# Patch type nucleotide sequence identities between genomes from many different species facilitate illegitimate recombination

**DOI:** 10.1038/s41598-026-44124-0

**Published:** 2026-03-30

**Authors:** Stefanie Weber, Christina M. Ramirez, Walter Doerfler

**Affiliations:** 1 Institute for Clinical and Molecular Virology, Friedrich-Alexander University Erlangen-Nürnberg, 91054 Erlangen, Germany; 2 Department of Biostatistics, UCLA Fielding School of Public Health, Los Angeles, CA 90095-1772 USA; 3 Institute for Genetics, University of Cologne, 50674 Cologne, Germany

**Keywords:** Patch-type nucleotide sequence identities, Illegitimate recombination, Genome plasticity, Evolution, Statistical properties, Built-in feature of four-letter genetic alphabet, *SARS-CoV-2*, Biochemistry, Evolution, Genetics, Microbiology, Molecular biology

## Abstract

Comparative analyses of nucleotide sequences across diverse taxa, including viruses, bacteria, plants, and mammals, consistently reveal patch-type sequence identities of around 45%. These identities consist of short stretches interspersed by mismatches. Similarly, identity patterns emerge in alignments of randomized shuffled or scrambled sequences. These findings suggest patch-type identities reflect intrinsic statistical properties of the four-letter genetic alphabet. Such patterns likely function as recognition signals for illegitimate recombination, a mechanism that promotes sequence insertions, exchanges, and rearrangements without extensive homology. Patch-type identities have been observed at integration sites of foreign DNA and may play a role in evolutionary innovation and rapid diversification (e. g. *SARS-CoV-2)*. Simulation data support the ideas that the frequency and length distribution of matching segments can be predicted by statistical models based on base composition, yet may also create local environments conducive to recombination. Further, the statistical architecture of the genetic alphabet encodes not only biological information, but also the potential for genome remodeling and adaptation during evolution. By bridging fundamental sequence properties with biological outcomes, this study provides a framework for exploring how randomness at the nucleotide sequence level can give rise to order and complexity across the tree of life.

**Supplementary Information:**

The online version contains supplementary material available at 10.1038/s41598-026-44124-0.

## Introduction

Studies of viral and host genomes have long leveraged sequence alignments to identify conserved regions and infer functional or evolutionary relationships. In an effort to better understand the genomic structure of *SARS-CoV-2* and its rapidly evolving variants during the pandemic, we compared the full-length *SARS-CoV-2*^1^ genome to those of *human Adenoviruses (Ad2* and *Ad12*)^2^. Surprisingly, we observed consistent patch-type sequence identities of about 45% across these otherwise unrelated genomes. These identities consisted of short, exact matches distributed irregularly throughout the sequence and were not restricted to specific genomic regions.

To investigate whether this pattern was specific to *SARS-CoV-2* or *Adenoviruses*, we expanded our analysis to include more than 90 genomes from a wide array of species including viruses, bacteria, plants, and mammals, spanning a range of GC content and nucleotide composition biases. Importantly, these sequences were selected to represent a broad spectrum of genome architectures, including both coding and non-coding regions and sequences with varying strand asymmetries. Remarkably, similar patch-type identities were observed across all comparisons, regardless of taxonomic distance or functional relevance.

Furthermore, alignments of randomized or shuffled sequences—preserving base composition but lacking biological organization—produced the same identity levels and structural patterns. This consistency suggests that such patch-type identities are not artifacts of alignment algorithms or evolutionary convergence but rather an inherent statistical feature of the four-letter genetic alphabet.

Previous studies have documented similar patch-type sequence identities at junction sites where foreign DNA integrates into host genomes, such as those involving retroviruses, transposons, and adenoviral DNA^3–20^. These integration events are often catalyzed by illegitimate recombination—a process that does not require extended homologies and can utilize short sequence matches as recombination signals.^3–20^ [Examples from earlier publications Figs. SA,^7^ and SB,^16^ under Supplemental Materials].

In this study, we hypothesize that patch-type nucleotide sequence identities arise from the statistical properties of the four-nucleotide genetic alphabet—including base composition and strand bias, and that these patterns have been evolutionarily co-opted as functional signals for illegitimate recombination. Simulations presented here model expected alignment patterns based on genome composition and reveal that patch-type identity levels emerge predictably even in unrelated sequence pairs. At the same time, localized identity-rich regions may act as potential recombination substrates in vivo. This mechanism could underlie a range of biological processes, including genome integration, transposition, and sequence exchange, and may have facilitated the rapid evolution of RNA viruses such as *SARS-CoV-2*.

To test this hypothesis, we systematically aligned both natural and shuffled nucleotide sequences across a diverse range of species using two independent alignment tools. We compared complete genomes, genomes of varying GC content [Tables 1, 2 and Tables S2–S4 under Supplemental materials], and sequences with known strand biases, as well as randomized and composition-matched controls. In addition, we used simulation-based control approaches to model the expected identity distributions based on base composition and sequence length. By analyzing the frequency, length distribution, and spatial distribution of matching sequences, we demonstrate that patch-type identities are a predictable statistical consequence of nucleotide sequence architecture. At the same time, certain regions exhibit elevated identity relative to random expectations, which may indicate functional recombination hotspots. These results provide a framework for understanding how statistical properties of the genetic code contribute to genome dynamics and evolutionary plasticity.

**Table 1:** Nucleotide sequence comparisons among the here-presented 14 genome pairs from a wide range of taxonomically diverse species. Common names for selected species include: *Ilex aquifolium* (English holly), *Bombus pascuorum* (common carder bee; see Fig. S5), *Latimeria chalumnae* (coelacanth, closely related to lungfish; *Cylas formicarius* (sweet potato weevil), *Lycium barbarum* (matrimony vine), *Zootoca vivipara* (viviparous lizard), *Sus scrofa* (wild boar) (Table S1), and *Triticum aestivum* (common wheat). Genomic coordinates for each alignment are provided in the leftmost column. The second column shows the GC content of the two DNA sequences compared. All comparisons span the full length of the shorter sequence in each pair. As detailed in the Materials and Methods section, alignments were conducted using two independent programs—Vector NTI and BLAST. The results from both methods are presented in adjacent columns and show high concordance, often yielding identical percentages.

Alignment	GC content	VNTI® Identity Positions	BLAST® Percent Identities
*Adenovirus type 2* (J01917.1; alignment complete genome) vs. *SARS-CoV-2 Wuhan-Hu-1* (NC_045512.2; alignment complete genome) *	55%*** vs. 38%***	43.2%	43%
*Adenovirus type 12* (X73487; alignment complete genome) vs *Homo sapiens* chromosome 1 (NC_000001.11; alignment from 11,783,698–11,817,823nt)**	46.5%*** vs. 51.27%	44.3%	44%
*Adenovirus type 12* (X73487; alignment complete genome) vs. *Homo sapiens* chromosome 13 (NC_000013.11; alignment from 34,882,059–34,916,184nt) **	46.5%*** vs. 39.23%	44.1%	44%
*Autographa californica nuclear polyhedrosis virus* (NC_001623.1; alignment from 42,843–75,223nt) vs. *SARS-CoV-2 Wuhan-Hu-1* (NC_045512.2; alignment complete genome) *	39.03% vs. 38%***	45.6%	45%
*Chlorocebus sabaeus* mitochondrion (NC_008066.1; alignment from 2,779–15,177nt) vs. *HERV-E* (AB062274.1; alignment complete genome) **	42.5% vs. 47.71%	44.5%	44%
*Hepatitis B Virus* (NC_003977.2; alignment complete genome) vs. *SARS-CoV-2 Wuhan-Hu-1* (NC_045512.2; alignment from 21,047–24,558nt) *	48.49% vs. 36.47%	45.7%	46%
*Homo sapiens* chromosome 1 (NC_000001.11; alignment from 11,783,698–11,817,823nt) vs. *Homo sapiens* chromosome 13 (NC_000013.11; alignment from 34,882,059–34,916,184nt) **	51.27% vs. 39.23%	44.5%	45%
*Human Immunodeficiency Virus 1* (K03455.1; alignment complete genome) vs. *SARS-CoV-2 Wuhan-Hu-1* (NC_045512.2; alignment from 699–11,486nt) *	42.5%*** vs. 36.99%	45.8%	46%
Phage *lambda* (*λ*) (NC_001416.1; alignment from 14,752–47,353nt) vs. *SARS-CoV-2 Wuhan-Hu-1* (NC_045512.2; alignment complete genome) *	46.97% vs. 38%***	45.3%	45%
*SARS-CoV-2 Wuhan-Hu-1* (NC_045512.2; alignment complete genome) vs. *Oryza sativa* chromosome 1 (BA000010.8; alignment from 9,891–41,663nt) **	38%*** vs. 41.52%	49.2%	46%
*Homo sapiens* chromosome 7 (NC_000007.14; alignment from 143,456–153,536nt) vs. *Ilex aquifolium* chromosome 11 (OX637401.1; alignment from 1,289–11,294nt) **	48.98% vs. 53.72%	43.7%	44%
*Homo sapiens* chromosome X (NC_000023.11; alignment from 604,089–614,089nt) vs. *Zootoca vivipara* chromosome W (NC_083293.1; alignment from 563,489–573,489nt) **	41.77% vs. 50.11%	42.2%	42%
*Mycobacterium tuberculosis* (AP018036.1; alignment from 4,403,362–4,413,362nt) vs. *Oryza sativa* chromosome 8 (NC_029263.1; alignment from 844,302–854,302nt) **	63.09% vs. 33.19%	40.2%	40%
*Mus musculus* strain C57BL/6 J chromosome 19 (NC_000085.7; alignment from 7,159,736–7,169,736nt) vs. *Triticum aestivum* cultivar Chinese Spring chromosome 6D (NC_057811.1; alignment from 5,380,293–5,390,293nt) **	55.1% vs. 44.91%	43.1%	43%

*Data as cited in Weber et al., 2022^30^. **New data. ***The GC content of the DNA sequence is taken from the NCBI database. All other GC values were determined using the online tool “GC Content Calculator” from VectorBuilder (https://en.vectorbuilder.com/tool/gc-content-calculator.html).

**Table 2:** Sequence comparisons involving selected human repetitive elements, including *Alu* elements, *LINE-1* elements, and *SINE* repeat regions, aligned against genomic sequences from a broad range of species. Patch-type sequence identities in these comparisons generally ranged from 41 to 48%, consistent with the results in Tables 1, 2 and S1–S4. Notably, in comparisons involving closely related human genomic segments—particularly among *Alu* elements—identity levels exceeded 89%, reflecting the high degree of conservation within these repetitive sequence families. All GC values were determined using the online tool “GC Content Calculator” from VectorBuilder(https://en.vectorbuilder.com/tool/gc-content-calculator.html).

Alignments	GC content	VNTI® Identity Positions
Human specific *Alu element HS C4N5* (X54177.1; alignment complete genome) vs. *Homo sapiens* chromosome 15 1000 genomes pilot1 *Alu locus 1 NA18953 hg18 49,778,223* genomic sequence (KT305395.1; alignment from 173 bp—508 bp)	55.83% vs. 54.76%	89,6%
Human specific *Alu element HS C4N5* (X54177.1; alignment complete genome) vs. *Homo sapiens* clone *AluYi6AH121 SINE* repeat region (AY190771.1; alignment from 142 bp—477 bp)	55.83% vs. 51.79%	81%
Human specific *Alu element HS C4N*5 (X54177.1; alignment complete genome) vs. Human *Mermaid LINE-1* element (U31059.1; alignment complete genome)	55.83% vs. 42.88%	35,8%
Human specific *Alu element HS C4N5* (X54177.1; alignment complete genome) vs. *Ad 12* (X73487; alignment from 15,189 bp—15,526 bp)	55.83% vs. 63.91%	50,1%
Human specific *Alu element HS C4N5* (X54177.1; alignment complete genome) vs. *Bombus pascuorum* chromosome 14 (NC_083501.1; alignment from 116,147 bp—116, 509 bp)	55.83% vs. 40.22%	45,1%
Human specific *Alu element HS C4N5* (X54177.1; alignment complete genome) vs. *Chlorocebus sabaeus* mitochondrion (NC_008066.1; alignment from 2,624 bp—2,941 bp)	55.83% vs. 44.03%	47,7%
Human specific *Alu element HS C4N5* (X54177.1; alignment complete genome) vs. *Oryza sativa* chromosome 2 (NC029257.1; alignment from 41,873 bp—42,223 bp)	55.83% vs. 37.04%	45,9%
Human specific *Alu element HS C4N5* (X54177.1; alignment complete genome) vs. *SARS-CoV-2 Wuhan Hu-1* (NC045512.2; alignment from 28,656 bp—29,009 bp)	55.83% vs. 49.72%	46,2%
*Homo sapiens* chromosome 15 1000 genomes pilot1 *Alu locus 1 NA18953 hg18 49,778,223* genomic sequence (KT305395.1; alignment complete genome) vs. *Homo sapiens* clone *AluYi6AH121 SINE* repeat region (AY190771.1; alignment complete genome)	52.04% vs. 48.58%	67%
*Homo sapiens* chromosome 15 1000 genomes pilot1 *Alu locus 1 NA18953 hg18 49,778,223* genomic sequence (KT305395.1; alignment from 1 bp—334 bp) vs. Human *Mermaid LINE-1* element (U31059.1; alignment complete genome)	52.04% vs. 42.88%	41,5%
*Homo sapiens* chromosome 15 1000 genomes pilot1 *Alu locus 1 NA18953 hg18 49,778,223* genomic sequence (KT305395.1; alignment complete genome) vs. *Ad 12* (X73487; alignment from 7,969 bp—8,564 bp)	52.04% vs. 56.38%	45,5%
*Homo sapiens* chromosome 15 1000 genomes pilot1 *Alu locus 1 NA18953 hg18 49,778,223* genomic sequence (KT305395.1; alignment complete genome) vs. *Bombus pascuorum* chromosome 14 (NC_083501.1; alignment from 120,025 bp—120,591 bp)	52.04% vs. 41.98%	45,9%
*Homo sapiens* chromosome 15 1000 genomes pilot1 *Alu locus 1 NA18953 hg18 49,778,223* genomic sequence (KT305395.1; alignment complete genome) vs. *Chlorocebus sabaeus* mitochondrion (NC_008066.1; alignment from 1,612 bp—2,190 bp)	52.04% vs. 40.76%	44,2%
*Homo sapiens* chromosome 15 1000 genomes pilot1 *Alu locus 1 NA18953 hg18 49,778,223* genomic sequence (KT305395.1; alignment complete genome) vs. *Oryza sativa* chromosome 2 (NC029257.1; alignment from 42,479 bp—43,045 bp)	52.04% vs. 50.44%	45,5%

## Materials and methods

### Selection and preparation for sequences for analysis

Patient-derived *SARS-CoV-2* variant sequences were obtained from the GISAID database^21^. To ensure high-quality alignments, only sequences classified as complete (i.e., > 29,000 nucleotides) and annotated with high coverage (defined as < 1% undefined bases, or “Ns”) were included. Sequences containing > 5% undefined nucleotides were excluded to avoid artifacts due to low-quality sequences or incomplete genomes.

All sequences were aligned to the reference *SARS-CoV-2* genome, *Wuhan-Hu-1* (NCBI Reference Sequence: NC_045512.2)^1,22–24^, the earliest publicly released *SARS-CoV-2* genome.

Additional complete genomic sequences representing viruses, bacteria, plants, and mammalian species were obtained from the NCBI nucleotide database (https://www.ncbi.nlm.nih.gov/nucleotide/) and were selected to represent a broad range of taxonomic groups, GC content [Tables 1, 2 and Tables S2 to S4 under Supplemental materials], genome architectures, and coding/non-coding structure. Special attention was paid to include sequences with known strand bias or compositional asymmetry to assess potential contributions to alignment behavior.

### Programs applied for pairwise alignments

All pairwise alignments were performed in duplicate using two independent tools: Vector NTI Advance® 11 (Thermo Fisher Scientific) and the Basic Local Alignment Search Tool (BLAST®), to ensure consistency across alignment platforms. Both tools were used to assess sequence identities across complete genomes or selected genomic segments from diverse species.

#### Vector NTI AlignX settings

Pairwise alignments were conducted using the AlignX module of Vector NTI Advance® 11^22–24^. The following alignment parameters were applied for nucleotide comparisons:

- K-tuple size: 2
- Number of best diagonals: 2
- Window size: 4
- Gap penalty: 5
- Gap opening penalty: 15
- Gap extension penalty: 6.66

Multiple sequence alignments, including comparisons between *SARS-CoV-2* variants (e.g., *BA.1*, *BA.2*) and the *Wuhan-Hu-1* reference genome, were also performed using AlignX. These alignments were based on ClustalW^25^, using the software’s predefined parameters:

- Gap opening penalty: 15
- Gap extension penalty: 6.66
- Gap separation penalty range: 8
- Identity threshold for alignment delay: 40%
- Transition weighting: 0

#### BLAST settings

Alignments were repeated using the BLAST® tool, applying the Needleman-Wunsch global alignment algorithm^26^. The default scoring parameters were:

- Match score: + 1
- Mismatch penalty: − 2
- Gap existence penalty: 5
- Gap extension penalty: 2

To assess the robustness of the results to scoring parameters, all available permutations of parameter configurations within the BLAST interface were tested across representative genome pairs. These variations yielded highly consistent results, confirming that the observed identity levels and patch-type patterns were not artifacts of specific parameter choices.

### Sequence alignment strategy

Complete genome sequences from a range of organisms were aligned in pairwise fashion to assess nucleotide sequence identity. For comparisons between genomes of substantially different lengths, we first performed a global alignment to identify the region within the longer genome with highest similarity to the shorter one. That region was then locally re-aligned to refine identity estimates. For example, a 7,140-nucleotide genome of *Fig badnavirus 1* was globally aligned to the 29,903-nucleotide genome of *SARS-CoV-2 Wuhan-Hu-1,* and the best matching region (nucleotides 1,978—9,647 in the *SARS-CoV-2* genome) was extracted and realigned, yielding a refined identity of 45.5% (see Figs. S9 and S10).

For genome pairs with similar lengths, a single global alignment was performed without the need for regional refinement. Comparisons involving individual chromosomes or genome segments were based on pre-selected regions of equivalent length to ensure valid pairwise evaluation.

These alignments were conducted using both the AlignX tool (Vector NTI Advance® 11) and the BLAST® program, as described in Sect. "Programs Applied for Pairwise Alignments". Results obtained from both tools were highly consistent across all comparisons.

To complement the AlignX/BLAST analyses, we implemented an automated best-segment overlap workflow in R (Biostrings, IRanges, pwalign, optparse, dplyr, ggplot2). For each genome pair we treated the shorter genome, here *SARS-CoV-2 Wuhan-Hu-1*. The longer genome was scanned in coarse steps (default 200 nt) to identify windows with elevated un-gapped similarity; the top-scoring windows (default 200) were then refined by dynamic-programming overlap alignments with ± 800 nt padding. Alignments were required to span ≥ 95% of the pattern segment, guaranteeing that the reported “best window” reflects a contiguous, full-length overlap. After identifying the best overlap on the native strand, we repeated the entire search against the reverse complement of the longer genome using the same parameters.

Unless otherwise stated, the pattern sequence was fixed to a 10 kb window of *SARS-CoV‑2 Wuhan-Hu-1* (nucleotides 9,952–19,951; 3,285–13,284 for the human mitochondrial comparison to avoid the control region), so every partner genome was queried with the same segment before the automated scan and refinement steps.

### Shuffling of sequences for randomized controls

To evaluate whether patch-type sequence identities arise from biological organization or inherent sequence statistics, we created several control datasets. First, shuffled sequences were generated with the Sequence Manipulation Suite [https://www.bioinformatics.org/sms2/shuffle_dna.html, by randomly permuting nucleotides within each natural sequence while preserving sequence length and base composition. Second, composition-matched random sequences were generated in R by sampling nucleotides based on the base frequency distribution of each genome, maintaining strand bias and compositional asymmetries but not nucleotide order. Third, we created fully randomized sequences by sampling A, T, C, and G with equal probability (25% each), eliminating both biological sequence structure and compositional bias. All control sequences were aligned to their original, unshuffled counterparts and to unrelated genomes, including *SARS-CoV-2*, *Adenoviruses*, *Mycobacterium tuberculosis*, and *Plasmodium falciparum*—as well as to human chromosomes 1 and 13. These comparisons were designed to evaluate whether patch-type identities could arise purely from statistical features of sequence composition, independent of biological function or evolutionary history.

### Quantitative assessment of identity patterns in control sequences

To further investigate the properties of patch-type sequence identities, we conducted detailed analyses on a representative set of nine genomic sequences. These included complete genomes from *SARS-CoV-2* (NC_045512.2), *Adenovirus 2* (AC_000007.1), *Adenovirus 5* (AC_000008.1), *Adenovirus 12* (X73487), and *Acidianus rod-shaped virus 1* (NC_009965.1), as well as endogenous retroelements and retrotransposons such as *LINE-1* (M80343), *HERV-E* (AB062274.1), *HERV-W* (NM_014590.4), and *HERV-K* (AF074086).

For each sequence, pairwise alignments were performed to compute percent identity. Frequencies of exact nucleotide matches ranging in lengths from 1-mer to 11-mer runs were quantitated in Figs. 4 and 5. Thus profiles of identity patterns for both related and unrelated genomic comparisons were produced.

To evaluate the statistical properties underlying these identity patterns, we performed control experiments using two classes of synthetic sequencies. First five hundred randomized sequences were generated by sampling nucleotides (A, T, C, G) at uniform frequencies. These sequences were constructed at two lengths—3,500 and 35,000—nucleotides representing the lower and upper bounds of the real genomic sequences used in the study. Second, additional control sequences were created by shuffling the nucleotide order within authentic genomic sequences, thereby preserving the original base composition while eliminating inherent biological structure. To explore the impact of compositional bias and base asymmetry, additional simulations were conducted using composition-matched controls and GC-rich versus GC-poor genomes, including *Mycobacterium tuberculosis* and *Plasmodium falciparum* (Table 3). These sequences were compared to real genomic sequences using a windowed alignment strategy that identifies the best-matching local region for each sequence pair, followed by a geometric analysis of run length distributions.

**Table 3:** Nucleotide sequence comparisons between the genome of *SARS-CoV-2 Wuhan-Hu-1* and genomes of three different species.

Comparison	GC% (target)	GC% (query)	Identity (%)
*SARS-CoV-2 Wuhan-Hu-1* vs. *Mycobacterium tuberculosis*	38%	66%	26.50%
*SARS-CoV-2 Wuhan-Hu-1* vs. *Plasmodium falciparum*	38%	36%	30.50%
*SARS-CoV-2 Wuhan-Hu-1* vs. *Ad2*	38%	51%	95.20%

The distributions of contiguous identity runs were visualized using bar plots, and statistical comparisons across groups were conducted using the Kruskal–Wallis test^27^. These analyses revealed that both natural and shuffled sequences consistently yielded patch-type identity distributions that conformed closely to geometric expectations based on their underlying base composition. Notably, while the overall patch-type structure was preserved in control sequences, certain localized regions in biological sequences exhibited higher identity than predicted by simulations, suggesting the potential presence of recombination-prone hotspots. Together, these data support the interpretation that patch-type sequence identities reflect a combination of statistical expectation and selective retention in biologically active regions.

The results of all pairwise nucleotide sequence alignments—including accession numbers, species names, and genomic coordinates—are summarized in Tables 1 and 2 (show selected entries) Tables S1 and S2 (show all data). These alignments span a wide taxonomic range and include both coding and non-coding genomic regions. To enable visual inspection of patch-type identity distributions, representative alignments are shown in Figs. 1–5, while full-length global alignments are provided as Supplemental Figs. S1–S15. These visualizations reveal the irregular, non-contiguous nature of identity patches, which typically consist of short exact matches interspersed with mismatches and gaps.

**Fig. 1:**
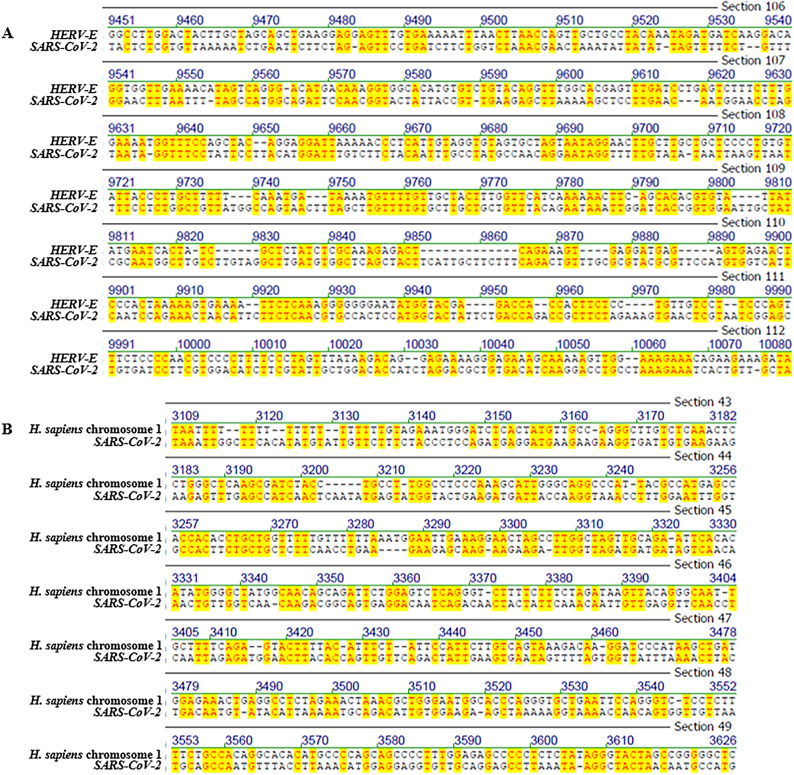

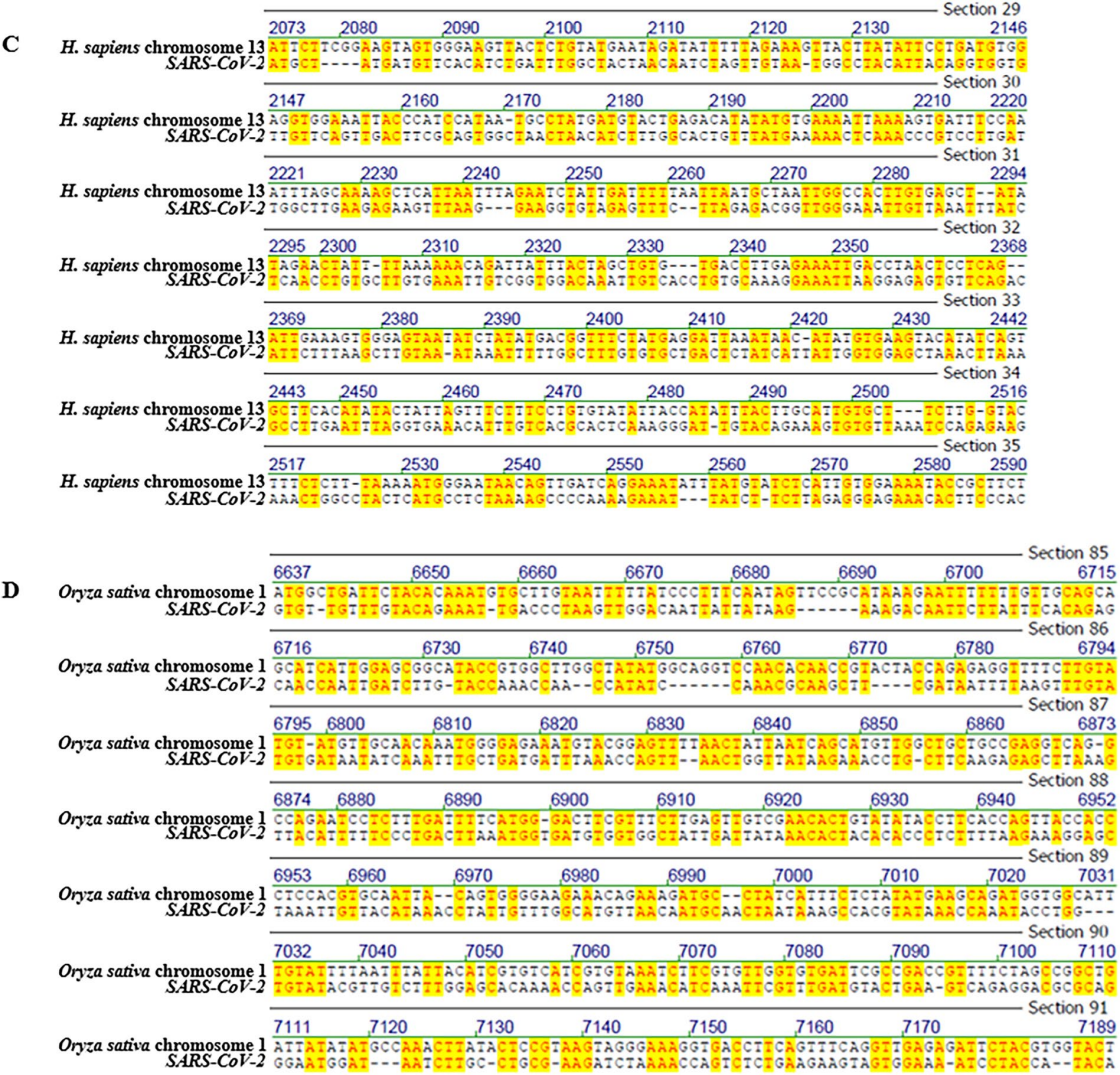
Representative screenshots of pairwise sequence alignments between the full-length *SARS-CoV-2 Wuhan-Hu-1* genome (29,903 nucleotides) and selected genomic regions from diverse species: (**A**) *human endogenous retrovirus type E* (*HERV-E*), (**B**) *Homo sapiens* chromosome 1, (**C**) *Homo sapiens* chromosome 13, and (**D**) *Oryza sativa* chromosome 1. These alignments illustrate the characteristic patch-type sequence identities, with short stretches of identical nucleotides interspersed with mismatches. Full-length scrollable alignments for each comparison can be examined in Supplementary Figs. S1–S4.

**Fig. 2:**
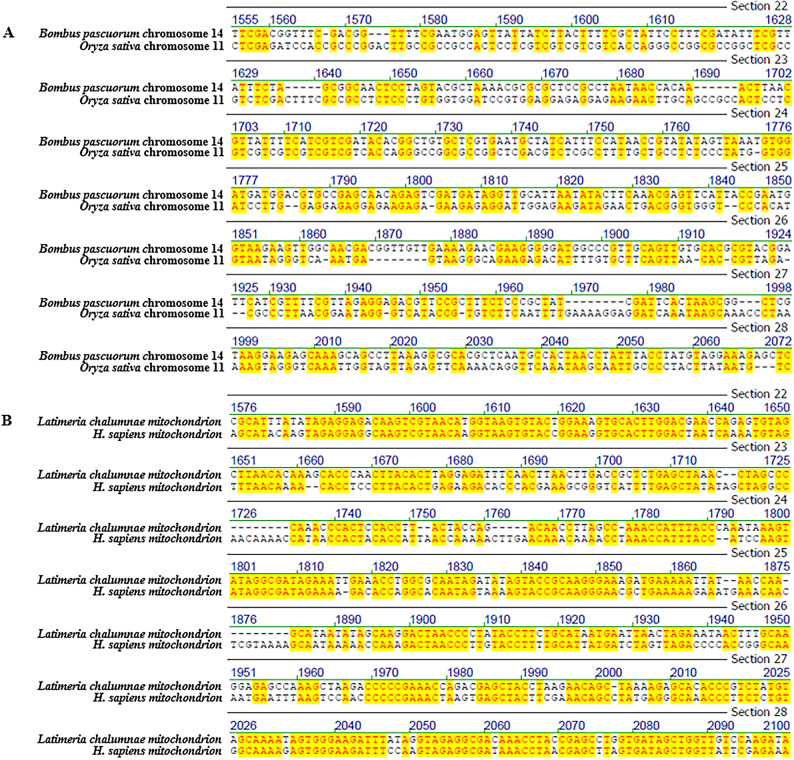

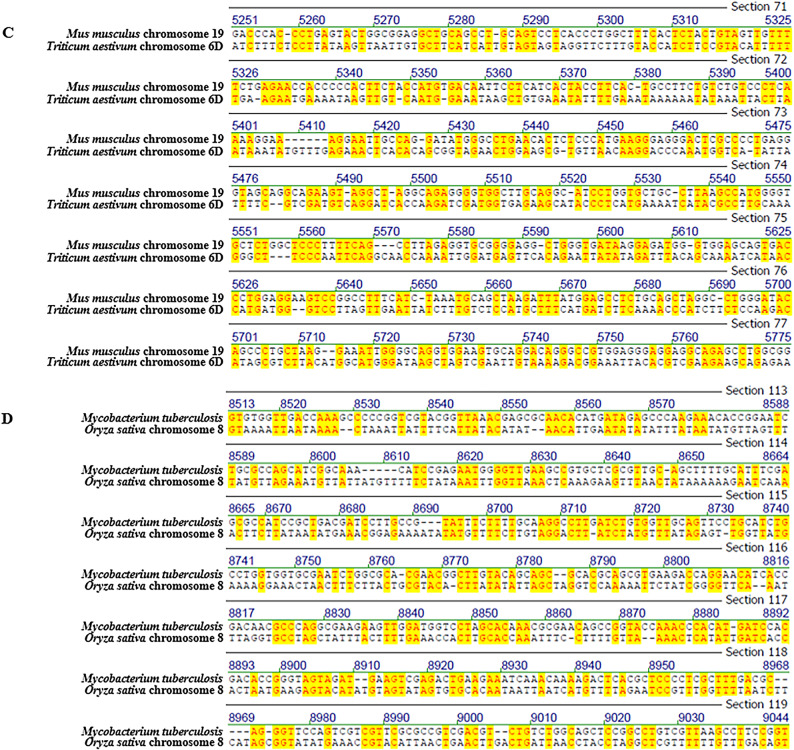
Representative screenshots of pairwise sequence alignments, illustrating patch-type sequence identities across distantly related genomes. Comparisons include: (**A**) *Bombus pascuorum* chromosome 14 vs. *Oryza sativa* chromosome 11, (**B**) *Homo sapiens* mitochondrion vs. *Latimeria chalumnae* mitochondrion, (**C**) *Mus musculus* strain C57BL/6 J chromosome 19 vs. *Triticum aestivum* cultivar Chinese Spring chromosome 6D, and (**D**) *Mycobacterium tuberculosis* vs. *Oryza sativa* chromosome 8. Complete scrollable alignments for each comparison are provided in Supplementary Figs. S5–S8. Additional alignment details can be found in Tables 1 and S1.

**Fig. 3:**
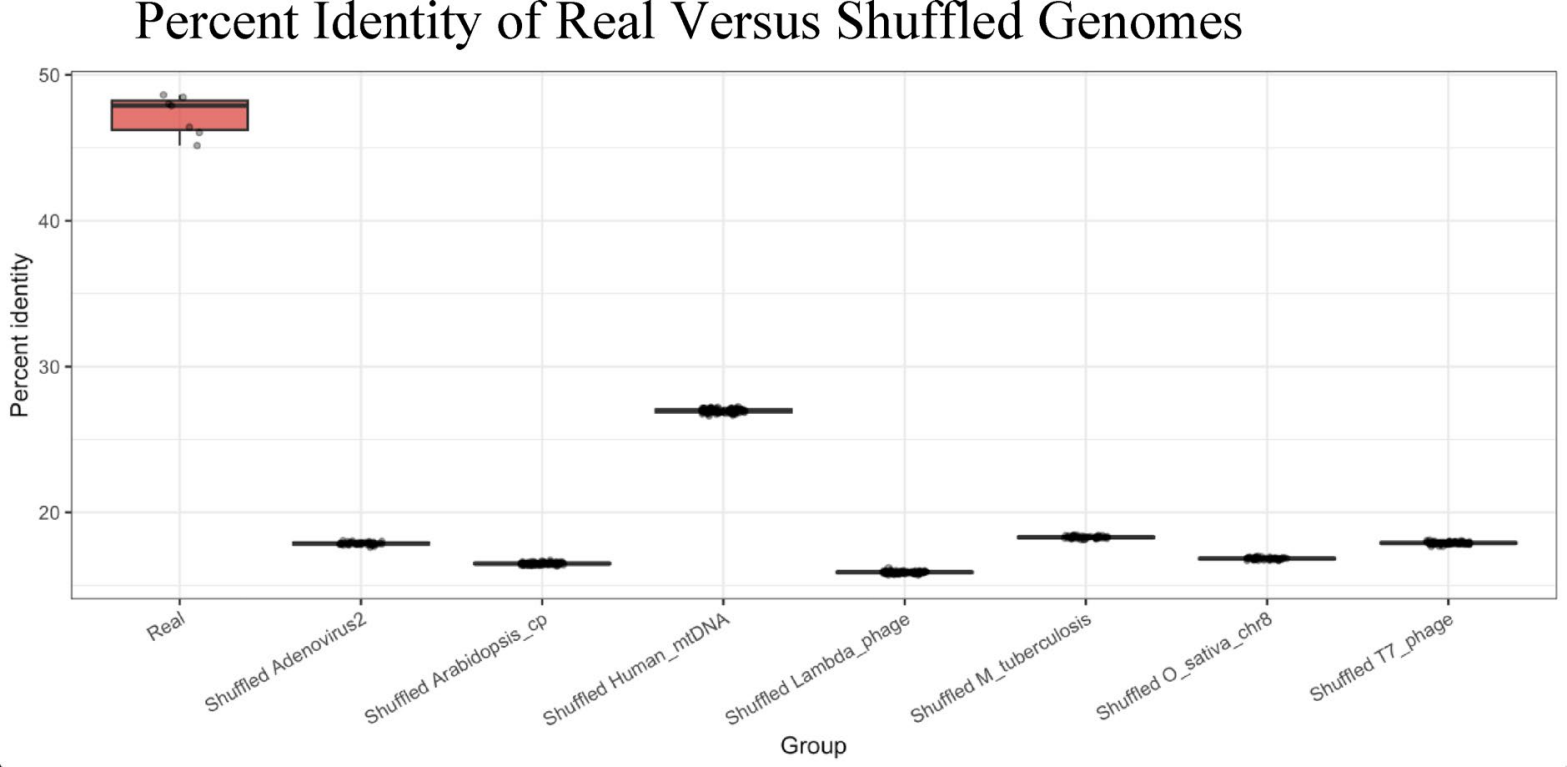
Boxplots show the real best-window identity (“Real”; n = 7) and the composition-matched shuffle cohorts for each comparison (50–100 shuffles per genome, labelled by partner). Dots represent individual percent identities. Real overlaps cluster near 45–49%, whereas shuffled windows fall between ~ 16% and 27%. The pooled Kruskal–Wallis’s^27^ test across all eight groups yields χ^2^ = 535.2 (df = 7, p < 2.2 × 10⁻^1^⁶), demonstrating that the biological overlaps are significantly higher than the composition-matched null distributions.

**Fig. 4:**
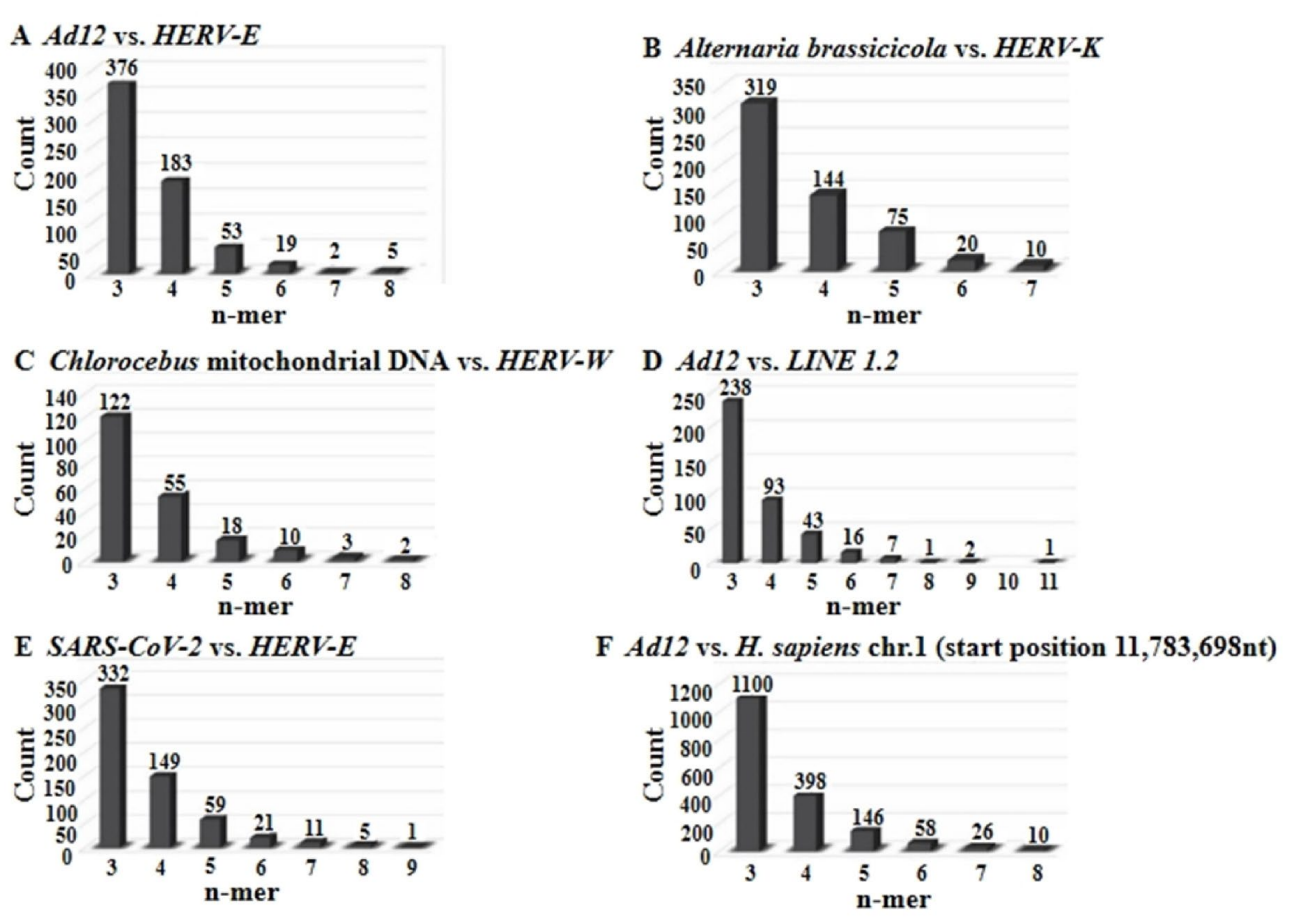
Bar graphs that summarize the results of quantitative determinations of 3-mers to maximally 11-mers of trans-species patchy nucleotide sequence identities in total sequence comparisons of very diverse species as described in panels A through F.

**Fig. 5:**
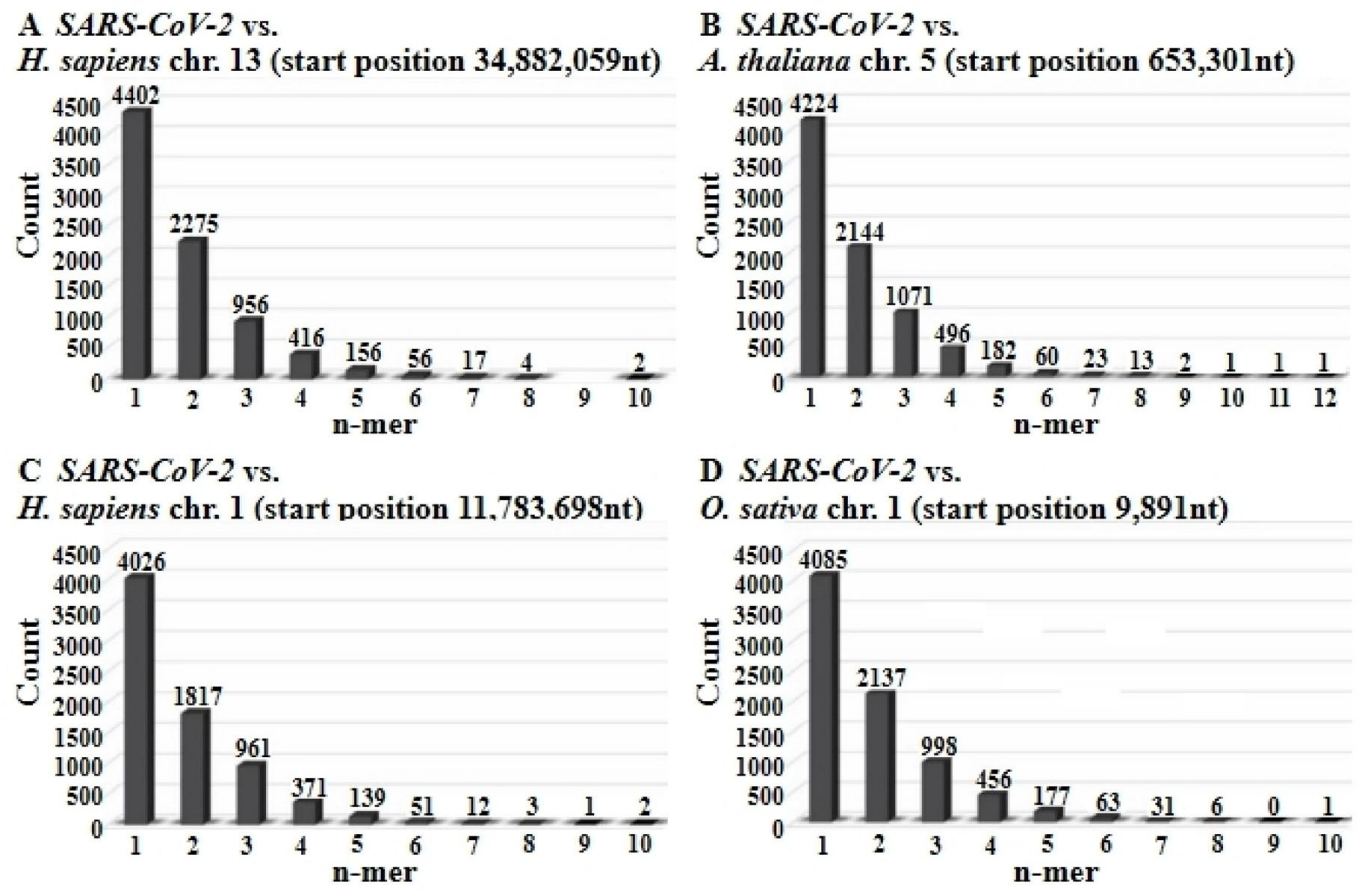
Bar graphs quantifying the frequency of contiguous identical nucleotide stretches (1-mers to 12-mers) in pairwise alignments between the full-length *SARS-CoV-2 Wuhan-Hu-1* genome (29,903 nucleotides) and selected genomic segments from other species. Panels A—D represent alignments with: (**A**) *Homo sapiens* chromosome 13 (49.4% overall identity), (**B**) *Arabidopsis thaliana* chromosome 5 (50.8%), (**C**) *Homo sapiens* chromosome 1 (44.0%), and (**D**) *Oryza sativa* chromosome 1 (49.2%). The graphs reflect the total number of identical sequences of each length observed across the entire *SARS-CoV-2* genome, illustrating the predominance of short identical stretches and the characteristic patch-type identity distribution.

In addition to alignments of natural sequences, comparisons with randomized and shuffled controls are presented in Table 4 and illustrated in Figs. 7–9. These controls reproduce the patch-type identity patterns observed in biological sequences, supporting the conclusion that such identities are a predictable statistical outcome of nucleotide composition and sequence length in the four-letter genetic system. The local alignments used in these simulations focused on identifying the best-matching regions within large genomes using adaptive windowing, ensuring computational feasibility while preserving biological relevance. Bar plots and histograms show that the lengths and distributions of identity runs are highly similar across biological and control datasets, with identity levels centering around 45% in most cases.

**Table 4:** Summary of pairwise alignments involving five independently shuffled versions of the *Ad12* (X73487) and *SARS-CoV-2 Wuhan-Hu-1* (NC_045512.2) genomes. Each shuffled sequence was aligned to the corresponding original (unshuffled) viral genome and to selected unshuffled segments from human chromosomes 1 and 13. The resulting identity percentages consistently ranged between 42 and 46%, comparable to values obtained from natural sequence comparisons. These results demonstrate that patch-type sequence identities are preserved even in randomized sequences and suggest that such identity patterns reflect a statistical property intrinsic to the four-letter genetic alphabet. Full alignments are available in Supplementary Figs. S14 and S15. These alignments can also be scrolled through their entire lengths.

Original sequence vs. original sequence	Original sequence	Shuffled sequences	Identity positions
	*SARS-CoV-2 Wuhan-Hu-1*	*SARS-CoV-2 Shuffle 1*	46.2%
		*SARS-CoV-2 Shuffle 2*	45.6%
		*SARS-CoV-2 Shuffle 3*	46.5%
		*SARS-CoV-2 Shuffle 4*	46.5%
		*SARS-CoV-2 Shuffle 5*	46.4%
*SARS-CoV-2 Wuhan-Hu-1* vs. *Ad12* = 43.2%	*Adenovirus 12*	*SARS-CoV-2 Shuffle 1*	42.8%
		*SARS-CoV-2 Shuffle 2*	43.2%
		*SARS-CoV-2 Shuffle 3*	42.8%
		*SARS-CoV-2 Shuffle 4*	42.3%
		*SARS-CoV-2 Shuffle 5*	43.4%
	*Adenovirus 12*	*Adenovirus 12 Shuffle 1*	44.8%
		*Adenovirus 12 Shuffle 2*	44.7%
		*Adenovirus 12 Shuffle 3*	44.8%
		*Adenovirus 12 Shuffle 4*	44.8%
		*Adenovirus 12 Shuffle 5*	44.7%
	*SARS-CoV-2 Wuhan-Hu-1*	*Adenovirus 12 Shuffle 1*	43.3%
		*Adenovirus 12 Shuffle 2*	43.3%
		*Adenovirus 12 Shuffle 3*	43.3%
		*Adenovirus 12 Shuffle 4*	43.6%
		*Adenovirus 12 Shuffle 5*	42.1%
*SARS-CoV-2 Wuhan-Hu-1* vs. *Homo sapiens* chromosome 1 NC_000001.11; 11,783,698–11,813,601 = 43.9%	*Homo sapiens* chromosome 1 NC_000001.11; 11,783,698–11,813,601	*SARS-CoV-2 Shuffle 1*	43.4%
		*SARS-CoV-2 Shuffle 2*	43.4%
		*SARS-CoV-2 Shuffle 3*	43.6%
		*SARS-CoV-2 Shuffle 4*	43.5%
		*SARS-CoV-2 Shuffle 5*	43.5%
*SARS-CoV-2 Wuhan-Hu-1* vs. *Homo sapiens* chromosome 13 NC_000013.11; 34,882,059–34,911,962 = 45.4%	*Homo sapiens* chromosome 13 NC_000013.11; 34,882,059–34,911,962	*SARS-CoV-2 Shuffle 1*	45.4%
		*SARS-CoV-2 Shuffle 2*	45.1%
		*SARS-CoV-2 Shuffle 3*	45.4%
		*SARS-CoV-2 Shuffle 4*	45.4%
		*SARS-CoV-2 Shuffle 5*	45.6%
*Ad12* vs. *Homo sapiens* chromosome 1 NC_000001.11; 11,783,698–11,817,823 = 44.3%	*Homo sapiens* chromosome 1 NC_000001.11; 11,783,698–11,817,823	*Adenovirus 12 Shuffle 1*	44.1%
		*Adenovirus 12 Shuffle 2*	44.1%
		*Adenovirus 12 Shuffle 3*	44.1%
		*Adenovirus 12 Shuffle 4*	44.1%
		*Adenovirus 12 Shuffle 5*	44.4%
*Ad12* vs. *Homo sapiens* chromosome 13 NC_000013.11; 34,882,059–34,916,184 = 44.1%	*Homo sapiens* chromosome 13 NC_000013.11; 34,882,059–34,916,184	*Adenovirus 12 Shuffle 1*	44.2%
		*Adenovirus 12 Shuffle 2*	44.9%
		*Adenovirus 12 Shuffle 3*	44.6%
		*Adenovirus 12 Shuffle 4*	44.7%
		*Adenovirus 12 Shuffle 5*	44.4%

**Fig. 6:**
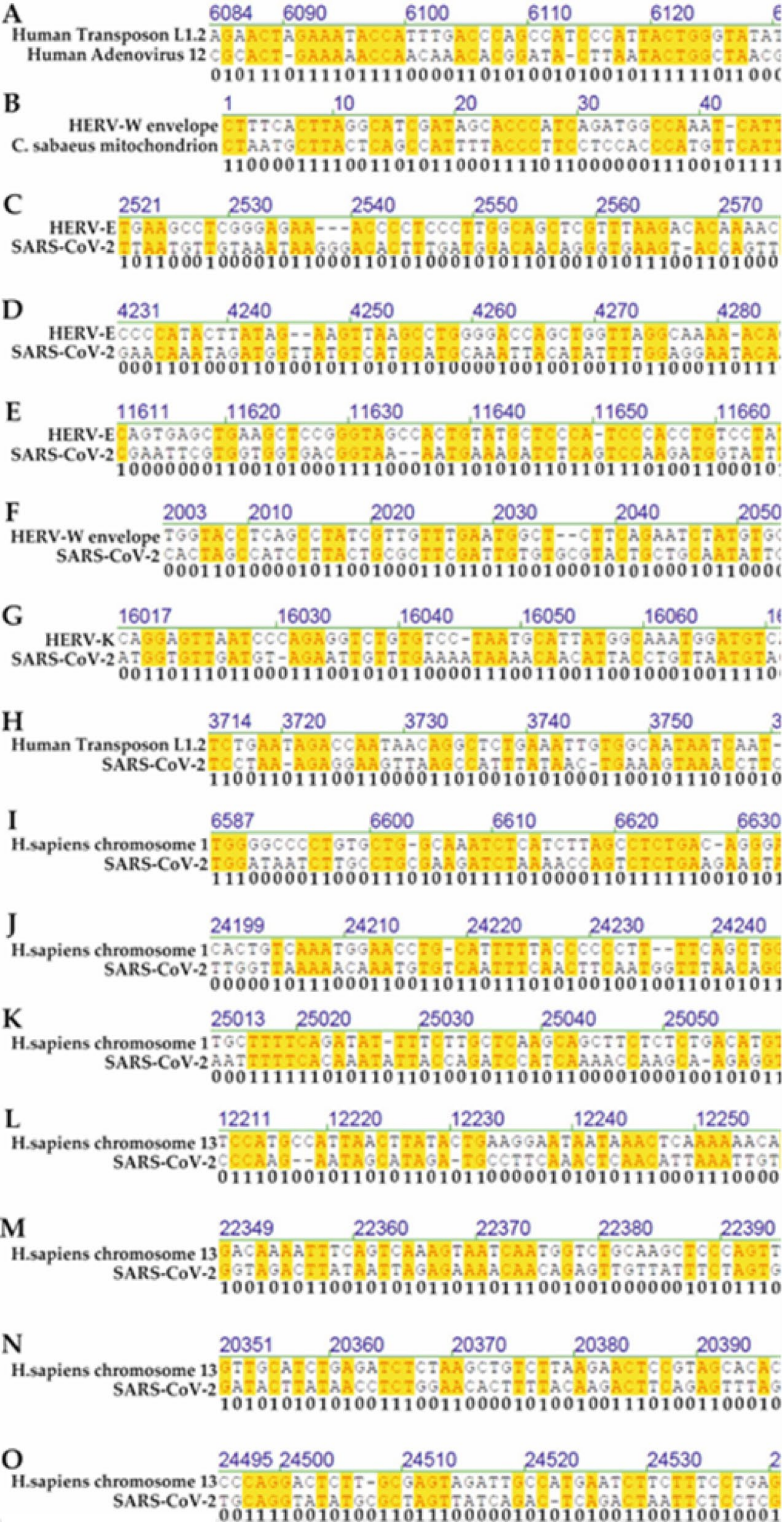
Representative examples of patch-type nucleotide sequence identities between genomes, as shown in panels A through O. Each alignment highlights short stretches of identity (n = 1 to 10) interspersed with mismatches (0). Identical nucleotides are highlighted red, with yellow shading marking regions of local identity. The binary scale beneath each alignment (0 = mismatch; 1 = match) visually depicts the distribution of sequence identities across the length of each comparison. Panels C to O specifically present alignments between the *SARS-CoV-2 Wuhan-Hu-1* genome (converted to DNA format) and various segments of the human genome, illustrating the widespread and non-localized nature of patch-type sequence identities.

**Fig. 7:**
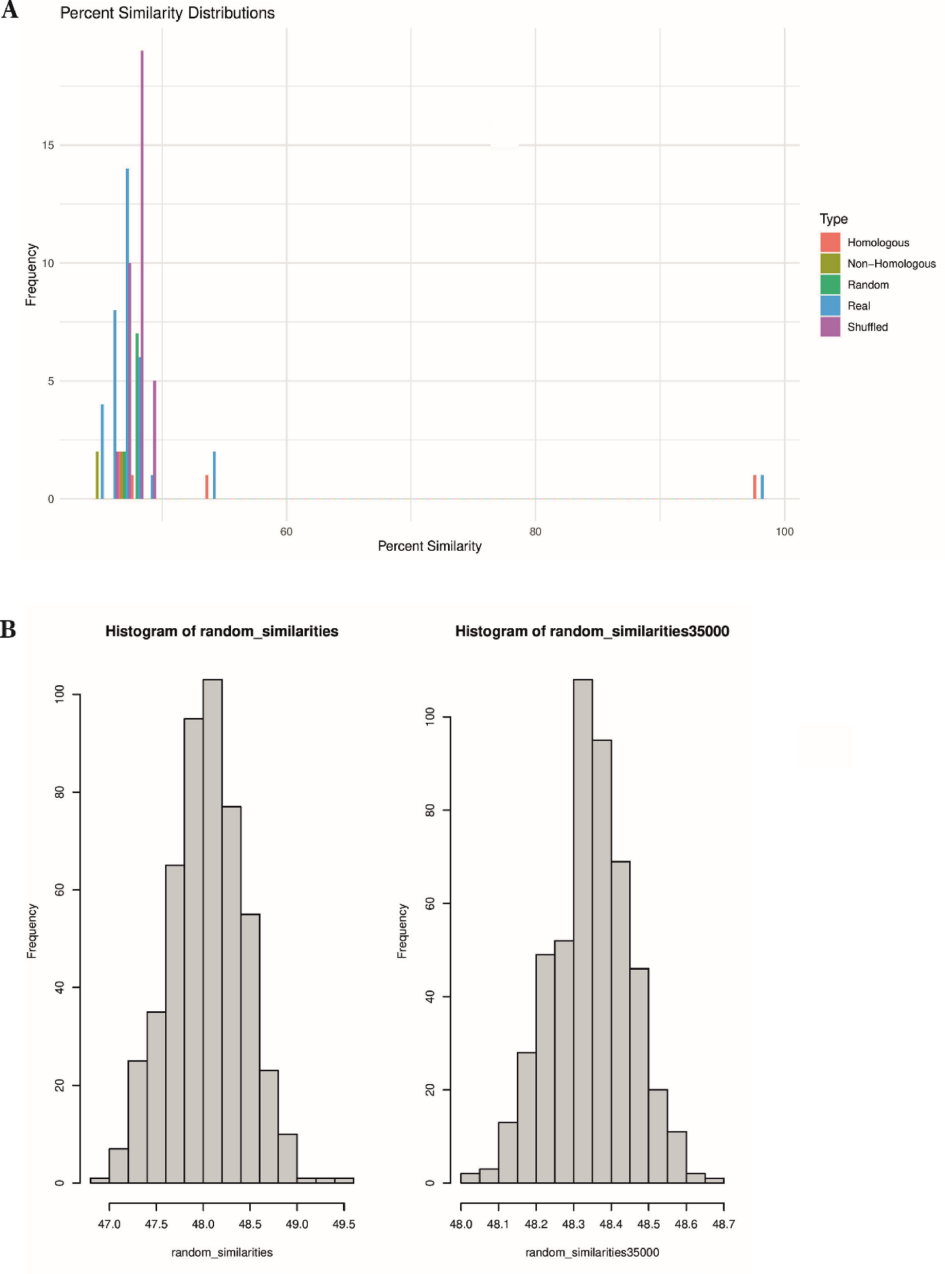
(**A**) Identity distributions from global sequence alignments across four groups: homologous sequences (*Adenovirus 2, Adenovirus 5, Adenovirus 12, HERV-E, HERV-W, HERV-K*), non-homologous sequences (e.g., *SARS-CoV-2*, *Adenovirus 2*, *Acidianus virus*, *LINE-1*, *HERV-E*), randomized real sequences (shuffled permutations of *SARS-CoV-2 Wuhan-Hu-1, Adenovirus 2, Adenovirus 5, Adenovirus 12*, and *Acidianus virus*), and synthetic random sequences composed of A, C, G, and T without constraint on nucleotide proportions. (**B**) Additional control comparing shuffled sequences of different lengths: 3,500 nucleotides (left panel) and 35,000 nucleotides (right panel). Across all conditions, identity levels were clustered around ~ 48%, indicating consistency in the frequency of sequence matches regardless of sequence origin or length.

**Fig. 8:**
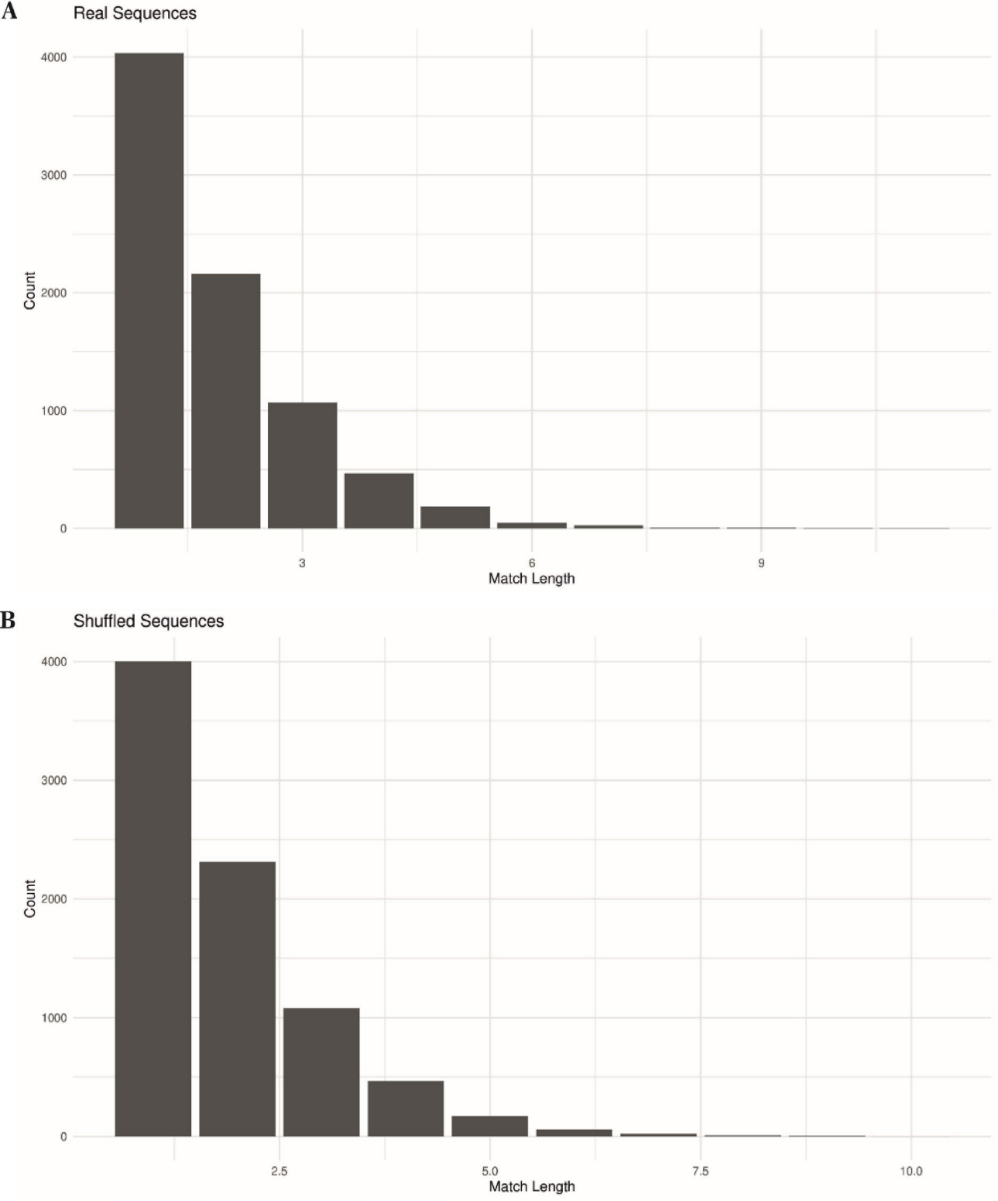
Bar graphs showing the frequency distribution of identical nucleotide stretches (1-mers to 11-mers) observed in pairwise sequence comparisons. (**A**) Summary of identity lengths from comparisons of homologous, non-homologous, shuffled real, and fully randomized sequences, as defined in Fig. 7. (**B**) Corresponding distributions for synthetic sequences generated by random sampling of A, C, G, and T bases.

To further highlight patterns of compositional influence, GC-rich and GC-poor genomes were included in the analyses, and simulations incorporating composition-matched random controls were performed [Tables 1, 2, and Tables S2–S4 under Supplemental materials]. These comparisons revealed that the shape and frequency of identity patches can vary slightly with base composition but still follow the same overall statistical structure. Collectively, these results provide strong empirical support for the hypothesis that patch-type sequence identities are an inherent property of the four-letter genetic system, while also identifying genomic regions with excess identity that may serve as candidates for recombination-prone hotspots.

In the automated best-segment workflow introduced above, we extended this quantitative framework in three ways. First, for each genome pair the optimal subject window was aligned globally against dinucleotide-preserving shuffles of itself, yielding a composition-matched null distribution of percent identities that was contrasted with the real overlap via the Kruskal–Wallis’s test^27^ and Cliff’s delta. Second, the contiguous match-run vector from every real alignment was compared to the geometric distribution implied by the observed base frequencies using the Kolmogorov–Smirnov statistic^28^, explicitly testing whether patch lengths exceed stochastic expectations. Third, we quantified base-class effects by enumerating matches and mismatches in AT, CG, and mixed contexts and evaluating enrichment with Fisher’s exact tests and logistic regression (match ~ base class, CG baseline). Sliding-window identity profiles (1 kb window, 250 nt step) were also computed across each alignment to highlight top decile windows as candidate hotspots.

### Rationale for choice of sequences analyzed

- The predominant factor in selecting was randomness.
- We preferentially investigated complete sequences that have been available.

We also included some viral sequences because of availability and relatedness to previous work on viruses from this laboratory.

## Results

### Interspecies patch-type sequence identities

Across 91 pairwise comparisons summarized in Tables 1, 2 (selected data), S1 and S2 (complete data), consistent patch-type sequence identities of around 45% were observed, even between sequences of distantly or un-related genomes. These patterns were identified using both Vector NTI Advance® 11 and BLAST, with strong concordance between tools, underscoring the robustness of the observed patterns. Comparisons among closely related *SARS-CoV-2* sequences, such as *Wuhan-Hu-1* aligned with *Omicron* subvariants *BA.1* and *BA.2*, yielded high identity levels ranging from 95 to 99%. In contrast, comparisons involving distantly related genomes, such as viral with human or plant sequences, consistently produced identities within the 42% to 48% range.

These patch-type sequence identities were evident across a broad phylogenetic spectrum, including organisms such as humans, plants, bacteria, and various viruses. Notably, even comparisons within the segmented *Influenzavirus* genome^29^—between hemagglutinin and neuraminidase coding segments—resulted in approximately 45% identity, reinforcing the ubiquity of these patterns across genomes. This recurring identity range suggests that patch-type matches are not solely the result of evolutionary conservation but also reflect a statistical property of the four-letter genetic system.

Tables 1, 2, S1 and S2 detail the alignment coordinates and accession numbers used in the comparisons. The only instances in which identity levels exceeded 89% occurred among human repeat elements such as *Alu* elements, consistent with their known sequence conservation. For all other interspecies alignments, patch-type identities consistently ranged between 41 and 48%. Tables 1, 2, S1 and S2 also list the GC-contents of all sequences investigated (see Sect. "Special Considerations for Mitochondrial DNAs").

Representative alignment screenshots (Figs. 1 and 2) illustrate the characteristic patchy structure of these identities: short runs of identical nucleotides interspersed with mismatches. These patch-type patterns extend across the full length of the shorter sequence in each comparison, not just within isolated regions as presented in screen shots. Due to space limitations, full-length alignments are presented in the Supplementary Figs. S1–S15. In these Figures, it is possible to scroll through the entire lengths of the alignments and find the identity patterns to spread along the alignments’ entire lengths.

To further quantify the morphology of these patterns, the frequency of contiguous identity stretches ranging from 1 to 10 nucleotides was analyzed (Figs. 4 and 5). These analyses revealed a preponderance of short matches, such as 1-mers and 2-mers, with declining frequency as match length increased. Notably, these patch-type patterns closely resemble those previously observed at integration junctions between host genomes and foreign DNA. Such junctions, reported in numerous studies^3–20^, suggest that patch-type identities may serve as recognition signals for illegitimate recombination events. Figures SA and SB under Supplemental Materials reproduce two such examples of patchy sequence identities at junction sites between integrated *Ad12* DNA and *hamster* cell DNA determined earlier in the senior author’s laboratory (7, 16, respectively).

Some of the entire alignments can be inspected under Supplemental Materials, Figs. S1–S15.

### Best-window reanalysis

To confirm that the patch-type regime persists when the highest-identity window is located automatically, we re-ran three paired comparisons (*SARS-CoV-2 Wuhan Hu-1* vs. *Adenovirus 2*, *Mycobacterium tuberculosis*, and *Oryza sativa* chromosome 8) with the best‑segment overlap pipeline described in Methods. Using a 10 kb *SARS-CoV-2* segment (nucleotides 9,952–19,951) we identified the single subject window that maximized percent identity in each partner genome and then aligned the same segment against 100 composition-matched Markov 1 shuffles of that window. Real best windows yielded 45–49% identity, whereas shuffled controls clustered near 17–18%. We extended this analysis to additional genomes spanning a wider GC range—*Lambda phage*, *T7 phage*, the *Arabidopsis thaliana* chloroplast genome, and the human mitochondrial genome—using the same 10 kb *SARS-CoV-2* segment (the mt-DNA comparison used nucleotides 3,285–13,284 to avoid the control region). Real overlaps for these genomes also fell between 45 and 49%, whereas their shuffled counterparts ranged from ~ 16% to 27% (Table S4). Pooling all seven real values against the seven shuffle cohorts (50 null alignments per pair) yielded a Kruskal–Wallis’s statistic of approximately 535.2 (df = 7, p < 10⁻^15^ Fig. 3), underscoring the clear separation between biological overlaps and composition-matched controls (Fig. 3). Kolmogorov–Smirnov tests^28^ comparing the observed run-length distributions to their geometric expectation were highly significant (D = 0.52–0.59, p = 10⁻^3^–10⁻^5^), demonstrating that the contiguous match structure of real alignments departs sharply from random expectations even when compositional bias is preserved. Base-class contingency tables and logistic regression further revealed strong enrichment of AT ↔ AT matches (odds ratios 5–8, q < 10⁻^25^⁰) and CG ↔ CG matches (odds ratios 3–6), whereas mixed pairs contributed negligibly. For each comparison we also re-ran the search against the reverse complement of the partner genome. RC identities (46.2–48.5%) closely matched the forward overlaps, confirming that the observed patches are not strand-specific artifacts. These results mirror the patch-type morphology observed in Figs. 1–2 and establish that the ~ 45% identity plateau is recapitulated by an automated window-search strategy that reports exact genomic coordinates for each best overlap.

### Special considerations for mitochondrial DNAs

Sequence identities between mitochondrial DNAs of *Homo sapiens* and sequences of *Chlorocebus* or *Latimeria columnae* have been shown to lie between ~ 60 to 80% (Table S1 and in Fig. S6). The most likely interpretation of this difference to most of the other sequence comparisons can be found in the classical evolutionary conservation of mitochondrial DNA.

### Do differences in GC-contents of alignment partners affect identity patterns?

The question has come up and the possibility exists that differences in the GC contents of the aligned sequence partners might alter the identity patterns of around 45%. In Tables 1, 2, S1 and S2, the outcome of such comparisons has been presented. Within the GC ranges occurring in the studied alignments, there is no evidence that differing GC contents between the compared sequences would alter the ~ 45% identity patterns, except in a few cases that have been described below. As stated in the previous paragraph, mitochondrial DNAs show higher identity values (Table S1 and in Fig. S6). Moreover, the *Wuhan* sequence of *SARS-CoV-2* and its recently evolved variants during the pandemic have very closely related sequences (Tables 1 or S1 and in Fig. S6).

In Tables 1, 2 and S2, S3, S4 sequence identity patterns have been related also when considering differences in GC contents of the aligned sequence partners (shown in column 2 in these Tables). See also Sect. "Best-window reanalysis".

Notable differences in comparisons have been found only in instances shown in Table 3

Specifically, we compared the *SARS-CoV-2 Wuhan-Hu-1* genome and three divergent reference genomes: *Mycobacterium tuberculosis*, *Plasmodium falciparum* and *Ad2*. In each case, the *SARS-CoV-2* genome was aligned to the full-length target genome using global alignment followed by local realignment within the best-matching window. As expected, the overall identities in random regions were low (26.5% and 30.5% for *M. tuberculosis* and *P. falciparum*, respectively), but in the case of *Ad2*, we identified a 21-nucleotide region of 95.2% identity, far exceeding the expected binomial match probability of 26.5% calculated from base frequencies (Table 3).

This result supports the idea that while patch-type identities are common and statistically predictable, some regions exhibit localized identity spikes that exceed background expectations. Such regions—especially when involving two unrelated viral genomes—may represent potential hotspots for illegitimate recombination. These findings reinforce the broader conclusion that patch-type identities arise from the inherent structure of nucleotide sequence space, but can also be exploited by biological systems during recombination, integration, or viral evolution.

Alignments with genomes differing in GC content from *SARS-CoV-2 Wuhan-Hu-1* . Unlike compositionally matched shuffled sequences, these mismatched natural genomes yield identity values close to the theoretical binomial expectation (~ 26%).

Automated best-window analyses confirmed these observations. When we restricted *SARS-CoV-2 Wuhan-Hu-1* to a 10 kb segment and asked the algorithm to find the single highest-identity window in *Adenovirus 2* (51% GC), *Mycobacterium tuberculosis* (66% GC), and *Oryza sativa* chromosome 8 (43% GC), the resulting overlaps remained in the 45–49% range even though the partner GC contents differed by as much as 28%. Compositional effects became apparent only when we removed biological structure entirely: dinucleotide-preserving shuffles of the optimal windows aligned back to *SARS-CoV-2* at ~ 18% identity, close to the binomial expectation (~ 26%) for random sequences with 38% GC. Thus, GC mis-matches alone do not collapse the patch-type plateau; instead, they set the baseline from which occasional high-identity “hotspots” (e.g., the 95.2% region in *Adenovirus 2*) emerge The same behavior held for *Lambda phage*, *T7 phage*, the *Arabidopsis thaliana* chloroplast genome, and human mitochondrial DNA: despite GC contents ranging from ~ 37% to 49%, their best overlaps with the 10 kb *SARS-CoV-2* segment remained within 45–49% identity, whereas shuffles of the optimal window dropped to 16–27% (Table S5).

### Quantitation of sequence identity patterns

To investigate the structure and distribution of patch-type sequence identities in greater detail, we quantified the frequency of contiguous identical nucleotide stretches ranging from 1 to 11 base pairs. Figures 4 and 5 summarize these patterns using bar graphs for specific genome comparisons as indicated in the Figures.

In all cases, short identical sequences (e.g., 1-mers and 2-mers) were the most frequently observed, with 1-mers occurring more than 4,000 times and 2-mers over 2,000 times per alignment. The frequency of longer identical runs decreased progressively, with 10-mers and 11-mers appearing only rarely. These trends were consistent across real sequences and mirrored in control simulations (Figs. 7 and 8), suggesting a geometric-like distribution. The quantitative distribution of match lengths reinforces the patchy nature of sequence identity observed in both natural alignments and in control alignments (see Sect. "Details of patch-type identities").

### Similar sequence identity patterns in coding and non-coding regions

To assess whether patch-type sequence identities differ between coding and non-coding genomic regions, we performed separate analyses in alignments between the *SARS-CoV-2 Wuhan-Hu-1* genome and segments of the human genome. Specifically, alignment with a non-coding region of chromosome 13 resulted in an overall sequence identity of 49.4% (Fig. 5A), while comparison with a coding region of chromosome 1 yielded a sequence identity of 44.0% (Fig. 5C). Despite these minor differences, the characteristic patch-type identity pattern was observed in both cases. These findings suggest that such identities are not confined to any specific functional genomic element and occur ubiquitously across both coding and non-coding regions.

### Details of patch-type identities

This section highlights representative examples of patch-type sequence identities observed between the 29,903-nucleotide *SARS-CoV-2 Wuhan-Hu-1* genome and various segments of the human genome (Fig. 6, panels C–O). These alignments illustrate the characteristic pattern of short stretches of identical nucleotides interspersed with non-identical regions. Patch-type identities were distributed along the full length of each alignment, further reinforcing their non-localized, intrinsic nature. In an alignment of the sequence of the *SARS-CoV-2 Wuhan-Hu-1 Omicron* variant *BA.2.86,* “*Pirola*” with the sequence of wildtype *SARS-CoV-2 Wuhan-Hu-1*, two 2- and two 4-patch-type identities were detected [our unpublished results].

The recurrence of such identity patterns in functionally important, mutationally dynamic regions underscores their potential relevance for recombination and adaptation. This raises the possibility that *SARS-CoV-2* may engage in sequence exchange not only with related viral genomes but also with host-derived nucleic acid fragments present in infected cells. Recombination events among positive-sense RNA viruses are well documented^31–40^, and further research is needed to investigate whether host-derived patch-type identities contribute to such processes in *SARS-CoV-2*. There is also direct evidence for recombination between RNA viral and host cell genomes^40^.

### Control experiments using randomized and shuffled sequences

To determine whether the observed patch-type identities are unique to biological sequences or reflect a more general property of nucleotide composition, we conducted a series of control experiments using randomized, shuffled, and composition matched sequences. Specifically, we generated 500 independently shuffled versions of the *SARS-CoV-2 Wuhan-Hu-1* and *Adenovirus type 12* (*Ad12*) genomes by permuting the nucleotide order while preserving the overall sequence length and base composition.

Pairwise alignments of each shuffled sequences with its original (unshuffled) counterpart revealed identity levels ranging from 42 to 46%, closely mirroring those seen in alignments between natural sequences. These identity levels were robust across five independently shuffled replicates per genome and remained consistent when aligning the shuffled genomes to human DNA regions of similar length and base composition.

Table 4 summarizes these control comparisons and shows that patch-type identities are preserved even in randomized sequences. These findings reinforce the hypothesis that patch-type identities are not necessarily the result of evolutionary relatedness or conserved functional elements, but rather reflect an inherent statistical property of the four-letter genetic system. Specifically, when sequences share overall base composition—especially in GC content—even randomly ordered nucleotides exhibit statistically enriched short identity tracts under global alignment conditions.

The results of all sequence comparisons under consideration of differing GC-contents between the aligned sequences have been included in Tables 1, 2, S2, S3 and S4 as described in Sect. "Special Considerations for Mitochondrial DNAs" above.

**Fig. 9:**
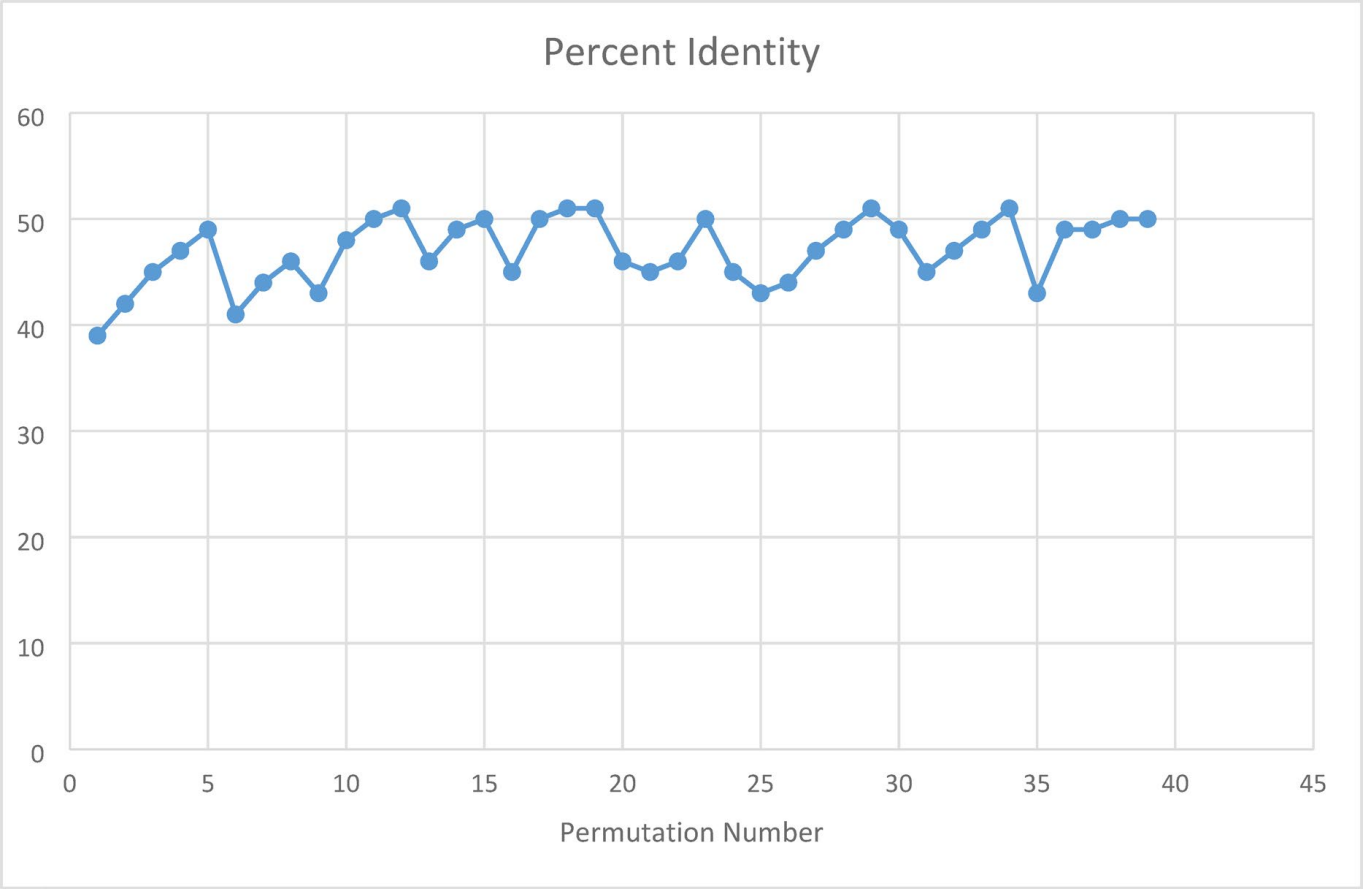
Percent identity between the *Adenovirus Type 2* genome (Accession Number NC_J01917.1) and the *SARS-CoV-2 Wuhan-Hu-1* genome (Accession Number NC_045512.2) computed across 40 different parameter permutations using the Needleman-Wunsch algorithm^26^ as implemented in the BLAST tool. Each permutation reflects a distinct combination of alignment scoring parameters (match, mismatch, gap penalties). Details of the parameter configurations and alignment procedures are provided in Materials and Methods Sects. "Programs Applied for Pairwise Alignments" and "Sequence Alignment Strategy".

In addition to these shuffling experiments, we also performed global alignments of fully synthetic sequences generated through uniform base sampling (Figs. 7 and 8), as well as parameter-permuted alignments using the Needleman-Wunsch algorithm^26^ (Fig. 9). Across all conditions, identity levels remain consistent in the 42% and 46% range. These additional controls further support the conclusion that patch-type sequence identities are a statistical property of sequence composition and not dependent on biologic structure or evolutionary relatedness. For details, please see legends to Figs. 7–9.

DNA alignments between *SARS-CoV-2 Wuhan-Hu-1* and shuffled DNAs from *Mycobacterium tuberculosis* [Table S3] as well as DNA alignments between shuffled DNA from *SARS-CoV-2 Wuhan-Hu-1* and shuffled DNA from *Mycobacterium tuberculosis* [Table S4] have now been performed. The same sequence identity results of between 41 and 42% have been obtained.

### Patch-type sequence identities as potential signals for recombination

The data presented in this study, along with numerous published reports^3–20^ and Figs. SA and SB illustrating previously published work from this laboratory^7,16^, support the hypothesis that patch-type nucleotide sequence identities may serve as recognition signals for illegitimate recombination or sequence exchange reactions. These patch-type identities, characterized by short interspersed runs of identical nucleotides separated by mismatches, closely resemble the patterns previously observed at known DNA integration junctions, including those involving *Adenoviruses*, *Retroviruses*, *Transposons*, and exogenous plasmid DNA.

Notably, we observed similar patch-type identity patterns in the *SARS-CoV-2 Wuhan-Hu-1* genome and its variants, particularly in regions of functional importance and high mutational variability, such as the spike protein-coding sequence (data not shown). These localized regions of elevated identity, which deviate from the expected baseline defined by composition-matched random controls, may represent molecular substrates for recombination. Given the critical role of the spike protein in viral entry, immune escape, and vaccine responsiveness, even subtle recombination events facilitated by patch-type sequence identities could drive significant phenotypic change and rapid evolutionary adaptation.

To explore this further, we used an adaptive windowing and alignment strategy to scan the *SARS-CoV-2 Wuhan-Hu-1* genome against viral and host genomes (*Mycobacterium tuberculosis*, *Plasmodium falciparum and Ad2*), identifying the most identity-rich regions. In comparisons with *Ad2*, for instance, the highest-identity region in the *SARS-CoV-2* genome achieved 95.2% identity over a 21-nucleotide window—substantially above the background expectation of ~ 26.5% based on base composition alone (Table 3). This result, while rare, exemplifies the emergence of identity hotspots that could serve as candidate substrates for sequence exchange in vivo (see Sect. "Special Considerations for Mitochondrial DNAs").

The presence of these identity-rich patches in both natural and shuffled alignments suggests that recombination signals may arise not only from biologically conserved regions, but also from the statistical structure of the four-letter genetic system itself. The findings underscore the possibility that the genome’s statistical architecture has been evolutionarily co-opted to support molecular mechanisms such as recombination, integration, and transposition. These mechanisms may, in turn, contribute to genome remodeling, viral plasticity, the emergence of novel viral variants. This intersection between randomness and function provides a new perspective on how intrinsic sequence properties may facilitate genome evolution across both viral and host systems.

### Statistical properties of patch-type identities

To better understand the statistical nature of patch-type sequence identities, we performed systematic simulations comparing natural, shuffled, and synthetic random sequences. Global alignments across all conditions revealed striking consistency in identity levels, typically ranging between 42 and 48%, regardless of sequence origin or base order (Fig. 7). These findings underscore that patch-type identities are a predictable outcome of sequence space defined by a four-nucleotide alphabet.

Figure 7A compares identity distributions from homologous sequences, non-homologous biological sequences, shuffled real genomes, and synthetic random sequences. As expected, homologous pairs displayed the highest identity values. However, identity levels among shuffled and random sequences closely mirrored those of the biological comparisons. Figure 7B further confirms the stability of this pattern across different sequence lengths, with simulated random sequences of 3,500 and 35,000 nucleotides showing nearly identical identity distributions.

To examine the structure of these matches in more detail, we quantified the frequency of contiguous identical stretches from 1-mers to 11-mers across all sequence types (Fig. 8). The frequency profiles were nearly identical among natural, shuffled, and synthetic sequences, consistently showing a steep decline in match frequency as segment length increased. These consistent profiles across diverse sequence types and lengths indicate that patch-type identities follow a statistically predictable pattern, arising from the basic combinatorics of the four-nucleotide system that has likely played a decisive role in selecting the four-letter system for all of biology.

We also tested the robustness of these findings by varying alignment parameters using the Needleman-Wunsch global alignment algorithm^26^. Figure 9 displays identity levels obtained from aligning the *SARS-CoV-2 Wuhan-Hu-1* genome with that of *Adenovirus type 2* across 40 different scoring configurations. While minor fluctuations were observed, identity levels remained well within the 42–46% range, indicating that the observed patterns are not sensitive to alignment parameters and further supporting their statistical basis.

Collectively, these analyses demonstrate that patch-type identities are a fundamental property of nucleotide sequence architecture. They persist across different sequence types, alignment methods, base compositions, and even alignment scoring schemes. These findings provide a robust statistical framework for interpreting the widespread presence of patch-type identities and their biological implications in genome dynamics and recombination.

In both panels, the frequency profiles are similar across categories, with total identity levels clustering around 48%, indicating that the patch-type identity pattern is maintained regardless of sequence origin or composition.

## Discussion

### Patch-type identities an intrinsic statistic property of the genetic alphabet

A central finding of this study is the consistent emergence of patch-type sequence identities—short contiguous stretches of matching nucleotides interspersed with mismatches—across a broad spectrum of interspecies comparisons. These patterns appeared not only in biologically unrelated sequences, such as viral versus human or plant genomes, but also in shuffled and fully randomized control sequences. The persistence of these patterns, even in sequences devoid of biological structure, suggests that patch-type identities are a statistical consequence of the four-letter nucleotide alphabet and has been selected during evolution probably for optimal biological function.

All control experiments demonstrated that alignments between randomized or composition-preserving shuffled sequences consistently yielded identity levels between 42 and 48%, comparable to the identity observed in many interspecies alignments of natural sequences. Aligning real genomes with slightly dissimilar base compositions and functional constraints leads to the same result, indicating that biological structure can actually suppress some of the stochastic identity expected from random symbol distributions. This counterintuitive result reinforces the conclusion that patch-type identities are not merely tolerated but are a statistical expectation when aligning any two sequences drawn from a four-letter alphabet with biased base frequencies.

### Evolutionary and functional implications of patch-type identities

Although patch-type sequence identities appear to arise as a statistical consequence of the four-letter genetic alphabet, their persistence across natural genomes and association with known biological processes suggest they may have been co-opted as functional elements through evolutionary time. Numerous studies have reported the presence of patch-type sequence identities at sites where foreign DNA integrates into host genomes^3–20^. These integration junctions frequently display interspersed stretches of nucleotide identity, consistent with the patch-type pattern observed in the current study (Figs. SA and SB).

**Fig. 10:**
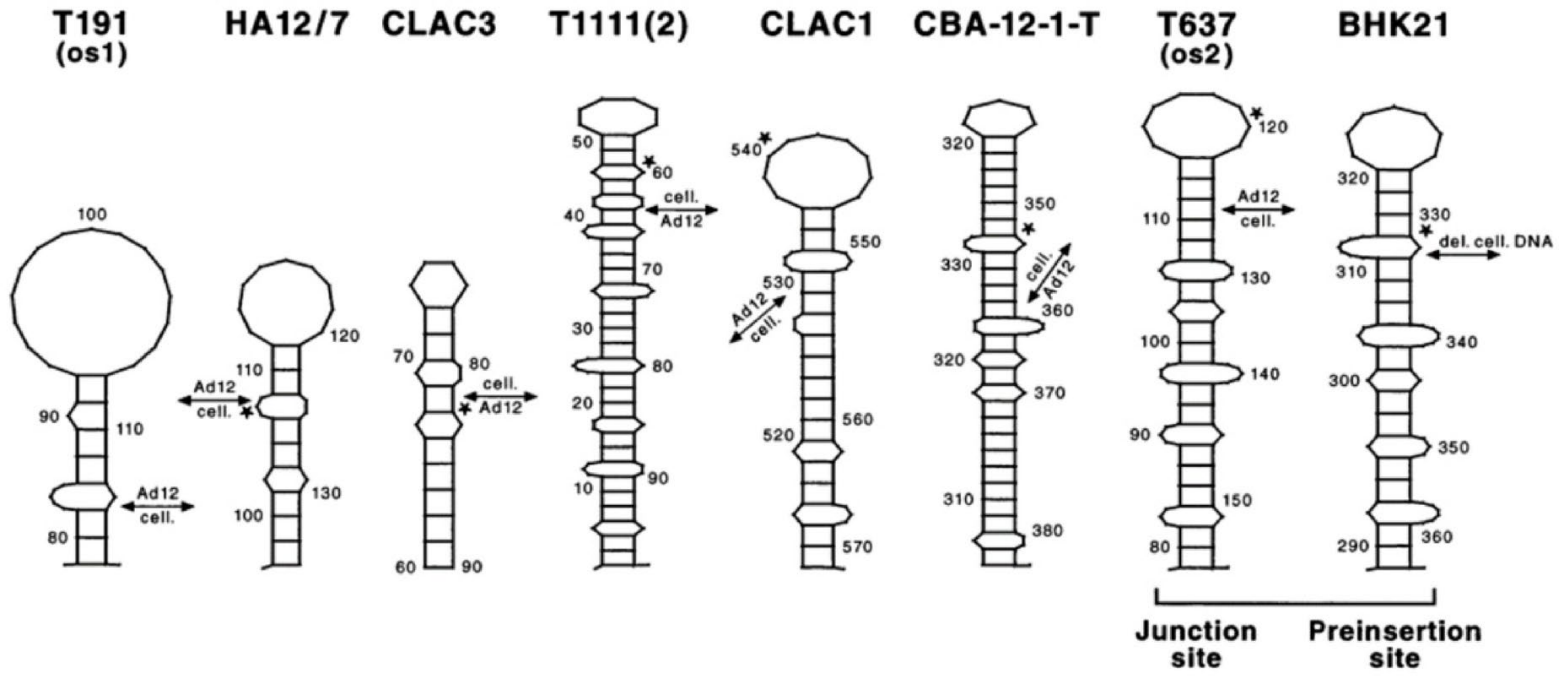
Stem-loop structures identified at junction sites between *Ad12* DNA and host cellular DNA in multiple *Ad12*-transformed hamster cell lines and *Ad12*-induced hamster or mouse tumor cell lines. The computer-generated graphs present stem-loop designs for *Ad12* insertion sites in the transformed or tumor cell lines T191, HA12/7, CLAC3, T1111, CLAC1, CBA-12–1-T, and T637 and the *Ad12* pre-insertion site in the parental BHK21 cells [for details, see reference 16]. In all structures, the virus-cell junctions were designated by double-headed arrows. Possible topoisomerase I recognition sites (asterisks)^41^ were indicated as well as nucleotide numbers for the individual nucleotide sequences. Stem-loop configurations were also reported for a number of non-homologous recombination events, e.g., the insertion site of unique and repetitive DNA fragments into the aprt locus in the hamster genome or a hotspot for novel amplification joints in *Alu*-like repeats and other structures or for virus-cell DNA integration sites [references 3–20]. Additionally, it has been shown that multiple *Adenovirus* integration sites are apparently randomly distributed over host cell genomes^18^. These authors also report illegitimate recombination to have led to *Adenovirus* DNA integration.

Such recurring patterns raise the possibility that patch-type identities facilitate illegitimate recombination events by serving as minimal recognition sites for cellular enzymes involved in integration, recombination, or transposition. Enzymes such as integrases, recombinases, or topoisomerases may use these short identity stretches to stabilize interactions during insertion or exchange, particularly in non-homologous recombination where extended identity is not required. For example, Fig. 10 shows previously characterized junctions between *Adenovirus type 12* (*Ad12*) and host cellular DNA in transformed and tumor cell lines, where patch-type identity regions coincide with stem-loop structures and putative topoisomerase I recognition motifs^16,41^.

Our new simulations further support the functional plausibility of patch-type identities as recombination substrates. When we compared the *SARS-CoV-2* genome to other viral and microbial genomes, we identified specific regions with identity levels exceeding random expectations that suggest some patch-type regions being under selection or serving as hotspots for sequence exchange. Notably, such identity-rich zones were observed in the *SARS-CoV-2* spike protein coding region, which is known for its high mutation rate and evolutionary adaptability (data not shown). Given the critical role of this region in host cell entry and immune escape, patch-type sequences within it may facilitate recombination with either related coronaviruses or host nucleic acid fragments present in infected cells.

### Statistical constraints as evolutionary opportunities

The consistent presence of patch-type sequence identities across natural, shuffled and fully randomized genomes reinforces the conclusion that these patterns are an intrinsic statistical feature of the four-letter nucleotide alphabet. Far from being biologically meaningless, this property may have provided a crucial scaffold for the emergence and diversification of early genomes. In primordial genetic systems that still lacked sophisticated recombination enzymes or high-fidelity replication machinery, short, chance-based partial identity patches could have facilitated recombination, rearrangement, and the incorporation of foreign sequences, acting as a scaffold for molecular innovation.

These local stretches of sequence identity may have served as natural anchors or minimal substrates for early recombination, sequence joining, or template switching. Even weak interactions, stabilized by just a few matching nucleotides, could have facilitated the integration of foreign or parasitic sequences, including early mobile elements, into host-like frameworks. In this way, what began as a stochastic consequence of limited sequence complexity may have enabled modular genome construction.

Over evolutionary time, such chance-driven integrations would have been filtered by natural selection, favoring recombinant products that preserved or enhanced biological function. The genomic architectures we observe today, rich in mosaic structures, repeats, and horizontally acquired segments, may be the long-term legacy of these early patch-mediated recombination events. Thus, a basic statistical constraint of nucleotide composition was not a barrier to genome evolution, but rather a source of opportunity, driving diversity and adaptability across the tree of life.

### Implications for viral evolution and SARS-CoV-2

The findings presented in this study may have potentially important implications for understanding the mechanisms underpinning RNA virus evolution and adaptability of RNA viruses, such as *SARS-CoV-2*. Our analyses revealed consistent patch-type sequence identities between the *SARS-CoV-2* genome and a diverse array of non-homologous sequences, including those from human, bacterial, plant, and microbial genomes. These results suggest that patch-type identities may serve as potential scaffolds for recombination, even in the absence of long contiguous homology.

In *SARS-CoV-2*-infected cells, viral RNA coexists with host RNA and potentially with degraded host DNA fragments. In such a context, short stretches of identity between viral and host sequences—arising as a statistical property of nucleotide composition—may facilitate template switching, illegitimate recombination, or the incorporation of host-derived sequence fragments. These interactions may occur during RNA replication, reverse transcription (in the case of co-infection with retroelements), or other nucleic acid processing events in the host cell. Extensive gene transfer between viral and host genomes has recently been reported^40^.

Given the high mutation rates and recombination potential of positive-sense RNA viruses, even rare sequence exchanges may have impacts on viral fitness. Patch-type identity regions could enable *SARS-CoV-2* to acquire new sequence motifs that alter key functional domains, such as those involved in spike protein structure, receptor binding, or immune evasion. In this way, a fundamentally statistical property of the genetic system in all biology may contribute to real-world evolutionary dynamics, helping to explain the rapid emergence of novel *SARS-CoV-2* variants with enhanced transmissibility and/or altered pathogenicity.

### Limitations and future directions

While this study provides robust evidence that patch-type sequence identities are a statistically intrinsic property of the four-letter genetic system and are consistently observed across diverse genomes, several important questions remain unresolved. Most notably, the molecular mechanisms by which these patch-type identities might facilitate recombination, integration, or other genomic rearrangements are still poorly understood. Experimental studies will be required to determine whether enzymes involved in homologous or illegitimate recombination—such as integrases, recombinases, or topoisomerases—specifically recognize or act upon regions exhibiting patch-type identity configurations. Biochemical or structural studies focused on enzyme–substrate interactions at patch-type regions would be particularly illuminating.

In addition, while our initial analyses showed that patch-type identities appear with similar frequency in both coding and non-coding regions, more genome-wide surveys are warranted. Such analyses may reveal subtle biases that relate to sequence function, regulatory architecture, or chromatin organization. For example, patch-type identities may be enriched in intergenic regions, retrotransposon sequences or enhancers which potentially influence genome accessibility, epigenetic marking, or susceptibility to recombination events.

The relationship between patch-type identities and repetitive or mobile elements such as *Alu* sequences, *LINEs*, or *endogenous retroviruses* deserves deeper investigation. These elements are known to drive genome evolution through duplication and insertion events, and their structure may be particularly compatible with the recombination-prone properties of patch-type identities. Exploring whether these statistical patterns contribute to the insertion preferences or structural motifs of mobile genetic elements could uncover new dimensions of genome plasticity.

Taken together, future work should combine computational, experimental, and comparative approaches to clarify whether patch-type sequence identities play a functional role beyond their statistical inevitability—and whether evolution has leveraged these patterns as molecular tools for genomic innovation.

### Conclusion

Patch-type nucleotide sequence identities represent a fundamental statistical property of the four-nucleotide genetic alphabet. Although these patterns are not exclusive to biological sequences, their widespread presence across diverse genomes suggests they may have been co-opted during evolution to serve functional roles. By acting as potential substrates for recombination, integration, or other genomic re-arrangements, patch-type identities may have contributed to the generation of genetic diversity and the emergence of complex genome architectures. In modern genomes, including those of RNA viruses such as *SARS-CoV-2*, these sequence features may continue to influence genomic plasticity and adaptation. Their presence in mutationally dynamic regions, such as the spike protein-coding sequence, raises the possibility that patch-type identities influence viral recombination, adaptation, and evolution in real time. Together, these findings highlight the importance of understanding how statistical properties intrinsic to the genetic system intersect with evolutionary and functional processes to shape the genomic landscape today. By bridging fundamental sequence properties with biological outcomes, this study provides a framework for exploring how randomness at the nucleotide level can give rise to order and complexity across the tree of life.

## Supplementary Information

Below is the link to the electronic supplementary material.

Supplementary Material 1

Supplementary Material 2

Supplementary Material 3

Supplementary Material 4

Supplementary Material 5

Supplementary Material 6

Supplementary Material 7

Supplementary Material 8

Supplementary Material 9

Supplementary Material 10

Supplementary Material 11

Supplementary Material 12

Supplementary Material 13

Supplementary Material 14

Supplementary Material 15

Supplementary Material 16

Supplementary Material 17

Supplementary Material 18

Supplementary Material 19

Supplementary Material 20

Supplementary Material 21

Supplementary Material 22

## Data Availability

Data from GISAID ^21^ and as listed under Weber et al., 2022^30^. All *SARS-CoV-2* variant sequences were obtained from the GISAID database (https://gisaid.org/) and are listed in legend to Table S1 with the corresponding GISAID accession numbers. All other genome sequences of viruses, bacteria, plants, and mammalian species are from the NCBI nucleotide database (https://www.ncbi.nlm.nih.gov/nucleotide/) and are listed with their corresponding NCBI accession numbers in Tables 1, 2, and Tables S1–S4 under Supplemental materials.
