# Supplementary material for: Patch type nucleotide sequence identities between genomes from many different species facilitate illegitimate recombination
Source: Sci Rep. 2026 Mar 30;16:10524. doi: 10.1038/s41598-026-44124-0 (PMC13035915; doi:10.1038/s41598-026-44124-0)
Supplement: Supplementary file 1 — Supplementary Material 1 [file 41598_2026_44124_MOESM1_ESM.pdf]

Section 1

```
                                        (1) 1        10        20        30        40        50        60        70        80        90
            HERV- E (AB062274.1)        (1) GGGCCCGCAGACACTC-AGCTAAAGATGTCTCATAGGTTGTCCTTGCAGCTAAGTGGGGCC--ATGAGACTAG-----GCTT-TAACCAG
SARS-CoV-2 (region 17170 - 29465)       (1) GCCGCTGTTGATGCACTATGTGAGAAGGCATTAAAATATTTGCCTATAGATAAATGTAGTAGAATTATACCTGCACGTGCTCGTGTAGAG
```

Section 2

```
                                       (91) 91       100       110       120       130       140       150       160       170       180
            HERV- E (AB062274.1)       (82) TGGGCTGAGAG--TTAAAGTGATTTTTGC-------CATTCTGTTTTTAGGAATGGATGTGTCT-GCCTGTG------GCAGATTATATT
SARS-CoV-2 (region 17170 - 29465)      (91) TGTTTTGATAAATTCAAAGTGAATTCAACATTAGAACAGTATGTCTTTTGTACTGTAAATGCATTGCCTGAGACGACAGCAGAT-ATAGT
```

Section 3

```
                                      (181) 181      190       200       210       220       230       240       250       260       270
            HERV- E (AB062274.1)      (156) TTTCAAAGATGACCACAAAAATATCTCCTATC-TCATGTGTGATTCTACAGTGGGGTCTATGTCCCCTCTTCTTGAATGTGTGTGCACTT
SARS-CoV-2 (region 17170 - 29465)     (180) TGTCTTTGATGAAATTTCAATGGCCACAAATTATGATTTGAGTGTTGTCAATG----CCAGATTACGTGCTAAGCACTATGTGTACATTG
```

Section 4

```
                                      (271) 271      280       290       300       310       320       330       340       350       360
            HERV- E (AB062274.1)      (245) GTGACTGCTTTGACTAACAGAGTATGGGGTAGGATGCCATGTGACTTCTGAGGCTGGGTCACGGAAAG--CAATT--GTTATAAGTTAAA
SARS-CoV-2 (region 17170 - 29465)     (266) GCGACCCTGCTCAATTACCT-GCACCACGCACATTGCTAACTAAGGGCACA--CTAGAACCAGAATATTTCAATTCAGTGTGTAGACTTA
```

Section 5

```
                                      (361) 361      370       380       390       400       410       420       430       440       450
            HERV- E (AB062274.1)      (331) TTGCATGTCCCCGCCCCCCACCAAATTCATATGATGCAGTCTTAAT--TCCTGCTGCC-----TCCGAATGTGACCTTATTTAGA--AAG
SARS-CoV-2 (region 17170 - 29465)     (353) TGAAAACTATAGGTCCAGA--CATGTTCCTCGGAACTTGTCGGCGTTGTCCTGCTGAAATTGTTGACACTGTGAG-TGCTTTGGTTTATG
```

Section 6

```
                                      (451) 451      460       470       480       490       500       510       520       530       540
            HERV- E (AB062274.1)      (412) AGAATCATCGTGGATCTAATTAGTTAAGATTAGGTCA-----TACTACAGTGTG-TGAGACCCTAATCCCGTAGGACTGGTCTT-----A
SARS-CoV-2 (region 17170 - 29465)     (440) ATAATAAGCTTAAAGCACATAAAGACAAATCAGCTCAATGCTTTAAAATGTTTTATAAGGGTGTTATCACGCATGATGTTTCATCTGCAA
```

Section 7

```
                                      (541) 541      550       560       570       580       590       600       610       620       630
            HERV- E (AB062274.1)      (491) TAAAAAGGGTA-ATTTGGGCACAGAAATAGACATGCATAGAGGGAAGATGATGTGAAGAAACACAGCTCAGACACCCATCTTCAAGGCAA
SARS-CoV-2 (region 17170 - 29465)     (530) TTAACAGGCCACAAATAGGCGTGGTAAGAGAATTCCTTACACGTAACCCTGCTTGGAGAAAAGCTG-TCTTTATTTCACCTTATA---AT
```

Section 8

```
                                 (631) 631      640       650       660       670       680       690       700       710      720
HERV- E (AB062274.1)             (580) GGAGAGAGGCCTGGAACAGATCAGAAGGAATCAACCCTGCCAACACCTTCATTTCAGAC-TGCTAGCCTCCAGAAC-TGTCGTTTAAACA
SARS-CoV-2 (region 17170 - 29465)(616) TCACAGAATGCTGTAGCC--TCA-AAGATTTTGGGACTACCAACTCAAACTGTTGATTCATCACAGGGCTCAGAATATGACTATGTCATA
```

Section 9

```
                                 (721) 721      730       740       750       760       770       780       790       800      810
HERV- E (AB062274.1)             (668) CCCAGTCTGTGACTCTTTACTATGGCAGTATGGTAGGAGACCACCACTTCTCCTGTTGTCCTTCCCAGTTTCTCCCCAACCTCCCCTTTT
SARS-CoV-2 (region 17170 - 29465)(703) TTCACTCAAAC-CACTGAAACAGCTCACTCTTGTAATGTA--AACAGATTTAATGTTGCTATTACCAGAGCAAAAGTAGGCATAC--TTT
```

Section 10

```
                                 (811) 811      820       830       840       850       860       870       880       890      900
HERV- E (AB062274.1)             (758) CCCTAGTTTATAAGACAG-----GAGAAAAGGGAGAAAGCAAAAAGT-TGGAAAGAAACAGAAGTAA----GATAAATAGCTAGACGACC
SARS-CoV-2 (region 17170 - 29465)(788) GCATAATGTCTGATAGAGACCTTTATGACAAGTTGCAATTTACAAGTCTTGAAATTCCACGTAGGAATGTGGCAACTTTACAAGCTGAAA
```

Section 11

```
                                 (901) 901      910       920       930       940       950       960       970       980      990
HERV- E (AB062274.1)             (838) TTG--GCGCCACCACCTGGCCCTGGTGGTTAAAATGATAATAATATTAACCCCTG-------ACCAAAACTACTG-GTGTTATCTGTAAA
SARS-CoV-2 (region 17170 - 29465)(878) ATGTAACAGGACTCTTTAAAGATTGTAGT-AAGGTAATCACTGGGTTACATCCTACACAGGCACCTACACACCTCAGTGTTGACACTAAA
```

Section 12

```
                                 (991) 991      1000      1010      1020      1030      1040      1050      1060      1070     1080
HERV- E (AB062274.1)             (918) TTCCAGAC--A----TTATATGA-GAAAGCACTGTAAAACTTTTTGTACTGTTAGCTGATGTAGGTAGCCCCCAGTCACGTTCCTCACAC
SARS-CoV-2 (region 17170 - 29465)(967) TTCAAAACTGAAGGTTTATGTGTTGACATACCTGGCATACCTAAGGACATG--ACCTATAGAAGACTCATCTCTATGATGGGTTTTAAAA
```

Section 13

```
                                 (1081) 1081     1090      1100      1110      1120      1130      1140      1150      1160     1170
HERV- E (AB062274.1)             (1001) TTACTTGA----TCTGTGATGACTCTTTCACGTAGA---CCC-CTTAGA-GTTGTAAGCCCTTCAAAGGGCTAGGAAT---TTCTTTTTC
SARS-CoV-2 (region 17170 - 29465)(1055) TGAATTATCAAGTTAATGGTTACCCTAACATGTTTATCACCCGCGAAGAAGCTATAAGACATGTAC-GTGCATGGATTGGCTTCGATGTC
```

Section 14

```
                                 (1171) 1171     1180      1190      1200      1210      1220      1230      1240      1250     1260
HERV- E (AB062274.1)             (1079) G--GGGAGCTCGGCT-CTTAAGATGC-GACTCTGCCGATGCTCCTGGCCGAATAAAAACCTCTTCCTTCTTTAATCCAGTGTCTG-----
SARS-CoV-2 (region 17170 - 29465)(1144) GAGGGGTGTCATGCTACTAGAGAAGCTGTTGGTACCAATTTACCTTTACAGCTAGGTTTTTCTACAGGTGTTAACCTAGTTGCTGTACCT
```

Section 15

(1261) 1261 1270 1280 1290 1300 1310 1320 1330 1340 1350

HERV- E (AB062274.1) (1160) AGGAGTTTTGTCTGCGACTCATCCTGCTACATTTCTTGGTTCCCTGCCTGGGAAGCGAGGTAATTGATGGACGGAGGGTCGAGGCAG-CC

SARS-CoV-2 (region 17170 - 29465) (1234) ACAGGTTATGT-TGATACACCTAATAATACAGATTTTTCCAGAGTTAGTGCTAAACCACCGCCTGGAGATCAATTTAAACACCTCATACC

Section 16

(1351) 1351 1360 1370 1380 1390 1400 1410 1420 1430 1440

HERV- E (AB062274.1) (1249) CCTTAGGTGGCTTAGGCCTGCCCTGTGGAGCATCCCTGTGGGGGACTCTGGCCAGCTTGAGTGACGCAGATCCTGAGACCACTCCCGGGT

SARS-CoV-2 (region 17170 - 29465) (1323) ACTTATGTA-CAAAGGACTTCCTTGGAATGTAGTGCGTATAAAGATTGTACAAATGTTAAGTGACACACTTAAAAATCTCTCTGACAGAG

Section 17

(1441) 1441 1450 1460 1470 1480 1490 1500 1510 1520 1530

HERV- E (AB062274.1) (1339) AGGCAATTGCCCCGGTGGAACGCCTCATC-AGAGCAGTGCGTGGCAGGCCCCTGTGGA----GGA--TCAACGCAGTGCTGAACACCGGG

SARS-CoV-2 (region 17170 - 29465) (1412) TCGTATTTGTCTTATGGGCACATGGCTTTGAGTTGACATCTATGAAGTATTTTGTGAAAATAGGACCTGAGCGCACCTGTTGTCTATGTG

Section 18

(1531) 1531 1540 1550 1560 1570 1580 1590 1600 1610 1620

HERV- E (AB062274.1) (1422) AAGGAATGGGCACTTGGAGTCCG---------GACATTTGAAACTTGGTAAGACTGGTCTTTGGAACTTGCCCAC-TCCATC-TGAGTGG

SARS-CoV-2 (region 17170 - 29465) (1502) ATAGACGTGCCACATGCTTTTCCACTGCTTCAGACACTT-ATGCCTGTTGGCATCATTCTATTGGATTTGATTACGTCTATAATCCGTTT

Section 19

(1621) 1621 1630 1640 1650 1660 1670 1680 1690 1700 1710

HERV- E (AB062274.1) (1501) AAGCGTGGCCTGATCACCCATGGCGTG----------CCTGTACCGGCACTTTGGTTTTTGTTTTTGACTTGACTTGAATTGC-----TT

SARS-CoV-2 (region 17170 - 29465) (1591) ATGATTGA--TGTTCAACAATGGGGTTTTACAGGTAACCTACAAAGCAACCATGATCTGTATTGTCAAGTCCA-TGGTAATGCACATGTA

Section 20

(1711) 1711 1720 1730 1740 1750 1760 1770 1780 1790 1800

HERV- E (AB062274.1) (1576) GATATTTTGGTTTTGGTTTTGACC---TGGCTTGGACTTCTGGA-TACTCGGATTTTGGTTTTGATTCTGGTTTGGTGTAAACTGAAAAA

SARS-CoV-2 (region 17170 - 29465) (1678) GCTAGTTGTGATGCAATCATGACTAGGTGTCTAGCTGTCCACGAGTGCTTTGTTAAGCGTGTTGA--CTGGACTATTGAATATCCTATAA

Section 21

(1801) 1801 1810 1820 1830 1840 1850 1860 1870 1880 1890

HERV- E (AB062274.1) (1662) GTGTGTGTGTGCCCTTTTTACCTGTTCTTTGTTCTGTGGTGTGT----GTGTGGTGTAAGCTTGGTGTTTTGTCTTGAGGAAACACGGGT

SARS-CoV-2 (region 17170 - 29465) (1766) TTGGTGATGAACTGAAGATTAATGCGGCTTGTAGAAAGGTTCAACACATGGTTGTTAAAGCTGCATTATTAGCA--GACAAATTCCCAGT

```
                                                                                                        Section 22
                                           (1891) 1891     1900      1910      1920      1930      1940      1950      1960      1970      1980
             HERV- E (AB062274.1)          (1748) CAGACACAAA----GTAAGCCTACTCCGCTAGGAACTGTGT---TGAAAAATTTTAAGAAGGGA-----TTTAATGGACA-CTATGGGGT
SARS-CoV-2 (region 17170 - 29465)          (1854) TCTTCACGACATTGGTAACCCTAAA--GCTATTAAGTGTGTACCTCAAGCTGATGTAGAATGGAAGTTCTATGATGCACAGCCTTGTAGT
                                                                                                        Section 23
                                           (1981) 1981     1990      2000      2010      2020      2030      2040      2050      2060      2070
             HERV- E (AB062274.1)          (1825) TACTATG---ACACCAGGGAA-ACTTAG----------A-----ACTTTGTGTGAAAT----AGATTG-----GCCAACATTAGAAGTGG
SARS-CoV-2 (region 17170 - 29465)          (1942) GACAAAGCTTATAAAATAGAAGAATTATTCTATTCTTATGCCACACATTCTGACAAATTCACAGATGGTGTATGCCTATTTTGGAATTGC
                                                                                                        Section 24
                                           (2071) 2071     2080      2090      2100      2110      2120      2130      2140      2150      2160
             HERV- E (AB062274.1)          (1887) GTTGGCCATCAGAAGGAAGCCTGGACAGGTCCCTTGTTTCTAAGGTATGGCACAAGGTAACTGGTAAGTCAGGACACTCAGACCAGTTCC
SARS-CoV-2 (region 17170 - 29465)          (2032) AATGTCGAT----AGATATCCTGCTAAT-TCCATTGTTTGTA-GATTTGACACTAG---AGTGCTATCTAACCTTAACTTGCCTGGTTGT
                                                                                                        Section 25
                                           (2161) 2161     2170      2180      2190      2200      2210      2220      2230      2240      2250
             HERV- E (AB062274.1)          (1977) CATGCATAGACACTTGGTTACAGCTGGTGCTAGAGCCCCCACAG------TGGCTAAGAGGGC---------AGGCAGCAGCAGTGCTAGT
SARS-CoV-2 (region 17170 - 29465)          (2113) GATGG-TGGCAGTTTGTATGTAAATAAACATGCATTCCACACACCAGCTTTTGATAAAAGTGCTTTTGTTAATTTAAAACAATTACCATT
                                                                                                        Section 26
                                           (2251) 2251     2260      2270      2280      2290      2300      2310      2320      2330      2340
             HERV- E (AB062274.1)          (2053) AG-CAAAGGGACAGATAG-CCAAGGAAGGATCCCGCTCCACC------GTCTGAGGGAAACCA---ACTCCTAAAGTTC--T----GTTC
SARS-CoV-2 (region 17170 - 29465)          (2202) TTTCTATTACTCTGACAGTCCATGTGAGTCTCATGGAAAACAAGTAGTGTCAGATATAGATTATGTACCACTAAAGTCTGCTACGTGTAT
                                                                                                        Section 27
                                           (2341) 2341     2350      2360      2370      2380      2390      2400      2410      2420      2430
             HERV- E (AB062274.1)          (2126) AACCCA----ACATCAGAAGATACATTGCAGGAGATGGCA--CCAGTGATCCCAG-TGGTGCC---CTCCCCTTACCAGG-------GAG
SARS-CoV-2 (region 17170 - 29465)          (2292) AACACGTTGCAATTTAGGTGGTGCTGT-CTGTAGACATCATGCTAATGAGTACAGATTGTATCTCGATGCTTATAACATGATGATCTCAG
                                                                                                        Section 28
                                           (2431) 2431     2440      2450      2460      2470      2480      2490      2500      2510      2520
             HERV- E (AB062274.1)          (2199) AGGGGCTCCCCACTTTTGATTCCACAGTGCTTGTACCTCCGCAAGACAAACATATCCTTAGGCCACC-CACAGTAGACAAGA-GAGGAGG
SARS-CoV-2 (region 17170 - 29465)          (2381) CTGGCTTTAGCTTGTGGGTTTACAAACAATTTGATACTTATAACCTCTGGAACACTTTTACAAGACTTCAGAGTTTAGAAAATGTGGCTT
```

Section 29

```
                                            (2521) 2521     2530      2540      2550      2560      2570      2580      2590      2600     2610
HERV- E (AB062274.1)                        (2287) TGAAGCCTCGGGAGAA---ACCCCTCCCTTGGCAGCTCGTTTAAGACACAAAACTGGGATACAAATGCCCCTGAGAGAGCAGCAGTATAC
SARS-CoV-2 (region 17170 - 29465)           (2471) TTAATGTTGTAAATAAGGGACACTTTGATGGACAACAGGGTGAAGT-ACCAGTTTCTATCATTAATAACACTGTTTACACAAAAGTTGA-
```

Section 30

```
                                            (2611) 2611     2620      2630      2640      2650      2660      2670      2680      2690     2700
HERV- E (AB062274.1)                        (2374) TGGTATAGATG-AGGATGGT---CACGTGGTGGAGAGGTGTGTTTTTGGGTACCA----GCCCTTCACCTCTGCCCACCTTCTCAACTGG
SARS-CoV-2 (region 17170 - 29465)           (2559) TGGTGTTGATGTAGAATTGTTTGAAAATAAAACAACATTACCTGTTAATGTAGCATTTGAGCTTTGGGCTAAGCGCAACATTAAACCAGT
```

Section 31

```
                                            (2701) 2701     2710      2720      2730      2740      2750      2760      2770      2780     2790
HERV- E (AB062274.1)                        (2456) AAAAG------CAATACCCCATCCTATACTGAAAAGCCACAAGCTCTAAT----TGATTTG---CTCCAAACT--ATTATCCAGACCCAT
SARS-CoV-2 (region 17170 - 29465)           (2649) ACCAGAGGTGAAAATACTCAATAATTTGG-GTGTGGACATTGCTGCTAATACTGTGATCTGGGACTACAAAAGAGATGCTCCAG-CACAT
```

Section 32

```
                                            (2791) 2791     2800      2810      2820      2830      2840      2850      2860      2870     2880
HERV- E (AB062274.1)                        (2531) AACCCCACTAGGGCTGATTGCCACCAGTTGCTCATGTTCCTCTTTAACACAGATGAAAGGCGGAGAGTGCTCCAAGCAGCAACTAAGTGG
SARS-CoV-2 (region 17170 - 29465)           (2737) ATATCTACTATTGGTGTTTGTT--CTATGACTGACATAGCCAAGAAAC-CAACTGAAA--CGATTTGTGCACCACTCACTGTCTTTTTG
```

Section 33

```
                                            (2881) 2881     2890      2900      2910      2920      2930      2940      2950      2960     2970
HERV- E (AB062274.1)                        (2621) CTAGAGGAAC--ATGCACC----GACTGATT-ACAAAAACCCCCAA-GAGGATGTAAGGACCCA-------GTTACTGGGAACCTACCCC
SARS-CoV-2 (region 17170 - 29465)           (2822) ATGGTAGAGTTGATGGTCAAGTAGACTTATTTAGAAATGCCCGTAATGGTGTTCTTATTACAGAAGGTAGTGTTAAAGGTTTACAACCAT
```

Section 34

```
                                            (2971) 2971     2980      2990      3000      3010      3020      3030      3040      3050     3060
HERV- E (AB062274.1)                        (2696) CAGTGGGACCCAAA----------TGAAAGACAGGATATGCAAAGGCTAAACCGATACAGGGAAGCTCTCTTGGAAGGATTAAAGAGGGG
SARS-CoV-2 (region 17170 - 29465)           (2912) CTGTAGGTCCCAAACAAGCTAGTCTTAATGGAGTCACATTAATTGGAGAAGCCGTAAAAACACAGTTCAATT---ATTATAAGAAAGTTG
```

Section 35

```
                                            (3061) 3061     3070      3080      3090      3100      3110      3120      3130      3140     3150
HERV- E (AB062274.1)                        (2776) AGCCCAGAAGGCCACAAACATTAACAAGGTCTCTGAGGTCATTCAGAG----AAA--AGAAGAAAGTCCAGC-ACAATTCTACGAGAGAC
SARS-CoV-2 (region 17170 - 29465)           (2999) AT----GGTGTTGTCCAACAATTACCTGAAA-CTTACTTTACTCAGAGTAGAAATTTACAAGAATTTAAACCCAGGAGTCAAATGGAAAT
```

Section 36

		3151 – 3240
	(3151)	3151 3160 3170 3180 3190 3200 3210 3220 3230 3240
HERV- E (AB062274.1)	(2859)	TG--TG-TGAGGCCTATCGTATGTATACTCCCTTTGATCCCGATAGCCCTGAAAATCAGTGCATGATTAACATGGCTT-TAGTTATTCAA
SARS-CoV-2 (region 17170 - 29465)	(3084)	TGATTTCTTAGAATTAGC-TATGGATGAATTCATTGAACGGTATAAATTAGAAGGCTATGCCTTCGAACATATCGTTTATGGAGATTTTA

Section 37

		3241 – 3330
	(3241)	3241 3250 3260 3270 3280 3290 3300 3310 3320 3330
HERV- E (AB062274.1)	(2945)	AGCACAGAAGACATTAGAAGAAAACTGCAGAAACAGGCTGGGTTTGC---AAGGATGAACACATCACAGTT---ATTAGAAATAGCTAAC
SARS-CoV-2 (region 17170 - 29465)	(3173)	GTCATAGTCAG--TTAGGTGGTT--TACATCTACTGATTGGACTAGCTAAACGTTTTAAGGAATCACCTTTTGAATTAGAAGATTTTATT

Section 38

		3331 – 3420
	(3331)	3331 3340 3350 3360 3370 3380 3390 3400 3410 3420
HERV- E (AB062274.1)	(3029)	CAGGTGTTTGTAAACAGGGATGCAGTAAGCCGTAAGGAAAACCAGAAAGAGAATGAACGTCAGGCCCAGCGAAACGC---TGATCTGTTA
SARS-CoV-2 (region 17170 - 29465)	(3259)	CCTATGGACAGTA-CAGTTAAAAACTATTTCATAAC-AGATGCGCAAACAGGTTCATC-TAAGTGTGTGTGTTCTGTTATTGATTTATTA

Section 39

		3421 – 3510
	(3421)	3421 3430 3440 3450 3460 3470 3480 3490 3500 3510
HERV- E (AB062274.1)	(3116)	GCTGCAGCAATC----AGAGGGGACCCCCCAAAGAGGCAAGGG--------AAGGGGGGCCCCGGGA--A----------AGAAACT-CA
SARS-CoV-2 (region 17170 - 29465)	(3346)	CTTGATGATTTTGTTGAAATAATAAAATCCCAAGATTTATCTGTAGTTTCTAAGGTTGTCAAAGTGACTATTGACTATACAGAAATTTCA

Section 40

		3511 – 3600
	(3511)	3511 3520 3530 3540 3550 3560 3570 3580 3590 3600
HERV- E (AB062274.1)	(3181)	-----GCCTGGCTGTCAGAGCTTGCAGCGTA--ATCAGTGTGCTTATTGTAAAGAAATAGGACATTGGAAGAACAAG----TGCT----C
SARS-CoV-2 (region 17170 - 29465)	(3436)	TTTATGCTTTGGTGTAA-AGATGGCCATGTAGAAACATTTTACCCAAAATTACAATCTAGTCAAGCGTGGCAACCGGGTGTTGCTATGCC

Section 41

		3601 – 3690
	(3601)	3601 3610 3620 3630 3640 3650 3660 3670 3680 3690
HERV- E (AB062274.1)	(3256)	TCAGCT----AAAAGGAAAACAAGGTGACTTGGAGCAGGAGGCACCAGACAAGGAGGAAGGGGCCCTGCTCAACCTGGCAGAAGGGTTAT
SARS-CoV-2 (region 17170 - 29465)	(3525)	TAATCTTTACAAAATGCAAAGAATGCTA-TTAGAAAAGTGTGACCTTCAAAATTATG--GTGATAGTGCAACAT-TACCTAAAGGCATAA

Section 42

		3691 – 3780
	(3691)	3691 3700 3710 3720 3730 3740 3750 3760 3770 3780
HERV- E (AB062274.1)	(3342)	TG--GACTGAGGGGGACTGGGCTCAAGGGCCCCC------AAAGAGCCTATGGTCAGGATGACAGT-TGGGGGTAAAGACATTGATTTTC
SARS-CoV-2 (region 17170 - 29465)	(3611)	TGATGAATGTCGCAAAATATACTCAACTGTGTCAATATTTAAACACATTAACATTAGCTGTACCCTATAATATGAGAGTTATACATTTTG

Section 43

	(3781)	3781 3790 3800 3810 3820 3830 3840 3850 3860 3870
HERV- E (AB062274.1)	(3423)	TTGTAGATACCGGTGCTGAACATTCGGTAGTAACCGCCCCGGTCGCCCCCTTATCCAAAAAGACTA-----TTGACATAATCGGAGCCAC
SARS-CoV-2 (region 17170 - 29465)	(3701)	GTGCTGGTTCTGATAAAGGAGTTGCACCAGGTACAGCTGTTTTAAGACAGTGGTTGCCTACGGGTACGCTGCTTGTCGATTCAGATCTTA

Section 44

	(3871)	3871 3880 3890 3900 3910 3920 3930 3940 3950 3960
HERV- E (AB062274.1)	(3508)	GGGAGTTTCAG-CAAAACAAGCTTTCTGCTTGCCCCGGACTTGT---ACTGTAGG---AGGACATAAAGTGATTCATCAGTTTTTGT-AC
SARS-CoV-2 (region 17170 - 29465)	(3791)	ATGACTTTGTCTCTGATGCAGATTCAACTTTGATTGGTGATTGTGCAACTGTACATACAGCTAATAAATGGGATC-TCATTATTAGTGAT

Section 45

	(3961)	3961 3970 3980 3990 4000 4010 4020 4030 4040 4050
HERV- E (AB062274.1)	(3590)	ATGCCTGACTGTCCCT-TGCCCTTGTTGGGAAGGGACTTGCTTAGCAAGCTGAGAGCCACTATCTCTTTTACAGAGCATGGCTCTTTGCT
SARS-CoV-2 (region 17170 - 29465)	(3880)	ATGTACGACCCTAAGACTAAAAATGTTACAAAAGAAAATGACTCTAAAG---AGGGT---TTTTTCACTTACATTTG-TGGGTTTATACA

Section 46

	(4051)	4051 4060 4070 4080 4090 4100 4110 4120 4130 4140
HERV- E (AB062274.1)	(3679)	GCTAAAGTTACCCAGAACGGGAGTCATTATGACCCTTACGGTCCCCCGAGAGGA--GGAATGGAGACTTTTCTTAACTGAGTTGAGCCAA
SARS-CoV-2 (region 17170 - 29465)	(3963)	ACAAAAGCTAGCTCTT---GGAGGTTCCGTGGCTATAAAGATAACAGAACATTCTTGGAATGCTGATCTTTATAAGCTCATGGGACACTT

Section 47

	(4141)	4141 4150 4160 4170 4180 4190 4200 4210 4220 4230
HERV- E (AB062274.1)	(3767)	GAGATAA-GACCAGCTCTGGCTAAGTGGTGGCCAAGAGTG-TGGCCTGAAGACAACCCTCCAGGGTTGGCAGT--C------AACCAAGC
SARS-CoV-2 (region 17170 - 29465)	(4050)	CGCATGGTGGACAGCCTTTGTTAC-TAATGTGAATGCGTCATCATCTGAAG-CATTTTTAATTGGATGTAATTATCTTGGCAAACCACGC

Section 48

	(4231)	4231 4240 4250 4260 4270 4280 4290 4300 4310 4320
HERV- E (AB062274.1)	(3847)	CCCCATACTTATAG--AAGTTAAGCCTGGGGACCAGCTGGTTAGGCAAAA-ACAGTACCCGGTCCCCAGAGAAGCT---CTTGAAGGTAT
SARS-CoV-2 (region 17170 - 29465)	(4138)	GAACAAATAGATGGTTATGTCATGCATGCAAATTACATATTTTGGAGGAATACAAATCCAATTCAGTTGTCTTCCTATTCTTTATTTGAC

Section 49

	(4321)	4321 4330 4340 4350 4360 4370 4380 4390 4400 4410
HERV- E (AB062274.1)	(3931)	CCAGGTCCATCTCAAGTGCC--TAAGAACCTTTGGAATTAAAGTTCCTTGTCAGTCTCCACGGAACACTCTCCTCCTGCCTGTTCCCAAG
SARS-CoV-2 (region 17170 - 29465)	(4228)	ATGAGTAAATTTCCCCTTAAATTAAGGGGTACTGCTGTTATGTCTTTAAAAGAAGGTCAAATCAATGATATGATTTTATCTCTTCTTAGT

```
                                                                                                              Section 50
                                    (4411) 4411     4420      4430      4440      4450      4460      4470      4480      4490     4500
            HERV- E (AB062274.1)   (4019) CCTGG--GACCAAGGACTACAGGCCGGTACAGGATT--------------TGCGCTTGGT------TAATCAAGCTACAGTGACTTTACA
SARS-CoV-2 (region 17170 - 29465)  (4318) AAAGGTAGACTTATAATTAGAGAAAACAACAGAGTTGTTATTTCTAGTGATGTTCTTGTTAACAACTAAACGAACAATGTTTGTTTTTCT
                                                                                                              Section 51
                                    (4501) 4501     4510      4520      4530      4540      4550      4560      4570      4580     4590
            HERV- E (AB062274.1)   (4087) TCCAACAGTAC--CTAACCCGTACACATTGTTGGGGTTGCTGCCAGCTGAGGACAGCTGCTTCACCTGCTTGGAC-----CTGAAAGACG
SARS-CoV-2 (region 17170 - 29465)  (4408) TGTTTTATTGCCACTAGTCTCTAGTCAGTGT-GTTAATCTTACAACCAGAACTCAATTAC--CCCCTGCATACACTAATTCTTTCACACG
                                                                                                              Section 52
                                    (4591) 4591     4600      4610      4620      4630      4640      4650      4660      4670     4680
            HERV- E (AB062274.1)   (4170) CTTTCTTTAGCATC-------AGATT--AGCCCCTGAG-----------AGCCAGAAGCTATT--TGCCTTTCAGTGGGAA-GATCCGGA
SARS-CoV-2 (region 17170 - 29465)  (4495) TGGTGTTTATTACCCTGACAAAGTTTTCAGATCCTCAGTTTTACATTCAACTCAGGACTTGTTCTTACCTTTCTTTTCCAATGTTACTTG
                                                                                                              Section 53
                                    (4681) 4681     4690      4700      4710      4720      4730      4740      4750      4760     4770
            HERV- E (AB062274.1)   (4237) GTCAGGTGTCACTACTCAGTACACTTGGACCCGGGTTCCCCAAGGGTTCAAGAACTCC--CCCACCATCTTTGGGGAGGTGTTGGCTC-G
SARS-CoV-2 (region 17170 - 29465)  (4585) GTTCCATG---CTATACATGTCTCTGGGACCAATGGTACTAAGAGGTTTGATAACCCTGTCCTACCAT-TTAATGATGGTGTTTATTTTG
                                                                                                              Section 54
                                    (4771) 4771     4780      4790      4800      4810      4820      4830      4840      4850     4860
            HERV- E (AB062274.1)   (4324) AGACCACC-AGAAGTTTCCCACCAGA-GACCTAGGCTGTGTGTTGCTCCAGTATGTT-GAT----------CTTTTGCTGGGACACCCCA
SARS-CoV-2 (region 17170 - 29465)  (4671) CTTCCACTGAGAAGTCTAACATAATAAGAGGCTGGATTTTTGGTACTACTTTAGATTCGAAGACCCAGTCCCTACTTATTGTTAATAACG
                                                                                                              Section 55
                                    (4861) 4861     4870      4880      4890      4900      4910      4920      4930      4940     4950
            HERV- E (AB062274.1)   (4401) CGGCAGTCGGGTGCGCCAAGGGAACAGACACACTACTCCGGCACC-TGGAGGACTGTGGGTATA--AGGTGTCCAAGAAAAAAGCTCAGA
SARS-CoV-2 (region 17170 - 29465)  (4761) CTACTAATGTTGTTATTAAAGTCTGTGAATTTCAATTTTGTAATGATCCATTTTTGGGTGTTTATTACCACAAAAACAACAAAAGTTGGA
                                                                                                              Section 56
                                    (4951) 4951     4960      4970      4980      4990      5000      5010      5020      5030     5040
            HERV- E (AB062274.1)   (4488) TC-----TGCCAACAGCACGTACATTACTTGGGAT--TTACTATCCGACAGGGGGAGCGCAGCCTG-------GGATCAGGAAG-AAAGC
SARS-CoV-2 (region 17170 - 29465)  (4851) TGGAAAGTGAGTTCAGAGTTTATTCTAGTGCGAATAATTGCACTTTTGAATATGTCTCTCAGCCTTTTCTTATGGACCTTGAAGGAAAAC
```

Section 57

```
                                          5041     5050      5060      5070      5080      5090      5100      5110      5120      5130
                                  (5041)
HERV- E (AB062274.1)              (4563)  AGGTCATTTGCAA---TCTGCCAGA------GCCTAAGACCA--GAAGGCAGGTGAGAGA---ATTCTTAGGAGCTGCGGGGTTTTGCAG
SARS-CoV-2 (region 17170 - 29465) (4941)  AGGGTAATTTCAAAAATCTTAGGGAATTTGTGTTTAAGAATATTGATGGTTATTTTAAAATATATTCTAAGCACACGCCTATTAATTTAG
```

Section 58

```
                                          5131     5140      5150      5160      5170      5180      5190      5200      5210      5220
                                  (5131)
HERV- E (AB062274.1)              (4639)  ACTGTGGA-TCCCAAAC--T-TTGCAGTATTAGCTAAGCCTTTGTGTGAGGTCACAA-AGTGGGGGGGACCGGGACCTTTTGAA-TGGGG
SARS-CoV-2 (region 17170 - 29465) (5031)  TGCGTGATCTCCCTCAGGGTTTTTCGGCTTTAG---AACCATTGGTAGATTTGCCAATAGGTATTAACATCACTAGGTTTCAAACTTTAC
```

Section 59

```
                                          5221     5230      5240      5250      5260      5270      5280      5290      5300      5310
                                  (5221)
HERV- E (AB062274.1)              (4723)  ATCCCAGCAACAGCAAGCCTTTCA--------TGAGT----TAAAGGAAAAACTTATGTCAGCCCCAGCCC--TG---GGGCTATCCGATC
SARS-CoV-2 (region 17170 - 29465) (5118)  TTGCTTTACATAGAAGTTATTTGACTCCTGGTGATTCTTCTTCAGGTTGGACAGCTGGT-GCTGCAGCTTATTATGTGGGTTATCT--TC
```

Section 60

```
                                          5311     5320      5330      5340      5350      5360      5370      5380      5390      5400
                                  (5311)
HERV- E (AB062274.1)              (4797)  TGACAAAGCCTTTTACATTGTATGTGTCAGAGAGAGAAAAGATGGCAGTTGGAGTT-----------TTAACCC------------AAACT
SARS-CoV-2 (region 17170 - 29465) (5205)  AACCTAGGACTTTTCTATTAAAATATAATGAAAATGGAACCATTACAGATGCTGTAGACTGTGCACTTGACCCTCTCTCAGAAACAAAGT
```

Section 61

```
                                          5401     5410      5420      5430      5440      5450      5460      5470      5480      5490
                                  (5401)
HERV- E (AB062274.1)              (4865)  GTGGGG------CCCTGGCTGAGGCCGGTGGCCTATCTCTCTAAACAACTAGACGGGGTTGCTAAAGGATGGCCCCCAT-GTTTGAGGGC
SARS-CoV-2 (region 17170 - 29465) (5295)  GTACGTTGAAATCCTTCACTGTAGAAAAAGGA--ATCTATC-AAACTTCTAACTTTAGAGTCCAACCAACAGAATCTATTGTTAGATTTC
```

Section 62

```
                                          5491     5500      5510      5520      5530      5540      5550      5560      5570      5580
                                  (5491)
HERV- E (AB062274.1)              (4948)  CT--TGGCACCAACT---GCCCTGCTAGTACAAG----A-----AGCAGATAAGCTGACTCTTGGGCA--AAACCTGAACATAAAGGCCC
SARS-CoV-2 (region 17170 - 29465) (5382)  CTAATATTACAAACTTGTGCCCTTTTGGTGAAGTTTTTAACGCCACCAGATTTGCATCTGTTTATGCTTGGAACAGGAAGAGAATCAGCA
```

Section 63

```
                                          5581     5590      5600      5610      5620      5630      5640      5650      5660      5670
                                  (5581)
HERV- E (AB062274.1)              (5022)  CCCATGCTG-TGGTGACTTTAACGAATACTAAAGGACATCATTAGCTAG---CGAATGCTA----GA--CTC--ACTAAGTACCA--AAG
SARS-CoV-2 (region 17170 - 29465) (5472)  ACTGTGTTGCTGATTATTCTGTCCTATATAATTCCGCATCATTTTCCACTTTTAAGTGTTATGGAGTGTCTCCTACTAAATTAAATGATC
```

Section 64

```
                                      (5671) 5671     5680      5690      5700      5710      5720      5730      5740      5750    5760
          HERV- E (AB062274.1)        (5098) TTTGCTCTGTGAAAATCCCCGTATAACCACT-GAAGTTTGTAACACCCTGAACCCCACTACCTTGCTCCCGGT--ATC---AGAGAGCCC
SARS-CoV-2 (region 17170 - 29465)     (5562) TCTGCTTTACTAATGTCTATGCAGATTCATTTGTAATTAG--AGGTGATGAAGTCAGACAAATCGCTCCAGGGCAAACTGGAAAGATTGC
```

Section 65

```
                                      (5761) 5761     5770      5780      5790      5800      5810      5820      5830      5840    5850
          HERV- E (AB062274.1)        (5182) TGTCAAGCATGATTGTGTAGAAGT--ATTGGA----CTCAGTTTACTCTAGCAGACCTGACC-TCTGGACC-AGCCTTGGGCATCAGTA
SARS-CoV-2 (region 17170 - 29465)     (5650) TGATTATAATTATAAATTACCAGATGATTTTACAGGCTGCGTTATAGCTTGGAATTCTAACAATCTTGATTCTAAGGTTGGTGGTAATTA
```

Section 66

```
                                      (5851) 5851     5860      5870      5880      5890      5900      5910      5920      5930    5940
          HERV- E (AB062274.1)        (5264) GACTGGGAACTATACGTGGATTGGAGCAGCTTCATC--AA-CCCACAAGGAGAGAGATTTGCAGGG-----TGTGCGGT-GGTA--ACCC
SARS-CoV-2 (region 17170 - 29465)     (5740) TAATTACCTGTATAGATTGTTTAG-GAAGTCTAATCTCAAACCTTTTGAGAGAGATATTTCAACTGAAATCTATCAGGCCGGTAGCACAC
```

Section 67

```
                                      (5941) 5941     5950      5960      5970      5980      5990      6000      6010      6020    6030
          HERV- E (AB062274.1)        (5343) TGGGCACTGTTGTTGAAGCCAGATCGTTGCCCCAGGGCACTTCAGCCCAGAAAGCTG--AACTCATTGCTTTCATTCAGGCCTTAGAACT
SARS-CoV-2 (region 17170 - 29465)     (5829) CTTGTAATGGTGTTGAAGGTTT-TAATTGTTACTTTCCTTTACAATC-ATATGGTTTCCAACCCACTAATGGTGTTGGTTACCAACCATA
```

Section 68

```
                                      (6031) 6031     6040      6050      6060      6070      6080      6090      6100      6110    6120
          HERV- E (AB062274.1)        (5431) CAGTGAAGGTAAGACTGTC-----AACATTTACACTGACTCTCGGTATGCCTTTTTAACCCTTCAAGTGCATGGAGCATTATATAAAGAA
SARS-CoV-2 (region 17170 - 29465)     (5917) CAGAGTAG-TAGTACTTTCTTTTGAACTTCTACA-TG-CACCAGCAACTGTTTGTGGACCTAAAAAGTCTACTAATTTGGTTAAAAACAA
```

Section 69

```
                                      (6121) 6121     6130      6140      6150      6160      6170      6180      6190      6200    6210
          HERV- E (AB062274.1)        (5516) AAGCGCCTATTG-AACT---CTGGGGGAAAGGACATAAAATATC-----------AACAAGAAATC-------TTGCAATTATT----AG
SARS-CoV-2 (region 17170 - 29465)     (6004) ATGTGTCAATTTCAACTTCAATGGTTTAACAGGCACAGGTGTTCTTACTGAGTCTAACAAAAAGTTTCTGCCTTTCCAACAATTTGGCAG
```

Section 70

```
                                      (6211) 6211     6220      6230      6240      6250      6260      6270      6280      6290    6300
          HERV- E (AB062274.1)        (5580) AAGCAGTATGGAAACCACACAAGGTGGCCGTTATGCATTG-CAGAGGACACCAGCGAGCTTCCACCTTGGTGGGTTTGCGGAATTCCCGC
SARS-CoV-2 (region 17170 - 29465)     (6094) AGACATTGCTGACACTACTGATGCTGTCCGTGATCCACAGACACTTGAGATTCTTGACATTACACCATGTTCTTTTGGTGGTGTCAGTGT
```

Section 71
```
                                          6301      6310      6320      6330      6340      6350      6360      6370      6380     6390
                                 (6301)
HERV- E (AB062274.1)             (5669)   GCTGACTTAGAGGCTCGAA-------AAGCAGCAT-CTGCCCCCT-TCCGGCATCAGT--CACAGCAC-CCCTGCTCC--CTCAAGCGC
SARS-CoV-2 (region 17170 - 29465) (6184)  TATAACACC-AGGAACAAATACTTCTAACCAGGTTGCTGTTCTTTATCAGGATGTTAACTGCACAGAAGTCCCTGTTGCTATTCATGCAG
```

Section 72
```
                                          6391      6400      6410      6420      6430      6440      6450      6460      6470     6480
                                 (6391)
HERV- E (AB062274.1)             (5745)   CTGATCTTTTACCTACTT---------ATTCTAAAGAAGAAAAGGACTTTC-------TCCAGGC--------AGAGGAGGACAAGTGA
SARS-CoV-2 (region 17170 - 29465) (6273)  ATCAACTTACTCCTACTTGGCGTGTTTATTCTACAGGTTCTAATGTTTTCAAACACGTGCAGGCTGTTTAATAGGGGCTGAACATGTCA
```

Section 73
```
                                          6481      6490      6500      6510      6520      6530      6540      6550      6560     6570
                                 (6481)
HERV- E (AB062274.1)             (5811)   TGGAGGAAGGATGGATTCGGTTACCAGATGGGAGAGAAGCTGTGCCACAGCTGCTAGGAGCCGC--AGTTGTGCTGGCTGTGCA--TA--
SARS-CoV-2 (region 17170 - 29465) (6363)  ACAACTCATATGAGTGTGACATACCCATTGGTGCAGGTA-TATGCGCTAGTTATCAGACTCAGACTAATTCTCCTCGGCGGGCACGTAGT
```

Section 74
```
                                          6571      6580      6590      6600      6610      6620      6630      6640      6650     6660
                                 (6571)
HERV- E (AB062274.1)             (5895)   ------AAACCACCCATCTAGGCCAGGA----GTCACTT------GAAAA----GTTG-TTAGTCCGGTATTTCTA----CAT---CTTG
SARS-CoV-2 (region 17170 - 29465) (6452)  GTAGCTAGTCAATCCATCATTGCCTACACTATGTCACTTGGTGCAGAAAATTCAGTTGCTTACTCTAATAACTCTATTGCCATACCCACA
```

Section 75
```
                                          6661      6670      6680      6690      6700      6710      6720      6730      6740     6750
                                 (6661)
HERV- E (AB062274.1)             (5957)   CATTTGTCAGCCCTTGCCAAAACGGTGACGC-AGCGGTGTGTTACCTGCCCAAAGCATAATGCGAA---GCAAGGTCCAGCTGTTCCGCC
SARS-CoV-2 (region 17170 - 29465) (6542)  AATTTTACTATTAGTGTTACCACAGAAATTCTACCAGTGTCTA---TGACCAAGACATCAGTAGATTGTACAATGTACATTTGTG-----
```

Section 76
```
                                          6751      6760      6770      6780      6790      6800      6810      6820      6830     6840
                                 (6751)
HERV- E (AB062274.1)             (6043)   CGTCATACAAGCTTATGGAGCAGCCCCCT-TTG-AAGATGTCCAGGTGGACTTCACAGAGATGCCAAAGTGTGGAGGTAACAAGTATTTA
SARS-CoV-2 (region 17170 - 29465) (6624)  -GTGATTCAACTGAATGCAGCAATCTTTTGTTGCAATATGGC-AGTTTTTGTACACAATTAAACCGTGCTTTAACTGGAATAGCTGTTGA
```

Section 77
```
                                          6841      6850      6860      6870      6880      6890      6900      6910      6920     6930
                                 (6841)
HERV- E (AB062274.1)             (6131)   -CTAG--------TTCTTGTGTGTACCTACTCTGGTGGGTGGAGGCT--TATCCAGCACAAACTGAGAAAGCTCGTGAAGTAACCTGTGT
SARS-CoV-2 (region 17170 - 29465) (6712)  ACAAGACAAAAACACCCAAGAAGTTTTTGCACAAGTCAAACAAATTTACAAAACACCACCAATTAAAGATTTTGGTGGTTTTAATTTTC
```

```
                                                                                                          Section 78
                                        (6931) 6931     6940      6950      6960      6970      6980      6990      7000      7010      7020
            HERV- E (AB062274.1)        (6210) GC---TTCTTCGAGATCCT---ATTCCTAG-ATTTGGACTGCCCTTACGGATCGGCTCA--GATAATGGGCCAGCATTTGTGGCTGACTT
SARS-CoV-2 (region 17170 - 29465)       (6802) ACAAATATTACCAGATCCATCAAAACCAAGCAAGAGGTCATTTATTGAAGATCTACTTTTCAACAAAGTGACACTTGCAGATGCTGGCTT
                                                                                                          Section 79
                                        (7021) 7021     7030      7040      7050      7060      7070      7080      7090      7100      7110
            HERV- E (AB062274.1)        (6291) GGT-ACAGAAGAGGGCAAAGGTATTGGGGAACACATGGAAACTGCATGCTGCCTACCGGCCTCAGAGTTCCAGAAAGGTGGAGTCGATGA
SARS-CoV-2 (region 17170 - 29465)       (6892) CATCAAACAATATGGTGATTGCCTTGGTGAT---ATTGCTGCTAGAGACCTCATTTGTGCACAAAAGTTTAACGGCCTTACTGTTTTGCC
                                                                                                          Section 80
                                        (7111) 7111     7120      7130      7140      7150      7160      7170      7180      7190      7200
            HERV- E (AB062274.1)        (6380) A---TTGGAC---TATCAAAA-ATAGCTTAGGGAAAGTATGTCAGGAAACAGGATTAAAATGGATACAGGCTCTCCCTATGGTATTATTT
SARS-CoV-2 (region 17170 - 29465)       (6979) ACCTTTGCTCACAGATGAAATGATTGCTCAATACACTTCTGCACTGTTAGCGGGT-ACAATCACTTCTGGTTGGACCTTTGGTGCAGGTG
                                                                                                          Section 81
                                        (7201) 7201     7210      7220      7230      7240      7250      7260      7270      7280      7290
            HERV- E (AB062274.1)        (6463) AAA-ATTAGATGTACCCCTT-CTAAAAGAACAGGATATTCCCCTTATGAAATATT-GTATCATAG---GCCCCCTCCCATATTGCA---G
SARS-CoV-2 (region 17170 - 29465)       (7068) CTGCATTACAAATACCATTTGCTATGCAAATGGCTTATAGGTTTAATGGTATTGGAGTTACACAGAATGTTCTCTATGAGAACCAAAAAT
                                                                                                          Section 82
                                        (7291) 7291     7300      7310      7320      7330      7340      7350      7360      7370      7380
            HERV- E (AB062274.1)        (6544) GGACTTCCAGGCACTCCCCGAGAGTTA--GGTGAAATTGA-GTTACAGCGACAACTACAG-----GCTTTAGGAAAAATTACACAAACAA
SARS-CoV-2 (region 17170 - 29465)       (7158) TGATTGCCAACCAATTTAATAGTGCTATTGGCAAAATTCAAGACTCACTTTCTTCCACAGCAAGTGCACTTGGAAAACTTCAAGATGTGG
                                                                                                          Section 83
                                        (7381) 7381     7390      7400      7410      7420      7430      7440      7450      7460      7470
            HERV- E (AB062274.1)        (6626) TCT--CAGCCTGGGTAAA--TGAGAGATGCCCTGTTAGCTTATTCTCCCCAGTT----CACCCTTTCTCCCCAGGTAATCGAGTGTGGAT
SARS-CoV-2 (region 17170 - 29465)       (7248) TCAACCAAAATGCACAAGCTTTAAACACGCTTGTTAAACAACTTAGCTCCAATTTTGGTGCAATTTCAAGTGTTTTAAATGATATCCTTT
                                                                                                          Section 84
                                        (7471) 7471     7480      7490      7500      7510      7520      7530      7540      7550      7560
            HERV- E (AB062274.1)        (6708) CAAGGACTGG-AACGTAACTTCTTT-GTGCCCAC-GGTGGAAAGACCGTAGTCTGTCGTCCTGACCACTCCCACCGCTGTGAAGGTAGAG
SARS-CoV-2 (region 17170 - 29465)       (7338) CACGTCTTGACAAAGTTGAGGCTGAAGTGCAAATTGATAGGTTGATCACAGGCAGACTTCAA-AGTTTGCAGACATATGTGACTCAACAA
```

Section 85

```
                                        (7561) 7561     7570      7580      7590      7600      7610      7620      7630      7640     7650
                HERV- E (AB062274.1)    (6795) GGAATCCCAGCCTGGATCCACCACAGCCG-TGTAAAACCTGCAGCACCTGAAACCTGGGAG---------GCAAGACCAAGCCCAGACGA
SARS-CoV-2 (region 17170 - 29465)       (7427) TTAATTAGAGC-TGCAGAAATCAGAGCTTCTGCTAATCTTGCTGCTACTAAAATGTCAGAGTGTGTACTTGGACAATCAAAAAGAGTTGA
```

Section 86

```
                                        (7651) 7651     7660      7670      7680      7690      7700      7710      7720      7730     7740
                HERV- E (AB062274.1)    (6875) CCCTTGCAGA--G-------------TGACCCTGAGGAAGACGACAAGCCCTGCTCCAGTCACACCCAGAAGCTGACTG--GTCCACGCA
SARS-CoV-2 (region 17170 - 29465)       (7516) TTTTTGTGGAAAGGGCTATCATCTTATGTCCTTCCCTCAGTCAGCACCTCATGGTGTAGTCTT--CTTGCATGTGACTTATGTCCCTGCA
```

Section 87

```
                                        (7741) 7741     7750      7760      7770      7780      7790      7800      7810      7820     7830
                HERV- E (AB062274.1)    (6948) CGGCCGAAGCATGAGGAAGCTCATCGTGGGATTCATTTTTCTTAAATTCTGGACTTACACAGT-AAGGG--CTTCAACTGACCTTACTCA
SARS-CoV-2 (region 17170 - 29465)       (7604) CAAGAAAAGAACTTCACAACTGCTCCTGCCATTTGTCATGATGGAAAAGCACACTTTCCTCGTGAAGGTGTCTTTGTTTCAAATGGCACA
```

Section 88

```
                                        (7831) 7831     7840      7850      7860      7870      7880      7890      7900      7910     7920
                HERV- E (AB062274.1)    (7035) AACTGGGGACTGTTCCCAGTGTATTCATCAGGTCACTGAGGTAGGACAGCAAATTAAAACAATCTTTCTGTTCTATAGTTATTATGAATA
SARS-CoV-2 (region 17170 - 29465)       (7694) CACTGGTTTGTAACACAAAGGAATTTTTATGAACCACAAATCATTACTACAGAC-AACACA---TTTGTGTCTGGTAACTGTGATGTTGT
```

Section 89

```
                                        (7921) 7921     7930      7940      7950      7960      7970      7980      7990      8000     8010
                HERV- E (AB062274.1)    (7125) TATGGGAAT-GTTAA-AAGAAACTTGTTTGTATAATGCCAC-TCAGTACAAGGTATGTAGCCTGGGAAATGACCCACCTGATGTGTGTTA
SARS-CoV-2 (region 17170 - 29465)       (7780) AATAGGAATTGTCAACAACACAGTTTATGATCCTTTGCAACCTGAATTAGACTCATTCAAGGAGGAGTTAGATAAATATTTTAAGAATCA
```

Section 90

```
                                        (8011) 8011     8020      8030      8040      8050      8060      8070      8080      8090     8100
                HERV- E (AB062274.1)    (7212) TAACCCATCTGAGCCCCCTGCAACCACCATTTTTGAAACAA--GATTAAGAACTAGCCTTTTCCTAGGTGACACAAGTAAAATAATAACT
SARS-CoV-2 (region 17170 - 29465)       (7870) TACATCACCAGATGTTGATTTAGGTGACATCTCTGGCATTAATGCTTCAGTTGTAAACATTCAAAAAGAAATTGACCGCCTCAATGAGGT
```

Section 91

```
                                        (8101) 8101     8110      8120      8130      8140      8150      8160      8170      8180     8190
                HERV- E (AB062274.1)    (7300) AGAACAGAAGAAAAAGGAATCCCCAAACAAATAATTTTAAGATTTGATGCTTGTGCAGCCATTAATAGTAACAAACTAGGAACAGAATG-
SARS-CoV-2 (region 17170 - 29465)       (7960) TGCCAAGAATTTAAATGAATCTCTCATCGATCTCCAAGAACTTGGAAAGTATGAGCAGTATATAAAATGGCCAT----GGTACATTTGGC
```

Section 92

Sequence	Position	8191 … 8280
	(8191)	8191 8200 8210 8220 8230 8240 8250 8260 8270 8280
HERV- E (AB062274.1)	(7389)	TGGTTCTCTTAACTGGGAAAGGAGCTACAGAGTAGAAAATAAATATGTTTGTCATGAGTCAGGGGATTGTGAAAATTGTGCCTTTTGGCC
SARS-CoV-2 (region 17170 - 29465)	(8046)	TAGGTTTTATAGCTGGCTTGATTGCCATAGTAATGGTGACAATTATGCTTTGC-TGTATGACCAGTTGCTGTAG-TTGT--CTCAAGGGC

Section 93

Sequence	Position	8281 … 8370
	(8281)	8281 8290 8300 8310 8320 8330 8340 8350 8360 8370
HERV- E (AB062274.1)	(7479)	ATGTGTTATTTGGGCTACT--TGGAAAAAGAACAAAAAAGACCCGGTTCATCTTCAGAAGGGGAAGCCAACCCTTCCTGTGCTGCCGGT
SARS-CoV-2 (region 17170 - 29465)	(8132)	TGTTGTTCTTGTGGATCCTGCTGCAAATTTGATGAAGACGACTCTGAGCCAGTGCTCAAAGG---AGTCAAA--TTACATTACA--CATA

Section 94

Sequence	Position	8371 … 8460
	(8371)	8371 8380 8390 8400 8410 8420 8430 8440 8450 8460
HERV- E (AB062274.1)	(7567)	CACTGTAACCCACTAGAACTAATAATTACCAATCCCCTAGATCCCCATTAGAAAAAGGGAG-AACGTGTAACCCTGGGGATCGATGGGAC
SARS-CoV-2 (region 17170 - 29465)	(8215)	AACG--AACTTA-TGGATTTGTTTATGAG-AATCTTCACAATTGGAACTGTAACTTTGAAGCAAGGTGAAATCAAGGATGCTACTCCTTC

Section 95

Sequence	Position	8461 … 8550
	(8461)	8461 8470 8480 8490 8500 8510 8520 8530 8540 8550
HERV- E (AB062274.1)	(7656)	GGGGTTAAACCCCCAAGTTGCCATTTTAATTAGAGGGGAGGTCCACAAGCGCTCTCCCAAGCCAGTATTTCAAACCTTTTATGAGGAGCT
SARS-CoV-2 (region 17170 - 29465)	(8301)	AGATTTTGTTCGCGCTACTGCAACGATACCGATACAAG----CCTCACTCCCTTTCGGATGGCT-TATTGTTGGCGTTGCACTTCTTGCT

Section 96

Sequence	Position	8551 … 8640
	(8551)	8551 8560 8570 8580 8590 8600 8610 8620 8630 8640
HERV- E (AB062274.1)	(7746)	GAATCTGCCAGCACCAG-AACTTCCGAAAAAGACAAAAAATTTGTTTATCCAATCAGCAGAAAATGTAGCTCATTCCCTTAATGTTACTT
SARS-CoV-2 (region 17170 - 29465)	(8386)	GTTTTTCAGAGCGCTTCCAAAATCATAACCCTCAAAAGAGATGGCAA--CTAGCACTCTCCAAGGGTGTTCACTTTGTT--TGCAACTT

Section 97

Sequence	Position	8641 … 8730
	(8641)	8641 8650 8660 8670 8680 8690 8700 8710 8720 8730
HERV- E (AB062274.1)	(7835)	CTTGTTAT-GTATG---CGGGGA---ACCACTATCG---GAGACCGATGGCCTTGGGAAGCCCGAGAGTTGGTGCCTACTGATCCAG--
SARS-CoV-2 (region 17170 - 29465)	(8472)	GCTGTTGTTGTTTGTAACAGTTTACTCACACCTTTTGCTCGTTGCTGCTGGCCTTG--AAGCCCCTTTTCTCTATCTTTATGCTTTAGTC

Section 98

Sequence	Position	8731 … 8820
	(8731)	8731 8740 8750 8760 8770 8780 8790 8800 8810 8820
HERV- E (AB062274.1)	(7913)	--CTCCTGATGTAATTCCAGTTCAGAAGGCCCAAGCTAGCAACTT--------CTGG-------GTCCTAAAAACC--TCAATTATTGGA
SARS-CoV-2 (region 17170 - 29465)	(8560)	TACTTCTTGCAGAGTATAAACTTTGTAAGAATAATAATGAGGCTTTGGCTTTGCTGGAAATGCCGTTCCAAAAACCCATTACTTTATGAT

Section 99

```
                                (8821) 8821     8830      8840      8850      8860      8870      8880      8890      8900     8910
          HERV- E (AB062274.1)  (7984) CAATACTGTATAGCTAGAGAAGGGAAAGTCTTTATCATCCCTGTAAGAAAGCTTA--ATTGTATAGGA-CAGAAGTTGT-ACAACAGCAC
SARS-CoV-2 (region 17170 - 29465) (8650) GCCAACTATTTTCTTTGCT--GGCATACTAATTGTTACGACTATTGTATACCTTACAATAGTGTAACTTCTTCAATTGTCATTACTTCAG
```

Section 100

```
                                (8911) 8911     8920      8930      8940      8950      8960      8970      8980      8990     9000
          HERV- E (AB062274.1)  (8070) AACA----AAGACAATTACTTGGTGGGGCCTAAACCACACTGAAAAGAACCCATTTAGTAAATTT----TCTAAATTAAAA-ACTGCCTG
SARS-CoV-2 (region 17170 - 29465) (8738) GTGATGGCACAACAAGTCCTATTTCTGAACATGACTAC-CAGATTGGTGGTTATACTGAAAAATGGGAATCTGGAGTAAAAGACTGTGTT
```

Section 101

```
                                (9001) 9001     9010      9020      9030      9040      9050      9060      9070      9080     9090
          HERV- E (AB062274.1)  (8151) GGCTCATCCAGAATCTCATCAGGACTGGGCAGCTCCCGCTGGACTATACTGGATATGTGGGCACGGAGCCTACATTCG-----GTTACCT
SARS-CoV-2 (region 17170 - 29465) (8827) GTATTACACAGTTACT--TCACTTCAGACTATTACCAGCTGTACTCAACTCAAT---TGAGTACAGACACTGGTGTTGAACATGTTACCT
```

Section 102

```
                                (9091) 9091     9100      9110      9120      9130      9140      9150      9160      9170     9180
          HERV- E (AB062274.1)  (8236) AATAAATGGGCAGGCAGTTGTGTTATTGGCACTATTAAGCCGTCCTTTTTCTTATTACCCATAAAACGGGTGAGCTCCTAGGTTTCCCT
SARS-CoV-2 (region 17170 - 29465) (8912) TCTTCATCTACAATAAAATTGTTGATGAGCCTGAAGAACATGTCCAAATTC-----ACACA-ATCGACGGTTCA--TCCGGAGTT-----
```

Section 103

```
                                (9181) 9181     9190      9200      9210      9220      9230      9240      9250      9260     9270
          HERV- E (AB062274.1)  (8326) GTCTATGCCTCCCAAGAGAGGCATAGTTAT-AGGAAACTGGAAAGATAATGAGTGGCCCCCTGAAAG--------GATCA-TACAGTATT
SARS-CoV-2 (region 17170 - 29465) (8989) GTTAATCCAGT--AATGGAACCA-ATTTATGATGAACCGACGACGACTACTAGCGTGCCTTTGTAAGCACAAGCTGATGAGTACGAACTT
```

Section 104

```
                                (9271) 9271     9280      9290      9300      9310      9320      9330      9340      9350     9360
          HERV- E (AB062274.1)  (8406) ATGGGC-CTGCCGCATGGGCACAAGACAGCTCATGGGGATACTGAATCCCCATCTACATGCTCAATCAGA--TCATATGGTTGCAGGCCA
SARS-CoV-2 (region 17170 - 29465) (9076) ATGTACTCATTCGTTTCGGAAGA-GACAGGT-ACGTTAATAGTTAATAGCGTACTTCTTTTTCTTGCTTTCGTGGTATTCTTGCTAGTTA
```

Section 105

```
                                (9361) 9361     9370      9380      9390      9400      9410      9420      9430      9440     9450
          HERV- E (AB062274.1)  (8493) TCTTAGAAATAATTACTAATGAAACTTTGACTGTTTTAGCTTGGCAGGAAAC----CCAAATGAGGAATGCTAT-CTCTCAGAATAGGCT
SARS-CoV-2 (region 17170 - 29465) (9164) CACTAGCCATCCTTACTGCG----CTTCGATTGTGTGCGTACTGCTGCAATATTGTTAACGTGAGTCTTGTAAAACCTTCTTTTTACGTT
```

Section 106

```
                                     (9451) 9451     9460      9470      9480      9490      9500      9510      9520      9530     9540
HERV- E (AB062274.1)                 (8578) GGCCTTGGACTACTTGCTAGCAGCTGAAGGAGGAGTTTGTGAAAATTTAACTTAACCAGTTGCTGCCTACAAATAGATGATCAAGGACA
SARS-CoV-2 (region 17170 - 29465)    (9250) TACTCTCGTGTTAAAAATCTGAATTCTTCTAG-AGTTCCTGATCTTCTGGTCTAAACGAACTAAATATTATAT-TAGTTTTTCT--GTTT
```

Section 107

```
                                     (9541) 9541     9550      9560      9570      9580      9590      9600      9610      9620     9630
HERV- E (AB062274.1)                 (8668) GGTGGTTGAAAACATAGTCAGGG-ACATGACAAAGGTGGCACATGTGTCTGTACAGGTTTGGCACGAGTTTGATCCTGAGTCTTTCTTTG
SARS-CoV-2 (region 17170 - 29465)    (9336) GGAACTTTAATTT-TAGCCATGGCAGATTCCAACGGTACTATTACCGT-TGAAGAGCTTAAAAAGCTCCTTGAAC---AATGGAACCTAG
```

Section 108

```
                                     (9631) 9631     9640      9650      9660      9670      9680      9690      9700      9710     9720
HERV- E (AB062274.1)                 (8757) GAAAATGGTTTCCAGCTAC--AGGAGGATTAAAAACCCTCATTGTAGGTGTAGTGCTAGTAATAGGAACTTGCTTGCTGCTCCCCTGTGT
SARS-CoV-2 (region 17170 - 29465)    (9421) TAATA-GGTTTCCTATTCCTTACATGGATTTGTCTTCTACAATTTGCCTATGCCAACAGGAATAGGTTTTTGTATA-TAATTAAGTTAAT
```

Section 109

```
                                     (9721) 9721     9730      9740      9750      9760      9770      9780      9790      9800     9810
HERV- E (AB062274.1)                 (8845) ATTACCCTTGCTTTTT---CAAATGA---TAAAATGTTTTGTTGCTACTTTGGTTCATCAAAAAACTTC-AGCACACGTGTA----TTAT
SARS-CoV-2 (region 17170 - 29465)    (9509) TTTCCTCTGGCTGTTATGGCCAGTAACTTTAGCTTGTTTTGTGCTTGCTGCTGTTTACAGAATAAATTGGATCACCGGTGGAATTGCTAT
```

Section 110

```
                                     (9811) 9811     9820      9830      9840      9850      9860      9870      9880      9890     9900
HERV- E (AB062274.1)                 (8924) ATGAATCACTA-TC------GCTCTATCTCGCAAAGAGACT-------------CAGAAAGT----GAGGATGAG-----AGTGAGAACT
SARS-CoV-2 (region 17170 - 29465)    (9599) CGCAATGGCTTGTCTTGTAGGCTTGATGTGGCTCAGCTACTTCATTGCTTCTTTCAGACTGTTTGCGCGTACGCGTTCCATGTGGTCATT
```

Section 111

```
                                     (9901) 9901     9910      9920      9930      9940      9950      9960      9970      9980     9990
HERV- E (AB062274.1)                 (8985) CCCACTAAAAAGTGAAAA--TTCTCAAAGGGGGGAATATGGTACGA----GACCA--CCACTTCTCC----TGTTGTCCT--TCCCAGT
SARS-CoV-2 (region 17170 - 29465)    (9689) CAATCCAGAAACTAACATTCTTCTCAACGTGCCACTCCATGGCACTATTCTGACCAGACCGCTTCTAGAAAGTGAACTCGTAATCGGAGC
```

Section 112

```
                                     (9991) 9991     10000     10010     10020     10030     10040     10050     10060     10070    10080
HERV- E (AB062274.1)                 (9061) TTCTCCCCAACCTCCCCTTTTCCCTAGTTTATAAGACAG--GAGAAAAGGGAGAAAGCAAAAAGTTGG--AAAGAAACAGAAGAAAGATA
SARS-CoV-2 (region 17170 - 29465)    (9779) TGTGATCCTTCGTGGACATCTTCGTATTGCTGGACACCATCTAGGACGCTGTGACATCAAGGACCTGCCTAAAGAAATCACTGTT-GCTA
```

Section 113

(10081) 10081 10090 10100 10110 10120 10130 10140 10150 10160 10170

HERV- E (AB062274.1) (9147) AATAGCTAGACGACCTTGGCGCCACCACCTGGC-----CCTGGTGGTTAAAATGATAATAATATTAA-CCCCTGACCAAAACTACTGG--

SARS-CoV-2 (region 17170 - 29465) (9868) CATCACGA-ACG-CTTTCTTATTACAAATTGGGAGCTTCGCAGCGTGTAGCAGGTGACTCAGGTTTTGCTGCATACAGTCGCTACAGGAT

Section 114

(10171) 10171 10180 10190 10200 10210 10220 10230 10240 10250 10260

HERV- E (AB062274.1) (9229) TGTTATCTGTAAATTC----CAGAC-ATTGTGTGAGAAAGCACTGTAAAACTTT--TTGTACTGTTAG---CT---GATGT---AGGTAG

SARS-CoV-2 (region 17170 - 29465) (9956) TGGCAACTATAAATTAAACACAGACCATTCCAGTAGCAGTGACAATATTGCTTTGCTTGTACAGTAAGTGACAACAGATGTTTCATCTCG

Section 115

(10261) 10261 10270 10280 10290 10300 10310 10320 10330 10340 10350

HERV- E (AB062274.1) (9303) CCCACAGTCACATTCCT----CACACTTACTTG-ATCTATTATGACT-CTTTCACGTAGACCCCTTAGAGTTGTAAGCCCTTCAAAGGGC

SARS-CoV-2 (region 17170 - 29465) (10046) TTGACTTTCAGGTTACTATAGCAGAGATATTACTAATTATTATGAGGACTTTTAAAGTTTCCATTTGGAATCTTGATTACATCATAAACC

Section 116

(10351) 10351 10360 10370 10380 10390 10400 10410 10420 10430 10440

HERV- E (AB062274.1) (9387) TAGGAATT---TCTTTTTCGGGG-----AGCTCGG--C------TCTTAA---GATGCGAGTCTGCTGATGCTCCCGG-----CCGAATA

SARS-CoV-2 (region 17170 - 29465) (10136) TCATAATTAAAAATTTATCTAAGTCACTAACTGAGAATAAATATTCTCAATTAGATGAAGAGCAACCAATGGAGATTGATTAAACGAACA

Section 117

(10441) 10441 10450 10460 10470 10480 10490 10500 10510 10520 10530

HERV- E (AB062274.1) (9453) AAAAACCTCTTCCTTCTTT----------AATCCGGTGTCTCAGGAGTTTTGTCT-------GCGACTCGTCCTGCTACAGCAGCCCTAG

SARS-CoV-2 (region 17170 - 29465) (10226) TGAAAATTATTCTTTTCTTGGCACTGATAACACTCGCTACTTGTGAGCTTTATCACTACCAAGAGTGTGTTAGAGGTACAACAGTACTTT

Section 118

(10531) 10531 10540 10550 10560 10570 10580 10590 10600 10610 10620

HERV- E (AB062274.1) (9526) CAAACTAA-----------------ATAGCAATGCCAT-CTTCATCCAATTCTCT----GGAAATAAGCGGGAAGCCAGCTACAATGTAA

SARS-CoV-2 (region 17170 - 29465) (10316) TAAAAGAACCTTGCTCTTCTGGAACATACGAGGGCAATTCACCATTTCATCCTCTAGCTGATAACAAATTTGCACTGACTTGCTTTAGCA

Section 119

(10621) 10621 10630 10640 10650 10660 10670 10680 10690 10700 10710

HERV- E (AB062274.1) (9594) GTCCAACTACCCCAAGAATATCGTGCTGGAG---AGCCCATTTGT-AGGTACGACAGTCAACAGCCCCGGCTGAACTCAACAGCCGGCAC

SARS-CoV-2 (region 17170 - 29465) (10406) CTC-AATTTGCTTTTGCTTGTCCTGACGGCGTAAAACACGTCTATCAGTTACGTGCCAGATCAGTTTCACCTAAACTGTTCATCAGACAA

Section 120

(10711) 10711 10720 10730 10740 10750 10760 10770 10780 10790 10800
HERV- E (AB062274.1) (9680) CAGCATGGGAGTGAGACTCCTCAGGCATCAGCCCGGTGGATCATTTAGATAACTGCAGATCTGCCAGCCTCTGATGGTATCCGCATGTGA
SARS-CoV-2 (region 17170 - 29465) (10495) GAGGAAGTTCAAGA-ACT--TTACTCTCCAATTTTTCTTATTGTTGCGGCAATAGTGTTTATAACA-CTTTGCTTCACACTCAAAAGAAA

Section 121

(10801) 10801 10810 10820 10830 10840 10850 10860 10870 10880 10890
HERV- E (AB062274.1) (9770) GACTCAAAGCGAGAACTGCTGAGGTGAGCGCTTCCTGGTCCTGACCCACAACATCATGAGTGACATACAATGGTTGTTTGAGGCTGCCAA
SARS-CoV-2 (region 17170 - 29465) (10581) GACAGAATGATTGAACT--TTCATTAATTGACTTCTATTTGTG---CTTTTTAGCCTTTCTGCTATTCCTTGTTTTAATTATGCTTATTA

Section 122

(10891) 10891 10900 10910 10920 10930 10940 10950 10960 10970 10980
HERV- E (AB062274.1) (9860) GTTTTAGGGT--AACTTATTATACAACATAGTAACCAGAGCACCTAC-CCTCTCCCTTCCCCCACTTGCCACT-------GGCTGGAACA
SARS-CoV-2 (region 17170 - 29465) (10666) TCTTTTGGTTCTCACTTGAACTGCAAGATCATAATGAAACTTGTCACGCCTAAACGAACATGAAATTTCTTGTTTTCTTAGGAATCATCA

Section 123

(10981) 10981 10990 11000 11010 11020 11030 11040 11050 11060 11070
HERV- E (AB062274.1) (9940) CAGATG-AGGT-CATGGCAG-------GCTGTCTTCAGTCCTGTCAATGACATAGATGAGGGCATGGGGAAGCCACAAATGAGGAGC-CT
SARS-CoV-2 (region 17170 - 29465) (10756) CAACTGTAGCTGCATTTCACCAAGAATGTAGTTTACAGTCATGTACTCAACATCAACCATATGTAGTTGATGACCCGTGTCCTATTCACT

Section 124

(11071) 11071 11080 11090 11100 11110 11120 11130 11140 11150 11160
HERV- E (AB062274.1) (10020) GGGCTTTTGGATGCCCTCGTGGAGCAG-AGAGGCCAGCTCCCCAGATGTCACTTGCATTTCAGGT--AAGAG-----AAATAAATTTCCT
SARS-CoV-2 (region 17170 - 29465) (10846) TCTATTCTAAATGGTATATTAGAGTAGGAGCTAGAAAATCAGCACCTTTAA-TTGAATTGTGCGTGGATGAGGCTGGTTCTAAATCACCC

Section 125

(11161) 11161 11170 11180 11190 11200 11210 11220 11230 11240 11250
HERV- E (AB062274.1) (10102) -TTCTGTTTAA-GTCACTGTTAATTCTATCTGTTT--TTTTAAGCCAAATCAATATCCTAGTAATATCATATAT--GTAAAACGAACAGC
SARS-CoV-2 (region 17170 - 29465) (10935) ATTCAGTACATCGATATCGGTAATTATA-CAGTTTCCTGTTTACCTTTTACAATTAATTGCCAGGAACCTAAATTGGGTAGTCTTGTAGT

Section 126

(11251) 11251 11260 11270 11280 11290 11300 11310 11320 11330 11340
HERV- E (AB062274.1) (10186) TCAGAGTCTAGCACATAAATAAGTGCTTTTTGGAAAAGGTAATTATTGCTATTATATTAATTAT-TATACTAATAATAGTTATTAATAAT
SARS-CoV-2 (region 17170 - 29465) (11024) GCGTTGTTCGTTCTATGAAGACTTT-TTAGAGTATCATGACGTTCGTGTTGTTTTAGATTTCATCTAAACGAACAA-ACTAAAATGTCTG

```
                                                                                                Section 127
                                      (11341) 11341    11350     11360     11370     11380     11390     11400     11410     11420     11430
               HERV- E (AB062274.1) (10275) ATTATT-ACCCTAAACTGCTCAACACTCTTCAAAATGAACATGAGGTGGACCC----ATGTCACTGATGGTCAATGAGACATGACAA---
SARS-CoV-2 (region 17170 - 29465) (11112) ATAATGGACCCCAAAATCAGCGAAATGCACCCCGCATTACGTTTGGTGGACCCTCAGATTCAACTGGCAGT-AACCAGAATGGAGAACGC
                                                                                                Section 128
                                      (11431) 11431    11440     11450     11460     11470     11480     11490     11500     11510     11520
               HERV- E (AB062274.1) (10357) -GCTGGGC-------AAAAATTGTCTGTGGTCATGCCTA---ATTCATACCAGGTCTCTCTTC---CATCCACCTTAAACTGACTAC---
SARS-CoV-2 (region 17170 - 29465) (11201) AGTGGGGCGCGATCAAAACAACGTCGGCCCCAAGGTTTACCCAATAATACTGCGTCTTGGTTCACCGCTCTCACTCAACATGGCAAGGAA
                                                                                                Section 129
                                      (11521) 11521    11530     11540     11550     11560     11570     11580     11590     11600     11610
               HERV- E (AB062274.1) (10430) -AGCT----CTCCCAGGCAGACCAATAGATA-AGGGATCACAGACACCAGCGCACA-GACACATCCTCCCACTCCCCAC---C---CACA
SARS-CoV-2 (region 17170 - 29465) (11291) GACCTTAAATTCCCTCGAGGACAAGGCGTTCCAATTAACACCAATAGCAGTCCAGATGACCAAATTGGCTACTACCGAAGAGCTACCAGA
                                                                                                Section 130
                                      (11611) 11611    11620     11630     11640     11650     11660     11670     11680     11690     11700
               HERV- E (AB062274.1) (10507) CAGTGAGCTGAAGCTCCGGGTAGCCACTGTATGCTCCCA-TCCCACCTGTCCTATCG-CAGGAGAGAAGACAGGCAGCAAAATGGGATGG
SARS-CoV-2 (region 17170 - 29465) (11381) CGAATTCGTGGTGGTGACGGTAA--AATGAAAGATCTCAGTCCAAGATGGTATTTCTACTACCTAGGAACTGGGCCAGAAGCTGGACTTC
                                                                                                Section 131
                                      (11701) 11701    11710     11720     11730     11740     11750     11760     11770     11780     11790
               HERV- E (AB062274.1) (10595) AAAGTGGGTCTAAGATA-ATGAGTGGGGAGAGGGAGCCAGAGAAACTAACTCCA--TCCCCAGGAAATCAGGCAAGGCTTCCGGGAGGAG
SARS-CoV-2 (region 17170 - 29465) (11469) CCTATGGTGCTAACAAAGACGGCATCATATGGGTTGCAACTGAGGGAGCCTTGAATACACCAAAAGATCACATT-GGCACCCGCAATCCT
                                                                                                Section 132
                                      (11791) 11791    11800     11810     11820     11830     11840     11850     11860     11870     11880
               HERV- E (AB062274.1) (10682) GTGACCACT------ATCCTG--AGAGTATCTTCCAGGCTGAAGAGAGGGAAAGGGCT-CTCTGCAAGCAGGGA-CAGTGGCCTGGGCAA
SARS-CoV-2 (region 17170 - 29465) (11558) GCTAACAATGCTGCAATCGTGCTACAACTTCCTCAAGGAACAACATTGCCAAAAGGCTTCTACGCA-GAAGGGAGCAGAGGCGGCAGTCA
                                                                                                Section 133
                                      (11881) 11881    11890     11900     11910     11920     11930     11940     11950     11960     11970
               HERV- E (AB062274.1) (10762) AGGCACAGAGCATCAGAATCCACATAG---CCAGTGACCTACAGCTGCCTCTTCAGCTTCACCCTTTCAGCTCTACCTCCAGGGAGGCTG
SARS-CoV-2 (region 17170 - 29465) (11647) AGCCTCTTCTCGTTCCTCATCACGTAGTCGCAACAGTTCAAGAAATTCAACTCCAGG--CAGCAGTAGGGGAACTTCTCCTGCTAGAATG
```

Section 134

```
                                   (11971) 11971    11980     11990     12000     12010     12020     12030     12040     12050     12060
          HERV- E (AB062274.1)     (10849) GAGGGCAGCACAGGGGAGGGTG------------GG-TGTCAGAGCCCTGTGGCCCCCTGGGGACC----TGT---GTCCAAGCAGGGAC
SARS-CoV-2 (region 17170 - 29465) (11735) GCTGGCAATGGCGGTGATGCTGCTCTTGCTTTGCTGCTGCTTGACAGATTGAACCAGCTTGAGAGCAAAATGTCTGGTAAAGGCCAACAA
```

Section 135

```
                                   (12061) 12061    12070     12080     12090     12100     12110     12120     12130     12140     12150
          HERV- E (AB062274.1)     (10919) CTCCTCTGC--AGCTATGGCAGAGTTA---GCTTTAGAGTCTTCT------CTTCTTCCAGAGCCA--TGGAACT---GCAAACAGTACT
SARS-CoV-2 (region 17170 - 29465) (11825) CAACAAGGCCAAACTGTCACTAAGAAATCTGCTGCTGAGGCTTCTAAGAAGCCTCGGCAAAAACGTACTGCCACTAAAGCATACAATGTA
```

Section 136

```
                                   (12151) 12151    12160     12170     12180     12190     12200     12210     12220     12230     12240
          HERV- E (AB062274.1)     (10993) TCAAAACCCTGATCCACACAGGGATGCCTGCAGTCTTCCTGTCCTTTTC---ACCCTCTCTGGGTCA---AAGGCTTTGGCCTCAACCCT
SARS-CoV-2 (region 17170 - 29465) (11915) ACACAAGCTTTCGGCAGACGTGGTCCAGAACAAACCCAAGGAAATTTTGGGGACCAGGAACTAATCAGACAAGGAACTGATTACAAACAT
```

Section 137

```
                                   (12241) 12241    12250     12260     12270     12280     12290     12300     12310     12320     12330
          HERV- E (AB062274.1)     (11077) GATTCTCCCCCTCATCTCCTAGCCCCCTGCA----AGATTTGGGCAGGTCTTGCTGAACTAGAGGCCCAGGAGACATCCCATAGATACAG
SARS-CoV-2 (region 17170 - 29465) (12005) TGGCCGCAAATTGCACAATTTGCCCCCAGCGCTTCAGCGTTCTTCGGAATGTCGCGCATT---GGCATGGAAGTCACACCTTCGGGA-AC
```

Section 138

```
                                   (12331) 12331    12340     12350     12360     12370     12380     12390     12400     12410     12420
          HERV- E (AB062274.1)     (11163) GAGGCTG--CAT--CTGGAGACATCCCATAG-AT-ACAG-GAGGCTGAGGAAGGAGACATAGGCCTTTCCTTCCTGAGGCCCCAGGAGGG
SARS-CoV-2 (region 17170 - 29465) (12091) GTGGTTGACCTACACAGGTGCCATCAAATTGGATGACAAAGATCCAAATTTCAAAGATCAAGTCATTTTGCTGAATAAGCATATTGACGC
```

Section 139

```
                                   (12421) 12421    12430     12440     12450     12460     12470     12480     12490     12500     12510
          HERV- E (AB062274.1)     (11246) AGACAAGACA----CAAAGACACAGAC---AGAAGTCTCAGTGAATAGAGCAGAGGCCCTGAGAGCTGGGCAGCCAGAGAGGGCAGGAAG
SARS-CoV-2 (region 17170 - 29465) (12181) ATACAAAACATTCCCACCAACAGAGCCTAAAAAGGACAAAAAGAAGAAGGCTGATGAAACTCAAGCCTTACCGC-AGAGACAGAAGAAAC
```

Section 140

```
                                   (12511) 12511    12520              12538
          HERV- E (AB062274.1)     (11329) AGTGCAATTAGGCAATCAAGGCAAGCTT
SARS-CoV-2 (region 17170 - 29465) (12270) AGCAAACTGTGACTCTTCTTCCT-GCTG
```
