# Supplementary material for: Patch type nucleotide sequence identities between genomes from many different species facilitate illegitimate recombination
Source: Sci Rep. 2026 Mar 30;16:10524. doi: 10.1038/s41598-026-44124-0 (PMC13035915; doi:10.1038/s41598-026-44124-0)
Supplement: Supplementary file 2 — Supplementary Material 2 [file 41598_2026_44124_MOESM2_ESM.pdf]

```
                                                                                                             Section 1
                                                       (1)  1        10                  20                  30                  40                  50                  60                 74
Homo sapiens chromosome 1 NC_000001.11: 11783698-...   (1)  -----------CTGCCCTCTGAGG---CTGAGGTTGCAAGATG-GAAAGCCAGT-GCTTTCACCTGTTACTGGT
      SARS-CoV-2 Reference Genome NC_045512.2          (1)  ATTAAAGGTTTATACCTTCCCAGGTAACAAACCAACCAACTTTCGATCTCTTGTAGATCTGTTCTCTAAACGAA
                                                                                                             Section 2
                                                      (75)  75  80                  90                  100                 110                 120                 130                148
Homo sapiens chromosome 1 NC_000001.11: 11783698-...  (59)  CGTTTACTGTTTTGTGGGAG--ATT----TGCAGGTGTA----ACT----TTGAATAAATG-TGACTTTTGACT
      SARS-CoV-2 Reference Genome NC_045512.2         (75)  CTTTAAAATCTGTGTGGCTGTCACTCGGCTGCATGCTTAGTGCACTCACGCAGTATAATTAATAACTAATTACT
                                                                                                             Section 3
                                                     (149)  149         160                 170                 180                 190                 200                 210            222
Homo sapiens chromosome 1 NC_000001.11: 11783698-... (118)  CT-GAGAACGAGACAAGCCCTCC-CATGCATCTGTTTTTGTTTTGTTTCATTTTGTTTTTTTGAGATGGAGTTT
      SARS-CoV-2 Reference Genome NC_045512.2        (149)  GTCGTTGACAGGACACGAGTAACTCGTCTATCTTCTGCAGGCTGCTTACGGTTTCGTCCGTGTTGCAGCCGAT-
                                                                                                             Section 4
                                                     (223)  223    230                 240                 250                 260                 270                 280              296
Homo sapiens chromosome 1 NC_000001.11: 11783698-... (190)  CACTCTTGTCACCCAGGCTGGAGTGCACTTGCGCGATCTCGGCTTACTGCAACCTCTGCCTCCTGGGTTCAGGC
      SARS-CoV-2 Reference Genome NC_045512.2        (222)  CA-TCAGCACATCTAGGTTTC-GTCCGGGTGTGACCGAAAGGTAAGATGGAGAGCCTTGTCCCTGGTTTCAACG
                                                                                                             Section 5
                                                     (297)  297           310                 320                 330                 340                 350                 360        370
Homo sapiens chromosome 1 NC_000001.11: 11783698-... (264)  AATTCTTCTGCCTCAGACTCCCAAGTAGCTGGGATTACAGGTGC-CCAC-------TACCAGGCCT-GGCTAAT
      SARS-CoV-2 Reference Genome NC_045512.2        (294)  AGAAAACACACGTCCAACTC--AGTTTGCCTGTTTTACAGGTTCGCGACGTGCTCGTACGTGGCTTTGGAGACT
                                                                                                             Section 6
                                                     (371)  371      380                 390                 400                 410                 420                 430              444
Homo sapiens chromosome 1 NC_000001.11: 11783698-... (329)  TTTTGTAT----TTTTAATAGAGACGGGGTTTCACC---ACGTTGG----TCAGGCTGGTCTCAAACTGACTTC
      SARS-CoV-2 Reference Genome NC_045512.2        (366)  CCGTGGAGGAGGTCTTATCAGAGGCACGTCAACATCTTAAAGATGGCACTTGTGGCTTAGTAGAAGTTTGAAAA
                                                                                                             Section 7
                                                     (445)  445  450                 460                 470                 480                 490                 500                518
Homo sapiens chromosome 1 NC_000001.11: 11783698-... (392)  AGGTGATCCACTCACCTTGG-CTTCCCAAAGTGCTG------GGATTACAAGCATGAGCCACCACACCTGGCCC
      SARS-CoV-2 Reference Genome NC_045512.2        (440)  GGCGTTTTGCCTCAACTTGAACAGCCCTATGTGTTCATCAAACGTTCGGATGCTCGAACTGCACCTCATGGTC-
```

Section 8

```
                                                      (519) 519        530       540       550       560       570       580     592
Homo sapiens chromosome 1 NC_000001.11: 11783698-...  (459) AATGTGTCTGTTTTGCAGCTA------CTTCCAGGCCTGGTGTGAGGTTGTAA----GAGACACGGGG-GTGCT
SARS-CoV-2 Reference Genome NC_045512.2               (513) -ATGTTATGGTTGAGCTGGTAGCAGAACTCGAAGGCATTCAGTACGGTCGTAGTGGTGAGACACTTGGTGTCCT
```

Section 9

```
                                                      (593) 593    600       610       620       630       640       650           666
Homo sapiens chromosome 1 NC_000001.11: 11783698-...  (522) ---CC-------GGGCCCAGCATCAA-GCCTTTCAGGATGG-----CCTGAGGAGAAGAGCACTGGGGGCCGTG
SARS-CoV-2 Reference Genome NC_045512.2               (586) TGTCCCTCATGTGGGCGAAATACCAGTGGCTTACCGCAAGGTTCTTCTTCGTAAGAACGGTAATAAAGGAGCTG
```

Section 10

```
                                                      (667) 667          680       690       700       710       720       730     740
Homo sapiens chromosome 1 NC_000001.11: 11783698-...  (580) TTTGCCACATGGCGGGAAGCCCAGGAAGTGGCAGCCGGGGCACAGGAGCAGCGTG----TGGCCC-----AGGC
SARS-CoV-2 Reference Genome NC_045512.2               (660) GTGGCCATAGTTACGGC-GCCGATCTAAAGTCATTTGACTTAGGCGACGAGCTTGGCACTGATCCTTATGAAGA
```

Section 11

```
                                                      (741) 741     750       760       770       780       790       800         814
Homo sapiens chromosome 1 NC_000001.11: 11783698-...  (645) CTCCCTTGCCCGCTGGAGAAGCTGCGGGCAGCAAGG------CCAG-GAAGATGGGCAGCAGAAGAAGGCCCGG
SARS-CoV-2 Reference Genome NC_045512.2               (733) TTTTCAAGAAAACTGGAACA-CTAAACATAGCAGTGGTGTTACCCGTGAACTCATGCGTGAGCTTAACGGAGGG
```

Section 12

```
                                                      (815) 815  820       830       840       850       860       870             888
Homo sapiens chromosome 1 NC_000001.11: 11783698-...  (712) GCCC-CACAGGCCTTCCC---AGCATGGCCAGTGGCCCCG--GGCATGCACCATGAGGCCCAGCAGCAGGCAGG
SARS-CoV-2 Reference Genome NC_045512.2               (806) GCATACACTCGCTATGTCGATAACAACTTCTGTGGCCCTGATGGCTACCCTCTTGAGTGCATTAAAGACCTTCT
```

Section 13

```
                                                      (889) 889        900       910       920       930       940       950     962
Homo sapiens chromosome 1 NC_000001.11: 11783698-...  (780) AGAGAGCGCTGG----GGCCCAGGCA----GCCC--AGTGCTGGACTTGGTGCTGGGCTCTGTGGGTGCATGAG
SARS-CoV-2 Reference Genome NC_045512.2               (880) AGCACGTGCTGGTAAAGCTTCATGCACTTTGTCCGAACAACTGGACTTTATTGACACTAAGAGGGGTGTAT-AC
```

Section 14

```
                                                      (963) 963    970       980       990       1000      1010      1020       1036
Homo sapiens chromosome 1 NC_000001.11: 11783698-...  (844) TCCTGTCGGGGCCAGGT-CAGCCGAGCCCATGCTTCCTGGAAGCCGC------GAGCC------TGGTGAGTGC
SARS-CoV-2 Reference Genome NC_045512.2               (953) TGCTGCCGTGAACATGAGCATGAAATTGCTTGGTACACGGAACGTTCTGAAAAGAGCTATGAATTGCAGACACC
```

Section 15

	(1037)	1037 1050 1060 1070 1080 1090 1100 1110
Homo sapiens chromosome 1 NC_000001.11: 11783698-...	(905)	TGTGG---TCTAAGTGCAGCCCCACTCTGTG--CTTCCA--GCTCAGGCCTCAAGCCTGTTTTTGGCAGGGGTA
SARS-CoV-2 Reference Genome NC_045512.2	(1027)	TTTTGAAATTAAATTGGCAAAGAAATTTGACACCTTCAATGGGGAATGTCCAAATTTTGTATTTCCCTTAAATT

Section 16

	(1111)	1111 1120 1130 1140 1150 1160 1170 1184
Homo sapiens chromosome 1 NC_000001.11: 11783698-...	(972)	GAGCGTAATTCATGGCTGGGTGGC--AGGGCAAAGGCTCAAGAGGGTGGTTGGGGGTGACAGGGAGAGGCCGCC
SARS-CoV-2 Reference Genome NC_045512.2	(1101)	C--CATAAT-CAAGACTATTCAACCAAGGGTTGAAAAGAAAAAGCTTGAT--GGCTTTATGGGTAGAATTCGAT

Section 17

	(1185)	1185 1190 1200 1210 1220 1230 1240 1258
Homo sapiens chromosome 1 NC_000001.11: 11783698-...	(1044)	CTGGCCATCTAGTCCTGGGCCACATCCTGACACCGCCTCTTGCTGTGTGTCCCCTCCTTGGGGAAGTCTCTAAT
SARS-CoV-2 Reference Genome NC_045512.2	(1170)	CTGTCTATCCAGT--TGCGTCACCAAATGAATGCAACCAAATGTGCCTTTCAACTC--TCATGAAGTGT-----

Section 18

	(1259)	1259 1270 1280 1290 1300 1310 1320 1332
Homo sapiens chromosome 1 NC_000001.11: 11783698-...	(1118)	GGTCATGCCTTGCAGTCACCAGCACAGCCAGCC-ACTCAGCAAGCGCCATAAACGCGCTCT-TGCCA--GGGAA
SARS-CoV-2 Reference Genome NC_045512.2	(1235)	GATCATTGTGGTGAAACTTCATGGCAGACGGGCGATTTTGTTAAAGCCACTTGCGAATTTTGTGGCACTGAGAA

Section 19

	(1333)	1333 1340 1350 1360 1370 1380 1390 1406
Homo sapiens chromosome 1 NC_000001.11: 11783698-...	(1188)	GGGGAGGCAAAGTGGCACTGGCAGGACTGTCCCCTCTCTGCCCCTAAACAGGATGCTTGTCCCCGTCTGTGATT
SARS-CoV-2 Reference Genome NC_045512.2	(1309)	TTTGACTAAAGAAGG---TGCCACTACT-TGTGGTTACTTACCCCAAA----ATGCTGTT----GTTAAAATTT

Section 20

	(1407)	1407 1420 1430 1440 1450 1460 1470 1480
Homo sapiens chromosome 1 NC_000001.11: 11783698-...	(1262)	TTCTCCTTCCAGGTCGCTCTCCATCCTGTTTACCC-AGATTCTCCCTCCAGAAAAGGGAGTGTGGGAGGTGGGT
SARS-CoV-2 Reference Genome NC_045512.2	(1371)	ATTGTCCAGCATGTCACAATTCAGAAGTAGGACCTGAGCATAGTCTTGCCGAATACC-ATAATGAATCTGGCTT

Section 21

	(1481)	1481 1490 1500 1510 1520 1530 1540 1554
Homo sapiens chromosome 1 NC_000001.11: 11783698-...	(1335)	GGGCCCCAGCCTGGGGCTGGGCCCGGAAGGCCCCCTCCCGCAGGGCACTGGGGGGGCTCCC-TGCAGAGAGTCC
SARS-CoV-2 Reference Genome NC_045512.2	(1444)	GAAAACCATTCTTCGTAAGGGT--GGTCGCACTATTGCCTTTGGAGGCTGTGTGTTCTCTTATGTTGGTTGCCA

Section 22

```
                                                        (1555) 1555 1560      1570      1580      1590      1600      1610           1628
Homo sapiens chromosome 1 NC_000001.11: 11783698-...  (1408) TGGCCTTTATGGCCCT-GGCTGCAGACTCCTTCCTCTCCCGCAGGGT-----CCTAGAGGCCTCGGTGCAGTCG
        SARS-CoV-2 Reference Genome NC_045512.2  (1516) TAACAAGTGTGCCTATTGGGTTCCACGTGCTAGCGCTAACATAGGTTGTAACCATACAGGTGTTGTTGGAGAAG
```

Section 23

```
                                                        (1629) 1629      1640      1650      1660      1670      1680      1690      1702
Homo sapiens chromosome 1 NC_000001.11: 11783698-...  (1476) GCGGTGCGCGGCGGTGTCCA-GCGAGCCATCCTCACCCAGCTCCGGCCGGCTGAGCTCAGGCGC----TTCCTG
        SARS-CoV-2 Reference Genome NC_045512.2  (1590) G---TTC-CGAAGGTCTTAATGACAACCTTCTTGAAATA-CTCCAAAAAGAGAAAGTCAACATCAATATTGTTG
```

Section 24

```
                                                        (1703) 1703   1710      1720      1730      1740      1750      1760          1776
Homo sapiens chromosome 1 NC_000001.11: 11783698-...  (1545) CGGACAGTGCAGCTCAGGGTGCGGCTGGGACTGCCAGGGGCCGGCAAGGTACG-CCCCAAGCCCCAGACTGACC
        SARS-CoV-2 Reference Genome NC_045512.2  (1659) GTGACTTTAAACTTAATGAAGAGATCGCCATTATTTTGGCATCTTTTTCTGCTTCCACAAGTGCTTTTGTGGAA
```

Section 25

```
                                                        (1777) 1777         1790      1800      1810      1820      1830      1840   1850
Homo sapiens chromosome 1 NC_000001.11: 11783698-...  (1618) TCACGCTCTGGCCCCGGATGGCCAGCAGGGGGAGCCTGCTGCTGGACGCCCCAGCCCCTTGGAAACAGGTGAAG
        SARS-CoV-2 Reference Genome NC_045512.2  (1733) ACTGTGAAAGGTTTGGATTATAAAGCATTCAAA--CAAATTGTTGAATCCTGTGGTAATTTTAAAGTTACAAAA
```

Section 26

```
                                                        (1851) 1851     1860      1870      1880      1890      1900      1910          1924
Homo sapiens chromosome 1 NC_000001.11: 11783698-...  (1692) GGAGC--CTAGGCAGGGCGGGAGGTCCAAAAATGACC----CTCGGACCACCGGCCTTAGAATCAGCTTGATTC
        SARS-CoV-2 Reference Genome NC_045512.2  (1805) GGAAAAGCTAAAAAAGGTGCCTGGAATATTGGTGAACAGAAATCAATACTGAGTCCTCTTTATGCATTTGCATC
```

Section 27

```
                                                        (1925) 1925 1930      1940      1950      1960      1970      1980           1998
Homo sapiens chromosome 1 NC_000001.11: 11783698-...  (1760) AGCCGGTGGTGGCAGCATGGCTGGATGCCTCCCCAGGGGCCCAGCAACCTCGTATGCACCACCTCTCCCCAGTT
        SARS-CoV-2 Reference Genome NC_045512.2  (1879) AGAGGCTGCTCGTGTTGTACGATCAATTTTCTCCCGCACTCTTGAAACTGCTCA---AAATTCTGTGCGTGTTT
```

Section 28

```
                                                        (1999) 1999     2010      2020      2030      2040      2050      2060          2072
Homo sapiens chromosome 1 NC_000001.11: 11783698-...  (1834) CTCAGTCAGCCTCTGACAAGCAACTCTGAATCCATCAGCAAAGATGAACCTGGCACCTCTCGAGTGCCA----G
        SARS-CoV-2 Reference Genome NC_045512.2  (1950) TACAGAAGGCCGCT-ATAA-CAATACTAGATGGAATTTCACAGTATTCACTGAGACTCATTGA-TGCTATGATG
```

```
                                                                  Section 29
                                               (2073) 2073   2080      2090      2100      2110      2120      2130          2146
Homo sapiens chromosome 1 NC_000001.11: 11783698-... (1904) GCCACTGCAGGTCCGGCCGCAAAGACCAGCATGTTGGTCACAGCCTGTTAGGGAGACATTGAAATGCATGCTT
      SARS-CoV-2 Reference Genome NC_045512.2 (2021) TTCACATCTGATTTGGCTACTAACA--ATCTAGTTG--TAATGGCCTACATTACAGGTGGTGTTGTTCAGTTGA
                                                                  Section 30
                                               (2147) 2147         2160      2170      2180      2190      2200      2210      2220
Homo sapiens chromosome 1 NC_000001.11: 11783698-... (1978) TCTCACGGTGCAGTTGAGGATCAGAATAGGTACACGAGGACAGTAGCATTTTTTTGTCAAGTTTTTTTGTTTAT
      SARS-CoV-2 Reference Genome NC_045512.2 (2091) CTTCGCAGTG--GCTAACTAACATCTTTGGCACTGTTTATGAAAAACTCAAACCCGTCC-------TTGATTGG
                                                                  Section 31
                                               (2221) 2221     2230      2240      2250      2260      2270      2280          2294
Homo sapiens chromosome 1 NC_000001.11: 11783698-... (2052) TTTTGAGACAGAGTCTGGCTCAATTGCCCAGGCTGAAGCAGAGGAGTGATCTCAGCTCACTGCAACCTCTGCCT
      SARS-CoV-2 Reference Genome NC_045512.2 (2156) CTTGAAGAGA-AGTTTAAGGAAGGTGTAGAGTTTCTTAGAGACGGTTGGGAAATTGTTAAATTTATCTCAACCT
                                                                  Section 32
                                               (2295) 2295 2300      2310      2320      2330      2340      2350              2368
Homo sapiens chromosome 1 NC_000001.11: 11783698-... (2126) CCCGGGTTCAAGTGATTCTCCCGCCTCAGCTT----CCTGAGTAGCTGGGACTACA-AGTGCACGCCACCACGC
      SARS-CoV-2 Reference Genome NC_045512.2 (2229) ---GTGCTTGTGAAATTGTCGGTGGACAAATTGTCACCTGTGCAAAGGAAATTAAGGAGAGTGTTCAGACATTC
                                                                  Section 33
                                               (2369) 2369       2380      2390      2400      2410      2420      2430      2442
Homo sapiens chromosome 1 NC_000001.11: 11783698-... (2195) CT--GGCTAATTTTTGTATTTTTAG--TAGAGA-CGGGGTTTCACCATATTGCCCAAGCTGGTCTCGAACTCCT
      SARS-CoV-2 Reference Genome NC_045512.2 (2300) TTTAAGCTTGTAAATAAATTTTTGGCTTTGTGTGCTGACTCTATCATTATTGGTGGAGCTAAACTTAAAGCCTT
                                                                  Section 34
                                               (2443) 2443   2450      2460      2470      2480      2490      2500          2516
Homo sapiens chromosome 1 NC_000001.11: 11783698-... (2264) GAACTCAAGTGATCCTCCCACCTCGACTTCCCAAAGTGCTGGGATTACAGGTGTG----AGCCACCGCGCCA--
      SARS-CoV-2 Reference Genome NC_045512.2 (2374) GAATTTAGGTGAAACATTTGTCACGCACTC--AAAGGGATTGTACAGAAAGTGTGTTAAATCCAGAGAAGAAAC
                                                                  Section 35
                                               (2517) 2517         2530      2540      2550      2560      2570      2580      2590
Homo sapiens chromosome 1 NC_000001.11: 11783698-... (2332) -GGCCAAGTTTTTTTTTTCTTATTGGAAAAGTGAAATATGCTTATTACAG---AAACCTCAGAAAATATAAAG
      SARS-CoV-2 Reference Genome NC_045512.2 (2446) TGGCCTACTCATGCCTCTAAAAGCCCCAAAAG--AAATTATCTTCTTAGAGGGAGAAACACTTCCCACAGAA-G
```

Section 36

	(2591)	2591 2600 2610 2620 2630 2640 2650 2664
Homo sapiens chromosome 1 NC_000001.11: 11783698-...	(2402)	TAGAAAAATACCAAAATTTCTTTCATAATTCTC-TCTAC--CCAAAGGCATCGGTCAGTCCCTCTCCAGACCAG
SARS-CoV-2 Reference Genome NC_045512.2	(2517)	TGTTAACAGAGGAAGTTGTCTTGAAAACTGGTGATTTACAACCATTAGAACAACCTACTAGTGAAGCTGTTGAA

Section 37

	(2665)	2665 2670 2680 2690 2700 2710 2720 2738
Homo sapiens chromosome 1 NC_000001.11: 11783698-...	(2473)	AAGCAGTTAGTTCTGACACCAACAAGTGGTGATAAG---AGGTTGATAGCCTAGCAAGGGGGAAGAAGCCACCA
SARS-CoV-2 Reference Genome NC_045512.2	(2591)	GCTCCATTGGTTGGTACACCAGTTTGTATTAACGGGCTTATGTTGCTCGAA-ATCAAAGACACAGAAAAGTACT

Section 38

	(2739)	2739 2750 2760 2770 2780 2790 2800 2812
Homo sapiens chromosome 1 NC_000001.11: 11783698-...	(2544)	CCAAACCAAACACAAAAAATGACACTGCACAGCAGTCTGGGGACATGTCCTTGAAGGACACAGCCCCAAAGATA
SARS-CoV-2 Reference Genome NC_045512.2	(2664)	GTGC-CCTTGCACCTAATATGATGGTAACAAACAATACCTTCACA----CTCAAAGG-CGGTGCACCAACAAAG

Section 39

	(2813)	2813 2820 2830 2840 2850 2860 2870 2886
Homo sapiens chromosome 1 NC_000001.11: 11783698-...	(2618)	CTGGCAGGTGAGGTAGTGTCCCTAAGATCAACAGGCATCATCATGAATCCTATTTCAGAATGCCACAAAGAATA
SARS-CoV-2 Reference Genome NC_045512.2	(2732)	GTTACTTTTG--GTGATGACACTGTGATAGAAGTGCAAGGTTAC-AAGAGTGT----GAATATCACTTTTGA-A

Section 40

	(2887)	2887 2900 2910 2920 2930 2940 2950 2960
Homo sapiens chromosome 1 NC_000001.11: 11783698-...	(2692)	CGTTTTAGAGAAGATTCTAAGAGAA--AAAGCTAAGGTGATTTTTTTTTTCAGTCGTTTTTTGTTCTCAGACAG
SARS-CoV-2 Reference Genome NC_045512.2	(2798)	CTTGAT-GAAAGGATTGATAAAGTACTTAATGAGAAGTGCTCTGCCTATACAGTTGAACTCGGTAC--AGA-AG

Section 41

	(2961)	2961 2970 2980 2990 3000 3010 3020 3034
Homo sapiens chromosome 1 NC_000001.11: 11783698-...	(2764)	TGTCTCACTCTGTCGCCCAGGCCAGAGTGCAG-TGGTGCGATCACAGCTCACTGCAGCCTCAACCTCCAGGGCT
SARS-CoV-2 Reference Genome NC_045512.2	(2868)	TAAATGAGT---TCGCCTGTGTT-GTG-GCAGATGCTGTCATAAAAACTT--TGCAACCAGTATCTGAATTACT

Section 42

	(3035)	3035 3040 3050 3060 3070 3080 3090 3108
Homo sapiens chromosome 1 NC_000001.11: 11783698-...	(2837)	CAAACGATCCTCCCACCTCAGCCACCTGAGTAGC-TGAGACTACAGGC---GTGTGCCACCACACCCGG----C
SARS-CoV-2 Reference Genome NC_045512.2	(2935)	TACACCA--CTGGGCATTGATTTAGATGAGTGGAGTATGGCTACATACTACTTATTTGATGAGTCTGGTGAGTT

```
                                                    Section 43
                                                (3109) 3109       3120      3130      3140      3150      3160      3170      3182
Homo sapiens chromosome 1 NC_000001.11: 11783698-... (2903) TAATTTT--TTTT--TTTTT--TTTTTTGTAGAAATGGGATCTCACTATGTTGCC-AGGGCTTGTCTCAAACTC
      SARS-CoV-2 Reference Genome NC_045512.2 (3007) TAAATTGGCTTCACATATGTATTGTTCTTTCTACCCTCCAGATGAGGATGAAGAAGAAGGTGATTGTGAAGAAG
                                                    Section 44
                                                (3183) 3183   3190      3200      3210      3220      3230      3240      3256
Homo sapiens chromosome 1 NC_000001.11: 11783698-... (2970) CTGGGCTCAAGCGATCTACC-----TGCCT-TGGCCTCCCAAAGCATTGGGCAGGCCCAT-TACGCCATGAGCC
      SARS-CoV-2 Reference Genome NC_045512.2 (3081) AAGAGTTTGAGCCATCAACTCAATATGAGTATGGTACTGAAGATGATTACCAAGGTAAACCTTTGGAATTTGGT
                                                    Section 45
                                                (3257) 3257         3270      3280      3290      3300      3310      3320      3330
Homo sapiens chromosome 1 NC_000001.11: 11783698-... (3037) ACCACACCTGCTGGTTTTTGTTTTTTAAATGGAATTGAAAGGAACTAGCCTTGGCTAGTTGCAGA-ATTCACAC
      SARS-CoV-2 Reference Genome NC_045512.2 (3155) GCCACTTCTGCTGCTCTTCAACCTGAA----GAAGAGCAAG-AAGAAGA-TTGGTTAGATGATGATAGTCAACA
                                                    Section 46
                                                (3331) 3331     3340      3350      3360      3370      3380      3390      3404
Homo sapiens chromosome 1 NC_000001.11: 11783698-... (3110) ATATGGGGCTATGGCAACAGCAGATTCTGGAGTCTCAGGGT-CTTTTCTTTCTAGATAAGTTACAGGGCAAT-T
      SARS-CoV-2 Reference Genome NC_045512.2 (3223) AACTGTTGGTCAA-CAAGACGGCAGTGAGGACAATCAGACAACTACTATTCAAACAATTGTTGAGGTTCAACCT
                                                    Section 47
                                                (3405) 3405 3410      3420      3430      3440      3450      3460          3478
Homo sapiens chromosome 1 NC_000001.11: 11783698-... (3182) GCTTTTCAGA--GTACTTTTAC-ATTTCT--ATTCCATTCTTGTCAGTAAAGACAA-GGATCCCATAAGCTGAT
      SARS-CoV-2 Reference Genome NC_045512.2 (3296) CAATTAGAGATGGAACTTACACCAGTTGTTCAGACTATTGAAGTGAATAGTTTTAGTGGTTATTTAAAACTTAC
                                                    Section 48
                                                (3479) 3479       3490      3500      3510      3520      3530      3540      3552
Homo sapiens chromosome 1 NC_000001.11: 11783698-... (3250) GGAGAAACTGAGGCCTCTAGAAACTAAACGCTGGGAATGGCACCCAGGGTGCTGAATTCCAGGGTC-TCCTCTT
      SARS-CoV-2 Reference Genome NC_045512.2 (3370) TGACAATGT-ATACATTAAAAATGCAGACATTGTGGAAGA-AGCTAAAAAGGTAAAACCAACAGTGGTTGTTAA
                                                    Section 49
                                                (3553) 3553   3560      3570      3580      3590      3600      3610      3626
Homo sapiens chromosome 1 NC_000001.11: 11783698-... (3323) TTCTGCCACAGGCACACATGCCCCAGCAGCCCCTTTGGAGAGCCCCCTCTCTATAGGGTACTAGCCGGGGGCTC
      SARS-CoV-2 Reference Genome NC_045512.2 (3442) TGCAGCCAATGTTTACCTTAAACATGGAGGAGGTGTTGCAGGAGCCTTAAATA-AGGCTACTAACAATGCCATG
```

Section 50

```
                                                        (3627) 3627         3640      3650      3660      3670      3680      3690      3700
Homo sapiens chromosome 1 NC_000001.11: 11783698-...   (3397) AGACTCTGGAGTCAGACGGCTTCCGAGGGATGAGGCCTCAGACAAGTAACTCAGCTTTGAAATGTTTTCAGTGG
SARS-CoV-2 Reference Genome NC_045512.2                (3515) CAAGTT--GAATCTGATGATTACATAGCTACTAATGGAC---CACTTAAAGTGGGTGGTAGTTGTGTTTTAAGC
```

Section 51

```
                                                        (3701) 3701     3710      3720      3730      3740      3750      3760          3774
Homo sapiens chromosome 1 NC_000001.11: 11783698-...   (3471) GGAGAGAAATAGCCCCCTCCCAGGGCTGTGCTCCGGGAA-CTGACTGAGGGCTAACTATTATGGGCCCACCCCA
SARS-CoV-2 Reference Genome NC_045512.2                (3584) GGACACAAT-----CTTGCTAAACACTGTCTTCATGTTGTCGGCCCAAATGTTAACAAAGGTGAAGACATTCAA
```

Section 52

```
                                                        (3775) 3775 3780      3790      3800      3810      3820      3830              3848
Homo sapiens chromosome 1 NC_000001.11: 11783698-...   (3544) TTGTTGGAATGTCTTCAGCCACATGGGGGAATGGGTGGAAGAGG--CCGGGATGTGTCCTGCCAGCTAG-AGCA
SARS-CoV-2 Reference Genome NC_045512.2                (3653) CTTCTTAAGAGTGCTTATGAAAATTTTA-ATCAGCACGAAGTTCTACTTGCACCATTATTATCAGCTGGTATTT
```

Section 53

```
                                                        (3849) 3849       3860      3870      3880      3890      3900      3910        3922
Homo sapiens chromosome 1 NC_000001.11: 11783698-...   (3615) GCGAAGAAATCCCAGCGGGGCCCCAGGAAGTCCAAGCCCATGGGGTTCAGGTGCTCCTCTCTGGCTAT---TCC
SARS-CoV-2 Reference Genome NC_045512.2                (3726) TTGGTGCTGACCCTATACATTCTTTAAGAGTTTGTGTAGATACTGTTC--GCACAAATGTCTACTTAGCTGTCT
```

Section 54

```
                                                        (3923) 3923   3930      3940      3950      3960      3970      3980            3996
Homo sapiens chromosome 1 NC_000001.11: 11783698-...   (3686) TTCAGACCCGCAGCTGCTGGACACAGGCCACAGAGCTGGTGCCTCCCGCGCCATCACTGCA-GTGCAGCCTGGG
SARS-CoV-2 Reference Genome NC_045512.2                (3798) TTGATAAAAATCTCTATGACAAACTTGTTTCAAGCTTTTTGGAAATGAAGAGTGAAAAGCAAGTTGAACAAAAG
```

Section 55

```
                                                        (3997) 3997         4010      4020      4030      4040      4050      4060      4070
Homo sapiens chromosome 1 NC_000001.11: 11783698-...   (3759) TGGACGGAGGAAGCCAAGGGGAACG----GCCTGGGCTCAGAGTAAGGAGACCTTGCCCCG----GGGGACAAA
SARS-CoV-2 Reference Genome NC_045512.2                (3872) ATCGCTGAGATTCCTAAAGAGGAAGTTAAGCCATTTATAACTGAAAGTAAACCTTCAGTTGAACAGAGAAAACA
```

Section 56

```
                                                        (4071) 4071     4080      4090      4100      4110      4120      4130          4144
Homo sapiens chromosome 1 NC_000001.11: 11783698-...   (3825) CGGTG-TCTGAAGTGCA------GGGGTGAAGCGGTCTCCAGTCCTCAGGGGCCTGGAAATCAGCTCCTTGG-G
SARS-CoV-2 Reference Genome NC_045512.2                (3946) AGATGATAAGAAAATCAAAGCTTGTGTTGAAGAAGTTACAACAACTCTGGAA---GAAACTAAGTTCCTCACAG
```

Section 57

```
                                                  (4145) 4145 4150      4160      4170      4180      4190      4200           4218
Homo sapiens chromosome 1 NC_000001.11: 11783698-... (3891) ACACGGTCTTA-TGTGTATTGAC--------CATTCT----CCCCATCCATTCCCTCTTCGGAATG----TTTC
  SARS-CoV-2 Reference Genome NC_045512.2 (4017) AAAACTTGTTACTTTATATTGACATTAATGGCAATCTTCATCCAGATTCTGCCACTCTTGTTAGTGACATTGAC
```

Section 58

```
                                                  (4219) 4219 4230 4240 4250 4260 4270 4280 4292
Homo sapiens chromosome 1 NC_000001.11: 11783698-... (3948) AGCTGTT-CTGAGA-AAACACTTTGCA--T--------TCCTGCC--CCGCCCCTGTGATTTCTCTTTGGGCGG
  SARS-CoV-2 Reference Genome NC_045512.2 (4091) ATCACTTTCTTAAAGAAAGATGCTCCATATATAGTGGGTGATGTTGTTCAAGAGGGTGTTTTAACTGCTGTGGT
```

Section 59

```
                                                  (4293) 4293 4300 4310 4320 4330 4340 4350 4366
Homo sapiens chromosome 1 NC_000001.11: 11783698-... (4008) CAGGCAGCCGGGAGAGGCTGGAGGC-CTGGGGAGATGGGGGC-AGGGCACAAGGCTAAGCTC-AGCA--CCTTC
  SARS-CoV-2 Reference Genome NC_045512.2 (4165) TATACCTACTAAAAAGGCTGGTGGCACTACTGAAATGCTAGCGAAAGCTTTGAGAAAAGTGCCAACAGACAATT
```

Section 60

```
                                                  (4367) 4367 4380 4390 4400 4410 4420 4430 4440
Homo sapiens chromosome 1 NC_000001.11: 11783698-... (4077) CTCCACCCTCCTCACTGTACTCAACTCCTAGGCGGGCACTAAGAGCAGCTGCCTTCCTGGACCCACCTTAACCT
  SARS-CoV-2 Reference Genome NC_045512.2 (4239) ATATAACCACTTACCCGGG-TCAGGGTTTAAATGGTTACACTGTAGAGGAGGCAAA---GACAGTGCTTAAAAA
```

Section 61

```
                                                  (4441) 4441 4450 4460 4470 4480 4490 4500 4514
Homo sapiens chromosome 1 NC_000001.11: 11783698-... (4151) CTTGTGACTCAACTCCCCTTTCCAGGGTGCAGGGAACATTCCCTCTGCCCTGCCTTCCAGCTCTGGCCACAGTG
  SARS-CoV-2 Reference Genome NC_045512.2 (4309) GT-GTAA---AAGTGCCTTTTACATTCTACCATCTATTATCTCTAATGAGAAGCAAGAAATTCTTGGAACTGTT
```

Section 62

```
                                                  (4515) 4515 4520 4530 4540 4550 4560 4570 4588
Homo sapiens chromosome 1 NC_000001.11: 11783698-... (4225) GCCTGGACAGAGTAGCTGGGTCCCAGGCCTGCCCCTGTGGACGAGGGACCAGGGACCAAGAGCGCACTCCAGCC
  SARS-CoV-2 Reference Genome NC_045512.2 (4379) TCTTGGA------ATTTGCGAGAAATGCTTGCACATGCAGAAGA---AACACGCAA-ATTAATGCCTGTCTGTG
```

Section 63

```
                                                  (4589) 4589 4600 4610 4620 4630 4640 4650 4662
Homo sapiens chromosome 1 NC_000001.11: 11783698-... (4299) CTGAGCCCAGAGCC-TGCAAAGCCCAAAGCAAGGCCCATAAACGGAGGCTACGGTAAGAGGAGCTGTGTGTGCA
  SARS-CoV-2 Reference Genome NC_045512.2 (4443) TGGAAACTAAAGCCATAGTTTCAACTATACAGCGTAAATATAAGGGTATTAAAATACAAGAGGGTGTGGTTG-A
```

```
                                                               Section 64
                                          (4663) 4663   4670      4680      4690      4700      4710      4720         4736
Homo sapiens chromosome 1 NC_000001.11: 11783698-... (4372) GTTTGGTGGGTCCGAGGGATGGGGGCAAGGGCTGAGGGGCTTGCCTGAGCCATGGCTACAG-CTGGGCCCTCTC
SARS-CoV-2 Reference Genome NC_045512.2             (4516) TTATGGTGC-TAGATTTTACTTTTACACCAGTAAAACAACTGTAGCGTCACTTATCAACACACTTAACGATCTA
                                                               Section 65
                                          (4737) 4737         4750      4760      4770      4780      4790      4800    4810
Homo sapiens chromosome 1 NC_000001.11: 11783698-... (4445) TGGCCAGTGCTCGTTCCTGCAGGATCCTGGAAAAGCAGGCCCAGGCCCATGGCTCTGCCCTCCTTCTGGCCCTG
SARS-CoV-2 Reference Genome NC_045512.2             (4589) AATGAAACTCTTGTTACAATGCCA-CTTGGCTATGTAACACATGGCTTAAATTTGGAAGAAGCTGCTCGGTATA
                                                               Section 66
                                          (4811) 4811     4820      4830      4840      4850      4860      4870         4884
Homo sapiens chromosome 1 NC_000001.11: 11783698-... (4519) AAGGGTCACGATGCTCTTGGCCATCAGGAGGA--AGTACCTGCAGC----GCCGGTGCCTCGAGGCACTGCTTC
SARS-CoV-2 Reference Genome NC_045512.2             (4662) TGAGATCTCT---CAAAGTGCCAGCTACAGTTTCTGTTTCTTCACCTGATGCTGTTACAGCGTATAA-TGGTTA
                                                               Section 67
                                          (4885) 4885 4890      4900      4910      4920      4930      4940            4958
Homo sapiens chromosome 1 NC_000001.11: 11783698-... (4587) ACGGGCTGCGGGGTTCCCAGCAGGCCAGGTGCCTGGCAGTGGCATGGCAGCACTGGGTGGATGCCCAAGGGGCA
SARS-CoV-2 Reference Genome NC_045512.2             (4732) TCTTACTTCTTCTTCTAAAACACCTGAAGAACATTTTATTGAAACCATCTCACTTGCTGGTTCCTATAAAGATT
                                                               Section 68
                                          (4959) 4959       4970      4980      4990      5000      5010      5020      5032
Homo sapiens chromosome 1 NC_000001.11: 11783698-... (4661) GAGCAGCTGGTGCGGACCCTGGTGAG-TGGGTGCACCCCT-CTCCACAC---TGACCATCTCCCATTTCAC-CT
SARS-CoV-2 Reference Genome NC_045512.2             (4806) GGTCCTATTCTGGACAATCTACACAACTAGGTATAGAATTTCTTAAGAGAGGTGATAAAAGTGTATATTACACT
                                                               Section 69
                                          (5033) 5033   5040      5050      5060      5070      5080      5090         5106
Homo sapiens chromosome 1 NC_000001.11: 11783698-... (4729) CATACTTAACCCAAACCCCTCTCAAA------AGTATGGCCCTTGGACCTACTGACTCCAAAACCTTGGGGGAC
SARS-CoV-2 Reference Genome NC_045512.2             (4880) AGTAATCCTACCACATTCCACCTAGATGGTGAAGTTATCACCTTTGACAATCTTAAGACACTTCTTTCTTTGAG
                                                               Section 70
                                          (5107) 5107         5120      5130      5140      5150      5160      5170    5180
Homo sapiens chromosome 1 NC_000001.11: 11783698-... (4797) AGGCCC-AGGAATCTGCAT-TCTTAATACTCCTCAGATGACACCC-CCTTGCCATAGCCCTTTCACTCTGGTGC
SARS-CoV-2 Reference Genome NC_045512.2             (4954) AGAAGTGAGGACTATTAAGGTGTTTACAACAGT-AGACAACATTAACCTCCACACGCAAGTTGTGGACATGTCA
```

Section 71

```
                                                  (5181) 5181     5190      5200      5210      5220      5230      5240          5254
Homo sapiens chromosome 1 NC_000001.11: 11783698-... (4868) AGCAGTGTCGGGGG--AGAAAGGCAGAAGGGCTGGGGACTGCGGGGGAGCGTGAGCACAAGAGGCCTTCTCTGC
SARS-CoV-2 Reference Genome NC_045512.2             (5027) ATGACATATGGACAACAGTTTGGTCCAACTTATTTGGA-TGGAGCTGATGTTACTAAAATAAAACCTCATAATT
```

Section 72

```
                                                  (5255) 5255 5260      5270      5280      5290      5300      5310            5328
Homo sapiens chromosome 1 NC_000001.11: 11783698-... (4940) CAACTGTCCCCAAAGCTCAGGGAGTGGCACCTGA-GATGGGCCTGGCAGACGTGGTGGCAGCGGA-TCCTGCGA
SARS-CoV-2 Reference Genome NC_045512.2             (5100) CACATGAAGGTAAAACATTTTATGTTTTACCTAATGATGACACTCTACGT-GTTGAGGCTTTTGAGTACTACCA
```

Section 73

```
                                                  (5329) 5329       5340      5350      5360      5370      5380      5390      5402
Homo sapiens chromosome 1 NC_000001.11: 11783698-... (5012) CTGCGAGTGGCTCAGCAGTTACAGCAGCAGGAAGATGGGTGGGTCCTTTCCCAGGTACT-GCCCTCTTCCCTGC
SARS-CoV-2 Reference Genome NC_045512.2             (5173) CA-CAACTGATCCT--AGTTTTCTGGGTAGGTACATGTCAGCATTAAATCACACTAAAAAGTGGAAATACCCAC
```

Section 74

```
                                                  (5403) 5403   5410      5420      5430      5440      5450      5460          5476
Homo sapiens chromosome 1 NC_000001.11: 11783698-... (5085) ATTCCACTCAGCCAGCCTTCCAGAAGTCTGTGCCACAGCCACGCACCGTGGAGCTTTCC-CTGC-TTGCCAGCC
SARS-CoV-2 Reference Genome NC_045512.2             (5244) AAGTTAATGGTTTAACTTCTATTAAATGGG-----CAGATAACAACTGTT-ATCTTGCCACTGCATTGTTAACA
```

Section 75

```
                                                  (5477) 5477          5490      5500      5510      5520      5530      5540    5550
Homo sapiens chromosome 1 NC_000001.11: 11783698-... (5157) CCGGGGCCCCTTCGTTTTCAGGGGAGGGGGTCAGTTCCATTA----CAGAACACACAGGCTGGATGCTGGAGCC
SARS-CoV-2 Reference Genome NC_045512.2             (5312) CTCCAACAAATAGAGTTGAAGTTTAATCCACCTGCTCTACAAGATGCTTATTACAGAGCAAGG--GCTGGTGAA
```

Section 76

```
                                                  (5551) 5551     5560      5570      5580      5590      5600      5610          5624
Homo sapiens chromosome 1 NC_000001.11: 11783698-... (5227) CTTGCTTTGATTTGTGGCCCCAGCTCAGGCCAGGCCCACCCGGTCCATCAGTTTGCGAAGAAGGTGTGGCATGA
SARS-CoV-2 Reference Genome NC_045512.2             (5384) GCTGCTAACTTTTGTGCACTTATCTTAGCCTACTGTAATAAGA--CAGTAGGTGAGTTAGGTGATGTTAGAGAA
```

Section 77

```
                                                  (5625) 5625 5630      5640      5650      5660      5670      5680              5698
Homo sapiens chromosome 1 NC_000001.11: 11783698-... (5301) GGCATG-GCTGCCACTCACA-CTAGCCCATGGAGGGCCTGG-------GTCCTGAGCGCCCCCTCCCTCATTTG
SARS-CoV-2 Reference Genome NC_045512.2             (5456) ACAATGAGTTACTTGTTTCAACATGCCAATTTAGATTCTTGCAAAAGAGTCTTGAACGTGGTGTGTAAAACTTG
```

Section 78

```
                                                     (5699) 5699       5710      5720      5730      5740      5750      5760      5772
Homo sapiens chromosome 1 NC_000001.11: 11783698-... (5366) CTCTGCTCTGAGTACAGC--TTCCCAGCTCACCATCAGATCTATTC-TGAGCTT--GTGCATTTGGCCTAGGCA
SARS-CoV-2 Reference Genome NC_045512.2              (5530) TGGACAACAGCAGACAACCCTTAAGGGTGTAGAAGCTGTTATGTACATGGGCACACTTTCTTATGAACAATTTA
```

Section 79

```
                                                     (5773) 5773  5780      5790      5800      5810      5820      5830          5846
Homo sapiens chromosome 1 NC_000001.11: 11783698-... (5435) AGACTGGCTGGCTGCTTGGGGCAGAGGGAGGGGCCCTGGGGACAAAAGGAGCTGGGT--ACAGGGGTTGC--TC
SARS-CoV-2 Reference Genome NC_045512.2              (5604) AGAAAGGTGTTCAGATACCTTGTACGTGTGGTAAACAAGCTACAAAAT-ATCTAGTACAACAGGAGTCACCTTT
```

Section 80

```
                                                     (5847) 5847         5860      5870      5880      5890      5900      5910 5920
Homo sapiens chromosome 1 NC_000001.11: 11783698-... (5505) TAGCAGGC--ACAGCACAGACCCCGGAGAAAGCCCACAACAATAG--CATTTCCTC-TCCTCCCTGGTTC-CAG
SARS-CoV-2 Reference Genome NC_045512.2              (5677) TGTTATGATGTCAGCACCACCTGCTCAGTATGAACTTAAGCATGGTACATTTACTTGTGCTAGTGAGTACACTG
```

Section 81

```
                                                     (5921) 5921     5930      5940      5950      5960      5970      5980        5994
Homo sapiens chromosome 1 NC_000001.11: 11783698-... (5573) GCCTTTAAGAA-GTGG-CACCAACGCCTGGCAGCCAGGAGCCCAA---------GGAGAGGAGCTGCCAGTAGC
SARS-CoV-2 Reference Genome NC_045512.2              (5751) GTAATTACCAGTGTGGTCACTATAAACATATAACTTCTAAAGAAACTTTGTATTGCATAGACGGTGCTTTACTT
```

Section 82

```
                                                     (5995) 5995 6000      6010      6020      6030      6040      6050            6068
Homo sapiens chromosome 1 NC_000001.11: 11783698-... (5636) CCAAGACCCTGGAGCAAGCCAGGCCCCA--AGGGC------CCCGAGAGTGGACAGGAAG----CCGCCAGAGC
SARS-CoV-2 Reference Genome NC_045512.2              (5825) ACAAAGTCCTCAGAATACAAAGGTCCTATTACGGATGTTTTCTACAAAGAAAACAGTTACACAACAACCATAAA
```

Section 83

```
                                                     (6069) 6069       6080      6090      6100      6110      6120      6130      6142
Homo sapiens chromosome 1 NC_000001.11: 11783698-... (5698) ACCGCGGGGTTGGGGGCTTGGGGCAGAGCATGGGGCCCAGCTGCAGCTGTGACTTGTTCTCATAGAATAAAAAA
SARS-CoV-2 Reference Genome NC_045512.2              (5899) ACCAGTTACTTATAAATTGGATGGTGTTGTTTGTACAGAAATTGACCC-TAAGTTGGACAATTATTATAAGAAA
```

Section 84

```
                                                     (6143) 6143  6150      6160      6170      6180      6190      6200          6216
Homo sapiens chromosome 1 NC_000001.11: 11783698-... (5772) CAGAACTGAACTTAGCCTTCCCAGGGAAGGAACCATGCCCCACAC-ATCCAGGGAGTGATGACAGAGGGGACAG
SARS-CoV-2 Reference Genome NC_045512.2              (5972) GACAATTCTTATTTCACAGAGCAACCAATTGATCTTGTACCAAACCAACCATATCCAAACGCAAGCTTCGATAA
```

Section 85

```
                                                     (6217) 6217         6230      6240      6250      6260      6270      6280     6290
Homo sapiens chromosome 1 NC_000001.11: 11783698-... (5845) CTTGAAGAGCTCTGAAGGACAATAAAACCCACTCCTCAGTCCTAGCTGCTGCTTTCAGTCTCTCCTAGCGGCTG
       SARS-CoV-2 Reference Genome NC_045512.2       (6046) TTTTAAG---TTTGTATGTGA-TAATATCAAAT---------TTGCTGATGATTTAAACCAGT--TAACTGGT-
```

Section 86

```
                                                     (6291) 6291     6300      6310      6320      6330      6340      6350         6364
Homo sapiens chromosome 1 NC_000001.11: 11783698-... (5919) CCCCTCTGCCCCAGCCAGTGAGGCCAGGAAGGGAGTAAAGAGGACATCCCACAATTGCGTCCACCCCCTTGGCC
       SARS-CoV-2 Reference Genome NC_045512.2       (6104) ----TATAAGAAACC---TGCTTCAAG--AGAGCTTAAAGTT-ACATTTTTCCCTGACTTAAA------TGGTG
```

Section 87

```
                                                     (6365) 6365 6370      6380      6390      6400      6410      6420             6438
Homo sapiens chromosome 1 NC_000001.11: 11783698-... (5993) ATCAGGCTGCTCTTCCA---CTACACGAAACAGCCCATCTGCACCTGCCAGTCAC--TGCCCCCAGGCCCTGGG
       SARS-CoV-2 Reference Genome NC_045512.2       (6162) ATGTGGTGGCTATTGATTATAAACACTACACACCCTCTTTTAAGAAAGGAGCTAAATTGTTACATAAACCTATT
```

Section 88

```
                                                     (6439) 6439       6450      6460      6470      6480      6490      6500        6512
Homo sapiens chromosome 1 NC_000001.11: 11783698-... (6062) GCTTG-CAGGTGACTCGTGGGG--AGCCAGGCCAGGCAGATAACCTCTGGGACAGGACGAGGGGATGAGGAAGG
       SARS-CoV-2 Reference Genome NC_045512.2       (6236) GTTTGGCATGTTAACAATGCAACTAATAAAGCCACGTATA-AACCA-------AATACCTGGTGTATACGTTGT
```

Section 89

```
                                                     (6513) 6513   6520      6530      6540      6550      6560      6570             6586
Homo sapiens chromosome 1 NC_000001.11: 11783698-... (6133) CAGCTGGCAGGACAAGGGCCTACCTGAAAAAT-ACACCAGGAAGCTGGGCTGCTG-CACAGGCCCAGGCAGATC
       SARS-CoV-2 Reference Genome NC_045512.2       (6302) CT--TTGGAGCACAAAACC--AGTTGAAACATCAAATTCGTTTGATGTACTGAAGTCAGAGGACGCGCAGGGAA
```

Section 90

```
                                                     (6587) 6587         6600      6610      6620      6630      6640      6650     6660
Homo sapiens chromosome 1 NC_000001.11: 11783698-... (6205) TGGGGCCCCTGTGCTG-GCAAATCTCATCTTAGCCTCTGAC-AGGGAACGGGGCAGCATCAGGCCTCCACAGCC
       SARS-CoV-2 Reference Genome NC_045512.2       (6372) TGGATAATCTTGCCTGCGAAGATCTAAAACCAGTCTCTGAAGAAGTAGTGGAAAATCCTACCATACAGAAAGAC
```

Section 91

```
                                                     (6661) 6661     6670      6680      6690      6700      6710      6720         6734
Homo sapiens chromosome 1 NC_000001.11: 11783698-... (6277) AGGCAAAAGGGGAAGGCCACGAGCCCCACCAAGCACAAGGGTTCAGACTGCCTTCAACCCTTAAGGGGCACTTT
       SARS-CoV-2 Reference Genome NC_045512.2       (6446) GTTCTTGAGTGTAATGTGAAAACTACCG--AAGTTGTAGGAGACATTATAC-TTAAACCAGCAAATAATAGTTT
```

```
                                                                                 Section 92
                                                   (6735) 6735 6740      6750      6760      6770      6780      6790         6808
Homo sapiens chromosome 1 NC_000001.11: 11783698-... (6351) TTCTCTCAAGGCTCAGAATAGACAACTGTGCCAAAAAGCAGCATG-GTAGGTGTCAGCCCAATGCCCATCCAGG
     SARS-CoV-2 Reference Genome NC_045512.2         (6517) AAAAATTACAGAAGAGGTTGGCCACACAGATCTAATGGCTGCTTATGTAGACAATTCTAGTCTTACTATTAAGA
                                                                                 Section 93
                                                   (6809) 6809       6820      6830      6840      6850      6860      6870    6882
Homo sapiens chromosome 1 NC_000001.11: 11783698-... (6424) GGGCTGGTGCCATGGACGGCTAAGTGGGCAGGCAGGGCAGTGGT-------GTGCCCCTTCAGTCACAAGTAG-
     SARS-CoV-2 Reference Genome NC_045512.2         (6591) AACCTAATGAATTATCTAGAGTATTAGGTTTGAAAACCCTTGCTACTCATGGTTTAGCTGCTGTTAATAGTGTC
                                                                                 Section 94
                                                   (6883) 6883   6890      6900      6910      6920      6930      6940      6956
Homo sapiens chromosome 1 NC_000001.11: 11783698-... (6490) --GTGGGGC-CC--AGCT------GCCCACCTCTAGGAGGTGGAAG--GGTACAGCACTTTGCCACATCTCTTC
     SARS-CoV-2 Reference Genome NC_045512.2         (6665) CCTTGGGATACTATAGCTAATTATGCTAAGCCTTTTCTTAACAAAGTTGTTAGTACAACTACTAACATAGTTAC
                                                                                 Section 95
                                                   (6957) 6957         6970      6980      6990      7000      7010      7020 7030
Homo sapiens chromosome 1 NC_000001.11: 11783698-... (6551) TACGATGCC--ACCAGTGCCTGATGCCCTTGCGTG--TTTTGCCTGTACTGCACGGGCTCCAA--GTGTAAGTT
     SARS-CoV-2 Reference Genome NC_045512.2         (6739) -ACGGTGTTTAAACCGTGTTTGTACTAATTATATGCCTTATTTCTTTACTTTATTG-CTACAATTGTGTACTTT
                                                                                 Section 96
                                                   (7031) 7031     7040      7050      7060      7070      7080      7090     7104
Homo sapiens chromosome 1 NC_000001.11: 11783698-... (6619) CACTGGAGTCCCAGGCTGGCTCTGGAGGCTGCCTTGG----TTCGAGGGCTTAGTTGGCAACAACCACTCTCC
     SARS-CoV-2 Reference Genome NC_045512.2         (6811) TACTAGAAGTACAAATTCTAGAATTAAAGCATCTATGCCGACTACTATAGCAAAGAATACTGTTAAGAGTGTCG
                                                                                 Section 97
                                                   (7105) 7105 7110      7120      7130      7140      7150      7160         7178
Homo sapiens chromosome 1 NC_000001.11: 11783698-... (6689) TCGCCTTCTCAGGAGGTGCCACCCACTGTGGTCCTCTGCAGG-ACGCAAGTGCTCCCCACCAAAAGAGCACAGA
     SARS-CoV-2 Reference Genome NC_045512.2         (6885) GTAAATTTTGTCTAGAGGCTTCA---TTTAATTATTTGAAGTCACCTAATTTTTCTAAACTGATAAATATTATA
                                                                                 Section 98
                                                   (7179) 7179       7190      7200      7210      7220      7230      7240    7252
Homo sapiens chromosome 1 NC_000001.11: 11783698-... (6762) CTCCTGGCTAGAGCAGAGAGTACTAGGTTCCCATGTGGGGCCGACTCA---GGTCCAGGAAGAGCCTGGAAGCC
     SARS-CoV-2 Reference Genome NC_045512.2         (6956) ATTTGGTTTTTACTATTAAGTGTTTGCCTAGGTTCTTTAATCTACTCAACCGCTGCTTTAGGTGTTTTAATGTC
```

```
                                                                Section 99
                                                (7253) 7253   7260      7270      7280      7290      7300      7310        7326
Homo sapiens chromosome 1 NC_000001.11: 11783698-... (6833) TCACTCCAGTCTAGCTGCC-ATTGTCAGGTGGGGGAGTGGAGGCCGGGGTGGGAGAGACACGAAGGAGAGTGGA
SARS-CoV-2 Reference Genome NC_045512.2              (7030) TAATTTAGGCATGCCTTCTTACTGTACTG-GTTACAGAGAAGGCTA---TTTGAACTCTACTAATGTCACTATT

                                                                Section 100
                                                (7327) 7327         7340      7350      7360      7370      7380      7390   7400
Homo sapiens chromosome 1 NC_000001.11: 11783698-... (6906) GTTCCAAGAGAAGCAGCACTGTGGAGGAGGAAGGCGGGACAGGAGTGGCTCCAACGCAGGGCGTCAGGACGCA
SARS-CoV-2 Reference Genome NC_045512.2              (7100) GCAACCTACTGTACTGGTTCTAT--ACCTTGTAGTGTTTGTCTTAGTGGTTTAGATTC------TTTAGACACC

                                                                Section 101
                                                (7401) 7401     7410      7420      7430      7440      7450      7460        7474
Homo sapiens chromosome 1 NC_000001.11: 11783698-... (6980) GGGTCATGGAGCCTCCGTTTCTCT-CGCATTCTGGGTGGGCCTGTTGAGAAGCTCCAATGTGTCTTCCACCACC
SARS-CoV-2 Reference Genome NC_045512.2              (7166) TATCCTT----CTTTAGAAACTATACAAATTACCATTTCATCTTTTAAATGGGATTTAACTGCTTTTGGCTTAG

                                                                Section 102
                                                (7475) 7475 7480      7490      7500      7510      7520      7530          7548
Homo sapiens chromosome 1 NC_000001.11: 11783698-... (7053) TGCCAGAGGCAGTTGTCCAGTGGGAAGTCAT-TGTCCACCAGGTTGACCAGGAAGTAGTTGTCGTGGATGTACT
SARS-CoV-2 Reference Genome NC_045512.2              (7236) TTGCAGAGTG-GTTTT----TGGCATATATTCTTTTCACTAGGTTTTTCT--ATGTACTTGGATTGGCTGCAAT

                                                                Section 103
                                                (7549) 7549         7560      7570      7580      7590      7600      7610   7622
Homo sapiens chromosome 1 NC_000001.11: 11783698-... (7126) GGATGATGGTGCGGGACGGGGACTCCTCCTCATACAGCTT-TCCCCACCGCTCAATCCACAGGGCAAAGGCCTC
SARS-CoV-2 Reference Genome NC_045512.2              (7303) C-ATGCAATTGTTTTTCAGCTATTTTGCAG--TACATTTTATTAGTAATTCTTGGCTTATGTGGTTAATAATTA

                                                                Section 104
                                                (7623) 7623   7630      7640      7650      7660      7670      7680        7696
Homo sapiens chromosome 1 NC_000001.11: 11783698-... (7199) GTCCTACACACACATACCCCCGCACACACGCACACAT-GCACACACA-CACAGAGGCAAGATTAACAGGGCTCT
SARS-CoV-2 Reference Genome NC_045512.2              (7374) ATCTTGTACAAA--TGGCCCCGATTTCAGCTATGGTTAGAATGTACATCTTCTTTGCATCATTTTATTATGTAT

                                                                Section 105
                                                (7697) 7697         7710      7720      7730      7740      7750      7760   7770
Homo sapiens chromosome 1 NC_000001.11: 11783698-... (7271) CCAAGGGCTCTGCTCAACAGGACATTTCCCGGAATAGAACAAGGCCCACTGCCAGTATCACTTCCCACCCCTGC
SARS-CoV-2 Reference Genome NC_045512.2              (7446) GGAAAAGTTATGTGCATGTTGTAGACGGTTGTAATTCATCAA------CTTGTATGATGTGTTACAAACGTAAT
```

Section 106

```
                                               (7771) 7771     7780          7790          7800          7810          7820          7830            7844
Homo sapiens chromosome 1 NC_000001.11: 11783698-... (7345) CCAGAAATAAACACCC--TGCATCAGCAGTGACATGGGTGAGTCAGAGCTGGGTGTGCCCTGAAGGCTGGGTCC
   SARS-CoV-2 Reference Genome NC_045512.2 (7514) AGAGCAACAAGAGTCGAATGTACAACTATTGTTAATGGTG--TTAGAAGGTCCTTTTATGTCTATGCTAATGGA
```

Section 107

```
                                               (7845) 7845 7850          7860          7870          7880          7890          7900            7918
Homo sapiens chromosome 1 NC_000001.11: 11783698-... (7417) AGTGTGGCCTTCAGC-----ACACGCTTGG----GACTTGGTCCCAATCCCTCCCCAC--GGTTTTCCAGGTGG
   SARS-CoV-2 Reference Genome NC_045512.2 (7586) GGTAAAGGCTTTTGCAAACTACACAATTGGAATTGTGTTAATTGTGATACATTCTGTGCTGGTAGTACATTTAT
```

Section 108

```
                                               (7919) 7919       7930          7940          7950          7960          7970          7980      7992
Homo sapiens chromosome 1 NC_000001.11: 11783698-... (7480) GCGGGGCAAGCTTGCCCCCGGCTC----CTTTACCTTCCAGAAC--ATGAAGCTGACGGGATCCACTACGGTGG
   SARS-CoV-2 Reference Genome NC_045512.2 (7660) TAGTGATGAAGTTGCGAGAGACTTGTCACTACAGTTTAAAAGACCAATAAATCCTACTGA--CCAGTCTTCTTA
```

Section 109

```
                                               (7993) 7993   8000          8010          8020          8030          8040          8050            8066
Homo sapiens chromosome 1 NC_000001.11: 11783698-... (7548) GCTGGATGATCTCTCGCCCAGGGAAGAT--GCCCCAAGTGACAGCATTCGGCT---GCAGTTCAGGGGCATTGG
   SARS-CoV-2 Reference Genome NC_045512.2 (7732) CATCGTTGATAG-TGTTACAGTGAAGAATGGTTCCATCCATCTTTACTTTGATAAAGCTGGTCAAAAGACTTAT
```

Section 110

```
                                               (8067) 8067          8080          8090          8100          8110          8120          8130      8140
Homo sapiens chromosome 1 NC_000001.11: 11783698-... (7617) TGATGTT-TTCACCCTGAGGGGATGTGGGGTGGGGTAAGACCTGGG--CCAAGAACAACTGGAGTCCCACTGC
   SARS-CoV-2 Reference Genome NC_045512.2 (7805) GAAAGACATTCTCTCTCTCATTTTGTTAACTTAG---ACAACCTGAGAGCTAATAACA-CTAAAGGTTCATTGC
```

Section 111

```
                                               (8141) 8141     8150          8160          8170          8180          8190          8200            8214
Homo sapiens chromosome 1 NC_000001.11: 11783698-... (7688) CTGGACAGCCTTGACTCAATGAGGCTGTCT----GGGCTCAATACCCTCTGCCTGCGCCTCCACTCCCGGCCTC
   SARS-CoV-2 Reference Genome NC_045512.2 (7875) CTATTAATGTTATAGTTTTTGATGGTAAATCAAAATGTGAAGAATCATCTGCAAAATCAGCGTCTGTTTAC-TA
```

Section 112

```
                                               (8215) 8215 8220          8230          8240          8250          8260          8270            8288
Homo sapiens chromosome 1 NC_000001.11: 11783698-... (7758) CACTCTCCCTGCGCCTCCACTCCCCTCTCCCTGTGCCTCCGCTCCTGGCCTCCTCTGCCTGCATCTGTGAATGA
   SARS-CoV-2 Reference Genome NC_045512.2 (7948) CAGTCAGCTTATGTGTCAAC----CTATAC-TGTTACTA-GATCA-GGCATTAG-TGTCTGA---TGTTGGTGA
```

Section 113

```
                                                         8289       8300      8310      8320      8330      8340      8350        8362
                                                 (8289)
Homo sapiens chromosome 1 NC_000001.11: 11783698-... (7832) TATCTCTCCCCACACAAGCCTGAACCTAGAGGCGT-CCTGGCCAC-TTCCCCTCTCCTCCTTGAGCACCCCCTG
       SARS-CoV-2 Reference Genome NC_045512.2 (8011) TAGTGCGGAAGTTGCAGTTAAAATGTTTGATGCTTACGTTAATACGTTTTCATCAACTTTTAACGTACCAA-TG
```

Section 114

```
                                                         8363   8370      8380      8390      8400      8410      8420          8436
                                                 (8363)
Homo sapiens chromosome 1 NC_000001.11: 11783698-... (7904) CTCACTCTCTGATCC---ATTCCCTCTTGTCTAGCCCACCT--CCAGGAGTGTGTTTGGCTTGGGTCACTCCAG
       SARS-CoV-2 Reference Genome NC_045512.2 (8084) GAAAAACTCAAAACACTAGTTGCAACTGCAGAAGCTGAACTTGCAAAGAATGTGT----CCTTAGACAAT---G
```

Section 115

```
                                                         8437         8450      8460      8470      8480      8490      8500  8510
                                                 (8437)
Homo sapiens chromosome 1 NC_000001.11: 11783698-... (7973) GGGTCTCTTCACTGGCCTCCCCGGCTCTAGCCGTCCCCAATCTCTCTCATCCGTATGACTATCCA--CTGT-GA
       SARS-CoV-2 Reference Genome NC_045512.2 (8151) TCTTATCTACTTTTATTTCAGCAGCTCGGCAAGGGTTTGTTGATTCAGATGTAGAA-ACTAAAGATGTTGTTGA
```

Section 116

```
                                                         8511     8520      8530      8540      8550      8560      8570        8584
                                                 (8511)
Homo sapiens chromosome 1 NC_000001.11: 11783698-... (8044) TTTGCCTAAAATAAAGCAGGAACCAAGCCACCCCACTGCTGGAGAAAGGCTTCCCTTGCATTGAGGGAAAACTC
       SARS-CoV-2 Reference Genome NC_045512.2 (8224) ATGTCTTAAATTGTCACATCAATCTGACATAGAAGTTACTGGCGATAG-------TTGTAATAACTATATGCTC
```

Section 117

```
                                                         8585 8590      8600      8610      8620      8630      8640          8658
                                                 (8585)
Homo sapiens chromosome 1 NC_000001.11: 11783698-... (8118) AA--ATTCCAGCAGGACAACCATGATCTCTGCGGGCCTGGCCCCTGCTTAACACTCCCGCCTCCTCCTGCTCAT
       SARS-CoV-2 Reference Genome NC_045512.2 (8291) ACCTATAACAAAGTTGAAAACATGACACCCCGTGACCTTGGTGCTTGTATTGACTGTAG--TGCGCGTCATATT
```

Section 118

```
                                                         8659       8670      8680      8690      8700      8710      8720        8732
                                                 (8659)
Homo sapiens chromosome 1 NC_000001.11: 11783698-... (8190) GTTCCCCGTTTCCCCAGATGGTGCAGCTCACTGCCTCTGCCTGTCCTCTGCCCTGTCCCAGGAATACCCTTCTC
       SARS-CoV-2 Reference Genome NC_045512.2 (8363) AATGCGCAGGTAGCAAAAAG-------TCACAACAT-TGCTT-TGATATGGAACGTTAAAGATTTCATGTCATT
```

Section 119

```
                                                         8733   8740      8750      8760      8770      8780      8790          8806
                                                 (8733)
Homo sapiens chromosome 1 NC_000001.11: 11783698-... (8264) TCCTGTTGGCCAACTCCTCTTTGTCCTCAGTGATTCCCACA-GATGACCCT--CTGAGAAGCCTTCCCTGGCCT
       SARS-CoV-2 Reference Genome NC_045512.2 (8428) GTCTGAACAACTACGAAAACAAATACGTAGTGCTGCTAAAAGAATAACTTACCTTTTAAGT-TGACATGTGCA
```

Section 120

	(8807)	8807 … 8880
Homo sapiens chromosome 1 NC_000001.11: 11783698-...	(8335)	CCCAGGTGAGATATCCCTCCTCTGGACCCGACAGCATCCTCTCCTGAACTTG---ATTTTCCCACT--TACACT
SARS-CoV-2 Reference Genome NC_045512.2	(8501)	ACTACTAGACAAGTTGTTAATGTTGTAACAACAAAGATAGCACTTAAGGGTGGTAAAATTGTTAATAATTGGTT

Section 121

	(8881)	8881 … 8954
Homo sapiens chromosome 1 NC_000001.11: 11783698-...	(8404)	G--GCTGGTGGTCACACATTGATCTGCCTCCCTCACTCTAG--GCTGGGAGCTCCTTAAGAGCAGG-GCCCCAG
SARS-CoV-2 Reference Genome NC_045512.2	(8575)	GAAGCAGTTAATTAAAGTTACACTTGTGTTCCTTTTTGTTGCTGCTATTTTCTATTTAATAACACCTGTTCATG

Section 122

	(8955)	8955 … 9028
Homo sapiens chromosome 1 NC_000001.11: 11783698-...	(8473)	TTCTGCCTGTATCTCTCTGTCTTGCACAATGCCTAGCCCAGG---CTAGGTGCTGGGTGTTTGCTCAACGAAGG
SARS-CoV-2 Reference Genome NC_045512.2	(8649)	TCATGTCTAAACATA-CTGACTTTTCAAGTGAA-ATCATAGGATACAAGGCTATTGATGGTGGTGTCACTCGTG

Section 123

	(9029)	9029 … 9102
Homo sapiens chromosome 1 NC_000001.11: 11783698-...	(8544)	GCCTGGTACTCTGTGGGAACCCTGGGGCCTGGCCTACCTTCACATTGACA-AGGTGGTAATTAACCCG-GAGCT
SARS-CoV-2 Reference Genome NC_045512.2	(8721)	ACATAGCA-TCTACAGATACTTGTTTTGCTAACAAACATGCTGATTTTGACACATGGT--TTAGCCAGCGTGGT

Section 124

	(9103)	9103 … 9176
Homo sapiens chromosome 1 NC_000001.11: 11783698-...	(8616)	CGTACTTCTTCAGCACTTGCAGAAGTGCTTCCGCTGTCTCGCGGGAAGTGAAAAACTCTAAGTAGGCCTGTGGG
SARS-CoV-2 Reference Genome NC_045512.2	(8792)	GGTAGTTATACT--AATGACAAAGCT--TGCCCATTGATTGCTGCAGTCATAACAAGAGAAGTGGG--TTTTGT

Section 125

	(9177)	9177 … 9250
Homo sapiens chromosome 1 NC_000001.11: 11783698-...	(8690)	AGAGACAGG--TGCCTGTCACCAACCCCAGCCCTGCCA--GACGCCCTTTCGCAGACACCTAAGGTATCAGGAC
SARS-CoV-2 Reference Genome NC_045512.2	(8860)	CGTGCCTGGTTTGCCTGGCACGATATTACGCACAACTAATGGTGACTTTTGCA------------TTTCTTACC

Section 126

	(9251)	9251 … 9324
Homo sapiens chromosome 1 NC_000001.11: 11783698-...	(8760)	TACTGGCAGCCATGTGGGCGGAATAATGAACTGTGGCGTTGGCACGTCCATAAGCAAAGGGTGAGGGGAGTG-G
SARS-CoV-2 Reference Genome NC_045512.2	(8923)	TAGAGTTTTTAGTGCAGTTGGTAACAT---CTGTTACACAC---CATCAAAACTTATAGAGTACACTGACTTTG

Section 127

```
                                                            9325 9330      9340      9350      9360      9370      9380              9398
                                                   (9325)
Homo sapiens chromosome 1 NC_000001.11: 11783698-... (8833) GGACGTGAGTTTCTCAGTCTGGACAGGTCTGAGGGCTATCAGCCACCCCTCTCCCAATCAGTCAGGGAAGACTC
      SARS-CoV-2 Reference Genome NC_045512.2        (8991) CAACATCAGCTTGT--GTTTTGGCTGCTGAATGTACAATT----------TTTAAAGATGCTTCTGGTAAGCCAG
```

Section 128

```
                                                            9399       9410      9420      9430      9440      9450      9460          9472
                                                   (9399)
Homo sapiens chromosome 1 NC_000001.11: 11783698-... (8907) CACGAAGAAGCCATTTCTGAACAGCAGCAACTGGTTTGACAGGCCGAGTCATGGAGAGAGGCCCCACACATTC
      SARS-CoV-2 Reference Genome NC_045512.2        (9054) TACCATATTG----TTATGATACCAATGTACTAGAAGGTTCTGTTGCTT-ATGAAAGTTTACGCCCTGACACAC
```

Section 129

```
                                                            9473   9480      9490      9500      9510      9520      9530              9546
                                                   (9473)
Homo sapiens chromosome 1 NC_000001.11: 11783698-... (8981) CATCCTCCCCAGTGCAAGCTGACAATGACATTGACATCTCCTCTAGAACCAGTGCCTATCAGGGATTAAGCTGG
      SARS-CoV-2 Reference Genome NC_045512.2        (9123) GTTATGTGCTCATGGA---TGGCTCTATTATTCA-ATTTCCT----AACACCTACCTTGAAGG--TTCTGTTAG
```

Section 130

```
                                                            9547         9560      9570      9580      9590      9600      9610      9620
                                                   (9547)
Homo sapiens chromosome 1 NC_000001.11: 11783698-... (9055) GTTTACTCAAACTCATTTGCTAGAATGCTGATAATACGGGAGGCTGGGCACAGTGAGCCCCACGTGAGGAAAGT
      SARS-CoV-2 Reference Genome NC_045512.2        (9187) AGTGGTAACAACT--TTTG-----ATTCTGAG--TACTGTAGGCACGGCACT-TGTGAAAGATCAGAAGCTGGT
```

Section 131

```
                                                            9621     9630      9640      9650      9660      9670      9680            9694
                                                   (9621)
Homo sapiens chromosome 1 NC_000001.11: 11783698-... (9129) GGGT-TCAGGGAAGTTGAGCACGTTGGCTACTCTGACAGTAAACAGTAGAGCTGGTCCTTAAACTCAGGTCTTT
      SARS-CoV-2 Reference Genome NC_045512.2        (9251) GTTTGTGTATCTACTAGTGGTAGATGGGTACT-TAACAATGATTATTACAGAT---CTTTACCAGGAGTTTTCT
```

Section 132

```
                                                            9695 9700      9710      9720      9730      9740      9750              9768
                                                   (9695)
Homo sapiens chromosome 1 NC_000001.11: 11783698-... (9202) CTGCTCCAGGGCCTGTGGGCCTTAGATCAGAACATAATGTTAACTCCAGCAGCCCTGGGAGAAAGGTGTCAACT
      SARS-CoV-2 Reference Genome NC_045512.2        (9321) GTGGTGTAGATGCTGTAAAT-TTACTTACTAATAT---GTTTACACCACTAATTCAACCT-ATTGGTG----CT
```

Section 133

```
                                                            9769       9780      9790      9800      9810      9820      9830          9842
                                                   (9769)
Homo sapiens chromosome 1 NC_000001.11: 11783698-... (9276) CAGGATAGTAACAGCAGCTGCCTTTCAGGGAGTGCTTCCTGCTCT-----TACCATG---TGCCAAGCACGTTA
      SARS-CoV-2 Reference Genome NC_045512.2        (9386) TTGGACA-TATCAGCATCTATAGTAGCTGGTGGTATTGTAGCTATCGTAGTAACATGCCTTGCCTACTATTTTA
```

```
                                                                   Section 134
                                                   (9843)  9843    9850     9860     9870     9880     9890     9900      9916
Homo sapiens chromosome 1 NC_000001.11: 11783698-... (9342)  CATGCTTTGTTAGGTTTCATGCCCAGAACACA-AGCTACATCCATTTTACAGACGAGAAACCAGGC-TCAGA
        SARS-CoV-2 Reference Genome NC_045512.2 (9459)  TGAGGTTTAGAAGAGCTTTTGGTGAATACAGTCATGTAGTTGCC-TTTAATACTTTACTATTCCTTATGTCATT

                                                                   Section 135
                                                   (9917)  9917        9930     9940     9950     9960     9970     9980   9990
Homo sapiens chromosome 1 NC_000001.11: 11783698-... (9414)  GAG-GTCGAGTGACCTGCCTCCATAGGTCTGATTTCTATCT--TTTTTTTTTTTTTTTGAGATGGAGTCTTGC
        SARS-CoV-2 Reference Genome NC_045512.2 (9532)  CACTGTACTCTGTT-TAACACCAGTTTACTCATTCTTACCTGGTGTTTATTCTGTTATTTACTTGTA--CTTGA

                                                                   Section 136
                                                   (9991)  9991  10000    10010    10020    10030    10040    10050   10064
Homo sapiens chromosome 1 NC_000001.11: 11783698-... (9485)  TCTGTTGCCCAGGCTGGAGTGCAGTGGCATGATCTCAGCTCAC-TGCAACCTCCGTCTCCCGGGTTCAAGCGAT
        SARS-CoV-2 Reference Genome NC_045512.2 (9603)  CATTTTATCTTA-CT--AATGATGTTTCTT--TTTTAGCACATATTCAGTGGATGGTTAT---GTTCACACCTT

                                                                   Section 137
                                                  (10065)  10065 10070   10080    10090    10100    10110    10120    10138
Homo sapiens chromosome 1 NC_000001.11: 11783698-... (9558)  TCTCCTGCCTCAGCCTCCCAAGTAGCTGCAACTACAGGTGCCCGCCACCACACCCGGCTAATTGTTGTATTTTT
        SARS-CoV-2 Reference Genome NC_045512.2 (9669)  TAGTACCTTTCTGGATAACAATT-GCTTATATCATTTGTATTT-CCACAAAGCATTTCTATTGGTTCTTTAGTA

                                                                   Section 138
                                                  (10139)  10139   10150    10160    10170    10180    10190    10200   10212
Homo sapiens chromosome 1 NC_000001.11: 11783698-... (9632)  AGTAGAGATGGGGTTTCATCATGTTTGTCAGGCTG----GTCTCGAACTCCTGACCTTG-TGATCTGCCCACCT
        SARS-CoV-2 Reference Genome NC_045512.2 (9741)  ATTACCTAAAGAGA--CGTGTAGTCTTTAATGGTGTTTCCTTTAGTACTTTTGAAGAAGCTGCGCTGTGCACCT

                                                                   Section 139
                                                  (10213)  10213 10220    10230    10240    10250    10260    10270    10286
Homo sapiens chromosome 1 NC_000001.11: 11783698-... (9701)  TGGCCTC--CTAAAGTGCTGGGATTACAG--GCAT---GAGCCACCAAGCCCAGCCAATCTTTTTTTTTTTTT
        SARS-CoV-2 Reference Genome NC_045512.2 (9813)  TTTTGTTAAATAAAGAAATGTATCTAAAGTTGCGTAGTGATGTGCTATTACCTCTTACGCAATATAATAGATAC

                                                                   Section 140
                                                  (10287)  10287      10300    10310    10320    10330    10340    10350 10360
Homo sapiens chromosome 1 NC_000001.11: 11783698-... (9768)  TTTTTTTTTTTTT-TGAGACAGGGTCTCACTTTGTGGGCCAGGCTGGAGTGCAG-AGGCACAAATATG--GCTT
        SARS-CoV-2 Reference Genome NC_045512.2 (9887)  TTAGCTCTTTATAATAAGTACAAGTATT--TTAGTGGAGCAA--TGGATACAACTAGCTACAGAGAAGCTGCTT
```

Section 141

	(10361)	10361 … 10434
Homo sapiens chromosome 1 NC_000001.11: 11783698-...	(9838)	ACTGGAGGCTCAACCTCTCGGACTCAAGCGATCCTCTCACCCCAGCCCCCTAAGTACCTGGGACTACAGGTGTA
SARS-CoV-2 Reference Genome NC_045512.2	(9957)	GTTGTCATCTCG--CAAAGGCTCTCAATGACTTCAGTAACTC-AGGTTCTGATGTTCTTTAC-CAAC-------

Section 142

	(10435)	10435 … 10508
Homo sapiens chromosome 1 NC_000001.11: 11783698-...	(9912)	CACCACCATGCCTCGGTCATTTTTGTATTTTTTGTAGAGAATGGGTTTCACCATGTTGCCCAGGCTGGTCTCGA
SARS-CoV-2 Reference Genome NC_045512.2	(10020)	CACCACAAA-CCTCTATCACCTCAGC-TGTTTTGCAGAG--TGGTTTTAGAAAAAATGGCATTCCCATCTGGTAA

Section 143

	(10509)	10509 … 10582
Homo sapiens chromosome 1 NC_000001.11: 11783698-...	(9986)	ACTCCTGGGCTCAAGTGATCCACCCGCCTTGGCCTCCCAAAGTGCTGGCCGGGTTCCGTGCCCAGCCAGGTCTA
SARS-CoV-2 Reference Genome NC_045512.2	(10090)	AGTTGAGGGTTGTA-TGGTACAAGTAACTTGTGGTACAACTACACTTAACGG--TCTTTGGCTTGAT-GACGTA

Section 144

	(10583)	10583 … 10656
Homo sapiens chromosome 1 NC_000001.11: 11783698-...	(10060)	TCTGACTT---AAAGCATGAGCTCCCATCCGCTCCAATCCATGCTCATTCAAAATTTAAGAGCCCAGGAGAGGC
SARS-CoV-2 Reference Genome NC_045512.2	(10160)	GTTTACTGTCCAAGACATGTGATCTGCACCTCTG-AAGACATGCTTAACCCTAATTATGAAGATTTACTCATTC

Section 145

	(10657)	10657 … 10730
Homo sapiens chromosome 1 NC_000001.11: 11783698-...	(10131)	AGGCTGCTCTGCCCTAAGCTCTGGGGAACCCACGGGTGCCGGTCAAGAGAGGG-----GGGCACCCCGTGCC--
SARS-CoV-2 Reference Genome NC_045512.2	(10233)	GTAAGTCTAATCA-TAATTTCTTGGTA-CAGGCTGGTAATGTTCAACTCAGGGTTATTGGACATTCTATGCAAA

Section 146

	(10731)	10731 … 10804
Homo sapiens chromosome 1 NC_000001.11: 11783698-...	(10198)	--TCCCTACCACACCTTCTGGAAGACATAGCCCCCGCTGGGGCCCCAGCCCACGA-TGGGGTCGGAGGACGGCT
SARS-CoV-2 Reference Genome NC_045512.2	(10305)	ATTGTGTACTTAAGCTTAAGGTTGATACAGCCAATCCTAAGACACCTAAGTATAAGTTTGTTCGCATTCAACCA

Section 147

	(10805)	10805 … 10878
Homo sapiens chromosome 1 NC_000001.11: 11783698-...	(10269)	TCCCGTTGATGTTGGGCTGTGAG-TTGATGGTGAGGATGCCC---TGGCGGTTCACCCGCAGC----AGCTCCT
SARS-CoV-2 Reference Genome NC_045512.2	(10379)	GGACAGACTTTTTCAG-TGTTAGCTTGTTACAATGGTTCACCATCTGGTGTTTACCAATGTGCTATGAGGCCCA

Section 148

	(10879)	10879 10890 10900 10910 10920 10930 10940 10952
Homo sapiens chromosome 1 NC_000001.11: 11783698-...	(10335)	CCTTCAGCAGGCTGGTCTCAGCCGCCAGGGGCTCATCGTTCCAGGGCAGGCAAGTCACCTGGGAGA-GACGGTG
SARS-CoV-2 Reference Genome NC_045512.2	(10452)	ATTTCACTATTAAGGGTTCATTCCTTAATGGTTCAT-GTGGTAGTGTTGGTTT-TAACATAGATTATGACTGTG

Section 149

	(10953)	10953 10960 10970 10980 10990 11000 11010 11026
Homo sapiens chromosome 1 NC_000001.11: 11783698-...	(10408)	AG-CTGGCTGGGGC---GACCATCAGGT-TTGGCACCCTGAGTCCCT-CTCACGGCCCCCAACAAAG-ACCCAG
SARS-CoV-2 Reference Genome NC_045512.2	(10524)	TCTCTTTTTGTTACATGCACCATATGGAATTACCAACTGGAGTTCATGCTGGCACAGACTTAGAAGGTAACTTT

Section 150

	(11027)	11027 11040 11050 11060 11070 11080 11090 11100
Homo sapiens chromosome 1 NC_000001.11: 11783698-...	(10475)	------CCTGTCTTTGCCTCCCTAA--GCCCTTCCAGGTGGAGGTCTCCCAACT--TACCCTTCTCCCTTTGCC
SARS-CoV-2 Reference Genome NC_045512.2	(10598)	TATGGACCTTTTGTTGACAGGCAAACAGCACAAGCAGCTGGTACGGACACAACTATTACAGTTAATGTTTTAGC

Section 151

	(11101)	11101 11110 11120 11130 11140 11150 11160 11174
Homo sapiens chromosome 1 NC_000001.11: 11783698-...	(10539)	ATG----TCCACAGCATGGAGGGGAGGGCACAGGATG-------GGGAAGTCACAGCCCCGCAGCCTGGCCTGC
SARS-CoV-2 Reference Genome NC_045512.2	(10672)	TTGGTTGTACGCTGCTGTTATAAATGGAGACAGGTGGTTTCTCAATCGATTTACCACAACTCTTAATGACTTTA

Section 152

	(11175)	11175 11180 11190 11200 11210 11220 11230 11248
Homo sapiens chromosome 1 NC_000001.11: 11783698-...	(10602)	AGCTGGGGTCAGGCCAGGGGCAGGGGATGAACCAGGGTCCCCACTCCAGCATCACTCACTTTGTGACCATTCCG
SARS-CoV-2 Reference Genome NC_045512.2	(10746)	ACCTTGTGGCTATGAAGTACAATT--ATGAACCTCTAACACAAGACCATGTTGACATACTAGGACCTCTTTCTG

Section 153

	(11249)	11249 11260 11270 11280 11290 11300 11310 11322
Homo sapiens chromosome 1 NC_000001.11: 11783698-...	(10676)	GTTTGGTTCTCCCGAGAGGTAAAGA-ACGAAGACTTCA--AAGACACTTTCTTCA--CTGGTCAGCTCCTCCCC
SARS-CoV-2 Reference Genome NC_045512.2	(10818)	CTCAAACTGGAATTGCCGTTTTAGATATGTGTGCTTCATTAAAAGAATTACTGCAAAATGGTATGAATGGACGT

Section 154

	(11323)	11323 11330 11340 11350 11360 11370 11380 11396
Homo sapiens chromosome 1 NC_000001.11: 11783698-...	(10745)	CCACATCTTCAGCAGCTCCTCCTTGGGGGACTTGCTCTTCAGGTAGAAGAGGTAGTAGTCCTT---CAG-----
SARS-CoV-2 Reference Genome NC_045512.2	(10892)	AC-CATATTGGGTAGTGCTTTATTAGAAGA-TGAATTTACACCTTTTGATGTTGTTAGACAATGCTCAGGTGTT

		Section 155
	(11397)	11397 11410 11420 11430 11440 11450 11460 11470
Homo sapiens chromosome 1 NC_000001.11: 11783698-...	(10811)	-CTCCCCAAAG-GCAGGGGAA-GAGGAAT--TG----CCCCTGGCAGAGGGGTGCCCAGAGGTCAGGGCACACT
SARS-CoV-2 Reference Genome NC_045512.2	(10964)	ACTTTCCAAAGTGCAGTGAAAAGAACAATCAAGGGTACACACCACTGGTTGTTACTCACAATTTTGACTTCACT

		Section 156
	(11471)	11471 11480 11490 11500 11510 11520 11530 11544
Homo sapiens chromosome 1 NC_000001.11: 11783698-...	(10876)	CCT-GACAGAGGGCAGTGCCACCACATGCCCAGGAGGCCATTCCTGTAA-ATTCTGCCCCTG--ACTCCTCCCA
SARS-CoV-2 Reference Genome NC_045512.2	(11038)	TTTAGTTTTAGTCCAGAGTACTCAATGGTCTTTGTTCTTTTTTTTGTATGAAAATGCCTTTTTACCTTTTGCTA

		Section 157
	(11545)	11545 11550 11560 11570 11580 11590 11600 11618
Homo sapiens chromosome 1 NC_000001.11: 11783698-...	(10946)	GGTCAACCACAAGCATGCAAACTTCTTCTGCCCTCCCGCTCC-CAAGAACAAAGATGTATTTGCAAGGAAGGTC
SARS-CoV-2 Reference Genome NC_045512.2	(11112)	TGGGTATTATTGCTATGT---CTGCTTTTGCAATGATGTTTGTCAAACATAAGCATGCATTTCTCTGTTTGTTT

		Section 158
	(11619)	11619 11630 11640 11650 11660 11670 11680 11692
Homo sapiens chromosome 1 NC_000001.11: 11783698-...	(11019)	TGCAGGCCCTCACCAGCGGCCGTTAGGGAACTCGTCCCACTCCTGGGTACGGTAGATGTAACTCTTTGGTCTGG
SARS-CoV-2 Reference Genome NC_045512.2	(11183)	TTGTTACCTTCTCTTGCCACTGT-----AGCTTATTTTAATATGGTCTATATGCCTGCTAGTTGGGTGATGCGT

		Section 159
	(11693)	11693 11700 11710 11720 11730 11740 11750 11766
Homo sapiens chromosome 1 NC_000001.11: 11783698-...	(11093)	AGGCCCAGAAGATGGGACGTACATCTTCCTCTCGGCGCTTGGGGTGGGCGCTGAGAGCCCAGGGTAGGGGACGC
SARS-CoV-2 Reference Genome NC_045512.2	(11252)	ATTATGACATGGTTGGATATGGTTGATACTAGTTTGTCT--GGTTTTAAGCTAAAAGACTGTGTTATGT-ATGC

		Section 160
	(11767)	11767 11780 11790 11800 11810 11820 11830 11840
Homo sapiens chromosome 1 NC_000001.11: 11783698-...	(11167)	CTGGGTGAGGATGGGGACAGAGAATTGAGACA--AGGGATTGGCTA----GAGGGAGCCGGAAGCAGGGGAGCA
SARS-CoV-2 Reference Genome NC_045512.2	(11323)	ATCAGCT-GTAGTGTTACTAATCCTTATGACAGCAAGAACTGTGTATGATGATGGTGCTAGGAGAGTGTGGACA

		Section 161
	(11841)	11841 11850 11860 11870 11880 11890 11900 11914
Homo sapiens chromosome 1 NC_000001.11: 11783698-...	(11235)	CCCCTGA--GGCTGAGTTCTTCCATCTGATGCCCTCTGAGACTGGAGCCTTCGATGTGCCCAGCTAGCCATG--
SARS-CoV-2 Reference Genome NC_045512.2	(11396)	CTTATGAATGTCTTGACACTCGTTTATAAAGTT-TATTATGGTAATGCTTTAGATCAAGCCATTT--CCATGTG

```
                                                                              Section 162
                                                    (11915) 11915 11920     11930     11940     11950     11960     11970            11988
Homo sapiens chromosome 1 NC_000001.11: 11783698-... (11305) -GCACAGACATCCATCTGTACCACCGTGGGGAGCCAG---CACTGCAGCCGGGGCTGCTCTTGGACCCTCCTCT
    SARS-CoV-2 Reference Genome NC_045512.2 (11467) GGCTCTTATAATC-TCTGTTACTTC-TAACTACTCAGGTGTAGTTACAACTGTCATGTTTTTGGCCAGAGGTAT
                                                                              Section 163
                                                    (11989) 11989      12000     12010     12020     12030     12040     12050       12062
Homo sapiens chromosome 1 NC_000001.11: 11783698-... (11375) GGGGATCTCTGGGCCACTGCCCTCACCTGGGGTCCTCAGTCCACATCCCCAGGCGCTTCAGCACCTCTGTGGTA
    SARS-CoV-2 Reference Genome NC_045512.2 (11539) TGTTTTTATGTGTGTTGAGTATTGCCCTATTTTCTTCATAACTGGTA---ATACACTTCAGTGTATAA-TGCTA
                                                                              Section 164
                                                    (12063) 12063   12070     12080     12090     12100     12110     12120           12136
Homo sapiens chromosome 1 NC_000001.11: 11783698-... (11449) GCCATCTCGCGGTTGAGGGTG---TAGAAGTGGAGGCCT-GGCACCAAGCCACTGGCCAGAAGCTCCTGGC-AC
    SARS-CoV-2 Reference Genome NC_045512.2 (11609) GTT-TATTGTTTCTTAGGCTATTTTTGTACTTGTTACTTTGGCCTCTTTTGTTTACTCAACCGCTACTTTAGAC
                                                                              Section 165
                                                    (12137) 12137         12150     12160     12170     12180     12190     12200  12210
Homo sapiens chromosome 1 NC_000001.11: 11783698-... (11518) AGGCTCACGGCCAGCTCGATGCCATAGTTGCGG-ATGGCAGCATCGTTGTCT---TTGATTGGCTC-AATCACG
    SARS-CoV-2 Reference Genome NC_045512.2 (11682) TGACTCTTGGTGTTTATGATTACTTAGTTTCTACACAGGAGTTTAGATATATGAATTCACAGGGACTACTCCCA
                                                                              Section 166
                                                    (12211) 12211    12220     12230     12240     12250     12260     12270         12284
Homo sapiens chromosome 1 NC_000001.11: 11783698-... (11587) TCCTTGATCTCCTGTGGCACCTCCAGCTTGGACAGCTTCACAAGCTGCCGAAGGGAGTGGTAGCCCT---GTCC
    SARS-CoV-2 Reference Genome NC_045512.2 (11756) CCCAAGAATAGCATAGATGCCTTCAAACTCAACA--TTAAATTGTTG--GGTGTTGGTGGCAAACCTTGTATCA
                                                                              Section 167
                                                    (12285) 12285 12290     12300     12310     12320     12330     12340            12358
Homo sapiens chromosome 1 NC_000001.11: 11783698-... (11658) AAG-----ACATGAGGGGCTG---TGGCGGCTGC----TGGCCAGCCCGTCTCCCACCCTAAATACTTCTCCCC
    SARS-CoV-2 Reference Genome NC_045512.2 (11826) AAGTAGCCACTGTACAGTCTAAAATGTCAGATGTAAAGTGCACATCAGTAGTCTTACTCTCAGTTTTGCAACAA
                                                                              Section 168
                                                    (12359) 12359      12370     12380     12390     12400     12410     12420       12432
Homo sapiens chromosome 1 NC_000001.11: 11783698-... (11720) CTTCCATCATCCATGCCAACCACAGTGTCAGGCAGAGGGACCCACGAAAGTCCGATCATATTCTAGGAGC----
    SARS-CoV-2 Reference Genome NC_045512.2 (11900) CTCAGAGTAGAATCATCATCTAAATTGTGGGCTCAATGTGTCCAGTTACA--CAATGACATTCTCTTAGCTAAA
```

Section 169

```
                                                                 12433 12440      12450      12460      12470      12480      12490        12506
                                                         (12433)
Homo sapiens chromosome 1 NC_000001.11: 11783698-... (11790) -ACAC--CTG--GGCTTGGCCTGCC-GTTTTCTCCTCTTTTT-TCTGTTTTTAGAGACTGAGTCTCACTGCTCT
SARS-CoV-2 Reference Genome NC_045512.2 (11972) GATACTACTGAAGCCTTTGAAAAAATGGTTTCACTACTTTCTGTTTTGCTTTCCATGCAGGGTG-CTGTAGACA
```

Section 170

```
                                                                 12507         12520      12530      12540      12550      12560      12570   12580
                                                         (12507)
Homo sapiens chromosome 1 NC_000001.11: 11783698-... (11857) CAAACGCCAGGGCTCAAGCCATCCT--CCTGCCT--CAGCTTCTCAAG---TAGCCGG-GACTACAGGCACGTG
SARS-CoV-2 Reference Genome NC_045512.2 (12045) TAAACAAGCTTTGTGAAGAAATGCTGGACAACAGGGCAACCTTACAAGCTATAGCCTCAGAGTTTAGTTCCCTT
```

Section 171

```
                                                                 12581    12590      12600      12610      12620      12630      12640        12654
                                                         (12581)
Homo sapiens chromosome 1 NC_000001.11: 11783698-... (11923) CCACTATGCCCAGCTTTC-CCACTTTTTACAGAGTAGAGCAGCTGCCCTAAGCATTCCAGCCTTGGGTTGAGCC
SARS-CoV-2 Reference Genome NC_045512.2 (12119) CCATCATATGCAGCTTTTGCTACTGCTCAAGAAGCTTATGAGCAGGCTGTTGCTAATGGTGATTCTGAAGTTGT
```

Section 172

```
                                                                 12655 12660      12670      12680      12690      12700      12710            12728
                                                         (12655)
Homo sapiens chromosome 1 NC_000001.11: 11783698-... (11996) AGCCTTTTAGCCCTAGATGTGTCTGTTGGTAACATGGTGTCA-TCTAACCCAGCTGTTCCCATGCTGCACTGAT
SARS-CoV-2 Reference Genome NC_045512.2 (12193) TCTTAAAAAGTTGAAGAAGTCTTTGAATGTGGCTAAATCTGAATTTGACCGTGATGCAGCCATGCAACG----T
```

Section 173

```
                                                                 12729      12740      12750      12760      12770      12780      12790      12802
                                                         (12729)
Homo sapiens chromosome 1 NC_000001.11: 11783698-... (12069) CATCCGAATCACCTGCCAAGCTCAAGAAGTAAAACACACATTCCGAGGACCTCCCTCAGATTTACTGAGTGAGA
SARS-CoV-2 Reference Genome NC_045512.2 (12263) AAGTTGGAAAAGATGGCT-GATCAAGCTATGACCCAAATGTATAAACAGGCTAGATCTGAGGACAAGAGGGCAA
```

Section 174

```
                                                                 12803 12810      12820      12830      12840      12850      12860            12876
                                                         (12803)
Homo sapiens chromosome 1 NC_000001.11: 11783698-... (12143) ACCTCCAGGGGTGGGACCTGGAGATCCACTTTTTGAAAAAGCTTATCAAGTGG--TTCTGATGA---CAGCCAC
SARS-CoV-2 Reference Genome NC_045512.2 (12336) AAGTT-ACTAGTGCTA--TGCAGACAATGCTTTTCACTATGCTTAGAAAGTTGGATAATGATGCACTCAACAAC
```

Section 175

```
                                                                 12877         12890      12900      12910      12920      12930      12940   12950
                                                         (12877)
Homo sapiens chromosome 1 NC_000001.11: 11783698-... (12212) CTTTGGGAAACACTGATTTAAGCAGGATTTGTAATGAGAATTAGAATCCCTTTTG--------GTGATGCT--T
SARS-CoV-2 Reference Genome NC_045512.2 (12407) ATTATC--AACAATGCAAGA-GATGGTTGTGTTCCCTTGAACATAATACCTCTTACAACAGCAGCCAAACTAAT
```

Section 176

(12951) 12951 12960 12970 12980 12990 13000 13010 13024

Homo sapiens chromosome 1 NC_000001.11: 11783698-... (12276) GTTGGCCAAAGTACAACA-AACCCCTCAACAGACACTGTTGCTGGG-------TT--TTGGGGGGAAAATTAGA
SARS-CoV-2 Reference Genome NC_045512.2 (12478) GGTTGTCATACCAGACTATAACACATATAAAAATACGTGTGATGGTACAACATTTACTTATGCATCAGCATTGT

Section 177

(13025) 13025 13030 13040 13050 13060 13070 13080 13098

Homo sapiens chromosome 1 NC_000001.11: 11783698-... (12340) GGTAACCAAAATGGGGTGGCCAAGCAACGCTGTGCAAGTTCTGGACCTGAGAGGAGATC-TGGGAAGAACTCAG
SARS-CoV-2 Reference Genome NC_045512.2 (12552) GGGAAATCCAACAGGTTGTAGATGCA--GATAGTAAAATTGTTCAACTTAGTGAAATTAGTATGGACAATTCAC

Section 178

(13099) 13099 13110 13120 13130 13140 13150 13160 13172

Homo sapiens chromosome 1 NC_000001.11: 11783698-... (12413) CGAACTCAGCACT-CCACCCAGAGCCCCCAGCCTGTGCGAGGACGGTGCGGTGAGAGTGGGGTGGAGGGAGCTT
SARS-CoV-2 Reference Genome NC_045512.2 (12624) CTAATTTAGCATGGCCTCTTATTGTAACAGCTTTAAGGGCCAATTCTGCTGTCAAATTACAGAATAATGAGCTT

Section 179

(13173) 13173 13180 13190 13200 13210 13220 13230 13246

Homo sapiens chromosome 1 NC_000001.11: 11783698-... (12486) ATGG--GCTCTCCTGGGCCCCTCACCTGGATGGGAAAGATCCCGGGGACGATGGGGCAAGTGATGCCCATGTCG
SARS-CoV-2 Reference Genome NC_045512.2 (12698) AGTCCTGTTGCACTACGACAGATGTCTTG-TGCTGCCGGTACTACACAAACTGCTTGCACTGATGACAATGCGT

Section 180

(13247) 13247 13260 13270 13280 13290 13300 13310 13320

Homo sapiens chromosome 1 NC_000001.11: 11783698-... (12558) GTGCATGC--CTTCACA--AAGCGGAAGAATGTGTCAGCCTCAAAGAAAAGCTGCGTGA--TGA--TGAAATCG
SARS-CoV-2 Reference Genome NC_045512.2 (12771) TAGCTTACTACAACACAACAAAGGGAGGTAGGTTTGTACTTGCACTGTTATCCGATTTACAGGATTTGAAATGG

Section 181

(13321) 13321 13330 13340 13350 13360 13370 13380 13394

Homo sapiens chromosome 1 NC_000001.11: 11783698-... (12624) GCTCCCGCAGACACCTTCTCCTTCAAGTGCTTCAGGTCAGCCTCAAAGCTCCCTGCTTCGGGGTGGCCTTTGGG
SARS-CoV-2 Reference Genome NC_045512.2 (12845) GCT-----AGA----TTC-CCTAAGAGTGATGGAACTGGTACTATCTATACAGAACT--GGAACCACCTTGTAG

Section 182

(13395) 13395 13400 13410 13420 13430 13440 13450 13468

Homo sapiens chromosome 1 NC_000001.11: 11783698-... (12698) GTAACCTGCCAATAGGGATGACAGTCAGGA-GAGGCTGGCCTCCACCTGTTCAAGGCGAGGGATGAAGAGACCA
SARS-CoV-2 Reference Genome NC_045512.2 (12907) GTTTGTTACAGACACACCTAAAGGTCCTAAAGTGAAGTATTTATACTTTATTAAAGGATTAAACAACCTAAATA

```
                                                                    Section 183
                                                   (13469) 13469      13480          13490          13500          13510          13520          13530     13542
Homo sapiens chromosome 1 NC_000001.11: 11783698-... (12771) CAGGGACTGGGCAGAGAGAGTCCTCTGCTTTGG--GGGCTCTGATCTTGCACCTTCCAACAGCACAGCACAGCC
SARS-CoV-2 Reference Genome NC_045512.2 (12981) GAGGTATGGTACTTGGTAGTTTAGCTGCCACAGTACGTCTACAAGCTGGTAA--TGCAACAGAAGTGC-CTGCC

                                                                    Section 184
                                                   (13543) 13543  13550          13560          13570          13580          13590          13600          13616
Homo sapiens chromosome 1 NC_000001.11: 11783698-... (12843) TGAACTAAAACAGTGAGTGGCTGGGCTCATGACTGATATGCTTTCTGACCCCAGAGATGACTCTCAGGGAGGTG
SARS-CoV-2 Reference Genome NC_045512.2 (13052) AATTCAACTGTATTATCTTTCTGTGCTTTTGCTGTAGATGCTGCTAAAGCTTACAAAGATTATCTAGCTAG-TG

                                                                    Section 185
                                                   (13617) 13617          13630          13640          13650          13660          13670          13680 13690
Homo sapiens chromosome 1 NC_000001.11: 11783698-... (12917) GCTCAGAGCCCAGTTCCAAGAGTGGGGGTGGATTGCTCCAAACTCCCACACTGTCCTGCCTTGAGCAGCCAAGC
SARS-CoV-2 Reference Genome NC_045512.2 (13125) GGGGACAACCAATCACTAATTGTGTTAAGATGTTG-TGTACAC----ACACTG----GTACTGGTCAGGCAATA

                                                                    Section 186
                                                   (13691) 13691     13700          13710          13720          13730          13740          13750      13764
Homo sapiens chromosome 1 NC_000001.11: 11783698-... (12991) AAATCAGGCCTGCACTTGGTGGCCATAGAAAAGAGAAAATGCTACAGTTA-GCACTGGCTGGGCAGCATCTGGG
SARS-CoV-2 Reference Genome NC_045512.2 (13190) A---CAGT--TACACCGGAAGCCAATATGGATCA-AGAATCCTTTGGTGGTGCATCGTGTTGTCTGTA-CTGCC

                                                                    Section 187
                                                   (13765) 13765 13770          13780          13790          13800          13810          13820          13838
Homo sapiens chromosome 1 NC_000001.11: 11783698-... (13064) GTAGTAAGCTTGACTTTTCTCTAGGCACCGGATTAAACATTGTTTTTAATGTAACACTGAAATTACTCAGGGGT
SARS-CoV-2 Reference Genome NC_045512.2 (13257) GTTGCCACATAGATCATCCAAATCCTAAAGGATTTTG--TGACTTAAAAGGTAAGTATG----TACAAATACCT

                                                                    Section 188
                                                   (13839) 13839      13850          13860          13870          13880          13890          13900     13912
Homo sapiens chromosome 1 NC_000001.11: 11783698-... (13138) CCAAGATAGTGAAAAGCTAAAAACCAAGTTACACAAGGAAGAACTGAAGAAAGTGATGTTTTCTTCAAAGAAAA
SARS-CoV-2 Reference Genome NC_045512.2 (13325) ACAACTT-GTGCTAATG-ACCCTGTGGGTTTTACACTTAAAAACACA-GTCTGTACCGTCTGCGGTATGTGGAA

                                                                    Section 189
                                                   (13913) 13913  13920          13930          13940          13950          13960          13970          13986
Homo sapiens chromosome 1 NC_000001.11: 11783698-... (13212) TAATGGATGGCAGGGTGCGGTGGCTCATGTCTGTAATCCCAGCACTTTGGGAGGCCAAGGCAGGA--GGATGAC
SARS-CoV-2 Reference Genome NC_045512.2 (13396) AGGTT-ATGGCTGTA-GTTGTGA-TCAACTCCGCGAACCCATG-CTTCAGTCAGCTGATGCACAATCGTTTTTA
```

Section 190

```
                                                              (13987) 13987        14000     14010     14020     14030     14040     14050 14060
Homo sapiens chromosome 1 NC_000001.11: 11783698-...          (13284) CTGAGGTCAGGAGTTCGAGACCAGCCTGGCCAACATGGTAAAACCCCGTCTCTACCAAAAATACAAAAATTAGC
SARS-CoV-2 Reference Genome NC_045512.2                       (13466) AACGGGTTTGCGGTGTAAGTGCAGCCCGTCTTACACCGTGCGGCACAGGCACTAGTACTGATGTCG----TATA
```

Section 191

```
                                                              (14061) 14061    14070     14080     14090     14100     14110     14120      14134
Homo sapiens chromosome 1 NC_000001.11: 11783698-...          (13358) CAGGTGTGGTGGTGTATGCCTGTAATAGCTACTCAGGAGGCTGAGTCAGGAAAATTGCTTGAACCCACGAGACA
SARS-CoV-2 Reference Genome NC_045512.2                       (13536) CAGGGCTTTTGACATCTAC----AAT-GATA---AAGTAGCTGGTTTTGCTAAATTCCTAAAAACTAATTGTTG
```

Section 192

```
                                                              (14135) 14135 14140     14150     14160     14170     14180     14190       14208
Homo sapiens chromosome 1 NC_000001.11: 11783698-...          (13432) GAGGTTGCAGTGAGCCGA-GATCGTACCATTGCACTCTAGCCTGGGCAACGAG---AGCGAAACTCCGTCTCAA
SARS-CoV-2 Reference Genome NC_045512.2                       (13602) TCGCTTCCAAGAAAAGGACGAAGATGACAATTTAATTGATTCTTACTTTGTAGTTAAGAGACACACTTTCTCTA
```

Section 193

```
                                                              (14209) 14209      14220     14230     14240     14250     14260     14270    14282
Homo sapiens chromosome 1 NC_000001.11: 11783698-...          (13502) AAAAAAAAAAAAAAAAAAGAATGAAAAGAATACAGAAACTCCTTTCAAATGT--CCAAAGGTCTGTCCTGGGGA
SARS-CoV-2 Reference Genome NC_045512.2                       (13676) ACTACCAACATGAAGAAACAATTTATAATTTACTTAAGGATTGTCCAGCTGTTGCTAAACATGACTTCTTTAAG
```

Section 194

```
                                                              (14283) 14283  14290     14300     14310     14320     14330     14340        14356
Homo sapiens chromosome 1 NC_000001.11: 11783698-...          (13574) AAAAGGATCCACCATAGGTGAGTTTGTTTTGTTTTGTTTCAGAGACAGGGTCTTGCTCTGTCACCCAGGCTAGA
SARS-CoV-2 Reference Genome NC_045512.2                       (13750) TTTAGAATAGAC----GGTGACATGGTACCACATA-TATCAC-GTCAACGTCTTACTAAATACACAATGGCAGA
```

Section 195

```
                                                              (14357) 14357        14370     14380     14390     14400     14410     14420 14430
Homo sapiens chromosome 1 NC_000001.11: 11783698-...          (13648) --------GTGCAGTGATACAATC--ATG--GCTCACTGTAGCC--TT----GAACTCCGGGCACA----AGT
SARS-CoV-2 Reference Genome NC_045512.2                       (13818) CCTCGTCTATGCTTTAAGGCATTTTGATGAAGGTAATTGTGACACATTAAAAGAAATACTTGTCACATACAATT
```

Section 196

```
                                                              (14431) 14431    14440     14450     14460     14470     14480     14490      14504
Homo sapiens chromosome 1 NC_000001.11: 11783698-...          (13700) GATCTCCCACT--TCAGTCTCCTGAGTAG--CTTGGACTACA--GATGCAAGCCACCACATCTGGCTAAT-TAA
SARS-CoV-2 Reference Genome NC_045512.2                       (13892) GTTGTGATGATGATTATTTCAATAAAAAGGACTGGTATGATTTTGTAGAAAACCCAGATATATTACGCGTATAC
```

Section 197

	(14505)	14505 14510 14520 14530 14540 14550 14560 14578
Homo sapiens chromosome 1 NC_000001.11: 11783698-...	(13767)	AAAAAATTTTTTTAAT---TAATTATTTATTTTTTTAGAGATGGGGTCTTGCTATCTTGTCCAG-GTTTGTCTGG
SARS-CoV-2 Reference Genome NC_045512.2	(13966)	GCCAACTTAGGTGAACGTGTACGCCAAGCTTTGTTAAAAACAGTACAATTCTGTGATGCCATGCGAAATGCTGG

Section 198

	(14579)	14579 14590 14600 14610 14620 14630 14640 14652
Homo sapiens chromosome 1 NC_000001.11: 11783698-...	(13837)	AACTTCTGGGCTCAAGCAATCCTCCCACCTTGGCCTCCC---AAAGTGCTGAGATTACAGGCATGAGCCACTGT
SARS-CoV-2 Reference Genome NC_045512.2	(14040)	TATTGTTGGTGTACTGACATTAGATAATCAAGATCTCAATGGTAACTGGTATGATTTCGGTGATTTCATACAA-

Section 199

	(14653)	14653 14660 14670 14680 14690 14700 14710 14726
Homo sapiens chromosome 1 NC_000001.11: 11783698-...	(13908)	ACCCAGCCCCCTAGTGAAGTTCTTTAT-TAG--TCATATTCTGTGTTGTTTCTAATGGCAGAACCAGCATCCGG
SARS-CoV-2 Reference Genome NC_045512.2	(14113)	ACCACGCCAGGTAGTGGAGTTCCTGTTGTAGATTCTTATTATTCATTGTT---AATGCCTATATTAACCTT--G

Section 200

	(14727)	14727 14740 14750 14760 14770 14780 14790 14800
Homo sapiens chromosome 1 NC_000001.11: 11783698-...	(13979)	TTCAGAGAGCTGGCT--AGAGT-ACTATAAGCCACTCACAAAACAATGAGCTCC------TGGAGTCTGAAAAC
SARS-CoV-2 Reference Genome NC_045512.2	(14182)	ACCAGGGCTTTAACTGCAGAGTCACATGTTGACACTGACTTAACAAAGCCTTACATTAAGTGGGATTTGTTAAA

Section 201

	(14801)	14801 14810 14820 14830 14840 14850 14860 14874
Homo sapiens chromosome 1 NC_000001.11: 11783698-...	(14044)	ACAGGGGCAGTGGATGCTGAAGACAAGATTCCAGAATTGC----TTAGGAGGTGGGATCAGATGT--C--CCAT
SARS-CoV-2 Reference Genome NC_045512.2	(14256)	ATATGACTTCACGGAAGAGAGGTTAAAACTCTTTGACCGTTATTTTAAATATTGGGATCAGACATACCACCCAA

Section 202

	(14875)	14875 14880 14890 14900 14910 14920 14930 14948
Homo sapiens chromosome 1 NC_000001.11: 11783698-...	(14110)	AGGCTCCTTACTGGCTCTGTGA-ACTCTCTGACCCCTTTCCTCAATGACTAAACTT--ACTAGCCGCCTTGCTC
SARS-CoV-2 Reference Genome NC_045512.2	(14330)	ATTGTGTTAACTGTTTGGATGACAGATGCATTCTGCATTGTGCAAACTTTAATGTTTTATTCTCTACAGTGTTC

Section 203

	(14949)	14949 14960 14970 14980 14990 15000 15010 15022
Homo sapiens chromosome 1 NC_000001.11: 11783698-...	(14181)	GGTCAGGCAAGCA--GGATGACCATAGGCCTTCATGAAACTACATGGAGTGC---TGGTTTCATCAGCCCAAGC
SARS-CoV-2 Reference Genome NC_045512.2	(14404)	CCACCTACAAGTTTTGGACCACTAGTGAGAAAAATATTTGTTGATGGTGTTCCATTTGTAGTTTCAACTGGATA

Section 204

	(15023)	15023 15030 15040 15050 15060 15070 15080 15096
Homo sapiens chromosome 1 NC_000001.11: 11783698-...	(14250)	CCAGGGCAGGACTCTGAGCCAGGCGGAAGGCCAAGCTGCTTTGCTCTGATAAGGC-AGTTCCGTTCTTAAGAAA
SARS-CoV-2 Reference Genome NC_045512.2	(14478)	CCACTTCAGAGAGCTAGGTGTTGTACATAATCAGGATG-TAAACTTACATAGCTCTAGACTTAGTTTTAAGGAA

Section 205

	(15097)	15097 15110 15120 15130 15140 15150 15160 15170
Homo sapiens chromosome 1 NC_000001.11: 11783698-...	(14323)	C--CCAGAGTCATCAAAAAATGAATATAAAAACTATTGTTTCAAGGGGTAAGGGAGTGGGGGATAAA-GCAATG
SARS-CoV-2 Reference Genome NC_045512.2	(14551)	TTACTTGTGTATGCTGCTGACCCTGCTATGCACGCT-GCTTCT---GGTAATCTATTACTAGATAAACGCACTA

Section 206

	(15171)	15171 15180 15190 15200 15210 15220 15230 15244
Homo sapiens chromosome 1 NC_000001.11: 11783698-...	(14394)	-GGGCTTCACACT---TGCAATAACAAAGCTATTTTCCCAGTAGGAATTTCAGAAACAACAAGGCTTTTTAATA
SARS-CoV-2 Reference Genome NC_045512.2	(14621)	CGTGCTTTTCAGTAGCTGCACTTACTAA-CAATGTTGCTTTTCAAACTGTCAAACCCGGTAA----TTTTAACA

Section 207

	(15245)	15245 15250 15260 15270 15280 15290 15300 15318
Homo sapiens chromosome 1 NC_000001.11: 11783698-...	(14464)	GCTGAAAAGTATAGCTA--CAATACCTAAATATCACCGAACAAATCTAGCATTTGATTACGTATAGATTTTGCT
SARS-CoV-2 Reference Genome NC_045512.2	(14690)	AA-GACTTCTATGACTTTGCTGTGTCTAAGGGTTTCTTTAAGGAAGGAAGTTCTGTTGAATTAAAACACTTCTT

Section 208

	(15319)	15319 15330 15340 15350 15360 15370 15380 15392
Homo sapiens chromosome 1 NC_000001.11: 11783698-...	(14536)	ATTTACTCTATTTTTCTATCATG--AACAGC--TGAGGCTTTCTTTTTTAAAATGTTTTTATTTTTTGTAGAG
SARS-CoV-2 Reference Genome NC_045512.2	(14763)	CTTTGCTCAGGATGGTAATGCTGCTATCAGCGATTATGACTACTATCGTTATAATCTACCAACAATGTGTGATA

Section 209

	(15393)	15393 15400 15410 15420 15430 15440 15450 15466
Homo sapiens chromosome 1 NC_000001.11: 11783698-...	(14606)	ACACGGTCTCACTGTGTTGCCCAGGCTGGTCTTGATCTCCTGCCTCAGTCTCCCAAAGCTCTGGGATTACAGGC
SARS-CoV-2 Reference Genome NC_045512.2	(14837)	TCAGACAACTACTAT-TTGTAGTTGAAGTTGTTGATAAGTACTTTGATTGTTACGATGGTGGCTGTATTAATGC

Section 210

	(15467)	15467 15480 15490 15500 15510 15520 15530 15540
Homo sapiens chromosome 1 NC_000001.11: 11783698-...	(14680)	ATGAGCTAC-CATGCCCAACAGGTTGGTGTTTTCTAATCCACTCATCTTC-ATTACCCACTTGATAAGCAAAGT
SARS-CoV-2 Reference Genome NC_045512.2	(14910)	-TAACCAAGTCATCGTCAACAACCTAGA-----CAAATCAGCTGGTTTTCCATTTAATAAATGGG--GTAAGGC

```
                                                                 Section 211
                                                  (15541) 15541    15550          15560          15570          15580          15590          15600        15614
Homo sapiens chromosome 1 NC_000001.11: 11783698-... (14752) AAGAAACCATTCAGCAACTGTGAGCTCACAGCTTCA-----CTAGTTTCCAATCATTATCACCTAACGCCCATC
   SARS-CoV-2 Reference Genome NC_045512.2 (14976) TAGACTTTATTATGATTCAATGAGTTATGAGGATCAAGATGCACTTTTCGCATATACAAAACGTAATGTC-ATC
                                                                 Section 212
                                                  (15615) 15615 15620          15630          15640          15650          15660          15670             15688
Homo sapiens chromosome 1 NC_000001.11: 11783698-... (14821) ACGGGTGCATATTCCCCCAGTAACTGAC-TAT---ATAATGGCTGATACAAGCGATGACAATTTAACAGTTGGA
   SARS-CoV-2 Reference Genome NC_045512.2 (15049) CCTACT--ATAACTCAAATGAATCTTAAGTATGCCATTAGTGCAAAGAATAGAGCTCGCACCGTAGCTGGTGTC
                                                                 Section 213
                                                  (15689) 15689        15700          15710          15720          15730          15740          15750     15762
Homo sapiens chromosome 1 NC_000001.11: 11783698-... (14891) AATGTCTGT---TCTCTGACTCCTAATCAGTG-CTTCCAGACTTAGCACCTAAACACCCCTGTTGACAGAATG
   SARS-CoV-2 Reference Genome NC_045512.2 (15121) TCTATCTGTAGTACTATGACCAATAGACAGTTTCATCAAAAATTATTGAAATCAATAGCCGCCACTAGAGGAGC
                                                                 Section 214
                                                  (15763) 15763  15770          15780          15790          15800          15810          15820          15836
Homo sapiens chromosome 1 NC_000001.11: 11783698-... (14961) GACATGTCCATCGGGGGTTAGGGACAAGGCCAAAGT--TACTCACAACATGT---AATTTTCTTGAAGCCACGT
   SARS-CoV-2 Reference Genome NC_045512.2 (15195) TAC-TGTAGTAATTGGAACAAGCA-AATTCTATGGTGGTTGGCACAACATGTTAAAAACTGTTTATAGTGATGT
                                                                 Section 215
                                                  (15837) 15837            15850          15860          15870          15880          15890          15900 15910
Homo sapiens chromosome 1 NC_000001.11: 11783698-... (15030) GGTTTGTTTTTTGGGTTTTTTGTTTTGTTTTTTTGAGACGGAGTCTGGCTCTGTTGCCCAGGCTACAGTGCAT
   SARS-CoV-2 Reference Genome NC_045512.2 (15267) AGAAAACCCTCACC-TTATGGGTTGGGATTATCCTAAATGTGA---------TAGAGCCATGCCTAACATGCTT
                                                                 Section 216
                                                  (15911) 15911    15920          15930          15940          15950          15960          15970        15984
Homo sapiens chromosome 1 NC_000001.11: 11783698-... (15104) TGGTGCGATCTCGGCTCACTGCAAGCTCCGCCTCCTGGGTTCAAGCGATTCTCCTGCCTCAGCCTACCAAGTAG
   SARS-CoV-2 Reference Genome NC_045512.2 (15331) AGAAT---TATGGCCTCACTT---GTTCTTGCTCG------CAAACATACAACGTGTTGTAGCTTGTCACACCG
                                                                 Section 217
                                                  (15985) 15985 15990          16000          16010          16020          16030          16040             16058
Homo sapiens chromosome 1 NC_000001.11: 11783698-... (15178) CTGGGACTACAGGCACGCGCCACCATGCCCGGCTAATTTTTGTATTTTTAGTAGAGACGGGGTTT-CACTATGT
   SARS-CoV-2 Reference Genome NC_045512.2 (15393) TTTCTA-TAGATTAGCTAATGAGTGTGCTCAAGTATTGAGTGAAATGGTCAT-GTGTGGCGGTTCACTATATGT
```

Section 218

	(16059)	16059 … 16132
Homo sapiens chromosome 1 NC_000001.11: 11783698-...	(15251)	TGGACAGGATGGTCTCAAACTCCTGATCTCATGATCCACCCGCCTTGGCCTCCCAAAGTGCTGGGATTATAGGC
SARS-CoV-2 Reference Genome NC_045512.2	(15465)	TAAACCAGGTGG----AACCTCATCAGGAGATGCCACAACTGCTTATGCTA---ATAGTGTT-----TTTAACA

Section 219

	(16133)	16133 … 16206
Homo sapiens chromosome 1 NC_000001.11: 11783698-...	(15325)	TTCAGCCACCGCACCTGGCCTTTTTTTTTTAGTTCATATCACTTTAGATGCTA-CTCCATAAC--ATAGCCATG
SARS-CoV-2 Reference Genome NC_045512.2	(15527)	TTTGTCAAGCTGTCACGGCCAATGTTAATGCACTTTTATCTACT--GATGGTAACAAAATTGCCGATAAGTATG

Section 220

	(16207)	16207 … 16280
Homo sapiens chromosome 1 NC_000001.11: 11783698-...	(15396)	TTTAATATAAAAATGGTAGGATTTCTCCCAGAATAT-TGGCAAAAAAACTGTCATCCCAGAAAATGTGCCAATC
SARS-CoV-2 Reference Genome NC_045512.2	(15599)	TCCGCAATTTACAACACAGACTTTATGAGTGTCTCTATAGAAATAGAGATGTTGACACAGACTTTGTG--AAT-

Section 221

	(16281)	16281 … 16354
Homo sapiens chromosome 1 NC_000001.11: 11783698-...	(15469)	CCGGGACTTCCTGTCCCTGGTGCACAC-TGTCTTGCCATACCTGAGCGCTGTCTGAGACGCTGAGGGCT-CAAT
SARS-CoV-2 Reference Genome NC_045512.2	(15670)	--GAGTTTTACGCATATTTGCGTAAACATTTCTCAATGATGATACTCTCTGACGATGCTGTTGTGTGTTTCAAT

Section 222

	(16355)	16355 … 16428
Homo sapiens chromosome 1 NC_000001.11: 11783698-...	(15541)	GCCAGCCTTCAGCTTCAAGGGTGGGAGAGAAGTCCAACCCACCCATTTCTGTTGGGGGAACCTGGTCTTGTGGC
SARS-CoV-2 Reference Genome NC_045512.2	(15742)	AGCACTTATGCATCTCAAGGTCTAGTGGCTAGCATAAAGAAC---TTTAAGT---------CAGTTCTTTATTA

Section 223

	(16429)	16429 … 16502
Homo sapiens chromosome 1 NC_000001.11: 11783698-...	(15615)	TGTGGCCAATGGTGATTCTCTTTCCTGGGAA-TTTGGAACGGAGGCCTGTCCATTAGCCTGGGGAACTGGCTGG
SARS-CoV-2 Reference Genome NC_045512.2	(15804)	TCAAAACAATGTTT-TTATGTCTGAAGCAAAATGTTGGACTGAGAC-TGACC-TTA-CTAAAGGACCT--CATG

Section 224

	(16503)	16503 … 16576
Homo sapiens chromosome 1 NC_000001.11: 11783698-...	(15688)	AACTGACACTCTGCACACTCAGGAGCAGGCCAGGTGTGTGGAAAAGCATGGGTATCAGGTCT-CCCAGATGAAG
SARS-CoV-2 Reference Genome NC_045512.2	(15872)	AATTTTG-CTCTCAACATACAATGCTAGTTAAACAGGGTG-ATGATTATGTGTACCTTCCTTACCCAGATCCAT

Section 225

```
                                                        (16577) 16577        16590     16600     16610     16620     16630     16640  16650
Homo sapiens chromosome 1 NC_000001.11: 11783698-... (15761) CACAGATGTGCAGTGGAAGCGGAGATG--GGGGACCCTGTGGCCCCAGAGGG------AATGAGGGAGGGCCT
SARS-CoV-2 Reference Genome NC_045512.2 (15944) CA-AGAATCCTAGGGGCCGGCTGTTTTGTAGATGATATCGTAAAAACAGATGGTACACTTATGATTGAACGGTT
```

Section 226

```
                                                        (16651) 16651    16660     16670     16680     16690     16700     16710      16724
Homo sapiens chromosome 1 NC_000001.11: 11783698-... (15827) TGCTTTTTGACCTCTTCCAGTGCCTTGTGAGGTCTGAGGCCATCTCCTGATCTTGGGTTCCCTGCCCTCTGGAA
SARS-CoV-2 Reference Genome NC_045512.2 (16017) CGTGTCTTTAGCTATAGA--TGCTTACCCA---CTTACTAAACATCCTAATCAGGAGTATGCTGATGTCTTTCA
```

Section 227

```
                                                        (16725) 16725 16730     16740     16750     16760     16770     16780      16798
Homo sapiens chromosome 1 NC_000001.11: 11783698-... (15901) TC--CTGTTAG--TAAATATCCCTCTTTTGTGGGGTGTTGTTTTTTTTTTTTTTTTGAGATGGAGTCTTGCTCT
SARS-CoV-2 Reference Genome NC_045512.2 (16086) TTTGTACTTACAATACATAAGAAAGCTACATGATGAGTTAACAGGACACATGTTA-GACATGTATTCT-GTTAT
```

Section 228

```
                                                        (16799) 16799      16810     16820     16830     16840     16850     16860    16872
Homo sapiens chromosome 1 NC_000001.11: 11783698-... (15971) GCC-ACTGAGGCTGG-ACTGCAGCGGCACCATCTCGGCTCACTGCAACCTCCGCCTCTTGGGTCCAAGAGATTC
SARS-CoV-2 Reference Genome NC_045512.2 (16158) GCTTACTAATGATAACACTTCAAGG------TATTGGG-------AACCTGAGTTTTATGAGGCTATGT-ACAC
```

Section 229

```
                                                        (16873) 16873  16880     16890     16900     16910     16920     16930      16946
Homo sapiens chromosome 1 NC_000001.11: 11783698-... (16043) TCCTGGCTCAGCCTCCTGAGTAGCTGGGACTACAG--CCGTGCACCAC-CACACCTGGCTAATTTTTGTATTTT
SARS-CoV-2 Reference Genome NC_045512.2 (16218) ACCGCATACAGTCTTACAGGCTGTTGGGGCTTGTGTTCTTTGCAATTCACAGACTTCATTAAGATGTGGTGCTT
```

Section 230

```
                                                        (16947) 16947        16960     16970     16980     16990     17000     17010  17020
Homo sapiens chromosome 1 NC_000001.11: 11783698-... (16114) TAGTAGAGACGGGGTTTCGCTATGTTGGCCAGGCAGGTCTCA--AACTCCTG---ACCTCAGGTGATCTG--CC
SARS-CoV-2 Reference Genome NC_045512.2 (16292) GCATACGTA-GACCATTCT-TATGTTGTAAATGCTGTTACGACCATGTCATATCAACATCACATAAATTAGTCT
```

Section 231

```
                                                        (17021) 17021    17030     17040     17050     17060     17070     17080      17094
Homo sapiens chromosome 1 NC_000001.11: 11783698-... (16181) TGCCTCGGCCTCCCAAAGTGTTGGG-GTTACAGGT-GTGA-GCCACCGCGCCCAGACCCCTCTTTCGTTTTAAA
SARS-CoV-2 Reference Genome NC_045512.2 (16364) TGTCTGTTAATCCGTATGTTTGCAATGCTCCAGGTTGTGATGTCACAGATGT--GACTCAACTTTACTTAGGAG
```

Section 232

	(17095)	17095 17100 17110 17120 17130 17140 17150 17168
Homo sapiens chromosome 1 NC_000001.11: 11783698-...	(16252)	GAAAAGAATAAGGCCAAGCAGGCTAAGGGCACAGCCAGGCAGAGGAACAAAAAGCTAGGGGTCGATGGTGGCAG
SARS-CoV-2 Reference Genome NC_045512.2	(16436)	G-------TATGAGCTATTATTGTAAAT-----------CACATAAACCACCCATTAGTTTTCCATTGTGTGCT

Section 233

	(17169)	17169 17180 17190 17200 17210 17220 17230 17242
Homo sapiens chromosome 1 NC_000001.11: 11783698-...	(16326)	GCCCAGGAGGGAGTGGGTGATACAAAAGGAGCAAGGACATGGTGCAGTTCACCT--CTGCATGTCCTTCA--T
SARS-CoV-2 Reference Genome NC_045512.2	(16492)	AATGGACAAGTTTTTGGTTTATATAAAAATAC--ATGTGTTGGTAGCGATAATGTTACTGACTTTAATGCAATT

Section 234

	(17243)	17243 17250 17260 17270 17280 17290 17300 17316
Homo sapiens chromosome 1 NC_000001.11: 11783698-...	(16396)	CCAGCATG-GACCC-ATGAGTGCCCTGATCAC-TGTGTCCTGAACCGAACACCGACAGGCTCTCTGGGCATCT
SARS-CoV-2 Reference Genome NC_045512.2	(16564)	GCAACATGTGACTGGACAAATGCTGGTGATTACATTTTAGCTAACACCTGTACTGAAAGACTCAA---GCTTTT

Section 235

	(17317)	17317 17330 17340 17350 17360 17370 17380 17390
Homo sapiens chromosome 1 NC_000001.11: 11783698-...	(16467)	CAGCCTCCCTAGCTCCATCCCCGCCACCAGGATGATCCAGCCACTCACCTGCCACACAGATGTCAAAGTAGTCA
SARS-CoV-2 Reference Genome NC_045512.2	(16635)	T-GCAGCAGAAACGC--TCAAAGCTACT--GAGGAGACATTTA---AACTGTCTTATGGTATTGCTACTGTACG

Section 236

	(17391)	17391 17400 17410 17420 17430 17440 17450 17464
Homo sapiens chromosome 1 NC_000001.11: 11783698-...	(16541)	CCAAACTCACTTCGGATGTGCTTCACCAGGTCCACTGCGTAGTTGAAGCCTCCCTCCTCCTCTTCCCACTGGTC
SARS-CoV-2 Reference Genome NC_045512.2	(16701)	TGAAGTGCTGTCTGACAGAGAATTACATCTTTCA-TGGGAAGTTG--GTAAACCTAGACCAC----CACTTAAC

Section 237

	(17465)	17465 17470 17480 17490 17500 17510 17520 17538
Homo sapiens chromosome 1 NC_000001.11: 11783698-...	(16615)	ACCTATTGGGTCTGCAGGGTTGGTGGAGGTCATAGTCACTGGGTCTCACCCAACCTCCAGAGACCCTTTCTGTT
SARS-CoV-2 Reference Genome NC_045512.2	(16768)	CGAAATTATGTCTTTACTGGTTATCGTGTAACTAAAAACAGTAAAGTAC--AAATAGGAGAGTACACCTTTGAA

Section 238

	(17539)	17539 17550 17560 17570 17580 17590 17600 17612
Homo sapiens chromosome 1 NC_000001.11: 11783698-...	(16689)	CCCTCTACCTAGCCCCGACACCCAGGACTGCGAGTCCCTGGAAGGCAGAAACTTCATATTATTTAGGTTTGGTT
SARS-CoV-2 Reference Genome NC_045512.2	(16840)	AAAGGTGACTATGGT-GATGCTGTTGTTTAC-----CGAGGTA--CAACAACTTACAA--ATTAAATGTTGGTG

Section 239

(17613)	17613 17620 17630 17640 17650 17660 17670 17686
Homo sapiens chromosome 1 NC_000001.11: 11783698-... (16763)	CGTCTCTTTCTAGCACTTGGTAAGACAGCAGGAGGGAAAAAAGCAGCC-CACCAGTTCAATATTCACTGCATGC
SARS-CoV-2 Reference Genome NC_045512.2 (16904)	ATTATTTTGTGCTGACATCACAT-ACAGTAATGCCATTAAGTGCACCTACACTAGTGCCACAAGAGCACTATGT

Section 240

(17687)	17687 17700 17710 17720 17730 17740 17750 17760
Homo sapiens chromosome 1 NC_000001.11: 11783698-... (16836)	TGACCCTGTGCTGGCGGTGGAGAATAGGAGACCACCGCTGCTTCTCACCGTGCCTGGAGGATTGGAGAGCCCTT
SARS-CoV-2 Reference Genome NC_045512.2 (16977)	T-AGAAT-TACTGGCTT------ATAC----CCAACACTCAATATCTCAGAT----GAGTTTTCTAGCAATGTT

Section 241

(17761)	17761 17770 17780 17790 17800 17810 17820 17834
Homo sapiens chromosome 1 NC_000001.11: 11783698-... (16910)	CCCTACTTCTACCTGGGCATGCCTGCCACCCTCTCC--CATCCCACGGCCTTGGGGCTCCTGCCTCCTGGGAGA
SARS-CoV-2 Reference Genome NC_045512.2 (17035)	GCAAA-TTATCAAAAGGTTGGTATGCAAAAGTATTCTACACTCCAGGGACCACCTGGTACTGGTAA--GAGTCA

Section 242

(17835)	17835 17840 17850 17860 17870 17880 17890 17908
Homo sapiens chromosome 1 NC_000001.11: 11783698-... (16982)	ATTTTCTACTCATCTCAAG-TGAAGGGGTCTTGTGGTAGGGAGCACGAGGCCATAG-GATCCCACCCATCAGGC
SARS-CoV-2 Reference Genome NC_045512.2 (17106)	TTTTGCTATTGGCCTAGCTCTCTACTACCCTTCTGCTCG----CAT-AGTGTATACAGCTTGCTCTCATGCCGC

Section 243

(17909)	17909 17920 17930 17940 17950 17960 17970 17982
Homo sapiens chromosome 1 NC_000001.11: 11783698-... (17054)	TCCCTCTTCCCACTCTTAGGTGGCCTGACAACAGCGGGCACCAGAGGCCTGACCGGCTATTCTCACTCCTGGAT
SARS-CoV-2 Reference Genome NC_045512.2 (17175)	TGTTGATGCACTATGTGAGAAGGCATTAAAATATTTGCCTATAGATAAATGTAGTAGAATTATACCTGCACG-T

Section 244

(17983)	17983 17990 18000 18010 18020 18030 18040 18056
Homo sapiens chromosome 1 NC_000001.11: 11783698-... (17128)	ACTCTGTTACTCTGTGATTGGGCCAAGTGCTGGGTGCAGCCAGCAATCGATGCAGT--GGTGTAGGCAAGGCAG
SARS-CoV-2 Reference Genome NC_045512.2 (17248)	GCTCGTGTAGAGTGTTTT---GATAAATTCAAAGTGAATTCAACATTAGAA-CAGTATGTCTTTTGTACTGTAA

Section 245

(18057)	18057 18070 18080 18090 18100 18110 18120 18130
Homo sapiens chromosome 1 NC_000001.11: 11783698-... (17200)	AGGGTTATCAGGCCGCGACTGCATTCCTGACGGAACTCCTATGTCGAGGTCAAGAGGTGGAAGAGGTGGCT-GC
SARS-CoV-2 Reference Genome NC_045512.2 (17318)	ATGCATTGCCTGAGACGACAGCAGATATAGTTGT-CTTTGATGAA-ATTTCAATGGCCACAAATTATGATTTGA

```
                                                                                Section 246
                                                         18131    18140     18150     18160     18170     18180     18190        18204
                                                (18131)
Homo sapiens chromosome 1 NC_000001.11: 11783698-... (17273) CTGTGTGTGGTGCCCAGGATGACTCACTACCCACAATGCCTACT-----GACACTACTGGCGGGGATGGGATCC
SARS-CoV-2 Reference Genome NC_045512.2 (17390) GTGT-TGTCAATGCCAG-ATTACGTGCTAAGCACTATGTGTACATTGGCGACCCTGCTCAATTACCTGC-ACCA
                                                                                Section 247
                                                         18205 18210     18220     18230     18240     18250     18260          18278
                                                (18205)
Homo sapiens chromosome 1 NC_000001.11: 11783698-... (17342) TGCGGATCCTCAGCGTGGGGCCCA--GGAAGAAGTAAAGGACCATTTCAGGAACCAAGTTCAGGCTACCAAGTG
SARS-CoV-2 Reference Genome NC_045512.2 (17461) CGCACATTGCTAACTAAGGGCACACTAGAACCAGAATATTTCAATT-CAGTGTGTAGACTTATGAAAACTATAG
                                                                                Section 248
                                                         18279      18290     18300     18310     18320     18330     18340     18352
                                                (18279)
Homo sapiens chromosome 1 NC_000001.11: 11783698-... (17414) GCCTCCGGGAAAGCCAGAACCCAGAGTGTAGGGGAGTGCTGGCTCCACACCTCCCCGCAGCGCCATGATGTTCT
SARS-CoV-2 Reference Genome NC_045512.2 (17534) G--TCCAGACATGTTCCT-CGGAACTTGTCGGCGTTGTCCTGCTGAAATTGTTGACACTG-----TGA-GTGCT
                                                                                Section 249
                                                         18353  18360     18370     18380     18390     18400     18410        18426
                                                (18353)
Homo sapiens chromosome 1 NC_000001.11: 11783698-... (17488) TCAGGCCCAGCTGCTTAGCTTTGTGCAGATGGCCCGTGATCTCCTCCAGGCGCTGACGGCAGCAGGTCATGTGC
SARS-CoV-2 Reference Genome NC_045512.2 (17599) TTGGTTTATGATAATAAGCTTAAAGCACATAAAGACAAATCAGCTCAATGCTTTAAA------ATGTTTTATA-
                                                                                Section 250
                                                         18427        18440     18450     18460     18470     18480     18490 18500
                                                (18427)
Homo sapiens chromosome 1 NC_000001.11: 11783698-... (17562) AGGATGGTCTCCAGGCCACAGTAGTTCA-CGGCGGTG--CTGGCGATCATCATGGAGGAGGTCTCCTTGTCTGA
SARS-CoV-2 Reference Genome NC_045512.2 (17666) AGGGTGTTATCACG--CATGATGTTTCATCTGCAATTAACAGGCCACAA--ATAGGCGTGGTAAGAGAATTCCT
                                                                                Section 251
                                                         18501    18510     18520     18530     18540     18550     18560        18574
                                                (18501)
Homo sapiens chromosome 1 NC_000001.11: 11783698-... (17633) GCCAGGGTCACCTGCTGGGTGCCAGGTCACGTCTATGTAGAGGGGGCCACCTGCTGCCATCCGGTCAAACCTGT
SARS-CoV-2 Reference Genome NC_045512.2 (17736) TACACGTAACCCTGCTTGGAGAAAAGCTGTCTTTATTT---------CACCTTATA--ATTCACAGAATGCTGT
                                                                                Section 252
                                                         18575 18580     18590     18600     18610     18620     18630          18648
                                                (18575)
Homo sapiens chromosome 1 NC_000001.11: 11783698-... (17707) AGGGGGTTTGTTTCTGAAGAGAGGTTCCAGGAGAAATAC--CATCCGTCACTGCCTTCCCATGGGGCCCGGGAG
SARS-CoV-2 Reference Genome NC_045512.2 (17799) AGCCTCAAAGATTTTG----GGACTACCAACTCAAACTGTTGATTCATCACAGGGCTCAGAATATGACTATGTC
```

```
                                                                                Section 253
                                                        (18649) 18649      18660    18670    18680    18690    18700    18710    18722
Homo sapiens chromosome 1 NC_000001.11: 11783698-... (17779) TTAGAGAAGCATCAATGAAGGTTGGAGACAGACCAATCAGCGTTCATATCCTGGCTT--TGCTACTTATAGCTG
   SARS-CoV-2 Reference Genome NC_045512.2 (17869) ATATTCACTCA------AACCACTGAAACAGCTCACTCTTGTAATGTAAACAGATTTAATGTTGCTATTACCAG
                                                                                Section 254
                                                        (18723) 18723 18730    18740    18750    18760    18770    18780    18796
Homo sapiens chromosome 1 NC_000001.11: 11783698-... (17851) TGTGATCTTGGG--TACATTACTTAATCTCTGTGAGTTCTGTTTCCTTATTTATAAGACACGGCAGATAGTAGT
   SARS-CoV-2 Reference Genome NC_045512.2 (17937) AGCAAAAGTAGGCATACTTTGCATAATGTCTGATAGAGA-----CCTTTATGACAAGTTGCA--ATTTACAAGT
                                                                                Section 255
                                                        (18797) 18797       18810    18820    18830    18840    18850    18860  18870
Homo sapiens chromosome 1 NC_000001.11: 11783698-... (17923) ACCCACTTCATAATAACAC-TAGGATGCTGGGCGTGGTGGCTCACGCCTGTAATCC--CAGCACT-TCAGGAGG
   SARS-CoV-2 Reference Genome NC_045512.2 (18004) -----CTTGA-AATTCCACGTAGGAATGTGGCAACTTTA---CAAGCTGAAAATGTAACAGGACTCTTTAAAGA
                                                                                Section 256
                                                        (18871) 18871   18880    18890    18900    18910    18920    18930    18944
Homo sapiens chromosome 1 NC_000001.11: 11783698-... (17993) CTGAAGTGAGTGGATCACTTGAGGTCAGGAGTTCCAGACCGCCTGGCCAACATAGTGAAACCTCATCTCTACTA
   SARS-CoV-2 Reference Genome NC_045512.2 (18069) TTGTAGTAAGGTAATCACTGGGTTACATCCTACACAGGCA-CCTACACACCTCAGTGTTGACAC---TAAATTC
                                                                                Section 257
                                                        (18945) 18945 18950    18960    18970    18980    18990    19000    19018
Homo sapiens chromosome 1 NC_000001.11: 11783698-... (18067) AAAATACAAAAATTAGCTGGGTGTGGTGGTGGGCACCTGTAATCCCAGCTACTTGAGA-GGCTGAGGCAGGAGA
   SARS-CoV-2 Reference Genome NC_045512.2 (18139) AAAACTGAAGGTTTATGTGT-TGACATACCTGGCATACCTAAGGACATGACCTATAGAAGACTCATCTCTATGA
                                                                                Section 258
                                                        (19019) 19019      19030    19040    19050    19060    19070    19080    19092
Homo sapiens chromosome 1 NC_000001.11: 11783698-... (18140) ATTGCTTGAGCCAGGAGGCAGAGGTTGCAATGAGCCAAGATCACACCACTGCACTCCAGCCTGGGCCACAGAAT
   SARS-CoV-2 Reference Genome NC_045512.2 (18212) TGGGTTTTAAAATGAATTATCAAGTT--AATG-GTTACCCTAACA----TGTTTATCACCCGCGAAGAAGCTAT
                                                                                Section 259
                                                        (19093) 19093 19100    19110    19120    19130    19140    19150    19166
Homo sapiens chromosome 1 NC_000001.11: 11783698-... (18214) GAGAC-TCTGTCT-CAAAAAAAAAAAAAAAAAAAAAAAAGCAACAA-CAACAACAAAAAACCATAACACTAGGA
   SARS-CoV-2 Reference Genome NC_045512.2 (18279) AAGACATGTACGTGCATGGATTGGCTTCGATGTCGAGGGGTGTCATGCTACTAGAGAAGCTGTTGGTACCAATT
```

Section 260

(19167)	19167 19180 19190 19200 19210 19220 19230 19240
Homo sapiens chromosome 1 NC_000001.11: 11783698-... (18285)	GAATATGACGGC-AGCTGGTTCCACAGAACTTAGAACAGTGCCTGGTGTACAAACCGAATAC-TCAGTATATGT
SARS-CoV-2 Reference Genome NC_045512.2 (18353)	TACCTTTACAGCTAGGTTTTTCTACAGGTGTTAACCTAGT---TGCTGTACCTACAGGTTATGTTGATACACCT

Section 261

(19241)	19241 19250 19260 19270 19280 19290 19300 19314
Homo sapiens chromosome 1 NC_000001.11: 11783698-... (18357)	GAGGTGCCGTTATAATCACTATTACAGAGGTAAGACACAACAACAGC---GCTATCATTTTGTGTGAGAGCAGC
SARS-CoV-2 Reference Genome NC_045512.2 (18424)	AA-------TAATACAGATTTTTCCAGAGTTAGTGCTAAACCACCGCCTGGAGATCAATTTA------AACACC

Section 262

(19315)	19315 19320 19330 19340 19350 19360 19370 19388
Homo sapiens chromosome 1 NC_000001.11: 11783698-... (18428)	ACTTCAGACTT---TTCACAGCGCTTGCCTG---TTTATTATCTCAGGTGAGTTAAGACATCATGATTGAGAGT
SARS-CoV-2 Reference Genome NC_045512.2 (18485)	TCATACCACTTATGTACAAAGGACTTCCTTGGAATGTAGTGCGTATAAAGATTGTACAAATGTTAAGTGACACA

Section 263

(19389)	19389 19400 19410 19420 19430 19440 19450 19462
Homo sapiens chromosome 1 NC_000001.11: 11783698-... (18496)	TAGGGGAATCCTAACTGTGGCAAGCACAGCCTCGCTGTAGGAACAGTTAGAAA-GAGG-GTCAGCAGACTCACT
SARS-CoV-2 Reference Genome NC_045512.2 (18559)	CTTAAAAATCT---CTCTGACAGAGTCGTATTTGTCTTATGGGCACATGGCTTTGAGTTGACATCTATGAAGTA

Section 264

(19463)	19463 19470 19480 19490 19500 19510 19520 19536
Homo sapiens chromosome 1 NC_000001.11: 11783698-... (18568)	TTCTTTCATGCCACCCCCCAAGC---CCTGTGCAGCAGCTTACAGTTATGC-AGAC-CAAGGCCAAG--TTCCG
SARS-CoV-2 Reference Genome NC_045512.2 (18630)	TTTTGTGAAAATAGGACCTGAGCGCACCTGTTGTCTATGTGATAGACGTGCCACATGCTTTTCCACTGCTTCAG

Section 265

(19537)	19537 19550 19560 19570 19580 19590 19600 19610
Homo sapiens chromosome 1 NC_000001.11: 11783698-... (18635)	AAACGAGACTCAAAAAAAAGCCTGGATGTGCA--CGAGTACTTGTGTTA-CAAGCTATCAACGGTGTATTTAAA
SARS-CoV-2 Reference Genome NC_045512.2 (18704)	ACACTTATGCCTGTTGGCATCATTCTATTGGATTTGATTACGTCTATAATCCGTTTATGATTGATGTTCAACAA

Section 266

(19611)	19611 19620 19630 19640 19650 19660 19670 19684
Homo sapiens chromosome 1 NC_000001.11: 11783698-... (18706)	CATCCTTGTGCATTTAAACTTCAAA--AACTTGGGAGTGACACGTGAGACGTGCATACAGTAAAAGGCATAAAC
SARS-CoV-2 Reference Genome NC_045512.2 (18778)	TGGGGTTTTACAGGTAACCTACAAAGCAACCATGATCTGTATTGTCAA--GTCCATG--GTAATGCACATGTAG

Section 267

	(19685)	19685 19690 19700 19710 19720 19730 19740 19758
Homo sapiens chromosome 1 NC_000001.11: 11783698-...	(18778)	TTAAAAAAAAACTGCAATTCATGA--AGTGCTAATGTAATTCAGTTGGCATTTCT--AGAGT-TCAAGAAGCA
SARS-CoV-2 Reference Genome NC_045512.2	(18848)	CTAGTTGTGA---TGCAAT-CATGACTAGGTGTCTAGCTGTCCACGAGTGCTTTGTTAAGCGTGTTGACTGGAC

Section 268

	(19759)	19759 19770 19780 19790 19800 19810 19820 19832
Homo sapiens chromosome 1 NC_000001.11: 11783698-...	(18847)	TAATCCAAATT-TCTAACT--TGCTGAAAACAATCTCACTGC--TTTGT-GACTTCCTAAACTGCACTCGTGAT
SARS-CoV-2 Reference Genome NC_045512.2	(18918)	TATTGAATATCCTATAATTGGTGATGAACTGAAGATTAATGCGGCTTGTAGAAAGGTTCAACA-CATGGTTGTT

Section 269

	(19833)	19833 19840 19850 19860 19870 19880 19890 19906
Homo sapiens chromosome 1 NC_000001.11: 11783698-...	(18915)	CCAGCCCCCAAGTTCCCTCAAAGAATACAGTGTGAGAATCAACATTTTTAACCCAT------TGAAGAGTGTGT
SARS-CoV-2 Reference Genome NC_045512.2	(18991)	AAAGCTGCATTATTAGCAGACA-AATTCCCAGTTCTTCACGACATTGGTAACCCTAAAGCTATTAAGTGTGTAC

Section 270

	(19907)	19907 19920 19930 19940 19950 19960 19970 19980
Homo sapiens chromosome 1 NC_000001.11: 11783698-...	(18983)	CTAAAATACCTTGACACTGTGGAGGGACTTTTACAGGCAACAGAGAAGTTGCAGATCAGATGACCCACTCTGCC
SARS-CoV-2 Reference Genome NC_045512.2	(19064)	CTCAAGCTGATGTAGAATG--GAAGTTCTATGATGCACAGCCTTGTAGTGACAAAGCTTATAAAATAGAAGAAT

Section 271

	(19981)	19981 19990 20000 20010 20020 20030 20040 20054
Homo sapiens chromosome 1 NC_000001.11: 11783698-...	(19057)	TTCTCCTTCCTGC--CCCCTCCGGCTGCTTTTTTCTGCCGGGTTAAAGGGCAGCCCAGCCTCAGGGCTGACAGT
SARS-CoV-2 Reference Genome NC_045512.2	(19136)	TATTCTATTCTTATGCCACACATTCTGACAAATTCACAGATGGTGTATGCCTATTTTGGAATTGCAATGTC-GA

Section 272

	(20055)	20055 20060 20070 20080 20090 20100 20110 20128
Homo sapiens chromosome 1 NC_000001.11: 11783698-...	(19129)	TTGCTCCCCAGGCACCACCACTCCCGCCTCCCACCTTAAC-CTTGCATG--AGTTTACCTTGAGATGAGATTGA
SARS-CoV-2 Reference Genome NC_045512.2	(19209)	TAGATATCCTGCTAATTCCATT---GTTTGTAGATTTGACACTAGAGTGCTATCTAACCTTAACTTGCC--TGG

Section 273

	(20129)	20129 20140 20150 20160 20170 20180 20190 20202
Homo sapiens chromosome 1 NC_000001.11: 11783698-...	(19200)	CAGCTCCCTCAGCAGTTCG------AGGAGGGAAGAATTCCAGGGAGAACCACTTGTCACCAGATTCCAATCGC
SARS-CoV-2 Reference Genome NC_045512.2	(19278)	TTGTGATGGTGGCAGTTTGTATGTAAATAAACATGCATTCCAC--ACACCAGCTTTTGATAAAGTGCTTTTGT

Section 274

(20203) 20203 20210 20220 20230 20240 20250 20260 20276

Homo sapiens chromosome 1 NC_000001.11: 11783698-... (19268) CGCCTCATCTTCTCCCGGAGTCTCTCATGCCGCTCGGGGTCCAGGCCCGGGG--TGGAA--CATCTCGAACTAT

SARS-CoV-2 Reference Genome NC_045512.2 (19350) TAATTTAAAACAATTACCATTTTTCTATTACTCTGACAGTCCATGTGAGTCTCATGGAAAACAAGTAGTGTCAG

Section 275

(20277) 20277 20290 20300 20310 20320 20330 20340 20350

Homo sapiens chromosome 1 NC_000001.11: 11783698-... (19338) CTTTGGAGCTCTCACTGCCACTG-CTGGCAC-TGCCCTCCAAGCAGGGGTTGAGGCTGCTGTTTCCTCT-GGCT

SARS-CoV-2 Reference Genome NC_045512.2 (19424) ATATAGATTATGTACCACTAAAGTCTGCTACGTGTATAACACGTTGCAATTTAGG-TGGTGCTGTCTGTAGACA

Section 276

(20351) 20351 20360 20370 20380 20390 20400 20410 20424

Homo sapiens chromosome 1 NC_000001.11: 11783698-... (19409) TCGT-----TCACCATGGCTGGGTTCCTACTGC-----ACAGGGTGGGG-CAGGGAGCATCAGGGGGCAGGCAG

SARS-CoV-2 Reference Genome NC_045512.2 (19497) TCATGCTAATGAGTACAGATTGTATCTCGATGCTTATAACATGATGATCTCAGCTGGCTTTAGCTTGTGGGTTT

Section 277

(20425) 20425 20430 20440 20450 20460 20470 20480 20498

Homo sapiens chromosome 1 NC_000001.11: 11783698-... (19472) CCACACCCCC-GACACATCAAGACACCTGAGTGGCAGGTTCAAGCCGGAGGCGCTGTATTTCCACACAGGAAGA

SARS-CoV-2 Reference Genome NC_045512.2 (19571) ACAAACAATTTGATACTTATA-ACCTCTGGAACACTTTTACAAGACTTCAGAGTTTAGAAAATGTGGCTTTTAA

Section 278

(20499) 20499 20510 20520 20530 20540 20550 20560 20572

Homo sapiens chromosome 1 NC_000001.11: 11783698-... (19545) AGGCCAAAAAAGGTGACACTGCCCCCTCCCAGTGGCTCCATGCTCCTCAGCTATGGCTGTCCGGGCCGCCTCAC

SARS-CoV-2 Reference Genome NC_045512.2 (19644) TGTTGTAAATAAGGGACACTTTGATGGACAACAGGGTGAAGTACCAGTTTCTATCATTAATAACACTGTTT-AC

Section 279

(20573) 20573 20580 20590 20600 20610 20620 20630 20646

Homo sapiens chromosome 1 NC_000001.11: 11783698-... (19619) TCAAAGCCTTGCCCTCCGCTGCTGCCAGGCTCCTTGCATGCAAGGCAGCCCCCACCCGGCACTGCAACAT--GC

SARS-CoV-2 Reference Genome NC_045512.2 (19717) ACAAAAGTTGATGGT--GTTGATGTAGAATTGTTTGAAAATAAAACAACATT-ACCTGTTAATGTAGCATTTGA

Section 280

(20647) 20647 20660 20670 20680 20690 20700 20710 20720

Homo sapiens chromosome 1 NC_000001.11: 11783698-... (19691) TCCTCGGTGACAGCTACCTGATGGAAGGTGGCACAGAGGACCTCACGCTGCCGACCTGCGAGGCCCATATTCCT

SARS-CoV-2 Reference Genome NC_045512.2 (19788) GCTTTGGGCTAAGCG-CAACATTAAACCAGTACCAGAGGTGAAAATACTCAATAATTTGGGTGTGGACATTGCT

Section 281

(20721) 20721 20730 20740 20750 20760 20770 20780 20794
Homo sapiens chromosome 1 NC_000001.11: 11783698-... (19765) AGCGAGGGGCAGTAGTGCCCAGCTGGGAGGACCCTGGCTTTTCGATGGTCCATCTCTGTCAGCTCAGGCCCAGA
SARS-CoV-2 Reference Genome NC_045512.2 (19861) -------GCTAATACTGTG-ATCTGGGACTACAAAAGAGATGCTCCAGCACATATATCTACTATTGGTGTTTGT

Section 282

(20795) 20795 20800 20810 20820 20830 20840 20850 20868
Homo sapiens chromosome 1 NC_000001.11: 11783698-... (19839) GTGCGTGGGGAGGGAACAGAGGCACCTTCCCCAGCGGGTGTGTTCAACTGGGCGGAGCTGCGACAAAGGAAGAG
SARS-CoV-2 Reference Genome NC_045512.2 (19927) TCTATGACTGACATAGCCAAGAAACCAACTGAAACGATT-TGTGCACCACTCACTGTCTTTTTTGATGGTAGAG

Section 283

(20869) 20869 20880 20890 20900 20910 20920 20930 20942
Homo sapiens chromosome 1 NC_000001.11: 11783698-... (19913) GCTTGA-GCTCCAGCCAGCACAAGGGG----GCGGGGGCAAGGCTTCC-AGGCGAGGAGGGAGGGCTGAGGCTC
SARS-CoV-2 Reference Genome NC_045512.2 (20000) --TTGATGGTCAAGTAGACTTATTTAGAAATGCCCGTAATGGTGTTCTTATTACAGAAGGTAGTGTTAAAGGTT

Section 284

(20943) 20943 20950 20960 20970 20980 20990 21000 21016
Homo sapiens chromosome 1 NC_000001.11: 11783698-... (19981) TAGTAAGGGGAGGCGGATGGAAGGA-GGGGGACTCAGGGGCAGAGACTGCAGGGGTTGGTGGAGATGAGGCCAA
SARS-CoV-2 Reference Genome NC_045512.2 (20072) TACAACCATCTGTAGGTCCCAAACAAGCTAGTCTTAATGG-AGTCACATTAAT------TGGAGAAGCCGTAAA

Section 285

(21017) 21017 21030 21040 21050 21060 21070 21080 21090
Homo sapiens chromosome 1 NC_000001.11: 11783698-... (20054) CAGGC-GTTCAGGGATACCAGATCACCCTCCAGAGAAGG--AACAGCTCACTCTGGCTGGCTCTGCCTGGAACA
SARS-CoV-2 Reference Genome NC_045512.2 (20139) AACACAGTTCAATTATTATAAGAAAGTTGATGGTGTTGTCCAACAATTACCTGAAACTTACTTTACTCAGAGTA

Section 286

(21091) 21091 21100 21110 21120 21130 21140 21150 21164
Homo sapiens chromosome 1 NC_000001.11: 11783698-... (20125) TCTGTTACTTGAAACAGTTCGTGCACAGGATGGAAGGGGTGAGGGCTTGACTATGCAGCCAGATTTCTCCTGAA
SARS-CoV-2 Reference Genome NC_045512.2 (20213) ---GAAATTTACAAGAATTTAAACCCAGGAGTCAAATGGAAATTGATTTCTTAG--AATTAGCTATGGA-TGAA

Section 287

(21165) 21165 21170 21180 21190 21200 21210 21220 21238
Homo sapiens chromosome 1 NC_000001.11: 11783698-... (20199) TTCATGCTACTCCA-AAAAGACCGTGTTGTGGGTTAGAACTTGATGCTGGCCATGTTCCAAAAATAGCCAGACC
SARS-CoV-2 Reference Genome NC_045512.2 (20281) TTCATTGAACGGTATAAATTAGAAGGCTATGCCTTCGAACATATCGTT-----TATGGAGATTTTAGTCATAG-

Section 288

(21239) 21239 21250 21260 21270 21280 21290 21300 21312
Homo sapiens chromosome 1 NC 000001.11: 11783698-... (20272) CAAGTTCAGTAAGGGCACGAATTTCTCAGAACAAAAGCTGCCCCATCTGTGCTTGCCTTGCAGGCTGCTCTTCT
SARS-CoV-2 Reference Genome NC_045512.2 (20349) -----TCAGTTAGGTGGTTTACATCT----ACTGAT--TGGACTAGCTAAACGT---TTTAAGGAATCACCTTT

Section 289

(21313) 21313 21320 21330 21340 21350 21360 21370 21386
Homo sapiens chromosome 1 NC 000001.11: 11783698-... (20346) TACACATCACAGAATTCAATTCAAAT---CATCACAGTGACCCAGCTACTGGGAAG-GCTGAGGCAGAAGGATT
SARS-CoV-2 Reference Genome NC_045512.2 (20409) TGAA--TTAGAAGATTTTATTCCTATGGACAGTACAGTTAAAAACTATTTCATAACAGATGCGCAAACAGGTTC

Section 290

(21387) 21387 21400 21410 21420 21430 21440 21450 21460
Homo sapiens chromosome 1 NC 000001.11: 11783698-... (20416) GCCTGAGCCCAGGAGTTCAAGGCCAGCCTGGGCAACATAGCGAGACTCCATCTCTTAAAAAAACAAAAAACACA
SARS-CoV-2 Reference Genome NC_045512.2 (20481) ATCTAAGTGTGTGTGTTCTGTTATTGATTT------ATTACTTGA-TGATTTTGTTGAAATAATAAAATCCCAA

Section 291

(21461) 21461 21470 21480 21490 21500 21510 21520 21534
Homo sapiens chromosome 1 NC 000001.11: 11783698-... (20490) CCTGTAATCCCAGCACTTTGGGAGGCCGAGGTGGGTGGATCACCTGAGGCCAGGAGTTTGAGACCAGCCTGGC-
SARS-CoV-2 Reference Genome NC_045512.2 (20548) GATTTATCTGTAGTTTCTAAGGTTGTCAAAGTGACTAT-TGACTATA---CAGAAATTTCATTTATGCTTTGGT

Section 292

(21535) 21535 21540 21550 21560 21570 21580 21590 21608
Homo sapiens chromosome 1 NC 000001.11: 11783698-... (20563) --CAACATGGTGAAATCCCGAC--TCTACTAAAAATATAAAAATTAGCCAGGTTTGGTGGCGGGCGCCTGTAAT
SARS-CoV-2 Reference Genome NC_045512.2 (20618) GTAAAGATGGCCATGTAGAAACATTTTACCCAAAAT-TACAATCTAGTCAAGCGTGGCAACCGGGTGTTGCTAT

Section 293

(21609) 21609 21620 21630 21640 21650 21660 21670 21682
Homo sapiens chromosome 1 NC 000001.11: 11783698-... (20633) CCCAGCTACTCTGGAGGCTGAGGCAGGAGAATCGCTTGAACCCGGAAGGCAGAGGTTGCAGTGAACCAAGATTG
SARS-CoV-2 Reference Genome NC_045512.2 (20691) GCC---TAATCTTTACAAA-ATGCAA-AGAAT-GCTATTAGAA--AAGTGTGACCTTCAAAATTATGGTGATAG

Section 294

(21683) 21683 21690 21700 21710 21720 21730 21740 21756
Homo sapiens chromosome 1 NC 000001.11: 11783698-... (20707) TGCCATAGCACTCCAGCCTGGGCGACAAAAGCAAAACTCCATCTCAAAAAAAAAAACAAAAACAAAAAAACAGAA
SARS-CoV-2 Reference Genome NC_045512.2 (20757) TGCAACATTACCTAAA-------GGCATAATGATGAATGTCGCAAAATATACTCAACTGTGTCAATATTTAAAC

```
                                                              Section 295
                                                       (21757) 21757        21770      21780      21790      21800      21810      21820  21830
Homo sapiens chromosome 1 NC_000001.11: 11783698-... (20781) ACAAGTA-ATCAGAGCCACCAGGGCACA-GACAGGCCAACAGAGGGCATAGGGTCATCTGGCAGTCTGGTTAAG
       SARS-CoV-2 Reference Genome NC_045512.2 (20824) ACATTAACATTAGCTGTACCCTATAATATGAGAGTTATACAT------TTTGGTG--CTGGT--TCTGATAAAG
                                                              Section 296
                                                       (21831) 21831    21840      21850      21860      21870      21880      21890      21904
Homo sapiens chromosome 1 NC_000001.11: 11783698-... (20853) AAAATAAGACAAACACTTAAAAACAAACAAAACACTTGCACAGCAATGGGCAAACACACAGTAGTGAATGGGTG
       SARS-CoV-2 Reference Genome NC_045512.2 (20888) GAGTTGCACCAGGTACAGCTGTTTTAAGACAGTGGTTGC----CTACGGGTACGCTGCTTGTCG--ATTCAGAT
                                                              Section 297
                                                       (21905) 21905 21910      21920      21930      21940      21950      21960      21978
Homo sapiens chromosome 1 NC_000001.11: 11783698-... (20927) TGTGGTGACATGGCACAACAGAGGAAAGCCCAGGTGAGGTTCATTTCTTTCAGACTGTCC--ACCACTAACCCT
       SARS-CoV-2 Reference Genome NC_045512.2 (20956) CTTAATGACTTTGTCT--CTGATGCAGATTCAACTTTGATTGGTGATTGTGCAACTGTACATACAGCTAATAAA
                                                              Section 298
                                                       (21979) 21979      21990      22000      22010      22020      22030      22040  22052
Homo sapiens chromosome 1 NC_000001.11: 11783698-... (20999) GTGTACCTAACCATTTGGGGTCT-TA-GACTCTT---TTGACAATCTGATTAAATGCACTG-CTCCCTTCCCCT
       SARS-CoV-2 Reference Genome NC_045512.2 (21028) TGGGATCTCATTATTAGTGATATGTACGACCCTAAGACTAAAAATGTTACAAAAGAAAATGACTCTAAAGAGGG
                                                              Section 299
                                                       (22053) 22053  22060      22070      22080      22090      22100      22110      22126
Homo sapiens chromosome 1 NC_000001.11: 11783698-... (21067) GAAATACACACACAGGTGCACAGACTACACGGGGATACATCCAAAGACAAGGAGTTGATTCCAGACTAAGAACC
       SARS-CoV-2 Reference Genome NC_045512.2 (21102) TTTTTTCACTTACATTTGTGG-GTTTATACA---ACAAAAGCTAGCTCTTGGAGGT--TCCGTGGCTATAAAGA
                                                              Section 300
                                                       (22127) 22127        22140      22150      22160      22170      22180      22190  22200
Homo sapiens chromosome 1 NC_000001.11: 11783698-... (21141) CTGGGCCAAAGCTTTCTCCCTCTAGGGCTGCTCTCTTTCATTCTGACCTCAAACTCCAGATTCTCAGTGGGGAT
       SARS-CoV-2 Reference Genome NC_045512.2 (21170) TAA---CAGAACATTCT---TGGAATGCTGATCT------TTATAAGCTCATGGGACAC-TTCGCATGGTGGAC
                                                              Section 301
                                                       (22201) 22201    22210      22220      22230      22240      22250      22260      22274
Homo sapiens chromosome 1 NC_000001.11: 11783698-... (21215) GGCTGTGGGGAGTGAGGAACGGGGGCGGGGGGGAGGAGTAACAGCAAGAGCAGCCAGTTGGCTTTCCTCCAGCG
       SARS-CoV-2 Reference Genome NC_045512.2 (21231) AGCCTTTGTTACTAATGTGAATGCGTCATCATCTGAAGCATTTTTAATTGGATGTAATTATCTTGGCAA-ACCA
```

Section 302

```
                                                        (22275) 22275 22280          22290          22300          22310          22320          22330          22348
Homo sapiens chromosome 1 NC_000001.11: 11783698-... (21289) TTTAAGGGCGCACCTCGTTAATTGCTTGCTGGAA----CAGAACCTAGAGTAGGAGGAAGCTGAACCACCTGTA
SARS-CoV-2 Reference Genome NC_045512.2 (21304) CGCGAACAAATAGATGGTTATGTCATGCATGCAAATTACATATTTTGGAGGAATACAAATCCAATTCAGTTGTC
```

Section 303

```
                                                        (22349) 22349 22360 22370 22380 22390 22400 22410 22422
Homo sapiens chromosome 1 NC_000001.11: 11783698-... (21359) AACCC--TCTG-ATTTTCAAAGACTTATTGCATTCCTAGTATATGCCACGCATGTGCCATATTTGTAAGCCAAC
SARS-CoV-2 Reference Genome NC_045512.2 (21378) TTCCTATTCTTTATTTGACATGAGTAAATTTCCCCTTAAATTAAGGGGTACTGCTGTTATGTCTTTAAAAGAAG
```

Section 304

```
                                                        (22423) 22423 22430 22440 22450 22460 22470 22480 22496
Homo sapiens chromosome 1 NC_000001.11: 11783698-... (21430) -TCCCACGATTTATCCGTTATCAGCTGGACTCCCATAGCACCCTGGGTTGTAACTGTGGGTGAAAAGGCCTGTC
SARS-CoV-2 Reference Genome NC_045512.2 (21452) GTCAAATCAATGATATGATTTTATCTCTTCTTAGTAA--AGGTAGACTTATAATTAGAGGAAAACAACAGAGTTG
```

Section 305

```
                                                        (22497) 22497 22510 22520 22530 22540 22550 22560 22570
Homo sapiens chromosome 1 NC_000001.11: 11783698-... (21503) TCCTTTCTCTTT---CCCCAGTTCCCTGAGACAGGACCGGTGTC----CTGCCTGGGGTATCCCCAGAGTTTGG
SARS-CoV-2 Reference Genome NC_045512.2 (21524) TTATTTCTAGTGATGTTCTTGTTAACAACTAAACGAACAATGTTTGTTTTTCTTGTTTTATTGCCACTAGTCTC
```

Section 306

```
                                                        (22571) 22571 22580 22590 22600 22610 22620 22630 22644
Homo sapiens chromosome 1 NC_000001.11: 11783698-... (21570) CACACGGTGATAGCCAACATTCACTGAGCGCCAAAGGGCCAGGTGCTGCCACT----CTCTCAAAATAAGCCTC
SARS-CoV-2 Reference Genome NC_045512.2 (21598) TAGTCAGTG-TGTTAATCTTACAACCAGAACTCAATTACCCCCTGCATACACTAATTCTTTCACACGTGGTGTT
```

Section 307

```
                                                        (22645) 22645 22650 22660 22670 22680 22690 22700 22718
Homo sapiens chromosome 1 NC_000001.11: 11783698-... (21640) TGCCACTTACTGAACAACTAGTCTGCGCCAAGCACTGGGATACTAAGCCTACGACTC--CCAGAAAGGTC----
SARS-CoV-2 Reference Genome NC_045512.2 (21671) TATTACC--CTGACAAAGTTTTCAGATCCT--CAGTTTTACATTCAACTCAGGACTTGTTCTTACCTTTCTTTT
```

Section 308

```
                                                        (22719) 22719 22730 22740 22750 22760 22770 22780 22792
Homo sapiens chromosome 1 NC_000001.11: 11783698-... (21708) CCGGCGGACCCCCGTGCGCAGCGGGACG---CAGTGGGCGCCAGGGACCGCAGTGCC-------CCGGGCCCAG
SARS-CoV-2 Reference Genome NC_045512.2 (21741) CCAATGTTACTTGGTTCCATGCTATACATGTCTCTGGGACCAATGGTACTAAGAGGTTTGATAACCCTGTCCTA
```

```
                                                           Section 309
                                                 (22793) 22793 22800 22810 22820 22830 22840 22850 22866
Homo sapiens chromosome 1 NC_000001.11: 11783698-... (21772) CGCTCTTCTCCAGGTGATCG----CCGGGGAGGCAGGTTTAAAAGGCAGGAGCCGGAAGCCGTCTCGGCGCCCA
SARS-CoV-2 Reference Genome NC_045512.2 (21815) CCATTTAATGATGGTGTTTATTTTGCTTCCACTGAGAAGTCTAACATAATAAGAGGCTGGATTTTTGGTACTAC
                                                           Section 310
                                                 (22867) 22867 22880 22890 22900 22910 22920 22930 22940
Homo sapiens chromosome 1 NC_000001.11: 11783698-... (21842) GGTGGC--CGAGAATCCAGTCACCAG-----GTCACTGAGTCACCGATGGGGGCGAGGACACGGGCCTGGGCCG
SARS-CoV-2 Reference Genome NC_045512.2 (21889) TTTAGATTCGAAGACCCAGTCCCTACTTATTGTTAATAACGCTACTAATGTTGTTATTAAA---GTCTGTGAAT
                                                           Section 311
                                                 (22941) 22941 22950 22960 22970 22980 22990 23000 23014
Homo sapiens chromosome 1 NC_000001.11: 11783698-... (21909) GTCAGAGGGGCGGGATCGTCACCCTGGCTCAGGGGCCCCTCCGTCCAGGCAGGGAGCCAAAGTCAGTCTTCGC
SARS-CoV-2 Reference Genome NC_045512.2 (21960) TTCAATTTTG-----TAATGATCCATTTTT---GGGTGTTTATTACCACAAAAACAACAAAAGTTGGA--TGGA
                                                           Section 312
                                                 (23015) 23015 23020 23030 23040 23050 23060 23070 23088
Homo sapiens chromosome 1 NC_000001.11: 11783698-... (21983) TTGAGGGTTGGCGGTCGCTGGAAGTGGTAGCCATTGGAGTTACACTAATCCCGCGAAGGGTGCGCA-AGGGAG
SARS-CoV-2 Reference Genome NC_045512.2 (22024) AAGTGAGTTCAGAGTTTATTCTAGTGCGAATAATTGCACTTTTGAATA-TGTCTCTCAGCCTTTTCTTATGGA-
                                                           Section 313
                                                 (23089) 23089 23100 23110 23120 23130 23140 23150 23162
Homo sapiens chromosome 1 NC_000001.11: 11783698-... (22056) GCGGCAGCCCCCCCCAAGAAGAGAGGCAGGCCGGCCTCCAGCGCTCCCCGCCCACAAGCACGTCCTTGCCCCCG
SARS-CoV-2 Reference Genome NC_045512.2 (22096) -------------CCTTGAAGGAAAACAGGGTAATTTCAAAAA-TCTTAGGGAATTTGT--GTTTAAGAATATT
                                                           Section 314
                                                 (23163) 23163 23170 23180 23190 23200 23210 23220 23236
Homo sapiens chromosome 1 NC_000001.11: 11783698-... (22130) GGAGGTTGTTTGCCGGCTCCAGGATCTCC-CTCCCGGCGACCCCGGCCTCGCCGTCACTCACCCTCCAGTCCCG
SARS-CoV-2 Reference Genome NC_045512.2 (22154) GATGGTTATTTTAAAAT---ATATTCTAAGCACACGCCTATTAATTTAGTGC-GTGA-TCTCCCTCAGGGTTTT
                                                           Section 315
                                                 (23237) 23237 23250 23260 23270 23280 23290 23300 23310
Homo sapiens chromosome 1 NC_000001.11: 11783698-... (22203) GGTGACTCAGAACGCTCGGCCGCCGGCCGCACCACTGTGGCCGCTGCAGGGCGGGGAACGCAGTGAAGGGGCG
SARS-CoV-2 Reference Genome NC_045512.2 (22223) TCGGCTTTAGAACCATTGGTAGATTTGCCAATAGGTATTAACATCACTAGGTTTCAAACTTTACTT--GCTTTA
```

```
                                                          Section 316
                                                    (23311) 23311         23320          23330          23340          23350          23360          23370            23384
Homo sapiens chromosome 1 NC_000001.11: 11783698-... (22277) CGGCGCAGGTGTCAGGTTGCTGGAGAGGCGGGCCTACAGGGCGGAAACCACCGGC-GCGGGTGACCGGAAGCCC
SARS-CoV-2 Reference Genome NC_045512.2 (22295) CATAGAAGTTATTTGACTCCTGGTGATTCTT--CTTCAGGTTGGACAGCTGGTGCTGCAGCTTATTATGTGGGT

                                                          Section 317
                                                    (23385) 23385 23390          23400          23410          23420          23430          23440                 23458
Homo sapiens chromosome 1 NC_000001.11: 11783698-... (22350) CGCCCCCAACAT-GGCCGTGCCCACAGGTCTCGT-TGGAGGCCGTGCCTTTATCG-TCATGTGACGCGAGTCCT
SARS-CoV-2 Reference Genome NC_045512.2 (22367) TATCTTCAACCTAGGACTTTTCTATTAAAATATAATGAAAATGGAACCATTACAGATGCTGTAGACTGTGCACT

                                                          Section 318
                                                    (23459) 23459           23470          23480          23490          23500          23510          23520       23532
Homo sapiens chromosome 1 NC_000001.11: 11783698-... (22421) CGCCCCACCCGTCTGCAGCCACTCCTGGTCTCAGTCCCAGAGCTCGAGAGGGCCACGTGACCGTCCCGGGGCCA
SARS-CoV-2 Reference Genome NC_045512.2 (22441) TGACCCTCTC-TCAGAAACAAAGTGTACGTTGAAATCCTTCACTGTAGAAA---AAGGAATCTATCAAACTTCT

                                                          Section 319
                                                    (23533) 23533   23540          23550          23560          23570          23580          23590              23606
Homo sapiens chromosome 1 NC_000001.11: 11783698-... (22495) GTCACGTGAGGCGCAGATCCTGGCTGGGAGGGG-GTTGGTAGAGGGGTCCAGAGTG--GCAGTAAAGGAGGAAG
SARS-CoV-2 Reference Genome NC_045512.2 (22511) AACTTTAGAGTCCAACCAACAGAATCTATTGTTAGATTTCCTAATATTACAAACTTGTGCCCTTTTGGTGAAGT

                                                          Section 320
                                                    (23607) 23607               23620          23630          23640          23650          23660          23670 23680
Homo sapiens chromosome 1 NC_000001.11: 11783698-... (22566) ATGGCGGGGTGCAGGGGGTCTCTGTGCTGCTGCT----GCAGGTGGTGCTGCTGCTGCGGTGAGCGTGAGACCC
SARS-CoV-2 Reference Genome NC_045512.2 (22585) TTTTAACGCCACCAGATTTGCATCTGTTTATGCTTGGAACAGGAAGAGAATCAGCAACTGTGTTGCTGATTATT

                                                          Section 321
                                                    (23681) 23681         23690          23700          23710          23720          23730          23740            23754
Homo sapiens chromosome 1 NC_000001.11: 11783698-... (22636) GCACCCCCGAGGAGCTGGTAAGAAGCCGGCGGAGTCGGCCTGGGGAGGGGCGGTTGCTAAGGGAC-TGAGAGA
SARS-CoV-2 Reference Genome NC_045512.2 (22659) CTGTCCTATATAATTCCGCATCATTTTCCACTTTTAAGTGTTATGGAGTGTCTCCTACTAAATTAAATGATCTC

                                                          Section 322
                                                    (23755) 23755 23760          23770          23780          23790          23800          23810                 23828
Homo sapiens chromosome 1 NC_000001.11: 11783698-... (22709) GGCGTTGCCGGGGGTGGGGCCGGGCCAATC-----TGGGCCCGCAGGTGGCAG-CGGGTGGGGCCTGGGG--AC
SARS-CoV-2 Reference Genome NC_045512.2 (22733) TGCTTTACTAATGTCTATGCAGATTCATTTGTAATTAGAGGTGATGAAGTCAGACAAATCGCTCCAGGGCAAAC
```

```
Section 323
                                                        (23829) 23829      23840      23850      23860      23870      23880      23890      23902
Homo sapiens chromosome 1 NC_000001.11: 11783698-... (22775) CGCAGCCAGGGCTCTCGAAGCGTCTACCCTGCTTCACGTGCCTA-AGTCTGGGGTCTTTTCTCATAACCCCTC-
     SARS-CoV-2 Reference Genome NC_045512.2 (22807) TGGAAAGATTGCTGATTATAATTATAAATTACCAGATGATTTTACAGGCTGCGTTATAGCTTGGAATTCTAACA

Section 324
                                                        (23903) 23903  23910      23920      23930      23940      23950      23960          23976
Homo sapiens chromosome 1 NC_000001.11: 11783698-... (22847) --CTGGAATCTGAGGGAGAATCCAGGCCAGCTCCAGTAAATCCCACCCATGTTTCCTTTTATTTCCATCTGAAA
     SARS-CoV-2 Reference Genome NC_045512.2 (22881) ATCTTGATTCTAAGGTTGGT----GGTAATTATAATTACCTGTATAGATTGTTTAGGA--AGTCTAATCTCAAA

Section 325
                                                        (23977) 23977        23990      24000      24010      24020      24030      24040  24050
Homo sapiens chromosome 1 NC_000001.11: 11783698-... (22919) AGAGTTTGGAATGAAACAAGCGTTTTCAGAATGAGCTTCCTCACAGGACT-TTGTGCTTATCATGCTGCTGTTT
     SARS-CoV-2 Reference Genome NC_045512.2 (22949) CC--TTTTGAGAGAGATA-----TTTCA-ACTGAAAT--CTATCAGGCCGGTAGCACACCTTGTAATGGTGTTG

Section 326
                                                        (24051) 24051    24060      24070      24080      24090      24100      24110      24124
Homo sapiens chromosome 1 NC_000001.11: 11783698-... (22992) TAGCTTCTCCAGTCCTTTCTCACTCTATCCCTCACCC--TTTCTGGGTCACTGACAGTCCAGCTTCCCC-CTGC
     SARS-CoV-2 Reference Genome NC_045512.2 (23013) AAGGTTTT--AATTGTTACTTTCCTTTACAATCATATGGTTTCCAACCCACTAATGGTGTTGGTTACCAACCAT

Section 327
                                                        (24125) 24125 24130      24140      24150      24160      24170      24180          24198
Homo sapiens chromosome 1 NC_000001.11: 11783698-... (23063) AAAGCATCCTGGGAAAA-CTTGAGTTGTGTTCCCAGAGTTGACAAGTCCTTTCTGATTTCAGA--T--ACGTCA
     SARS-CoV-2 Reference Genome NC_045512.2 (23085) ACAGAGTAGTAGTACTTTCTTTTGAACTTCTACATGCACCAGCAACTGTTTGTGGACCTAAAAAGTCTACTAAT

Section 328
                                                        (24199) 24199      24210      24220      24230      24240      24250      24260      24272
Homo sapiens chromosome 1 NC_000001.11: 11783698-... (23132) CACTGTCAAATGGAACCTG-CATTTTTACCCCCCTT--TTCAGCTGGCGTTGGACATCTTTGAG-GTGTCCTGA
     SARS-CoV-2 Reference Genome NC_045512.2 (23159) TTGGTTAAAAACAAATGTGTCAATTTCAACTTCAATGGTTTAACAGGCACAGGTGTTCTTACTGAGTCTAACAA

Section 329
                                                        (24273) 24273  24280      24290      24300      24310      24320      24330          24346
Homo sapiens chromosome 1 NC_000001.11: 11783698-... (23202) AGAGGAGGGTGGTTATTTGGAGACTATCAGTGGAAATGAAGAGCTCAGCTGATGGCTGTA---GATCCTCACCC
     SARS-CoV-2 Reference Genome NC_045512.2 (23233) AAAGTTTCTGCCTTTCCAACAATTTGGCAGAGACATTGCTGA-CACTACTGATG-CTGTCCGTGATCCACAGAC
```

Section 330

(24347) 24347 24360 24370 24380 24390 24400 24410 24420
Homo sapiens chromosome 1 NC_000001.11: 11783698-... (23273) ACATGG--TCCCTCACCCCTGAA-AGGTTTTTTTTGAGGGCTGGTTCCTATTTGATAATGGCTCCAGATGATTT
SARS-CoV-2 Reference Genome NC_045512.2 (23305) ACTTGAGATTCTTGACATTACACCATGTTCTTTTGGTGGTGTCAGTGTTATAACACCAGGAACAAATACTTCTA

Section 331

(24421) 24421 24430 24440 24450 24460 24470 24480 24494
Homo sapiens chromosome 1 NC_000001.11: 11783698-... (23344) AGAAGCTAGCCACTGATT-TCAGTAAAT--ACTTC-------TGCCTCCTTCCACTCCTAACCACTAAAAAAGG
SARS-CoV-2 Reference Genome NC_045512.2 (23379) ACCAGGTTGCTGTTCTTTATCAGGATGTTAACTGCACAGAAGTCCCTGTTGCTATTCATGCAGATCAACTTACT

Section 332

(24495) 24495 24500 24510 24520 24530 24540 24550 24568
Homo sapiens chromosome 1 NC_000001.11: 11783698-... (23408) CTCCCTCTGCGGATCTGTTTTTCTAGA-CCATCCTTGGAGAAACACA-GGAGGAGGAGGATGAGATTCTTC-CA
SARS-CoV-2 Reference Genome NC_045512.2 (23453) CCTACTTGGCGTGTTTATTCTACAGGTTCTAATGTTTTTCAAACACGTGCAGGCTGTTTAATAGGGGCTGAACA

Section 333

(24569) 24569 24580 24590 24600 24610 24620 24630 24642
Homo sapiens chromosome 1 NC_000001.11: 11783698-... (23479) AGGAAAGAC----TATGAG-GTGAGCT--CCTTTGATACTGCT---TGGGCAACTAAAGT-AGTGAGTGGCCTG
SARS-CoV-2 Reference Genome NC_045512.2 (23527) TGTCAACAACTCATATGAGTGTGACATACCCATTGGTGCAGGTATATGCGCTAGTTATCAGACTCAGACTAATT

Section 334

(24643) 24643 24650 24660 24670 24680 24690 24700 24716
Homo sapiens chromosome 1 NC_000001.11: 11783698-... (23542) GTCA-CTTCAGGC-CGAAGCATTATGATTCCAACCAGAGATGCT----CTTTGACCTTCTGTCCCAGGGAGTGA
SARS-CoV-2 Reference Genome NC_045512.2 (23601) CTCCTCGGCGGGCACGTAGTGTAGCTAGTCAATCCATCATTGCCTACACTATGTCACTTGGTGCAGAAAATTCA

Section 335

(24717) 24717 24730 24740 24750 24760 24770 24780 24790
Homo sapiens chromosome 1 NC_000001.11: 11783698-... (23610) GTGGGTAAG----AGAACTTCCTT------CT-AGGAAAGAGACTGTT----T-AGCA-AGCCCTTTTCTCACC
SARS-CoV-2 Reference Genome NC_045512.2 (23675) GTTGCTTACTCTAATAACTCTATTGCCATACCCACAAATTTTACTATTAGTGTTACCACAGAAATTCTACCAGT

Section 336

(24791) 24791 24800 24810 24820 24830 24840 24850 24864
Homo sapiens chromosome 1 NC_000001.11: 11783698-... (23667) TT--AACCCCAAAACAAGGTCTGGTGTTTTTACACGTTTATAAATCTGTGGCACCTTAACCTGGATAGAGTA--
SARS-CoV-2 Reference Genome NC_045512.2 (23749) GTCTATGACCAAGACA---TCAGTAGATTGTACAA---TGTACATTTGTGGTGATTCAAC-TGAATGCAGCAAT

Section 337

(24865) 24865 24870 24880 24890 24900 24910 24920 24938

Homo sapiens chromosome 1 NC_000001.11: 11783698-... (23737) -TGTATTTGCCATTAAACTGTTTGCTTTTACAGCTTACAGAGAGACTTAGAGTGAAAGGCACTGGCAGGCAGTA

SARS-CoV-2 Reference Genome NC_045512.2 (23816) CTTTTGTTGCAATATGGCAGTTTTTGTACACAA-TTAAACCGTGCTTTAACTGGAATAGCTGTTGAACAAGACA

Section 338

(24939) 24939 24950 24960 24970 24980 24990 25000 25012

Homo sapiens chromosome 1 NC_000001.11: 11783698-... (23810) TGTGCCCCTAACAGGTGTGTGC-----TTATAAACTGTTAAGGAGGCCTTGCTTCTC----TTCTAGGAGAGCT

SARS-CoV-2 Reference Genome NC_045512.2 (23889) AAAACACCCAAGAAGTTTTTGCACAAGTCAAACAAATTTACAAAACACCACCAATTAAAGATTTTGGTGGTTTT

Section 339

(25013) 25013 25020 25030 25040 25050 25060 25070 25086

Homo sapiens chromosome 1 NC_000001.11: 11783698-... (23875) TGCTTTTCAGATAT-TTTCTTGCTCAAGCAGCTTCTCTCTGACATGTTCTTGATT-AAGTTCCACCTTTCATGT

SARS-CoV-2 Reference Genome NC_045512.2 (23963) AATTTTTCACAAATATTACCAGATCCATCAAAACCAAGCA-AGAGGTCATTTATTGAAGATCTACTTTTCAACA

Section 340

(25087) 25087 25100 25110 25120 25130 25140 25150 25160

Homo sapiens chromosome 1 NC_000001.11: 11783698-... (23947) ATGGATTCTTAGAGTATGTGTTTTTAGTAAAGCCAACACTGCTGGGGTGAAACAACATGCGTGACTTTTTATC

SARS-CoV-2 Reference Genome NC_045512.2 (24036) AAGTGACACTTGCAGATGCTGGCTTCATCAAACAATATGGTGATTGCCTTGGTGATATTGCTGCTAGAGACCTC

Section 341

(25161) 25161 25170 25180 25190 25200 25210 25220 25234

Homo sapiens chromosome 1 NC_000001.11: 11783698-... (24021) ATTAAATTCTTAAACGTAATAAAGAAGGTAGAGAAAAGCCTA-TAACTCCATTCATTAAAAGATGACTGTTAAC

SARS-CoV-2 Reference Genome NC_045512.2 (24110) ATTTG-TGCACAAAAGTT-TAACGGCCTTACTGTTTTGCCACCTTTGCTCACAGATGAAATGATTGCTCAATAC

Section 342

(25235) 25235 25240 25250 25260 25270 25280 25290 25308

Homo sapiens chromosome 1 NC_000001.11: 11783698-... (24094) ACCT-TACTAAGTATCTTTCTACATCCTTACCCATGCTTTTATATACATGATACATGCTTTTTTTAAGTAACAA

SARS-CoV-2 Reference Genome NC_045512.2 (24182) ACTTCTGCACTGTTAGCGGGTACAATC--ACTTCTGGTTGGACCTTTGGTGCAGGTGCTGCATTACAAATACCA

Section 343

(25309) 25309 25320 25330 25340 25350 25360 25370 25382

Homo sapiens chromosome 1 NC_000001.11: 11783698-... (24167) AAATGAGATTCCACTAAATCATCTATTTTAACCTGTCCCACTCACCCCCTTTATTATACTTTTTGTATCATTAG

SARS-CoV-2 Reference Genome NC_045512.2 (24254) TT-TGCTATGCAAATGGCTTATAGGTTTAATGGTATTGGAGTTACACAGAATGTTCT-CTATGAGAACCAAAAA

Section 344

	(25383)	25383 25390 25400 25410 25420 25430 25440 25456
Homo sapiens chromosome 1 NC_000001.11: 11783698-...	(24241)	GTAATTTTCCAC-AACCTTTTCTTTTTTTTGACAGAGTCTCACTCTTCCATAAATTCTTCATGCTGTAGAATTT
SARS-CoV-2 Reference Genome NC_045512.2	(24326)	TTGATTGCCAACCAATTTAATAGTGCTATTGGCAAAAT-TCAAGACTCACTTTCTTCCACAGCAAGTGCACTTG

Section 345

	(25457)	25457 25470 25480 25490 25500 25510 25520 25530
Homo sapiens chromosome 1 NC_000001.11: 11783698-...	(24314)	AGTGTCAGCGTTAGGATTTGTAGCACTTTTTTGTTTAA-CTTTTATTATGAAGGATTT-CAACT-ATATGCAAA
SARS-CoV-2 Reference Genome NC_045512.2	(24399)	---GAAAACTTCAAGATGTGGTCAACCAAAATGCACAAGCTTTAAACACGCTTGTTAAACAACTTAGCTCCAAT

Section 346

	(25531)	25531 25540 25550 25560 25570 25580 25590 25604
Homo sapiens chromosome 1 NC_000001.11: 11783698-...	(24385)	AACAGAATAATCTGTTGAAACCCCT-TGTCCTCATCACACAGCTTCCACAGTGATTTAGCTCAAGCCCAGTCTT
SARS-CoV-2 Reference Genome NC_045512.2	(24470)	TTTGGTGCAATTTCAAGTGTTTTAAATGATATCCTTTCACGTCTTG-ACAAAGTTGAGGCTGAAGTGCAAATTG

Section 347

	(25605)	25605 25610 25620 25630 25640 25650 25660 25678
Homo sapiens chromosome 1 NC_000001.11: 11783698-...	(24458)	CTTTTGTCTGTGCTTTC-GTTATCCACTTCTCCTTTCTCATATTATT--ATTTTTTATTGTATGTGTGTGTGTG
SARS-CoV-2 Reference Genome NC_045512.2	(24543)	ATAGGTTGATCACAGGCAGACTTCAAAGTTTGCAGACATATGTGACTCAACAATTAATTAGAGCTGCAGAAATC

Section 348

	(25679)	25679 25690 25700 25710 25720 25730 25740 25752
Homo sapiens chromosome 1 NC_000001.11: 11783698-...	(24529)	TTTGTGTGTGTGTGTGTGTGTGTGTGT---GTGTGTGTGTGTGTGTGTTTTAAAGTAAAGACAGTCTCCCTATG
SARS-CoV-2 Reference Genome NC_045512.2	(24617)	AGAGCTTCTGCTAATCTTGCTGCTACTAAAATGTCAGAGTGTGTACTTGGACAATCAAAAAGAGTTGATTTTTG

Section 349

	(25753)	25753 25760 25770 25780 25790 25800 25810 25826
Homo sapiens chromosome 1 NC_000001.11: 11783698-...	(24600)	TTGCCCAGGCTGTTCTCGAACTCCTTGACACCAGCGATCCTCCCATGTCAGCCTCCCAAAGTTCTGGGATTACA
SARS-CoV-2 Reference Genome NC_045512.2	(24691)	TGGAAAGGGCTATCATCTTATGTCCTTCCCTCAGTCAGCACCTCATGGTGTAGTC------TTCTTGCATGTGA

Section 350

	(25827)	25827 25840 25850 25860 25870 25880 25890 25900
Homo sapiens chromosome 1 NC_000001.11: 11783698-...	(24674)	GTCATGATCTACCATACCTGGCCGCCCTTCCCATGTTATTTTGAAAGAAATTCTG-TACATCATATTATTTCAC
SARS-CoV-2 Reference Genome NC_045512.2	(24759)	CTTATGTCCCTGCACAAGAAAA-GAACTTCACAACTGCTCCTGCCATTTGTCATGATGGAAAAGCACACTTTCC

Section 351

(25901) 25901 25910 25920 25930 25940 25950 25960 25974
Homo sapiens chromosome 1 NC_000001.11: 11783698-... (24747) CCATAATTCTATATGCATCTCTAATGGATAAGGACTTTTAAAAAGCATAACCCAATACTATTACCACATCTAAA
SARS-CoV-2 Reference Genome NC_045512.2 (24832) TCGTGAAGGTGTCTTTGTTTCAAATGGCACAC-ACTGGTTTGTAACACAAAGGAAT--TTTTATGAACCACAAA

Section 352

(25975) 25975 25980 25990 26000 26010 26020 26030 26048
Homo sapiens chromosome 1 NC_000001.11: 11783698-... (24821) AAATTAGGAATTTCTTGATATCAAATATCTACTGTCCAGATTTTCCAGTTTTCTTGTAAATGTCATATGTATTT
SARS-CoV-2 Reference Genome NC_045512.2 (24903) TCATTACTACA-----GACAACACATTTGT---GTCTGG---TAACTGTGATGTTGTAATAGGAATTGTCAACA

Section 353

(26049) 26049 26060 26070 26080 26090 26100 26110 26122
Homo sapiens chromosome 1 NC_000001.11: 11783698-... (24895) TAACTTTATTTGAATTAGGTTCCAAAAGTCCTTTTTTATT-TGTTTGATAAATCTGTTAAATCTCTTTTAATCT
SARS-CoV-2 Reference Genome NC_045512.2 (24966) ACACAGTTTATGA--TCCTTTGCAA----CCTGAATTAGACTCATTCAAGGAGGAGTTAGATAAATATT----T

Section 354

(26123) 26123 26130 26140 26150 26160 26170 26180 26196
Homo sapiens chromosome 1 NC_000001.11: 11783698-... (24968) GTAGCTTCTTCCTTCATCTTTTTTTTTTTTTTGTAAGTTATTTGACAACATAATTAGGACCTGGGATTTTCTAC
SARS-CoV-2 Reference Genome NC_045512.2 (25030) TAAGAATCATACATCACCAGATGTTGATTTAGGTGACATCTCTGGCA------TTAATGCTTCAG-TTGTAAAC

Section 355

(26197) 26197 26210 26220 26230 26240 26250 26260 26270
Homo sapiens chromosome 1 NC_000001.11: 11783698-... (25042) TGTCTA---GATTTTG-CCATTTGGAGCCGTTTGTATAGT-TTAATATGCTACTCTGATGGGACTGCTTTGAAT
SARS-CoV-2 Reference Genome NC_045512.2 (25097) ATTCAAAAAGAAATTGACCGCCTCAATGAGGTTGCCAAGAATTTAAATGAATCTCTCATCGATCTCCAAGAACT

Section 356

(26271) 26271 26280 26290 26300 26310 26320 26330 26344
Homo sapiens chromosome 1 NC_000001.11: 11783698-... (25111) --GAACCAAAAAGCACTGTATCCACTG-CTGTGTTTTGTTCTATTTCTTTTTTTTCT--TTTTTTTAAATTTG
SARS-CoV-2 Reference Genome NC_045512.2 (25171) TGGAAAGTATGAGCAGTATATAAAATGGCCATGGTACATTTGGCTAGGTTTTATAGCTGGCTTGATTGCCATAG

Section 357

(26345) 26345 26350 26360 26370 26380 26390 26400 26418
Homo sapiens chromosome 1 NC_000001.11: 11783698-... (25180) AGATGGAGTCTCACTCTTAGTGCCCAGGTTGGAGTGCAATGGCTCGATCTCAGCTCACTGCAACCTCCACCCCA
SARS-CoV-2 Reference Genome NC_045512.2 (25245) TAATGGTG----ACAATTA-TGCTTTGCTGTATGACCAGTTGCTGTA-----GTTGTCT-CAAGGGCTGTTGTT

Section 358

(26419)	26419 26430 26440 26450 26460 26470 26480 26492
Homo sapiens chromosome 1 NC_000001.11: 11783698-... (25254)	CCCTAGGTTCAAGCGGTTCTTCTGCCCCAGCCTCCCGAGTAGATGGGATTACAGGCGCCCACCACCATGCC---
SARS-CoV-2 Reference Genome NC_045512.2 (25308)	CTTGTGGATCCTGCTGCAAATTTGATGAAGACGACTCTGAGCCAGTGCTCAAAGGAGTCAAATTACATTACACA

Section 359

(26493)	26493 26500 26510 26520 26530 26540 26550 26566
Homo sapiens chromosome 1 NC_000001.11: 11783698-... (25325)	-CGGCTAATTTTTGTATTTTTAGTAGAGATAGGGTTTCACCATGTTGGCCAGGCTGGTCTCGAACTCC-TGACC
SARS-CoV-2 Reference Genome NC_045512.2 (25382)	TAAACGAACTTATGGATTTGTTTATGAGAATC---TTCACAAT--TGGAACTGTAACTTTGAAGCAAGGTGAAA

Section 360

(26567)	26567 26580 26590 26600 26610 26620 26630 26640
Homo sapiens chromosome 1 NC_000001.11: 11783698-... (25397)	TCAGGTGATACTGCTTCGGTCTCCCAGAGTGCTGGGATTACAGGTATGAGCCACTATACCTGGCCATTTTCTTC
SARS-CoV-2 Reference Genome NC_045512.2 (25451)	TCAAG-GATGCTACTCCT---TCAGATTTTGTTCGCGCTACTGCAACGATAC-CGATACAAGCC----TCACTC

Section 361

(26641)	26641 26650 26660 26670 26680 26690 26700 26714
Homo sapiens chromosome 1 NC_000001.11: 11783698-... (25471)	TGTTTCGTAAG--TATTTGTTTTC-TTGTACATAGTTGAGAAGTTTGCAGGAATT--AGTATAAGATACTCCAT
SARS-CoV-2 Reference Genome NC_045512.2 (25516)	CCTTTCGGATGGCTTATTGTTGGCGTTGCACTTCTTGCTGTTTTTCAGAGCGCTTCCAAAATCATAACCCTCAA

Section 362

(26715)	26715 26720 26730 26740 26750 26760 26770 26788
Homo sapiens chromosome 1 NC_000001.11: 11783698-... (25540)	ATTGTTCTGTCACCTTGCACTCCCTCACTGAACCCTGTTACATCATCCTGACTGTATCACCAGGACTGAGACTG
SARS-CoV-2 Reference Genome NC_045512.2 (25590)	AAAGAGATGGCAACTAGCACTCTCCAAGGG-----TGTT----CACTTTGTTTGCA--ACTTGCTGTTGTTGTT

Section 363

(26789)	26789 26800 26810 26820 26830 26840 26850 26862
Homo sapiens chromosome 1 NC_000001.11: 11783698-... (25614)	TCTCACAGCCTTCATCCTACTTCTAGGAGCTCACCAAGGCTTCCCCATTGACACCCAGGCTCCCTCACCCCTTC
SARS-CoV-2 Reference Genome NC_045512.2 (25653)	TGTAACAGTTTACTCACACCTTTT----GCTCGTTGCTGCTGGCC--TTGAAGCCCCTTTTCTCTATCT--TTA

Section 364

(26863)	26863 26870 26880 26890 26900 26910 26920 26936
Homo sapiens chromosome 1 NC_000001.11: 11783698-... (25688)	TACTAAATTGTACTTACATTTGTAATGTGCTTGCCTTCAGCTGATATGCATATTCATGTAAATTTTTGTTGTTC
SARS-CoV-2 Reference Genome NC_045512.2 (25719)	TGCTTTAGTCTACTTC---TTGCAGAGTATAAACTTTG------TAAGAATAATAATG-AGGCTTTGGCTTTGC

Section 365

	(26937)	26937 26950 26960 26970 26980 26990 27000 27010
Homo sapiens chromosome 1 NC_000001.11: 11783698-...	(25762)	TTGTCAT--TGTTTGAGACAGAG-TCTCACTCTGTCGCCCAGGCTGGAATGCAGTGGTACGGTCTTGGATCACT
SARS-CoV-2 Reference Genome NC_045512.2	(25783)	TGGAAATGCCGTTCCAAAAACCCATTACTTTATGATGCCAACTATTTTCTTTGCTGGCATACTAATTGTT-ACG

Section 366

	(27011)	27011 27020 27030 27040 27050 27060 27070 27084
Homo sapiens chromosome 1 NC_000001.11: 11783698-...	(25833)	GCGACCTCCACCCCGCCTCTGAGTTCAAGCTGTTCTCCTACCTCAGCCTCCCAAGTAGCTGGGACTACATG-C
SARS-CoV-2 Reference Genome NC_045512.2	(25856)	ACTAT-TGTATACCTTACAAT-AGTGTAACTTCTTCAATTGTCATTACTTC---AGGTGATGGCACAACAAGTC

Section 367

	(27085)	27085 27090 27100 27110 27120 27130 27140 27158
Homo sapiens chromosome 1 NC_000001.11: 11783698-...	(25906)	ATACACC--ACCATGCCTGGCTAAGTTTGTATTTTAGT-AGAGATGGGGTTTCACCATGTTGGTCAGGCTGG
SARS-CoV-2 Reference Genome NC_045512.2	(25925)	CTATTTCTGAACATGACTACC--AGATTGGTGGTTATACTGAAAAATGGGAATCTGGAGTAAAAGACTGTGTTG

Section 368

	(27159)	27159 27170 27180 27190 27200 27210 27220 27232
Homo sapiens chromosome 1 NC_000001.11: 11783698-...	(25977)	TCTTGAACTCCTGACCTCAGGTGATCCACCCACCTCAGCCTCCCAAAGTACTGGGATTACAAGCG-TGAGCCAC
SARS-CoV-2 Reference Genome NC_045512.2	(25997)	TATTACACAGTT-ACTTCACTTCAGACTATTACCAGCTGTACTCAACTCAATTG-AGTACAGACACTGGTGTTG

Section 369

	(27233)	27233 27240 27250 27260 27270 27280 27290 27306
Homo sapiens chromosome 1 NC_000001.11: 11783698-...	(26050)	CACGCGTGGCCATATTCATGTACATTTCATGGTTAATGTTCTTTTGATATTGGTCATTGATATTTCTCAACCA
SARS-CoV-2 Reference Genome NC_045512.2	(26069)	AACATGTTACCTTCTTCATCTACAATAAAATTGTTGATGAGCCTGAAGAACATGTCCA----AATTCACA-CAA

Section 370

	(27307)	27307 27320 27330 27340 27350 27360 27370 27380
Homo sapiens chromosome 1 NC_000001.11: 11783698-...	(26124)	TCTTCAAGAATGAGCTTCTTGAGAGCACTTGCT--TTGTGTAA--GAACAAATTCAGGCACTTAAACAGAAAAG
SARS-CoV-2 Reference Genome NC_045512.2	(26138)	TC-----GACGGTTCATCCGGAG-----TTGTTAATCCAGTAATGGAACCAATTTATGA--TGAACCGACGACG

Section 371

	(27381)	27381 27390 27400 27410 27420 27430 27440 27454
Homo sapiens chromosome 1 NC_000001.11: 11783698-...	(26194)	TCTGGATTTTATTTATTCAATAAATAAATCAACTTGACACAAAAAGTATAATAAAGAGGGGAGTGAGTGG-GAC
SARS-CoV-2 Reference Genome NC_045512.2	(26200)	ACT--ACTAGCGTGCCTTTGTAAGCACAAGCTGATGAGTACGAACTTATG-TACTCATTCGTTTCGGAAGAGAC

```
                                                                    Section 372
                                                 (27455) 27455 27460      27470      27480      27490      27500      27510              27528
Homo sapiens chromosome 1 NC_000001.11: 11783698-... (26267) AGGTTAA--AATAGTTGATTTAGTGGAATCTCATTTTTGTTATTTAAAACACGTGTTAAAAGCATGTGTATAA
     SARS-CoV-2 Reference Genome NC_045512.2 (26271) AGGTACGTTAATAGTTAAT--AGCGTACT-TCTTTTTCTTGCTTT------CGTGGTAT-----TCTTGCTAGT
                                                                    Section 373
                                                 (27529) 27529      27540      27550      27560      27570      27580      27590      27602
Homo sapiens chromosome 1 NC_000001.11: 11783698-... (26339) AACGTGGGTAAGGATGTAGAAGGATATTTAGTAAG-GTATTAATAGTCATATT-TTAATGAATGGAGT-TATAG
     SARS-CoV-2 Reference Genome NC_045512.2 (26331) TACACTAGCCATCCTTACTGCGCTTCGATTGTGTGCGTACTGCT-GCAATATTGTTAACGTGAGTCTTGTAAAA
                                                                    Section 374
                                                 (27603) 27603  27610      27620      27630      27640      27650      27660          27676
Homo sapiens chromosome 1 NC_000001.11: 11783698-... (26410) GGTTTTCTCTACCTTGATAGTCAGTCAACTAGAAAACCCTTCTGTTGAATATCTTTCTAAAAAGTGCCCCTTCT
     SARS-CoV-2 Reference Genome NC_045512.2 (26404) CCTTCTTTTTACGTT--TACTC--TCGTGTTAAAAATC-------TGAAT-TCTT-CTAGA--GTTCCTGATCT
                                                                    Section 375
                                                 (27677) 27677        27690      27700      27710      27720      27730      27740  27750
Homo sapiens chromosome 1 NC_000001.11: 11783698-... (26484) TCCTGTCTCAG-GAAGAGTTCATTGCACATATTTGACTCTTTATTTGATAAATTTAGGGTCTTACAAAATGTTA
     SARS-CoV-2 Reference Genome NC_045512.2 (26463) TCTGGTCTAAACGAACTAAATATTATATTAGTTTTTCTGTTTGGAACTTTAATTTTAGCCATGGCAGATTCCAA
                                                                    Section 376
                                                 (27751) 27751    27760      27770      27780      27790      27800      27810          27824
Homo sapiens chromosome 1 NC_000001.11: 11783698-... (26557) GAATCCCATTTTGACATCCATTCTCATTATAGGTGGTTTGAAAAATGGTAACTTTCTGTGAGATGTGCCATTGC
     SARS-CoV-2 Reference Genome NC_045512.2 (26537) CGGTACTATTAC--CGTTGAAGAGCTTAAAAAGCTCCTTGAACAATGG-AACCTAGTAATAGGTTTCCTATTCC
                                                                    Section 377
                                                 (27825) 27825 27830      27840      27850      27860      27870      27880              27898
Homo sapiens chromosome 1 NC_000001.11: 11783698-... (26631) AAACA-GGAATGTTTTCATGCAGGTCACACAGTCTATTTATATTGTACCTTTGAATTAGAAAGAGGCACACTCA
     SARS-CoV-2 Reference Genome NC_045512.2 (26608) TTACATGGATTTGTCTTCTACAATTTGCCTATGCCAACAGGAATAGGTTTTTGTAT-ATAATTAAGTTAATTTT
                                                                    Section 378
                                                 (27899) 27899      27910      27920      27930      27940      27950      27960      27972
Homo sapiens chromosome 1 NC_000001.11: 11783698-... (26704) GAACGTAAGACTTAGAATTAAAGGCCTCCCCCACTGGCCATAGCAGCACCCCTAGCTCACAACCAGTTGAAGGT
     SARS-CoV-2 Reference Genome NC_045512.2 (26681) ---CCTCTGGCTG-----TTATGGCCAGTAACTTTAGCTTGTTTTGTGCTTGCTGCTGTTTAC-AGAATAAATT
```

```
                                                                   Section 379
                                                        (27973) 27973  27980     27990     28000     28010     28020     28030         28046
Homo sapiens chromosome 1 NC_000001.11: 11783698-...  (26778) GGTTTT--GGTGTGAACACTAA--GATGCCCCTTCCTGGTTGAATTGGGGCTGGTTCAGCCCCCACCTCCCTCC
SARS-CoV-2 Reference Genome NC_045512.2               (26746) GGATCACCGGTGGAATTGCTATCGCAATGGCTTGTCTTGTAGGCTTGATG-TGGCTCAGCTACTTCATTGCTTC
                                                                   Section 380
                                                        (28047) 28047         28060     28070     28080     28090     28100     28110  28120
Homo sapiens chromosome 1 NC_000001.11: 11783698-...  (26848) CGGCCAGGTGCCATTTTGCTCGTAACCCCCTGTAACAGCCCTGCAAGACACAGA-TAACATTTTATTTTTTAA
SARS-CoV-2 Reference Genome NC_045512.2               (26819) TTTCAGACTG----TTTGCGCGTACGCGTTCCATGTGGTCATTCAATCCAGAAACTAACATTCTTCTC------
                                                                   Section 381
                                                        (28121) 28121    28130     28140     28150     28160     28170     28180         28194
Homo sapiens chromosome 1 NC_000001.11: 11783698-...  (26921) AAAGTTGTTAGTCCCCTAAACCAAAGAGCAACATATACTGCAGAAATAAAAAGTTAGTCATAGCAAAGGAGAAG
SARS-CoV-2 Reference Genome NC_045512.2               (26883) -AACGTGCCACTCCATGGCACTATTCTG-ACCAGA--CCGCTTCTAGAAAGTGA-ACTCGTA--ATCGGAGCTG
                                                                   Section 382
                                                        (28195) 28195 28200     28210     28220     28230     28240     28250             28268
Homo sapiens chromosome 1 NC_000001.11: 11783698-...  (26995) TAACAGTATCTGGTTTTAATAATAGCTCATATATTCCAT-TGGTACATATTTACA--AAGGGCCAAACTCAACT
SARS-CoV-2 Reference Genome NC_045512.2               (26950) TGATCCTTCGTGGACATCTTCGTATTGC-TGGACACCATCTAGGACGCTGTGACATCAAGGACCTGCCTAAAGA
                                                                   Section 383
                                                        (28269) 28269       28280     28290     28300     28310     28320     28330      28342
Homo sapiens chromosome 1 NC_000001.11: 11783698-...  (27066) AATTTAT-TTAAAAAAAAAAGAAGCGTGGCACAGTGGCTCACGCCTATATGCTCAGCACTTTGGGAGGCCAAGG
SARS-CoV-2 Reference Genome NC_045512.2               (27023) AATCACTGTTGCTACATCACGAACGCTTTCTTATTA-CAAATTGGGAGCTTCGCAGCG-TGTAGCAGGTGACTC
                                                                   Section 384
                                                        (28343) 28343  28350     28360     28370     28380     28390     28400         28416
Homo sapiens chromosome 1 NC_000001.11: 11783698-...  (27139) CAGGAGGATCGCTTAAAGCCAGGAGTTCAAGACCATCCTGGGCAATAAAGTGAGACTTTGTTCCTACAAAAAAA
SARS-CoV-2 Reference Genome NC_045512.2               (27095) ----AGGTTTTGCTGCATACAGTCGCTACAGG--ATTGGCAACTATAAATTAA-ACACAGACCATTCCAGTAGC
                                                                   Section 385
                                                        (28417) 28417         28430     28440     28450     28460     28470     28480  28490
Homo sapiens chromosome 1 NC_000001.11: 11783698-...  (27213) TTTGTTCAAAATTAGCAGGGGGCCGGGTGCAGTGGCCCACACCTGTTACCCCC---ACACTTT-AGGAGGCTGA
SARS-CoV-2 Reference Genome NC_045512.2               (27162) AGTGA-CAATATT-GCTTTGCTTGTACAGTAAGTGACAACAGATGTTTCATCTCGTTGACTTTCAGGTTACTAT
```

Section 386

```
                                                         (28491) 28491    28500     28510     28520     28530     28540     28550        28564
Homo sapiens chromosome 1 NC_000001.11: 11783698-...     (27283) GGCAGGTGGATCACT---TGAGCCCAGGACTTCATACCAGCCTGGGCAACATGGCGAGACTCCATC-TCAACAA
SARS-CoV-2 Reference Genome NC_045512.2                  (27234) AGCAGAGATATTACTAATTATTATGAGGACTTTTAAAGTTTCCATTTGGAAT--CTTGATTACATCATAAACCT
```

Section 387

```
                                                         (28565) 28565 28570    28580     28590     28600     28610     28620             28638
Homo sapiens chromosome 1 NC_000001.11: 11783698-...     (27353) CAAATACGAAAAAATTAACGAGGTGTGGTGGCTAACCTGTGGTCCCCCCTACTCAGGAGATCGAAGCAGGTGAA
SARS-CoV-2 Reference Genome NC_045512.2                  (27306) CATA-ATTAAAAATTTATCTAAGTCA-----CTAAC-TGAGAATAAATATTCTCAAT---TAGATGAAGAGCAA
```

Section 388

```
                                                         (28639) 28639      28650     28660     28670     28680     28690     28700     28712
Homo sapiens chromosome 1 NC_000001.11: 11783698-...     (27427) TTGCTTGAGCCCAGGAGACGGAGGTTGCAGAGAGCTGTGATCACGCCACTGCACTCCAGCCTGGGTGACAGAGC
SARS-CoV-2 Reference Genome NC_045512.2                  (27370) CCAATGGAGATTGATTAAACGAACATGAAAATTATTCTTTTCTTGGCACTG-ATAACA--CTCGCTACTTGTGA
```

Section 389

```
                                                         (28713) 28713  28720     28730     28740     28750     28760     28770           28786
Homo sapiens chromosome 1 NC_000001.11: 11783698-...     (27501) GAGACCCTGACAAAAAAAAAAGGGATTCTGGTGTTCTAGCGTTGCGGTTGTTTCTAAATGCCCTTCTTAGCAGT
SARS-CoV-2 Reference Genome NC_045512.2                  (27441) G---CTTTATCACTACCAAGAGTGTGTTAGAGGTACAA------CAGTACTTTTAAAAGAACCTT-------GC
```

Section 390

```
                                                         (28787) 28787        28800     28810     28820     28830     28840     28850 28860
Homo sapiens chromosome 1 NC_000001.11: 11783698-...     (27575) TTTATTGCACTATAATTGGCATACTATTCAGTTCACCCATGTAGA-GTGTACAA---TTCAATGG--GGTTTTA
SARS-CoV-2 Reference Genome NC_045512.2                  (27499) TCTTCTGGAACATACGAGGGCAATTCACCATTTCATCC-TCTAGCTGATAACAAATTTGCACTGACTTGCTTTA
```

Section 391

```
                                                         (28861) 28861    28870     28880     28890     28900     28910     28920        28934
Homo sapiens chromosome 1 NC_000001.11: 11783698-...     (27643) AAAATTAGTATATTCACAGAGTTGTACAACCATTCCTACAGTCAATTTTTTTTTTTTTTTGAGACGGAGTTTCG
SARS-CoV-2 Reference Genome NC_045512.2                  (27572) GCACTCA--ATTTGCTTTTGCTTGTCCTGACGGCGTAAAACACGTCTATCAGTTACGTGCCAGATC-AGTTTCA
```

Section 392

```
                                                         (28935) 28935 28940    28950     28960     28970     28980     28990             29008
Homo sapiens chromosome 1 NC_000001.11: 11783698-...     (27717) CTCT---TGTTGCCCAGGCAAGAGTGCAATGTTGTGATCTCAGCTCACTGCAACCTCTGCCTCCCGGGTTCA-A
SARS-CoV-2 Reference Genome NC_045512.2                  (27643) CCTAAACTGTTCATCAGACAAGAGG--AAGTTCAAGAACTTTACTCTCCAATTTTTCTTATTGTTGCGGCAATA
```

Section 393

(29009) 29009 29020 29030 29040 29050 29060 29070 29082
Homo sapiens chromosome 1 NC_000001.11: 11783698-... (27787) GTGATTCTC-C--T--GCCTCAGCCTCCCAAGTAGCTGGGATTACAGGCGTCTGCCACCACACCCGGCTAATTT
SARS-CoV-2 Reference Genome NC_045512.2 (27715) GTGTTTATAACACTTTGCTTCACACTCAAAAGAAAGACAGAATGATTGAACTTTCATTAATTGACTTCTA--TT

Section 394

(29083) 29083 29090 29100 29110 29120 29130 29140 29156
Homo sapiens chromosome 1 NC_000001.11: 11783698-... (27856) TGTATTTTTAGTAGAGACGAGGCTTCTCCATGTTGGTCAGGCTGGTCTTGAACTCCTGGC-CTCGGGTGATCCG
SARS-CoV-2 Reference Genome NC_045512.2 (27787) TGTGCTTTT--TAGCCTTTCTGCTATTCCTTGTTT-TAATTATGCTTATTATCTTTTGGTTCTCACTTGAACTG

Section 395

(29157) 29157 29170 29180 29190 29200 29210 29220 29230
Homo sapiens chromosome 1 NC_000001.11: 11783698-... (27929) CCCGCCTTGGCCTCCCAAAGTGCTGGGATTACAGACGTGAGCCACTGCGCCCAGCTAATTCCTACAGTCAATTT
SARS-CoV-2 Reference Genome NC_045512.2 (27858) CAAGA-------TCATAA-----TGAAACTTGTCACG----CCTAAACGAACATGAAATTTCTTGTTTT---TCT

Section 396

(29231) 29231 29240 29250 29260 29270 29280 29290 29304
Homo sapiens chromosome 1 NC_000001.11: 11783698-... (28003) TAGAATATTTTCATCACCTGAGAGGAAACCCTGCACTCAT-TAGCAGCCACTGCCTGTTCTCCTTGCCCCTGAC
SARS-CoV-2 Reference Genome NC_045512.2 (27913) TAGGA-ATCATCACAACTGTAGCTGCATTTCACCAAGAATGTAGTTTACAGT-CATGTACTCAACATCA---AC

Section 397

(29305) 29305 29310 29320 29330 29340 29350 29360 29378
Homo sapiens chromosome 1 NC_000001.11: 11783698-... (28076) TCCAGGCAACCAGTGATCTCCTTTCTGTCTATTTGCCTATTCTAGATATCTAAAAACCTTCTAAATGTTTACGG
SARS-CoV-2 Reference Genome NC_045512.2 (27982) CATATGTAGTTGATGACCCGTGTCCTATTCACTT--CTATTCTAAATGGTATATTAGAGTAGGA--GCTAGAAA

Section 398

(29379) 29379 29390 29400 29410 29420 29430 29440 29452
Homo sapiens chromosome 1 NC_000001.11: 11783698-... (28150) AACCATATCATTATTAGATGGAACCATCAATGAAGTACTTGACCAGTAATTTCCATACATATTTTGGATGAAGG
SARS-CoV-2 Reference Genome NC_045512.2 (28052) ATCAGCACCTTTAATTGAATTGTGCGTGGATGAGG--CTGGTTCTAAATCACCCATTCAGTACATCGATATCGG

Section 399

(29453) 29453 29460 29470 29480 29490 29500 29510 29526
Homo sapiens chromosome 1 NC_000001.11: 11783698-... (28224) CAAGGGTATTGTTTCTCCTCTGCTCTCAGGCACCACTCGC--GGAACACTGCTAACACCGATATGTGGAG-GTT
SARS-CoV-2 Reference Genome NC_045512.2 (28124) TAATTATACAGTTTCCTGTTTACCTTTTACAATTAATTGCCAGGAACCTAAATTGGGTAGTCTTGTAGTGCGTT

```
Section 400
                                                              (29527) 29527        29540    29550    29560    29570    29580    29590 29600
Homo sapiens chromosome 1 NC_000001.11: 11783698-... (28295) GTTTCCCCACTACTCAGGAGGCTGAGGTGGGAGGATGGCTTAAGCCCAGGAGTTGGAGGTTGCAGTGAACCGAG
    SARS-CoV-2 Reference Genome NC_045512.2 (28198) GTT--CGTTCTATGAAGACTTTTTAGA--GTATCATGACGTTCGT---GTTGTTTTAGATTTCATCTAAACGAA
```

```
Section 401
                                                              (29601) 29601     29610    29620    29630    29640    29650    29660    29674
Homo sapiens chromosome 1 NC_000001.11: 11783698-... (28369) ATTATGCCACTG-CACTCCATG---CTGGGTGACAGAGAAAG--ACCCCATCTCAAAAAAAAAAAGTAGTAGGT
    SARS-CoV-2 Reference Genome NC_045512.2 (28265) CAAACTAAAATGTCTGATAATGGACCCCAAAATCAGCGAAATGCACCCCGCATTACGTTTGGTGGACCCTCAGA
```

```
Section 402
                                                              (29675) 29675 29680    29690    29700    29710    29720    29730       29748
Homo sapiens chromosome 1 NC_000001.11: 11783698-... (28437) TGTCACCTGCATTTCTCAGCAGCTGGCAGTGAATTGAAGGTTCACAGGACTCCCTCCTTGAGTTTGGTTAACTT
    SARS-CoV-2 Reference Genome NC_045512.2 (28339) TTCAACTGGCAGTAACCAGAATGGAGAACGCAGTGGGGCGCGATCAAAACAACGTCGGCCCCAAGGTTTACCCA
```

```
Section 403
                                                              (29749) 29749      29760    29770    29780    29790    29800    29810   29822
Homo sapiens chromosome 1 NC_000001.11: 11783698-... (28511) GCTAGAGCGGGTCAGAAAACACAGAAATGCTACTTACATTTGCTAGTTTATTAATAAAGGATATC----ATAAA
    SARS-CoV-2 Reference Genome NC_045512.2 (28413) ATAATACTGCGTCTTGGTTCACCGCTCTC--ACTCAACATGGCAAGGAAGACCTTAAATTCCCTCGAGGACAAG
```

```
Section 404
                                                              (29823) 29823   29830    29840    29850    29860    29870    29880       29896
Homo sapiens chromosome 1 NC_000001.11: 11783698-... (28581) GGATACAGAAGAACAGCCAGATGGAGAGATTCAAGGAACGTGGGAGGGGGCACGGAGCTTTCATGCCCTCTCCA
    SARS-CoV-2 Reference Genome NC_045512.2 (28485) GCGTTCCAATTAACACCAATAGCAGTCCAGATGACCAAATTGGCTACTACCGAAGAGCTACCAGACGAATTCGT
```

```
Section 405
                                                              (29897) 29897        29910    29920    29930    29940    29950    29960 29970
Homo sapiens chromosome 1 NC_000001.11: 11783698-... (28655) GGC----ACGCTACCCTCCAGGAACCTCCAAACGGTTCAGCTGTCTGGAAGCTCTCTGAACCTTGTCATTT--T
    SARS-CoV-2 Reference Genome NC_045512.2 (28559) GGTGGTGACGGTAAAATGAAAGATCT--CAGTCCAAGATGGTATTTCTACTACCTAGGAACTGGGCCAGAAGCT
```

```
Section 406
                                                              (29971) 29971     29980    29990    30000    30010    30020    30030    30044
Homo sapiens chromosome 1 NC_000001.11: 11783698-... (28723) GGAGTT--TTATGGAAGCTTTTTTATGCAG--TCATGATTGATTACATCACCAGC--CCTTGGTGATCAGCTTA
    SARS-CoV-2 Reference Genome NC_045512.2 (28631) GGACTTCCCTATGGT-GCTAACAAAGACGGCATCAT-ATGGGTTGCAACTGAGGGAGCCTTGA--ATACACCAA
```

Section 407

```
                                                    (30045) 30045 30050     30060     30070     30080     30090     30100             30118
Homo sapiens chromosome 1 NC_000001.11: 11783698-... (28791) ACCTTCAGCTTCGGTCTCCTCCCTGGAGGGATGGGTG-TCCCACCCCGCTAATCCTGCCTT---GGTCTTTCTG
   SARS-CoV-2 Reference Genome NC_045512.2 (28701) AAGATCACATTGGCAC-CCGCAATCCTGCTAACAATGCTGCAATCGTGCTACAACTTCCTCAAGGAACAACATT
```

Section 408

```
                                                    (30119) 30119      30130     30140     30150     30160     30170     30180     30192
Homo sapiens chromosome 1 NC_000001.11: 11783698-... (28861) GTGACCAGCCC-CTTTTCTGAAGCCATCTAAGGGCCCCAGCCGCCACTCATCTCATTAGCAAACAAA-AGATA
   SARS-CoV-2 Reference Genome NC_045512.2 (28774) GCCAAAAGGCTTCTACGCAGAAGGGAGCAGAGG--CGGCAGTCAAGCCTCTTCTCGTTCCTCATCACGTAGTCG
```

Section 409

```
                                                    (30193) 30193  30200     30210     30220     30230     30240     30250          30266
Homo sapiens chromosome 1 NC_000001.11: 11783698-... (28933) CCACGGCTCAAAAAAAAAAA---AAGACAA-AGTATGTTTGTGTGTG-TGTGTGTGTGTGTGTGTGTGTGTG
   SARS-CoV-2 Reference Genome NC_045512.2 (28846) CAACAGTTCAAGAAATTCAACTCCAGGCAGCAGTAGGGGAACTTCTCCTGCTAGAATGGCTGGCAATGGCGGTG
```

Section 410

```
                                                    (30267) 30267        30280     30290     30300     30310     30320     30330 30340
Homo sapiens chromosome 1 NC_000001.11: 11783698-... (29002) TGTGTGTGTGTGTATTGTTGGGGCTCAGAAAATGATACC---T-GGAAATATGGTGCTTTGATATGCTGAACTC
   SARS-CoV-2 Reference Genome NC_045512.2 (28920) ATGCTGCTCTTGCTTTGCTGCTGCTTGACAGATTGAACCAGCTTGAGAGCAAAATGTCTGGTAAAGGCCAACAA
```

Section 411

```
                                                    (30341) 30341     30350     30360     30370     30380     30390     30400         30414
Homo sapiens chromosome 1 NC_000001.11: 11783698-... (29072) TCCACCCTCACACTTTGTCTCTCCCAAAGCACAGGATGAGGCTCTTCTCTGCCGTTCCCTTATCTACCTAGAAA
   SARS-CoV-2 Reference Genome NC_045512.2 (28994) -CAACAAGGCCAAACTGTCACTAAGAAATCTGCTGCTGAGGCT--TCTAAGAAGCCTCGGCAAAAACGTACTGC
```

Section 412

```
                                                    (30415) 30415 30420     30430     30440     30450     30460     30470             30488
Homo sapiens chromosome 1 NC_000001.11: 11783698-... (29146) CTGGACCTACCAGAGAGGAACACA-GTTGTCTTTGATCCCTTTCCTGAAATGTCATTAACCA------GGGAAC
   SARS-CoV-2 Reference Genome NC_045512.2 (29065) CACTAAAGCATACAATGTAACACAAGCTTTCGGCAGACGTGGTCCAGAACAAACCCAAGGAAATTTTGGGGACC
```

Section 413

```
                                                    (30489) 30489      30500     30510     30520     30530     30540     30550     30562
Homo sapiens chromosome 1 NC_000001.11: 11783698-... (29213) ATTAAAACTCATCTTACAGAGGAGACTGAAGACCAAACAACATACCTAGAGCCTGGACAAACTGTCCCAACCG-
   SARS-CoV-2 Reference Genome NC_045512.2 (29139) AGGAA--CTAATCAGACA-AGGA-ACTGATTAC-AAACATTGGCCGCAAAT--TGCACAATTTGCCCCCAGCGC
```

```
                                                        Section 414
                                                  (30563) 30563 30570 30580 30590 30600 30610 30620 30636
Homo sapiens chromosome 1 NC_000001.11: 11783698-... (29286) TTGTTTGTTC-----ACTGTCCCATACAATTTCCAAAGAGAATCATTCAC-AAGATGATATCTGCTTCCCAGAT
SARS-CoV-2 Reference Genome NC_045512.2 (29206) TTCAGCGTTCTTCGGAATGTCGCGCATTGGCATGGAAGTCACACCTTCGGGAACGTGGT-TGACCTACACAGGT

                                                        Section 415
                                                  (30637) 30637 30650 30660 30670 30680 30690 30700 30710
Homo sapiens chromosome 1 NC_000001.11: 11783698-... (29354) CCATTCA---TTCCCCACTAAGAATT--ATTTCCTC--CCACATCCCTAT-CTCCCCTTCCCCTGTGAAGAAGA
SARS-CoV-2 Reference Genome NC_045512.2 (29279) GCCATCAAATTGGATGACAAAGATCCAAATTTCAAAGATCAAGTCATTTTGCTGAATAAGCATATTGACGCATA

                                                        Section 416
                                                  (30711) 30711 30720 30730 30740 30750 30760 30770 30784
Homo sapiens chromosome 1 NC_000001.11: 11783698-... (29420) GTATAGA-----AGCGTCTGTACCTCGCTGGCATTATT-------GGGTAATCATTCTGCAG---TTCCCCTGT
SARS-CoV-2 Reference Genome NC_045512.2 (29353) CAAAACATTCCCACCAACAGAGCCTAAAAAGGACAAAAAGAAGAAGGCTGATGAAACTCAAGCCTTACCGCAGA

                                                        Section 417
                                                  (30785) 30785 30790 30800 30810 30820 30830 30840 30858
Homo sapiens chromosome 1 NC_000001.11: 11783698-... (29479) G-CA-ATGCATGTTAAATAAGTGGATGCCTTTGCTCCCATTAATCTGCCT--TTTGTCGGTTCACTTTCAGCAG
SARS-CoV-2 Reference Genome NC_045512.2 (29427) GACAGAAGAAACAGCAAACTGTGACTCTTCTTCCTGCTGCAGATTTGGATGATTTCTCCAAACAATTGCAACAA

                                                        Section 418
                                                  (30859) 30859 30870 30880 30890 30900 30910 30920 30932
Homo sapiens chromosome 1 NC_000001.11: 11783698-... (29549) ACCTTTAG-AGAG-TGAA--AGGGGAAGCTTTTCCTCTTG--GCCGCTACAATACATATGTAGGTGCATATATG
SARS-CoV-2 Reference Genome NC_045512.2 (29501) TCCATGAGCAGTGCTGACTCAACTCAGGCCTAAACTCATGCAGACCACACAAGGCAGATGG-GCTATATAAACG

                                                        Section 419
                                                  (30933) 30933 30940 30950 30960 30970 30980 30990 31006
Homo sapiens chromosome 1 NC_000001.11: 11783698-... (29617) TTTAATCACTTATGGATGAGTCTAGTTTCTAAAAG-TACTTTTTTCATCACTGTGTT-TCTTTTCTGATCAGTA
SARS-CoV-2 Reference Genome NC_045512.2 (29574) TTT--TCGCTTTTCC----GTTTACGATATATAGTCTACTCTTGTGCAGAATGAATTCTCGTAACTACATAGCA

                                                        Section 420
                                                  (31007) 31007 31020 31030 31040 31050 31060 31070 31080
Homo sapiens chromosome 1 NC_000001.11: 11783698-... (29689) -AAAAAGATTTTTTTTTTTTTTTTGAGACCGAG---TCTC-ACTCTGTCGCCTAGGCTGGAGTGCAGTAGCACA
SARS-CoV-2 Reference Genome NC_045512.2 (29642) CAAGTAGATGTAGTTAACTTTAATCTCACATAGCAATCTTTAATCAGTGTGTAACATTAGGGAGGACTTGAAAG
```

Section 421

	(31081)	31081 … 31154
Homo sapiens chromosome 1 NC_000001.11: 11783698-...	(29758)	ATCTCGGCTCACTGCAGCCTCTGCCTC-CTGGGTTCAAGCGATTCTCCTGCCTCAGCCACCCTAGT---AGCTG
SARS-CoV-2 Reference Genome NC_045512.2	(29716)	AGC-CACCACATTTTCACCGAGGCCACGCGGAGTACGATCGAGTGTACAG--TGAACAATGCTAGGGAGAGCTG

Section 422

	(31155)	31155 … 31228
Homo sapiens chromosome 1 NC_000001.11: 11783698-...	(29828)	GGATTACAAGTGTGTGCCACCACACCCACCTAATTTGTGTATTTTTGGTAGAGACGGGGTTTCGCCATGTTGGC
SARS-CoV-2 Reference Genome NC_045512.2	(29787)	CCTATATGGAAGAGCCCTAATGTGTAAAATTAATTTTAGTAGTGCTA-TCCCCATGTGATTTTAATAGCTTCTT

Section 423

	(31229)	31229 … 31272
Homo sapiens chromosome 1 NC_000001.11: 11783698-...	(29902)	CGG---
SARS-CoV-2 Reference Genome NC_045512.2	(29860)	AGGAGAATGACAAAAAAAAAAAAAAAAAAAAAAAAAAAAAAAAA
