# Supplementary material for: Patch type nucleotide sequence identities between genomes from many different species facilitate illegitimate recombination
Source: Sci Rep. 2026 Mar 30;16:10524. doi: 10.1038/s41598-026-44124-0 (PMC13035915; doi:10.1038/s41598-026-44124-0)
Supplement: Supplementary file 3 — Supplementary Material 3 [file 41598_2026_44124_MOESM3_ESM.pdf]

```
                                                                        Section 1
                                                    (1)   1       10        20        30        40        50        60           74
Homo sapiens chromosome 13 NC_000013.11: 34882059... (1)  -------TTTTAT--C----CAGAT----TAACTTCTTCTCTTAAATGTACAGCAGTCCAGAGCTAAAAATGAA
       SARS-CoV-2 Reference Genome NC_045512.2      (1)   ATTAAAGGTTTATACCTTCCCAGGTAACAAACCAACCAACTTTCGATCTCTTGTAGATCTGTTCTCTAAACGAA
                                                                        Section 2
                                                    (75)  75   80        90        100       110       120       130            148
Homo sapiens chromosome 13 NC_000013.11: 34882059... (58) ACACAAAAAAATTGCAATTACTTTTTAAATGAAAAAATCCCTGTACTGCCATG-AGATTAGTCCTTAAACCTGT
       SARS-CoV-2 Reference Genome NC_045512.2      (75)  CTTTAAAATCTGTG---TGGCTGTCACTCGGCTGCATGCTTAGTGCACTCACGCAGTATAATTAATAACTAATT
                                                                        Section 3
                                                    (149) 149        160       170       180       190       200       210         222
Homo sapiens chromosome 13 NC_000013.11: 34882059... (131) AAGATTTGGGGAAAGAAATAATTTTTTGCTCCATTACCCTAAAACATTCCAAAGGAATATTAAAT-TTGAATGA
       SARS-CoV-2 Reference Genome NC_045512.2      (146) ACTGTCGTTGACAGGACACGAGTAACTCGTCTATCTTCTGCAGGC-TGCTTACGGTTTCGTCCGTGTTGCAGCC
                                                                        Section 4
                                                    (223) 223     230       240       250       260       270       280          296
Homo sapiens chromosome 13 NC_000013.11: 34882059... (204) GAACATTTAAAAATACCA-TTTAAAAAATTTTTCACTGATGATTAAACTGATGTTAAATTATTTACACTTATTTT
       SARS-CoV-2 Reference Genome NC_045512.2      (219) GATCATCAGCACATCTAGGTTTCGTCCGGGTGTGACCGAAAGGTAA---GATGGAGAGCCTTGTCCCTGGTTTC
                                                                        Section 5
                                                    (297) 297          310       320       330       340       350       360     370
Homo sapiens chromosome 13 NC_000013.11: 34882059... (277) CTTGACAATTTATAAAACTAACC-----TCTCTCTTTCAAA----CTCCATTATATTTTTAGTCCTGTAGAATT
       SARS-CoV-2 Reference Genome NC_045512.2      (290) AACGAGAAAACACACGTCCAACTCAGTTTGCCTGTTTTACAGGTTCGCGACGTGCTCGTACGTGGCTTTGGAGA
                                                                        Section 6
                                                    (371) 371      380       390       400       410       420       430         444
Homo sapiens chromosome 13 NC_000013.11: 34882059... (342) CCATTTGGATGTTATCTTGTTATTTTTG-GTCAGTCTTTGAAGAATAACACCAACGATTATGCACCAGCAACCT
       SARS-CoV-2 Reference Genome NC_045512.2      (364) CTCCGTGGAGGAGGTCTTATCAGAGGCACGTCAACATCTTAAAGATGGCACTTGTGGCTTAGTAGAAG-----T
                                                                        Section 7
                                                    (445) 445  450       460       470       480       490       500            518
Homo sapiens chromosome 13 NC_000013.11: 34882059... (415) GCCACTAAGTCACCTGCTTAAATAAGCGAGAGGCCGTCACTACTCAATACACTCTTCTTTTAATGAGCTAATAA
       SARS-CoV-2 Reference Genome NC_045512.2      (433) TGAAAAAGGCGTTTTGCCTCAACTTG--AACAGCCCTATGTGTTCATCAAACGTTCGGATGCTCGAACTGCACC
```

Section 8

		519 … 592
	(519)	519 530 540 550 560 570 580 592
Homo sapiens chromosome 13 NC_000013.11: 34882059...	(489)	TTAAAGCTTTATTTTGAATATAAATAGTGCCATTTATACACAGAA------ACACACATGAAGATTATTTACTA
SARS-CoV-2 Reference Genome NC_045512.2	(505)	TCATGGTCATGTTATGGTTG-AGCTGGTAGCAGAACTCGAAGGCATTCAGTACGGTCGTAGTGGTGAGACACTT

Section 9

		593 … 666
	(593)	593 600 610 620 630 640 650 666
Homo sapiens chromosome 13 NC_000013.11: 34882059...	(557)	AATCTCCATAT---TGGTGTTTATGAATCAAAATTTCTTCATTAAAATATTACCCTATTTAGTATATGAATTTT
SARS-CoV-2 Reference Genome NC_045512.2	(578)	GGTGTCCTTGTCCCTCATGTGGGCGAAATACCAGTGGCTTACCGCAAGGTTCTTCTTCGTAAGAACGGTAATAA

Section 10

		667 … 740
	(667)	667 680 690 700 710 720 730 740
Homo sapiens chromosome 13 NC_000013.11: 34882059...	(628)	AAAAAATAAAAAAT-TATTTCCCAAGTCCATTCTAAAACCCTC---CCTTGGTTATTATAAGGTTAAT-ATATT
SARS-CoV-2 Reference Genome NC_045512.2	(652)	AGGAGCTGGTGGCCATAGTTACGGCGCCGAT-CTAAAGTCATTTGACTTAGGCGACGAGCTTGGCACTGATCCT

Section 11

		741 … 814
	(741)	741 750 760 770 780 790 800 814
Homo sapiens chromosome 13 NC_000013.11: 34882059...	(697)	TAAGAATTTGTCTGAACTTATTTCCATCTCTCCTACACAAAGATTAAGCATGAAACAATATATCCCCAGAGTAG
SARS-CoV-2 Reference Genome NC_045512.2	(725)	TATGAAGATTT-TCAAGAAAACTGGAACACT---AAACATAGCAGTGG--TGTTACCCGTGAACTCATGCGTGA

Section 12

		815 … 888
	(815)	815 820 830 840 850 860 870 888
Homo sapiens chromosome 13 NC_000013.11: 34882059...	(771)	ACTGAATTCTGTGTGCAGACTAAATAACTGTCTACA-CAACTTGATTTTAAA-GATTGTCCAACTCTCAA---C
SARS-CoV-2 Reference Genome NC_045512.2	(793)	GCTTAACGGAGGG-GCATACACTCGCTATGTCGATAACAACTTCTGTGGCCCTGATGGCTACCCTCTTGAGTGC

Section 13

		889 … 962
	(889)	889 900 910 920 930 940 950 962
Homo sapiens chromosome 13 NC_000013.11: 34882059...	(840)	ATTGAA-ATCTCTTAATACTATCTG--AAAG--TAGGGACCTTTGTACCTCC--TTGCACTGTTATTGTCAACT
SARS-CoV-2 Reference Genome NC_045512.2	(866)	ATTAAAGACCTTCTAGCACGTGCTGGTAAAGCTTCATGCACTTTGTCCGAACAACTGGACT-TTATTGACA---

Section 14

		963 … 1036
	(963)	963 970 980 990 1000 1010 1020 1036
Homo sapiens chromosome 13 NC_000013.11: 34882059...	(907)	GCTAACACCTGGCCATGGTAGATTAACAAATTAGATTTCCTATTAGCGTCAAACACTAAAGGAACAGAACAGAG
SARS-CoV-2 Reference Genome NC_045512.2	(936)	-CTAAGAGGGGTGTATACTGCTGCCGTGAACATGAGCATGAAATTGCTTGGTACACGGAACGTTCTGAAAAGAG

Section 15

		1037 1050 1060 1070 1080 1090 1100 1110
	(1037)	
Homo sapiens chromosome 13 NC_000013.11: 34882059...	(981)	AATCAATGAGTTTCATAGAG---TAGAAGACAGGCAGGAAGAAAGGTCAGAATGACTGCATGGAGCTCTGAGCA
SARS-CoV-2 Reference Genome NC_045512.2	(1009)	---CTATGAATTGCAGACACCTTTTGAAATTAAATTGGCAAAGAAATTTGACACCTTCAATGGGGAATGTCCAA

Section 16

		1111 1120 1130 1140 1150 1160 1170 1184
	(1111)	
Homo sapiens chromosome 13 NC_000013.11: 34882059...	(1052)	GCTAGGAAGCATCAGAAAAGCACTGGGCCACTAAACTTCTGAGAGTAGGGAAGGCACTAAATAAAAGGTAGGGA
SARS-CoV-2 Reference Genome NC_045512.2	(1080)	ATTTTGTATTTCCCTTAAATTCCATAATCA--AGACTATTCAACCAAGGGTTGAAAAGAAAAAGCTTGATGGCT

Section 17

		1185 1190 1200 1210 1220 1230 1240 1258
	(1185)	
Homo sapiens chromosome 13 NC_000013.11: 34882059...	(1126)	ATGTCAAAAGACACTCTAAAGAGAAATCCACGAGCTTCAC----TGAATCCAC---ATTTTAAATAGCATTATT
SARS-CoV-2 Reference Genome NC_045512.2	(1152)	TTATGGGTAGA-ATTCGATCTGTCTATCCAGTTGCGTCACCAAATGAATGCAACCAAATGTGCCTTTCAACTCT

Section 18

		1259 1270 1280 1290 1300 1310 1320 1332
	(1259)	
Homo sapiens chromosome 13 NC_000013.11: 34882059...	(1193)	TATATTTAGTGA-CATTTTC--AAAATTTAATGTCAGAATCATGACGTTGA-AAATAGATTCTGAAACTAAGGG
SARS-CoV-2 Reference Genome NC_045512.2	(1225)	CATGAAGTGTGATCATTGTGGTGAAACTTCATGGCAGACGGGCGATTTTGTTAAAGCCACTTGCGAATTTTGTG

Section 19

		1333 1340 1350 1360 1370 1380 1390 1406
	(1333)	
Homo sapiens chromosome 13 NC_000013.11: 34882059...	(1263)	GGAAAAATATTCCCTTAATTAAGCTCATGGAGGTAGTTG-GCTGACTTTGGC--AA-TGAAAT--TTAACTTAT
SARS-CoV-2 Reference Genome NC_045512.2	(1299)	GCACTGAGAATT--TGACTAAAGAAGGTGCCACTACTTGTGGTTACTTACCCCAAAATGCTGTTGTTAAAATTT

Section 20

		1407 1420 1430 1440 1450 1460 1470 1480
	(1407)	
Homo sapiens chromosome 13 NC_000013.11: 34882059...	(1331)	TCTTTTTTCCAT-TCTC---TCATACGTACA--CTCTGCTACACCTTACTGAACAAGCTGAAAATTCAATAAAT
SARS-CoV-2 Reference Genome NC_045512.2	(1371)	ATTGTCCAGCATGTCACAATTCAGAAGTAGGACCTGAGCATAGTCTTGCCGAATACCATAATGAATCTGGCTTG

Section 21

		1481 1490 1500 1510 1520 1530 1540 1554
	(1481)	
Homo sapiens chromosome 13 NC_000013.11: 34882059...	(1399)	AAATGCATTCGTTAATGATTTAGTATAACCCGAAAGGAAAGGAAAGCTGCTGGAGCCACTCCACAGGTCTCCCA
SARS-CoV-2 Reference Genome NC_045512.2	(1445)	AAAACCATTC-TTCGTAAGGGTGG-TCGCACTATTGCCTTTGGAGGCTGTGTGTTCTCTTATGTTGGT-TGCCA

Section 22

	(1555)	1555 1560 1570 1580 1590 1600 1610 1628
Homo sapiens chromosome 13 NC_000013.11: 34882059...	(1473)	CTTTGCCAAAAGCGATTACTGTGTGTCAGGGCTGATGGCATGAACCCAGCTGTGGGAGGCCAGGGATGCGGGGA
SARS-CoV-2 Reference Genome NC_045512.2	(1516)	---TAACAAGTGTGCCTATTGGGTTCCACG--TGCTAGCGCTAACATAGGT-TGTAA--CCATACAGGTGTTGT

Section 23

	(1629)	1629 1640 1650 1660 1670 1680 1690 1702
Homo sapiens chromosome 13 NC_000013.11: 34882059...	(1547)	GGCTGCTGCCCCTGCAGCTGTTGCTGTGAGAGATGCTGAAGGCCTT-AGAATCTGCACTCCCACAGTGACTGAG
SARS-CoV-2 Reference Genome NC_045512.2	(1582)	TGGAGAAGGTTCCGAAGGTCTTAATGACAACCTTCTTGAAATACTCCAAAAAGAGAAAGTCAACA-TCAAT-AT

Section 24

	(1703)	1703 1710 1720 1730 1740 1750 1760 1776
Homo sapiens chromosome 13 NC_000013.11: 34882059...	(1620)	AAATGAGGGATTTGAATATGTTTATGTGAAGGG--TTCTTCCTTCTTCCCTTCATTATGCGCCAAAGGATGTT
SARS-CoV-2 Reference Genome NC_045512.2	(1654)	TGTTGGTGACTTTAAACTTAATGAAGAGATCGCCATTATTTTGGCATCTTTTTC-TGCTTCCACAAGTGCTTTT

Section 25

	(1777)	1777 1790 1800 1810 1820 1830 1840 1850
Homo sapiens chromosome 13 NC_000013.11: 34882059...	(1692)	CTAGTAAAACTCAAGACATCAGCTTAGAATATGTTACTGATCATAAATTAAATTGCTCACAGAAGGTGATCAAT
SARS-CoV-2 Reference Genome NC_045512.2	(1727)	GTGGAAACTGTGAAAGGTTTGGATTATAAAGCATTCA--AACAAATTGTTGAATCCTGTGGTAATTTTAA-AGT

Section 26

	(1851)	1851 1860 1870 1880 1890 1900 1910 1924
Homo sapiens chromosome 13 NC_000013.11: 34882059...	(1766)	TTTTAATGTTTACACTCAAGATGTTCTTTCTAATTAACACTTAGCTACATTTGTATTCTGTGTTCACTGTA-GA
SARS-CoV-2 Reference Genome NC_045512.2	(1798)	TACAAAAGGAAAAGCTAAAAAAGGTGCCTGGAAT-ATTGGTGAAC-AGAAATCAATACTGAGTCCTCTTTATGC

Section 27

	(1925)	1925 1930 1940 1950 1960 1970 1980 1998
Homo sapiens chromosome 13 NC_000013.11: 34882059...	(1839)	AAATG--TCTGAGCTTGCA-GAGAACTGCAATAAGTTGTA---AGA---CATCAAACTGC-CATAATTTTCAAA
SARS-CoV-2 Reference Genome NC_045512.2	(1870)	ATTTGCATCAGAGGCTGCTCGTGTTGTACGATCAATTTTCTCCCGCACTCTTGAAACTGCTCAAAATTCTGTGC

Section 28

	(1999)	1999 2010 2020 2030 2040 2050 2060 2072
Homo sapiens chromosome 13 NC_000013.11: 34882059...	(1903)	GT---T-ACACTCTTCAGCTAAAGAAAAA--GGATTTAGCTTCAGAGCACTGACACCATAAAGGGACCTAAGTT
SARS-CoV-2 Reference Genome NC_045512.2	(1944)	GTGTTTTACAGAAGGCCGCTATAACAATACTAGATGGAATTTCACAGTATTCACT-------GAGAC-TCATTG

Section 29

	(2073)	2073 … 2146
Homo sapiens chromosome 13 NC_000013.11: 34882059...	(1971)	ATTCTTCGGAAGTAGTGGGAAGTTACTCTGTATGAATAGATATTTTTAGAAAGTTACTTATATTCCTGATGTGG
SARS-CoV-2 Reference Genome NC_045512.2	(2010)	ATGCT----ATGATGTTCACATCTGATTTGGCTACTAACAATCTAGTTGTAA-TGGCCTACATTACAGGTGGTG

Section 30

	(2147)	2147 … 2220
Homo sapiens chromosome 13 NC_000013.11: 34882059...	(2045)	AGGTGGAAATTACCCATCCATAA-TGCCTATGATGTACTGAGACATATATGTGAAAATTAAAAGTGATTTCCAA
SARS-CoV-2 Reference Genome NC_045512.2	(2079)	TTGTTCAGTTGACTTCGCAGTGGCTAACTAACATCTTTGGCACTGTTTATGAAAAACTCAAACCCGTCCTTGAT

Section 31

	(2221)	2221 … 2294
Homo sapiens chromosome 13 NC_000013.11: 34882059...	(2118)	ATTTAGCAAAAGCTCATTAATTTAGAATCTATTGATTTTTAATTAATGCTAATTGGCCACTTGTGAGCT--ATA
SARS-CoV-2 Reference Genome NC_045512.2	(2153)	TGGCTTGAAGAGAAGTTTAAG---GAAGGTGTAGAGTTTC--TTAGAGACGGTTGGGAAATTGTTAAATTTATC

Section 32

	(2295)	2295 … 2368
Homo sapiens chromosome 13 NC_000013.11: 34882059...	(2190)	TAGAACTATT-TTAAAAAACAGATTATTTACTAGCTGTG---TGACCTTGAGAAATTGACCTAACTCCTCAG--
SARS-CoV-2 Reference Genome NC_045512.2	(2222)	TCAACCTGTGCTTGTGAAATTGTCGGTGGACAAATTGTCACCTGTGCAAAGGAAATTAAGGAGAGTGTTCAGAC

Section 33

	(2369)	2369 … 2442
Homo sapiens chromosome 13 NC_000013.11: 34882059...	(2258)	ATTGAAAGTGGGAGTAATATCTATATGACGGTTTCTATGAGGATTAAATAAC-ATATGTGAAGTACATATCAGT
SARS-CoV-2 Reference Genome NC_045512.2	(2296)	ATTCTTTAAGCTTGTAA-ATAAATTTTTGGCTTTGTGTGCTGACTCTATCATTATTGGTGGAGCTAAACTTAAA

Section 34

	(2443)	2443 … 2516
Homo sapiens chromosome 13 NC_000013.11: 34882059...	(2331)	GCTTCACATATACTATTAGTTTCTTTCCTGTGTATATTACCATATTTACTTGCATTGTGCT---TCTTG-GTAC
SARS-CoV-2 Reference Genome NC_045512.2	(2369)	GCCTTGAATTTAGGTGAAACATTTGTCACGCACTCAAAGGGAT-TGTACAGAAAGTGTGTTAAATCCAGAGAAG

Section 35

	(2517)	2517 … 2590
Homo sapiens chromosome 13 NC_000013.11: 34882059...	(2401)	TTTCTCTT-TAAAAATGGGAATAACAGTTGATCAGGAAATATTTATGTATCTCATTGTGGAAAATACCGCTTCT
SARS-CoV-2 Reference Genome NC_045512.2	(2442)	AAACTGGCCTACTCATGCCTCTAAAAGCCCCAAAAGAAAT---TATCT-TCTTAGAGGGAGAAACACTTCCCAC

Section 36

(2591) 2591 2600 2610 2620 2630 2640 2650 2664
Homo sapiens chromosome 13 NC_000013.11: 34882059... (2474) GGCAGTGTCTGGGGTCCTGCGCCAAGACAGGAACAATTCAGGGAAGCAGGCCAAGGAC-----TTTGACTTACT
SARS-CoV-2 Reference Genome NC_045512.2 (2512) AGAAGTGTT--------------AACAGAGGAAGTTGTCTTGAAAACTGGTGATTTACAACCATTAGAACAAC-

Section 37

(2665) 2665 2670 2680 2690 2700 2710 2720 2738
Homo sapiens chromosome 13 NC_000013.11: 34882059... (2543) CTGCAAAGGAAAAGGTACTATATGTCAGTTATTTATGTATTTATTGGAGACAGGACCCCACTCTGCCACTCAGG
SARS-CoV-2 Reference Genome NC_045512.2 (2571) CTACTAGTGAAGCTGTTGAAGCTC-CATTGGTTGGTACACCAGTTTGTATTA--ACGGGCTTATGTTGCTCGAA

Section 38

(2739) 2739 2750 2760 2770 2780 2790 2800 2812
Homo sapiens chromosome 13 NC_000013.11: 34882059... (2617) CTGGAGTGCAGTGGTGAGATCATAATTCATTGCAACCTCAAACTCCTGGGTCCAAGCAATCCTCCTTCATCAGT
SARS-CoV-2 Reference Genome NC_045512.2 (2642) ATCAAAGACACAGAAAAGTAC-TGTGCCCTTGCA-CCTAATA-TGATGGTAACAAACAATAC--CTTCA-CACT

Section 39

(2813) 2813 2820 2830 2840 2850 2860 2870 2886
Homo sapiens chromosome 13 NC_000013.11: 34882059... (2691) CTCCCAAGTAGCTAGGACTAGAGACATTCTCCATCATGACCAGCTAATTTGTTTTCCCCTTTTCATGCAGAGAC
SARS-CoV-2 Reference Genome NC_045512.2 (2710) CAAAGGCGGTGCACCAACAAAGGTTACTTTTGGTGATGACACTGTGATAGAAGTGCAAGGTTACA---AGAGTG

Section 40

(2887) 2887 2900 2910 2920 2930 2940 2950 2960
Homo sapiens chromosome 13 NC_000013.11: 34882059... (2765) AGGCTCTCACTATGTTGCCCAAGCTG---GTCTTGAACTCTTGGCCTCAAGCTATCCTCCCACCTTGGCCTCTA
SARS-CoV-2 Reference Genome NC_045512.2 (2781) TGAATATCACTTT--TGAACTTGATGAAAGGATTGA--TAAAGTACTTAATGAGAAGTGC----TCTGCCTATA

Section 41

(2961) 2961 2970 2980 2990 3000 3010 3020 3034
Homo sapiens chromosome 13 NC_000013.11: 34882059... (2836) GAGTTGCTGAGATTACAGGCATGAGCCACCACACCCAGACATGAGTCATTTAGAGGAA-AACTAGTGTCCACAG
SARS-CoV-2 Reference Genome NC_045512.2 (2847) CAGTTGAACTCGGTACAGAAGTAAATGAGTTCGCCT-GTGTTGTGGCAGATGCTGTCATAAAAACTTTGCAACC

Section 42

(3035) 3035 3040 3050 3060 3070 3080 3090 3108
Homo sapiens chromosome 13 NC_000013.11: 34882059... (2909) AAAACAGACATTTGCCGGACACAGTGGCTCATGCCTGTAATCCTAACACTTTGGAGTCCAGGCAGGAAGATCG
SARS-CoV-2 Reference Genome NC_045512.2 (2920) AGTATCTGAATTACTTACAC-CACTGGGCATTGATTTAGATGAGTGGAGTATGGC--TACATACTACTTATTTG

Section 43

		3109 … 3120 … 3130 … 3140 … 3150 … 3160 … 3170 … 3182
	(3109)	
Homo sapiens chromosome 13 NC_000013.11: 34882059...	(2983)	CTTGAGCTCAGGAGTTCAAGAATGGCTTGGGCAA--CATGGTGAGACCTCGTGTCTACAGGAAATAAAATTAGC
SARS-CoV-2 Reference Genome NC_045512.2	(2991)	ATGAGTCTGGTGAGTTTAA-ATTGGCTTCACATATGTATTGTTCTTTCTACCCTCCAGATGAGGATGAAGAAGA

Section 44

		3183 … 3190 … 3200 … 3210 … 3220 … 3230 … 3240 … 3256
	(3183)	
Homo sapiens chromosome 13 NC_000013.11: 34882059...	(3055)	CGGGCATGGTGGACCACACCTGTA-GTCCCAGCTACTCGGGAGGCTGAGGCAGGAGGATTGCTTGAGCCCAGGA
SARS-CoV-2 Reference Genome NC_045512.2	(3064)	AGGTGATTGTGAAGAAGAAGAGTTTGAGCCATCAACTCAATATGAGTATGGTACTGAAGATGATTA--CCA--A

Section 45

		3257 … 3270 … 3280 … 3290 … 3300 … 3310 … 3320 … 3330
	(3257)	
Homo sapiens chromosome 13 NC_000013.11: 34882059...	(3128)	GGTAGAGGCT-GCAGTGAGCTGAGATCCCTTCACTGCACTCCAGCCTGGTGAAACCCAGCCTGA------GTGA
SARS-CoV-2 Reference Genome NC_045512.2	(3134)	GGTAAACCTTTGGAATTTGGTGCCACTTCTGC--TGCTCTTCAACCTGAAGAAGAGCAAGAAGAAGATTGGTTA

Section 46

		3331 … 3340 … 3350 … 3360 … 3370 … 3380 … 3390 … 3404
	(3331)	
Homo sapiens chromosome 13 NC_000013.11: 34882059...	(3195)	CAGAGCGAGAGCCTGCCC-CTGCACCCCAAAAAATGGAAAGAAAAGA-AAACAGATATCCATGGAGCATGGGTA
SARS-CoV-2 Reference Genome NC_045512.2	(3206)	GATGATGATAGTCAACAAACTGTTGGTCAACAAGACGGCAGTGAGGACAATCAGACAACTACTATTCAAACAAT

Section 47

		3405 … 3410 … 3420 … 3430 … 3440 … 3450 … 3460 … 3478
	(3405)	
Homo sapiens chromosome 13 NC_000013.11: 34882059...	(3267)	CAATGGCTTTCATGGTGCCATTGCCAATTCTA--TAGAAAAGAAGATTAGAAGAGTACAAATAATTGTTTAAAA
SARS-CoV-2 Reference Genome NC_045512.2	(3280)	TGTTGAGGTTCAACCT-CAATTAGAGATGGAACTTACACCAGTTGTTCAGACTATTGAAGTGAATAGTTTTAGT

Section 48

		3479 … 3490 … 3500 … 3510 … 3520 … 3530 … 3540 … 3552
	(3479)	
Homo sapiens chromosome 13 NC_000013.11: 34882059...	(3339)	TATAATGGACTCAGTAAGGCGCGGCAGATCACGCCTGTAATCCCAACACTTTGG--GAAGCCAAGGTGGGCAGA
SARS-CoV-2 Reference Genome NC_045512.2	(3353)	GGTTATTTAAAACTTACTGA-CAATGTAT-ACATTAAAAATGCAGACATTGTGGAAGAAGCTAAAAAGGTAAAA

Section 49

		3553 … 3560 … 3570 … 3580 … 3590 … 3600 … 3610 … 3626
	(3553)	
Homo sapiens chromosome 13 NC_000013.11: 34882059...	(3411)	TCACCTGAGGTCAGGAGTTCGAGACCAGCCTGGCC---AACATGGCAAAACCCT-------GTCTCTACTAAAA
SARS-CoV-2 Reference Genome NC_045512.2	(3425)	CCAACAGTGGTTGTTAATGC-AGCCAATGTTTACCTTAAACATGGAGGAGGTGTTGCAGGAGCCTTAAATAAGG

```
                                                                     Section 50
                                               (3627) 3627         3640      3650      3660      3670      3680      3690     3700
Homo sapiens chromosome 13 NC_000013.11: 34882059... (3475) ATACAAAATTAGCCGAGCATGGTGGCACA-AGCCTGTAATCCCAGCTACTCGGGAGGCTGAGGTATGAGAATC
  SARS-CoV-2 Reference Genome NC_045512.2 (3498) CTACTAACAAT-GCCATGCAAGTTGAATCTGATGATTACATAGCTACTAATGGACCACTTAAAGTGGGTGGTAG
                                                                     Section 51
                                               (3701) 3701     3710      3720      3730      3740      3750      3760         3774
Homo sapiens chromosome 13 NC_000013.11: 34882059... (3548) ACTTGAACCCAGGAGGCAGAGGTT----A--CAGTGAGTGGA-GATCACACCACTGCACTCCAGCCTGGGCAAT
  SARS-CoV-2 Reference Genome NC_045512.2 (3571) TTGTGTTTTAAGCGGACACAATCTTGCTAAACACTGTCTTCATGTTGTCGGCCCAAATGTTAACAAAGGTGAAG
                                                                     Section 52
                                               (3775) 3775 3780      3790      3800      3810      3820      3830               3848
Homo sapiens chromosome 13 NC_000013.11: 34882059... (3615) GGAGTAAGACTTCATTGAAAGAAAGAGAGAGAGAGAGAGGAAGGAAGGAAGGGAGGGAGGGAGGGAGGGAAGGG
  SARS-CoV-2 Reference Genome NC_045512.2 (3645) ACATTCA-ACTTC-TTAAGAGTGCTTATGAAAATTTTAATCAGCACGAAGTTCTACTTGCACCATTATTATCAG
                                                                     Section 53
                                               (3849) 3849        3860      3870      3880      3890      3900      3910     3922
Homo sapiens chromosome 13 NC_000013.11: 34882059... (3689) AAGGAA---GGGGAAAAGAAAAAAGAAAGAAAGAGAAAGAAAGAAAGAAAGAAAGAAAGAAAGAAAGAA-AGAA
  SARS-CoV-2 Reference Genome NC_045512.2 (3717) CTGGTATTTTTGGTGCTGACCCTATACATTCTTTAAGAGTTTGTGT-AGATACTGTTCGCACAAATGTCTACTT
                                                                     Section 54
                                               (3923) 3923   3930      3940      3950      3960      3970      3980          3996
Homo sapiens chromosome 13 NC_000013.11: 34882059... (3759) AGAAAGAAAGAAAGAAAGAAAGAAAGAGAGAGA-GAGGAAGGAAGGAAGGGAAGGAAGGG-GAAAAGAAAAAGA
  SARS-CoV-2 Reference Genome NC_045512.2 (3790) AGCTGTCTTTGATAAAAATCTCTATGACAAACTTGTTTCAAGCTTTTTGGAAATGAAGAGTGAAAAGCAAGTTG
                                                                     Section 55
                                               (3997) 3997         4010      4020      4030      4040      4050      4060     4070
Homo sapiens chromosome 13 NC_000013.11: 34882059... (3831) AAGAAGAAAGAGAAAAAAGGAAAGAGAGAAAGAAAGGAAAGAAAGAAAGAAAGAGAAAGAAAGAAAGAAAGAAA
  SARS-CoV-2 Reference Genome NC_045512.2 (3864) AACAAAAGATCGCTGA---GATTCCTAAAGAGGAAGTTAAGCCATTTATAACTGAAAGTAAACCTTCAGTTGAA
                                                                     Section 56
                                               (4071) 4071     4080      4090      4100      4110      4120      4130         4144
Homo sapiens chromosome 13 NC_000013.11: 34882059... (3905) GAAAGAAAGAAAGAAAGAAAGAACGAACGAAAGAAAGAAAGAAAGAAAGAACGAACGAAAGAAAGAAAGAAAGA
  SARS-CoV-2 Reference Genome NC_045512.2 (3935) CAGAGAAAACAAGATGATAAGAA--AATCAAAGCTTGTGTTGAAGAAGTTAC-AAC--AACTCTGGAAGAAACT
```

Section 57

```
                                                      (4145) 4145 4150      4160      4170      4180      4190      4200            4218
Homo sapiens chromosome 13 NC_000013.11: 34882059...  (3979) AAGAAAGAAAGAAAGAAAGAGAAAGGAAAGAAAAGTGAGGGAGGGAGGGAGGCAGGAAGGAAGGAAGGAAGAGA
SARS-CoV-2 Reference Genome NC_045512.2               (4004) AAGTTCCTCACAGAAAACTTGTTACTTTATATT-GACATTAATGGCAATCTTCATCCAG--ATTCTGCCACTCT
```

Section 58

```
                                                      (4219) 4219       4230      4240      4250      4260      4270      4280         4292
Homo sapiens chromosome 13 NC_000013.11: 34882059...  (4053) AGTAAATAAAATAGCATGGACTCTTCTCATCCTTATATTCTCTCTAAAT--TGCATTTTTGTTTTCTTTTGTGT
SARS-CoV-2 Reference Genome NC_045512.2               (4075) TGTTAGTGACATTGACATCACT-TTCTTAAAGAAAGATGCTCCATATATAGTGGGTGATGTTGTTCAAGAGGGT
```

Section 59

```
                                                      (4293) 4293   4300      4310      4320      4330      4340      4350            4366
Homo sapiens chromosome 13 NC_000013.11: 34882059...  (4125) TTTTAAGGATCTGCAAGAATATCT-CTAACATGGAGATTTCCAAGTTTTCTTAGTTTGATGATAGAGGAAATTT
SARS-CoV-2 Reference Genome NC_045512.2               (4148) GTTTTAACTGCTGTGGTTATACCTACTAAAAAGGCTGGTGGCAC-------TACTGAAATGCTAGCGAAA----
```

Section 60

```
                                                      (4367) 4367         4380      4390      4400      4410      4420      4430      4440
Homo sapiens chromosome 13 NC_000013.11: 34882059...  (4198) CAGGCTTAGACGATGATAGTAACAAAGGAGAA--AAATTGGCACTAGGATAGGTGGGTGGAGCCAAAACAGATT
SARS-CoV-2 Reference Genome NC_045512.2               (4211) ---GCTTTGA-GA--AAAGTGCCAACAGACAATTATATAACCACT----TACCCGGGTCAGGGTTTAAATGGTT
```

Section 61

```
                                                      (4441) 4441     4450      4460      4470      4480      4490      4500          4514
Homo sapiens chromosome 13 NC_000013.11: 34882059...  (4270) TTACAGACTGATGAAAGTATGATTTTGGCAAAATGGACAGAAATTTTTGGCTCTGTATATGCT-CAAAATAGCT
SARS-CoV-2 Reference Genome NC_045512.2               (4275) ACACTGTA-GAGGAGGCAAAGACAGTGCTTAAAAAGTGTAAAAGT----GCCTTTTACATTCTACCATCTATTA
```

Section 62

```
                                                      (4515) 4515 4520      4530      4540      4550      4560      4570            4588
Homo sapiens chromosome 13 NC_000013.11: 34882059...  (4343) GTTGGGATGATAAAATATTAAATCAT--AAATGTATAAT---TTTTACTAGTGGTGCACTAAC-TTCCTAGGAA
SARS-CoV-2 Reference Genome NC_045512.2               (4344) TCTCTAATGAGAAGCAAGAAATTCTTGGAACTGTTTCTTGGAATTTGCGAGAAATGCTTGCACATGCAGAAGAA
```

Section 63

```
                                                      (4589) 4589       4600      4610      4620      4630      4640      4650         4662
Homo sapiens chromosome 13 NC_000013.11: 34882059...  (4411) GATCTCAAGACATGACTCTGAAATTAAGTATCCTAAAGTGTGTTTCTAGAGCTATAAATAACCTCCTTCTATGT
SARS-CoV-2 Reference Genome NC_045512.2               (4418) ACACGCAAATTAATGC-CTGTCTGTGTGGAAACTAAAG-------CCATAGTTTCAACTATACAGCGTAAATAT
```

```
                                                                 Section 64
                                                (4663) 4663   4670      4680      4690      4700      4710      4720          4736
Homo sapiens chromosome 13 NC_000013.11: 34882059... (4485) GTAATTCCAATAATCCATGCAAATGTTGCTTTCAGATATCCATGGAAATTTTTGGTACATGACTAATTAAAATT
     SARS-CoV-2 Reference Genome NC_045512.2 (4484) AAGGGTATTAAAATACAAGAGGGTGTGG-TTGATTATGGTGCTAGATTTTACTTTTACACCAGTAA----AACA
                                                                 Section 65
                                                (4737) 4737         4750      4760      4770      4780      4790      4800    4810
Homo sapiens chromosome 13 NC_000013.11: 34882059... (4559) ATTGTTTAAGCCCATGGCAAGTCACTGGT-GCACTAAACAAGACAAT-GAGAGTGTGCATGATTGTCATATTTA
     SARS-CoV-2 Reference Genome NC_045512.2 (4553) ACTGTAGCGTCACTTATCAACACACTTAACGATCTAAATGAAACTCTTGTTACAATGCCAC-TTGGC-TATGTA
                                                                 Section 66
                                                (4811) 4811     4820      4830      4840      4850      4860      4870          4884
Homo sapiens chromosome 13 NC_000013.11: 34882059... (4631) AACCAAAGCTGTGAGAGAGCTTCCTGGTCCACTGTTTCTGGGATATCTATTATAATTTGCCACACGTTGAACTA
     SARS-CoV-2 Reference Genome NC_045512.2 (4625) ACACATGGCT-TAAATTTGGAAGAAGCTGCTCGGTATATGAGATCTCTCAAAGTGCCAGCTACAGTTTCTGTTT
                                                                 Section 67
                                                (4885) 4885 4890      4900      4910      4920      4930      4940          4958
Homo sapiens chromosome 13 NC_000013.11: 34882059... (4705) TAAAAAATGATGTGTCTTTAGCTAACTCAACTTTAATTTTCTTGTATCTTACCCAATTTCCCAA--ATATTTTC
     SARS-CoV-2 Reference Genome NC_045512.2 (4698) CTTCACCTGATGCTGTTACAGCGTA-TAATGGTTATCTTACTTCT-TCTTCTAAAACACCTGAAGAACATTTTA
                                                                 Section 68
                                                (4959) 4959        4970      4980      4990      5000      5010      5020     5032
Homo sapiens chromosome 13 NC_000013.11: 34882059... (4777) T---AAGTATTGCAAT--GTGA-----ATAAATTTTCTAACAGTAAAACAGGATATAGTTTTATGGAGTTTTAT
     SARS-CoV-2 Reference Genome NC_045512.2 (4770) TTGAAACCATCTCACTTGCTGGTTCCTATAAAGATTGGTCCTATTCTGGACAATCTACACAACTAGGTATAGAA
                                                                 Section 69
                                                (5033) 5033   5040      5050      5060      5070      5080      5090          5106
Homo sapiens chromosome 13 NC_000013.11: 34882059... (4841) TTTCTGAAGTAGATAACTCAAGAATGTAAGGAAGTAAAGCAAAAAAAGAGACTCAAG--TGATTTTTTTGTTA
     SARS-CoV-2 Reference Genome NC_045512.2 (4844) TTTCTTAAG-AGAGGTGATAAAAGTGTATATTACACTAGTAATCCTACCACATTCCACCTAGATGGTGAAGTTA
                                                                 Section 70
                                                (5107) 5107         5120      5130      5140      5150      5160      5170    5180
Homo sapiens chromosome 13 NC_000013.11: 34882059... (4913) GCCTGATAATTAGTTTTTGTTTTCTTTTTTAATCTGAAGATCTGTGGAGGA--AATGATTTTACTTTATAAATA
     SARS-CoV-2 Reference Genome NC_045512.2 (4917) TCACCTTTGACAATCTTAAGACACTTCTTTCTTTGAGAGAAGTGAGGACTATTAAGGTGTTTAC---AACAGTA
```

Section 71

(5181) 5181 5190 5200 5210 5220 5230 5240 5254

Homo sapiens chromosome 13 NC_000013.11: 34882059... (4985) AGAAACTTGGACTTATATCAGCAAG--------AATTCTATTGGCTGGGCTCACACAATCTGGATTAAC-----

SARS-CoV-2 Reference Genome NC_045512.2 (4988) GACAACATTAACCTCCACACGCAAGTTGTGGACATGTCAATGACATATGGACA-ACAGTTTGGTCCAACTTATT

Section 72

(5255) 5255 5260 5270 5280 5290 5300 5310 5328

Homo sapiens chromosome 13 NC_000013.11: 34882059... (5046) ---AGGGAACACTCCTTGAAACTTTTTATTCTTTTTATTTTGTTAAAC-TATTTCAGATTTACTTTTTATATGA

SARS-CoV-2 Reference Genome NC_045512.2 (5061) TGGATGGAGCTGATGTTACTAAAATAAAACCTCATAATTCACATGAAGGTAAAACAT-TTTATGTTTTACCTAA

Section 73

(5329) 5329 5340 5350 5360 5370 5380 5390 5402

Homo sapiens chromosome 13 NC_000013.11: 34882059... (5116) CACTTGAGTCACTATGAGTCAAA-CTGGGTAGTGATATCATCTAACCTCTGGAAGGGTAAATAGTTCAAAAAAT

SARS-CoV-2 Reference Genome NC_045512.2 (5134) TGAT-GACACTCTACGTGTTGAGGCTTTTGAGTACTACCACACAACTGATCCTAGTTTTC-TGGGTAGGTACAT

Section 74

(5403) 5403 5410 5420 5430 5440 5450 5460 5476

Homo sapiens chromosome 13 NC_000013.11: 34882059... (5189) AGTAGCTCTGATCAATGCTTCAAAATTCTGTTGCACATTAGATTCAGCAG--AGTACCATTCAAAAA-CATATA

SARS-CoV-2 Reference Genome NC_045512.2 (5206) GTCAGCATTAAATCACACTAAAAAGTGGAAATACCCACAAGTTAATGGTTTAACTTCTATTAAATGGGCAGATA

Section 75

(5477) 5477 5490 5500 5510 5520 5530 5540 5550

Homo sapiens chromosome 13 NC_000013.11: 34882059... (5260) ATACCCAGGTCCCAGGTGGGTCATGTCTTAGTATTAGTTCTTTAAAAACGCTACTCAGTTGATTTTAATGTGCA

SARS-CoV-2 Reference Genome NC_045512.2 (5280) ACAAC----TGTTATCTTGCCACTGCATTGTTAACACTCCAACAAATAGAGTTGAAGTTTAATCCACCTGCTCT

Section 76

(5551) 5551 5560 5570 5580 5590 5600 5610 5624

Homo sapiens chromosome 13 NC_000013.11: 34882059... (5334) GATAGA-----ATTA-AAAACTACTGCTTCAGATAAAGCATTTTTTTTTTTTTACATAGAAAAGAGCACTTCAA

SARS-CoV-2 Reference Genome NC_045512.2 (5350) ACAAGATGCTTATTACAGAGCAAGGGCTGGTGAAGCTGC--TAACTTTTGTGCACTTATCTTAGCCTACTGTAA

Section 77

(5625) 5625 5630 5640 5650 5660 5670 5680 5698

Homo sapiens chromosome 13 NC_000013.11: 34882059... (5402) AGGGTTATGTCCATCACAGTTCCCAGAATATGGAGAATGATCAA--TAAAAGTTAGAACTTCAACTATTTGAAT

SARS-CoV-2 Reference Genome NC_045512.2 (5422) TAAGACA-GTAGGTGA--GTTAGGTGATGTTAGAGAAACAATGAGTTACTTGTTTCAACATGCCAATTTAGATT

Section 78

		5699 … 5772
Homo sapiens chromosome 13 NC_000013.11: 34882059...	(5474)	C---CATCAGG---TTAAACTTTTTCTGTAA---T-GGGTCTGACAGTAAATA--CCTTA--GGTGTAGAAGGT
SARS-CoV-2 Reference Genome NC_045512.2	(5493)	CTTGCAAAAGAGTCTTGAACGTGGTGTGTAAAACTTGTGGACAACAGCAGACAACCCTTAAGGGTGTAGAAGCT

Section 79

		5773 … 5846
Homo sapiens chromosome 13 NC_000013.11: 34882059...	(5534)	TATAGGGTGCCTGTCGCAGCTACT------CAACTCTGCT--GGTATTCA---ATCCTATCATTGTAGCACAAA
SARS-CoV-2 Reference Genome NC_045512.2	(5567)	GTTATGTACATGGGCACACTTTCTTATGAACAATTTAAGAAAGGTGTTCAGATACCTTGTACGTGTGGTAAACA

Section 80

		5847 … 5920
Homo sapiens chromosome 13 NC_000013.11: 34882059...	(5597)	AGCAGCCACAGA-------CAATATGAACCAAATAT-GTCTAGCTGT--GCTCCAATAAAACTTTATTTACCAA
SARS-CoV-2 Reference Genome NC_045512.2	(5641)	AGCTACAAAATATCTAGTACAACAGGAGTCACCTTTTGTTATGATGTCAGCACCACCTGCTCAGTATGAACTTA

Section 81

		5921 … 5994
Homo sapiens chromosome 13 NC_000013.11: 34882059...	(5661)	AACAGGCAACATGTTGGATTTGGGCCACTGGTCATAGT-----TTGCCAACCCTGGCAATACATCACCTCTCAA
SARS-CoV-2 Reference Genome NC_045512.2	(5715)	AGCATGGTACATTT---ACTTGTGCTAGTGAGTACACTGGTAATTACCAGTGTGGTCACTATAA-ACATATAAC

Section 82

		5995 … 6068
Homo sapiens chromosome 13 NC_000013.11: 34882059...	(5730)	TTTT--TGCAACTTTTTGCAATTTTATCACTATAGCTTTATTTT-ATAGT--TTAAAATATTATTTGTTTATAA
SARS-CoV-2 Reference Genome NC_045512.2	(5785)	TTCTAAAGAAACTTTGT---ATTGCATAGACGGTGCTTTACTTACAAAGTCCTCAGAATACAAAGGTCCTATTA

Section 83

		6069 … 6142
Homo sapiens chromosome 13 NC_000013.11: 34882059...	(5799)	A--ATATTGCCATGGATTAAACCACTAACT-AGCTATTATAT-----GTGA-TTATTAAAGAGTTAGTGTAGCC
SARS-CoV-2 Reference Genome NC_045512.2	(5856)	CGGATGTTTTCTACAAAGAAAACAGTTACACAACAACCATAAAACCAGTTACTTATAAATTGGATGGTGTTGTT

Section 84

		6143 … 6216
Homo sapiens chromosome 13 NC_000013.11: 34882059...	(5864)	---AGACACGTTGGCTCACGGCTGTAATCCCAGCACTTTAGGAGGCTGAGGCGGGTGGATCACCTGAGGTCAGG
SARS-CoV-2 Reference Genome NC_045512.2	(5930)	TGTACAGAAATTGACCCTAAGTTGGA---CAATTATTATAAGAAA--GACAATTCTTATTTCACAGAGCA-ACC

```
                                                                      Section 85
                                                    (6217) 6217         6230      6240      6250      6260      6270      6280    6290
Homo sapiens chromosome 13 NC_000013.11: 34882059... (5935) AGTTCAAGACCAGCCTGGCCAAC-ATGGTGAAACCCCATCTTTACTAAAAATACAAAAATTAGCTGGACGTGGT
          SARS-CoV-2 Reference Genome NC_045512.2 (5998) AATTGATCTTGTACCAAACCAACCATATCCAAACGCAAGCTT--CGATAATTTTAAGTTTGTATGTGATAATAT
                                                                      Section 86
                                                    (6291) 6291     6300      6310      6320      6330      6340      6350          6364
Homo sapiens chromosome 13 NC_000013.11: 34882059... (6008) GGCATGCACCTGTAATTTCAGTTATTTGGGAGGCTGAGGCAGGAGAATCGCTTGAACCCAGGAGACAGAGGTTG
          SARS-CoV-2 Reference Genome NC_045512.2 (6070) CAAATTTGCTGATGATTTAAACCAGTTAACTGGTTAT---AAGAAACCTGCTTCAAGAGAGCTTAAAG---TTA
                                                                      Section 87
                                                    (6365) 6365 6370      6380      6390      6400      6410      6420              6438
Homo sapiens chromosome 13 NC_000013.11: 34882059... (6082) CAGTGAGCCGAGATCACACCACTGCACTCTAGCC---TGGGCAACAGAGCAAGACTCCATCTCAAAAAAAAATA
          SARS-CoV-2 Reference Genome NC_045512.2 (6138) CATTTTTCCCTGACTTAAATGGTGATGTGGTGGCTATTGATTATAAACACTACACACCCTCTTTTAAGAAAGGA
                                                                      Section 88
                                                    (6439) 6439       6450      6460      6470      6480      6490      6500        6512
Homo sapiens chromosome 13 NC_000013.11: 34882059... (6153) AGTAAATAAAATTTAAAAAATAAAGAGTTAG------TATAAATAAAAATGA-----CCAAATAATAACCTCTG
          SARS-CoV-2 Reference Genome NC_045512.2 (6212) GCTAAATTG--TTACATAAACCTATTGTTTGGCATGTTAACAATGCAACTAATAAAGCCACGTATAAACCAAAT
                                                                      Section 89
                                                    (6513) 6513   6520      6530      6540      6550      6560      6570            6586
Homo sapiens chromosome 13 NC_000013.11: 34882059... (6216) CCCTTTTG-ATGAGATTACCATCTCTAAAGATACTGGTTTTTAGAG--AATTTAATTTA---ACTTTATTTAGC
          SARS-CoV-2 Reference Genome NC_045512.2 (6284) ACCTGGTGTATACGTTGTCTTTGGAGCACAAAACCAGTTGAAACATCAAATTCGTTTGATGTACTGAAGTCAGA
                                                                      Section 90
                                                    (6587) 6587         6600      6610      6620      6630      6640      6650    6660
Homo sapiens chromosome 13 NC_000013.11: 34882059... (6284) CTA-GCTAATATAATTTATAAATTATACTTACTGGGATATGAAGTGATTAAATAGAAATTTCCTAGTGCTACTA
          SARS-CoV-2 Reference Genome NC_045512.2 (6358) GGACGCGCAGGGAATGGATAATCT-TGCCTGCGAAGATCTAAAACCAGTCTCTGAAGAAGTAGTGGAA--AATC
                                                                      Section 91
                                                    (6661) 6661     6670      6680      6690      6700      6710      6720          6734
Homo sapiens chromosome 13 NC_000013.11: 34882059... (6357) CTTCTAGAGGACAAGAGCTCTTTAATTGTATTTCATAGAAAGTACTGTAGTCAAGAGTAGAAAATATGAAATAT
          SARS-CoV-2 Reference Genome NC_045512.2 (6429) CTACCATACAGAAAGACGTTCTTGAGTGTAATG--TGAAAACTACCGAAGTTGT-AGGAGACATTATACTTAAA
```

Section 92

```
                                                    (6735) 6735 6740      6750      6760      6770      6780      6790              6808
Homo sapiens chromosome 13 NC_000013.11: 34882059... (6431) ACAT-AAGGGCCACTTTCCTCTCTAGGAAAGACATTG-CCAATCAATTATACCTGATCCTGGATGT-GATCTCC
   SARS-CoV-2 Reference Genome NC_045512.2          (6500) CCAGCAAATAATAGTTTAAAAATTACAGAAGAGGTTGGCCACACAGATCTAATGGCTGCTT-ATGTAGACAATT
```

Section 93

```
                                                    (6809) 6809       6820      6830      6840      6850      6860      6870          6882
Homo sapiens chromosome 13 NC_000013.11: 34882059... (6502) CAATACTTCTTACTATTACTCC--AGGCAGTCACCACAAATCAAAGTTTGCAGACAAGATGAAATC-TATGTGA
   SARS-CoV-2 Reference Genome NC_045512.2          (6573) CTAGTCTTACTATTAAGAAACCTAATGAATTATCTAGAGTATTAGGTTTGAAAACCCTTGCTACTCATGGTTTA
```

Section 94

```
                                                    (6883) 6883   6890      6900      6910      6920      6930      6940              6956
Homo sapiens chromosome 13 NC_000013.11: 34882059... (6573) CATTCTTAACACAAGAGGAGATGTGTTTCCAGCTCCAACAACGTGCAGGAAATTATTCTTTGCTCCAAGGTAT
   SARS-CoV-2 Reference Genome NC_045512.2          (6647) GCTGCTGTTAATAGTGTCCCTTGGGATACTATAGCTAATTATGCTAAGCC---TTTTCTTAAC---AAAGTTGT
```

Section 95

```
                                                    (6957) 6957         6970      6980      6990      7000      7010      7020    7030
Homo sapiens chromosome 13 NC_000013.11: 34882059... (6647) AAATGCCCTTCCCACCACCACACTGA-AGAGTGTGCTGATCTTACAG--AGGAAGCATA-GAATCACATGTTAA
   SARS-CoV-2 Reference Genome NC_045512.2          (6715) TAGTACAACT---ACTAACATAGTTACACGGTGTTTAAACCGTGTTTGTACTAATTATATGCCTTATTTCTTTA
```

Section 96

```
                                                    (7031) 7031     7040      7050      7060      7070      7080      7090          7104
Homo sapiens chromosome 13 NC_000013.11: 34882059... (6717) --TGTTTGTTAGAGATGCAGAAAATTGCAGCAAGT-CACATCTTATC--CAAAGTAGGTAGTCAGCATTTT---
   SARS-CoV-2 Reference Genome NC_045512.2          (6786) CTTTATTGCTACAATTGTGTACTTTTACTAGAAGTACAAATTCTAGAATTAAAGCATCTATGCCGACTACTATA
```

Section 97

```
                                                    (7105) 7105 7110      7120      7130      7140      7150      7160              7178
Homo sapiens chromosome 13 NC_000013.11: 34882059... (6783) GCATAGCATGCTTGGAAGAAAG-----AAAAGATTCCCATAGAAATCAATTGATTAAAA-AAAAAATCTA---T
   SARS-CoV-2 Reference Genome NC_045512.2          (6860) GCAAAGAATACTGTTAAGAGTGTCGGTAAATTTTTGTCTAGAGGCTTCATTTAATTATTTGAAGTCACCTAATTT
```

Section 98

```
                                                    (7179) 7179       7190      7200      7210      7220      7230      7240          7252
Homo sapiens chromosome 13 NC_000013.11: 34882059... (6848) AGCTAAAGGCTGTAATTTAAACTTAAGCAAAAGGAAATAGTAAAAACTTTTCTA--TAATTTACATAAAATAAA
   SARS-CoV-2 Reference Genome NC_045512.2          (6934) TTCTAAA--CTGATAAATATTATAATTTGGTTTTTACTATTAAGTGTTTGCCTAGGTTCTTTAATCTACTCAAC
```

Section 99

```
                                                     (7253) 7253      7260            7270            7280            7290            7300            7310                7326
Homo sapiens chromosome 13 NC 000013.11: 34882059... (6920) CTCTGAAGCAGAGGTTTTGGGGC---AGCTAGTATGGGACTAGAGAAGTGTGGGGCACACAAAAGGAGATTTAG
      SARS-CoV-2 Reference Genome NC_045512.2        (7006) CGCTGCTTTAGGTGTTTTAATGTCTAATTTAGGCATGCCTTCTTACTGTACTGGTTACAGAGAAGGCTATTTGA
```

Section 100

```
                                                     (7327) 7327         7340            7350            7360            7370            7380            7390        7400
Homo sapiens chromosome 13 NC 000013.11: 34882059... (6991) ATTGAACCGGAACTTTTTCCTGTCTCATTCTTCATGACTGGT-----AGCTGGCACTGGCTTTAACCGAGAAAT
      SARS-CoV-2 Reference Genome NC_045512.2        (7080) ACTCTACT--AA--TGTCACTATTGCAACCTACTGTACTGGTTCTATACCTTGTAGTG--TTTGTCTTAGTGGT
```

Section 101

```
                                                     (7401) 7401     7410            7420            7430            7440            7450            7460              7474
Homo sapiens chromosome 13 NC 000013.11: 34882059... (7060) CTCAATTCTCCTCTGGCTTCTCATCCTCCAG----TGTACTAGATCAGCTTCCTTATATGAGAGTGGTGTTGGG
      SARS-CoV-2 Reference Genome NC_045512.2        (7148) TTAGATTCTTTAGACACCTATCCTTCTTTAGAAACTATACAAATTACCATTTCATCTTTTAAATGGGATTTAAC
```

Section 102

```
                                                     (7475) 7475 7480            7490            7500            7510            7520            7530                  7548
Homo sapiens chromosome 13 NC 000013.11: 34882059... (7130) GGACATTC-CAAGAAAGCAAAGGCCTTTGAGGCCTAGATTCCTGAACTTACACAACATCAGTTCAATTACATAA
      SARS-CoV-2 Reference Genome NC_045512.2        (7222) TGCTTTTGGCTTAGTTGCAGAGTGGTTTTTGGCATATATTCTT-----TTCACTAGGTTTTTCTATGTACTTGG
```

Section 103

```
                                                     (7549) 7549       7560            7570            7580            7590            7600            7610          7622
Homo sapiens chromosome 13 NC 000013.11: 34882059... (7203) TCTATTCGAAGCAAG-ATTCAAGAGGTAGGAAAAGAGACTCCACCTCTTGGTAGGAGGCACTATAAAATACTGG
      SARS-CoV-2 Reference Genome NC_045512.2        (7291) ---ATTGGCTGCAATCATGCAATTGTTTTTCAGCTATTTTGCAGTACATTTTATTAGTAATTCTTGGCTTATGT
```

Section 104

```
                                                     (7623) 7623      7630            7640            7650            7660            7670            7680                7696
Homo sapiens chromosome 13 NC 000013.11: 34882059... (7276) AGTTATGTTTTTCAATCTGCCACTCACAGTGGAGCTGT-AGTCTTACTTAACACATTCTTCTGATT--CTTCAT
      SARS-CoV-2 Reference Genome NC_045512.2        (7362) GGTTAATAATT--AATCTTGTACAAATGGCCCCGATTTCAGCTATGGTTAGAATGTACATCTTCTTTGCATCAT
```

Section 105

```
                                                     (7697) 7697         7710            7720            7730            7740            7750            7760        7770
Homo sapiens chromosome 13 NC 000013.11: 34882059... (7347) AAAGTTCTGAATTAAATTATTCTGATTATTATGTATGGACAGCAGGA------CCAAATGGGGCCTCTCTGACA
      SARS-CoV-2 Reference Genome NC_045512.2        (7434) TTTATTATGTATGGAAAAGTTATGTGCATGTTGTA--GACGGTTGTAATTCATCAACTTGTATGATGTGTTACA
```

Section 106

```
                                                      (7771) 7771     7780           7790           7800           7810           7820           7830        7844
Homo sapiens chromosome 13 NC_000013.11: 34882059...  (7415) A-CATCTTA--------AGATTCTGATGCTCAGAGCAGCAAAAATTGTCTAATAAAATTCCATCAGAAAGAAGC
SARS-CoV-2 Reference Genome NC_045512.2               (7506) AACGTAATAGAGCAACAAGAGTCGAATGTACA-ACTATTGTTAATGGTGTTAGAAGGTCCTTTTATGTCTATGC
```

Section 107

```
                                                      (7845) 7845 7850           7860           7870           7880           7890           7900        7918
Homo sapiens chromosome 13 NC_000013.11: 34882059...  (7480) ATATTTCTGTCTAAGGATGCTACATCTTACATA-TGTAAGATCTTTCATGTTTGATTTCAATCTTTCATGTTTG
SARS-CoV-2 Reference Genome NC_045512.2               (7579) TAATGGAGGTA-AAGGCTTTTGCAAACTACACAATTGGAATTGTGTTAATTGTGATA-CATTCTGTGCTGGTAG
```

Section 108

```
                                                      (7919) 7919       7930           7940           7950           7960           7970           7980   7992
Homo sapiens chromosome 13 NC_000013.11: 34882059...  (7553) ---ATTTCATAGAATCTTAATATTAAGAAAAAG----AAAGAAACTT--AAATATCATCCAATTCCAC-GTTAA
SARS-CoV-2 Reference Genome NC_045512.2               (7651) TACATTTATTAG--TGATGAAGTTGCGAGAGACTTGTCACTACAGTTTAAAAGACCAATAAATCCTACTGACCA
```

Section 109

```
                                                      (7993) 7993   8000           8010           8020           8030           8040           8050        8066
Homo sapiens chromosome 13 NC_000013.11: 34882059...  (7617) G--------CAAAATTGCCAAT-TCACAAAAAATATACAACTTTTATTTCTTTAAAAGAATAGATACAGGCCCA
SARS-CoV-2 Reference Genome NC_045512.2               (7723) GTCTTCTTACATCGTTGATAGTGTTACAGTGAAGAATGGTTCCATCCATCTTTACTTTGATAAAG-CTGGTCAA
```

Section 110

```
                                                      (8067) 8067         8080           8090           8100           8110           8120           8130   8140
Homo sapiens chromosome 13 NC_000013.11: 34882059...  (7682) CGGAGGG--GAATAATATTTT-TCTACAATTATGCTACCTT------CTTTATTGAATTGTACAATACAGTTAT
SARS-CoV-2 Reference Genome NC_045512.2               (7796) AAGACTTATGAAAGACATTCTCTCTCTCATTTTGTTAACTTAGACAACCTGAGAGCTAATAACACTAAAGGTTC
```

Section 111

```
                                                      (8141) 8141     8150           8160           8170           8180           8190           8200        8214
Homo sapiens chromosome 13 NC_000013.11: 34882059...  (7747) TTTAGTAGCTCAAGGTTTAATGTCTGGTTATACTTCAGGAGAAGCAGCCAGGTGGGCAACATGACCAG----TT
SARS-CoV-2 Reference Genome NC_045512.2               (7870) ATTGCCTATTAATGTTATAGTTTTTGATGGTAAATCAAAATGTGAAGAATCATCTGCAAAATCAGCGTCTGTTT
```

Section 112

```
                                                      (8215) 8215 8220           8230           8240           8250           8260           8270        8288
Homo sapiens chromosome 13 NC_000013.11: 34882059...  (7817) CCTTGATTTTCCAGGTAAGA-AACAGAGACAGGGA--AACTAAGG-----GTCT-TGCTCAAGGTTACAGAACT
SARS-CoV-2 Reference Genome NC_045512.2               (7944) ACTACAGTCAGCTTATGTGTCAACCTATACTGTTACTAGATCAGGCATTAGTGTCTGATGTTGGTGATAGTGCG
```

		Section 113
	(8289)	8289 8300 8310 8320 8330 8340 8350 8362
Homo sapiens chromosome 13 NC_000013.11: 34882059...	(7882)	AAC--TACTGGCAAAAT--T-GATGCCACAAGACAACACCCTTGC---------TCCCAAAACAATGTTTC--C
SARS-CoV-2 Reference Genome NC_045512.2	(8018)	GAAGTTGCAGTTAAAATGTTTGATGCTT-ACGTTAATACGTTTTCATCAACTTTTAACGTACCAATGGAAAAAC
		Section 114
	(8363)	8363 8370 8380 8390 8400 8410 8420 8436
Homo sapiens chromosome 13 NC_000013.11: 34882059...	(7940)	TCATTGCTCCATTAGGA----CAGTTACTCTAATT-CAGGAAAGTCATTATTCTCTAAGCCTTA-CTGATCTG
SARS-CoV-2 Reference Genome NC_045512.2	(8091)	TCAAAACACTAGTTGCAACTGCAGAAGCTGAACTTGCAAAGAATGTGTCCTTAGACAATGTCTTATCTACTTTT
		Section 115
	(8437)	8437 8450 8460 8470 8480 8490 8500 8510
Homo sapiens chromosome 13 NC_000013.11: 34882059...	(8008)	GTGAGACCAGAG-GTCAGCAGCCTGTTTCTGGAAAGGGCCACATAAAAATTACTTTAGATTTTGCAAGCCTGGA
SARS-CoV-2 Reference Genome NC_045512.2	(8165)	ATTTCAGCAGCTCGGCAAGGGTTTGTTGATTCAGATGTAGAAACTAAAGATGTTGTTGAATGTCTTAAATTGTC
		Section 116
	(8511)	8511 8520 8530 8540 8550 8560 8570 8584
Homo sapiens chromosome 13 NC_000013.11: 34882059...	(8081)	A----AGGGCCACATAAAAATTACTTTAGATT-TTGTAAGCCATACAGTCTCT---G-------GTTGCCACTA
SARS-CoV-2 Reference Genome NC_045512.2	(8239)	ACATCAATCTGACATAGAAGTTACTGGCGATAGTTGTAATAACTATATGCTCACCTATAACAAAGTTGAAAACA
		Section 117
	(8585)	8585 8590 8600 8610 8620 8630 8640 8658
Homo sapiens chromosome 13 NC_000013.11: 34882059...	(8140)	TGCAACCCCAGCATTGTACCACAAAAGCAGACTGACAATACATTTTTTAAATGACCATTCTGTG----GTCCA
SARS-CoV-2 Reference Genome NC_045512.2	(8313)	TGACACCCCGTGACCTTGGTGCTTGTATTGACTGTAGTGCGCGTCATATTAATGCGCAGGTAGCAAAAAGTCAC
		Section 118
	(8659)	8659 8670 8680 8690 8700 8710 8720 8732
Homo sapiens chromosome 13 NC_000013.11: 34882059...	(8210)	ATCAAATTGTTTTTATAGAA----ACAGGCAACAGGCCAG-GTTTGACCCACAGCCAATACTTTTCC--AATAC
SARS-CoV-2 Reference Genome NC_045512.2	(8387)	AACATTGCTTTGATATGGAACGTTAAAGATTTCATGTCATTGTCTGAACAACTACGAAAACAAATACGTAGTGC
		Section 119
	(8733)	8733 8740 8750 8760 8770 8780 8790 8806
Homo sapiens chromosome 13 NC_000013.11: 34882059...	(8277)	CACGCACTTGATTAAAGTCTAAACTATTAAGACCCCAATTCCTCATCCCTTTATGCATTCACATCCT-TTGCCA
SARS-CoV-2 Reference Genome NC_045512.2	(8461)	TGCTAAAAAGAATAA---CTTACCTTTTAAGTTGACATGTGCAACTAC---TAGACAAGTTGTTAATGTTGTAA

Section 120

```
(8807) 8807 8820 8830 8840 8850 8860 8870 8880
Homo sapiens chromosome 13 NC_000013.11: 34882059... (8350) TGTCATCATTATGAGTTCAGTTTGT--AAGTCCTTTATTTGGCTTGACCCTTTA-TTATGGGTCAAATTGTT
SARS-CoV-2 Reference Genome NC_045512.2 (8529) CAACAAAGATAGCACTTAAGGGTGGTAAAATTGTTAATAATTGGTTGAAGCAGTTAATTAAAGTTACACTTGTG
```

Section 121

```
(8881) 8881 8890 8900 8910 8920 8930 8940 8954
Homo sapiens chromosome 13 NC_000013.11: 34882059... (8421) TCCCCCCAGAATTCATATGTCGAAGTCCGAACTCCCAGTACCTCAGAATATGACTGTATGTGGACATAGGGTCT
SARS-CoV-2 Reference Genome NC_045512.2 (8603) TTCCTTT----TTGTTGCTGCTATTTTCTATTTAATAACACCTGTTCATGTCA---TGTCTAAACATACTGACT
```

Section 122

```
(8955) 8955 8960 8970 8980 8990 9000 9010 9028
Homo sapiens chromosome 13 NC_000013.11: 34882059... (8495) TTATAGAGGTAATCAGCTAAAATGAGGTTATC-AAGATAGGCCCAATCCAATCTGACTGGGGTCCTTGTAAAAA
SARS-CoV-2 Reference Genome NC_045512.2 (8670) TTTCAAGTGAAATCATAGGATACAAGGCTATTGATGGTGGTGTCACTCG----TGACATAG---CATCTACAGA
```

Section 123

```
(9029) 9029 9040 9050 9060 9070 9080 9090 9102
Homo sapiens chromosome 13 NC_000013.11: 34882059... (8568) GGAGAAATTTGGAAACAGACACGCATACAGGGAAAACACCACGTGAACATGAACACAGCCATCTCCAAGGCAAG
SARS-CoV-2 Reference Genome NC_045512.2 (8737) TACTTGTTTTGCTAACAAACATGCTGATTTTGA------CACATGG---TTTAGCCAGCGTGGTGGTAGTTATA
```

Section 124

```
(9103) 9103 9110 9120 9130 9140 9150 9160 9176
Homo sapiens chromosome 13 NC_000013.11: 34882059... (8642) AGAGGGACCTGGAACAGATCCTTCCCTCACAGCCTTCAGAAGGAACCAAACCTGTCAACCTCTTGGTCTTGGAC
SARS-CoV-2 Reference Genome NC_045512.2 (8802) CTAATGACAAAG--CTTGCCCATTGATTGCTGCAGTCATAACAAGAGAAGTGGGT-------TTTGTCGTGCCT
```

Section 125

```
(9177) 9177 9190 9200 9210 9220 9230 9240 9250
Homo sapiens chromosome 13 NC_000013.11: 34882059... (8716) TTTTAGTCTCCAGAGCTGCGAGATAATAAATTTCTGTTGTTGAAGCCACCCAGTCTGTAG--ACATTTTTTAGG
SARS-CoV-2 Reference Genome NC_045512.2 (8867) GGTTTGCCT-----GGCACGATATTACGCACAACTAATGGTGACTTTTTGCATTTCTTACCTAGAGTTTTTAGT
```

Section 126

```
(9251) 9251 9260 9270 9280 9290 9300 9310 9324
Homo sapiens chromosome 13 NC_000013.11: 34882059... (8788) ACAGCCA-TAGGA-CACTAATATACCATGTGA-CTTGTTGTGCCCAGTGATGCTGGGCTAGAAGTGGCAAATGC
SARS-CoV-2 Reference Genome NC_045512.2 (8936) GCAGTTGGTAACATCTGTTACACACCATCAAAACTTATAGAGTACACTGA--CTTTGCAA-------CATCAGC
```

Section 127

	(9325)	9325 9330 9340 9350 9360 9370 9380 9398
Homo sapiens chromosome 13 NC_000013.11: 34882059...	(8859)	CAGTCTGTAGACTAGGTCTTAAAAAACTTTGAGTTTTTCCACTTGCTGTCTCACCCACCTTCCATTGCTATGAG
SARS-CoV-2 Reference Genome NC_045512.2	(9001)	TTGTGTTTTGGCTGCTGAATGTACAATTTTTAAAGATGCTTCTGGTAAGC-CAGT-ACCAT--ATTGTTATGAT

Section 128

	(9399)	9399 9410 9420 9430 9440 9450 9460 9472
Homo sapiens chromosome 13 NC_000013.11: 34882059...	(8933)	AAAATTATGCCCTAATGCTATTCTGCTGGTCTAAG---AAGCA-TGAGACACATT------TGAACCAGAGCCA
SARS-CoV-2 Reference Genome NC_045512.2	(9071)	ACCAATGTACTAGAAGGTTCTGTTGCTTATGAAAGTTTACGCCCTGACACACGTTATGTGCTCATGGATGGCTC

Section 129

	(9473)	9473 9480 9490 9500 9510 9520 9530 9546
Homo sapiens chromosome 13 NC_000013.11: 34882059...	(8997)	TTCCAGCCAATC-CCTACCTCCGGATGACCTGCAGATATACCAGCCATAATAAATTAT--TTCTG-----TAAA
SARS-CoV-2 Reference Genome NC_045512.2	(9145)	TATTATTCAATTTCCTAACACCTACCTTGAAGGTTCTGTTAGAGTGGTAACAACTTTTGATTCTGAGTACTGTA

Section 130

	(9547)	9547 9560 9570 9580 9590 9600 9610 9620
Homo sapiens chromosome 13 NC_000013.11: 34882059...	(9063)	GCCACTGAGTTTTAGAATAGATTACTACATAGCAACAACTAATTGATTTAAATGTTAAATTTAACATTAGTTAA
SARS-CoV-2 Reference Genome NC_045512.2	(9219)	GGCACGGCACTTGTGAA-AGATCAGAAGCTGGTGTTTGTGTATCTACT--AGTGGTAGATGGG----TACTTAA

Section 131

	(9621)	9621 9630 9640 9650 9660 9670 9680 9694
Homo sapiens chromosome 13 NC_000013.11: 34882059...	(9137)	ATTCCCAAAACACAGCTGTTTACATTCAATATAGAACTATGACTTTTTTCTGCATGTGTTTTGCCTTTTCCACT
SARS-CoV-2 Reference Genome NC_045512.2	(9286)	CAATGATTATTACAGATCTTTACCAGGAGTTT-----TCTGTGGTGTAGATGC-TGTAAATTTACTT----ACT

Section 132

	(9695)	9695 9700 9710 9720 9730 9740 9750 9768
Homo sapiens chromosome 13 NC_000013.11: 34882059...	(9211)	GAAAA-TTTAAGTCAATTCCTAAACACGTGGTTTGCTATCCACA----AGCCTTCTCTCTTCCTTTTTTCCTTC
SARS-CoV-2 Reference Genome NC_045512.2	(9350)	AATATGTTTACACCACTAATTCAACCTATTGGT-GCTTTGGACATATCAGCATCTATAGTAGCTGGTGGTATTG

Section 133

	(9769)	9769 9780 9790 9800 9810 9820 9830 9842
Homo sapiens chromosome 13 NC_000013.11: 34882059...	(9280)	CAGTGAAAATAGTGATATGAACTGATGAATAAATGAAGTCGGCTTGCTCGTCTTCTTCTAACTTAGGCCGGCAA
SARS-CoV-2 Reference Genome NC_045512.2	(9423)	TAGCTATCGTAGTAACATGCCTTGCCTACTATTTTATGAGGTTTAGAAGAGCTTTTGGTGAATACAGTCATGTA

Section 134

(9843) 9843 9850 9860 9870 9880 9890 9900 9916

Homo sapiens chromosome 13 NC_000013.11: 34882059... (9354) AATTGCTATGTAATCCCACTCTTTTCCACAGTTGCAAAAATGTGCTCCGTTTTGCTTTCAGCAGCCTCGTTTCA

SARS-CoV-2 Reference Genome NC_045512.2 (9497) G-TTGCCTT-TAATACTTTACTATTCCTTATGTCATTCACTGTACTCTGTTTAACAC-CAGTTTACTCATTCTT

Section 135

(9917) 9917 9930 9940 9950 9960 9970 9980 9990

Homo sapiens chromosome 13 NC_000013.11: 34882059... (9428) TCAGTCAGACTATTTCTTAAGAGATGTATCTATAGAGGGATAGTTTGCTGTTGTTGTTATTGTTGTTAATTAAA

SARS-CoV-2 Reference Genome NC_045512.2 (9568) ACCTGGTGTTTATTCTGTTATTTACTTGTACTTGACATTTTATCTTACTAATGATGTT--TCTTTTTTAGCACA

Section 136

(9991) 9991 10000 10010 10020 10030 10040 10050 10064

Homo sapiens chromosome 13 NC_000013.11: 34882059... (9502) TAATGCGATCCATTTTTAAACACACAGTTGTAAAATACCCTTTGAACAGACACACAGTTTGTTTGTTGGTTTGT

SARS-CoV-2 Reference Genome NC_045512.2 (9640) TATTCAG-TGGATGGTTATGTTCACACCTTTAGTA---CCTTTCTGGATA---ACAATTGCTTATATCATTTGT

Section 137

(10065) 10065 10070 10080 10090 10100 10110 10120 10138

Homo sapiens chromosome 13 NC_000013.11: 34882059... (9576) TTGTTTGTTTGTTTGTTTGTGACAGGGTCTCTCTCTGTTGCCTAGGCTGGAGTCAGTGATCATACCTCACTGCA

SARS-CoV-2 Reference Genome NC_045512.2 (9707) AT-TTCCACAAAGCATTTCT-ATTGGTTCTTTAGTAATTACCTAAAGAGACGT--GTAGTCTTTAATGG-TGTT

Section 138

(10139) 10139 10150 10160 10170 10180 10190 10200 10212

Homo sapiens chromosome 13 NC_000013.11: 34882059... (9650) GCATTCAGCTCCTGGGCTCAAG-TGATCCTCCCACCCCAGTCTCTGAGTAGCTAGGACTACAAGCACGTACCAC

SARS-CoV-2 Reference Genome NC_045512.2 (9776) TCCTTTAGTACTTTTGAAGAAGCTGCGCTGTGCACCTTTTTGT-TAAATAAAGAAATGTATCTAAA-GTTGCGT

Section 139

(10213) 10213 10220 10230 10240 10250 10260 10270 10286

Homo sapiens chromosome 13 NC_000013.11: 34882059... (9723) CATGCTCAGCTAATTAAAAAAAAAAAAAAAATTGTAGAGACAGGGTCTCTCTATGTTGCCTAGGCTAGTCTTGA

SARS-CoV-2 Reference Genome NC_045512.2 (9848) AGTGATGTGCTATT--ACCTCTTACGCAATATAATAGATACTTAG-CTCTTTATAATAAGTA--CAAGTATTTT

Section 140

(10287) 10287 10300 10310 10320 10330 10340 10350 10360

Homo sapiens chromosome 13 NC_000013.11: 34882059... (9797) ACTCCTGGCCTTAAAACAATCCTCTAGCCTCTGAAAGTGCTTG--GATTGCAGGCATGAGCCACCACTCCCAGC

SARS-CoV-2 Reference Genome NC_045512.2 (9917) AGTGGAGCAATGGATACAACTAGCTA--CAGAGAAGCTGCTTGTTGTCATCTCGCAAAGGCTCTCAATGACTTC

```
                                                                                                         Section 141
                                                     (10361) 10361    10370     10380     10390     10400     10410     10420        10434
Homo sapiens chromosome 13 NC_000013.11: 34882059...  (9869) CATACACATAGATTCTTAACTTGTTGGTACTTTTCACAAATTACCTAACATTTTGGTCTTAGTTTTTGATTGGC
        SARS-CoV-2 Reference Genome NC_045512.2       (9989) AGTA-ACTCAGGTTCTGATGTTCTT--TACCAACCACCACAAACCTC----TATCACCTCAGCTGTT---TTGC
                                                                                                         Section 142
                                                     (10435) 10435 10440     10450     10460     10470     10480     10490             10508
Homo sapiens chromosome 13 NC_000013.11: 34882059...  (9943) AGAATGAAATTAGACTAATTGGTGTGATGTATGGTGGGGGCCTTGAGCCATGATGTGTCAGTTTGACCTCAGGA
        SARS-CoV-2 Reference Genome NC_045512.2      (10053) AGAGTGGTTTTAGAAAAATGGCATTCCCATCTGGTAAAG---TTGAGGGTTGTATGGTACAAGTAACTTGTGGT
                                                                                                         Section 143
                                                     (10509) 10509      10520     10530     10540     10550     10560     10570      10582
Homo sapiens chromosome 13 NC_000013.11: 34882059... (10017) GGAACTGGAAACCGAGTAACAAAGGTCAGTCACACCATGAACTTCA-TGCCTA---CATGT------CCCAATT
        SARS-CoV-2 Reference Genome NC_045512.2      (10124) ACAACT--ACACT---TAACGGTCTTTGGCTTGATGACGTAGTTTACTGTCCAAGACATGTGATCTGCACCTCT
                                                                                                         Section 144
                                                     (10583) 10583  10590     10600     10610     10620     10630     10640          10656
Homo sapiens chromosome 13 NC_000013.11: 34882059... (10081) AAAACCCTGGAACTGGCTGGGTATGGTGGTTCATGCCT--GTAA-TCCCAGCACTTTGGGATGCTG-AGGCGGG
        SARS-CoV-2 Reference Genome NC_045512.2      (10193) GAAGACATGCTTAACCCTAATTATGAAGATTTACTCATTCGTAAGTCTAATCATAATTTCTTGGTACAGGCTGG
                                                                                                         Section 145
                                                     (10657) 10657        10670     10680     10690     10700     10710     10720  10730
Homo sapiens chromosome 13 NC_000013.11: 34882059... (10151) CAGATCATGAGATCAGGAAATAGAGACCACCCTGTCTAATACAATG-----AAACCTCATCTCTATTAAA--AA
        SARS-CoV-2 Reference Genome NC_045512.2      (10267) TA-ATGTTCAACTCAGGGT-TATTGGACATTCTATGCAAAATTGTGTACTTAAGCTTAAGGTTGATACAGCCAA
                                                                                                         Section 146
                                                     (10731) 10731    10740     10750     10760     10770     10780     10790        10804
Homo sapiens chromosome 13 NC_000013.11: 34882059... (10218) TAAAAATACAAAAAATTAGCCAAGCATGGTGGCACGTGCCTGTAGTCCCAGCTACTCAGGAGGCTGAAGTAGGA
        SARS-CoV-2 Reference Genome NC_045512.2      (10339) TCCTAAGACACCTAAGTAT--AAGTTTGTTCGCATTCAACCAG-GACAGACTTTTTCAGTGTTAGCTTGTTACA
                                                                                                         Section 147
                                                     (10805) 10805 10810     10820     10830     10840     10850     10860             10878
Homo sapiens chromosome 13 NC_000013.11: 34882059... (10292) GAATCTCTTGAACCTGGGAGACGGAGCTTGCAGTGAGCCGAGATTGCACCA------CTGCACTCCAGCCTGGG
        SARS-CoV-2 Reference Genome NC_045512.2      (10410) ATGGTTCACCATCTGGTGTTTACCAATGTGCTATGAGGCCCAATTTCACTATTAAGGGTTCATTCCTTAATGGT
```

Section 148

	(10879)	10879 10890 10900 10910 10920 10930 10940 10952
Homo sapiens chromosome 13 NC_000013.11: 34882059...	(10360)	CAACAGAATGAGATTCCGTCTCAAAAAAAAACAAAAAAACCCTGGAACCAAAGTTTGGGTGAACC-TCTC-ATT
SARS-CoV-2 Reference Genome NC_045512.2	(10484)	TCATGTGGT-AGTGTTGGTTTTAACATAGATT---ATGACTGTGTCTCTTTTTGTTACATGCACCATATGGAAT

Section 149

	(10953)	10953 10960 10970 10980 10990 11000 11010 11026
Homo sapiens chromosome 13 NC_000013.11: 34882059...	(10432)	TGGCAAT---ACTCTGTGCTGTCACACATCATTGCTGGAAAAATTCA-GTACTGCTCATATGACTT-CACAGGG
SARS-CoV-2 Reference Genome NC_045512.2	(10554)	TACCAACTGGAGTTCATGCTGGCACAGA-CTTAGAAGGTAACTTTTATGGACCTTTTGT-TGACAGGCAAACAG

Section 150

	(11027)	11027 11040 11050 11060 11070 11080 11090 11100
Homo sapiens chromosome 13 NC_000013.11: 34882059...	(10501)	AAGGGACAGCTGGAAACTTG--AACTTGTTCT-TTCCTGGTGACTTTAACTTGTACCCTTTTGCTGTAATAAAC
SARS-CoV-2 Reference Genome NC_045512.2	(10626)	CACAAGCAGCTGGTACGGACACAACTATTACAGTTAATGTTTTAGCTTGGTTGTACGCT---GCTGTTATAAAT

Section 151

	(11101)	11101 11110 11120 11130 11140 11150 11160 11174
Homo sapiens chromosome 13 NC_000013.11: 34882059...	(10572)	CATAACTGTGAGTATAACAGCT--TTTGCCAGTTCTGTGAGCCCTTCTAGCAAGTCATTGAAACTGAGGGTGTT
SARS-CoV-2 Reference Genome NC_045512.2	(10697)	GGAGACAGGTGGTTTCTCAATCGATTTACCACAACTCTTAATGACTTTAACC--TTGTGGCTA-TGAAG-TACA

Section 152

	(11175)	11175 11180 11190 11200 11210 11220 11230 11248
Homo sapiens chromosome 13 NC_000013.11: 34882059...	(10644)	CTTGGGGACTCCCAAACACATAACTATGCAAACATAC-ATGAAAAGAAAATA-TAAGATTGTGATTAATAACTT
SARS-CoV-2 Reference Genome NC_045512.2	(10767)	ATTATGAAC-CTCTAACACAAGACCATGTTGACATACTAGGACCTCTTTCTGCTCAAACTGGAATTGCCGTTTT

Section 153

	(11249)	11249 11260 11270 11280 11290 11300 11310 11322
Homo sapiens chromosome 13 NC_000013.11: 34882059...	(10716)	CTCCATTTGTTATATGTGTAATCACTGTACCCTGCCCCACTGTATGCAAAGACTGTGACATGTGTGCAAAGACT
SARS-CoV-2 Reference Genome NC_045512.2	(10840)	AGATATGTGTGCTTCATTAAAAGAAT-TAC--TGCAAAATGGTATGAATGGAC-GTACCATATTGGGTAGTGCT

Section 154

	(11323)	11323 11330 11340 11350 11360 11370 11380 11396
Homo sapiens chromosome 13 NC_000013.11: 34882059...	(10790)	CCCAAGCCCATGTGTAAGTGAGGAAGCCACTGAAGCTCGTGAGAGCCTAAG-TTAGGACTTACCATTTGATAAA
SARS-CoV-2 Reference Genome NC_045512.2	(10910)	TT-------ATTAGAAGATGAATTTACACCTTTTGATGTTGTTAGACAATGCTCAGGTGTTAC---TTTCCAAA

```
                                                                   Section 155
                                                     (11397) 11397        11410     11420     11430     11440     11450     11460   11470
Homo sapiens chromosome 13 NC_000013.11: 34882059... (10863) ATGATGTCAACATTACCATGG-GCTTCCCCAGTCCTTAGAGGATGTGGATCACTCTTTAACCAGTCGCACGGAA
    SARS-CoV-2 Reference Genome NC_045512.2          (10974) GTGCAGTGAAAAGAACAATCAAGGGTACACACCACT----GGTTGTTACTCACAATTTTGACT-TCACTTTTAG
                                                                   Section 156
                                                     (11471) 11471    11480     11490     11500     11510     11520     11530        11544
Homo sapiens chromosome 13 NC_000013.11: 34882059... (10936) AACTTGCCCTCCATGGTCTTGCAAGACCTAAGAACAAGGGTTAGC----AATTCCCAGTAAACCATTTGCTAT-
    SARS-CoV-2 Reference Genome NC_045512.2          (11043) TTTTAG---TCCAGAGTACTCAATGGTCTTTGTTCTTTTTTTTGTATGAAAATGCCTTTTTACCTTTTGCTATG
                                                                   Section 157
                                                     (11545) 11545 11550     11560     11570     11580     11590     11600           11618
Homo sapiens chromosome 13 NC_000013.11: 34882059... (11005) ----TTATGAGATCCTGGGCAAATTCCATGAACTCTCTGAACCTTAGG------TTCCTGGTTTTAAATAGGAA
    SARS-CoV-2 Reference Genome NC_045512.2          (11114) GGTATTATTGCTATGTCTGCTTTTGCAATGATGTTTGTCAAACATAAGCATGCATTTCTCTGTTTGTTTTTGTT
                                                                   Section 158
                                                     (11619) 11619      11630     11640     11650     11660     11670     11680    11692
Homo sapiens chromosome 13 NC_000013.11: 34882059... (11069) AGCAATAAGTACCACACAGAATTATT--ATTAAGTTTAACAAAGATAATGCATGTGTGATTGAGTAAAAATTAA
    SARS-CoV-2 Reference Genome NC_045512.2          (11188) ACCTTCTCTTGCCACTGTAGCTTATTTTAATATGGTCTATATGCCTGCTAGTTGGGTGAT-GCGTATTATGACA
                                                                   Section 159
                                                     (11693) 11693  11700     11710     11720     11730     11740     11750          11766
Homo sapiens chromosome 13 NC_000013.11: 34882059... (11141) TAACAGGATA-GCTCTTCACCACCAACT-TCCTTGTGAA--AGAAGATTTTGTC-TGTCT-CATCTTCTCCCCC
    SARS-CoV-2 Reference Genome NC_045512.2          (11261) TGGTTGGATATGGTTGATACTAGTTTGTCTGGTTTTAAGCTAAAAGACTGTGTTATGTATGCATCAGCTGTAGT
                                                                   Section 160
                                                     (11767) 11767        11780     11790     11800     11810     11820     11830   11840
Homo sapiens chromosome 13 NC_000013.11: 34882059... (11209) TTCCCCA--CCTTCCGAGAAGGATTCTGCTTTGCCCGTTGATTATTTGGAATTTGATAGTGCAGAATCAGAAAA
    SARS-CoV-2 Reference Genome NC_045512.2          (11335) GTTACTAATCCTTATGACAGCAAGAA--CTGTGTATGATGATGGT--GCTAGGAGAGTGTGGACACTTATGAAT
                                                                   Section 161
                                                     (11841) 11841    11850     11860     11870     11880     11890     11900        11914
Homo sapiens chromosome 13 NC_000013.11: 34882059... (11281) GAGAAAGTGGTAGAAGGAGAAAAGGTATATATATAGACTAAGGCTGTGGGACAAGC--TTTTCAGGAAGACTTT
    SARS-CoV-2 Reference Genome NC_045512.2          (11405) GTCT---TGACACTCGTTTATAAAGTTTAT-TATGG--TAATGCTTTAGATCAAGCCATTTCCATGTGGGCTCT
```

Section 162

```
(11915)                                               11915 11920     11930     11940     11950     11960     11970            11988
Homo sapiens chromosome 13 NC_000013.11: 34882059... (11353) GAGAGACAGAGAGAGATCTGGCAGCCCCTACAGAGGAGTAGAAAGTGA-ATGGGATATAAAAGGAAGAATGTTA
SARS-CoV-2 Reference Genome NC_045512.2 (11473)              TATAATCTCTGTTACTTCT---AACTACTCAGGTGTAGTTACAACTGTCATGTTTTTGGCCAGAGGTATTGTTT
```

Section 163

```
(11989)                                               11989      12000     12010     12020     12030     12040     12050        12062
Homo sapiens chromosome 13 NC_000013.11: 34882059... (11426) AGAAGTGCGTATCCTCTTTATAAGTAATATTTGAGAATCAGAAATATAGACCTGAGTAACCTCAAACCAACTTA
SARS-CoV-2 Reference Genome NC_045512.2 (11544)              TTATGTGTGTTGAGTATTGCCCTATTTTCTTC-ATAACTGGTAATACACTTCAGTGTA---TAATGCTAGTTTA
```

Section 164

```
(12063)                                               12063   12070     12080     12090     12100     12110     12120           12136
Homo sapiens chromosome 13 NC_000013.11: 34882059... (11500) TA--TAAAAATCCTATTTGAGAGAAATAGTACAAACTGAAGAATAGCCTTTCTATTTTTCTGTGGATTAGGCCA
SARS-CoV-2 Reference Genome NC_045512.2 (11614)              TTGTTTCTTAGGCTATTTTTG-----TACTTGTTACTTTGGCCTCTTTTGTTTACTCAACCGCTACTTTAGACT
```

Section 165

```
(12137)                                               12137         12150     12160     12170     12180     12190     12200   12210
Homo sapiens chromosome 13 NC_000013.11: 34882059... (11572) GATCACATCAAGCTTCATATGCTTCTATTTGT---CTTGC-TTTCCAGAAATTCTTTCAGAGTTTCCCTTAAGA
SARS-CoV-2 Reference Genome NC_045512.2 (11683)              GA-CTCTTGGTGTTTATGATTACTTAGTTTCTACACAGGAGTTTAGATATATGAATTCACAGGGACTACTCCCA
```

Section 166

```
(12211)                                               12211    12220     12230     12240     12250     12260     12270          12284
Homo sapiens chromosome 13 NC_000013.11: 34882059... (11642) TCCATGCCATTAACTTATACTGAAGGAATAATAAACTCAAAAAACATATTTTATTTCGGGAGATTTTTTTTTCA
SARS-CoV-2 Reference Genome NC_045512.2 (11756)              CCCAAG--AATAGCATAGA-TGCCTTCAAACTCAACATTAAATTGTTGGGTGTTGGTGGCAAACCTTGTATCAA
```

Section 167

```
(12285)                                               12285 12290     12300     12310     12320     12330     12340            12358
Homo sapiens chromosome 13 NC_000013.11: 34882059... (11716) -GT-GTGATGTCACGCTCAAGAAATTTCACTTAGAACATAAAGCCAGAGGAATTTCTCTCCTAAAGGTGTAATA
SARS-CoV-2 Reference Genome NC_045512.2 (11827)              AGTAGCCACTGTACAGTCTA-AAATGTCAGATGTAAAGTGCA-CATCAGTAGTCTTACTCTCAGTTTTGCAACA
```

Section 168

```
(12359)                                               12359      12370     12380     12390     12400     12410     12420        12432
Homo sapiens chromosome 13 NC_000013.11: 34882059... (11788) CGTTTTCCCTTCTGTAACGCTGACAAACGTGTGATTTTGGCTACCATTAAGTTCAGAGTCAAATTTCTAGCTC-
SARS-CoV-2 Reference Genome NC_045512.2 (11899)              ACTCAGAG-TAGAATCATCATCTAAATTGTGGGCTCAATGTGTCCAGTTACA-CAATGACATTCTCTTAGCTAA
```

Section 169

(12433) 12433 12440 12450 12460 12470 12480 12490 12506
Homo sapiens chromosome 13 NC_000013.11: 34882059... (11861) -GACTTTTGTTAATGATTTAAATTAA--ATTTAAATGATTTTTAAACTTCTGGCCGGATGTGGTGGCTCACACC
SARS-CoV-2 Reference Genome NC_045512.2 (11971) AGATACTACTGAAGCCTTTGAAAAAATGGTTTCACTACTTTCTGTTTTGCTTTCCATGCAGGGTGCTGTAGACA

Section 170

(12507) 12507 12520 12530 12540 12550 12560 12570 12580
Homo sapiens chromosome 13 NC_000013.11: 34882059... (11932) TGTAG----TTCCAGCACTTTGGGAGGCCAAGCGGGGAGGATT----GCTTGAGCCCAGGAGTTTGAGACCAGC
SARS-CoV-2 Reference Genome NC_045512.2 (12045) TAAACAAGCTTTGTGAAGAAATGCTGGACAACAGGGCAACCTTACAAGCTATAGCCTCAGAGTTTAGTTCCCTT

Section 171

(12581) 12581 12590 12600 12610 12620 12630 12640 12654
Homo sapiens chromosome 13 NC_000013.11: 34882059... (11998) CTATACAACTGAGACCTCATCTCCAAAAAAAATTAAAAATTAGCCAAGCATGGTGTTGCCCACCTGTAGTCCCA
SARS-CoV-2 Reference Genome NC_045512.2 (12119) CCAT-CATATGCAGCTTTTGCTACTGCTCAAG---AAGCTTATG--AGCAGGCTGTTGCTAATGGTGATTCTGA

Section 172

(12655) 12655 12660 12670 12680 12690 12700 12710 12728
Homo sapiens chromosome 13 NC_000013.11: 34882059... (12072) GCTACTTGGGAGACTGAGGTGGGAGGATCGTTAGAGCCCATGACATCAAGGCTGCAGTGAGTTGTGATCATGCC
SARS-CoV-2 Reference Genome NC_045512.2 (12187) AGTTGTTCTTAAAAAGTTGAAGAAG--TCTTTGAATGTGGCTAAATCTGAATTTGACCGTGATGCAGCCATGCA

Section 173

(12729) 12729 12740 12750 12760 12770 12780 12790 12802
Homo sapiens chromosome 13 NC_000013.11: 34882059... (12146) ACTGTACTGGGCAACAGAGTGAGAACCTGTCTCAAAGAAAACTTAAAATTAAAAATAAA-TAATTCTAAGTTCT
SARS-CoV-2 Reference Genome NC_045512.2 (12259) ACGTAAGTTGGAAA-AGATGGCTGATCAAGCT-----ATGACCCAAATGTATAAACAGGCTAGATCTGAGGACA

Section 174

(12803) 12803 12810 12820 12830 12840 12850 12860 12876
Homo sapiens chromosome 13 NC_000013.11: 34882059... (12219) ATCTATCTGAATTTGAGC-TGCCTTCTTCCCACTTTCACTTGCTTACATCAGATCATTGGGAATGGATGTAGAT
SARS-CoV-2 Reference Genome NC_045512.2 (12327) AGAGGGCAAAAGTTACTAGTGCTATGCAGACAATGCTTTTCACTATGCTTAGAAAGTTGGATAATGATGCACTC

Section 175

(12877) 12877 12890 12900 12910 12920 12930 12940 12950
Homo sapiens chromosome 13 NC_000013.11: 34882059... (12292) AATAATACCAG-AACAGGAGGATTTATTTATTTATTTATTTATTTATTTAGATAGAGTCTCACTCTGTCACCC
SARS-CoV-2 Reference Genome NC_045512.2 (12401) AACAACATTATCAACAATGCAAGAGATGGTTGTGTTCCCTTGAACATAATACCT-----CTTACAACAGCAGCC

Section 176

```
                                                    (12951) 12951    12960           12970           12980           12990           13000           13010       13024
Homo sapiens chromosome 13 NC_000013.11: 34882059... (12365) AGGCTGGAGTACAGTGGTGCAATCTC-AACTCACTGAAATCTCTGCCTGCTGGGCTTAAGCAATT-CTTCTGCC
SARS-CoV-2 Reference Genome NC_045512.2 (12470) AAACTAATGGTTGTCATACCAGACTATAACACATATAAAAATACGTGTGATGG--TACAACATTTACTTATGCA
```

Section 177

```
                                                    (13025) 13025 13030          13040           13050           13060           13070           13080             13098
Homo sapiens chromosome 13 NC_000013.11: 34882059... (12437) TCAGCCCCC-----AAAGTAGCTGAGATTACAGGCACCTGCCACCATGCCTGACTATTTTTTTTTTTTTGAGGT
SARS-CoV-2 Reference Genome NC_045512.2 (12542) TCAGCATTGTGGGAAATCCAACAGGTTGTAGATGCAGATAGTAAAATTGTTCA--ACTTAGTGAAATTAGTATG
```

Section 178

```
                                                    (13099) 13099      13110           13120           13130           13140           13150           13160       13172
Homo sapiens chromosome 13 NC_000013.11: 34882059... (12506) GAAATTTCATTCTTGTTGCCCAGGCTGGAGTGCAGTGGCACAATCTCGACTCACTGCAACCTCCACCTCCTGGA
SARS-CoV-2 Reference Genome NC_045512.2 (12614) GACAATTCAC-CTAATTTAGCATGGCCTCTTATTGTAACAGCTTTAAGGGCCAATTCTGCTGTCAAATTACAGA
```

Section 179

```
                                                    (13173) 13173  13180           13190           13200           13210           13220           13230           13246
Homo sapiens chromosome 13 NC_000013.11: 34882059... (12580) TTCAAGAGATTC-TCCTGTCTCAGCCTCCCAAGTAGCTGGGATTACAGGTGCCCGCCAC----CATGCCCAGCT
SARS-CoV-2 Reference Genome NC_045512.2 (12687) ATAATGAGCTTAGTCCTGTTGCACTACGACAGATGTCTTGTGCTGCCGGTACTACACAAACTGCTTGCACTGAT
```

Section 180

```
                                                    (13247) 13247        13260           13270           13280           13290           13300           13310  13320
Homo sapiens chromosome 13 NC_000013.11: 34882059... (12649) AATTTTTTGTATTTTTAGTATAGACGGGGTTTCACCATGTTGGCCAGGCTGGT-CTCAACCTGCTGACCT----
SARS-CoV-2 Reference Genome NC_045512.2 (12761) GACAATGCGTTAGCTTACTACA-ACACA-----ACAAAGGGAGGTAGGTTTGTACTTGCACTGTTATCCGATTT
```

Section 181

```
                                                    (13321) 13321    13330           13340           13350           13360           13370           13380       13394
Homo sapiens chromosome 13 NC_000013.11: 34882059... (12718) -CAGGTGATCAGCCCACCTTAGCCTCCCAAA--GTGCTGGAATTACAGGCATGAGCCAC-CACGCCTGGC-CCA
SARS-CoV-2 Reference Genome NC_045512.2 (12829) ACAGGATTTGAAATGGGCT-AGATTCCCTAAGAGTGATGGAACT---GGTACTATCTATACAGAACTGGAACCA
```

Section 182

```
                                                    (13395) 13395 13400          13410           13420           13430           13440           13450             13468
Homo sapiens chromosome 13 NC_000013.11: 34882059... (12787) GAACAGAGGGATTTTGAAGTTGCTGGGAGAGGACAAAGAGGTAAC-AGACTTCCTTTGTGTGTCGGTGGGAGGA
SARS-CoV-2 Reference Genome NC_045512.2 (12899) CCTTGTAGGTTTGTTACAGACACACCTAAAGGTCCTAAAGTGAAGTATTTATACTTTAT-TAAAGGATTAAACA
```

Section 183

	(13469)	13469 13480 13490 13500 13510 13520 13530 13542
Homo sapiens chromosome 13 NC_000013.11: 34882059...	(12860)	GTGTCAGTGGGAGTAGCCT--TAAGATAATAATCAATCAACACACATTTAAGAGCTATAGTAAGCCAACAAGAG
SARS-CoV-2 Reference Genome NC_045512.2	(12972)	ACCTAAATAGAGGTATGGTACTTGGTAGTTTAGCTGCCACAGTACGTCTACAAGCTG--GTAATGCAACAGAAG

Section 184

	(13543)	13543 13550 13560 13570 13580 13590 13600 13616
Homo sapiens chromosome 13 NC_000013.11: 34882059...	(12932)	T--------AGAGCAGTTTATTG-------GAGCTT---CAAGAGTCAAGGCTAAATGACACAAGGCTAAAAGA
SARS-CoV-2 Reference Genome NC_045512.2	(13044)	TGCCTGCCAATTCAACTGTATTATCTTTCTGTGCTTTTGCTGTAGATGCTGCTAAAGCTTACAAAGATTATCTA

Section 185

	(13617)	13617 13630 13640 13650 13660 13670 13680 13690
Homo sapiens chromosome 13 NC_000013.11: 34882059...	(12988)	TCCTTATGAATCTTGACCCAGCT-TAAAAGATCTAAG-TGATGAGAACA---ACTTAAAGTGTTTTGGTTCTAG
SARS-CoV-2 Reference Genome NC_045512.2	(13118)	GC-TAGTGGGGGACAACCAATCACTAATTGTGTTAAGATGTTGTGTACACACACTGGTACTGGTCAGGCAATAA

Section 186

	(13691)	13691 13700 13710 13720 13730 13740 13750 13764
Homo sapiens chromosome 13 NC_000013.11: 34882059...	(13057)	AATTTA-----GAATTCT-TCTGCGTGAAGAAT------TTA----ATCAGGTG------ACTGCAAAGTGTAC
SARS-CoV-2 Reference Genome NC_045512.2	(13191)	CAGTTACACCGGAAGCCAATATGGATCAAGAATCCTTTGGTGGTGCATCGTGTTGTCTGTACTGCCGTTGCCAC

Section 187

	(13765)	13765 13770 13780 13790 13800 13810 13820 13838
Homo sapiens chromosome 13 NC_000013.11: 34882059...	(13109)	AGTTTTCATTTTAAAATAACAGGAGATTGAACCTTGGAGCCCCACCAGAAAAGACTAAATGTGCTTAGGGATGA
SARS-CoV-2 Reference Genome NC_045512.2	(13265)	ATAGATCATCCAAATCCTAAAGGATTTTGTGACTTAAAAGGTAAGTATGTACAAATACCTACAACTTGTGCTAA

Section 188

	(13839)	13839 13850 13860 13870 13880 13890 13900 13912
Homo sapiens chromosome 13 NC_000013.11: 34882059...	(13183)	TGGCTTAGAGAGGTTTAACCAGCTGGAGGTGAAACAGTTTGCAA---CTGAACAATCTAGAT-GGTTATG--TG
SARS-CoV-2 Reference Genome NC_045512.2	(13339)	TGACCCTGTGGGTTTTA--CACTTAAAA---ACACAGTCTGTACCGTCTGCGGTATGTGGAAAGGTTATGGCTG

Section 189

	(13913)	13913 13920 13930 13940 13950 13960 13970 13986
Homo sapiens chromosome 13 NC_000013.11: 34882059...	(13251)	AAGTACTGTGCCATGCAGAGTCCTCATAATTTATCTCTGTAATTATAATATCAACCCTAGACCTTCATTTAAAA
SARS-CoV-2 Reference Genome NC_045512.2	(13408)	TAGTTGTGATCAACTCCGCGAACCCATGCTTCAG-TCAGCTGATGCACAATCGTTTTTAAACGGGTTTGCGGTG

Section 190

```
(13987)                                                       13987        14000     14010     14020     14030     14040     14050  14060
Homo sapiens chromosome 13 NC_000013.11: 34882059... (13325)  GAAGAAAATATTTGAACCATTATTGACTCCAGAAGGCAATATAAGGAGCCTTTTTTTTTTTTTTTTTTTTTTT
SARS-CoV-2 Reference Genome NC_045512.2 (13481)               TAAG-------TGCAGCCCGTCTT-ACACCGTGCGGCACAGGCACTAGTACTGATGTCGTATACAGGGCTTTTG
```

Section 191

```
(14061)                                                       14061    14070     14080     14090     14100     14110     14120       14134
Homo sapiens chromosome 13 NC_000013.11: 34882059... (13399)  TCAGAAACCTTTT---ATTGGTTTCATTAACAGAATCTGGCATGTCCACTTTAGTTCTCCTTAGCTAAACTTAA
SARS-CoV-2 Reference Genome NC_045512.2 (13547)               ACATCTACAATGATAAAGTAGCTGGTTTTGCTAAATTCCTAAAACTAATT--GTTGTCGCTTCCAAGAAAAGG
```

Section 192

```
(14135)                                                       14135 14140     14150     14160     14170     14180     14190         14208
Homo sapiens chromosome 13 NC_000013.11: 34882059... (13470)  ACAGACATTTCAATGTAAGTC-TACTTAAGTAAACTAAAAACAAGACACATAGAGTCACGCCCAACAAGAACCT
SARS-CoV-2 Reference Genome NC_045512.2 (13619)               ACGAAGATGACAATTTAATTGATTCTTACTTTGTAGTTAAGAGACACACTTTCTCTAACTACCAACATGAA---
```

Section 193

```
(14209)                                                       14209      14220     14230     14240     14250     14260     14270   14282
Homo sapiens chromosome 13 NC_000013.11: 34882059... (13543)  TAGAAACCATCTAGTCCTAGGCCGGGCACAGTGGCTCATGTCTGTAATCCCAGCACTTTGGGAGGCTGAGGCAG
SARS-CoV-2 Reference Genome NC_045512.2 (13690)               --GAAACAATTTATAATTTACTTAAGGATTGTCCAGC-TGTTGCTAAAC--ATGACTTCTTTAAGTTTAGAATA
```

Section 194

```
(14283)                                                       14283  14290     14300     14310     14320     14330     14340         14356
Homo sapiens chromosome 13 NC_000013.11: 34882059... (13617)  GCTAATCACCTGAGGTCAGG-AGTTCGAGACCA-GCCTGGCCAA---CACAGTTAAACTCCATCTCTATTAAAA
SARS-CoV-2 Reference Genome NC_045512.2 (13759)               GACGGTGACATGGTACCACATATATCACGTCAACGTCTTACTAAATACACAATGGCAGACCTCGTCTATGCTTT
```

Section 195

```
(14357)                                                       14357        14370     14380     14390     14400     14410     14420  14430
Homo sapiens chromosome 13 NC_000013.11: 34882059... (13686)  ATACAAAATTAGCTGGGCGTGGTGGTG-CA---TGCCTGTAATCCCAGCTACTCAGGAGGCAGGAGAATCTCTT
SARS-CoV-2 Reference Genome NC_045512.2 (13833)               A-AGGCATTTTGATGAAGGTAATTGTGACACATTAAAAGAAATACTTGTCACATACAATTGTTGTGATGATGAT
```

Section 196

```
(14431)                                                       14431    14440     14450     14460     14470     14480     14490       14504
Homo sapiens chromosome 13 NC_000013.11: 34882059... (13756)  GAACTCGGGAAGCAGA---GGTTGCAGTGAGCCGAGATTGC-GCCATTGCACTCCAGCCTGGGCAACAAGAGCG
SARS-CoV-2 Reference Genome NC_045512.2 (13906)               TATTTCAATAAAAAGGACTGGTATGATTTTGTAGAAACCCAGATATATTACGCGTATACGC-CAACTTAGGTG
```

Section 197

```
                                                          (14505) 14505 14510     14520     14530     14540     14550     14560            14578
Homo sapiens chromosome 13 NC_000013.11: 34882059... (13826) AAACTCTATCTCAAAAATTAATAAATAAATAAATAAATAAGAAAAGAAAAGAAATTTCCTAGTCCTAGCTCCTC
SARS-CoV-2 Reference Genome NC_045512.2 (13979) AACGTGTACGCCAAGCTTTGTTAAAAACAGTAC-AATTCTGTGATGCCATGCGAAATGCTGGTATT-GTTGGTG
```

Section 198

```
                                                          (14579) 14579      14590     14600     14610     14620     14630     14640       14652
Homo sapiens chromosome 13 NC_000013.11: 34882059... (13900) ATTTTACAGGTTGGAAACCAAAATTGATAAAGGCCAGAATCACATGTCTTAGCTAATGGCAAGAACCTGAGGCC
SARS-CoV-2 Reference Genome NC_045512.2 (14051) TACTGACAT-TAGATAATCAAGATC--TCAATGGTA-ACTGGTATGATTTCGGTGATTTCATACAAACCACGCC
```

Section 199

```
                                                          (14653) 14653  14660     14670     14680     14690     14700     14710           14726
Homo sapiens chromosome 13 NC_000013.11: 34882059... (13974) TCTGGATGCTCTGCGATCCTGTTCTGTCAGAAATGCATGAGGTTACTCTGCCCAAACCCCACCAATGCAAGTTA
SARS-CoV-2 Reference Genome NC_045512.2 (14121) AGGTAGTG----GAGTTCCTGTTGT-----AGATTCTTA---TTATTCATTGTTAATGCCTATATT--AACCTT
```

Section 200

```
                                                          (14727) 14727        14740     14750     14760     14770     14780     14790   14800
Homo sapiens chromosome 13 NC_000013.11: 34882059... (14048) TAAAAATGATTTTGAAGCTGTTTCTGAGGCAAAAACAGAGACCCACAGCTCTGCAATTCCATTTGCCAGCCCCA
SARS-CoV-2 Reference Genome NC_045512.2 (14181) GACCAGGGCTTTAACTGCAGAGTCACATGTTGACACTGA----CTTAACAAAGCC-TTACATTAAGTGGGATTT
```

Section 201

```
                                                          (14801) 14801    14810     14820     14830     14840     14850     14860         14874
Homo sapiens chromosome 13 NC_000013.11: 34882059... (14122) GTTCACATTTG-CTTTTTTTTCTATCAATTATACAAATTAAACTTCTCTAGTTTTCAAATTCCAGAATAATTTG
SARS-CoV-2 Reference Genome NC_045512.2 (14250) GTTAAAATATGACTTCACGGAAGAGAGGTTAAAACTCTTTGAC--CGTTATTTTAAATATTG--GGATCAG---
```

Section 202

```
                                                          (14875) 14875 14880     14890     14900     14910     14920     14930            14948
Homo sapiens chromosome 13 NC_000013.11: 34882059... (14195) CATATTCCAATAAATTCCCAGGTGACTTTATTGAGCATAGGCGTTAGCAGTATAGA-CAAATTTCATCTTTTGA
SARS-CoV-2 Reference Genome NC_045512.2 (14317) -ACATACCACCCAAATTGT-GTTAACTGTTTGGATGACAGATGCATTCTGCATTGTGCAAACTTTAATGTTTTA
```

Section 203

```
                                                          (14949) 14949      14960     14970     14980     14990     15000     15010       15022
Homo sapiens chromosome 13 NC_000013.11: 34882059... (14268) CTTGTTGCTACATGTCTTCGTTTAGGAATCCCTTCTGGCTTTTATGACTCAGGATTTTCTTTAAAATCTCCTTG
SARS-CoV-2 Reference Genome NC_045512.2 (14389) -TTCT--CTACA-GTGTTCCCACCTACAAGTTTTGGACCACTAGTGAGAAAAATATTTGTTGATGGTGTTCCAT
```

Section 204

```
                                                          15023  15030     15040     15050     15060     15070     15080          15096
                                               (15023)
Homo sapiens chromosome 13 NC_000013.11: 34882059... (14342) GACCAATATTTCTATATTTTCACTAAGGTTCAGTTAGCTCTTTTAAAT--TCAAAACTGTAATCTGC---AGCT
     SARS-CoV-2 Reference Genome NC_045512.2 (14459) TTGTAGTTTCAACTGGATACCACTTCAGAG-AGCTAGGTGTTGTACATAATCAGGA-TGTAAACTTACATAGCT
```

Section 205

```
                                                          15097         15110     15120     15130     15140     15150     15160  15170
                                               (15097)
Homo sapiens chromosome 13 NC_000013.11: 34882059... (14411) ATGAAATCTGTTGCCCAGCAAGGTAAGCATACTTTGCAATTCTTTGGCAACCTTCTTTTT-CTCTCCAACT--T
     SARS-CoV-2 Reference Genome NC_045512.2 (14531) CTAGACTTAGTTTT-----AAGGAAT---TACTTGTGTATGCTGCTG-ACCCTGCTATGCACGCTGCTTCTGGT
```

Section 206

```
                                                          15171    15180     15190     15200     15210     15220     15230        15244
                                               (15171)
Homo sapiens chromosome 13 NC_000013.11: 34882059... (14482) CTTCCATCCCATAATATTTACACTAAGT-CTTATTTGTTCTTACTTTCACTCCATGAGTATTTCTTCTACCTAC
     SARS-CoV-2 Reference Genome NC_045512.2 (14596) AATCTATTACTAGATAAACGCACTACGTGCTTTTCAGTAGCTGCACTTACTAACAATGTTGCTTTTCAAACTGT
```

Section 207

```
                                                          15245 15250     15260     15270     15280     15290     15300            15318
                                               (15245)
Homo sapiens chromosome 13 NC_000013.11: 34882059... (14555) CAAGCACCCTCAGTCCAAGTTCAAGGAAAGATACAGAACAGAATCAAAAG--TACATAAAATAATAGCAGATAC
     SARS-CoV-2 Reference Genome NC_045512.2 (14670) CAAACCCGGTAATTTTAACAAAGACTTCTATGACTTTGCTGTGTCTAAGGGTTTCTTTAAGGAA--GGAAGTTC
```

Section 208

```
                                                          15319      15330     15340     15350     15360     15370     15380       15392
                                               (15319)
Homo sapiens chromosome 13 NC_000013.11: 34882059... (14627) ATTTTCAGGATAATTGTTAATCCTTGTTTTG---TGTACT-----TTACAATGTGTGGGCACACCATT-TTGTT
     SARS-CoV-2 Reference Genome NC_045512.2 (14742) TGTTGAATTAAAACACTTCTTCTTTGCTCAGGATGGTAATGCTGCTATCAGCGATTATGACTACTATCGTTATA
```

Section 209

```
                                                          15393  15400     15410     15420     15430     15440     15450          15466
                                               (15393)
Homo sapiens chromosome 13 NC_000013.11: 34882059... (14692) TTTTAATAACAATATCTTTCATCAGTTAAAAAAC----GTAGTTTCTATTTTTCCTATTATAATATGGCCGGTT
     SARS-CoV-2 Reference Genome NC_045512.2 (14816) ATCTACCAACAATGTGTGATATCAGACAACTACTATTTGTAGTTGAAGTTGTTGATAAG-TACTTTGATTGTTA
```

Section 210

```
                                                          15467         15480     15490     15500     15510     15520     15530  15540
                                               (15467)
Homo sapiens chromosome 13 NC_000013.11: 34882059... (14762) --ATTTCCTCT-TATTTTTTAAAAATACCATTTTTGCTGTATCCTGTGTAACTGATACAGAAATA-AGTTCACA
     SARS-CoV-2 Reference Genome NC_045512.2 (14889) CGATGGTGGCTGTATTAATGCTAACCAAGTCATCGTCAACAACCTAGACAAATCAGCTGGTTTTCCATTTAATA
```

```
                                                                    Section 211
                                                      (15541) 15541    15550     15560     15570     15580     15590     15600        15614
Homo sapiens chromosome 13 NC_000013.11: 34882059... (14832) AAAG-----ATTATAAATTTTTTTTTGCAATAAGGAGTTAGTCAAGGCCAGTCGCGGTGG-CTCACACTTGTAA
     SARS-CoV-2 Reference Genome NC_045512.2 (14963) AATGGGGTAAGGCTAGACTTTATTATGATTCAATGAGTTATGAGGATCAAGATGCACTTTTCGCATATACAAAA
                                                                    Section 212
                                                      (15615) 15615 15620      15630     15640     15650     15660     15670          15688
Homo sapiens chromosome 13 NC_000013.11: 34882059... (14900) TCCCATCTCAAAACAAAAACAAACAAACAAACAAACAAAAACAAAGAGTTAGTCAAAACAAGT-AGCTTGG-CC
     SARS-CoV-2 Reference Genome NC_045512.2 (15037) CGTAATGTCATCCCTACTATAACTCAA--ATGAATCTTAAGTATGCCATTAGTGCAAAGAATAGAGCTCGCACC
                                                                    Section 213
                                                      (15689) 15689      15700     15710     15720     15730     15740     15750      15762
Homo sapiens chromosome 13 NC_000013.11: 34882059... (14972) GGATATGGTGCTCACGCCTGTAATCCCAGCACCTTTGGAGGCCGAGGCAGGCTGATCACCTGAGGTCAGGAGTT
     SARS-CoV-2 Reference Genome NC_045512.2 (15109) GTAGCTGGTGTCTCTATCTGTAGTACTATGACCAATAGA--CAGTTTCATCAAAAATTATTGAAATCAATAGCC
                                                                    Section 214
                                                      (15763) 15763  15770     15780     15790     15800     15810     15820          15836
Homo sapiens chromosome 13 NC_000013.11: 34882059... (15046) ----CGAGACCAGC--CTGGCCAACATGGTGAAACCTCATCTCTACTAAAAATACAAAAACAAATTAGCCAG-G
     SARS-CoV-2 Reference Genome NC_045512.2 (15181) GCCACTAGAGGAGCTACTGTAGTAATTGGAACAAGCAAAT-TCTATGGTGGTTGGCACAACATGTTAAAAACTG
                                                                    Section 215
                                                      (15837) 15837        15850     15860     15870     15880     15890     15900  15910
Homo sapiens chromosome 13 NC_000013.11: 34882059... (15113) CATCCACTGTGGCACAAGCCTGTAATCCCAGCTACTCAGGG-AGCTGAGGCAGGAGAATTGCTTGAACCTAGGA
     SARS-CoV-2 Reference Genome NC_045512.2 (15254) TTTATAGTGATGTAGAAAACCCTCACCTTATGGGTTGGGATTATCCTAAATGTGATAGAGCCATG--CCTAACA
                                                                    Section 216
                                                      (15911) 15911    15920     15930     15940     15950     15960     15970        15984
Homo sapiens chromosome 13 NC_000013.11: 34882059... (15186) AGTGGAGG-TTGTGGTCAGCTGTGGTCAAGC-CACTGCACTCCAGCCTGGGTGACACAGTGAGACTCCATCTCA
     SARS-CoV-2 Reference Genome NC_045512.2 (15326) TGCTTAGAATTATGGCCTCACTTGTTCTTGCTCGCAAACATACAACGTG--TTGTAGCTTGTCACACCGTTTCT
                                                                    Section 217
                                                      (15985) 15985 15990      16000     16010     16020     16030     16040          16058
Homo sapiens chromosome 13 NC_000013.11: 34882059... (15258) AAAAAACAAAACAAACAAGTAGCTAATTTTTGGAAGACTTTTTGCAAGGATTAAATTCACAAATCTTAGATGAG
     SARS-CoV-2 Reference Genome NC_045512.2 (15398) ATAGATTAGCTAATGAGTGTGCTCAAGTATTGAGTGAAATGGTCATGTGTGGCGGTTCAC------TATATGTT
```

Section 218

```
                                                              (16059) 16059      16070     16080     16090     16100     16110     16120     16132
Homo sapiens chromosome 13 NC_000013.11: 34882059... (15332) CCAAGAGCTGGAAACTTC-CAGCAGATGCAGTCAGAGAAGTGAGTAAGAATGAGTCTTACCCCTTGCA---TTT
SARS-CoV-2 Reference Genome NC_045512.2 (15466)             AAACCAGGTGGAACCTCATCAGGAGATGCCACAACTGCTTATGCTAATAGTGTTTTTAACATTTGTCAAGCTGT
```

Section 219

```
                                                              (16133) 16133 16140     16150     16160     16170     16180     16190     16206
Homo sapiens chromosome 13 NC_000013.11: 34882059... (15402) ATTGGGTTCTCTTA-TGAGATTTAATATGATAAATG-AGCACGGTGGCTCAGGCCTGTAATCACAGCACTTTGG
SARS-CoV-2 Reference Genome NC_045512.2 (15540)             CACGGCCAATGTTAATGCACTTTTATCTACTGATGGTAACAAAATTGCCGATAAGT---ATGTCCGCAATTTAC
```

Section 220

```
                                                              (16207) 16207        16220     16230     16240     16250     16260     16270 16280
Homo sapiens chromosome 13 NC_000013.11: 34882059... (15474) GAGACCGAG---GAGGGTAGATCCCTTGAAGACAGGAATTTCAGACCAGCCTGGCCACCATGGTGAAACCCCGT
SARS-CoV-2 Reference Genome NC_045512.2 (15611)             AACACAGACTTTATGAGTGTCTCTATAGAAATAGAGATGTTGACACAGACTTTGTGAATGAGTTTTACGCATAT
```

Section 221

```
                                                              (16281) 16281    16290     16300     16310     16320     16330     16340     16354
Homo sapiens chromosome 13 NC_000013.11: 34882059... (15545) CTCTACTAAAAATACAAAAATTAGCTGGACT-TGGTGGCACGTATCTGTAATCC--TAGCTACTCAGGAGGCTG
SARS-CoV-2 Reference Genome NC_045512.2 (15685)             TTGCGTAAACATTTCTCAATGATGATACTCTCTGACGATGCTGTTGTGTGTTTCAATAGC-ACTTATGCATCTC
```

Section 222

```
                                                              (16355) 16355 16360     16370     16380     16390     16400     16410     16428
Homo sapiens chromosome 13 NC_000013.11: 34882059... (15616) ACA--CTTGAGATTCACTT---GAACCT---GGGAGGT-TGCAAGATTGCACCACTACACTCCAGCCTGTCTCA
SARS-CoV-2 Reference Genome NC_045512.2 (15758)             AAGGTCTAGTGGCTAGCATAAAGAACTTTAAGTCAGTTCTTTATTATCAAAACAATGTTTTTATGTCTGAAGCA
```

Section 223

```
                                                              (16429) 16429      16440     16450     16460     16470     16480     16490     16502
Homo sapiens chromosome 13 NC_000013.11: 34882059... (15681) AAAAAAGAAAAAATTAATTAATTAATTAAATATATGTATATATTTA--TCATATTTTTACATGATTTTTATATA
SARS-CoV-2 Reference Genome NC_045512.2 (15832)             AAATGTTGGACTGAGACTGACCTTACTAAAGGACCTCATGAATTTTGCTCTCAACATACAATGCTAGTTAAACA
```

Section 224

```
                                                              (16503) 16503 16510     16520     16530     16540     16550     16560     16576
Homo sapiens chromosome 13 NC_000013.11: 34882059... (15753) TGATAAATGAACCAATTAGACTCCATTATGTTTTTCAATGCCCCACTCCCCAAATTTGCCACCATTTAAAATTC
SARS-CoV-2 Reference Genome NC_045512.2 (15906)             GGGTGA-TGATTATGTGTACCTTCCTTACCCAGATCCAT-CAAGAATCCTAGGGGCCGGCTGTTTTGTAGATG-
```

Section 225

	(16577)	16577 16590 16600 16610 16620 16630 16640 16650
Homo sapiens chromosome 13 NC_000013.11: 34882059...	(15827)	ACCAAAAGCTAAAATTTGGATCATTGCTTTATGACTAAAAAGTCTGTGAATCT-GCTA-----------CCAAC
SARS-CoV-2 Reference Genome NC_045512.2	(15977)	----ATATCGTAAAAACAGATGGTACACTTATGATTGAACGGTTCGTGTCTTTAGCTATAGATGCTTACCCACT

Section 226

	(16651)	16651 16660 16670 16680 16690 16700 16710 16724
Homo sapiens chromosome 13 NC_000013.11: 34882059...	(15889)	TA-TAAAATCCCTTCACAAGTGTAT----ATTCCCTTAGGAAAGA--TCAAATTCATCAGAAGGGCAACTT---
SARS-CoV-2 Reference Genome NC_045512.2	(16047)	TACTAAACATCCTAATCAGGAGTATGCTGATGTCTTTCATTTGTACTTACAATACATAAGAAAGCTACATGATG

Section 227

	(16725)	16725 16730 16740 16750 16760 16770 16780 16798
Homo sapiens chromosome 13 NC_000013.11: 34882059...	(15953)	--TCAAGAGTATGAATTTTAAGCCA-TATTAAGAGTTCTTCCCAAAGCATCTCAGAGAAACATGTTCAGAAGTC
SARS-CoV-2 Reference Genome NC_045512.2	(16121)	AGTTAACAGGACACATGTTAGACATGTATTCTGTTATGCTTACTAATGATAACACTTCAAGGTATTGGGAACCT

Section 228

	(16799)	16799 16810 16820 16830 16840 16850 16860 16872
Homo sapiens chromosome 13 NC_000013.11: 34882059...	(16024)	CAGCCTCATG--GC------CACAGCCCAAACAATCCTGGAGTTATTTTAGTGCTCTGCGATATTTTCG-TCCC
SARS-CoV-2 Reference Genome NC_045512.2	(16195)	GAGTTTTATGAGGCTATGTACACACCGCATACAGTCTTACAGGC-TGTTGGGGCT-TGTGTTCTTTGCAATTCA

Section 229

	(16873)	16873 16880 16890 16900 16910 16920 16930 16946
Homo sapiens chromosome 13 NC_000013.11: 34882059...	(16089)	CATCCTTTGTCCTCCTTTT---CTATAGTATCGATTTCAGGATTAGTATCACTATCATATAACTACACACACAC
SARS-CoV-2 Reference Genome NC_045512.2	(16267)	CAGACTTCATTAAGATGTGGTGCTTGCATACGTAGACCATTCTTATGTTGTAAATGCTGTTACGACCATGTCAT

Section 230

	(16947)	16947 16960 16970 16980 16990 17000 17010 17020
Homo sapiens chromosome 13 NC_000013.11: 34882059...	(16160)	ACACACATTAAA--AACCTGTAT-GTAGGTATATTTTCATGTTGCCATTTGCTTCTGATTATCCTTGATCTACA
SARS-CoV-2 Reference Genome NC_045512.2	(16341)	ATCAACATCACATAAATTAGTCTTGTCTGTTAATCCGTATGTTTGCAAT-GCTCCAGGTTGTGATGTCAC-AGA

Section 231

	(17021)	17021 17030 17040 17050 17060 17070 17080 17094
Homo sapiens chromosome 13 NC_000013.11: 34882059...	(16231)	TGCATATATGCTTTTAAAATAATGAGCCTTATTTATTTTTCCAATTTCATTTTACCTTAGAACTGTTCATTTGC
SARS-CoV-2 Reference Genome NC_045512.2	(16413)	TGTGACTCAACTTT--ACTTAGGAGGTATGAGCTATTATTGTAAATCACATAAACC----ACCCATTAGTTTTC

Section 232

(17095)		17095 17100 17110 17120 17130 17140 17150 17168
Homo sapiens chromosome 13 NC_000013.11: 34882059...	(16305)	ATAAGAGTAGTCAGTGAAATGTATACTTCTAATTGTTCATTTTTACTTGTGTCAAAACCAGATATCCTTCATCT
SARS-CoV-2 Reference Genome NC_045512.2	(16481)	CATTGTGTGCTAATGGACAAGTTT---TTGGTTTATATAAAAATACATGTGTTGGTAGC-GATAATGTTA--CT

Section 233

(17169)		17169 17180 17190 17200 17210 17220 17230 17242
Homo sapiens chromosome 13 NC_000013.11: 34882059...	(16379)	TTTTTTCCTGACTTTACATCGT-TCACAAGCACAACCCATTTCCTTCAAAATTTTCTCACTGCAC-ACAGTAAT
SARS-CoV-2 Reference Genome NC_045512.2	(16549)	GACTTTAATGCAATTGCAACATGTGACTGGACAAATGCTGGTGATTACATTTTAGCTAACACCTGTACTGAAAG

Section 234

(17243)		17243 17250 17260 17270 17280 17290 17300 17316
Homo sapiens chromosome 13 NC_000013.11: 34882059...	(16451)	TTTCCAGTGGTAGGAA--AGAATGCTTCATGGGTA-TTTCAAAACATCT--TCTGTCCTAACCCTTCGTTCTTT
SARS-CoV-2 Reference Genome NC_045512.2	(16623)	ACTCAAGCTTTTTGCAGCAGAAACGCTCAAAGCTACTGAGGAGACATTTAAACTGTCTTATGGTATTGCTACTG

Section 235

(17317)		17317 17330 17340 17350 17360 17370 17380 17390
Homo sapiens chromosome 13 NC_000013.11: 34882059...	(16520)	TCTCACTTTGGATCTCTGGTGTCTTCCCACA-CAAAAAGGGGAATATGCCATCTCCCTTTAGGAAATCAAG-AT
SARS-CoV-2 Reference Genome NC_045512.2	(16697)	TACGTGAAGTGCTGTCTGACAGAGAATTACATCTTTCATGGGAAGTTGGTAAACC----TAGACCACCACTTAA

Section 236

(17391)		17391 17400 17410 17420 17430 17440 17450 17464
Homo sapiens chromosome 13 NC_000013.11: 34882059...	(16592)	TCTAAAATATCTTTCCTCTCTTGACCGTTTTGG-----ACTGTGG-GTATTCATA----ACCTCACATATAAAA
SARS-CoV-2 Reference Genome NC_045512.2	(16767)	CCGAAATTATGTCTTTACTGGTTATCGTGTAACTAAAAACAGTAAAGTACAAATAGGAGAGTACACCTTTGAAA

Section 237

(17465)		17465 17470 17480 17490 17500 17510 17520 17538
Homo sapiens chromosome 13 NC_000013.11: 34882059...	(16656)	TTGGCATAAATGTATTTATTTATTTATTTATTTATTTATTT-ATTTTATTTATTTATTTTGAGACAGTGTCTCG
SARS-CoV-2 Reference Genome NC_045512.2	(16841)	AAGG--TGACTATGGTGATGCTGTTGTTTACCGAGGTACAACAACTTACAAATTAAATGTTGGTGATTATTTTG

Section 238

(17539)		17539 17550 17560 17570 17580 17590 17600 17612
Homo sapiens chromosome 13 NC_000013.11: 34882059...	(16729)	CTCTGTCG-CCCAGGCTGGAGTGCAGTGGCGCGATCTCAGCTCACTGCAAGCTCTGCCTCCCGGGTTCCTGCCA
SARS-CoV-2 Reference Genome NC_045512.2	(16913)	TGCTGACATCACATACAGTAATGCCATTAAGTGCACCTACACTAGTGCCACAAGAGCACTATGTTAGAATTACT

Section 239

(17613)		17613 17620 17630 17640 17650 17660 17670 17686
Homo sapiens chromosome 13 NC_000013.11: 34882059...	(16802)	TTCTCCTGCCTCAGCCTCCTGAGTAGCTGGGACTACATGCGCCCGCCACCACGCCTGGCTAATTTTTTGTATTT
SARS-CoV-2 Reference Genome NC_045512.2	(16987)	GGCTTATACCCAACACTC---AATATCTCAGA-----TGAGTTTTCTAGCAATGTTGC--AAATTATCAAAAGG

Section 240

(17687)		17687 17700 17710 17720 17730 17740 17750 17760
Homo sapiens chromosome 13 NC_000013.11: 34882059...	(16876)	TTAGTAGAGACGGGGTTTCACCGTGTTAGCCAGGA--TGGT-CTCGATCTCCTGACCTCGTGATCTGCCCGCCT
SARS-CoV-2 Reference Genome NC_045512.2	(17051)	TTGGTATGCAAAAGTATTCTACACTCCAGGGACCACCTGGTACTGGTAAGAGTCATTTTGCTATTGGCCTAGCT

Section 241

(17761)		17761 17770 17780 17790 17800 17810 17820 17834
Homo sapiens chromosome 13 NC_000013.11: 34882059...	(16947)	CGGC-CTCCCAAAGTGCTGGGATTACAGGCGTGAGACACCACGCCTGGCCTAATTTATTTTTTAAGTAATAAGC
SARS-CoV-2 Reference Genome NC_045512.2	(17125)	CTCTACTACCCTTCTGCTCGCAT-AGTGTATACAGCTTGCTCTCATGCCGCTGTTGATGCACTATGTGAGAAGG

Section 242

(17835)		17835 17840 17850 17860 17870 17880 17890 17908
Homo sapiens chromosome 13 NC_000013.11: 34882059...	(17020)	TTTAATCAGTTATGATAAAACATCAGTTATGAACCACATTGTTAGTTTTTGTTACTCTTCTTTCACTATTATAT
SARS-CoV-2 Reference Genome NC_045512.2	(17198)	CATTAAAATATTTGCCTATAGATAA---ATGTAGTAGAATTATACCTGCACGTGCTCGTGTAG-AGTGTTTTGA

Section 243

(17909)		17909 17920 17930 17940 17950 17960 17970 17982
Homo sapiens chromosome 13 NC_000013.11: 34882059...	(17094)	TTCAGTAAATATTGCCAAATGCATTTTAAT----TGTCTTTGATATTTTAATAGCTTCATGTACCCTTGAAGAG
SARS-CoV-2 Reference Genome NC_045512.2	(17268)	TAAATTCAA-AGTGAATTCAACATTAGAACAGTATGTCTTTTGTACTGTAAATGCAT----TGCCTGAGACGAC

Section 244

(17983)		17983 17990 18000 18010 18020 18030 18040 18056
Homo sapiens chromosome 13 NC_000013.11: 34882059...	(17164)	ATGTGCTGCCAGTCTAATTATTTTCAA--AAAGTTAAATTATCCCTCAA-ATGA---GACTTTCTTCATTTTAA
SARS-CoV-2 Reference Genome NC_045512.2	(17337)	A------GCAGATATAGTTGTCTTTGATGAAATTTCAATGGCCACAAATTATGATTTGAGTGTTGTCAATGCCA

Section 245

(18057)		18057 18070 18080 18090 18100 18110 18120 18130
Homo sapiens chromosome 13 NC_000013.11: 34882059...	(17232)	AATTTCCTCATCTACCCATAGTGTTTGTTGATAGATTTCCTCAACTA--TTTTCTAGGTCTCTTGTTTTCT---
SARS-CoV-2 Reference Genome NC_045512.2	(17405)	GATTACGTGCTAAGCACTATGTGTACATTGGCGACCCTGCTCAATTACCTGCACCACGCACATTGCTAACTAAG

```
                                                              Section 246
                                                 (18131) 18131    18140    18150    18160    18170    18180    18190    18204
Homo sapiens chromosome 13 NC_000013.11: 34882059... (17301) ------CTTGTGTTGTAATTAATCAGTT-AGTGTTT----TCATGCA---T--AGGGAAACACATGTTGTTCTC
SARS-CoV-2 Reference Genome NC_045512.2 (17479) GGCACACTAGAACCAGAATATTTCAATTCAGTGTGTAGACTTATGAAAACTATAGGTCCAGACATGTTCCTCGG
                                                              Section 247
                                                 (18205) 18205 18210    18220    18230    18240    18250    18260    18278
Homo sapiens chromosome 13 NC_000013.11: 34882059... (17359) ATCAAGTTTCTTTCAGTTG-CTTACTTAATCTTCTTCACAGCTTCCAGAAATATCTTTACTC--CATCTG-TAG
SARS-CoV-2 Reference Genome NC_045512.2 (17553) AAC----TTGTCGGCGTTGTCCTGCTGAAA-TTGTTGACA-CTGTGAGTGCTTTGGTTTATGATAATAAGCTTA
                                                              Section 248
                                                 (18279) 18279    18290    18300    18310    18320    18330    18340    18352
Homo sapiens chromosome 13 NC_000013.11: 34882059... (17429) AATTACAGGTTTAGAATACAGTT---TCCTTTCCA--GGTTATTGACTCTGTTTTCCCCTGTGATGTTGCAATT
SARS-CoV-2 Reference Genome NC_045512.2 (17621) AAGCACATAAAGACAAATCAGCTCAATGCTTTAAAATGTTTTATAAGGGTGTTATCACGCATGATGTTTCATCT
                                                              Section 249
                                                 (18353) 18353 18360    18370    18380    18390    18400    18410    18426
Homo sapiens chromosome 13 NC_000013.11: 34882059... (17498) TTTGTTCACTAGCCA-----AGGAGTATTAACTGGTGTACTCATTCGTTTGGGAACTTAATTTTTTGGGTTCTA
SARS-CoV-2 Reference Genome NC_045512.2 (17695) GCAATTAACAGGCCACAAATAGGCGTGGTAAGAGAATTCCTTACACGTAACCCTGCTTGGAGAAAAGCTGTCT-
                                                              Section 250
                                                 (18427) 18427    18440    18450    18460    18470    18480    18490    18500
Homo sapiens chromosome 13 NC_000013.11: 34882059... (17567) TTATTTTCCTTTTTTAGGTGACATTATTTTTTTTTTACTGCATATAGAAATAAGGAATAACCTGACACTAGATG
SARS-CoV-2 Reference Genome NC_045512.2 (17768) TTATTTCACCTTATAAT-TCACAGAATGCTGTAGCCTC-----AAAGATTTTGGGACTACCA--ACTCAAACTG
                                                              Section 251
                                                 (18501) 18501    18510    18520    18530    18540    18550    18560    18574
Homo sapiens chromosome 13 NC_000013.11: 34882059... (17641) TTACTTCCTCTACCGTACCATCTTTCCAGAACGCCATAGACTTGTACCACAAAATACAGTCATTTTTAAATAGT
SARS-CoV-2 Reference Genome NC_045512.2 (17834) TTGATTCATCACAGGGCTCAGAATATGACTATGTCATATTCA-----CTCAAACCACTG---------AAACAGC
                                                              Section 252
                                                 (18575) 18575 18580    18590    18600    18610    18620    18630    18648
Homo sapiens chromosome 13 NC_000013.11: 34882059... (17715) TGTGCTTGATTTTTTTCTTGTTAACTTCCTATGATGATTGCTGCACGGTATAGTAAAATAGATGAGCATATTAT
SARS-CoV-2 Reference Genome NC_045512.2 (17895) TC------ACTCTTGTAATGTAAAC-----AGATTTAATGTTGCTATTACCAGAGCAAAAGTAG-GCATACTTT
```

```
                                                                                 Section 253
                                                        (18649) 18649      18660     18670     18680     18690     18700     18710   18722
Homo sapiens chromosome 13 NC_000013.11: 34882059... (17789) TTTAAAAGTATTTTTCATGCCTTTAACAGGCTTTGTTTGCTGTGTAAGGTTTGAACA-CTAAGTGATAATGACC
SARS-CoV-2 Reference Genome NC_045512.2 (17957) GCATAATGTCTGATAGAGACCTTTATGACAAGTTGCAA--TTTACAAGTCTTGAAATTCCACGTAGGAATGTGG

                                                                                 Section 254
                                                        (18723) 18723 18730     18740     18750     18760     18770     18780          18796
Homo sapiens chromosome 13 NC_000013.11: 34882059... (17862) CATAAT-ATAAACTAATTCATGCTCATAGATCCCTCCAAGTAGGGATTTCGAGTAAATTAGAGGCAATCTTTTT
SARS-CoV-2 Reference Genome NC_045512.2 (18029) CAACTTTACAAGCTGAAA-ATG-TAACAGGACTCTTTAAAGATTGTAGTAAGGTAATCACTGGGTTACATCCTA

                                                                                 Section 255
                                                        (18797) 18797         18810     18820     18830     18840     18850     18860 18870
Homo sapiens chromosome 13 NC_000013.11: 34882059... (17935) CA-ACCGAGCAACTTTTCTTAGAGCACAGAACAACTGCCATCCTCTGCCTTGATGCGGGGAG----CTGCAATT
SARS-CoV-2 Reference Genome NC_045512.2 (18101) CACAGGCACCTACACACCTCAGTGTTGACACTAAATTCAAAACTGAAGGTTTATGTGTTGACATACCTGGCATA

                                                                                 Section 256
                                                        (18871) 18871    18880     18890     18900     18910     18920     18930      18944
Homo sapiens chromosome 13 NC_000013.11: 34882059... (18004) GCTGAAGGTA-GATACGTTCACGACCTAAGTTAGAGTTTAGCCTTTAACC---TTACCAAGGGTATCATTCCAA
SARS-CoV-2 Reference Genome NC_045512.2 (18175) CCTAAGGACATGACCTATAGAAGACTCATCTCTATGATGGGTTTTAAAATGAATTATCAAGTTAATGGTTACCC

                                                                                 Section 257
                                                        (18945) 18945 18950     18960     18970     18980     18990     19000          19018
Homo sapiens chromosome 13 NC_000013.11: 34882059... (18074) CAGCCATCTACCTCACCACCTAAAGCAGGAAAACGAAATGTCTTTTCTT---TTGTCATTGGCTTTCTACTCCA
SARS-CoV-2 Reference Genome NC_045512.2 (18249) TAAC-ATGTTTATCACCCGCGAA-GAAGCTATAAGACATGTACGTGCATGGATTGGC-TTCGATGTCGAGGGGT

                                                                                 Section 258
                                                        (19019) 19019      19030     19040     19050     19060     19070     19080   19092
Homo sapiens chromosome 13 NC_000013.11: 34882059... (18145) TTAAAACTA-TAGAGAATA-GTTGGTATAACTGTAAAAT-ATAG--AGCCTCTTCCCAAGGT-TAAAATGGGTT
SARS-CoV-2 Reference Genome NC_045512.2 (18320) GTCATGCTACTAGAGAAGCTGTTGGTACCAATTTACCTTTACAGCTAGGTTTTTCTACAGGTGTTAACCTAGTT

                                                                                 Section 259
                                                        (19093) 19093 19100     19110     19120     19130     19140     19150          19166
Homo sapiens chromosome 13 NC_000013.11: 34882059... (18213) GTTATATTTTGTCTTTCACACACACAAAAAAGACCTCCTGCTTCTTTTTCCTTCAAGCAGGGAAAAGTGGCAAG
SARS-CoV-2 Reference Genome NC_045512.2 (18394) GCTGTA------CCTACAGGTTATGTTGATACACCTAATAATACAGATTT-TTCCAG---------------AG
```

Section 260

	(19167)	19167 19180 19190 19200 19210 19220 19230 19240
Homo sapiens chromosome 13 NC_000013.11: 34882059...	(18287)	TCTGTCTTGAACCATTG--TGCCTTTCACATTCTCCTCCCCAAAGCCATTTAAGCTCCTTCGACCTGCTTCTCT
SARS-CoV-2 Reference Genome NC_045512.2	(18446)	TTAGTGCTAAACCACCGCCTGGAGATCAATTTAAACACCTCATA-CCACTTATGTACAAAGGACTTCCTTGGA-

Section 261

	(19241)	19241 19250 19260 19270 19280 19290 19300 19314
Homo sapiens chromosome 13 NC_000013.11: 34882059...	(18359)	ATTTCAAGAGG-TCTTTTCTTTACATTTGCATAACTTAAAGG--TATCTATCTCCATAACAAAGAAACCCATTG
SARS-CoV-2 Reference Genome NC_045512.2	(18518)	ATGTAGTGCGTATAAAGATTGTACAAATGT-TAAGTGACACACTTAAAAATCTCTCTGACAGAGTCGTATTTGT

Section 262

	(19315)	19315 19320 19330 19340 19350 19360 19370 19388
Homo sapiens chromosome 13 NC_000013.11: 34882059...	(18430)	CCT---GGAAGGTGTGTTGGAGTTTCCACTTCTCACCTAATAA---AGACTTCTTGCAGTTTTTAAATAATGAA
SARS-CoV-2 Reference Genome NC_045512.2	(18591)	CTTATGGGCACATGGCTTTGAGTTGACATCTATGAAGTATTTTGTGAAAATAGGACCTGAGCGCACCTGTTGTC

Section 263

	(19389)	19389 19400 19410 19420 19430 19440 19450 19462
Homo sapiens chromosome 13 NC_000013.11: 34882059...	(18498)	-ATGTGAGAGACTGGGGAGA-----TTACACTCTGTCATCCATGCCTCCGGCTTTTTTCAAAACCC--ATGGGT
SARS-CoV-2 Reference Genome NC_045512.2	(18665)	TATGTGATAGACGTGCCACATGCTTTTCCACTGCTTCAGACA--CTTATGCCTGTTGGCATCATTCTATTGGAT

Section 264

	(19463)	19463 19470 19480 19490 19500 19510 19520 19536
Homo sapiens chromosome 13 NC_000013.11: 34882059...	(18564)	CTGGGAAA---TCTGATACAATTCTGCATAGTCTGCAAGACATAGTGCTATGCATTTATTGCATTCAAGTTGTT
SARS-CoV-2 Reference Genome NC_045512.2	(18737)	TTGATTACGTCTATAATCCGTTTATGATTGATGTTCAACA-ATGGGGTTTTACAGGTAACCTACA-AAGCAACC

Section 265

	(19537)	19537 19550 19560 19570 19580 19590 19600 19610
Homo sapiens chromosome 13 NC_000013.11: 34882059...	(18635)	TTCATCCAATTG---AGTGC-TGACTGTGTACCTGGCTCTGTCTGTCAATGAACAACAAAGACAAAACTTTCT
SARS-CoV-2 Reference Genome NC_045512.2	(18809)	ATGATCTGTATTGTCAAGTCCATGGTAATGCACATGTAGCTAGTTGTGATGCAATCATGACTAGGTGTCTAGCT

Section 266

	(19611)	19611 19620 19630 19640 19650 19660 19670 19684
Homo sapiens chromosome 13 NC_000013.11: 34882059...	(18705)	CCCCTCATCCAACTAGTAAAAGGAGCAC-CTGTCCTGAACAAAATGAGGAAGTTAAATGTGTATTATATAGCTG
SARS-CoV-2 Reference Genome NC_045512.2	(18883)	GTCCACGAGTGCTTTGTTAAGCGTGTTGACTGGACTATTGAATATCCTATAATTGGTGATGAACTGAAGATTAA

```
                                                                Section 267
                                              (19685) 19685 19690     19700     19710     19720     19730     19740             19758
Homo sapiens chromosome 13 NC_000013.11: 34882059... (18778) TGAATACTATTAAAAAAAAAAAAGGAAAAAGGGGAGATTTGGAGCTAGGGTAGAGAGAGCTGTAAGTCATTCAG
    SARS-CoV-2 Reference Genome NC_045512.2 (18957) TGCGGCTTGTAGAAAGGTTCAACACATGGTTGTTAAAGCTGCATTATTAGCAGACAAAT-TCCCAGTTCTTCAC
                                                                Section 268
                                              (19759) 19759      19770     19780     19790     19800     19810     19820        19832
Homo sapiens chromosome 13 NC_000013.11: 34882059... (18852) G-CAAGTT--CC--AAAGGAAGGAAGAGTGT---TACAGGCAAAGACTAATGGAAGGAACAGGCCTGGAGACT
    SARS-CoV-2 Reference Genome NC_045512.2 (19030) GACATTGGTAACCCTAAAGCTATTAAGTGTGTACCTCAAGCTGATGTAGAATGGAAGTTCTATGA-TGCACAGC
                                                                Section 269
                                              (19833) 19833   19840     19850     19860     19870     19880     19890          19906
Homo sapiens chromosome 13 NC_000013.11: 34882059... (18918) GGAGCA-TGCCTGGGGTTTTAGAAGAACTGCAAGGAAGCTAAAGATAAGAACCCAGAAACT-ACAGCA-CTTAG
    SARS-CoV-2 Reference Genome NC_045512.2 (19103) CTTGTAGTGACAAAGCTTATAAAATAGAAG-AATTATTCTATTCTTATGC-CACACATTCTGACAAATTCACAG
                                                                Section 270
                                              (19907) 19907        19920     19930     19940     19950     19960     19970   19980
Homo sapiens chromosome 13 NC_000013.11: 34882059... (18989) AACTCCTATGACTAATATATGATTTCGCATTTTCTTTTTTTATTTTATTTTATTTTATTTTATTTATTTATTT
    SARS-CoV-2 Reference Genome NC_045512.2 (19175) ATGGTGTATGCCTATTTTGGAATT--GCAATGTCGATAGATATCCTGCTA-ATTCCATTGT---TTGTAGATTT
                                                                Section 271
                                              (19981) 19981    19990     20000     20010     20020     20030     20040        20054
Homo sapiens chromosome 13 NC_000013.11: 34882059... (19063) ATTTATTTATTATTATTATACTTTAAGTTTTAGGGTACATGTGCACAATGTGCAGGTTACTTACATATGTATAC
    SARS-CoV-2 Reference Genome NC_045512.2 (19243) GACACTAGAGTGCTATCTAACCTTAACTTGCCTGGT---TGTG-ATGGTG-GCAGTTTG--TATGTAAATAAAC
                                                                Section 272
                                              (20055) 20055 20060     20070     20080     20090     20100     20110             20128
Homo sapiens chromosome 13 NC_000013.11: 34882059... (19137) ATGTGCCATGCTGGTGTGTTGCACCCACTAACTTGTCATCTAGCATTAGGTATATCTCCCAATGCTATCCCTCC
    SARS-CoV-2 Reference Genome NC_045512.2 (19310) ATG---CATTC----------CACACACCAGCTTTTGATAAA-----AGTGCTTTTGTTAATTTAAAACAATTA
                                                                Section 273
                                              (20129) 20129      20140     20150     20160     20170     20180     20190        20202
Homo sapiens chromosome 13 NC_000013.11: 34882059... (19211) CCCCTCCCCCCACCCCACAACAGTCCCCA-GAGTGTGATGTTCCCCTTCCTGTGTCATGGGTTCTCATTGTTT
    SARS-CoV-2 Reference Genome NC_045512.2 (19366) CCATTTTTCTATTACTCTGACAGTCCATGTGAGTCTCATGGAAAACAAGTAGTGTCA---GATATAGATTATGT
```

Section 274

```
                                                          (20203) 20203  20210     20220     20230     20240     20250     20260       20276
Homo sapiens chromosome 13 NC_000013.11: 34882059...      (19284) AATTCCCACCTATGAGTGAGAATATGCGGTGTTTGGTTTTTTGTTCTTGCGATAGTTTAC-TGAAAATGATGAT
SARS-CoV-2 Reference Genome NC_045512.2                   (19437) ACCACTAAAGTCTGC-TACGTGTATAACACGTTGCAATTTAGGTGGTGCTGTCTGTAGACATCATGCTAATGAG
```

Section 275

```
                                                          (20277) 20277        20290     20300     20310     20320     20330     20340 20350
Homo sapiens chromosome 13 NC_000013.11: 34882059...      (19357) TTCCAATTTCATCCATGTCCCTACAA-AGGACGTGAACTCATCATTTTTAAGCTTTC---TTGAC------CCT
SARS-CoV-2 Reference Genome NC_045512.2                   (19510) TACAGATTGTATCTCGATGCTTATAACATGA--TGATCTCAGCTGGCTTTAGCTTGTGGGTTTACAAACAATTT
```

Section 276

```
                                                          (20351) 20351    20360     20370     20380     20390     20400     20410       20424
Homo sapiens chromosome 13 NC_000013.11: 34882059...      (19421) GTTGCATCTGAGATCTCTAAGCTGTCTTAAGAACTCCGTAGCACACAAAAGGAG--TTAGAAGCATCC---TTC
SARS-CoV-2 Reference Genome NC_045512.2                   (19582) GATACTTATAACCTCTGGAACACTTTTACAAGACTTCAGAGTTTAGAAAATGTGGCTTTTAATGTTGTAAATAA
```

Section 277

```
                                                          (20425) 20425 20430     20440     20450     20460     20470     20480           20498
Homo sapiens chromosome 13 NC_000013.11: 34882059...      (19490) AGCATTCCCTGAAGCTTAGGCCAGTGTATTCTGAGCAACCATTTCTTCTAAATGCATAGACCCTCA---TGAAA
SARS-CoV-2 Reference Genome NC_045512.2                   (19656) GGGACACTTTGATGGACAACAGGGTGAAGTACCAGTTTCTATCATTAATAACACTGTTTACACAAAAGTTGATG
```

Section 278

```
                                                          (20499) 20499      20510     20520     20530     20540     20550     20560     20572
Homo sapiens chromosome 13 NC_000013.11: 34882059...      (19561) CCTTTTTTGGATCTATTTTTGTTTTTCTTAC--CCCTGGCTGTGTTCGTA--ATT---GCTTCACGATGACATC
SARS-CoV-2 Reference Genome NC_045512.2                   (19730) GTGTTGATGTAGAATTGTTTGAAAATAAAACAACATTACCTGTTAATGTAGCATTTGAGCTTTGGGCTAAGCGC
```

Section 279

```
                                                          (20573) 20573  20580     20590     20600     20610     20620     20630         20646
Homo sapiens chromosome 13 NC_000013.11: 34882059...      (19628) TCATTGACAGATGCAAGGCTGCTGACACCACGCCAGACTCTCTGAAATGAAATCATTTGACACCTGAGGGATC
SARS-CoV-2 Reference Genome NC_045512.2                   (19804) AACATTAAACCAGTACCAGAGGTGAAAATACTCAATAATTTGGGTGTGGACATTGCTGCTAA--TACTGTGATC
```

Section 280

```
                                                          (20647) 20647        20660     20670     20680     20690     20700     20710 20720
Homo sapiens chromosome 13 NC_000013.11: 34882059...      (19702) TGGGCCTTCTGGACTCTTCATTCCTGGCTGAGAACT-CTGT-GCCCTACTTTGAGGGTTTGAACCCTCAGGAA
SARS-CoV-2 Reference Genome NC_045512.2                   (19876) TGGGACTACAAAAGAGATGCTCCAGCACATATATCTACTATTGGTGTTTGTTCTATGACTGA---CATAGCCAA
```

```
                                                              Section 281
                                                     (20721) 20721    20730     20740     20750     20760     20770     20780        20794
Homo sapiens chromosome 13 NC_000013.11: 34882059... (19774) GAAGCTAACTTAGGA-ATTCTCGTTGCCCTCATTGCTCATCTCTGA-GCTACCTTAGCACCTCAAAAAGGGCCC
   SARS-CoV-2 Reference Genome NC_045512.2 (19947) GAAACCAACTGAAACGATTTGTGCACCACTCACTG-TCTTTTTTGATGGTAGAGTTGATGGTCAAGTAGACTTA
                                                              Section 282
                                                     (20795) 20795 20800     20810     20820     20830     20840     20850           20868
Homo sapiens chromosome 13 NC_000013.11: 34882059... (19846) GCAAATCCTGTCCCT-----T--TCTTTATTCACAAA----CCTCAAATTTTGGTGACAGAGTGCAGGTGCC--
   SARS-CoV-2 Reference Genome NC_045512.2 (20020) TTTAGAAATGCCCGTAATGGTGTTCTTATTACAGAAGGTAGTGTTAAAGGTTTACAACCATCTGTAGGTCCCAA
                                                              Section 283
                                                     (20869) 20869      20880     20890     20900     20910     20920     20930        20942
Homo sapiens chromosome 13 NC_000013.11: 34882059... (19907) ACAGG---GACTTA---GAGTCA---TGGTTGAACAAGG-----AAGGGCTGTACGTTCAGATGCCTACAGAGG
   SARS-CoV-2 Reference Genome NC_045512.2 (20094) ACAAGCTAGTCTTAATGGAGTCACATTAATTGGAGAAGCCGTAAAAACACAGTTCAATTATTATAAGAAAGTTG
                                                              Section 284
                                                     (20943) 20943  20950     20960     20970     20980     20990     21000          21016
Homo sapiens chromosome 13 NC_000013.11: 34882059... (19967) CTACTCCGGTCACATATGTCAGCGAGAAGTTAGATAAGTGAGA-------TCCACAGCAATTGTGAGCTTGTGT
   SARS-CoV-2 Reference Genome NC_045512.2 (20168) ATGGTGTTGTCCAACAATTACCTGA-AACTTACTTTACTCAGAGTAGAAATTTACAAGAATTTAAACCCAG-GA
                                                              Section 285
                                                     (21017) 21017        21030     21040     21050     21060     21070     21080  21090
Homo sapiens chromosome 13 NC_000013.11: 34882059... (20034) GTCTTTAGGGGAGAGCGAATTCTCAGATCTAGCC--------AGTCATTGAAATGTGGAAATAACAGTCCAGTG
   SARS-CoV-2 Reference Genome NC_045512.2 (20240) GTCA--AATGGAAATTGATTTCTTAGAATTAGCTATGGATGAATTCATTGAACGGTATAAATTAGAAGGCTATG
                                                              Section 286
                                                     (21091) 21091    21100     21110     21120     21130     21140     21150        21164
Homo sapiens chromosome 13 NC_000013.11: 34882059... (20100) TTTCCAAATCTTATCGTAAAAG-ACAAGACAGCAAT--CCAAATATTTGTATGAAATTTCCTGAT--------G
   SARS-CoV-2 Reference Genome NC_045512.2 (20312) CCTTCGAA-CATATCGTTTATGGAGATTTTAGTCATAGTCAGTTAGGTGGTTTACATCTACTGATTGGACTAGC
                                                              Section 287
                                                     (21165) 21165 21170     21180     21190     21200     21210     21220           21238
Homo sapiens chromosome 13 NC_000013.11: 34882059... (20163) T-----TTTAATGCTGACAACTAGT-AATCCCCCCAAAACAAACAAACAAACAA-ACAAACAAAACCACTGTTG
   SARS-CoV-2 Reference Genome NC_045512.2 (20385) TAAACGTTTTAAGGAATCACCTTTTGAATTAGAAGATTTTATTCCTATGGACAGTACAGTTAAAA--ACTATTT
```

Section 288

(21239) 21239 21250 21260 21270 21280 21290 21300 21312
Homo sapiens chromosome 13 NC_000013.11: 34882059... (20230) AATTACAC-----CAAACAAGAGCTATCTGTA-GCCTGGGTTCTGCCTGGGGGCTCTCATTCTATGACTTC--T
SARS-CoV-2 Reference Genome NC_045512.2 (20457) CATAACAGATGCGCAAACAGGTTC-ATCTAAGTGTGTGTGTTCTGTTATTGATTTATTACTTGATGATTTTGTT

Section 289

(21313) 21313 21320 21330 21340 21350 21360 21370 21386
Homo sapiens chromosome 13 NC_000013.11: 34882059... (20296) GAAACAATCATCTTTGATCCTGTCACTC-ATTGTCCA----TTTCATGGTGTTTGTGGGAGTTTAAAATTTTTT
SARS-CoV-2 Reference Genome NC_045512.2 (20530) GAAATAATAAAATCCCAAGATTTATCTGTAGTTTCTAAGGTTGTCAAAGTGACTATTGACTATACAGAAATTTC

Section 290

(21387) 21387 21400 21410 21420 21430 21440 21450 21460
Homo sapiens chromosome 13 NC_000013.11: 34882059... (20365) AAATAAGATGTGGAACAAGGATGGCAAATAATTTCTAAATTGCATGTCACCTCTGATTTATTAGCAGTGGCTGC
SARS-CoV-2 Reference Genome NC_045512.2 (20604) ATTTATGCTTTGGTGTAAAGATGGCCA----TGTAGAAA---CATTTTACC-CAAAATTACAATCTA-GTCAAG

Section 291

(21461) 21461 21470 21480 21490 21500 21510 21520 21534
Homo sapiens chromosome 13 NC_000013.11: 34882059... (20439) CGTGAGCACTACATTTAGGATTCTGAGGCTGTTTCTAGGTCTGCAGAAAAAGCAT-GTGTTAAATTATCAATGT
SARS-CoV-2 Reference Genome NC_045512.2 (20669) CGTGGCAACCG------GG----TGTTGCTATGCCTAA-TCTTTACAAAATGCAAAGAATGCTATTAGAAAAGT

Section 292

(21535) 21535 21540 21550 21560 21570 21580 21590 21608
Homo sapiens chromosome 13 NC_000013.11: 34882059... (20512) TGAATGATGGTACACAAGGCCTGTATTTGCAA--TTCCCCAACCAAACAATCCCCATGAAGGTTAGTGTTGGTG
SARS-CoV-2 Reference Genome NC_045512.2 (20732) GTGACCTTCAAAATTATGG--TGATAGTGCAACATTACCTAAAGGCATAATG---ATGAATGTCGCAAAATATA

Section 293

(21609) 21609 21620 21630 21640 21650 21660 21670 21682
Homo sapiens chromosome 13 NC_000013.11: 34882059... (20584) CTTGAATAAATGATTTCAGTTACTCCTCCTCCTTT-CTTAGCTCTTTAATTCAAGTGTGCTTCTCTCATCTACT
SARS-CoV-2 Reference Genome NC_045512.2 (20801) CTCAACTGTGTCAATATTTAAACACATTAACATTAGCTGTACCCTATAATATGAGAGTTATACATTTTGGTGCT

Section 294

(21683) 21683 21690 21700 21710 21720 21730 21740 21756
Homo sapiens chromosome 13 NC_000013.11: 34882059... (20657) GTTCCCCA---AGGCCTGGGAAAATGGAGCACTGACTTTCAGCGAATGGCGGAGGCGGGGGTGGGGTGGGGGTC
SARS-CoV-2 Reference Genome NC_045512.2 (20875) GGTTCTGATAAAGGAGTTGCACCAGGTACAGCTGT-TTTAAGACAGTGGTTGCCTACGGG-TACGCTGCTTGTC

Section 295

(21757) 21757 21770 21780 21790 21800 21810 21820 21830

Homo sapiens chromosome 13 NC_000013.11: 34882059... (20728) CTGCCAGGTCATCAGGTGTCTTTGCTTATAACTCATGAGGCATAAATCAAGCCAATGCATTCAACACTACAGGA

SARS-CoV-2 Reference Genome NC_045512.2 (20947) GATTCAGATCTTAA--TGACTTTGT------CTC-TGATGCAGA---------------TTCAACTTTGATTG-

Section 296

(21831) 21831 21840 21850 21860 21870 21880 21890 21904

Homo sapiens chromosome 13 NC_000013.11: 34882059... (20802) GGAGAGTAACTGATCTATCACTCAGACACCTGTTATAATTTTTTCAGATTTCTTGTGAT--GTAAAAATATCAG

SARS-CoV-2 Reference Genome NC_045512.2 (20996) -----GTGATTGTGCAACTGTACATACAGCTAATA-AATGGGATCTCATTATTAGTGATATGTACGACCCTAAG

Section 297

(21905) 21905 21910 21920 21930 21940 21950 21960 21978

Homo sapiens chromosome 13 NC_000013.11: 34882059... (20874) AGCAAAGTTGTACTACTCTAGACCAATCAGGCTCCAGATGATAAATATTTTAATCTGAGTTGGTATTCTAGCTC

SARS-CoV-2 Reference Genome NC_045512.2 (21064) ACTAAAAATGT--TACAAAAGAA-AATGACTCTAAAGAGGGTTTTTTCACTTACATTTGTGGGT-TTATACAAC

Section 298

(21979) 21979 21990 22000 22010 22020 22030 22040 22052

Homo sapiens chromosome 13 NC_000013.11: 34882059... (20948) ATTTTAATATATCAAAATGAGTTTACTTTGGAATAAAAATGACCTTAAATACAA--ATTTCAGATACTT-TATT

SARS-CoV-2 Reference Genome NC_045512.2 (21134) AA---AAGCTAGCTCTTGGAGGTTCCGTGGCTATAAAGATAACAGAACATTCTTGGAATGCTGATCTTTATAAG

Section 299

(22053) 22053 22060 22070 22080 22090 22100 22110 22126

Homo sapiens chromosome 13 NC_000013.11: 34882059... (21019) TTAAAGAGAAAC----AATAAAAACCAACACTGGAAAACAACCTA-------CAACAT-TTAATTATTTTTAGT

SARS-CoV-2 Reference Genome NC_045512.2 (21205) CTCATGGGACACTTCGCATGGTGGACAGCCTTTGTTACTAATGTGAATGCGTCATCATCTGAAGCATTTTTAAT

Section 300

(22127) 22127 22140 22150 22160 22170 22180 22190 22200

Homo sapiens chromosome 13 NC_000013.11: 34882059... (21081) --GTTTTAGTTCTTGATAGGGAAACTGAACCCAAGAAAGTAATTGAAAAATGTTATCCTCCACAAGTA--TATC

SARS-CoV-2 Reference Genome NC_045512.2 (21279) TGGATGTAATTATC--TTGGCAAACC-ACGCGAACAAATAGATGGTTA--TGTCATGCATGCAAATTACATATT

Section 301

(22201) 22201 22210 22220 22230 22240 22250 22260 22274

Homo sapiens chromosome 13 NC_000013.11: 34882059... (21151) TTAGAGGTAAGAGCATTTGAATGTTAAGAAATATATATTTTTAAACGTCCAC--TTATGTTTACTTTGGAAGGA

SARS-CoV-2 Reference Genome NC_045512.2 (21348) TTGGAGG-AATACAAATCCAATTCAGTTGTCTTCCTATTCTTTATTTGACATGAGTAAATTTCCCCTTAAATTA

Section 302

```
                                                  (22275) 22275 22280     22290     22300     22310     22320     22330            22348
Homo sapiens chromosome 13 NC_000013.11: 34882059... (21223) ATTTCTTATAGAAAAATGCTAGCAAATAGATAAGTTCTTACCTACTAATTCACTCTGTTTTCTCATAATCTTCT
SARS-CoV-2 Reference Genome NC_045512.2 (21421) AGGGGTACTGCTGTTATGTCTTTAAA---AGAAGGTCAAATCAATGATATGATTTTATCTCTTCTTAGTAAA--
```

Section 303

```
                                                  (22349) 22349      22360     22370     22380     22390     22400     22410        22422
Homo sapiens chromosome 13 NC_000013.11: 34882059... (21297) GACAAAATTTCAGTCAAAGTAATCAATGGTCTGCAAGCTCCCAGTTTCCAACAATTGCTAACTCAGTAACTAAC
SARS-CoV-2 Reference Genome NC_045512.2 (21490) GGTAGACTTATAATTAGAGAAAACAACAGAGTTGTTATTTCTAGTGATGTTC--TTGTTAACAACTAAACGAAC
```

Section 304

```
                                                  (22423) 22423  22430     22440     22450     22460     22470     22480           22496
Homo sapiens chromosome 13 NC_000013.11: 34882059... (21371) ATCAACTACTTGAGTCAATAGAATAGGTAGTAGTTGGCCTATATGTGATTTTAGACCACATATAGAGGATTTGA
SARS-CoV-2 Reference Genome NC_045512.2 (21562) AATGTTTGTTTTTCTTGTTTTATT-GCCACTAGTCT-CTAGTCAGTGTGTTAATCTTACAACCAGA--ACTCAA
```

Section 305

```
                                                  (22497) 22497        22510     22520     22530     22540     22550     22560 22570
Homo sapiens chromosome 13 NC_000013.11: 34882059... (21445) TATAAAAAAATCTCCCCACAATGCCCCAAATCTCTTCTTCATCAACACGATGTTGATGCCTGAAACTCAGCAAT
SARS-CoV-2 Reference Genome NC_045512.2 (21632) TTACCCCCTGCATACACTAATTCTTTCACACGTGGTGTTTATTACCCTGACAAAGTTTTCAGATCCTCAGTTTT
```

Section 306

```
                                                  (22571) 22571    22580     22590     22600     22610     22620     22630         22644
Homo sapiens chromosome 13 NC_000013.11: 34882059... (21519) -CAGACTCCTTCTGAATT-TTTATAATTCATTATTGCAATATTGTTTAATTGGAGTATCAAAATGTCATTGTCT
SARS-CoV-2 Reference Genome NC_045512.2 (21706) ACATTCAACTCAGGACTTGTTCTTACCTTTCTTTTCCAATGTTACTTGGTTCCATGCTATACATGTCTCTGGGA
```

Section 307

```
                                                  (22645) 22645 22650     22660     22670     22680     22690     22700            22718
Homo sapiens chromosome 13 NC_000013.11: 34882059... (21591) TTGAGACTATTAAAAA-----ATAGTGCTTCAATATAAACTATTTATTGTCTACAGTACATTCTTCCATTTATT
SARS-CoV-2 Reference Genome NC_045512.2 (21780) CCAATGGTACTAAGAGGTTTGATAACCCTGTCCTACCATTTAATGATGGTGTTTATTT--TGCTTCCACTGAGA
```

Section 308

```
                                                  (22719) 22719      22730     22740     22750     22760     22770     22780        22792
Homo sapiens chromosome 13 NC_000013.11: 34882059... (21660) TGATTCTGATTATATAGATTTCTTCCAATTGTAACT--TGTAGGTAGCTAATGGCATTGCAAAATAATTTTTGT
SARS-CoV-2 Reference Genome NC_045512.2 (21852) AGTCTAACATAATA-AGAGGCTGGATTTTTGGTACTACTTTAGATTCGAAGACCCAGTCCC---TACTTATTGT
```

Section 309

(22793) 22793 22800 22810 22820 22830 22840 22850 22866
Homo sapiens chromosome 13 NC_000013.11: 34882059... (21732) CCATAGACATGCAACTGAACTGTTCTCCCGCTGTGGAAAAAATAGTTTTGTC----TCCATTT--GCACATTTA
SARS-CoV-2 Reference Genome NC_045512.2 (21922) TAATAACGCTACTAATGT--TGTTATTAAAGTCTGTGAATTTCAATTTTGTAATGATCCATTTTTGGGTGTTTA

Section 310

(22867) 22867 22880 22890 22900 22910 22920 22930 22940
Homo sapiens chromosome 13 NC_000013.11: 34882059... (21800) TTTCAATAGTCTTGATCTAGAAATGTGTCTGTTTAATTGTTTGTTTTTGGATTTTCTTTTGAATTGCTTATAAT
SARS-CoV-2 Reference Genome NC_045512.2 (21994) TTACCACAAA----AACAACAAAAGTTG--GATGGAAAGTGAGTTCA--GAGTTTATTCT--AGTGCGAATAAT

Section 311

(22941) 22941 22950 22960 22970 22980 22990 23000 23014
Homo sapiens chromosome 13 NC_000013.11: 34882059... (21874) ATCTGCCTAGT-TCTCTCACTGAACAATGAGTTAAGTAAGATCTTT-AATACATGTTTGCTTTATATCTGTATG
SARS-CoV-2 Reference Genome NC_045512.2 (22058) TGCACTTTTGAATATGTCTCTCAGCCTTTTCTTATGGACCTTGAAGGAAAACAGGGTAATTTCAAAAATCTTAG

Section 312

(23015) 23015 23020 23030 23040 23050 23060 23070 23088
Homo sapiens chromosome 13 NC_000013.11: 34882059... (21946) AGTCATGATTTTACTTCTTTTTGAAAATTATCTTTTAATGATATTTTCCTATTTGATCAATATAAATTCTTGAC
SARS-CoV-2 Reference Genome NC_045512.2 (22132) GGAATTTGTGTTTAAGAATATTGATGGTTAT-TTTAAA--ATATATTCTAAGCACACGCCTATTAATTTAGTGC

Section 313

(23089) 23089 23100 23110 23120 23130 23140 23150 23162
Homo sapiens chromosome 13 NC_000013.11: 34882059... (22020) AAGTAATTCC--AATATTTATCCACATTTATGAAAAAAGACCCTTTGA-AAAAGTAAGAAACTTGATTAATAAT
SARS-CoV-2 Reference Genome NC_045512.2 (22203) GTGATCTCCCTCAGGGTTTTTCGGCTTTAGAACCATTGGTAGATTTGCCAATAGGTATTAACATCACTAGG--T

Section 314

(23163) 23163 23170 23180 23190 23200 23210 23220 23236
Homo sapiens chromosome 13 NC_000013.11: 34882059... (22091) TTGCAATTTTATTTTATTCTACCAAAATGTTGTT-GA-TAAATGTGTTTCTGAAAAGTATATTCCACAGCTATG
SARS-CoV-2 Reference Genome NC_045512.2 (22275) TTCAAACTTTACTTGCTT-TACATAGAAGTTATTTGACTCCTGGTGATTCTTCTTC--AGGTTGGACAGCTG--

Section 315

(23237) 23237 23250 23260 23270 23280 23290 23300 23310
Homo sapiens chromosome 13 NC_000013.11: 34882059... (22163) TGTCCTGACCTATTTTATCCTTAAAATCTTATTTAACTTCAATTTTGGAAAATGTAATAAGGAAAATA-AACA
SARS-CoV-2 Reference Genome NC_045512.2 (22344) -GTGCTGCAGCTTATTATGTGGGTTATCTTCA--ACCTAGGACTTTTCTATTAAAATATAATGAAAATGGAACC

```
                                                                                    Section 316
                                                  (23311) 23311    23320     23330     23340     23350     23360     23370      23384
Homo sapiens chromosome 13 NC_000013.11: 34882059... (22236) ATTAAAAGCAATTTAAAAAGTAATTATTGAGCACGTGCTCTATTCCAGACT---TGCTCAAATATCTGACCAA
      SARS-CoV-2 Reference Genome NC_045512.2 (22415) ATTACAGATGC-TGTAGACTGTGCAC-TTGACCCTCT-CTCAGAAACAAAGTGTACGTTGAAATCCTTCACTGT
                                                                                    Section 317
                                                  (23385) 23385 23390      23400     23410     23420     23430     23440      23458
Homo sapiens chromosome 13 NC_000013.11: 34882059... (22307) GGATCCCGGTATGCGTCTCACTTTTGAAAAGCCCTTTTAAAGTCCATCTAAGAGCAAATTCATTTCTCTAGCAT
      SARS-CoV-2 Reference Genome NC_045512.2 (22486) AGAAAAAGGAATCTATCAAACTTCTAAC--------TTTAGAGTCCAACCAACAGAATCTATTGTTAGATTTCCT
                                                                                    Section 318
                                                  (23459) 23459      23470     23480     23490     23500     23510     23520   23532
Homo sapiens chromosome 13 NC_000013.11: 34882059... (22381) CTACTGAAAAAC----AGCCAGTCAAAGAATATGTT--CCACACC----CTCCAAGTGTTTCTCCAATGTAACA
      SARS-CoV-2 Reference Genome NC_045512.2 (22553) AATATTACAAACTTGTGCCCTTTTGGTGAAGTTTTTAACGCCACCAGATTTGCATCTGTTTATGCT-TGGAACA
                                                                                    Section 319
                                                  (23533) 23533   23540     23550     23560     23570     23580     23590      23606
Homo sapiens chromosome 13 NC_000013.11: 34882059... (22445) GCCACTG--TCAGCA---GGGTGACTGGGCCC-CTTCCCCAGGCTGTCACGTCTGATGCTTCC--TTCA-GTGA
      SARS-CoV-2 Reference Genome NC_045512.2 (22626) GGAAGAGAATCAGCAACTGTGTTGCTGATTATTCTGTCCTATATAATTCCGCATCATTTCCACTTTTAAGTGT
                                                                                    Section 320
                                                  (23607) 23607        23620     23630     23640     23650     23660     23670 23680
Homo sapiens chromosome 13 NC_000013.11: 34882059... (22510) CCCGG-------CCTTCTAATTCAGCTTAACCTTCTCC-CTTCAACCTCAGCCCTGTCACCAGGACAGCCTCT
      SARS-CoV-2 Reference Genome NC_045512.2 (22700) TATGGAGTGTCTCCTACTAAATTAAATGATCTCTGCTTTACTAATGTCTATGCAGATTCATTTGTAATTAGAGG
                                                                                    Section 321
                                                  (23681) 23681    23690     23700     23710     23720     23730     23740      23754
Homo sapiens chromosome 13 NC_000013.11: 34882059... (22576) CATTCCACTCAGTCCTG-CCTTCCAGTGGGAATCGGATGAGCGACTCTACATCCATGGTGGTCAAGGAAATGGG
      SARS-CoV-2 Reference Genome NC_045512.2 (22774) TGATGAAGTCAGACAAATCGCTCCAGGGCAAACTGGAAAGATTGCTGATTATA-ATTATAAATTACCAGATGAT
                                                                                    Section 322
                                                  (23755) 23755 23760      23770     23780     23790     23800     23810      23828
Homo sapiens chromosome 13 NC_000013.11: 34882059... (22649) TTGCCGTGATGTGGGAGTGGAATTCGAGGTGCAGACCTTCTATTGTTTAGAAACAGAGCTATTGA--ATCCCCC
      SARS-CoV-2 Reference Genome NC_045512.2 (22847) TTTACAGGCTGCGTTATAGC--TTGGAATTCTA-ACAATCTTGATTCTAAGGTTGGTGGTAATTATAATTACCT
```

Section 323

```
                                                      (23829) 23829      23840     23850     23860     23870     23880     23890    23902
Homo sapiens chromosome 13 NC_000013.11: 34882059... (22721) AGGGTAAACCAAAACTAAGAGTGCTGAGGA-AGACCTAGAGTCAGAGGCCGTAACACTCCCATGAATGTAGGAA
 SARS-CoV-2 Reference Genome NC_045512.2 (22918) ---GTATAGATTGTTTAGGAAGTCTAATCTCAAACCTTTTGAGAGAGATATTTCAACTGAAATCTATC-AGGCC
```

Section 324

```
                                                      (23903) 23903  23910     23920     23930     23940     23950     23960         23976
Homo sapiens chromosome 13 NC_000013.11: 34882059... (22794) AGTAACTTGAGAACAGTTGACGTAGTAAATAAAAGAGGAGCTTTTGCATGATAGCAGAATAGTCTAGAATCAGC
 SARS-CoV-2 Reference Genome NC_045512.2 (22988) GGTAGCACACCTTGTAATGGTGTTGAAGGTTTTAATTGTTACTTTCCTTTACAATCATATGGTTTCCAACCCAC
```

Section 325

```
                                                      (23977) 23977        23990     24000     24010     24020     24030     24040  24050
Homo sapiens chromosome 13 NC_000013.11: 34882059... (22868) AGTGGGAGCAAGAT---AAGCA-AGATGGCAGCACTACTCTCCTGCCCTAGAGGGATTACAGTCTTGAGAGGGA
 SARS-CoV-2 Reference Genome NC_045512.2 (23062) TAATGGTGTTGGTTACCAACCATACAGAGTAGTAGTACTTTCTT----TTGAACTTCTACATGCACCAGCAACT
```

Section 326

```
                                                      (24051) 24051   24060     24070     24080     24090     24100     24110        24124
Homo sapiens chromosome 13 NC_000013.11: 34882059... (22938) GAC---AGACCAACAAA--CAAGCAATTACAATATTGTCTGATCTGTAGATGATCAGGGAATTCTAAGGATGCC
 SARS-CoV-2 Reference Genome NC_045512.2 (23132) GTTTGTGGACCTAAAAAGTCTACTAATTTGGTTAAAAACAAATGTGTCAATT-TCA---ACTTCAATGGTTTAA
```

Section 327

```
                                                      (24125) 24125 24130     24140     24150     24160     24170     24180           24198
Homo sapiens chromosome 13 NC_000013.11: 34882059... (23007) CACCGAAGGTAAATGGATCCCAGTCTGGGTCAGTGT---GGAGTATTCACAAA--GGGAAGAATTAATGGGGA-
 SARS-CoV-2 Reference Genome NC_045512.2 (23202) CAGGCACAGGTGTTCTTACTGAGTCTAACAAAAAGTTTCTGCCTTTCCAACAATTTGGCAGAGACATTGCTGAC
```

Section 328

```
                                                      (24199) 24199      24210     24220     24230     24240     24250     24260    24272
Homo sapiens chromosome 13 NC_000013.11: 34882059... (23075) ACTGGGCACTGTTTATTCAACATGATCCAGAGA-----GAAACACTAAATATGAGCAAAATGAACTTTC-----
 SARS-CoV-2 Reference Genome NC_045512.2 (23276) ACT----ACTGATGCTGTC-CGTGATCCACAGACACTTGAGATTCTTGACATTA-CACCATGTTCTTTTGGTGG
```

Section 329

```
                                                      (24273) 24273  24280     24290     24300     24310     24320     24330         24346
Homo sapiens chromosome 13 NC_000013.11: 34882059... (23139) ---CAGT-------CACTGGGAAAGCATCTCTGTAGGTGAAGTAGAAATGATTGAATAAGCATCGCCACAGAGC
 SARS-CoV-2 Reference Genome NC_045512.2 (23344) TGTCAGTGTTATAACACCAGGAACAAATACTTCTAACC-AGGTTGCTGTTCTTTA-TCAGGATGTTAACTGCAC
```

Section 330

	(24347)	24347 24360 24370 24380 24390 24400 24410 24420
Homo sapiens chromosome 13 NC_000013.11: 34882059...	(23203)	A-ATGAATCTCTC-CCATGCTTACTGATCAACATTCTGATTATTAGTGTATCT-TCCTGGGTACCCTAATCTCA
SARS-CoV-2 Reference Genome NC_045512.2	(23416)	AGAAGTCCCTGTTGCTATTCATGCAGATCAACTTACTCCTACTTGGCGTGTTTATTCTACAGGTTCTAATGTTT

Section 331

	(24421)	24421 24430 24440 24450 24460 24470 24480 24494
Homo sapiens chromosome 13 NC_000013.11: 34882059...	(23274)	GCAAAGGAACATCCTGGATGGGGATACATGCACTCCAG--CAACTAGAACTGGATATCTCCCACTATAAATTA
SARS-CoV-2 Reference Genome NC_045512.2	(23490)	TTCAAACA-CGTGCAGGCTGTTTAATAGGGGCTGAACATGTCAACAACTCATATGAGTGTGACATACCCATTGG

Section 332

	(24495)	24495 24500 24510 24520 24530 24540 24550 24568
Homo sapiens chromosome 13 NC_000013.11: 34882059...	(23346)	CCCAGGACTCTT-GCGAGTAGATTGCCATGAATCTTCTTTCCTGAGGCTTTTCCACTTTGAGAAATT-GACAGG
SARS-CoV-2 Reference Genome NC_045512.2	(23563)	TGCAGGTATATGCGCTAGTTATCAGAC-TCAGACTAATTCTCCTCGGCGGG--CACGTAGTGTAGCTAGTCAAT

Section 333

	(24569)	24569 24580 24590 24600 24610 24620 24630 24642
Homo sapiens chromosome 13 NC_000013.11: 34882059...	(23418)	GC-TGAAGGCC-AGACTGGGGGCCTTTCTAAATTCACCAAAGGTAATGCCACCTGTGAGGCTTCTTGGCAG---
SARS-CoV-2 Reference Genome NC_045512.2	(23634)	CCATCATTGCCTACACTATGTCACTTGGTGCAGAAAATTCAGTTGCTTACTCTAATAACTCTA-TTGCCATACC

Section 334

	(24643)	24643 24650 24660 24670 24680 24690 24700 24716
Homo sapiens chromosome 13 NC_000013.11: 34882059...	(23487)	-GCAGTTTTCAGCATTCTCCTGGAAGCACCCTAACCTTCTCTACAGCGCCTTTGA-----AATTTTTAACAATG
SARS-CoV-2 Reference Genome NC_045512.2	(23707)	CACAAATTTTACTATTAG--TGTTACCACAGAAA--TTCTAC-CAGTGTCTATGACCAAGACATCAGTAGATTG

Section 335

	(24717)	24717 24730 24740 24750 24760 24770 24780 24790
Homo sapiens chromosome 13 NC_000013.11: 34882059...	(23555)	GGCATAGTGAATATGAATTAGATGTCACT-ATGTCACCAATCAGCTCTGTGGAAACATAAAACCTCTTTGTTTT
SARS-CoV-2 Reference Genome NC_045512.2	(23776)	TACAATGTACATTTGTGGT-GATTCAACTGAATGCAGCAATCT--TTTGTTGCAATATGGCAGTT-TTTGTACA

Section 336

	(24791)	24791 24800 24810 24820 24830 24840 24850 24864
Homo sapiens chromosome 13 NC_000013.11: 34882059...	(23628)	GATGGCTACACATACTTTTATTTGACAAGCCCTGTAACTGCTCAGCACCAGA-AAAACTAAATTGGTCCATTCA
SARS-CoV-2 Reference Genome NC_045512.2	(23846)	CAATTAACAC-CGTGCTTTAACTGGAATAGCTGTTGAACAAGACAAAACACCCAAGAAGTTTTTGCACAAGTCA

Section 337

(24865) 24865 24870 24880 24890 24900 24910 24920 24938

Homo sapiens chromosome 13 NC_000013.11: 34882059... (23701) GAAAACTTGGTCCAACCTAGTAAATTTTTCAGTTTCTGCTAGCTTAAAATTGAG-CATCTCTCAGGTAAATATT

SARS-CoV-2 Reference Genome NC_045512.2 (23919) AACAAATT--TACAAAACACCACCAATTAAAGATTTTGGTGGTTTTAATTTTTCACAAATATTACCAGATCCAT

Section 338

(24939) 24939 24950 24960 24970 24980 24990 25000 25012

Homo sapiens chromosome 13 NC_000013.11: 34882059... (23774) CAATCAAATGCAAGTG-TAATTCTGTCACTGTCCAGTTCAATGCTCGTTCTCTTCCTACAATCCCTCCACTGTC

SARS-CoV-2 Reference Genome NC_045512.2 (23991) CAAAACCAAGCAAGAGGTCATTTATTGAAGATCTACTTTTCAACAAAGTGACA-CTTGCAGATGCTGGCTTCAT

Section 339

(25013) 25013 25020 25030 25040 25050 25060 25070 25086

Homo sapiens chromosome 13 NC_000013.11: 34882059... (23847) CATCCAACTCTCAGGAGGCCTCTGAGAGGCCTCTGGCCCAACACCTGTGCAGTGGAAGACCTAAAATTAAGACC

SARS-CoV-2 Reference Genome NC_045512.2 (24064) CAAACAATATGGTGATTGCCTTGGTGATATTGCTG-CTAGAGACCT---CATTTGTGCACAAAAGTTTAACGGC

Section 340

(25087) 25087 25100 25110 25120 25130 25140 25150 25160

Homo sapiens chromosome 13 NC_000013.11: 34882059... (23921) CAATACTATGT-GCTGCCTT----GACATCTGGAAAAATCAGCAGGACCTCAAATGGCCTGATCCCAAGT----

SARS-CoV-2 Reference Genome NC_045512.2 (24134) CT-TACTGTTTTGCCACCTTTGCTCACAGATGAAATGATTGCTCAATACACTTCTGCACTGTTAGCGGGTACAA

Section 341

(25161) 25161 25170 25180 25190 25200 25210 25220 25234

Homo sapiens chromosome 13 NC_000013.11: 34882059... (23986) TCACCTCCTGCT---CCCATGGTTAAAGTCCCCCAGTCCAAAAAT--TCTCCTTAGCCAGTGGACCAGGCACAG

SARS-CoV-2 Reference Genome NC_045512.2 (24207) TCACTTCTGGTTGGACCTTTGGTGCAGGTGCTGCATTACAAATACCATTTGCTATGCAAATGGCTTAT--AGGT

Section 342

(25235) 25235 25240 25250 25260 25270 25280 25290 25308

Homo sapiens chromosome 13 NC_000013.11: 34882059... (24055) TTTCTGCTTATCCCTAAGTAGTGGGTTTCAGTTCCCAGCCTACCTAAGGAATTATT-CACACAAACCAATCACA

SARS-CoV-2 Reference Genome NC_045512.2 (24279) TTAATGGTATTGG--AGTTACACAGAATGTTCTCTATGAGAACCAAAA-ATTGATTGCCAACCAATTTAATAGT

Section 343

(25309) 25309 25320 25330 25340 25350 25360 25370 25382

Homo sapiens chromosome 13 NC_000013.11: 34882059... (24128) TCCTCTCACAGAAATCAGGAGTCACCTCACCCTCTTGATACTACAGTCTGCCTT-TCCCAGCCCTGGCTCGTTC

SARS-CoV-2 Reference Genome NC_045512.2 (24350) GCTATTGGCAAAATTCAAGACTCACTTTCTTCCACAGCAAGTGCACTTGGAAAACTTCAAGATGTGG-TCAACC

Section 344

(25383)		25383 25390 25400 25410 25420 25430 25440 25456
Homo sapiens chromosome 13 NC_000013.11: 34882059...	(24201)	ACTCAGTTTTCGAGTGGAACTCTCATGTGACCCTGCATGGCGTGCTGTGTTCTCCTTCCCCAGGTTATGAGGAC
SARS-CoV-2 Reference Genome NC_045512.2	(24423)	AAAATGCACAAGCTTTAAACACGCTTGTTAAACAACTTAGCTCCAATTTTGGTGCAATTTCAAGTGTTT-----

Section 345

(25457)		25457 25470 25480 25490 25500 25510 25520 25530
Homo sapiens chromosome 13 NC_000013.11: 34882059...	(24275)	ATGTAACTAATAAAGTTACAACACCTC-CTCTGCCCAGTGTTGGGAGTCATGTGTTTGGCT-ATCCCAGTA--A
SARS-CoV-2 Reference Genome NC_045512.2	(24492)	---TAAATGATATCCTTTCACGTCTTGACAAAGTTGAGGCTGAAGTGCAAATTGATAGGTTGATCACAGGCAGA

Section 346

(25531)		25531 25540 25550 25560 25570 25580 25590 25604
Homo sapiens chromosome 13 NC_000013.11: 34882059...	(24345)	CTCTAAAGCATCAAAGGGTAAAT-AGGAGGTGATTAAAACACCCTGCACTAAGCAGCAGGACGG--AATGGAGA
SARS-CoV-2 Reference Genome NC_045512.2	(24563)	CTTCAAAGTTTGCAGACATATGTGACTCAACAATTAATTAGAGCTGCAGAAATCAGAGCTTCTGCTAATCTTGC

Section 347

(25605)		25605 25610 25620 25630 25640 25650 25660 25678
Homo sapiens chromosome 13 NC_000013.11: 34882059...	(24416)	TGCGTC--ACCTGACCGGCTG----CATGGTGGCTGTAAGGTGGCTTCCTCAGGTGGGTCTTGCTGGTAGCTAG
SARS-CoV-2 Reference Genome NC_045512.2	(24637)	TGCTACTAAAATGTCAGAGTGTGTACTTGGACAATCAAAAAGAGTTGATTTTTGTGGAAAGGGCTATCATCTTA

Section 348

(25679)		25679 25690 25700 25710 25720 25730 25740 25752
Homo sapiens chromosome 13 NC_000013.11: 34882059...	(24484)	AGTCCTTCCCTAGTCTAGTCTCTGGCTC-TGGTGTTTTATCACTACCAGCTATTTGGAGTGGTCTGGTAGGTGG
SARS-CoV-2 Reference Genome NC_045512.2	(24711)	TGTCCTTCCCTC----AGTCAGCACCTCATGGTGTAGTCTTCTTGCATGTGACTTA---TGTCCCTGCACAAG-

Section 349

(25753)		25753 25760 25770 25780 25790 25800 25810 25826
Homo sapiens chromosome 13 NC_000013.11: 34882059...	(24557)	TGGATAACAGCCTCGAAAGGCTTAGGGCGGCTTCCAGGTTTGGGTAATCCAATAAGGTGATCCTTACATGCTAA
SARS-CoV-2 Reference Genome NC_045512.2	(24777)	---AAAAGAACTTCACAA--CT----GCTCCTGCCA--TTTG--TCATGATGGAAAAGCACACTTTCCTCGTGA

Section 350

(25827)		25827 25840 25850 25860 25870 25880 25890 25900
Homo sapiens chromosome 13 NC_000013.11: 34882059...	(24631)	ATCCTCCAAGGTCTACCTCACAGGCTTAGGACATGAGGTCTTGTCCCCACTGAGAAACTTCTTGTGAGATGGCT
SARS-CoV-2 Reference Genome NC_045512.2	(24838)	AGGTGTCTTTGTTT----CAAATG----GCACACACTGGTTTGTAAC-ACAAAGGAATTTTT------ATGAAC

```
Section 351
                                                           (25901) 25901         25910           25920           25930           25940           25950           25960           25974
Homo sapiens chromosome 13 NC_000013.11: 34882059... (24705) GACAAGCCAGCAAGGACCTCAGCTACCTAGTTCCCTAGTTAATTCCAAGCCTACAGGAAACCTAGAAATTTCCC
SARS-CoV-2 Reference Genome NC_045512.2 (24897) CACAAATCATTACTACAGACAAC-ACATTTGTGTCTGGTAACTGTGATGTT----GTAA---TAGGAATTGTCA

Section 352
                                                           (25975) 25975 25980           25990           26000           26010           26020           26030           26048
Homo sapiens chromosome 13 NC_000013.11: 34882059... (24779) CCAGCTGAGCTTG-GCTCCAGAGGAAGCTGAAGGTGATCTTTGCCTTCTCTTGCCTCATATTGCTATCTCCTCA
SARS-CoV-2 Reference Genome NC_045512.2 (24963) ACAACACAGTTTATGATCCTTTGCAACCTGAATTAGACTCATTCAAGGAGGAG--TTAGATAAATATTT--TAA

Section 353
                                                           (26049) 26049            26060           26070           26080           26090           26100           26110      26122
Homo sapiens chromosome 13 NC_000013.11: 34882059... (24852) GGGTCCTTCTTGCTCCAAAGGCGACAGAGGATTCTGCAGATTCTATTGCTCAGCAAACTCTAGAAATTGTGTGT
SARS-CoV-2 Reference Genome NC_045512.2 (25033) GAATCATACATCACCAGATGTTGATTTAGGTGACATCTCTGGC-ATTAATGCTTCAGTTGTAAACATTCAAAAA

Section 354
                                                           (26123) 26123    26130           26140           26150           26160           26170           26180           26196
Homo sapiens chromosome 13 NC_000013.11: 34882059... (24926) CATTTTCTTACTTATCTGCCTACAGCTGCAAA-ACTAAAAATGATGCTTTAGAATGTTTCTCGTGTGTAGACAT
SARS-CoV-2 Reference Genome NC_045512.2 (25106) GAAATTG--ACCGC-CTCAATGAGGTTGCCAAGAATTTAAATGAATCTCTC--ATCGATCTCCA----AGAACT

Section 355
                                                           (26197) 26197            26210           26220           26230           26240           26250           26260      26270
Homo sapiens chromosome 13 NC_000013.11: 34882059... (24999) GTGGTCAGAATAAATCTTCTCACTCAAATGCTCTCTTTTCCTCCTTTTAGTGTCCTCTCCTGACTCTCCTTCCT
SARS-CoV-2 Reference Genome NC_045512.2 (25171) -TGGAAAGTATGAGCAGTATA--TAAAATGGCCATGGTACATTTGGCTAGGTTTTATAGCTGGCTTGATTGCCA

Section 356
                                                           (26271) 26271         26280           26290           26300           26310           26320           26330           26344
Homo sapiens chromosome 13 NC_000013.11: 34882059... (25073) TTCACA-GCCAAGCCTCAAGAATGGTTCTATGCTCACTTCCCCTACTTCCTCCCATCACATTCACTCTTTGCTG
SARS-CoV-2 Reference Genome NC_045512.2 (25242) TAGTAATGGTGACAATTATGCTTTGCTGTATGACCAGTTGCTGTAGTTGTCTCAAGGGCTGTTGTTCTTG--TG

Section 357
                                                           (26345) 26345 26350           26360           26370           26380           26390           26400           26418
Homo sapiens chromosome 13 NC_000013.11: 34882059... (25146) CAGTCTGACTCTAGTCCCTGAAACTCCTTTCCATAA-AGTCATAATTTATCCTCAAATT-GTGAAATTCAATGA
SARS-CoV-2 Reference Genome NC_045512.2 (25314) GATCCTG----------CTGCAAATTTGATGAAGACGACTCTGAGCCAGTGCTCAAAGGAGTCAAATTACATTA
```

Section 358

```
                                                   (26419) 26419      26430          26440          26450          26460          26470          26480      26492
Homo sapiens chromosome 13 NC_000013.11: 34882059... (25218) CTATGTCCTGGCCTTATTGAACTACAGTGTGGCACAATGTGCTGCCCACTCCCTCTTTAAAACTCTCACTTTAG
        SARS-CoV-2 Reference Genome NC_045512.2 (25378) CACATAAACGAACTTATGGATTT---GTTTATGAGAAT---CTTCACAAT-------TGGAACTGTAACTTTGA
```

Section 359

```
                                                   (26493) 26493  26500          26510          26520          26530          26540          26550          26566
Homo sapiens chromosome 13 NC_000013.11: 34882059... (25292) TGTTAG-TGACATCATTCTTGC--CTAAGTATCATCTCCTTCCCTGAGCATCTTTCTGTCTCATGCATGCAGGC
        SARS-CoV-2 Reference Genome NC_045512.2 (25439) AGCAAGGTGAAATCAAGGATGCTACTCCTTCAGATTTGTTCGCGCTACTGCAACGATACCGATACAAGC---C
```

Section 360

```
                                                   (26567) 26567           26580          26590          26600          26610          26620          26630  26640
Homo sapiens chromosome 13 NC_000013.11: 34882059... (25363) TCTTCCAATTTCCCCTCAGTGATGGTGCTGCCACTGCTGAGGACCTGCTTTTTGCACTGTGCTCTTCCATCCAT
        SARS-CoV-2 Reference Genome NC_045512.2 (25510) TCACTCCCTTTCGGATGGCTTATTGT--TGGCGTTGC--ACTTCTTGCTGTTTTT-CAGAGCGCTTCCAAAATC
```

Section 361

```
                                                   (26641) 26641    26650          26660          26670          26680          26690          26700       26714
Homo sapiens chromosome 13 NC_000013.11: 34882059... (25437) ATAGTTCTCATTAGGTGACCACATGGAAGCCTCTGTTTTCCTTCAACTACCACTGTGTATGACCCAATGCCTCC
        SARS-CoV-2 Reference Genome NC_045512.2 (25579) ATAACCCTCAAAAAGAGATGGCAACTAGCACTCT-----CCAAGGGTGTTCACTTTGTTTG--CAACTTGCTGT
```

Section 362

```
                                                   (26715) 26715 26720          26730          26740          26750          26760          26770            26788
Homo sapiens chromosome 13 NC_000013.11: 34882059... (25511) AGCTCCTTCTA---CTGAGCTCAGACATCATCCCCG--GGTTCTGGACCCACATAATGTGTTCTTTATTTGAAG
        SARS-CoV-2 Reference Genome NC_045512.2 (25646) TGTTGTTTGTAACAGTTTACTCACACCTTTGCTCGTTGCTGCTGGCCTTGAAGCCCCTTTTCTCTAT------
```

Section 363

```
                                                   (26789) 26789      26800          26810          26820          26830          26840          26850      26862
Homo sapiens chromosome 13 NC_000013.11: 34882059... (25580) AGAAATGATGACTTTATGGAAAAAAGTTTCTGCCTGAAACTTGCCTAGGCACTTTATACTTAACATGGATTATA
        SARS-CoV-2 Reference Genome NC_045512.2 (25714) -----------CTTTATGC---------TTTAGTCTACTTCTTGCAGAG----TATAAACTTTGTAAGAATAATA
```

Section 364

```
                                                   (26863) 26863  26870          26880          26890          26900          26910          26920          26936
Homo sapiens chromosome 13 NC_000013.11: 34882059... (25654) AGCCCTTCTTCCCTCTGTATTCCCCTAGTTGAAGGCACATTCATCCAAGGACCTAGTATTCTAGCCATTCTGTC
        SARS-CoV-2 Reference Genome NC_045512.2 (25765) ATGAGGCTTTGGCTTTG-----CTGGAAATGCCGTTCCAAAAACCCATTACTTTATGATGCCAACTATTTTCTT
```

```
                                                                    Section 365
                                                        (26937) 26937          26950       26960       26970       26980       26990       27000   27010
Homo sapiens chromosome 13 NC_000013.11: 34882059... (25728) TGCCTCTTCTATCCTCATTGCCTAAGAGTCTGATTTACTTCCTA-AATGTACATGTTGGGCTCT--TTTCTT
   SARS-CoV-2 Reference Genome NC_045512.2 (25834) TGC---TGGCATACTAATTGT-TACGACTATTGTATACCTTACAATAGTGTAACTTCTTCAATTGTCATTACTT
                                                                    Section 366
                                                        (27011) 27011      27020       27030       27040       27050       27060       27070       27084
Homo sapiens chromosome 13 NC_000013.11: 34882059... (25799) CCCTTCTATTGCACTGCCATGGTCTTGGTTTAGGCCCTCATTGCTTT-TTGCCAGGATTATTGTTCTCTCAGCC
   SARS-CoV-2 Reference Genome NC_045512.2 (25904) CAGGTG-ATGGCACAACAA--GTCCTATTTCTGAACATGACTACCAGATTGGTGGTTATACTGAAAAATGGGAA
                                                                    Section 367
                                                        (27085) 27085 27090        27100       27110       27120       27130       27140         27158
Homo sapiens chromosome 13 NC_000013.11: 34882059... (25872) TCTAGACTACACAAGCCCTTTCTCCTACCCAA--CCTCTACCAGAGTGATTTTTTAACTGTAAATCTGCTCGTG
   SARS-CoV-2 Reference Genome NC_045512.2 (25975) TCTGGAGTAAAAGACTGTGTTGTATTACACAGTTACTTCACTTCAGACTATTACCAGCTGTAC-TCAACTCAAT
                                                                    Section 368
                                                        (27159) 27159         27170       27180       27190       27200       27210       27220     27232
Homo sapiens chromosome 13 NC_000013.11: 34882059... (25944) TCAGGTTACCTTCCTGCTTAAAACCTTTCAGGATTCCGTCACATTCAGGATAAAGGTCAAACTCCTCAGCTAGG
   SARS-CoV-2 Reference Genome NC_045512.2 (26048) TGAGTACAGACACTGGTGTTGAACATGTTACCTT--CTTCATCTACAATAAAATTGTTGA-----TGAGCCTGA
                                                                    Section 369
                                                        (27233) 27233    27240       27250       27260       27270       27280       27290         27306
Homo sapiens chromosome 13 NC_000013.11: 34882059... (26018) CACACTAAGCCCATCATTAATACTTTGGCATCACACTCCACAGTACTAGTTGTGAGAGTGTAGACAAGTTACT-
   SARS-CoV-2 Reference Genome NC_045512.2 (26115) AGAAC-ATGTCCAA-ATTCACACAATCGACGGTTCATCCGGAGTTGTTAATCCAGTAATGGAACCAATTTATGA
                                                                    Section 370
                                                        (27307) 27307          27320       27330       27340       27350       27360       27370   27380
Homo sapiens chromosome 13 NC_000013.11: 34882059... (26091) TAACCTTACTATGCCTCAGTTTCCTCCATGTGTAA--AATAGA-GATAAGATAGCACGTGTGATAAGATCATTG
   SARS-CoV-2 Reference Genome NC_045512.2 (26187) TGAACCGACGACGACT-ACTAGCGTGCCTTTGTAAGCACAAGCTGATGAGTACGAACTTATGTACTCATTCGTT
                                                                    Section 371
                                                        (27381) 27381      27390       27400       27410       27420       27430       27440       27454
Homo sapiens chromosome 13 NC_000013.11: 34882059... (26162) TCAGCACAGAATGAACTAAGGCATATAAAGTACTTAGGCACGTAGTAAGCAATCTGTTTTTTGCTTCTTTGTT
   SARS-CoV-2 Reference Genome NC_045512.2 (26260) TCGGAAGAGAC-------AGGTACGTTAA-TAGTTAATAGCGTACT------TCTTTTTCTTGCTTTCGTGGTA
```

Section 372

	(27455)	27455 27460 27470 27480 27490 27500 27510 27528
Homo sapiens chromosome 13 NC_000013.11: 34882059...	(26236)	TGTTTATTTTTTAGACAGGG---TCTTACTCTGTCACCAAGGCTGGAGTACAGTGACATGATC--TC-----AG
SARS-CoV-2 Reference Genome NC_045512.2	(26320)	TTCTTGCTAGTTACACTAGCCATCCTTACTGCGCTTCGATTGTGTGCGTACTGCTGCAATATTGTTAACGTGAG

Section 373

	(27529)	27529 27540 27550 27560 27570 27580 27590 27602
Homo sapiens chromosome 13 NC_000013.11: 34882059...	(26300)	CTCACTGCAACCT--CTTTGCCTCAGCCTCTCAAGT---AGCTGGGACTACAGGAGCATGCCACCATGCCCAG-
SARS-CoV-2 Reference Genome NC_045512.2	(26394)	TCTTGTAAAACCTTCTTTTTACGTTTACTCTCGTGTTAAAAATCTGAATTCTTCTAGAGTTCCTGATCTTCTGG

Section 374

	(27603)	27603 27610 27620 27630 27640 27650 27660 27676
Homo sapiens chromosome 13 NC_000013.11: 34882059...	(26368)	-CTAATCAATGTTTGTTTGTTTGTTTGTTTGTTTGTTTCTTTCTTTCTTTCTTTCTTTCTTTCTTTCTTTCTTT
SARS-CoV-2 Reference Genome NC_045512.2	(26468)	TCTAAACGAACTAAATAT-TATATTAGTTTTTCTGTTTGGAACTTTAATTTTAGCCATGGCAGATTCCAACGGT

Section 375

	(27677)	27677 27690 27700 27710 27720 27730 27740 27750
Homo sapiens chromosome 13 NC_000013.11: 34882059...	(26441)	CTTTCTCTTTCTTTCTCTCTTTCTCTCTTTCTCTCTCTTTCTTCCCTCCCTCCCTTTTTCCT--TCCCTCCCT-
SARS-CoV-2 Reference Genome NC_045512.2	(26541)	ACTATTACCGTTGAAGAGCTTAAAAAGCTCCTTGAACAATGGAACCTAGTAATAGGTTTCCTATTCCTTACATG

Section 376

	(27751)	27751 27760 27770 27780 27790 27800 27810 27824
Homo sapiens chromosome 13 NC_000013.11: 34882059...	(26512)	-CTCTTCCTTCCTTCCTTCCCTCCCTCCCTCTCTCCCTCCTTCCCTTCCTTCCTTCCCTCCTTCTTTC-CTTTT
SARS-CoV-2 Reference Genome NC_045512.2	(26615)	GATTTGTCTTCTACAATTTGCCTATGCCAACAGGAATAGGTTTTTGTATATAATTAAGTTAATTTTCCTCTGGC

Section 377

	(27825)	27825 27830 27840 27850 27860 27870 27880 27898
Homo sapiens chromosome 13 NC_000013.11: 34882059...	(26584)	TTTTGTAGATATGGGCTTTCACT-GTGTTGCCCAAGCTGCTCCAGAACTCCTGAGCTCAAGTGATCCACCAGCC
SARS-CoV-2 Reference Genome NC_045512.2	(26689)	TGTTATGGCCAGTAACTTTAGCTTGTTTTGTGCTTGCTGCTGTTTA----CAGAA-TAAATTGGATCACCGGTG

Section 378

	(27899)	27899 27910 27920 27930 27940 27950 27960 27972
Homo sapiens chromosome 13 NC_000013.11: 34882059...	(26657)	TAG--GCCTCCCAAAGTGCTGGGATTACAGGCATGAGCCACTGCACCCAGCCAGCAATCTGTTTTTGATAGTAG
SARS-CoV-2 Reference Genome NC_045512.2	(26758)	GAATTGCTATCGCAATGGCTTGTCTTGTAGGCTTGATGTGGCTCAGCTA--CTTCATTGCTTCTTTCAGACTGT

Section 379

(27973) 27973 27980 27990 28000 28010 28020 28030 28046
Homo sapiens chromosome 13 NC_000013.11: 34882059... (26729) CTATCTTTAT---TACCATTTTAAAATCTCACCTCCCTTCTCTTCC---CTTCCCTTCG-GCCACCATACTGGT
SARS-CoV-2 Reference Genome NC_045512.2 (26830) TTGCGCGTACGCGTTCCATGTGGTCAT-TCAA-TCCAGAAACTAACATTCTTCTCAACGTGCCACTCCATGGCA

Section 380

(28047) 28047 28060 28070 28080 28090 28100 28110 28120
Homo sapiens chromosome 13 NC_000013.11: 34882059... (26796) CTACT-TGACATTTCTGAATATTGCCTGTGAGTTCATCCTCCTGGGCTTACTTATGGTCCTGCTTCCTC-TGTG
SARS-CoV-2 Reference Genome NC_045512.2 (26902) CTATTCTGACCAGACCGCTTCTAGAAAGTGAACTCGTAATCG-GAGCTG-----TGATCCTTCGTGGACATCTT

Section 381

(28121) 28121 28130 28140 28150 28160 28170 28180 28194
Homo sapiens chromosome 13 NC_000013.11: 34882059... (26868) TGTAATACTTTCCCCCTCTTTCTTCATCTAGCTAACAGGTACTTAGTTTTAAAGACCCTTCTCACATCACTTCT
SARS-CoV-2 Reference Genome NC_045512.2 (26970) CGTATTGCTGGACACCATCTAGGACGCTGTGACATCAAGGACCTGCCTAAAGAAATCACTGTTGCTACAT----

Section 382

(28195) 28195 28200 28210 28220 28230 28240 28250 28268
Homo sapiens chromosome 13 NC_000013.11: 34882059... (26942) TCCAGAAACTCTTCCTTGAT-CTTCTTGCTCCAGTCGGGGTTTAATTTTCCATGTTTTTGTAACTCTTCATTAA
SARS-CoV-2 Reference Genome NC_045512.2 (27040) --CACGAACGCTTTCTTATTACAAATTGGGAGCTTCGCAGCGTG---TAGCAGGTGACTC-AGGTTTTGCTGCA

Section 383

(28269) 28269 28280 28290 28300 28310 28320 28330 28342
Homo sapiens chromosome 13 NC_000013.11: 34882059... (27015) TACATTTATCCCAACACTCAATAACTCTGCATTGGGCTTGATTACATGTTTGTCTCCTATACAAGACTGTGGAT
SARS-CoV-2 Reference Genome NC_045512.2 (27108) TACAGTCGCTACAGGATTGGC-AACTATAAATTAAACAC-AGACCATTCCAGTAGCAGTGACAATATTGCT--T

Section 384

(28343) 28343 28350 28360 28370 28380 28390 28400 28416
Homo sapiens chromosome 13 NC_000013.11: 34882059... (27089) TCCTTAAAGGCAGAATACAACTTTGTCTGAGTTCCAACGAAAGGCTTGTCAA--TGTTGAACCAGGGATTCAG
SARS-CoV-2 Reference Genome NC_045512.2 (27178) TGCTTGTA--CAGTAAGTGACAACAGATG--TTTCATCTCGTTGACTT-TCAGGTTACTATAGCAGAGATATTA

Section 385

(28417) 28417 28430 28440 28450 28460 28470 28480 28490
Homo sapiens chromosome 13 NC_000013.11: 34882059... (27161) TAAATGACTGCTGGGTGAATTAAAAGATTGCATCAGGTGCTCAGAGACTCTAGCACACCTT-TGCTTCAAAAAG
SARS-CoV-2 Reference Genome NC_045512.2 (27247) CTAATTATTA-TGAGGACTTTTAAAGTTTCCATTTGGAATCTTGATTACATCATAACCTCATAATTAAAAATT

Section 386

Sequence	Start	28491 – 28564
	(28491)	
Homo sapiens chromosome 13 NC_000013.11: 34882059...	(27234)	CACCTTACTT-CAAAATTATTATCTCATTTCTTCCTCTATTTAAAGTCCCTATTTGTAAATTGT------GAAA
SARS-CoV-2 Reference Genome NC_045512.2	(27320)	TATCTAAGTCACTAACTGAGAATAAATATTCTCAATTAGATGAAGAGCAACCAATGGAGATTGATTAAACGAAC

Section 387

Sequence	Start	28565 – 28638
	(28565)	
Homo sapiens chromosome 13 NC_000013.11: 34882059...	(27301)	TTAGGCACTATTAGTTTACTGTCAGCGATGAAACTAA--GCTAGTCAGCTGAAAATTTATTACAATTCATTTAT
SARS-CoV-2 Reference Genome NC_045512.2	(27394)	ATGAAAATTATTCTTTTCTTGGCACTGATAACACTCGCTACTTGTGAGCTTTATCACTACCAAGAGTGTGTTAG

Section 388

Sequence	Start	28639 – 28712
	(28639)	
Homo sapiens chromosome 13 NC_000013.11: 34882059...	(27373)	ACTTCTAAAGCGTATATTTACCCTTTCAAACAAGGAACTTGTA-AAGATAATAGGATGAATTTTAGTGATGAGT
SARS-CoV-2 Reference Genome NC_045512.2	(27468)	AGGTACAACA-GTACTTTTAAAAG----AACCTTGCTCTTCTGGAACATACGAGGGC-AATTCACCATTTCATC

Section 389

Sequence	Start	28713 – 28786
	(28713)	
Homo sapiens chromosome 13 NC_000013.11: 34882059...	(27446)	TTTAAGGAAATATAATAGGAGGAAAGGGTGTAGTGTGAGAAGATAGGAAGAATAATTTTTTTCTGCCCAGGCAC
SARS-CoV-2 Reference Genome NC_045512.2	(27536)	CTCTAGCTGATAACAAATTTGCACTGACTT--GCTTTAGCACTCA-----ATTTGCTTTTGCTTGTCCTGACGG

Section 390

Sequence	Start	28787 – 28860
	(28787)	
Homo sapiens chromosome 13 NC_000013.11: 34882059...	(27520)	AGTGGCGCATGCCTATAATCCCAGCACTTTGGGAGGCCAAAGCAGGAGGATCACTTGAGGCCAGTAGTTCAAGG
SARS-CoV-2 Reference Genome NC_045512.2	(27603)	CGTAAAACACGTCTAT--------CAGTTACGT--GCCAGATCAGTT---TCACCTAAA--CTGTTCATCA-GA

Section 391

Sequence	Start	28861 – 28934
	(28861)	
Homo sapiens chromosome 13 NC_000013.11: 34882059...	(27594)	CTACAGTGAGCAATGATCATGC-CATTGCACTCCAGTCTGGGCCACAGAGGAAGACTCAGTTTCTAAAAAAAAA
SARS-CoV-2 Reference Genome NC_045512.2	(27661)	CAA----GAGGAA-GTTCAAGAACTTTACTCTCCAATTTTTCTTATTGTTGCGGCAATAGTGTTTATAACACTT

Section 392

Sequence	Start	28935 – 29008
	(28935)	
Homo sapiens chromosome 13 NC_000013.11: 34882059...	(27667)	ATTAAATACAGAAAAAATAAAAAAATAAAAAATAAATTTTATGGGAGAGTTGCTTGGAACTCCTTAAATAACAT
SARS-CoV-2 Reference Genome NC_045512.2	(27730)	-TGCTTCACACTCAAAAGAAAGACAGAATGATTGAACTTTCATTAATTGACTTCTATTTGTGCTTTT-TAGCCT

Section 393

```
                                                              (29009) 29009      29020      29030      29040      29050      29060      29070      29082
Homo sapiens chromosome 13 NC_000013.11: 34882059... (27741) CAAGTGGGAATCCAGCCTCTACTGGTAGGGGTAAGCTGATGGCCTACTATATGAGGGGATTTGACTGGAAAAGC
SARS-CoV-2 Reference Genome NC_045512.2 (27802) TTC-TGCTATTCCTTGTTTTAATTATGCTTATTATCTTTTGGTTCTCACT-TGA---------ACTGCAAGATC
```

Section 394

```
                                                              (29083) 29083  29090      29100      29110      29120      29130      29140      29156
Homo sapiens chromosome 13 NC_000013.11: 34882059... (27815) ACCCAGAAGCTTTTCATAATTAAACTAGTAGCTGAAATACTGAACTAATATCACTAATAAAAA----GGCAGCC
SARS-CoV-2 Reference Genome NC_045512.2 (27865) ATAATGAAACTTGTCACGCCTAAACGAACATGAAATTTCTTGTTTTCTTAGGAATCATCACAACTGTAGCTGCA
```

Section 395

```
                                                              (29157) 29157      29170      29180      29190      29200      29210      29220   29230
Homo sapiens chromosome 13 NC_000013.11: 34882059... (27885) TGACAACAAAA--GAACT--AAAATCTTG-ATACATGATACAACATGCTAAGTGAAGGAAGCCAGACAAAAAAA
SARS-CoV-2 Reference Genome NC_045512.2 (27939) TTTCACCAAGAATGTAGTTTACAGTCATGTACTCAACATCAACCATATGTAGTTGATGACCCGTGTCCTATTCA
```

Section 396

```
                                                              (29231) 29231    29240      29250      29260      29270      29280      29290      29304
Homo sapiens chromosome 13 NC_000013.11: 34882059... (27954) CCCATATGCTTCATG-TGTATGAAA----TGTCCAGAATAAGCAAACCCATAGT----CAGTGAGTGGATCAGG
SARS-CoV-2 Reference Genome NC_045512.2 (28013) CTTCTATTCTAAATGGTATATTAGAGTAGGAGCTAGAAAATCAGCACCTTTAATTGAATTGTGCGTGGATGAGG
```

Section 397

```
                                                              (29305) 29305 29310      29320      29330      29340      29350      29360      29378
Homo sapiens chromosome 13 NC_000013.11: 34882059... (28019) --GGCTGGGGAACAGGGAGTGATTTCTAATGAGTATGGGTTTCTTTGGGGCTGA-TAAAAATGTTCTGAAATTA
SARS-CoV-2 Reference Genome NC_045512.2 (28087) CTGGTTCTAAATCACCCATTCAGTAC--ATCGATATCGGTAATTATACAGTTTCCTGTTTACCTTTTACAATTA
```

Section 398

```
                                                              (29379) 29379      29390      29400      29410      29420      29430      29440      29452
Homo sapiens chromosome 13 NC_000013.11: 34882059... (28090) AATAGTGGTGATGGGTGCATAACCCTTTGAATATACTAAAATCTACTGAATTGTACATTTTAA-AAGTGTTATT
SARS-CoV-2 Reference Genome NC_045512.2 (28159) ATT------GCCAGGAACCTAAA---TTGGGTAGTCT----TGTAGTGCGTTGTTCGTTCTATGAAGACTTTTT
```

Section 399

```
                                                              (29453) 29453  29460      29470      29480      29490      29500      29510      29526
Homo sapiens chromosome 13 NC_000013.11: 34882059... (28163) T--TTATGATATGTTAAATTATATTGCAATAAAAATATGTCAGAAAAAAGTGATATTCCAGGAAATACTGACAA
SARS-CoV-2 Reference Genome NC_045512.2 (28220) AGAGTATCATGACGTTCGTGTTGTTTTAGATTTCATCTAAACGAACAAACTAAAATGTCTGATAATGGACCCCA
```

Section 400

		29527 29540 29550 29560 29570 29580 29590 29600
	(29527)	
Homo sapiens chromosome 13 NC_000013.11: 34882059...	(28235)	TAGAAAAAAAACAGCATGAGTTATTTATTATACTATTTGAAGGTCCACAAATAAAATTATATTTTCTAAGTATG
SARS-CoV-2 Reference Genome NC_045512.2	(28294)	AAATCAGCGAAATGCACCCCGCATT------ACGTTTGGTGGACCCTCAGATTCAACTGGCAGTAACCAGAATG

Section 401

		29601 29610 29620 29630 29640 29650 29660 29674
	(29601)	
Homo sapiens chromosome 13 NC_000013.11: 34882059...	(28309)	AAGGATTATATAGCAAGTCATTTTAATAGCTTA--CATAAAGCAGTTTCATATTTCTTTTCCTCTAGTTTCACA
SARS-CoV-2 Reference Genome NC_045512.2	(28362)	GAGAACGCAGTGGGGCGCGATCAAAACAACGTCGGCCCCAAGGTTTACCCAATAATACTGCGTCTTGGTTCACC

Section 402

		29675 29680 29690 29700 29710 29720 29730 29748
	(29675)	
Homo sapiens chromosome 13 NC_000013.11: 34882059...	(28381)	GAACTCTTTTGATGTATTGTGTCATTAATTAATAGGCTCTAAAAACTACCGTTGTGTTAGTTGGTGTTTTGTTT
SARS-CoV-2 Reference Genome NC_045512.2	(28436)	GCTCTCACTCAACAT-----GGCAAGGA-----AGACCTTAAA---TTCCCTCGAGG-ACAAGGCGTTCCAATT

Section 403

		29749 29760 29770 29780 29790 29800 29810 29822
	(29749)	
Homo sapiens chromosome 13 NC_000013.11: 34882059...	(28455)	CTGTTTTA-AACTTTCAATTTTTTTTTTTTTTTTGAGATGGAGTCTCACTCTGTCACCCAGGCTGGAGTGCAGT
SARS-CoV-2 Reference Genome NC_045512.2	(28496)	AACACCAATAGCAGTCCAGATGACCAAATTGGCTACTACCGAAGAGCTACCAG--ACGAATTCGTG-GTGGTGA

Section 404

		29823 29830 29840 29850 29860 29870 29880 29896
	(29823)	
Homo sapiens chromosome 13 NC_000013.11: 34882059...	(28528)	GGCTGGAGTGCAACCTCCACCTCCCAGGTTCAAGTGATTCTCCTGCCT--GAGCTTAACAAGTAACTGGGATTA
SARS-CoV-2 Reference Genome NC_045512.2	(28567)	CGGTAAAATGAAAGATCTCAGTCCAAGATG---GTATTTCTACTACCTAGGAACTGGGCCAGAAGCTGGACTTC

Section 405

		29897 29910 29920 29930 29940 29950 29960 29970
	(29897)	
Homo sapiens chromosome 13 NC_000013.11: 34882059...	(28600)	CAGGCACCTGCCACCAAACCCGGC-TAATTT---TTGTATTTTAGTAGAGACGGAGTTTCACTATGTTAGCCAG
SARS-CoV-2 Reference Genome NC_045512.2	(28638)	CCTATGG-TGCTAACAAAGACGGCATCATATGGGTTGCAACTGAGG-GAGCCTTGAATACACCAAAGATCACA

Section 406

		29971 29980 29990 30000 30010 30020 30030 30044
	(29971)	
Homo sapiens chromosome 13 NC_000013.11: 34882059...	(28670)	GTTGCTCTCAAACTCCTG---AC----CTGAA---GTGATCCACCCGCCTCAGCCTCCCAAGGTGCCA---GGA
SARS-CoV-2 Reference Genome NC_045512.2	(28710)	TTGGCACCCGCAATCCTGCTAACAATGCTGCAATCGTGCTACAACTTCCTCAAGGAACAACATTGCCAAAGGC

```
                                                              Section 407
                                                  (30045) 30045 30050     30060     30070     30080     30090     30100            30118
Homo sapiens chromosome 13 NC_000013.11: 34882059... (28731) TTACAGGTGTGAGCCACCACGCCTGGCCCTAAACTCAATTTTTAAAAAATAATTTATTTTGGTTTTATAATGG
     SARS-CoV-2 Reference Genome NC_045512.2 (28784) TTCTACGCAGAAGGGAGCAGAGGCGGCAGTCAAGCC--TCTTCTCGTTCCTCATC-ACGTAGTCGCAACAGTTC
                                                              Section 408
                                                  (30119) 30119      30130     30140     30150     30160     30170     30180    30192
Homo sapiens chromosome 13 NC_000013.11: 34882059... (28805) TCTCAATTTAGCTCATTCAATCAAAACAATAACCTATACTACTT---TCGGTT-CAATTCAAATGTTCCTTTTG
     SARS-CoV-2 Reference Genome NC_045512.2 (28855) AAGAAATTCAACTCCAGGCAGCAGTAGGGGAACTTCTCCTGCTAGAATGGCTGGCAATGGCGGTGATGCTGCTC
                                                              Section 409
                                                  (30193) 30193  30200     30210     30220     30230     30240     30250          30266
Homo sapiens chromosome 13 NC_000013.11: 34882059... (28875) ACTATTAATCCCAGTTTTA-AGAT-GAAC---ATTTATAACTAT---CCAAGTTGATGCCA-------CA--GA
     SARS-CoV-2 Reference Genome NC_045512.2 (28929) TTGCTTTGCTGCTGCTTGACAGATTGAACCAGCTTGAGAGCAAAATGTCTGGTAAAGGCCAACAACAACAAGGC
                                                              Section 410
                                                  (30267) 30267        30280     30290     30300     30310     30320     30330 30340
Homo sapiens chromosome 13 NC_000013.11: 34882059... (28932) TATGCTGTAATTAATAACATTTTATGTTTC--CTTTTATTTATCATAGTGATATAATTTTATATTACTGTCCAC
     SARS-CoV-2 Reference Genome NC_045512.2 (29003) CAAACTGTCACTAAGAA-ATCTGCTGCTGAGGCTTCTAAGAAGCCTCGGCAAAAACGT--------ACTG-CCAC
                                                              Section 411
                                                  (30341) 30341    30350     30360     30370     30380     30390     30400    30414
Homo sapiens chromosome 13 NC_000013.11: 34882059... (29004) CATAAATGTCAGTTGAACAGAGGCTTAATTGAAACATTCTTTAGATCACAGAAAAAGATAGTGATGTTATGCTT
     SARS-CoV-2 Reference Genome NC_045512.2 (29068) TAAAGCATACAATGTAACACAAGCTTTCGGCAGACGTGGTCCAGAACAAACCCAAGGAAATTTTGGGGAC-CAG
                                                              Section 412
                                                  (30415) 30415 30420     30430     30440     30450     30460     30470            30488
Homo sapiens chromosome 13 NC_000013.11: 34882059... (29078) GAAATATTTCTCGAAGATTATACAAGGTTAAGAAGTCATTGAACAAATGTTTATTGGAGGTCTACTATGTAAAC
     SARS-CoV-2 Reference Genome NC_045512.2 (29141) GAACTA-----------ATCAGACAAGG-----AACTGATT--ACAAAC----ATTGGCCG-CAAAT-TGCACAA
                                                              Section 413
                                                  (30489) 30489      30500     30510     30520     30530     30540     30550    30562
Homo sapiens chromosome 13 NC_000013.11: 34882059... (29152) TATGGCTCTCGGCCTTCTTA-TATTTCCAAGTGTACCTTGAAAACCAACTCTTCTGAGCAACAACTTGGAGAGA
     SARS-CoV-2 Reference Genome NC_045512.2 (29192) TTTGCCCCCAGCGCTTCAGCGTTCTTCGGAATGTCGCG------CATTGGCATGGAAGTCACACCTTCGGGAAC
```

```
                                                                  Section 414
                                                  (30563) 30563  30570     30580     30590     30600     30610     30620     30636
Homo sapiens chromosome 13 NC_000013.11: 34882059... (29225) TTAAATGTGCCACACACGTCAGCACTAATTACACTGAAATCCCAGAAAATCATGGCTCATAGTGTCTGTGAAAG
      SARS-CoV-2 Reference Genome NC_045512.2 (29260) GTGGTTGACCTACACAGGTGCCATCAAATTGGA-TGA-----CAAAGATCCAAATTTCAAAGATCAAGTCATTT
                                                                  Section 415
                                                  (30637) 30637        30650     30660     30670     30680     30690     30700  30710
Homo sapiens chromosome 13 NC_000013.11: 34882059... (29299) TGTCCAGTCAGCACACTATTTAGTTGTTTACACTTTTAC--ACTGAGGCCTTGTTGAAGAAGAAGAAATAGTCT
      SARS-CoV-2 Reference Genome NC_045512.2 (29328) TGCTGAATAAGCATATTGACGCATACAAAACATTCCCACCAACAGAGCCTAAAAAGGACAAAAAGAAGAAGGCT
                                                                  Section 416
                                                  (30711) 30711    30720     30730     30740     30750     30760     30770        30784
Homo sapiens chromosome 13 NC_000013.11: 34882059... (29371) CTTCCTTGACAATCCTCTTTTATATACTCAGACATAAGGGTTATTTTATGTTATAGTAAACACACATAATCCAG
      SARS-CoV-2 Reference Genome NC_045512.2 (29402) GATGAAACTCAAGCCT-TACCGCAGAGACAGAAGAAAACAGCAAACTGTGACTCTTCTT------CCTGCTGCAG
                                                                  Section 417
                                                  (30785) 30785 30790     30800     30810     30820     30830     30840           30858
Homo sapiens chromosome 13 NC_000013.11: 34882059... (29445) ATATCCCAAAATTTTCTAAAATTTGATGTCTTGTTTTTTATAATACCCAAATAAAACAGCTCCTCCTCTTTAC-
      SARS-CoV-2 Reference Genome NC_045512.2 (29469) ATTTGGATGATTTCTCCAAACAAT-----------TGCAACAATCCATGAGCAGTGCTGACTCAACTCAGGCCT
                                                                  Section 418
                                                  (30859) 30859      30870     30880     30890     30900     30910     30920    30932
Homo sapiens chromosome 13 NC_000013.11: 34882059... (29518) ACTCTCTGGGATACCAGCACCCTCCCTTCTCCTCCATCA--GTATTTTCTTGCACCTTTCAGAGGTGCACATTT
      SARS-CoV-2 Reference Genome NC_045512.2 (29532) AAACTCATGCAGACCACACAAGGCAGATGGGCTATATAAACGTTTTCGCTTTTCCGTTTACGATAT----ATAG
                                                                  Section 419
                                                  (30933) 30933  30940     30950     30960     30970     30980     30990        31006
Homo sapiens chromosome 13 NC_000013.11: 34882059... (29590) TCTCAGATGTTGCAGC-TGCTTTCCCACTGTGGTTTGACCATGAACCATTATTTGTGG----CTTCCATGTTAG
      SARS-CoV-2 Reference Genome NC_045512.2 (29602) TCTACTCTTGTGCAGAATGAATTCTC---GTAACT--ACATAGCACAAGTAGATGTAGTTAACTTTAATCTCAC
                                                                  Section 420
                                                  (31007) 31007        31020     31030     31040     31050     31060     31070  31080
Homo sapiens chromosome 13 NC_000013.11: 34882059... (29659) TTGTTATCCAGTTAAAAGCAGAAAACAGTCTAGGGTTCTGTATTTGTCAGCTTGAACTACTATAACAAAATGTC
      SARS-CoV-2 Reference Genome NC_045512.2 (29671) ATAGCAATCTTTAATCAGTGTGTAACATT--AGGGA---GGACTTGAAAGAGCCACCACATTTTCACCGAGGCC
```

Section 421

```
                                                    (31081) 31081    31090     31100     31110     31120     31130     31140         31154
Homo sapiens chromosome 13 NC_000013.11: 34882059... (29733) ACAGACAGGGTGACTTAAGGAACAGAGATTTATTTATTATCTATTTTATTTATATCTCACAGTTTTAGACTCTA
SARS-CoV-2 Reference Genome NC_045512.2              (29740) ACGCGGAGTACGATCGAGTGTACAGTGAACAATGCTAGGGAGAGCTGCCT-ATATGGAAGAGCCCTAATGTGTA
```

Section 422

```
                                                    (31155) 31155 31160     31170     31180     31190     31200     31210             31228
Homo sapiens chromosome 13 NC_000013.11: 34882059... (29807) AAA--AATTCAAGAACAAGATGCCTTGGTCTTACCTTCTCTCTGCGTCCTCACCTGGCAGACACTCTGTTGTCT
SARS-CoV-2 Reference Genome NC_045512.2              (29813) AAATTAATTTTAGTAG----TGCTATCCCCATGTGATTTTAATAGCTTCTTAGGAGAATGACAAAAAAAAAAAA
```

Section 423

```
                                                    (31229) 31229      31240        31254
Homo sapiens chromosome 13 NC_000013.11: 34882059... (29879) CTTCTTTTAGGACACCAACGCTGTTG
SARS-CoV-2 Reference Genome NC_045512.2              (29883) AAAAAAAAAAAAAAAAAAAAA-----
```
