# Supplementary material for: Patch type nucleotide sequence identities between genomes from many different species facilitate illegitimate recombination
Source: Sci Rep. 2026 Mar 30;16:10524. doi: 10.1038/s41598-026-44124-0 (PMC13035915; doi:10.1038/s41598-026-44124-0)
Supplement: Supplementary file 4 — Supplementary Material 4 [file 41598_2026_44124_MOESM4_ESM.pdf]

```
                                                          Section 1
                                           (1)  1        10        20        30        40        50        60                 79
Oryza sativa chromosome 1 region 9.891-41.663nt   (1)  ACAAACTGCGGCATCCCGCCGGGGTGGAACTGGACGTACCAGTTCCAGGTGAAGGACCAGGTCGGCAGCTTCTTCTACG
SARS-CoV-2 Reference Genome NC_045512.2          (1)  ATTAAAGGTTTATACCTTCCCAGGT--AACAA-ACCAACCAACTTTCGATCT-----CTTGTAGATCTGTTCTCTAAAC
                                                          Section 2
                                          (80)  80       90        100       110       120       130       140               158
Oryza sativa chromosome 1 region 9.891-41.663nt  (80)  CCCCCTCCACCGCCCTCCACCGCGCCGCCGGCGGC-TAC--GGCGCCATCACCATCAACAACCGCGACGTCATCCCCTT
SARS-CoV-2 Reference Genome NC_045512.2         (72)  GAACTTTAAAATCTGTGTGGCTGTCACTCGGCTGCATGCTTAGTGCACTCACGCAGTATAATTAATAACTAATTAC--T
                                                          Section 3
                                         (159)  159        170       180       190       200       210       220              237
Oryza sativa chromosome 1 region 9.891-41.663nt (156)  GCCCTTCCCCCTCCCCGACGGCGGCGAC-ATCACCCTCTTCCTCGCCGACTGGTACGCCAGGGACCACAGGGCCCTGCG
SARS-CoV-2 Reference Genome NC_045512.2        (149)  GTCGTTGACAGGACACGAGTAACTCGTCTATCTTCTGCAGGCT-GCTTACGGTTTCGTCCGTGTTG-CAG--CCGATCA
                                                          Section 4
                                         (238)  238         250       260       270       280       290       300             316
Oryza sativa chromosome 1 region 9.891-41.663nt (234)  CCGCGCCCTCGACGCCGGCGACCCCCTCGGCCCCCCCGACGGGGTGCTCATCAACGCCCTCGGCCCCTACCGCTACAAC
SARS-CoV-2 Reference Genome NC_045512.2        (224)  TCAGCACATCTAGGTTT-CGTCCGGGTGTGACCGAAAGGTAAGATGGAGAGCCTTGTCCCTGGTTTCAACGAGAAAACA
                                                          Section 5
                                         (317)  317          330       340       350       360       370       380           395
Oryza sativa chromosome 1 region 9.891-41.663nt (313)  GACACCCTCGTCCCGCCGGGCGTCACCTACGAGCGCATCAATGTCGACCCGGGG--AGGACCTACCGCCTGCGCGTCCA
SARS-CoV-2 Reference Genome NC_045512.2        (302)  CACGTCCAACTCAGTTTGCCTGTTTTACAGGTTCGCGACGTGCTCGTACGTGGCTTTGGAGACTCCGTGGAGGAGGTCT
                                                          Section 6
                                         (396)  396           410       420       430       440       450       460          474
Oryza sativa chromosome 1 region 9.891-41.663nt (390)  CAACGTCGGCGTCGCCACCAGCCTCAATTTCAGAATCCAGGGCCACAACCTGCTGCTCGTCGAGGCCGAGGGCTCCTAC
SARS-CoV-2 Reference Genome NC_045512.2        (381)  TATCAGAGGCA-CGTCAACATCTTAAAGATGGCACTTGTGGCTTAGTAGAAGTTGAAAA---AGGCGTTTTGC--CTCA
                                                          Section 7
                                         (475)  475  480        490       500       510       520       530       540            553
Oryza sativa chromosome 1 region 9.891-41.663nt (469)  ACTTCACAGCAGAACTACACCAACATGGACATCCATGTCGGCCAGTCCTACTCCTTCCTTCTCACCATGGACCAGAACG
SARS-CoV-2 Reference Genome NC_045512.2        (454)  ACTTGAA--CAGCCCTATGTGTTCAT---CAAACGT-TCGGATGCTCGAACTGCA-CCT------CATGGTCATGTTAT
```

Section 8

```
                                                      554  560       570       580       590       600       610       620          632
Oryza sativa chromosome 1 region 9.891-41.663nt (548) CCTCCACCGACTACTACGTCGTCGCCAGCGCCCGTTTCGTCCCCGACGCCGACAAGCTCACCGGCGTCGCCATCCTCCA
SARS-CoV-2 Reference Genome NC_045512.2         (520) GGTTGAGCTGGTAGCAGAAC-TCGAAGGCATTCAGTACGGTCGTAGTGGTGAGACACTTGGTGTCCTTGTC--CCTCAT
```

Section 9

```
                                                      633    640       650       660       670       680       690       700        711
Oryza sativa chromosome 1 region 9.891-41.663nt (627) CTACTCCAACTCCCAG-GGCCCGCCCTCTGGCTCT-CTCCCTGATGCCCCAGATGACCAGTACGACACCGCTTTCTCCA
SARS-CoV-2 Reference Genome NC_045512.2         (596) GTGGGCGAAATACCAGTGGCTTACCGCAAGGTTCTTCTTCGTAAGAACGGTAATAAAGGAGCTGGTGGCCATAGTTACG
```

Section 10

```
                                                      712     720       730       740       750       760       770       780       790
Oryza sativa chromosome 1 region 9.891-41.663nt (704) TCAACCAAGCAAGATCAATCAGGTCAGTAGCCTGCATTGCCATTACTGCTCATCCATTCATTCATCGGTCCTACTCACT
SARS-CoV-2 Reference Genome NC_045512.2         (675) GCGCCGATCTAAAGTCATTTGACTTAGGCGACGAGCTTGGCACTGATCCTTATGAAGATTTTCAAGAAAACTGGA-ACA
```

Section 11

```
                                                      791      800       810       820       830       840       850                 869
Oryza sativa chromosome 1 region 9.891-41.663nt (783) CTCATTGACTCTCTCTCTCTCTCACACACACCCAGATGGAATGTTACAGCCAGTGGTGCCCGCCCCAATCCGCAGGGCT
SARS-CoV-2 Reference Genome NC_045512.2         (753) CTAAACATAGCAGTGG-TGTTACCCGTGAACTCATGCGTGAGCTTAACG---GAGGGGCATACAC---TC------GCT
```

Section 12

```
                                                      870       880       890       900       910       920       930                948
Oryza sativa chromosome 1 region 9.891-41.663nt (862) CATTCCATTACGGCGACATTACCGTCACCGATGTGTACCTGCTGCAGAGCATGCCACCGGAGCTCATAGACGGCCAGAT
SARS-CoV-2 Reference Genome NC_045512.2         (819) ATGTCGATAACAACTTCTGT---GGCCCTGATGGCTACCCTCTTGAGTGCATTAAA---GACCTTCTAG-----CACGT
```

Section 13

```
                                                      949        960       970       980       990       1000      1010             1027
Oryza sativa chromosome 1 region 9.891-41.663nt (941) GCGTGCTACTCTCAATGGAATTTCTTATATTGCTCCATCGACTCCTTTGATGCTCGCTCAGCTCTTTAATG-TCCCGGG
SARS-CoV-2 Reference Genome NC_045512.2         (887) GCTGGTAAAGCTTCATGCACTTTGTCCGAA--CAAC-TGGACTTTATTGACACTAAGAGGGGTGTATACTGCTGCCGTG
```

Section 14

```
                                                       1028        1040      1050      1060      1070      1080      1090            1106
Oryza sativa chromosome 1 region 9.891-41.663nt (1019) AGTCTACAAACTGGATTTTCCTAATCGTCCTATGAACAGGTTACCAAAGCTTGACACCTCCATTATCAATGGCACCTAC
SARS-CoV-2 Reference Genome NC_045512.2          (963) AACATG--AGCATGAAATTGCT--TGGTACACGGAAC--GTTCTGAAA-----AGAGCT-----ATGAATTGCAGACAC
```

**Section 15**	(1107)	1107 1120 1130 1140 1150 1160 1170 1185
Oryza sativa chromosome 1 region 9.891-41.663nt	(1098)	AAAGGCTTTATGGAGATCATATTCCAAAACAACGCCACATCCGTGCAGAGCTACCACTTGGATGGATATGCATTCTTTG
SARS-CoV-2 Reference Genome NC_045512.2	(1026)	-----CTTTT--GAAATTAAATTGGCAAAGAA------ATTTG---ACACCTTCAATGGGGA---ATGTCCAAATTTTG
**Section 16**	(1186)	1186 1200 1210 1220 1230 1240 1250 1264
Oryza sativa chromosome 1 region 9.891-41.663nt	(1177)	TTGTTGGGTAAAAGCTTGCAGCACTCCCCCTTTCCCCTGCCCTGGATGTACTACTTATTTACTTATATATGGATATGGT
SARS-CoV-2 Reference Genome NC_045512.2	(1086)	TATTTCCCTTAAA--TTCCATAATCAAGACTATTCAA--CCAAGGGTTGAAAAGAAAAAGCTTGATGGCTTTATG-GGT
**Section 17**	(1265)	1265 1270 1280 1290 1300 1310 1320 1330 1343
Oryza sativa chromosome 1 region 9.891-41.663nt	(1256)	GGTTACTTCTCTCAGGATGGACTATGGACTGTGGACAGACAACAGCCGCGGTACATACAACAAATGGGATGGCGTTGCA
SARS-CoV-2 Reference Genome NC_045512.2	(1160)	AG--AATTCGATCTGT-----CTATCCAGTTGCGTCACCAAA---------TGAATGCAACCAAATG--TGCCTTTCAA
**Section 18**	(1344)	1344 1350 1360 1370 1380 1390 1400 1410 1422
Oryza sativa chromosome 1 region 9.891-41.663nt	(1335)	CGCTCCACAATTCAGGTGCTCAAAATTTTCATGTCTCCCATTGCTTTCTATGGATGCCGGTTTTTAACTATGACACCTG
SARS-CoV-2 Reference Genome NC_045512.2	(1221)	CTCTCATGAAGT---GTGATCATTGTGGTGAAACTTC--ATGGCA-------GACGGGCGATTTTGTTAAAGCCACTTG
**Section 19**	(1423)	1423 1430 1440 1450 1460 1470 1480 1490 1501
Oryza sativa chromosome 1 region 9.891-41.663nt	(1414)	GTAGCTAGTCTGCTGC-CTGA-AACCCAAAGAAACTAGTACTGTGCTATTACATCTG-TTACTTGATGGAGATGGAAGT
SARS-CoV-2 Reference Genome NC_045512.2	(1288)	CGA---ATTTTGTGGCACTGAGAATTTGACTAAAGAAG----GTGCCACTACTTGTGGTTACTTACCCCAAAATGCTGT
**Section 20**	(1502)	1502 1510 1520 1530 1540 1550 1560 1570 1580
Oryza sativa chromosome 1 region 9.891-41.663nt	(1490)	TAGCAGCCGATAT-GTGGGACTCGT-ACTATGTAGTAACAGAGCGGCACGGTGCACCTTCTTGTAGAAAA--ATCATGT
SARS-CoV-2 Reference Genome NC_045512.2	(1360)	TGTTAAAATTTATTGTCCAGCATGTCACAATTCAGAAGTAGGAC----CTGAGCATAGTCTTGCCGAATACCATAATGA
**Section 21**	(1581)	1581 1590 1600 1610 1620 1630 1640 1659
Oryza sativa chromosome 1 region 9.891-41.663nt	(1565)	ATGTCTTTCTATGGGGGTGGTTAGTTTTTTGAGGGATGTTGTCATATATCCATATCATTCAGATATGGAGTGGCTAAT
SARS-CoV-2 Reference Genome NC_045512.2	(1435)	ATCTGGCTTGAAAACCATTCTTCGT------AAGGGTGGTCGCACTATTGCC------TTTGGAGGCTGTGTGTTCTCT

```
                                                                Section 22
                                                  (1660) 1660      1670      1680      1690      1700      1710      1720              1738
Oryza sativa chromosome 1 region 9.891-41.663nt   (1644) TATGTGGTCAACCAATGATTCCTTTGGCATGGCAGTTTTGATGGTATAGAAACCCTCATTGTTTGTTTGGTTAGTGAT
SARS-CoV-2 Reference Genome NC_045512.2           (1502) TATGTTGGTTGCCA-TAACAAGTGTGCCTATTG--GGTTCCACGTGCTAGCG-CTAACATAGGTTGTAACCATACAGGT
                                                                Section 23
                                                  (1739) 1739       1750      1760      1770      1780      1790      1800             1817
Oryza sativa chromosome 1 region 9.891-41.663nt   (1723) AAACTTCATGCACTGGTTCAAAGTTTCAGACGACTGCATTTT--ATCTTGAGAAAACCTTCGAATTCAGGA--AATACT
SARS-CoV-2 Reference Genome NC_045512.2           (1577) GTTGTTGGAGAAG-GTTCCGAAGGTCTTAATGACAACCTTCTTGAAATACTCCAAAAAGAGAAAGTCAACATCAATATT
                                                                Section 24
                                                  (1818) 1818        1830      1840      1850      1860      1870      1880            1896
Oryza sativa chromosome 1 region 9.891-41.663nt   (1798) GTC--TGAAATATAACCTACTCATGAACTCTATACAATATTCTGGTACAGCCTTTAGCTTG-ACATGTC--TTTGTGCA
SARS-CoV-2 Reference Genome NC_045512.2           (1655) GTTGGTGACTTTAAACTTAATGAAGAGATCGC--CATTATTTTGGCATCTTTTTCTGCTTCCACAAGTGCTTTTGTGGA
                                                                Section 25
                                                  (1897) 1897          1910      1920      1930      1940      1950      1960        1975
Oryza sativa chromosome 1 region 9.891-41.663nt   (1872) AGACTGCAGGTATTCCCTGGCGCTTGGACGGCTGTCCTTGTCTTTCTTGACAA-TGCGGGTATATGGAATCTGCGGGTG
SARS-CoV-2 Reference Genome NC_045512.2           (1732) A-ACTGT--GAAAGGTTTGGA--TTATAAAGCATTCAAACAAATTGTTGAATCCTGTGGTAATTTAAAGTTACAAAAG
                                                                Section 26
                                                  (1976) 1976           1990      2000      2010      2020      2030      2040       2054
Oryza sativa chromosome 1 region 9.891-41.663nt   (1950) GAGAACCTGGACGCCTGGTACCTGGGGCAAGAAGTGTACATCAGT--GTGGTTAACCCGGAGGATAGCAGCAACAAGAC
SARS-CoV-2 Reference Genome NC_045512.2           (1806) GAAAAGCTAAAAAA--GGTGCCTGGAATATTG-GTGAACAGAAATCAATACTGAGTCCTCTTTAT-GCATTTGCATCA-
                                                                Section 27
                                                  (2055) 2055 2060      2070      2080      2090      2100      2110      2120         2133
Oryza sativa chromosome 1 region 9.891-41.663nt   (2027) CGTGCTTCCTCTTCCCGACAACGCGATTTTCTGTGGTGCGCTGTCAAGTTTACAAAAGTGAGGCTTCTCATGACAGCGG
SARS-CoV-2 Reference Genome NC_045512.2           (1880) -GAGGCTGCTCGTGTTGTACGATCAATTTTCTCCCGCACTCTTGAAACTGCTCAAAA-------TTCT---GTGCGTGT
                                                                Section 28
                                                  (2134) 2134  2140      2150      2160      2170      2180      2190      2200        2212
Oryza sativa chromosome 1 region 9.891-41.663nt   (2106) TTTTCAGTTTGCAGCTCAGGGATGCCTACCTGTTATATAACA-TGTATAGCGTTTGTTGTTGATTCCGTGCAGGGAGCA
SARS-CoV-2 Reference Genome NC_045512.2           (1948) TTTACAGAAGGCCGCTATAACAATACTAGATGGAATTTCACAGTATTCACTGAGACTCATTGATGCTATGATGT-----
```

Section 29

	(2213)	2213 2220 2230 2240 2250 2260 2270 2280 2291
Oryza sativa chromosome 1 region 9.891-41.663nt	(2184)	ATCGCATA-GATTCCAATACTCAGAAGCTTCTCAGATAACGCAGCTGTGGAAGATGGTTTTCTTCATGGCATGGTTGGC
SARS-CoV-2 Reference Genome NC_045512.2	(2022)	-TCACATCTGATTTGGCTACTAACAATCTAGTTGTA-ATGGCCTACATTACAGGTGGTGTTGTTCA-------GTTGAC

Section 30

	(2292)	2292 2300 2310 2320 2330 2340 2350 2360 2370
Oryza sativa chromosome 1 region 9.891-41.663nt	(2262)	CCTTTGGTAGAGCTTAATTAGATCTGTCTGCTGGGGTGATCCGGAGACGAGAGAGCTCAGAGCTGGTCCCAAACATGGA
SARS-CoV-2 Reference Genome NC_045512.2	(2092)	--TTCGCAGTGGCTAACTAACATCTTTG-GCACTGTTTAT---GAAA------AACTCAAACCCG-TCC------TTGA

Section 31

	(2371)	2371 2380 2390 2400 2410 2420 2430 2449
Oryza sativa chromosome 1 region 9.891-41.663nt	(2341)	TGAGCTCTCAGCTCAAGTTA-GTAGGGTGGTGAATTTTGTCAGATTCGAATTGGTTAGATTGGACTTGAGTGATGATC-
SARS-CoV-2 Reference Genome NC_045512.2	(2152)	TTGGCTTGAAGAGAAGTTTAAGGAAGGTGTAGAGTTTCTT-AGAGACGGTTGGG--AAATTG---TTAAAT--TTATCT

Section 32

	(2450)	2450 2460 2470 2480 2490 2500 2510 2528
Oryza sativa chromosome 1 region 9.891-41.663nt	(2418)	CTGTCTGGGAAGGGCAAAGAGTAGGAGGAAGCATTGGGAAG-GCATACACACGCTTGACTTGATTTGGCTTGAAGTCG-
SARS-CoV-2 Reference Genome NC_045512.2	(2223)	CAACCTGTGCTTGTGAAATTGTCGGTGGACAAATTGTCACCTGTGCAAAGGAAATTAAGGAGAGTGTTCAGACATTCTT

Section 33

	(2529)	2529 2540 2550 2560 2570 2580 2590 2607
Oryza sativa chromosome 1 region 9.891-41.663nt	(2495)	--AGCTAGCAGGTTGCTCATTAGCCGTTGTGTGATATCTGAAG-ATCAACTTACTACCCAAACCTAGACTCACAGAAAC
SARS-CoV-2 Reference Genome NC_045512.2	(2302)	TAAGCTTGTAAATAAATTTTTGGCT-TTGTGTGCTGACTCTATCATTATTGGTGGAGCTAAACTTAAAGCCTT-GAATT

Section 34

	(2608)	2608 2620 2630 2640 2650 2660 2670 2686
Oryza sativa chromosome 1 region 9.891-41.663nt	(2571)	CAATTGATTGATATGGATTAAGACCTGAAGCTTCTTGGGTTTTCCTAAGTAATCCATTTGTTG-GTGGTTAC-GGTATC
SARS-CoV-2 Reference Genome NC_045512.2	(2379)	TAGGTGAAACATTTG--TCACGCACTCAAAGGGATTG---TACAGAAAGTGTGTTAAATCCAGAGAAGAAACTGGCCTA

Section 35

	(2687)	2687 2700 2710 2720 2730 2740 2750 2765
Oryza sativa chromosome 1 region 9.891-41.663nt	(2648)	CTTTTTCTTGTATTGACAGCAAGCAGAAAGTTGTTTTAAAAGAACAGCAGGCAGAAAGAAAATAATTATGGTAGTTGGG
SARS-CoV-2 Reference Genome NC_045512.2	(2453)	CTCATGCCTCTAAAAGCCCCAAA-AGAAA-TTATCTTCTTAGA------GGGAGAAACAC--TTCCCACAGAAGTGT--

Section 36

	(2766)	2766 2780 2790 2800 2810 2820 2830 2844
Oryza sativa chromosome 1 region 9.891-41.663nt	(2727)	CCTAACTGAATTAGTATTCTAAGAATATGTCAGTTTACAATCTTAATTCTTAAGAAAGTCTAAAAGTCGTGCATGTGCG
SARS-CoV-2 Reference Genome NC_045512.2	(2520)	--TAACAGAGGAAGTTGTCTTGAAAACTGGTGATTTACAACCAT------TAGAACAACCTACTAGTGAAGCTGTTGA-

Section 37

	(2845)	2845 2850 2860 2870 2880 2890 2900 2910 2923
Oryza sativa chromosome 1 region 9.891-41.663nt	(2806)	TTCCGAGCACACACTTTTTCGTATGAAAGTTTCAGATAAAAGTTTACGGATGAAAGTTTTTTTTTTGAAAAAAAAAAGA
SARS-CoV-2 Reference Genome NC_045512.2	(2590)	-----AGCTC-CATTGGTTGGTACACCAGTTTG---TA----TTAACGGGCTTATGTTGCTCGAAATCAAAGACACAGA

Section 38

	(2924)	2924 2930 2940 2950 2960 2970 2980 2990 3002
Oryza sativa chromosome 1 region 9.891-41.663nt	(2885)	TGCGCGCTGGAAATGCATTTCCGATTTCATGCAAGGTTTTATAAGGTACTTTTTTTTTAAAACCTTTTATATATAAATA
SARS-CoV-2 Reference Genome NC_045512.2	(2656)	AAAGTACTG----TGCCCTTGCACCTAATATGATGGTAACAAACAATACCTTCACACTCAAAGGCGGTGCACC--AACA

Section 39

	(3003)	3003 3010 3020 3030 3040 3050 3060 3070 3081
Oryza sativa chromosome 1 region 9.891-41.663nt	(2964)	TAAGGTATTAGGCTTGATCTCACAAGTGTAGGGAAATGGAATCTGACTTTAGATGACAAGTGGTGGCTTTGTTTGCTCC
SARS-CoV-2 Reference Genome NC_045512.2	(2729)	AAGGTTACTTTTGGTGATGACACT-GTG-ATAGAAGTGCAAGGTTACAAGAG-TGTGAATA--TCACTTT-TGAACTTG

Section 40

	(3082)	3082 3090 3100 3110 3120 3130 3140 3150 3160
Oryza sativa chromosome 1 region 9.891-41.663nt	(3043)	TTGCCTCCTTGGCCAGGGGAG--AA-GGCAGTTGACCTACCTAGGTAAAAATTACACAAGGAAAAAAAGAGA--GAGTT
SARS-CoV-2 Reference Genome NC_045512.2	(2802)	ATGAAAGGATTGATAAAGTACTTAATGAGAAGTGCTCTGCCTAT---ACAGTTGAACTCGGTACAGAAGTAAATGAGTT

Section 41

	(3161)	3161 3170 3180 3190 3200 3210 3220 3239
Oryza sativa chromosome 1 region 9.891-41.663nt	(3117)	TCTCTTTCTTTTTCT--TTTCTCTCTTTGTTTTTGAGCTGGAGGATTCTTCTCTCTTCTTAAGCTCATCAGGGCCAGCT
SARS-CoV-2 Reference Genome NC_045512.2	(2878)	CGCCTGTGTTGTGGCAGATGCTGTCATAAAAACTTTGCAACCAGTATCTGAATTA--CTTA--CACCACTGGGCAT--T

Section 42

	(3240)	3240 3250 3260 3270 3280 3290 3300 3318
Oryza sativa chromosome 1 region 9.891-41.663nt	(3194)	AAGCCAGCACAA-GCAAAAGGACTCTGTATTTTCTGTGTTGTCTCCTCTAGTCCTCTCCTCCCTTCTGTTCCAGTCAGG
SARS-CoV-2 Reference Genome NC_045512.2	(2951)	GATTTAGATGAGTGGAGTATGGCTACATACTACTTAT-TTGATGAGTCTGGTGAGTT--TAAATTGGCTTC--------

```
                                                                                    Section 43
                                            (3319) 3319       3330           3340           3350           3360           3370           3380                3397
Oryza sativa chromosome 1 region 9.891-41.663nt (3272) ACATACAAAGCAAAGATCCCTCCCTCTGCTGTGCAGGCTGCAGCAGGCGAGGAGAGGCAACCAGAGGGAGGGTAGGTTG
SARS-CoV-2 Reference Genome NC_045512.2         (3019) ACATATGTATTGTTCTTTCTACCCTC--CAGATGAGGATGAAGAAGAAGGTGATTGTGAAGAAGAAG------AGTTTG
                                                                                    Section 44
                                            (3398) 3398         3410           3420           3430           3440           3450           3460                3476
Oryza sativa chromosome 1 region 9.891-41.663nt (3351) GTTTGGAAGGAAGGAAGGAGAAGAGATAAACAAGTTAGGAGGAGACAAGCATCCGATTCCGAACTTTGATGGATCCCCT
SARS-CoV-2 Reference Genome NC_045512.2         (3090) AGCCATCAACTCAATATGAGTATGG-TACTGAAGAT--GATTACCAAGGTAAACCTTT--GGAATTTGGTG--------
                                                                                    Section 45
                                            (3477) 3477         3490           3500           3510           3520           3530           3540                3555
Oryza sativa chromosome 1 region 9.891-41.663nt (3430) CCATATCTATCTTCCATCCAATCCAATCTCTGCCCTCAATTGAGATTCGAGAGCTAAGCTAAGCCACGCCAAATTAAG
SARS-CoV-2 Reference Genome NC_045512.2         (3156) CCACTTCTG-CTGCTCTTCAACCTGAAGAAGAGCAAGAAGA--AGATTGGTTAGATGATGATAGTCAACAAACTGTTGG
                                                                                    Section 46
                                            (3556) 3556           3570           3580           3590           3600           3610           3620                3634
Oryza sativa chromosome 1 region 9.891-41.663nt (3509) CCATGGCGTGCACGGCGGCGCGCATGTTCGCCTCCAACGCCACCCTCTGCGCCTGCGAGCCGGGCTTCTACCTCTCCGC
SARS-CoV-2 Reference Genome NC_045512.2         (3232) TCAACAAG---ACGGCAGTGAGGACAATCAGA--CAACTAC----TATTCAAACAATTGTTGAGGTTCAACCTCAATT-
                                                                                    Section 47
                                            (3635) 3635 3640           3650           3660           3670           3680           3690           3700                3713
Oryza sativa chromosome 1 region 9.891-41.663nt (3588) CGCCATCAACGGCACCTGCCTCGGCCTGCCCGACGGCGGCTGGCAGGTCGGCTCCGTCGGCGCCTCCCGCAACCAGAGC
SARS-CoV-2 Reference Genome NC_045512.2         (3301) ----AGAGATGGAACTTACACCAGTTGTTCAGACTATTGAAGTGAA-TAGTTTTAGTGGTTATTTAAAACTTACTGACA
                                                                                    Section 48
                                            (3714) 3714 3720           3730           3740           3750           3760           3770           3780                3792
Oryza sativa chromosome 1 region 9.891-41.663nt (3667) TTCTACTTCCTCACCCCCGTCCTCTCCCTCGACGTCGTCCGCCGCCTCACCCAGTCCCAGGCCCTCCTCCTCGAGGCCA
SARS-CoV-2 Reference Genome NC_045512.2         (3375) ATGTA-TACATTAAAAATGCAGACATTGTGGAAGAAG-----CTAAAAAGGTAAAACCAA-CAGTGGTTGTTAATGCAG
                                                                                    Section 49
                                            (3793) 3793  3800           3810           3820           3830           3840           3850           3860                3871
Oryza sativa chromosome 1 region 9.891-41.663nt (3746) CCATCGCTGCCCTCCTCTCCTGGCTTGCCTTTTGTGCCTTCGCCAGGTTCACCGGACACGACCCCACCGGCAACAAGAG
SARS-CoV-2 Reference Genome NC_045512.2         (3447) CCAATGTTTACCTTAAA-CATGGAGGAGGTGTTG---------CAGGAGC--CTTAAATAAGGCTACT---AACAA---
```

Section 50

(3872) 3872 3880 3890 3900 3910 3920 3930 3940 3950
Oryza sativa chromosome 1 region 9.891-41.663nt (3825) GCTCTTCCGCGCGCGCTTCTGGGTCAGCCGCCTCGACTGCATCTACGACACCACCCACTG-GGCGGTTAGTTTTCTTTT
SARS-CoV-2 Reference Genome NC_045512.2 (3508) ----TGCCATGCAAGTT---GAATCTGATGATTACATAGCTACTAATGGACCACTTAAAGTGGGTGGTAGTTGTGTTTT

Section 51

(3951) 3951 3960 3970 3980 3990 4000 4010 4029
Oryza sativa chromosome 1 region 9.891-41.663nt (3903) TTTCCCCTTTTTTTTACTAATAACATCTTGTTTCTTACAAATTTCTTTTGCCCTCGCCGAACTTAGATTTTCATCAAGG
SARS-CoV-2 Reference Genome NC_045512.2 (3580) AAGCGG-------------ACACAATCTTGCTAAACACTGTCTTCATGTTGTCGGCCCAAATGTTAACAAAGGTGAAGA

Section 52

(4030) 4030 4040 4050 4060 4070 4080 4090 4108
Oryza sativa chromosome 1 region 9.891-41.663nt (3982) CCAAATTAATTGCTTTTGAGTCCTTCTACTCATGTCGAAATTGAGGATTGCTTTGCTTCGCTTGTTAGGAATCTCCTCT
SARS-CoV-2 Reference Genome NC_045512.2 (3646) C--ATTCAACTTCTTAAGAGTGCTT------ATGAAAATTTTAATCAGCACGAAGTTCTACTTGC-ACCATTATTATCA

Section 53

(4109) 4109 4120 4130 4140 4150 4160 4170 4187
Oryza sativa chromosome 1 region 9.891-41.663nt (4061) CCTAAGGAACAAGTTTTTGCTTTGCTACTACTTTAATTTCTTATCCATCACACTCCTTTTGCCGCTCCTTCCTACTTCG
SARS-CoV-2 Reference Genome NC_045512.2 (3716) GCT--GGTA----TTTTTGGT--GCTGACCCTATACATTCTT-----TAAGAGT--TTGTGTAGATACTG-----TTCG

Section 54

(4188) 4188 4200 4210 4220 4230 4240 4250 4266
Oryza sativa chromosome 1 region 9.891-41.663nt (4140) GCACATTGCACTTTCTTACCAGTATTAGATATTGGATATGGATATTATACATTATACACTACGAACACTCCTTATATCC
SARS-CoV-2 Reference Genome NC_045512.2 (3775) -CACAAATGTCTA-CTTAGCTGTCTTTGATA----------AAAATCTCTATGACAAACTTGTTTCAAGCTTTTT----

Section 55

(4267) 4267 4280 4290 4300 4310 4320 4330 4345
Oryza sativa chromosome 1 region 9.891-41.663nt (4219) AAGGAAATTTATTTTCTGCACTAATTTCTACTTTTCCAAGTTGCATTGAATATTGATGTACCTTGCCGTACTGTAGGAT
SARS-CoV-2 Reference Genome NC_045512.2 (3838) --GGAAATGAAGAGTGAAAAG---------------CAAGTTGAACAAAAGATCGCTG-AGATTCC--TAAAG-AGGAA

Section 56

(4346) 4346 4360 4370 4380 4390 4400 4410 4424
Oryza sativa chromosome 1 region 9.891-41.663nt (4298) GACCAGCAAGTACTGAGGAAAAGAAAAACAGAGTTGGGTGGTAC-GTGCTCAGTAGCTAGCTTGATACTCTTCGTTGGA
SARS-CoV-2 Reference Genome NC_045512.2 (3896) GTTAAGCCATTTATAACTGAAAGTAAACC----TTCAGTTGAACAGAGAAAACAAGATGATAAGAAAATCAAAGCTTG-

Section 57

```
                                                  (4425) 4425 4430      4440      4450      4460      4470      4480      4490     4503
Oryza sativa chromosome 1 region 9.891-41.663nt   (4376) TTAGTCACTGTGTAAGTGCTTAACAAAGTCTAGTGTTGGAAGGAA-TTTGTTACTCACTCACTAACAGGGTGTCTTCTT
SARS-CoV-2 Reference Genome NC_045512.2           (3970) --------TGTTGAAGAAGTTA-CAACAACTC----TGGAAGAAACTAAGTTCCTCACAGAA-AACTTGTTACTTTATA
```

Section 58

```
                                                  (4504) 4504 4510      4520      4530      4540      4550      4560      4570     4582
Oryza sativa chromosome 1 region 9.891-41.663nt   (4454) TCCTTGCTTGTTGCAGGCTGCTG---TACCAAGCCATCCAAAGACGCAACATTGAAGTGCATCGGGTCAAACCAGCCAA
SARS-CoV-2 Reference Genome NC_045512.2           (4035) TTGACATTAATGGCAATCTTCATCCAGATTCTGCCACTCTTGTTAGTGACATTGACATCACTTTCTTAAAGAAAG---A
```

Section 59

```
                                                  (4583) 4583 4590      4600      4610      4620      4630      4640      4650     4661
Oryza sativa chromosome 1 region 9.891-41.663nt   (4530) TGCTCCTGATTTGTTGTCTTTTGTCAATGATATCGAGTTCCACATAACCACCATCTCTAGCATGTCTTGCTCTCAGTTG
SARS-CoV-2 Reference Genome NC_045512.2           (4111) TGCTCCATATATAGTGGGTGATGT---TGTTCAAGAGGGTGTTTTAAC-------------------TGCTGT-----G
```

Section 60

```
                                                  (4662) 4662 4670      4680      4690      4700      4710      4720      4730     4740
Oryza sativa chromosome 1 region 9.891-41.663nt   (4609) GTTGCCCCTTCTACAATTGCAATGGGAACACCTGGATCTATGGACTTCAGGCTGCTGCCTTTGTCAACATTGTTGACAT
SARS-CoV-2 Reference Genome NC_045512.2           (4163) GTTATACCTACTAAAAAGGCTGGTGGCACTACTGAA---ATG--CTAGCGAAAGCT----TTGAGAAAAGTGCCAACAG
```

Section 61

```
                                                  (4741) 4741 4750      4760      4770      4780      4790      4800               4819
Oryza sativa chromosome 1 region 9.891-41.663nt   (4688) ACAATTGCCAGAACACGAGCCAGGGGCCATCTGTTTCACTCAAGTGCAATGGCTGCAGAATCCCGCCAAGAGACCACTA
SARS-CoV-2 Reference Genome NC_045512.2           (4233) ACAATTATATAACCACTTACCCGGGTCAGG--GTTT-----AAATGGTTACACTGTAGAGGAG-GCAAAGACAGTGCTT
```

Section 62

```
                                                  (4820) 4820 4830      4840      4850      4860      4870      4880               4898
Oryza sativa chromosome 1 region 9.891-41.663nt   (4767) CGTATCCTGGCAATTTATTGACCTGCCAAGGCAACCAGCTGCCGCTGTTGGCTTCCAGTTTAATCTC-ACTGCGAAGCA
SARS-CoV-2 Reference Genome NC_045512.2           (4304) AAAAAG-TGTAAA---AGTGCCTTTTACATTCTACCATCTA-------------------TTATCTCTAATGAGAAGCA
```

Section 63

```
                                                  (4899) 4899 4910      4920      4930      4940      4950      4960               4977
Oryza sativa chromosome 1 region 9.891-41.663nt   (4845) ACATGGAGATGACAAACATGTGAGCTCTGTCAGTGGAACTATAAACTCCGACAATT--TTACTGATGACAAGCTGAAGA
SARS-CoV-2 Reference Genome NC_045512.2           (4360) A---------GAAATTCTTG-GAACTGTTCT-TGGAATT------TGCGAGAAATGCTTGCACATGC--AGAAGAA-A
```

```
                                                             Section 64
                                         (4978) 4978        4990      5000      5010      5020      5030      5040            5056
Oryza sativa chromosome 1 region 9.891-41.663nt (4922) CATTCAGAGGGAGAGATTCCAATGTGCTCAAGATT-CAATTGTTTCCTCAAACATACATC--AATCATCACAATTTGAA
   SARS-CoV-2 Reference Genome NC_045512.2 (4419) CACGCAAATTAATGCCTGTCTGTGTGGAAACTAAAGCCATAGTTTC---AACTATACAGCGTAAATATAAGGGTATTAA
                                                             Section 65
                                         (5057) 5057         5070      5080      5090      5100      5110      5120           5135
Oryza sativa chromosome 1 region 9.891-41.663nt (4998) GCTTCTGCAACCTCTGGTTCAGGACTTCACTCAAGGTTCTACCTTTTCAAATGTTAGA-AACTTGAATGCTTCCTTGCA
   SARS-CoV-2 Reference Genome NC_045512.2 (4495) AATACAAGAGGGTGTGGTTGATTATGGTGCT--AGATTTTACTTTTACACCAGTAAAACAACTGTAGCGTCACTTATCA
                                                             Section 66
                                         (5136) 5136          5150      5160      5170      5180      5190      5200          5214
Oryza sativa chromosome 1 region 9.891-41.663nt (5076) AAACCCT-ATGGATGGAATAATAAATACTACACTCTACATAAGCTATCTTTCCAACTACATTGTGGAGATTAGCAACGA
   SARS-CoV-2 Reference Genome NC_045512.2 (4572) ACACACTTAACGATCTAA-ATGAAACTCTTGTTACAATGCCACTTGGCTATGTAAC-ACATGGCTTAAATTTGGAAG--
                                                             Section 67
                                         (5215) 5215 5220      5230      5240      5250      5260      5270      5280         5293
Oryza sativa chromosome 1 region 9.891-41.663nt (5154) AAATGTGCTAGGCCCAGGTGAGCTTTTCTAGTGATTGTTGATACCTACATAAGTCATCTTTCCAGCCTATTGAAAATGA
   SARS-CoV-2 Reference Genome NC_045512.2 (4647) -AAGCTGCTCGGTATATGAGATCTCTCAAAGTGCCAGCT-ACAGTTTC-TGTTTCTTCACCTGATGCTGTT-ACAGCGT
                                                             Section 68
                                         (5294) 5294  5300      5310      5320      5330      5340      5350      5360        5372
Oryza sativa chromosome 1 region 9.891-41.663nt (5233) AGTCTCTTTACTTGTGAATTGTGGTGTTAATGTTACTAAGATTTACGCTACTAAGTACCAATACAAATATTTGAAATCC
   SARS-CoV-2 Reference Genome NC_045512.2 (4722) ATAATGGTTA--TCTTACTTCTTCTTCTAAAACACCTGAAGAACATTTTATTGAA-ACCATCTCACTTGCTGGT--TCC
                                                             Section 69
                                         (5373) 5373   5380      5390      5400      5410      5420      5430      5440       5451
Oryza sativa chromosome 1 region 9.891-41.663nt (5312) CGGTGCAAATATTTTCTCCATGATGGCTTTTTTAAATTTTTGGGCTTCTACTAATCTGAATAGTTTGTATTACTAGTAT
   SARS-CoV-2 Reference Genome NC_045512.2 (4796) ---TATAAAGATTGGTCCTATTCTGGACAATCTACACAACTAGGTATAGAATT-TCTTAAGAGAG-GTGATAAAAGTGT
                                                             Section 70
                                         (5452) 5452    5460      5470      5480      5490      5500      5510      5520      5530
Oryza sativa chromosome 1 region 9.891-41.663nt (5391) CTAATATGCTTATTTTCTGCAGTCAGTATACTTGCAAGTATTGGTGGCCTTTATGCCTT---CAGTGTGGCAATATTTC
   SARS-CoV-2 Reference Genome NC_045512.2 (4870) ATATTACACTAGTAATCCTACCACATTCCACCTAGA-----TGGTGAAGTTATCACCTTTGACAATCTTAAGACACTTC
```

Section 71

```
                                                  (5531)  5531     5540      5550      5560      5570      5580      5590                5609
Oryza sativa chromosome 1 region 9.891-41.663nt  (5467)  TTTGCCTTATGGCTCAAGTAAGTCATCACAAATCTATCCCTGTTGTTTGTTTAAGATAAACTGCCAGTTTTTTTTTTCA
SARS-CoV-2 Reference Genome NC_045512.2          (4944)  TTT--CTT-TGAGAGAAGTGAGGACTATTAA----------GGTGTTT-----ACAACAGTAGACAACATTAACCTCCA
```

Section 72

```
                                                  (5610)  5610      5620      5630      5640      5650      5660      5670              5688
Oryza sativa chromosome 1 region 9.891-41.663nt  (5546)  CTTTCGAGTTTTA-ACTAGCCTAACACTTGTTGACCTCTGAATCTTTGCATCTTTATGGGGTTTTCTGTTCCACTCAGA
SARS-CoV-2 Reference Genome NC_045512.2          (5005)  CACGCAAGTTGTGGACATGTCAATGACATATGGACAACAGTTTGGTCCAACTTATTTGGATGGAGCTGATG--------
```

Section 73

```
                                                  (5689)  5689       5700      5710      5720      5730      5740      5750            5767
Oryza sativa chromosome 1 region 9.891-41.663nt  (5624)  ATTACTAATAGGCCATGAAAATTTTCTGATATTTTTTAAATGAATTGAATTAACATACTTGCAATTTCCGCTCATTATC
SARS-CoV-2 Reference Genome NC_045512.2          (5076)  -TTACTAAAA-----TAAAAC----CTCATAATTC--ACATGAAGGTAA--AACAT--TTTATGTTTTACCTAATGATG
```

Section 74

```
                                                  (5768)  5768        5780      5790      5800      5810      5820      5830           5846
Oryza sativa chromosome 1 region 9.891-41.663nt  (5703)  ATGGTTCAAGGCGTGTG-CTCTGGTGCACATCCTTCCTCGACAGCC-AGCTGTCGATGTAAAACTTTTCTTGTAACCTT
SARS-CoV-2 Reference Genome NC_045512.2          (5139)  ACACTCTACGTGTTGAGGCTTTTGAGTACTACCACACAACTGATCCTAGTTTTCTGGGTAGG----TACATGTCAGCAT
```

Section 75

```
                                                  (5847)  5847         5860      5870      5880      5890      5900      5910          5925
Oryza sativa chromosome 1 region 9.891-41.663nt  (5780)  TTCTTCCTATCTTAATTACATTGAATGACCTCCTTCGGTCATTCCGGCAAAAAAGAAGATGACTTAGTATGCAGCTGTA
SARS-CoV-2 Reference Genome NC_045512.2          (5214)  TAAATCACA-CTAAA--AAGTGGAAATACCCACAA-GTTAAT---GGTTTAACTTCTATTAAATGGGCAGATAAC---A
```

Section 76

```
                                                  (5926)  5926          5940      5950      5960      5970      5980      5990         6004
Oryza sativa chromosome 1 region 9.891-41.663nt  (5859)  ATTGATGCTTTGTCTTTCCAGTGTGAAGCTAGGATAAAAAAGCTTCGAGACGAGGATTCCAGAATGCTAAAAATACTGA
SARS-CoV-2 Reference Genome NC_045512.2          (5283)  ACTGTTATCTTGCCACTGCATTGTTAAC--ACTCCAACAAA---TAGAGTTGAAGTTT----AATCC---ACCTGCTCT
```

Section 77

```
                                                  (6005)  6005 6010      6020      6030      6040      6050      6060      6070          6083
Oryza sativa chromosome 1 region 9.891-41.663nt  (5938)  GGAAAAGACGTGCTCAACAGAATTGGGATAAGGTGAAGAATGTCCTGATCTAATGATGAACATTGTACCTACTAGACTG
SARS-CoV-2 Reference Genome NC_045512.2          (5350)  ACAAGATGCTTATTACAGAGCAA-GGGCT--GGTGAAG------CTGCTA-ACTTTTGTGCACT-TATCT--TAGCCTA
```

```
Section 78
                                                      (6084) 6084  6090      6100      6110      6120      6130      6140      6150      6162
Oryza sativa chromosome 1 region 9.891-41.663nt       (6017) CATCCCTGATCAAGCATATCATTTTTCTTCCTAATAGGTGAGGAAGTTTGTCATGTATACCTGGGGCCCTAGCAACTTG
SARS-CoV-2 Reference Genome NC_045512.2               (5416) CTGTAATAAGACAGTAGGTGAGTTAGGTG----ATGTTAGAGAAACAATG--AGTTACTTGTTTCAACATGCCAATTTA

Section 79
                                                      (6163) 6163   6170      6180      6190      6200      6210      6220      6230      6241
Oryza sativa chromosome 1 region 9.891-41.663nt       (6096) GATCCAAGTGATAGAAG-TGGCAAATGGCCTGAAAGTTCAGTGATGGATTCTCTCCATGGATCCTTCCACAAGAAAAGA
SARS-CoV-2 Reference Genome NC_045512.2               (5489) GATTCTTGCAAAAGAGTCTTGAACGTGGTGTGTAAA---ACTTGTGGACAACAGCAGACAACCCTTAAGGGTGTAGAAG

Section 80
                                                      (6242) 6242    6250      6260      6270      6280      6290      6300      6310      6320
Oryza sativa chromosome 1 region 9.891-41.663nt       (6174) AAACCGATTAGACGAGCAACCTCGAATGGTAACAAACCTAAGAGAAATCCTGCTGACGCGGTAAAGTAACTGTCCTTTT
SARS-CoV-2 Reference Genome NC_045512.2               (5565) CTGTTATGTACATGGGCACACTTTCTTATGAACAATT-TAAGAAGGTGTT-CAGATACCTT---GTACGTGTGGTAAA

Section 81
                                                      (6321) 6321     6330      6340      6350      6360      6370      6380               6399
Oryza sativa chromosome 1 region 9.891-41.663nt       (6253) CAA-CTACTCTTTATGGCACTATTTATACATTTGCCATTCAATACAGTAAAATACGTTTATACCAGAAAAAGGGTACCC
SARS-CoV-2 Reference Genome NC_045512.2               (5639) CAAGCTACAAAATAT----CTAGTACAACAGGAGTCA--CCTTTTGTTATGAT--GTCAGCACCACC-------TGCTC

Section 82
                                                      (6400) 6400      6410      6420      6430      6440      6450      6460               6478
Oryza sativa chromosome 1 region 9.891-41.663nt       (6331) TGTGTACATTTTTCGATACT-CTTTTTTTTTACTTCATTCTTTCTCTGACAAGGTTTTACTTCATTCCTTGTTGAATGA
SARS-CoV-2 Reference Genome NC_045512.2               (5703) AGTATGAACTTAAGCATGGTACATTTACTTGTGCTAGTGAGTACACTGGTAA----TTACCAGTGTGGTCACT--ATAA

Section 83
                                                      (6479) 6479       6490      6500      6510      6520      6530      6540             6557
Oryza sativa chromosome 1 region 9.891-41.663nt       (6409) TTATCTTATTTTTCCTGTTTCAATG-ATATCACAGGGAGTTATTGACATCGAAAGAGTTGGGGAAATGCAGGAGTCGAG
SARS-CoV-2 Reference Genome NC_045512.2               (5776) ACATATAACTTCTAAAGAAACTTTGTATTGCATAGACGGTGCTTTACTTACAAAGTCCTCAG--AATACAAAGGTCC--

Section 84
                                                      (6558) 6558        6570      6580      6590      6600      6610      6620             6636
Oryza sativa chromosome 1 region 9.891-41.663nt       (6487) TAGTTCTAGACAGGCTTAATGGATGAAAGCTGTGGATGCTATTGGTCATTGAAATGTGATTGCTCTTAGTCTCTGACTC
SARS-CoV-2 Reference Genome NC_045512.2               (5851) TATTAC--GGATGTTTTCTACAAAGAAAACAGT---TACACAACAACCATAAAACCAGTTACTTATAAAT---TGGATG
```

```
                                                         Section 85
                                              (6637) 6637         6650      6660      6670      6680      6690      6700          6715
Oryza sativa chromosome 1 region 9.891-41.663nt (6566) ATGGCTGATTCTACACAAATGTGCTTGTAATTTTTATCCCTTTCAATAGTTCCGCATAAAGAATTTTTTTGTTGCAGCA
SARS-CoV-2 Reference Genome NC_045512.2         (5922) GTGT-TGTTTGTACAGAAAT-TGACCCTAAGTTGGACAATTATTATAAG------AAAGACAATTCTTATTTCACAGAG
                                                         Section 86
                                              (6716) 6716           6730      6740      6750      6760      6770      6780          6794
Oryza sativa chromosome 1 region 9.891-41.663nt (6645) GCATCATTGGAGCGGCATACCGTGGCTTGGCTATATGGCAGGTCCAACACAACCGTACTACCAGAGAGGTTTTCTTGTA
SARS-CoV-2 Reference Genome NC_045512.2         (5993) CAACCAATTGATCTTG-TACCAAACCAA--CCATATC------CAAACGCAAGCTT----CGATAATTTTAAGTTTGTA
                                                         Section 87
                                              (6795) 6795 6800      6810      6820      6830      6840      6850      6860          6873
Oryza sativa chromosome 1 region 9.891-41.663nt (6724) TGT-ATGTTGCAACAAATGGGGAGAAATGTACGGAGTTTTAACTATTAATCAGCATGTTGGCTGCTGCCGAGGTCAG-G
SARS-CoV-2 Reference Genome NC_045512.2         (6059) TGTGATAATATCAAATTTGCTGATGATTTAAACCAGTT--AACTGGTTATAAGAAACCTG-CTTCAAGAGAGCTTAAAG
                                                         Section 88
                                              (6874) 6874  6880      6890      6900      6910      6920      6930      6940          6952
Oryza sativa chromosome 1 region 9.891-41.663nt (6801) CCAGAATCCTCTTTGATTTTCATGG-GACTTCGTTTCTTGAGTTGTCGAACACTGTATATACCTTCACCAGTTACCACC
SARS-CoV-2 Reference Genome NC_045512.2         (6135) TTACATTTTTCCCTGACTTAAATGGTGATGTGGTGGCTATTGATTATAAACACTACACACCCTCTTTTAAGAAAGGAGC
                                                         Section 89
                                              (6953) 6953   6960      6970      6980      6990      7000      7010      7020          7031
Oryza sativa chromosome 1 region 9.891-41.663nt (6879) CTCCACGTGCAATTA--CAGTGGGGAAGAAACAGAAAGATGC--CTATCATTTCTCTATATGAAGCAGATGGTGGCATT
SARS-CoV-2 Reference Genome NC_045512.2         (6214) TAAATTGTTACATAAACCTATTGTTTGGCATGTTAACAATGCAACTAATAAAGCCACGTATAAACCAAATACCTGG---
                                                         Section 90
                                              (7032) 7032     7040      7050      7060      7070      7080      7090      7100        7110
Oryza sativa chromosome 1 region 9.891-41.663nt (6954) TGTATTTTAATTTATTACATCGTGTCATCGTGTAAATCTTCGTGTTGGTGTGATTCGCCGACCGTTTTCTAGCCGGCTG
SARS-CoV-2 Reference Genome NC_045512.2         (6290) TGTATACGTTGTCTTTGGAGCACAAAACCAGTTGAAACATCAAATTCGTTTGATGTACTGAA-GTCAGAGGACGCGCAG
                                                         Section 91
                                              (7111) 7111     7120      7130      7140      7150      7160      7170                7189
Oryza sativa chromosome 1 region 9.891-41.663nt (7033) ATTATATATGCCAAACTTATACTCCGTAAGTAGGGAAAGGTGACCTTCAGTTTCAGGTTGAGAGATTCTACGTGGTACT
SARS-CoV-2 Reference Genome NC_045512.2         (6368) GGAATGGAT---AATCTTGC-CTGCG-AAGATCTAAAACCAGTCTCTGAAGAAGTAGTGGAAA-ATCCTACCA--TACA
```

Section 92

	(7190)	7190 7200 7210 7220 7230 7240 7250 7268
Oryza sativa chromosome 1 region 9.891-41.663nt	(7112)	GTGATA-GCTCTTGCTTTTGATGGACATGTAATGTGTTGTTGTTGACTATGTGGTACTTTGTGATGCTTGGACATGTTT
SARS-CoV-2 Reference Genome NC_045512.2	(6439)	GAAAGACGTTCTTGAGTGTAATG----TGAAAACTACCGAAGTTG--TAGGAG--ACATT---ATACTTAAACCAGCAA

Section 93

	(7269)	7269 7280 7290 7300 7310 7320 7330 7347
Oryza sativa chromosome 1 region 9.891-41.663nt	(7190)	ATATGTGGTGCTATGTTAAAAAATCCTGTTGAAATTGTGTCAATTATTGCTGAAATTGTGTCAAAATTCTATCTTATAT
SARS-CoV-2 Reference Genome NC_045512.2	(6507)	ATAA-------TAGTTTAAAAATTACAGAAGAGGTTG-GCCACACAGATCT-AATGGCTGCTTATGTAGACAATTCTAG

Section 94

	(7348)	7348 7360 7370 7380 7390 7400 7410 7426
Oryza sativa chromosome 1 region 9.891-41.663nt	(7269)	GTAAAATTTTGGCAAAAATCAGGTCAGATTTGAAATACTATATATGTCAAAATTATTGCTGGAATTGATTTTTTTTATG
SARS-CoV-2 Reference Genome NC_045512.2	(6577)	TCTTACTATTAAGAAACCTAATGAATTATCTAGAGTATTAGGTTTG--AAAACCCTTGCTACTCATGGTTTAGCTGCTG

Section 95

	(7427)	7427 7440 7450 7460 7470 7480 7490 7505
Oryza sativa chromosome 1 region 9.891-41.663nt	(7348)	GCGATTGAGTTGTGTGTGTGTAAGAAATC-AATTATACTAGCCAACAATTGAGAAAAAAGAGAGTTGCATAGGTGGAAG
SARS-CoV-2 Reference Genome NC_045512.2	(6654)	TTAATAGTGTCCCTTGGGA-TACTATAGCTAATTATGCTAAGC--CTTTTCTTAACAAAG---------TTGTTAGTAC

Section 96

	(7506)	7506 7520 7530 7540 7550 7560 7570 7584
Oryza sativa chromosome 1 region 9.891-41.663nt	(7426)	AAGTTCTGGCTGCCTAGGAATATGGGGGCAACTATCTACTGGCTTGACACTCAATGACATAGAGTAATTTTTCTCTACC
SARS-CoV-2 Reference Genome NC_045512.2	(6721)	AACTACTAACA---TAGTTACACGGTG-----TTTAAACCGTGTTTGTACT-AATTATATGCCTTA---TTTCTTTACT

Section 97

	(7585)	7585 7590 7600 7610 7620 7630 7640 7650 7663
Oryza sativa chromosome 1 region 9.891-41.663nt	(7505)	CTTAAACCCTTGGAACAAAGGTTTTTTTTTATAAGAA-TACAACATTTGGGAAGAATTTCCGTGTACCGCACAAGGGTC
SARS-CoV-2 Reference Genome NC_045512.2	(6788)	-TTA-----TTGCTACAATTGTGTACTTTTACTAGAAGTACAAATTCTAGAATTAAAGCATCTATGCCG-ACTACTATA

Section 98

	(7664)	7664 7670 7680 7690 7700 7710 7720 7730 7742
Oryza sativa chromosome 1 region 9.891-41.663nt	(7583)	CATCTGAATGTTGGTATTGAATTCGCCCTATTG-----AAGGAATTCCTTTAGTCATGATTCAACTTCCATTGGGATTG
SARS-CoV-2 Reference Genome NC_045512.2	(6860)	GCAAAGAATACTGTTAAGAGTGTCGGTAAATTTTGTCTAGAGGCTTCATTTAATTAT--TTGAAGTCACCTAATTTTTC

Section 99

```
                                                   (7743) 7743   7750      7760      7770      7780      7790      7800      7810     7821
Oryza sativa chromosome 1 region 9.891-41.663nt    (7657) TAATCGATCTAAAAATTCCTATTTTTCCATAACCCTGACATTTTTTGGGGGGCTTTTTATCCCGGTTGAAGAAAATGGA
SARS-CoV-2 Reference Genome NC_045512.2            (6937) TAAACTGA-TAAATATTATAATTTGGTTTTTACTATTAAGTGTTTGCCTAGGTTCTTTAATCTACTC-----AACCGCT
```

Section 100

```
                                                   (7822) 7822    7830      7840      7850      7860      7870      7880      7890     7900
Oryza sativa chromosome 1 region 9.891-41.663nt    (7736) GTTGACAATGGGGAAAAAAAAACCTATGTATTTGTTGAAGAAGTCTTGCGTCCCCAAAAGGTTTGCCATTTTGCGGTA
SARS-CoV-2 Reference Genome NC_045512.2            (7010) GCTTTAGGTGTTTTAATGTCTAATTTAGGCATGCCTTCTTACTGTACTGGTTACAGAGAAGGCT----ATTT----GAA
```

Section 101

```
                                                   (7901) 7901     7910      7920      7930      7940      7950      7960               7979
Oryza sativa chromosome 1 region 9.891-41.663nt    (7815) GTTGATAAAGCCTACGAATCATTGCAA--AATTAGAATAAATAAATGCAAAGTTGTATTCCATTTACTCTCAATAATGA
SARS-CoV-2 Reference Genome NC_045512.2            (7081) CTCTACTAATGTCACTA----TTGCAACCTACTGTACTGGTTCTATACCTTGTAGTGTTTGTCTTAGTGGTTTAGATTC
```

Section 102

```
                                                   (7980) 7980      7990      8000      8010      8020      8030      8040               8058
Oryza sativa chromosome 1 region 9.891-41.663nt    (7892) TATTGAGAT-TTTAATAATTGAAAAATCGAAATCAACAATTAGTTAATCAT--AACCGATATGGACTTACACTTA--AT
SARS-CoV-2 Reference Genome NC_045512.2            (7156) TTTAGACACCTATCCTTCTTTAGAAA-CTATACAAATTACCATTTCATCTTTTAAATGGGATTTAACTGCTTTTGGCTT
```

Section 103

```
                                                   (8059) 8059       8070      8080      8090      8100      8110      8120            8137
Oryza sativa chromosome 1 region 9.891-41.663nt    (7966) AAATGTAGTGTTGTACATGG--TGTGGAATTTAAACTATATTTTTATGTGACAAAATAATTTCTAAATTCAGACCAATA
SARS-CoV-2 Reference Genome NC_045512.2            (7234) AGTTGCAGAGTGGTTTTTGGCATATATTCTTTTCACTAGGTTTTTCTATGTACTTGGATTGGCTGCAATCATGCAATTG
```

Section 104

```
                                                   (8138) 8138        8150      8160      8170      8180      8190      8200           8216
Oryza sativa chromosome 1 region 9.891-41.663nt    (8043) GATACAAGTTGTACTGGATTTGAAATCTCATTATCACTTTTTTCCAAATTTAAT-AGTACTAAGTTTTGGAACCAACTA
SARS-CoV-2 Reference Genome NC_045512.2            (7313) TTTTTCAGCTATTTTGCAGT--ACATTTTATTAGTAATTCTTGGCTTATGTGGTTAATAATTAATCTTGTA--CAAATG
```

Section 105

```
                                                   (8217) 8217         8230      8240      8250      8260      8270      8280          8295
Oryza sativa chromosome 1 region 9.891-41.663nt    (8121) GTAGGAAGTTACATGTCTCAACTTAGTATCTAACTACCATAGGGTTTTAAATAACATTCATTGATAC---AAATCAAAA
SARS-CoV-2 Reference Genome NC_045512.2            (7388) GCCCCGATTT-CAGCTATGG--TTAGAATGTACATCTTCTTTGCATCATTTTATTATGTATGGAAAAGTTATGTGCATG
```

Section 106

(8296) 8296 8310 8320 8330 8340 8350 8360 8374
Oryza sativa chromosome 1 region 9.891-41.663nt (8197) TCCTAAACCATATATTACAATTGAAGATAAATGATTAAGG--AAATATAATCATAGAAACAATAATATCAAACTACAAC
SARS-CoV-2 Reference Genome NC_045512.2 (7464) TTGTAGACGGT-TGTAATTCATCAACTTGTATGATGTGTTACAAACGTAAT-AGAGCAACAAGAGTCG-AATGTACAAC

Section 107

(8375) 8375 8380 8390 8400 8410 8420 8430 8440 8453
Oryza sativa chromosome 1 region 9.891-41.663nt (8274) TAACTTTG--GCCGATACGATGATTTAATATCATGAAAATTATATAATTAAATCATTTTTTTCATTTAGCTAT---AAA
SARS-CoV-2 Reference Genome NC_045512.2 (7540) TATTGTTAATGGTGTTAGAAGGTCCTTTTATGTCTATGCTAATGGAGGTAAAGGCTTTTGCAAACTACACAATTGGAAT

Section 108

(8454) 8454 8460 8470 8480 8490 8500 8510 8520 8532
Oryza sativa chromosome 1 region 9.891-41.663nt (8348) TAATTCAATACAGAGGAAAATTATAA-GTTTCATCAATTTTAATGGATCAAGTAGTACTAAAACTTATAGTGTAAATGA
SARS-CoV-2 Reference Genome NC_045512.2 (7619) TGTGTTAATTGTGATACATTCTGTGCTGGTAGTACATTTATTAGTGATGAAGTTGCGAGAGACTTGTCACTACAGTTTA

Section 109

(8533) 8533 8540 8550 8560 8570 8580 8590 8600 8611
Oryza sativa chromosome 1 region 9.891-41.663nt (8426) AAAAAAAACTGAAATTAGCTAGAATTTTTTTTTAAAAAAAACTTATTTTAGCCTATAGATGAAATTTTGATCCATTAAT
SARS-CoV-2 Reference Genome NC_045512.2 (7698) AAAGACCAATAAATCCTACTGACCAGTCTTCTTACATCGTTGATAGTGT----TACAG-TGAAGAATGGTTCCATCCAT

Section 110

(8612) 8612 8620 8630 8640 8650 8660 8670 8680 8690
Oryza sativa chromosome 1 region 9.891-41.663nt (8505) TCTGAATGGGTATCCGTTTCCATTCATATTCCTATGGTTTCAATATTACGTGAGTGGTCCATAAGCATGATTAGGCTAG
SARS-CoV-2 Reference Genome NC_045512.2 (7772) CTTTACTTTGATAAAGCTGG--TCAAAAGACTTATGAAAGACATTCTCTCTCTCATTTTGTTAAC-----TTAGACAAC

Section 111

(8691) 8691 8700 8710 8720 8730 8740 8750 8769
Oryza sativa chromosome 1 region 9.891-41.663nt (8584) ACCAGAAATGAATAGTACTGAATAAGCATTGCTCATCATCTTCCCTAGTCGAAGGCGAGAAAAAAAAAAGAGAAGAGAA
SARS-CoV-2 Reference Genome NC_045512.2 (7844) CTGAGAGCT-AATAACACTAAAGGTTCATTGCCTATTAATGTTA-TAGTTTTTGATGGTAAATCAAAATGTGAAGA-AT

Section 112

(8770) 8770 8780 8790 8800 8810 8820 8830 8848
Oryza sativa chromosome 1 region 9.891-41.663nt (8663) GATGGGCG---TCAGTGCTCCGATGACTAAACGGCGGTGAGGAGAGTGGTAGGACGTGTCGCACAACATGAATCATCCA
SARS-CoV-2 Reference Genome NC_045512.2 (7920) CATCTGCAAAATCAGCG-TCTGTTTACTACAGTCAGCTTATGTGTCAACCTATACTGTTACTAGATCAGGCATTAGTGT

Section 113

	(8849)	8849 8860 8870 8880 8890 8900 8910 8927
Oryza sativa chromosome 1 region 9.891-41.663nt	(8739)	TAGATGGTGGAGGTTTCATCCATAGATGGTGGAGGTTGATAATGAAGAAAACCATAATTGTAGGAACACGGCCGGCAAC
SARS-CoV-2 Reference Genome NC_045512.2	(7998)	CTGATGTTGGTG----------ATAGT--GCGGAAGTTGCAGTTAAAATGTTTGATGCTTACGTTAATACGTTTT-CATC

Section 114

	(8928)	8928 8940 8950 8960 8970 8980 8990 9006
Oryza sativa chromosome 1 region 9.891-41.663nt	(8818)	GACGTG----GTCCCAATAGAGAGAAATAATAACAACG-----AACT-CTGTTGGTGAACACG-ATCGGATGAGTGCAT
SARS-CoV-2 Reference Genome NC_045512.2	(8065)	AACTTTTAACGTACCAATGGAAA-AACTCAAAACACTAGTTGCAACTGCAGAAGCTGAACTTGCAAAGAATGTGTCCTT

Section 115

	(9007)	9007 9020 9030 9040 9050 9060 9070 9085
Oryza sativa chromosome 1 region 9.891-41.663nt	(8886)	-GAAAATGTGTT-----CCTGGATAAAAGGGTTTCAG------TTTCTTGAACGCGTAGGAGGAATGAAAGACGGAGAA
SARS-CoV-2 Reference Genome NC_045512.2	(8143)	AGACAATGTCTTATCTACTTTTATTTCAGCAGCTCGGCAAGGGTTTGTTGATTCAGATGTAGAAACTAAAGATGTTGTT

Section 116

	(9086)	9086 9100 9110 9120 9130 9140 9150 9164
Oryza sativa chromosome 1 region 9.891-41.663nt	(8953)	AAAATGACTAACACTGTT-CATCG-TCTTCGCTAGCCGAAACGAGAAATATAATTGGAGGAAAGATCGGCGTCGGTGCT
SARS-CoV-2 Reference Genome NC_045512.2	(8222)	GAA-TGTCTTAAATTGTCACATCAATCTGACATAGAAGTTACTGGCGATAG--TTGTAATAACTATATGCTCACCTATA

Section 117

	(9165)	9165 9170 9180 9190 9200 9210 9220 9230 9243
Oryza sativa chromosome 1 region 9.891-41.663nt	(9030)	TC---GGCGACCAAACGACGACGAGGGAGGTGGCAGGACGCGTCCCACGAGGTGGCTTGTCCATAGAAGGTGGCAGTTG
SARS-CoV-2 Reference Genome NC_045512.2	(8298)	ACAAAGTTGAAAACATGACACCCCGTGACCTTGGTGCTTGTATTGACTGTAGTG-CGCGTC-ATATTAATGCGCAGGTA

Section 118

	(9244)	9244 9250 9260 9270 9280 9290 9300 9310 9322
Oryza sativa chromosome 1 region 9.891-41.663nt	(9106)	GCAATGAAAAAACAAAAAACAGTGGAAACGCA--GTCAGCAATGACATGGTCCCGATGGAGATAGAAACAACAACAATT
SARS-CoV-2 Reference Genome NC_045512.2	(8375)	GCAAA-AAGTCACAACATTGCTTTGATATGGAACGTTAAAGATTTCATGTCATTGTCTGA-ACAACTACGAAAACAAAT

Section 119

	(9323)	9323 9330 9340 9350 9360 9370 9380 9390 9401
Oryza sativa chromosome 1 region 9.891-41.663nt	(9183)	AGCTC-TGGTGGTGAACACGACCAGGTGAGTGT---GTCAAAATGCGT---TTCCGGACAAGGGGTTATGGTTTCCTTA
SARS-CoV-2 Reference Genome NC_045512.2	(8452)	ACGTAGTGCTGCTAAAAAGAATAACTTACCTTTTAAGTTGACATGTGCAACTACTAGACAAGTTGTTAATGTTGTAACA

Section 120

		9402 … 9480
	(9402)	
Oryza sativa chromosome 1 region 9.891-41.663nt	(9255)	AACGCGCTGGAGAAACGAAAGACGGAAAAAAGAGAATGCATCATATAGGAGGGAAAGAGGGACGGGGAGGAGAGGGTTG
SARS-CoV-2 Reference Genome NC_045512.2	(8531)	ACAAAGAT--AGCACTTAAGGGTGGTAAAA------TTGTTAATAATTGGTTGAAGCAGTTA-----ATTAAAGT-TAC

Section 121

		9481 … 9559
	(9481)	
Oryza sativa chromosome 1 region 9.891-41.663nt	(9334)	CCATGAAAACTTTCTACTCCCTCCATCAATGTGACAATGAATCTAGCAGCTAGATTCATTTAGGATTTATGTGTCAAAT
SARS-CoV-2 Reference Genome NC_045512.2	(8596)	ACTTGTGTTCCTTTTTGTTGCTGCT---ATTTTCTATTTAAT--AACACCTG--TTCATGTCA--------TGTCTAAA

Section 122

		9560 … 9638
	(9560)	
Oryza sativa chromosome 1 region 9.891-41.663nt	(9413)	CCAACCAAAATCTC---TTATATTTTAGGACGGAGGGAGTATATAGAAGTTACTTTAAAATATCAAATAT-ATCTCTTT
SARS-CoV-2 Reference Genome NC_045512.2	(8660)	CATACTGACTTTTCAAGTGAAATCATAGGATACAAGGC---TATTGATGGTGGTGTCACTCGTGACATAGCATCTACAG

Section 123

		9639 … 9717
	(9639)	
Oryza sativa chromosome 1 region 9.891-41.663nt	(9488)	GTCAAGTTTGTAATAACTAAAA--CTTAATTAATCATATGTTATTGGTTTTCTCGTTTTACGTGCACAAACT--TAATC
SARS-CoV-2 Reference Genome NC_045512.2	(8736)	ATACTTGTTTTGCTAACAAACATGCTGATTTTGACACATGGTTTAGCCAGCGTGGTGGTAGTTATACTAATGACAAAGC

Section 124

		9718 … 9796
	(9718)	
Oryza sativa chromosome 1 region 9.891-41.663nt	(9563)	TTCACCATCATCAATTTTAAAGTTTAAACACCATGTTTAGGGTTT--AGGGATAAGATT--CAAAATCAATATCCAGCA
SARS-CoV-2 Reference Genome NC_045512.2	(8815)	TTGCCCAT--TGATTGCTGCAGTCATAACAAGAGAAGTGGGTTTTGTCGTGCCTGGTTTGCCTGGCACGATATTACGCA

Section 125

		9797 … 9875
	(9797)	
Oryza sativa chromosome 1 region 9.891-41.663nt	(9638)	-AGCTATATATAGCAGAGTTGGACT-CTTATTTTAA--TCTGAG-GCA--TGATAATATTT-TTATACAGCGTAAATGG
SARS-CoV-2 Reference Genome NC_045512.2	(8892)	CAACTA-ATGGTGACTTTTTGCATTTCTTACCTAGAGTTTTTAGTGCAGTTGGTAACATCTGTTACACACCATCAAAA-

Section 126

		9876 … 9954
	(9876)	
Oryza sativa chromosome 1 region 9.891-41.663nt	(9709)	ATGGCCATATTCATTTATCCGA--TTGATATCTAATTTTATCCCTCACAAGTCACAGGGAGGAGTCGAAAAGAGTCAAT
SARS-CoV-2 Reference Genome NC_045512.2	(8969)	-----CTTATAGAGTACACTGACTTTGCAACATCAGCTTGTGTTTTGGCTGCTGAATGTACAATTTTTAAAGATGCTT-

Section 127

```
                                                   9955 9960      9970      9980      9990      10000     10010     10020        10033
                                           (9955)
Oryza sativa chromosome 1 region 9.891-41.663nt   (9786) ACCAGCCGCGCCACCGCTCCCTCGCTGACCTGCCCGTGTTC-----GCCCCTCTCGCCGGCCGCCGACCCGCCCCTGCT
SARS-CoV-2 Reference Genome NC_045512.2           (9042) --CTGGTAAGCCAGTACCATATTGTTATGATACCAATGTACTAGAAGGTTCTGTTGCTTATGAAAGTTTACGCCCTGA-
```

Section 128

```
                                                   10034 10040     10050     10060     10070     10080     10090     10100       10112
                                           (10034)
Oryza sativa chromosome 1 region 9.891-41.663nt   (9860) GCTCACCGCTCCAGCCTCCGCTCATCCCAAGCCTTCCACCACAACCCCCCACCCCTGCTCGCCCGCGCACCCAGCTCAC
SARS-CoV-2 Reference Genome NC_045512.2           (9118) -CACACGTTATGTGCTCATGGATGGCTCTATTATTCAATTTCCTAACACCTACCTTGAAGGTTCTGTTAGAGTGGTAAC
```

Section 129

```
                                                   10113 10120     10130     10140     10150     10160     10170     10180       10191
                                           (10113)
Oryza sativa chromosome 1 region 9.891-41.663nt   (9939) CAGCGCACCCGTCTGGGGTCTGCCTGC-CGCTTCTCCCTCCCGTGCAGGTGCAGGGGAA---GAGGCAAGGAGGCAAG
SARS-CoV-2 Reference Genome NC_045512.2           (9196) AA-CTTTTGATTCTGAGTACTGTAGGCACGGCACTTGTGAAAGATCAGAAGCTGGTGTTTGTGTATCTACTAGTGGTAG
```

Section 130

```
                                                   10192 10200     10210     10220     10230     10240     10250     10260     10270
                                           (10192)
Oryza sativa chromosome 1 region 9.891-41.663nt  (10014) ACCGGGGCG---CAGTGGGGAGTACGGAGGA--AGCTGGTGCAGCGTGGGAGAGAGGGAGCTGGAAAAGAAGGAAGAAA
SARS-CoV-2 Reference Genome NC_045512.2           (9274) ATGGGTACTTAACAATGATTATTACAGATCTTTACCAGGAGTTTTCTGTG-GTGTAGATGCTGTAAATTTACTTACTAA
```

Section 131

```
                                                   10271 10280     10290     10300     10310     10320     10330               10349
                                           (10271)
Oryza sativa chromosome 1 region 9.891-41.663nt  (10088) G--GGGGGCAGTACAAGGGGGAC---TTGGGAGGAGGAAGAAGGGGGAATATTTTGAGTATATGATC--AATTTCGCCG
SARS-CoV-2 Reference Genome NC_045512.2           (9352) TATGTTTACACCACTAATTCAACCTATTGGTGCTTTGGACATATCAGCATCTAT--AGTAGCTGGTGGTATTGTAGCTA
```

Section 132

```
                                                   10350 10360     10370     10380     10390     10400     10410               10428
                                           (10350)
Oryza sativa chromosome 1 region 9.891-41.663nt  (10160) TCCAATCCAGATGTGC-GCCAACCAATTCGCTATGGT-ATAATTGATAATAATGA-TACCTTCCTT--GTTGTATCTAT
SARS-CoV-2 Reference Genome NC_045512.2           (9429) TCGTAGTAACATGCCTTGCCTACTATTTTATGAGGTTTAGAAGAGCTTTTGGTGAATACAGTCATGTAGTTGCCTTTAA
```

Section 133

```
                                                   10429 10440     10450     10460     10470     10480     10490               10507
                                           (10429)
Oryza sativa chromosome 1 region 9.891-41.663nt  (10234) CTATCTATCTATCTATCTATCTATCTATCTA-TCTATCTA-------TCTA-TCTATCTATC--GATTTCTTATCT-TT
SARS-CoV-2 Reference Genome NC_045512.2           (9508) TACTTTACTATTCCTTATGTCATTCACTGTACTCTGTTTAACACCAGTTTACTCATTCTTACCTGGTGTTTATTCTGTT
```

```
                                                            Section 134
                                           (10508) 10508       10520     10530     10540     10550     10560     10570          10586
Oryza sativa chromosome 1 region 9.891-41.663nt (10301) CTTTGCTCATGCCTGACGATGTCATGTCACAGCTGCTATGCTATAGCAATCTCCTTACTTCAGGATTATCATGTATGTA
   SARS-CoV-2 Reference Genome NC_045512.2  (9587) ATTTACTTGTACTTGAC-ATTTTATCTTACTAATGATGTTTCTTTTTTAGCACATATTCAGTGGATGGTTATGTTC--A

                                                            Section 135
                                           (10587) 10587        10600     10610     10620     10630     10640     10650          10665
Oryza sativa chromosome 1 region 9.891-41.663nt (10380) AATCACTAGTAAATGTATTACGAGAATATATACAATTAAGCAT-GTATCACATCGATGTAATTGGCTTAGTACAATAAA
   SARS-CoV-2 Reference Genome NC_045512.2  (9663) CACCTTTAGTACCTTTCTG--GATAACAATTGCTTATATCATTTGTATTTCCACAAAGCATTTCTATTGGTTCTTTAG-

                                                            Section 136
                                           (10666) 10666          10680     10690     10700     10710     10720     10730        10744
Oryza sativa chromosome 1 region 9.891-41.663nt (10458) GCTTTCATTATCTAAAAAAATTACAGTTCTTGTGTTGCTCTGTGCGAACGAAACTTTAAACTGGAACAG-GTTGAGGAG
   SARS-CoV-2 Reference Genome NC_045512.2  (9739) ----TAATTACCTAAAGAGACGTGTAGTCTT-TAATGGTGTTTCCTTTAGTACTTTTGAA--GAAGCTGCGCTGTGCAC

                                                            Section 137
                                           (10745) 10745 10750     10760     10770     10780     10790     10800     10810        10823
Oryza sativa chromosome 1 region 9.891-41.663nt (10536) CTGGCTTCACTCATCAAGGACAACCTCTACAGCAAGCACCTCGTCCTCTCTACTGAGGAAACCCTCGTCGGGATCCTAC
   SARS-CoV-2 Reference Genome NC_045512.2  (9811) CTTT-TTGTTAAATAAAGAAATGTATCTAAAGTTG---CGTAGTGATGTGCTATTA------CCTCTTACGCA----AT

                                                            Section 138
                                           (10824) 10824 10830     10840     10850     10860     10870     10880     10890        10902
Oryza sativa chromosome 1 region 9.891-41.663nt (10615) AGAACCAGTACCACAACTCTGACGATGACGAGGATGAGGACGATATAGTGGCA--GCATACCGCGGGACAAATAGGAAT
   SARS-CoV-2 Reference Genome NC_045512.2  (9876) ATAATAGATACTT-AGCTCTTT--ATAATAAGTACAAGTATTTTAGTGGAGCAATGGATACAACTAGCTACAGAG-AAG

                                                            Section 139
                                           (10903) 10903 10910     10920     10930     10940     10950     10960     10970        10981
Oryza sativa chromosome 1 region 9.891-41.663nt (10692) ATCCTAGAGCTCCAACCTGCCAGCTCCTATCAACGTCTTCTTTTGCATCGCCTTGCCGACATCTACGGGTGCTCTTT-C
   SARS-CoV-2 Reference Genome NC_045512.2  (9951) CTGCTTGTTGTC--ATCTCGCAAAGGCTCTCAATGACTTCAGTAACT------------CAGGTTCTGATGTTCTTTAC

                                                            Section 140
                                           (10982) 10982 10990     11000     11010     11020     11030     11040     11050  11060
Oryza sativa chromosome 1 region 9.891-41.663nt (10770) CAATTCCATTCGACCTGCTTCACCTACCCTCTTCTCAAATACTAACTTAACTGGATAGAGTAATATTTGCAACTACTAA
   SARS-CoV-2 Reference Genome NC_045512.2 (10016) CAACCACCACAAACCTCTATCACCTCAGCTGTTTTGCAG-----------AGTGGTTTTAGAAAATG-GCA--TTCCCA
```

Section 141

	(11061)	11061 … 11139
Oryza sativa chromosome 1 region 9.891-41.663nt	(10849)	TGTCTTGCTTTTGTGTCGCTGCTAGGTTTGTTCATGAATCTGTTGGTGAAGGTGAAGATCGACACTTAGTTCTCCAGCG
SARS-CoV-2 Reference Genome NC_045512.2	(10082)	TCTGGTAAAGTTGAG-----GGTTGTATGGTACAAGTAACTTGTGGT--------ACAACTACACTTAACGGTCTTTGG

Section 142

	(11140)	11140 … 11218
Oryza sativa chromosome 1 region 9.891-41.663nt	(10928)	CTGCCCTGAAACTGCCATGTATGATACCAAAAGTGACCACACATCTCTTTGTTATTGAGTGTCGTCTTATGTCTCCCTA
SARS-CoV-2 Reference Genome NC_045512.2	(10148)	CT----TGA---TGAC--GTAGTTTAC------TGTCCA---AGACATGTGATCTGCA----CCTCTGAAGACATGCTT

Section 143

	(11219)	11219 … 11297
Oryza sativa chromosome 1 region 9.891-41.663nt	(11007)	TGCCCTCATTTCGCTGTTTCTTATACTACAAACATGTACAGTTTAATTTCCACCTGCATCATTCTTCAATGCAATAGAA
SARS-CoV-2 Reference Genome NC_045512.2	(10205)	AACCCTAATTATGAAGATT-TACTCATTCGTAAGTCTA-ATCATAATTTCTTGGTACAGGCTGGT--AATG---TTCAA

Section 144

	(11298)	11298 … 11376
Oryza sativa chromosome 1 region 9.891-41.663nt	(11086)	AAAAGGGTTAATAGAAAAACACATTGTTTCCACACAAAAAACTGT-CTTCAGGGAAATAAAACAAAAGCTATCCCGTAA
SARS-CoV-2 Reference Genome NC_045512.2	(10277)	CTCAGGGTTATTGGACA----------TTCTATGCAAAATTGTGTACTTAAGCTTAAGGTTGATACAGCCAATCCTAAG

Section 145

	(11377)	11377 … 11455
Oryza sativa chromosome 1 region 9.891-41.663nt	(11164)	ACTCATGATCACATCTTGTTTGTTATTCCTTGCCGTCTTGCTCCAATTGCATCCCTAG-TTCCACCTGTGGCGAACCAT
SARS-CoV-2 Reference Genome NC_045512.2	(10346)	ACACCTAAGTATAAGTTTGTTCGCATTCAACCAGGACAGACTT---TTTCAGTGTTAGCTTGTTACAATGGTTCACCAT

Section 146

	(11456)	11456 … 11534
Oryza sativa chromosome 1 region 9.891-41.663nt	(11242)	TTGGCTGCTCACACTTTAAGATAATCACGCCATTTGCCTCCCATAGCTATTGTGTTGACTCAGAGCACGGAAGCTGAAA
SARS-CoV-2 Reference Genome NC_045512.2	(10422)	CTGGTGTTTACCAATGTGCTATGAGGCC-CAATTTCACTATTAAGGGTCATTCCTTAATG-GTTCATGTG----GTAG

Section 147

	(11535)	11535 … 11613
Oryza sativa chromosome 1 region 9.891-41.663nt	(11321)	TCACACTTTTCTTGTGACCTACACATGAGACATTATTCTGTAGTTCTATTACCTCTGATTACTTATT--ATTTCATATT
SARS-CoV-2 Reference Genome NC_045512.2	(10495)	TGTTGGTTTTAACATAGATTATGACTGTGTCTCTTTTTGTTACATGCACCATATGGAATTACCAACTGGAGTTCATGCT

```
                                                                                            Section 148
                                                          (11614) 11614 11620     11630     11640     11650     11660     11670     11680      11692
Oryza sativa chromosome 1 region 9.891-41.663nt  (11398) GCGCAGCCCACCTGTCCTCGTAAGCGACGTATTATGGGAGTAT-GACAACAAAGATACATCGACATCTGTTGTAGTGAA
SARS-CoV-2 Reference Genome NC_045512.2 (10574) G-GCA------CAGACTTAGAAGGTAACTT-TTATGGACCTTTTGTTGACAGGCAAACAGCACAAGCAGCTGGTACGGA
                                                                                            Section 149
                                                          (11693) 11693  11700     11710     11720     11730     11740     11750     11760      11771
Oryza sativa chromosome 1 region 9.891-41.663nt  (11476) GAGGAAAGATACAGGTGAATTGTTATAATTTCATTTTGTGGATCATTCATGATGAAGCATTTTGATGGGGTGACACTAC
SARS-CoV-2 Reference Genome NC_045512.2 (10645) CACAACTATTACAGTTAA--TGTTTTAGCTTGGTTGTACGCTGCTGTTATAA--ATGGAGACAGGTGGTTTCTCAAT-C
                                                                                            Section 150
                                                          (11772) 11772   11780     11790     11800     11810     11820     11830     11840   11850
Oryza sativa chromosome 1 region 9.891-41.663nt  (11555) CCTGTAGCA---CCCATGCTGCCAATTCTGTTTGTGCTTT--AGTA-AATGGTCCATGGAGAAGATTCGTGTGTACTTA
SARS-CoV-2 Reference Genome NC_045512.2 (10719) GATTTACCACAACTCTTAATGACTTTAACCTTGTGGCTATGAAGTACAATTATGAACCTCTAACACAAGACCATGTTGA
                                                                                            Section 151
                                                          (11851) 11851    11860     11870     11880     11890     11900     11910           11929
Oryza sativa chromosome 1 region 9.891-41.663nt  (11628) TGATCCGTTGTTCGGCTTGTCCGGGCTCAAGAAAATTTCTGGATCCTCCTCTGTTTTGTTCCCCTACATAACCACTGTA
SARS-CoV-2 Reference Genome NC_045512.2 (10798) C-ATAC-TAGGACCTCTT-TCTGCTCAAACTGGAATTGCCGTTTTAGA-TATGTGTGCTTCATTAAAAGAATTACTGCA
                                                                                            Section 152
                                                          (11930) 11930     11940     11950     11960     11970     11980     11990           12008
Oryza sativa chromosome 1 region 9.891-41.663nt  (11707) TTACCTGACCAATATATTTTTTTTTCTGAAAACAATCATAAT-GCCCTTTACTCATAACCCTAAGATACCTTCCCTAAT
SARS-CoV-2 Reference Genome NC_045512.2 (10873) AAATGGTATGAATGGACGTACCATATTGGGTAGTGCTTTATTAGAAGATGAATTTACACCTTTTGATGTTGTTAGACAA
                                                                                            Section 153
                                                          (12009) 12009      12020     12030     12040     12050     12060     12070           12087
Oryza sativa chromosome 1 region 9.891-41.663nt  (11785) TAATGAAATGGG--T--CCATAGCGGAT---CAAGAAAAACTAAAAAAAAGTATGGGAGGATAAAATGAAGAATCAGGG
SARS-CoV-2 Reference Genome NC_045512.2 (10952) TGCTCAGGTGTTACTTTCCAAAGTGCAGTGAAAAGAACAATCAAGGGTACACACCACTGGTTGTTACTCACAATTTTGA
                                                                                            Section 154
                                                          (12088) 12088       12100     12110     12120     12130     12140     12150           12166
Oryza sativa chromosome 1 region 9.891-41.663nt  (11857) CA-CACTAGAAATTCTCTTCGCAAAAAAATTATGAAATGATTCCTCTTGATGGTAGTGACCAAATCTGGAAATAGGAA-
SARS-CoV-2 Reference Genome NC_045512.2 (11031) CTTCACTTTTAGTTTTAGTCCAGAGTACTCAATGGTCTTTGTTCTTTTTTTTGTA-TGAAAATGCCTTTTACCTTTTG
```

Section 155

	(12167)	12167 12180 12190 12200 12210 12220 12230 12245
Oryza sativa chromosome 1 region 9.891-41.663nt	(11934)	CTAGGAAGTCATTGTGCAGTGCCTTAGCCTGAATTTATTTCACCCTCTCATAAAAAGGGATTAAAAATGCACAGTATGG
SARS-CoV-2 Reference Genome NC_045512.2	(11109)	CTATGG-GTATTATTGCTATGTCTGCTTTTGCAATGATGTTTGTCAAACATAAGCATGCATTT------CTCTGTTTGT

Section 156

	(12246)	12246 12260 12270 12280 12290 12300 12310 12324
Oryza sativa chromosome 1 region 9.891-41.663nt	(12013)	TCGTTGAGCATGATTCGCGTGACAAAGGAG------TAAAGCTGGCTGCTGGATGCCAGCTGGAGAAATACTGGTATTT
SARS-CoV-2 Reference Genome NC_045512.2	(11181)	TTTTGTTACCT---TCTCTTGCCACTGTAGCTTATTTTAATATGGT--CTATATGCCTGCTAGTTGGGTGATGCG-TAT

Section 157

	(12325)	12325 12330 12340 12350 12360 12370 12380 12390 12403
Oryza sativa chromosome 1 region 9.891-41.663nt	(12086)	TTTTCCATGTGAGTTTGAAGTGGGTAGTCAATTTGACTTACATTTTCATCGTTCCACAGGCATAGTTTGATGTGTCATA
SARS-CoV-2 Reference Genome NC_045512.2	(11254)	TATGACATGGTTGGATATGGTTGATACT-AGTTTGTCTGG--TTTTAAGCTA---AAAGAC---------TGTGTTATG

Section 158

	(12404)	12404 12410 12420 12430 12440 12450 12460 12470 12482
Oryza sativa chromosome 1 region 9.891-41.663nt	(12165)	AGACATTCAGTTCCATAGATATCTTCTTGTTATTCCCTTAAAAAAGATATTTCTTATTATTCTCTCAAGAAATTCTAAG
SARS-CoV-2 Reference Genome NC_045512.2	(11318)	TATGCATCAGCTGTAGTGTTA-CTAATCCTTATGACAGCAAGAAC--TGTGTATGATGATGGTGCTAGGAGAGT----G

Section 159

	(12483)	12483 12490 12500 12510 12520 12530 12540 12550 12561
Oryza sativa chromosome 1 region 9.891-41.663nt	(12244)	TGCAC-CATCTGCACAAAGAAAAAC-CTATTCTACA-TGTATAAATGTGATGCACT-GTGCAATCTTATGTGTGTGTAA
SARS-CoV-2 Reference Genome NC_045512.2	(11390)	TGGACACTTATGAATGTCTTGACACTCGTTTATAAAGTTTATTATGGTAATGCTTTAGATCAAGCC-ATTTCCATGT--

Section 160

	(12562)	12562 12570 12580 12590 12600 12610 12620 12630 12640
Oryza sativa chromosome 1 region 9.891-41.663nt	(12319)	CAGGTTAATAATTAGCATTTTGAATTCTCTTCTAACAGATCTTGAAGAAGCCTGGAAGGAAGATGCCCAAGAAAATATT
SARS-CoV-2 Reference Genome NC_045512.2	(11466)	--GGCTCTTATAATCTCTGTTACTTCTAACTACTCAGGTGTAGTTACAAC-TGTCATGTTTTTGGCCAGAGGTATTGT

Section 161

	(12641)	12641 12650 12660 12670 12680 12690 12700 12719
Oryza sativa chromosome 1 region 9.891-41.663nt	(12398)	TCTGCTGAAATCTCGCATTTGAAAAATGATGCAGGTACGTTGCGCTTGCTG-TCCACCTC--TCAACAAAGCGTCTTTC
SARS-CoV-2 Reference Genome NC_045512.2	(11542)	TTTTATG---TGTG----TTGAGTATTGCCCTATTTTCTTCATAACTGGTAATACACTTCAGTGTATAATGCTAGTTT-

Section 162

```
                                             (12720) 12720     12730     12740     12750     12760     12770     12780          12798
 Oryza sativa chromosome 1 region 9.891-41.663nt (12474) AAACTGTCTTAGGGTGTGTTTGGAACTCCAAGTTCCCAACTCCATTGCTTTGTTTTCGTACGCTT-TTCAAACTGTTAA
SARS-CoV-2 Reference Genome NC_045512.2 (11613) ATTGTTTCTTAGGCTATTTTTGTACTT----GTTACTTTGGCCTCTTTTGTTTACTCAACCGCTACTTTAGACTG--AC
```

Section 163

```
                                             (12799) 12799      12810     12820     12830     12840     12850     12860          12877
 Oryza sativa chromosome 1 region 9.891-41.663nt (12552) ACGTTGCGTTTTTTTGCAAAAAGTTTCTATACAA-AGTT--GCTTTTAAAAATCATATTGATCCAATTTTGAAAAAAAT
SARS-CoV-2 Reference Genome NC_045512.2 (11686) TCTTGGTGTTTATGATTACTTAGTTTCTACACAGGAGTTTAGATATATGAATTCACAGGGACTACTCCCACCCAAGAAT
```

Section 164

```
                                             (12878) 12878       12890     12900     12910     12920     12930     12940          12956
 Oryza sativa chromosome 1 region 9.891-41.663nt (12628) AGCAAATACTTAATTAATCATGTACTAATGGACCGCTCCGTTTTCCGTGCCAACTGTTTGGGATGGGAACCGGCATATG
SARS-CoV-2 Reference Genome NC_045512.2 (11765) AGCATAGATGCCTTCAAAC-TCAAC-ATTAAATTGTTGGGTGTTGGTGGCAAACCTTGTATCAAAGTAGCCACTGTA--
```

Section 165

```
                                             (12957) 12957        12970     12980     12990     13000     13010     13020          13035
 Oryza sativa chromosome 1 region 9.891-41.663nt (12707) CGAACGCAGCCTTAAGCTTGGTTCTCTGCATAATTTCAGTCGAGGAATTTAAGTCT-GAAAAAAAACCCAATCAACTCT
SARS-CoV-2 Reference Genome NC_045512.2 (11840) CAGTCTAAAATGTCAGAT--GTAAAGTGCACATCAGTAGTCTT--ACTCTCAGTTTTGCAACAACTCAGAGTAGAATCA
```

Section 166

```
                                             (13036) 13036         13050     13060     13070     13080     13090     13100          13114
 Oryza sativa chromosome 1 region 9.891-41.663nt (12785) TTTATTTCAGATTTGAAGGCTTTGCAAAAGTC-AGTTGCACCCCCAGCACCGTCACTCAAGGAGAGGGAAGCTGCTTAT
SARS-CoV-2 Reference Genome NC_045512.2 (11915) TCA---TCTAAATTGTGGGCTCA--ATGTGTCCAGTTACACA---ATGACATTCTCTTAGCTA-----AAGATACTACT
```

Section 167

```
                                             (13115) 13115 13120     13130     13140     13150     13160     13170     13180          13193
 Oryza sativa chromosome 1 region 9.891-41.663nt (12863) CGAGCTGCTCGCGAGCGTATCTTCTCAGCGCATGATGCCAAGGGA--AATGG-GACAGCAGT-GGCAAAACCTAGGCAT
SARS-CoV-2 Reference Genome NC_045512.2 (11981) GAAGCCTTT---GAAAAAATGGTTTCACTACTTTCTGTTTTGCTTTCCATGCAGGGTGCTGTAGACATAAACAAGCTTT
```

Section 168

```
                                             (13194) 13194  13200     13210     13220     13230     13240     13250     13260          13272
 Oryza sativa chromosome 1 region 9.891-41.663nt (12938) GTTCCTGC-TGTTGCTCAAC-GGATGATCGCGCATGCACTTGGCAAAAAAGTTGAGAGCCCGACAGAGACA-GCAGCCG
SARS-CoV-2 Reference Genome NC_045512.2 (12057) GTGAAGAAATGCTGGACAACAGGGCAACCTTACAAGCTATAGCCTCAGA-GTTTAGTTCCCTTCCATCATATGCAGCTT
```

Section 169

		13273 … 13351
	(13273)	
Oryza sativa chromosome 1 region 9.891-41.663nt	(13014)	TGAAGAATGGCAAAGGA---AAGGAGCCAGCGGAAAGTAGTAGGAAC----AAGTTAAACCCACGCACA-GCAGGTGGC
SARS-CoV-2 Reference Genome NC_045512.2	(12135)	TTGCTACTGCTCAAGAAGCTTATGAGCAGGCTGTTGCTAATGGTGATTCTGAAGTTGTTCTTAAAAAGTTGAAGAAGTC

Section 170

		13352 … 13430
	(13352)	
Oryza sativa chromosome 1 region 9.891-41.663nt	(13085)	AAGGAAGATAGCAGGTATGTTGAGAATGGCAGAATGAGGTTGCACACCGGGAATCCATGTAAGCAGAGCTGGC-GTACA
SARS-CoV-2 Reference Genome NC_045512.2	(12214)	TTTGAATGTGGCTA--AATCTGAATTTGACCG--TGA---TGCAGCCATGCAACGTAAGTTGGAAAAGATGGCTGATCA

Section 171

		13431 … 13509
	(13431)	
Oryza sativa chromosome 1 region 9.891-41.663nt	(13163)	AG-TAACAGTAGAGCTGCTAGTAGTGTTAGTCCTGATGAACTGAAGAGGGAGCAGGTTGGAGCGGCAA---AGAGGATG
SARS-CoV-2 Reference Genome NC_045512.2	(12286)	AGCTATGACCCAAATGTATAAACAGGCTAGATCTGAGGAC---AAGAGGGCAAAAGTTACTAGTGCTATGCAGACAATG

Section 172

		13510 … 13588
	(13510)	
Oryza sativa chromosome 1 region 9.891-41.663nt	(13238)	TTTGTGCATGCCCTGCGGCTGCCTGGTGTTGAAGGAAGTGATGGTCCGGTGCGGAAAGGCAAGTAAGAGTCGAGTTGAG
SARS-CoV-2 Reference Genome NC_045512.2	(12362)	CTTTTCACTATGCTTAGAAAGTTGGATAATGATGCAC-TCAACAACATTATCAACAATGCAAGAGATGGTTGTGTTCCC

Section 173

		13589 … 13667
	(13589)	
Oryza sativa chromosome 1 region 9.891-41.663nt	(13317)	ATGGAGGTGTAATGTAGGTATTAGGAT-GGAGTGAAATGGCAACTCTGTGATGATTTTCATCTGGTGATACTATGCTG
SARS-CoV-2 Reference Genome NC_045512.2	(12440)	TTGAACA---TAAT--ACCTCTTACAACAGCAGCCAAAC----------TAATGGTTGTCAT--------ACCAGACT-

Section 174

		13668 … 13746
	(13668)	
Oryza sativa chromosome 1 region 9.891-41.663nt	(13395)	GTATAAGATGAGATGGAAGGCCTGGTATCCCTTGGTGGAAGAAGTTGAAGCGCAAAATGCAATGCTGGGGATATGCAAG
SARS-CoV-2 Reference Genome NC_045512.2	(12495)	--ATAACAC-ATATAAAAATACGTGTGA----TGGTACAACATTTACTTATGCATCA-GCATTGT--GGGAAATCCAAC

Section 175

		13747 … 13825
	(13747)	
Oryza sativa chromosome 1 region 9.891-41.663nt	(13474)	TGGAAGAAGTTGAAGCGCAAAATGCAATGCTGGGGATA-TGCAAGTGGAAGTGGTGAGAGCTAGCTGCTTGCCGATGG
SARS-CoV-2 Reference Genome NC_045512.2	(12564)	AGGTTGTAGATGCAG---ATAGTAAAATTGTTCAACTTAGTGAAATTAGTA-TGGACAATTC-ACCTAATTTAGCATGG

Section 176

```
                                              (13826) 13826         13840     13850     13860     13870     13880     13890        13904
Oryza sativa chromosome 1 region 9.891-41.663nt (13552) CAGCAG-TTGTGGCAATATTGAGTGCAATTGACAGGTCTTTGTTTGTTTTGTTTTTTTTTTGCCAGAAGCTGCAGTTT
SARS-CoV-2 Reference Genome NC_045512.2 (12638) CCTCTTATTGTAACAGCTTTAAGGGCCAATT-CTGCTGTCAAATTACAGAATAATGAGCTTAGTCCT--GTTGCACTAC
```

Section 177

```
                                              (13905) 13905 13910     13920     13930     13940     13950     13960     13970        13983
Oryza sativa chromosome 1 region 9.891-41.663nt (13630) AGCAGGTCCTTATTTTCGTCAGAAGTTACAAAGAGAGAATATGCCAATTTTTAGCTTTATATTTTGCTGAAGGCAGACA
SARS-CoV-2 Reference Genome NC_045512.2 (12714) GACAGATGTCTTGTGCTGCCGGTACT-ACACAAACTGCTTGCACTGATGACAATGCGT-TAGCTTACTACA---ACACA
```

Section 178

```
                                              (13984) 13984 13990     14000     14010     14020     14030     14040     14050        14062
Oryza sativa chromosome 1 region 9.891-41.663nt (13709) AGTTGTAGAGGAATATGCTGATCCTTAGAAAGAACTTTG-TGTAGCCTATTTGGATGGTTTCAGACTTTCAGTGATAGA
SARS-CoV-2 Reference Genome NC_045512.2 (12788) ACAAAGGGAGG--TAGGTTTGTACTTGCACTGTTATCCGATTTACAGGATTTGAAATGGGCTAGA--TTCCCTAAGAGT
```

Section 179

```
                                              (14063) 14063  14070     14080     14090     14100     14110     14120     14130      14141
Oryza sativa chromosome 1 region 9.891-41.663nt (13787) GTCGGAGCAATGTTGGTCTCATTTTCGTTTGCTGCTGGTTACTTGTATTAATACATTATAGAAAATGAGTACATAAATA
SARS-CoV-2 Reference Genome NC_045512.2 (12863) GATGGAAC--TGGTACTATCTATACAGAA--CTGGAACCACCTTGTAGGTTTG--TTACAGACAC-----ACCTAAAGG
```

Section 180

```
                                              (14142) 14142   14150     14160     14170     14180     14190     14200     14210   14220
Oryza sativa chromosome 1 region 9.891-41.663nt (13866) TACATGACGATATGATCCTGTAAGATATTCTATTCCATTATCAAAAAAAAT-GAAATCCCTGCCGTCCGATGCGATGG
SARS-CoV-2 Reference Genome NC_045512.2 (12931) TCC-TAAAGTGAAGTATTTATACTTTATTAAAGG--ATTAAACAACCTAAATAGAGGTATGGTACTTGGTAGTTTAGCT
```

Section 181

```
                                              (14221) 14221    14230     14240     14250     14260     14270     14280               14299
Oryza sativa chromosome 1 region 9.891-41.663nt (13944) GCGATGGTAAAACC-CTAGCTCGAAGCCAAGCACAGCCTGGGCCGGGCCCGGCCCAGCCCAACAAGTAGTCCGCTGTTA
SARS-CoV-2 Reference Genome NC_045512.2 (13007) GCCACAGTACGTCTACAAGCTGGTAATGCAACAGAA---GTGCCTG--CCAATTCAACTGTATTATCTTTCTGTGCTTT
```

Section 182

```
                                              (14300) 14300     14310     14320     14330     14340     14350     14360              14378
Oryza sativa chromosome 1 region 9.891-41.663nt (14022) TAAAGGAAGGTAGGCATCTCCCCCGCGCGCCGCGA-CTCGTTAGGCTACTCCACCCACTC-TTGTTTAGGGTTTCCC
SARS-CoV-2 Reference Genome NC_045512.2 (13081) TGCTGTAGATGCTGCTAAAGCTTACAAAGATTATCTAGCTAGTGGGGACAACCAATCACTAATTGTGTTAAGAT----
```

Section 183

(14379)	14379 14390 14400 14410 14420 14430 14440 14457
Oryza sativa chromosome 1 region 9.891-41.663nt (14099)	TCAGCCGCCGCCGCCGCATCCACGCTCAGCAGCCATGGCGGTGGTCGAGCAGCCGCAGCAGCAGGTGGTGAAGCTCTTC
SARS-CoV-2 Reference Genome NC_045512.2 (13156)	------------GTTGTGTACACACACTGGTAC--TGGTCAGGCAATAACAGTTACACCGGAAGCCAATATGGATCAAG

Section 184

(14458)	14458 14470 14480 14490 14500 14510 14520 14536
Oryza sativa chromosome 1 region 9.891-41.663nt (14178)	AACTGCTGGTCCTTCGAAGACGTTCAGGTTTGCCTGTCCTCCCATTATACTAGTAAGCAAGTAGGGGTACTCGTCTGGT
SARS-CoV-2 Reference Genome NC_045512.2 (13221)	AA-------TCCTTTGGTGGTGCATCGTGTTGTCTGTACTGCCGTTGCCACATAGATCATCCAAATCCTAAAGGATTTT

Section 185

(14537)	14537 14550 14560 14570 14580 14590 14600 14615
Oryza sativa chromosome 1 region 9.891-41.663nt (14257)	CTGTCG-ATAACTCTCGTTGGCGCAGGTGAACGACATATCCCTCGCCGACTACCTCGCGGTGTCCTCGACGAAGCACGC
SARS-CoV-2 Reference Genome NC_045512.2 (13293)	GTGACTTAAAAGGTAAGTATGTACAAAT-ACCTACAACTTGT--GCTAATGACCCTGTGGGTTTACACTTAAAAACAC

Section 186

(14616)	14616 14630 14640 14650 14660 14670 14680 14694
Oryza sativa chromosome 1 region 9.891-41.663nt (14335)	CACCTACCTGCCGCACACGGCTGGCCGCTACTCGGCGAAGCGCTTCCGCAAGGCGCAGTGCCCCTCGTGGAGCGCCTC
SARS-CoV-2 Reference Genome NC_045512.2 (13369)	AGTCT-------GTAC-CGTCTG--CGGTATGTGGAAAGGTTATGGCTGTAGTTGTGAT--CAACTCCGCGAACCCATG

Section 187

(14695)	14695 14700 14710 14720 14730 14740 14750 14760 14773
Oryza sativa chromosome 1 region 9.891-41.663nt (14414)	ACCAACTCCCTCATGATGCACGGCCGCAA--CAACGGCAAGAAGATCATGGCTGTCCGCATCGTCAAGCACGCCATGGA
SARS-CoV-2 Reference Genome NC_045512.2 (13436)	CTTCAGTCAGC--TGATGCACAATCGTTTTTAACGGGTTTGCGGT---GTAAGTGCAGCCCGTCTTACAC--CGTGCG

Section 188

(14774)	14774 14780 14790 14800 14810 14820 14830 14840 14852
Oryza sativa chromosome 1 region 9.891-41.663nt (14491)	GATCATCCACCTCCTCACCGACGCCAACCCCATCCAGGTCATCGTAGAT--GCCATCATCAAC-AGGTACCACCGTCCA
SARS-CoV-2 Reference Genome NC_045512.2 (13508)	GCACAGGCAC-TAGT-ACTGATGTCGT----ATACAGGGCTTTTGACATCTACAATGATAAAGTAGCTGGTTTTGCTAA

Section 189

(14853)	14853 14860 14870 14880 14890 14900 14910 14920 14931
Oryza sativa chromosome 1 region 9.891-41.663nt (14567)	ATTCCCAATTCCTTTCAGTTGCGCACTTCAATTGTCCAATTCACTTTTTTTTCAATCACCCTCACCCTCTTATATGCAA
SARS-CoV-2 Reference Genome NC_045512.2 (13581)	ATTCCTAAAAACTAATTGTTGT-CGCTTCCAAGA---AAAGGACGAAGATGACAATTTAATTGATTCT-TACTTTGTAG

Section 190

```
                                         (14932) 14932   14940     14950     14960     14970     14980     14990     15000   15010
Oryza sativa chromosome 1 region 9.891-41.663nt  (14646) ATGCTTATCACTGTC-CAATCACCCCCACCCTCACTCTTATATGCAAATGCTCATA-ACTGTAGTATTTTTTTTCTTTT
SARS-CoV-2 Reference Genome NC_045512.2 (13655) TTAAGAGACACACTTTCTCTAACTACCAACATGA-----AGAAACAATTTATAATTTACTTAAGGATTGTCCAGCTGTT
```

Section 191

```
                                         (15011) 15011    15020     15030     15040     15050     15060     15070             15089
Oryza sativa chromosome 1 region 9.891-41.663nt  (14723) --TACAAAT-ACTTCTATCCC---AAAATACACTCCTCTATTTTGCCCGTGCAATTAATGTTATCTTACTAGCCAAAAA
SARS-CoV-2 Reference Genome NC_045512.2 (13729) GCTAAACATGACTTCTTTAAGTTTAGAATAGACGGTGACATGGTACCACATATATCAC-GTCAACGTCTTA--CTAAAT
```

Section 192

```
                                         (15090) 15090     15100     15110     15120     15130     15140     15150           15168
Oryza sativa chromosome 1 region 9.891-41.663nt  (14796) TCAAGACGGGAATATATATATATATATATATATATATATATATATATATATATATATATATATATATATATATATATAT
SARS-CoV-2 Reference Genome NC_045512.2 (13805) ACACAATGGCAGACCTCGTCTATGCTTTAAGGCATTTTGATGAAGGTAATTGTG-ACACATTAAAAGAAATACTTGTC-
```

Section 193

```
                                         (15169) 15169      15180     15190     15200     15210     15220     15230           15247
Oryza sativa chromosome 1 region 9.891-41.663nt  (14875) ATATATATATATTGTTTGGTACGAGTAGTACATACGAAAATGTTTGTGTTACATGTCTAATGTGACAATTTACTACTAT
SARS-CoV-2 Reference Genome NC_045512.2 (13882) ACATACAATTGTTGTGATG-ATGATTATTTCA-ATAAAAAGGACTGGTATGATTTTGTA----GAAAACCCAGATATAT
```

Section 194

```
                                         (15248) 15248       15260     15270     15280     15290     15300     15310         15326
Oryza sativa chromosome 1 region 9.891-41.663nt  (14954) CACTGAATGAAATGAACACAAATAAACAAGCATG---ATTGTTGTTCACAGCGGCCCGCGTGAG-GACGCAACCCGTAT
SARS-CoV-2 Reference Genome NC_045512.2 (13955) TACGCGTATACGCCAACTTAGGTGAACGTGTACGCCAAGCTTTGTTAAAAACAGTACAATTCTGTGATGCCATGCGAAA
```

Section 195

```
                                         (15327) 15327         15340     15350     15360     15370     15380     15390       15405
Oryza sativa chromosome 1 region 9.891-41.663nt  (15029) CGGCTCTGCTGGCGCTGTGAGGAGGC-AGGCTGTGGATATCTCTCCCTTGAGGAGGGTCAACCAGGCAATCTACCTCCT
SARS-CoV-2 Reference Genome NC_045512.2 (14034) TGCTGGTATTGTTGGTGTACTGACATTAGATAATCAAGATCTCAATGGTAACTGGTATGATTTCGGTGATTT-CATACA
```

Section 196

```
                                         (15406) 15406          15420     15430     15440     15450     15460     15470      15484
Oryza sativa chromosome 1 region 9.891-41.663nt  (15107) CACCACTGGCGCCAGGGAGAGTGCCTTTAGGAACATCAAGACCATTGCTGAGTGCCTTGCTGATGAGCTCATCAATGCC
SARS-CoV-2 Reference Genome NC_045512.2 (14112) AACCACGCCAGGTAGTG-GAGTTCCTGTTGTAGATTCTT-ATTATT--------CATTGTTAATGCCTATATTAACCTT
```

Section 197

```
                                                  (15485) 15485 15490     15500     15510     15520     15530     15540     15550        15563
Oryza sativa chromosome 1 region 9.891-41.663nt   (15186) GCCAAGGGCTCATCCAACAGGTAAATCTTGCTTCTCATTTGATGAAATGAGGCTACTGACACATCCATGCTCTAATTCT
SARS-CoV-2 Reference Genome NC_045512.2           (14181) GACCAGGGCTT----------TAACTGCAGAGTCACATGT--TGACACT-GACT--TAACAAAGCCTTACATTAAGTGG
```

Section 198

```
                                                  (15564) 15564 15570     15580     15590     15600     15610     15620     15630        15642
Oryza sativa chromosome 1 region 9.891-41.663nt   (15265) GATATGTTACAGCTATGCCATCAAG-AAGA-AGGATGAGATTGAGCGTGTTGCCAAGGCGAACCGTTGAGTGCTGA---
SARS-CoV-2 Reference Genome NC_045512.2           (14245) GATTTGTTAAAA-TATGACTTCACGGAAGAGAGGTTAAAACTCTT--TGACCGTTATTTTAAATATTGGGATCAGACAT
```

Section 199

```
                                                  (15643) 15643 15650     15660     15670     15680     15690     15700     15710        15721
Oryza sativa chromosome 1 region 9.891-41.663nt   (15339) ACCCATCTCAGTGTGCTCGAGCTATTGCCTTGCACTGTTGCCAG-TACTTTTGCTAGTCTTTTTGGGCTTTGC----C
SARS-CoV-2 Reference Genome NC_045512.2           (14321) ACCACCCAAATTGTGTT--AACTGTTTGGATG-ACAGATGCATTCTGCATTGTGCAAACTTTAATGTTTTATTCTCTAC
```

Section 200

```
                                                  (15722) 15722 15730     15740     15750     15760     15770     15780     15790        15800
Oryza sativa chromosome 1 region 9.891-41.663nt   (15413) AGAGTTACCTGTGATTTTCTGTTTAATGATAAGTGCTGAGAAAATGTAGTAGTCTGTTCGCTATCGACAT--TGTTTTA
SARS-CoV-2 Reference Genome NC_045512.2           (14397) AGTGTTCCC----ACCTACAAGTTTTGGACCACTAGTGAGAAAAA-TATTTGT-TGATGGTGTTCCATTTGTAGTTTCA
```

Section 201

```
                                                  (15801) 15801 15810     15820     15830     15840     15850     15860               15879
Oryza sativa chromosome 1 region 9.891-41.663nt   (15490) TTACACCCCCAATCCTTGTTCCTGCATGATCTGAGTTCTTTATGTGTGAT-TGACTGATGATTGGTTCTGTTTCTGATT
SARS-CoV-2 Reference Genome NC_045512.2           (14470) ACTGGATACCACTTCAGAGAGCTAGGTGTTGTACATAATCAGGATGTAAACTTACATAGCTCTAGACTTAGTTTTAAGG
```

Section 202

```
                                                  (15880) 15880 15890     15900     15910     15920     15930     15940               15958
Oryza sativa chromosome 1 region 9.891-41.663nt   (15568) ATGTTACTTGAATTTCAAGCTGTAGATGATAGCTTAGATTAGGTTCATTATGT-TTACTCGACTTAGG--ATGTGTTCG
SARS-CoV-2 Reference Genome NC_045512.2           (14549) AA-TTACTTGTGTATGCTGCTGACCCTGCTATGCACGCTGCTTCTGGTAATCTATTACTAGATAAACGCACTACGTGCT
```

Section 203

```
                                                  (15959) 15959 15970     15980     15990     16000     16010     16020               16037
Oryza sativa chromosome 1 region 9.891-41.663nt   (15644) TTTGGGTGATAGGGAGTGATAATGCACATCGTTTTCCGCGCGCACGCTTCCCAAACTACTAAATGGTGTGTAAATGGTG
SARS-CoV-2 Reference Genome NC_045512.2           (14627) TTTCAGT-AGCTGCACTTACTAACAATGTTGCTTTTCAAACTGTCAAACCCGGTAATTTAACAAAGACTTCTATGACT
```

Section 204

```
(16038) 16038 16050 16060 16070 16080 16090 16100 16116
Oryza sativa chromosome 1 region 9.891-41.663nt  (15723) TGTTTTTTGCAAAAATTTTCTATATGAAAGTTGCTTTAAAAAAATCATATTAATTCATTTTTGAAATTTAAAATAGTTA
SARS-CoV-2 Reference Genome NC_045512.2 (14705) TTGCTGTGTCTAAGGGTTTCTTTAAGGAAGGAAGTTCTGTTGAATTAAAACACTTCTTCTTTGC---TCAGGATGGT-A
```

Section 205

```
(16117) 16117 16130 16140 16150 16160 16170 16180 16195
Oryza sativa chromosome 1 region 9.891-41.663nt  (15802) ATACTCAATTAATCATGAGTTAATGGCTCATCTCGTTTTGCGTATCTTCCCAATCTCCTCAATCCCCTTTTCCTCAAAC
SARS-CoV-2 Reference Genome NC_045512.2 (14780) ATGCTGCT---ATCA-GCGATTATGACTACTATCGTTATA---ATCTACCAA-------CAATGTGTGATA--TCAGAC
```

Section 206

```
(16196) 16196 16210 16220 16230 16240 16250 16260 16274
Oryza sativa chromosome 1 region 9.891-41.663nt  (15881) ACACCCTTAGTTAAGCTTGCTGGTCTTGGTATGT-CTCTGAAATACTCGCTGCTTGCATGCCATATGCAAAGTTTATCA
SARS-CoV-2 Reference Genome NC_045512.2 (14843) A-ACTACTATTTGTAGTTGAAGTTGTTGATAAGTACTTTGATTGTTACGATGGTGGC-TG---TATT-AATGCTAACCA
```

Section 207

```
(16275) 16275 16280 16290 16300 16310 16320 16330 16340 16353
Oryza sativa chromosome 1 region 9.891-41.663nt  (15959) ACTAACTTCGTATCTGCACCACCCTAAATGGAATAAGAAAATTCTTATTTGTGAATGAACTCGGGCAAATGGCCGCCTA
SARS-CoV-2 Reference Genome NC_045512.2 (14916) AGTCA--TCGT-----CAACAACCTA----GACAAATCAGCTGGTTTTCCATTTAATAAATGGGGTAA--GGCTAGACT
```

Section 208

```
(16354) 16354 16360 16370 16380 16390 16400 16410 16420 16432
Oryza sativa chromosome 1 region 9.891-41.663nt  (16038) CCAGTTTGGTATCAGCAC--AAGTAAATTATGGTGTATGTATTTCACATGAACAGTCAATTCCAATTGTTGAGTTAAGC
SARS-CoV-2 Reference Genome NC_045512.2 (14982) TTATTATGATTCAATGAGTTATGAGGATCAAGATGCACTTTTCGCATATACAAAACGTAATGTCATCCCTACTATAA-C
```

Section 209

```
(16433) 16433 16440 16450 16460 16470 16480 16490 16500 16511
Oryza sativa chromosome 1 region 9.891-41.663nt  (16115) TGAAAATTTGATATATCATTTGCAACGTGGATAAATGAGATTGCTGAAATCTGGGGTAGTTCTGTGTGAGGTTGCTATC
SARS-CoV-2 Reference Genome NC_045512.2 (15060) TCAAA--TGAATCTTAAGTATGCCATTAGTGCAAAGAATAGAGCTCGCACC----GTAGCTG-GTGTCTCTAT-CTGTA
```

Section 210

```
(16512) 16512 16520 16530 16540 16550 16560 16570 16580 16590
Oryza sativa chromosome 1 region 9.891-41.663nt  (16194) ATATTATAAACTTAAGATTGTAAACATCCAAAATTTGGCCAAAAACTTGAAATCTGATATTTCGATTATTTTATCCAAA
SARS-CoV-2 Reference Genome NC_045512.2 (15131) GTACTATGACCAATAGACAGTTT-CATCAAAAATTATTGAAATCAATAG----CCGCCACTA-GAGGAGCTACTGTAGT
```

```
                                                               Section 211
                                          (16591) 16591 16600 16610 16620 16630 16640 16650 16669
Oryza sativa chromosome 1 region 9.891-41.663nt (16273) TTTTGGAAGTTTTTGTCTTAACGGTATTTTGGCCATTTCGACAAATTTGTACGGTACTAATGGAGGCTAACTGGACGGC
SARS-CoV-2 Reference Genome NC_045512.2 (15204) AATTGGAACAAGCAAATTCTATGGTGGTTGGCACAACATGTTAAAAACTGTTTATAGTGATGTAGAAAACCCTCACCTT
                                                               Section 212
                                          (16670) 16670 16680 16690 16700 16710 16720 16730 16748
Oryza sativa chromosome 1 region 9.891-41.663nt (16352) GATGGTAAATTGTAGAAATTAAGAAAAAGTCGATGGCATATTGTAGAACGTGATAAAGTTAGTGGCAAATCGTAGATGT
SARS-CoV-2 Reference Genome NC_045512.2 (15283) -ATGG------GTTGGGATTATCCTAAATGTGATAGAGCCATGCCTAACATGCTTAGAATTATGGCCTCACTTGTTCTT
                                                               Section 213
                                          (16749) 16749 16760 16770 16780 16790 16800 16810 16827
Oryza sativa chromosome 1 region 9.891-41.663nt (16431) G-TGACAAAGTCAGTGGCATATAATAGGTTGTCCCATGTAATA--GAATCTTTCCCTGCCTTAAAAAGAAGTTTGAAAG
SARS-CoV-2 Reference Genome NC_045512.2 (15355) GCTCGCAAA--------CATACAACGTGTTGTAGCTTGTCACACCGTTTCTATAGATTAGCTAATGAGTGTGCTCAAGT
                                                               Section 214
                                          (16828) 16828 16840 16850 16860 16870 16880 16890 16906
Oryza sativa chromosome 1 region 9.891-41.663nt (16507) GGCAAAAGAAAGAGAGA-GAGAGAGAATT---AATATGTCATCTTATGATTATAATTATATGCCAATCCAACATTCGTG
SARS-CoV-2 Reference Genome NC_045512.2 (15426) ATTGAGTGAAATGGTCATGTGTGGCGGTTCACTATATGTTAAACCAGGTGGAACCTCATCAGGAGATGCCACAACTGCT
                                                               Section 215
                                          (16907) 16907 16920 16930 16940 16950 16960 16970 16985
Oryza sativa chromosome 1 region 9.891-41.663nt (16582) TAAATATAAATATATTTCCATGCCGTAAAAAAGTTTGAAAGGGCTAAAGAAAAAGAGGAGAGAGAGAGAGAGAGGGG
SARS-CoV-2 Reference Genome NC_045512.2 (15505) TA--TGCTAATAGTGTTTTTTAACATTTGTCAAGCT-GTCACGGCCAATGTTAATGCACTTTTATCTACTGATGGTAACA
                                                               Section 216
                                          (16986) 16986 17000 17010 17020 17030 17040 17050 17064
Oryza sativa chromosome 1 region 9.891-41.663nt (16661) GGGGGGGGGGCGGGGTGGTGTGGAATTGATGATAGGCCAAAGGTGGTGAGGAAGAATCTGGGTCGTCCAGGCAAGATCC
SARS-CoV-2 Reference Genome NC_045512.2 (15581) AAATTGCCGATAAGTATGTCCGCAATTTACAACA---CAGACTTTATGAGTGTCTCTATAGAAAT---AGAGATGTTGA
                                                               Section 217
                                          (17065) 17065 17070 17080 17090 17100 17110 17120 17130 17143
Oryza sativa chromosome 1 region 9.891-41.663nt (16740) CACGGGCGATGCGAACGAGCA--CCGGATCCGCTGCGGCTGCTCGGCGTCGGGTCGGAGGTGAGGTCTCGAAACCCTAG
SARS-CoV-2 Reference Genome NC_045512.2 (15654) CACAGACTTTGTGAATGAGTTTTACGCATATT-TGCGTAAACATTTC-TCAATGATGATACTCTCTGACGATGCTGTTG
```

```
                                                                                                                   Section 218
                                            (17144) 17144 17150     17160     17170     17180     17190     17200     17210    17222
  Oryza sativa chromosome 1 region 9.891-41.663nt  (16817) C-TGCTCCGACGACGATCGTGGCAGCCGCCGTCGCCATGGTGCTGGGAAAGATCGTCATCGTCATCGGCTCCGGTAGGG
SARS-CoV-2 Reference Genome NC_045512.2 (15731) TGTGTTTCAATAGCACTTATGCATCTCAAGGTCTAG-TGGCTAGCATAAAGAACTTTAA-GTCAGTT-CTTTATTATCA
                                                                                                                   Section 219
                                            (17223) 17223 17230     17240     17250     17260     17270     17280     17290    17301
  Oryza sativa chromosome 1 region 9.891-41.663nt  (16895) ATCCACTCTCGCGATTTCTGTGCGTCTAATCTGTATGTATAGGGTAGCTTGGCCCAATCCCCCTCTCTCTCGTTAGCC
SARS-CoV-2 Reference Genome NC_045512.2 (15807) AAACAATGTTTTTATGTCTGAAGCAAAATGTTGGACTGAGACTG-ACCTTACTAAAGGACCTCATGAATTTTGCT---C
                                                                                                                   Section 220
                                            (17302) 17302 17310     17320     17330     17340     17350     17360     17370    17380
  Oryza sativa chromosome 1 region 9.891-41.663nt  (16974) TCTAGCTCGATTCGCTCCTGCTCCTGTTTGTTGCTTCATTTACTGGAGTAGCTTCGATTAGCTTTACTTGTTTGGAAAT
SARS-CoV-2 Reference Genome NC_045512.2 (15882) TCAACATACAAT-GCTAGTTAAACAGGGTGATGATT-ATGT------GTACCTTCC-TTACCCAGATCCATCAAGAA-T
                                                                                                                   Section 221
                                            (17381) 17381 17390     17400     17410     17420     17430     17440              17459
  Oryza sativa chromosome 1 region 9.891-41.663nt  (17053) TGCAGGAATCGTTGGGACATTACTCACCAGCGGAGAGGCCAAGATTGCGCTCCCCGATTTTAGGGATGTGCTCTCTGGT
SARS-CoV-2 Reference Genome NC_045512.2 (15951) CCTAGGGGCCGGCTGTTTTGTAGATGATATCGTAAAAACAGATGGTACACTTAT-GATTGAACGGTTCGTGTCTTTAG-
                                                                                                                   Section 222
                                            (17460) 17460 17470     17480     17490     17500     17510     17520              17538
  Oryza sativa chromosome 1 region 9.891-41.663nt  (17132) GCCTTCAAGGTTGTTCTTTTTTCTTTCAATTTCAATCATTCCTTCCTTTCAGTAGTATTTTGTTTTTATTTAGAGTAAA
SARS-CoV-2 Reference Genome NC_045512.2 (16028) -CTATAGATGCTTACCCACTTACTAAACATCCTAATCAGGAGTA---TGCTGATGTCTTTCATTTGTACTTACAATACA
                                                                                                                   Section 223
                                            (17539) 17539 17550     17560     17570     17580     17590     17600              17617
  Oryza sativa chromosome 1 region 9.891-41.663nt  (17211) TTTCACAAAACTACAT----ATTTATGGTTCACGTTGCAGAAAACCACA-TATTTGAT-ACTTGGCACTTAAGTACA
SARS-CoV-2 Reference Genome NC_045512.2 (16103) TA--AGAAAGCTACATGATGAGTTAACAGGACACATGTTAGACATGTATTCTGTTATGCTTACTAATGATAACACTTCA
                                                                                                                   Section 224
                                            (17618) 17618 17630     17640     17650     17660     17670     17680              17696
  Oryza sativa chromosome 1 region 9.891-41.663nt  (17284) TATATTTTGGTAGTTTAGTTTCACAAAACCACACTATCGATGAATGGATTCACTTGTGATATGACGACGTGG-TTGTTT
SARS-CoV-2 Reference Genome NC_045512.2 (16180) AGGTATTGGGAACCTGAGTTTTATGAGGCTATGTACACACCGCATACAGTC-----TTACAGGCTGTTGGGCTTGTGT
```

Section 225

```
                                                  (17697) 17697        17710     17720     17730     17740     17750     17760          17775
Oryza sativa chromosome 1 region 9.891-41.663nt   (17362) CATCTAGTATGAGGACGTGGCATCATATTAATTTCGTGCGATGGCTGGTAATAGGATGCCATGTCCTCGTCCTAGGTGA
SARS-CoV-2 Reference Genome NC_045512.2           (16254) TCTTTGCAATTCACA---GACTTCATTAAGATGTGGTGCT-TGCATACGTAGACCATTC--TTATGTTGTAAATGCTGT
```

Section 226

```
                                                  (17776) 17776          17790     17800     17810     17820     17830     17840         17854
Oryza sativa chromosome 1 region 9.891-41.663nt   (17441) AACAGCCACATCATCTCGCGGGTGAATCTATACATCGATAATGTAGTTTTGTGAAACTACACGACCAAAATATATGTAC
SARS-CoV-2 Reference Genome NC_045512.2           (16327) TACGACCATGTCATATC---------AACATCACATAAATTAGTCTTGTCTGTTAATCCGTATGTTTGCAATG------C
```

Section 227

```
                                                  (17855) 17855 17860     17870     17880     17890     17900     17910     17920       17933
Oryza sativa chromosome 1 region 9.891-41.663nt   (17520) TCAAGTGCCAAGTGTCAAAGTGCGTGTGGTTTTCTGCAACTTGTACCACAAAATATGTAGTTTTGTGAAATTTACTCTT
SARS-CoV-2 Reference Genome NC_045512.2           (16392) TCCAGGTTGTGATGTCACAGA---TGTGACT-----CAACTT-TAC-------TTAGGAGGTATGAGCTATTA--TTGT
```

Section 228

```
                                                  (17934) 17934 17940     17950     17960     17970     17980     17990     18000       18012
Oryza sativa chromosome 1 region 9.891-41.663nt   (17599) TTATTTAAGACATAAAAGGTTCCTTTGCTCCTTTGCTCTGCTCT-CTAGTTTGTGACCAAGCAAGACAAAAAGGATGGC
SARS-CoV-2 Reference Genome NC_045512.2           (16453) AAATCACATAAACCACCCATTAGTTTTCCATTGTGTGCTAATGGACAAGTTTTTGGTTTAT--ATAAAAATA-------
```

Section 229

```
                                                  (18013) 18013  18020     18030     18040     18050     18060     18070     18080      18091
Oryza sativa chromosome 1 region 9.891-41.663nt   (17677) CCATCCACCAGCAGCCCTCACGCTGCTCACTTGCTATCTCAGGTCTCCCACTCGTCATCTCTTACTGTACTTCATCGCT
SARS-CoV-2 Reference Genome NC_045512.2           (16523) -CATGTGTTGGTAGCGATAATGTTACTGACTT------TAATG-----CAATTGCAACATGTGACTGGACA--AATGCT
```

Section 230

```
                                                  (18092) 18092   18100     18110     18120     18130     18140     18150     18160  18170
Oryza sativa chromosome 1 region 9.891-41.663nt   (17756) GGATGGCTCCATGATTAACCCACTGCTCATCTAACTATTAGGTCAATCATCTCAGAGAGGACCTCCAACTTTTGTCCAG
SARS-CoV-2 Reference Genome NC_045512.2           (16588) GG-TGATTACATT-TTAGCTAACACCTGTACTGA--AAGAC-TCAAGCTTTTGCAGCAGA-----AACGCT----CAA
```

Section 231

```
                                                  (18171) 18171    18180     18190     18200     18210     18220     18230              18249
Oryza sativa chromosome 1 region 9.891-41.663nt   (17835) GTCAAATCAAGTTGCTATTGTCACCGTCG-ATGGTAGACCAGGTCTCTCCCATTCTTCTCTGTTCTGTTGTATCTGCAG
SARS-CoV-2 Reference Genome NC_045512.2           (16653) AGCTACTGAGGAGAC-ATTTAAACTGTCTTATGGTA-------TTGCTACTGTACGTGAA-GTGCTGTCTGACAGAGAA
```

```
                                                                                                   Section 232
                                                   (18250) 18250     18260     18270     18280     18290     18300     18310            18328
Oryza sativa chromosome 1 region 9.891-41.663nt    (17913) TGTGCTTTTTTCCTGGAA--TTATTAACCATAACCTATTAACCTAGTGATATTTATGTTTTCTTTACCGTCCATATCGT
SARS-CoV-2 Reference Genome NC_045512.2            (16723) T-TACATCTTTCATGGGAAGTTGGTAAACCTAGACCACCACTTAACCGAAATT-ATGT---CTTTACTGGT--TATCGT
```

```
                                                                                                   Section 233
                                                   (18329) 18329      18340     18350     18360     18370     18380     18390           18407
Oryza sativa chromosome 1 region 9.891-41.663nt    (17990) TTTTCCTGCCAACCATTATCTTGGAGGAATACATTAAGCCCATTTTCTGATCTAACTAATTTATGGAAACTTGCTCGTG
SARS-CoV-2 Reference Genome NC_045512.2            (16795) GTAAC-TAAAAACAGTAAAGTACAAATAGGAGAGTACACC-----TTTGAAAAAGGTGACT-ATGGTGA--TGCTGTTG
```

```
                                                                                                   Section 234
                                                   (18408) 18408       18420     18430     18440     18450     18460     18470          18486
Oryza sativa chromosome 1 region 9.891-41.663nt    (18069) AA-ATCGTTTGATGCCCTTTTGGACACCAAATTTTTGTATGTAATTTTTGCAGCTACTTCATTGTCTTCTATACTAACG
SARS-CoV-2 Reference Genome NC_045512.2            (16865) TTTACCGAGGTACAACAACTTACAAATTAAATGTTGGTGATTATTTTGTGCTG--ACATCACA------TACAGTAATG
```

```
                                                                                                   Section 235
                                                   (18487) 18487        18500     18510     18520     18530     18540     18550       18565
Oryza sativa chromosome 1 region 9.891-41.663nt    (18147) CTGACAAATTCTT-TCTTTTGCAGGCCCTGGTGCT-TATGGTATAACAGCTGTTGTTGCTGGGGCTATCGGCTATTTAT
SARS-CoV-2 Reference Genome NC_045512.2            (16936) CCATTAAGTGCACCTACACTAGTGCCACAAGAGCACTATGTTAGAATTACTG-----GCTTATACC--CAACACTCAAT
```

```
                                                                                                   Section 236
                                                   (18566) 18566         18580     18590     18600     18610     18620     18630      18644
Oryza sativa chromosome 1 region 9.891-41.663nt    (18224) ACATAAGGTGGAAGGTAAAAGTAATCACTGATTCTCAACTTGGCAGAAGGGTGCTATTGAACAATTATAAACACATCAT
SARS-CoV-2 Reference Genome NC_045512.2            (17008) ATCTCAGATG-AGTTTTCTAGCAATG------TTGCAAATTATCAAAGGTTGGTATGCAAAAGTATTCTACACTCCAG
```

```
                                                                                                   Section 237
                                                   (18645) 18645 18650     18660     18670     18680     18690     18700     18710    18723
Oryza sativa chromosome 1 region 9.891-41.663nt    (18303) CTTAGACTATCAGACAA-GCAAAGTTTTCTCTTTTTCTCTCTCCTTCCTTTTCATTTTAGGGCAACTGTCATAATCCAC
SARS-CoV-2 Reference Genome NC_045512.2            (17080) G---GACCACCTGGTACTGGTAAGAGTCATTTTGCTAT-TGGCCTAGCTCTCTACTACCCTTCTGCTCGCATAGTGTAT
```

```
                                                                                                   Section 238
                                                   (18724) 18724 18730     18740     18750     18760     18770     18780     18790    18802
Oryza sativa chromosome 1 region 9.891-41.663nt    (18381) AAAATGGGGCAATCAAGCTAACTCGGTGATTTACAGGGTTGGAAACTTTCTGATTTGATGTTTGTGACGAAGCGTGGCT
SARS-CoV-2 Reference Genome NC_045512.2            (17155) ACAGCTTG-CTCTCATGCCG-CT-GTTGATGCACTATGTGAGAAGGCATTAAAATATTTGCCTATAGATAAATGTAGTA
```

```
Section 239
                                            (18803) 18803  18810     18820     18830     18840     18850     18860     18870    18881
Oryza sativa chromosome 1 region 9.891-41.663nt (18460) TATCTGATGCCTGCGATGTGGTTGGCAAACAGTTGGAACATGTTTCAGAGAATGTTAATGTAAGTTTCTCCTTCGTTCA
SARS-CoV-2 Reference Genome NC_045512.2 (17231) GAATT-ATACCTGC-ACGTGCTCG---------TGTAGAGTGTTTT-GATAAATTCAAAGTGAATTCAACATTAGAACA

Section 240
                                            (18882) 18882   18890     18900     18910     18920     18930     18940     18950    18960
Oryza sativa chromosome 1 region 9.891-41.663nt (18539) CTGGAATATGAATATTGCACTATTTGAAGCTTTTACATGCATACTGCAATGATCCTATATTTCTCTCTTTGGGAAGTTG
SARS-CoV-2 Reference Genome NC_045512.2 (17298) GT-----ATGTCTTTTGTACTGTA--AATGCATTGCCTG-AGACGACA--GCAGATATAGTTGTCT-TTGATGAAATTT

Section 241
                                            (18961) 18961    18970     18980     18990     19000     19010     19020             19039
Oryza sativa chromosome 1 region 9.891-41.663nt (18618) GAACTGTTGTTTGTTGAATGGAGTTCAATCCTTTAACATCCCGTTAAAGTTTGTGGCATACTGTATGTCAATCACTATT
SARS-CoV-2 Reference Genome NC_045512.2 (17366) CAATGGCCACAAATT--ATG-ATTTGAGTGTTGTCAATGCCAGATTACGTGCTAAGCA--CTATGTGT-----AC-ATT

Section 242
                                            (19040) 19040     19050     19060     19070     19080     19090     19100             19118
Oryza sativa chromosome 1 region 9.891-41.663nt (18697) GGACTGCCACCTCAAATGAGAGAGTACACATCAGGCCGCTCAATTAAAATGGTTAAAGGGGAAATATCTAGTGAAAACC
SARS-CoV-2 Reference Genome NC_045512.2 (17434) GGCGACCCTGCTCAATTACCTG------CACCA---CGCACATTGCTAAC---TAAGGGCACACTAGAACCAGAATATT

Section 243
                                            (19119) 19119      19130     19140     19150     19160     19170     19180            19197
Oryza sativa chromosome 1 region 9.891-41.663nt (18776) ATCAAATAAATG--AAAACCTGTGAAAACC---GGACCGAATCTAAATTCATAACAGTTTCCTATTACTGTTCACCTGA
SARS-CoV-2 Reference Genome NC_045512.2 (17501) -TCAATTCAGTGTGTAGACTTATGAAAACTATAGGTCCAGACATG--TTCCTCGGAACTTGTCGGCGTTGTCCTGCTGA

Section 244
                                            (19198) 19198       19210     19220     19230     19240     19250     19260           19276
Oryza sativa chromosome 1 region 9.891-41.663nt (18850) AATTTGTCTTTTTTGTTCCTCCCAAATGAAGTTGAGTTTTTACTTAAGATTTGGTGGGAATGTTGCCTCTAGTTTTTTT
SARS-CoV-2 Reference Genome NC_045512.2 (17577) AATT-GTTGACACTGTGAGTGCTTTG-GTTTATGATAATAAGCTTAAAGCACATAAAGACAAAT-CAGCTCAATGCTTT

Section 245
                                            (19277) 19277        19290     19300     19310     19320     19330     19340          19355
Oryza sativa chromosome 1 region 9.891-41.663nt (18929) TTCATGAATTTAGAGAACTTTTAGGTCCGGTTTTCACTTATTTGATGGTTTTCACCAGATATGTCCACATGGTTAGAAT
SARS-CoV-2 Reference Genome NC_045512.2 (17653) AAAATGT-TTTATAAGGGTGTTATCACGCATGATGTTTCATCTGCAA-TTAACAGGCCACAAATAGGCGTGGTAAGAGA
```

Section 246

(19356) 19356 19370 19380 19390 19400 19410 19420 19434

Oryza sativa chromosome 1 region 9.891-41.663nt (19008) ACCTTGGATTTGCAGGATGACCTTTCTTGGATATGAACATTTACAATTATGTGTTGTTTTTGTATATGGCATCTTTATA

SARS-CoV-2 Reference Genome NC_045512.2 (17730) A--TTCC--TTACACG-TAACCCTGCTTGGA---GAAAAGCTGTCTTTATTTCACCTTATAATTCACAGAATGCTGTAG

Section 247

(19435) 19435 19440 19450 19460 19470 19480 19490 19500 19513

Oryza sativa chromosome 1 region 9.891-41.663nt (19087) ATTCTATTAAGCATTCTTGACCTATTTGGACCAAACAAGAAAAGTTAACCTGAGAAAACAAAACAGAATCTATGATTCC

SARS-CoV-2 Reference Genome NC_045512.2 (17801) CCTC----AAAGATTTTGGGACTACCAACTCAAACTGTTGATTCATCACAGGGCTCAGAATATGACTATGTCATATTCA

Section 248

(19514) 19514 19520 19530 19540 19550 19560 19570 19580 19592

Oryza sativa chromosome 1 region 9.891-41.663nt (19166) CACCTCCCACT---AGATATGCCTCTGAACATTTTGAGGCTAGCTTTGTTATACTGTTGCATCTGC----GTGTGCTTA

SARS-CoV-2 Reference Genome NC_045512.2 (17876) CTCAAACCACTGAAACAGCTCACTCTTGTAATGTAAA--CAGATTTAATGTTGCTATTACCAGAGCAAAAGTAGGCATA

Section 249

(19593) 19593 19600 19610 19620 19630 19640 19650 19660 19671

Oryza sativa chromosome 1 region 9.891-41.663nt (19238) CT---CCCACTGTTTGATTCAAA--TTCGTGTTTATGTGCAATCTTCTGATATC-AGGCTGCAAAGAGGCATCTGGCAG

SARS-CoV-2 Reference Genome NC_045512.2 (17953) CTTTGCATAATGTCTGATAGAGACCTTTATGACAAGTTGCAATTTACAAGTCTTGAAATTCCACGTAGGAATGTGGCAA

Section 250

(19672) 19672 19680 19690 19700 19710 19720 19730 19740 19750

Oryza sativa chromosome 1 region 9.891-41.663nt (19311) GAAGAATAGATCA---TGTAGATTGTACTTTAGATGAATGCCAGGAAATTACAGAATCCACAAGAAAAGAGGTTTGAAT

SARS-CoV-2 Reference Genome NC_045512.2 (18032) CTTTACAAGCTGAAAATGTA-ACAGGACTCTTTAAAGATTGTAGTAAGGTAATCACTGGGTTACATCCTACACAGGCAC

Section 251

(19751) 19751 19760 19770 19780 19790 19800 19810 19829

Oryza sativa chromosome 1 region 9.891-41.663nt (19387) GCTCAAGCATTA-TGTTAATAGGATTTTATATAGTTTA---TTGGAGGTTGATACAAAATTAATTCAATAGGTTACAGT

SARS-CoV-2 Reference Genome NC_045512.2 (18110) CTACACACCTCAGTGTTGACACTAAATTCAAAACTGAAGGTTTATGTGTTGACATACCTGGCATACCTAAGG--ACA-T

Section 252

(19830) 19830 19840 19850 19860 19870 19880 19890 19908

Oryza sativa chromosome 1 region 9.891-41.663nt (19462) CATCCAT-GAAGATATAAGTGCCTTCCAGGAGGAAATGCAATCAGTTCATCTTGTGGTTCGT-ACTCTGGTAGGTGCCT

SARS-CoV-2 Reference Genome NC_045512.2 (18186) GACCTATAGAAGACTCATCT--CTATGATG-GGTTTTAAAATGAATT-ATCAAGTTAATGGTTACCCTAACATGT---T

```
                                                                Section 253
                                                 (19909) 19909      19920     19930     19940     19950     19960     19970           19987
Oryza sativa chromosome 1 region 9.891-41.663nt  (19539) TGTATTCCGCATACACTTCTTTTTCAAGATAACTCTTTTCCTCACTGGTGCTAGATGGAAACTGACTTCTTCCCTTGTA
SARS-CoV-2 Reference Genome NC_045512.2          (18258) TATCACCCGCGAAGAAG-CTAT---AAGACATGTACGTGCATGGATTGGCTTCGATGTCGAGGGGTGTCATGC----TA
                                                                Section 254
                                                 (19988) 19988       20000     20010     20020     20030     20040     20050          20066
Oryza sativa chromosome 1 region 9.891-41.663nt  (19618) CTACAGGAGACAAAGCTTGGACGTCTTTCATATACCCAAGTAATTA-AGAGTTTGCAACAATATAACTTGCTTTACCCC
SARS-CoV-2 Reference Genome NC_045512.2          (18329) CTAGAGAAGCTGTTGGTACCAATTTACCTTTACAGCTAGGTTTTTCTACAGGTGTTAACCT----AGTTGCTGTACCTA
                                                                Section 255
                                                 (20067) 20067        20080     20090     20100     20110     20120     20130         20145
Oryza sativa chromosome 1 region 9.891-41.663nt  (19696) CAGAA-ATATGCATTTTGCTAA-----CCTCTTTTACTGAAATGTCTTAGGATCGTACAGCACGAG--GAATTTATGA-
SARS-CoV-2 Reference Genome NC_045512.2          (18404) CAGGTTATGTTGATACACCTAATAATACAGATTTTTCCAGAGTTAGTGCTAAACCACCGCCTGGAGATCAATTTAAACA
                                                                Section 256
                                                 (20146) 20146         20160     20170     20180     20190     20200     20210       20224
Oryza sativa chromosome 1 region 9.891-41.663nt  (19766) -CTTATGCGAGTTCACTA-AAAGG------CTGGATAAGAGCCCCAAAACTGATACTCGTCAGGCAAGTTAGCACAATG
SARS-CoV-2 Reference Genome NC_045512.2          (18483) CCTCATACCACTTATGTACAAAGGACTTCCTTGGAATGTAGTGCGTATAAAGATTGTACAAATGTTAAGTGACACACTT
                                                                Section 257
                                                 (20225) 20225 20230     20240     20250     20260     20270     20280     20290     20303
Oryza sativa chromosome 1 region 9.891-41.663nt  (19837) CCCAAAAGCTATTTTTGGCAGACCTTGCTATGTTGCTAGCCTGAACTTCTATTCCCTTACGATCCCATACTAGAACATT
SARS-CoV-2 Reference Genome NC_045512.2          (18562) ----AAAAATCTCTCTGACAGAG--TCGTATTTGTCTTATGGGCAC----ATGGCTTTGAGTTGACATCTATGAAGTAT
                                                                Section 258
                                                 (20304) 20304  20310     20320     20330     20340     20350     20360     20370    20382
Oryza sativa chromosome 1 region 9.891-41.663nt  (19916) ATTGATATAAAAAGGCATACACTTACACAT-GAGTTTCTGATAGATTGTGTAAAATG-------ACTGCT-CAGACATT
SARS-CoV-2 Reference Genome NC_045512.2          (18631) TTTGTGAAAATAGGACCTGAGCGCACCTGTTGTCTATGTGATAGAC-GTGCCACATGCTTTTCCACTGCTTCAGACACT
                                                                Section 259
                                                 (20383) 20383   20390     20400     20410     20420     20430     20440     20450   20461
Oryza sativa chromosome 1 region 9.891-41.663nt  (19986) GAT-----TTGGCATTATAAGTTATGATATGGCTCC-TACATGACCAACTTTAATCTATTTCTTTGATCCATGAGATA
SARS-CoV-2 Reference Genome NC_045512.2          (18709) TATGCCTGTTGGCATCATTCTATTGGATTTGATTACGTCTATAATC--CGTTTATG-ATTGATGTTCAACAATGGGGTT
```

Section 260

```
                                                     (20462) 20462   20470     20480     20490     20500     20510     20520     20530   20540
Oryza sativa chromosome 1 region 9.891-41.663nt      (20059) TCCCTCAGGCAATGTCCCTCGACAC-AGTTTCTGGCGTGATACCAATTACGATGTATTAAGTGGCACCATGAAGCT--T
SARS-CoV-2 Reference Genome NC_045512.2              (18785) TTAC--AGGTAACCTACAAAGCAACCATGATCTGTATTGT---CAAGTCCATGGTAAT-----GCAC-ATGTAGCTAGT
```

Section 261

```
                                                     (20541) 20541    20550     20560     20570     20580     20590     20600            20619
Oryza sativa chromosome 1 region 9.891-41.663nt      (20135) TCTTATATCATCCTTCTATACTGATAGTCTTGATTTTTCTTAATCGATTTCCAGGTCTTATCA-TCAACTCCTCTTCCA
SARS-CoV-2 Reference Genome NC_045512.2              (18853) TGTGATGCAATCATG----ACTAGGTGTCTAGCTGTCCACGAGTGCTTTGTTAAGCGTGTTGACTGGACTATTGAATAT
```

Section 262

```
                                                     (20620) 20620     20630     20640     20650     20660     20670     20680           20698
Oryza sativa chromosome 1 region 9.891-41.663nt      (20213) GCTATTGAATTGCCAGAGAGAATTACAAGGGTAAAGTTGTGCACCTTTTTGAATGTTAACATGATTTGGATTATCTGAC
SARS-CoV-2 Reference Genome NC_045512.2              (18928) CCTAT--AATTG---GTGATGAACTGAAGATTAA-----TGCGGCTTGTAGAAAGGTT-CAACACATGG-TTGTTAAAG
```

Section 263

```
                                                     (20699) 20699      20710     20720     20730     20740     20750     20760          20777
Oryza sativa chromosome 1 region 9.891-41.663nt      (20292) CT--TTTATTTTCTTACCACAGGCTGCTTCTTTGCCTCCAAGTTCAGAACCGGAGTTTTCTGGCCCTCGATCACCTGTA
SARS-CoV-2 Reference Genome NC_045512.2              (18995) CTGCATTATTAGCAGACAAATTCCCAGTTCTT---CACGACATTGGTAACCCTAAAGCTATTAAGTGTGT--ACCTCAA
```

Section 264

```
                                                     (20778) 20778       20790     20800     20810     20820     20830     20840         20856
Oryza sativa chromosome 1 region 9.891-41.663nt      (20369) ACAGAGGCATCTAAGGTCAGGTCTGGTTTTGTGTCTGAGGACATGTT-TGACCTGGGTTATTGCATCTAATTATTTTTC
SARS-CoV-2 Reference Genome NC_045512.2              (19069) GCTGATGTAG--AATGGAAGTTCTA----TGATGCACAGC-CTTGTAGTGACAAAGCTTATAAAATAGAAGAATTATTC
```

Section 265

```
                                                     (20857) 20857        20870     20880     20890     20900     20910     20920        20935
Oryza sativa chromosome 1 region 9.891-41.663nt      (20447) TTTTGCAGGTTGTGCATTCTCCTACAACTATGTCGGCATCAGGACTGAGTATGCTAGTTGAAACATCAATGCCACCTAA
SARS-CoV-2 Reference Genome NC_045512.2              (19141) TATT-CTTATGCCACACATTCTGACAAAT-TCACAG-ATGGTGTATGCCTATT----TTGGAATTGCAATGTCG----A
```

Section 266

```
                                                     (20936) 20936         20950     20960     20970     20980     20990     21000       21014
Oryza sativa chromosome 1 region 9.891-41.663nt      (20526) AAGGGTAATATCAT-CTAACATGCTTTTAAATTGAATAGAGCGACTCATGGCCTATTAAGTGTTAATGCTTACGCTGGG
SARS-CoV-2 Reference Genome NC_045512.2              (19209) TAG----ATATCCTGCTAATTCCATTGTTTGTAGATTTGAC--ACTAGAGTGCTATCTAACCTTAA--CTTGC-CTGGT
```

```
Section 267
                                                  (21015) 21015 21020      21030      21040      21050      21060      21070      21080        21093
Oryza sativa chromosome 1 region 9.891-41.663nt  (20604) AAAGATGAAG-CACTTCGCCTGATGATTAT-AGACATTGGAAATACTAGCTGTTTACAAAGACGATGGTGTATTGACTA
SARS-CoV-2 Reference Genome NC_045512.2          (19279) TGTGATGGTGGCAGTTTGTATGTAAATAAACATGCATTCCACACACCAGCTTTTGATAAA-----------AGTGCTTT

Section 268
                                                  (21094) 21094  21100      21110      21120      21130      21140      21150      21160      21172
Oryza sativa chromosome 1 region 9.891-41.663nt  (20681) TGCTTGAGTGAACACATTAATAGTGAGTCTGCGTTATACTTTCTGGCAATATGCCCGAGCTTTTATCACTTTTCGTTGC
SARS-CoV-2 Reference Genome NC_045512.2          (19347) TG-TTAATTTAAAACAATTACCATTTTTCTA--TTACTCTGACAGTCCATGTGAGTCT-CATGGAAAACAAGTAGTGTC

Section 269
                                                  (21173) 21173   21180      21190      21200      21210      21220      21230      21240      21251
Oryza sativa chromosome 1 region 9.891-41.663nt  (20760) TCGACTAGG---TGTGCCATTGTTGTTACCTTCCAAAACTCTATAGCACTAAATGCAAACCAGAACGTAAGGAGTGTGT
SARS-CoV-2 Reference Genome NC_045512.2          (19422) AGATATAGATTATGTACCACTAAAGTCTGCTACG-----TGTATAACACGT--TGCAATTTAGGTGGT--GCTGTCTGT

Section 270
                                                  (21252) 21252    21260      21270      21280      21290      21300      21310      21320   21330
Oryza sativa chromosome 1 region 9.891-41.663nt  (20836) ATGCCCTAAACTGAGCAGCTTAAGTTGT--CTTAATCCTG----CATGAATCTCCAGGGTGTTTTGAGCCGAGCAAGTT
SARS-CoV-2 Reference Genome NC_045512.2          (19492) AGACATCATGCTAATGAGTACAGATTGTATCTCGATGCTTATAACATGATGATCTCAGCTGGCTTTAGCTTGTGGGTTT

Section 271
                                                  (21331) 21331     21340      21350      21360      21370      21380      21390            21409
Oryza sativa chromosome 1 region 9.891-41.663nt  (20909) CTATGAAAGAAGGATCCCAGGAGCTATCAAATGGGTCATCAAG--TTCAGGGGAACCAACCACTGGGAGG-AATGTTCC
SARS-CoV-2 Reference Genome NC_045512.2          (19571) ACAAACAATTTGATACTTATAACCTCTGGAACACTTTTACAAGACTTCAGAGTTTAGAAAATGTGGCTTTTAATGTTGT

Section 272
                                                  (21410) 21410      21420      21430      21440      21450      21460      21470            21488
Oryza sativa chromosome 1 region 9.891-41.663nt  (20985) AAATTCAAGACTGTTTG--------AGGGTTTGGT----TTTTTGAAGAGTTCTGCCAGTTGAGATACATGGCATTGT
SARS-CoV-2 Reference Genome NC_045512.2          (19650) AAATAAGGGACACTTTGATGGACAACAGGGTGAAGTACCAGTTTCTATCATTAATAACACTGTTTACACAAAAGTTGAT

Section 273
                                                  (21489) 21489      21500      21510      21520      21530      21540      21550            21567
Oryza sativa chromosome 1 region 9.891-41.663nt  (21052) CATGGTTTTACCCAAAGGAGTTGGATTGA----CCTTTACGTGTATATGTATTGCTAGCTACTAGGTGCCAGTAGAAAA
SARS-CoV-2 Reference Genome NC_045512.2          (19729) GGTGTTGATGTAGAATTGTTTGAAAATAAACAACATTACCTGTTAATGTAGCATTTGAGCTTTGGGCTAAGCGCAACA
```

```
                                                                      Section 274
                                                  (21568) 21568       21580     21590     21600     21610     21620     21630            21646
Oryza sativa chromosome 1 region 9.891-41.663nt   (21127) ATATGCAGTTGCCCCATGGCATCACTTTCACCTATT-GGAAGTGCCAATTTTTTGTAGAAATTAATTAATCTATATACC
SARS-CoV-2 Reference Genome NC_045512.2           (19808) TTAAACCAGTACCAGAGGTGAAAATACTCAATAATTTGGGTGTGGACATTGCTGCTAATACTG---TGATCTGGGACTA
                                                                      Section 275
                                                  (21647) 21647        21660     21670     21680     21690     21700     21710          21725
Oryza sativa chromosome 1 region 9.891-41.663nt   (21205) AAATACAAATGCATTGCAG-AGATATACTGTTTGTCGGTTGTGGTTGTAAAAAAAACTCGTAGGGGCAGAAAGAGACA
SARS-CoV-2 Reference Genome NC_045512.2           (19884) CAAAAGAGATGC--TCCAGCACATATATCTACTATTGGTGTTTGTTCTATGACTGACATAGCCAAGAAACCAACTGAAA
                                                                      Section 276
                                                  (21726) 21726         21740     21750     21760     21770     21780     21790        21804
Oryza sativa chromosome 1 region 9.891-41.663nt   (21283) CCTGAT-TGCGTCTTGTTGCAGCAAGTTTGTTTGGGGAG--GATGTACTGGAATAAGGGTATAGTAGTAGTAGGAATTA
SARS-CoV-2 Reference Genome NC_045512.2           (19961) CGATTTGTGCACCACTCACTGTCTTTTTTGATGGTAGAGTTGATGGTCAAGTAGACTTATTTAGAAATGCCCGTAATGG
                                                                      Section 277
                                                  (21805) 21805 21810     21820     21830     21840     21850     21860     21870    21883
Oryza sativa chromosome 1 region 9.891-41.663nt   (21359) TTATGGCACATTTGCATGCTTTGGCATATGGCACTCTGAGTTTTATTGATCTCAGATTTTCGTCACTTTAATATACT-A
SARS-CoV-2 Reference Genome NC_045512.2           (20040) TGTTCTTATTACAGAAGGTAGTGTTAAA-GGTTTACAACCATCTGTAGGTCCCAAACAAGCTAGTCTT-AATGGAGTCA
                                                                      Section 278
                                                  (21884) 21884 21890     21900     21910     21920     21930     21940     21950    21962
Oryza sativa chromosome 1 region 9.891-41.663nt   (21437) CCTCCATCCTAAAATGTTTGA---CGCCATCGAATTTTTTAAAAATGTTTGACCGTCTTATTCAAAAAATTTAAGTAAT
SARS-CoV-2 Reference Genome NC_045512.2           (20117) CATTAATTGGAGAAGCCGTAAAAACACAGTTCAATTATTATAAGAAAGTTGATGGTGTTGTCCAACAATT------ACC
                                                                      Section 279
                                                  (21963) 21963 21970     21980     21990     22000     22010     22020     22030    22041
Oryza sativa chromosome 1 region 9.891-41.663nt   (21513) TGTTAATTCTTTTTCTATCATTTGATTTATTGTTAAATATACTTTTATGTATACATATAGTTTTACACATATCACAAA-
SARS-CoV-2 Reference Genome NC_045512.2           (20190) TGAAACTTACTTTACTCAGAGTAGAAATTTACAAGAATTTAAACCCAGGAGT-CAAATGGAAATTGATTTCTTAGAATT
                                                                      Section 280
                                                  (22042) 22042  22050     22060     22070     22080     22090     22100     22110  22120
Oryza sativa chromosome 1 region 9.891-41.663nt   (21591) AGTTTT---TGAATAAAACGAACGGTCAAACATGTTTAAAAAAGTCAACGGTGTCAAACATTTAGGAAATGAGGGAGTA
SARS-CoV-2 Reference Genome NC_045512.2           (20268) AGCTATGGATGAATTCATTGAACGGTATAAATT------AGAAGGCTATGCCTTCGAACATATCGTTTATG---GAGAT
```

```
                                                                              Section 281
                                                         (22121) 22121    22130     22140     22150     22160     22170     22180                   22199
Oryza sativa chromosome 1 region 9.891-41.663nt (21667) TATAGGAGTATATATACTCCAGGTGTGGTGTGGGTGTAGTACGTATGAAGTATGATGGATTTGTTGTACTTTGGTTTTC
  SARS-CoV-2 Reference Genome NC_045512.2 (20338) TTTAGTC------ATAGTCA--GTTAGGTG---GTTTACATCTACTGAT------TGGACTAGCTAAAC----GTTTTA
                                                                              Section 282
                                                         (22200) 22200     22210     22220     22230     22240     22250     22260                 22278
Oryza sativa chromosome 1 region 9.891-41.663nt (21746) ACGGCGAGCCATCTTGTTGTAGCTGTCGGAGTAGGAGGGCAGCCATATTGATGGATGAAGGTCAAATAATTTGGGCCTA
  SARS-CoV-2 Reference Genome NC_045512.2 (20396) AGG---AATCACCTT-TTGAATTAG-------AAGATTTTATTCCTATGGACAGTACAGTTAAAAACTATTT---CATA
                                                                              Section 283
                                                         (22279) 22279      22290     22300     22310     22320     22330     22340                22357
Oryza sativa chromosome 1 region 9.891-41.663nt (21825) ATCGATGGTGATAC---TCCTACTAAACGCTCGCTCTTCTGTGAGGCAGGCTCCTTTTGTTGACCTGACTGAGTGAGTG
  SARS-CoV-2 Reference Genome NC_045512.2 (20461) ACAGATGCGCAAACAGGTTCATCTAAGTGTGTG-TGTTCTGTTATTGATTTATTACTTGATGATTTTGTTGAAATAATA
                                                                              Section 284
                                                         (22358) 22358       22370     22380     22390     22400     22410     22420               22436
Oryza sativa chromosome 1 region 9.891-41.663nt (21901) ACTTGTGGCCACAAATTAGGTCAATCTTGATAGGCCCAATTACTC-CTATTGCTACTACTGTACATGATTTTCAGTAAC
  SARS-CoV-2 Reference Genome NC_045512.2 (20539) AAATC---CCAAGATTTATCTGTAGTTTCTAAGGTTGTCAAAGTGACTATTG-----ACTATACAGAAATTTCA-----
                                                                              Section 285
                                                         (22437) 22437        22450     22460     22470     22480     22490     22500              22515
Oryza sativa chromosome 1 region 9.891-41.663nt (21979) ATTTTAACATTAATTTATTTATATT-ATTTATTAACAACTAA---AAGGTTAGTAAGTACTTTAAAATACAATCTAG
  SARS-CoV-2 Reference Genome NC_045512.2 (20605) --TTTATGCTTTGGTGTAAAGATGGCCATGTAGAAACATTTTACCCAAAATTACAATCTAGTCAAGCGTGGCAACCGGG
                                                                              Section 286
                                                         (22516) 22516          22530     22540     22550     22560     22570     22580            22594
Oryza sativa chromosome 1 region 9.891-41.663nt (22054) TGTTATAACGCATAATACTTATAATTTTTATTAGTCTAAAGTTAGTGAA-TTTGATTTTCTTTGAAACAAGGACATCGA
  SARS-CoV-2 Reference Genome NC_045512.2 (20682) TGTTGCTATGCCTAATCTTTACAAAATGCAA-AGAATGCTATTAGAAAAGTGTGACCTTCAAAATTATGGTGATAGTGC
                                                                              Section 287
                                                         (22595) 22595 22600     22610     22620     22630     22640     22650     22660            22673
Oryza sativa chromosome 1 region 9.891-41.663nt (22132) ATCATTTGGGACAGAAGTAATAGTATATTACTCCATCCGTCCCCTGATATAAGAGGTTTAATATTTACTTGTACTGT
  SARS-CoV-2 Reference Genome NC_045512.2 (20760) AACATTACCTAAAGGCATAATGATGAATGTCGCAAAATATACTCAACT-------GTGTCAATATTTAA-----ACACA
```

```
                                                          Section 288
                                          (22674) 22674 22680     22690     22700     22710     22720     22730     22740     22752
Oryza sativa chromosome 1 region 9.891-41.663nt (22211) TTGACTATTCGTCTTATTCCAAAAAAATAGAATTATTATTTATTTTATTTGTGACTTACTTTATTATCAAAAGTACTTT
SARS-CoV-2 Reference Genome NC_045512.2 (20827) TTAAC-ATTAGCTGTACCCTATAATATGAGAGTTATACATT------TTGGTG-CTGGTTCTGATAAAGGAGTTGCACC

                                                          Section 289
                                          (22753) 22753 22760     22770     22780     22790     22800     22810     22820     22831
Oryza sativa chromosome 1 region 9.891-41.663nt (22290) AAGCATACCTTTTTGTTTTTTATATTTGCATAATTCTTTTAAATAAGACGAGT-AG-TCCAAAAGTCAAAATCCCT---
SARS-CoV-2 Reference Genome NC_045512.2 (20898) AGGTACAGCTGTTTTAAGACAGTGGTTGCCTACGGGTACGCTGCTTGTCGATTCAGATCTTAATGACTTTGTCTCTGAT

                                                          Section 290
                                          (22832) 22832 22840     22850     22860     22870     22880     22890     22900     22910
Oryza sativa chromosome 1 region 9.891-41.663nt (22364) TTAATTTGAGGACGGAGAGAGAGTATTAGGAGGAGATCTTTTTACACCTAATGAAATCAATGTGAAAATAAAAGGAAA-
SARS-CoV-2 Reference Genome NC_045512.2 (20977) GCAGATTCA--ACTTTGATTGGTGATT--GTGCAACTGTACATACAGCTAATAAATGGGATCTCATTATTAGTGATATG

                                                          Section 291
                                          (22911) 22911 22920     22930     22940     22950     22960     22970     22989
Oryza sativa chromosome 1 region 9.891-41.663nt (22442) TATGCAGCGGAGAATGGGACCCCTTCTA--GAAAGTGGATTTAATACTAGTGTGTGCTCCTACTTGGGAAGTTGGGTGG
SARS-CoV-2 Reference Genome NC_045512.2 (21052) TACGACCCTAAGACTAAAAATGTTACAAAAGAAAATGACTCTAAAGAGGGTTTTTTCACTTACATTTGTGGGTTTATAC

                                                          Section 292
                                          (22990) 22990 23000     23010     23020     23030     23040     23050     23068
Oryza sativa chromosome 1 region 9.891-41.663nt (22519) GATTGGCGTCGTGTTCGTGTAGCTCCTCTTCTCCTCATTGCTGGCTTGGCCTTTGGCTATACCTTCCCTTCATCACCTC
SARS-CoV-2 Reference Genome NC_045512.2 (21131) AACAAAAG-CTAGCTCTTGGAGGTTC-CGTGGCTATAAAGATAACAGAACATTCTTGGAATGCTGATCTTTATAAGCTC

                                                          Section 293
                                          (23069) 23069 23080     23090     23100     23110     23120     23130     23147
Oryza sativa chromosome 1 region 9.891-41.663nt (22598) CTAGTATAAAGCAATTCAATTCCCAATCCCCCCGGCCCGAAGTGAAGGAAGCGGATCATGGAGCTCGCCATGGATTTTG
SARS-CoV-2 Reference Genome NC_045512.2 (21208) ATGGGA----------CACTTCGCATGGTGGACAGCCTTT-GTTACTAATGTGAATGCGTCATCATCTGAAGCATTTTT

                                                          Section 294
                                          (23148) 23148 23160     23170     23180     23190     23200     23210     23226
Oryza sativa chromosome 1 region 9.891-41.663nt (22677) CGCTCCGCCTCCGCGACGCCGCCAACCACCACCTCTCCCGCTACGAGCCTCTCGTGCTCCTCGCCGCGCCCCTCCTCGC
SARS-CoV-2 Reference Genome NC_045512.2 (21276) AATTGGATGTAATTATCTTGGCAAACCACG------CGAACAAATAGATGGTTATG-TCATGCATGCAAATTACATA--
```

Section 295

```
(23227) 23227 23240 23250 23260 23270 23280 23290 23305
Oryza sativa chromosome 1 region 9.891-41.663nt (22756) CCTCCTCGCTGCCCGCACCCTCCACGCCGCCGCTGCCGCCGTCGCCGACCGCGGTC-TCCGCACCGTCCTCCTCGCTCT
SARS-CoV-2 Reference Genome NC_045512.2 (21346) --TTTTGGAGGAATACAAA-TCCAATTCA--GTTGTCTTCCTATTCTTTATTTGACATGAGTAAATTTCCCCTTAAATT
```

Section 296

```
(23306) 23306 23320 23330 23340 23350 23360 23370 23384
Oryza sativa chromosome 1 region 9.891-41.663nt (22834) AGCCATGACAGCCATCAAGTAAGCTTTAATCGACT-TCAATCTCCTCCTCTTTTCTTACTTGTTGCTGCTATTACTATT
SARS-CoV-2 Reference Genome NC_045512.2 (21420) AAGGGGTACTGCTGTTATGT---CTTTAAAAGAAGGTCAAATCAATGATATGATTTTATCTCTTCTTAGTAAAGGTAGA
```

Section 297

```
(23385) 23385 23390 23400 23410 23420 23430 23440 23450 23463
Oryza sativa chromosome 1 region 9.891-41.663nt (22912) ATTATCCGTTCATACATACACACGCAGGTTGCTTCCGGGAGTTAGCGCCTACATCAACGCTGAGAAGAGGAAGGTCAGC
SARS-CoV-2 Reference Genome NC_045512.2 (21496) CTTATAATTAGAGAAAACAACAGAGTTGTTATTTCT---AGTGATGTTCTTGTTAACAACTAA-ACGAACAATGT----
```

Section 298

```
(23464) 23464 23470 23480 23490 23500 23510 23520 23530 23542
Oryza sativa chromosome 1 region 9.891-41.663nt (22991) AGCACACGCATCTGAGTACCTCTTCAATTCTCTCTCTCCTTTTCTTGT-AGTAATCGCTCGCTAGTGGCTTTTATTTTT
SARS-CoV-2 Reference Genome NC_045512.2 (21567) ---------------------------TTGTTTTTCTTGTTTTATTGCCACTAGTCTCTAGTCAGTG--TGTTAATCTT
```

Section 299

```
(23543) 23543 23550 23560 23570 23580 23590 23600 23610 23621
Oryza sativa chromosome 1 region 9.891-41.663nt (23069) AAAATGAAAAATAAAAATACTTTCTTTTCTACTCTACTCATTTTTTTACATATGCATATGCCTACCCTACATATGTTAA
SARS-CoV-2 Reference Genome NC_045512.2 (21617) ACAACCAGAACTCAAT-TACCCCCTGCA-TACACTA---ATTCTTTCACACGTGGTGTTTATTACCCT------GACAA
```

Section 300

```
(23622) 23622 23630 23640 23650 23660 23670 23680 23690 23700
Oryza sativa chromosome 1 region 9.891-41.663nt (23148) ATTTTTTGCTTCATCTTTTTTTTTCTTTCCTATGGGAATGCTC---CGTGGGTATTCAGATGCTACGTTCGTTCAGTTT
SARS-CoV-2 Reference Genome NC_045512.2 (21685) AGTTTTCAGATCCTCAGTTTTACATTCAACTCAGGACTTGTTCTTACCTTTCTTTTCCAATGTTAC-TTGGTTCCATGC
```

Section 301

```
(23701) 23701 23710 23720 23730 23740 23750 23760 23779
Oryza sativa chromosome 1 region 9.891-41.663nt (23224) GACATATGCAGCCGCGCGCATTAACTGGGAATGCTCGCGTGCACGCAACTCAGCCAGGCGTACCGTCTCTCTTATGATT
SARS-CoV-2 Reference Genome NC_045512.2 (21763) TATACATGTCTCTGGGACCAAT----GGTACTAAGAGGTTTGA------TAACCCTGTCCTACCATTTA----ATGATG
```

```
                                                        Section 302
                                              (23780) 23780     23790     23800     23810     23820     23830     23840              23858
Oryza sativa chromosome 1 region 9.891-41.663nt (23303) GTGAATGCCATTGTGAATGCGATTGATTTAAGACCATGTTCATGTCAAAACAAAAATAAAAAAAAGTACTTCTACCTAC
SARS-CoV-2 Reference Genome NC_045512.2        (21828) GTGTTT---ATTTTGCTTCC-ACTGAG--AAGTCTAACATAATA--AGAGGCTGGATTTTTGGTACTACTT-TAGATTC

                                                        Section 303
                                              (23859) 23859      23870     23880     23890     23900     23910     23920             23937
Oryza sativa chromosome 1 region 9.891-41.663nt (23382) ATATCCCCGGATATCTCATTATTGGTGCTATA-CTTATATTGTAACAGGTTGTTGACCAGCTGCAGTCAGGTGGCACGT
SARS-CoV-2 Reference Genome NC_045512.2        (21898) GAAGACCCAG-TCCCTACTTATTGTTAATAACGCTACTAATGTT----GTTATTAA--------AGTC---TGTGAATT

                                                        Section 304
                                              (23938) 23938       23950     23960     23970     23980     23990     24000           24016
Oryza sativa chromosome 1 region 9.891-41.663nt (23460) CCACAAAAAGTACTCTGCGGACCGAGTTGCCAACTGTTGGGCTTTCCAACCAAGTGATAAACGATCTCGAAACACTGAA
SARS-CoV-2 Reference Genome NC_045512.2        (21961) TCA-ATTTTGTAAT-----GATCCATTTTTGGG-TGTTTA--TTACCACAAAAACAACAAAAGTTGGATGGAAAGTGAG

                                                        Section 305
                                              (24017) 24017        24030     24040     24050     24060     24070     24080          24095
Oryza sativa chromosome 1 region 9.891-41.663nt (23539) AGCTAGAGATGTAAACTGGCAGGGAAAATG---CTCTGGCACAGTGTATGTGCATCCCATTAATTCTCACACAAAATAA
SARS-CoV-2 Reference Genome NC_045512.2        (22031) TTC-AGAGTT-TATTCTAGTGCGAATAATTGCACTTTTGAATA-TGTCTCT-CAGCCTTTTCTTATGGACCTTGAAGGA

                                                        Section 306
                                              (24096) 24096         24110     24120     24130     24140     24150     24160         24174
Oryza sativa chromosome 1 region 9.891-41.663nt (23615) ACCCAGTAAATTAAACGGTTCAATCATTCTATTGTGGTTCTTTTCGTTATGCTGTACACTCTACACATATTACTAGCAT
SARS-CoV-2 Reference Genome NC_045512.2        (22106) AAACAGGGTAAT------TTCAAAAATCTTA----GGGAATTTGTGTT-------------TAAGAATATTGATGGTTA

                                                        Section 307
                                              (24175) 24175 24180     24190     24200     24210     24220     24230     24240      24253
Oryza sativa chromosome 1 region 9.891-41.663nt (23694) TCCGCATGTCAAATAACTTTTTTTTTAATATTGCAGTTATATTGCTGGGAGTGAATCCGAAGGTCATTTTGCATTGATA
SARS-CoV-2 Reference Genome NC_045512.2        (22162) T-----TTTAAAATA--TATTCTAAGCACACGCCTATTAATTTAGTGCGTGATCTCCCTCAGGGTTTTTCGGCTTTAGA

                                                        Section 308
                                              (24254) 24254  24260     24270     24280     24290     24300     24310     24320    24332
Oryza sativa chromosome 1 region 9.891-41.663nt (23773) AACAAGGCTTACTCTATGTAAGACTTGGGATTT---TGACCCTGTCTCAATCTCTATTTAAATAGAATATATAGACAAT
SARS-CoV-2 Reference Genome NC_045512.2        (22234) ACCA----TTGGTAGATTTGCCAATAGGTATTAACATCACTAGGTTTCAAACTTTACTTGCTT----TACATAGA-AGT
```

Section 309

	(24333)	24333 24340 24350 24360 24370 24380 24390 24400 24411
Oryza sativa chromosome 1 region 9.891-41.663nt	(23849)	AATTGGTCACCATGTTTTCCAATAGGAGGGCGACTTAAGAAAGTATATAATAAAGTGTATTTATTGTTTTATTTATGCT
SARS-CoV-2 Reference Genome NC_045512.2	(22304)	TATTTGACTCCTGGTGATTCTTCTTCAGGTTG------GACAGCTGGTGCTGCAGCTTATTATGTGGGTTATCTTCAAC

Section 310

	(24412)	24412 24420 24430 24440 24450 24460 24470 24480 24490
Oryza sativa chromosome 1 region 9.891-41.663nt	(23928)	CTGTTGCGTTGGTGGAAGAAAAAAAGTAATTTGCATAATTTTATTTTTTTCCCTAGGGAATACCGTCATAAAATTCATG
SARS-CoV-2 Reference Genome NC_045512.2	(22377)	CTAGGACTTTTCTATTAAAATATAATGAAAATGGAA--------------CCATTACAGATGCTGT-AGACTGTGCACT

Section 311

	(24491)	24491 24500 24510 24520 24530 24540 24550 24569
Oryza sativa chromosome 1 region 9.891-41.663nt	(24007)	TCAGCCTGTCTCAACTATCAAGATAAATAGTCCACTAGATTACTTCAAATGTATAGTAGGGAAAC-AT--ATGTTCT--
SARS-CoV-2 Reference Genome NC_045512.2	(22441)	TGACCCTCTCTCAG------AAACAAAGTGTACGTTGAAATCCTTCAC-TGTAGAAAAAGGAATCTATCAAACTTCTAA

Section 312

	(24570)	24570 24580 24590 24600 24610 24620 24630 24648
Oryza sativa chromosome 1 region 9.891-41.663nt	(24081)	-TCCAGATTCAAACCAAGTGCCTCTGTTAGTAT-TTTGGTGAATTGACTTATTTATAACTCATATTTCAAAGTTACCAT
SARS-CoV-2 Reference Genome NC_045512.2	(22513)	CTTTAGAGTCCAACCAACAGAATCTATTGTTAGATTTCCTAATATTACAAACTTGTGCC-CTTTTGGTGAAGTT----T

Section 313

	(24649)	24649 24660 24670 24680 24690 24700 24710 24727
Oryza sativa chromosome 1 region 9.891-41.663nt	(24158)	GTAGTCCCTCCATCCCAATTGATCATCATATATATTTATGCACCAAGACCAAA----GATAATTTAAATCACATCAATC
SARS-CoV-2 Reference Genome NC_045512.2	(22587)	TTAACGCCACCAG---ATTTG--CATC-TGTTTATGCTTGGAACAGGAAGAGAATCAGCAACTGTGTTGCTGATTATTC

Section 314

	(24728)	24728 24740 24750 24760 24770 24780 24790 24806
Oryza sativa chromosome 1 region 9.891-41.663nt	(24233)	GAGACACCATG----CACA-CATTCTCCCCACATACATGC-ATCGATTAAAACATCATGCCAATTACATCCT-TAGCAT
SARS-CoV-2 Reference Genome NC_045512.2	(22660)	TGTCCTATATAATTCCGCATCATTTTCCACTTTTAAGTGTTATGGAGTGTCTCCTACTA--AATTAAATGATCTCTGCT

Section 315

	(24807)	24807 24820 24830 24840 24850 24860 24870 24885
Oryza sativa chromosome 1 region 9.891-41.663nt	(24305)	GTACACAT-TCTCCCTTGCTACATT---AATTGTATTCTGATGAAACCCGTGTCCATGCGGTTATGTAATGATCAATTT
SARS-CoV-2 Reference Genome NC_045512.2	(22737)	TTACTAATGTCTATGCAGATTCATTTGTAATTAGAGG-TGATGAAGTCAGA--CAAATCGCTCCAG----GGCAAACTG

```
                                                              Section 316
                                                      (24886) 24886         24900     24910     24920     24930     24940     24950           24964
Oryza sativa chromosome 1 region 9.891-41.663nt        (24380) GAGAAATTTTGGATT-TCATTATATGATGATCAATT--TGGGAAGGAGGGAGTAACTTTTTCTAGTTTTTATAGGCCTC
SARS-CoV-2 Reference Genome NC_045512.2                (22809) GAAAGATTGCTGATTATAATTATAA-ATTACCAGATGATTTTACAGGCTGCGTTATAGCTTGGAATTCTAACAATCTTG
                                                              Section 317
                                                      (24965) 24965 24970     24980     24990     25000     25010     25020     25030           25043
Oryza sativa chromosome 1 region 9.891-41.663nt        (24456) CATCTAATTTTAGGC----TTCTAATTTACCATAAATATTCTTAATCCTTTC-AGGTTTTCACAC----ACCAACCCAC
SARS-CoV-2 Reference Genome NC_045512.2                (22887) ATTCTAAGGTTGGTGGTAATTATAATTACCTGTATAGATTGTTTAGGAAGTCTAATCTCAAACCTTTTGAGAGAGATAT
                                                              Section 318
                                                      (25044) 25044 25050     25060     25070     25080     25090     25100     25110           25122
Oryza sativa chromosome 1 region 9.891-41.663nt        (24526) TTCACCAGGACGTATTCAAGAGTGTGGCACAATTAG--AGGCTGAAGTAGTTGCAATGACAGCTGCTTTGCTTGGCA-T
SARS-CoV-2 Reference Genome NC_045512.2                (22966) TTCAACTGAAATCTATCAGGCCGGTAGCACACCTTGTAATGGTGTTGAAGGTTTTA--ATTGTTACTTTCCTTTACAAT
                                                              Section 319
                                                      (25123) 25123 25130     25140     25150     25160     25170     25180     25190           25201
Oryza sativa chromosome 1 region 9.891-41.663nt        (24602) CAAAGAAAAATCATCCGGTGGACAGATTTGTGGTAATATGACATCGGGCGGAACTGAAAGCATATTATTAGCAGTAAAA
SARS-CoV-2 Reference Genome NC_045512.2                (23043) CATATGGTTTCCAACCCACTAATGGTGTTG--GTTACCAACCATACAGAGTAGTAGTACTTTCTTTTGAACTTCTACAT
                                                              Section 320
                                                      (25202) 25202 25210     25220     25230     25240     25250     25260     25270           25280
Oryza sativa chromosome 1 region 9.891-41.663nt        (24681) ACATCACGCGACTATATGCGAACAAAGAAGGGCATTACAAAGCCTGAAATGTGAGATAAATGATGGATTATGTTATGCA
SARS-CoV-2 Reference Genome NC_045512.2                (23120) GCACCA-GCAACTGTTTGTGGACCTAAAAAGTC--TACTAATTTGGTTAA---AAACAAATGTGTCAATTTCAACTTCA
                                                              Section 321
                                                      (25281) 25281 25290     25300     25310     25320     25330     25340                     25359
Oryza sativa chromosome 1 region 9.891-41.663nt        (24760) TTGTGATCACCTTATCTCCATGGTAATAAAGGCTTGTGATACAGGATAATTGCCGAATCAGCACATTCTGCATACGATA
SARS-CoV-2 Reference Genome NC_045512.2                (23193) ATGGTTTAACAGG--CACAGGTGTTCTTACTGAGTCTAACAAAAAGTTTCTGCCTTTCCAACA-ATTTGGCAGA-GACA
                                                              Section 322
                                                      (25360) 25360 25370     25380     25390     25400     25410     25420                     25438
Oryza sativa chromosome 1 region 9.891-41.663nt        (24839) AAGCTGCTCAATATTTCAATATTAAAGTGCGGCGTGTGCCAGTGAACAAAGAGTTTCTTGCAGATGTGAAAGGATTCAA
SARS-CoV-2 Reference Genome NC_045512.2                (23268) TTGCTGA---------CACTACTGATGCTGTCCGTGATCCACAGACACTTGAGATTCTTG-ACATTACACCATGTTCTT
```

Section 323

(25439) 25439 25450 25460 25470 25480 25490 25500 25517

Oryza sativa chromosome 1 region 9.891-41.663nt (24918) AAGGTGTATAAACGGAAATACGATAATGGTGAGAT---TTTATCCATATTCCTGTTTCTCTAGTTAGCTTAAAGCTGTT

SARS-CoV-2 Reference Genome NC_045512.2 (23337) TTGGTGGTGTCAGTGTTATAACACCAGGAACAAATACTTCTAACCAGGTTGCTGTT-CTTTATCAGGATGTTAACTGCA

Section 324

(25518) 25518 25530 25540 25550 25560 25570 25580 25596

Oryza sativa chromosome 1 region 9.891-41.663nt (24994) CTGATGCCTCCATTC----ACCTG-AGATGAAAGATATTTGCATTTGTCTTTCCTAAGGAAAGATTAGTATGTACCTT

SARS-CoV-2 Reference Genome NC_045512.2 (23415) CAGAAGTCCCTGTTGCTATTCATGCAGATCAACT-TACTCCTACTTGGCGTGTTTATTCTACAGGTTCTAATGTTT-TT

Section 325

(25597) 25597 25610 25620 25630 25640 25650 25660 25675

Oryza sativa chromosome 1 region 9.891-41.663nt (25068) CTGACCCTGTGACCTTTTATAAGGAAAGATTAGTCTTTACCTTTACCTTCTTCCTACTCTTACATTTCTTAGTGTACAT

SARS-CoV-2 Reference Genome NC_045512.2 (23492) CAAACACGTGCAGGCTGTTTAATAGGGGCTGAACATGT-CAACAAC--TCATATGAGTGTGACATACCCATTGGTGCAG

Section 326

(25676) 25676 25690 25700 25710 25720 25730 25740 25754

Oryza sativa chromosome 1 region 9.891-41.663nt (25147) GTAAATATGTAAATGTTC-----CCTAACAATTTTCT--GTTGCACAGATGGTGG-GGTCTGCA--CCAGGATTTCCT-

SARS-CoV-2 Reference Genome NC_045512.2 (23568) GTATATGCGCTAGTTATCAGACTCAGACTAATTCTCCTCGGCGGGCACGTAGTGTAGCTAGTCAATCCATCATTGCCTA

Section 327

(25755) 25755 25760 25770 25780 25790 25800 25810 25820 25833

Oryza sativa chromosome 1 region 9.891-41.663nt (25215) CATGGT-TTAATTGATCCT--AT-TGAGGTATGTTAATCAATCATCGTTATGT---TAAAGAAAACCAAAAATATT--T

SARS-CoV-2 Reference Genome NC_045512.2 (23647) CACTATGTCACTTGGTGCAGAAAATTCAGTTGCTTACTCTAATAACTCTATTGCCATACCCACAAATTTTACTATTAGT

Section 328

(25834) 25834 25840 25850 25860 25870 25880 25890 25900 25912

Oryza sativa chromosome 1 region 9.891-41.663nt (25285) TTTCCTACTGAAATTGTGC-ACTG-CAAGTTCAGTGATTTCTTGTATCCCGGATCATCTTCAGGAACTTGGGGAGTTGG

SARS-CoV-2 Reference Genome NC_045512.2 (23726) GTTACCACAGAAATTCTACCAGTGTCTATGACCAAGACATCA-GTAGATTGTACAATGTACATTTG--TGGTGATTCAA

Section 329

(25913) 25913 25920 25930 25940 25950 25960 25970 25980 25991

Oryza sativa chromosome 1 region 9.891-41.663nt (25362) CTTCACGATATGACATTTGCTTGCATGTTGATCTCTGCCTTGGTGGATTTGTATTGCCTTTCGCTCGTAAGCTTGGGTA

SARS-CoV-2 Reference Genome NC_045512.2 (23802) CTGAATGCAGCAATCTTTTGTTGCAATATGG---CAGTTTTTGTACACAATTAAACCGT---GCT--TTAACTGGAATA

```
                                                                                  Section 330
                                                   (25992) 25992   26000     26010     26020     26030     26040     26050     26060  26070
Oryza sativa chromosome 1 region 9.891-41.663nt    (25441) CCTACTTACTTCATAACATGATATCTTACCATTATTATTAGCACTTG---AGCAAATTTTGAATAGCTCGCCAATATAC
SARS-CoV-2 Reference Genome NC_045512.2            (23873) GCTGTTGA----ACAAGACAAAAACACCCAAGAAGTTTTTGCACAAGTCAAACAAATTTACAAAACACCACCAATTAAA
                                                                                  Section 331
                                                   (26071) 26071    26080     26090     26100     26110     26120     26130           26149
Oryza sativa chromosome 1 region 9.891-41.663nt    (25517) ACATTTAATCATTTTC---TTACAGCAACATGATGGTTTGCCTATTAATGATTGCTTTAATTTCAATAGAGGAGGTCTC
SARS-CoV-2 Reference Genome NC_045512.2            (23948) GATTTTGGTGGTTTTAATTTTTCACAAATATTACCAGATCCATCAAAACCAA-GCAAGAGGTCATTTATTGAAGATCT-
                                                                                  Section 332
                                                   (26150) 26150     26160     26170     26180     26190     26200     26210           26228
Oryza sativa chromosome 1 region 9.891-41.663nt    (25593) AAACTGTCTACATAA-GCAATTTGTTGATACCTGTATTATATTGCTTTTCACCTATAATCTTGGG--TATTCCTTCATG
SARS-CoV-2 Reference Genome NC_045512.2            (24025) -ACTTTTCAACAAAGTGACACTTGCAGATGCTGGCTTCATCAAACAATATGGTGATTGCCTTGGTGATATTGCTGCTAG
                                                                                  Section 333
                                                   (26229) 26229      26240     26250     26260     26270     26280     26290         26307
Oryza sativa chromosome 1 region 9.891-41.663nt    (25669) ACCATTCATTTGTTCCC-----TTTATCTGGATTTACT-------CGACTGTTCTCAGATGATGTTCTGATTTCTTATC
SARS-CoV-2 Reference Genome NC_045512.2            (24103) AGACCTCATTTGTGCACAAAAGTTTAAC-GGCCTTACTGTTTTGCCACCTTTGCTCACA-GATGAAATGATTGCTCAAT
                                                                                  Section 334
                                                   (26308) 26308       26320     26330     26340     26350     26360     26370        26386
Oryza sativa chromosome 1 region 9.891-41.663nt    (25736) AGTAATTTCAGCATTTGCATGAACTTTGAAGAACT--TGCCAGAACATTACGTAATATTCACTTTTTTAATCATGTAAC
SARS-CoV-2 Reference Genome NC_045512.2            (24180) AC--ACTTCTGCACTGTTA-GCGGGTACAATCACTTCTGGTTGGACCTTTGGTGCAGGTG-CTGCATTA--CAAATACC
                                                                                  Section 335
                                                   (26387) 26387        26400     26410     26420     26430     26440     26450       26465
Oryza sativa chromosome 1 region 9.891-41.663nt    (25813) ATGATGGTTTGCCTGTTAATGATTGTTTTAATTTTGTCCCGAACTGCCACCAACTGTGAACTA--AAAACCCAAAGGTT
SARS-CoV-2 Reference Genome NC_045512.2            (24253) ATT-TGCTATGCAAATGGCTTATAGGTTTAATGGTATT--GGAGTTACACAGAATGTTCTCTATGAGAACCAAAAATTG
                                                                                  Section 336
                                                   (26466) 26466         26480     26490     26500     26510     26520     26530      26544
Oryza sativa chromosome 1 region 9.891-41.663nt    (25890) ATGGCC-----ATGTATAACCACATATGGAACAAGTGTGTGCTTACTATCGCAATACCCAAGCTAATGTGAAGTGAACA
SARS-CoV-2 Reference Genome NC_045512.2            (24329) ATTGCCAACCAATTTAATAGTGCTATTGGCAAAATTCAAGACTCACTTTC----TTCCACAGCAA--GTGCACTTGGAA
```

Section 337

```
                                              (26545) 26545 26550          26560          26570          26580          26590          26600          26610        26623
Oryza sativa chromosome 1 region 9.891-41.663nt (25964) AATATGCAAATTTTTGCTAATCCCTAGTCATAGATCAGGTCTGATGAATCTGG----ACTGGTTTAAAGCAATCTTATG
SARS-CoV-2 Reference Genome NC_045512.2 (24402) AACTTCAAGATGT--GGTCAACCAAA---ATGCACAAGCTTTAAACACGCTTGTTAAACAACTTAGCTCCAATTTTGGT
```

Section 338

```
                                              (26624) 26624 26630          26640          26650          26660          26670          26680          26690        26702
Oryza sativa chromosome 1 region 9.891-41.663nt (26039) ATAGTTACTTATCTTAATGTTCATTTATGTCCCATTTATTGGTTTTAGATACTTGAAGTATATGTCTCTTTTGTTAGAA
SARS-CoV-2 Reference Genome NC_045512.2 (24476) GCAATTTCAAGTGTT----TTAAATGATATCC--TTTCACGTCTTGACAAAGTTGAGGCTGAAGTGCAAATTGATAGGT
```

Section 339

```
                                              (26703) 26703 26710          26720          26730          26740          26750          26760          26770        26781
Oryza sativa chromosome 1 region 9.891-41.663nt (26118) ATGATGTGTTATA-ACTTCAATCATATATGGCATCCATTT-TGTCAAGGCCATTG-TTACATCT-CTGAT---TGAACT
SARS-CoV-2 Reference Genome NC_045512.2 (24549) -TGATCACAGGCAGACTTCAA----AGTTTGCAGACATATGTGACTCAACAATTAATTAGAGCTGCAGAAATCAGAGCT
```

Section 340

```
                                              (26782) 26782 26790          26800          26810          26820          26830          26840          26850        26860
Oryza sativa chromosome 1 region 9.891-41.663nt (26190) AATGTTATGTGTACTAATTCTATGACTTCTCTGCAA-TACAGGTACCCTATTCCTCCATTCGACTTCTGTGTCAAGGGA
SARS-CoV-2 Reference Genome NC_045512.2 (24623) TCTGCTAATCTTGCTGCTACTAAAATGTCAGAGTGTGTACTTGGACAATCAAAAAGAGTT-GATTTTTGTGGAAAGGGC
```

Section 341

```
                                              (26861) 26861 26870          26880          26890          26900          26910          26920                      26939
Oryza sativa chromosome 1 region 9.891-41.663nt (26268) GTTACATCAATCTCAACTGATGTTCATAAGTATGGATTAGCCCCAAAAGGGACAAGCATTGTCCTATATAAAAATCATG
SARS-CoV-2 Reference Genome NC_045512.2 (24701) ----------TATCATCTTATGTCCTTCCCTCAG--TCAGCACCTCATGGTGTAGTCTTCTTGC---ATGTGACTTATG
```

Section 342

```
                                              (26940) 26940 26950          26960          26970          26980          26990          27000                      27018
Oryza sativa chromosome 1 region 9.891-41.663nt (26347) AAATTAGAAAGGTAAAAATAGCCTTGGAGTATTTTTCTCTTTACTTTTCTCA-GATATTCTAACATTATTCCCTCCATC
SARS-CoV-2 Reference Genome NC_045512.2 (24765) TCCCTGCACAAG--AAAAGAACTTCACAAC----TGCTCCTGCCATTTGTCATGATGGAAAAGCACACTTTCCTCG-TG
```

Section 343

```
                                              (27019) 27019 27030          27040          27050          27060          27070          27080                      27097
Oryza sativa chromosome 1 region 9.891-41.663nt (26425) TATTTGGCACTGGCATCTTGGATACTAC-CACCAAATTC-AGCATCAATTTGTTGCTGGTAAGCTTCAATCATTTCCTA
SARS-CoV-2 Reference Genome NC_045512.2 (24837) AAGGTGTCTTTG---TTTCAAATGGCACACACTGGTTTGTAACACAAAGGAATTTTTATGAACCACAAATCATTACTAC
```

```
                                                     Section 344
                                                (27098) 27098       27110      27120      27130      27140      27150      27160       27176
Oryza sativa chromosome 1 region 9.891-41.663nt (26502) TGA-AACTTCTCTCT-TGAGTAAACCTTTTTAAGGGGTTCTCACTTCTCAATAAAA----TTTTGA-CCTCT---ATAT
SARS-CoV-2 Reference Genome NC_045512.2         (24913) AGACAACACATTTGTGTCTGGTAACTGTGATGTTGTAATAGGAATTGTCAACAACACAGTTTATGATCCTTTGCAACCT

                                                     Section 345
                                                (27177) 27177        27190      27200      27210      27220      27230      27240       27255
Oryza sativa chromosome 1 region 9.891-41.663nt (26571) TTATTCGACAGATATCATGGTTGTCGTTACATAACTATTACAGCTTAACAGATATGATCATGTTGCAAAACTGCACGAA
SARS-CoV-2 Reference Genome NC_045512.2         (24992) GAATTAGACTCAT-TCAAGGAGGA-GTTAGATAAATATT-----TTAAGA------ATCAT--------ACATCACCAG

                                                     Section 346
                                                (27256) 27256         27270      27280      27290      27300      27310      27320      27334
Oryza sativa chromosome 1 region 9.891-41.663nt (26650) AATGTTCAAACAGGGTAGAATATTTCTTACATGATTATGGTAACTTTGGTTGGAAGAACGTAGATTCATGCAATGCAAA
SARS-CoV-2 Reference Genome NC_045512.2         (25050) A-TGTTGATTTAGGTGACA---TCTCTGGCATTA--ATG----CTTCAGTTGTAA------ACATTCAA--AAAGAAAT

                                                     Section 347
                                                (27335) 27335 27340      27350      27360      27370      27380      27390      27400      27413
Oryza sativa chromosome 1 region 9.891-41.663nt (26729) TGGTAGTTTGCGTCCTAGTAGCCCTTGGCTTACTTTCCACTATCTGACATGAGCAACGAATCTTGAACCAAAGTTTGAA
SARS-CoV-2 Reference Genome NC_045512.2         (25111) TGACCGCCTCAATGAG-GTTGCCAAGAATTTAAATGAATCTCTCATCGATCTCCAA-GAA-CTTGGA---AAGTATGAG

                                                     Section 348
                                                (27414) 27414 27420      27430      27440      27450      27460      27470      27480      27492
Oryza sativa chromosome 1 region 9.891-41.663nt (26808) TAGTGGTTTAATGTGGGGATTTTGCTTCCTTATTTTTTTTCAGTTACTGAATGGACTGGTGGGCTCTATGTTTCACCTA
SARS-CoV-2 Reference Genome NC_045512.2         (25184) CAGTA-TATAAAATGGCCATGGTACATTTGGCTAGGTTTTA--TAGCTGGCTTGATTGCCATAGTAATGGTGACA---A

                                                     Section 349
                                                (27493) 27493  27500      27510      27520      27530      27540      27550      27560     27571
Oryza sativa chromosome 1 region 9.891-41.663nt (26887) CTATTGCTGGAAGTAGGCCTGGTGGGTTGATAGCGGGAGCTTGGGCTGCTATGACGTCTCTTGGACTGAACGGTAACAG
SARS-CoV-2 Reference Genome NC_045512.2         (25257) TTATGCTTTGCTGTATGACCAGTTGCTG--TAGTTGTCTCAAGGGCTGTTGT----TCTTGTGGATCCTGCTGCAAAT-

                                                     Section 350
                                                (27572) 27572   27580      27590      27600      27610      27620      27630      27640   27650
Oryza sativa chromosome 1 region 9.891-41.663nt (26966) GTTTATTCAGAAGTTGACCAGTCCAGTGGT-AGTTTAGTAGTGGAACAGTATTTCTAATGGATACTTGTGCTTGGTTTC
SARS-CoV-2 Reference Genome NC_045512.2         (25329) -TTGATGAAGACGACTCTGAG-CCAGTGCTCAAAGGAGTCAAATTACATTACACATAAACGA-ACTTATGGATTTGTTT
```

Section 351

(27651) 27651 27660 27670 27680 27690 27700 27710 27729
Oryza sativa chromosome 1 region 9.891-41.663nt (27044) CAGGTTATATGGAAAATACAGGTCATATCATGGAAGTGTCGAAGAAAATACAAAGAGGGTAAACTTGCCTGTTTATCAT
SARS-CoV-2 Reference Genome NC_045512.2 (25405) ATGAGAATCTTCACAATTGGAACTGTAACTTTGAAG---CAAGGTGAAATCAAGGATGCTACTCCTTCAGATTT-----

Section 352

(27730) 27730 27740 27750 27760 27770 27780 27790 27808
Oryza sativa chromosome 1 region 9.891-41.663nt (27123) CCTTGGAAGC-CTAGAGTATAAACATATATA-AAGACTGACTACTGTGGTGTTTTGGTATCAGTTAGTTTGGGAAAT-T
SARS-CoV-2 Reference Genome NC_045512.2 (25476) ---TGTTCGCGCTACTGCAACGATACCGATACAAGCCTCACTCCCTTTCGGATGGCTTATT-GTTGGCGTTGCACTTCT

Section 353

(27809) 27809 27820 27830 27840 27850 27860 27870 27887
Oryza sativa chromosome 1 region 9.891-41.663nt (27199) TTTTGAACAGAAGTGCCATTCACATCAATATTATTCCTCATAATTGTAGGGATGCACATTGTGTATAGTCAAATG-GCA
SARS-CoV-2 Reference Genome NC_045512.2 (25551) TGCTGTTTTTCAGAGCGCTTCCAA--AATCATAACCCTCAAAA----AGAGATGGCAACTAGCACTCTCCAAGGGTGTT

Section 354

(27888) 27888 27900 27910 27920 27930 27940 27950 27966
Oryza sativa chromosome 1 region 9.891-41.663nt (27277) AACTTTAGATGCCCTTTCATGTAGATGTGCTGCATCTGAATTCTTGAGAGCACGTGTTTAAAGAGAGAAATGTA-TTGA
SARS-CoV-2 Reference Genome NC_045512.2 (25624) CACTTTGTTTGCAACTTGCTGTTGTTGTT-TGTAACAGTTTACTCA----CACCTTTTGCTCGTTGCTGCTGGCCTTGA

Section 355

(27967) 27967 27980 27990 28000 28010 28020 28030 28045
Oryza sativa chromosome 1 region 9.891-41.663nt (27355) ATGCA--TTGCGCAGGATTGAAGATATCCCAGGGTTGTTTGTGATTGGAAAGCCAGACATGACTGTCGTG-GCATTTGG
SARS-CoV-2 Reference Genome NC_045512.2 (25698) AGCCCCTTTTCTCTATCTTTATGCTTTAGTCTACTTCTTGCAGAGTATAAACTTTGTAAGAATAATAATGAGGCTTTGG

Section 356

(28046) 28046 28060 28070 28080 28090 28100 28110 28124
Oryza sativa chromosome 1 region 9.891-41.663nt (27431) CTCAGATTCGGTTGATATATTTGAGGTGAACGAC---ATAATGTCATCAAAAG-----GCTGGCACCTTAATGCT--CT
SARS-CoV-2 Reference Genome NC_045512.2 (25777) CTTTGCTGGAAATGCCGTTCCAAAAACCCATTACTTTATGATGCCAACTATTTTCTTTGCTGGCATACTAATTGTTACG

Section 357

(28125) 28125 28130 28140 28150 28160 28170 28180 28190 28203
Oryza sativa chromosome 1 region 9.891-41.663nt (27500) GCAGAGACCCAACAGGTAAAACAAAAACATAGTCTGGTTTCAGA--TTCAGACAAATAACCATAGTGTAACGTATTTAT
SARS-CoV-2 Reference Genome NC_045512.2 (25856) ACTATTGTATACCTTACAATAGTGTAACTTCTTCAATTGTCATTACTTCAGGTGATGGCACAACAAGT--CCTATTTCT

Section 358

```
                                                    28204 28210     28220     28230     28240     28250     28260     28270     28282
                                           (28204)
Oryza sativa chromosome 1 region 9.891-41.663nt  (27577) GTACGAGTGTAGCCTGACGATGTTTTTT--------TCTGTTTCAGTT-TACACATTTGTGTGACACTGCAGCACACGG
SARS-CoV-2 Reference Genome NC_045512.2 (25933) GAACATGACTACCAGATTGGTGGTTATACTGAAAAATGGGAATCTGGAGTAAAAGACTGTGTTGTATTACACAGTTACT
```

Section 359

```
                                                    28283 28290     28300     28310     28320     28330     28340     28350     28361
                                           (28283)
Oryza sativa chromosome 1 region 9.891-41.663nt  (27647) TCATATATGAGGAATTCCTGAAGGA-TCTGAAAGATTCAGTGGACACTGTGAGTTACCAACATATG-CATGTTTTGTCT
SARS-CoV-2 Reference Genome NC_045512.2 (26012) TCACTTCAGACTATTACCAGCTGTACTCAACTCAATTGAGTACAGACACTG-GTGTTGAACATGTTACCTTCTTCATCT
```

Section 360

```
                                                    28362 28370     28380     28390     28400     28410     28420     28430     28440
                                           (28362)
Oryza sativa chromosome 1 region 9.891-41.663nt  (27724) TGA-TAGAGTAGTATATGATTGTAGTGAAGCTA-CTAAGGTAACTAATTTTTCTGTGTAAATTGCGATGCTGTAGGTAA
SARS-CoV-2 Reference Genome NC_045512.2 (26090) ACAATAAAATTGTTGATGAGCCTGAAGAACATGTCCAAATTCACACAATCGACGGTTCATCCGGAGTTGTTAATCCAGT
```

Section 361

```
                                                    28441 28450     28460     28470     28480     28490     28500               28519
                                           (28441)
Oryza sativa chromosome 1 region 9.891-41.663nt  (27801) AAGCAAATCCAGGGCCTATTAGCGGGGGGCGAGCTCCGATTTACGGAGCTGCTGGCAAGATGCCG--GACAGAGGCATG
SARS-CoV-2 Reference Genome NC_045512.2 (26169) AATGGAACCAATTTATGATGAACCGACGACGACTACTAGCGTGCCTTTGTAAGCACAAGCTGATGAGTACGAACTTATG
```

Section 362

```
                                                    28520     28530     28540     28550     28560     28570     28580               28598
                                           (28520)
Oryza sativa chromosome 1 region 9.891-41.663nt  (27878) GTCAGGGAGCTGCTGGTGGAGTTCATGGACGCGTCTTGCTGAACATATAATATATCCCCTGTTCTTGTTATTGTGGCAT
SARS-CoV-2 Reference Genome NC_045512.2 (26248) TACTCATTCGTTTCGGAAGAGA-CAGGTACGTTAATAGTTAA-----TAGCGTACTTCTTTTTCTTGCTTTCGTGGTAT
```

Section 363

```
                                                    28599      28610     28620     28630     28640     28650     28660              28677
                                           (28599)
Oryza sativa chromosome 1 region 9.891-41.663nt  (27957) T---GCTGATTACTGTTGTACTAGTA--TTATCATC--ATGTTCATGTACTCACCATAGAACCAAAACATAGCCATAGA
SARS-CoV-2 Reference Genome NC_045512.2 (26321) TCTTGCTAGTTACACTAGCCATCCTTACTGCGCTTCGATTGTGTGCGTACTGCTGCAATATTGTTAACGTGAGTCTTGT
```

Section 364

```
                                                    28678       28690     28700     28710     28720     28730     28740           28756
                                           (28678)
Oryza sativa chromosome 1 region 9.891-41.663nt  (28029) ATAAAATGCTTATTTGATCCAATAATAATAAAATATAGCTGCATGCAGTTC-AGTTCAT--TTCACTTATCTCTGTCAA
SARS-CoV-2 Reference Genome NC_045512.2 (26400) AAAACCTTCTTTTTACGTTTACTC-TCGTGTTAAAAATCTGAATTCTTCTAGAGTTCCTGATCTTCTGGTCTAAACGAA
```

Section 365

```
                                                   (28757) 28757        28770     28780     28790     28800     28810     28820          28835
Oryza sativa chromosome 1 region 9.891-41.663nt    (28105) AGGAAGGTTTTAACCATCTCTCTCTAAAGGACTCTG-TTCCTGCTATGATA-AAGAAAACGTTAACATGACTAGTGAGT
SARS-CoV-2 Reference Genome NC_045512.2            (26478) CTAAATATTATATTAGTTTTCTGTTTGGAACTTTAATTTTAGCCATGGCAGATTCCAACGGTACTATTACCGTTGAA-
```

Section 366

```
                                                   (28836) 28836          28850     28860     28870     28880     28890     28900          28914
Oryza sativa chromosome 1 region 9.891-41.663nt    (28182) GGATCTCCTCGCGCAGCTAGATTTTAAAGGAA--TTAAATTTGATATACTACAACT--CTTCCCTTTTGATTCTATAAT
SARS-CoV-2 Reference Genome NC_045512.2            (26556) -GAGCTTAAAAAGCTCCTTGAAC--AATGGAACCTAGTAATAGGTTTCCTATTCCTTACATGGATTTGTCTTCTACAAT
```

Section 367

```
                                                   (28915) 28915 28920   28930     28940     28950     28960     28970     28980          28993
Oryza sativa chromosome 1 region 9.891-41.663nt    (28257) TGTATTCTCTATGCAACACATTTAAAAATCAGATTGAATTTTATATAGAGTGAATTGCATATTGGGCTAGTTATTATG
SARS-CoV-2 Reference Genome NC_045512.2            (26632) T----TGCCTATGCCA-ACAGGAATA----------GGTTTTGTATATAATTAAGTAATTTCCTCTGGCTGTTATG
```

Section 368

```
                                                   (28994) 28994 29000     29010     29020     29030     29040     29050     29060       29072
Oryza sativa chromosome 1 region 9.891-41.663nt    (28336) ACCAAAGTTGCACTTTAGACCAGGGCACATCCAACATTTTCAGTTTAGACCAGAATCTTTTACCTTGGGTTGCACATTA
SARS-CoV-2 Reference Genome NC_045512.2            (26696) GCCA--GTA--ACTTTAG-CTTGTTTTGTGCTTGC---TGCTGTTTA---CAGAAT-----AAATTGGAT--CACCGGT
```

Section 369

```
                                                   (29073) 29073  29080     29090     29100     29110     29120     29130     29140       29151
Oryza sativa chromosome 1 region 9.891-41.663nt    (28415) GACCACCCTATC-CATTTCCAGTTCCACATCCCCTGTTTTCACTCAGCCACTGC-TTGCTCCTACATCAAGCTGCTGCC
SARS-CoV-2 Reference Genome NC_045512.2            (26757) GGAATTGCTATCGCAATGGCTTGTCTTGTAGGCTTGATGTGGCTCAGCTACTTCATTGCTTCTT--TCAGACTGTT---
```

Section 370

```
                                                   (29152) 29152   29160     29170     29180     29190     29200     29210     29220   29230
Oryza sativa chromosome 1 region 9.891-41.663nt    (28492) TGCTGCTAATTGTCTCTAACTTTGGCACTGACATTGGGTCAAGCCAGAAGATGAGTACAACACCACTGGCATGAGGTAT
SARS-CoV-2 Reference Genome NC_045512.2            (26831) TGCGCGTACGCGT-TCCA--TGTGGT-----CATT----CAATCCAGAAACT------AACATTCTTCTCA--ACGT--
```

Section 371

```
                                                   (29231) 29231    29240     29250     29260     29270     29280     29290              29309
Oryza sativa chromosome 1 region 9.891-41.663nt    (28571) TGAAAACCACGGGAATGGCCTTGAAGAGAGGAGCTCTGTCTATGTTGCCTTTGAGCTTGAGCTCAAGCTCCATCTGTG
SARS-CoV-2 Reference Genome NC_045512.2            (26888) -GCCACTCCATGGCACTATTCT-GACCAGACCGCTTCTAGAAA-GTGAACTCGTAATCGGAGCTGT-GATCCTTC-GTG
```

Section 372

	(29310)	29310 29320 29330 29340 29350 29360 29370 29388
Oryza sativa chromosome 1 region 9.891-41.663nt	(28650)	GCCACCTTTGC-TAGCTGTGCTTGAGCTCGACCTCCGC-ATACCATGCCCATGCTCGATCGCTCCAC--TTGC-ACCTC
SARS-CoV-2 Reference Genome NC_045512.2	(26962)	GACATCTTCGTATTGCTGGACACCATCTAGGACGCTGTGACATCAAGGACCTGCCTAAAGAAATCACTGTTGCTACATC

Section 373

	(29389)	29389 29400 29410 29420 29430 29440 29450 29467
Oryza sativa chromosome 1 region 9.891-41.663nt	(28724)	CATGGCGCCTCGATAGCCCTGCCTCGAAGCTACTCTTTTTTCTCCCATGTG-------TTGTGCTCATTGCTCTTGCAA
SARS-CoV-2 Reference Genome NC_045512.2	(27041)	ACGAACGCTTTCTTATTACAAATTGGGAGCTTCGCAGCGTG-TAGCAGGTGACTCAGGTTTTGCTGCATACAGTCGCTA

Section 374

	(29468)	29468 29480 29490 29500 29510 29520 29530 29546
Oryza sativa chromosome 1 region 9.891-41.663nt	(28796)	CAGTAGTGGCAGCAGGAATTGGTCTAGTGCTTGATCTAGTCATGAGCTCCTAATCTAGATGCTCAAGCTT-GAGGGTAA
SARS-CoV-2 Reference Genome NC_045512.2	(27119)	CAGGATTGGCAACTATAAATTAAACACAGACCATTCCAGT-AGCAGTGAC-AATATTGCTTTGCTTGTACAGTAAGTGA

Section 375

	(29547)	29547 29560 29570 29580 29590 29600 29610 29625
Oryza sativa chromosome 1 region 9.891-41.663nt	(28874)	CAACAGATGGGCAGCTAGACCGCTATAGGCAGGCTGGAAAGGGAAGTGGAGGCAGCGATAGTGGGGATGGGGATGACTG
SARS-CoV-2 Reference Genome NC_045512.2	(27196)	CAACAGATGT-----TTCATCTCGTT----------------GACTTTCAGGTTACTATAGCAGAGAT----ATTACTA

Section 376

	(29626)	29626 29640 29650 29660 29670 29680 29690 29704
Oryza sativa chromosome 1 region 9.891-41.663nt	(28953)	GTCTAGAGGAGGAAGAAAGAAGGAGAAGAGGGAGAGACACCTGG--TCTGGCTTACATG-TGGGCCTC-TAGTCCAAAT
SARS-CoV-2 Reference Genome NC_045512.2	(27250)	ATTATTATGAGGACTTTTAAAGTTTC-----------CATTTGGAATCTTGATTACATCATAAACCTCATAATTAAAAA

Section 377

	(29705)	29705 29710 29720 29730 29740 29750 29760 29770 29783
Oryza sativa chromosome 1 region 9.891-41.663nt	(29028)	TGCATGCTCAAGTAACTATGGCTGGTTGAAAGTGAAAATGTTTGATAACACCTAGTTCATTGTGAAACATGGGTAAGAA
SARS-CoV-2 Reference Genome NC_045512.2	(27318)	TTTAT-CT-AAGTCACTA------------ACTGAGAATAAATATTCTCAATTAGATGAAGAGCAACCAATGGAGATTG

Section 378

	(29784)	29784 29790 29800 29810 29820 29830 29840 29850 29862
Oryza sativa chromosome 1 region 9.891-41.663nt	(29107)	AATATAGTCCAACATGCAATTCACTCTTAAAATATACAACTCTTAGCTTATATTTCATAGATATAATCTTTTTTTCTT
SARS-CoV-2 Reference Genome NC_045512.2	(27383)	ATTAAA--CGAACATGAAAATTATTCTT------TTCTTGGCACTGATAACACTCGC-TACTTGTGAGCTTTATCACTA

Section 379	(29863)	29863 29870 29880 29890 29900 29910 29920 29930 29941
Oryza sativa chromosome 1 region 9.891-41.663nt	(29186)	ACATACATAACTCTTAGCTTTTATAGATACAATTGTATTTTCTATATAATATCTATATTGATCAATCTACACCGCCATG
SARS-CoV-2 Reference Genome NC_045512.2	(27453)	CCAAGAGTG---------TGTTAGAGGTACAACAGTACTTTTAAAAGAACCTTGCTCTTCTGGAACATACGAGGGCA--
Section 380	(29942)	29942 29950 29960 29970 29980 29990 30000 30010 30020
Oryza sativa chromosome 1 region 9.891-41.663nt	(29265)	TATTGAACATATAATGATCGGGAGCTTAAGATCTTTATTTTTTTCCCAACTAATGTCTCTAGAGCAAACATGGTTTCTA
SARS-CoV-2 Reference Genome NC_045512.2	(27521)	-ATTCACCATTTCATCCTCTAGCTGATAACAAATTTG---------CACTGACTTGCTTTAGCACTCAATTTGCTTTTG
Section 381	(30021)	30021 30030 30040 30050 30060 30070 30080 30099
Oryza sativa chromosome 1 region 9.891-41.663nt	(29344)	CCTGTAATATCTAATAGACTTATAATCATGTAGTAATGTTTTAGCATGGCCAGCAACAAGGACCATGTATCCTTGATG
SARS-CoV-2 Reference Genome NC_045512.2	(27590)	CTTGTCCTGAC-----GGCGTAAAA-CACGTCT--ATCAGTTA-CGTG-CCAG-ATCA--GTTTCACCTAAACT----G
Section 382	(30100)	30100 30110 30120 30130 30140 30150 30160 30178
Oryza sativa chromosome 1 region 9.891-41.663nt	(29423)	TTCAAATCTTTAAATAAGTGAAAAGTGAAACCCTTCACACAAAAATATCACGCCACGT--CATTAAGAAAACATGGATC
SARS-CoV-2 Reference Genome NC_045512.2	(27652)	TTCA-----TCAGACAAGAGGAAGTTCAAGAACTTTACTCTCCAATTTTTCTTATTGTTGCGGCAATAGTGTTTATAAC
Section 383	(30179)	30179 30190 30200 30210 30220 30230 30240 30257
Oryza sativa chromosome 1 region 9.891-41.663nt	(29500)	G-TCCGATTACTCCTCTAAACCATG-CATGCCGCTTGATCTCTCAATTGGTGAAATTTCATTTGCACCCTTGAGGAAAC
SARS-CoV-2 Reference Genome NC_045512.2	(27726)	ACTTTGCTTCACACTCAAAAGAAAGACAGAATGATTGAACTTTCATTAATTGACTTCT-ATTTGTGCTTTTTAGC----
Section 384	(30258)	30258 30270 30280 30290 30300 30310 30320 30336
Oryza sativa chromosome 1 region 9.891-41.663nt	(29577)	TTCTTTCACAAATGAAGTCCTCCCATATCCCTACTCGTGGAGCCGCTTCGTCCTCCGGTTTCCACCTCCCATTCGATAG
SARS-CoV-2 Reference Genome NC_045512.2	(27800)	--CTTTCTGCTAT----TCCTTGTTT-----TAATTATG----CTTATTATCTTTTGGTTCTCACTT-------GAACT
Section 385	(30337)	30337 30350 30360 30370 30380 30390 30400 30415
Oryza sativa chromosome 1 region 9.891-41.663nt	(29656)	GCAACAGCCCGACGCTGCTCGCTACCTCCTTCCACCTCAACGCCACCACCGTCTCCTCTCCACCATGTTCTCTCACTC
SARS-CoV-2 Reference Genome NC_045512.2	(27857)	GCAAGATCATAATGAAACTTGTCACG--------CCTAACG-----AACATGAAATTTCT-----TGTTTTCTTAGGA

```
Section 386
                                                    (30416) 30416         30430     30440     30450     30460     30470     30480       30494
Oryza sativa chromosome 1 region 9.891-41.663nt     (29735) TTCCCTCAACTTCCTTTGCCTCTACCCATCCCCATCCCCGACGCGCCTGC---GACGCTGCTCCTTAACCA-TTGGTG
SARS-CoV-2 Reference Genome NC_045512.2             (27918) ATCATCACAACTGTAGCTGCATTTCACCAAGAATGTAGTTTACAGTCATGTACTCAACATCAACCATATGTAGTTGATG

Section 387
                                                    (30495) 30495 30500     30510     30520     30530     30540     30550     30560       30573
Oryza sativa chromosome 1 region 9.891-41.663nt     (29810) TGCCCTCTTCGATTCGTCACCAGATCTACGTGTTTTATTCTGGAATCTAATCCAAGAACTCGGCAGATTATATCTCGAA
SARS-CoV-2 Reference Genome NC_045512.2             (27997) ACCCGTGTCCTATTCACTTCTAT-TCTAAATGGTATATTA--GAGTAGGAGCTAGAAAATCAGCACCTTTAATT--GAA

Section 388
                                                    (30574) 30574 30580     30590     30600     30610     30620     30630     30640       30652
Oryza sativa chromosome 1 region 9.891-41.663nt     (29889) CTCCACATTCTAGGTTATCGTCATTGCAATTGTTCTGCAGAGGGGAGGAACCAGGGAAGGTGATGCCATATTGTCGTCG
SARS-CoV-2 Reference Genome NC_045512.2             (28071) TTGTGCGTG---GATGAGGCTGGTTCTAAATCACCCATTCAGTACATCGATATCGGTAATT-ATACAGTTTCCTGTTTA

Section 389
                                                    (30653) 30653 30660     30670     30680     30690     30700     30710     30720       30731
Oryza sativa chromosome 1 region 9.891-41.663nt     (29968) CCTATCGCATAATAGTTTCCCGCAGGGACCTCGTCTTTGCTCCAGCTTGCGTTGGGGAGGTACGACTCTCTTCTCAATT
SARS-CoV-2 Reference Genome NC_045512.2             (28146) CCTTTTACA-ATTAATTGCC---AGGAACCTAAA--TTGGGTAGTCTTGTAGTGCGTTGTT----CGTTCTA-TGAAGA

Section 390
                                                    (30732) 30732 30740     30750     30760     30770     30780     30790     30800       30810
Oryza sativa chromosome 1 region 9.891-41.663nt     (30047) CTTATTGCAGCTATCACAGATGTTATGCTCGATTTGTGTCGAAACAAGATTTCGGCAATAATAAAAGGGGGTGGCTATC
SARS-CoV-2 Reference Genome NC_045512.2             (28214) CTTTTTAGAG-TATCAT-GACGTTCGTGTTGTTTTAGATTTCATCTAAA---CGAACAAACTAAAATGTC-TGATAATG

Section 391
                                                    (30811) 30811     30820     30830     30840     30850     30860     30870             30889
Oryza sativa chromosome 1 region 9.891-41.663nt     (30126) AAGCTAGGAAAGTGGATGGGTTTAGAGACAAGGAATTTTATACAGGTTCAGGCCTTCTTATTCAAGAAGTAATA-CCCT
SARS-CoV-2 Reference Genome NC_045512.2             (28287) GACCCC--AAAATCAGCGAAATGC---ACCCCGCATTACGTTT-GGTGGA--CCCTCAGATTCAACTGGCAGTAACCAG

Section 392
                                                    (30890) 30890     30900     30910     30920     30930     30940     30950             30968
Oryza sativa chromosome 1 region 9.891-41.663nt     (30204) ACTCCTGTCCGGGGATGATTCCGCCGAGTGTGTTATTGATTGTGTGATTGAGAGAAAAAGAATCGTTCCGAGAGGAACC
SARS-CoV-2 Reference Genome NC_045512.2             (28358) AATGGAGAACGCAGTGGGCGCGATCAAAACAACGTCGGCCCCAAGGTTTACCCAATAATACTGCGTCTTGGTTCA--C
```

```
                                                          Section 393
                                                    (30969) 30969      30980     30990     31000     31010     31020     31030          31047
Oryza sativa chromosome 1 region 9.891-41.663nt     (30283) CGGACCCCTCCTTATATAGGGGAAGGGATCCGT---ATTACAA--AGTACAGCCCATATC--CTAACGGAACATGGAT
SARS-CoV-2 Reference Genome NC_045512.2             (28435) CG---CTCTCACTCAACATGGCAAGGAAGACCTTAAATTCCCTCGAGGACAAGGCGTTCCAATTAACACCA-ATAGCAG

                                                          Section 394
                                                    (31048) 31048       31060     31070     31080     31090     31100     31110          31126
Oryza sativa chromosome 1 region 9.891-41.663nt     (30355) TACAGATAAATACAATCGTA--ACCGATTAGGATCCCTGGTA-----TCTTCTTGACATACAAGTTTAGAATCTAAC-
SARS-CoV-2 Reference Genome NC_045512.2             (28510) TCCAGATGACCAAATTGGCTACTACCGAAGAGCTACCAGACGAATTCGTGGTGGTGACGGTAAAATGAAAGATCTCAGT

                                                          Section 395
                                                    (31127) 31127        31140     31150     31160     31170     31180     31190          31205
Oryza sativa chromosome 1 region 9.891-41.663nt     (30426) CCGAGTCCGCATAAAGATATTCTATCTACTGGAATGTAACCGCCATGTGGATTTGGGG-TAACCATAATCTCCACA---
SARS-CoV-2 Reference Genome NC_045512.2             (28589) CCAAGATGGTATTTCTACTACCTAGGAACTGGGCCAGAAGCTGGACTTCCCTATGGTGCTAACAAAGACGGCATCATAT

                                                          Section 396
                                                    (31206) 31206         31220     31230     31240     31250     31260     31270        31284
Oryza sativa chromosome 1 region 9.891-41.663nt     (30501) -G-TTGCCCCGAGA---CCTTCACAGTTGACCAAAATAGTCTTCTCGAAACAAAAAACAATAATTCGAGTGCTTCAA-
SARS-CoV-2 Reference Genome NC_045512.2             (28668) GGGTTGCAACTGAGGGAGCCTTGAA--TACACCAAAAGA-TCACATTGGCACCCGCAATCCTGCTAACAATGCTGCAAT

                                                          Section 397
                                                    (31285) 31285 31290     31300     31310     31320     31330     31340     31350       31363
Oryza sativa chromosome 1 region 9.891-41.663nt     (30574) -----TTCAACTTGCTCCGGCTTGCCGAGTGCTAAAAGACTGTGAAGGCGAAAATAAAACATGGTGCA-TCGAG----
SARS-CoV-2 Reference Genome NC_045512.2             (28744) CGTGCTACAACTTCCTCAAGGAACAACATTGCCAAAAGGCT-TCTACGCAGAAGGGAGCAGAGGCGGCAGTCAAGCCTC

                                                          Section 398
                                                    (31364) 31364  31370     31380     31390     31400     31410     31420     31430        31442
Oryza sativa chromosome 1 region 9.891-41.663nt     (30643) TAGATGCACCTAATTAGGTA-TAGCCCCGACTATGTGATTAGTTGAAAAACTAAACATATAGTCAAGAGCCGAGTGGT
SARS-CoV-2 Reference Genome NC_045512.2             (28822) TTCTCGTTCCTCATCACGTAGTCGCAACAGTTCAAGAAATTCAACTCCAGGC--AGCAG-TAGGGGAACTTCTCCTGCT

                                                          Section 399
                                                    (31443) 31443   31450     31460     31470     31480     31490     31500     31510     31521
Oryza sativa chromosome 1 region 9.891-41.663nt     (30721) TTTATAGCGAAGCACACATGGTACTTAGTAAGATACCCAGCCGACTGCTTCTTAATTTGAAT-ATATCAAGGACAAAAA
SARS-CoV-2 Reference Genome NC_045512.2             (28898) AGAATGGCTG-GCAATGGCGGTGATGCTGCTCTTGCTTTGCTG-CTGCTTGACAGATTGAACCAGCTTGAGAGCAAAAT
```

Section 400

```
                                              (31522) 31522   31530      31540      31550      31560      31570      31580      31590 31600
Oryza sativa chromosome 1 region 9.891-41.663nt (30799) ATACAAGAAGGGCCGAGTGACAAACACTCAAAAGGTCCAAAAATAGATATATTTGGTAGGAATCCGAGCAAGAAACTCT
SARS-CoV-2 Reference Genome NC_045512.2 (28975) GTCTGGTAAAGGCCAACA-ACAACAAGGCCAAACTGTCACTAAGAAATCTGCTGCTGAGGCTTCTAAG-AAGCCTCGGC
```

Section 401

```
                                              (31601) 31601    31610      31620      31630      31640      31650      31660        31679
Oryza sativa chromosome 1 region 9.891-41.663nt (30878) TAAAATGA---TCCACCAAA---TACGATAAAGATCATGGTAATTAAGAAGTTCATTAGCCTAGGCTATAACCCTTACC
SARS-CoV-2 Reference Genome NC_045512.2 (29052) AAAAACGTACTGCCACTAAAGCATACAATGTAACACAAGCT--TTCGGCAGACG--TGGTCCAGAACAAACCCAAGGAA
```

Section 402

```
                                              (31680) 31680     31690      31700      31710      31720      31730      31740        31758
Oryza sativa chromosome 1 region 9.891-41.663nt (30951) ATAATCAAAATAAGCATATAA-CATGCTAGGAGAATGACTACTAATAAACCAGTCATCTCAAATTAATCCAACAGCTCT
SARS-CoV-2 Reference Genome NC_045512.2 (29127) ATTTTGGGGACCAGGAACTAATCAGACAAGGA-ACTGATTACAAA-----CATTGGCCGCAAATTGCACAATTTGCCCC
```

Section 403

```
                                              (31759) 31759      31770      31780      31790      31800      31810      31820        31837
Oryza sativa chromosome 1 region 9.891-41.663nt (31029) TGTAGCCTAAAAAAGCTTACAAAAATGGTATAACACCTTTCTGTCGGGCACCTTTTCATGTGCATCATAATAATTCCAA
SARS-CoV-2 Reference Genome NC_045512.2 (29200) CAGCGCTTCAGCGTTCTTCGGAATGTCGCGCATTGGCAT---GGAAGTCACACCTTCGGGAACGTGGTTGACCTACACA
```

Section 404

```
                                              (31838) 31838       31850      31860      31870      31880      31890      31900      31916
Oryza sativa chromosome 1 region 9.891-41.663nt (31108) AGAATTATTGAATCGCGTTAAAAAGG---AT-TTTAACAGCATCGGGCCGTGTCATTGTGAGCACATATTGCTAAAGCA
SARS-CoV-2 Reference Genome NC_045512.2 (29276) GGTGCCATCAAATTGGATGACAAAGATCCAAATTTCAAAG-ATCAAGTCATTTTGCTGAATAAGCATATTG---ACGCA
```

Section 405

```
                                              (31917) 31917        31930      31940      31950      31960      31970      31980      31995
Oryza sativa chromosome 1 region 9.891-41.663nt (31183) AAAAGCGCCTAAGTCCCTAGGGATCACAACAGGCCACGCTGAAAACATAATTGGGCTGAAGTACTGTACAGGCCTAGGC
SARS-CoV-2 Reference Genome NC_045512.2 (29351) TACAAAACATTCCCACCAACAGAGCCTAAAAAGG-AC-----AAAAAGAAGAAGGCTGATGAAACT--CAAGCCT----
```

Section 406

```
                                              (31996) 31996         32010      32020      32030      32040      32050      32060    32074
Oryza sativa chromosome 1 region 9.891-41.663nt (31262) CCATTAGTACTCCCGATACCCTAAAAATACCGAAGTCCAAACATCGCTTCGTCTTCCCCGCCGAGACTCTCGCCATT-C
SARS-CoV-2 Reference Genome NC_045512.2 (29418) -------TACCGCAGAGACAGAAGAAACAGC-------AAACTGTGACTCTTCTTCCTGCTGCAGATTTGGATGATTTC
```

Section 407

(32075) 32075 32080 32090 32100 32110 32120 32130 32140 32153
Oryza sativa chromosome 1 region 9.891-41.663nt (31340) CCCACTCCGT-GCTACAGTACA-GAACCGCGCCGACG-AAGTTAGGTTCATCAATCCGCTCCAATCCATCCTCAAAAAC
SARS-CoV-2 Reference Genome NC_045512.2 (29483) TCCAAACAATTGCAACAATCCATGAGCAGTGCTGACTCAACTCAGG--CCTAAACTCA-TGCAGACCA----CACAAGG

Section 408

(32154) 32154 32160 32170 32180 32190 32200 32210 32220 32232
Oryza sativa chromosome 1 region 9.891-41.663nt (31416) TTCACGAAATTTCTCCCCGCATCCACCGCTCCGATTCCCCATCCCTTTCCAGCCGTATCTTGAACTAAATCAAATCATC
SARS-CoV-2 Reference Genome NC_045512.2 (29555) CAGATGGGCTATATAAACGTTTTCGCTTTTCCGTTTACGA-----TATATAGTCTACTCTTGTGCAGAATGAATTCTCG

Section 409

(32233) 32233 32240 32250 32260 32270 32280 32290 32300 32311
Oryza sativa chromosome 1 region 9.891-41.663nt (31495) TAGTTCACATTCATGGCGGAAGAAAA---AGAAAGCTTCGAGAGTCAGTGGGCGCCCTCCGATGTGACAGAAGATAATC
SARS-CoV-2 Reference Genome NC_045512.2 (29629) TA----AC-TACATAGCACAAGTAGATGTAGTTAACTTTAATC-TCACATAGCAATCTTTAATCAGTGTGTA-ACATTA

Section 410

(32312) 32312 32320 32330 32340 32350 32360 32370 32380 32390
Oryza sativa chromosome 1 region 9.891-41.663nt (31571) TGAAGGAGATGGTGGCGC-ACGGCGTTCTCCCAGCTAAGGAGATTATCGGATGGCGACCGAC-GTTCGGCGAAGCATTC
SARS-CoV-2 Reference Genome NC_045512.2 (29701) GGGAGGACTTGAAAGAGCCACCACATTTTCACCG---AGGCCACG--CGGAGTACGATCGAGTGTACAGTGAACAATGC

Section 411

(32391) 32391 32400 32410 32420 32430 32440 32450 32469
Oryza sativa chromosome 1 region 9.891-41.663nt (31648) CCGACCCC-CGACACACACGAAATCGTGGTATTTGCTCA--TTTCTTCTATGGCGGATTTTCTCTTCCAACCTCAAGAT
SARS-CoV-2 Reference Genome NC_045512.2 (29775) TAGGGAGAGCTGCCTATATGGAAGAGCCCTAATGTGTAAAATTAATTTTAGTAGTGCTATCCCCATGTGATTTTAATAG

Section 412

(32470) 32470 32480 32490 32500 32519
Oryza sativa chromosome 1 region 9.891-41.663nt (31724) TCTTCCGGGGGATTCTGAACTTCTACGGGATTAGCTTGCATCACTTGAAC
SARS-CoV-2 Reference Genome NC_045512.2 (29854) CTTCTTAGGAGAATGACAAAAAAAAAAAAAAAAAAAAAAAAAAAAAAAAA
