# Supplementary material for: Patch type nucleotide sequence identities between genomes from many different species facilitate illegitimate recombination
Source: Sci Rep. 2026 Mar 30;16:10524. doi: 10.1038/s41598-026-44124-0 (PMC13035915; doi:10.1038/s41598-026-44124-0)
Supplement: Supplementary file 5 — Supplementary Material 5 [file 41598_2026_44124_MOESM5_ESM.pdf]

Bombus pascuorum ~~chromosome 14~~ vs. Oryza sativa ~~chromosome 11~~

Section 1

	(1)	1 10 20 30 40 50 60 74
Bombus pascuorum chromosome 14 NC_083501.1 region...	(1)	------------------------ACCGTG-ATACGT-GTAATTAATGTCG---A-TCATTCGATTTCACTACT
Oryza sativa chromosome 11 NC_029266.1 region from 8...	(1)	GTATGCAGTGTAATAGAATCAGATTCATTGGATAGGAAGCAATGAATGACTCATAATCATTTGAAAGTAAGGAA

Section 2

	(75)	75 80 90 100 110 120 130 148
Bombus pascuorum chromosome 14 NC_083501.1 region...	(45)	AAATTTTTATAAATTTCTATAAATTTCACATTATTATTAATAAACACCTAAAATTCTTAACAAACGTTATTTCT
Oryza sativa chromosome 11 NC_029266.1 region from 8...	(75)	AAAAAGAGAGACGAGTCCATT--TTACATGTCAACTTTCGTCAAAATA-AAAATTCATCCCTAAACTTCAAAAT

Section 3

	(149)	149 160 170 180 190 200 210 222
Bombus pascuorum chromosome 14 NC_083501.1 region...	(119)	GATATTCAATGTTATTCAATA-TTATTAATAACCGAATGT----AAAACAATTTAAGAGAAAATCTTTGAATTT
Oryza sativa chromosome 11 NC_029266.1 region from 8...	(146)	TGGATACTAT-TTATCCCTTAGTTTTTAATACCGTTTTCTTTTGAAAAGTATGTACATCATTTTTTTTATATTT

Section 4

	(223)	223 230 240 250 260 270 280 296
Bombus pascuorum chromosome 14 NC_083501.1 region...	(188)	CGAAGCGAATACTTTTATATATCTGATCGTTG-TGTCCCCTGTTTGTTAAATATATCGCTTTTGTCGTTTATAC
Oryza sativa chromosome 11 NC_029266.1 region from 8...	(219)	TTTCTC-AATCATTTACTAACTTTTATAATTAATATATATTTTAACCTATTTGAATCGGTCAAAA-GATGAAAA

Section 5

	(297)	297 310 320 330 340 350 360 370
Bombus pascuorum chromosome 14 NC_083501.1 region...	(261)	GAGCATTCTATCGATATCGTTTCTATCTGAATCGATCGCGTTCATTCACATTTCTTATCTTGGAAGCGAATACC
Oryza sativa chromosome 11 NC_029266.1 region from 8...	(291)	GTAAATGGTTGAGGGATGAATAATGTCTGGTTT--TGAAGTTTATGAATAAATGTTAGACTTGATGAAAATTGG

Section 6

	(371)	371 380 390 400 410 420 430 444
Bombus pascuorum chromosome 14 NC_083501.1 region...	(335)	AATCACGTTCCATAATATTACGTTATTAACGACATTACTGACGTTAACGT-TACAACAAC-TAACCGTTCATTG
Oryza sativa chromosome 11 NC_029266.1 region from 8...	(363)	AG--ATGTAAAGGGATTTTTC--TATTAAAAGAATATGCGATTTTGACATGTACAAATGGATAAGCTCAGGCCG

Section 7

	(445)	445 450 460 470 480 490 500 518
Bombus pascuorum chromosome 14 NC_083501.1 region...	(407)	TGCCGTAAAATATACATACAT-ACATACAGTACCGATATCGTAATAAAAATTAACTTTAAGATTATCAATGAA-
Oryza sativa chromosome 11 NC_029266.1 region from 8...	(433)	GCACGTCCATTGCGGTTTCACCAGAGCCAATCCATCTTGCAAGATTCCAATGAAATAGAGGCATA-CATGGAAT

Section 8

	(519)	519 530 540 550 560 570 580 592
Bombus pascuorum chromosome 14 NC_083501.1 region...	(479)	ACAATTTGAACAGATATAGGTAATGGATATAGAAATGGAAC--TGGCACGGAGAACAATCCTCGAATTGTGTTG
Oryza sativa chromosome 11 NC_029266.1 region from 8...	(506)	ATATTCTGGAGACTGTGAAGATTTGAAGATGTTACAGATAAGATGATTTGAACTATATAATTCCAGCTACGTAA

Section 9

	(593)	593 600 610 620 630 640 650 666
Bombus pascuorum chromosome 14 NC_083501.1 region...	(551)	AAACGGTATGGAAATGATTAT---TGAAAT-----CATATAGGGAAACCGTTTCGATCTTGCTAAAAGACTATG
Oryza sativa chromosome 11 NC_029266.1 region from 8...	(580)	AAAAGCTAAGACAATGATTATATATGAGATGGTAGCATAT-GTGAAATGGTTTAGTGAATACTTAAA-ACTTTA

Section 10

	(667)	667 680 690 700 710 720 730 740
Bombus pascuorum chromosome 14 NC_083501.1 region...	(617)	CTGATTAATTAAGTAAAAGCTGCTGCGTGTCTCGATTTTTATTTGAAAAGGAAATAGTCGTAAGAGAGAAAGAA
Oryza sativa chromosome 11 NC_029266.1 region from 8...	(652)	TTTAGGTGTTTGGTTAGGGAGAGAGAGAGTGGCGATAATTAT---AAGGGACAATTGTT-TATTTGACTTTGTT

Section 11

	(741)	741 750 760 770 780 790 800 814
Bombus pascuorum chromosome 14 NC_083501.1 region...	(691)	A-GAAGGAAAAGTGAAACTCGTCGTTTAAACGTACTCGATGG--TATTAG--TGGTTTTCATCGGTGATTTA-C
Oryza sativa chromosome 11 NC_029266.1 region from 8...	(722)	TTGAAGCCTAACTACCAATTTGACTCTATTTTTTCTAGTTTGCTTATTTGCCTGCTTTTCGAAAATGAACACTC

Section 12

	(815)	815 820 830 840 850 860 870 888
Bombus pascuorum chromosome 14 NC_083501.1 region...	(759)	TGTCTC-CATAGTAATGGAA-ATCTAGAAAAGTACGATCGTACAATGTGTTATATCGTATTATATTTATGTATC
Oryza sativa chromosome 11 NC_029266.1 region from 8...	(796)	CGTCTGACCTGTTTCTACAACACCGTTAAGAGTTCATTTTAAAAAAGAGGAAAA----ATAAAAATCACATTTC

Section 13

	(889)	889 900 910 920 930 940 950 962
Bombus pascuorum chromosome 14 NC_083501.1 region...	(831)	TAATCTACGGCAAGTTGTTAAGTATAAGTTGTCGAGTTAGAGATGGAGAGCGGAAGTGCGTGCTCGTCGATCGA
Oryza sativa chromosome 11 NC_029266.1 region from 8...	(866)	ATAATGACTAAAATACCCTCCAACTCTATCCTCATCTAACCCTTTTCTCTCCCGGTCTCTCTCTC-TCTATCAT

Section 14

	(963)	963 970 980 990 1000 1010 1020 1036
Bombus pascuorum chromosome 14 NC_083501.1 region...	(905)	ATCGTACCTAAGTTAAAGAATTAAAGAAAGCGTTGTAGGTGTGTCTGAATGAGACAAATAAATGGGTCGAAGCT
Oryza sativa chromosome 11 NC_029266.1 region from 8...	(939)	ATCCCTTCTCTCTCCGGC-------GAGGGAGCTGTGGCGGGGAAGGTAAGCGGCAGAGCCGGCTGGTGGAGGG

Bombus pascuorum chromosome 14 vs. Oryza sativa chromosome 11

Section 15

	(1037)	1037 1050 1060 1070 1080 1090 1100 1110
Bombus pascuorum chromosome 14 NC_083501.1 region...	(979)	TGGTTAAACTGGGGTC----GCCTCTCATTGATAAATTAGATCGAGTATCGTTGCAAGTATGATAAATTATTTC
Oryza sativa chromosome 11 NC_029266.1 region from 8...	(1006)	AGGGACCACCGGCGGCAACGGCGGGCCATGGAGGGAGGGGATCCACGCCGGCGCCAACT-TGCGGAGGGAGGGC

Section 16

	(1111)	1111 1120 1130 1140 1150 1160 1170 1184
Bombus pascuorum chromosome 14 NC_083501.1 region...	(1049)	ATTTATGATAAATTACGCTGTGGACAAGTAGCTTTCTAACAAGATGAACGCAATCGAATAAACTTGCAGAAA-G
Oryza sativa chromosome 11 NC_029266.1 region from 8...	(1079)	GGCTATGCCGACGTG-GATCAGCGGCAGCGCCGGCTGATGGAGAGGGGAGCACCGGCGGCGGCGGGGGGCCGTG

Section 17

	(1185)	1185 1190 1200 1210 1220 1230 1240 1258
Bombus pascuorum chromosome 14 NC_083501.1 region...	(1122)	CATCTACGATATTACCGATAATACCAA-TGAAGCCGATATTCCTTGTATTCGGGATATATCTATTACACTTATT
Oryza sativa chromosome 11 NC_029266.1 region from 8...	(1152)	GAGGGATGGGATCCACTTCGGTGTCAACTCGCGGTGGGAGGGCGGCTGCGCCGAGCA---CGGATCCACGGCTG

Section 18

	(1259)	1259 1270 1280 1290 1300 1310 1320 1332
Bombus pascuorum chromosome 14 NC_083501.1 region...	(1195)	TGTTATTCCATTAAAAAGGAAGGAAATTATGTTTACGTCTATAGTTTTGTA--G-TAAAAAATCCACCGTTTTT
Oryza sativa chromosome 11 NC_029266.1 region from 8...	(1223)	TGCCGCTGGTGGAGGGAGGGAGCACCGGCGGCGGCGGCCGGCCGTGGAGGAAGGGGATCCGCGCCGCCGTCAAC

Section 19

	(1333)	1333 1340 1350 1360 1370 1380 1390 1406
Bombus pascuorum chromosome 14 NC_083501.1 region...	(1266)	TCCTATGCATAGGTTGCATTA-----------ATATACTTTGTATATTTTGTATATATTATTTGAAAATATTAT
Oryza sativa chromosome 11 NC_029266.1 region from 8...	(1297)	TTGTAGAGGGAGGGCGGCTGCGCCGAGCGCAGATCTGCGGCGGCGCCGGCGGAGTTTCAACCCATAGGAAGTCC

Section 20

	(1407)	1407 1420 1430 1440 1450 1460 1470 1480
Bombus pascuorum chromosome 14 NC_083501.1 region...	(1329)	ATTAATGTGTATATTAATGTGAAAGTAAAAATAAACATAACGTA-AGTAAAATAAGTGAAATTATAATATAAGT
Oryza sativa chromosome 11 NC_029266.1 region from 8...	(1371)	ACCCCTGCTGCTTCCAGCCCGCCGGCGAGGGGGAGCTCGTTGTCCAGGCCAAGCAGAGCAGGCCGATGGC--GT

Section 21

	(1481)	1481 1490 1500 1510 1520 1530 1540 1554
Bombus pascuorum chromosome 14 NC_083501.1 region...	(1402)	TATTAATAATATTTTATTAAGTAAAGTATAATTACGTTAGGTTA-AGCAG--ACGGTCGTTTCAAAGTCCTTTT
Oryza sativa chromosome 11 NC_029266.1 region from 8...	(1443)	GGCCAACGCGGGCGCAGGCGCTCGAATGGAGGGAGG--AGGTGGCAGCGGCAACGCCGGATTCGCCGACCGCAG

Bombus pascuorum chromosome 14 vs. Oryza sativa chromosome 11

		**Section 22**
	(1555)	1555 1560 1570 1580 1590 1600 1610 1628
Bombus pascuorum chromosome 14 NC_083501.1 region...	(1473)	TTCGACGGTTTC-GACGG---TTTTCGAATGGAGTTATTATCTTACTTTTCGCTATTCCTTTCGATATTTCGTT
Oryza sativa chromosome 11 NC_029266.1 region from 8...	(1515)	CTCGAGATCCACCGCCCGGACTTGCCGCCGCCACTCCTCGTCGTCGTCGTCACCAGGGCCGGCGCCGGCTCGCC
		**Section 23**
	(1629)	1629 1640 1650 1660 1670 1680 1690 1702
Bombus pascuorum chromosome 14 NC_083501.1 region...	(1543)	ATTTCTA-----GCGGCAACTCCTAGTACGCTAAAACGCGCGCTCCGCCTAATAACCACAA------ACTTAAC
Oryza sativa chromosome 11 NC_029266.1 region from 8...	(1589)	GTCTCGACTTTCGCCGCCTCTCCCTGTGGTGGATCCGTGGAGGAGAGGAGAAGAACTTGCAGCCGCCACTCCTC
		**Section 24**
	(1703)	1703 1710 1720 1730 1740 1750 1760 1776
Bombus pascuorum chromosome 14 NC_083501.1 region...	(1606)	GTTATTTTCATCGTCGATACACGGCTGTGCTCGTGAATGCTATCATTTCCATAACCGTATATAGTTAAATGTGG
Oryza sativa chromosome 11 NC_029266.1 region from 8...	(1663)	GTCGTCGTCGTCGTCGTCACCAGGGCCGGCGCCGGCTCGACGTCTCGCCTTTTGCTGCCTCTCCCTATG-GTGG
		**Section 25**
	(1777)	1777 1790 1800 1810 1820 1830 1840 1850
Bombus pascuorum chromosome 14 NC_083501.1 region...	(1680)	ATGATGGACGTGCCGAGCAACAGAGTCGATGATAGGTTGCATTAATATACTTCAAACGAGTTCATTACCGAATG
Oryza sativa chromosome 11 NC_029266.1 region from 8...	(1736)	ATCCTTG--GAGGAGAGGAGAAGAGA-GAAGAGAGGATTGGAGAAGATAGAACTGACGGGTGGGT--CCCACAT
		**Section 26**
	(1851)	1851 1860 1870 1880 1890 1900 1910 1924
Bombus pascuorum chromosome 14 NC_083501.1 region...	(1754)	GTAAGAAGTTGGCAACGACGGTTGTTGAAAAGAACGAAGGGGGATGGCCCGTTGCAGTTGTGCACGCGTACGGA
Oryza sativa chromosome 11 NC_029266.1 region from 8...	(1805)	GTAATAGGGTCA-AATGA--------GTAAGGGCAGAAGAGACATTTTGTGCTTCAGTTAA-CAC-CGTTAGA-
		**Section 27**
	(1925)	1925 1930 1940 1950 1960 1970 1980 1998
Bombus pascuorum chromosome 14 NC_083501.1 region...	(1828)	TTCATCGTTTTCGTTAGAGGAGACGTTCCGCTTTCTCCCGCTAT--------CGATTCACTAAGCGG---CTCG
Oryza sativa chromosome 11 NC_029266.1 region from 8...	(1867)	--CGCCCTTAACGGAATAGG-GTCATACCG-TGTCTTCAATTTTGAAAAGGAGGATCAAATAAGCAAACCCTAA
		**Section 28**
	(1999)	1999 2010 2020 2030 2040 2050 2060 2072
Bombus pascuorum chromosome 14 NC_083501.1 region...	(1891)	TAAGGAAGAGCAAAGCAGCCTTAAAGGCGCACGCTCAATGCCACTAACCTATTTACCTATGTAGGAAAGAGCTC
Oryza sativa chromosome 11 NC_029266.1 region from 8...	(1937)	AAAGTAGGGTCAAATTGGTAGTTAGAGTTCAAAACAGGTTCAAATAAGCAATTGCCCCTACTTATAATG---TC

Section 29

(2073) 2073 2080 2090 2100 2110 2120 2130 2146
Bombus pascuorum chromosome 14 NC_083501.1 region... (1965) GGCTGACTCATTTCCATCGATCGGCGTGCACGTGCACATATCTCGAA-AGGGACCGGGATTCTCTCGAATTCTC
Oryza sativa chromosome 11 NC_029266.1 region from 8... (2008) AACTAGTTGATACCTCGCGCTTTGC-TGCGGG-----ATATATGGATGATTGCATGTTATACATTATAGAAATG

Section 30

(2147) 2147 2160 2170 2180 2190 2200 2210 2220
Bombus pascuorum chromosome 14 NC_083501.1 region... (2038) GTTAATTCTTTTGTTCAGACAGCCTTTTGCAACGATGCTTTTATACACCATTCGTAAAAAGATCTACGATGAGT
Oryza sativa chromosome 11 NC_029266.1 region from 8... (2076) ATAGATGTATATGTCTAAATAA--TGTAAAAATGGTGTGGAGGTA-ATGATTCGACATTGCTTGCGTATTGAGT

Section 31

(2221) 2221 2230 2240 2250 2260 2270 2280 2294
Bombus pascuorum chromosome 14 NC_083501.1 region... (2112) A-TCGATTTTCAAGATCGTCTAGAAATAAAAGATAGG-CTTGTAGGCGTATTTTATTCGATGGTTTTGTTATCT
Oryza sativa chromosome 11 NC_029266.1 region from 8... (2147) TCTAAAAATACAATAAAAT-TAATTATAAATGATATGTCATGTTTGATGATATT-TAAAATACTCTAGATAA--

Section 32

(2295) 2295 2300 2310 2320 2330 2340 2350 2368
Bombus pascuorum chromosome 14 NC_083501.1 region... (2184) TACTTATACGTGCGT-----TG--GCTACCCTTGCAGATAGTCACCTATCCT-TCGTTGGTTAGTCGAAGCCTG
Oryza sativa chromosome 11 NC_029266.1 region from 8... (2217) TAATTACCAGTGGGTGATAATGTGGC-ACGCTTGCATGTGATTTAAAATACTCTAGATAATAATTGCTAGT-TG

Section 33

(2369) 2369 2380 2390 2400 2410 2420 2430 2442
Bombus pascuorum chromosome 14 NC_083501.1 region... (2250) ATCGACTGTGAATGCCAATTTCTAGTCTATTTCCAAAAAAAAGCGTGATATAAATGAGTTCAGCTAAAGAA-AT
Oryza sativa chromosome 11 NC_029266.1 region from 8... (2289) GT-GATGATGTGGCACACTTGCATGTTGAGTTTTAGGAATTAGTGGGC-ATCAACTA---CATAGAAAGTATAG

Section 34

(2443) 2443 2450 2460 2470 2480 2490 2500 2516
Bombus pascuorum chromosome 14 NC_083501.1 region... (2323) CTAAATATCCGGAGCGAACAAAGAAAACCTGA---GGG-AAACGATGAGCTGTTA----GACGGGGAAGTT---
Oryza sativa chromosome 11 NC_029266.1 region from 8... (2358) TTATAGATGCGGGCACACCCAAGGCAATTTGAAAGGGGGTTATGGT-AGCTGTTATGAAGAGGGGAATGTATCA

Section 35

(2517) 2517 2530 2540 2550 2560 2570 2580 2590
Bombus pascuorum chromosome 14 NC_083501.1 region... (2386) TAGCCGGAGAAGCTGCCTGAGGAAATCGTGGAATTTTCAGCGTGTTTTAAAAAGCGACCGATCGTTAACAGTAA
Oryza sativa chromosome 11 NC_029266.1 region from 8... (2431) TTGCAGGAGAAGATTCTAGTGCACTGATTTTTTTTTACTAATGCCTTATTGTA-GACTCATAGTTA-CAG--A

Bombus pascuorum chromosome 14 vs. Oryza sativa chromosome 11

Section 36

	(2591)	2591 2600 2610 2620 2630 2640 2650 2664
Bombus pascuorum chromosome 14 NC_083501.1 region...	(2460)	GCGTATATCTGCAGCTTTGAGACATAAAAATGTCTCTTTGTCCATTCTGCAGCCGCGATCGTTTCGCGGAGGAA
Oryza sativa chromosome 11 NC_029266.1 region from 8...	(2501)	GCATATGATAAAATCGTCATTCTATAATAATG-CTCGGTGCTAACTTATCTCTATCAAACTATTATTGAATGAT

Section 37

	(2665)	2665 2670 2680 2690 2700 2710 2720 2738
Bombus pascuorum chromosome 14 NC_083501.1 region...	(2534)	TTCTCGCGGAAGAGGAAGAAAAGTACGGTAAAGGCGCACCCCGGCT-----ATTGCATATTGTA-TGCAGCGTT
Oryza sativa chromosome 11 NC_029266.1 region from 8...	(2574)	TTC-------AGTACTCCTTATGTATTCATTATTCCCATGCTTGATAGTAGATTGAATTTCATAGTGAAGCATT

Section 38

	(2739)	2739 2750 2760 2770 2780 2790 2800 2812
Bombus pascuorum chromosome 14 NC_083501.1 region...	(2602)	--TCAACTTTAAATTGCTTTCTTTTAATCCGAAAACAACGC----AACTGAAAAAAGAACT-ACGTAGTTGACG
Oryza sativa chromosome 11 NC_029266.1 region from 8...	(2641)	AGTAAAAGTTGCACAAGTTTTTTTTAAAGGAAAAACAAAATGATATACTGTTGCAGGCACAGAAGGAGGCCATG

Section 39

	(2813)	2813 2820 2830 2840 2850 2860 2870 2886
Bombus pascuorum chromosome 14 NC_083501.1 region...	(2669)	TTCTAATGGTTCTTTCGT-GTGGCTGA--TAAAAATGTAACTTGGT-CCCTGCAACTCGTATATTTCTAACAAC
Oryza sativa chromosome 11 NC_029266.1 region from 8...	(2715)	GTGGCGTGGAGAGAAAGGAGCAGCAGCAGCAGAGGGGTTACCAGCTGCCCCGTGACTCCCGGGGATCACTCGAG

Section 40

	(2887)	2887 2900 2910 2920 2930 2940 2950 2960
Bombus pascuorum chromosome 14 NC_083501.1 region...	(2739)	AAGGGTAAAGAGTCTGTAACGATCTCGA--GGAAAATTTCCTGTAATTTGTGAAAACGACTGTGGAAAGAGACA
Oryza sativa chromosome 11 NC_029266.1 region from 8...	(2789)	GTGTTCAACCCCTCATCCGCTTCCTCCTTCCGCACTGCTGCTGCCGCCCCCAAGTCCGCCTCCCCCTTC---CT

Section 41

	(2961)	2961 2970 2980 2990 3000 3010 3020 3034
Bombus pascuorum chromosome 14 NC_083501.1 region...	(2811)	TGTGAGAACAGACTGGATAAACATAAATTCATAAGTAGATCATCGGAGTTTGTACTTTAATTTATT--TAGTGA
Oryza sativa chromosome 11 NC_029266.1 region from 8...	(2860)	TGCTATTCCCGACAGGGAAGAGGATAATGTGGTGGCACAGCAGCGGGCAGCAGAGTGGGGCCTCGTCCTCCAGA

Section 42

	(3035)	3035 3040 3050 3060 3070 3080 3090 3108
Bombus pascuorum chromosome 14 NC_083501.1 region...	(2883)	TATTTTACCGTTTATAATTACCACAATATCATCCATTGTATTATTATTGCTTACCTTATTTATAATAATTTCTC
Oryza sativa chromosome 11 NC_029266.1 region from 8...	(2934)	CCGACCACCACACAGGCCTCCCCCAGGG-CGTCTCC-GCACGACCCTCCTCTGGCTCTGCACGCACCAGCTCTG

Section 43

	(3109)	3109 3120 3130 3140 3150 3160 3170 3182
Bombus pascuorum chromosome 14 NC_083501.1 region...	(2957)	AAG-CAATTTTTTGCTAATTTTTTGC--CAATTTTTTGCCAATTTTATGCCAATTTTTTGCAAATTTTTTGCCA
Oryza sativa chromosome 11 NC_029266.1 region from 8...	(3006)	AGGACAACCCACAGCAACAGCAGAGCGCCGCCGCCATCCCACGGGTGTCGGAGGAGCTACGGGCGGCACTATCA

Section 44

	(3183)	3183 3190 3200 3210 3220 3230 3240 3256
Bombus pascuorum chromosome 14 NC_083501.1 region...	(3028)	ATTTTTTGCTAAT--TTTATGCCAACGGATTGTTGCAACGAGAATAATATTGGAATAGAAAGGTGGAAAGAGTT
Oryza sativa chromosome 11 NC_029266.1 region from 8...	(3080)	GTGTTCCAACAGACGTTTGTGGTGTCTGATGCCACCCACCCCAACCACCCCATCATGTACGCCAGCGCTGGCTT

Section 45

	(3257)	3257 3270 3280 3290 3300 3310 3320 3330
Bombus pascuorum chromosome 14 NC_083501.1 region...	(3100)	TGACAAAGTTATTATAGTTAAATTAAAGGCATGAAACCGGAATATTTAGTAGAAGAA--AAGTACACAAACAGA
Oryza sativa chromosome 11 NC_029266.1 region from 8...	(3154)	CTTCAACATGACCGGCTATACCTCCAAGGAGGTTGTCGGCAGGAACTGGTTCGATCCTCACACACACACACATA

Section 46

	(3331)	3331 3340 3350 3360 3370 3380 3390 3404
Bombus pascuorum chromosome 14 NC_083501.1 region...	(3172)	AAGACGATGATAGGCT-AGATAATTATTAACGGAAATGGTGATAGGAAAGAGACAGTTAAAGTAGGTTAAACGA
Oryza sativa chromosome 11 NC_029266.1 region from 8...	(3228)	AACACATCAATCCAATTATTCAATTAATAATC--AATTGTGTGTGCAGCCGCTTCCTTCAAGGCTCTGGCACC-

Section 47

	(3405)	3405 3410 3420 3430 3440 3450 3460 3478
Bombus pascuorum chromosome 14 NC_083501.1 region...	(3245)	AGAATAGCGTAAAAAAAAAAAAATTGACGTTGATGTTTGCGGAAAGAGACAATTCGAACGATTCGATAATTATT
Oryza sativa chromosome 11 NC_029266.1 region from 8...	(3299)	-GACCCCCATGAGATTGACAAGATCA--GGCAATCCCT-CGCAAATGGC----TCCAACTATTGTGGCCGCATC

Section 48

	(3479)	3479 3490 3500 3510 3520 3530 3540 3552
Bombus pascuorum chromosome 14 NC_083501.1 region...	(3319)	TTCAAATTCATTTTCGACGA-ACG---TTC-----CAATTATACGATA--C-CTTTGTAGTTTAATTACTGCAA
Oryza sativa chromosome 11 NC_029266.1 region from 8...	(3365)	CTCAACTACAAGAAGGACGGCACGCCATTCTGGAACCTCTTAACCATTGCCCCTATCAAGGATGAGGACGGCAG

Section 49

	(3553)	3553 3560 3570 3580 3590 3600 3610 3626
Bombus pascuorum chromosome 14 NC_083501.1 region...	(3381)	CAGCTTATCCTTATCATTGTTAGAGCAATAATTATCTCA----AAGAGCTTTAATCTC-TGATAATTGTACACG
Oryza sativa chromosome 11 NC_029266.1 region from 8...	(3439)	--GCTCCTCAAGTTCATCGGGTCAGCTATTTTCCCATCACGTTAATTACATACATCACATAAACAGATTATTAC

Bombus pascuorum chromosome 14 vs. Oryza sativa chromosome 11

Section 50

	(3627)	3627 3640 3650 3660 3670 3680 3690 3700
Bombus pascuorum chromosome 14 NC_083501.1 region...	(3450)	AAATC-GAAACATAATATTCTTTTAACCATTGCAATCCT-TTATATGCGGTACGTTGAAATAACGTATTATACG
Oryza sativa chromosome 11 NC_029266.1 region from 8...	(3511)	ATTTCAGCACCATATTTTTATTTGGCTGTTTGCATTGTTATTCCAGGATGCAAGTGGAAGTTAGTAAATACACT

Section 51

	(3701)	3701 3710 3720 3730 3740 3750 3760 3774
Bombus pascuorum chromosome 14 NC_083501.1 region...	(3522)	GATAAACAAGAATATACTCCGGATCAGGTTTAATCGAATTCATGAATGAAACGTCTTTTTACGCGTAAAAGAAC
Oryza sativa chromosome 11 NC_029266.1 region from 8...	(3585)	GAAGGGAAAAAGGATACTGTTGTTC-GTCCAAATGGACTTTCAGAATCACTCATCAAATACGATGGTACTACTA

Section 52

	(3775)	3775 3780 3790 3800 3810 3820 3830 3848
Bombus pascuorum chromosome 14 NC_083501.1 region...	(3596)	GCTGTTGTACATTTAATAAATAACGATTTCCAG-ATTAGGCGTTAACAAAACATAAATGTTCGTTTCAA----C
Oryza sativa chromosome 11 NC_029266.1 region from 8...	(3658)	AATGTTTATCTTCTGCTACAAAAGGGTTTTATCCATAAGGTCTTGGAAATTTACAACTTTTGGTTTCAAACTAA

Section 53

	(3849)	3849 3860 3870 3880 3890 3900 3910 3922
Bombus pascuorum chromosome 14 NC_083501.1 region...	(3665)	AGCTGCTATTTAAACGATCC-TTAATATAAATAAC---ATAGAA------------TAGGTTT-TGCCGAAACG
Oryza sativa chromosome 11 NC_029266.1 region from 8...	(3732)	AAAAGAGAAAAAAATCAACAATTAATATTTACAGCTCGACAGAAGGATCACGCCCGTAGCTCAGTGTCTGAGCT

Section 54

	(3923)	3923 3930 3940 3950 3960 3970 3980 3996
Bombus pascuorum chromosome 14 NC_083501.1 region...	(3722)	TTTACTTCCTCGGAATATTTTTCGTGGAGAGGAA--ATCACG--ATGAAACCA-AAGTAGAAGAGTTGCTGATC
Oryza sativa chromosome 11 NC_029266.1 region from 8...	(3806)	CTTGTTGGCTCTCAAAAATCCACGCTCATTGTCAGAATCAAGCAATAACACCTTAAAAAGAAAATCACAAGAAT

Section 55

	(3997)	3997 4010 4020 4030 4040 4050 4060 4070
Bombus pascuorum chromosome 14 NC_083501.1 region...	(3791)	CAGCGCGCAGGG--ATGAAAGAAAACC--AGGAAAACTACTCGTGCAGCTCTCTTACCACGTAACGCGTGTA--
Oryza sativa chromosome 11 NC_029266.1 region from 8...	(3880)	CCCTAAGCATGTCAATGAGCGAAGTTCCTAGTAAGAGAAGCTCTGAAAGTGGATCTCGCCGAAATTCTCGTAGC

Section 56

	(4071)	4071 4080 4090 4100 4110 4120 4130 4144
Bombus pascuorum chromosome 14 NC_083501.1 region...	(3859)	--AACTCTGCGC-CACTGCGTAATATATAACGCGT-CCTATTC---TTAGCCGCTTTATCACGCCTTTTCGATT
Oryza sativa chromosome 11 NC_029266.1 region from 8...	(3954)	GGAACAAGGAGCTCGCTTCAGAAGATCAATGAAGTACCTGATCAAGTGAATAGAACTAGAAAATCTGGTCTGCG

Bombus pascuorum chromosome 14 vs. Oryza sativa chromosome 11

Section 57

```
                                                   (4145) 4145 4150      4160      4170      4180      4190      4200              4218
Bombus pascuorum chromosome 14 NC_083501.1 region... (3926) TCCATCTCCC---TTTGAATCCATCTCGCGTTGAACGAAGAATTTGTCCGCGCG-ATGACGATGCCAATCTTTG
Oryza sativa chromosome 11 NC_029266.1 region from 8... (4028) TGCATTTATGGGGTATGCATACAAGTCAAATTATAT-AACATGTTGTTTGCATTTATCTTGTTTGTCTTACTTA
```

Section 58

```
                                                   (4219) 4219       4230      4240      4250      4260      4270      4280         4292
Bombus pascuorum chromosome 14 NC_083501.1 region... (3996) CTTCGTCGAGGCAGAATGCCGCAGTAACGAGCCTG----TTTTCACTGCAACGATATCGAGACGTTGAGCTGTC
Oryza sativa chromosome 11 NC_029266.1 region from 8... (4101) ATTTGTACATGCAGTTTTCTTG-GTATGGCCATGGAAGTGTAGAGAAGAAC-ATGCTGAAACCTAGAGATGAA
```

Section 59

```
                                                   (4293) 4293   4300      4310      4320      4330      4340      4350          4366
Bombus pascuorum chromosome 14 NC_083501.1 region... (4066) TCTTTGGCGACGGGAATGGAGCGTGGAAACGTGATCGTACCGACTTTGAATTCTTCCGTTAGGGCATGATAG
Oryza sativa chromosome 11 NC_029266.1 region from 8... (4173) GATCCACTGATCGATAGTGATGA-TGAAAGACCTGAGAG------CTTTGAAGA-TGAGTTTAGG--AGGAAAG
```

Section 60

```
                                                   (4367) 4367         4380      4390      4400      4410      4420      4430     4440
Bombus pascuorum chromosome 14 NC_083501.1 region... (4140) ATTTTTAATTGGGGTGTTGATCGTTGCGCGAGTCTTCTGCCCGATTAGGATTACATGTTGTTTGGAAA--TGGA
Oryza sativa chromosome 11 NC_029266.1 region from 8... (4237) AAATG-AGGAGGGGTATAGA--------CTTGGCTACTACAC--TT--GA--ACGTATTGAGAAGAATTTTGTC
```

Section 61

```
                                                   (4441) 4441     4450      4460      4470      4480      4490      4500          4514
Bombus pascuorum chromosome 14 NC_083501.1 region... (4212) ATCAAGTACAATGAACGTTGCGTGATGTGATACTTATGTACGGATGTAGGTGCAGGGACCTTAAA---TTAGTC
Oryza sativa chromosome 11 NC_029266.1 region from 8... (4296) ATCAC--AGATCCAAGGTTGCCTGATA--ATCCCATTGTAAG-ATCCATATGGAATCTCTTTACCTCTTACAC
```

Section 62

```
                                                   (4515) 4515 4520      4530      4540      4550      4560      4570              4588
Bombus pascuorum chromosome 14 NC_083501.1 region... (4283) CTGCTCTCTTCTTTCGCTTATCTTCTCCGCTTAATTTTCTAACGTGAAAATCGTTATCTATATGCATGATTTTT
Oryza sativa chromosome 11 NC_029266.1 region from 8... (4365) CGCACCTGTTCTTTTTATTAATATGGATATTAAATTTTCATGAATGTATGTTTGGTTCATATTTCAGATATTT
```

Section 63

```
                                                   (4589) 4589       4600      4610      4620      4630      4640      4650         4662
Bombus pascuorum chromosome 14 NC_083501.1 region... (4357) CGTTTCGAATGAACAAAATCGAAATATAAAGATTCAAGGGGATTCGTAGCGGTTAATTTTAAATTGGGATTTCA
Oryza sativa chromosome 11 NC_029266.1 region from 8... (4439) GCATCCGATAG--CTTTCTGCAATTGACAGAATACAACCGT----GAAGA----AATATTAGGGAGAAACTGCA
```

Section 64

	(4663)	4663 4670 4680 4690 4700 4710 4720 4736
Bombus pascuorum chromosome 14 NC_083501.1 region...	(4431)	GTTAAACAGAATGCTGCTTTGTTTTACTTAGCTATCGGCGTAACATTATATTTCGTTATTGTAATCGTTTGCAA
Oryza sativa chromosome 11 NC_029266.1 region from 8...	(4503)	GGTAATGATGATCCATCTTGCCCCTTTT-GCT----GCACAATTTACTGGAAGGTTTTTAAATTTTATAGAAA

Section 65

	(4737)	4737 4750 4760 4770 4780 4790 4800 4810
Bombus pascuorum chromosome 14 NC_083501.1 region...	(4505)	ATAGTAGGCAAGTCGCGGTAATGATTAGGTTTTCGTTTTTAAATTAACAGCAGGACTATAGTAACGTATACACA
Oryza sativa chromosome 11 NC_029266.1 region from 8...	(4572)	ATTTCAGGCTTTT----GTAAA-ATT---TTTGCACTTTAACGGTATAAGTTAG-CTTT--TCATGTAAA-ATA

Section 66

	(4811)	4811 4820 4830 4840 4850 4860 4870 4884
Bombus pascuorum chromosome 14 NC_083501.1 region...	(4579)	TCCGGGCATAGGTATCAGGGTGTCCGCTCGTTACGTGTTTGTGGAAACTCTGAGTTTTCCTAATCTG--CCTG
Oryza sativa chromosome 11 NC_029266.1 region from 8...	(4634)	CTAGTGAACAACTTGCCTGGTGCTTTCTTCTGCCCTTTGTGCAGGTTTCTTCAAGGCC-CTGAAACTGATCGTG

Section 67

	(4885)	4885 4890 4900 4910 4920 4930 4940 4958
Bombus pascuorum chromosome 14 NC_083501.1 region...	(4651)	ATACAGCTGAGATGC-ATGATCTGTGTTCCTTTACGTGGCAGAAGATGGCGAA-ATCTGGAGAGTTCGTCAAAG
Oryza sativa chromosome 11 NC_029266.1 region from 8...	(4707)	CAACAGTTAGGAAGATAAGAGATGCCATAGATAACCAGGCAGAGGTCACAGTTCAGCTCATAAATTATACAAAA

Section 68

	(4959)	4959 4970 4980 4990 5000 5010 5020 5032
Bombus pascuorum chromosome 14 NC_083501.1 region...	(4723)	GGCGTAGACGGATGAAAGTCGCGCGATCGGGACACCGGTTTATTAAACAGCGACCTTGCCGATATTTAGGACGA
Oryza sativa chromosome 11 NC_029266.1 region from 8...	(4781)	AGTG--GTAGGCTACTCGTCACTTG-TCTGCATGTTTTTGTTCTCAACTTATAAAT-----ATAATAGGTAGTT

Section 69

	(5033)	5033 5040 5050 5060 5070 5080 5090 5106
Bombus pascuorum chromosome 14 NC_083501.1 region...	(4797)	GACACGGCGGCCGTATCAGCGAGCAACGCACTT-AGCGCATGGTCATAATTCGATTGTA-GGTATATTAATAGG
Oryza sativa chromosome 11 NC_029266.1 region from 8...	(4847)	GATACCCGCGCTTTGTTGCGGGATATGTATATGAATACATGCTATACATTATAGAAAATGGTAGACGTATATA

Section 70

	(5107)	5107 5120 5130 5140 5150 5160 5170 5180
Bombus pascuorum chromosome 14 NC_083501.1 region...	(4869)	ATTGCATTA--TAGATACGTCACCGTTAGACGGAGTATCCAGGCAACGCAT-TAGTG---TAGTCGCGTCACGT
Oryza sativa chromosome 11 NC_029266.1 region from 8...	(4921)	TTTAAATAAGGTGAAAATGATGTGGAGATAATGATTTGACATTGCTTGCATATTGAGTTCTAAAATATAACAA

```
                                                                  Section 71
                                                  (5181) 5181     5190      5200      5210      5220      5230      5240          5254
Bombus pascuorum chromosome 14 NC_083501.1 region... (4937) AATTACAGATACTAAGC-------GAATCGCGACGCGAAGGCACCGGATGTTAACTCC----GCGCCATTGTTC
Oryza sativa chromosome 11 NC_029266.1 region from 8... (4995) AATTGTAAATGATATGGCATGTTTGCATGATATTTAAAATACTCTAGATAATAATTGCTATTGGGTGATGATGC
                                                                  Section 72
                                                  (5255) 5255 5260      5270      5280      5290      5300      5310              5328
Bombus pascuorum chromosome 14 NC_083501.1 region... (5000) CGTATATTCGTGTTTCGCATTTCTGGTAAACT---TGTTCGTT-CGAGTAACTGATAATTCGATATGGTTAT-T
Oryza sativa chromosome 11 NC_029266.1 region from 8... (5069) GGTACACTTGTATGTGG--TTTAAAATACTCTAGATGATAATTACTTGTGAGTGATAATGTGGCACACTTGCAT
                                                                  Section 73
                                                  (5329) 5329       5340      5350      5360      5370      5380      5390        5402
Bombus pascuorum chromosome 14 NC_083501.1 region... (5069) ATTGGATATGAGAAGGTAATCGT--TTGACTCATTGCTAA--TTGGACGTTATTCATCCCGGTATCGATGCAAC
Oryza sativa chromosome 11 NC_029266.1 region from 8... (5141) GTTGTATTTTAAGAGTTAGTGGACATCAATTTTATAATAAGATAGGATATGTCTTTTCACAGTAAAGTTACCTT
                                                                  Section 74
                                                  (5403) 5403   5410      5420      5430      5440      5450      5460            5476
Bombus pascuorum chromosome 14 NC_083501.1 region... (5139) TGCGGTATTACTACAATTGCGCGATCAACGATATACGTATATGACTTGCTACCGATAGAAACAAT-CGTAATAC
Oryza sativa chromosome 11 NC_029266.1 region from 8... (5215) CCCTTCATTACTTCA-TTACTTGGACAGGTAAAAAGTTTTGGAACCTCTTTCACTTGCAACCAATGCGTGATCA
                                                                  Section 75
                                                  (5477) 5477         5490      5500      5510      5520      5530      5540    5550
Bombus pascuorum chromosome 14 NC_083501.1 region... (5212) AACACAGCTCACGTTATTTGTGTTTTTTATTCGGGAAATTTCGTTTGTGTT--TCAAATTATTATATTATTATA
Oryza sativa chromosome 11 NC_029266.1 region from 8... (5288) AA---AGGT-ACTATA---GTGACAGACATGGGAAAAGTTTCTGCTGAGTTATTCAACATATTTGTCTACAATT
                                                                  Section 76
                                                  (5551) 5551     5560      5570      5580      5590      5600      5610          5624
Bombus pascuorum chromosome 14 NC_083501.1 region... (5284) GCTTCCTATACGAATATAGCAATTTGAATATCAAGTTCATATTTAT-AGAGTACCCTTCACATTAACTTTTACG
Oryza sativa chromosome 11 NC_029266.1 region from 8... (5355) CTTTTGTATGT-AATTTGACA---TGAGTCCAATTTGCCTGTGGATCAGGGTGATGTTCAG-TACTTTATTGGG
                                                                  Section 77
                                                  (5625) 5625 5630      5640      5650      5660      5670      5680              5698
Bombus pascuorum chromosome 14 NC_083501.1 region... (5357) TTTCTA--GGTTCTATCGTTAGATTATAAATCTTTTACCTCAAGTCTTTCTTTATATGTAACATATTTTGTTCG
Oryza sativa chromosome 11 NC_029266.1 region from 8... (5424) GTTCAATTGGATGGAACTGAGCATGTTCAGGATGATGCTGCAAAAGAGGGAGTCG-TGCTGGTTTGTATCTTGA
```

Section 78

```
(5699)                                                       5699       5710      5720      5730      5740      5750      5760          5772
Bombus pascuorum chromosome 14 NC_083501.1 region...   (5429) TATAGAAGAAATAGGTAGAGGTAGATAGATAGATTGATAGATGTCGGAATTCCAGCCCTCGGGAGATCATAGAA
Oryza sativa chromosome 11 NC_029266.1 region from 8... (5497) AAAATCATATTTGTGCAACTGACAAAAGCAACACAAATAGATATGGGCAGTCCCATTGTCATGTTATGTTTTGT
```

Section 79

```
(5773)                                                       5773  5780      5790      5800      5810      5820      5830          5846
Bombus pascuorum chromosome 14 NC_083501.1 region...   (5503) CGGACTTGGTTTATAATATTTCTTATAAACACTCATCCCTCCAAACCATAACCACACC-GCCTTCTGATCTGTA
Oryza sativa chromosome 11 NC_029266.1 region from 8... (5571) AGTCTGATGCTTCTGGAGTTT--TATTAAGAACGAAGGCTATGTTCATTTGCAAGAGTTGGAAACACAGCTGTT
```

Section 80

```
(5847)                                                       5847         5860      5870      5880      5890      5900      5910    5920
Bombus pascuorum chromosome 14 NC_083501.1 region...   (5576) TCGCACGCAGTACGGTACTTTCTTTGGTCCTGTTACTTCTCACCTCTTA--------CTTTTCACTCTCACTTT
Oryza sativa chromosome 11 NC_029266.1 region from 8... (5643) GCGCACGGGAAACGGAGCGGTCCATTAGCACATTATTAATTAAGTATTAGCCAATTTCTTTTCAAAAATGGATC
```

Section 81

```
(5921)                                                       5921     5930      5940      5950      5960      5970      5980          5994
Bombus pascuorum chromosome 14 NC_083501.1 region...   (5642) AGAAGGTATCATCTTCAACATC-TACGTA-AGTTACATAGCCCGT---ACGCATCTATATACA--TTGAAACAC
Oryza sativa chromosome 11 NC_029266.1 region from 8... (5717) AATATGATTTTTTTAAAGCAACATTCGTATAGAAACTTTTTGCAAAAAACGCACTGTTTAACAATTTGAATAAC
```

Section 82

```
(5995)                                                       5995 6000      6010      6020      6030      6040      6050              6068
Bombus pascuorum chromosome 14 NC_083501.1 region...   (5709) GCGCGAAACACATCCAATGACGAAATTTCACACTGAAAGTTATAGTTAACAAATAAC---AAGTAAAATT--AT
Oryza sativa chromosome 11 NC_029266.1 region from 8... (5791) GTGGGCATGGAATACAA----GAGAGATGGGTTGGGAACATAGAGAAAAAAACTCAGCCTAACTGGAATTGCAA
```

Section 83

```
(6069)                                                       6069       6080      6090      6100      6110      6120      6130          6142
Bombus pascuorum chromosome 14 NC_083501.1 region...   (5778) TAATTCGTGAGATTTTTGTTCTCTCCTTAGTGAAGCAATATCTTTA--ACGTTAACCAAAAAATTCGATCGCAT
Oryza sativa chromosome 11 NC_029266.1 region from 8... (5861) AAATGCTCTATACCCGAAAGTCAAAACTAGTGCTACAGAACCTGTGATACATCTCACATATTATAAAATCTAAT
```

Section 84

```
(6143)                                                       6143  6150      6160      6170      6180      6190      6200          6216
Bombus pascuorum chromosome 14 NC_083501.1 region...   (5850) CTATTTATCGGAGTTGAAAATTTCGAAATAGCGTGAACAGTGAACTTGGTTTTATTAATTT-------CGTTT
Oryza sativa chromosome 11 NC_029266.1 region from 8... (5935) CTACGTGACTAGCATTCTCTTCTGGAAATAA--TTAATAGTTTTCTCTTATCTTTTCCATTTTTTAACACGTTG
```

Bombus pascuorum chromosome 14 vs. Oryza sativa chromosome 11

Section 85

	(6217)	6217 … 6290
Bombus pascuorum chromosome 14 NC_083501.1 region...	(5917)	GTTAATTCG-CTGAAGAATCGCCATATTGAAAAAGAAAAGAAAAAAAGAAGAAGAAAAAGAGAAAAGAAAAAAGA
Oryza sativa chromosome 11 NC_029266.1 region from 8...	(6007)	TTTCATTTTTCCTCATAATTTACCCTTTTATGTCTCCATGAGTGAGTGAATTTTGATTAAA-ATTTCTCACATT

Section 86

	(6291)	6291 … 6364
Bombus pascuorum chromosome 14 NC_083501.1 region...	(5990)	AGAGAAGATTGATATTCTGTTAAAACTTGTTGCCTGACTAAACTATATTCGCTGCATCATTCACGTATTAATAT
Oryza sativa chromosome 11 NC_029266.1 region from 8...	(6080)	AGATTACTGTGTCAATCCTGCCAAATTGTTTG--TGAATATTAGTGACTAATTGCACCAAATTTGGAC-AAGGC

Section 87

	(6365)	6365 … 6438
Bombus pascuorum chromosome 14 NC_083501.1 region...	(6064)	AATCCTCCGAATGTATTTTAATTTGCTTCAATCGCTAATAATGCTTCCATTAGATATCACTTTACATGATGAAA
Oryza sativa chromosome 11 NC_029266.1 region from 8...	(6151)	AAGCCTAGAAACACTGCATGAGTAAA--CGAGTTGTTGCATTGGTCCGGATAAACAAAAGTTGTTGCATTG---

Section 88

	(6439)	6439 … 6512
Bombus pascuorum chromosome 14 NC_083501.1 region...	(6138)	GTGAGGATACGAAAGAGTAAATAGGTCAA-GGTTACAAATTATAGCAGTAGT-----AGCTTTTAGAAGG----
Oryza sativa chromosome 11 NC_029266.1 region from 8...	(6220)	GTCCGGAT--GAAC---TAAACAGTTCAATGTTTACTAAACTTTGCTATGGACCTTCAACTTTATGAAAGCATA

Section 89

	(6513)	6513 … 6586
Bombus pascuorum chromosome 14 NC_083501.1 region...	(6202)	AAAGCGTAGCTACTTGCAATCTGGTCATGAACCGACAATACTAGTTTCAACGGCTGTTCGTTCCAAAGTTCAAA
Oryza sativa chromosome 11 NC_029266.1 region from 8...	(6289)	AAAGCATAGTTTGTTGGAAAA-----ATAAACTGGAATGTTTATGTGCCA-GGTTAAGAAAACCGCAGATAATA

Section 90

	(6587)	6587 … 6660
Bombus pascuorum chromosome 14 NC_083501.1 region...	(6276)	CACTCGCGCAATTTGCGTCGACAAACGCAAATAC-AATATCTTCT--TGCAAACTAATTCCGTTTCTTCCTCGA
Oryza sativa chromosome 11 NC_029266.1 region from 8...	(6357)	---TCGATGAGGCTGCAAAGGAACTCCCGGATGCTAATTTGGTCAGATTAAAGCATCACTCTTTGATTCATCAT

Section 91

	(6661)	6661 … 6734
Bombus pascuorum chromosome 14 NC_083501.1 region...	(6347)	TTACACAGTCGTAATTTCACCAATGTAGACGGCGCTT-CGAGCAGAATTTCGTTTGAAAATCCCGTGGGGTT-C
Oryza sativa chromosome 11 NC_029266.1 region from 8...	(6428)	ATATGTGCTTGCAGTTCTAACA--GTAACCCATGCTCACATTCAGA-----GACCGAAAGATCTATGGGCTAAC

```
                                                                      Section 92
                                                        (6735) 6735 6740      6750      6760      6770      6780      6790         6808
Bombus pascuorum chromosome 14 NC_083501.1 region...    (6419) AAGGCTAAAGTCGTTC-GTTTCATCGGGAACTTCACGGT-CTCGAGCACGCTTGGCAGCTGTACGCGGACGAAA
Oryza sativa chromosome 11 NC_029266.1 region from 8... (6495) CACTCAAAAGTAGTCCTGCCAAATCCACATATGAAGGATACTGCATCATGGAGAGCCATCCAAAAGGTATGATT
                                                                      Section 93
                                                        (6809) 6809       6820      6830      6840      6850      6860      6870   6882
Bombus pascuorum chromosome 14 NC_083501.1 region...    (6491) CCGCGGTAATAACAATAAATAATCGCTGGAAATTTGCGAGCACTGCCAGTGAGTCGCGGTTTCCAAT-TTCGCA
Oryza sativa chromosome 11 NC_029266.1 region from 8... (6569) TGACTATAGTT-CACTGAATTCGCTACGCAAACATCACTTTAAGGTTATTGCATATAGCAGGCTCATCTTTTCG
                                                                      Section 94
                                                        (6883) 6883   6890      6900      6910      6920      6930      6940         6956
Bombus pascuorum chromosome 14 NC_083501.1 region...    (6564) CTGACGCCGTTTGTCATCCAACCAACCAACCAACGACTTTCCAA-CAAAGC---------GGTGGTTTCTTCGA
Oryza sativa chromosome 11 NC_029266.1 region from 8... (6642) CTTATGC---TTATCAGCCAAAATTTGAATTTTCAACCTTAAATTCGAAGCCGATTTTGAGGTTTTTTCATCGA
                                                                      Section 95
                                                        (6957) 6957         6970      6980      6990      7000      7010      7020   7030
Bombus pascuorum chromosome 14 NC_083501.1 region...    (6628) ATGATTTTCTTCGGTCCTTTCGGTCCACTACGGTACTTTCTAGCGAATAAAAGAACGCCTTTCTCATGATGTTT
Oryza sativa chromosome 11 NC_029266.1 region from 8... (6713) AGTTTATTTTTCAG-CTTTTGCTTTTAGATCACTAAGAACATGTATATAAAAGCTTCATTTACAAATTACTTTT
                                                                      Section 96
                                                        (7031) 7031     7040      7050      7060      7070      7080      7090      7104
Bombus pascuorum chromosome 14 NC_083501.1 region...    (6702) CGCTTTCAGG-AT-TCATTT--CTCATTCATTGGCTC-GCTGGTAAATC--GTGGTAATTTTCAAGACTTGTAT
Oryza sativa chromosome 11 NC_029266.1 region from 8... (6786) CATTTGCAAATATGTCGTTTGGCTTATTCCGTAGATAAGCCAAACAATGGGGTTGTAAGTGTCATTATCTGGAC
                                                                      Section 97
                                                        (7105) 7105 7110      7120      7130      7140      7150      7160         7178
Bombus pascuorum chromosome 14 NC_083501.1 region...    (6769) CATTATATTTTTGCCAATTTACAATTTTCACGCGCCACTACTACTACTACTACTACTACTACTCATCCGCTTTT
Oryza sativa chromosome 11 NC_029266.1 region from 8... (6860) AAACTTGGAAGAGTTA-TATATCATTATCTAGACAAACATGGGAGAGTGATT----TACATGTCAACCTCTAT-
                                                                      Section 98
                                                        (7179) 7179       7190      7200      7210      7220      7230      7240   7252
Bombus pascuorum chromosome 14 NC_083501.1 region...    (6843) CGGCAAAATGTTTAATCGTGAAACGTGTCCTTTTGCTTCTTTAGCTTTAGCGCAATAATACCTATCAGAGATC
Oryza sativa chromosome 11 NC_029266.1 region from 8... (6928) -GTTTTAAAAGTGTCATTTT-AATCTCATCACTATTCTGTTGTAGAAT-------ACAACAGCCTTTATAGAAC
```

```
                                                                  Section 99
                                                (7253) 7253  7260      7270      7280      7290      7300      7310       7326
Bombus pascuorum chromosome 14 NC_083501.1 region... (6917) ATTTAAATTACGTGA-TACGAAGCGTCACGGACTTTCATTAATCCGTGTTAAGCTGTGAAATTAATCGAGATTA
Oryza sativa chromosome 11 NC_029266.1 region from 8... (6993) ACT--AGTTATTTGCCTACTATATTATATGCCATCACAGCCAGGAGATTTATACTGATGTATAATACAATAGCC
                                                                  Section 100
                                                (7327) 7327        7340      7350      7360      7370      7380      7390   7400
Bombus pascuorum chromosome 14 NC_083501.1 region... (6990) GATAGGAAAAATTAATCGAGAAATACAACTGCACAATTGACCGATCGATTTTAGTTAAAAATTGTGTGTCTATC
Oryza sativa chromosome 11 NC_029266.1 region from 8... (7065) TTTACAGAATATTAATA-ACTAATATCTCTTGTAGGTTC-----TCGAGAGTGGTGAAAGTATTGGTTTAAAAC
                                                                  Section 101
                                                (7401) 7401     7410      7420      7430      7440      7450      7460       7474
Bombus pascuorum chromosome 14 NC_083501.1 region... (7064) GATATAACCTGATAAAGAATTTAGCATGAAATTTTCAGCTTTTAAAGTTCTAATCGATTAACAACTGAAATAAA
Oryza sativa chromosome 11 NC_029266.1 region from 8... (7133) ATTTTAGGCCAGTAAAACCATTAGGATC---TGGTGACACTGGAAGGTA--AATCATTTCATGGTTTGGTTAAA
                                                                  Section 102
                                                (7475) 7475 7480      7490      7500      7510      7520      7530          7548
Bombus pascuorum chromosome 14 NC_083501.1 region... (7138) GTTGAAAAGAAAAAGAAACAAAGTTATATTATTCGATCGTAACTTTCTCAACTAATTATCAGATTCGTCCGATT
Oryza sativa chromosome 11 NC_029266.1 region from 8... (7202) TTGGATAA----------ACA---TTGAATTGTTGC-------CTTTTATTGCTAAT----ACATTTGACTCATT
                                                                  Section 103
                                                (7549) 7549       7560      7570      7580      7590      7600      7610    7622
Bombus pascuorum chromosome 14 NC_083501.1 region... (7212) TTTAGATTTATCGTAGAATGCCGATAATTTTACGTGATATTTTTGCTTTTTCAGCCGGTATAAAGGATTAATAT
Oryza sativa chromosome 11 NC_029266.1 region from 8... (7253) TTACAATTGTTGTTTCAACAATGTGTATTATGATTGGAATTGAAG--TGTACACTTGGTAGAGTTGTTGAATA-
                                                                  Section 104
                                                (7623) 7623  7630      7640      7650      7660      7670      7680       7696
Bombus pascuorum chromosome 14 NC_083501.1 region... (7286) GCCCGAAACATTCTTTGCTCTTCGAGGTTTCTGAAAATTACAGAAAAAACGCTTTTTCGCGATCA---TCGACC
Oryza sativa chromosome 11 NC_029266.1 region from 8... (7324) --CAGGAGAATACTTTGCCATGAAAGCTATG-GATAAAAGCATCAT----GCTTAACCGCAATAAGGTTTGATG
                                                                  Section 105
                                                (7697) 7697        7710      7720      7730      7740      7750      7760   7770
Bombus pascuorum chromosome 14 NC_083501.1 region... (7357) GCCATATATATTTTCATCAGAGCGACGTTCCTCGTTATCGCTGCTCGGATTCGACCATTTGCTTTCCAACTCTA
Oryza sativa chromosome 11 NC_029266.1 region from 8... (7391) CTTGTTTCACCTTTTAAAATGTATACATTTTTGT------------GAATAAAAGACATTGTTTATACTTCTA
```

Section 106

```
                                                            (7771) 7771     7780      7790      7800      7810      7820      7830         7844
Bombus pascuorum chromosome 14 NC_083501.1 region...        (7431) GATCAACCGAAAGGAAGAAATACTGGAATTACGATAATACTAATACTA----CACAGAGAAA-TAAAAAGCGAA
Oryza sativa chromosome 11 NC_029266.1 region from 8...     (7453) GTTATGTCTATTGCATTATAT-CTCTGAGTAC-ATTATAATGTCTCTAGGTTCACAGAGCTACTGCTGAACGCC
```

Section 107

```
                                                            (7845) 7845 7850      7860      7870      7880      7890      7900              7918
Bombus pascuorum chromosome 14 NC_083501.1 region...        (7500) AAAAGAATGATTTGTCCAATCTCTTTACCATTTCTTC--GCTTTGTAAT-ATCAGAATATTTAAAGAGAAAGAA
Oryza sativa chromosome 11 NC_029266.1 region from 8...     (7525) AAATCCTTGATCTATTAGATCAC----CCATTCCTTCCAACATTATATGCATCATTTCAGGTTTGCTCTATCAA
```

Section 108

```
                                                            (7919) 7919       7930      7940      7950      7960      7970      7980        7992
Bombus pascuorum chromosome 14 NC_083501.1 region...        (7571) TGTTCTCGTTGGAATGG--TATTAACGTTACAAAGAAATAAAAAATAAAAGAAAAAAAGAAAATTGGGCAGTAT
Oryza sativa chromosome 11 NC_029266.1 region from 8...     (7595) TAATCTAGCTAAGATGTCCTATTTATGT-ATAATCATTTCCATTTCACTCTATAATGTGGAAGCTATCCCTATT
```

Section 109

```
                                                            (7993) 7993   8000      8010      8020      8030      8040      8050            8066
Bombus pascuorum chromosome 14 NC_083501.1 region...        (7643) AGGTAGAGTGACTAAAGAA--GTAGCCAATTCGTTGACCTCTTGTCGCAGTACCACTCCACTTTTGCTACTAT
Oryza sativa chromosome 11 NC_029266.1 region from 8...     (7668) ATCTAGTCTTAATACCCCAACATAGCTA-----TAGAAATCTTTTTGCAGTACATCAGTTTTATGTGGTATTTT
```

Section 110

```
                                                            (8067) 8067         8080      8090      8100      8110      8120      8130     8140
Bombus pascuorum chromosome 14 NC_083501.1 region...        (7715) ---ACGCGTGTTTCCATTTGAAGCAAAGACCAATGAAACGAAAGAAAAGCAATCGATCGTTTCGCGTTTCAAAT
Oryza sativa chromosome 11 NC_029266.1 region from 8...     (7737) CTTATGAAT-TTTCCTTTTCAATATATGGCAGACAAAAACGCATATATGCC-----TCATTACTGACTACTGCC
```

Section 111

```
                                                            (8141) 8141     8150      8160      8170      8180      8190      8200        8214
Bombus pascuorum chromosome 14 NC_083501.1 region...        (7786) CTATTATTATTCTTTCGCTTCTGTCGTCGATTTTCTAACTCGG-----TCGAGAAAGATATAAAGTTAGTTTAC
Oryza sativa chromosome 11 NC_029266.1 region from 8...     (7805) CGGGTGGTGAGCTCTTTGTGCT-TC-TAGATAATCAACCTCTGAAGGTTCTACATGAAGATGCAGTCAGGTCAT
```

Section 112

```
                                                            (8215) 8215 8220      8230      8240      8250      8260      8270              8288
Bombus pascuorum chromosome 14 NC_083501.1 region...        (7855) GATACGAAAGAGAGAATTCTC---GCTTTAATCG--TCGACTCTGCAACTTCGAAACAAACAAACGGAAG-AG-
Oryza sativa chromosome 11 NC_029266.1 region from 8...     (7877) CATTTAAACTATTGTTTTCGCTTAGTTCTGATGGATGATGTGTGTGGCTTTGTTTTGAACACACGTTGGCAGC
```

Section 113

(8289) 8289 8300 8310 8320 8330 8340 8350 8362
Bombus pascuorum chromosome 14 NC_083501.1 region... (7922) ATATTCGAGAATGAATG--ACAACGATATTCT-CCATAATCTGTTATCCTTTCTTCGCTTTTAGCCGGACCACT
Oryza sativa chromosome 11 NC_029266.1 region from 8... (7951) ATATGTGCTAATCATTGTGGCAAAGACATGCTACTTTGATCTGTTGTGATTTTGTTTGATTCA-----ATTATT

Section 114

(8363) 8363 8370 8380 8390 8400 8410 8420 8436
Bombus pascuorum chromosome 14 NC_083501.1 region... (7993) GGACTAACACCAAAGTAACCAG--GAAGAAGACATACTCGAGTTGGAATAACAT-TACTATCACAAACTAGTAG
Oryza sativa chromosome 11 NC_029266.1 region from 8... (8020) GTA-TGAGAAGACAATAAGCATTTGTATAATAC-TATTTCATTGTTCACAGGTTCTATGCTGCCGAAGTGGTTG

Section 115

(8437) 8437 8450 8460 8470 8480 8490 8500 8510
Bombus pascuorum chromosome 14 NC_083501.1 region... (8064) ACGAGAAACCCAAGAGAGAAGTAACCCAAGTAATCTTCGACTCTTGTTACGTACTTTGATCACATTGACACTTT
Oryza sativa chromosome 11 NC_029266.1 region from 8... (8092) TTGCACTAG--AATACCTACATTGCCAAGGTACATATACATCATTGTTGATAACTTGCATG-CATTAAAAATTC

Section 116

(8511) 8511 8520 8530 8540 8550 8560 8570 8584
Bombus pascuorum chromosome 14 NC_083501.1 region... (8138) GGTCATCGGATTTCCAATTTGGAATACGAATGTTCGATATAACACGTTTCTTCGTTCTCAATTGTTGTTCGTTC
Oryza sativa chromosome 11 NC_029266.1 region from 8... (8163) TCTTATTTCAATGATCATCAATATATCTAGGGATGCCTATCA-ACTTTACATAAAAAGAACATGGCCTTAATAC

Section 117

(8585) 8585 8590 8600 8610 8620 8630 8640 8658
Bombus pascuorum chromosome 14 NC_083501.1 region... (8212) GTTTTAATTGTCAACGTTCCGACACCAGAGTCAGGCGAAAGAAAAATCATGGAAAAATCACACTGGCGTTCATA
Oryza sativa chromosome 11 NC_029266.1 region from 8... (8236) TCATAATTTATCCACTTT--GACTGCAT------GCCATACATAAAT-ATTGAACCATAACA--GCACTTCTTC

Section 118

(8659) 8659 8670 8680 8690 8700 8710 8720 8732
Bombus pascuorum chromosome 14 NC_083501.1 region... (8286) TGGAAAATTATGTGAGAAGCAAAGAGAGAAAAACTGATTTTCTCGCTCCGTATTATCGGTCCTTTGTTCCAACC
Oryza sativa chromosome 11 NC_029266.1 region from 8... (8299) AATACAGTT----GAATCCTCACAAGAAATCCATGCATTTACTTGGTGGGTAATAGATGTC--TTGTCTCTTTT

Section 119

(8733) 8733 8740 8750 8760 8770 8780 8790 8806
Bombus pascuorum chromosome 14 NC_083501.1 region... (8360) CTATACTCGATAACTCCAGTCCGACGAAAAAGAAGAAGATACTCAAACGCAAATCCACTCTTTGATCAAGCGCA
Oryza sativa chromosome 11 NC_029266.1 region from 8... (8367) TGACAATTCATGAT---ATTTAGTTGGAAAATAGTTACTCCCTCCATTTCAAATT-ACTAGT--------CGTG

Bombus pascuorum chromosome 14 vs. Oryza sativa chromosome 11

Section 120

	(8807)	8807 8820 8830 8840 8850 8860 8870 8880
Bombus pascuorum chromosome 14 NC_083501.1 region...	(8434)	TCGATTCACGTTCAACCAGAGAATTTGCACACGTGTACTCGCGGCGAGAG-TACAAGAACCAGCCTCTCTAAG
Oryza sativa chromosome 11 NC_029266.1 region from 8...	(8429)	TAGATTTGTCCTAAATCAAATATGTTTATCTTTGGCTATTTTTTTAGAAATCTATGTAGTTTAACATGTG-AAT

Section 121

	(8881)	8881 8890 8900 8910 8920 8930 8940 8954
Bombus pascuorum chromosome 14 NC_083501.1 region...	(8507)	AACACGAAAGGGACGACAAAGTCTGCAGGCAATTTCCTCTTTCTTCGGAAACTTTTCCATTTTCTCCTTTCGCT
Oryza sativa chromosome 11 NC_029266.1 region from 8...	(8502)	TACTCCCTCTGTCCCTAAATATTTA-ACGCCGTTGACTTTTT--TAAGCATGTTTAACTGTTTGTCTTATTCAA

Section 122

	(8955)	8955 8960 8970 8980 8990 9000 9010 9028
Bombus pascuorum chromosome 14 NC_083501.1 region...	(8581)	GTACATATTGGTTTTCTCTTCTAGCTCTCCTGGCCAGGACAGACTCCCGGCTAAGAAATTTTTATTTATTAAGA
Oryza sativa chromosome 11 NC_029266.1 region from 8...	(8573)	AAAAATTTAAATAATTATTAATTCTTTTCCTATCATTT---GATTCATTGTTAACTACTTTT-ATGTATACATA

Section 123

	(9029)	9029 9040 9050 9060 9070 9080 9090 9102
Bombus pascuorum chromosome 14 NC_083501.1 region...	(8655)	ATATTCCTTCCGACGGATCTCTCGAAATGCTAGACACGCGTGTAAATACACCTTCCTCGGGAAGCTTTCCACTC
Oryza sativa chromosome 11 NC_029266.1 region from 8...	(8643)	TAGTT--TT---ACATATTTCACAAAAA---AAA------TTTGAATAAAAC---------GAATAGTCAAACAT

Section 124

	(9103)	9103 9110 9120 9130 9140 9150 9160 9176
Bombus pascuorum chromosome 14 NC_083501.1 region...	(8729)	GTTATTTGGATTACAACGTCCGTGCCAGACGGTTGAGGGCAAATGCAGACGAAAATGAAAATATCTTGGCGCTC
Oryza sativa chromosome 11 NC_029266.1 region from 8...	(8695)	GTTTAAAAAAGT-CAACG---GTGTCAAATATTTAGGGACAGAGGGAGTATATAGTATGAAAGTATTTGTCATG

Section 125

	(9177)	9177 9190 9200 9210 9220 9230 9240 9250
Bombus pascuorum chromosome 14 NC_083501.1 region...	(8803)	GTCCAACGAATAAA-ATCAATATCGCCAGTGTTTTTGCTACACGGTGAATGAGATTTTGTTAATGTACACGGTT
Oryza sativa chromosome 11 NC_029266.1 region from 8...	(8765)	GTGGATCTAAAAAAATATCAATCTTATTATGTCAAATATTAAAATGTT--TGACTTAGGACAAACCTAAAATGAC

Section 126

	(9251)	9251 9260 9270 9280 9290 9300 9310 9324
Bombus pascuorum chromosome 14 NC_083501.1 region...	(8876)	AATTCGTTGAAACGTGTTGTCGAGTCGCCGAATTTCATCGTTTTTAGTCGGTTTTTTCTTCGTTGATTCGT-TG
Oryza sativa chromosome 11 NC_029266.1 region from 8...	(8837)	GAGTAATTTAAAACAGGGGAG-GACACTACTTCTAAATGTGTATTAT---TTCCTTCTTTGTCTTCAGGTATA

Bombus pascuorum chromosome 14 vs. Oryza sativa chromosome 11

Section 127

(9325) 9325 9330 9340 9350 9360 9370 9380 9398
Bombus pascuorum chromosome 14 NC_083501.1 region... (8949) ATTCGTTGATTCGTTGATTCGCCGATTCT-TCGTTGCCGCGATATTCTCTCATGGTTGAAAGAAATATCGATCG
Oryza sativa chromosome 11 NC_029266.1 region from 8... (8907) ATATACCGAGACTTAAAACCAGAGAATATCTTACTTCATAGAGATGGGCACATCTCCCTTACAGACTTCGATT-

Section 128

(9399) 9399 9410 9420 9430 9440 9450 9460 9472
Bombus pascuorum chromosome 14 NC_083501.1 region... (9022) TGACAAGTACAAGATATTACTTGCGGTTTATTAGAATTAGTTAGAGTATTTAGGATTAAT-TATCTAGAATTCA
Oryza sativa chromosome 11 NC_029266.1 region from 8... (8980) TGTCTTGTCTAACATCTTGCAGACCGCAGGTAATAATTTGATAAATCTCTTCTCATAAAGTAATATTAATTCA

Section 129

(9473) 9473 9480 9490 9500 9510 9520 9530 9546
Bombus pascuorum chromosome 14 NC_083501.1 region... (9095) TTAAGCGAAGTAAATTGTCACGGTAGCGACGGTTTTTTCTAA--ACGATTTTCTAA-GCTAAAAT-----ACTT
Oryza sativa chromosome 11 NC_029266.1 region from 8... (9054) TGAACCTCTTTCTCTTCCAAAAAGAATCATAAAACGTCACAATTATAATGCGCTAATGAGAAAATGAACCACAT

Section 130

(9547) 9547 9560 9570 9580 9590 9600 9610 9620
Bombus pascuorum chromosome 14 NC_083501.1 region... (9161) CAAGACTAAAGATTTG--GTACAATTAGAATGTTGTGTTCCTTGAATCATCGAATTATCGAATTATTGAAGCAA
Oryza sativa chromosome 11 NC_029266.1 region from 8... (9128) TTGATTTAAATATGAGTAGTAAAATTCACTTGCAGTATCACTGCTTGCAAGGAGGGTGCAGTTTAGTCACTCAT

Section 131

(9621) 9621 9630 9640 9650 9660 9670 9680 9694
Bombus pascuorum chromosome 14 NC_083501.1 region... (9233) CATCTGTATAACACA---CAGTTACGATAATATCCACGCAGTGCTAAAAATATTTTTTATT--ATTATC----G
Oryza sativa chromosome 11 NC_029266.1 region from 8... (9202) CTTGCAAATTGCACAAATAAGTCAC--TCATCTTGCTTCGATGGTGCAAATCTTACCAAATACATTGTCACATG

Section 132

(9695) 9695 9700 9710 9720 9730 9740 9750 9768
Bombus pascuorum chromosome 14 NC_083501.1 region... (9298) GAATAATTGACGGAAATATTTTAGAT-ATATTCGATGAAATGGATATTTGTTCCTAAAAGAATGTCTGTTCAAA
Oryza sativa chromosome 11 NC_029266.1 region from 8... (9274) GATTATTTGTTTTTGA-ATTGGAGGTCATATTGACCTAACATCATGCTTTTTT-TAGCCATTTGGCAAGGCAAG

Section 133

(9769) 9769 9780 9790 9800 9810 9820 9830 9842
Bombus pascuorum chromosome 14 NC_083501.1 region... (9371) TTTGGAAAACTTTACGGAATTTGTCTAAAAATTGTCAGCGGTTAACGGACGTTCTCCCCCT-TC---GTACTTT
Oryza sativa chromosome 11 NC_029266.1 region from 8... (9346) CATTAAAG---TTACAAACTAATACTCATTTTTTTCCATGTATATGCTAC-TACTCCCGCTGTCAATGAACACG

Bombus pascuorum chromosome 14 vs. Oryza sativa chromosome 11

```
                                                                        Section 134
                                                            (9843)  9843      9916
Bombus pascuorum chromosome 14 NC_083501.1 region...        (9441)  CTCTTATTTTTCTGCTCTCTTATGACTGTTTAGCATGCTATCGTTGAACTCCCTAACGTT--TATGTAACATCT
Oryza sativa chromosome 11 NC_029266.1 region from 8...     (9416)  ACCATATTGTGCTTGTCAATCAATAACAAAATTCATCTTTAATTGGATTTTACTACTGGTGGTATTGCACGTTC
                                                                        Section 135
                                                            (9917)  9917      9990
Bombus pascuorum chromosome 14 NC_083501.1 region...        (9513)  TTTTGTTTA---AT--TGTACCCA----TAGAATACACCATATAATATACCCGGAAAGTTTGGCTGCTGAACCT
Oryza sativa chromosome 11 NC_029266.1 region from 8...     (9490)  TCTTATTTATGGATCATGTGACCATGTTTGCAAAACAAATTAACATAGAGCATAACTTCATTGCGAACAACCCA
                                                                        Section 136
                                                            (9991)  9991      10064
Bombus pascuorum chromosome 14 NC_083501.1 region...        (9578)  T--GGAACAGCCTGTATATAAATATACGTTTAGAAAAACGATAAGAAAAGCGTAAC-GAGTAATTCAT----TC
Oryza sativa chromosome 11 NC_029266.1 region from 8...     (9564)  CAAGGCACATGTTTTATGTGCTT-TGCACCCCCTAAAACAAGATGAGTGGCTTGTTTGAGCATTTTACAATATG
                                                                        Section 137
                                                           (10065)  10065     10138
Bombus pascuorum chromosome 14 NC_083501.1 region...        (9645)  GCCAATTATAGTCGAGATAAAAGACCTGGAA-GGTACTGGGGAAGCGTGATTGCAATGGGATTTCAGATGA-CA
Oryza sativa chromosome 11 NC_029266.1 region from 8...     (9637)  GGTGACTAAATTGCACCCACCTCACAAGTTGCGGTACTAGCGTTGCATTTTACTCATTAAACATGCTTTGCTCA
                                                                        Section 138
                                                           (10139)  10139     10212
Bombus pascuorum chromosome 14 NC_083501.1 region...        (9717)  G-GTGCGGCGAGAGGGTGAGGGCGAAGG-CAATGGAGGAAAACA-AGGAGATGGATCAAGTTCAGCGGAAAACC
Oryza sativa chromosome 11 NC_029266.1 region from 8...     (9711)  TCGTGTGGTAAGTTTTTTTTTTTTAACAACAGTATACTAATCCTCAAGGGATAGCACATATTATTATACTAGTG
                                                                        Section 139
                                                           (10213)  10213     10286
Bombus pascuorum chromosome 14 NC_083501.1 region...        (9788)  TAGAACTCTACACGCTGGGGGCGGGGAGCAAGCGGATTGTGTAATAGGGCCATTGGCATCGGTCCAAAGTGCA-
Oryza sativa chromosome 11 NC_029266.1 region from 8...     (9785)  TAATATTGTTTTCAATCTATCGTACACTTCAGCATGTATGTCAATAAGTTCATTATCATC---CCAAGTTACAA
                                                                        Section 140
                                                           (10287)  10287     10360
Bombus pascuorum chromosome 14 NC_083501.1 region...        (9861)  ---CGATCTCCGTGGCCGTGCCAGAGCCGCCAGAGCCGTCAGAGCTCCAGAATCCAACGGCAATGCAGAGTGCA
Oryza sativa chromosome 11 NC_029266.1 region from 8...     (9856)  TAAGTATTTTTATGTTATTTGGTAGGTCTTTCTTCCCGAAGATGCTGATGAGAAAAAAGGGAGAAAAAACGGGA
```

Section 141

```
                                                         10361    10370     10380     10390     10400     10410     10420         10434
                                               (10361)
Bombus pascuorum chromosome 14 NC_083501.1 region... (9932) GGAG---GATCGCCGACGGAGAACTCTAGGTCGAGTCGAACCCGTGT-TGGAAATTGGGTCATACAGCGAGCAC
Oryza sativa chromosome 11 NC_029266.1 region from 8... (9930) GTTACCCCATATTTTTTGCTGAACCTATGCGAGCATCCAATTCATTTGTTGGCACGGAGGAGTACATTGCGC--
```
