# Supplementary material for: Patch type nucleotide sequence identities between genomes from many different species facilitate illegitimate recombination
Source: Sci Rep. 2026 Mar 30;16:10524. doi: 10.1038/s41598-026-44124-0 (PMC13035915; doi:10.1038/s41598-026-44124-0)
Supplement: Supplementary file 6 — Supplementary Material 6 [file 41598_2026_44124_MOESM6_ESM.pdf]

Homo sapiens mitochondrion, complete genome vs. Latimeria chalumnae mitochondrion

```
                                                                  Section 1
                                          (1) 1        10        20        30        40        50        60            75
Homo sapiens mitochondrion, complete genome NC_01...  (1) GATCACAGGTCTATCACCCTATTAACCACTCACGGGAGCTCTCCATGCATTTGGTATTTTCGTCTGGGGGGTATG
Latimeria chalumnae mitochondrion NC_001804.1  (1) ---------------------------------------------------------------------------
                                                                  Section 2
                                          (76) 76            90        100       110       120       130       140       150
Homo sapiens mitochondrion, complete genome NC_01...  (76) CACGCGATAGCATTGCGAGACGCTGGAGCCGGAGCACCCTATGTCGCAGTATCTGTCTTTGATTCCTGCCTCATC
Latimeria chalumnae mitochondrion NC_001804.1  (1) ---------------------------------------------------------------------------
                                                                  Section 3
                                          (151) 151      160       170       180       190       200       210           225
Homo sapiens mitochondrion, complete genome NC_01...  (151) CTATTATTTATCGCACCTACGTTCAATATTACAGGCGAACATACTTACTAAAGTGTGTTAATTAATTAATGCTTG
Latimeria chalumnae mitochondrion NC_001804.1  (1) ---------------------------------------------------------------------------
                                                                  Section 4
                                          (226) 226           240       250       260       270       280       290       300
Homo sapiens mitochondrion, complete genome NC_01...  (226) TAGGACATAATAATAACAATTGAATGTCTGCACAGCCACTTTCCACACAGACATCATAACAAAAAATTTCCACCA
Latimeria chalumnae mitochondrion NC_001804.1  (1) ---------------------------------------------------------------------------
                                                                  Section 5
                                          (301) 301      310       320       330       340       350       360           375
Homo sapiens mitochondrion, complete genome NC_01...  (301) AACCCCCCCTCCCCCGCTTCTGGCCACAGCACTTAAACACATCTCTGCCAAACCCCAAAAACAAAGAACCCTAAC
Latimeria chalumnae mitochondrion NC_001804.1  (1) ---------------------------------------------------------------------------
                                                                  Section 6
                                          (376) 376           390       400       410       420       430       440       450
Homo sapiens mitochondrion, complete genome NC_01...  (376) ACCAGCCTAACCAGATTTCAAATTTTATCTTTTGGCGGTATGCACTTTTAACAGTCACCCCCCAACTAACACATT
Latimeria chalumnae mitochondrion NC_001804.1  (1) ---------------------------------------------------------------------------
                                                                  Section 7
                                          (451) 451      460       470       480       490       500       510           525
Homo sapiens mitochondrion, complete genome NC_01...  (451) ATTTTCCCCTCCCACTCCCATACTACTAATCTCATCAATACAACCCCCGCCCATCCTACCCAGCACACACACACC
Latimeria chalumnae mitochondrion NC_001804.1  (1) ---------------------------------------------------------------------------
```

Homo sapiens mitochondrion, complete genome vs. Latimeria chalumnae mitochondrion

```
                                                                                                        Section 8
                                                           (526) 526          540       550       560       570       580       590     600
Homo sapiens mitochondrion, complete genome NC_01...       (526) GCTGCTAACCCCATACCCCGAACCAACCAAACCCCAAAGACACCCCCCACAGTTTATGTAGCTTACCTCCTCAAA
    Latimeria chalumnae mitochondrion NC_001804.1            (1) ---------------------------------------------------GTGAGTGTAGCTTAATAA---AAA
                                                                                                        Section 9
                                                           (601) 601      610       620       630       640       650       660          675
Homo sapiens mitochondrion, complete genome NC_01...       (601) GCAATACACTGAAAATGTTTAGACGGGCT-CACATCACCCCATAAACAAATAGGTTTGGTCCTAGCCTTTCTATT
    Latimeria chalumnae mitochondrion NC_001804.1           (22) GCATAGCACTGAAGATGCTAAGATGAATTACAAAATATTCCACTGACACAAAGGTTTGGTCCCAGCCTTGCTATC
                                                                                                        Section 10
                                                           (676) 676          690       700       710       720       730       740     750
Homo sapiens mitochondrion, complete genome NC_01...       (675) AGCTCTTAGTAAGATTACACATGCAAGCATCCCCGTTCCAGTGAGTTCACCCTCTAAATCACC----ACGATCAA
    Latimeria chalumnae mitochondrion NC_001804.1           (97) AATTTTAACCAGGATTACACATGCAAGCATCAACTCCCCAGTGAGAATGCCCCTGACTTATCCGTCAAAGATAAC
                                                                                                        Section 11
                                                           (751) 751      760       770       780       790       800       810          825
Homo sapiens mitochondrion, complete genome NC_01...       (746) AAGGAACAAGCATCAAGCACGCAGCAAT---GC-AGCTCAAAACGCTTAGCCTAGCCACACCCCCACGGGAAA-C
    Latimeria chalumnae mitochondrion NC_001804.1          (172) AGGGAGTAGGTATCAGGCACACAACATTAACGCTAGCCCAAGACACCTTGTCCAGCCACACCCCCAAGGGAACTC
                                                                                                        Section 12
                                                           (826) 826          840       850       860       870       880       890     900
Homo sapiens mitochondrion, complete genome NC_01...       (816) AGCAGTGATTAACCTTTAGCAATAAACGAAAGTTTAACTAAGCTATACTAACCCCAGGGTTGGTCAATTTCGTGC
    Latimeria chalumnae mitochondrion NC_001804.1          (247) AGCAGTGATAGACATTGAATAATAAGTGAAAACTTGACTCAGCCATGGTTACA--AGGGCCGGTCAACTCCGTGC
                                                                                                        Section 13
                                                           (901) 901      910       920       930       940       950       960          975
Homo sapiens mitochondrion, complete genome NC_01...       (891) CAGCCACCGCGGTCACACGATTAACCCAAGTCAATAGAAG--CCGGCGTAAAGAGTGTTTTAGATCACCCCCTCC
    Latimeria chalumnae mitochondrion NC_001804.1          (320) CAGCCACCGCGGTTACACGGAAGACCCAAAATGATAACACTACCGGCGTAAAGCGTGATT-AAAGGACACCCACC
                                                                                                        Section 14
                                                           (976) 976          990       1000      1010      1020      1030      1040   1050
Homo sapiens mitochondrion, complete genome NC_01...       (964) CCAATAAAGCTAAAACTCACCTGAGTTGTAAAAAACTCCAGTTGACACAAAATAGACT----ACGAAAGTGGCTT
    Latimeria chalumnae mitochondrion NC_001804.1          (394) ATAATGGAGCCACAAATAACTAAAGCTGTTATACGC---ACTTAAAAAAATATGCTCATCACACGAAAGTAACTC
```

Section 15

```
                                                        (1051) 1051     1060      1070      1080      1090      1100      1110          1125
Homo sapiens mitochondrion, complete genome NC_01...    (1035) TAACATAT---------CTGAACACACAATAGCTAAGACCCAAACTGGGATTAGATACCCCACTATGCTTAGCCC
Latimeria chalumnae mitochondrion NC_001804.1            (466) CAGCACCCAAAGGAACCCTGAACCCACGAAAGCTAAGAAACAAACTGGGATTAGATACCCCACTATGCTCAGCCC
```

Section 16

```
                                                        (1126) 1126          1140      1150      1160      1170      1180      1190    1200
Homo sapiens mitochondrion, complete genome NC_01...    (1101) TAAACCTCAACAGTTAAATCAACAAAACTGCTCGCCAGAACA-CTACGAGCCACAGCTTAAAACTCAAAGGACCT
Latimeria chalumnae mitochondrion NC_001804.1            (541) TAAACACAAACAATTCAAACA--CACACTGTTCGCCAGGGGAACTACAAGCGCCAGCTTCAAACCCAAAGGACTT
```

Section 17

```
                                                        (1201) 1201     1210      1220      1230      1240      1250      1260          1275
Homo sapiens mitochondrion, complete genome NC_01...    (1175) GGCGGTGCTTCATATCCCTCTAGAGGAGCCTGTTCTGTAATCGATAAACCCCGATCAACCTCACCACCTCTTGCT
Latimeria chalumnae mitochondrion NC_001804.1            (614) GGCGGCACTTCAAACCCACCTAGAGGAGCCTGTTCTAAAACTGACAACCCCCACCTAACCTCACCATCCCTAGCC
```

Section 18

```
                                                        (1276) 1276          1290      1300      1310      1320      1330      1340    1350
Homo sapiens mitochondrion, complete genome NC_01...    (1250) -------CAGCCTATATACCGCCATCTTCAGCAAACCCTGATGAAGGCTACAAAGTAAGCGCAAGTACCCACGT-
Latimeria chalumnae mitochondrion NC_001804.1            (689) ATTAAACCAGCCTATATACCGCCGTCGCCAGCCCACCCTG-TGAAGGAAATACAATGGGCAAAAATAAAAAAATT
```

Section 19

```
                                                        (1351) 1351     1360      1370      1380      1390      1400      1410          1425
Homo sapiens mitochondrion, complete genome NC_01...    (1317) AAAGACGTTAGGTCAAGGTGTAGCCCATGAGGTGGCAAGAAATGGGCTACATTTTCTACCCC-AGAAAACTACGA
Latimeria chalumnae mitochondrion NC_001804.1            (763) AAAAACGTCAGGTCGAGGTGTAGCAAATGAGATGGGAAGAAATGGGCTACATTTTCTAAATATAGAATATTACGA
```

Section 20

```
                                                        (1426) 1426          1440      1450      1460      1470      1480      1490    1500
Homo sapiens mitochondrion, complete genome NC_01...    (1391) TAGCCCTTAT-GAAACTTAAGGGTCGAAGGTGGATTTAGCAGTAAACTAAGAGTAGAGTGCTTAGTTGAACAGGG
Latimeria chalumnae mitochondrion NC_001804.1            (838) AAAAATACAGCGAAACCTGTACTTTGAAGGAGGATTTAGCAGTAAAAGGGGAATAGAGAGCCCCTCTGAAACCGG
```

Section 21

```
                                                        (1501) 1501     1510      1520      1530      1540      1550      1560          1575
Homo sapiens mitochondrion, complete genome NC_01...    (1465) CCCTGAAGCGCGTACACACCGCCCGTCACCCTCCTCAAGTATACTTCAAAGGACATTTAACTAAA---ACCCCTA
Latimeria chalumnae mitochondrion NC_001804.1            (913) CCCTGAAATGCGCACACACCGCCCGTCACTCTCCTCACCCAAAATCGGCCCCATCTTTTAATAAACAAAAAACCA
```

Section 22

```
                                                         (1576) 1576         1590      1600      1610      1620      1630      1640      1650
Homo sapiens mitochondrion, complete genome NC_01...     (1537) CGCATTTATATAGAGGAGACAAGTCGTAACATGGTAAGTGTACTGGAAAGTGCACTTGGACGAACCAGAGTGTAG
     Latimeria chalumnae mitochondrion NC_001804.1        (988) AGCATACAAGTAGAGGAGGCAAGTCGTAACAAGGTAAGTGTACCGGAAGGTGCACTTGGACTAATCAAAATGTAG
```

Section 23

```
                                                         (1651) 1651     1660      1670      1680      1690      1700      1710          1725
Homo sapiens mitochondrion, complete genome NC_01...     (1612) CTTAACACAAAGCACCCAACTTACACTTAGGAGATTTCAACTTAACTTGACCGCTCTGAGCTAAAC--CTAGCCC
     Latimeria chalumnae mitochondrion NC_001804.1       (1063) TTTAACAAAA--CACCTCCCTTACACTGAGAAGACACCCACGAAAGCGGGTCATTTTGAGCTATATAGCTAGGCC
```

Section 24

```
                                                         (1726) 1726         1740      1750      1760      1770      1780      1790      1800
Homo sapiens mitochondrion, complete genome NC_01...     (1685) --------CAAACCCACTCCACCTT--ACTACCAG-----ACAACCTTAGCC-AAACCATTTACCCAAATAAAGT
     Latimeria chalumnae mitochondrion NC_001804.1       (1136) AACAAAACCATAACCACTACACCATTAACCAAAAACTTGAACAAACAAAACCTAAACCATTTACC--ATCCAAGT
```

Section 25

```
                                                         (1801) 1801     1810      1820      1830      1840      1850      1860          1875
Homo sapiens mitochondrion, complete genome NC_01...     (1744) ATAGGCGATAGAAATTGAAACCTGGCGCAATAGATATAGTACCGCAAGGAAAGATGAAAAATTAT--AACCAA-
     Latimeria chalumnae mitochondrion NC_001804.1       (1209) ATAGGCGATAGAAAA-GACACCAGGCACAATAGTAAAAGTACCGCAAGGAACGCTGAAAAAGAAATGAAACAAC
```

Section 26

```
                                                         (1876) 1876         1890      1900      1910      1920      1930      1940      1950
Homo sapiens mitochondrion, complete genome NC_01...     (1816) --------GCATAATATAGCAAGGACTAACCCCTATACCTTCTGCATAATGAATTAACTAGAAATAACTTTGCAA
     Latimeria chalumnae mitochondrion NC_001804.1       (1283) TCGTAAAAGCAATAAAAACCAAAGACTAACCCTTGTACCTTTTGCATTATGATCTAGTTAGACCCCACCGGGCAA
```

Section 27

```
                                                         (1951) 1951     1960      1970      1980      1990      2000      2010          2025
Homo sapiens mitochondrion, complete genome NC_01...     (1883) GGAGAGCCAAAGCTAAGACCCCCGAAACCAGACGAGCTACCTAAGAACAGC-TAAAAGAGCACACCCGTCTATGT
     Latimeria chalumnae mitochondrion NC_001804.1       (1358) AATGAATTTAAGTCCAACCCCCGAAACTAAGTGAGCTACTTCGAAACAGCCTATGAGGGCAAACCCTTCTCTGT
```

Section 28

```
                                                         (2026) 2026         2040      2050      2060      2070      2080      2090      2100
Homo sapiens mitochondrion, complete genome NC_01...     (1957) AGCAAAATAGTGGAAGATTTATAGGTAGAGGCGACAAACCTACCGAGCCTGGTGATAGCTGGTTGTCCAAGATA
     Latimeria chalumnae mitochondrion NC_001804.1       (1433) GGCAAAAGAGTGGAAGATTTCCAAGTAGAGGCGATAAACCTAACGAGCTTAGTGATAGCTGGTTATTCGAGAAA
```

Section 29

	(2101)	2101 2110 2120 2130 2140 2150 2160 2175
Homo sapiens mitochondrion, complete genome NC_01...	(2032)	-GAATCTTAGTTCAACTT-TAAATTTGCCCACAGAACCCTCTAAATCCCCTTGTAAATTTAACTG--TTAGTCCA
Latimeria chalumnae mitochondrion NC_001804.1	(1508)	TGATTTTTAGTTCAGCCCCTCGGCCTCCAAACCCTAACAACATAAAACCAATGTGAGCCAGAGAGAGTTATTCAA

Section 30

	(2176)	2176 2190 2200 2210 2220 2230 2240 2250
Homo sapiens mitochondrion, complete genome NC_01...	(2103)	AAGAGGAACAGCTCTTTGGACACTAGGAAAAAACCTTGTA-GAGAGAGTAAAAAAT---TTAACAC---------
Latimeria chalumnae mitochondrion NC_001804.1	(1583)	AGGGGGTACAGCCCCTTTGA-AAAAGGACACAACCTTCTAAGACAGGATAAAGACTATATTAACTCAAGGATCAG

Section 31

	(2251)	2251 2260 2270 2280 2290 2300 2310 2325
Homo sapiens mitochondrion, complete genome NC_01...	(2165)	---CCATAGTAGGCCTAAAAGCAGCCACCAATTAAGAAAGCGTTCAAGCTCAACACCCACTAC-CTAAAAAATCC
Latimeria chalumnae mitochondrion NC_001804.1	(1657)	CTGCCCAAGTGGACCTAAAAGCAGTCACC--T-AAAAAAGCGTTAAAGCTTAAGCAGCACTAAACCAACCTATAC

Section 32

	(2326)	2326 2340 2350 2360 2370 2380 2390 2400
Homo sapiens mitochondrion, complete genome NC_01...	(2236)	CAA-ACATATAACTGAACTCCTCACACCCAATTGGACCAATCTATCACCCTATAGAAGAACTAATGTTAGTATAA
Latimeria chalumnae mitochondrion NC_001804.1	(1729)	TAATAAATTTAACCTCATTCCACCATCACTATCGAATTATTCTATATGC--ATAGAAGAATAAATGCTAGAATTA

Section 33

	(2401)	2401 2410 2420 2430 2440 2450 2460 2475
Homo sapiens mitochondrion, complete genome NC_01...	(2310)	GTAACATGAA--------AACATTCTCCTCC-GCATAAGCCTGCGTCAGATTA----AAACACTGA--ACTGACA
Latimeria chalumnae mitochondrion NC_001804.1	(1802)	GTAACAAGAAGGCCATTAAGCCTTCTCTAACTGCATAAGTGTACATCAGATTAGATTAACCACTGATAATTAACG

Section 34

	(2476)	2476 2490 2500 2510 2520 2530 2540 2550
Homo sapiens mitochondrion, complete genome NC_01...	(2370)	ATT---AACAGCCCAATATCTACAATCAACCAACAAGTCATTATTACCCTCACTGTCAACCCAACACAGGCATGC
Latimeria chalumnae mitochondrion NC_001804.1	(1877)	ACTTCAAAGAGAATACTATG-ACATAAAACAAGAAAAGCACACACGCCCACATCGTTAATCCAACACAGGAATGC

Section 35

	(2551)	2551 2560 2570 2580 2590 2600 2610 2625
Homo sapiens mitochondrion, complete genome NC_01...	(2442)	TCATAAGGAAAGGTTAAAAAAAGTAAAAGGAACTCGGCAAATCTTA--CCCCGCCTGTTTACCAAAAACATCACC
Latimeria chalumnae mitochondrion NC_001804.1	(1951)	A-ACCACGAAAGATTAAAAGAAAGAAAAGGAACTCGGCAAACTATAAGCCCCGCCTGTTTACCAAAAACATCGCC

Homo sapiens mitochondrion, complete genome vs. Latimeria chalumnae mitochondrion

```
                                                          Section 36
                                                   (2626) 2626          2640      2650      2660      2670      2680      2690    2700
Homo sapiens mitochondrion, complete genome NC_01... (2515) TCTAGC----ATCACCAGTATTAGAGGCACCGCCTGCCCAGTGACACATGTTTAACGGCCGCGGTACCCTAACCG
   Latimeria chalumnae mitochondrion NC_001804.1 (2025) TCCCGCCAACAACAGAAGTATTGGAGGTCCCGCCTGCCCAGTGACA-AGATTTAACGGCCGCGGTATCCTGACCG
                                                          Section 37
                                                   (2701) 2701     2710      2720      2730      2740      2750      2760          2775
Homo sapiens mitochondrion, complete genome NC_01... (2586) TGCAAAGGTAGCATAATCACTTGTTCCTTAAATAGGGACCTGTATGAATGGCTCCACGAGGGTTCAGCTGTCTCT
   Latimeria chalumnae mitochondrion NC_001804.1 (2099) TGCAAAGGTAGCGAAATCACTTGTCTTTTAAATGAAGACCTGTATGAATGGCACCACGAGGGCTTAACTGTCTCC
                                                          Section 38
                                                   (2776) 2776          2790      2800      2810      2820      2830      2840    2850
Homo sapiens mitochondrion, complete genome NC_01... (2661) TACTTTTAACCAGTGAAATTGACCTGCCCGTGAAGAGGCGGGCATAACACAGCAAGACGAGAAGACCCTATGGAG
   Latimeria chalumnae mitochondrion NC_001804.1 (2174) TCTTTCCAATCAGTAAAATTGATCTGTCCGTGCAGAAGCGGACTTAACTACATTAGACGAGAAGACCCTGTGGAG
                                                          Section 39
                                                   (2851) 2851     2860      2870      2880      2890      2900      2910          2925
Homo sapiens mitochondrion, complete genome NC_01... (2736) CTTTA-----------ATTTATTAATGCAAACAGTACCTAACAAACCCACAGGTCCTAAACTACCAAACCTGCAT
   Latimeria chalumnae mitochondrion NC_001804.1 (2249) CTTCAGACACAAAGCCAACTACAAATAAACACT-TAGATAATAAATTGATAAAACCAGTAGCCTAATACTGGCCC
                                                          Section 40
                                                   (2926) 2926          2940      2950      2960      2970      2980      2990    3000
Homo sapiens mitochondrion, complete genome NC_01... (2800) TAAAAATTTCGGTTGGGGCGACCTCGGAGCAGAACCCAACCTCCGAGCAGT--------ACATG--CTAAGACT-
   Latimeria chalumnae mitochondrion NC_001804.1 (2323) TATTGTCTTTGGTTGGGGCGACCACGGAGAAAAAACAATCCTCCAAGCCGATTGGTACCACCTGTACTAAAACAA
                                                          Section 41
                                                   (3001) 3001     3010      3020      3030      3040      3050      3060          3075
Homo sapiens mitochondrion, complete genome NC_01... (2864) ----TCACCAGTCAAAGCGA-ACTACTATACTC--AATTGATCCAATAACT-----TGACCAACGGAACAAGTTA
   Latimeria chalumnae mitochondrion NC_001804.1 (2398) AGGGTAACACCCCAAAGTAATAAAAATTTTATCGGACATGACCCAGGGACTAAACCTGATCAACGAACCAAGTTA
                                                          Section 42
                                                   (3076) 3076          3090      3100      3110      3120      3130      3140    3150
Homo sapiens mitochondrion, complete genome NC_01... (2927) CCCTAGGGATAACAGCGCAATCCTATTCTAGAGTCCATATCAACAATAGGGTTTACGACCTCGATGTTGGATCAG
   Latimeria chalumnae mitochondrion NC_001804.1 (2473) CCCCAGGGATAACAGCGCAATCCTTTCCAAGAGTCCAAATCGACGAAAGGGTTTACGACCTCGATGTTGGATCAG
```

Homo sapiens mitochondrion, complete genome vs. Latimeria chalumnae mitochondrion

Section 43

```
                                                               3151     3160      3170      3180      3190      3200      3210          3225
                                                       (3151)
Homo sapiens mitochondrion, complete genome NC_01...   (3002)  GACATCCCGATGGTGCAGCCGCTATTAAAGGTTCGTTTGTTCAACGATTAAAGTCCTACGTGATCTGAGTTCAGA
Latimeria chalumnae mitochondrion NC_001804.1          (2548)  GACACCCCAATGGTGAAGCCGCTATTAAAGGTTTGTTTGTTCAACAATTAAAGTCCTACGTGATCTGAGTTCAGA
```

Section 44

```
                                                               3226          3240      3250      3260      3270      3280      3290    3300
                                                       (3226)
Homo sapiens mitochondrion, complete genome NC_01...   (3077)  CCGGAGTAATCCAGGTCGGTTTCTATCTACNTTCAAATTCCTCCCTGTACGAAAGGACAAGAGAAATAAGGCCTA
Latimeria chalumnae mitochondrion NC_001804.1          (2623)  CCGGAGAAATCCAGGTCAGTTTCTATCTATGATGTTAATTCTCCCAGTACGAAAGGACCGGAGAACTTGAGCCAA
```

Section 45

```
                                                               3301     3310      3320      3330      3340      3350      3360          3375
                                                       (3301)
Homo sapiens mitochondrion, complete genome NC_01...   (3152)  CTTCACAAAGCGCCTTCCCCCGTAA----ATGATATCATCTCAA-CTTAGTATTATACCCACACCCACCC-----
Latimeria chalumnae mitochondrion NC_001804.1          (2698)  TGCCACAA-GCACGCTCATCTTCAACCTGATGAAAACAACTAAAACAGATAAAGAAGAACACAACCGCTCCCGTA
```

Section 46

```
                                                               3376          3390      3400      3410      3420      3430      3440    3450
                                                       (3376)
Homo sapiens mitochondrion, complete genome NC_01...   (3217)  AAGAACAGGGTTTGTTAAGATGGCAGAGCCCGG-TAATCGCATAAAACTTAAAACTTTACAGTCAGAGGTTCAAT
Latimeria chalumnae mitochondrion NC_001804.1          (2772)  AACAACGGGCAATGCCGGGGTGGCAGAGCCTGGCTAAATGCAAGAGGCCTAAACTCTCTCTCTCAGAAGTTCAAA
```

Section 47

```
                                                               3451     3460      3470      3480      3490      3500      3510          3525
                                                       (3451)
Homo sapiens mitochondrion, complete genome NC_01...   (3291)  TCCTCTTCTTA------ACAACA-----TACCCA--TGGCCAACCTCCTACTCCTCATTGTACCCATTCTAATCG
Latimeria chalumnae mitochondrion NC_001804.1          (2847)  TCCTCTCCCCAGCTATGACAAAAATTATTACACACCTGCTAAACCCACTAGCTGTCATCATTCCAATTTTACTAG
```

Section 48

```
                                                               3526          3540      3550      3560      3570      3580      3590    3600
                                                       (3526)
Homo sapiens mitochondrion, complete genome NC_01...   (3353)  CAATGGCATTCCTAATGCTTACCGAACGAAAAATTCTAGGCTATATACAACTACGCAAAGGCCCCAACGTTGTAG
Latimeria chalumnae mitochondrion NC_001804.1          (2922)  CCGTAGCGTTCCTGACACTCATCGAACGAAAAGTACTAGGTTATATGCAACTACGAAAGGGTCCTAATATCGTAG
```

Section 49

```
                                                               3601     3610      3620      3630      3640      3650      3660          3675
                                                       (3601)
Homo sapiens mitochondrion, complete genome NC_01...   (3428)  GCCCCTACGGGCTACTACAACCCTTCGCTGACGCCATAAAACTCTTCACCAAAGAGCCCCTAAAACCCGCCACAT
Latimeria chalumnae mitochondrion NC_001804.1          (2997)  GCCCATATGGTCTCCTACAACCCCTAGCAGATGGACTAAAACTATTCATTAAGGAGCCAGTACGACCTTCTACAT
```

Section 50

	(3676)	3676 3690 3700 3710 3720 3730 3740 3750
Homo sapiens mitochondrion, complete genome NC_01...	(3503)	CTACCATCACCCTCTACATCACCGCCCCGACCTTAGCTCTCACCATCGCTCTTCTACTATGAACCCCCCTCCCCA
Latimeria chalumnae mitochondrion NC_001804.1	(3072)	CATCCCCGCTACTCTTCATTACCACTCCAATACTAGCACTCACCATAGCACTAACTCTATGACTACCACTACCCC

Section 51

	(3751)	3751 3760 3770 3780 3790 3800 3810 3825
Homo sapiens mitochondrion, complete genome NC_01...	(3578)	TACCCAACCCCCTGGTCAACCTCAACCTAGGCCTCCTATTTATTCTAGCCACCTCTAGCCTAGCCGTTTACTCAA
Latimeria chalumnae mitochondrion NC_001804.1	(3147)	TCCCACATCCAATAACAAACCTAAACCTAGGAATATTATTTATCCTAGCAATCTCAAGTCTAACAGTATACTCAA

Section 52

	(3826)	3826 3840 3850 3860 3870 3880 3890 3900
Homo sapiens mitochondrion, complete genome NC_01...	(3653)	TCCTCTGATCAGGGTGAGCATCAAACTCAAACTACGCCCTGATCGGCGCACTGCGAGCAGTAGCCCAAACAATCT
Latimeria chalumnae mitochondrion NC_001804.1	(3222)	TTCTAGGCTCCGGCTGAGCATCAAACTTAAAATATGCCCTAATTGGGGCCCTCCGAGCAGTCGCACAAACAATCT

Section 53

	(3901)	3901 3910 3920 3930 3940 3950 3960 3975
Homo sapiens mitochondrion, complete genome NC_01...	(3728)	CATATGAAGTCACCCTAGCCATCATTCTACTATCAACATTACTAATAAGTGGCTCCTTTAACCTCTCCACCCTTA
Latimeria chalumnae mitochondrion NC_001804.1	(3297)	CCTATGAAGTAAGCCTAGGACTTATCCTACTGGCCATAATCATCTTCGCAGGCGGTTTTACACTAACAACATTTA

Section 54

	(3976)	3976 3990 4000 4010 4020 4030 4040 4050
Homo sapiens mitochondrion, complete genome NC_01...	(3803)	TCACAACACAAGAACACCTCTGATTACTCCTGCCATCATGACCCTTGGCCATAATATGATTTATCTCCACACTAG
Latimeria chalumnae mitochondrion NC_001804.1	(3372)	ATACATCACAGGAAACCATTTGACTTCTAACGCCAGGATGACCACTCGCAGCAATATGATACATCTCAACCTTAG

Section 55

	(4051)	4051 4060 4070 4080 4090 4100 4110 4125
Homo sapiens mitochondrion, complete genome NC_01...	(3878)	CAGAGACCAACCGAACCCCCTTCGACCTTGCCGAAGGGGAGTCCGAACTAGTCTCAGGCTTCAACATCGAATACG
Latimeria chalumnae mitochondrion NC_001804.1	(3447)	CAGAAACCAACCGAGCCCCATTTGACCTCACAGAAGGAGAATCAGAACTTGTATCGGGGTTTAATGTGGAATATG

Section 56

	(4126)	4126 4140 4150 4160 4170 4180 4190 4200
Homo sapiens mitochondrion, complete genome NC_01...	(3953)	CCGCAGGCCCCTTCGCCCTATTCTTCATAGCCGAATACACAAACATTATTATAATAAACACCCTCACCACTACAA
Latimeria chalumnae mitochondrion NC_001804.1	(3522)	CAGGAGGACCATTTGCACTATTCTTTCTAGCAGAATATGCAAATATTCTACTAATAAACACACTATCAACTATTC

Homo sapiens mitochondrion, complete genome vs. Latimeria chalumnae mitochondrion

Section 57

		(4201) 4201 … 4275
Homo sapiens mitochondrion, complete genome NC_01...	(4028)	TCTTCCTAGGAACAACATATGACGCACTCTCCCCTGAACTCTACACAACATATTTTGTCACCAAGACCCTACTTC
Latimeria chalumnae mitochondrion NC_001804.1	(3597)	TATTTATAGGAGCCATACACAACCCAATCACACCAGAACTAACCTCAATTAACCTAATGATTAAAGCCTCCGCAC

Section 58

		(4276) 4276 … 4350
Homo sapiens mitochondrion, complete genome NC_01...	(4103)	TAACCTCCCTGTTCTTATGAATTCGAACAGCATACCCCCGATTCCGCTACGACCAACTCATACACCTCCTATGAA
Latimeria chalumnae mitochondrion NC_001804.1	(3672)	TATCAATACTCTTCCTATGAGTACGAGCCTCATACCCACGATTCCGATATGACCAGCTAATACACTTAGTATGAA

Section 59

		(4351) 4351 … 4425
Homo sapiens mitochondrion, complete genome NC_01...	(4178)	AAAACTTCCTACCACTCACCCTAGCATTACTTATATGATATGTCTCCATACCCATTACAATCTCCAGCATTCCCC
Latimeria chalumnae mitochondrion NC_001804.1	(3747)	AAAACTTCTTACCAATCACCCTAGCCATGATCCTATGACACACCTCCCTTCCAATTTTTACAGGAAGCTTACCAC

Section 60

		(4426) 4426 … 4500
Homo sapiens mitochondrion, complete genome NC_01...	(4253)	CTCAAACCTAA--GAAATATGTCTGA-TAAAAGAGTT--ACTTTGATAGAGTAAATAATAGGAGCTTAAACCCCC
Latimeria chalumnae mitochondrion NC_001804.1	(3822)	CACAAACCTAAAGGAAACGTGCCCGAATAAACAAGGACCACTTTGATAGAGTGGACTATAGGAGTTAAAACCTCC

Section 61

		(4501) 4501 … 4575
Homo sapiens mitochondrion, complete genome NC_01...	(4323)	TTATTTC-TAGGACTATGAGAATCGAACCCATCCCTGAGAATCCAAAATTCTCCGTGCCACCTATCACACCCCAT
Latimeria chalumnae mitochondrion NC_001804.1	(3897)	TCGCTTCCTAGGAAAATAGGACTTGAACCTATACTAAAGAGATCAAAACTCTTGGTGCTACC-ACTACACCATCT

Section 62

		(4576) 4576 … 4650
Homo sapiens mitochondrion, complete genome NC_01...	(4397)	CCTAAAGTAAGGTCAGCTAAATAAGCTATCGGGCCCATACCCCGAAAATGTTGGTTATACC--CTTCCCGTACTA
Latimeria chalumnae mitochondrion NC_001804.1	(3971)	CCTA--GTAAAGTCAGCTAAAAAAGCTTTCGGGCCCATACCCCGAACATGTTGGTTAAACCTCCCTCCTTTACTA

Section 63

		(4651) 4651 … 4725
Homo sapiens mitochondrion, complete genome NC_01...	(4470)	ATTAATCCCCTGGCCCAACCCGTCATCTACTCTACCATCTTTGCA---GGCACACTCATCACAGCGCTAAGCTCG
Latimeria chalumnae mitochondrion NC_001804.1	(4044)	ATGAG--CCCTTACGTAACAATAATCCTTATCT-CAAGCCTTGGACTCGGGACAACAATTACATTTACAAGCTCA

```
                                                                                              Section 64
                                                    (4726) 4726          4740      4750      4760      4770      4780      4790    4800
Homo sapiens mitochondrion, complete genome NC_01... (4542) CACTGATTTTTACCTGAGTAGGCCTAGAAATAAACATGCTAGCTTTTATTCCAGTTCTAACCAAAAAAATAAAC
    Latimeria chalumnae mitochondrion NC_001804.1 (4116) TCCTGACTGATAGCTTGAATAGGTCTAGAAATTAATACCCTAGCCATCACCCCCCTAATAGTAAAACAACATCAC
                                                                                              Section 65
                                                    (4801) 4801     4810      4820      4830      4840      4850      4860         4875
Homo sapiens mitochondrion, complete genome NC_01... (4617) CCTCGTTCCACAGAAGCTGCCATCAAGTATTTCCTCACGCAAGCAACCGCATCCATAATCCTTCTAATAGCTATC
    Latimeria chalumnae mitochondrion NC_001804.1 (4191) CCTCGGGCAACTGAAGCCACAACAAAATATTTTCTTACCCAGGCAACAGCATCAGGACTGCTATTATTCGCAACC
                                                                                              Section 66
                                                    (4876) 4876          4890      4900      4910      4920      4930      4940    4950
Homo sapiens mitochondrion, complete genome NC_01... (4692) CTCTTCAACAATATACTCTCCGGACAATGAACCATAACCAATACTACCAATCAATAC-TCATCATTAATAATCAT
    Latimeria chalumnae mitochondrion NC_001804.1 (4266) CTTAACAACGCTTGGATAACAGGAGAATGAAACACAAT-AGAACTATCAAACAATTTATCCGCCCCAATAATTAC
                                                                                              Section 67
                                                    (4951) 4951     4960      4970      4980      4990      5000      5010         5025
Homo sapiens mitochondrion, complete genome NC_01... (4766) AATAGCTATAGCAATAAAACTAGGAATAGCCCCCTTTCACTTCTGAGTCCCAGAGGTTACCCAAGGCACCCCTCT
    Latimeria chalumnae mitochondrion NC_001804.1 (4340) AATAGCCCTCGCACTAAAGATAGGAGTAGCACCAATACACTTCTGATTACCAGAAGTGCTACAAGGACTCCCCCT
                                                                                              Section 68
                                                    (5026) 5026          5040      5050      5060      5070      5080      5090    5100
Homo sapiens mitochondrion, complete genome NC_01... (4841) GACATCCGGCCTGCTTCTTCTCACATGACAAAAACTAGCCCCCATCTCAATCATATACCAAATCTCTCCCTCACT
    Latimeria chalumnae mitochondrion NC_001804.1 (4415) ACTAACTGGACTTATCCTGTCGACTTGACAAAAACTAGCCCCCTTCACCCTACTATATATAACATCACATGAATT
                                                                                              Section 69
                                                    (5101) 5101     5110      5120      5130      5140      5150      5160         5175
Homo sapiens mitochondrion, complete genome NC_01... (4916) AAACGTAAGCCTTCTCCTCACTCTCTCAATCTTATCCATCATAGCAGGCAGTTGAGGTGGATTAAACCAAACCCA
    Latimeria chalumnae mitochondrion NC_001804.1 (4490) AAACACAACAACAATAACAATCCTAGGATTGACATCAACAATTATCGGTGGCCTTGGTGGATTAAACCAAACTCA
                                                                                              Section 70
                                                    (5176) 5176          5190      5200      5210      5220      5230      5240    5250
Homo sapiens mitochondrion, complete genome NC_01... (4991) GCTACGCAAAATCTTAGCATACTCCTCAATTACCCACATAGGATGAATAATAGCAGTTCTACCGTACAACCCTAA
    Latimeria chalumnae mitochondrion NC_001804.1 (4565) ACTGCGAAAGTCCTAGCTTACTCATCAATTGCACACCTCGGATGAATAGTTATCATTATCCAATACT-CCAAAA
```

Section 71

	(5251)	5251 5260 5270 5280 5290 5300 5310 5325
Homo sapiens mitochondrion, complete genome NC_01...	(5066)	CA-TAACCATTCTTAATTTAACTATTTATATTATCCTAACTACTACCGCATTCCTACTACTCAACTTAAACTCCA
Latimeria chalumnae mitochondrion NC_001804.1	(4639)	CACTAGCCCTACTAAACCTACTGCTATATATTACAATAACATCAACAGCATTTTTAACACTTAT----GACTCTA

Section 72

	(5326)	5326 5340 5350 5360 5370 5380 5390 5400
Homo sapiens mitochondrion, complete genome NC_01...	(5140)	GCA-CCACGACCCTACTACTATC-TCGCACCTGAAACAAGCTAACATGACTAAC---ACCCTTAATTCCATCCAC
Latimeria chalumnae mitochondrion NC_001804.1	(4710)	TCAGCCACAAAAAT--TAATACCCTATCAACA-AAATGAGCAACAACCCCTATCGCAACTATAACTGCAATACTA

Section 73

	(5401)	5401 5410 5420 5430 5440 5450 5460 5475
Homo sapiens mitochondrion, complete genome NC_01...	(5210)	CCTCCTCTCCC--TAGGAGGCCTGCCCCCGCTAACCGGCTTTTTGCCCAAATGGGCCATTATCGAAGAATTCACA
Latimeria chalumnae mitochondrion NC_001804.1	(4782)	GCTCTACTAGCATTAGGAGGTCTCCCACCACTAACAGGATTTATACCAAAATGACTAATTTTACAAGAACTTACC

Section 74

	(5476)	5476 5490 5500 5510 5520 5530 5540 5550
Homo sapiens mitochondrion, complete genome NC_01...	(5283)	AAAAACAATAGCCTCATCATCCCCACCATCATAGCCACCATCACCCTCCTTAA-CCTCTACTTCTACCTACGCCT
Latimeria chalumnae mitochondrion NC_001804.1	(4857)	AAGCAAAATTTACCCGCTCTAGCCACACTAATAGCC-CTATCAGCCCTATTAAGCCTCTTCTTCTACCTACGAAT

Section 75

	(5551)	5551 5560 5570 5580 5590 5600 5610 5625
Homo sapiens mitochondrion, complete genome NC_01...	(5357)	AATCTACTCCACCTCAATCACACTACTCCCCATAT-CTAACAACGTAAAAATAAAATGACAGTTTGAACATACAA
Latimeria chalumnae mitochondrion NC_001804.1	(4931)	ATGCCATACAATAACCCTTACAAT-CTCACCAAACACAAACAATAACATAATTACATGACGAAAGAAACCTGGCC

Section 76

	(5626)	5626 5640 5650 5660 5670 5680 5690 5700
Homo sapiens mitochondrion, complete genome NC_01...	(5431)	AACCCACCCCATTCCTCCCCACACTCATCGCCCTTACCACGCTACTCCTACCTATCTCCCCTTTTATACTA----
Latimeria chalumnae mitochondrion NC_001804.1	(5005)	AGAAAGCACTACCCCTAGCCATACTAAGCATCATAACCCTCATAGCACTCCCAACAACCCCAACAATAGTAGCCA

Section 77

	(5701)	5701 5710 5720 5730 5740 5750 5760 5775
Homo sapiens mitochondrion, complete genome NC_01...	(5502)	--ATAATCTTATA-GAAATTTAGGT-TAAATACAGACCAAGAGCCTTCAAAGCCCTCAGTAAGT-TGCAATACT-
Latimeria chalumnae mitochondrion NC_001804.1	(5080)	TCATAAACTAATAAGAGACTTAGGAATAAACCAAGACCAAGAGCCTTCAAAGCCCTCAGCAGGAGTGAAAACCTC

Homo sapiens mitochondrion, complete genome vs. Latimeria chalumnae mitochondrion

Section 78

	(5776)	5776 5790 5800 5810 5820 5830 5840 5850
Homo sapiens mitochondrion, complete genome NC_01...	(5571)	-TAATTTCTGTAACAGCTAAGGACTGCAAAACCCCACTCTGCATCAACTGAACGCAAATCAGCCACTTTAATTAA
Latimeria chalumnae mitochondrion NC_001804.1	(5155)	CTAGTCTCTGGA-----TAAGACCTGTAGGATACTACCCCACATATTCTGAATGCAAACCAGACACTTTAATTAA

Section 79

	(5851)	5851 5860 5870 5880 5890 5900 5910 5925
Homo sapiens mitochondrion, complete genome NC_01...	(5645)	GCTAAGCCCTT-ACTAGACCAATGGGACTTAAACCCACAAACACTTAGTTAACAGCTAAGCACCCTAATCAACTG
Latimeria chalumnae mitochondrion NC_001804.1	(5225)	GCTAAGGCCTCCACTAGATGGGTAGGCCTCGATCCTACAAAATATTAGTTAACAGCTAAAAGCCCAAACCGGCGA

Section 80

	(5926)	5926 5940 5950 5960 5970 5980 5990 6000
Homo sapiens mitochondrion, complete genome NC_01...	(5719)	GCTTCAATCTACTTCTCCCGCCGCCGGGAAAAAAGGCGGGA--GAAGCCCCGGCAGGTTTGAAGCTGCTTCTTCG
Latimeria chalumnae mitochondrion NC_001804.1	(5300)	GCATCCATC-----CTAACATTCCAAGAAAAAAAGGAATGTTTGAAGCCTCAACAAGCACCACT-TGTCCCTCCA

Section 81

	(6001)	6001 6010 6020 6030 6040 6050 6060 6075
Homo sapiens mitochondrion, complete genome NC_01...	(5792)	AATTTGCAATTCAATATGAAAATCACCTCGGAGCT-GGTAAAAAGAGGCCTAACCCCTGTCTTTA--GATTTACA
Latimeria chalumnae mitochondrion NC_001804.1	(5369)	GGTTTGCAACCTGACATGCTAAACACCATGAGGCTTGGTAAGAGGAGGAATTGAACCTCCCTACACGGGGCTACA

Section 82

	(6076)	6076 6090 6100 6110 6120 6130 6140 6150
Homo sapiens mitochondrion, complete genome NC_01...	(5864)	GTCCAATGCTT----CACTCAGCCATTTTACCTCACCCCCACTGATGTTCGCCGACCGTTGACTATTCTCTACAA
Latimeria chalumnae mitochondrion NC_001804.1	(5444)	ACCCGCCGCTAAGAGCAATCAGCCATCTTACCTG--------TGATAATCACT--C-GTTGACTATTCTCAACCA

Section 83

	(6151)	6151 6160 6170 6180 6190 6200 6210 6225
Homo sapiens mitochondrion, complete genome NC_01...	(5935)	ACCACAAAGACATTGGAACACTATACCTATTATTCGGCGCATGAGCTGGAGTCCTAGGCACAGCTCTAAGCCTCC
Latimeria chalumnae mitochondrion NC_001804.1	(5508)	ACCATAAAGACATTGGTACCCTATACATGATCTTCGGTGCCTGAGCTGGAATAGTTGGAACCGCCCTAAGCCTGC

Section 84

	(6226)	6226 6240 6250 6260 6270 6280 6290 6300
Homo sapiens mitochondrion, complete genome NC_01...	(6010)	TTATTCGAGCCGAGCTGGGCCAGCCAGGCAACCTTCTAGGTAACGACCACATCTACAACGTTATCGTCACAGCCC
Latimeria chalumnae mitochondrion NC_001804.1	(5583)	TTATTCGAGCTGAACTCAGCCAACCTGGGGCTCTCCTGGGCGATGACCAAATTTATAATGTAGTCGTTACAGCAC

Homo sapiens mitochondrion, complete genome vs. Latimeria chalumnae mitochondrion

Section 85

(6301) 6301 6310 6320 6330 6340 6350 6360 6375
Homo sapiens mitochondrion, complete genome NC_01... (6085) ATGCATTTGTAATAATCTTCTTCATAGTAATACCCATCATAATCGGAGGCTTTGGCAACTGACTAGTTCCCCTAA
Latimeria chalumnae mitochondrion NC_001804.1 (5658) ATGCATTCGTGATAATCTTCTTTATAGTAATACCGATCATAATCGGCGGGTTTGGCAACTGATTAATTCCCCTGA

Section 86

(6376) 6376 6390 6400 6410 6420 6430 6440 6450
Homo sapiens mitochondrion, complete genome NC_01... (6160) TAATCGGTGCCCCCGATATGGCGTTTCCCCGCATAAACAACATAAGCTTCTGACTCTTACCTCCCTCTCTCCTAC
Latimeria chalumnae mitochondrion NC_001804.1 (5733) TGATTGGGGGCACCCGACATAGCATTTCCACGTATAAACAACATAAGCTTCTGACTACTACCACCCTCACTCCTAC

Section 87

(6451) 6451 6460 6470 6480 6490 6500 6510 6525
Homo sapiens mitochondrion, complete genome NC_01... (6235) TCCTGCTCGCATCTGCTATAGTGGAGGCCGGAGCAGGAACAGGTTGAACAGTCTACCCTCCCTTAGCAGGGAACT
Latimeria chalumnae mitochondrion NC_001804.1 (5808) TCCTACTAGCATCTTCTGGAGTAGAAGCAGGAGCAGGCACAGGATGGACAGTATACCCTCCACTAGCGGGCAACC

Section 88

(6526) 6526 6540 6550 6560 6570 6580 6590 6600
Homo sapiens mitochondrion, complete genome NC_01... (6310) ACTCCCACCCTGGAGCTCCGTAGACCTAACCATCTTCTCCTTACACCTAGCAGGTGTCTCCTCTATCTTAGGGG
Latimeria chalumnae mitochondrion NC_001804.1 (5883) TCGCCCATGCAGGAGCATCCGTAGATTTAACAATTTTCTCCTTACATCTAGCCGGTGTATCCTCAATCTTAGGGG

Section 89

(6601) 6601 6610 6620 6630 6640 6650 6660 6675
Homo sapiens mitochondrion, complete genome NC_01... (6385) CCATCAATTTCATCACAACAATTATCAATATAAAACCCCCTGCCATAACCCAATACCAAACGCCCCTCTTCGTCT
Latimeria chalumnae mitochondrion NC_001804.1 (5958) CCATCAACTTCATCACAACAGTAATCAACATAAAACCCCCAACAATAACACAGTATCAGACACCACTATTTATCT

Section 90

(6676) 6676 6690 6700 6710 6720 6730 6740 6750
Homo sapiens mitochondrion, complete genome NC_01... (6460) GATCCGTCCTAATCACAGCAGTCCTACTTCTCCTATCTCTCCCAGTCCTAGCTGCTGGCATCACTATACTACTAA
Latimeria chalumnae mitochondrion NC_001804.1 (6033) GATCAGTCTTAGTGACCGCCGTACTACTCCTACTCTCGCTACCGGTGCTAGCTGCCGGAATTACCATACTACTGA

Section 91

(6751) 6751 6760 6770 6780 6790 6800 6810 6825
Homo sapiens mitochondrion, complete genome NC_01... (6535) CAGACCGCAACCTCAACACCACCTTCTTCGACCCCGCCGGAGGAGGAGACCCCATTCTATACCAACACCTATTCT
Latimeria chalumnae mitochondrion NC_001804.1 (6108) CAGATCGAAATCTAAACACAACATTCTTTGACCCTGCTGGAGGAGGAGACCCTATTCTATACCAACACCTATTCT

Homo sapiens mitochondrion, complete genome vs. Latimeria chalumnae mitochondrion

Section 92

	(6826)	6826 6840 6850 6860 6870 6880 6890 6900
Homo sapiens mitochondrion, complete genome NC_01...	(6610)	GATTTTTCGGTCACCCTGAAGTTTATATTCTTATCCTACCAGGCTTCGGAATAATCTCCCATATTGTAACTTACT
Latimeria chalumnae mitochondrion NC_001804.1	(6183)	GATTCTTCGGCCATCCTGAAGTATACATCCTAATTTTACCAGGATTTGGTATAATCTCACACATTGTGGCCTACT

Section 93

	(6901)	6901 6910 6920 6930 6940 6950 6960 6975
Homo sapiens mitochondrion, complete genome NC_01...	(6685)	ACTCCGGAAAAAAAGAACCATTTGGATACATAGGTATGGTCTGAGCTATGATATCAATTGGCTTCCTAGGGTTTA
Latimeria chalumnae mitochondrion NC_001804.1	(6258)	ACTCTGGAAAGAAAGAACCATTCGGGTATATAGGTATAGTATGAGCTATAATGGCAATTGGACTTCTAGGCTTCA

Section 94

	(6976)	6976 6990 7000 7010 7020 7030 7040 7050
Homo sapiens mitochondrion, complete genome NC_01...	(6760)	TCGTGTGAGCACACCATATATTTACAGTAGGAATAGACGTAGACACACGAGCATATTTCACCTCCGCTACCATAA
Latimeria chalumnae mitochondrion NC_001804.1	(6333)	TCGTATGAGCCCATCATATATTTACCGTAGGAATGGATGTTGACACACGAGCATACTTTACATCAGCAACCATAA

Section 95

	(7051)	7051 7060 7070 7080 7090 7100 7110 7125
Homo sapiens mitochondrion, complete genome NC_01...	(6835)	TCATCGCTATCCCCACCGGCGTCAAAGTATTTAGCTGACTCGCCACACTCCACGGAAGCAATATGAAATGATCTG
Latimeria chalumnae mitochondrion NC_001804.1	(6408)	TTATTGCCATCCCAACAGGAGTAAAAGTGTTCAGCTGACTAGCGACACTTCACGGAGGAGTGACCAAATGAGACA

Section 96

	(7126)	7126 7140 7150 7160 7170 7180 7190 7200
Homo sapiens mitochondrion, complete genome NC_01...	(6910)	CTGCAGTGCTCTGAGCCCTAGGATTCATCTTTCTTTTCACCGTAGGTGGCCTGACTGGCATTGTATTAGCAAACT
Latimeria chalumnae mitochondrion NC_001804.1	(6483)	CACCCCTGCTATGAGCACTAGGATTTATCTTTCTTTTTACAGTAGGAGGCCTAACAGGCATCGTACTGGCAAACT

Section 97

	(7201)	7201 7210 7220 7230 7240 7250 7260 7275
Homo sapiens mitochondrion, complete genome NC_01...	(6985)	CATCACTAGACATCGTACTACACGACACGTACTACGTTGTAGCCCACTTCCACTATGTCCTATCAATAGGAGCTG
Latimeria chalumnae mitochondrion NC_001804.1	(6558)	CATCACTAGACATCATCCTACATGACACTTATTACGTAGTAGCACACTTCCACTATGTCCTATCAATAGGAGCAG

Section 98

	(7276)	7276 7290 7300 7310 7320 7330 7340 7350
Homo sapiens mitochondrion, complete genome NC_01...	(7060)	TATTTGCCATCATAGGAGGCTTCATTCACTGATTTCCCCTATTCTCAGGCTACACCCTAGACCAAACCTACGCCA
Latimeria chalumnae mitochondrion NC_001804.1	(6633)	TATTTGCAATCATAGGGGGACTCGTGCACTGATTTCCACTAATAACAGGATATACCTTACACAACACATGAACAA

Section 99

	(7351)	7351 7360 7370 7380 7390 7400 7410 7425
Homo sapiens mitochondrion, complete genome NC_01...	(7135)	AAATCCATTTCACTATCATATTCATCGGCGTAAATCTAACTTTCTTCCCACAACACTTTCTCGGCCTATCCGGAA
Latimeria chalumnae mitochondrion NC_001804.1	(6708)	AAATCCACTTTGGTGTAATATTCACAGGAGTAAACCTAACATTTTTCCCACAACACTTCCTCGGACTAGCAGGAA

Section 100

	(7426)	7426 7440 7450 7460 7470 7480 7490 7500
Homo sapiens mitochondrion, complete genome NC_01...	(7210)	TGCCCCGACGTTACTCGGACTACCCCGATGCATACACCACATGAAACATCCTATCATCTGTAGGCTCATTCATTT
Latimeria chalumnae mitochondrion NC_001804.1	(6783)	TACCACGACGTTACTCAGACTATCCAGATGCCTATACTTTATGAAACACAGTATCATCAATTGGCTCTCTAATTT

Section 101

	(7501)	7501 7510 7520 7530 7540 7550 7560 7575
Homo sapiens mitochondrion, complete genome NC_01...	(7285)	CTCTAACAGCAGTAATATTAATAATTTTCATGATTTGAGAAGCCTTCGCTTCGAAGCGAAAAGTCCTAATAGTAG
Latimeria chalumnae mitochondrion NC_001804.1	(6858)	CACTAATTGCCGTAATCATATTTATATTTATCCTGTGAGAAGCTTTCTCTGCCAAACGAGAAGTACTAATTGTAG

Section 102

	(7576)	7576 7590 7600 7610 7620 7630 7640 7650
Homo sapiens mitochondrion, complete genome NC_01...	(7360)	AAGAACCCTCCATAAACCTGGAGTGACTATATGGATGCCCCCCACCCTACCACACATTCGAAGAACCCGTATACA
Latimeria chalumnae mitochondrion NC_001804.1	(6933)	AAATAACAACAACAAATGTAGAATGGCTGCACGGATGCCCACCACCACACCACACATATGAAGAACCAGCATTCG

Section 103

	(7651)	7651 7660 7670 7680 7690 7700 7710 7725
Homo sapiens mitochondrion, complete genome NC_01...	(7435)	TAAAATCTA---GACAAAA--------AAGGAAGGAATCGAACCCCCCAAAGCTGGTTTCAAGCCAACCCCATGG
Latimeria chalumnae mitochondrion NC_001804.1	(7008)	TACAAGCTCCTCGATAAAACACTGAGAAAAGAGGGAATCGAACCCCCGTCAGCTAATTTCAAGACAGCCGCATCA

Section 104

	(7726)	7726 7740 7750 7760 7770 7780 7790 7800
Homo sapiens mitochondrion, complete genome NC_01...	(7499)	CCTCCATGACTTTTTC---AAAAAGGTATTAGAAAAACCATTT-CATAACTTTGTCAAAGTTAATTATAGGCTA
Latimeria chalumnae mitochondrion NC_001804.1	(7083)	CCACTCTGCCATTTTCTTTATTAAGATTCTAGTAAAACAAATTACACTACCTTGTCAAGATAGAATTATGAGTGA

Section 105

	(7801)	7801 7810 7820 7830 7840 7850 7860 7875
Homo sapiens mitochondrion, complete genome NC_01...	(7570)	AATCCT-ATATATCTT-----AATGGCACATGCAGCGCAAGTAGGTCTACAAGACGCTACTTCCCCTATCATAGA
Latimeria chalumnae mitochondrion NC_001804.1	(7158)	AAACCTCATGAACCTTGACCAAATGGCACACCCATCACAGTTAGGATTACAAGATGCAGCTTCTCCCGTTATAGA

Section 106

(7876) 7876 7890 7900 7910 7920 7930 7940 7950
Homo sapiens mitochondrion, complete genome NC_01... (7639) AGAGCTTATCACCTTTCATGATCACGCCCTCATAATCATTTTCCTTATCTGCTTCCTAGTCCTGTATGCCCTTTT
Latimeria chalumnae mitochondrion NC_001804.1 (7233) AGAACTCCTCCACTTTCACGATCATGCACTAATAATTGTATTTTTAATTAGCACATTAGTATTTTACATTATTCT

Section 107

(7951) 7951 7960 7970 7980 7990 8000 8010 8025
Homo sapiens mitochondrion, complete genome NC_01... (7714) CCTAACACTCACAACAAAACTAACTAATACTAACATCTCAGACGCTCAGGAAATAGAAACCGTCTGAACTATCCT
Latimeria chalumnae mitochondrion NC_001804.1 (7308) AGCCATAATAACAACAAAAATAACTGACAAATATATCTTAGACGCACAAGAAATTGAAATTGTGTGAACACTACT

Section 108

(8026) 8026 8040 8050 8060 8070 8080 8090 8100
Homo sapiens mitochondrion, complete genome NC_01... (7789) GCCCGCCATCATCCTAGTCCTCATCGCCCTCCCATCCCTACGCATCCTTTACATAACAGACGAGGTCAACGATCC
Latimeria chalumnae mitochondrion NC_001804.1 (7383) CCCAGCAATCGTCCTAATCCTAGTTGCCCTACCCTCGCTACGAATCCTATATCTAATTGATGAAGTCGAAAACCC

Section 109

(8101) 8101 8110 8120 8130 8140 8150 8160 8175
Homo sapiens mitochondrion, complete genome NC_01... (7864) CTCCCTTACCATCAAATCAATTGGCCACCAATGGTACTGAACCTACGAGTACACCGACTACGGCGGACTAATCTT
Latimeria chalumnae mitochondrion NC_001804.1 (7458) TCACCTAACAATTAAAGCAATAGGCCACCAATGATACTGAAGCTATGAGTACACGGACTATGAAGAACTAAGCTT

Section 110

(8176) 8176 8190 8200 8210 8220 8230 8240 8250
Homo sapiens mitochondrion, complete genome NC_01... (7939) CAACTCCTACATACTTCCCCCATTATTCCTAGAACCAGGCGACCTGCGACTCCTTGACGTTGACAATCGAGTAGT
Latimeria chalumnae mitochondrion NC_001804.1 (7533) CGACTCATACATAACACCACTACAAGACCTAAACCCGGGCCAATTCCGCTTGCTGGAAACAGACCATCGAATGGT

Section 111

(8251) 8251 8260 8270 8280 8290 8300 8310 8325
Homo sapiens mitochondrion, complete genome NC_01... (8014) ACTCCCGATTGAAGCCCCCATTCGTATAATAATTACATCACAAGACGTCTTGCACTCATGAGCTGTCCCCACATT
Latimeria chalumnae mitochondrion NC_001804.1 (7608) TATCCCAATAGAGTCGCTTATCCGAGTACTAATTTCAGCTGAAGACGTACTACACTCATGAGCAGTCCCAGCCCT

Section 112

(8326) 8326 8340 8350 8360 8370 8380 8390 8400
Homo sapiens mitochondrion, complete genome NC_01... (8089) AGGCTTAAAAACAGATGCAATTCCCGGACGTCTAAACCAAACCACTTTCACCGCTACACGACCGGGGGTATACTA
Latimeria chalumnae mitochondrion NC_001804.1 (7683) AGGAGTAAAAATAGATGCAGTCCCAGGGCGACTCAACCAAATTACATTCATAATTTCCCGACCAGGACTATATTA

```
                                                                         Section 113
                                                     (8401) 8401     8410      8420      8430      8440      8450      8460          8475
Homo sapiens mitochondrion, complete genome NC_01... (8164) CGGTCAATGCTCTGAAATCTGTGGAGCAAACCACAGTTTCATGCCCATCGTCCTAGAATTAATTCCCCTAAAAAT
   Latimeria chalumnae mitochondrion NC_001804.1     (7758) TGGACAATGCTCAGAGATTTGTGGAGCAAACCACAGCTTTATACCCATCGTACTTGAAGCAATCCCACTAGACCC
                                                                         Section 114
                                                     (8476) 8476           8490      8500      8510      8520      8530      8540  8550
Homo sapiens mitochondrion, complete genome NC_01... (8239) CTTTGAAATAGGGCCCGTATTTACCCTATAGCACCCCCTCTACCCCCTCTAGAGCCCACTGTAAAGCTAACTTAG
   Latimeria chalumnae mitochondrion NC_001804.1     (7833) CTTCGAAGACTGATCTTCATCAATGCTGGAAGAAGCCT------CACTGAGAAGCTAAATAGAAAGCGA------
                                                                         Section 115
                                                     (8551) 8551     8560      8570      8580      8590      8600      8610          8625
Homo sapiens mitochondrion, complete genome NC_01... (8314) CATTAACCTTTTAAGTTAAAGATTAAGAGAACCAACACCTCTTTACAGTGAAATGCCCCAACTAAATACTACCGT
   Latimeria chalumnae mitochondrion NC_001804.1     (7896) ---TAGCCTTTTAAGCTAGAGACTGGTGAAAACAAACCCACCCT-CAGTGCCATGCCACAACTAAACCCCTCCCC
                                                                         Section 116
                                                     (8626) 8626           8640      8650      8660      8670      8680      8690  8700
Homo sapiens mitochondrion, complete genome NC_01... (8389) ATGGCCCACCATAATTACCCCCATACTCCTTACACTATTCCTCATCACCCAACTAAAAATATTAAACACAAACTA
   Latimeria chalumnae mitochondrion NC_001804.1     (7967) CTG----ACTACTAATCCTGCTATTCTCCTGACTC--ATCTTCTTAACTATACTCCCC-TCTAAGACACAATTAC
                                                                         Section 117
                                                     (8701) 8701     8710      8720      8730      8740      8750      8760          8775
Homo sapiens mitochondrion, complete genome NC_01... (8464) CCACCTACCTC--CCTCACCAAAGCCCATAAAAATAAAAAATTATAACAAACCCTGAGAACCAAA--ATGAACGA
   Latimeria chalumnae mitochondrion NC_001804.1     (8035) ACACCTTCCCAAACATGCCATCAACACAAAATATATGCAAACAAGAACCAGAACCATGAACCTGACCATGAGCCT
                                                                         Section 118
                                                     (8776) 8776           8790      8800      8810      8820      8830      8840  8850
Homo sapiens mitochondrion, complete genome NC_01... (8535) AAATCTGTTCGCTTCATTCATTGCCCCCACAATCCTAGGCCTACCCGCCGCAGTACTG-ATCATTCTATTTCCCC
   Latimeria chalumnae mitochondrion NC_001804.1     (8110) AAACTTCTTTGACCAATTTATGAGCCCAACACTATTAGGAGTACCA--CTCATTGCTGTAGCAATAATATTCCCA
                                                                         Section 119
                                                     (8851) 8851     8860      8870      8880      8890      8900      8910          8925
Homo sapiens mitochondrion, complete genome NC_01... (8609) ----CTCTATTGATCCCCACCTCCAAATATCTCA-TCAACAACCGACTAATCACCACCCAACAATGACTAATCAA
   Latimeria chalumnae mitochondrion NC_001804.1     (8183) TGGACCCTATT-ACCAACACCAACCAACCGATGACTTAATAACCGAACACTAACACTACAAAACTGATTTATCGG
```

```
                                                                    Section 120
                                                       (8926) 8926          8940      8950      8960      8970      8980      8990      9000
Homo sapiens mitochondrion, complete genome NC_01...   (8679) ACTAACCTCAAAACAAATGATAACCATACACAACACTAAAGGACGAACCTGATCTCTTATACTAGTATCCTTAAT
Latimeria chalumnae mitochondrion NC_001804.1          (8257) CCGCTTCACTAATCAACTACTACAACCATTAAACACTGGAGGACACAAATGAGCAATAATCTTAATATCACTA--

                                                                    Section 121
                                                       (9001) 9001     9010      9020      9030      9040      9050      9060           9075
Homo sapiens mitochondrion, complete genome NC_01...   (8754) CATTTTTATTGCCACAACTAACCTCCTCGGACTCCTGCCTCACTCATTTACACCAACCACCCAACTATCTATAAA
Latimeria chalumnae mitochondrion NC_001804.1          (8330) -A------------------ACCTCCTGGGACTTCTACCGTATACATTCACACCAACAACACAACTATCACTAAA

                                                                    Section 122
                                                       (9076) 9076          9090      9100      9110      9120      9130      9140      9150
Homo sapiens mitochondrion, complete genome NC_01...   (8829) CCTAGCCATGGCCATCCCCTTATGAGCGGGCACAGTGATTATAGGCTTTCGCTCTAAGATTAAAAATGCCCTAGC
Latimeria chalumnae mitochondrion NC_001804.1          (8386) CATGGGACTTGCTATTCCATTCTGACTAGCAACAGTATTACTGGGACTGCGTAACCAACCCACTGCCGCGCTAGG

                                                                    Section 123
                                                       (9151) 9151     9160      9170      9180      9190      9200      9210           9225
Homo sapiens mitochondrion, complete genome NC_01...   (8904) CCACTTCTTACCACAAGGCACACCTACACCCCTTATCCCCATACTAGTTATTATCGAAACCATCAGCCTACTCAT
Latimeria chalumnae mitochondrion NC_001804.1          (8461) ACACCTTCTCCCAGAAGGAACACCAACCCTGCTAATCCCAATCCTAATTATTATTGAAACAATCAGCCTACTTAT

                                                                    Section 124
                                                       (9226) 9226          9240      9250      9260      9270      9280      9290      9300
Homo sapiens mitochondrion, complete genome NC_01...   (8979) TCAACCAATAGCCCTGGCCGTACGCCTAACCGCTAACATTACTGCAGGCCACCTACTCATGCACCTAATTGGAAG
Latimeria chalumnae mitochondrion NC_001804.1          (8536) CCGCCCCTTCGCCCTAGGAGTACGACTAACAGCCAATCTCACAGCAGGCCACCTCCTAATACAATTAATTGCTAC

                                                                    Section 125
                                                       (9301) 9301     9310      9320      9330      9340      9350      9360           9375
Homo sapiens mitochondrion, complete genome NC_01...   (9054) CGCCACCCTAGCAATATCAACCATTAACCTTCCCTCTACACTTATCATCTTCACAATTCTAATTC-TACTGACTA
Latimeria chalumnae mitochondrion NC_001804.1          (8611) CGCCGCCTTCGTACTC-CTACCTATAATACCAACAGTAGCATTATTAACAACATTAGTCCTATTCCTCCTGACCC

                                                                    Section 126
                                                       (9376) 9376          9390      9400      9410      9420      9430      9440      9450
Homo sapiens mitochondrion, complete genome NC_01...   (9128) TCCTAGAAATCGCTGTCGCCTTAATCCAAGCCTACGTTTTCACACTTCTAGTAAGCCTCTACCTGCACGACAACA
Latimeria chalumnae mitochondrion NC_001804.1          (8685) TGCTAGAAATTGCCGTAGCAATAATCCAAGCCTACGTGTTTGTTCTATTACTAAGCCTCTATCTACAAGAAAATG
```

Section 127

(9451)	9451 … 9525
Homo sapiens mitochondrion, complete genome NC_01... (9203)	CATAATGACCCACCAATCACATGCCTATCATATAGTAAAACCCAGCCCATGACCCCTAACAGGGGCCCTCTCAGC
Latimeria chalumnae mitochondrion NC_001804.1 (8760)	TCTAATGGCCCACCAAGCACACGCATATCACATAGTTGACCCAAGCCCATGACCCATTACAGGGGCCACGGCCGC

Section 128

(9526)	9526 … 9600
Homo sapiens mitochondrion, complete genome NC_01... (9278)	CCTCCTAATGACCTCCGGCCTAGCCATGTGATTTCACTTCCACTCCATAACGCTCCTCATACTAGGCCTACTAAC
Latimeria chalumnae mitochondrion NC_001804.1 (8835)	CCTACTTGTAACCTCAGGCCTAGCAGCGTGATTTCACTTCAACTCAATAATCTTAATTTTAATAGGACTAACACT

Section 129

(9601)	9601 … 9675
Homo sapiens mitochondrion, complete genome NC_01... (9353)	CAACACACTAACCATATACCAATGATGGCGCGATGTAACACGAGAAAGCACATACCAAGGCCACCACACACCACC
Latimeria chalumnae mitochondrion NC_001804.1 (8910)	ATTGCTACTAACTATGTATCAATGATGACGAGATATTATTCGAGAAAGCACATTCCAAGGTCACCACACACTACC

Section 130

(9676)	9676 … 9750
Homo sapiens mitochondrion, complete genome NC_01... (9428)	TGTCCAAAAAGGCCTTCGATACGGGATAATCCTATTTATTACCTCAGAAGTTTTTTTCTTCGCAGGATTTTTCTG
Latimeria chalumnae mitochondrion NC_001804.1 (8985)	TGTACAAAAAAGCCTACGATATGGTATAATCCTGTTCATTACATCCGAAGTATTCTTCTTCCTAGGGTTCTTCTG

Section 131

(9751)	9751 … 9825
Homo sapiens mitochondrion, complete genome NC_01... (9503)	AGCCTTTTACCACTCCAGCCTAGCCCCTACCCCCCAATTAGGAGGGCACTGGCCCCCAACAGGCATCACCCCGCT
Latimeria chalumnae mitochondrion NC_001804.1 (9060)	AGCCTTTTACCATTCAAGTCTGGCACCCACTCCTGAACTCGGAGGACTCTGACCTCCCACTGGAATTACACCCCT

Section 132

(9826)	9826 … 9900
Homo sapiens mitochondrion, complete genome NC_01... (9578)	AAATCCCCTAGAAGTCCCACTCCTAAACACATCCGTATTACTCGCATCAGGAGTATCAATCACCTGAGCTCACCA
Latimeria chalumnae mitochondrion NC_001804.1 (9135)	AGATCCATTTGAAGTACCACTATTAAACACAGCAGTTCTACTAGCCTCGGGAATTACAGTCACATGAGCCCATCA

Section 133

(9901)	9901 … 9975
Homo sapiens mitochondrion, complete genome NC_01... (9653)	TAGTCTAATAGAAAACAACCGAAACCAAATAATTCAAGCACTGCTTATTACAATTTTACTGGGTCTCTATTTTAC
Latimeria chalumnae mitochondrion NC_001804.1 (9210)	CAGCCTAATAGAGGGGCAACGAAAAGAGGCTATCCAATCACTATTTATCACAGTTCTGTTAGGACTATACTTTAC

Homo sapiens mitochondrion, complete genome vs. Latimeria chalumnae mitochondrion

Section 134

	(9976)	9976 9990 10000 10010 10020 10030 10040 10050
Homo sapiens mitochondrion, complete genome NC_01...	(9728)	CCTCCTACAAGCCTCAGAGTACTTCGAGTCTCCCTTCACCATTTCCGACGGCATCTACGGCTCAACATTTTTTGT
Latimeria chalumnae mitochondrion NC_001804.1	(9285)	AGCACTGCAAGCCACAGAATACTACGAATCCCCATTTACAATCGCTGACGGAGCCTATGGCTCAACCTTTTTTGT

Section 135

	(10051)	10051 10060 10070 10080 10090 10100 10110 10125
Homo sapiens mitochondrion, complete genome NC_01...	(9803)	AGCCACAGGCTTCCACGGACTTCACGTCATTATTGGCTCAACTTTCCTCACTATCTGCTTCATCCGCCAACTAAT
Latimeria chalumnae mitochondrion NC_001804.1	(9360)	AGCAACCGGATTCCACGGTCTACATGTCATTATTGGCTCTACATTCCTAATCGTATGCCTAGTACGACAAACACA

Section 136

	(10126)	10126 10140 10150 10160 10170 10180 10190 10200
Homo sapiens mitochondrion, complete genome NC_01...	(9878)	ATTTCACTTTACATCCAAACATCACTTTGGCTTCGAAGCCGCCGCCTGATACTGGCATTTTGTAGATGTGGTTTG
Latimeria chalumnae mitochondrion NC_001804.1	(9435)	ATACCACTTCACATCAAACCACCACTTTGGCTTTGAAGCAGCAGCATGATACTGACATTTCGTAGACGTAGTCTG

Section 137

	(10201)	10201 10210 10220 10230 10240 10250 10260 10275
Homo sapiens mitochondrion, complete genome NC_01...	(9953)	ACTATTTCTGTATGTCTCCATCTATTGATGAGGGTCTTA--CTCTTTTAGTATAAA-TAGTACCGTTAACTTCCA
Latimeria chalumnae mitochondrion NC_001804.1	(9510)	ACTATTCTTATACGTATCAATCTACTGATGAGGCTCATAAACCCCTTTAGTACAAAATAATACAAATGACTTCCA

Section 138

	(10276)	10276 10290 10300 10310 10320 10330 10340 10350
Homo sapiens mitochondrion, complete genome NC_01...	(10025)	ATTAACTAGTTTTGACAACATTCAAAAAA-GAGTAATAAACTTCGCCTTAATTT---TAATAATCAACACCCTCC
Latimeria chalumnae mitochondrion NC_001804.1	(9585)	ATCATTTAATCTTGGTTATACCCCAAGAATGGGTAATGAACCTGATTCTAGCGGGCCTACTTATCATAAGCATCC

Section 139

	(10351)	10351 10360 10370 10380 10390 10400 10410 10425
Homo sapiens mitochondrion, complete genome NC_01...	(10096)	TAGCCTTACTACTAATAATTATTACATTTTGACTACCACAACTCAACGGCTACATAGAAAAATCCACCCCTTACG
Latimeria chalumnae mitochondrion NC_001804.1	(9660)	TCTCTATAATTTTAGCTATAATCGCATTCTGACTACCAAACATGACCCCTGATACAGAAAAACTATCTCCCTACG

Section 140

	(10426)	10426 10440 10450 10460 10470 10480 10490 10500
Homo sapiens mitochondrion, complete genome NC_01...	(10171)	AGTGCGGCTTCGACCCTATATCCCCCGCCCGCGTCCCTTTCTCCATAAAATTCTTCTTAGTAGCTATTACCTTCT
Latimeria chalumnae mitochondrion NC_001804.1	(9735)	AATGTGGCTTTGATCCTCTAGGATCCGCACGACTCCCATTCTCCCTACGATTTTTCCTAGTAGCAATCCTATTCC

Section 141

(10501) 10501 10510 10520 10530 10540 10550 10560 10575
Homo sapiens mitochondrion, complete genome NC_01... (10246) TATTATTTGATCTAGAAATTGCCCTCCTTTTACCCCTACCATGAGCCCTACAAACAACTAACCTGCCACTAATAG
Latimeria chalumnae mitochondrion NC_001804.1 (9810) TGCTATTTGACCTAGAAATTGCATTATTATTACCCCTACCCTGGGCAGACCAACTAACAAACCCAACACTTGCAT

Section 142

(10576) 10576 10590 10600 10610 10620 10630 10640 10650
Homo sapiens mitochondrion, complete genome NC_01... (10321) TTATGTCATCCCTCTTATTAATCATCATCCTAGCCCTAAGTCTGGCCTATGAGTGACTACAAAAAGGATTAGACT
Latimeria chalumnae mitochondrion NC_001804.1 (9885) TAACCTGGACAACAAGCATCATCGCCCTACTAACACTAGGACTAATCCACGAATGAACTCAAGGAGGCCTCGAAT

Section 143

(10651) 10651 10660 10670 10680 10690 10700 10710 10725
Homo sapiens mitochondrion, complete genome NC_01... (10396) GAACCGAATTGGTATATAGTTTAAACAAAACGAATGATTTCGACTCATTAAATTATGATAAT----CATATTTAC
Latimeria chalumnae mitochondrion NC_001804.1 (9960) GGGCAGAATAGGTAGTTAGTTTAAA-AAAACCACTAATTTCGACTTAGTTAACTGTGGTGAAACCCCACAACTAT

Section 144

(10726) 10726 10740 10750 10760 10770 10780 10790 10800
Homo sapiens mitochondrion, complete genome NC_01... (10467) CAA-ATGCCCCTCATTTACATAAATATTATACTAGCATTTACCATCTCACTTCTAGGAATACTAGTATATCGCTC
Latimeria chalumnae mitochondrion NC_001804.1 (10034) CTTCATGACCCCAGTACAACTCAGCTTTAACACTGCATTCACACTAGGCTTAATAGGAGTAACATTCCACCGAGC

Section 145

(10801) 10801 10810 10820 10830 10840 10850 10860 10875
Homo sapiens mitochondrion, complete genome NC_01... (10541) ACACCTCATATCCTCCCTACTATGCCTAGAAGGAATAATACTATCGCTGTTCATTATAGCTACTCTCATAACCCT
Latimeria chalumnae mitochondrion NC_001804.1 (10109) CCATCTGCTATCAGCATTACTCTGCCTAGAAGGAATAATATTATCCCTGTATAT-AGGACTGTCCCTATGACCAA

Section 146

(10876) 10876 10890 10900 10910 10920 10930 10940 10950
Homo sapiens mitochondrion, complete genome NC_01... (10616) CAACACCCACTCCCTCTTAGCCAATATTGTGCCTATTGCCA-TACTAGTCTTTGCCGCCTGCGAAGCAGCGGTGG
Latimeria chalumnae mitochondrion NC_001804.1 (10183) TGCAACTAGAATCAACTACATACATAACCACACCACTACTACTACTCGCCTTCTCAGCCTGTGAGGCTGGAGCAG

Section 147

(10951) 10951 10960 10970 10980 10990 11000 11010 11025
Homo sapiens mitochondrion, complete genome NC_01... (10690) GCCTAGCCCTACTAGTCTCAATCTCCAACACATATGGCCTAGACTACGTACATAACCTAAACCTACTCCAATGCT
Latimeria chalumnae mitochondrion NC_001804.1 (10258) GCCTAGCCCTCATAGTAGCAACATCCCGCACACATGGTACGGACCACCTCCAAAACCTAAACTTACTACAATGCT

Section 148

(11026) 11026 11040 11050 11060 11070 11080 11090 11100
Homo sapiens mitochondrion, complete genome NC_01... (10765) AAAACTAATCGTCCCAACAATTATATTACTACCACTGACATGACTTTCCAAAAAACACATAATTTGAATCAACAC
Latimeria chalumnae mitochondrion NC_001804.1 (10333) AAAAGTTTTAATACCAACAATTATGCTTATCTTAACCACATGA-TTAACAAAACCTGCATGACTCTGACCAACAA

Section 149

(11101) 11101 11110 11120 11130 11140 11150 11160 11175
Homo sapiens mitochondrion, complete genome NC_01... (10840) -AACCACCCACAGCCTAATTATTAGCATCATCCCTCTACTATTTTTTAACCA--AATCAACAACAACCTATTTAG
Latimeria chalumnae mitochondrion NC_001804.1 (10407) TAACAACCAATAGCCTACTCGTAGCTACCATCAGCTTAACCTGACTAAAATGGGACTCAGAGTCAGGATGAAAAT

Section 150

(11176) 11176 11190 11200 11210 11220 11230 11240 11250
Homo sapiens mitochondrion, complete genome NC_01... (10912) CTGTTCCCCAACCTTTTCCT-CCGACCCCCTAACAACCCCCCTCCTAATACTAACTACCTGACTCCTACCCCTCA
Latimeria chalumnae mitochondrion NC_001804.1 (10482) CTCTCAACAGCTCAATGGCTACCGACCCCCTATCTACACCATTACTAATCCTCACATGCTGGCTTCTACCCCTCA

Section 151

(11251) 11251 11260 11270 11280 11290 11300 11310 11325
Homo sapiens mitochondrion, complete genome NC_01... (10986) CAATCATGGCAAGCCAACGCCACTTATCCAGTGAACCACTATCACGAAAAAAACTCTACCTCTCTATACTAATCT
Latimeria chalumnae mitochondrion NC_001804.1 (10557) TAATTCTCGCAAGCCAAAACCACATGTTTATAGAACCACTAAACCGCCAACGATCATTCATCTCCCTACTCATCT

Section 152

(11326) 11326 11340 11350 11360 11370 11380 11390 11400
Homo sapiens mitochondrion, complete genome NC_01... (11061) CCCTACAAATCTCCTTAATTATAACATTCACAGCCACAGAACTAATCATATTTTATATCTTCTTCGAAACCACAC
Latimeria chalumnae mitochondrion NC_001804.1 (10632) CCCTACAAACATTCCTAATTATAGCATTTGGTGCCACTGAAATCATCCTATTTTACATTATATTTGAAGCAACCC

Section 153

(11401) 11401 11410 11420 11430 11440 11450 11460 11475
Homo sapiens mitochondrion, complete genome NC_01... (11136) TTATCCCCACCTTGGCTATCATCACCCGATGAGGCAACCAGCCAGAACGCCTGAACGCAGGCACATACTTCCTAT
Latimeria chalumnae mitochondrion NC_001804.1 (10707) TAATCCCAACACTAATTATTATTACCCGATGGGGTAATCAAACAGAGCGACTAAACGCAGGAACATACTTTTTAT

Section 154

(11476) 11476 11490 11500 11510 11520 11530 11540 11550
Homo sapiens mitochondrion, complete genome NC_01... (11211) TCTACACCCTAGTAGGCTCCCTTCCCCTACTCATCGCACTAATTTACACTCACAACACCCTAGGCTCACTAAACA
Latimeria chalumnae mitochondrion NC_001804.1 (10782) TTTATACAGTAATAGGGTCACTACCACTATTAGTTGCACTTTTAATAACACAAAATAACCTTGGTACCCTATCAA

Section 155

(11551)	11551 11560 11570 11580 11590 11600 11610 11625
Homo sapiens mitochondrion, complete genome NC_01... (11286)	TTCTACTACTCACTCTCACTGCCCAAGAACTA--TCAAACTCCTGAGCCAACAACTTAATATGACTAGCTTACAC
Latimeria chalumnae mitochondrion NC_001804.1 (10857)	TACCGCTCATCCAACACATATACCAAATAAAACTTCATACACATGGAG--ACATGATATGATGAACAGCCTGCCT

Section 156

(11626)	11626 11640 11650 11660 11670 11680 11690 11700
Homo sapiens mitochondrion, complete genome NC_01... (11359)	AATAGCTTTATAGTAAAGATACCTCTTTACGGACTCCACTTATGACTCCCTAAAGCCCATGTCGAAGCCCCCAT
Latimeria chalumnae mitochondrion NC_001804.1 (10930)	ATTAGCCTTCTTAGTAAAAATACCACTATACGGAGTCCACCTTTGACTCCCAAAAGCCCATGTAGAAGCCCCAAT

Section 157

(11701)	11701 11710 11720 11730 11740 11750 11760 11775
Homo sapiens mitochondrion, complete genome NC_01... (11434)	CGCTGGGTCAATAGTACTTGCCGCAGTACTCTTAAAACTAGGCGGCTATGGTATAATACGCCTCACACTCATTCT
Latimeria chalumnae mitochondrion NC_001804.1 (11005)	TGCAGGATCAATAGTACTAGCCGCCGTCCTACTAAAACTAGGAGGATACGGAATAATACGACTAATCATAATATT

Section 158

(11776)	11776 11790 11800 11810 11820 11830 11840 11850
Homo sapiens mitochondrion, complete genome NC_01... (11509)	CAACCCCCTGACAAAACACATAGCCTACCCCTTCCTTGTACTATCCCTATGAGGCATAATTATAACAAGCTCCAT
Latimeria chalumnae mitochondrion NC_001804.1 (11080)	AGCTCCAATAACAAAAACCCTAGCCTATCCATTCATCATCCTCGCCCTATGAGGAATCATTATAACTGGATCAAT

Section 159

(11851)	11851 11860 11870 11880 11890 11900 11910 11925
Homo sapiens mitochondrion, complete genome NC_01... (11584)	CTGCCTACGACAAACAGACCTAAAATCGCTCATTGCATACTCTTCAATCAGCCACATAGCCCTCGTAGTAACAGC
Latimeria chalumnae mitochondrion NC_001804.1 (11155)	CTGCTTACGACAAACAGACCTAAAATCCCTAATCGCCTACTCATCAGTAGGCCACATAGGACTAGTGGCAGCAGG

Section 160

(11926)	11926 11940 11950 11960 11970 11980 11990 12000
Homo sapiens mitochondrion, complete genome NC_01... (11659)	CATTCTCATCCAAACCCCCTGAAGCTTCACCGGCGCAGTCATTCTCATAATCGCCCACGGGCTTACATCCTCATT
Latimeria chalumnae mitochondrion NC_001804.1 (11230)	TATCCTAACACAAACACCATGAGGCTTTACAGGAGCTACTGTTCTAATAATTGCTCACGGTCTTACATCCTCAGC

Section 161

(12001)	12001 12010 12020 12030 12040 12050 12060 12075
Homo sapiens mitochondrion, complete genome NC_01... (11734)	ACTATTCTGCCTAGCAAACTCAAACTACGAACGCACTCACAGTCGCATCATAATCCTCTCTCAAGGACTTCAAAC
Latimeria chalumnae mitochondrion NC_001804.1 (11305)	CCTATTCTGTCTAGCAAACACAAACTATGAACGAACCCATAGCCGAACCATGATCCTAGCACGAGGAATGCAAGT

Section 162

(12076)		12076 12090 12100 12110 12120 12130 12140 12150
Homo sapiens mitochondrion, complete genome NC_01...	(11809)	TCTACTCCCACTAATAGCTTTTTGATGACTTCTAGCAAGCCTCGCTAACCTCGCCTTACCCCCCACTATTAACCT
Latimeria chalumnae mitochondrion NC_001804.1	(11380)	TATCCTCCCACTCATGACATTCTGATGACTTATAATAAATTTAGCTAACTTAGCCTTACCCCCATCCACTAATCT

Section 163

(12151)		12151 12160 12170 12180 12190 12200 12210 12225
Homo sapiens mitochondrion, complete genome NC_01...	(11884)	ACTGGGAGAACTCTCTGTGCTAGTAACCACGTTCTCCTGATCAAATATCACTCTCCTACTTACAGGACTCAACAT
Latimeria chalumnae mitochondrion NC_001804.1	(11455)	AATAGGAGAACTACTTATTATTACAATAACCTTCAACTGATCAAACTGGACACTAACTATAACAGGACTGGGCAT

Section 164

(12226)		12226 12240 12250 12260 12270 12280 12290 12300
Homo sapiens mitochondrion, complete genome NC_01...	(11959)	ACTAGTCACAGCCCTATACTCCCTCTACATATTTACCACAACACAATGGGGCTCACTCACCCACCACATTAACAA
Latimeria chalumnae mitochondrion NC_001804.1	(11530)	ACTAATCACAGCTATCTACTCATTACACATGTTCCTCACAACACAACGAGGCCTGATAACAAACCACATTATCTC

Section 165

(12301)		12301 12310 12320 12330 12340 12350 12360 12375
Homo sapiens mitochondrion, complete genome NC_01...	(12034)	CATAAAACCCTCATTCACACGAGAAAACACCCTCATGTTCATACACCTATCCCCCATTCTCCTCCTATCCCTCAA
Latimeria chalumnae mitochondrion NC_001804.1	(11605)	AATTGAACCCTCCCACACCCGAGAACACCTACTAATAACAATACATGCCCTCCCAATACTATTGCTAATCCTTAA

Section 166

(12376)		12376 12390 12400 12410 12420 12430 12440 12450
Homo sapiens mitochondrion, complete genome NC_01...	(12109)	CCCCGACATCATTACCGGGTTTTCCTCTTGTAAATATAGTTTAACCAAAACATCAGATTGTGAATCTGACAACAG
Latimeria chalumnae mitochondrion NC_001804.1	(11680)	ACCAGAACTGATCTGAGGCTGATCCTACTGTAAATATAGTTTGAAAAAAACATTAGACTGTGGCTCTAAAAATAA

Section 167

(12451)		12451 12460 12470 12480 12490 12500 12510 12525
Homo sapiens mitochondrion, complete genome NC_01...	(12184)	AGGCTTACGACCCTTATTTACCGAGAA-----AGCTCACAA-GAACTGCTAACTCA-TGCCCCCATGTCT-AAC
Latimeria chalumnae mitochondrion NC_001804.1	(11755)	GAGTTAAATCCTCTTTATTAACCGAGAGGGGCTAGAGCACTACGAACTGCTAATTCTGTAGTACCATGGTTCAAT

Section 168

(12526)		12526 12540 12550 12560 12570 12580 12590 12600
Homo sapiens mitochondrion, complete genome NC_01...	(12251)	AACATGGCTTTCTCAACTTTTAAAGGATAACAGCT-ATCCATTGGTCTTAGGCCCCAAAAATT-TTGGTGCAACT
Latimeria chalumnae mitochondrion NC_001804.1	(11830)	CCCATGGCCCACTCAGCCCCTAAAGGATAATAGCTCATCCATTGGTCTTAGGAGCCAAAAACTCTTGGTGCAACT

Section 169
(12601) 12601 12610 12620 12630 12640 12650 12660 12675
Homo sapiens mitochondrion, complete genome NC_01... (12324) CCAAATAA-----AAGTAATAACCATGCACACTACTATAACCACCC-TAACCCTGACTTCC---CTAATTCCCC
Latimeria chalumnae mitochondrion NC_001804.1 (11905) CCAAGTAGCGGCCATGTACACAACATTAATTTTTAACTCAACACTTATAACCATATTTACCATCCTAACAGCCCC

Section 170
(12676) 12676 12690 12700 12710 12720 12730 12740 12750
Homo sapiens mitochondrion, complete genome NC_01... (12390) CATCCTTACCACCCTCGTTAACCCTAACAAAAAAAACTCATAC----CCCCATTATG--TAAAATCCATTGTCGC
Latimeria chalumnae mitochondrion NC_001804.1 (11980) AATCTTAACCACCCT---TAACCCTATCAAACCCAACAAAAAGTGAACAGAATTATGAGTAAAAACCGCCGTA-C

Section 171
(12751) 12751 12760 12770 12780 12790 12800 12810 12825
Homo sapiens mitochondrion, complete genome NC_01... (12459) ATCCACCTTTATTATCA-GTCTCTTCCCCACAACAATATTCATGTGCCTAGACCAAGAAGTT------ATTATCT
Latimeria chalumnae mitochondrion NC_001804.1 (12051) AGCTAGCCTTCTTCACAAGTCTAATCC------CGCTCTTCATCTATCTTGACCAAGGAATCGAGACCATTACAA

Section 172
(12826) 12826 12840 12850 12860 12870 12880 12890 12900
Homo sapiens mitochondrion, complete genome NC_01... (12527) CGAACTGACACTGAGCCACAACCCAAACAACCCAGCTCTCCCTAAGCTTCAAACTAGACTACTTCTCCATAATAT
Latimeria chalumnae mitochondrion NC_001804.1 (12120) CAAACTGACAATGAATAAACACCAACACATTTAACATTAACATCAGCTTTAAATTTGACCAATACTCAATTGTAT

Section 173
(12901) 12901 12910 12920 12930 12940 12950 12960 12975
Homo sapiens mitochondrion, complete genome NC_01... (12602) TCATCCCTGTAGCATTGTTCGTTACATGGTCCATCATAGAATTCTCACTGTGATATATAAACTCAGACCCAAACA
Latimeria chalumnae mitochondrion NC_001804.1 (12195) TCATCCCAATCGCACTGTATGTCACATGATCAATTCTAGAATTCGCCAACTGATACATGCACCAAGACCCAAAAA

Section 174
(12976) 12976 12990 13000 13010 13020 13030 13040 13050
Homo sapiens mitochondrion, complete genome NC_01... (12677) TTAATCAGTTCTTCAAATATCTACTCATCTTCCTAATTACCATACTAATCTTAGTTACCGCTAACAACCTATTCC
Latimeria chalumnae mitochondrion NC_001804.1 (12270) TAAACCAATTTTTCAAATACCTGCTCCTATTCCTAATCACCATAATAATCTTAGTTACAGCAAACAACATGTTCC

Section 175
(13051) 13051 13060 13070 13080 13090 13100 13110 13125
Homo sapiens mitochondrion, complete genome NC_01... (12752) AACTGTTCATCGGCTGAGAGGGCGTAGGAATTATATCCTTCTTGCTCATCAGTTGATGATACGCCCGAGCAGATG
Latimeria chalumnae mitochondrion NC_001804.1 (12345) AACTATTTATCGGCTGAGAAGGAGTAGGAATTATATCATTCCTATTAATTGGCTGATGACATGGCCGAGCTAACG

Homo sapiens mitochondrion, complete genome vs. Latimeria chalumnae mitochondrion

Section 176

(13126) 13126 13140 13150 13160 13170 13180 13190 13200
Homo sapiens mitochondrion, complete genome NC_01... (12827) CCAACACAGCAGCCATTCAAGCAATCCTATACAACCGTATCGGCGATATCGGTTTCATCCTCGCCTTAGCATGAT
Latimeria chalumnae mitochondrion NC_001804.1 (12420) CCAATACCGCAGCCCTACAAGCAGTCATCTACAATCGAGTAGGAGACATTGGGTTAACCCTAAGCATGGTCTGAT

Section 177

(13201) 13201 13210 13220 13230 13240 13250 13260 13275
Homo sapiens mitochondrion, complete genome NC_01... (12902) TTATCCTACACTCCAACTCATGAGACCCACAACAAATAGCCCTTCTAAACGCTAATCCAAGCCTCACC---CCAC
Latimeria chalumnae mitochondrion NC_001804.1 (12495) TTGCAATCAACCTAAATACATGAGAAATACAACAAATATTTATCATATCCTACAACACCGACATAACCATCCCCC

Section 178

(13276) 13276 13290 13300 13310 13320 13330 13340 13350
Homo sapiens mitochondrion, complete genome NC_01... (12974) TACTAGGCCTCCTCCTAGCAGCAGCAGGCAAATCAGCCCAATTAGGTCTCCACCCCTGACTCCCCTCAGCCATAG
Latimeria chalumnae mitochondrion NC_001804.1 (12570) TACTAGGCCTAACTCTGGCAGCAGCAGGAAAATCAGCCCAATTTGGATTACACCCATGACTACCAGCAGCTATAG

Section 179

(13351) 13351 13360 13370 13380 13390 13400 13410 13425
Homo sapiens mitochondrion, complete genome NC_01... (13049) AAGGCCCCACCCCAGTCTCAGCCCTACTCCACTCAAGCACTATAGTTGTAGCAGGAATCTTCTTACTCATCCGCT
Latimeria chalumnae mitochondrion NC_001804.1 (12645) AAGGTCCAACACCGGTCTCTGCCCTACTACACTCAAGTACCATAGTGGTTGCCGGAATCTTCCTGCTTATCCGAC

Section 180

(13426) 13426 13440 13450 13460 13470 13480 13490 13500
Homo sapiens mitochondrion, complete genome NC_01... (13124) TCCACCCCCTAGCAGAAAATAGCCCACTAATCCAAACTCTAACACTATGCTTAGGCGCTATCACCACTCTGTTCG
Latimeria chalumnae mitochondrion NC_001804.1 (12720) TACACCCCCTCATAGACAATAATAAACTAATCCTCACTACCTGCCTCTGCCTAGGAGCACTAACCACCCTATTTA

Section 181

(13501) 13501 13510 13520 13530 13540 13550 13560 13575
Homo sapiens mitochondrion, complete genome NC_01... (13199) CAGCAGTCTGCGCCCTTACACAAAATGACATCAAAAAAATCGTAGCCTTCTCCACTTCAAGTCAACTAGGACTCA
Latimeria chalumnae mitochondrion NC_001804.1 (12795) CTGCCGCATGCGCACTCACCCAAAACGATATTAAAAAAATTATTGCATTTTCAACATCAAGTCAACTAGGACTAA

Section 182

(13576) 13576 13590 13600 13610 13620 13630 13640 13650
Homo sapiens mitochondrion, complete genome NC_01... (13274) TAATAGTTACAATCGGCATCAACCAACCACACCTAGCATTCCTGCACATCTGTACCCACGCCTTCTTCAAAGCCA
Latimeria chalumnae mitochondrion NC_001804.1 (12870) TAATGGTAGCAATTGGACTAAATCAACCACAACTAGCATTCCTCCACATCTGCACCCACGCTTTCTTTAAAGCAA

```
                                                                 Section 183
                                              (13651) 13651    13660     13670     13680     13690     13700     13710          13725
Homo sapiens mitochondrion, complete genome NC_01... (13349) TACTATTTATGTGCTCCGGGTCCATCATCCACAACCTTAACAATGAACAAGATATTCGAAAAATAGGAGGACTAC
      Latimeria chalumnae mitochondrion NC_001804.1 (12945) TGCTGTTCCTATGCTCCGGATCAATTATTCACAGTCTAAATGATGAACAAGATATCCGAAAAATAGGTGGCTTAC
                                                                 Section 184
                                              (13726) 13726         13740     13750     13760     13770     13780     13790   13800
Homo sapiens mitochondrion, complete genome NC_01... (13424) TCAAAACCATACCTCTCACTTCAACCTCCCTCACCATTG---GCAGCCTAGCATTAGCAGGAATACCTTTCCTCA
      Latimeria chalumnae mitochondrion NC_001804.1 (13020) ACAA--C-ATGCTCCCAACAACAAGCTCCTGTACAATAGTTAGCAGCATAGCCCTAACAGGAATGCCATTCCTAG
                                                                 Section 185
                                              (13801) 13801    13810     13820     13830     13840     13850     13860          13875
Homo sapiens mitochondrion, complete genome NC_01... (13496) CAGGTTTCTACTCCAAAGACCACATCATCGAAACCGCAAACATATCATACACAAACGCCTGAGCCCTATCTATTA
      Latimeria chalumnae mitochondrion NC_001804.1 (13092) CAGGCTTCTTCTCAAAAGACGCAATCATCGAATCACTAAACTCCTCTCACCTAAACGCCTGAGCCCTAACTACTA
                                                                 Section 186
                                              (13876) 13876         13890     13900     13910     13920     13930     13940   13950
Homo sapiens mitochondrion, complete genome NC_01... (13571) CTCTCATCGCTACCTCCCTGACAAGCGCCTATAGCACTCGAATAATTCTTCTCACCCTAACAGGTCAACCTCGCT
      Latimeria chalumnae mitochondrion NC_001804.1 (13167) CACTAATAGCCACATCATTCACCGCAGTCTATAGCCTCCGAATTATTTACCTTGTAATGATAAACTATCCACGAT
                                                                 Section 187
                                              (13951) 13951    13960     13970     13980     13990     14000     14010          14025
Homo sapiens mitochondrion, complete genome NC_01... (13646) TCCCCACCCTTACTAACATTAACGAAAATAACCCCACCCTACTAAACCCCATTAAACGCCTGGCAGCCGGAAGCC
      Latimeria chalumnae mitochondrion NC_001804.1 (13242) TTCAAAGCCTGTCCCCCATCGATGAAAATTATACAAAAACACGTAACCCAATTAAACGACTAGCATGAGGAAGCG
                                                                 Section 188
                                              (14026) 14026         14040     14050     14060     14070     14080     14090   14100
Homo sapiens mitochondrion, complete genome NC_01... (13721) TATTCGCAGGATTTCTCATTACTAACAACATTTCCCCCGCATCCCCCTTCCAAACAA---CAATCCCCCTCTACC
      Latimeria chalumnae mitochondrion NC_001804.1 (13317) TAATCGCAGGAATAATGAT--CTTCCTAAACATCCTAC-CAACTAAATCACAAACAATGACCATACCCACCCATA
                                                                 Section 189
                                              (14101) 14101    14110     14120     14130     14140     14150     14160          14175
Homo sapiens mitochondrion, complete genome NC_01... (13793) TAAAACTCACAGCCCTCGCTGTCACTTTCCTAGGACTTCTAACAGCCCTAGACCTCAACTACCTAACCAACA---
      Latimeria chalumnae mitochondrion NC_001804.1 (13389) TAAAAACAGCCGCAATCATAGTTACAATCCTAGGAGTCCTCACCGCTATAGAACTAACAAAACTTACAAGCACAC
```

Homo sapiens mitochondrion, complete genome vs. Latimeria chalumnae mitochondrion

Section 190

(14176) 14176 14190 14200 14210 14220 14230 14240 14250
Homo sapiens mitochondrion, complete genome NC_01... (13865) AACTTAAAATAA----AATCCCCACTATGCACATTTTATTTCTCCAACATACTCGGATTCTACCCTAGCATCACA
Latimeria chalumnae mitochondrion NC_001804.1 (13464) AACTAAAAATCACCCCAAACGTCAACATACACA----ACTCATCCAACATACTAGGATACTTCCCAAACATCATT

Section 191

(14251) 14251 14260 14270 14280 14290 14300 14310 14325
Homo sapiens mitochondrion, complete genome NC_01... (13936) CACCGCACAATCCCCTATCTAGGCCTTCTTACGAGCCAAAACCTGCCCCTACTCCTCCTAGACCTAACCTGACTA
Latimeria chalumnae mitochondrion NC_001804.1 (13535) CACCGACTTCTACCACAAACAAACTTATATCTAGGACAAAAAATAGCAACACACCTAACAGATCAAACATGATTT

Section 192

(14326) 14326 14340 14350 14360 14370 14380 14390 14400
Homo sapiens mitochondrion, complete genome NC_01... (14011) GAAAAGCTATTACCTAAAACAATTTCACAGCACCAAATCTCCACCTCCA---TCATCACCTCAACCCAAAAAGGC
Latimeria chalumnae mitochondrion NC_001804.1 (13610) GAAAAAATCGGGCCAAAAGGAATTTTAGCCCTACAAATCCCTACAACTAAAATAATCAACAACTCGCAACAAGGA

Section 193

(14401) 14401 14410 14420 14430 14440 14450 14460 14475
Homo sapiens mitochondrion, complete genome NC_01... (14083) ATAATTAAACTTTACTTC-CTCTCTTTCTTCTTCCCACTCATCC-----------TAACCCTACTC--------C
Latimeria chalumnae mitochondrion NC_001804.1 (13685) CTAATCAAAACATACCTGACCCTATTTTTTCTAACAACAGTTCTATTTACTACAATAACCATAATCTAACTGCTC

Section 194

(14476) 14476 14490 14500 14510 14520 14530 14540 14550
Homo sapiens mitochondrion, complete genome NC_01... (14138) TAATCACATAACCTATT--CCCCCGAGCAATCTCAATTACAATATATACACCAACAAACAATGTTCAACCAGTAA
Latimeria chalumnae mitochondrion NC_001804.1 (13760) GAAGCGCACCACGGGACAACCCACGAGTAATCTCCAAAACCACAAACAAAGCCAACAATAACACCCACCCAGTCA

Section 195

(14551) 14551 14560 14570 14580 14590 14600 14610 14625
Homo sapiens mitochondrion, complete genome NC_01... (14211) CTACTACTAATCAACGCCCATAATCATACAAAGCCCCCGCACCAATAGGATCCTCCCGAATCAACCCTGACCCCT
Latimeria chalumnae mitochondrion NC_001804.1 (13835) CCAACAACAAAAATCCCCCAGAAGAATAAACTATGGGCACACCACTGACATCC-CCTGA--CAAAACTGATATCT

Section 196

(14626) 14626 14640 14650 14660 14670 14680 14690 14700
Homo sapiens mitochondrion, complete genome NC_01... (14286) CTCCTTCATAAATTATTCAGCTTCCTACACTATTAAAGTTTACCACAACCACCACCCCATCATACTCTTTCACCC
Latimeria chalumnae mitochondrion NC_001804.1 (13907) CACC---AAAAA--------CATCCAATGATATTCAATAAAAATCATACCACCACCC-ATAACAATAATACCCAA

Section 197

	(14701)	14701 14710 14720 14730 14740 14750 14760 14775
Homo sapiens mitochondrion, complete genome NC_01...	(14361)	ACAGCACCAATCCTACCTCCATCGCTAACCCCACTAAAACACTCACCAAGACCTCAACCCCTGACCCCCATGCCT
Latimeria chalumnae mitochondrion NC_001804.1	(13970)	CAAGTAAAAAGCCAAAC---------AAATAAACCAAAATA-TAAAGAAAAACCGACCAACT--CCCCCAAGACT

Section 198

	(14776)	14776 14790 14800 14810 14820 14830 14840 14850
Homo sapiens mitochondrion, complete genome NC_01...	(14436)	CAGGATACTCCTCAATAGCCATCGCTGTAGTATATCCAAAGACAACCATCATTCCCCCTAAATAAATTAAAAAAA
Latimeria chalumnae mitochondrion NC_001804.1	(14033)	CAGGATAAGGCTCTGCTGCTAAAGCAGCAGAATAAGCAAAAACAACCAATATCCCCCCCAAATAAATCAAAAACA

Section 199

	(14851)	14851 14860 14870 14880 14890 14900 14910 14925
Homo sapiens mitochondrion, complete genome NC_01...	(14511)	CTATTAAACCCATATAACCTCCCCCAAAATTCAGAATAATAACACACCCGACCACACCGCTAACAATCAATACTA
Latimeria chalumnae mitochondrion NC_001804.1	(14108)	GTACTAAGGACAAAAAAGAGCCACCGTACCCAACCAAAAAGCCACAGCCAACAACTGCAGAAAATACTAACCCAA

Section 200

	(14926)	14926 14940 14950 14960 14970 14980 14990 15000
Homo sapiens mitochondrion, complete genome NC_01...	(14586)	AACCCCCATAAATAGGAGAAGGCTTAGAAGAAAACCCCACAAACCCCATTACTAAACCCAC--ACTCAACAGAAA
Latimeria chalumnae mitochondrion NC_001804.1	(14183)	GAGCAGCAAAATAAGGAGCTGGATTAGACGCAACTGCCATCAAACCAACCACGAGTCCCATCAACACAACAAAAA

Section 201

	(15001)	15001 15010 15020 15030 15040 15050 15060 15075
Homo sapiens mitochondrion, complete genome NC_01...	(14659)	CAAAGCATACATCATTATTCTCGCACGGACTACAACCACGACCAATGATATGAAAAACCATCGTTGT-ATTTCAA
Latimeria chalumnae mitochondrion NC_001804.1	(14258)	CAAA--ATAAATCATAATTCTTGCCCGGACTTTAACCAGAACCAATGACTTGAAAAACCACCGTTGTCAATTCAA

Section 202

	(15076)	15076 15090 15100 15110 15120 15130 15140 15150
Homo sapiens mitochondrion, complete genome NC_01...	(14733)	CTACAAGAACACCAATGACCCCAATACGCAAAACTAACCCCCTAATAAAATTAATTAACCACTCATTCATCGACC
Latimeria chalumnae mitochondrion NC_001804.1	(14331)	CTACA--AAAACCCATGACAAACATCCGAAAGACACACCCGCTAATTAAAATTATCAACGAAACCATCATCGACC

Section 203

	(15151)	15151 15160 15170 15180 15190 15200 15210 15225
Homo sapiens mitochondrion, complete genome NC_01...	(14808)	TCCCCACCCCATCCAACATCTCCGCATGATGAAACTTCGGCTCACTCCTTGGCGCCTGCCTGATCCTCCAAATCA
Latimeria chalumnae mitochondrion NC_001804.1	(14404)	TCCCCACACCATCAAACATCTCAATCTGATGAAATTTTGGGTCACTACTAGGAATTTGTTTAATTACACAAATCG

Section 204

(15226) 15226 15240 15250 15260 15270 15280 15290 15300
Homo sapiens mitochondrion, complete genome NC_01... (14883) CCACAGGACTATTCCTAGCCATGCACTACTCACCAGACGCCTCAACCGCCTTTTCATCAATCGCCCACATCACTC
Latimeria chalumnae mitochondrion NC_001804.1 (14479) TAACAGGCCTATTCTTAGCAATACACTACACAGCTGACATTACAACAGCATTCTCATCAGTAGCCCACATCTGCC

Section 205

(15301) 15301 15310 15320 15330 15340 15350 15360 15375
Homo sapiens mitochondrion, complete genome NC_01... (14958) GAGACGTAAATTATGGCTGAATCATCCGCTACCTTCACGCCAATGGCGCCTCAATATTCTTTATCTGCCTCTTCC
Latimeria chalumnae mitochondrion NC_001804.1 (14554) GAGATGTAAACTATGGATGACTAATCCGAAGTACCCATGCCAACGGAGCCTCTCTATTCTTCATCTGCATCTACC

Section 206

(15376) 15376 15390 15400 15410 15420 15430 15440 15450
Homo sapiens mitochondrion, complete genome NC_01... (15033) TACACATCGGGCGAGGCCTATATTACGGATCATTTCTCTACTCAGAAACCTGAAACATCGGCATTATCCTCCTGC
Latimeria chalumnae mitochondrion NC_001804.1 (14629) TACATGTAGCACGTGGACTCTACTATGGGTCATACTTACAAAAAGAAACCTGAAACATCGGAGTTATCCTCCTCA

Section 207

(15451) 15451 15460 15470 15480 15490 15500 15510 15525
Homo sapiens mitochondrion, complete genome NC_01... (15108) TTGCAACTATAGCAACAGCCTTCATAGGCTATGTCCTCCCGTGAGGCCAAATATCATTCTGAGGGGCCACAGTAA
Latimeria chalumnae mitochondrion NC_001804.1 (14704) TGCTAGTTATGATTACTGCCTTCGTGGGATATGTCCTCCCCTGAGGCCAAATATCATTCTGAGGGGCAACCGTCA

Section 208

(15526) 15526 15540 15550 15560 15570 15580 15590 15600
Homo sapiens mitochondrion, complete genome NC_01... (15183) TTACAAACTTACTATCCGCCATCCCATACATTGGGACAGAC-CTAGTTCAATGAATCTGAGGAGGCTACTCAGTA
Latimeria chalumnae mitochondrion NC_001804.1 (14779) TCACAAACCTCCTGTCAGCAGTACCCTATATTGG-AGATACACTAGTTCAATGAATCTGAGGAGGTTTTTCCGTA

Section 209

(15601) 15601 15610 15620 15630 15640 15650 15660 15675
Homo sapiens mitochondrion, complete genome NC_01... (15257) GACAGTCCCACCCTCACACGATTCTTTACCTTTCACTTCATCTTGCCCTTCATTATTGCAGCCCTAGCAACACTC
Latimeria chalumnae mitochondrion NC_001804.1 (14853) GACAACGCCACACTCACGCGATTCTTTGCCTTCCACTTCCTCCTGCCATTCGTAATCTCAGGAGCCTCGATTATT

Section 210

(15676) 15676 15690 15700 15710 15720 15730 15740 15750
Homo sapiens mitochondrion, complete genome NC_01... (15332) CACCTCCTATTCTTGCACGAAACGGGATCAAACAACCCCCTAGGAATCACCTCCCATTCCGATAAAATCACCTTC
Latimeria chalumnae mitochondrion NC_001804.1 (14928) CACCTGCTCTTCCTTCATGAGACAGGATCCAACAACCCAACTGGCCTTAACTCTGACGCAGATAAAGTAACCTTC

Section 211

```
                                                        (15751) 15751    15760     15770     15780     15790     15800     15810        15825
Homo sapiens mitochondrion, complete genome NC_01... (15407) CACCCTTACTACACAATCAAAGACGCCCTCGGCTTACTTCTCTTCCTTCTCTCCTTAATGACATTAACACTATTC
Latimeria chalumnae mitochondrion NC_001804.1 (15003) CACCCGTACTTCTCGTACAAAGACATGTTAGGATTCCTAATTACACTAACAACACTAGCATTCCTAACCCTATTC
```

Section 212

```
                                                        (15826) 15826         15840     15850     15860     15870     15880     15890   15900
Homo sapiens mitochondrion, complete genome NC_01... (15482) TCACCAGACCTCCTAGGCGACCCAGACAATTATACCCTAGCCAACCCCTTAAACACCCCTCCCCACATCAAGCCC
Latimeria chalumnae mitochondrion NC_001804.1 (15078) ACTCCAAACCTGTTAGGAGACCCCGAAAACTTCACACCAGCAAACCCATTAACCACCCCGCCACACATCAAACCA
```

Section 213

```
                                                        (15901) 15901    15910     15920     15930     15940     15950     15960        15975
Homo sapiens mitochondrion, complete genome NC_01... (15557) GAATGATATTTCCTATTCGCCTACACAATTCTCCGATCCGTCCCTAACAAACTAGGAGGCGTCCTTGCCCTATTA
Latimeria chalumnae mitochondrion NC_001804.1 (15153) GAATGATACTTCCTATTCGCCTATGCAATTCTACGATCCATCCCCAACAAACTAGGAGGAGTTCTAGCTCTAGTC
```

Section 214

```
                                                        (15976) 15976         15990     16000     16010     16020     16030     16040   16050
Homo sapiens mitochondrion, complete genome NC_01... (15632) CTATCCATCCTCATCCTAGCAATAATCCCATCCTCCATATATCCAAACAACAAAGCATAATATTTCGCCCACTA
Latimeria chalumnae mitochondrion NC_001804.1 (15228) TCCTCTATTCTAGTACTACTACTAGTGCCAATCTTACACACCTCAAAACAACGAGGAAACACCTTCCGCCCAATT
```

Section 215

```
                                                        (16051) 16051    16060     16070     16080     16090     16100     16110        16125
Homo sapiens mitochondrion, complete genome NC_01... (15707) AGCCAATCACTTTATTGACTCCTAGCCGCAGACCTCCTCATTCTAACCTGAATCGGAGGACAACCAGTAAGCTAC
Latimeria chalumnae mitochondrion NC_001804.1 (15303) ACCCAAATACTATTCTGAGCCCTTGTAGCAGACATGCTAATCCTAACCTGAATCGGAGGCCAACCAGTAGAATAT
```

Section 216

```
                                                        (16126) 16126         16140     16150     16160     16170     16180     16190   16200
Homo sapiens mitochondrion, complete genome NC_01... (15782) CCTTTACCATCATTGGACAAGTAGCATCCGTACTATACTTCACAACAATCCTAATCCTAATACCAACTATCTCC
Latimeria chalumnae mitochondrion NC_001804.1 (15378) CCATTCATGACAATCGGACAAATTGCTTCAATCACCTACTTCAGCCTATTCCTCATCCTAATCCCAA-TAACTGG
```

Section 217

```
                                                        (16201) 16201    16210     16220     16230     16240     16250     16260        16275
Homo sapiens mitochondrion, complete genome NC_01... (15857) CTAATT-GAAAACAAAATACTCAAATGGCCTGTCCTT-GTAGTATAAACTAATA-CACCAGTCTTGTAAACCGG
Latimeria chalumnae mitochondrion NC_001804.1 (15452) ATGACTAGAAAACAAGGCCATAAACTGAAACTAGCCTTAGTAGCTTAAACCCAAAGCACCGGCCTTGTAAGCCGG
```

Homo sapiens mitochondrion, complete genome vs. Latimeria chalumnae mitochondrion

```
                                                              Section 218
                                                     (16276) 16276         16290     16300     16310     16320     16330     16340  16350
Homo sapiens mitochondrion, complete genome NC_01... (15929) AGAT-GAAAAC----CTTTTTCCAAGGACAAATCAGAGAAAAA-GTCTTTAACTCC-ACCATTAGCACCCAAAGC
Latimeria chalumnae mitochondrion NC_001804.1        (15527) AGATTGAGAACTGACCATTCTCCTAAAGCTT-TCAGAGAAAAAAGGATCAAACTTTTACCCTTAGCTCCCAAAGC
                                                              Section 219
                                                     (16351) 16351    16360     16370     16380     16390     16400     16410         16425
Homo sapiens mitochondrion, complete genome NC_01... (15997) TAAGATTCTAATTTAAACTATTCTCTGTTCTTTCATGGGGAAGCAGATTTGGGTACCACCCAAGTATTGACTCAC
Latimeria chalumnae mitochondrion NC_001804.1        (15601) TAAAATTCTAATT-AAACTATTCTCTGTTC---CCAAGCTCTGCCCATCCACTCAATAACCCCCC--TCCTTCTA
                                                              Section 220
                                                     (16426) 16426         16440     16450     16460     16470     16480     16490  16500
Homo sapiens mitochondrion, complete genome NC_01... (16072) CCATCAACAACCGCTATGTATTTCGTACATT----ACTGCCAGCCACCATGAATATTGTACGGTA-----CCATA
Latimeria chalumnae mitochondrion NC_001804.1        (15670) GTACGTACTATCTCTATGTATATCGAACATTCGTTACTCTCAAGTACATTATACATTACATGTTAATTTACCATT
                                                              Section 221
                                                     (16501) 16501    16510     16520     16530     16540     16550     16560         16575
Homo sapiens mitochondrion, complete genome NC_01... (16138) AATACTTG---ACCACCTGTAGTACATAAAAACC-CAATCCA--CATCAA-----AACCCCCTCCCCATGCTTAC
Latimeria chalumnae mitochondrion NC_001804.1        (15745) AGAACTGTCACACCACATTTAGGGCGAGAAACCAGCAATACTTGCATAAATGATTAATTAATTTACGATGTGGTT
                                                              Section 222
                                                     (16576) 16576         16590     16600     16610     16620     16630     16640  16650
Homo sapiens mitochondrion, complete genome NC_01... (16202) AAGCAAGTACAGC--AATCAACCCTCAACTATCACACAT-----CAACTGCAACT---CCAAAGCCACCCCTC-A
Latimeria chalumnae mitochondrion NC_001804.1        (15820) AGACACTTATTTCTTAATCTAAACTTGGCTTTATCCATTACTGGCCACTGGTACTGTGCGATGGAGAATAATAGA
                                                              Section 223
                                                     (16651) 16651    16660     16670     16680     16690     16700     16710         16725
Homo sapiens mitochondrion, complete genome NC_01... (16266) CCCACT---AGGATACCAACAAACCTACC-----CACCC--TTAACAGTACATAGTACATAAAGCCATTTA---C
Latimeria chalumnae mitochondrion NC_001804.1        (15895) AAGATTTATATGATTATAAATTATCTATTACTGGCATCTGGTTTTGGGTTTAGTGAGGGGAAGGGCTTTTTAACC
                                                              Section 224
                                                     (16726) 16726         16740     16750     16760     16770     16780     16790  16800
Homo sapiens mitochondrion, complete genome NC_01... (16328) CGTA---CA-TAGCACATTACAGTCAA----ATCCCTTCTCGTCCCCATGGATGACCCCCCTCAGATAGGGGTCC
Latimeria chalumnae mitochondrion NC_001804.1        (15970) CGTAACTCAGTATCACTTTACTATACTGGCCAGCCGTTATTTTTTTGGGGGGGGGAACGTGAAGTTAGACAGCA
```

Homo sapiens mitochondrion, complete genome vs. Latimeria chalumnae mitochondrion

```
                                                                            Section 225
                                                      (16801) 16801    16810     16820     16830     16840     16850     16860          16875
Homo sapiens mitochondrion, complete genome NC_01... (16395) CTTGACCACCATCCTCCGTGAAATCAATATCCCGCACAAGAGTGCTACTCTCCTCGCTCCGGGCCCATAACACTT
      Latimeria chalumnae mitochondrion NC_001804.1 (16045) CTTAACTTGCGTCAAACGTCGTTT-ATGATTGGACTTTTAGTCGACACTCAAGTACTTTTGGATCTATGACAAAG
                                                                            Section 226
                                                      (16876) 16876          16890     16900     16910     16920     16930     16940  16950
Homo sapiens mitochondrion, complete genome NC_01... (16470) GGGGGTAGCTAAAGTGAACTGTATCCGACATCTGGTTCCTACTTCAGGGTCATA-----AAGCCTAAATAGCCCA
      Latimeria chalumnae mitochondrion NC_001804.1 (16119) GATAATCAGTTAA-TGATAGATA---GACATATAATGAATGATAGATAGACATATAATGAATGATAAATAGAC-A
                                                                            Section 227
                                                      (16951) 16951    16960     16970     16980     16990     17000     17010          17025
Homo sapiens mitochondrion, complete genome NC_01... (16540) CACGTTCCCCTTAAATAAGACATCACGATG---------------------------------------------
      Latimeria chalumnae mitochondrion NC_001804.1 (16189) TATAATGAATGATAGTTAGATATATAGATTAATGTAAGAAAGATAATTGAACCCATGACAGAGGACATACTTTTA
                                                                            Section 228
                                                      (17026) 17026          17040     17050     17060     17070     17080     17090  17100
Homo sapiens mitochondrion, complete genome NC_01... (16570) ---------------------------------------------------------------------------
      Latimeria chalumnae mitochondrion NC_001804.1 (16264) ATGATTTCAGGACATAAACACCATGCACATCAGATTGTATCAACTAAACCCCTCCCAACCCCTAAAACCCAGGAC
                                                                            Section 229
                                                      (17101) 17101    17110     17120     17130     17140     17150          17169
Homo sapiens mitochondrion, complete genome NC_01... (16570) ---------------------------------------------------------------------
      Latimeria chalumnae mitochondrion NC_001804.1 (16339) TCGCTAAACACATCAACCCATATATTTTTTCAGTATATACATTGTTATACATCAAATAAATAATGTAAT
```
