# Supplementary material for: Patch type nucleotide sequence identities between genomes from many different species facilitate illegitimate recombination
Source: Sci Rep. 2026 Mar 30;16:10524. doi: 10.1038/s41598-026-44124-0 (PMC13035915; doi:10.1038/s41598-026-44124-0)
Supplement: Supplementary file 7 — Supplementary Material 7 [file 41598_2026_44124_MOESM7_ESM.pdf]

Mus musculus chromosome 19 vs. Triticum aestivum chromosome 6D

```
                                                                     Section 1
                                                         (1)   1        10        20        30        40        50        60             75
Mus musculus strain C57BL/6J chromosome 19 NC_000...     (1)   GCGTCGTCTCTGACTGGGGTCTGCTGTGCCCAGCCCAGGTCTTGGCCCCTCCTCTCCTGCCCCAGCA-AGGCTTC
Triticum aestivum cultivar Chinese Spring chromosome 6...(1)   --------------------------------TCCTTTATCTTAGTCACAATAC--CAGCCAAAAAACAGACTTG
                                                                     Section 2
                                                         (76)  76            90        100       110       120       130       140      150
Mus musculus strain C57BL/6J chromosome 19 NC_000...     (75)  CTGCCAACCCATTTGGCACTGTGCCCTCTC-TGAGCAGGAGCCAGCTTGTTTGCAGCCGGTGTTAATTAAGAGAG
Triticum aestivum cultivar Chinese Spring chromosome 6...(42)  CTGC--ACCCTTTTC-CATTGATCTCTGAAATGAGGGAAAGGCAATCATTTTCCAA---GTGAACAGGACCATCG
                                                                     Section 3
                                                         (151) 151      160       170       180       190       200       210            225
Mus musculus strain C57BL/6J chromosome 19 NC_000...     (149) AGGTG--GTGGGGGGAGGTG---GGTAGGGCCGCGGAGGGCAGCTTC----CGACAGCCGCCAGCGCTGCC-CCA
Triticum aestivum cultivar Chinese Spring chromosome 6...(111) TGGTGTGGTAGGACAACTTCTAAGCTACGTATGCCCACAATAGCTTCAACACTTTGGCATTGTGGGATGCAGCCC
                                                                     Section 4
                                                         (226) 226           240       250       260       270       280       290      300
Mus musculus strain C57BL/6J chromosome 19 NC_000...     (214) GGGGCTCAGAGGGCTGGAGTCAG---GCCTGGGCCCACCTGAGATGCCTCATCTCCC-ACTGTCGGTTCCCACGA
Triticum aestivum cultivar Chinese Spring chromosome 6...(186) CAGGCTCATAGTCATAGAAGCACACAGCCTGTATCCTCCCACAATGCTGCACCCCATGAACCTGAATTCTCAAAA
                                                                     Section 5
                                                         (301) 301      310       320       330       340       350       360            375
Mus musculus strain C57BL/6J chromosome 19 NC_000...     (285) GCTCCTGCCGGAGTGCGTCCTTCGCGTTTGCAACCAAGCCTGCCCTTGCCGGGCGTGGAGCAATCGAGGCTGAGG
Triticum aestivum cultivar Chinese Spring chromosome 6...(261) CCTGCATCAAAATTCAGT--TTCGC-----CCACCCAGC-TGCCGGTTTTGTCCAACAATGAATCAA--CTTACA
                                                                     Section 6
                                                         (376) 376           390       400       410       420       430       440      450
Mus musculus strain C57BL/6J chromosome 19 NC_000...     (360) TAATAGAGGCTCCAGAGGGCTCCTTGCTTTGAGGGAGTAGGCGTGGCCAGGAGGTGTAACCATG-TAACCGTGCC
Triticum aestivum cultivar Chinese Spring chromosome 6...(326) TT-TCCATGCTCTTTTAT-CTCATCAATCATGAAGAGGGGGCTTTTCCTTTTGCATCCACAAGGATTTCAGTGGC
                                                                     Section 7
                                                         (451) 451      460       470       480       490       500       510            525
Mus musculus strain C57BL/6J chromosome 19 NC_000...     (434) TCTTGGTGGCCTTACCT-TCCAGT-------TCAGAGAGACCCAGGTTTAGTCTTTATTTATTTGGAACCAAGGT
Triticum aestivum cultivar Chinese Spring chromosome 6...(399) TCTTCTATCCCTATCCTATACAGAGGTCATATAAGAGTCAAAAAAATTGAAGATTGTTGGATGGCACACTTACCA
```

```
                                                                   Section 8
                                                      (526) 526           540       550       560       570       580       590       600
Mus musculus strain C57BL/6J chromosome 19 NC_000...  (501) CTCCTGTAGCCCCGGTTGGTT-TAGAATTCCTTACATAATAGAAGATGACCTTGAACTTCTGATCCTTCCTGCCT
Triticum aestivum cultivar Chinese Spring chromosome 6... (474) TCACTGAAAATTGAGTTGTTCCTAAAATGCCATAATCTCCACCAGA-GAAGTAGTAGTTTTTCTC--TCATGGAC
                                                                   Section 9
                                                      (601) 601      610       620       630       640       650       660            675
Mus musculus strain C57BL/6J chromosome 19 NC_000...  (575) CCACCTCCCAGAGTGATAA-AATTACAAATTACGGGCATCAGCTACTATGTC---TGGTATACACATGGAAATCT
Triticum aestivum cultivar Chinese Spring chromosome 6... (546) TCATCAATGTTACTAAGAAGAAGAAGAACCCAATCATATCATGTA-TATCCGAGTTGTTGTTCATCAGGTAATGG
                                                                   Section 10
                                                      (676) 676           690       700       710       720       730       740       750
Mus musculus strain C57BL/6J chromosome 19 NC_000...  (646) GTATA-TCTGGTATATACATGGTTGGAAATCCAACCCAAGGCTTCCTACATGCACAAGCCCTTGAAATGTACCCA
Triticum aestivum cultivar Chinese Spring chromosome 6... (620) CAATTCTCTTCT-TAGGCAATGACGAAGGGCCCACACTTTTGTGCAGGATTTCATAGCGTGTTAGCTTGTTTCAG
                                                                   Section 11
                                                      (751) 751      760       770       780       790       800       810            825
Mus musculus strain C57BL/6J chromosome 19 NC_000...  (720) GCTCCA-GCTTCC-----TACACCACCCTATACCTGCTTTCTCTCTCTCTCTCTCTCTCTCTCTCT---CTCTCTCT
Triticum aestivum cultivar Chinese Spring chromosome 6... (694) GTTCCATGCCACCGATGTTATAGGTAGCAAAATGGTCCATATTTCTCTTGTTC-CGATGTGCCTGAACTCCTAGA
                                                                   Section 12
                                                      (826) 826           840       850       860       870       880       890       900
Mus musculus strain C57BL/6J chromosome 19 NC_000...  (786) CTCTCTCTCTCTCTCTCTCTCCCTCTCTCCCTCCCTCTCCCTCCCTCTCCCTCTCCCTCTCCCTCTCCCTCCCTC
Triticum aestivum cultivar Chinese Spring chromosome 6... (768) GTGTTCCTATCAAGTTTCCAACCAAAGACTAACCTTTAGAGGCACATTTGCTTTCCAGATCAGATTCCATAATTC
                                                                   Section 13
                                                      (901) 901      910       920       930       940       950       960            975
Mus musculus strain C57BL/6J chromosome 19 NC_000...  (861) CCCTTCAGTTGGCCCATCTGAACTTCGTTTTCCTTCTCTGTAAAATGGGAC-CAGGAGGCCCTCCCTGGTGACTT
Triticum aestivum cultivar Chinese Spring chromosome 6... (843) ACTCTCACCTG---CAGGTTTGCTGCTCATGCCTGCATCATTGCTGGCAACTCCATAGCTAGTCTATATGCACTC
                                                                   Section 14
                                                      (976) 976           990       1000      1010      1020      1030      1040      1050
Mus musculus strain C57BL/6J chromosome 19 NC_000...  (935) GGCTCTGGGATAAACAGGGTCCTTTAAGAAGAATGGCA-GCCTATCTCGCTAAGTATTTACCCTTCCATATGAT
Triticum aestivum cultivar Chinese Spring chromosome 6... (915) TTAACCGGGGAAAGCGCATTCTTTTCATAA---TGCCATGCAAATGAAATCATCTCCTGTCAATCTTGATATTCT
```

Section 15

```
                                                       (1051) 1051      1060      1070      1080      1090      1100      1110          1125
   Mus musculus strain C57BL/6J chromosome 19 NC_000... (1009) CATGTCCTGGAGAGCCTCTG----GGGACCAGAGTGGGACTTCAGTACCC--TGCCCCTTCTGGGGTCCTTGGTG
Triticum aestivum cultivar Chinese Spring chromosome 6... (987) AATTTGTGACACTTGATCTGTATCTGTAGCATGAAACGACTTCCTGACCAAATGTTCTTTC---GCCCATTTGTG
```

Section 16

```
                                                       (1126) 1126          1140      1150      1160      1170      1180      1190      1200
   Mus musculus strain C57BL/6J chromosome 19 NC_000... (1078) GCTG-ACCTCTTA--CCCGGTAGTGTCTCCCAGGTTGCTTGCCTACATGCTCAGCTAGC-TCCTTCTTCGTTCCC
Triticum aestivum cultivar Chinese Spring chromosome 6... (1059) CCTGGATCAAGTAAGTCCGAGACCCACCTCAATCTAGAGTTTGTA---GCTTTCCTAGAATTTTTAAATTTACTC
```

Section 17

```
                                                       (1201) 1201      1210      1220      1230      1240      1250      1260          1275
   Mus musculus strain C57BL/6J chromosome 19 NC_000... (1149) ATGGCCTCCACTT---AGTTCTAGGAGCTCCCGGCCGCTCCTT--GCCTCTCCATCTTAATTAGCTGCTT----G
Triticum aestivum cultivar Chinese Spring chromosome 6... (1131) ATGGAATCCAGTTGTCCATCCAAATTTTATTTTGGTGCTCAATTAGATTCTCCAAATAACACCCTTTTTTCGAGG
```

Section 18

```
                                                       (1276) 1276          1290      1300      1310      1320      1330      1340      1350
   Mus musculus strain C57BL/6J chromosome 19 NC_000... (1215) TATTAATC-TCGAAAGTAACGTGGCAACTTGCAGCCCCGGGTTTTCAGGCTGTGGAATTAATTAT-TCCCCAAGC
Triticum aestivum cultivar Chinese Spring chromosome 6... (1206) TCTAAACCATACATAATTCCTTGCCAACATGGTGAAGTACTTTGAATAACGGTCGTGTCAATCAAATCACCTGAA
```

Section 19

```
                                                       (1351) 1351      1360      1370      1380      1390      1400      1410          1425
   Mus musculus strain C57BL/6J chromosome 19 NC_000... (1288) GAGGAGCTGTCAGGTCTGGGAGACCAGCCACTGCGAGCCACCCCGACACCACTTAACAGCATCCATAGCTTCCGC
Triticum aestivum cultivar Chinese Spring chromosome 6... (1281) GGGTAATATTTCGTTGTATGAACGCAAGCACAAAGACCCTGCA-GCTAAGATGAGTCTACATGCTT-GCTTT-GC
```

Section 20

```
                                                       (1426) 1426          1440      1450      1460      1470      1480      1490      1500
   Mus musculus strain C57BL/6J chromosome 19 NC_000... (1363) AAAAAAAGCCCTGGGCCACAGTCTGGCCCCTAACCCTAAAGCAGAAAGCCCAGGAAGGTGAGGTAAGAGTCAACC
Triticum aestivum cultivar Chinese Spring chromosome 6... (1353) CAGAAGAGCTTGATTAAATAGTCTG------AAATCTCAAAAACCAATGCCACCCTG-------AATCTTCTGTT
```

Section 21

```
                                                       (1501) 1501      1510      1520      1530      1540      1550      1560          1575
   Mus musculus strain C57BL/6J chromosome 19 NC_000... (1438) CTGGGAGGTGCTGAAGGCCCAGCCTGGGA--GTCAGGCTC--CCTGGGCCTCTAACCTGGGCAGCAACCAGGTCT
Triticum aestivum cultivar Chinese Spring chromosome 6... (1415) TTGTCATTTTATCCAAAGCCATCCAGTGAACTTTAATCTTATCTTAATCTTCCCACCAAAAAATCCTAATAATAT
```

Mus musculus chromosome 19 vs. Triticum aestivum chromosome 6D

Section 22

	(1576)	1576 1590 1600 1610 1620 1630 1640 1650
Mus musculus strain C57BL/6J chromosome 19 NC_000...	(1509)	GGGGAACCTGTGGCCTCCCCTAACTCCTGGTCTGGTTGGCACTGGG--ATTTCTTAGGGATGTAGACCTTGTGGC
Triticum aestivum cultivar Chinese Spring chromosome 6...	(1490)	GGGACACCTCACTACTAGGGAAAAGCCTAGTAG---TAGCACGGGTTAAATGCTTATCA--GTAGCGCGGGTGGC

Section 23

	(1651)	1651 1660 1670 1680 1690 1700 1710 1725
Mus musculus strain C57BL/6J chromosome 19 NC_000...	(1582)	TAGAAGGGGACTCCAGGCTGAGCAGGAGCAATGATCAGCACAGCCCTAGCATTCTAGAAGAGGGGCTGCCAGTCC
Triticum aestivum cultivar Chinese Spring chromosome 6...	(1560)	C-GCTCTACTTTGGCGTCCGTCCTGCATACCCCCCGGTCACAATACTTCTTTGCGATAGTTTTTTTGTGCAGTGC

Section 24

	(1726)	1726 1740 1750 1760 1770 1780 1790 1800
Mus musculus strain C57BL/6J chromosome 19 NC_000...	(1657)	CCCTTACCTATCGTCAGCCACAGGGCCACGTGGTA--AGGCAGGAGGGGCATTCA-----GCTGGCAGAGA-AG
Triticum aestivum cultivar Chinese Spring chromosome 6...	(1634)	CACGAAAGGATCTACCTGGTCTGGGTGTTGTAGCACTATCAAGTTTGCTCTGTCAAAGTCGTTGCGTCGATCTAA

Section 25

	(1801)	1801 1810 1820 1830 1840 1850 1860 1875
Mus musculus strain C57BL/6J chromosome 19 NC_000...	(1724)	GATGGCTATG-GC--TACCTGGGGAAAGTGGTAGCCATGATGGCTGTCAGGCC---CACTCTGCCTGAGGAGGGT
Triticum aestivum cultivar Chinese Spring chromosome 6...	(1709)	GTGGGCCATGTGCAGTTCCTTGCGACCGTTGGAGTGACCTTCTCCTTCAAGCCTCCCATCGTGCTTGTTAACGGG

Section 26

	(1876)	1876 1890 1900 1910 1920 1930 1940 1950
Mus musculus strain C57BL/6J chromosome 19 NC_000...	(1793)	CCCCTCCACACCAGGCAGAAAG-CAGGGCACTCTCCTATAGCCAGGCC---AGAACTG-ACTCCTTTTCAGGAC
Triticum aestivum cultivar Chinese Spring chromosome 6...	(1784)	CAAACCAACACCAGGCGGCTGGTCTGCGCCATATAGAAAATTCTCGCAGAAAGAGTTGCACTCTTGTGTCCGATA

Section 27

	(1951)	1951 1960 1970 1980 1990 2000 2010 2025
Mus musculus strain C57BL/6J chromosome 19 NC_000...	(1863)	CTTCACCCCAACCCTGTGAGCT----GGGGTATTTTTCCCAGTTTACATTGGA--AACAGAGGCCTGGGGCCAGG
Triticum aestivum cultivar Chinese Spring chromosome 6...	(1859)	GCCCTGGCCGATGCTTCTATCCGGACGGGACATGTTACGAACGAATCCTTTGATGATCCCATTCATCCTCTCAAA

Section 28

	(2026)	2026 2040 2050 2060 2070 2080 2090 2100
Mus musculus strain C57BL/6J chromosome 19 NC_000...	(1932)	CTGCCCTGCTCAGTGGGTGCTGGCTGGCACTCAGACTTTTGACAAGGCCCACAGTGTGGCTTCCC---TCCACCA
Triticum aestivum cultivar Chinese Spring chromosome 6...	(1934)	CGGCATCATGTTGTGCAGGAATGACGGCCCCGGGTCTATT-ATGTCGTCCACAATATGGACACACAGATGCACCA

Mus musculus chromosome 19 vs. Triticum aestivum chromosome 6D

Section 29

	(2101)	2101 2110 2120 2130 2140 2150 2160 2175
Mus musculus strain C57BL/6J chromosome 19 NC_000...	(2004)	CATATTCCAGACTCTGTGGCCTTCTGCACA---CACGCTGCCTTCT---TCTCTTTGGTGTTTT----TAG---T
Triticum aestivum cultivar Chinese Spring chromosome 6...	(2008)	TGACGTCAAAGAACGCGGGCAGAAAGTACATCTCAAGCT-CATTCAGTATCACAACGATCTCTTCCTGTAGCCTT

Section 30

	(2176)	2176 2190 2200 2210 2220 2230 2240 2250
Mus musculus strain C57BL/6J chromosome 19 NC_000...	(2066)	CAGGGTCTCTACTTAGTCCTCACTGTCCTGGAGCTCACT------AAGTAGACCAGCCTGGCCTCGGATTCAGAG
Triticum aestivum cultivar Chinese Spring chromosome 6...	(2082)	CGGAGCTGCTTCACACTGATCGACTTTCGAGAGATGACGTCGAAAAAGTTGCAGAGACCAATAAGCGTGTCACAT

Section 31

	(2251)	2251 2260 2270 2280 2290 2300 2310 2325
Mus musculus strain C57BL/6J chromosome 19 NC_000...	(2135)	A--TCCATGTGCCTCTGCCTCCTGAGAGCTGGATTAAGAGTGTGTG-CTGCCGGGCGTGATGGTGCACGCCTTTA
Triticum aestivum cultivar Chinese Spring chromosome 6...	(2157)	ACGTGCTTGTCCATTATCCCTCTAAGGGCAACAGGTAGTATCTGCGTCATCATCACATGACAGTCATGGGACTTC

Section 32

	(2326)	2326 2340 2350 2360 2370 2380 2390 2400
Mus musculus strain C57BL/6J chromosome 19 NC_000...	(2207)	ATCCCA--GCACTTGGGAGGCAGAGGCAGGTGGATTTCTGAGTTCGAGGCCAGCCTGGTCTACAAAGTGAGTTCC
Triticum aestivum cultivar Chinese Spring chromosome 6...	(2232)	ATCCCGCTGAACCTTTTCTTCTGCGTGTC-TAGATATCTTCTTATTTTGCCA--CAGTAACTGAAACTAACTTTG

Section 33

	(2401)	2401 2410 2420 2430 2440 2450 2460 2475
Mus musculus strain C57BL/6J chromosome 19 NC_000...	(2280)	AGGACAGCCAGGGCTATACAGAGAAACCCTGTCTCAAAAAAAAAAAAAAAAAAAAAAAAAAAAAAAAAAGTATGT
Triticum aestivum cultivar Chinese Spring chromosome 6...	(2304)	ACTCTGGTAAGGCACTTGAAGAATTGATCGATCTCAGCCGGACTTAAGGTGAAGCAAGAAGGGGGCAATA-ATCC

Section 34

	(2476)	2476 2490 2500 2510 2520 2530 2540 2550
Mus musculus strain C57BL/6J chromosome 19 NC_000...	(2355)	GCCACCGTGCCCCATGAGCACTTCCTTCTTGGTG-----GCCTCCAACTAGGCAGCCTGTGGGCCATGAGGGTCA
Triticum aestivum cultivar Chinese Spring chromosome 6...	(2378)	TCTTCCTTTTCTTTTTTGCCATTCTTCTCCCGCGCCTCTGTCTCCATCTCTGTGTCCTCTGACTTTTTACGGGGC

Section 35

	(2551)	2551 2560 2570 2580 2590 2600 2610 2625
Mus musculus strain C57BL/6J chromosome 19 NC_000...	(2425)	GGACCATGCTCTGGCCTCCCCATACCTATGCTGAAGGCTGGACACTTCCGGGTCTCAAGAAACGCT--GTGAAGG
Triticum aestivum cultivar Chinese Spring chromosome 6...	(2453)	GG--CATGTGCAGATCTTCCCTGATTTTCAAATCTTGCAAGTCTTTTCTTGCCTTCGGCGCATCCTTGGTCTTAT

Mus musculus chromosome 19 vs. Triticum aestivum chromosome 6D

```
Section 36
                                                  (2626) 2626          2640      2650      2660      2670      2680      2690    2700
 Mus musculus strain C57BL/6J chromosome 19 NC_000... (2498) AGAGCAGGCAGAATACAGGCCGGGAGTAAACACCCATAATTCCAGTGGAGGCAGGAGCACTACAGAGCCATCCCC
Triticum aestivum cultivar Chinese Spring chromosome 6... (2526) CCGGCATGTTCATTAGTGTC-GCAAGCAAGCTCTCA-ACGACATTCTTATAGATATGCACTT--GATCAAGGCAA
Section 37
                                                  (2701) 2701     2710      2720      2730      2740      2750      2760          2775
 Mus musculus strain C57BL/6J chromosome 19 NC_000... (2573) TGTACATAGT-GAATTGAAGCCAGT-CTAGAATTCAGGAAACTCTGTCTTTAAAAAATAAGGGGGTGAGGCTGG
Triticum aestivum cultivar Chinese Spring chromosome 6... (2597) TGAGGTGTGTCGATTTTGTGCCAGTACTCCAAGTCCCAGAAAACAGACCTCGGCTTCCATACCT--------TCG
Section 38
                                                  (2776) 2776          2790      2800      2810      2820      2830      2840    2850
 Mus musculus strain C57BL/6J chromosome 19 NC_000... (2646) GCAGTGGGGGCACGAGCCTTTAATCC-CAGTGAAGGCAGGTGGATCTCTGAGTTCAAGGCCAGCCTGGTCCACAA
Triticum aestivum cultivar Chinese Spring chromosome 6... (2664) GCAGCGGCTCCGCGCCTTTTTCATCGTCTTTCCCGGCGCGGGGCACT----GTTCCCAGTTTTTCAACAGCTCAT
Section 39
                                                  (2851) 2851     2860      2870      2880      2890      2900      2910          2925
 Mus musculus strain C57BL/6J chromosome 19 NC_000... (2720) AGAGAGTTTCAGGACAGCCAGGGCTACACAGAGAAACCCTATATCAGAAAAAAAGCACAGTGTGGAGGTGGGGGG
Triticum aestivum cultivar Chinese Spring chromosome 6... (2735) CGA---TTTCAGCGCCGCAACGCTTACGTGGAGGTCCTCGATG-CTCAGCCTCACCATTGAATAGATCTCCACGG
Section 40
                                                  (2926) 2926          2940      2950      2960      2970      2980      2990    3000
 Mus musculus strain C57BL/6J chromosome 19 NC_000... (2795) GCAGAGGGCATCAAGATTGTTCAGTGAACAAAGGCGTCTGCCAC-TAAGCCTGACAGCCTGAAGTCCAACCACGG
Triticum aestivum cultivar Chinese Spring chromosome 6... (2806) TTT-----CTCCACGGGTCGTCCTTCTCGA--GCCAGATGCCCCATGTACACGATTTTCCCA-GACCCACCATCT
Section 41
                                                  (3001) 3001     3010      3020      3030      3040      3050      3060          3075
 Mus musculus strain C57BL/6J chromosome 19 NC_000... (2869) GATCCACTTTGTGGAAGGAGAGAACCAACTCCTGCCAGTTGTCCTCTGACCTGCACATACATGC-TGTAACCCTG
Triticum aestivum cultivar Chinese Spring chromosome 6... (2873) TTCCTTGATGTTAGCTGCTGAGACGTCGTATCATCCA-TGCACCGCGTGCATTCGCA-ATATCCATGGCACACCT
Section 42
                                                  (3076) 3076          3090      3100      3110      3120      3130      3140    3150
 Mus musculus strain C57BL/6J chromosome 19 NC_000... (2943) TGTACTGCTCCTTCTGGGTGGTCAGGGGTAGCCTTGC----TTGTGCCCACTTCCTGATCCCCTATAAGCTCAGG
Triticum aestivum cultivar Chinese Spring chromosome 6... (2946) GGT-CTGCGACATATCTGTAACC-GAGATAGTCGTGCACCATCGTGATCAGCGCGAC-TCTCGTATAGAAATAGT
```

```
                                                                                        Section 43
                                                            (3151) 3151     3160      3170      3180      3190      3200      3210          3225
Mus musculus strain C57BL/6J chromosome 19 NC_000...        (3014) CCCCGTTAGGAGCTCCCCAACCCCCATCATTGGC--TCTTGCACAAAGGGCTGAGTATCCTATACCCAGAGTCTC
Triticum aestivum cultivar Chinese Spring chromosome 6...   (3018) CGCCTTT----GCTGCCGT-CCCATGTCTTGGCCGGTGTTTCCATAACGTGTAATATTATTTCTGGTTGTTTC
                                                                                        Section 44
                                                            (3226) 3226          3240      3250      3260      3270      3280      3290   3300
Mus musculus strain C57BL/6J chromosome 19 NC_000...        (3087) GGACCCAGGCTC--CCTGCTCCCAGCTGCCTGACTGTCCCTCTACCCCATGTAGCCCGTGCCTTTGACCCCTCCC
Triticum aestivum cultivar Chinese Spring chromosome 6...   (3088) GGCCCTTGAATCAGCATGCTCA----TG--TGTATGTACTTCGACTTCATGCACAACCTGGGGGAGGTTGTACA
                                                                                        Section 45
                                                            (3301) 3301     3310      3320      3330      3340      3350      3360          3375
Mus musculus strain C57BL/6J chromosome 19 NC_000...        (3160) GCACTGCA--CTTACCTGACTTTGTCTCTCTCTTTCTCTGTCTTGCAGCCAACAGCTCCCTGCTTGGAGGCGGAG
Triticum aestivum cultivar Chinese Spring chromosome 6...   (3157) TCCATACAAACACAGGCGACGTGCTATGGTTGGTGTTCTGG-TTGC--CAAACGGATTCATGCCATC-GGTACAC
                                                                                        Section 46
                                                            (3376) 3376          3390      3400      3410      3420      3430      3440   3450
Mus musculus strain C57BL/6J chromosome 19 NC_000...        (3233) GGGGTGAGTACTAGACCTTCCT-GAGTCTCCTGTAAGGGTGGAGTGGGGGTGGGGTATGGGTCTC--CTG--TTT
Triticum aestivum cultivar Chinese Spring chromosome 6...   (3228) GCGCCGAGCAC--GATGTTCCTTGCATCACCTTAAAAATTCTGATATTCGCCATTGAACGCTCTCCACTGGCTTC
                                                                                        Section 47
                                                            (3451) 3451     3460      3470      3480      3490      3500      3510          3525
Mus musculus strain C57BL/6J chromosome 19 NC_000...        (3303) CCTTCTTCAGGTCCCTACAATTTCAGTCAGTCCCC----TCTGGTGTATTCAT-------TGATAAAGCTAGAGG
Triticum aestivum cultivar Chinese Spring chromosome 6...   (3301) CATCCTTGACGTGTCT-CAGCTTCGGATCATCTCCATCATCGGGCTTCTCCCTCTCCGCGTGCCAGCGCATGAGC
                                                                                        Section 48
                                                            (3526) 3526          3540      3550      3560      3570      3580      3590   3600
Mus musculus strain C57BL/6J chromosome 19 NC_000...        (3367) CCTGGCTCCT----CTGTAGGGTA-ACCTCCCCACCC------CCACCCCTGAC-ACACCTGAACT------CAA
Triticum aestivum cultivar Chinese Spring chromosome 6...   (3375) TTTGCTTCCTTGGGATCTACGAAATACCGCTGGAGACGGGGAGTGATCGGTAAGTACCATAGAACTTTCTGGGGA
                                                                                        Section 49
                                                            (3601) 3601     3610      3620      3630      3640      3650      3660          3675
Mus musculus strain C57BL/6J chromosome 19 NC_000...        (3424) GCCTCCCTCCCGGCTTGTACCTGAACACGCTGGAGCTGGAGGCTGGGGCTGGTAGACAAAGCCTCACTCTGTGC
Triticum aestivum cultivar Chinese Spring chromosome 6...   (3450) GATTTCTTCCCGGCCTT--CTTGTAT--GGAGAAGCATTGCACACCGGATAGCTTGTTTTTTCCTCGTGCTCCTT
```

Section 50

```
                                                        (3676) 3676          3690      3700      3710      3720      3730      3740    3750
Mus musculus strain C57BL/6J chromosome 19 NC_000...    (3499) CTGATGCCGGCTGGGC-CTTTGAAAGCTCCTTGCTAATTGTCCT-CAGCTTTTCCACTGT-CTC-TGCACCTCAT
Triticum aestivum cultivar Chinese Spring chromosome 6... (3521) CCGATGAATTATGCAATCGTTGATGCATGCATGGTATCTAACGTGCGGCAGATCAAGAGGGCACATGATCTTCAT
```

Section 51

```
                                                        (3751) 3751     3760      3770      3780      3790      3800      3810          3825
Mus musculus strain C57BL/6J chromosome 19 NC_000...    (3570) CCATCTCGGCACTCAAAAACCCCAGGTGTCTGCCAGGTAGTATCTCAGACCAGCGCTACAGCACCAGGT-AGGGA
Triticum aestivum cultivar Chinese Spring chromosome 6... (3596) CGCCTTATCCACACTAGTAGGACATAAGATTCCCCGTGGGAAGAACATCCTAGAGGTACTTGAGATGCTCATCGA
```

Section 52

```
                                                        (3826) 3826          3840      3850      3860      3870      3880      3890    3900
Mus musculus strain C57BL/6J chromosome 19 NC_000...    (3644) A-CGGGTGAGTGACAAGGGGACAAAGGCCAGTG-CAGCAGGTGCCTCGAGAGGGGCAGCCCTGTGGCCCCACATC
Triticum aestivum cultivar Chinese Spring chromosome 6... (3671) GGCTAGTGTCGGTCCATTTGTTTTTAGCCTTCGTCTTTAAGAGT-TCGAGCGTGAAACTCAAGCGGGTC-ACCTC
```

Section 53

```
                                                        (3901) 3901     3910      3920      3930      3940      3950      3960          3975
Mus musculus strain C57BL/6J chromosome 19 NC_000...    (3717) TGCCT--CAACCAGCCTCCCACCACCCACTCAGCCCCTCAGCCCCCAGCTGCCGGGCAGCCACTGTCTGCGCAGT
Triticum aestivum cultivar Chinese Spring chromosome 6... (3744) AGGATTGCAACCATGATACAATGTAGCGTTCGAGTCTACCACCAGTTGCTGCAGCTGGGCCTC---CTCTCTAGA
```

Section 54

```
                                                        (3976) 3976          3990      4000      4010      4020      4030      4040    4050
Mus musculus strain C57BL/6J chromosome 19 NC_000...    (3790) GGCGGCAGGATGATTGGTGAGC-CACGGGGCAGGGGTTAATGCGCCGCGGGATAAATCCCGGGGGCCATGTTTAC
Triticum aestivum cultivar Chinese Spring chromosome 6... (3816) AGCAGCTCTCTCAGTACTCGTCTCCTTGCGAAGCACTACTTGAACATGAGGGTCCCGCACGATTGAACTTAGTAC
```

Section 55

```
                                                        (4051) 4051     4060      4070      4080      4090      4100      4110          4125
Mus musculus strain C57BL/6J chromosome 19 NC_000...    (3864) -GGCAGCGGTGGCGGGGCCCAGGCCAGGTTTTAAAAGGGGAAAA----ATAAAAGGGGCAGCGA-AGGTCAGGA
Triticum aestivum cultivar Chinese Spring chromosome 6... (3891) TGACAAATTCTACGGCGTGGAGTCCATGTTCTCTCCGCCGCCATGTCCGACACCTTCTCCATCGGCATGTCACTA
```

Section 56

```
                                                        (4126) 4126          4140      4150      4160      4170      4180      4190    4200
Mus musculus strain C57BL/6J chromosome 19 NC_000...    (3933) ATAAATAAGCACGCACTAAGAGCTGTCGGTGACCAGCCAGGAT--GCAGGGGCTGTTTGTGACTGAGGATGCCT
Triticum aestivum cultivar Chinese Spring chromosome 6... (3966) CTAGGAAAACCCTTACCAGTAGCGCTGTTTTTTTTGCTATTAGTAGCGCTGGCAGGCGCGCTACTGCTACGGCGC
```

```
                                                                Section 57
                                                     (4201) 4201     4210      4220      4230      4240      4250      4260           4275
Mus musculus strain C57BL/6J chromosome 19 NC_000... (4006) TCCCTCCCCACTCTCCTCTAGCTGTCCC---CGTGGAGGTCACCGCTTCCCAGGATGGGGGTTTGGTGCTCATCT
Triticum aestivum cultivar Chinese Spring chromosome 6... (4041) TAAA-----ACTATTTTGTAGCAGTAGCGCTTGTTTAGGCCAGCGCTACT---------GGTATGTT---CAGCT
                                                                Section 58
                                                     (4276) 4276          4290      4300      4310      4320      4330      4340      4350
Mus musculus strain C57BL/6J chromosome 19 NC_000... (4078) ATAGGCCCTTGGGGGCCTCTTTACAAGCCTGACCTTTAAGAGGAACAGGTCCCTCTGTGGTTGGTGACACCACAC
Triticum aestivum cultivar Chinese Spring chromosome 6... (4099) ACTAG---TAGCGCGCCTCTGGA-AAGCGGTACTGGTAA-ATGAGCACTGCTGGTAATATTTTGGCCCGCGCCAC
                                                                Section 59
                                                     (4351) 4351     4360      4370      4380      4390      4400      4410           4425
Mus musculus strain C57BL/6J chromosome 19 NC_000... (4153) TCTAACCACAGTACCCTTCCCACACCTATTCATTCCTCACTCGGTGCTCT-CCTGTGTGCACAGAGCTGTGAGCT
Triticum aestivum cultivar Chinese Spring chromosome 6... (4169) TGCTA--AAATTTATGTTC---------TTTTTTCGTCTTTTGGCGCTGTACATGTTTAAACAGATATATCTTTT
                                                                Section 60
                                                     (4426) 4426          4440      4450      4460      4470      4480      4490      4500
Mus musculus strain C57BL/6J chromosome 19 NC_000... (4227) GGACACTGACAGACCAGCCACGACAGGATAGTGGGAGGTCCCAGGTACATCAGGGCTCAGCATGAACACCGGGAA
Triticum aestivum cultivar Chinese Spring chromosome 6... (4233) ATACAATGGCAACTCATCATGAACTAGTCTGTTTAAA-TAGCATATTCATTACCACTAGTCATCACCACCAAACA
                                                                Section 61
                                                     (4501) 4501     4510      4520      4530      4540      4550      4560           4575
Mus musculus strain C57BL/6J chromosome 19 NC_000... (4302) AATTTCAGGAAGGAAAGTGGGGCCTGGACTGGTGAGGAGAGGTGGGAAG-AGT-GTCCTGAGTGGAGGGAACAGA
Triticum aestivum cultivar Chinese Spring chromosome 6... (4307) ATGTTCGTCAATGAGA------CATCATATATATAACA-AGTTGGTCACTAGTCGTAATCACAACTCCTCATCCT
                                                                Section 62
                                                     (4576) 4576          4590      4600      4610      4620      4630      4640      4650
Mus musculus strain C57BL/6J chromosome 19 NC_000... (4375) CAGAAAGGCCCCAAGATGAAGGGAGCAGGGTATTGAAGAGCCTCAGCTGGCCAGAGGCCTCAGATGGCAGCCCAG
Triticum aestivum cultivar Chinese Spring chromosome 6... (4375) CCTCATCAACTCTAACACATTGTAGCACATTATAACACATTCTACCTAGGACCTACTCCTCTCATAGGACCTACT
                                                                Section 63
                                                     (4651) 4651     4660      4670      4680      4690      4700      4710           4725
Mus musculus strain C57BL/6J chromosome 19 NC_000... (4450) AGACCTGAAGAGCAACGAGAACAGAAACCTTGGGAGGAGCTAAGGTGTTAGCATTTCCTCTCGGGAAGGCAGAGA
Triticum aestivum cultivar Chinese Spring chromosome 6... (4450) CTACCCT-----CTCTTAGGTAAAATATCATAAAACAAGATAGG-------CATTGACTCTCCATTAAGGAAA-A
```

Mus musculus chromosome 19 vs. Triticum aestivum chromosome 6D

Section 64

		4726 … 4800
	(4726)	4726 4740 4750 4760 4770 4780 4790 4800
Mus musculus strain C57BL/6J chromosome 19 NC_000...	(4525)	TGGAGGACGAATGTGGGCTTTCCAGCTTGCTATTCTTTTTTTGTGTTTTTGT-TTTGTTTTGTTTTGTTTTCGAG
Triticum aestivum cultivar Chinese Spring chromosome 6...	(4512)	TGGACTCTCCATAATGAAGAACGGAGATC--ATCCTGTCTCCAAGTCTTGGCCTACGCACAATGTTGCTTCCAAG

Section 65

		4801 … 4875
	(4801)	4801 4810 4820 4830 4840 4850 4860 4875
Mus musculus strain C57BL/6J chromosome 19 NC_000...	(4599)	ACAGCGTTTCTCTGTGTAGCCCTGGCTGTCCTGGAACTCACTTTGTAGACCAGGCTGGCCTCGAACTTTGAAATC
Triticum aestivum cultivar Chinese Spring chromosome 6...	(4585)	T-AGC---TCTCTCCGATGGTCGAATTTTTTTTCCAATCATTTATTAT-CATGGCTT-CCTTGCTTTTAGAAATC

Section 66

		4876 … 4950
	(4876)	4876 4890 4900 4910 4920 4930 4940 4950
Mus musculus strain C57BL/6J chromosome 19 NC_000...	(4674)	CACCTCCCTCTGCCTCTGCTGAATTAGAGGCGTGCGCCACCACGCCCTGCTCCAGCTTGCCATTCTTGACCCGCT
Triticum aestivum cultivar Chinese Spring chromosome 6...	(4654)	CGGTATGGACAGGAG----TGATGTGGATAC-TGCGGCT----GACCTGGCCGTGGTGGCCGT-----AAGCTCA

Section 67

		4951 … 5025
	(4951)	4951 4960 4970 4980 4990 5000 5010 5025
Mus musculus strain C57BL/6J chromosome 19 NC_000...	(4749)	CAAAACCCTCACGTCC--ATGAGAATCCCATCTAAGGCCCTCACTGTGGCTCAGAAGGACCAGCCACCTCTCAGC
Triticum aestivum cultivar Chinese Spring chromosome 6...	(4715)	TAACATCAACACGACCTCTTGGCAACATCAGACGAGGCACACAATC-----CATCGGGATTTTCTAT---TCAAA

Section 68

		5026 … 5100
	(5026)	5026 5040 5050 5060 5070 5080 5090 5100
Mus musculus strain C57BL/6J chromosome 19 NC_000...	(4822)	CTCCCTGCACTCCAGGCCTTCCCCACAACCACACCTGCCTCCCTGCACTGGGTTAGGTACACTCGCCTCAGGGCC
Triticum aestivum cultivar Chinese Spring chromosome 6...	(4782)	AACATAGTAATAA----CTTTGTAGTTAGCAATGTAGTTTAGTTTTAGAAGAATTTATGCAAAAGATGCACGGAT

Section 69

		5101 … 5175
	(5101)	5101 5110 5120 5130 5140 5150 5160 5175
Mus musculus strain C57BL/6J chromosome 19 NC_000...	(4897)	TGGGTACT-GTGGGTGGCAGGAG-AGGCACACTGCACCAGCTGTTCTCCCATCCTTCCGTGTG------TGGGGG
Triticum aestivum cultivar Chinese Spring chromosome 6...	(4853)	GTCGTAATAGTGGAAAATCGTACCATGCTATCTCCATT-GATGTTACCGTAGCACACCGCGTGCACTAGTGACAC

Section 70

		5176 … 5250
	(5176)	5176 5190 5200 5210 5220 5230 5240 5250
Mus musculus strain C57BL/6J chromosome 19 NC_000...	(4964)	GTAGGGATG-TGCTTTTCCCGAGGGCATCATCCCCTCTTACAAGCCTTTACTTGAATACGGGTTTCC--CAGAGG
Triticum aestivum cultivar Chinese Spring chromosome 6...	(4927)	ATATTGACCATAATTTTAAGGAATTCGATAATAGCTATTGTAATTCTCAAGATCAGTAAAAAATGCAATAAGACC

Section 71

	(5251)	5251 … 5325
Mus musculus strain C57BL/6J chromosome 19 NC_000...	(5036)	GACCCAC-CCTGAGTACTGGCGGAGGCTGCAGCCT-GCAGTCCTCACCCTGGCTTTCACTCTACTGTAGTTGTTT
Triticum aestivum cultivar Chinese Spring chromosome 6...	(5002)	ATCTTTCTCCTTATAAGTTAATTGTGCTTCATCATTGTAGTAGTAGGTTCTTTGTACCATCTTCCGTACATTTTT

Section 72

	(5326)	5326 … 5400
Mus musculus strain C57BL/6J chromosome 19 NC_000...	(5109)	TCTGAGAACCACCCCCACTTCTACCATGTGACAATTCCTCATCACTACCTTCAC-TGCCTTCTGTCTGTCCCTCA
Triticum aestivum cultivar Chinese Spring chromosome 6...	(5077)	TGA-AGAATGAAAATAAGTTGT-CAATG-GAAATAAGCTGTGAAATATTTTGAAATAAAAAATATAAATTACTTA

Section 73

	(5401)	5401 … 5475
Mus musculus strain C57BL/6J chromosome 19 NC_000...	(5183)	AAAGGAA------AGGAATTGCCAG-GATATGGGCCTGAACACTCTCCCATGAAGGGAGGGACTCGCCCCTGAGG
Triticum aestivum cultivar Chinese Spring chromosome 6...	(5149)	ATAAATATGTTTGAGAAACTCACACAGCGGTAGAACTGGAAGCG-TGTTAACAAGGACCCAAATGGTCA-TATTA

Section 74

	(5476)	5476 … 5550
Mus musculus strain C57BL/6J chromosome 19 NC_000...	(5251)	GTAGCAGGCAGAAGT-AGGCT-AGGCAGAGGGGTGGCTTGCAGGC-ATCCTGGTGCTGC-CTTAAGCCATGGGGT
Triticum aestivum cultivar Chinese Spring chromosome 6...	(5222)	TTTTC--GTCGATGTCAGGATCACCAAGATCGATGGTGAGAAGCATACCCTCATGAAAATCATACGCCTTGCAAA

Section 75

	(5551)	5551 … 5625
Mus musculus strain C57BL/6J chromosome 19 NC_000...	(5322)	GCTCTGGCTCCCTTTTCAG---CCTTAGAGGTGCGGGGAGG-CTGGGTGATAAGGAGATGG-GTGGAGCAGTGAC
Triticum aestivum cultivar Chinese Spring chromosome 6...	(5295)	GGGCT---TCCCAATTCAGGCAACCAAAATTGGATGAGTTCACAGAATTATATAGATTTACAGCAAAATCATAAC

Section 76

	(5626)	5626 … 5700
Mus musculus strain C57BL/6J chromosome 19 NC_000...	(5392)	CCTGGAGGAAGTCCGGCCTTTCATC-TAAATGCAGCTAAGATTTATGGAGCCTCTGCAGCTAGGC-CTGGGATAC
Triticum aestivum cultivar Chinese Spring chromosome 6...	(5367)	CATGATGG--GTCCTTAGTTGAATTATCTTTGTCTCCATGCTTTCATGATCTTCAAAACCCATCTTCTCCAAGAC

Section 77

	(5701)	5701 … 5775
Mus musculus strain C57BL/6J chromosome 19 NC_000...	(5465)	AGCCCTGCTAAG--GAAATTGGGGCAGGTGGAAGTGCAGGACAGGGCCGTGGAGGGAGGAGGCAGAGCCTGGCGG
Triticum aestivum cultivar Chinese Spring chromosome 6...	(5440)	ATAGCGTCTTACATGGCATGGGATAAGCTAGTCGAATTGTAAAAGACGGAAATTACACGTCGAAGAAGCAGAGAA

Section 78

(5776) 5776 5790 5800 5810 5820 5830 5840 5850
Mus musculus strain C57BL/6J chromosome 19 NC_000... (5538) GCCTGGCATCTGAAGTTGAAATGCAGCCTGCCAGTTACCCGGACATACCCCTTCA-CATATGACACATCTGTCA-
Triticum aestivum cultivar Chinese Spring chromosome 6... (5515) GTTGTGCAAAAAATAACAAAAAACA-CTTGTCGTTGTTGCGTACGGTTTTCAACATCGAAGGTCTCTTGTAGCTT

Section 79

(5851) 5851 5860 5870 5880 5890 5900 5910 5925
Mus musculus strain C57BL/6J chromosome 19 NC_000... (5611) ----CCCCATCACCAACCCTG-ACCCCAGGAAGACCCTGGGACCGATCGTGGGGGCCTGAAGTTCTTCCTGTAAG
Triticum aestivum cultivar Chinese Spring chromosome 6... (5589) GATGCTGAAGCGCCGACCTTCTACCCGGTGAGGCCTGTCGCACAGACCGCG-----CTCATCTT--TGCAGTACT

Section 80

(5926) 5926 5940 5950 5960 5970 5980 5990 6000
Mus musculus strain C57BL/6J chromosome 19 NC_000... (5681) AGCTCAGGTCTCTACCTGCCCTTATCTGTCCTCC-TCTCAGGAT-GGCCCTGGAGAAGCCTTGTAATGTCAGCCA
Triticum aestivum cultivar Chinese Spring chromosome 6... (5657) CGCACA--TAGCGAATTCCCTTTCGTCGTCAGACATTTCCTAATAGGTAATTAATAATCCATC--ATCGAATACA

Section 81

(6001) 6001 6010 6020 6030 6040 6050 6060 6075
Mus musculus strain C57BL/6J chromosome 19 NC_000... (5754) CAGACCAGCAGACATGCTACCAAGCTCTTGTCCCTAGCTCTGGGCC-CTCAGACTGTACAGTGCTCAGTGCTCTA
Triticum aestivum cultivar Chinese Spring chromosome 6... (5728) TATAT-AGTAGTT-TGTAGCAAAGATCATCATATAACAACTAAAACACCTAAAATTTGTAGTTTGTAGTAGTTTG

Section 82

(6076) 6076 6090 6100 6110 6120 6130 6140 6150
Mus musculus strain C57BL/6J chromosome 19 NC_000... (5828) CCACCAGGATGTCTTTCCTGAGAAGGTCAGGACCTAGGACACTGAGCTC-TGGAGCCCCTCACGGTGACCTCTGC
Triticum aestivum cultivar Chinese Spring chromosome 6... (5801) TAGCAAAGATCATCATCGAACCTAGTTT-GTAGCAAAGATCATCATATAATCCATCATCTAATACATATATAGTA

Section 83

(6151) 6151 6160 6170 6180 6190 6200 6210 6225
Mus musculus strain C57BL/6J chromosome 19 NC_000... (5902) TCAGCTAGCACAAGCGGGGCAGACTCTATGCTCTCCGTGTGTCTGCCCAGGGCTGAGGGTCCGT----GGGTACT
Triticum aestivum cultivar Chinese Spring chromosome 6... (5875) GTTTCCAGCACAAACCACGCTCATCTTTTGCATTCAATAAGCAAATAAGTAATTAATAATCCATCATCGAATACA

Section 84

(6226) 6226 6240 6250 6260 6270 6280 6290 6300
Mus musculus strain C57BL/6J chromosome 19 NC_000... (5973) CAGGGCTTTGCTCTGCAGTCAAG--CGGCCCAATTTAGAAGTAGGTT-TCTCAAGCAAAGGATGCTGGGACTTGG
Triticum aestivum cultivar Chinese Spring chromosome 6... (5950) TATATAGTAGTT-TGTAGCAAAGATCATCATATAATATCACTAATACATCTCGAATAATAGCTCCT----CTAGG

Section 85

	(6301)	6301 6310 6320 6330 6340 6350 6360 6375
Mus musculus strain C57BL/6J chromosome 19 NC_000...	(6045)	GCCTTGAAGACTGAATAGGAGTTTAGCAGGCTAGCAGG-ATGGAGAGATATGAGGGACGATGACCTGATGGCCTG
Triticum aestivum cultivar Chinese Spring chromosome 6...	(6020)	TTCA-GTGGGCCGCGGGGGACACCCAAAGAGAAGGAACCATCACCGGATCATAGCTCCGGTGA---GATCCCCAA

Section 86

	(6376)	6376 6390 6400 6410 6420 6430 6440 6450
Mus musculus strain C57BL/6J chromosome 19 NC_000...	(6119)	GGGAAGTCCTAGTCTCCTGGTGGAGCTGGTGACTACCTGGGCCACTCTCTTCCCATGCCTCTGCCCTATCTCCCT
Triticum aestivum cultivar Chinese Spring chromosome 6...	(6091)	AGAATCTGCCAGG-TATTGGAGAACCGGCCGTTCGCCAATGCGACCATGTAGCACTGGACGTGCGCGTTCTCCTC

Section 87

	(6451)	6451 6460 6470 6480 6490 6500 6510 6525
Mus musculus strain C57BL/6J chromosome 19 NC_000...	(6194)	GTGGAAAGCCAGTTATTGGCTCAATAAAAAGATGTCTGAAACA-CTCTCCTATTGTATGGTAAGGAGGGAGGCGG
Triticum aestivum cultivar Chinese Spring chromosome 6...	(6165)	CGTGACA--CGGTGCTGTACCACCTGCGCGGGGGACTCAAGCCTCTGCACTAATGCAGGCCCATGTGACCGCCA-

Section 88

	(6526)	6526 6540 6550 6560 6570 6580 6590 6600
Mus musculus strain C57BL/6J chromosome 19 NC_000...	(6268)	GTCCTAAATCAGGATAGGGATCGACAAGGCCCAAGCGCCTCAACTGTTTATTAGCACTGAGATGTTTACTTGGCG
Triticum aestivum cultivar Chinese Spring chromosome 6...	(6237)	---CCAAAGAAGCTCCAGG-TCGACGACGGGCTGGCTCCTCACTAAGTTGCGCTCACCAGAAGGTATCACAACCC

Section 89

	(6601)	6601 6610 6620 6630 6640 6650 6660 6675
Mus musculus strain C57BL/6J chromosome 19 NC_000...	(6343)	ATGAGGAGCCGGCTTTCTCGGGGAGATTTGTGC-GTTCTTTTTCAATTACCCCTCGTCT---TTCTCTGCCACTT
Triticum aestivum cultivar Chinese Spring chromosome 6...	(6308)	AGTACCAGCCCG-------GCGGAGCCCAGTCCCGGACATGGCCCCTCGGCTCAAGCAGGAGTCCTCCGCCGAGT

Section 90

	(6676)	6676 6690 6700 6710 6720 6730 6740 6750
Mus musculus strain C57BL/6J chromosome 19 NC_000...	(6414)	TTGCACTAAAGGGATAATAAATGTATCGGAG-CGTTTCAATTATTCATCTAAATCATGACTGCGCCTGCCATGGC
Triticum aestivum cultivar Chinese Spring chromosome 6...	(6376)	CGACGACGATGATGCGGGATAGGCATCGTCGACGTCGAGAACAAAATTCTTAATTATGAAAACAAAATTAATAAA

Section 91

	(6751)	6751 6760 6770 6780 6790 6800 6810 6825
Mus musculus strain C57BL/6J chromosome 19 NC_000...	(6488)	CAAGTCTTAGTGACAGATAAATTCAGGGCCGCCGCCTCTGCCGCCATGGCTGCCATCTCTGCCCCAGCCAACCAG
Triticum aestivum cultivar Chinese Spring chromosome 6...	(6451)	CA---CTTAG---CA---AAATTCGG---CATGACCTTTGCTAAAA-GCAGGACATATCGAGCGCCTGAAATTTG

Section 92

	(6826)	6826 6840 6850 6860 6870 6880 6890 6900
Mus musculus strain C57BL/6J chromosome 19 NC_000...	(6563)	CGCCCCAAAGGGCCAACACTGGGAGCCAGCGGTTGGGGTCAGACAGGTCCTTGGCAGGAGGGCAAGGCAGGCTGG
Triticum aestivum cultivar Chinese Spring chromosome 6...	(6513)	C----CAGAACGTAAAC-----GAATCGACATTTCTGGCAAAACATAGGCCACTCGGAAAAATATGACATCTTCA

Section 93

	(6901)	6901 6910 6920 6930 6940 6950 6960 6975
Mus musculus strain C57BL/6J chromosome 19 NC_000...	(6638)	GAAGGGGGAAGGAGCCCTTGTCCTCACCCTCTACC-------CCTGCTTGGGCCTCACGTGTTATCAGCCCCCCA
Triticum aestivum cultivar Chinese Spring chromosome 6...	(6579)	AAATATGACATGTCCACTACAAATTTGCATATAACAGGAAAAATTGCTTTAACTAAAAAAGATAACAACAATTTC

Section 94

	(6976)	6976 6990 7000 7010 7020 7030 7040 7050
Mus musculus strain C57BL/6J chromosome 19 NC_000...	(6706)	TGAAGCTACAGGAA--CTGAGGGTCCTACCTATCTGTTCACACATCGGCTCCCGGGCC-ACCCTGCACTGCCTCA
Triticum aestivum cultivar Chinese Spring chromosome 6...	(6654)	TTTAACTAAAAAAATACCTAGAATTCTGA-TAACTACACCTAAAACAACTAAAACACCTAAAATTCTGTTAACTA

Section 95

	(7051)	7051 7060 7070 7080 7090 7100 7110 7125
Mus musculus strain C57BL/6J chromosome 19 NC_000...	(6778)	CCCCTACAGCCA-TATCCCAGCACCACATGCTGGGAGAGGCCAGGATGGTGGTGCATCCTTCCAGCCATCCCCTG
Triticum aestivum cultivar Chinese Spring chromosome 6...	(6728)	CACCTAAAACAACTAAAACACCTAAAACTCTTTTAAATACAGCTAAAACACCTAAATTCTATTAACTA-CACCTA

Section 96

	(7126)	7126 7140 7150 7160 7170 7180 7190 7200
Mus musculus strain C57BL/6J chromosome 19 NC_000...	(6852)	GCATGGGACCTTTGTACCTGCTGCTGCTCCTCCGCCCTCCTGCCCAGAGTCCTACCTGACAATAGCTCCTGCTGG
Triticum aestivum cultivar Chinese Spring chromosome 6...	(6802)	ATTAAAAACCTAGCTAAAT--TTCTG----TCCTAACTTTA------AAACCTAGCTAAATTTAAAAAC-GCAGG

Section 97

	(7201)	7201 7210 7220 7230 7240 7250 7260 7275
Mus musculus strain C57BL/6J chromosome 19 NC_000...	(6927)	CCACACCCCAAA-ATCTAGGGATGGAGAGCCTAGCACATGTCATCTCTATC-TTGAACCAT--CCT-CAGTATTC
Triticum aestivum cultivar Chinese Spring chromosome 6...	(6864)	GTTCATACAAATTAACTAGGGTTC-ATA-CCTA--ATGTGTCCTAGCTAGGGTTCGATCATGACCTACATTATTC

Section 98

	(7276)	7276 7290 7300 7310 7320 7330 7340 7350
Mus musculus strain C57BL/6J chromosome 19 NC_000...	(6997)	TGTCACCCTTGA------CAACTATTCAAGAT-CTGTCTGCCCCATGTGCACCATCAGGACCACTGATGGCACAG
Triticum aestivum cultivar Chinese Spring chromosome 6...	(6935)	TAAGAACCTACATTTTTTCAACTATATAGGATTCAAAATAACCACTCTCTAATAACTATAACTAGGGAGGGAG-G

Section 99

(7351) 7351 7360 7370 7380 7390 7400 7410 7425
Mus musculus strain C57BL/6J chromosome 19 NC_000... (7065) GCACAGGTGGACAGCACGTGTTGGGAGCACAC--TTGTCAACTGCCGCTGTGCTCGGCCATAGCCTCATACTTGT
Triticum aestivum cultivar Chinese Spring chromosome 6... (7009) GAAGAGGAGGA-AGGAGGGAGGAGTACCTCTCGGTGGAGGAGGGCCGGTGCGGGGGG-----GCGCCCTGCGGGG

Section 100

(7426) 7426 7440 7450 7460 7470 7480 7490 7500
Mus musculus strain C57BL/6J chromosome 19 NC_000... (7138) GTGTGCAGCCTGCGTCCTCTCCCCTCACACAGGATATGTATGAGTACACTGTGGCAAGCTCACACTTCAGCGGCT
Triticum aestivum cultivar Chinese Spring chromosome 6... (7078) GAGGATGGCCGATGCGAGCGGCCGGCCGGCAGGGGAGGAGGGCCGGCGCGGGG--AAGCGCGGCCAACGGGGGAA

Section 101

(7501) 7501 7510 7520 7530 7540 7550 7560 7575
Mus musculus strain C57BL/6J chromosome 19 NC_000... (7213) CACCGGGCCTCTCAGTGGCTCCTTTCTATCTGCCCGGATCTCTGTCCCTTTGGCTCACCCATCCTTTTCTACCGC
Triticum aestivum cultivar Chinese Spring chromosome 6... (7151) GAGAGGCCCGCGCGGGGGAGGGCGGC---CGGAGTAGGACGCGGGGGCGGCGGCCGGATCGGGGGACGATTGAGG

Section 102

(7576) 7576 7590 7600 7610 7620 7630 7640 7650
Mus musculus strain C57BL/6J chromosome 19 NC_000... (7288) TCTTCCCTCTGCTTTCTTTTCCCCTCGCTTTCCTTTGTTTCTGCGAGG----ATTAGCA---AGGCCTTTGAGTA
Triticum aestivum cultivar Chinese Spring chromosome 6... (7223) GATTCGGTCTGTGAGTGTGAGCGACAGAGTGTGAGTGAGTGTGAGAGGGGAGAGGGGCGCGCGGGCCGGTGCGGG

Section 103

(7651) 7651 7660 7670 7680 7690 7700 7710 7725
Mus musculus strain C57BL/6J chromosome 19 NC_000... (7356) TGGGACCCAGCCTGGCCTTCCTGTCAATTCTTTGCAAGCAGCTAGCTGCC-ATCACTGCTGCTAAT-CTGTGATT
Triticum aestivum cultivar Chinese Spring chromosome 6... (7298) AGGAAGCAGAGTTGGGCTTAGCGGCA-------GCGTGGGGTGGAGGCCCAGCGCTATAGCTAAAGCTGCAACA

Section 104

(7726) 7726 7740 7750 7760 7770 7780 7790 7800
Mus musculus strain C57BL/6J chromosome 19 NC_000... (7429) GTTTCCGTGGCCTCTGGGAAAGCT--GGCACTGCTGCCCTGATGAATGGTAGCCTGACTCAGTGGAGAACTTAAC
Triticum aestivum cultivar Chinese Spring chromosome 6... (7366) GGGGGCCCAGAGCGTGGCCCATCAACGGCAGCGCTGGCCGG--GAA----ATCCGGCGCTACTGC------TAAC

Section 105

(7801) 7801 7810 7820 7830 7840 7850 7860 7875
Mus musculus strain C57BL/6J chromosome 19 NC_000... (7502) CTCCACAGCCTGTGGGCCGCGGTTGGTCGGTCGGTGCTCCAGTGCTCCGGAAGCCAGGGTGTGTGAAGTTGCCCC
Triticum aestivum cultivar Chinese Spring chromosome 6... (7429) GTGC---GCTTGTAGCACTGGTGTTGTC---CGGCGCTACTGCTAAATAGCAGT-AGGGTGGG-GTCTCTAACCA

```
                                                                                    Section 106
                                                     (7876) 7876          7890      7900      7910      7920      7930      7940    7950
   Mus musculus strain C57BL/6J chromosome 19 NC_000... (7577) ACCCCTCTTTGGCCACCTGTGGGACTCAAAAGCAGCAGGTAGCTTGAGAACTCCCAAGAGCCAGGAAGCTTGCTG
Triticum aestivum cultivar Chinese Spring chromosome 6... (7496) ACGCTACT---GCTAAATGTGTACCTATAA----GCATTTTCCT--AGTAGT-----GTGTCCGGCCCCTTCTCC
                                                                                    Section 107
                                                     (7951) 7951     7960      7970      7980      7990      8000      8010          8025
   Mus musculus strain C57BL/6J chromosome 19 NC_000... (7652) GACTCAGTGTTGAGCCTGGGCCTATCTGCAAGAGTAGGGGTGCAGCTTTAGCACAAAGAGGGACCCTCAAAAATG
Triticum aestivum cultivar Chinese Spring chromosome 6... (7557) GCCTCCATGTCCGTTCTCTCCCCTGCCGCCATGCCAGG----CCCCTTCTCCGCC----GCCATTATCGATCATT
                                                                                    Section 108
                                                     (8026) 8026          8040      8050      8060      8070      8080      8090    8100
   Mus musculus strain C57BL/6J chromosome 19 NC_000... (7727) GCTACGTGCTGGGGGCTGGAGAGATGGCTCAGTGGTTAAGAGGTCCTGTGTTCAGTTGCCAACAGCCACATGGTG
Triticum aestivum cultivar Chinese Spring chromosome 6... (7624) TCTTCGTCTTGCCCCGTGTCGTCATTGC-CTGCCCCGTCGGCGTCCTCTA--CAT----CATCATCCTCATCTTC
                                                                                    Section 109
                                                     (8101) 8101     8110      8120      8130      8140      8150      8160          8175
   Mus musculus strain C57BL/6J chromosome 19 NC_000... (7802) GCTCACAACCATCT-GTAATTGGGTCCAATGCTCTCTTCTGGTGTATTTGAAGACAGTGA-GAGTGTACTCACAT
Triticum aestivum cultivar Chinese Spring chromosome 6... (7692) ACTTAT--CCATCGAGTATAGCCATCCATGAAACCATTCATGAGCAGGTGCGCTTCGACACGACCATCGTCATAG
                                                                                    Section 110
                                                     (8176) 8176          8190      8200      8210      8220      8230      8240    8250
   Mus musculus strain C57BL/6J chromosome 19 NC_000... (7875) ATAT--ATATATATTTTTTTTTAAATCTCAAAAAGACAAAACAAAACAAAACAAAACAAGG-CATCCGGGCAGT
Triticum aestivum cultivar Chinese Spring chromosome 6... (7765) GGGTCGAGCCAAACTCGTCCTTTGCATCTTTGACACATCATAAAACCTTTGTTAGGTTATTTTCTTTCATGTCCT
                                                                                    Section 111
                                                     (8251) 8251     8260      8270      8280      8290      8300      8310          8325
   Mus musculus strain C57BL/6J chromosome 19 NC_000... (7947) GG--CGCATGCCTTTATTCCCAG--CACTTGGGAGGCAGAGGCAAGCAGATTTCTGAGTTCAAGGCCAGCCTGAT
Triticum aestivum cultivar Chinese Spring chromosome 6... (7840) GCACCGCGGGCCACAACCACCTGTCCACCATCGTTCCACAGACCATCA--TGAACGCCTGCACGGTAA---TAAT
                                                                                    Section 112
                                                     (8326) 8326          8340      8350      8360      8370      8380      8390    8400
   Mus musculus strain C57BL/6J chromosome 19 NC_000... (8018) CTACAGAGTGAGTTCCAGGA--CAGCCAGGGCTACACAGAGAAACCCTGTCTTGAAAAACCAAAAACCAAAAACC
Triticum aestivum cultivar Chinese Spring chromosome 6... (7910) AAACAAAATTATTATAAAAATGCATGCATG-CAACGAAGTCATACAAAATTTCGGCATGACCTTCCCTAAAAATA
```

Mus musculus chromosome 19 vs. Triticum aestivum chromosome 6D

Section 113

```
                                                              (8401) 8401     8410      8420      8430      8440      8450      8460           8475
Mus musculus strain C57BL/6J chromosome 19 NC_000...          (8091) AAAAACCAAAAAAAAAAAAAAAAAAAAAAAAAAAAAAAAAAAAGGCTAGATGCAAGCTGAGGGTGTTCTTCACAA
Triticum aestivum cultivar Chinese Spring chromosome 6...     (7984) GGACATATAGAGCTTGCCCGGAATTCACCGAAACGGAAATAAAT-CAACATTTCGGCAAAACATAGTC-----AA
```

Section 114

```
                                                              (8476) 8476          8490      8500      8510      8520      8530      8540      8550
Mus musculus strain C57BL/6J chromosome 19 NC_000...          (8166) CTGGCTAGGCACAGGCGTTAGGGTGATTCCTAGGTGCCTTACCCACAGTTCCTGCCCCTAGGACTGCAGTCCTGC
Triticum aestivum cultivar Chinese Spring chromosome 6...     (8053) CTCACAAG-CACAAT--TTGGCGTCAACTCA---TGCCACACACACAATTTCCATCATATCGCCCG-ATTATGTC
```

Section 115

```
                                                              (8551) 8551     8560      8570      8580      8590      8600      8610           8625
Mus musculus strain C57BL/6J chromosome 19 NC_000...          (8241) ACGTCTGCCCCCAGATTTACAGGCTGGGACTCTCTTTGGTCCCTCCCAGTATGTTCTATTACCCAGGGCTCCAGT
Triticum aestivum cultivar Chinese Spring chromosome 6...     (8121) ATATC-GCACACATATACACATATTTCCATTCTTGCAAAAGCATAAATTTTTAATCACCTATCTAGAGATCGAGC
```

Section 116

```
                                                              (8626) 8626          8640      8650      8660      8670      8680      8690      8700
Mus musculus strain C57BL/6J chromosome 19 NC_000...          (8316) ACAATATCCCATGCTAGCGGGACATTTTGGACCACTGAATGGATGTATTCACAAACCTCCCACCTCCTGTCCCGT
Triticum aestivum cultivar Chinese Spring chromosome 6...     (8195) ACACGGTGAGTTGATGATGT---AGTTAGGAAGACGAAAAG------TGGACAAAGCTCT----TCTTGACAAGG
```

Section 117

```
                                                              (8701) 8701     8710      8720      8730      8740      8750      8760           8775
Mus musculus strain C57BL/6J chromosome 19 NC_000...          (8391) -TCCATCTTCTCATGTCAGCCCTGTTGCCTCTTCCCT----GGTAGTGTGACACACAGGACTGGTCTGT-GGCTT
Triticum aestivum cultivar Chinese Spring chromosome 6...     (8257) CTAGATCTAGTTAGGGGTACATAGGT--CACTTCACTAAATGCTACATCTACCTAGCTAGCTAGTTTGGAGGAGA
```

Section 118

```
                                                              (8776) 8776          8790      8800      8810      8820      8830      8840      8850
Mus musculus strain C57BL/6J chromosome 19 NC_000...          (8460) CCCCATTACATGTTCGCTACAG--GGTACAGTGTATAGCTTCCCAGGGCCAGTGACTTGCTGTCACAGTTG--AA
Triticum aestivum cultivar Chinese Spring chromosome 6...     (8330) CAAGTTCAAATAGTGGAGGGGGAAGGAAAAGAGAAAGGAGATTCATTAATGG-GAGGTGGTGT---AGTTAGCTA
```

Section 119

```
                                                              (8851) 8851     8860      8870      8880      8890      8900      8910           8925
Mus musculus strain C57BL/6J chromosome 19 NC_000...          (8531) GTTTTCATATTGTG-GATTGGTGTGAGCCACAAAAGGTTTCATGAGCC-GAGTGTGATAGGTCTTTCCTTACCCA
Triticum aestivum cultivar Chinese Spring chromosome 6...     (8401) GGAGTAATAAAGAGAGAGAAAGGTAGGTAGAAGAAGGAGTAATGGGGGAGAAGTAGTGAGGAAGAAGCAAAGTGA
```

```
                                                              Section 120
                                                 (8926) 8926          8940      8950      8960      8970      8980      8990      9000
 Mus musculus strain C57BL/6J chromosome 19 NC_000... (8604) GGCAGAG---------GCCCTAGGTCA---CCAGCTTCCACTTCCTGACCATCACTAGGAGAACAGTGCCCTGGG
Triticum aestivum cultivar Chinese Spring chromosome 6... (8476) GGGAGAGAGGAGGTGGGAGCTAGGAGAAAGTGAGGAAGAGAGGATGGGGGAGGGGGAGGGGAAAAGTGGTATATG
                                                              Section 121
                                                 (9001) 9001     9010      9020      9030      9040      9050      9060          9075
 Mus musculus strain C57BL/6J chromosome 19 NC_000... (8667) TAGCTCCTCCCCCTGAGTGACACTGGAGTCTCTCTGCAGCTTCATTTTGCAGATGAGGAAACTGAGGCACCCTAC
Triticum aestivum cultivar Chinese Spring chromosome 6... (8551) TTGCGGCTTAGCAATAGCGCTGGTGGTAAACCGC-GCTACTGC--TATGCTAATAAGATAACAGCAGCGCGCTTC
                                                              Section 122
                                                 (9076) 9076          9090      9100      9110      9120      9130      9140      9150
 Mus musculus strain C57BL/6J chromosome 19 NC_000... (8742) CCTTCCTAGCAACTGGCCTTCCATGAGTAAGGGG-TGTGAGAATTATCTTCCTT--CAGCATCTCTGCTCCCTGC
Triticum aestivum cultivar Chinese Spring chromosome 6... (8623) TAGAACACGCG-CTGCTGCTAAGCGAGGCCCGCCATGTGGCCAGTTTCAAAATTAGCAGTAGCGTCACCCCCAAA
                                                              Section 123
                                                 (9151) 9151     9160      9170      9180      9190      9200      9210          9225
 Mus musculus strain C57BL/6J chromosome 19 NC_000... (8814) CTCATGGCCACTGCACTCATGCCCTGCCCCCAGTAGGCAGGATGCCACCAGGCCCTGC-ACAC---GGCTGCCGA
Triticum aestivum cultivar Chinese Spring chromosome 6... (8697) AAACGCGCTACTACTATTACTTTATTAGTAGAGCGGTTAGCTAGAG-CGTGCTACTGCTAAACCTAGGGTGGCGG
                                                              Section 124
                                                 (9226) 9226          9240      9250      9260      9270      9280      9290      9300
 Mus musculus strain C57BL/6J chromosome 19 NC_000... (8885) GGATCTTTAATA-ACTCGAGT-GCTGTGCATGATGAATGAGGCCCGTGGTTGTGTACATAATGAAGCCCTAATGT
Triticum aestivum cultivar Chinese Spring chromosome 6... (8771) GAATGGTCAATCGACTTTAGTAGCAGCGTCTTTCTACCGAGCCGCGCTACTGCTAAGTTAGT--AGCAGTAGCGC
                                                              Section 125
                                                 (9301) 9301     9310      9320      9330      9340      9350      9360          9375
 Mus musculus strain C57BL/6J chromosome 19 NC_000... (8958) TTTCAAATCCCCATTTAAGGAGCTGATCTCACCCCGTAATGAATGTCAGATCACATCACAGTGATGTCGTCCATC
Triticum aestivum cultivar Chinese Spring chromosome 6... (8844) CTGTCTGTGTCCCGCGCACTAGCGCCTCTTGCT--GTCCCGCGCAGCTGCTAA---------GATTTTGTGTATA
                                                              Section 126
                                                 (9376) 9376          9390      9400      9410      9420      9430      9440      9450
 Mus musculus strain C57BL/6J chromosome 19 NC_000... (9033) CATCTTCTCCCCGGGGCGCCCACCCTCCTCGGCACCTCCAGGAT----GCAGCCTCGGGAGGGTGGGCTCTG-TG
Triticum aestivum cultivar Chinese Spring chromosome 6... (8908) AGGTTTTCCTTAGTAGTGCCTCATCGCATAGGCCCATAGAGAATTTAAGCACACTCAT-AGCGTAAGTACAGATC
```

Mus musculus chromosome 19 vs. Triticum aestivum chromosome 6D

Section 127

		9451 9460 9470 9480 9490 9500 9510 9525
	(9451)	
Mus musculus strain C57BL/6J chromosome 19 NC_000...	(9103)	TCCTT--CCCTGGGCCCT--GTACAGGTCTTGTGTTGAGCAGACATCGAAGGA---GGGCTGTCTGGGAAATGAA
Triticum aestivum cultivar Chinese Spring chromosome 6...	(8982)	GCCTAAGCCACCGACATTACGAAAGTGTCTTTTGTTGCCCAGACATATATTTATCTGACCAGTCAGAGCACTTTT

Section 128

		9526 9540 9550 9560 9570 9580 9590 9600
	(9526)	
Mus musculus strain C57BL/6J chromosome 19 NC_000...	(9171)	GGGCCAGAAGTTGGCTGTTTGGGTTCTAACCGGGCAACATCAGTATCTCTGTTCACAAGTTCTATCCA--GAAAG
Triticum aestivum cultivar Chinese Spring chromosome 6...	(9057)	TCAGCA-ATCTCTCTTTAGTCGGTTGAAAATTTTCAATTT-----TCATTCTCCTCTAGGACTAGCAAATGAAGG

Section 129

		9601 9610 9620 9630 9640 9650 9660 9675
	(9601)	
Mus musculus strain C57BL/6J chromosome 19 NC_000...	(9244)	--------AGGAAGAGAGTGTGGGGCAAGC--CAGAAGAGA------AAGGTCCCAGGACTCAGTGGATGGTCTT
Triticum aestivum cultivar Chinese Spring chromosome 6...	(9126)	TACTTCTAATCAAAAGAAGGTGATTCAACATTCAGAATAGATGCTGCACTTTCACCATCTTCCTTGGAGCATTTT

Section 130

		9676 9690 9700 9710 9720 9730 9740 9750
	(9676)	
Mus musculus strain C57BL/6J chromosome 19 NC_000...	(9303)	AGCCCTCGTG-----------GAGGGGGGAGTCCAAAGGTCAGCAGT-------TCAGGAATCTGCCTCAGCGCC
Triticum aestivum cultivar Chinese Spring chromosome 6...	(9201)	TGACCTAGTATGATTGAACATGTGGCCGGACATGAAAGTTGTCCAGTACCCCTTTCATAAACCTGGAGAACTCCA

Section 131

		9751 9760 9770 9780 9790 9800 9810 9825
	(9751)	
Mus musculus strain C57BL/6J chromosome 19 NC_000...	(9360)	TTAGTCC----AGTGGACTCAGCTCCCCATCTGTTCAGCCAAGCTGTTCTT--CTGAGAAG-GAAATGGACAAGG
Triticum aestivum cultivar Chinese Spring chromosome 6...	(9276)	TTAACCCGAGAAGCTTGCTTAGAATTACCACTGAAAAGAAAGAATGATGTTGTCTGAAAACAGCAAATGTGATAT

Section 132

		9826 9840 9850 9860 9870 9880 9890 9900
	(9826)	
Mus musculus strain C57BL/6J chromosome 19 NC_000...	(9428)	ACCAGTGC-------ACAGGGTCTGGCTGGCAGCTGTATGCTGCACCAAGGTG--TGGCTGCTGTGAGCTGTCCT
Triticum aestivum cultivar Chinese Spring chromosome 6...	(9351)	ACCGGGGCTTTTTTAACAGATTTT---TTGCTCTTTTATTTTTC-CTAAGTTGATTTGTTGATG-GAATAGAAAC

Section 133

		9901 9910 9920 9930 9940 9950 9960 9975
	(9901)	
Mus musculus strain C57BL/6J chromosome 19 NC_000...	(9494)	CAGGTCAGAGTCCATGCAGGACTGAATGTCCAGATGGGGAGAGG---TCCCCTATACCT-------TCTTAGACA
Triticum aestivum cultivar Chinese Spring chromosome 6...	(9421)	GAGAT-ACCCTCAGTGATGAAGTGAAA-TAAATAAGGAGATAGGGGATCCCCTTGTCGTAGGAATCTCGTAGGTT

Mus musculus chromosome 19 vs. Triticum aestivum chromosome 6D

```
                                                                  Section 134
                                            (9976)  9976          9990      10000     10010     10020     10030     10040   10050
Mus musculus strain C57BL/6J chromosome 19 NC_000...  (9559)  GAGA----GTCCCTGCCCTTGCCCCGCCCCCACCCC--AGCCTGTC-GTCCTAGAACAGTGCCGCAGTGCT-CAG
Triticum aestivum cultivar Chinese Spring chromosome 6...  (9494)  CAAATCTTTTCAATAGTTTTCCGTTGAACCGAACCGAAAGCAAGACCGTGTTCACACA-TGCCATAAACCTACCG
                                                                  Section 135
                                            (10051) 10051    10060     10070     10080     10090     10100     10110          10125
Mus musculus strain C57BL/6J chromosome 19 NC_000...  (9626)  GTCTCTGCCCTTG----GCTGTGAGTGTGACAGCGTGATAGACAAAGGAGCAGCCTCTTCCCAAGCCCACACCAA
Triticum aestivum cultivar Chinese Spring chromosome 6...  (9568)  ATCCACTTACATGCAAAGCTGATTTTTTCATTGCTTCTTCTCAAAAATGCCAA--TCTACTCAGTCACATGACTT
                                                                  Section 136
                                            (10126) 10126         10140     10150     10160     10170     10180     10190   10200
Mus musculus strain C57BL/6J chromosome 19 NC_000...  (9697)  GCAACAAGGGCTTCCTGACAGCCAAGGAAACAGGTACAGAGACTGCACCT-TGGAGACAAGGTGGAAGAGCCCAG
Triticum aestivum cultivar Chinese Spring chromosome 6...  (9641)  G--ATAAGGTCATGCTTAGAGGCACAAAACTTCCTTGAATTATTCGACCCCTACTGCAGAGCATGAATACATTCC
                                                                  Section 137
                                            (10201) 10201    10210     10220     10230     10240     10250     10260          10275
Mus musculus strain C57BL/6J chromosome 19 NC_000...  (9771)  AAAGGGGAACTTGTCTTGGCAAGCTCCTTGCCTGTGTCCCCAAGGCCAGGTGGGCTGTCACTCTACAAACTGGAT
Triticum aestivum cultivar Chinese Spring chromosome 6...  (9714)  AAAGTGATAATTGCATTATCAGTAATCATCAGCGCAGAATGAAGGCACTTTGTGTTGGGGA---AAAAAGGAATT
                                                                  Section 138
                                            (10276) 10276         10290     10300     10310     10320     10330     10340   10350
Mus musculus strain C57BL/6J chromosome 19 NC_000...  (9846)  CCCTATTCCAGGATGGGCAGGGCAGAGGTGGGAGCTACCCAGAATCCTCTCTTGCTCATGGCTGTGCCTCTCTGA
Triticum aestivum cultivar Chinese Spring chromosome 6...  (9786)  ATAAAAACAAAGGCTACCTAGGTATATGTGGGAGTTACTAA-AATATACTGCTATGTAGGTATATGTCGCA-TGA
                                                                  Section 139
                                            (10351) 10351    10360     10370     10380     10390     10400     10410          10425
Mus musculus strain C57BL/6J chromosome 19 NC_000...  (9921)  ACCT-----TTGC--CTCCACTTCTTGTGAGACAGGGCCT-AGCAGTAGAGA----CCTTGGCTTCTGCTCCACT
Triticum aestivum cultivar Chinese Spring chromosome 6...  (9859)  ACATCCAGGTTGTTAATATATTTTATGTATTCAAGTGCATTATCAGTAATCATCCGCCCCGGAATAAAAGC-ACT
                                                                  Section 140
                                            (10426) 10426         10440     10450     10460     10470     10480       10494
Mus musculus strain C57BL/6J chromosome 19 NC_000...  (9984)  TCCAGCTGTGAGATCTTG---------------------------------------------------
Triticum aestivum cultivar Chinese Spring chromosome 6...  (9933)  TTGCGTTGGGGAAAGAAGAAATTATAACTACAAAGTCTATATAGGTATATGTGATGTAGGTATATGTCA
```
