# Supplementary material for: Patch type nucleotide sequence identities between genomes from many different species facilitate illegitimate recombination
Source: Sci Rep. 2026 Mar 30;16:10524. doi: 10.1038/s41598-026-44124-0 (PMC13035915; doi:10.1038/s41598-026-44124-0)
Supplement: Supplementary file 8 — Supplementary Material 8 [file 41598_2026_44124_MOESM8_ESM.pdf]

Mycobacterium tuberculosis vs. Oryza sativa chromosome 8

Section 1

		1 — 76
	(1)	`1 10 20 30 40 50 60 76`
Mycobacterium tuberculosis DNA AP018036.1 region fr...	(1)	`------------------------------------------------------------ACCCGCGGACACGCCC`
Oryza sativa chromosome 8 NC_029263.1 region fro...	(1)	`GGAGAAAGAGAGAAATTAAAGTATTTCCATTTTTCTCTTTGAGAGAGAGAGAGAGAGTGATGCTGATGAGAGTTGA`

Section 2

		77 — 152
	(77)	`77 90 100 110 120 130 140 152`
Mycobacterium tuberculosis DNA AP018036.1 region fr...	(17)	`GACAAACTGGCCGTTTTTGCGGTCGCGC---CGCACTGCAGCGCCGCCGATACCGGGTTGTTGGCTAGCACCGTGG`
Oryza sativa chromosome 8 NC_029263.1 region fro...	(77)	`GACTTCCTTCATATGTAATGAGTAGCTCTAGCTTGTTTTAACTTTTAAGGTTTAGGGTCACTGGCCGGCACACTGC`

Section 3

		153 — 228
	(153)	`153 160 170 180 190 200 210 228`
Mycobacterium tuberculosis DNA AP018036.1 region fr...	(90)	`-TCC--CCCGCGCATGATGGGT-CTGGGTGCTGTCGCTCGCCT----------GCGGGAACAGCAGTGCCTACGCT`
Oryza sativa chromosome 8 NC_029263.1 region fro...	(153)	`CTCCATCCATAGGAAAAAGTTTGCTCTCTGCTAGTACTCCTCTAAACTATTTCGACGGAACA-CATAATTAACCCT`

Section 4

		229 — 304
	(229)	`229 240 250 260 270 280 290 304`
Mycobacterium tuberculosis DNA AP018036.1 region fr...	(152)	`GGCGTTCGTTG-TCTCAAGATCTGCC-CTCGCACTCGAAAGGCTCGCATGACC--GCCCCG-CC--T-----GTCC`
Oryza sativa chromosome 8 NC_029263.1 region fro...	(228)	`GACCTTTTTGGCTCACTTGACCAAACACACTGTTTAAAACGAACCACTTTACTTTGTACCGACATTTCTTTTGTTT`

Section 5

		305 — 380
	(305)	`305 310 320 330 340 350 360 370 380`
Mycobacterium tuberculosis DNA AP018036.1 region fr...	(216)	`ATGACCGCG--CACACCACCC--CGTTCGCG-AC--GTGATCGTTATCGGCTCCGGTCCCGCGG--GGTACACTGC`
Oryza sativa chromosome 8 NC_029263.1 region fro...	(304)	`CTCACAATTAACACACGACCCATAGTTCTATCACTAGTGAGTGAAGTTGGGTAATTTTTTATTATAGTTTAACGGC`

Section 6

		381 — 456
	(381)	`381 390 400 410 420 430 440 456`
Mycobacterium tuberculosis DNA AP018036.1 region fr...	(283)	`GG---CGCTCTACGCCGCCCGTGCCCAGCTGGCGCCGCTGGTCTTCGAGGG-CACGTCTTTCGGCGGCGCGCTGAT`
Oryza sativa chromosome 8 NC_029263.1 region fro...	(380)	`TAAAACACTTTAACGTAATAGTTTCAAGTACTAGAATAAACTTTTCGGTTCACAAATATTATTTACTAGTATTTAT`

Section 7

		457 — 532
	(457)	`457 470 480 490 500 510 520 532`
Mycobacterium tuberculosis DNA AP018036.1 region fr...	(355)	`GACCACCA-----CCGACGTGGAGAACTACCCGGGATTTCGCAACGGCATCACCGGTCCAGAGTTG--ATGGATGA`
Oryza sativa chromosome 8 NC_029263.1 region fro...	(456)	`TTCCTTAAGAAATTTTACGACTATTAATTAAGGAGAAACCGAGAGGTAGTAGGAGATATAAAATTTACATTTAAAA`

Mycobacterium tuberculosis vs. Oryza sativa chromosome 8

Section 8

```
                                                    (533) 533    540            550            560            570            580            590                608
Mycobacterium tuberculosis DNA AP018036.1 region fr...  (424) GATGCGGGAACAGGCGCTGCGATTCGGCGCGGACCTGCGTATGGAAGACGTCGAGTCGGTATCACTTCACGGGCCG
Oryza sativa chromosome 8  NC_029263.1 region fro...   (532) GATTACTCTAAAGGATGTTAACATAGTCAAG-ACTAGTTCATCAAC----TCAAAACAATATCCCATCTCTCACCT
```

Section 9

```
                                                    (609) 609        620            630            640            650            660            670          684
Mycobacterium tuberculosis DNA AP018036.1 region fr...  (500) CTGAAATCGGTCGTCACCGCC--GACGGACAGACCCACCGGGCCCGAGCCGT-GATCCTGGC--AATGGGCGCAGC
Oryza sativa chromosome 8  NC_029263.1 region fro...   (603) CTCCTCTCCATCCCCTCCACCATGGCAGGCGCTACCTCCATGTCAACATTGTCGTTGCTGACTCAAAGTCAAGAGC
```

Section 10

```
                                                    (685) 685  690           700            710            720            730            740            750      760
Mycobacterium tuberculosis DNA AP018036.1 region fr...  (571) --GGCACGCTATCTGCAG-GTGCCCGGCGAACAGGAATTGCTCGGGCGCGGGGTGAGCTC-GTGC---GCCACCTG
Oryza sativa chromosome 8  NC_029263.1 region fro...   (679) TAGCTAGCCTTGCTGCGGCGCACCACTCGCTCAAGCTTCGCACTTATGAGGAGAAAGATTAGAGCAGTGAGCCTTG
```

Section 11

```
                                                    (761) 761     770            780            790            800            810            820          836
Mycobacterium tuberculosis DNA AP018036.1 region fr...  (640) CGACGGATTCTTCTTCCG-CGATCAGGACATCGCC--GTCATCGGCGGCGGTGACTCGGCAA----TGGAGGAAGC
Oryza sativa chromosome 8  NC_029263.1 region fro...   (755) GAATTACTAGATCTTGCGGCGAT--GGACCTCATCTAGTAATGGCTCGTCTCGAGACTGTTAGATCTGGCACTAGC
```

Section 12

```
                                                    (837) 837            850            860            870            880            890            900          912
Mycobacterium tuberculosis DNA AP018036.1 region fr...  (709) TACCTTCCTGAC---CCGATTCGCTCGCAGTGTGACGCTGGTGCATCGCCGCGACGAGTTCCGGGCTTCCAAAATC
Oryza sativa chromosome 8  NC_029263.1 region fro...   (829) TGGCCTCAGGACAGGCGGCGGCGGCAGCAGTGGCTCACAATGCCAGCGCTCC-ACCTCCTCGACGATAGCGAGAGC
```

Section 13

```
                                                    (913) 913    920            930            940            950            960            970                988
Mycobacterium tuberculosis DNA AP018036.1 region fr...  (782) ATGCTCGATCGCGCCCGCAACAACGACAAGATACGGTTCCTCACCAACCACACCGTGG-TCGCGGTGGACGGGAC
Oryza sativa chromosome 8  NC_029263.1 region fro...   (904) TTCCACT-TGGCAACCACAACGACGACCA--TAGGGTAGGTCTGCTTCT---CGGTGGCTCAAGGGCGTTGACGGT
```

Section 14

```
                                                    (989) 989        1000           1010           1020           1030           1040           1050        1064
Mycobacterium tuberculosis DNA AP018036.1 region fr...  (857) ACCACAGTGACCGGCTTGCGGGTACGC--GACACC-AACACCGGTGCCGAAACCACCCTGCCGGTAACCGGTGTTT
Oryza sativa chromosome 8  NC_029263.1 region fro...   (974) GCTGCTCTACTAGCCTCCAAGGTCCGGCTGGCACCTAACTACTGTTCGCTCGCTCACTTGCTACTATGATTTTTTT
```

Section 15

	(1065)	1065 1070 1080 1090 1100 1110 1120 1130 1140
Mycobacterium tuberculosis DNA AP018036.1 region fr...	(930)	TCGTCGCGATCGGCCACGAGCCGCGGTCGGGCTTG-GTGCGCGAGGCCATCGACGTCGACCCGGACGG--CTACGT
Oryza sativa chromosome 8 NC_029263.1 region fro...	(1050)	TGTGAAGTTTGGGGAATTTTGTGAATTCGTGTTTGTGTCTACAATG--ATACACA-CGATGTGAATGGATCTAAAT

Section 16

	(1141)	1141 1150 1160 1170 1180 1190 1200 1216
Mycobacterium tuberculosis DNA AP018036.1 region fr...	(1003)	GTTGG---TGCAGGGGCGTACCACCAGCACCTCACTGCCGGGCGTGTTCGCTGCCGGCGACCTGGTGGATCGCACC
Oryza sativa chromosome 8 NC_029263.1 region fro...	(1123)	TTTTGAATTGCAGGCTGATTCGATCTGAATTTTCTTTTC-------TACACTTAGGCCGCTTTAGTGGACCGCTTG

Section 17

	(1217)	1217 1230 1240 1250 1260 1270 1280 1292
Mycobacterium tuberculosis DNA AP018036.1 region fr...	(1076)	TATCGCCAGGCGGTTACCGCAGCGGGCAGTGGCTGCGCCGCGGCTATCGACGCCGAGCGCTGGCTCGCCGAGCACG
Oryza sativa chromosome 8 NC_029263.1 region fro...	(1192)	TTAA---AATCAATTTTTATGTTGGGTATCTAACGTGAT-CATATATGAAAATTGATTTTTTATATGC--AGTTTT

Section 18

	(1293)	1293 1300 1310 1320 1330 1340 1350 1368
Mycobacterium tuberculosis DNA AP018036.1 region fr...	(1152)	CAGCAACCGGAGAAGCT-GACAGTAC-CGACGCATTGATAGGAGCACAACGATGACC-GATTCCGAGAAG--TCCG
Oryza sativa chromosome 8 NC_029263.1 region fro...	(1262)	GAACAACTAACGAAAATTGACAATTTTCCATGCCTTC-TGTTAGAGGACTGTTCACCCGCCTCCCAAAATCATTTT

Section 19

	(1369)	1369 1380 1390 1400 1410 1420 1430 1444
Mycobacterium tuberculosis DNA AP018036.1 region fr...	(1223)	CCACCATCAAAGTTACCGACGCATCCTTTGCCACCGACGTGCTATCCAGC---AACAAGCCTGTGCTG-GTTGACT
Oryza sativa chromosome 8 NC_029263.1 region fro...	(1337)	TTACCATCT--GGAAAAGACTTTTTCATAGC-AGTGATATAATTTGCATCCTTAACACATATGTGAAATGAGTAGT

Section 20

	(1445)	1445 1450 1460 1470 1480 1490 1500 1510 1520
Mycobacterium tuberculosis DNA AP018036.1 region fr...	(1295)	TTTGGGCGACATG---GTGTGGACCTTGCAAGATGG---TAGCGCCCGTTCTCGAGGAAATCGCCACCGAGCGCGC
Oryza sativa chromosome 8 NC_029263.1 region fro...	(1410)	TATTTGCCACATTCACCTATTTACCTAGATATATATCACTAATACAATTTGTCTCTACAATTTCCCCCTAAAGTGG

Section 21

	(1521)	1521 1530 1540 1550 1560 1570 1580 1596
Mycobacterium tuberculosis DNA AP018036.1 region fr...	(1365)	AACAGACCTCACCGTCGCCAAGCTCGACGTGGACACCAACCCGGAGACCGCCCGCAACTTCCAG-GTCGTCTCGAT
Oryza sativa chromosome 8 NC_029263.1 region fro...	(1486)	CAGTGA----AGAATAGGAGATCGTTAATTAC-CAGCGTTACGGT-ACAGAGATTAATTAAGAGAGTTGTAGCTAA

Mycobacterium tuberculosis vs. Oryza sativa chromosome 8

Section 22

	(1597)	1597 1610 1620 1630 1640 1650 1660 1672
Mycobacterium tuberculosis DNA AP018036.1 region fr...	(1440)	CCCTACCCTGATCTTGTTCAAGGACGGCCA--GCCGGTGAAACGAATCGTTGGCGCCAAGGGTAAGGCTGCGTTGC
Oryza sativa chromosome 8 NC_029263.1 region fro...	(1556)	---TAGCCC-ATCCA--TCACGTACGGCTAAAGCCAGAGAATCCGGTGGATATGGCCACCGGAAATACATATATAC

Section 23

	(1673)	1673 1680 1690 1700 1710 1720 1730 1748
Mycobacterium tuberculosis DNA AP018036.1 region fr...	(1514)	TGCGCGAGCTCTCAG-ACGTGGTTCCCAACCTCAACTAGCCCCCGCGGTTAGCCTGGGGTTTTCCCGAAATCGGCA
Oryza sativa chromosome 8 NC_029263.1 region fro...	(1626)	GGCGTACTATGTGCGTACATCGT-CAGTACGT--ACGGGCCATCGTACTT-GATTGGCATTATTCGT---TTTGTA

Section 24

	(1749)	1749 1760 1770 1780 1790 1800 1810 1824
Mycobacterium tuberculosis DNA AP018036.1 region fr...	(1589)	AGGATCTGCGACAATACCGGTTGGCTGGTCCGCATTGTCAACGATGTGAGCTAATCCCGGAGGGCCCTTGGTATGC
Oryza sativa chromosome 8 NC_029263.1 region fro...	(1695)	TTAAGTTGCTA-ATTGCACGTACAATTA----CAGTGTATAGTATCTTAGCTTGTACGGTGTAAAACATTATATAT

Section 25

	(1825)	1825 1830 1840 1850 1860 1870 1880 1890 1900
Mycobacterium tuberculosis DNA AP018036.1 region fr...	(1665)	CGAGTCCGCGCCGCGAAGACGGCGATGCGCTGCGCTGTGGCGACCGCAGTGCGGCCGTCACCGAGA-TCCGGGCTG
Oryza sativa chromosome 8 NC_029263.1 region fro...	(1766)	ACGGTCCTTTTTTCCTTTCCATAAGAGTGTGTGTATATATCCCACTCGATCTGATCTAAAATGGTACTTTCATCTG

Section 26

	(1901)	1901 1910 1920 1930 1940 1950 1960 1976
Mycobacterium tuberculosis DNA AP018036.1 region fr...	(1740)	CGCTGACCGCGTTAGGGATGCTGGATCATCAGGAAGA---AGACCTGACGACGGGCCGTAACGTCGCCCTTGAGTT
Oryza sativa chromosome 8 NC_029263.1 region fro...	(1842)	TGCT--CGGTGCTGGTAATCCTACAT-ATCTATAAAACTCATCCCTG-CTAAGTGAGTTAATGTTAATTTTCTCTT

Section 27

	(1977)	1977 1990 2000 2010 2020 2030 2040 2052
Mycobacterium tuberculosis DNA AP018036.1 region fr...	(1813)	GTTCGACGCGCAGCTC-GAC--CAGGCGGTCCGTGCCTTCCAA-----CAGCATCGCGGCCTGCTGGTGGACGGCA
Oryza sativa chromosome 8 NC_029263.1 region fro...	(1914)	CTCCTTAAGACATATATGACCTCAATTTTTTTTAGCCTTTGTATGATTCACCAATGGTGCAAATTATATCCCTTCT

Section 28

	(2053)	2053 2060 2070 2080 2090 2100 2110 2128
Mycobacterium tuberculosis DNA AP018036.1 region fr...	(1881)	TCGTCGGTGAGGCCACCTA----CCGCGCGTTGAAAGAAGCCTCCTACCGGCTCGG--GGCCCGCACGCTGTACCA
Oryza sativa chromosome 8 NC_029263.1 region fro...	(1990)	CTCTAAAAGCCACAACATATAACCCAATCATTTATAACATTTGGATAATCGATCGATCGAGATACAAAATGTGTAA

Mycobacterium tuberculosis vs. Oryza sativa chromosome 8

Section 29

```
                                                          (2129) 2129       2140           2150           2160           2170           2180           2190             2204
Mycobacterium tuberculosis DNA AP018036.1 region fr...  (1951) CCAATTCGGCGCCCCGCTCTACGGGGACGACGTCGCTACACTGCAGGC-CCGGCTGCAGGATCTTGGTTTCTACAC
  Oryza sativa chromosome 8  NC_029263.1 region fro...  (2066) CAAACAAAAAAGAAAATCATATAGTAAATAAGAAAGAAAATTTTACTATCCATATCTACTAT-TTATCACATAATT
```

Section 30

```
                                                          (2205) 2205 2210           2220           2230           2240           2250           2260           2270   2280
Mycobacterium tuberculosis DNA AP018036.1 region fr...  (2026) CGGGCTGGTCGACGGTCATTTCGGGT---TGCAGACCCACAATGCGTTGATGTCCTATCAGCGTGAGT-ACGGACT
  Oryza sativa chromosome 8  NC_029263.1 region fro...  (2141) CTCCCCCGGCAACG--CATGTGGAACCCATCTAGTTAATCAATATGATCAAGGCCATTGTGAGTCCGTGACTGTGG
```

Section 31

```
                                                          (2281) 2281     2290           2300           2310           2320           2330           2340             2356
Mycobacterium tuberculosis DNA AP018036.1 region fr...  (2098) TGCCGCAGACGGTATCTGCGGCCCAGAAACGTTGCGCTCCTTGTACTTTCTAAGTTCGCGAGTCAGCGGTGGCTCG
  Oryza sativa chromosome 8  NC_029263.1 region fro...  (2215) TGCCACTCACACTCTCAAAGGC--AGTAGAG--GAGGTCACTGAATTCTCTTT-TTAATTATTAGACTATTAATTA
```

Section 32

```
                                                          (2357) 2357          2370           2380           2390           2400           2410           2420       2432
Mycobacterium tuberculosis DNA AP018036.1 region fr...  (2174) CCACATGCGATTCGCGAAGAAGAGCTGGTCCGCAGCTCGGGGCCGAAGCTGTCTGGCAAACGGATCATCATTGA-T
  Oryza sativa chromosome 8  NC_029263.1 region fro...  (2286) TATCAGTTGCTTCTAAATAATTATTT--TTCAATGATACGC----AAGCTGACATGCACACATACCACTACAGACT
```

Section 33

```
                                                          (2433) 2433   2440           2450           2460           2470           2480           2490             2508
Mycobacterium tuberculosis DNA AP018036.1 region fr...  (2249) CCCGGTCGCGGCGGCGTGGACCACGGACTTATCGCGCAAGGTCCG-GCTGGGCCCATCAGCGAAGCAGACTTGTTG
  Oryza sativa chromosome 8  NC_029263.1 region fro...  (2356) ATACATGTAAACAAAGTGT------GTCTTCTCGTATATTATCAAAGATATGGACACCAATGTTAAATTTTTGTCG
```

Section 34

```
                                                          (2509) 2509       2520           2530           2540           2550           2560           2570             2584
Mycobacterium tuberculosis DNA AP018036.1 region fr...  (2324) ----TGGGACTTGGCAA-GTC--GGCTCGAAGGACGGATGGCAGCTATCGGTATGGAGACCC---ACCTGTCCCGT
  Oryza sativa chromosome 8  NC_029263.1 region fro...  (2426) CCATTAGACCCTATCAATGTCTTGTTTGAAATGAAGAAATTTTACAAAAAATTTAGAGAAAATGGACCAATTCCAT
```

Section 35

```
                                                          (2585) 2585 2590           2600           2610           2620           2630           2640           2650   2660
Mycobacterium tuberculosis DNA AP018036.1 region fr...  (2390) CCGACCAACCGTAGTCCGTCCGACG-CAGAGCGTGCCGCCACCGCCAACGCCGTTGGCGCAGACCTGATGATCAGC
  Oryza sativa chromosome 8  NC_029263.1 region fro...  (2502) TCAAATGCTGTAAAATTTCTAAGTTCCGAAGGAACCGTAAAATGCATCTGCATATGTGTAA---TTATGAACAAT
```

Mycobacterium tuberculosis vs. Oryza sativa chromosome 8

Section 36
```
                                                      (2661) 2661     2670           2680           2690           2700           2710           2720                2736
Mycobacterium tuberculosis DNA AP018036.1 region fr... (2465) CTGCGCTGCGAGACCAGACC--AGTCTCGCGGCCAACGGCGTGGCTTCCTTTCACTTCGGCAACTCGCACGGCTC
 Oryza sativa chromosome 8  NC_029263.1 region fro... (2575) -TGTATTTGGATACCGTGAGTGGAGTCATGCGCCCA------TGTCTAACCATCAC-----------GCCTGTTGT
```

Section 37
```
                                                      (2737) 2737               2750           2760           2770           2780           2790           2800           2812
Mycobacterium tuberculosis DNA AP018036.1 region fr... (2539) GGTGTCTACCATCGGCCGCAATCTTGCCGATTTCATTCAACGAGAAGTGGTGGCGCGCACCGGTTTACGGGATT-G
 Oryza sativa chromosome 8  NC_029263.1 region fro... (2633) GTTTTCCTACTTTTATATAAATTTTGCCA--TTCAGATATTTAAAAATAGAGTT------CTTTTTAAGGAATTTG
```

Section 38
```
                                                      (2813) 2813   2820           2830           2840           2850           2860           2870                2888
Mycobacterium tuberculosis DNA AP018036.1 region fr... (2614) CCGTGTGCATGGTCGAACGTGGGATCTGTTGCGGCTGACCAGGATGCCGACC--GTTCAGGTCGATATCG----GC
 Oryza sativa chromosome 8  NC_029263.1 region fro... (2701) CCAAATTCAAAGAACATTTCAAGGTATGTTT-GTTTGAGGTAAGTTACAACCAATTTAAATTTTAGAAGTTAATTC
```

Section 39
```
                                                      (2889) 2889             2900           2910           2920           2930           2940           2950           2964
Mycobacterium tuberculosis DNA AP018036.1 region fr... (2684) TACATCACCAACCCCCACGATCGTGGGATGCTGGTCTCAACGCAGACGCGCGATGCCATCGCCGAAGGCATTCTCG
 Oryza sativa chromosome 8  NC_029263.1 region fro... (2776) TAAATTTTAAATCAACAT-AGCTTAGGAG--TAGTTTTGTTTTTTTCCCCTTTTTCTATTT--GTAGTTTTTTAAG
```

Section 40
```
                                                      (2965) 2965 2970           2980           2990           3000           3010           3020           3030      3040
Mycobacterium tuberculosis DNA AP018036.1 region fr... (2760) CCGCGG-TCAAACGGCTGTATCTGTTAGGCAAGAACGATCGGCCCACCGGCACATTCACTTTCGCCGAGTTGCTGG
 Oryza sativa chromosome 8  NC_029263.1 region fro... (2847) AGGAACATGCAGCGTGGGTATCCTCTAAATAAAAAAATAATTTCGAGGATGC--TCACGCTAGCTT-GTTGCTTT
```

Section 41
```
                                                      (3041) 3041     3050           3060           3070           3080           3090           3100                3116
Mycobacterium tuberculosis DNA AP018036.1 region fr... (2835) CCCACGAACTGTCTGTCGAGC-GAGCGGGTAGACTCGG-CGGTTCTTAAGCCCAG---TGGCCGCGTGGGGTT-TA
 Oryza sativa chromosome 8  NC_029263.1 region fro... (2920) TAAAAATTTTCAAATAGAAGCAGAGCTAAAATAATAGAACTCATCCTAATCTATGAAATGGATATGCCAGGCCCTA
```

Section 42
```
                                                      (3117) 3117               3130           3140           3150           3160           3170           3180           3192
Mycobacterium tuberculosis DNA AP018036.1 region fr... (2905) CGACGTGTTGCCGGCCGTCGACCCCGCTGCTATCGGCTCTTGCAGTCGAGCATTCTCCAGCAAGCGTTCAAGAGCG
 Oryza sativa chromosome 8  NC_029263.1 region fro... (2996) ACCTTTATTGAAGATCAGAGGCCG-GCTGAAATCCAACAATGCTG-CTTCCATACATTAGAA----TTAAAAATTA
```

Section 43

```
(3193) 3193 3200 3210 3220 3230 3240 3250 3268
Mycobacterium tuberculosis DNA AP018036.1 region fr... (2981) GCCTCGACTTCGGCTTTCCACCCCAGCCCTTTG---TCCAGTTCGAGGCGTAGCCTCGGAAAGTACGGGTGCGGTG
Oryza sativa chromosome 8  NC_029263.1 region fro... (3066) GGTATGA--TCA-CAAAGCACCTTTTTTCTTTAAACTAAAGAGAGATTCGATTCCTCTTGAA-TCGGGTAATTTT
```

Section 44

```
(3269) 3269 3280 3290 3300 3310 3320 3330 3344
Mycobacterium tuberculosis DNA AP018036.1 region fr... (3054) CCACCACGACGAAACCCACGTCCATCAAGAAGTTCGCGTCGATGATGCAGTGTTCGACACAGCAGTCGCCGAGGGC
Oryza sativa chromosome 8  NC_029263.1 region fro... (3138) AAAATGCTTCTTTCCAAATATAATTTATGGAGACCGAGTATATT------TGTTTTTCAATG--GTTACAGATGCT
```

Section 45

```
(3345) 3345 3350 3360 3370 3380 3390 3400 3410 3420
Mycobacterium tuberculosis DNA AP018036.1 region fr... (3130) CTCCAACACCGGCCGC-ACATCAGGTGTTACCGC---GCCCGGGTTTTGCAA-ATCGGTGGCCGC-TGGTGTCCGG
Oryza sativa chromosome 8  NC_029263.1 region fro... (3206) CTGCTTAACCAACCCATATATAATGATTAATCGGTTTGTTCTCTTTTGACAAGATAATTTACCGTGTGAACAAAAG
```

Section 46

```
(3421) 3421 3430 3440 3450 3460 3470 3480 3496
Mycobacterium tuberculosis DNA AP018036.1 region fr... (3200) CC---GAAAGCTTCCAGCGCC---CGGACGCCGCGCCG-AACCAACTCTTCAATCACCCGGGCAATCAGACTGTGC
Oryza sativa chromosome 8  NC_029263.1 region fro... (3282) TCAATGAGAGCATCAAGTGCAAATTGATCGATTCATCGCAACTAATTTTTGTAGCGTACAGAAAGTTATAC-GTAC
```

Section 47

```
(3497) 3497 3510 3520 3530 3540 3550 3560 3572
Mycobacterium tuberculosis DNA AP018036.1 region fr... (3269) GGTAAGTCGTCATCTGCTTGCCCGCGCTCGATGCCCAT-CGACGTAAGCAGCACCGCGTCCGCCGACACCGG-CGC
Oryza sativa chromosome 8  NC_029263.1 region fro... (3357) AAAAAG--GTACAGCGAACAAACTAACACG-TACGTATACGAAGTACAAATTAAAACGAATTCCGACAGTGTACTT
```

Section 48

```
(3573) 3573 3580 3590 3600 3610 3620 3630 3648
Mycobacterium tuberculosis DNA AP018036.1 region fr... (3343) GGTAGGAAACCGCTGGGCTCGCGGCACCGCACTGGGCGGAGCGTAGAGCACATACCCGAGGCAGGGTGGTTCGGCG
Oryza sativa chromosome 8  NC_029263.1 region fro... (3430) GGTGAAATTGAATTGGGAGAGTTATTTTAAACAGATTCGAGCG---AGAAAATATTGGA----------TTGCTCG
```

Section 49

```
(3649) 3649 3660 3670 3680 3690 3700 3710 3724
Mycobacterium tuberculosis DNA AP018036.1 region fr... (3419) TGGCTGCGCTCATCCGGGACTGCCGTTGCGACCTGACCGCACGAACCCCACTCCAGCATCACCATCGACAACCAGG
Oryza sativa chromosome 8  NC_029263.1 region fro... (3493) TAACCG---TAATCACGTACAATTGTAGCCTCGTA--CATACGTACGTTAATTAACTGGTGCTTTGGA-ATCCTCT
```

Mycobacterium tuberculosis vs. Oryza sativa chromosome 8

Section 50

```
(3725) 3725 3730 3740 3750 3760 3770 3780 3790 3800
Mycobacterium tuberculosis DNA AP018036.1 region fr... (3495) CTTCCTTTTCGAATTCGGGGTCGGCGAGGTGGTCGTCTTTGCCGAGAATCGCGGGTCGACCTCCCAGAAAACGCA
Oryza sativa chromosome 8  NC_029263.1 region fro... (3563) CGTTTGTTGCCAACC---GGCCGGACTCTTGATA--CTTTACATATGTACTCCG--TCCGTTTCCTCAATATATTA
```

Section 51

```
(3801) 3801 3810 3820 3830 3840 3850 3860 3876
Mycobacterium tuberculosis DNA AP018036.1 region fr... (3571) GCGTCGCGCATGCTTGGGGAGCTGCTCGAAGGCTTCGAGTCGTAACGCTGTGATACGAGCGGACACTAGTCTCCTG
Oryza sativa chromosome 8  NC_029263.1 region fro... (3632) TTTATTATCGTTCTAGTATATCTTACACTGAACTAGAAGATGTGATAAATT-ATATTAGT--ACCCTTATTAATTA
```

Section 52

```
(3877) 3877 3890 3900 3910 3920 3930 3940 3952
Mycobacterium tuberculosis DNA AP018036.1 region fr... (3647) GCC--TCCGTGCGGCATTGCAACCGATGGCCCTACACCTCCGCGGGCCAATGTGCACCAGCAACCCTTCTAGAATA
Oryza sativa chromosome 8  NC_029263.1 region fro... (3705) GTCCATCCATCC--CATTTTAAGTGCAG-CCATACTTTTCCGTACTCAACTTTG----ATCGTCCGTCTTATTTGA
```

Section 53

```
(3953) 3953 3960 3970 3980 3990 4000 4010 4028
Mycobacterium tuberculosis DNA AP018036.1 region fr... (3721) AGAGAGTCGATCGCTATCGGGCCAGTATTCG-CGATGCCACTCCAGCCGACTTGCACCGCATCGTGTCCGGCCGGT
Oryza sativa chromosome 8  NC_029263.1 region fro... (3774) AAAAATTTGAAAC-ATAAGTCACACATAAGTCCGTCTTATTTGAAAAAATTGAAAACATAAGTCACACATAAAG
```

Section 54

```
(4029) 4029 4040 4050 4060 4070 4080 4090 4104
Mycobacterium tuberculosis DNA AP018036.1 region fr... (3796) GACAATTGTCCGGTCCATTGCCCGTC-CAATCTCGAATCCGCTTGCCGCACACCGCGTCTCCGTTGATTCCCGCT
Oryza sativa chromosome 8  NC_029263.1 region fro... (3849) TACTATT-TATGTTTTATCATCTCATAACAACAAAAAAAATACTAATTACAAAAAAT-TCAAATAAGACGGACG
```

Section 55

```
(4105) 4105 4110 4120 4130 4140 4150 4160 4170 4180
Mycobacterium tuberculosis DNA AP018036.1 region fr... (3871) CCCCGCAGCGGGTTGGCTTAGGCGCCGGAACCGGCGCGTTGTCACAGTGAC-GTAATTACAGAGCGTCCCTGTGCA
Oryza sativa chromosome 8  NC_029263.1 region fro... (3923) ATCAAAATTTGGCACGGAAACTCATGGTTGCACTTAAAATGGGACGGAGGGAGTATATACTAATTATCTTTATGAA
```

Section 56

```
(4181) 4181 4190 4200 4210 4220 4230 4240 4256
Mycobacterium tuberculosis DNA AP018036.1 region fr... (3946) G---GCCTTTA---TCTCGGCCATCAGTGGTCATCAAACCGACTATGCGCGCTAAATCATCGACCGAGCCGAACTC
Oryza sativa chromosome 8  NC_029263.1 region fro... (3999) ATCAGCATTAAATTTCTTTGTAATAATACTATTAGTAATTTAGTATG---GTTGTATGGATGAAGGGTAGGAAGAA
```

Mycobacterium tuberculosis vs. Oryza sativa chromosome 8

```
                                                                                                                          Section 57
                                                   (4257) 4257         4270           4280           4290           4300           4310           4320       4332
Mycobacterium tuberculosis DNA AP018036.1 region fr... (4016) CACCACAA-TCTTACCCTTGCG-TTTGCCCAGACTGACGGTCACCCGCGTGTCAAAGGTGGTCGATAGACGCTCAG
  Oryza sativa chromosome 8  NC_029263.1 region fro... (4072) AAAGACTTCTCTAAATGAAGTGCTTTGCTTAAATTAAATAT--------TTTGACATATATTTTAAATGCTCT-AG
                                                                                                                          Section 58
                                                   (4333) 4333    4340           4350           4360           4370           4380           4390           4408
Mycobacterium tuberculosis DNA AP018036.1 region fr... (4090) CAACATCTTGGAGGCCAGGCATCTGAATCGGCTTACGCCGCGGCGGCGCGGGTG---TAGTCGCGTCGCTGTGATG
  Oryza sativa chromosome 8  NC_029263.1 region fro... (4139) TACAATA---------AGTAATTTGAA----CTGAAGGAAGGGAGTACCTTTTAACTTAATAGTGTCTCAGATGCT
                                                                                                                          Section 59
                                                   (4409) 4409        4420           4430           4440           4450           4460           4470       4484
Mycobacterium tuberculosis DNA AP018036.1 region fr... (4163) GGCTTGGCGATTGGCCTCGTGATTGGCCAG-CGTGACCGTCTCCTCGGTGGCTCGCACCGACAGGCCCTCCGCGAC
  Oryza sativa chromosome 8  NC_029263.1 region fro... (4202) TGTGTGAATATTTTTTTATCTATTTATAAGTCGAAAGCAT-------GTGTAT------GAAATGGCAAAAAATAA
                                                                                                                          Section 60
                                                   (4485) 4485 4490           4500           4510           4520           4530           4540           4550   4560
Mycobacterium tuberculosis DNA AP018036.1 region fr... (4238) GATCCGGCTCGCCAGCTCCTCTTGCGCCTCCGGTCCGGCC-TCGAGCGACAGCAGGGCGCGAGCATGCCCGGCCGA
  Oryza sativa chromosome 8  NC_029263.1 region fro... (4265) TTTTAGGATAAAATTTTATACTTATAC-TCTGAGCAGTATATGAAACAAAAGAGAGACGAGAAAAGGACCCTTAAA
                                                                                                                          Section 61
                                                   (4561) 4561      4570           4580           4590           4600           4610           4620           4636
Mycobacterium tuberculosis DNA AP018036.1 region fr... (4313) CAG-CACGCCGGCGGCCACTCGCCGCTGTACCGGGATGGGGAGTTTGAGCAATCGGA--TCATGTTGGTGATCAAG
  Oryza sativa chromosome 8  NC_029263.1 region fro... (4340) CAGTCAAGCCTTCTTTTTTCTTCAACTTTAGCACTATA-----TATATGGAATTTTACTTCATATGAAAAATTATT
                                                                                                                          Section 62
                                                   (4637) 4637         4650           4660           4670           4680           4690           4700       4712
Mycobacterium tuberculosis DNA AP018036.1 region fr... (4386) GGCCGCGAGCGGCCGATGCGCGCCGCCAGTTCATCGTGGGTGACCCCGAATTCGT-----CGA--GCAATTGCTGG
  Oryza sativa chromosome 8  NC_029263.1 region fro... (4411) TAATGTGAACTAAATATGGGTCCCGCA---TCTGTGCAGGTTAGACAAAATACTTGCTCTCTATTGGAATCGCTCC
                                                                                                                          Section 63
                                                   (4713) 4713    4720           4730           4740           4750           4760           4770           4788
Mycobacterium tuberculosis DNA AP018036.1 region fr... (4455) TAT-GCCGCCGCTTCTTCTAACGG--ATTCAGCTGTACT-CGATGAATATTTTCCAGGAGGGCGTCGCGCAGCAGA
  Oryza sativa chromosome 8  NC_029263.1 region fro... (4484) AAGAGAATATGGGACCCAAAATGGTAACTCTGCTTTCTTTCAATAATTAATTTGCAATTTGG--TACCAAAGTAT-
```

Mycobacterium tuberculosis vs. Oryza sativa chromosome 8

Section 64

```
                                                     (4789) 4789       4800      4810      4820      4830      4840      4850          4864
Mycobacterium tuberculosis DNA AP018036.1 region fr... (4527) TTATCGTCGC-CGGTCTCACGCACGATGGCCGGGATGGTGGCCAAGCC------CGCCTCTTGGCAGCCC-GCCA
  Oryza sativa chromosome 8  NC_029263.1 region fro... (4557) TCATCGTGGCACAGGCACATGCA-GACGCAGTTGCTGATGACCAATCTAAATCTTAGCACTTGCAGAGATGAGACA
```

Section 65

```
                                                     (4865) 4865 4870      4880      4890      4900      4910      4920      4930      4940
Mycobacterium tuberculosis DNA AP018036.1 region fr... (4595) GCGC-----CGCTCCCCCATCACTATCT-GGTAGCGCACG---CCGGTTTGGGATCCAGCCAATGACCGCACCACG
  Oryza sativa chromosome 8  NC_029263.1 region fro... (4632) GGGCATGAGCAATTTACTATTATTTTTAAGGTGCCACACTAATCAAAATAGAGGATCATATCAGGCCCCAAATCTA
```

Section 66

```
                                                     (4941) 4941     4950      4960      4970      4980      4990      5000           5016
Mycobacterium tuberculosis DNA AP018036.1 region fr... (4662) ATCGGCTGCAGGAGACCGAATTCGCGGATGGAGTGCACCAACTCGGCCAGTGCCTCTT-CGTCGAACACCTGACGC
  Oryza sativa chromosome 8  NC_029263.1 region fro... (4708) AACAATAATAATATCCAGAAGAAAAGGAAACCTTGCAGAAGATCTCTCAGTGCATCATGCATGGATTGATTAACCG
```

Section 67

```
                                                     (5017) 5017         5030      5040      5050      5060      5070      5080        5092
Mycobacterium tuberculosis DNA AP018036.1 region fr... (4737) GGCTGACGGGGATTAGCCTCGATGG-----CGCT-CGGTGGGATTTCCCGATAGATGGCGCCCATCACGGAAGTGT
  Oryza sativa chromosome 8  NC_029263.1 region fro... (4784) ATCGAAAGGAGACTGATTTCAATTATATAATACTACTGATTGATTAGTTCTCAAGTGGCGAATTTAACTAATCAAC
```

Section 68

```
                                                     (5093) 5093   5100      5110      5120      5130      5140      5150              5168
Mycobacterium tuberculosis DNA AP018036.1 region fr... (4807) CCGGGACCGGTCC-----GCCGATTACGACATCTGCCGT--------GGCAGATCCC-ATCCGGGGACCCAAGGTC
  Oryza sativa chromosome 8  NC_029263.1 region fro... (4860) AGGAGATCGATCCATGGTTATGATTAATACATGTCGAGTAGTACGTAGGTACATGACTAACAAACGACACTGGACG
```

Section 69

```
                                                     (5169) 5169       5180      5190      5200      5210      5220      5230          5244
Mycobacterium tuberculosis DNA AP018036.1 region fr... (4869) GGTGGCCCCGATTCTCCG-TCTGCCGGGCC--AGTCGGGATCAGC-----GCAGCCAGGCCACGGCCGAGGCCACC
  Oryza sativa chromosome 8  NC_029263.1 region fro... (4936) ATTGTCGCCAAGTGTACGATGTACCATGTTTAATTCGGGTGCAAATATATATATTCATGTATAGATGCATGTCGCC
```

Section 70

```
                                                     (5245) 5245 5250      5260      5270      5280      5290      5300      5310      5320
Mycobacterium tuberculosis DNA AP018036.1 region fr... (4937) CTTTCTGCGTGACGGCTGGGTCATGGTCG--TCCCTTCGCGGATGGTGGTCGGTCACGCTCGGCAAGTTCGCGGCT
  Oryza sativa chromosome 8  NC_029263.1 region fro... (5012) GCACGCACGCGAATATTCAATTGTACATGAATACATATATAAATGAATGTTAGTGTTTTTTGGCCGGGACA-GGTT
```

Section 71

(5321) 5321 5330 5340 5350 5360 5370 5380 5396
Mycobacterium tuberculosis DNA AP018036.1 region fr... (5011) CGCGTCGAGGTAACTCATCGCGCCGCGCGAACCGGGATCGTAATCG-ATGATGGTCATGCTGTAGCCCGGCGCTTC
Oryza sativa chromosome 8 NC_029263.1 region fro... (5087) ATACT-GAT-TAATTAA--GCTTCTAAAGATTTTCTTTCCTAGGGGTATTTTAGATTTTGTGAACTTTTTTTCTTT

Section 72

(5397) 5397 5410 5420 5430 5440 5450 5460 5472
Mycobacterium tuberculosis DNA AP018036.1 region fr... (5086) GGAAACCTTGACGCTGCGTGGAATCACCGTCCGCAACACTTTGCTTCCGAAATACT--GACGGACCTCGTCGGCTA
Oryza sativa chromosome 8 NC_029263.1 region fro... (5159) GGAATTTTTGAGCTTATATATAATTACCTCCCTCTGTTTTCTGATTCTTATAGTCGTCTACAGGAATTGAAAAAAA

Section 73

(5473) 5473 5480 5490 5500 5510 5520 5530 5548
Mycobacterium tuberculosis DNA AP018036.1 region fr... (5160) CTTGATCGGCGAGCTTTGTCCGGCCGTCATACATGGTAAGGATCACGGTGGTGACCTCGAGTTGGGGGTTGAGGTG
Oryza sativa chromosome 8 NC_029263.1 region fro... (5235) AGAAGTGCTCTTGCTGTGTCCTAA-GTTACACA-GAAATAAACCATATTTGTTTATTT-ATTTATTTATTGAAATT

Section 74

(5549) 5549 5560 5570 5580 5590 5600 5610 5624
Mycobacterium tuberculosis DNA AP018036.1 region fr... (5236) GGCCTTCACCATCTCGATGTTGCGCATAAGCTGCGACACACCCTCCAACGCGTAGTACTCGCATTGGATCGGGATC
Oryza sativa chromosome 8 NC_029263.1 region fro... (5308) AACATATTT-ATTGATATTATATTATTAAGAAAGGTAAAAAAGTGAGCCACTATGTTCTCTCTAGAGATTTAATTA

Section 75

(5625) 5625 5630 5640 5650 5660 5670 5680 5690 5700
Mycobacterium tuberculosis DNA AP018036.1 region fr... (5312) ATCACCTCCGGTGCCGCGACGAGTGCGTTGATGGTCAGCAGCCCCAGCGAGGGCGGGCAATCGACGAAAACGTAGT
Oryza sativa chromosome 8 NC_029263.1 region fro... (5383) GAAAGTACCATTTATCAGAAAACAGAGTTTTTTTTTTGTTAGTCCGGATTTTTGTAGTGTACATAGATAAAGTTGT

Section 76

(5701) 5701 5710 5720 5730 5740 5750 5760 5776
Mycobacterium tuberculosis DNA AP018036.1 region fr... (5388) CGAAGTCGAAGTTGTCGAGTGCGGCCAGGGCGGTGCGCAACCGGTTCTCGCGCGCCACCATGCTCACCAATTCGAT
Oryza sativa chromosome 8 NC_029263.1 region fro... (5459) ATACACAAAATCTAT--AATGCTATCTA--CGGAAACAAAATTAAAGTTGATCGATAAAGTGAAATCCAATTGGAT

Section 77

(5777) 5777 5790 5800 5810 5820 5830 5840 5852
Mycobacterium tuberculosis DNA AP018036.1 region fr... (5464) TTCGGCGCCGG-----CCAGATCGA-TCGTCGCCGGGAT-GCAGAACAGCCGCTCGCTGTGCGGGCTGCGCCGTAG
Oryza sativa chromosome 8 NC_029263.1 region fro... (5531) TTTTAAAATATATATACAAAATAGAATTAATGCATCTATAGAAAAAAAGAGCTAATTAAAAGTTAGACATTGAAT

Mycobacterium tuberculosis vs. Oryza sativa chromosome 8

Section 78

```
                                                       (5853) 5853                                                                      5928
Mycobacterium tuberculosis DNA AP018036.1 region fr... (5533) CGC------CGTGTGCAACGAAACCTCGCCGATAAGCATCTCGTAGGACGAGGGTGTGCCGGATTGCCGGTCGGTG
  Oryza sativa chromosome 8  NC_029263.1 region fro... (5607) CCCTTTTATCTTTTAAAATCTAGTTGCTGCAAAAAAAAACCATATTTACAAAATCGTGATAAAAACATGTTTGGGC
```

Section 79

```
                                                       (5929) 5929                                                                      6004
Mycobacterium tuberculosis DNA AP018036.1 region fr... (5603) ATACCCA---ATGCGGTGCTCGCGTTGCCCTGGGGATCGAGATCGATCACGAGTGTCTTGAGGCCCTGC-ACAGCA
  Oryza sativa chromosome 8  NC_029263.1 region fro... (5683) AAATGCATAAAAAAATTTCTTTTTTAAGGAAGACAAATGCGCCACATTTGAAAAAGCTTAATAAGTTACCACATTT
```

Section 80

```
                                                       (6005) 6005                                                                      6080
Mycobacterium tuberculosis DNA AP018036.1 region fr... (5675) AGCGCGGCAGCGATATTGACGGCGGTGGTCGTCTTACCGACCCCGCCCTTCTGATTCGCGATGGTGAGCACCCGGC
  Oryza sativa chromosome 8  NC_029263.1 region fro... (5759) TGATTTTAAGA-ATATATATATATATGTAGCTATAAAATAAAAAAA-----TAAAT-GAGAAGTCATAAACTAATT
```

Section 81

```
                                                       (6081) 6081                                                                      6156
Mycobacterium tuberculosis DNA AP018036.1 region fr... (5751) GTCGACCCGGCCGCTGCAGCGGCTCGTGGGTGGTGTGCAGGACCCGCATCGCACGTTCTGCTGCAGCGCCGATGGG
  Oryza sativa chromosome 8  NC_029263.1 region fro... (5828) AAAAACCTAACATTTTCAGATTTT--TAAAAGCTATTCAACAACCA-ATC-CAGTTGCTTTT-CAGAATTTCAAGC
```

Section 82

```
                                                       (6157) 6157                                                                      6232
Mycobacterium tuberculosis DNA AP018036.1 region fr... (5827) GGTGTCGAATTCTGTCG---ATGTTTC--ACGTGAAACATTCATC-GTCGGATTGTGCGCGGCCTCAGGCGTCGGT
  Oryza sativa chromosome 8  NC_029263.1 region fro... (5899) TACGT-GAATTCAGACCGAAAAGTTACCAACGCAAATCAACCGTATGTCCAGTAAAAAAGGGGGACCCGTTTAGCT
```

Section 83

```
                                                       (6233) 6233                                                                      6308
Mycobacterium tuberculosis DNA AP018036.1 region fr... (5897) GTCGGTGGTGTCATTTCCCGCTGGAATGGTTCGATAGTTGAAGCCTGGCCCGACCTTACGAGC--GCGGACGGTCC
  Oryza sativa chromosome 8  NC_029263.1 region fro... (5974) AACCGTATGTATATAACAACACACAATAATGACAACAGTAAAGG-TGATTATAATTAGCTAGCTGGTTGCTGGTCC
```

Section 84

```
                                                       (6309) 6309                                                                      6384
Mycobacterium tuberculosis DNA AP018036.1 region fr... (5971) AG-CGGC-CACCGGGCCCCACGGAGCACTCACGCCGTCCCTCC-ACTCGCCATCCGTGCCGACCCTC--GGGCGAT
  Oryza sativa chromosome 8  NC_029263.1 region fro... (6049) GTACGGCTCCCATAGATAGATTGATAATGCAATGTGAGAGTGAGACCGACCAAACCGGCCGGCCATGCTGCCCCAT
```

Section 85

```
                                                  (6385) 6385 6390      6400      6410      6420      6430      6440      6450     6460
Mycobacterium tuberculosis DNA AP018036.1 region fr... (6042) CTGCTTTCCACGT-CGCGCGAACACCACGGTCGCGGGCGGACGCAAATAGTTCGCGCCACATGTCACCACCCTGAC
Oryza sativa chromosome 8  NC_029263.1 region fro... (6125) ATATTGTCCAGGTGCGTGCATGGTCCATACTC-CTATCTG-CTAACCTATCTGGTTTTTCTTTTATCTGCCGTACT
```

Section 86

```
                                                  (6461) 6461     6470      6480      6490      6500      6510      6520          6536
Mycobacterium tuberculosis DNA AP018036.1 region fr... (6117) ATCAACCGCGCCCGATGCGATCATCACACGCCGGTGCTACCGTACTTCGTCGTG-AGCCCGCTCGCCTTTGATGGC
Oryza sativa chromosome 8  NC_029263.1 region fro... (6199) GTGCACTCCCGGCAATGCTTAGCTGCAGTGACAGCATCAGCATGTTGTTTGGTTCAGTATGCA-GCTATAAAAGTT
```

Section 87

```
                                                  (6537) 6537         6550      6560      6570      6580      6590      6600     6612
Mycobacterium tuberculosis DNA AP018036.1 region fr... (6192) GAGCATTCGCCCGTTCGGCCGTATCAACGGCATGCTCCATTTCGTCAACTTGTCCAACGCGGCCACCGCCCGTGAC
Oryza sativa chromosome 8  NC_029263.1 region fro... (6274) TTATATATATATATATATATATATATATATATATATATATATATATATATATATATATATATATATATATATGTAT
```

Section 88

```
                                                  (6613) 6613   6620      6630      6640      6650      6660      6670          6688
Mycobacterium tuberculosis DNA AP018036.1 region fr... (6268) ACCGCAGCGTCGCTGCCGCCCAATTGGTCCTGCACCCAGGACTCCTCGGCGCGCCCCCGCACGATCTCAACGGCCA
Oryza sativa chromosome 8  NC_029263.1 region fro... (6350) ATATATATATATATGTATATATATATCTTAAACTATATGTTCCCTTGAGCTATCTCTAGTAGAAAAAGGGTGCACA
```

Section 89

```
                                                  (6689) 6689       6700      6710      6720      6730      6740      6750     6764
Mycobacterium tuberculosis DNA AP018036.1 region fr... (6344) CGCCCAGATCTGTCACCATCTCTCGAAGAAACTCGGTGCGGCGCAGTAGCGGGTCTAGGAGAACTACCTGGAGGTC
Oryza sativa chromosome 8  NC_029263.1 region fro... (6426) CATATAGCTTTTTGAACTTTTTGTTTATCTTATCCATCCCCTTC-GTAT------TATCATTATTCCTTCAAGTGC
```

Section 90

```
                                                  (6765) 6765 6770      6780      6790      6800      6810      6820      6830     6840
Mycobacterium tuberculosis DNA AP018036.1 region fr... (6420) CGGCCGCGCTATCGCCAATGGCACGCCC--GGCAACCCGGCTCCGCTACCGATATCCACGACCCGGTCACCGCGTT
Oryza sativa chromosome 8  NC_029263.1 region fro... (6495) AGTGCCAGCTTAAAATAGTTTTATAACCTAGACAAATATCACTAGAAAAGTATTTTCA-GAGACACTA--CTCTTT
```

Section 91

```
                                                  (6841) 6841     6850      6860      6870      6880      6890      6900          6916
Mycobacterium tuberculosis DNA AP018036.1 region fr... (6494) CGAGGAGCTCACCGATCACGGCGCAGTTCAGTAGATGCCGGTCCCATAGC-CTACCGACTTCGCGGGGTCCCACCA
Oryza sativa chromosome 8  NC_029263.1 region fro... (6568) GTA-----TCAGTGTACAGGCAATCGAAACATAGCCGCTTGTACAATAAAGCTAGGGAGGCCACGGTTTTAAATCG
```

Mycobacterium tuberculosis vs. Oryza sativa chromosome 8

Section 92

```
                                                   (6917) 6917         6930      6940      6950      6960      6970      6980        6992
Mycobacterium tuberculosis DNA AP018036.1 region fr... (6569) GCCCCCGCTCCACACCGGGTCCC----GCCAACGCTTCGGCGTACCGCCGAGCAAGGCCAAGCCGCGGTCCGAAGA
   Oryza sativa chromosome 8  NC_029263.1 region fro... (6639) TCTCTCTAT--AAATTGGTTTACAGAGGCGGACGCTAATTAAGACAACCGCCT-----CTAGAAATGGTTTG--GT
```

Section 93

```
                                                   (6993) 6993   7000      7010      7020      7030      7040      7050              7068
Mycobacterium tuberculosis DNA AP018036.1 region fr... (6641) TCGCAGACGCCGCGGGCTCGATCGGAGACATTACGCACTCCGCCGGCTCGTGAGGTCTGTGTCATGTTTCACGTGA
   Oryza sativa chromosome 8  NC_029263.1 region fro... (6706) TTTCAGATATAGTTGTCTCTA--GAAATCAATTTTCAGA--GATGGTTGCTTATGTCCCGTCCACCTATG--GAAA
```

Section 94

```
                                                   (7069) 7069       7080      7090      7100      7110      7120      7130          7144
Mycobacterium tuberculosis DNA AP018036.1 region fr... (6717) AACATTCTCCGCTCTCGAGACGCTGGC---CCAGCCGCTCGGCCACGCATCGCTTACTGCGGCGTCGGTCGGAGCC
   Oryza sativa chromosome 8  NC_029263.1 region fro... (6776) TTGATTTTTATGTCTAGAAATTATTTTTTTGGAACCACATATATACACATAACTTTTTCATATGTCATATAAAGAT
```

Section 95

```
                                                   (7145) 7145 7150      7160      7170      7180      7190      7200      7210     7220
Mycobacterium tuberculosis DNA AP018036.1 region fr... (6790) GCTGGCTCGCGAGCTAGTCGCGGAGCACAACGA--CTCGGCGTTCTGGCTCCACGCCTTCGCTTTCGCTGTGCACA
   Oryza sativa chromosome 8  NC_029263.1 region fro... (6852) AAA--CTCTAAAAAAAGTTGTAGAGCTCGACAAATCTATAACTTTAAAGTAGAAAGTTATTTTTTAGTTGAGATTA
```

Section 96

```
                                                   (7221) 7221     7230      7240      7250      7260      7270      7280              7296
Mycobacterium tuberculosis DNA AP018036.1 region fr... (6864) CCTGGCACCGCTGCAACCGCA-TCGTGGACGATCTTCC--GTTCG---AACGGCGTCATTGGAACGAGTTCCTCGC
   Oryza sativa chromosome 8  NC_029263.1 region fro... (6926) TTTG-CATTTCTAAATATACACTATAGGTTCTTGTACCTATTTAGATAAACATATTTTTTTAAAAAAATTGCACGA
```

Section 97

```
                                                   (7297) 7297         7310      7320      7330      7340      7350      7360        7372
Mycobacterium tuberculosis DNA AP018036.1 region fr... (6934) GGTCACCGGTTTCGGCCACTCGCCGCGCCACCTCGTCGGCCAGCGCCGCCAATTCCTCCCGGCGCCGCCGTCGCCA
   Oryza sativa chromosome 8  NC_029263.1 region fro... (7001) CCTAAATAGAGAAATACTCAATATCAAAATTATAGAATTTGAT-GATATCAAAAACTTTTGTACTAGTTGATGACT
```

Section 98

```
                                                   (7373) 7373   7380      7390      7400      7410      7420      7430              7448
Mycobacterium tuberculosis DNA AP018036.1 region fr... (7010) CCTCGCGATGTCTAGC-ATCAACCGGCTCCGCACACCGGTCTTCTG-ATGCACCGCCAACCGGGTGAGTTCCTGCA
   Oryza sativa chromosome 8  NC_029263.1 region fro... (7076) TTTTTT-ATTTGAAGTTATATACACACTACTGAT---GGTCGTCTGGATGCGAC--CATCTATGAAAATTAATACT
```

```
                                                                                                                             Section 99
                                                        (7449) 7449       7460      7470      7480      7490      7500      7510          7524
Mycobacterium tuberculosis DNA AP018036.1 region fr... (7084) GAGCGTCGAGCACCTCGCCCCCGCGCCCGACCAACTTGTTCAGGTCGTCACTGCCGTCGATGCTCACCACCGCACG
  Oryza sativa chromosome 8  NC_029263.1 region fro... (7146) G------GATCTATTCTCCCATGTTAATAATTAATTAGTTG-GGACGAAATAACA----AAATCAACGAAAATTAG
                                                                                                                             Section 100
                                                        (7525) 7525 7530      7540      7550      7560      7570      7580      7590      7600
Mycobacterium tuberculosis DNA AP018036.1 region fr... (7160) ATTGCCTTCGACA-TCGAGGTCGATGTCGCCATCGAAGTCCAACACGTCCAATAACTCTTCCAGGTAGTCGCCTGC
  Oryza sativa chromosome 8  NC_029263.1 region fro... (7211) ACTGAAGTGAACAATCAAACTAACAG-----AAAGAACTGTA-CTGATCTGATCTCTCAGTATAATA-TAAAAAGA
                                                                                                                             Section 101
                                                        (7601) 7601     7610      7620      7630      7640      7650      7660           7676
Mycobacterium tuberculosis DNA AP018036.1 region fr... (7235) AATCTCGCCCTCGGC--GACCAATCTCTCTTCTTGATCGTCGGCCTCGTCAGCATCCGTCGCCGT-GTCCTCCCGG
  Oryza sativa chromosome 8  NC_029263.1 region fro... (7280) GATGTAACCAACAACCAGACAAAGAAAATTTTGCCCCCAGCTAGCTTATTATTGGCTGGCCACGTTGATCGTTGGC
                                                                                                                             Section 102
                                                        (7677) 7677         7690      7700      7710      7720      7730      7740      7752
Mycobacterium tuberculosis DNA AP018036.1 region fr... (7308) ACGCCTCCACCCGGTGCTTCTGCGTCGACGTCGAAGTCGGTGGTGTCAGCGTCGGCCATGGCTTGCTCTCCCCTCG
  Oryza sativa chromosome 8  NC_029263.1 region fro... (7356) ACGCCTCAAA----TATATATGTGCCCG-GTCAATCTCGATTGTATCTGCTAGTAGCTACTTACAATCAAACGATG
                                                                                                                             Section 103
                                                        (7753) 7753  7760      7770      7780      7790      7800      7810               7828
Mycobacterium tuberculosis DNA AP018036.1 region fr... (7384) TCTGCAGGTGG--GTTGTGTTTGTGGACCGCCTGCCCGGCTGCCCGGAAGGATTGTCAACGTTTGCGTTTT-TC
  Oryza sativa chromosome 8  NC_029263.1 region fro... (7427) GAAGCAGGACGACGTCGAGATTAAAGAA--GATAAAAAGGATT----GAA--AATAACAGCACATGCATGGCAGTA
                                                                                                                             Section 104
                                                        (7829) 7829       7840      7850      7860      7870      7880      7890          7904
Mycobacterium tuberculosis DNA AP018036.1 region fr... (7457) GGTCGCACCCCGGGCCGCGGCGTACGGGCGCTCGGGCCGCTGTTGCGTCTGGCCGGATT-GGACGTGTCGGCTGGT
  Oryza sativa chromosome 8  NC_029263.1 region fro... (7495) TGGTACATACCGATCAGGGGCCTAAGTAAAAAACAAAAGTTCCTAATTAAACACGATTTTGGCAGGATCAACTGGT
                                                                                                                             Section 105
                                                        (7905) 7905 7910      7920      7930      7940      7950      7960      7970      7980
Mycobacterium tuberculosis DNA AP018036.1 region fr... (7532) CGCTCAGTG---CTGGCGTCCGACTCTGCCCCGTCATCGGT-GTCCCCGGCT-TCTGTTGGGGCTGCCGCATTGGT
  Oryza sativa chromosome 8  NC_029263.1 region fro... (7571) TAATTAATTAATATATTTACCTGTTTAACACTATATCCAGTAGTTACCAGCTATATGAGTTATCTGCAAAACCCAT
```

Section 106

```
                                                           (7981) 7981     7990           8000           8010           8020           8030           8040           8056
Mycobacterium tuberculosis DNA AP018036.1 region fr...     (7603) CGCTGGAGCGGTCTTCGGCTCCGCTTGGGCTTAGCTCCCGGGGCCGGCGCGTTGGCCG---CCCGGCGCCGGACC
 Oryza sativa chromosome 8  NC_029263.1 region fro...     (7647) GCCATTAATTAAC----GGTGACACTGCAAATTTGCAGTCACTTCATGCATTATAGCCTTTTCCCGGCT--GGTT
```

Section 107

```
                                                           (8057) 8057         8070           8080           8090           8100           8110           8120           8132
Mycobacterium tuberculosis DNA AP018036.1 region fr...     (7676) GCCTCCTGCTTTTTGGCCTCCTCCTCCTTTCGATCATG---CCGAAGACGTAATGCTGCTGCCCGAACGTCCAGA
 Oryza sativa chromosome 8  NC_029263.1 region fro...     (7717) TACTC--GCTTTTTTTCCAGAAGGATGTGTCCACGTCTGACACCAAACTTCAGAGGCTAATACACAGTAGCCTACT
```

Section 108

```
                                                           (8133) 8133   8140           8150           8160           8170           8180           8190                8208
Mycobacterium tuberculosis DNA AP018036.1 region fr...     (7749) TATTGTTCGAGAACCAATACAAGATGATCGCCAGTGGCAGGAACGGTCCGCCGACGACTACGCCGAGCGGAAATAC
 Oryza sativa chromosome 8  NC_029263.1 region fro...     (7791) AATTAAGCTTAATTTACATCTAGAAACTTTCTCCTACCGGCAATTAGCCGCAGACGCTT--GCCTGCTTCAAATTC
```

Section 109

```
                                                           (8209) 8209        8220           8230           8240           8250           8260           8270           8284
Mycobacterium tuberculosis DNA AP018036.1 region fr...     (7825) GTACAGCGCCAGCTTGTTCATCATCGCGGTCTGTGGATTCGCAGCCGCCTCGGCGCTCTGCCGCGCGATAGACGCG
 Oryza sativa chromosome 8  NC_029263.1 region fro...     (7865) --AATTCACCAGTTGTTAGTTTAGAGTATGCTGAAATTATACTTTTGGTTGTTTCCTATAACTATCTGTGTATGCA
```

Section 110

```
                                                           (8285) 8285 8290           8300           8310           8320           8330           8340           8350     8360
Mycobacterium tuberculosis DNA AP018036.1 region fr...     (7901) CGACTGTTGAAGTACGTCGCGATGCCGGCCAAGATCA--TCACCGGCACA--CCCACCGCGATCAACGCAGGGC-G
 Oryza sativa chromosome 8  NC_029263.1 region fro...     (7939) --ATTGAT-AATTTTTTTTTAATTTCATTACAGATCAACTCATTGGATTTGTCTTATGGTAGTTATTTAAAAAATG
```

Section 111

```
                                                           (8361) 8361     8370           8380           8390           8400           8410           8420           8436
Mycobacterium tuberculosis DNA AP018036.1 region fr...     (7972) ACTGAAATCGACGAACGCATCCAACCCGGACCGTTGCGTCATGTACGCCCCGATCG---GAGCGCCGAACAAGTTG
 Oryza sativa chromosome 8  NC_029263.1 region fro...     (8012) TCATTAATCAA--AATGTTTACTACTAATTTATATATATAGTTGACTCCAAAATTTTTTGATTAACATCCACTCTC
```

Section 112

```
                                                           (8437) 8437         8450           8460           8470           8480           8490           8500           8512
Mycobacterium tuberculosis DNA AP018036.1 region fr...     (8045) GCATCTAGGAAGTGGCCGACGTCGACCGGGCTAAA--GACGTAGTTCCCAGTCAGTCGGTTCTCGATCACCGACAA
 Oryza sativa chromosome 8  NC_029263.1 region fro...     (8086) TCCGTTTTATATTATAAGTCGCTTTGTTTTTTAAATTAAATTTGTTTATATTTGATCAAATTTATAGAAGAAATTA
```

Mycobacterium tuberculosis vs. Oryza sativa chromosome 8

Section 113

```
(8513)                                              8513   8520      8530      8540      8550      8560      8570            8588
Mycobacterium tuberculosis DNA AP018036.1 region fr... (8119) GTGTGGTTGACCAAAGCCCCCGGTCGTACGGTTAAACGAGCGCAACACATGATAGAGCCCAAGAAACACCGGAATC
Oryza sativa chromosome 8  NC_029263.1 region fro... (8162) GTAAAATTAATAAAA--CTAAATTATTTTCATTATACATAT--AACATTGAATATATATTTATAATATGTTAGTTT
```

Section 114

```
(8589)                                              8589       8600      8610      8620      8630      8640      8650        8664
Mycobacterium tuberculosis DNA AP018036.1 region fr... (8195) TGCGCCAGCATCGGCAAA-----CATCCGAGAATGGGGTTGAAGCCGTGCTCGCGTTGC-AGCTTTTGCATTTCGA
Oryza sativa chromosome 8  NC_029263.1 region fro... (8234) TATGTTAGAAATGTTATTATGTTTTTCTATAAATTTGGTTAAACTCAAAGAAGTTTAACTATAAAAAAGAATCAAA
```

Section 115

```
(8665)                                              8665 8670      8680      8690      8700      8710      8720      8730     8740
Mycobacterium tuberculosis DNA AP018036.1 region fr... (8265) GCGCCATCCGCTGACGATCCTTGCCG---TATTTCTTTTGCAAGGCCTTGATCTGTGGTTGCAGTTCCTGCATCTG
Oryza sativa chromosome 8  NC_029263.1 region fro... (8310) ACTTCTTATAATATGAAACGGAGAAAATATATGTTTTCTTGTAGGACTT-ATCTATGTTTATAGAGT-TGGTTATG
```

Section 116

```
(8741)                                              8741     8750      8760      8770      8780      8790      8800            8816
Mycobacterium tuberculosis DNA AP018036.1 region fr... (8338) CCTGGTGGTGCGAATCTGGCGCA-CGAACGGCTTGTACAGCAGC--GCACGCAGCGTGAAGACCAGGAACATCACC
Oryza sativa chromosome 8  NC_029263.1 region fro... (8384) AAAAGGAAACTAACTTTCTTACTGCGTACA-CTTATATATTAGCTAGGTCCAAAAATTCTATCGGGGTTCA--AAT
```

Section 117

```
(8817)                                              8817         8830      8840      8850      8860      8870      8880      8892
Mycobacterium tuberculosis DNA AP018036.1 region fr... (8411) GACAACGCCCAGGCGAAGAAGTTGGATGGTCCTAGCACAAACGCGAACAGCCGGTACCAAACCCACAT-GATCCAC
Oryza sativa chromosome 8  NC_029263.1 region fro... (8457) TTAGGTGCCTAGCTATTTACTTTTGAAACCACTTGCACCAAATTTC-CTTTTGTTA--AAACTCATATTGATCACC
```

Section 118

```
(8893)                                              8893   8900      8910      8920      8930      8940      8950            8968
Mycobacterium tuberculosis DNA AP018036.1 region fr... (8486) GACACCGGGTAGTAGAT--GAAGTCGAGACTGAAGAAATCAAACAAAAGACTCACGCTCCCCTCGCTTTGACGC--
Oryza sativa chromosome 8  NC_029263.1 region fro... (8530) ACTAATGAAGAGTACATATGTAGTATAGTGTGCACAATAATTAATCATGTTTTAGAATCCGTTTGGTTTTAATCTT
```

Section 119

```
(8969)                                              8969       8980      8990      9000      9010      9020      9030        9044
Mycobacterium tuberculosis DNA AP018036.1 region fr... (8558) ---AG-GGTTCCAGTCGTCGTTCGCGCCGTCGACGT--CTGTCTGGCAGCTCCGGCCTGTCGTTAAGCCTTCCGGT
Oryza sativa chromosome 8  NC_029263.1 region fro... (8606) CATAGCGGTATATGAAACCGTACATTAACTGAACTTGACTGATTAACCTACCTAGGCTCGTTTTTTGTTTGACAGT
```

```
                                                                  Section 120
                                                   (9045) 9045 9050      9060      9070      9080      9090      9100      9110     9120
Mycobacterium tuberculosis DNA AP018036.1 region fr... (8628) ATCGGATCCCATCCTCCCCGATGCCATGGTCCGCACTTTGCGAGCCTGATCATGGTCAACC--AGCTTCCCCGCAA
  Oryza sativa chromosome 8  NC_029263.1 region fro... (8682) AAGTGTGTCCATTAGTCCTGC-GAGAGTGACATTACTGTACTATCATGAT-ATGATATATAAAAGCTCTGGAGCAA
                                                                  Section 121
                                                   (9121) 9121      9130      9140      9150      9160      9170      9180           9196
Mycobacterium tuberculosis DNA AP018036.1 region fr... (8702) CAGGCCATACTCGGTGAGCGCATCGACGGCGTACTGACTACA-----GGTAGGACAAAGCGGCACGACGCCGGTC
  Oryza sativa chromosome 8  NC_029263.1 region fro... (8756) AAG---AGACTTGGC-ATCCCATTTGTAACTACCAGAGAAGAAACTTTGGAGAAATGAAGTTTGACAAGGCCCATC
                                                                  Section 122
                                                   (9197) 9197         9210      9220      9230      9240      9250      9260      9272
Mycobacterium tuberculosis DNA AP018036.1 region fr... (8773) GTAGCGGCGAAAGCATGTGCCGATAGACCT--GGATAACGAAAATCAACCCCCGCGCTGATGCTCTACCGGTAACC
  Oryza sativa chromosome 8  NC_029263.1 region fro... (8828) AGCTAGATAACACTACAGGACAATGAAATTAAAGTTATTAATTAATTACTAGCTAATAGTTGCATGCAAGTTAATT
                                                                  Section 123
                                                   (9273) 9273    9280      9290      9300      9310      9320      9330           9348
Mycobacterium tuberculosis DNA AP018036.1 region fr... (8847) CGGACCACGCGCCCACAGCTTTGCCTAGACAGACTCACCGATCACTACCTGCCAGTTCGACAGCCCTCCGCAAGCC
  Oryza sativa chromosome 8  NC_029263.1 region fro... (8904) GGGAATTATATAGTTTGTCGCTATATGTATAGATCTA--GTTAATTACCT--CAGCAT-ATATATATATGCAGGTT
                                                                  Section 124
                                                   (9349) 9349        9360      9370      9380      9390      9400      9410        9424
Mycobacterium tuberculosis DNA AP018036.1 region fr... (8923) GCATCGCAGT--TGCTGCTCCAACCGAGCCGAGGAGACATGCCGGCTGCTCGGC-AGCGCGCGGATCACCACATGA
  Oryza sativa chromosome 8  NC_029263.1 region fro... (8975) GCAGCCTCGTACAGCTACTTTATAGGTGCTATAGAGATATGTAAGTATATATACTACAGTAGTGTTGATCAC-TGA
                                                                  Section 125
                                                   (9425) 9425 9430      9440      9450      9460      9470      9480      9490     9500
Mycobacterium tuberculosis DNA AP018036.1 region fr... (8996) TCGGACGGGTGGAGTTCTTTGACGATCGACCCAGCCACGTGCCGCAGCCGACGTGCCACGCGGTGGCGTTCCACGG
  Oryza sativa chromosome 8  NC_029263.1 region fro... (9050) TCA-ACCTGAGTAATTAATTAATTAATTAACAGTGCACATACATAATTACATATGTTGGATGTATTCTCCGAAATG
                                                                  Section 126
                                                   (9501) 9501      9510      9520      9530      9540      9550      9560           9576
Mycobacterium tuberculosis DNA AP018036.1 region fr... (9072) CCGACCCCACCGACTTGGCGATAATCAGTCCGACGCGCGGCCCACCGCCACTCCCACGCCACCAATAAACGACCAT
  Oryza sativa chromosome 8  NC_029263.1 region fro... (9125) GAAAATTAATTAATGTGCATATATACAGACACACACGTG-----CAGGCAAAGCAGAAAGATCAACTTTGTGTTAG
```

Mycobacterium tuberculosis vs. Oryza sativa chromosome 8

Section 127

(9577) 9577 9590 9600 9610 9620 9630 9640 9652

Mycobacterium tuberculosis DNA AP018036.1 region fr... (9148) GTCAGACCGCACGGTACGCATCCCGTGCTTCACCGTTGTTTCAAAATCCGCTGACCGCCTCATGCGGTTGCGTGCA

Oryza sativa chromosome 8 NC_029263.1 region fro... (9196) TTGCCACCTAATATTAATTAATTTAATATTCAGA-TAGTTTGACCAAACGG-GACGTTGTTTT----TTGAAATAA

Section 128

(9653) 9653 9660 9670 9680 9690 9700 9710 9728

Mycobacterium tuberculosis DNA AP018036.1 region fr... (9224) CGAAGCACCGCAAATAAGCCCGGTGTT-GCAATCAAGCACTGAGCGTGCGCCGACC-CTTGCGTCGCCGGCTGGAC

Oryza sativa chromosome 8 NC_029263.1 region fro... (9266) AAATATATAGAGGAGGGGGACAGTCTTTGAGAAAAAAAATTCAGAGTTAGCAAGCTGCCTGTGGCCATGGTAGAAA

Section 129

(9729) 9729 9740 9750 9760 9770 9780 9790 9804

Mycobacterium tuberculosis DNA AP018036.1 region fr... (9298) ACAATTGACCTCCCGGCGCGGGTACGCAT---CCGTAAGCGGAAA-CCGTGAACACGAGCTCGCCGCCGGTTGTTC

Oryza sativa chromosome 8 NC_029263.1 region fro... (9342) ATATTTCCTCGCAAAAGAAAAGAAAATATTTCCCTTTTTCATCGATCCATGTA-ATATCATTATTGTAGTATTTCA

Section 130

(9805) 9805 9810 9820 9830 9840 9850 9860 9870 9880

Mycobacterium tuberculosis DNA AP018036.1 region fr... (9370) GGCTGGAAGGTCCTTTTGCCCTTGGTCACGGGCGTCTCCTCGCTATGTCTGGCAACATCACCATCCGGCCACTCAC

Oryza sativa chromosome 8 NC_029263.1 region fro... (9417) ATCATTAAGTTCAGTAAGCATGTGACCTATATCGTGTCA-CTAAATGAAAAAAAAAAAAACTCTTGGGTGC-----

Section 131

(9881) 9881 9890 9900 9910 9920 9930 9940 9956

Mycobacterium tuberculosis DNA AP018036.1 region fr... (9446) TGCCTTCCAACTCGATTGGCCCGCGGGACAGTCGGAGGTGGTTTTCGCTGCTGGCCGGCGCGGTCCCTGGACTAAT

Oryza sativa chromosome 8 NC_029263.1 region fro... (9487) -GCATAAAAA----ATTAACTATCTTCAAATTTTAGGGGAAATTGTTCTCAAAGACTA-GAAATTGGTGTA-TAGT

Section 132

(9957) 9957 9970 9980 9990 10000 10010 10020 10032

Mycobacterium tuberculosis DNA AP018036.1 region fr... (9522) CCAGGTCGCAGCCGCATCGCCGACTTTCGGGCGACTGTTCG--AGGGTACTTACGCGCCTTCGCCTGGTCAAACCT

Oryza sativa chromosome 8 NC_029263.1 region fro... (9556) CCTTTTGTTAGTTTTTTTCTTGATATTTAAGATATTGATATTAATTGTCTCTATTGGACAT-GCTTGATCATTTTT

Section 133

(10033) 10033 10040 10050 10060 10070 10080 10090 10108

Mycobacterium tuberculosis DNA AP018036.1 region fr... (9596) CGCCCACCCGGCAACCGCTTCAGGGCATCCTGCCCGCTAAGCTGCTCACCATCCGTACACCCGAGACCGCCACACT

Oryza sativa chromosome 8 NC_029263.1 region fro... (9631) TTCTGATTATATAAATG-TAAAGGTACACGTGAAAGAACTTTTTATCAAAAATCGAATAACATAATTTTTCACATA

Section 134

	(10109)	10109 10120 10130 10140 10150 10160 10170 10184
Mycobacterium tuberculosis DNA AP018036.1 region fr...	(9672)	CACAAAGAACCCACCACAACGCAAAACAACGGTTGGCAGCCGTACGGAAAACTGTT--AGCTTCGGGCGGTGTAGT
Oryza sativa chromosome 8 NC_029263.1 region fro...	(9706)	TTAAATCTTAACATTTTTGTATATATTAGCGAGTTAGAACATCCGGTTTAACTATACATGATATAAAACAACTAGT

Section 135

	(10185)	10185 10190 10200 10210 10220 10230 10240 10250 10260
Mycobacterium tuberculosis DNA AP018036.1 region fr...	(9746)	TATCACGCCGTTTCAGCGTGGAAACGGCACTCGACAATCAAGCGAGGATGGCGGATCGACTAGCGGCCCGGACAAC
Oryza sativa chromosome 8 NC_029263.1 region fro...	(9782)	AAAAATA--GTATGAGAAATTTTACCGTCCTTTATAAATACTA-AGTACCACACATTTTCTAATAT-----AAAAG

Section 136

	(10261)	10261 10270 10280 10290 10300 10310 10320 10336
Mycobacterium tuberculosis DNA AP018036.1 region fr...	(9822)	TTGAACCGGGTGTTTTCAACACGAGGATCGCGAGCCGTTGCCGGTAGGTTGCGGCTGGTTATCGACGGTACTGTCC
Oryza sativa chromosome 8 NC_029263.1 region fro...	(9850)	TAGTATATA-TCATTACATTTTAAGCAACATAAGAAATAGAAATGAAATCACTA-TGGAAAGAGA--GAAATCTCA

Section 137

	(10337)	10337 10350 10360 10370 10380 10390 10400 10412
Mycobacterium tuberculosis DNA AP018036.1 region fr...	(9898)	ACATTTGTGGATAGCCATGTGGACAGTTCACCTGCCCACAACAACGGTTGTAGCTCGACCCGGAACCAAGACCCGG
Oryza sativa chromosome 8 NC_029263.1 region fro...	(9922)	AAATTAA-GGATAG--ATGTACACATGTAGTGTTAAGTAATTAA---TTATTTATATATATGTCTCTTAAA-----

Section 138

	(10413)	10413 10420 10430 10440
Mycobacterium tuberculosis DNA AP018036.1 region fr...	(9974)	AACTAACGAGAACCAGGGAGATACGTCG
Oryza sativa chromosome 8 NC_029263.1 region fro...	(9987)	-ATTTACTATATCCCT------------
