# Supplementary material for: Patch type nucleotide sequence identities between genomes from many different species facilitate illegitimate recombination
Source: Sci Rep. 2026 Mar 30;16:10524. doi: 10.1038/s41598-026-44124-0 (PMC13035915; doi:10.1038/s41598-026-44124-0)
Supplement: Supplementary file 9 — Supplementary Material 9 [file 41598_2026_44124_MOESM9_ESM.pdf]

SARS-CoV-2 & Fig badnavirus 2

```
                                                        Section 1
                                      (1)   1        10        20        30        40        50        60        70        80        92
SARS-CoV-2 region 1.978-9.647nt       (1)   TGGAATTTCACAG-TATTCACTGAGACTCATTGATGCTATGATGTTCACATCTGATTTGGCTACTAACAATCTAGTTGTAATGGCCTACATT
Fig badnavirus 1 NC_017830.1          (1)   TGGTA--TCAGAGCTAGTTA-TGAGTGAGTAGGTTCTTATAAGGTATTCGGGTAAACAGGG----AAAGATTTATTATGCCTAGATTGATTT
                                                        Section 2
                                      (93)  93     100       110       120       130       140       150       160       170       184
SARS-CoV-2 region 1.978-9.647nt       (92)  ACAGGTGGTGTTGTTCAGTTGACT---TCGCAGTGGCTAACT-----AACATCTTTGGCACTGTTTATGAAAAACTCA--AACCCGTCCTTG
Fig badnavirus 1 NC_017830.1          (86)  GATAATCTTGTTTCTCAGATCTCTGAGTTGTAGTATCTAAAAGGAAAAAGTCCTTGGGCACTATATCTGGTTAAGGCAGGAGGCCGTG--TG
                                                        Section 3
                                      (185) 185  190       200       210       220       230       240       250       260       276
SARS-CoV-2 region 1.978-9.647nt       (174) ATTGGCTTGAAGAGAAGTTTAAGGAAGGTGTAGAGTTTCTTAGAGACGGTTGGGAAATTGTTAAATTTATCTCAACCTGTGCTTGTGAAATT
Fig badnavirus 1 NC_017830.1          (176) ATACCAGTGAA---AC-CCTGAGGTGATCCTGATGGATATGAGTCATGTCTGAGAAATGGGAAAGGTCAATTCAGGAT-TGGTATAATAATT
                                                        Section 4
                                      (277) 277          290       300       310       320       330       340       350       368
SARS-CoV-2 region 1.978-9.647nt       (266) GTCGGTGGACAAATTGTCACCTGTGCAAAGGAAATTAAGGAGAGTGTTCAGACATTCTTTAAGCTTGTAAATAAATTTTTGGCTTTGTGTGC
Fig badnavirus 1 NC_017830.1          (263) CTAG-------AACTGCGAACCTTG--------AATACCTTGATTTAGCAGAAAGGAAAAACC-----AACAAATTCTCACCTTTA---CC
                                                        Section 5
                                      (369) 369         380       390       400       410       420       430       440       450     460
SARS-CoV-2 region 1.978-9.647nt       (358) TGACTCTATCATTATTGGTGGAGCTAAACTTAAAGCCTTGAATTTAGGTGAAACATTTGTCACGCACTCAAAGGGATTGTACAGAAAGTGTG
Fig badnavirus 1 NC_017830.1          (332) ATAATCTAGCAGTA--GTTTACGATAGACT--AAACTTGCAATCTCGTGTTAACCTCAAAAAC---CTTAAGGGTATTTT--AGAAAGAGTG
                                                        Section 6
                                      (461) 461      470       480       490       500       510       520       530       540       552
SARS-CoV-2 region 1.978-9.647nt       (450) TTAAATCCAGAGAAGAAACTGGCCTACTCATGCCTCTAAAAGCCCCAAAAGAAATTATCTTCTTAGAGGGAGAAACACTTCCCACAGAAGTG
Fig badnavirus 1 NC_017830.1          (415) GAAAAACAAGAGAGAAAACTTGAGAGTTTAGAACT---A--GCAGTAAGGAACCTTACCCAAGTTTTGTTGAAAATAAGCCTTTAACA---
                                                        Section 7
                                      (553) 553    560       570       580       590       600       610       620       630       644
SARS-CoV-2 region 1.978-9.647nt       (542) TTAACAGAGGAAGTTGTCTTGAAAACTGGTGATTTACAACCATTAGAACAACCTACTAGTGAAGCTGTTGAAGCTCCATTGGTTGGTACACC
Fig badnavirus 1 NC_017830.1          (499) ---ACAACTGAAGTT----AGAAGGCTTGT--TTACGAAATCTCTCAGCAACCAAAGCTTGTAGAGCAGGAGGCTCTAAGGTTAACTGAAGA
```

```
                                                                                                         Section 8
                                 (645) 645  650            660            670            680            690            700            710            720            736
SARS-CoV-2 region 1.978-9.647nt  (634) AGTTTGT-ATTAACGGGCTTATGTTGCT-CGAAATCAAAGACACAGAAAAGTACTGTGCCCTTGCACCTAATATGATGGTAACAAACAATAC
   Fig badnavirus 1 NC_017830.1  (582) ACTTCGTCAAAAACTCGAAAGAGTTGAGGCGATAGTAAAGAAAGTTGAAAGT--TGGACCTCTTCATGAGTAGTTATATTGTCTCCCAAGGG
                                                                                                         Section 9
                                 (737) 737          750            760            770            780            790            800            810            828
SARS-CoV-2 region 1.978-9.647nt  (724) CTTCACA-CTCAAAGGCGGTGCACCAACAAAGGTTACTTTTGGTGATGACACTGTGATAGAAGTGCAAGGTTACAAGAGTGTGAATATCACT
   Fig badnavirus 1 NC_017830.1  (672) ACAAATAGCTACAAGGAGG-----CCATAAAAGCTACTGAAGGAATAGAATCCCCAGCAGCAGGGTTCG-TGAGACCAGCC-GACTTCCAGG
                                                                                                         Section 10
                                 (829) 829         840            850            860            870            880            890            900            910      920
SARS-CoV-2 region 1.978-9.647nt  (815) TTTGAACTT--GATGAAAGGATT--GATAAAGTACTTAATGAGAAGTGCTCTGCCTATACAGTTGAACTCGGTACAGAAGTAAATGAGTTCG
   Fig badnavirus 1 NC_017830.1  (757) GAGGAACATCGGCAGCGAGGGTTCAGATTAAGCAGAATAATACCCTTATTCAGCTTCTA--ATCCAGATCGCTGAGTCCCTGAAGGACATC-
                                                                                                         Section 11
                                 (921) 921      930            940            950            960            970            980            990            1000     1012
SARS-CoV-2 region 1.978-9.647nt  (903) CCTGTGTTGTGGCAGATGCTGTCATAAAAACTTTGCAACCAGTATCTGAATTA-----CTTACACCACTGGGCATTGATTTAGATGAGTGGA
   Fig badnavirus 1 NC_017830.1  (846) CGAGAAGAGCAGCGAGTCCTAAAGGAGGAGGTTAGGCAACTGCAGAAGGACAAAGCAGCTTCAATCACAGAAGAGCTTGTGGAAAGACTTCA
                                                                                                         Section 12
                                (1013) 1013   1020           1030           1040           1050           1060           1070           1080           1090     1104
SARS-CoV-2 region 1.978-9.647nt  (990) GTATGGCTACATACTACTT--ATTTGATGAGTCTGGTGAGTTTAAATTGGCTTCACA--TATGTATTGTTCTTTCTACCCTCCA-GATGAGG
   Fig badnavirus 1 NC_017830.1  (938) AAGTCTCTCGTTAGGAGTCCCAGAAAAGAAGGTAGCTGAGAAGAAAAGAACTTTTAGGGTATTCAAAGACCCTTTAAGAATCCTTGAAGAGG
                                                                                                         Section 13
                                (1105) 1105 1110           1120           1130           1140           1150           1160           1170           1180     1196
SARS-CoV-2 region 1.978-9.647nt (1077) ATGAAGAAGAAGGTG--ATTGTGAAGAAGAAGAGTT---TGAGCCATCAACT-CAA--TATGAGTATGGTACTGAAG-ATGA--TTACCAAG
   Fig badnavirus 1 NC_017830.1 (1030) AAAAAGAA-AAGCTGCCAAAATGAACAGGTCGAGAACGGTGACTCAGCAGCTGCCAGCTGTGAGTACCAGACGGAGGAAGAATTAACAGG
                                                                                                         Section 14
                                (1197) 1197         1210           1220           1230           1240           1250           1260           1270           1288
SARS-CoV-2 region 1.978-9.647nt (1158) G-TAAACCTTTGGAATTTGGTGCCACTTCTGCTGCTCTTCAACCTGAAGAAGAGCAAGAAGAAGATTGGTTAGATGATGATAGTCAACAAAC
   Fig badnavirus 1 NC_017830.1 (1121) GATCAACCTCTG----TTCGAGG---ATCAGATCCGTGACTACCGGAGGAATCA-AAGGCGAAGGTTTGT-AGCTG---------AACAAGC
```

SARS-CoV-2 & Fig badnavirus 2

Section 15

```
                                     1289       1300            1310      1320      1330      1340      1350      1360      1370      1380
                             (1289)
SARS-CoV-2 region 1.978-9.647nt (1249) TGTTGGTCAACAAGACGG-CAGTGAGGACAATCAGACAACTACTATTCAACAATTGTTGAGGTTCAACCTCAATT-AGAGATGGAACTTAC
Fig badnavirus 1 NC_017830.1 (1195) CGT--------AAGACGAACCATGGGAAGAATGCGAGGACGACGATACAACCAAACTCTGGAGCAGATCGTAGATCCAGAGGTAGAGCTGAA
```

Section 16

```
                                     1381     1390      1400      1410      1420      1430      1440      1450      1460      1472
                             (1381)
SARS-CoV-2 region 1.978-9.647nt (1339) ACCAGTTGTTCAG--ACTATTGAAGTGAATAGTTTTAGTGGTTATTTAAAACTTACT----GACAATGTATAC-ATTAAAAATGCAGACATT
Fig badnavirus 1 NC_017830.1 (1279) CCAATCTATGCAGGAAAGAGCGAACCTAGTACCTGCAGAAGTTCTGTACCGCTCAAGAAGAGACAACATAAACCATCAGATCTATAATCATA
```

Section 17

```
                                     1473   1480      1490      1500      1510      1520      1530      1540      1550      1564
                             (1473)
SARS-CoV-2 region 1.978-9.647nt (1424) GTG--GAAGAAGCTAAA--------AAGGTAAAACCAACAGTGGTTGTTAATGCAGCCAAT--GTTTACCTTAAACATGGAGGAGGTGT-TG
Fig badnavirus 1 NC_017830.1 (1371) GATCCGAGGAAGCGATGCTTTGTGTAGGAGAACAGCAAGACAGGATGTTTATCCAACCTAACAGCTTCCAAGAACTACAGAGGAGTGGTATG
```

Section 18

```
                                     1565 1570      1580      1590      1600      1610      1620      1630      1640      1656
                             (1565)
SARS-CoV-2 region 1.978-9.647nt (1503) CAGGAGCCTTAAATAAGGCTACTAACAATGCCATGCAAGTT-GAATCTGATGATTACATAGCTACTAATGGACCACTTAAAGTGGGTGGTAG
Fig badnavirus 1 NC_017830.1 (1463) CAGTT--CATACATCTGGGAGTTT---------TGCAAGTAAGACTCCAGATACTGCATAGGG-CAGATGGAGGAAC-AATGGCCTTGGTAG
```

Section 19

```
                                     1657         1670      1680      1690      1700      1710      1720      1730      1748
                             (1657)
SARS-CoV-2 region 1.978-9.647nt (1594) TTGTGTTTTAAGCGGACACAATCTTGCTAAACACTGTCTTCATGTTGTCGGCCCAAATGTTAACAAAGGTGAAGACATTCAACTTCTTAAGA
Fig badnavirus 1 NC_017830.1 (1542) TC-----TTCAG------------AGATAACCGATGGCA--AGGAGATCAGTC--AATCTTCGCAATCTC--AGA-AGTTGACTTGACTAGA
```

Section 20

```
                                     1749       1760      1770      1780      1790      1800      1810      1820      1830      1840
                             (1749)
SARS-CoV-2 region 1.978-9.647nt (1686) GTGCTTATGAAAATTTTAATCAGCACGAAGTTCTACTTG-CACCATTATTATCAGCTGGTATTTTGGTGCTGACCCTATACAT-TCTTTAA
Fig badnavirus 1 NC_017830.1 (1610) G-GATC--GCAAATAGTATAC---------GTGATACCTGACACGATGATGATTATCGGTGACTTCTACAGA--AACGTTCAGATCTCTATCC
```

Section 21

```
                                     1841     1850      1860      1870      1880      1890      1900      1910      1920      1932
                             (1841)
SARS-CoV-2 region 1.978-9.647nt (1776) GAGTTTGTGTAGAT-ACTGTTCGCACAAATGTCTACTTAGCTGTCTTTGATAAAAATCTCTATGACAAACTTGTT-TCAAGCTTTTTGGAAA
Fig badnavirus 1 NC_017830.1 (1689) AGACCAGTGGATATGAGAATTGGCAGAA-TGGAGAGGCAAATTTGCT-GATCACAAGAGGCATGACGGGAAGGTTATCCAACACTCC--AAA
```

SARS-CoV-2 & Fig badnavirus 2

Section 22

```
                                        (1933) 1933 1940 1950 1960 1970 1980 1990 2000 2010 2024
SARS-CoV-2 region 1.978-9.647nt (1866) TGAAGAGTGAAAAGCAAGTTGAACAAAAGATCGCTGAGATT-CCTAAAGAGGAAGTTAAGCCATT-TATAACTGAAAGTAAACCTTCAGTTG
Fig badnavirus 1 NC_017830.1 (1777) TGTTG---GATTTGCTTACAGAATAAGCCATGTGACGGATTACCTAGCAAGCAGGGGAGTCCAAGCTTTGCCGGGAAG--AAGATACAGAT-
```

Section 23

```
                                        (2025) 2025 2030 2040 2050 2060 2070 2080 2090 2100 2116
SARS-CoV-2 region 1.978-9.647nt (1956) AACAGAGAAAACAAGATGATAAGAAAATCAAAGCTTGTGTTGAAGAAGTTACAA-CAACTCTGGAAGAAACTAAGTTCCTCACAGAAAACTT
Fig badnavirus 1 NC_017830.1 (1863) --CGGAGATGCTACGAGGA--AGAAACTGGATCATAAGGCAGCCACAGATCCAGGCGGCAATGATGCCAAGGAACGTGGAAACAAGGAACCT
```

Section 24

```
                                        (2117) 2117 2130 2140 2150 2160 2170 2180 2190 2208
SARS-CoV-2 region 1.978-9.647nt (2047) GTTACTTTATATTGACATTAATGGCAATCTTCATCCAGATTCTGCCACTCTTGTTAGTGA-CATTGACATCACTTTCTTAAAGAAAGATGCT
Fig badnavirus 1 NC_017830.1 (1951) GATAGATG----GAACAATCTCTGCAAGATC--TGCAGATTA---CACTCAGGCACCAGAACCACGACAACCCCAT-TACAATGAGCAGGAC
```

Section 25

```
                                        (2209) 2209 2220 2230 2240 2250 2260 2270 2280 2290 2300
SARS-CoV-2 region 1.978-9.647nt (2138) CCATATATAGTGGGTGATGTTGTTCAAGAGGGTGTTTTAACTGCTGTGGTTATACCTACTAAAAGGCTG-GTGGCACTACTGAAATGCTAG
Fig badnavirus 1 NC_017830.1 (2033) GAAGAAGTGGCGAGTGATGAGG---AAGAGCTGGCAGAAACTCAACAGC----ACATAGTAGCAATGCTGCGATTTCCGGATTATGTGG-AG
```

Section 26

```
                                        (2301) 2301 2310 2320 2330 2340 2350 2360 2370 2380 2392
SARS-CoV-2 region 1.978-9.647nt (2229) CGAAAGC--TTTGAGA-AAAGTGCCAACAGACAATTATATAACCACTTACCCGGGTCAGGGTTTAAATGGTTACACTGTAGAGGAGGCAAAG
Fig badnavirus 1 NC_017830.1 (2117) ATACAGGAGTTTAAGCCAAAGTACAAAGAG-CAATACGACAAGTACAAAAG-GGAGCAGGGCTG----GGATACCTG----GGAG---AAC
```

Section 27

```
                                        (2393) 2393 2400 2410 2420 2430 2440 2450 2460 2470 2484
SARS-CoV-2 region 1.978-9.647nt (2318) ACAGTGCTTAAAAAGTGTAAAAGTGCCTTTTACATTCTACCATCTATTATCTCTAATGAGAAGCAAGAAATTCTTGGAACTGTTTCTTGGAA
Fig badnavirus 1 NC_017830.1 (2196) CAAGCGGAAAGTATGACTACTAC-GTCCGCTACACTGCACCTCCAGCGA---CAACGCCGATAGAAGATATTCAGCCAACAGGCTGGGAGGA
```

Section 28

```
                                        (2485) 2485 2490 2500 2510 2520 2530 2540 2550 2560 2576
SARS-CoV-2 region 1.978-9.647nt (2410) TTTGCGAGAAATGCTTGCACATGCAGAAGAAACACGCAAATTAATGCCTGTCTGTGTGGAAACTAAAGCCATAGTTTCAACTATACAGCGTA
Fig badnavirus 1 NC_017830.1 (2284) GGAGCCTAAATGGATGATTCACCCGACGAGAAATCATTAGCAACAATTCGATTCGAGGAATACAAGGAAGAAGAATC-----TCAATCGGA
```

SARS-CoV-2 & Fig badnavirus 2

```
                                                                                       Section 29
                                   (2577) 2577         2590      2600      2610      2620      2630      2640      2650              2668
SARS-CoV-2 region 1.978-9.647nt    (2502) AATATAAGGGTATTAAAATACAAGAGGGTGTGGTTGATTATGG-TGCTAGATTTTACTTTTACACCAGTAAAACAACTGTAGCGTCACTTAT
Fig badnavirus 1 NC_017830.1       (2371) TGAAGAACATTATCAA----CCAGATGCTGAAGCAGAATTTGACTACCCGCTAAAAAGGATTCATTACCTAAATC-CTTTTTCGGAAGGTGG
                                                                                       Section 30
                                   (2669) 2669       2680      2690      2700      2710      2720      2730      2740      2750   2760
SARS-CoV-2 region 1.978-9.647nt    (2593) CAACACACTTAACGATCTAAATGAAACTCTTGTTACAATGCCACTTGGCTATGTAACACATGGCTTAAATTTGGAAGAAGCTGCTCGGTATA
Fig badnavirus 1 NC_017830.1       (2458) TGGGAAAGATGACCATTTAAAT-----TCT-GAAGAAATGG-ACTAC-CC--GCAAAGAATGAAGAAAATAGAAAAGTTACTA-TCCACCAG
                                                                                       Section 31
                                   (2761) 2761    2770      2780      2790      2800      2810      2820      2830      2840        2852
SARS-CoV-2 region 1.978-9.647nt    (2685) TGAGATCTCTCAAAGTGCCAGCTACAGTTTCTGTTTCTTCACCTGATGCTGTTACAGCGTATAATGGTTATCTTACTTCTTCTTCTAAAACA
Fig badnavirus 1 NC_017830.1       (2539) TGAGGTAACT---AGT-CCA--TACAG-------ACCACCAGAAGATGCAGCCATGGGAAGACCAAGTTATC---CACCAGCTAGGACAATA
                                                                                       Section 32
                                   (2853) 2853   2860      2870      2880      2890      2900      2910      2920      2930          2944
SARS-CoV-2 region 1.978-9.647nt    (2777) CCTGAAGAACATTTTATTGAAACCATCTCACTTGCTGGTTCCTATAAAGATTGGTCCTATTCTGGACAATCTACACAACTAGGTATAGAATT
Fig badnavirus 1 NC_017830.1       (2615) CCAGGAGGA---------GGGGCAAGCTCAAGCGCTGAA-CCTCCAAGGTTTGATGC-AGCCAGAACAAGATTTAAAGG--GGGATACAATG
                                                                                       Section 33
                                   (2945) 2945 2950      2960      2970      2980      2990      3000      3010      3020           3036
SARS-CoV-2 region 1.978-9.647nt    (2869) TCTTAAGAGAGGTGATAAAAGTGTATATTACACTAGTAATCCTACCACATTCCACCTAGATGGTGAAGTTATCACCTTTGACAATCTTAAGA
Fig badnavirus 1 NC_017830.1       (2694) ACG-AGATGTGGACACTACCATCAGCCCAACAGAAG-AATGGAGCCATGTTCGTAATACCAG--------AACAACTTGGGCTATTCAATGA
                                                                                       Section 34
                                   (3037) 3037         3050      3060      3070      3080      3090      3100      3110              3128
SARS-CoV-2 region 1.978-9.647nt    (2961) CACTTCTTTCTTTGAGAGAAGTGAGGACTATTAAGGTGTTTACAACAGTAGACAACATTAACCTCCACACGCAAGTTGTGGACATGTCAATG
Fig badnavirus 1 NC_017830.1       (2776) TGCTT-TCTCTAGATGGGAATCAGTCACTAAAAACCATGTGGCTACACAAGGTTTTACTGATACCCGAGATAAAATAAAGTACATGGAAAAT
                                                                                       Section 35
                                   (3129) 3129       3140      3150      3160      3170      3180      3190      3200      3210   3220
SARS-CoV-2 region 1.978-9.647nt    (3053) ACATATGGACAACAGTTTGGTCCAACTTATTTGGATGGAGCTGATGTTACTAAAATAAAACCTCATAATTCACATGAAGGTAAAACATTTTA
Fig badnavirus 1 NC_017830.1       (2867) CTACTCGGAGAAA----TTGAGAAGCTGATTTGGATACAGTGGAG---AATGACGTATG-CCACGGAATAC----GAAGCCTTAGTAGCCAT
```

```
Section 36
                                        (3221) 3221 3230 3240 3250 3260 3270 3280 3290 3300 3312
SARS-CoV-2 region 1.978-9.647nt (3145) TGTTTTACCTAATGATGACACTCTACGTGTTGAGGCTTTTGAGTACTACCACACAACTGATCCTAGTTTTCTGGGTAGGTACATGTCAGCAT
   Fig badnavirus 1 NC_017830.1 (2947) CGGAGAAGGCAGGGATGGTACTC-A---------------GAACATTCTCTCGCAAATGAAGAGTGTTTTCCTTAGAAGATCCGGCGCAG

Section 37
                                        (3313) 3313 3320 3330 3340 3350 3360 3370 3380 3390 3404
SARS-CoV-2 region 1.978-9.647nt (3237) TAAATCACACTAAAAAGTGGAAATACCCACAAGTTAATGGTTTAACT-TCTATTAAATGGGCAGATAACAACTGTTATCTTGCCACTGCATT
   Fig badnavirus 1 NC_017830.1 (3023) GGTTCGACAGTAATCCAGGATGAAGCCTACAGAGAGTTGGAAAGAATGTCTTGTACAGACGTTAAATACATCA-TTCCATTTCTAAATGAGT

Section 38
                                        (3405) 3405 3410 3420 3430 3440 3450 3460 3470 3480 3496
SARS-CoV-2 region 1.978-9.647nt (3328) GTTAACACTCCAACAAATAGAGTTGAAGTTTAATCCACCTGCTCTACAAGATGCTTATTACAGAGCAAGGGCTGG-TGAAGCTGCTA----A
   Fig badnavirus 1 NC_017830.1 (3114) ATATGCGTTTGGCAGCAAAGACGGGAAGGCTATTC-------TTAGGAGGAGAACTAT--CTGAG-AAGATCTGGATGAAGATGCCCGGAGA

Section 39
                                        (3497) 3497 3510 3520 3530 3540 3550 3560 3570 3588
SARS-CoV-2 region 1.978-9.647nt (3415) CTTTTGTGCACTTATCTTAGCCTACTGTAATAAGACAGTAGGTG-AGTTAGGTGATGTTAGAGAAACAATGAGTTACTTGTTTCAACATGCC
   Fig badnavirus 1 NC_017830.1 (3196) TTTAAGAAAAACAATAAAAGAAGACTTTGACAACAAATACCCAGGAGTCGTATCAGGGTGGTACCCAGGGTATTATTTGCTT--ACAAG--

Section 40
                                        (3589) 3589 3600 3610 3620 3630 3640 3650 3660 3670 3680
SARS-CoV-2 region 1.978-9.647nt (3506) AATTTAGATTCTTGCAAAAGAGTCTTGAACGTGGTGTGTAAAACTTGTGGACAACAGCAGACAACCCTTAAGGGTGTAGAAGCTGTTATGTA
   Fig badnavirus 1 NC_017830.1 (3284) --------TTCTTGGAGAAG------GAATGCAGAGATGCAGCATTCAAGAGGTCCCTAAAGAACCTGTCATTTTGTAGTTCAATTCCTATA

Section 41
                                        (3681) 3681 3690 3700 3710 3720 3730 3740 3750 3760 3772
SARS-CoV-2 region 1.978-9.647nt (3598) CATGGCACACTTTCTTATGAACAATTTAAGAAAGGTGTTCAGATACCTTGTACGTGTGGTAAACAAGCTACAAAATATCTAGTACAACAGG
   Fig badnavirus 1 NC_017830.1 (3362) CCAGG--ATACTAT------AAAAATACAGGCAAGAAGTATGGA------GTGCGAA-GATCCAAGACGTACAAAG--GCAAGCCTCACGAG

Section 42
                                        (3773) 3773 3780 3790 3800 3810 3820 3830 3840 3850 3864
SARS-CoV-2 region 1.978-9.647nt (3690) AGTCACCTTTTGTTATGATGTCAGCACCACCTGCTCAGTATGAACTTAAGCATGGTACATTTACTTGTGCTAGTGAGTACACTGGTAATTAC
   Fig badnavirus 1 NC_017830.1 (3437) AGCCATGCTAGGATTGAAAGCGAAAGCACCTGGTCCGCAACAAAGATGCAAGTGCTTCCTATGTGGACAAGAAGGTCATTTTGCAAGAA-
```

```
                                                                                       Section 43
                                  (3865) 3865 3870       3880       3890       3900       3910       3920       3930       3940       3956
SARS-CoV-2 region 1.978-9.647nt   (3782) CAGTGTGGTCACTATAAACATATAACTTCTAAAGAAACTTTGTATTGCATAGACGGTGCTTTACTTACAAAGTCCTCAGAATACAAAGGTCC
Fig badnavirus 1 NC_017830.1      (3528) -AGTGTCCTAATGATAAA---AGGAGCTCTAAA-------------------AGGATGGCTATGTTTGAAAATCTGGAAATACCAGAAG---
                                                                                       Section 44
                                  (3957) 3957          3970       3980       3990       4000       4010       4020       4030        4048
SARS-CoV-2 region 1.978-9.647nt   (3874) TATTACGGATGTTTTCTACAAAGAAAACAGTTACACAACAACCATAAAACCAGTTACTTATAAATTGGATGGTGTTGTTTGTACAGAAATTG
Fig badnavirus 1 NC_017830.1      (3594) -ATTGCG-ATATAGTCTCTGTAGAAGAAGG--AGAGGATATGAGTGATGCCATATACTCA-ATATCCGAAGGAGAAGATGGAGCAGA--T-G
                                                                                       Section 45
                                  (4049) 4049        4060       4070       4080       4090       4100       4110       4120       4130   4140
SARS-CoV-2 region 1.978-9.647nt   (3966) ACCCTAAGTTGGACAATTATTATAAGAAAGACAATTCTTATTTCACAGAGCAACCAATTGATCTTGTACCAAACCAACCATATCCAAACGCA
Fig badnavirus 1 NC_017830.1      (3678) AGCTGCAGAAAT-CACTCCAAACATTGGAAATTGTTCTTATGTTAG-GAGAAATTGATGGAG--GGTACCGGCCG---CAGATCAAGCTTCC
                                                                                       Section 46
                                  (4141) 4141     4150       4160       4170       4180       4190       4200       4210       4220        4232
SARS-CoV-2 region 1.978-9.647nt   (4058) AGCTTCGATAATTTTAAGTTTGTATGTGATAATATCAAATTTGCTGATGATTTAAACCAGTTAACTGGTTATAAGAAACCTGCTTCAAGAGA
Fig badnavirus 1 NC_017830.1      (3763) TGA--GGAGCAGATGAATTGTGTCCATAACTGGATACATAATGGGGATATCTTTGTACAAAGAGCTGTTCAT-----TGCAGCTTCTGCAGA
                                                                                       Section 47
                                  (4233) 4233   4240       4250       4260       4270       4280       4290       4300       4310        4324
SARS-CoV-2 region 1.978-9.647nt   (4150) GCTTAAAGTTACATTTTTCCCTGACTTAAATGGTGATGTGGTGGCTATTGA----TTATAAACACTACACACC---CTCTTTAAGAAAGGA
Fig badnavirus 1 NC_017830.1      (3848) AGGGAAAGCATAAAAAGGGCAAGAAT-ACATTGTCCTTTG-TGTCTCCTAACAGCTTGCAATCTGTGCGGACCTTACTACTTGAAGATAGAA
                                                                                       Section 48
                                  (4325) 4325 4330       4340       4350       4360       4370       4380       4390       4400        4416
SARS-CoV-2 region 1.978-9.647nt   (4235) GCTAAATTGTTACATAAACCTATTGTTTGGCATGTTAACAATGCAACTAATAAAGCCACGTATAAACCAAATACC-TGGTGTATACGTTGTC
Fig badnavirus 1 NC_017830.1      (3938) GTACCGGTGGAACCAGCACC-ATCAACCCCGATAAATCCGAGAAGAATTATTCAGGAGCAGCACAACTACATCGGATGGTGTG-AAGTAGAA
                                                                                       Section 49
                                  (4417) 4417          4430       4440       4450       4460       4470       4480       4490        4508
SARS-CoV-2 region 1.978-9.647nt   (4326) TTTGGAGCACAAAACCAGT---TGAAACATCAAATTCGTTTGATGTACTGAAGTCAGAGGACGCGCAGGGAATGGATAATCTT-GCCTGCGA
Fig badnavirus 1 NC_017830.1      (4028) ATAGAGAGACTAAACAAGGAAGTGAAGCACTACAAAGG---GCTATAC-GAAGCCTTACTAATGGA--GAAATCCTTAAGGAAAGACTACGA
```

```
                                                                                                  Section 50
                                   (4509) 4509       4520      4530      4540      4550      4560      4570      4580      4590     4600
SARS-CoV-2 region 1.978-9.647nt    (4414) AGATCTAAAACCAGTCTCTGAAGAAGTAGTGGAAAATCCTACCATACAGAAAGACGTTCTTGAGTGTAATGTGAAAACTACCGAAGTTGTAG
Fig badnavirus 1 NC_017830.1       (4114) GGAGCTGAAAGAAGGA----GATAAGGAGCGAAGAAAGGG--CATAATGATACAAGAACCAGA---AGAAG--TTAACTATCTGGGA-GAAG
                                                                                                  Section 51
                                   (4601) 4601     4610      4620      4630      4640      4650      4660      4670      4680       4692
SARS-CoV-2 region 1.978-9.647nt    (4506) GAGACATTATACTTAAACCAGCAA-ATAATAGTTTAA-AAATTACAGAAGAGGTTGGCCACACAGATCTAATGGCTGCTTATGTAGACAATT
Fig badnavirus 1 NC_017830.1       (4194) ATGAGAAGATTCTTGCAGCAGGAACAGAGGAAAAGAAGAAATTCGTCAAGAA-TATGCTTTATAACTTTGATAT-TGAATTTG-AGATTCCA
                                                                                                  Section 52
                                   (4693) 4693   4700      4710      4720      4730      4740      4750      4760      4770         4784
SARS-CoV-2 region 1.978-9.647nt    (4596) CTAGTCTTACTATTAAGAAACCTAATGAATTATCTAGAGTATTAGGTTTGAAAACCCTTGCTACTCATGGTTTAGCTGCTGTTAATAGTGTC
Fig badnavirus 1 NC_017830.1       (4283) GATGTCTCAAGATTCAGA---GTAAGAGCAATTCTTGATACAGGAGCTTCAACATGCTG--TATCAATGAAGGAGCAGTTCCAAA-AGAAGC
                                                                                                  Section 53
                                   (4785) 4785 4790      4800      4810      4820      4830      4840      4850      4860           4876
SARS-CoV-2 region 1.978-9.647nt    (4688) CCTTGGGATACTATAGCTAATTATGCTAAGCCTTTTCTTAACAAAGTTGTTAGTACAACTACTAACATAGTTACACGGTGTTTAAACCGTGT
Fig badnavirus 1 NC_017830.1       (4369) CCTC--GAAGAAAGCCCTTATGAAGTTCAGCTGAATGGGGTCAACTCTGTGCAGAAAACAAAGAAGA-AGCTCAAGTACGGGAGAATGATGA
                                                                                                  Section 54
                                   (4877) 4877         4890      4900      4910      4920      4930      4940      4950             4968
SARS-CoV-2 region 1.978-9.647nt    (4780) TTGTACTAATTATATGCCTTATTTCTTTACTTTATTGCTACAATTGTGTACTTTTACTAGAAGTACAAATTCTAGA--ATTAAAGCATCTAT
Fig badnavirus 1 NC_017830.1       (4458) TCGGATGAATACGTTCAGAATCCCTTT------TACATACAGTCTGCCACTGGTCATCGGAG-ATAACATCCAGATGATTGTAGGTTGTA-
                                                                                                  Section 55
                                   (4969) 4969       4980      4990      5000      5010      5020      5030      5040      5050     5060
SARS-CoV-2 region 1.978-9.647nt    (4870) GCCGACTACTATAGCAAAGAATACTGTTAAGAGTGTCGGTAAATTTTGTCTAGAGGCTTCATTTAATTATTTGAAGTCACCTAATTTTTCTA
Fig badnavirus 1 NC_017830.1       (4542) -------ACTTCATCAGAGCAATGTATGGAG-GAGTGAGAATAGAAGGGAATGAGGTAACCTTTTACAAGA--ACCTCACACGGATTAGCAC
                                                                                                  Section 56
                                   (5061) 5061     5070      5080      5090      5100      5110      5120      5130      5140       5152
SARS-CoV-2 region 1.978-9.647nt    (4962) AACTGATAAATATTATAATTTGGTTTTTACTATTAAGT-GTTTGCCTAGGTTCTTTAATCTACTCAACCGCTGCTTTAGGTGTTTT--AATG
Fig badnavirus 1 NC_017830.1       (4624) ATCCCAGAAG--TCTCGGTGAATTCCCTGGATGAAGAAGTTTGCGAAG----AAGAATATTTGCAGGTGCAGGAGATGGTGATCTGCAACA
```

Section 57

(5153) 5153 5160 5170 5180 5190 5200 5210 5220 5230 5244
SARS-CoV-2 region 1.978-9.647nt (5051) TCTAATTTAGGCATGCCTTCTTACTGTACTGGTTACAGAGAAGGCTATTTGAACTCTACTAAT----GTCACTATTGCAACCTACTGTACTG
Fig badnavirus 1 NC_017830.1 (4710) TTGGAGAAAGCCGAAAATCATTTCTTAAGAAGTTCAGGCCTTTGCTGGAAGAACTTAAGGAAGCAGGGTTTATAGGAGAAAATCCATTACAG

Section 58

(5245) 5245 5250 5260 5270 5280 5290 5300 5310 5320 5336
SARS-CoV-2 region 1.978-9.647nt (5139) GTTCTATACCTTGTAGTGTTGTCTTAGTGGTTTAGATTCTTTAGACACCTATCCTTCTTTAGAAACTATACAAATTACCATTTCATCTTTT
Fig badnavirus 1 NC_017830.1 (4802) CATTGGAAGAAAAATGGGATTTTATGCCAACTTGATATCAAAAATCCTGATTTCATAATT--GAAGATAGGCCACTGAAGAATTTAACGCCA

Section 59

(5337) 5337 5350 5360 5370 5380 5390 5400 5410 5428
SARS-CoV-2 region 1.978-9.647nt (5231) AAATGGGATTTAACTGCTTTTGGCTTAGTTGCAGAGTGGTTTTTGGCATATATTCTTTTCACTAGGTTTTTCTATGTACTTGGATTGGCTGC
Fig badnavirus 1 NC_017830.1 (4892) CAGATGAAGG--AGTCCTTCAAGAAGCATATCAA----GGTTTTGCTGGATTTAGGAGTCATCAGG-----GCAAGTAAAAGCCGCCACAG-

Section 60

(5429) 5429 5440 5450 5460 5470 5480 5490 5500 5510 5520
SARS-CoV-2 region 1.978-9.647nt (5323) AATCATGCAATTGTTTTTCAGCTATTTTGCAGTACATTTTATTAGTAATTCTTGGCTTATGTGGTTAATAATTAATCTTGTACAAATGGCCC
Fig badnavirus 1 NC_017830.1 (4972) AACGACGGCAATGCTAGTCAACTCAGGAACA--ACGGTGGATCCG-AAAACAGGG---AAGGAGATCAAAAGGAAG---GAAAGAATGGTCT

Section 61

(5521) 5521 5530 5540 5550 5560 5570 5580 5590 5600 5612
SARS-CoV-2 region 1.978-9.647nt (5415) CGATTTCAGCTATGGTTAGAATGTACATC--TTCTTTGCATCATTTTATTATGTATGGAAAAGTTATGTGCATGTTGTAGACGGTTGTAATT
Fig badnavirus 1 NC_017830.1 (5055) TCAACT-ACAAAAGGTT-GAATG-ACATCACTCATAAGGATCAGTATAGTCTTCCAGGAATAAATACG---ATCCTCAAGAAGGTTGGAAAT

Section 62

(5613) 5613 5620 5630 5640 5650 5660 5670 5680 5690 5704
SARS-CoV-2 region 1.978-9.647nt (5505) CATCAACTTGTATGATGTGTTACAAACGTAATAGAGCAACAAGAGTCGAATGTACAACTATTGTTAATGGTGTTAGAAGGTCCTTTTATGTC
Fig badnavirus 1 NC_017830.1 (5141) -AGCAA---G----ATATTCAGCAAGTTTGATCTA-----AAGAG-CGGATTT-CACCAAGTTGCTATGCA---------TCCT-----GAC

Section 63

(5705) 5705 5710 5720 5730 5740 5750 5760 5770 5780 5796
SARS-CoV-2 region 1.978-9.647nt (5597) TATGCTAATGGAGGTAAAGGCTTTTGCAAACTACACAATTGGAATTGTGTTAATTGTGATACATTCTGTGCTGGTAGTACATTTATTAGTGA
Fig badnavirus 1 NC_017830.1 (5204) T---CAATAGAATGGACAGCTTTTGGGTCCCAGACGGATTGTAT-GAGTGGCTTGTCATGCCATTCGGACTCAAAA-ACGCTCCTTC----

```
                                                                                     Section 64
                                       (5797) 5797         5810      5820      5830      5840      5850      5860      5870                 5888
SARS-CoV-2 region 1.978-9.647nt (5689) TGAAGTTGCGAGAGACTTGTCACTACAGTTTAAAAGACCAATAAATCCTACTGACCAGTCTTCTTACATCGTTGATAGTGTTACAGTGAAGA
   Fig badnavirus 1 NC_017830.1 (5287) TGTTTTTCAAAGAAAGATGGATGAATGCTTCAAAGGGACAGAAGATTTTATTG--CAGTC----TACATTGACGATATTCTT----------
                                                                                     Section 65
                                       (5889) 5889       5900      5910      5920      5930      5940      5950      5960      5970       5980
SARS-CoV-2 region 1.978-9.647nt (5781) ATGGTTCCATCCATCTTTACTTTGATAAAGCTGGTCAAAAGACTTATGAAAGACATTCTCTCTCATTTTGTTAACTTAGACAAC-CTGAG
   Fig badnavirus 1 NC_017830.1 (5363) --GTTTTC-TC-----TGAAAACGAGAAAGATCATGCGAAG-CACTTGAAGGCCATGTTGGAGATCTGCAAAAGAAATGGGTTAGTTCTAAG
                                                                                     Section 66
                                       (5981) 5981     5990      6000      6010      6020      6030      6040      6050      6060          6072
SARS-CoV-2 region 1.978-9.647nt (5872) AGCTAATAACACTAAAGGTTCATTGCCTATTAATGTTATAGTTTTTGATGGTAAATCAAAATGTGAAGAATCATCTGCAAAATCAGCGTCTG
   Fig badnavirus 1 NC_017830.1 (5446) CCCATCTAAAA-TGAAGATAGCAGTCCAAGAGGTGGAATT-TCTTGGAGCCCAAATCGGAAATCAAAGGATC-----CGACTTCAGCCACAT
                                                                                     Section 67
                                       (6073) 6073   6080      6090      6100      6110      6120      6130      6140      6150            6164
SARS-CoV-2 region 1.978-9.647nt (5964) TTTACTACAGTCAGCTTATGTGTCAACCTATACTGTTACTAGATCAGGCATTAGTGTCTGATGTTGGTGATAGTGCGGAAGTTGCAGTTAAA
   Fig badnavirus 1 NC_017830.1 (5531) GTTA-TAAAGAAGATCGTTGAGTTCAATGAGGCTG--AGTTGAAGGAGAAAAAGGGCATGA----GATCATGGTTAGGAATTCTCAACTA--
                                                                                     Section 68
                                       (6165) 6165 6170      6180      6190      6200      6210      6220      6230      6240              6256
SARS-CoV-2 region 1.978-9.647nt (6056) ATGTTTGATGCTTACGTTAATACGTTTTCATCAACTTTTAA-CGTAC-CAATGGAAAAACTCAAAACACTAGTTGCAACTGCAGAAGCTGAA
   Fig badnavirus 1 NC_017830.1 (5614) -TGCTCGC-GCATACATTCCTAACTTGGGC-CGCCTTTTAAGCCCACTCTATGCCAAAAC----AAGTCCAACTGGAGATA-AGAGGATGAA
                                                                                     Section 69
                                       (6257) 6257         6270      6280      6290      6300      6310      6320      6330                 6348
SARS-CoV-2 region 1.978-9.647nt (6146) CTTGCAAAGAATGTGTCCTTAGACAATGTCTTATCTACTTTTATTTCAGCAGCTCGGCAAGGGTTTGTTGATTCAGATGTAGAAACTAAAGA
   Fig badnavirus 1 NC_017830.1 (5698) --TGCCCAGGATTGG----------AAGCTCGTGGCCCAAATAAAAGAAGAAGTCCAGAA---GCTGCCAGATCTGGAGCTACCACCCGAGGA
                                                                                     Section 70
                                       (6349) 6349       6360      6370      6380      6390      6400      6410      6420      6430       6440
SARS-CoV-2 region 1.978-9.647nt (6238) TGTTGTTGAATGTCTTAAATTGTCACATCAATCTGACATAGAAGTTACTGGCGATAGTTGTAATAACTATATGCTCACCTATAACAAAGTTG
   Fig badnavirus 1 NC_017830.1 (5776) T--TGTTTCATC-----A-TTCTTGAAACAGACGGCTGTATGACTGGTTGGGGAGGAATTTG-TAAATGGAAGCCCAAAGAACGACCCAA
```

SARS-CoV-2 & Fig badnavirus 2

Section 71

```
                                       (6441) 6441     6450      6460      6470      6480      6490      6500      6510      6520      6532
SARS-CoV-2 region 1.978-9.647nt (6330) AAAACATG-ACACCCCGTGACCTTGGTGCTTGTATTGACTGTAGTGCGC----GTCAT--ATTAATGCGCAGGTAGCAAAAGTCAC-AACA
  Fig badnavirus 1 NC_017830.1 (5859) GAAGGATGGAGAAAGTTTGCGCCTA-TGCTAGTGGGAAGTTTAGTCCAATCAAGTCAACCATTGATGCCGAAATC-CATGCAGTCATGAACA
```

Section 72

```
                                       (6533) 6533   6540      6550      6560      6570      6580      6590      6600      6610      6624
SARS-CoV-2 region 1.978-9.647nt (6414) TTGCTTTGATATGGAACGTTAAAGATTTCATGTCATTGTCTGAACAACTACGAAACAAATACGTAGTGCTGCTAAAAAGAATAACTTACCT
  Fig badnavirus 1 NC_017830.1 (5949) ---CTCTG----GAAAAATTAAAGATTTATTATCTTGATAAAAAGGAGTT-GATCATCAGAACTGACTGCC---AAGCT--ATCATCAGCTT
```

Section 73

```
                                       (6625) 6625 6630      6640      6650      6660      6670      6680      6690      6700      6716
SARS-CoV-2 region 1.978-9.647nt (6506) TTTAAGTTGACATGTGCAACTACTAGACAAGTTGTTAATGTTGTAACAACAAAGATAGCACTTAAGGGTGGTA--AAATTGTTAATAATTGG
  Fig badnavirus 1 NC_017830.1 (6028) CTTCAAT--AAATCCGCC----CTAAATAAACCCTCTAGAGTG-----AGATGGGTAGCCTTTACGGATTTTATCACAGGGATAGGAATTAA
```

Section 74

```
                                       (6717) 6717         6730      6740      6750      6760      6770      6780      6790         6808
SARS-CoV-2 region 1.978-9.647nt (6596) T-TGAAGCAGTTAATTAAAGTTACACTTGTGTTCCTTTTTGTTGCTGCTATTTTCTATTTAATAACACCTGTTCATGTCATGTCTAAACATA
  Fig badnavirus 1 NC_017830.1 (6109) TGTGGAGTTCGAGCATATAGATGGAAGGGATAATGTGCTTGCTGATAGCCTTTCAAGGTTAATTA-ACAGTTTCTT----T--CTTACAGAA
```

Section 75

```
                                       (6809) 6809       6820      6830      6840      6850      6860      6870      6880      6890   6900
SARS-CoV-2 region 1.978-9.647nt (6687) CTGACTTTTCAAGTGAAATCATAGGATACAAGGCTATTGATGGTGGTGTCACTCGTGACATAGCATCTA-CAGATACTTGTTTTGCTAACAA
  Fig badnavirus 1 NC_017830.1 (6194) TGGAGTCTGAAAAAGGAGGAAGCAGTAGCAGAGCTAGAGAAGCTTATGGAAGACAA-ACAATGCAGGAAGCAACTCCTACCATTAATCAACA
```

Section 76

```
                                       (6901) 6901     6910      6920      6930      6940      6950      6960      6970      6980      6992
SARS-CoV-2 region 1.978-9.647nt (6778) ACATGCTGATTTTGACACATGGTTTAGCCAGCGTGGTGGTAGTTATACTAATGACAAAGCTTGCCATTGATTGCTGCAGTCATAACAAGAG
  Fig badnavirus 1 NC_017830.1 (6285) AAATGACGAACTGTTTCAACAATACCAGAACCTTGCTG---------AGGAAAGAGAAAGGAT--CCTTCGAT--CAGGAGGAATATCTTTTG
```

Section 77

```
                                       (6993) 6993   7000      7010      7020      7030      7040      7050      7060      7070      7084
SARS-CoV-2 region 1.978-9.647nt (6870) AAGTGGGTTTTGTCGTGCCTGGTTTGC-CTGGCACGATATTACGCACAACTAATGGTGACTTTTTGCATTTCTTACCTAGAGTTTTTAGTGC
  Fig badnavirus 1 NC_017830.1 (6365) CCAGAGACAATGAATTCAGAAAACAACTCAGGGAGGTT-----GAAGAAGAATCTGCAAGAAGAGCCATTAAAGCCCTGGAACAATACAGGC
```

```
                                                                                                     Section 78
                                   (7085) 7085 7090      7100      7110      7120      7130      7140      7150      7160         7176
SARS-CoV-2 region 1.978-9.647nt (6961) AGTTGGTAACATCTGTTACACACCATCAAAACTTATAGAGTACACTGACTTTGCAACATCAGCTTGTGTTT---TGGCTGCTGAATGT-ACA
  Fig badnavirus 1 NC_017830.1 (6452) AGATTCACACACTTAAAACAGCCCAATGTGCATCATGGAGTTCACCCGGAAGGGATGGGAATTATTGGTCTGATTGCTTGCCAAGCGTTAGG
                                                                                                     Section 79
                                   (7177) 7177        7190      7200      7210      7220      7230      7240      7250           7268
SARS-CoV-2 region 1.978-9.647nt (7049) ATTTTTAAAGATGCTTCTGGTAAGCCAGTACCATATTGTTATGATACCAATG-TACTAGAAGGTTCTGTTGCTTATGAAAGTTTACGCCCTG
  Fig badnavirus 1 NC_017830.1 (6544) CGATATGACGCAAGATTAGAAAAGGTCCTACAAGACTTGAAGGACGCGTCAGCTGACAGCCTAAAGTTCCGCATTTGAAGCATGATGGGCCC
                                                                                                     Section 80
                                   (7269) 7269       7280      7290      7300      7310      7320      7330      7340      7350      7360
SARS-CoV-2 region 1.978-9.647nt (7140) ACACACGTTATGTGCTCATGGATGGCTCTATTATTCAATTTCCTAACACCTACCTTGAAGGTTCTGTTAGAGTGGTAACAACTTTTGATTCT
  Fig badnavirus 1 NC_017830.1 (6636) AGAAAAGTG----GTTGAGGCGTCTCACTAT-AATCGATGCCCTTATCTTTTCCTTTGTCATTTTGTAATAATGGAATCGTCTTTTTGACCC
                                                                                                     Section 81
                                   (7361) 7361     7370      7380      7390      7400      7410      7420      7430      7440        7452
SARS-CoV-2 region 1.978-9.647nt (7232) GAGTACTGTAGGCACGGCACTTGTGAAA-GATCAGAAGCTGGTGTTTGTGTATCTACTAGTGGTAGATGG---GTACTTAACAATGATTATT
  Fig badnavirus 1 NC_017830.1 (6723) AAAGGCTGCCGTC------CATGTGCAGTGATTC----CTTTAGTTTGCGTGTTTATCCGCTTACACGGATTGTCCTTTGTAACCACTTTC
                                                                                                     Section 82
                                   (7453) 7453   7460      7470      7480      7490      7500      7510      7520      7530          7544
SARS-CoV-2 region 1.978-9.647nt (7320) ACAGATCTTTACCAGGAGTTTTCTGTGGTGTAGATGCTGTAAATT-TACTTACTAATATGTTTACACCACTAATTCAACCTATTGGTGCTTT
  Fig badnavirus 1 NC_017830.1 (6805) ACGTGGTTTTGC------TTTTCTTTAGTGCGCCTCTTTTGCGCCCTTGTTTTTAGCGTTAGTACGCTTTGAGTCAAGTTTCCTTATCATTT
                                                                                                     Section 83
                                   (7545) 7545 7550      7560      7570      7580      7590      7600      7610      7620           7636
SARS-CoV-2 region 1.978-9.647nt (7411) GGACATATCAGCATCTATAGTAGCTGGTGGTATTGTAGCTATCGTAGTAACATGCCTTGCCTACTATTTTATGAGGTTTAGAAGAGCTTTTG
  Fig badnavirus 1 NC_017830.1 (6891) GGGAATCTTTTGAGCTGTCGATG--GGGCCCAATGAAGCACCCG-----AGCTCTTTTAATTCCTATATAAGGAGCTTTAGTTTCG-TTGCA
                                                                                                     Section 84
                                   (7637) 7637        7650      7660      7670      7680      7690      7700      7710           7728
SARS-CoV-2 region 1.978-9.647nt (7503) GTGAATACAGTCATGTAGTTGCCTTTAATACTTTACTATTCCTTATGTCATTCACTGTACTCTGTTTAACACCAGTTTACTCATTCTTACCT
  Fig badnavirus 1 NC_017830.1 (6975) AGGCACACAACAATCAAGCAACCCTTGAGCCTT-ACTTCGAAAAGAGTCTGTAAAAGTTTAGAGTCTGTAATCAGTGTG--AAGTTTGAAGA
```

Section 85

```
                                     7729       7740      7750      7760      7770      7780      7790           7805
                             (7729)
SARS-CoV-2 region 1.978-9.647nt (7595) GGTGTTTATTCTGTTATTTACTTGTACTTGACATTT-TATCTTACTAATGATGTTTCTTTTTTAGCACATATTCAGT
 Fig badnavirus 1 NC_017830.1 (7064) AATAAATTAGAGGTAATTCAGTTTAAAATCTCTTTTATTTCTTGTTTTCGATCCATTTTATTTGGGGGACACACAAA
```
