# Supplementary material for: Patch type nucleotide sequence identities between genomes from many different species facilitate illegitimate recombination
Source: Sci Rep. 2026 Mar 30;16:10524. doi: 10.1038/s41598-026-44124-0 (PMC13035915; doi:10.1038/s41598-026-44124-0)
Supplement: Supplementary file 10 — Supplementary Material 10 [file 41598_2026_44124_MOESM10_ESM.pdf]

SARS-CoV-2 & Fig badnavirus

```
                                                     Section 1
                                           (1) 1        10        20        30        40        50        60        70           83
SARS-CoV-2 Reference Genome NC_045512.2    (1) ATTAAAGGTTTATACCTTCCCAGGTAACAAACCAACCAACTTTCGATCTCTTGTAGATCTGTTCTCTAAACGAACTTTAAAAT
     Fig badnavirus 1 NC_017830.1          (1) -----------------------------------------------------------------------------------
                                                     Section 2
                                          (84) 84    90        100       110       120       130       140       150            166
SARS-CoV-2 Reference Genome NC_045512.2   (84) CTGTGTGGCTGTCACTCGGCTGCATGCTTAGTGCACTCACGCAGTATAATTAATAACTAATTACTGTCGTTGACAGGACACGA
     Fig badnavirus 1 NC_017830.1          (1) -----------------------------------------------------------------------------------
                                                     Section 3
                                         (167) 167          180       190       200       210       220       230               249
SARS-CoV-2 Reference Genome NC_045512.2  (167) GTAACTCGTCTATCTTCTGCAGGCTGCTTACGGTTTCGTCCGTGTTGCAGCCGATCATCAGCACATCTAGGTTTCGTCCGGGT
     Fig badnavirus 1 NC_017830.1          (1) -----------------------------------------------------------------------------------
                                                     Section 4
                                         (250) 250       260       270       280       290       300       310       320          332
SARS-CoV-2 Reference Genome NC_045512.2  (250) GTGACCGAAAGGTAAGATGGAGAGCCTTGTCCCTGGTTTCAACGAGAAAACACACGTCCAACTCAGTTTGCCTGTTTTACAGG
     Fig badnavirus 1 NC_017830.1          (1) -----------------------------------------------------------------------------------
                                                     Section 5
                                         (333) 333    340       350       360       370       380       390       400            415
SARS-CoV-2 Reference Genome NC_045512.2  (333) TTCGCGACGTGCTCGTACGTGGCTTTGGAGACTCCGTGGAGGAGGTCTTATCAGAGGCACGTCAACATCTTAAAGATGGCACT
     Fig badnavirus 1 NC_017830.1          (1) -----------------------------------------------------------------------------------
                                                     Section 6
                                         (416) 416           430       440       450       460       470       480              498
SARS-CoV-2 Reference Genome NC_045512.2  (416) TGTGGCTTAGTAGAAGTTGAAAAAGGCGTTTTGCCTCAACTTGAACAGCCCTATGTGTTCATCAAACGTTCGGATGCTCGAAC
     Fig badnavirus 1 NC_017830.1          (1) -----------------------------------------------------------------------------------
                                                     Section 7
                                         (499) 499         510       520       530       540       550       560       570        581
SARS-CoV-2 Reference Genome NC_045512.2  (499) TGCACCTCATGGTCATGTTATGGTTGAGCTGGTAGCAGAACTCGAAGGCATTCAGTACGGTCGTAGTGGTGAGACACTTGGTG
     Fig badnavirus 1 NC_017830.1          (1) -----------------------------------------------------------------------------------
```

Section 8

```
                                               (582) 582     590             600             610             620             630             640             650                 664
SARS-CoV-2 Reference Genome NC_045512.2        (582) TCCTTGTCCCTCATGTGGGCGAAATACCAGTGGCTTACCGCAAGGTTCTTCTTCGTAAGAACGGTAATAAAGGAGCTGGTGGC
Fig badnavirus 1 NC_017830.1                     (1) -----------------------------------------------------------------------------------
```

Section 9

```
                                               (665) 665  670             680             690             700             710             720             730                     747
SARS-CoV-2 Reference Genome NC_045512.2        (665) CATAGTTACGGCGCCGATCTAAAGTCATTTGACTTAGGCGACGAGCTTGGCACTGATCCTTATGAAGATTTTCAAGAAAACTG
Fig badnavirus 1 NC_017830.1                     (1) -----------------------------------------------------------------------------------
```

Section 10

```
                                               (748) 748         760             770             780             790             800             810             820         830
SARS-CoV-2 Reference Genome NC_045512.2        (748) GAACACTAAACATAGCAGTGGTGTTACCCGTGAACTCATGCGTGAGCTTAACGGAGGGGCATACACTCGCTATGTCGATAACA
Fig badnavirus 1 NC_017830.1                     (1) -----------------------------------------------------------------------------------
```

Section 11

```
                                               (831) 831      840             850             860             870             880             890             900             913
SARS-CoV-2 Reference Genome NC_045512.2        (831) ACTTCTGTGGCCCTGATGGCTACCCTCTTGAGTGCATTAAAGACCTTCTAGCACGTGCTGGTAAAGCTTCATGCACTTTGTCC
Fig badnavirus 1 NC_017830.1                     (1) -----------------------------------------------------------------------------------
```

Section 12

```
                                               (914) 914   920             930             940             950             960             970             980                 996
SARS-CoV-2 Reference Genome NC_045512.2        (914) GAACAACTGGACTTTATTGACACTAAGAGGGGTGTATACTGCTGCCGTGAACATGAGCATGAAATTGCTTGGTACACGGAACG
Fig badnavirus 1 NC_017830.1                     (1) -----------------------------------------------------------------------------------
```

Section 13

```
                                               (997) 997          1010            1020            1030            1040            1050            1060                   1079
SARS-CoV-2 Reference Genome NC_045512.2        (997) TTCTGAAAAGAGCTATGAATTGCAGACACCTTTTGAAATTAAATTGGCAAAGAAATTTGACACCTTCAATGGGGAATGTCCAA
Fig badnavirus 1 NC_017830.1                     (1) -----------------------------------------------------------------------------------
```

Section 14

```
                                              (1080) 1080      1090            1100            1110            1120            1130            1140            1150          1162
SARS-CoV-2 Reference Genome NC_045512.2       (1080) ATTTTGTATTTCCCTTAAATTCCATAATCAAGACTATTCAACCAAGGGTTGAAAAGAAAAAGCTTGATGGCTTTATGGGTAGA
Fig badnavirus 1 NC_017830.1                     (1) -----------------------------------------------------------------------------------
```

```
                                                                                                    Section 15
                                        (1163) 1163   1170      1180      1190      1200      1210      1220      1230           1245
SARS-CoV-2 Reference Genome NC_045512.2 (1163) ATTCGATCTGTCTATCCAGTTGCGTCACCAAATGAATGCAACCAAATGTGCCTTTCAACTCTCATGAAGTGTGATCATTGTGG
          Fig badnavirus 1 NC_017830.1     (1) -----------------------------------------------------------------------------------
                                                                                                    Section 16
                                        (1246) 1246          1260      1270      1280      1290      1300      1310               1328
SARS-CoV-2 Reference Genome NC_045512.2 (1246) TGAAACTTCATGGCAGACGGGCGATTTTGTTAAAGCCACTTGCGAATTTTGTGGCACTGAGAATTTGACTAAAGAAGGTGCCA
          Fig badnavirus 1 NC_017830.1     (1) -----------------------------------------------------------------------------------
                                                                                                    Section 17
                                        (1329) 1329       1340      1350      1360      1370      1380      1390      1400        1411
SARS-CoV-2 Reference Genome NC_045512.2 (1329) CTACTTGTGGTTACTTACCCCAAAATGCTGTTGTTAAAATTTATTGTCCAGCATGTCACAATTCAGAAGTAGGACCTGAGCAT
          Fig badnavirus 1 NC_017830.1     (1) -----------------------------------------------------------------------------------
                                                                                                    Section 18
                                        (1412) 1412    1420      1430      1440      1450      1460      1470      1480           1494
SARS-CoV-2 Reference Genome NC_045512.2 (1412) AGTCTTGCCGAATACCATAATGAATCTGGCTTGAAAACCATTCTTCGTAAGGGTGGTCGCACTATTGCCTTTGGAGGCTGTGT
          Fig badnavirus 1 NC_017830.1     (1) -----------------------------------------------------------------------------------
                                                                                                    Section 19
                                        (1495) 1495 1500      1510      1520      1530      1540      1550      1560                1577
SARS-CoV-2 Reference Genome NC_045512.2 (1495) GTTCTCTTATGTTGGTTGCCATAACAAGTGTGCCTATTGGGTTCCACGTGCTAGCGCTAACATAGGTTGTAACCATACAGGTG
          Fig badnavirus 1 NC_017830.1     (1) -----------------------------------------------------------------------------------
                                                                                                    Section 20
                                        (1578) 1578        1590      1600      1610      1620      1630      1640      1650    1660
SARS-CoV-2 Reference Genome NC_045512.2 (1578) TTGTTGGAGAAGGTTCCGAAGGTCTTAATGACAACCTTCTTGAAATACTCCAAAAAGAGAAAGTCAACATCAATATTGTTGGT
          Fig badnavirus 1 NC_017830.1     (1) -----------------------------------------------------------------------------------
                                                                                                    Section 21
                                        (1661) 1661     1670      1680      1690      1700      1710      1720      1730         1743
SARS-CoV-2 Reference Genome NC_045512.2 (1661) GACTTTAAACTTAATGAAGAGATCGCCATTATTTTGGCATCTTTTTCTGCTTCCACAAGTGCTTTTGTGGAAACTGTGAAAGG
          Fig badnavirus 1 NC_017830.1     (1) -----------------------------------------------------------------------------------
```

Section 22

```
                                        (1744) 1744 1750      1760      1770      1780      1790      1800      1810           1826
SARS-CoV-2 Reference Genome NC_045512.2 (1744) TTTGGATTATAAAGCATTCAAACAAATTGTTGAATCCTGTGGTAATTTTAAAGTTACAAAAGGAAAAGCTAAAAAAGGTGCCT
        Fig badnavirus 1 NC_017830.1    (1)    -----------------------------------------------------------------------------------
```

Section 23

```
                                        (1827) 1827         1840      1850      1860      1870      1880      1890           1909
SARS-CoV-2 Reference Genome NC_045512.2 (1827) GGAATATTGGTGAACAGAAATCAATACTGAGTCCTCTTTATGCATTTGCATCAGAGGCTGCTCGTGTTGTACGATCAATTTTC
        Fig badnavirus 1 NC_017830.1    (1)    -----------------------------------------------------------------------------------
```

Section 24

```
                                        (1910) 1910      1920      1930      1940      1950      1960      1970      1980        1992
SARS-CoV-2 Reference Genome NC_045512.2 (1910) TCCCGCACTCTTGAAACTGCTCAAAATTCTGTGCGTGTTTACAGAAGGCCGCTATAACAATACTAGATGGAATTTCACAG-T
        Fig badnavirus 1 NC_017830.1    (1)    --------------------------------------------------------------------TGGTA--TCAGAGCT
```

Section 25

```
                                        (1993) 1993   2000      2010      2020      2030      2040      2050      2060           2075
SARS-CoV-2 Reference Genome NC_045512.2 (1992) ATTCACTGAGACTCATTGATGCTATGATGTTCACATCTGATTTGGCTACTAACAATCTAGTTGTAATGGCCTACATTACAGGT
        Fig badnavirus 1 NC_017830.1    (14)   AGTTA-TGAGTGAGTAGGTTCTTATAAGGT----ATTCGGGTAAACAGGGAAAGATTTATTATGCCTAGATTGATTTGATAAT
```

Section 26

```
                                        (2076) 2076          2090      2100      2110      2120      2130      2140           2158
SARS-CoV-2 Reference Genome NC_045512.2 (2075) GGTGTTGTTCAGTTGACT---TCGCAGTGGCTAACT-----AACATCTTTGGCACTGTTTATGAAAAACTCA--AACCCGTCC
        Fig badnavirus 1 NC_017830.1    (92)   CTTGTTTCTCAGATCTCTGAGTTGTAGTATCTAAAAGGAAAAAGTCCTTGGGCACTATATCTGGTTAAGGCAGGAGGCCGT--
```

Section 27

```
                                        (2159) 2159        2170      2180      2190      2200      2210      2220      2230      2241
SARS-CoV-2 Reference Genome NC_045512.2 (2148) TTGATTGGCTTGAAGAGAAGTTTAAGGAAGGTGTAGAGTTTCTTAGAGACGGTTGGAAATTGTTAAATTTATCTCAACCTGT
        Fig badnavirus 1 NC_017830.1    (173)  GTGATACCAGTGAA----ACCCTGAGGTGATCCTGATGGATATGAGTCATGTCTGAGAAATGGGAAAGGTCAATTCAGGAT-T
```

Section 28

```
                                        (2242) 2242    2250      2260      2270      2280      2290      2300      2310           2324
SARS-CoV-2 Reference Genome NC_045512.2 (2231) GCTTGTGAAATTGTCGGTGGACAAATTGTCACCTGTGCAAAGGAAATTAAGGAGAGTGTTCAGACATTCTTTAAGCTTGTAAA
        Fig badnavirus 1 NC_017830.1    (251)  GGTATAATAATTCTAG-------AACTGCGAACCTTG--------AATACCTTGATTTAGCAGAAAGGAAAAACC-----AA
```

Section 29

```
                                           (2325) 2325 ... 2407
SARS-CoV-2 Reference Genome NC_045512.2    (2314) TAAATTTTTGGCTTTGTGTGCTGACTCTATCATTATTGGTGGAGCTAAACTTAAAGCCTTGAATTTAGGTGAAACATTTGTCA
Fig badnavirus 1 NC_017830.1               (314) CAAATTCTCACCTTT---ACCATAATCTAGCAGTAGTTTACGA--TAGACTAAA--CTTGCAATCTCGTGTTAACCTCAAAAA
```

Section 30

```
                                           (2408) 2408 ... 2490
SARS-CoV-2 Reference Genome NC_045512.2    (2397) CGCACTCAAAGGGATTGTACAGAAAGTGTGTTAAATCCAGAGAAGAAACTGGCCTACTCATGCCTCTAAAAGCCCCAAAAGAA
Fig badnavirus 1 NC_017830.1               (390) C---CTTAAGGGTATTTT--AGAAAGAGTGGAAAAACAAGAGAGAAAACTTGAGAGTTTAGAACT-----AGCAGTAAGGAAC
```

Section 31

```
                                           (2491) 2491 ... 2573
SARS-CoV-2 Reference Genome NC_045512.2    (2480) ATTATCTTCTTAGAGGGAGAAACACTTCCCACAGAAGTGTTAACAGAGGAAGTTGTCTTGAAAACTGGTGATTTACAACCATT
Fig badnavirus 1 NC_017830.1               (463) CTTACCCAAGTTTTTGTTGAAAATAAGCCTT------TAACAACAACTGAAGTTA----GAAGGCT--TGTTTACGAAATCTC
```

Section 32

```
                                           (2574) 2574 ... 2656
SARS-CoV-2 Reference Genome NC_045512.2    (2563) AGAACAACCTACTAGTGAAGCTGTTGAAGCTCCATTGGTTGGTACACCAGTTTGT-ATTAACGGGCTTATGTTGCT-CGAAAT
Fig badnavirus 1 NC_017830.1               (534) TCAGCAACCAAAGCTTGTAGAGCAGGAGGCTCTAAGGTTAACTGAAGAACTTCGTCAAAAACTCGAAAGAGTTGAGGCGATAG
```

Section 33

```
                                           (2657) 2657 ... 2739
SARS-CoV-2 Reference Genome NC_045512.2    (2644) CAAAGACACAGAAAAGTACTGTGCCCTTGCACCTAATATGATGGTAACAAACAATACCTTCACA-CTCAAAGGCGGTGCACCA
Fig badnavirus 1 NC_017830.1               (617) TAAAGAAAGTTGAAAGT--TGGACCTCTTCATGAGTAGTTATATTGTCTCCCAAGGGACAAATAGCTACAAGGAGG----CC-
```

Section 34

```
                                           (2740) 2740 ... 2822
SARS-CoV-2 Reference Genome NC_045512.2    (2726) ACAAAGGTTACTTTTGGTGATGACACTGTGATAGAAGTGCAAGGTTACAAGAGTGTGAATATCACTTTTGAACTT--GATGAA
Fig badnavirus 1 NC_017830.1               (693) ATAAAAGCTACTGAAGGAATAGAATCCCCAGCAGCAGGGTTCG-TGAGACCAGCC-GACTTCCAGGGAGGAACATCGGCAGCG
```

Section 35

```
                                           (2823) 2823 ... 2905
SARS-CoV-2 Reference Genome NC_045512.2    (2807) AGGATT--GATAAAGTACTTAATGAGAAGTGCTCTGCCTATACAGTTGAACTCGGTACAGAAGTAAATGAGTTCGCCTGTGTT
Fig badnavirus 1 NC_017830.1               (774) AGGGTTCAGATTAAGCAGAATAATACCCTTATTCAGCTTCTA--ATCCAGATCGCTGAGTCCCTGAAGGACATC-CGAGAAGA
```

Section 36

(2906) 2906 2920 2930 2940 2950 2960 2970 2988

SARS-CoV-2 Reference Genome NC_045512.2 (2888) GTGGCAGATGCTGTCATAAAAACTTTGCAACCAGTATCTGAATTA-----CTTACACCACTGGGCATTGATTTAGATGAGTGG

Fig badnavirus 1 NC_017830.1 (854) GCAGCGAGTCCTAAAGGAGGAGGTTAGGCAACTGCAGAAGGACAAAGCAGCTTCAATCACAGAAGAGCTTGTGGAAAGACTTC

Section 37

(2989) 2989 3000 3010 3020 3030 3040 3050 3060 3071

SARS-CoV-2 Reference Genome NC_045512.2 (2966) AGTATGGCTACATACTACTT--ATTTGATGAGTCTGGTGAGTTTAAATTGGCTTCACA--TATGTATTGTTCTTTCTACCCTC

Fig badnavirus 1 NC_017830.1 (937) AAAGTCTCTCGTTAGGAGTCCCAGAAAAGAAGGTAGCTGAGAAGAAAAGAACTTTTAGGGTATTCAAAGACCCTTTAAGAATC

Section 38

(3072) 3072 3080 3090 3100 3110 3120 3130 3140 3154

SARS-CoV-2 Reference Genome NC_045512.2 (3045) CA-GATGAGGATGAAGAAGAAGGTG--ATTGTGAAGAAGAAGAGTT---TGAGCCATCAACT-CAA--TATGAGTATGGTACT

Fig badnavirus 1 NC_017830.1 (1020) CTTGAAGAGGAAAAAGAA-AAGCTGCCAAAATGAACAGGTCGAGAACGGTGACTCAGCAGCTGCCAGCTGTGAGTACCAGACG

Section 39

(3155) 3155 3160 3170 3180 3190 3200 3210 3220 3237

SARS-CoV-2 Reference Genome NC_045512.2 (3119) GAAG-ATGA--TTACCAAGG-TAAACCTTTGGAATTTGGTGCCACTTCTGCTGCTCTTCAACCTGAAGAAGAGCAAGAAGAAG

Fig badnavirus 1 NC_017830.1 (1102) GAGGGAAGAAATTAACAGGGATCAACCTCTG----TTCGAGG---ATCAGATCCGTGACTACCGGAGGAATCA-AAGGCGAAG

Section 40

(3238) 3238 3250 3260 3270 3280 3290 3300 3310 3320

SARS-CoV-2 Reference Genome NC_045512.2 (3198) ATTGGTTAGATGATGATAGTCAACAAACTGTTGGTCAACAAGACGG-CAGTGAGGACAATCAGACAACTACTATTCAAACAAT

Fig badnavirus 1 NC_017830.1 (1177) GTTTGT-AGCTGA--------A-CAAGCCGT--------AAGACGAACCATGGGAAGAATGCGAGGACGACGATACAACCAAA

Section 41

(3321) 3321 3330 3340 3350 3360 3370 3380 3390 3403

SARS-CoV-2 Reference Genome NC_045512.2 (3280) TGTTGAGGTTCAACCTCAATT-AGAGATGGAACTTACACCAGTTGTTCAG--ACTATTGAAGTGAATAGTTTAGTGGTTATT

Fig badnavirus 1 NC_017830.1 (1242) CTCTGGAGCAGATCGTAGATCCAGAGGTAGAGCTGAACCAATCTATGCAGGAAAGAGCGAACCTAGTACCTGCAGAAGTTCTG

Section 42

(3404) 3404 3410 3420 3430 3440 3450 3460 3470 3486

SARS-CoV-2 Reference Genome NC_045512.2 (3360) TAAAACTTACT----GACAATGTATAC-ATTAAAAATGCAGACATTGTG--GAAGAAGCTAAA--------AAGGTAAAACCA

Fig badnavirus 1 NC_017830.1 (1325) TACCGCTCAAGAAGAGACAACATAAACCATCAGATCTATAATCATAGATCCGAGGAAGCGATGCTTTGTGTAGGAGAACAGCA

Section 43

```
                                      (3487) 3487         3500      3510      3520      3530      3540      3550              3569
SARS-CoV-2 Reference Genome NC_045512.2 (3428) ACAGTGGTTGTTAATGCAGCCAAT--GTTTACCTTAAACATGGAGGAGGTGT-TGCAGGAGCCTTAAATAAGGCTACTAACAA
     Fig badnavirus 1 NC_017830.1 (1408) AGACAGGATGTTTATCCAACCTAACAGCTTCCAAGAACTACAGAGGAGTGGTATGCAGTT--CATACATCTGGGAGTTT----
```

Section 44

```
                                      (3570) 3570      3580      3590      3600      3610      3620      3630      3640        3652
SARS-CoV-2 Reference Genome NC_045512.2 (3508) TGCCATGCAAGTT-GAATCTGATGATTACATAGCTACTAATGGACCACTTAAAGTGGGTGGTAGTTGTGTTTTAAGCGGACAC
     Fig badnavirus 1 NC_017830.1 (1485) -----TGCAAGTAAGACTCCAGATACTGCATAGGG-CAGATGGAGGAAC-AATGGCCTTGGTAGTCTT------------CAG
```

Section 45

```
                                      (3653) 3653   3660      3670      3680      3690      3700      3710      3720           3735
SARS-CoV-2 Reference Genome NC_045512.2 (3590) AATCTTGCTAAACACTGTCTTCATGTTGTCGGCCCAAATGTTAACAAAGGTGAAGACATTCAACTTCTTAAGAGTGCTTATGA
     Fig badnavirus 1 NC_017830.1 (1549) A-----GATAACCGATGGC--AAGGAGATCAGTC--AATCTTCGCAATCT--CAGAAGTTGA-CTTGACTAGAG-GATC--GC
```

Section 46

```
                                      (3736) 3736          3750      3760      3770      3780      3790      3800            3818
SARS-CoV-2 Reference Genome NC_045512.2 (3673) AAATTTTAATCAGCACGAAGTTCTACTTG-CACCATTATTATCAGCTGGTATTTTGGTGCTGACCCTATACAT-TCTTTAAG
     Fig badnavirus 1 NC_017830.1 (1617) AAATAGTATAC--------GTGATACCTGACACGATGATGATTATCGGTGACTTCTACAG--AAACGTTCAGATCTCTATCCA
```

Section 47

```
                                      (3819) 3819       3830      3840      3850      3860      3870      3880      3890       3901
SARS-CoV-2 Reference Genome NC_045512.2 (3754) AGTTTGTGTAGAT-ACTGTTCGCACAAATGTCTACTTAGCTGTCTTTGATAAAAATCTCTATGACAAACTTGTT-TCAAGCTT
     Fig badnavirus 1 NC_017830.1 (1690) GACCAGTGGATATGAGAATTGGCAGAA-TGGAGAGGCAAATTTGCT-GATCACAAGAGGCATGACGGGAAGGTTATCCAACAC
```

Section 48

```
                                      (3902) 3902    3910      3920      3930      3940      3950      3960      3970          3984
SARS-CoV-2 Reference Genome NC_045512.2 (3835) TTTGGAAATGAAGAGTGAAAAGCAAGTTGAACAAAAGATCGCTGAGATT-CCTAAAGAGGAAGTTAAGCCATT-TATAACTGA
     Fig badnavirus 1 NC_017830.1 (1771) TC--CAAATGTTG---GATTTGCTTACAGAATAAGCCATGTGACGGATTACCTAGCAAGCAGGGGAGTCCAAGCTTTGCCGGG
```

Section 49

```
                                      (3985) 3985 3990      4000      4010      4020      4030      4040      4050                4067
SARS-CoV-2 Reference Genome NC_045512.2 (3916) AAGTAAACCTTCAGTTGAACAGAGAAAACAAGATGATAAGAAAATCAAAGCTTGTGTTGAAGAAGTTACAA-CAACTCTGGAA
     Fig badnavirus 1 NC_017830.1 (1849) AAG--AAGATACAG---ATCGGAGATGCTACGAGGA--AGAAACTGGATCATAAGGCAGCCACAGATCCAGGCGGCAATGATG
```

Section 50

	(4068)	4068 4080 4090 4100 4110 4120 4130 4140 4150
SARS-CoV-2 Reference Genome NC_045512.2	(3998)	GAAACTAAGTTCCTCACAGAAAACTTGTTACTTTATATTGACATTAATGGCAATCTTCATCCAGATTCTGCCACTCTTGTTAG
Fig badnavirus 1 NC_017830.1	(1925)	CCAAGGAACGTGGAAACAAGGAACCTGATAGATG----GAACAATCTCTGCAAGAT--CTGCAGATTA---CACTCAGGCACC

Section 51

	(4151)	4151 4160 4170 4180 4190 4200 4210 4220 4233
SARS-CoV-2 Reference Genome NC_045512.2	(4081)	TGA-CATTGACATCACTTTCTTAAAGAAAGATGCTCCATATATAGTGGGTGATGTTGTTCAAGAGGGTGTTTTAACTGCTGTG
Fig badnavirus 1 NC_017830.1	(1999)	AGAACCACGACAACCCCAT-TACAATGAGCAGGACGAAGAAGTGGCGAGTGATGAGG---AAGAGCTGGCAGAAACTCAACAG

Section 52

	(4234)	4234 4240 4250 4260 4270 4280 4290 4300 4316
SARS-CoV-2 Reference Genome NC_045512.2	(4163)	GTTATACCTACTAAAAAGGCTG-GTGGCACTACTGAAATGCTAGCGAAAGC--TTTGAGA-AAAGTGCCAACAGACAATTATA
Fig badnavirus 1 NC_017830.1	(2078)	----CACATAGTAGCAATGCTGCGATTTCCGGATTATGTGG-AGATACAGGAGTTTAAGCCAAAGTACAAAGAG-CAATACGA

Section 53

	(4317)	4317 4330 4340 4350 4360 4370 4380 4399
SARS-CoV-2 Reference Genome NC_045512.2	(4242)	TAACCACTTACCCGGGTCAGGGTTTAAATGGTTACACTGTAGAGGAGGCAAAGACAGTGCTTAAAAAGTGTAAAAGTGCCTTT
Fig badnavirus 1 NC_017830.1	(2155)	CAAGTACA-AAAGGGAGCAGGGCT----GGGATACCCTG----GGAG---AACCAAGCGGAAAGTATGACTACTAC-GTCCGC

Section 54

	(4400)	4400 4410 4420 4430 4440 4450 4460 4470 4482
SARS-CoV-2 Reference Genome NC_045512.2	(4325)	TACATTCTACCATCTATTATCTCTAATGAGAAGCAAGAAATTCTTGGAACTGTTTCTTGGAATTTGCGAGAAATGCTTGCACA
Fig badnavirus 1 NC_017830.1	(2225)	TACACTGCACCTCCAGCGA---CAACGCCGATAGAAGATATTCAGCCAACAGGCTGGGAGGAGGAGCCTAAATGGGATGATTC

Section 55

	(4483)	4483 4490 4500 4510 4520 4530 4540 4550 4565
SARS-CoV-2 Reference Genome NC_045512.2	(4408)	TGCAGAAGAAACACGCAAATTAATGCCTGTCTGTGTGGAAACTAAAGCCATAGTTTCAACTATACAGCGTAAATATAAGGGTA
Fig badnavirus 1 NC_017830.1	(2305)	ACCCGACGAGAAATCATTAGCAACAATTCGATTCGAGGAATACAAGGAAGAAG-----AATCTCAATCGGATGAAGAACATTA

Section 56

	(4566)	4566 4580 4590 4600 4610 4620 4630 4648
SARS-CoV-2 Reference Genome NC_045512.2	(4491)	TTAAAATACAAGAGGGTGTGGTTGATTATGG-TGCTAGATTTTACTTTTACACCAGTAAACAACTGTAGCGTCACTTATCAA
Fig badnavirus 1 NC_017830.1	(2383)	TCAA----CCAGATGCTGAAGCAGAATTTGACTACCCGCTAAAAAGGATTCATTACCTAAATC-CTTTTTCGGAAGGTGGTGG

Section 57

```
                                       (4649) 4649       4660      4670      4680      4690      4700      4710      4720      4731
SARS-CoV-2 Reference Genome NC_045512.2 (4573) CACACTTAACGATCTAAATGAAACTCTTGTTACAATGCCACTTGGCTATGTAACACATGGCTTAAATTTGGAAGAAGCTGCTC
     Fig badnavirus 1 NC_017830.1 (2461) GAAAGATGACCATTTAAAT-----TCTGAAGA-AATGG-ACTACCC---GCAAAGAATGAAGAAAATAGAAAAGTTACTA-TC
```

Section 58

```
                                       (4732) 4732    4740      4750      4760      4770      4780      4790      4800      4814
SARS-CoV-2 Reference Genome NC_045512.2 (4656) GGTATATGAGATCTCTCAAAGTGCCAGCTACAGTTTCTGTTTCTTCACCTGATGCTGTTACAGCGTATAATGGTTATCTTACT
     Fig badnavirus 1 NC_017830.1 (2533) CACCAGTGAGGTAACT---AGT-CCA--TACAGAC-------CACCAGAAGATGCAGCCATGGGAAGACCAAGTTATC---CA
```

Section 59

```
                                       (4815) 4815 4820      4830      4840      4850      4860      4870      4880      4897
SARS-CoV-2 Reference Genome NC_045512.2 (4739) TCTTCTTCTAAAACACCTGAAGAACATTTTATTGAAACCATCTCACTTGCTGGTTCCTATAAAGATTGGTCCTATTCTGGACA
     Fig badnavirus 1 NC_017830.1 (2600) CCAGCTAGGACAATACCAGGAGGA---------GGGGCAAGCTCAAGCGCTGAA-CCTCCAAGGTTTGATGC-AGCCAGAACA
```

Section 60

```
                                       (4898) 4898        4910      4920      4930      4940      4950      4960      4970      4980
SARS-CoV-2 Reference Genome NC_045512.2 (4822) ATCTACACAACTAGGTATAGAATTTCTTAAGAGAGGTGATAAAAGTGTATATTACACTAGTAATCCTACCACATTCCACCTAG
     Fig badnavirus 1 NC_017830.1 (2672) AGATTTAAAG--GGGGATACAATGACGAGAT-GTGGACACTACCATCAGCCCAACAGAAG-AATGGAGCCATGTTCGTAATAC
```

Section 61

```
                                       (4981) 4981     4990      5000      5010      5020      5030      5040      5050      5063
SARS-CoV-2 Reference Genome NC_045512.2 (4905) ATGGTGAAGTTATCACCTTTGACAATCTTAAGACACTTCTTTCTTTGAGAGAAGTGAGGACTATTAAGGTGTTTACAACAGTA
     Fig badnavirus 1 NC_017830.1 (2751) CAG--------AACAACTTGGGCTATTCAATGATGCTT-TCTCTAGATGGGAATCAGTCACTAAAAACCATGTGGCTACACAA
```

Section 62

```
                                       (5064) 5064  5070      5080      5090      5100      5110      5120      5130      5146
SARS-CoV-2 Reference Genome NC_045512.2 (4988) GACAACATTAACCTCCACACGCAAGTTGTGGACATGTCAATGACATATGGACAACAGTTTGGTCCAACTTATTTGGATGGAGC
     Fig badnavirus 1 NC_017830.1 (2825) GGTTTTACTGATACCCGAGATAAAATAAAGTACATGGAAAATCTACTCGGAGAAA----TTGAGAAGCTGATTTGGATACAGT
```

Section 63

```
                                       (5147) 5147         5160      5170      5180      5190      5200      5210               5229
SARS-CoV-2 Reference Genome NC_045512.2 (5071) TGATGTTACTAAAATAAAACCTCATAATTCACATGAAGGTAAAACATTTTATGTTTTACCTAATGATGACACTCTACGTGTTG
     Fig badnavirus 1 NC_017830.1 (2904) GGAG---AATGACGTATG-CCACGGAATAC----GAAGCCTTAGTAGCCATCGGAGAAGGCAGGGATGGTACTCA--------
```

Section 64

```
                                         (5230) 5230      5240      5250      5260      5270      5280      5290      5300      5312
SARS-CoV-2 Reference Genome NC_045512.2  (5154) AGGCTTTTGAGTACTACCACACAACTGATCCTAGTTTTCTGGGTAGGTACATGTCAGCATTAAATCACACTAAAAAGTGGAAA
          Fig badnavirus 1 NC_017830.1  (2971) --------GAACATTCTCTCGCAAATGAGAAGAGTGTTTTCCTTAGAAGATCCGGCGCAGGGTTCGACAGTAATCCAGGATGA
```

Section 65

```
                                         (5313) 5313   5320      5330      5340      5350      5360      5370      5380      5395
SARS-CoV-2 Reference Genome NC_045512.2  (5237) TACCCACAAGTTAATGGTTTAACT-TCTATTAAATGGGCAGATAACAACTGTTATCTTGCCACTGCATTGTTAACACTCCAAC
          Fig badnavirus 1 NC_017830.1  (3046) AGCCTACAGAGAGTTGGAAAGAATGTCTTGTACAGACGTTAAATACATCA-TTCCATTTCTAAATGAGTATATGCGTTTGGCA
```

Section 66

```
                                         (5396) 5396          5410      5420      5430      5440      5450      5460      5478
SARS-CoV-2 Reference Genome NC_045512.2  (5319) AAATAGAGTTGAAGTTTAATCCACCTGCTCTACAAGATGCTTATTACAGAGCAAGGGCTGG-TGAAGCTGCTA----ACTTTT
          Fig badnavirus 1 NC_017830.1  (3128) GCAAAGACGGGAAGGCTATTC----TTAG---GAGGAGAACTAT--CTGAG-AAGATCTGGATGAAGATGCCCGGAGATTTAA
```

Section 67

```
                                         (5479) 5479       5490      5500      5510      5520      5530      5540      5550      5561
SARS-CoV-2 Reference Genome NC_045512.2  (5397) GTGCACTTATCTTAGCCTACTGTAATAAGACAGTAGGTG-AGTTAGGTGATGTTAGAGAAACAATGAGTTACTTGTTTCAACA
          Fig badnavirus 1 NC_017830.1  (3201) GAAAAACAATAAAAGAAGACTTTGACAACAAATACCCAGGAGTCGTATCAGGGGTGGTACCCAGGGTATTATTTGCTT--ACA
```

Section 68

```
                                         (5562) 5562    5570      5580      5590      5600      5610      5620      5630      5644
SARS-CoV-2 Reference Genome NC_045512.2  (5479) TGCCAATTTAGATTCTTGCAAAAGAGTCTTGAACGTGGTGTGTAAAACTTGTGGACAACAGCAGACAACCCTTAAGGGTGTAG
          Fig badnavirus 1 NC_017830.1  (3282) AG----------TTCTTGGAGAAG------GAATGCAGAGATGCAGCATTCAAGAGGTCCCTAAAGAACCTGTCATTTTGTAG
```

Section 69

```
                                         (5645) 5645 5650      5660      5670      5680      5690      5700      5710      5727
SARS-CoV-2 Reference Genome NC_045512.2  (5562) AAGCTGTTATGTACATGGGCACACTTTCTTATGAACAATTTAAGAAAGGTGTTCAGATACCTTGTACGTGTGGTAAACAAGCT
          Fig badnavirus 1 NC_017830.1  (3349) TTCAATTCCTATACCAGGATAC-------TAT-AAAAATACAGGCAAGAAGTATGGA------GTGCGAA-GATCCAAGACGT
```

Section 70

```
                                         (5728) 5728        5740      5750      5760      5770      5780      5790      5800  5810
SARS-CoV-2 Reference Genome NC_045512.2  (5645) ACAAAATATCTAGTACAACAGGAGTCACCTTTTGTTATGATGTCAGCACCACCTGCTCAGTATGAACTTAAGCATGGTACATT
          Fig badnavirus 1 NC_017830.1  (3417) ACAAAG--GCAAGCCTCACGAGAGCCATGCTAGGATTGAAAAGCGAAAGCACCTGGTCCGCAACAAAAGATGCAAGTGCTTCC
```

Section 71

```
                                        (5811) 5811     5820      5830      5840      5850      5860      5870      5880      5893
SARS-CoV-2 Reference Genome NC_045512.2 (5728) TACTTGTGCTAGTGAGTACACTGGTAATTACCAGTGTGGTCACTATAAACATATAACTTCTAAAGAAACTTTGTATTGCATAG
Fig badnavirus 1 NC_017830.1           (3498) TATGTGGACAAGAAGGTCATTTTGCAAGAA--AGTGTCCTAATGATAAA---AGGAGCTCTAA----A---------------
```

Section 72

```
                                        (5894) 5894  5900      5910      5920      5930      5940      5950      5960      5976
SARS-CoV-2 Reference Genome NC_045512.2 (5811) ACGGTGCTTTACTTACAAAGTCCTCAGAATACAAAGGTCCTATTACGGATGTTTTCTACAAAGAAAACAGTTACACAACAACC
Fig badnavirus 1 NC_017830.1           (3557) AGGATGGCTATGTTTGAAAATCTGGAAATACCAGAAG----ATTGCG-ATATAGTCTCTGTAGAAGAAGG--AGAGGATATGA
```

Section 73

```
                                        (5977) 5977         5990      6000      6010      6020      6030      6040      6059
SARS-CoV-2 Reference Genome NC_045512.2 (5894) ATAAAACCAGTTACTTATAAATTGGATGGTGTTGTTTGTACAGAAATTGACCCTAAGTTGGACAATTATTATAAGAAAGACAA
Fig badnavirus 1 NC_017830.1           (3633) GTGATGCCATATACTCA-ATATCCGAAGGAGAAGATGGAGCAGA---TGAGCTGCAGAAAT-CACTCCAAACATTGGAAATTG
```

Section 74

```
                                        (6060) 6060      6070      6080      6090      6100      6110      6120      6130      6142
SARS-CoV-2 Reference Genome NC_045512.2 (5977) TTCTTATTTCACAGAGCAACCAATTGATCTTGTACCAAACCAACCATATCCAAACGCAAGCTTCGATAATTTTAAGTTTGTAT
Fig badnavirus 1 NC_017830.1           (3711) TTCTTATGTTAG-GAGAAATTGATGGAG--GGTACCGGCCG---CAGATCAAGCTTCCTGA--GGAGCAGATGAATTGTGTCC
```

Section 75

```
                                        (6143) 6143   6150      6160      6170      6180      6190      6200      6210      6225
SARS-CoV-2 Reference Genome NC_045512.2 (6060) GTGATAATATCAAATTTGCTGATGATTTAAACCAGTTAACTGGTTATAAGAAACCTGCTTCAAGAGAGCTTAAAGTTACATTT
Fig badnavirus 1 NC_017830.1           (3786) ATAACTGGATACATAATGGGGATATCTTTGTACAAAGAGCTGTTCATTGCA-----GCTTCTGCAGAAGGGAAAGCATAAAAA
```

Section 76

```
                                        (6226) 6226          6240      6250      6260      6270      6280      6290      6308
SARS-CoV-2 Reference Genome NC_045512.2 (6143) TTCCCTGACTTAAATGGTGATGTGGTGGCTATTGA----TTATAAACACTACACACC---CTCTTTTAAGAAAGGAGCTAAAT
Fig badnavirus 1 NC_017830.1           (3864) GGGCAAGAAT-ACATTGTCCTTTG-TGTCTCCTAACAGCTTGCAATCTGTGCGGACCTTACTACTTGAAGATAGAAGTACCGG
```

Section 77

```
                                        (6309) 6309       6320      6330      6340      6350      6360      6370      6380      6391
SARS-CoV-2 Reference Genome NC_045512.2 (6219) TGTTACATAAACCTATTGTTTGGCATGTTAACAATGCAACTAATAAAGCCACGTATAAACCAAATACC-TGGTGTATACGTTG
Fig badnavirus 1 NC_017830.1           (3945) TGGAACCAGCACC-ATCAACCCCGATAAATCCGAGAAGAATTATTCAGGAGCAGCACAACTACATCGGATGGTGTG-AAGTAG
```

```
                                                                                                         Section 78
                                       (6392) 6392    6400      6410      6420      6430      6440      6450      6460      6474
SARS-CoV-2 Reference Genome NC_045512.2 (6301) TCTTTGGAGCACAAAACCAGT---TGAAACATCAAATTCGTTTGATGTACTGAAGTCAGAGGACGCGCAGGGAATGGATAATC
   Fig badnavirus 1 NC_017830.1 (4026) AAATAGAGAGACTAAACAAGGAAGTGAAGCACTACAAAGG---GCTATAC-GAAGCCTTACTAATGGA--GAAATCCTTAAGG
                                                                                                         Section 79
                                       (6475) 6475 6480      6490      6500      6510      6520      6530      6540          6557
SARS-CoV-2 Reference Genome NC_045512.2 (6381) TT-GCCTGCGAAGATCTAAAACCAGTCTCTGAAGAAGTAGTGGAAAATCCTACCATACAGAAAGACGTTCTTGAGTGTAATGT
   Fig badnavirus 1 NC_017830.1 (4103) AAAGACTACGAGGAGCTGAAAGAAG----GAGATAAGGAGCGAAGAAAGGG--CATAATGATACAAGAACCAGA-----AGAA
                                                                                                         Section 80
                                       (6558) 6558        6570      6580      6590      6600      6610      6620      6630    6640
SARS-CoV-2 Reference Genome NC_045512.2 (6463) GAAAACTACCGAAGTTGTAGGAGACATTATACTTAAACCAGCAA-ATAATAGTTTAA-AAATTACAGAAGAGGTTGGCCACAC
   Fig badnavirus 1 NC_017830.1 (4175) GTTAACTATCTGGGA-GAAGATGAGAAGATTCTTGCAGCAGGAACAGAGGAAAAGAAGAAATTCGTCAAGAATATG-CTTTAT
                                                                                                         Section 81
                                       (6641) 6641     6650      6660      6670      6680      6690      6700      6710         6723
SARS-CoV-2 Reference Genome NC_045512.2 (6544) AGATCTAATGGCTGCTTATGTAGACAATTCTAGTCTTACTATTAAGAAACCTAATGAATTATCTAGAGTATTAGGTTTGAAAA
   Fig badnavirus 1 NC_017830.1 (4256) AACTTTGATAT-TGAATTTG-AGATTCCAGATGTCTCAAGATTCAGAGTAAGAGC-AATTCTTGATAC-AGGAGCTTCAACAT
                                                                                                         Section 82
                                       (6724) 6724  6730      6740      6750      6760      6770      6780      6790          6806
SARS-CoV-2 Reference Genome NC_045512.2 (6627) CCCTTGCTACTCATGGTTTAGCTGCTGTTAATAGTGTCCCTTGGGATACTATAGCTAATTATGCTAAGCCTTTTCTTAACAAA
   Fig badnavirus 1 NC_017830.1 (4335) GCTGTATCAATGAAGGAGCAGTTCC----AAAAGAAGCCCTC--GAAGAAAGCCCTTATGAAGTTCAGCTGAATGGGGTCAAC
                                                                                                         Section 83
                                       (6807) 6807          6820      6830      6840      6850      6860      6870            6889
SARS-CoV-2 Reference Genome NC_045512.2 (6710) GTTGTTAGTACAACTACTAACATAGTTACACGGTGTTTAAACCGTGTTTGTACTAATTATATGCCTTATTTCTTTACTTTATT
   Fig badnavirus 1 NC_017830.1 (4412) TCTGTGCAGAAAACAAAGAAGA-AGCTCAAGTACGGGAGAATGATGATCGGGATGAATACGTTCAGAATCCCTTT----TAC-
                                                                                                         Section 84
                                       (6890) 6890      6900      6910      6920      6930      6940      6950      6960        6972
SARS-CoV-2 Reference Genome NC_045512.2 (6793) GCTACAATTGTGTACTTTTACTAGAAGTACAAATTCTAGA--ATTAAAGCATCTATGCCGACTACTATAGCAAAGAATACTGT
   Fig badnavirus 1 NC_017830.1 (4489) -ATACAGTCTGCCACTGGTCATCGGAG-ATAACATCCAGATGATTGTAGGTTGTA--------ACTTCATCAGAGCAATGTAT
```

SARS-CoV-2 & Fig badnavirus

```
                                                                                        Section 85
                                        (6973) 6973   6980           6990           7000           7010           7020           7030           7040         7055
SARS-CoV-2 Reference Genome NC_045512.2 (6874) TAAGAGTGTCGGTAAATTTTGTCTAGAGGCTTCATTTAATTATTTGAAGTCACCTAATTTTTCTAAACTGATAAATATTATAA
        Fig badnavirus 1 NC_017830.1    (4562) GGAG-GAGTGAGAATAGAAGGGAATGAGGTAACCTTTTACAAGA--ACCTCACACGGATTAGCACATCCCCAGAAG--TCTCG
```

```
                                                                                        Section 86
                                        (7056) 7056                 7070           7080           7090           7100           7110           7120         7138
SARS-CoV-2 Reference Genome NC_045512.2 (6957) TTTGGTTTTTACTATTAAGT-GTTTGCCTAGGTTCTTTAATCTACTCAACCGCTGCTTTAGGTGTTTT--AATGTCTAATTTA
        Fig badnavirus 1 NC_017830.1    (4640) GTGAATTCCCTGGATGAAGAAGTTTGCGAAGAA----GAATATTTGCAGGTGCAGGAGATGGTGATCTGCAACATTGGAGAAA
```

```
                                                                                        Section 87
                                        (7139) 7139      7150           7160           7170           7180           7190           7200           7210     7221
SARS-CoV-2 Reference Genome NC_045512.2 (7037) GGCATGCCTTCTTACTGTACTGGTTACAGAGAAGGCTATTTGAACTCTACTAAT----GTCACTATTGCAACCTACTGTACTG
        Fig badnavirus 1 NC_017830.1    (4719) GCCGAAAATCATTTCTTAAGAAGTTCAGGCCTTTGCTGGAAGAACTTAAGGAAGCAGGGTTTATAGGAGAAAATCCATTACAG
```

```
                                                                                        Section 88
                                        (7222) 7222    7230           7240           7250           7260           7270           7280           7290         7304
SARS-CoV-2 Reference Genome NC_045512.2 (7116) GTTCTATACCTTGTAGTGTTTGTCTTAGTGGTTTAGATTCTTTAGACACCTATCCTTCTTTAGAAACTATACAAATTACCATT
        Fig badnavirus 1 NC_017830.1    (4802) CATTGGAAGAAAAATGGGATTTTATGCCAACTTGATATCAAAAATCCTGATTTCATAATT--GAAGATAGGCCACTGAAGAAT
```

```
                                                                                        Section 89
                                        (7305) 7305 7310           7320           7330           7340           7350           7360           7370              7387
SARS-CoV-2 Reference Genome NC_045512.2 (7199) TCATCTTTTAAATGGGATTTAACTGCTTTTGGCTTAGTTGCAGAGTGGTTTTTGGCATATATTCTTTTCACTAGGTTTTTCTA
        Fig badnavirus 1 NC_017830.1    (4883) TTAACGCCACAGATGAAG--GAGTCCTTCAAGAAGCATATCAAGGT----TTTGCTGGATTTAGGAGTCATCAGGGC-----A
```

```
                                                                                        Section 90
                                        (7388) 7388        7400           7410           7420           7430           7440           7450           7460   7470
SARS-CoV-2 Reference Genome NC_045512.2 (7282) TGTACTTGGATTGGCTGCAATCATGCAATTGTTTTTCAGCTATTTTGCAGTACATTTTATTAGTAATTCTTGGCTTATGTGGT
        Fig badnavirus 1 NC_017830.1    (4955) AGTAAAAGCCGCCACAG-AACGACGGCAATGCTAGTCAACTCA--GGAACAACGGTGGATCCG-AAAACAGGG---AAGGAGA
```

```
                                                                                        Section 91
                                        (7471) 7471     7480           7490           7500           7510           7520           7530           7540         7553
SARS-CoV-2 Reference Genome NC_045512.2 (7365) TAATAATTAATCTTGTACAAATGGCCCCGATTTCAGCTATGGTTAGAATGTACATC--TTCTTTGCATCATTTTATTATGTAT
        Fig badnavirus 1 NC_017830.1    (5031) TCAAAGGAAG---GAAAGAATGGTCTTCAACT-ACAAAAGGTT-GAATG-ACATCACTCATAAGGATCAGTATAGTCTTCCA
```

```
                                                      Section 92
                                       (7554) 7554 7560 7570 7580 7590 7600 7610 7620 7636
SARS-CoV-2 Reference Genome NC_045512.2 (7446) GGAAAAGTTATGTGCATGTTGTAGACGGTTGTAATTCATCAACTTGTATGATGTGTTACAAACGTAATAGAGCAACAAGAGTC
     Fig badnavirus 1 NC_017830.1       (5108) GGAATAAATACG---ATCCTCAAGAAGGTTGGAAAT-AGCAA---G----ATATTCAGCAAGTTTGATCTA---A--AGAG-C

                                                      Section 93
                                       (7637) 7637 7650 7660 7670 7680 7690 7700 7719
SARS-CoV-2 Reference Genome NC_045512.2 (7529) GAATGTACAACTATTGTTAATGGTGTTAGAAGGTCCTTTTATGTCTATGCTAATGGAGGTAAAGGCTTTTGCAAACTACACAA
     Fig badnavirus 1 NC_017830.1       (5174) GGAT-TTCACCAAGTTGCTATG------------CATCCTGACTCAATAG-AATGGAC----AGCTTTTTGGGTCCCAGACGG

                                                      Section 94
                                       (7720) 7720 7730 7740 7750 7760 7770 7780 7790 7802
SARS-CoV-2 Reference Genome NC_045512.2 (7612) TTGGAATTGTGTTAATTGTGATACATTCTGTGCTGGTAGTACATTTATTAGTGATGAAGTTGCGAGAGACTTGTCACTACAGT
     Fig badnavirus 1 NC_017830.1       (5239) ATTGTAT-GAGTGGCTTGTCATGCCATTCGGACTCAAAA-ACGCTCCTTC----TGTTTTTCAAAGAAAGATGGATGAATGCT

                                                      Section 95
                                       (7803) 7803 7810 7820 7830 7840 7850 7860 7870 7885
SARS-CoV-2 Reference Genome NC_045512.2 (7695) TTAAAAGACCAATAAATCCTACTGACCAGTCTTCTTACATCGTTGATAGTGTTACAGTGAAGAATGGTTCCATCCATCTTTAC
     Fig badnavirus 1 NC_017830.1       (5316) TCAAAGGGACAGAAGATTTTATTG--CAGTC----TACATTGACGATATTCTT-------------GTTTTCTC-----TGAA

                                                      Section 96
                                       (7886) 7886 7900 7910 7920 7930 7940 7950 7968
SARS-CoV-2 Reference Genome NC_045512.2 (7778) TTTGATAAAGCTGGTCAAAAGACTTATGAAAGACATTCTCTCTCTCATTTTGTTAACTTAGACAAC-CTGAGAGCTAATAACA
     Fig badnavirus 1 NC_017830.1       (5375) AACGAGAAAGATCATGCGAAGCACT-TGAAGGCCATGTTGGAGATCTGCAAAAGAAATGGGTTAGTTCTAAGCCCATCTAAAA

                                                      Section 97
                                       (7969) 7969 7980 7990 8000 8010 8020 8030 8040 8051
SARS-CoV-2 Reference Genome NC_045512.2 (7860) CTAAAGGTTCATTGCCTATTAATGTTATAGTTTTTGATGGTAAATCAAAATGTGAAGAATCATCTGCAAAATCAGCGTCTGTT
     Fig badnavirus 1 NC_017830.1       (5457) -TGAAGATAGCAGTCCAAGAGGTGGAATT-TCTTGGAGCCCAAATCGGAAATCAAAGGATC-----CGACTTCAGCCACATGT

                                                      Section 98
                                       (8052) 8052 8060 8070 8080 8090 8100 8110 8120 8134
SARS-CoV-2 Reference Genome NC_045512.2 (7943) TACTACAGTCAGCTTATGTGTCAACCTATACTGTTACTAGATCAGGCATTAGTGTCTGATGTTGGTGATAGTGCGGAAGTTGC
     Fig badnavirus 1 NC_017830.1       (5533) TA-TAAAGAAGATCGTTGAGTTCAATGAGGCTG--AGTTGAAGGAGAAAAAGGGCATGA----GATCATGGTTAGGAATTCTC
```

Section 99

	(8135)	8135 8140 8150 8160 8170 8180 8190 8200 8217
SARS-CoV-2 Reference Genome NC_045512.2	(8026)	AGTTAAAATGTTTGATGCTTACGTTAATACGTTTTCATCAACTTTTAA-CGTAC-CAATGGAAAACTCAAAACACTAGTTGC
Fig badnavirus 1 NC_017830.1	(5609)	AACT---ATGCTCGC-GCATACATTCCTAACTTGGGC-CGCCTTTTAAGCCCACTCTATGCCAAAAC----AAGTCCAACTGG

Section 100

	(8218)	8218 8230 8240 8250 8260 8270 8280 8290 8300
SARS-CoV-2 Reference Genome NC_045512.2	(8107)	AACTGCAGAAGCTGAACTTGCAAAGAATGTGTCCTTAGACAATGTCTTATCTACTTTTATTTCAGCAGCTCGGCAAGGGTTTG
Fig badnavirus 1 NC_017830.1	(5683)	AGATA-AGAGGATGAA--TGCCCAGGAT-------TGG--AAGCTCGTGGCCCAAATAAAAGAAGAAGTCCAGAA--GC-TGC

Section 101

	(8301)	8301 8310 8320 8330 8340 8350 8360 8370 8383
SARS-CoV-2 Reference Genome NC_045512.2	(8190)	TTGATTCAGATGTAGAAACTAAAGATGTTGTTGAATGTCTTAAATTGTCACATCAATCTGACATAGAAGTTACTGGCGATAGT
Fig badnavirus 1 NC_017830.1	(5751)	CAGATCTGGAGCTACCACCCGAGGA--TTGTTTCATC------ATTCTTGAAACAGACGGCTGTATGACTGGTTGGGGAGGAA

Section 102

	(8384)	8384 8390 8400 8410 8420 8430 8440 8450 8466
SARS-CoV-2 Reference Genome NC_045512.2	(8273)	TGTAATAACTATATGCTCACCTATAACAAAGTTGAAAACATG-ACACCCCGTGACCTTGGTGCTTGTATTGACTGTAGTGCGC
Fig badnavirus 1 NC_017830.1	(5826)	TTTG-TAAATGGAAGCCCAAAAGAAACGACCCAAGAAGGATGGAGAAAGTTTGCGCCTA-TGCTAGTGGGAAGTTTAGTCCAA

Section 103

	(8467)	8467 8480 8490 8500 8510 8520 8530 8549
SARS-CoV-2 Reference Genome NC_045512.2	(8355)	----GTCAT--ATTAATGCGCAGGTAGCAAAAGTCAC-AACATTGCTTTGATATGGAACGTTAAAGATTTCATGTCATTGTC
Fig badnavirus 1 NC_017830.1	(5907)	TCAAGTCAACCATTGATGCCGAAATC-CATGCAGTCATGAACA---CTCTG----GAAAAATTAAAGATTTATTATCTTGATA

Section 104

	(8550)	8550 8560 8570 8580 8590 8600 8610 8620 8632
SARS-CoV-2 Reference Genome NC_045512.2	(8431)	TGAACAACTACGAAACAAATACGTAGTGCTGCTAAAAAGAATAACTTACCTTTTAAGTTGACATGTGCAACTACTAGACAAG
Fig badnavirus 1 NC_017830.1	(5982)	AAAAGGAGTT-GATCATCAGAACTGACTGCC-----AAGCTATCATCAGCTTCTTCAAT--AAATCCGC----CCTAAATAAA

Section 105

	(8633)	8633 8640 8650 8660 8670 8680 8690 8700 8715
SARS-CoV-2 Reference Genome NC_045512.2	(8514)	TTGTTAATGTTGTAACAACAAAGATAGCACTTAAGGGTGGTA--AAATTGTTAATAATTGGT-TGAAGCAGTTAATTAAAGTT
Fig badnavirus 1 NC_017830.1	(6053)	CCCTCTAGAGTG-----AGATGGGTAGCCTTTACGGATTTTATCACAGGGATAGGAATTAATGTGGAGTTCGAGCATATAGAT

Section 106

```
                                         8716          8730      8740      8750      8760      8770      8780            8798
                                 (8716)  
SARS-CoV-2 Reference Genome NC_045512.2 (8594) ACACTTGTGTTCCTTTTTGTTGCTGCTATTTTCTATTTAATAACACCTGTTCATGTCATGTCTAAACATACTGACTTTTCAAG
      Fig badnavirus 1 NC_017830.1 (6131) GGAAGGGATAATGTGCTTGCTGATAGCCTTTCAAGGTTAATTA-ACAGTTTC-TT-----TCTTACAGAATGGAGTCTGAAAA
```

Section 107

```
                                         8799       8810      8820      8830      8840      8850      8860      8870      8881
                                 (8799)
SARS-CoV-2 Reference Genome NC_045512.2 (8677) TGAAATCATAGGATACAAGGCTATTGATGGTGGTGTCACTCGTGACATAGCATCTA-CAGATACTTGTTTTGCTAACAAACAT
      Fig badnavirus 1 NC_017830.1 (6207) AGGAGGAAGCAGTAGCAGAGCTAGAGAAGCTTATGGAAGACAA-ACAATGCAGGAAGCAACTCCTACCATTAATCAACAAAAT
```

Section 108

```
                                         8882     8890      8900      8910      8920      8930      8940      8950          8964
                                 (8882)
SARS-CoV-2 Reference Genome NC_045512.2 (8759) GCTGATTTTGACACATGGTTTAGCCAGCGTGGTGGTAGTTATACTAATGACAAAGCTTGCCCATTGATTGCTGCAGTCATAAC
      Fig badnavirus 1 NC_017830.1 (6289) GACGAACTGTTTCAACAATACCAGAACCTTGCTG--------AGGAAAGAGAAAGGAT--CCTTCGAT--CAGGAGGAATATC
```

Section 109

```
                                         8965 8970      8980      8990      9000      9010      9020      9030              9047
                                 (8965)
SARS-CoV-2 Reference Genome NC_045512.2 (8842) AAGAGAAGTGGGTTTTGTCGTGCCTGGTTTGC-CTGGCACGATATTACGCACAACTAATGGTGACTTTTTGCATTTCTTACCT
      Fig badnavirus 1 NC_017830.1 (6360) TTTTGCCAGAGACAATGAATTCAGAAAACAACTCAGGGAGGTT-----GAAGAAGAATCTGCAAGAAGAGCCATTAAAGCCCT
```

Section 110

```
                                         9048        9060      9070      9080      9090      9100      9110      9120      9130
                                 (9048)
SARS-CoV-2 Reference Genome NC_045512.2 (8924) AGAGTTTTTAGTGCAGTTGGTAACATCTGTTACACACCATCAAAACTTATAGAGTACACTGACTTTGCAACATCAGCTTGTGT
      Fig badnavirus 1 NC_017830.1 (6438) GGAACAATACAGGCAGATTCACACACTTAAAACAGCCCAATGTGCATCATGGAGTTCACCCGGAAGGGATGGGAATTATTGGT
```

Section 111

```
                                         9131     9140      9150      9160      9170      9180      9190      9200          9213
                                 (9131)
SARS-CoV-2 Reference Genome NC_045512.2 (9007) TT---TGGCTGCTGAATGT-ACAATTTTTAAAGATGCTTCTGGTAAGCCAGTACCATATTGTTATGATACCAATG-TACTAGA
      Fig badnavirus 1 NC_017830.1 (6521) CTGATTGCTTGCCAAGCGTTAGGCGATATGACGCAAGATTAGAAAAGGTCCTACAAGACTTGAAGGACGCGTCAGCTGACAGC
```

Section 112

```
                                         9214  9220      9230      9240      9250      9260      9270      9280              9296
                                 (9214)
SARS-CoV-2 Reference Genome NC_045512.2 (9085) AGGTTCTGTTGCTTATGAAAGTTTACGCCCTGACACACGTTATGTGCTCATGGATGGCTCTATTATTCAATTTCCTAACACCT
      Fig badnavirus 1 NC_017830.1 (6604) CTAAAGTTCCGCATTTGAAGCATGATGGGCCCAGAAAAGTGGT----TGAGGCGTCTCACTATAAT-CGATGCCCTTATCTTT
```

SARS-CoV-2 & Fig badnavirus

Section	Sequence	Start	Alignment
Section 113		(9297)	9297 … 9379
	SARS-CoV-2 Reference Genome NC_045512.2	(9168)	ACCTTGAAGGTTCTGTTAGAGTGGTAACAACTTTTGATTCTGAGTACTGTAGGCACGGCACTTGTGAAA-GATCAGAAGCTGG
	Fig badnavirus 1 NC_017830.1	(6682)	TCCTTTGTCATTTTGTAATAATGGAATCGTCTTTTTGACCCAAAGGCTGCCGTC------CATGTGCAGTGATTC----CTTT
Section 114		(9380)	9380 … 9462
	SARS-CoV-2 Reference Genome NC_045512.2	(9250)	TGTTTGTGTATCTACTAGTGGTAGATGG---GTACTTAACAATGATTATTACAGATCTTTACCAGGAGTTTTCTGTGGTGTAG
	Fig badnavirus 1 NC_017830.1	(6755)	AGTTTGCGTGTTTTATCCGCTTACACGGATTGTCCTTTGTAACCACTTTCACGTGGTTTTGC------TTTTCTTTAGTGCGC
Section 115		(9463)	9463 … 9545
	SARS-CoV-2 Reference Genome NC_045512.2	(9330)	ATGCTGTAAATT-TACTTACTAATATGTTTACACCACTAATTCAACCTATTGGTGCTTTGGACATATCAGCATCTATAGTAGC
	Fig badnavirus 1 NC_017830.1	(6832)	CTCTTTTGCGCCCTTGTTTTTAGCGTTAGTACGCTTTGAGTCAAGTTTCCTTATCATTTGGGAATCTTTTGAGCTGTCGATG-
Section 116		(9546)	9546 … 9628
	SARS-CoV-2 Reference Genome NC_045512.2	(9412)	TGGTGGTATTGTAGCTATCGTAGTAACATGCCTTGCCTACTATTTTATGAGGTTTAGAAGAGCTTTTGGTGAATACAGTCATG
	Fig badnavirus 1 NC_017830.1	(6914)	-GGGCCCAATGAAGCACCCG-----AGCTCTTTTAATTCCTATATAAGGAGCTTTAGTTTCG-TTGCAAGGCACACAACAATC
Section 117		(9629)	9629 … 9711
	SARS-CoV-2 Reference Genome NC_045512.2	(9495)	TAGTTGCCTTTAATACTTTACTATTCCTATGTCATTCACTGTACTCTGTTTAACACCAGTTTACTCATTCTTACCTGGTGTT
	Fig badnavirus 1 NC_017830.1	(6990)	AAGCAACCCTTGAGCCTT-ACTTCGAAAAGAGTCTGTAAAAGTTTAGAGTCTGTAATCAGTGTG--AAGTTTGAAGAAATAAA
Section 118		(9712)	9712 … 9794
	SARS-CoV-2 Reference Genome NC_045512.2	(9578)	TATTCTGTTATTTACTTGTACTTGACATTT-TATCTTACTAATGATGTTTCTTTTTTAGCACATATTCAGTGGATGGTTATGT
	Fig badnavirus 1 NC_017830.1	(7070)	TTAGAGGTAATTCAGTTTAAAATCTCTTTTATTTCTTGTTTTCGATCCATTTTATTTGGGGACACACAAA-------------
Section 119		(9795)	9795 … 9877
	SARS-CoV-2 Reference Genome NC_045512.2	(9660)	TCACACCTTTAGTACCTTTCTGGATAACAATTGCTTATATCATTTGTATTTCCACAAAGCATTTCTATTGGTTCTTTAGTAAT
	Fig badnavirus 1 NC_017830.1	(7141)	---

```
                                                                                                      Section 120
                                          (9878)  9878        9890      9900      9910      9920      9930      9940      9950     9960
SARS-CoV-2 Reference Genome NC_045512.2   (9743)  TACCTAAAGAGACGTGTAGTCTTTAATGGTGTTTCCTTTAGTACTTTTGAAGAAGCTGCGCTGTGCACCTTTTTGTTAAATAA
     Fig badnavirus 1 NC_017830.1         (7141)  -----------------------------------------------------------------------------------
                                                                                                      Section 121
                                          (9961)  9961     9970      9980      9990      10000     10010     10020     10030    10043
SARS-CoV-2 Reference Genome NC_045512.2   (9826)  AGAAATGTATCTAAAGTTGCGTAGTGATGTGCTATTACCTCTTACGCAATATAATAGATACTTAGCTCTTTATAATAAGTACA
     Fig badnavirus 1 NC_017830.1         (7141)  -----------------------------------------------------------------------------------
                                                                                                      Section 122
                                         (10044)  10044 10050     10060     10070     10080     10090     10100     10110         10126
SARS-CoV-2 Reference Genome NC_045512.2   (9909)  AGTATTTTAGTGGAGCAATGGATACAACTAGCTACAGAGAAGCTGCTTGTTGTCATCTCGCAAAGGCTCTCAATGACTTCAGT
     Fig badnavirus 1 NC_017830.1         (7141)  -----------------------------------------------------------------------------------
                                                                                                      Section 123
                                         (10127)  10127        10140     10150     10160     10170     10180     10190         10209
SARS-CoV-2 Reference Genome NC_045512.2   (9992)  AACTCAGGTTCTGATGTTCTTTACCAACCACCACAAACCTCTATCACCTCAGCTGTTTTGCAGAGTGGTTTTAGAAAAATGGC
     Fig badnavirus 1 NC_017830.1         (7141)  -----------------------------------------------------------------------------------
                                                                                                      Section 124
                                         (10210)  10210     10220     10230     10240     10250     10260     10270     10280     10292
SARS-CoV-2 Reference Genome NC_045512.2  (10075)  ATTCCCATCTGGTAAAGTTGAGGGTTGTATGGTACAAGTAACTTGTGGTACAACTACACTTAACGGTCTTTGGCTTGATGACG
     Fig badnavirus 1 NC_017830.1         (7141)  -----------------------------------------------------------------------------------
                                                                                                      Section 125
                                         (10293)  10293  10300     10310     10320     10330     10340     10350     10360         10375
SARS-CoV-2 Reference Genome NC_045512.2  (10158)  TAGTTTACTGTCCAAGACATGTGATCTGCACCTCTGAAGACATGCTTAACCCTAATTATGAAGATTTACTCATTCGTAAGTCT
     Fig badnavirus 1 NC_017830.1         (7141)  -----------------------------------------------------------------------------------
                                                                                                      Section 126
                                         (10376)  10376         10390     10400     10410     10420     10430     10440             10458
SARS-CoV-2 Reference Genome NC_045512.2  (10241)  AATCATAATTTCTTGGTACAGGCTGGTAATGTTCAACTCAGGGTTATTGGACATTCTATGCAAAATTGTGTACTTAAGCTTAA
     Fig badnavirus 1 NC_017830.1         (7141)  -----------------------------------------------------------------------------------
```

```
                                                                                                   Section 127
                                          (10459) 10459      10470     10480     10490     10500     10510     10520     10530 10541
SARS-CoV-2 Reference Genome NC_045512.2 (10324) GGTTGATACAGCCAATCCTAAGACACCTAAGTATAAGTTTGTTCGCATTCAACCAGGACAGACTTTTTCAGTGTTAGCTTGTT
         Fig badnavirus 1 NC_017830.1   (7141) -----------------------------------------------------------------------------------
                                                                                                   Section 128
                                          (10542) 10542   10550     10560     10570     10580     10590     10600     10610     10624
SARS-CoV-2 Reference Genome NC_045512.2 (10407) ACAATGGTTCACCATCTGGTGTTTACCAATGTGCTATGAGGCCCAATTTCACTATTAAGGGTTCATTCCTTAATGGTTCATGT
         Fig badnavirus 1 NC_017830.1   (7141) -----------------------------------------------------------------------------------
                                                                                                   Section 129
                                          (10625) 10625 10630     10640     10650     10660     10670     10680     10690         10707
SARS-CoV-2 Reference Genome NC_045512.2 (10490) GGTAGTGTTGGTTTTAACATAGATTATGACTGTGTCTCTTTTTGTTACATGCACCATATGGAATTACCAACTGGAGTTCATGC
         Fig badnavirus 1 NC_017830.1   (7141) -----------------------------------------------------------------------------------
                                                                                                   Section 130
                                          (10708) 10708       10720     10730     10740     10750     10760     10770     10780 10790
SARS-CoV-2 Reference Genome NC_045512.2 (10573) TGGCACAGACTTAGAAGGTAACTTTTATGGACCTTTTGTTGACAGGCAAACAGCACAAGCAGCTGGTACGGACACAACTATTA
         Fig badnavirus 1 NC_017830.1   (7141) -----------------------------------------------------------------------------------
                                                                                                   Section 131
                                          (10791) 10791    10800     10810     10820     10830     10840     10850     10860     10873
SARS-CoV-2 Reference Genome NC_045512.2 (10656) CAGTTAATGTTTTAGCTTGGTTGTACGCTGCTGTTATAAATGGAGACAGGTGGTTTCTCAATCGATTTACCACAACTCTTAAT
         Fig badnavirus 1 NC_017830.1   (7141) -----------------------------------------------------------------------------------
                                                                                                   Section 132
                                          (10874) 10874 10880     10890     10900     10910     10920     10930     10940         10956
SARS-CoV-2 Reference Genome NC_045512.2 (10739) GACTTTAACCTTGTGGCTATGAAGTACAATTATGAACCTCTAACACAAGACCATGTTGACATACTAGGACCTCTTTCTGCTCA
         Fig badnavirus 1 NC_017830.1   (7141) -----------------------------------------------------------------------------------
                                                                                                   Section 133
                                          (10957) 10957        10970     10980     10990     11000     11010     11020         11039
SARS-CoV-2 Reference Genome NC_045512.2 (10822) AACTGGAATTGCCGTTTTAGATATGTGTGCTTCATTAAAAGAATTACTGCAAAATGGTATGAATGGACGTACCATATTGGGTA
         Fig badnavirus 1 NC_017830.1   (7141) -----------------------------------------------------------------------------------
```

SARS-CoV-2 & Fig badnavirus

```
                                                                                                         Section 134
                                        (11040) 11040     11050     11060     11070     11080     11090     11100     11110   11122
SARS-CoV-2 Reference Genome NC_045512.2 (10905) GTGCTTTATTAGAAGATGAATTTACACCTTTTGATGTTGTTAGACAATGCTCAGGTGTTACTTTCCAAAGTGCAGTGAAAAGA
        Fig badnavirus 1 NC_017830.1     (7141) -----------------------------------------------------------------------------------
                                                                                                         Section 135
                                        (11123) 11123  11130     11140     11150     11160     11170     11180     11190          11205
SARS-CoV-2 Reference Genome NC_045512.2 (10988) ACAATCAAGGGTACACACCACTGGTTGTTACTCACAATTTTGACTTCACTTTTAGTTTTAGTCCAGAGTACTCAATGGTCTTT
        Fig badnavirus 1 NC_017830.1     (7141) -----------------------------------------------------------------------------------
                                                                                                         Section 136
                                        (11206) 11206         11220     11230     11240     11250     11260     11270           11288
SARS-CoV-2 Reference Genome NC_045512.2 (11071) GTTCTTTTTTTTGTATGAAAATGCCTTTTTACCTTTTGCTATGGGTATTATTGCTATGTCTGCTTTTGCAATGATGTTTGTCA
        Fig badnavirus 1 NC_017830.1     (7141) -----------------------------------------------------------------------------------
                                                                                                         Section 137
                                        (11289) 11289      11300     11310     11320     11330     11340     11350     11360   11371
SARS-CoV-2 Reference Genome NC_045512.2 (11154) AACATAAGCATGCATTTCTCTGTTTGTTTTTGTTACCTTCTCTTGCCACTGTAGCTTATTTTAATATGGTCTATATGCCTGCT
        Fig badnavirus 1 NC_017830.1     (7141) -----------------------------------------------------------------------------------
                                                                                                         Section 138
                                        (11372) 11372   11380     11390     11400     11410     11420     11430     11440         11454
SARS-CoV-2 Reference Genome NC_045512.2 (11237) AGTTGGGTGATGCGTATTATGACATGGTTGGATATGGTTGATACTAGTTTGTCTGGTTTTAAGCTAAAAGACTGTGTTATGTA
        Fig badnavirus 1 NC_017830.1     (7141) -----------------------------------------------------------------------------------
                                                                                                         Section 139
                                        (11455) 11455 11460     11470     11480     11490     11500     11510     11520           11537
SARS-CoV-2 Reference Genome NC_045512.2 (11320) TGCATCAGCTGTAGTGTTACTAATCCTTATGACAGCAAGAACTGTGTATGATGATGGTGCTAGGAGAGTGTGGACACTTATGA
        Fig badnavirus 1 NC_017830.1     (7141) -----------------------------------------------------------------------------------
                                                                                                         Section 140
                                        (11538) 11538       11550     11560     11570     11580     11590     11600     11610 11620
SARS-CoV-2 Reference Genome NC_045512.2 (11403) ATGTCTTGACACTCGTTTATAAAGTTTATTATGGTAATGCTTTAGATCAAGCCATTTCCATGTGGGCTCTTATAATCTCTGTT
        Fig badnavirus 1 NC_017830.1     (7141) -----------------------------------------------------------------------------------
```

Section 141

```
                                           (11621) 11621   11630     11640     11650     11660     11670     11680     11690       11703
SARS-CoV-2 Reference Genome NC_045512.2 (11486) ACTTCTAACTACTCAGGTGTAGTTACAACTGTCATGTTTTTGGCCAGAGGTATTGTTTTTATGTGTGTTGAGTATTGCCCTAT
          Fig badnavirus 1 NC_017830.1   (7141) -----------------------------------------------------------------------------------
```

Section 142

```
                                           (11704) 11704 11710     11720     11730     11740     11750     11760     11770       11786
SARS-CoV-2 Reference Genome NC_045512.2 (11569) TTTCTTCATAACTGGTAATACACTTCAGTGTATAATGCTAGTTTATTGTTTCTTAGGCTATTTTTGTACTTGTTACTTTGGCC
          Fig badnavirus 1 NC_017830.1   (7141) -----------------------------------------------------------------------------------
```

Section 143

```
                                           (11787) 11787        11800     11810     11820     11830     11840     11850           11869
SARS-CoV-2 Reference Genome NC_045512.2 (11652) TCTTTTGTTTACTCAACCGCTACTTTAGACTGACTCTTGGTGTTTATGATTACTTAGTTTCTACACAGGAGTTTAGATATATG
          Fig badnavirus 1 NC_017830.1   (7141) -----------------------------------------------------------------------------------
```

Section 144

```
                                           (11870) 11870     11880     11890     11900     11910     11920     11930     11940   11952
SARS-CoV-2 Reference Genome NC_045512.2 (11735) AATTCACAGGGACTACTCCCACCCAAGAATAGCATAGATGCCTTCAAACTCAACATTAAATTGTTGGGTGTTGGTGGCAAACC
          Fig badnavirus 1 NC_017830.1   (7141) -----------------------------------------------------------------------------------
```

Section 145

```
                                           (11953) 11953  11960     11970     11980     11990     12000     12010     12020       12035
SARS-CoV-2 Reference Genome NC_045512.2 (11818) TTGTATCAAAGTAGCCACTGTACAGTCTAAAATGTCAGATGTAAAGTGCACATCAGTAGTCTTACTCTCAGTTTTGCAACAAC
          Fig badnavirus 1 NC_017830.1   (7141) -----------------------------------------------------------------------------------
```

Section 146

```
                                           (12036) 12036         12050     12060     12070     12080     12090     12100          12118
SARS-CoV-2 Reference Genome NC_045512.2 (11901) TCAGAGTAGAATCATCATCTAAATTGTGGGCTCAATGTGTCCAGTTACACAATGACATTCTCTTAGCTAAAGATACTACTGAA
          Fig badnavirus 1 NC_017830.1   (7141) -----------------------------------------------------------------------------------
```

Section 147

```
                                           (12119) 12119      12130     12140     12150     12160     12170     12180     12190   12201
SARS-CoV-2 Reference Genome NC_045512.2 (11984) GCCTTTGAAAAAATGGTTTCACTACTTTCTGTTTTGCTTTCCATGCAGGGTGCTGTAGACATAAACAAGCTTTGTGAAGAAAT
          Fig badnavirus 1 NC_017830.1   (7141) -----------------------------------------------------------------------------------
```

```
Section 148
                                          (12202) 12202 12210 12220 12230 12240 12250 12260 12270 12284
SARS-CoV-2 Reference Genome NC_045512.2 (12067) GCTGGACAACAGGGCAACCTTACAAGCTATAGCCTCAGAGTTTAGTTCCCTTCCATCATATGCAGCTTTTGCTACTGCTCAAG
         Fig badnavirus 1 NC_017830.1   (7141) -----------------------------------------------------------------------------------

Section 149
                                          (12285) 12285 12290 12300 12310 12320 12330 12340 12350 12367
SARS-CoV-2 Reference Genome NC_045512.2 (12150) AAGCTTATGAGCAGGCTGTTGCTAATGGTGATTCTGAAGTTGTTCTTAAAAAGTTGAAGAAGTCTTTGAATGTGGCTAAATCT
         Fig badnavirus 1 NC_017830.1   (7141) -----------------------------------------------------------------------------------

Section 150
                                          (12368) 12368 12380 12390 12400 12410 12420 12430 12440 12450
SARS-CoV-2 Reference Genome NC_045512.2 (12233) GAATTTGACCGTGATGCAGCCATGCAACGTAAGTTGGAAAAGATGGCTGATCAAGCTATGACCCAAATGTATAAACAGGCTAG
         Fig badnavirus 1 NC_017830.1   (7141) -----------------------------------------------------------------------------------

Section 151
                                          (12451) 12451 12460 12470 12480 12490 12500 12510 12520 12533
SARS-CoV-2 Reference Genome NC_045512.2 (12316) ATCTGAGGACAAGAGGGCAAAAGTTACTAGTGCTATGCAGACAATGCTTTTCACTATGCTTAGAAAGTTGGATAATGATGCAC
         Fig badnavirus 1 NC_017830.1   (7141) -----------------------------------------------------------------------------------

Section 152
                                          (12534) 12534 12540 12550 12560 12570 12580 12590 12600 12616
SARS-CoV-2 Reference Genome NC_045512.2 (12399) TCAACAACATTATCAACAATGCAAGAGATGGTTGTGTTCCCTTGAACATAATACCTCTTACAACAGCAGCCAAACTAATGGTT
         Fig badnavirus 1 NC_017830.1   (7141) -----------------------------------------------------------------------------------

Section 153
                                          (12617) 12617 12630 12640 12650 12660 12670 12680 12699
SARS-CoV-2 Reference Genome NC_045512.2 (12482) GTCATACCAGACTATAACACATATAAAAATACGTGTGATGGTACAACATTTACTTATGCATCAGCATTGTGGGAAATCCAACA
         Fig badnavirus 1 NC_017830.1   (7141) -----------------------------------------------------------------------------------

Section 154
                                          (12700) 12700 12710 12720 12730 12740 12750 12760 12770 12782
SARS-CoV-2 Reference Genome NC_045512.2 (12565) GGTTGTAGATGCAGATAGTAAAATTGTTCAACTTAGTGAAATTAGTATGGACAATTCACCTAATTTAGCATGGCCTCTTATTG
         Fig badnavirus 1 NC_017830.1   (7141) -----------------------------------------------------------------------------------
```

```
                                                                                                                          Section 155
                                          (12783) 12783  12790          12800          12810          12820          12830          12840          12850            12865
SARS-CoV-2 Reference Genome NC_045512.2 (12648) TAACAGCTTTAAGGGCCAATTCTGCTGTCAAATTACAGAATAATGAGCTTAGTCCTGTTGCACTACGACAGATGTCTTGTGCT
          Fig badnavirus 1 NC_017830.1   (7141) -----------------------------------------------------------------------------------
                                                                                                                          Section 156
                                          (12866) 12866             12880          12890          12900          12910          12920          12930            12948
SARS-CoV-2 Reference Genome NC_045512.2 (12731) GCCGGTACTACACAAACTGCTTGCACTGATGACAATGCGTTAGCTTACTACAACACAACAAAGGGAGGTAGGTTTGTACTTGC
          Fig badnavirus 1 NC_017830.1   (7141) -----------------------------------------------------------------------------------
                                                                                                                          Section 157
                                          (12949) 12949          12960          12970          12980          12990          13000          13010          13020     13031
SARS-CoV-2 Reference Genome NC_045512.2 (12814) ACTGTTATCCGATTTACAGGATTTGAAATGGGCTAGATTCCCTAAGAGTGATGGAACTGGTACTATCTATACAGAACTGGAAC
          Fig badnavirus 1 NC_017830.1   (7141) -----------------------------------------------------------------------------------
                                                                                                                          Section 158
                                          (13032) 13032   13040          13050          13060          13070          13080          13090          13100          13114
SARS-CoV-2 Reference Genome NC_045512.2 (12897) CACCTTGTAGGTTTGTTACAGACACACCTAAAGGTCCTAAAGTGAAGTATTTATACTTTATTAAAGGATTAAACAACCTAAAT
          Fig badnavirus 1 NC_017830.1   (7141) -----------------------------------------------------------------------------------
                                                                                                                          Section 159
                                          (13115) 13115 13120          13130          13140          13150          13160          13170          13180            13197
SARS-CoV-2 Reference Genome NC_045512.2 (12980) AGAGGTATGGTACTTGGTAGTTTAGCTGCCACAGTACGTCTACAAGCTGGTAATGCAACAGAAGTGCCTGCCAATTCAACTGT
          Fig badnavirus 1 NC_017830.1   (7141) -----------------------------------------------------------------------------------
                                                                                                                          Section 160
                                          (13198) 13198           13210          13220          13230          13240          13250          13260          13270     13280
SARS-CoV-2 Reference Genome NC_045512.2 (13063) ATTATCTTTCTGTGCTTTTGCTGTAGATGCTGCTAAAGCTTACAAAGATTATCTAGCTAGTGGGGGACAACCAATCACTAATT
          Fig badnavirus 1 NC_017830.1   (7141) -----------------------------------------------------------------------------------
                                                                                                                          Section 161
                                          (13281) 13281    13290          13300          13310          13320          13330          13340          13350          13363
SARS-CoV-2 Reference Genome NC_045512.2 (13146) GTGTTAAGATGTTGTGTACACACACTGGTACTGGTCAGGCAATAACAGTTACACCGGAAGCCAATATGGATCAAGAATCCTTT
          Fig badnavirus 1 NC_017830.1   (7141) -----------------------------------------------------------------------------------
```

```
                                                                                                          Section 162
                                          (13364) 13364 13370     13380     13390     13400     13410     13420     13430           13446
SARS-CoV-2 Reference Genome NC_045512.2 (13229) GGTGGTGCATCGTGTTGTCTGTACTGCCGTTGCCACATAGATCATCCAAATCCTAAAGGATTTTGTGACTTAAAAGGTAAGTA
          Fig badnavirus 1 NC_017830.1   (7141) -----------------------------------------------------------------------------------
                                                                                                          Section 163
                                          (13447) 13447        13460     13470     13480     13490     13500     13510               13529
SARS-CoV-2 Reference Genome NC_045512.2 (13312) TGTACAAATACCTACAACTTGTGCTAATGACCCTGTGGGTTTTACACTTAAAAACACAGTCTGTACCGTCTGCGGTATGTGGA
          Fig badnavirus 1 NC_017830.1   (7141) -----------------------------------------------------------------------------------
                                                                                                          Section 164
                                          (13530) 13530     13540     13550     13560     13570     13580     13590     13600       13612
SARS-CoV-2 Reference Genome NC_045512.2 (13395) AAGGTTATGGCTGTAGTTGTGATCAACTCCGCGAACCCATGCTTCAGTCAGCTGATGCACAATCGTTTTTAAACGGGTTTGCG
          Fig badnavirus 1 NC_017830.1   (7141) -----------------------------------------------------------------------------------
                                                                                                          Section 165
                                          (13613) 13613  13620     13630     13640     13650     13660     13670     13680           13695
SARS-CoV-2 Reference Genome NC_045512.2 (13478) GTGTAAGTGCAGCCCGTCTTACACCGTGCGGCACAGGCACTAGTACTGATGTCGTATACAGGGCTTTTGACATCTACAATGAT
          Fig badnavirus 1 NC_017830.1   (7141) -----------------------------------------------------------------------------------
                                                                                                          Section 166
                                          (13696) 13696         13710     13720     13730     13740     13750     13760              13778
SARS-CoV-2 Reference Genome NC_045512.2 (13561) AAAGTAGCTGGTTTTGCTAAATTCCTAAAAACTAATTGTTGTCGCTTCCAAGAAAAGGACGAAGATGACAATTTAATTGATTC
          Fig badnavirus 1 NC_017830.1   (7141) -----------------------------------------------------------------------------------
                                                                                                          Section 167
                                          (13779) 13779      13790     13800     13810     13820     13830     13840     13850      13861
SARS-CoV-2 Reference Genome NC_045512.2 (13644) TTACTTTGTAGTTAAGAGACACACTTTCTCTAACTACCAACATGAAGAAACAATTTATAATTTACTTAAGGATTGTCCAGCTG
          Fig badnavirus 1 NC_017830.1   (7141) -----------------------------------------------------------------------------------
                                                                                                          Section 168
                                          (13862) 13862   13870     13880     13890     13900     13910     13920     13930           13944
SARS-CoV-2 Reference Genome NC_045512.2 (13727) TTGCTAAACATGACTTCTTTAAGTTTAGAATAGACGGTGACATGGTACCACATATATCACGTCAACGTCTTACTAAATACACA
          Fig badnavirus 1 NC_017830.1   (7141) -----------------------------------------------------------------------------------
```

Section 169

```
                                          (13945) 13945 13950     13960     13970     13980     13990     14000     14010            14027
SARS-CoV-2 Reference Genome NC_045512.2 (13810) ATGGCAGACCTCGTCTATGCTTTAAGGCATTTTGATGAAGGTAATTGTGACACATTAAAAGAAATACTTGTCACATACAATTG
      Fig badnavirus 1 NC_017830.1  (7141) -----------------------------------------------------------------------------------
```

Section 170

```
                                          (14028) 14028        14040     14050     14060     14070     14080     14090     14100   14110
SARS-CoV-2 Reference Genome NC_045512.2 (13893) TTGTGATGATGATTATTTCAATAAAAAGGACTGGTATGATTTTGTAGAAAACCCAGATATATTACGCGTATACGCCAACTTAG
      Fig badnavirus 1 NC_017830.1  (7141) -----------------------------------------------------------------------------------
```

Section 171

```
                                          (14111) 14111    14120     14130     14140     14150     14160     14170     14180       14193
SARS-CoV-2 Reference Genome NC_045512.2 (13976) GTGAACGTGTACGCCAAGCTTTGTTAAAAACAGTACAATTCTGTGATGCCATGCGAAATGCTGGTATTGTTGGTGTACTGACA
      Fig badnavirus 1 NC_017830.1  (7141) -----------------------------------------------------------------------------------
```

Section 172

```
                                          (14194) 14194 14200     14210     14220     14230     14240     14250     14260            14276
SARS-CoV-2 Reference Genome NC_045512.2 (14059) TTAGATAATCAAGATCTCAATGGTAACTGGTATGATTTCGGTGATTTCATACAAACCACGCCAGGTAGTGGAGTTCCTGTTGT
      Fig badnavirus 1 NC_017830.1  (7141) -----------------------------------------------------------------------------------
```

Section 173

```
                                          (14277) 14277         14290     14300     14310     14320     14330     14340            14359
SARS-CoV-2 Reference Genome NC_045512.2 (14142) AGATTCTTATTATTCATTGTTAATGCCTATATTAACCTTGACCAGGGCTTTAACTGCAGAGTCACATGTTGACACTGACTTAA
      Fig badnavirus 1 NC_017830.1  (7141) -----------------------------------------------------------------------------------
```

Section 174

```
                                          (14360) 14360     14370     14380     14390     14400     14410     14420     14430       14442
SARS-CoV-2 Reference Genome NC_045512.2 (14225) CAAAGCCTTACATTAAGTGGGATTTGTTAAAATATGACTTCACGGAAGAGAGGTTAAAACTCTTTGACCGTTATTTTAAATAT
      Fig badnavirus 1 NC_017830.1  (7141) -----------------------------------------------------------------------------------
```

Section 175

```
                                          (14443) 14443  14450     14460     14470     14480     14490     14500     14510          14525
SARS-CoV-2 Reference Genome NC_045512.2 (14308) TGGGATCAGACATACCACCCAAATTGTGTTAACTGTTTGGATGACAGATGCATTCTGCATTGTGCAAACTTTAATGTTTTATT
      Fig badnavirus 1 NC_017830.1  (7141) -----------------------------------------------------------------------------------
```

```
                                                   Section 176
                                        (14526) 14526         14540     14550     14560     14570     14580     14590              14608
SARS-CoV-2 Reference Genome NC_045512.2 (14391) CTCTACAGTGTTCCCACCTACAAGTTTTGGACCACTAGTGAGAAAAATATTTGTTGATGGTGTTCCATTTGTAGTTTCAACTG
            Fig badnavirus 1 NC_017830.1 (7141) -----------------------------------------------------------------------------------
                                                   Section 177
                                        (14609) 14609      14620     14630     14640     14650     14660     14670     14680     14691
SARS-CoV-2 Reference Genome NC_045512.2 (14474) GATACCACTTCAGAGAGCTAGGTGTTGTACATAATCAGGATGTAAACTTACATAGCTCTAGACTTAGTTTTAAGGAATTACTT
            Fig badnavirus 1 NC_017830.1 (7141) -----------------------------------------------------------------------------------
                                                   Section 178
                                        (14692) 14692   14700     14710     14720     14730     14740     14750     14760          14774
SARS-CoV-2 Reference Genome NC_045512.2 (14557) GTGTATGCTGCTGACCCTGCTATGCACGCTGCTTCTGGTAATCTATTACTAGATAAACGCACTACGTGCTTTTCAGTAGCTGC
            Fig badnavirus 1 NC_017830.1 (7141) -----------------------------------------------------------------------------------
                                                   Section 179
                                        (14775) 14775 14780     14790     14800     14810     14820     14830     14840            14857
SARS-CoV-2 Reference Genome NC_045512.2 (14640) ACTTACTAACAATGTTGCTTTTCAAACTGTCAAACCCGGTAATTTTAACAAAGACTTCTATGACTTTGCTGTGTCTAAGGGTT
            Fig badnavirus 1 NC_017830.1 (7141) -----------------------------------------------------------------------------------
                                                   Section 180
                                        (14858) 14858       14870     14880     14890     14900     14910     14920     14930    14940
SARS-CoV-2 Reference Genome NC_045512.2 (14723) TCTTTAAGGAAGGAAGTTCTGTTGAATTAAAACACTTCTTCTTTGCTCAGGATGGTAATGCTGCTATCAGCGATTATGACTAC
            Fig badnavirus 1 NC_017830.1 (7141) -----------------------------------------------------------------------------------
                                                   Section 181
                                        (14941) 14941    14950     14960     14970     14980     14990     15000     15010         15023
SARS-CoV-2 Reference Genome NC_045512.2 (14806) TATCGTTATAATCTACCAACAATGTGTGATATCAGACAACTACTATTTGTAGTTGAAGTTGTTGATAAGTACTTTGATTGTTA
            Fig badnavirus 1 NC_017830.1 (7141) -----------------------------------------------------------------------------------
                                                   Section 182
                                        (15024) 15024 15030     15040     15050     15060     15070     15080     15090            15106
SARS-CoV-2 Reference Genome NC_045512.2 (14889) CGATGGTGGCTGTATTAATGCTAACCAAGTCATCGTCAACAACCTAGACAAATCAGCTGGTTTTCCATTTAATAAATGGGGTA
            Fig badnavirus 1 NC_017830.1 (7141) -----------------------------------------------------------------------------------
```

```
                                                                                        Section 183
                                            (15107) 15107        15120     15130     15140     15150     15160     15170             15189
SARS-CoV-2 Reference Genome NC_045512.2 (14972) AGGCTAGACTTTATTATGATTCAATGAGTTATGAGGATCAAGATGCACTTTTCGCATATACAAAACGTAATGTCATCCCTACT
          Fig badnavirus 1 NC_017830.1   (7141) -----------------------------------------------------------------------------------
                                                                                        Section 184
                                            (15190) 15190     15200     15210     15220     15230     15240     15250     15260     15272
SARS-CoV-2 Reference Genome NC_045512.2 (15055) ATAACTCAAATGAATCTTAAGTATGCCATTAGTGCAAAGAATAGAGCTCGCACCGTAGCTGGTGTCTCTATCTGTAGTACTAT
          Fig badnavirus 1 NC_017830.1   (7141) -----------------------------------------------------------------------------------
                                                                                        Section 185
                                            (15273) 15273  15280     15290     15300     15310     15320     15330     15340          15355
SARS-CoV-2 Reference Genome NC_045512.2 (15138) GACCAATAGACAGTTTCATCAAAAATTATTGAAATCAATAGCCGCCACTAGAGGAGCTACTGTAGTAATTGGAACAAGCAAAT
          Fig badnavirus 1 NC_017830.1   (7141) -----------------------------------------------------------------------------------
                                                                                        Section 186
                                            (15356) 15356         15370     15380     15390     15400     15410     15420             15438
SARS-CoV-2 Reference Genome NC_045512.2 (15221) TCTATGGTGGTTGGCACAACATGTTAAAAACTGTTTATAGTGATGTAGAAAACCCTCACCTTATGGGTTGGGATTATCCTAAA
          Fig badnavirus 1 NC_017830.1   (7141) -----------------------------------------------------------------------------------
                                                                                        Section 187
                                            (15439) 15439      15450     15460     15470     15480     15490     15500     15510   15521
SARS-CoV-2 Reference Genome NC_045512.2 (15304) TGTGATAGAGCCATGCCTAACATGCTTAGAATTATGGCCTCACTTGTTCTTGCTCGCAAACATACAACGTGTTGTAGCTTGTC
          Fig badnavirus 1 NC_017830.1   (7141) -----------------------------------------------------------------------------------
                                                                                        Section 188
                                            (15522) 15522   15530     15540     15550     15560     15570     15580     15590         15604
SARS-CoV-2 Reference Genome NC_045512.2 (15387) ACACCGTTTCTATAGATTAGCTAATGAGTGTGCTCAAGTATTGAGTGAAATGGTCATGTGTGGCGGTTCACTATATGTTAAAC
          Fig badnavirus 1 NC_017830.1   (7141) -----------------------------------------------------------------------------------
                                                                                        Section 189
                                            (15605) 15605 15610     15620     15630     15640     15650     15660     15670            15687
SARS-CoV-2 Reference Genome NC_045512.2 (15470) CAGGTGGAACCTCATCAGGAGATGCCACAACTGCTTATGCTAATAGTGTTTTTAACATTTGTCAAGCTGTCACGGCCAATGTT
          Fig badnavirus 1 NC_017830.1   (7141) -----------------------------------------------------------------------------------
```

Section 190

```
                                           (15688) 15688       15700     15710     15720     15730     15740     15750     15760     15770
SARS-CoV-2 Reference Genome NC_045512.2 (15553) AATGCACTTTTATCTACTGATGGTAACAAAATTGCCGATAAGTATGTCCGCAATTTACAACACAGACTTTATGAGTGTCTCTA
          Fig badnavirus 1 NC_017830.1   (7141) -----------------------------------------------------------------------------------
```

Section 191

```
                                           (15771) 15771    15780     15790     15800     15810     15820     15830     15840        15853
SARS-CoV-2 Reference Genome NC_045512.2 (15636) TAGAAATAGAGATGTTGACACAGACTTTGTGAATGAGTTTTACGCATATTTGCGTAAACATTTCTCAATGATGATACTCTCTG
          Fig badnavirus 1 NC_017830.1   (7141) -----------------------------------------------------------------------------------
```

Section 192

```
                                           (15854) 15854 15860     15870     15880     15890     15900     15910     15920           15936
SARS-CoV-2 Reference Genome NC_045512.2 (15719) ACGATGCTGTTGTGTGTTTCAATAGCACTTATGCATCTCAAGGTCTAGTGGCTAGCATAAAGAACTTTAAGTCAGTTCTTTAT
          Fig badnavirus 1 NC_017830.1   (7141) -----------------------------------------------------------------------------------
```

Section 193

```
                                           (15937) 15937        15950     15960     15970     15980     15990     16000              16019
SARS-CoV-2 Reference Genome NC_045512.2 (15802) TATCAAAACAATGTTTTTATGTCTGAAGCAAAATGTTGGACTGAGACTGACCTTACTAAAGGACCTCATGAATTTTGCTCTCA
          Fig badnavirus 1 NC_017830.1   (7141) -----------------------------------------------------------------------------------
```

Section 194

```
                                           (16020) 16020     16030     16040     16050     16060     16070     16080     16090       16102
SARS-CoV-2 Reference Genome NC_045512.2 (15885) ACATACAATGCTAGTTAAACAGGGTGATGATTATGTGTACCTTCCTTACCCAGATCCATCAAGAATCCTAGGGGCCGGCTGTT
          Fig badnavirus 1 NC_017830.1   (7141) -----------------------------------------------------------------------------------
```

Section 195

```
                                           (16103) 16103  16110     16120     16130     16140     16150     16160     16170          16185
SARS-CoV-2 Reference Genome NC_045512.2 (15968) TTGTAGATGATATCGTAAAAACAGATGGTACACTTATGATTGAACGGTTCGTGTCTTTAGCTATAGATGCTTACCCACTTACT
          Fig badnavirus 1 NC_017830.1   (7141) -----------------------------------------------------------------------------------
```

Section 196

```
                                           (16186) 16186         16200     16210     16220     16230     16240     16250               16268
SARS-CoV-2 Reference Genome NC_045512.2 (16051) AAACATCCTAATCAGGAGTATGCTGATGTCTTTCATTTGTACTTACAATACATAAGAAAGCTACATGATGAGTTAACAGGACA
          Fig badnavirus 1 NC_017830.1   (7141) -----------------------------------------------------------------------------------
```

```
                                                        Section 197
                                        (16269) 16269      16280      16290      16300      16310      16320      16330      16340      16351
SARS-CoV-2 Reference Genome NC_045512.2 (16134) CATGTTAGACATGTATTCTGTTATGCTTACTAATGATAACACTTCAAGGTATTGGGAACCTGAGTTTTATGAGGCTATGTACA
       Fig badnavirus 1 NC_017830.1      (7141) -----------------------------------------------------------------------------------
                                                        Section 198
                                        (16352) 16352   16360      16370      16380      16390      16400      16410      16420      16434
SARS-CoV-2 Reference Genome NC_045512.2 (16217) CACCGCATACAGTCTTACAGGCTGTTGGGGCTTGTGTTCTTTGCAATTCACAGACTTCATTAAGATGTGGTGCTTGCATACGT
       Fig badnavirus 1 NC_017830.1      (7141) -----------------------------------------------------------------------------------
                                                        Section 199
                                        (16435) 16435 16440      16450      16460      16470      16480      16490      16500      16517
SARS-CoV-2 Reference Genome NC_045512.2 (16300) AGACCATTCTTATGTTGTAAATGCTGTTACGACCATGTCATATCAACATCACATAAATTAGTCTTGTCTGTTAATCCGTATGT
       Fig badnavirus 1 NC_017830.1      (7141) -----------------------------------------------------------------------------------
                                                        Section 200
                                        (16518) 16518       16530      16540      16550      16560      16570      16580      16590   16600
SARS-CoV-2 Reference Genome NC_045512.2 (16383) TTGCAATGCTCCAGGTTGTGATGTCACAGATGTGACTCAACTTTACTTAGGAGGTATGAGCTATTATTGTAAATCACATAAAC
       Fig badnavirus 1 NC_017830.1      (7141) -----------------------------------------------------------------------------------
                                                        Section 201
                                        (16601) 16601    16610      16620      16630      16640      16650      16660      16670      16683
SARS-CoV-2 Reference Genome NC_045512.2 (16466) CACCCATTAGTTTTCCATTGTGTGCTAATGGACAAGTTTTTGGTTTATATAAAAATACATGTGTTGGTAGCGATAATGTTACT
       Fig badnavirus 1 NC_017830.1      (7141) -----------------------------------------------------------------------------------
                                                        Section 202
                                        (16684) 16684 16690      16700      16710      16720      16730      16740      16750      16766
SARS-CoV-2 Reference Genome NC_045512.2 (16549) GACTTTAATGCAATTGCAACATGTGACTGGACAAATGCTGGTGATTACATTTTAGCTAACACCTGTACTGAAAGACTCAAGCT
       Fig badnavirus 1 NC_017830.1      (7141) -----------------------------------------------------------------------------------
                                                        Section 203
                                        (16767) 16767         16780      16790      16800      16810      16820      16830      16849
SARS-CoV-2 Reference Genome NC_045512.2 (16632) TTTTGCAGCAGAAACGCTCAAAGCTACTGAGGAGACATTTAAACTGTCTTATGGTATTGCTACTGTACGTGAAGTGCTGTCTG
       Fig badnavirus 1 NC_017830.1      (7141) -----------------------------------------------------------------------------------
```

```
                                                                                                                                       Section 204
                                        (16850) 16850     16860     16870     16880     16890     16900     16910     16920      16932
SARS-CoV-2 Reference Genome NC_045512.2 (16715) ACAGAGAATTACATCTTTCATGGGAAGTTGGTAAACCTAGACCACCACTTAACCGAAATTATGTCTTTACTGGTTATCGTGTA
         Fig badnavirus 1 NC_017830.1    (7141) -----------------------------------------------------------------------------------
                                                                                                                                       Section 205
                                        (16933) 16933  16940     16950     16960     16970     16980     16990     17000          17015
SARS-CoV-2 Reference Genome NC_045512.2 (16798) ACTAAAAACAGTAAAGTACAAATAGGAGAGTACACCTTTGAAAAAGGTGACTATGGTGATGCTGTTGTTTACCGAGGTACAAC
         Fig badnavirus 1 NC_017830.1    (7141) -----------------------------------------------------------------------------------
                                                                                                                                       Section 206
                                        (17016) 17016         17030     17040     17050     17060     17070     17080              17098
SARS-CoV-2 Reference Genome NC_045512.2 (16881) AACTTACAAATTAAATGTTGGTGATTATTTTGTGCTGACATCACATACAGTAATGCCATTAAGTGCACCTACACTAGTGCCAC
         Fig badnavirus 1 NC_017830.1    (7141) -----------------------------------------------------------------------------------
                                                                                                                                       Section 207
                                        (17099) 17099      17110     17120     17130     17140     17150     17160     17170      17181
SARS-CoV-2 Reference Genome NC_045512.2 (16964) AAGAGCACTATGTTAGAATTACTGGCTTATACCCAACACTCAATATCTCAGATGAGTTTTCTAGCAATGTTGCAAATTATCAA
         Fig badnavirus 1 NC_017830.1    (7141) -----------------------------------------------------------------------------------
                                                                                                                                       Section 208
                                        (17182) 17182   17190     17200     17210     17220     17230     17240     17250          17264
SARS-CoV-2 Reference Genome NC_045512.2 (17047) AAGGTTGGTATGCAAAAGTATTCTACACTCCAGGGACCACCTGGTACTGGTAAGAGTCATTTTGCTATTGGCCTAGCTCTCTA
         Fig badnavirus 1 NC_017830.1    (7141) -----------------------------------------------------------------------------------
                                                                                                                                       Section 209
                                        (17265) 17265 17270     17280     17290     17300     17310     17320     17330              17347
SARS-CoV-2 Reference Genome NC_045512.2 (17130) CTACCCTTCTGCTCGCATAGTGTATACAGCTTGCTCTCATGCCGCTGTTGATGCACTATGTGAGAAGGCATTAAAATATTTGC
         Fig badnavirus 1 NC_017830.1    (7141) -----------------------------------------------------------------------------------
                                                                                                                                       Section 210
                                        (17348) 17348       17360     17370     17380     17390     17400     17410     17420      17430
SARS-CoV-2 Reference Genome NC_045512.2 (17213) CTATAGATAAATGTAGTAGAATTATACCTGCACGTGCTCGTGTAGAGTGTTTTGATAAATTCAAAGTGAATTCAACATTAGAA
         Fig badnavirus 1 NC_017830.1    (7141) -----------------------------------------------------------------------------------
```

```
                                                   Section 211
                                          (17431) 17431    17440     17450     17460     17470     17480     17490     17500        17513
SARS-CoV-2 Reference Genome NC_045512.2 (17296) CAGTATGTCTTTTGTACTGTAAATGCATTGCCTGAGACGACAGCAGATATAGTTGTCTTTGATGAAATTTCAATGGCCACAAA
       Fig badnavirus 1 NC_017830.1   (7141) -----------------------------------------------------------------------------------
                                                   Section 212
                                          (17514) 17514 17520     17530     17540     17550     17560     17570     17580          17596
SARS-CoV-2 Reference Genome NC_045512.2 (17379) TTATGATTTGAGTGTTGTCAATGCCAGATTACGTGCTAAGCACTATGTGTACATTGGCGACCCTGCTCAATTACCTGCACCAC
       Fig badnavirus 1 NC_017830.1   (7141) -----------------------------------------------------------------------------------
                                                   Section 213
                                          (17597) 17597         17610     17620     17630     17640     17650     17660            17679
SARS-CoV-2 Reference Genome NC_045512.2 (17462) GCACATTGCTAACTAAGGGCACACTAGAACCAGAATATTTCAATTCAGTGTGTAGACTTATGAAAACTATAGGTCCAGACATG
       Fig badnavirus 1 NC_017830.1   (7141) -----------------------------------------------------------------------------------
                                                   Section 214
                                          (17680) 17680     17690     17700     17710     17720     17730     17740     17750     17762
SARS-CoV-2 Reference Genome NC_045512.2 (17545) TTCCTCGGAACTTGTCGGCGTTGTCCTGCTGAAATTGTTGACACTGTGAGTGCTTTGGTTTATGATAATAAGCTTAAAGCACA
       Fig badnavirus 1 NC_017830.1   (7141) -----------------------------------------------------------------------------------
                                                   Section 215
                                          (17763) 17763  17770     17780     17790     17800     17810     17820     17830          17845
SARS-CoV-2 Reference Genome NC_045512.2 (17628) TAAAGACAAATCAGCTCAATGCTTTAAAATGTTTTATAAGGGTGTTATCACGCATGATGTTTCATCTGCAATTAACAGGCCAC
       Fig badnavirus 1 NC_017830.1   (7141) -----------------------------------------------------------------------------------
                                                   Section 216
                                          (17846) 17846          17860     17870     17880     17890     17900     17910          17928
SARS-CoV-2 Reference Genome NC_045512.2 (17711) AAATAGGCGTGGTAAGAGAATTCCTTACACGTAACCCTGCTTGGAGAAAAGCTGTCTTTATTTCACCTTATAATTCACAGAAT
       Fig badnavirus 1 NC_017830.1   (7141) -----------------------------------------------------------------------------------
                                                   Section 217
                                          (17929) 17929      17940     17950     17960     17970     17980     17990     18000  18011
SARS-CoV-2 Reference Genome NC_045512.2 (17794) GCTGTAGCCTCAAAGATTTTGGGACTACCAACTCAAACTGTTGATTCATCACAGGGCTCAGAATATGACTATGTCATATTCAC
       Fig badnavirus 1 NC_017830.1   (7141) -----------------------------------------------------------------------------------
```

SARS-CoV-2 & Fig badnavirus

```
                                                                                                 Section 218
                                          (18012) 18012   18020     18030     18040     18050     18060     18070     18080       18094
SARS-CoV-2 Reference Genome NC_045512.2 (17877) TCAAACCACTGAAACAGCTCACTCTTGTAATGTAAACAGATTTAATGTTGCTATTACCAGAGCAAAAGTAGGCATACTTTGCA
        Fig badnavirus 1 NC_017830.1    (7141) -----------------------------------------------------------------------------------
                                                                                                 Section 219
                                          (18095) 18095 18100     18110     18120     18130     18140     18150     18160         18177
SARS-CoV-2 Reference Genome NC_045512.2 (17960) TAATGTCTGATAGAGACCTTTATGACAAGTTGCAATTTACAAGTCTTGAAATTCCACGTAGGAATGTGGCAACTTTACAAGCT
        Fig badnavirus 1 NC_017830.1    (7141) -----------------------------------------------------------------------------------
                                                                                                 Section 220
                                          (18178) 18178       18190     18200     18210     18220     18230     18240     18250 18260
SARS-CoV-2 Reference Genome NC_045512.2 (18043) GAAAATGTAACAGGACTCTTTAAAGATTGTAGTAAGGTAATCACTGGGTTACATCCTACACAGGCACCTACACACCTCAGTGT
        Fig badnavirus 1 NC_017830.1    (7141) -----------------------------------------------------------------------------------
                                                                                                 Section 221
                                          (18261) 18261    18270     18280     18290     18300     18310     18320     18330       18343
SARS-CoV-2 Reference Genome NC_045512.2 (18126) TGACACTAAATTCAAAACTGAAGGTTTATGTGTTGACATACCTGGCATACCTAAGGACATGACCTATAGAAGACTCATCTCTA
        Fig badnavirus 1 NC_017830.1    (7141) -----------------------------------------------------------------------------------
                                                                                                 Section 222
                                          (18344) 18344 18350     18360     18370     18380     18390     18400     18410         18426
SARS-CoV-2 Reference Genome NC_045512.2 (18209) TGATGGGTTTTAAAATGAATTATCAAGTTAATGGTTACCCTAACATGTTTATCACCCGCGAAGAAGCTATAAGACATGTACGT
        Fig badnavirus 1 NC_017830.1    (7141) -----------------------------------------------------------------------------------
                                                                                                 Section 223
                                          (18427) 18427        18440     18450     18460     18470     18480     18490           18509
SARS-CoV-2 Reference Genome NC_045512.2 (18292) GCATGGATTGGCTTCGATGTCGAGGGGTGTCATGCTACTAGAGAAGCTGTTGGTACCAATTTACCTTTACAGCTAGGTTTTTC
        Fig badnavirus 1 NC_017830.1    (7141) -----------------------------------------------------------------------------------
                                                                                                 Section 224
                                          (18510) 18510     18520     18530     18540     18550     18560     18570     18580     18592
SARS-CoV-2 Reference Genome NC_045512.2 (18375) TACAGGTGTTAACCTAGTTGCTGTACCTACAGGTTATGTTGATACACCTAATAATACAGATTTTTCCAGAGTTAGTGCTAAAC
        Fig badnavirus 1 NC_017830.1    (7141) -----------------------------------------------------------------------------------
```

```
                                                                                                               Section 225
                                             (18593) 18593 18600     18610     18620     18630     18640     18650     18660          18675
SARS-CoV-2 Reference Genome NC_045512.2 (18458) CACCGCCTGGAGATCAATTTAAACACCTCATACCACTTATGTACAAAGGACTTCCTTGGAATGTAGTGCGTATAAAGATTGTA
        Fig badnavirus 1 NC_017830.1    (7141) -----------------------------------------------------------------------------------
                                                                                                               Section 226
                                             (18676) 18676         18690     18700     18710     18720     18730     18740               18758
SARS-CoV-2 Reference Genome NC_045512.2 (18541) CAAATGTTAAGTGACACACTTAAAAATCTCTCTGACAGAGTCGTATTTGTCTTATGGGCACATGGCTTTGAGTTGACATCTAT
        Fig badnavirus 1 NC_017830.1    (7141) -----------------------------------------------------------------------------------
                                                                                                               Section 227
                                             (18759) 18759      18770     18780     18790     18800     18810     18820     18830      18841
SARS-CoV-2 Reference Genome NC_045512.2 (18624) GAAGTATTTTGTGAAAATAGGACCTGAGCGCACCTGTTGTCTATGTGATAGACGTGCCACATGCTTTTCCACTGCTTCAGACA
        Fig badnavirus 1 NC_017830.1    (7141) -----------------------------------------------------------------------------------
                                                                                                               Section 228
                                             (18842) 18842   18850     18860     18870     18880     18890     18900     18910          18924
SARS-CoV-2 Reference Genome NC_045512.2 (18707) CTTATGCCTGTTGGCATCATTCTATTGGATTTGATTACGTCTATAATCCGTTTATGATTGATGTTCAACAATGGGGTTTTACA
        Fig badnavirus 1 NC_017830.1    (7141) -----------------------------------------------------------------------------------
                                                                                                               Section 229
                                             (18925) 18925 18930     18940     18950     18960     18970     18980     18990          19007
SARS-CoV-2 Reference Genome NC_045512.2 (18790) GGTAACCTACAAAGCAACCATGATCTGTATTGTCAAGTCCATGGTAATGCACATGTAGCTAGTTGTGATGCAATCATGACTAG
        Fig badnavirus 1 NC_017830.1    (7141) -----------------------------------------------------------------------------------
                                                                                                               Section 230
                                             (19008) 19008       19020     19030     19040     19050     19060     19070     19080     19090
SARS-CoV-2 Reference Genome NC_045512.2 (18873) GTGTCTAGCTGTCCACGAGTGCTTTGTTAAGCGTGTTGACTGGACTATTGAATATCCTATAATTGGTGATGAACTGAAGATTA
        Fig badnavirus 1 NC_017830.1    (7141) -----------------------------------------------------------------------------------
                                                                                                               Section 231
                                             (19091) 19091    19100     19110     19120     19130     19140     19150     19160          19173
SARS-CoV-2 Reference Genome NC_045512.2 (18956) ATGCGGCTTGTAGAAAGGTTCAACACATGGTTGTTAAAGCTGCATTATTAGCAGACAAATTCCCAGTTCTTCACGACATTGGT
        Fig badnavirus 1 NC_017830.1    (7141) -----------------------------------------------------------------------------------
```

```
                                                                                                                 Section 232
                                          (19174) 19174 19180     19190     19200     19210     19220     19230     19240          19256
SARS-CoV-2 Reference Genome NC_045512.2 (19039) AACCCTAAAGCTATTAAGTGTGTACCTCAAGCTGATGTAGAATGGAAGTTCTATGATGCACAGCCTTGTAGTGACAAAGCTTA
           Fig badnavirus 1 NC_017830.1   (7141) -----------------------------------------------------------------------------------
                                                                                                                 Section 233
                                          (19257) 19257        19270     19280     19290     19300     19310     19320          19339
SARS-CoV-2 Reference Genome NC_045512.2 (19122) TAAAATAGAAGAATTATTCTATTCTTATGCCACACATTCTGACAAATTCACAGATGGTGTATGCCTATTTTGGAATTGCAATG
           Fig badnavirus 1 NC_017830.1   (7141) -----------------------------------------------------------------------------------
                                                                                                                 Section 234
                                          (19340) 19340     19350     19360     19370     19380     19390     19400     19410     19422
SARS-CoV-2 Reference Genome NC_045512.2 (19205) TCGATAGATATCCTGCTAATTCCATTGTTTGTAGATTTGACACTAGAGTGCTATCTAACCTTAACTTGCCTGGTTGTGATGGT
           Fig badnavirus 1 NC_017830.1   (7141) -----------------------------------------------------------------------------------
                                                                                                                 Section 235
                                          (19423) 19423  19430     19440     19450     19460     19470     19480     19490          19505
SARS-CoV-2 Reference Genome NC_045512.2 (19288) GGCAGTTTGTATGTAAATAAACATGCATTCCACACACCAGCTTTTGATAAAAGTGCTTTTGTTAATTTAAAACAATTACCATT
           Fig badnavirus 1 NC_017830.1   (7141) -----------------------------------------------------------------------------------
                                                                                                                 Section 236
                                          (19506) 19506         19520     19530     19540     19550     19560     19570          19588
SARS-CoV-2 Reference Genome NC_045512.2 (19371) TTTCTATTACTCTGACAGTCCATGTGAGTCTCATGGAAAACAAGTAGTGTCAGATATAGATTATGTACCACTAAAGTCTGCTA
           Fig badnavirus 1 NC_017830.1   (7141) -----------------------------------------------------------------------------------
                                                                                                                 Section 237
                                          (19589) 19589      19600     19610     19620     19630     19640     19650     19660   19671
SARS-CoV-2 Reference Genome NC_045512.2 (19454) CGTGTATAACACGTTGCAATTTAGGTGGTGCTGTCTGTAGACATCATGCTAATGAGTACAGATTGTATCTCGATGCTTATAAC
           Fig badnavirus 1 NC_017830.1   (7141) -----------------------------------------------------------------------------------
                                                                                                                 Section 238
                                          (19672) 19672   19680     19690     19700     19710     19720     19730     19740          19754
SARS-CoV-2 Reference Genome NC_045512.2 (19537) ATGATGATCTCAGCTGGCTTTAGCTTGTGGGTTTACAAACAATTTGATACTTATAACCTCTGGAACACTTTTACAAGACTTCA
           Fig badnavirus 1 NC_017830.1   (7141) -----------------------------------------------------------------------------------
```

```
                                                Section 239
                                  (19755) 19755 19760 19770 19780 19790 19800 19810 19820 19837
SARS-CoV-2 Reference Genome NC_045512.2 (19620) GAGTTTAGAAAATGTGGCTTTTAATGTTGTAAATAAGGGACACTTTGATGGACAACAGGGTGAAGTACCAGTTTCTATCATTA
       Fig badnavirus 1 NC_017830.1      (7141) -----------------------------------------------------------------------------------
                                                Section 240
                                  (19838) 19838 19850 19860 19870 19880 19890 19900 19910 19920
SARS-CoV-2 Reference Genome NC_045512.2 (19703) ATAACACTGTTTACACAAAAGTTGATGGTGTTGATGTAGAATTGTTTGAAAATAAAACAACATTACCTGTTAATGTAGCATTT
       Fig badnavirus 1 NC_017830.1      (7141) -----------------------------------------------------------------------------------
                                                Section 241
                                  (19921) 19921 19930 19940 19950 19960 19970 19980 19990 20003
SARS-CoV-2 Reference Genome NC_045512.2 (19786) GAGCTTTGGGCTAAGCGCAACATTAAACCAGTACCAGAGGTGAAAATACTCAATAATTTGGGTGTGGACATTGCTGCTAATAC
       Fig badnavirus 1 NC_017830.1      (7141) -----------------------------------------------------------------------------------
                                                Section 242
                                  (20004) 20004 20010 20020 20030 20040 20050 20060 20070 20086
SARS-CoV-2 Reference Genome NC_045512.2 (19869) TGTGATCTGGGACTACAAAAGAGATGCTCCAGCACATATATCTACTATTGGTGTTTGTTCTATGACTGACATAGCCAAGAAAC
       Fig badnavirus 1 NC_017830.1      (7141) -----------------------------------------------------------------------------------
                                                Section 243
                                  (20087) 20087 20100 20110 20120 20130 20140 20150 20169
SARS-CoV-2 Reference Genome NC_045512.2 (19952) CAACTGAAACGATTTGTGCACCACTCACTGTCTTTTTTGATGGTAGAGTTGATGGTCAAGTAGACTTATTTAGAAATGCCCGT
       Fig badnavirus 1 NC_017830.1      (7141) -----------------------------------------------------------------------------------
                                                Section 244
                                  (20170) 20170 20180 20190 20200 20210 20220 20230 20240 20252
SARS-CoV-2 Reference Genome NC_045512.2 (20035) AATGGTGTTCTTATTACAGAAGGTAGTGTTAAAGGTTTACAACCATCTGTAGGTCCCAAACAAGCTAGTCTTAATGGAGTCAC
       Fig badnavirus 1 NC_017830.1      (7141) -----------------------------------------------------------------------------------
                                                Section 245
                                  (20253) 20253 20260 20270 20280 20290 20300 20310 20320 20335
SARS-CoV-2 Reference Genome NC_045512.2 (20118) ATTAATTGGAGAAGCCGTAAAAACACAGTTCAATTATTATAAGAAAGTTGATGGTGTTGTCCAACAATTACCTGAAACTTACT
       Fig badnavirus 1 NC_017830.1      (7141) -----------------------------------------------------------------------------------
```

Section 246

```
                                        (20336) 20336              20350        20360        20370        20380        20390        20400                20418
SARS-CoV-2 Reference Genome NC_045512.2 (20201) TTACTCAGAGTAGAAATTTACAAGAATTTAAACCCAGGAGTCAAATGGAAATTGATTTCTTAGAATTAGCTATGGATGAATTC
        Fig badnavirus 1 NC_017830.1    (7141) -----------------------------------------------------------------------------------
```

Section 247

```
                                        (20419) 20419           20430        20440        20450        20460        20470        20480        20490     20501
SARS-CoV-2 Reference Genome NC_045512.2 (20284) ATTGAACGGTATAAATTAGAAGGCTATGCCTTCGAACATATCGTTTATGGAGATTTTAGTCATAGTCAGTTAGGTGGTTTACA
        Fig badnavirus 1 NC_017830.1    (7141) -----------------------------------------------------------------------------------
```

Section 248

```
                                        (20502) 20502   20510        20520        20530        20540        20550        20560        20570           20584
SARS-CoV-2 Reference Genome NC_045512.2 (20367) TCTACTGATTGGACTAGCTAAACGTTTTAAGGAATCACCTTTTGAATTAGAAGATTTTATTCCTATGGACAGTACAGTTAAAA
        Fig badnavirus 1 NC_017830.1    (7141) -----------------------------------------------------------------------------------
```

Section 249

```
                                        (20585) 20585 20590        20600        20610        20620        20630        20640        20650             20667
SARS-CoV-2 Reference Genome NC_045512.2 (20450) ACTATTTCATAACAGATGCGCAAACAGGTTCATCTAAGTGTGTGTGTTCTGTTATTGATTTATTACTTGATGATTTTGTTGAA
        Fig badnavirus 1 NC_017830.1    (7141) -----------------------------------------------------------------------------------
```

Section 250

```
                                        (20668) 20668          20680        20690        20700        20710        20720        20730        20740   20750
SARS-CoV-2 Reference Genome NC_045512.2 (20533) ATAATAAAATCCCAAGATTTATCTGTAGTTTCTAAGGTTGTCAAAGTGACTATTGACTATACAGAAATTTCATTTATGCTTTG
        Fig badnavirus 1 NC_017830.1    (7141) -----------------------------------------------------------------------------------
```

Section 251

```
                                        (20751) 20751    20760        20770        20780        20790        20800        20810        20820          20833
SARS-CoV-2 Reference Genome NC_045512.2 (20616) GTGTAAAGATGGCCATGTAGAAACATTTTACCCAAAATTACAATCTAGTCAAGCGTGGCAACCGGGTGTTGCTATGCCTAATC
        Fig badnavirus 1 NC_017830.1    (7141) -----------------------------------------------------------------------------------
```

Section 252

```
                                        (20834) 20834 20840        20850        20860        20870        20880        20890        20900             20916
SARS-CoV-2 Reference Genome NC_045512.2 (20699) TTTACAAAATGCAAAGAATGCTATTAGAAAAGTGTGACCTTCAAAATTATGGTGATAGTGCAACATTACCTAAAGGCATAATG
        Fig badnavirus 1 NC_017830.1    (7141) -----------------------------------------------------------------------------------
```

```
                                                               Section 253
                                            (20917) 20917        20930      20940      20950      20960      20970      20980           20999
SARS-CoV-2 Reference Genome NC_045512.2 (20782) ATGAATGTCGCAAAATATACTCAACTGTGTCAATATTTAAACACATTAACATTAGCTGTACCCTATAATATGAGAGTTATACA
          Fig badnavirus 1 NC_017830.1   (7141) -----------------------------------------------------------------------------------
                                                               Section 254
                                            (21000) 21000      21010      21020      21030      21040      21050      21060      21070    21082
SARS-CoV-2 Reference Genome NC_045512.2 (20865) TTTTGGTGCTGGTTCTGATAAAGGAGTTGCACCAGGTACAGCTGTTTTAAGACAGTGGTTGCCTACGGGTACGCTGCTTGTCG
          Fig badnavirus 1 NC_017830.1   (7141) -----------------------------------------------------------------------------------
                                                               Section 255
                                            (21083) 21083  21090      21100      21110      21120      21130      21140      21150        21165
SARS-CoV-2 Reference Genome NC_045512.2 (20948) ATTCAGATCTTAATGACTTTGTCTCTGATGCAGATTCAACTTTGATTGGTGATTGTGCAACTGTACATACAGCTAATAAATGG
          Fig badnavirus 1 NC_017830.1   (7141) -----------------------------------------------------------------------------------
                                                               Section 256
                                            (21166) 21166          21180      21190      21200      21210      21220      21230           21248
SARS-CoV-2 Reference Genome NC_045512.2 (21031) GATCTCATTATTAGTGATATGTACGACCCTAAGACTAAAAATGTTACAAAAGAAAATGACTCTAAAGAGGGTTTTTTCACTTA
          Fig badnavirus 1 NC_017830.1   (7141) -----------------------------------------------------------------------------------
                                                               Section 257
                                            (21249) 21249       21260      21270      21280      21290      21300      21310      21320   21331
SARS-CoV-2 Reference Genome NC_045512.2 (21114) CATTTGTGGGTTTATACAACAAAAGCTAGCTCTTGGAGGTTCCGTGGCTATAAAGATAACAGAACATTCTTGGAATGCTGATC
          Fig badnavirus 1 NC_017830.1   (7141) -----------------------------------------------------------------------------------
                                                               Section 258
                                            (21332) 21332   21340      21350      21360      21370      21380      21390      21400        21414
SARS-CoV-2 Reference Genome NC_045512.2 (21197) TTTATAAGCTCATGGGACACTTCGCATGGTGGACAGCCTTTGTTACTAATGTGAATGCGTCATCATCTGAAGCATTTTTAATT
          Fig badnavirus 1 NC_017830.1   (7141) -----------------------------------------------------------------------------------
                                                               Section 259
                                            (21415) 21415 21420      21430      21440      21450      21460      21470      21480         21497
SARS-CoV-2 Reference Genome NC_045512.2 (21280) GGATGTAATTATCTTGGCAAACCACGCGAACAAATAGATGGTTATGTCATGCATGCAAATTACATATTTTGGAGGAATACAAA
          Fig badnavirus 1 NC_017830.1   (7141) -----------------------------------------------------------------------------------
```

```
                                                       Section 260
                                            (21498) 21498      21510     21520     21530     21540     21550     21560     21570   21580
SARS-CoV-2 Reference Genome NC_045512.2 (21363) TCCAATTCAGTTGTCTTCCTATTCTTTATTTGACATGAGTAAATTTCCCCTTAAATTAAGGGGTACTGCTGTTATGTCTTTAA
         Fig badnavirus 1 NC_017830.1    (7141) -----------------------------------------------------------------------------------
                                                       Section 261
                                            (21581) 21581    21590     21600     21610     21620     21630     21640     21650       21663
SARS-CoV-2 Reference Genome NC_045512.2 (21446) AAGAAGGTCAAATCAATGATATGATTTTATCTCTTCTTAGTAAAGGTAGACTTATAATTAGAGAAAACAACAGAGTTGTTATT
         Fig badnavirus 1 NC_017830.1    (7141) -----------------------------------------------------------------------------------
                                                       Section 262
                                            (21664) 21664 21670     21680     21690     21700     21710     21720     21730           21746
SARS-CoV-2 Reference Genome NC_045512.2 (21529) TCTAGTGATGTTCTTGTTAACAACTAAACGAACAATGTTTGTTTTTCTTGTTTTATTGCCACTAGTCTCTAGTCAGTGTGTTA
         Fig badnavirus 1 NC_017830.1    (7141) -----------------------------------------------------------------------------------
                                                       Section 263
                                            (21747) 21747        21760     21770     21780     21790     21800     21810               21829
SARS-CoV-2 Reference Genome NC_045512.2 (21612) ATCTTACAACCAGAACTCAATTACCCCCTGCATACACTAATTCTTTCACACGTGGTGTTTATTACCCTGACAAAGTTTTCAGA
         Fig badnavirus 1 NC_017830.1    (7141) -----------------------------------------------------------------------------------
                                                       Section 264
                                            (21830) 21830     21840     21850     21860     21870     21880     21890     21900     21912
SARS-CoV-2 Reference Genome NC_045512.2 (21695) TCCTCAGTTTTACATTCAACTCAGGACTTGTTCTTACCTTTCTTTTCCAATGTTACTTGGTTCCATGCTATACATGTCTCTGG
         Fig badnavirus 1 NC_017830.1    (7141) -----------------------------------------------------------------------------------
                                                       Section 265
                                            (21913) 21913  21920     21930     21940     21950     21960     21970     21980          21995
SARS-CoV-2 Reference Genome NC_045512.2 (21778) GACCAATGGTACTAAGAGGTTTGATAACCCTGTCCTACCATTTAATGATGGTGTTTATTTTGCTTCCACTGAGAAGTCTAACA
         Fig badnavirus 1 NC_017830.1    (7141) -----------------------------------------------------------------------------------
                                                       Section 266
                                            (21996) 21996         22010     22020     22030     22040     22050     22060             22078
SARS-CoV-2 Reference Genome NC_045512.2 (21861) TAATAAGAGGCTGGATTTTTGGTACTACTTTAGATTCGAAGACCCAGTCCCTACTTATTGTTAATAACGCTACTAATGTTGTT
         Fig badnavirus 1 NC_017830.1    (7141) -----------------------------------------------------------------------------------
```

```
                                                                                Section 267
                                           (22079) 22079      22090          22100          22110          22120          22130          22140          22150    22161
SARS-CoV-2 Reference Genome NC_045512.2 (21944) ATTAAAGTCTGTGAATTTCAATTTTGTAATGATCCATTTTTGGGTGTTTATTACCACAAAAACAACAAAAGTTGGATGGAAAG
       Fig badnavirus 1 NC_017830.1   (7141) -----------------------------------------------------------------------------------
                                                                                Section 268
                                           (22162) 22162   22170          22180          22190          22200          22210          22220          22230          22244
SARS-CoV-2 Reference Genome NC_045512.2 (22027) TGAGTTCAGAGTTTATTCTAGTGCGAATAATTGCACTTTTGAATATGTCTCTCAGCCTTTTCTTATGGACCTTGAAGGAAAAC
       Fig badnavirus 1 NC_017830.1   (7141) -----------------------------------------------------------------------------------
                                                                                Section 269
                                           (22245) 22245 22250          22260          22270          22280          22290          22300          22310          22327
SARS-CoV-2 Reference Genome NC_045512.2 (22110) AGGGTAATTTCAAAAATCTTAGGGAATTTGTGTTTAAGAATATTGATGGTTATTTTAAAATATATTCTAAGCACACGCCTATT
       Fig badnavirus 1 NC_017830.1   (7141) -----------------------------------------------------------------------------------
                                                                                Section 270
                                           (22328) 22328        22340          22350          22360          22370          22380          22390          22400   22410
SARS-CoV-2 Reference Genome NC_045512.2 (22193) AATTTAGTGCGTGATCTCCCTCAGGGTTTTTCGGCTTTAGAACCATTGGTAGATTTGCCAATAGGTATTAACATCACTAGGTT
       Fig badnavirus 1 NC_017830.1   (7141) -----------------------------------------------------------------------------------
                                                                                Section 271
                                           (22411) 22411    22420          22430          22440          22450          22460          22470          22480          22493
SARS-CoV-2 Reference Genome NC_045512.2 (22276) TCAAACTTTACTTGCTTTACATAGAAGTTATTTGACTCCTGGTGATTCTTCTTCAGGTTGGACAGCTGGTGCTGCAGCTTATT
       Fig badnavirus 1 NC_017830.1   (7141) -----------------------------------------------------------------------------------
                                                                                Section 272
                                           (22494) 22494 22500          22510          22520          22530          22540          22550          22560          22576
SARS-CoV-2 Reference Genome NC_045512.2 (22359) ATGTGGGTTATCTTCAACCTAGGACTTTTCTATTAAAATATAATGAAAATGGAACCATTACAGATGCTGTAGACTGTGCACTT
       Fig badnavirus 1 NC_017830.1   (7141) -----------------------------------------------------------------------------------
                                                                                Section 273
                                           (22577) 22577         22590          22600          22610          22620          22630          22640              22659
SARS-CoV-2 Reference Genome NC_045512.2 (22442) GACCCTCTCTCAGAAACAAAGTGTACGTTGAAATCCTTCACTGTAGAAAAAGGAATCTATCAAACTTCTAACTTTAGAGTCCA
       Fig badnavirus 1 NC_017830.1   (7141) -----------------------------------------------------------------------------------
```

```
                                                                                                                    Section 274
                                         (22660) 22660     22670     22680     22690     22700     22710     22720     22730    22742
SARS-CoV-2 Reference Genome NC_045512.2 (22525) ACCAACAGAATCTATTGTTAGATTTCCTAATATTACAAACTTGTGCCCTTTTGGTGAAGTTTTTAACGCCACCAGATTTGCAT
      Fig badnavirus 1 NC_017830.1  (7141) -----------------------------------------------------------------------------------
                                                                                                                    Section 275
                                         (22743) 22743  22750     22760     22770     22780     22790     22800     22810          22825
SARS-CoV-2 Reference Genome NC_045512.2 (22608) CTGTTTATGCTTGGAACAGGAAGAGAATCAGCAACTGTGTTGCTGATTATTCTGTCCTATATAATTCCGCATCATTTTCCACT
      Fig badnavirus 1 NC_017830.1  (7141) -----------------------------------------------------------------------------------
                                                                                                                    Section 276
                                         (22826) 22826         22840     22850     22860     22870     22880     22890             22908
SARS-CoV-2 Reference Genome NC_045512.2 (22691) TTTAAGTGTTATGGAGTGTCTCCTACTAAATTAAATGATCTCTGCTTTACTAATGTCTATGCAGATTCATTTGTAATTAGAGG
      Fig badnavirus 1 NC_017830.1  (7141) -----------------------------------------------------------------------------------
                                                                                                                    Section 277
                                         (22909) 22909      22920     22930     22940     22950     22960     22970     22980    22991
SARS-CoV-2 Reference Genome NC_045512.2 (22774) TGATGAAGTCAGACAAATCGCTCCAGGGCAAACTGGAAAGATTGCTGATTATAATTATAAATTACCAGATGATTTTACAGGCT
      Fig badnavirus 1 NC_017830.1  (7141) -----------------------------------------------------------------------------------
                                                                                                                    Section 278
                                         (22992) 22992   23000     23010     23020     23030     23040     23050     23060         23074
SARS-CoV-2 Reference Genome NC_045512.2 (22857) GCGTTATAGCTTGGAATTCTAACAATCTTGATTCTAAGGTTGGTGGTAATTATAATTACCTGTATAGATTGTTTAGGAAGTCT
      Fig badnavirus 1 NC_017830.1  (7141) -----------------------------------------------------------------------------------
                                                                                                                    Section 279
                                         (23075) 23075 23080     23090     23100     23110     23120     23130     23140            23157
SARS-CoV-2 Reference Genome NC_045512.2 (22940) AATCTCAAACCTTTTGAGAGAGATATTTCAACTGAAATCTATCAGGCCGGTAGCACACCTTGTAATGGTGTTGAAGGTTTTAA
      Fig badnavirus 1 NC_017830.1  (7141) -----------------------------------------------------------------------------------
                                                                                                                    Section 280
                                         (23158) 23158       23170     23180     23190     23200     23210     23220     23230    23240
SARS-CoV-2 Reference Genome NC_045512.2 (23023) TTGTTACTTTCCTTTACAATCATATGGTTTCCAACCCACTAATGGTGTTGGTTACCAACCATACAGAGTAGTAGTACTTTCTT
      Fig badnavirus 1 NC_017830.1  (7141) -----------------------------------------------------------------------------------
```

```
                                                                                                                     Section 281
                                          (23241) 23241    23250     23260     23270     23280     23290     23300     23310     23323
SARS-CoV-2 Reference Genome NC_045512.2 (23106) TTGAACTTCTACATGCACCAGCAACTGTTTGTGGACCTAAAAAGTCTACTAATTTGGTTAAAAACAAATGTGTCAATTTCAAC
            Fig badnavirus 1 NC_017830.1   (7141) -----------------------------------------------------------------------------------
                                                                                                                     Section 282
                                          (23324) 23324 23330     23340     23350     23360     23370     23380     23390     23406
SARS-CoV-2 Reference Genome NC_045512.2 (23189) TTCAATGGTTTAACAGGCACAGGTGTTCTTACTGAGTCTAACAAAAAGTTTCTGCCTTTCCAACAATTTGGCAGAGACATTGC
            Fig badnavirus 1 NC_017830.1   (7141) -----------------------------------------------------------------------------------
                                                                                                                     Section 283
                                          (23407) 23407         23420     23430     23440     23450     23460     23470          23489
SARS-CoV-2 Reference Genome NC_045512.2 (23272) TGACACTACTGATGCTGTCCGTGATCCACAGACACTTGAGATTCTTGACATTACACCATGTTCTTTTGGTGGTGTCAGTGTTA
            Fig badnavirus 1 NC_017830.1   (7141) -----------------------------------------------------------------------------------
                                                                                                                     Section 284
                                          (23490) 23490     23500     23510     23520     23530     23540     23550     23560     23572
SARS-CoV-2 Reference Genome NC_045512.2 (23355) TAACACCAGGAACAAATACTTCTAACCAGGTTGCTGTTCTTTATCAGGATGTTAACTGCACAGAAGTCCCTGTTGCTATTCAT
            Fig badnavirus 1 NC_017830.1   (7141) -----------------------------------------------------------------------------------
                                                                                                                     Section 285
                                          (23573) 23573  23580     23590     23600     23610     23620     23630     23640     23655
SARS-CoV-2 Reference Genome NC_045512.2 (23438) GCAGATCAACTTACTCCTACTTGGCGTGTTTATTCTACAGGTTCTAATGTTTTTCAAACACGTGCAGGCTGTTTAATAGGGGC
            Fig badnavirus 1 NC_017830.1   (7141) -----------------------------------------------------------------------------------
                                                                                                                     Section 286
                                          (23656) 23656         23670     23680     23690     23700     23710     23720          23738
SARS-CoV-2 Reference Genome NC_045512.2 (23521) TGAACATGTCAACAACTCATATGAGTGTGACATACCCATTGGTGCAGGTATATGCGCTAGTTATCAGACTCAGACTAATTCTC
            Fig badnavirus 1 NC_017830.1   (7141) -----------------------------------------------------------------------------------
                                                                                                                     Section 287
                                          (23739) 23739      23750     23760     23770     23780     23790     23800     23810     23821
SARS-CoV-2 Reference Genome NC_045512.2 (23604) CTCGGCGGGCACGTAGTGTAGCTAGTCAATCCATCATTGCCTACACTATGTCACTTGGTGCAGAAAATTCAGTTGCTTACTCT
            Fig badnavirus 1 NC_017830.1   (7141) -----------------------------------------------------------------------------------
```

```
                                                                                                    Section 288
                                      (23822) 23822 23830     23840     23850     23860     23870     23880     23890       23904
SARS-CoV-2 Reference Genome NC_045512.2 (23687) AATAACTCTATTGCCATACCCACAAATTTTACTATTAGTGTTACCACAGAAATTCTACCAGTGTCTATGACCAAGACATCAGT
        Fig badnavirus 1 NC_017830.1   (7141) -----------------------------------------------------------------------------------
                                                                                                    Section 289
                                      (23905) 23905 23910     23920     23930     23940     23950     23960     23970       23987
SARS-CoV-2 Reference Genome NC_045512.2 (23770) AGATTGTACAATGTACATTTGTGGTGATTCAACTGAATGCAGCAATCTTTTGTTGCAATATGGCAGTTTTTGTACACAATTAA
        Fig badnavirus 1 NC_017830.1   (7141) -----------------------------------------------------------------------------------
                                                                                                    Section 290
                                      (23988) 23988       24000     24010     24020     24030     24040     24050     24060 24070
SARS-CoV-2 Reference Genome NC_045512.2 (23853) ACCGTGCTTTAACTGGAATAGCTGTTGAACAAGACAAAAACACCCAAGAAGTTTTTGCACAAGTCAAACAAATTTACAAAACA
        Fig badnavirus 1 NC_017830.1   (7141) -----------------------------------------------------------------------------------
                                                                                                    Section 291
                                      (24071) 24071    24080     24090     24100     24110     24120     24130     24140      24153
SARS-CoV-2 Reference Genome NC_045512.2 (23936) CCACCAATTAAAGATTTTGGTGGTTTTAATTTTTCACAAATATTACCAGATCCATCAAAACCAAGCAAGAGGTCATTTATTGA
        Fig badnavirus 1 NC_017830.1   (7141) -----------------------------------------------------------------------------------
                                                                                                    Section 292
                                      (24154) 24154 24160     24170     24180     24190     24200     24210     24220       24236
SARS-CoV-2 Reference Genome NC_045512.2 (24019) AGATCTACTTTTCAACAAAGTGACACTTGCAGATGCTGGCTTCATCAAACAATATGGTGATTGCCTTGGTGATATTGCTGCTA
        Fig badnavirus 1 NC_017830.1   (7141) -----------------------------------------------------------------------------------
                                                                                                    Section 293
                                      (24237) 24237        24250     24260     24270     24280     24290     24300          24319
SARS-CoV-2 Reference Genome NC_045512.2 (24102) GAGACCTCATTTGTGCACAAAAGTTTAACGGCCTTACTGTTTTGCCACCTTTGCTCACAGATGAAATGATTGCTCAATACACT
        Fig badnavirus 1 NC_017830.1   (7141) -----------------------------------------------------------------------------------
                                                                                                    Section 294
                                      (24320) 24320     24330     24340     24350     24360     24370     24380     24390     24402
SARS-CoV-2 Reference Genome NC_045512.2 (24185) TCTGCACTGTTAGCGGGTACAATCACTTCTGGTTGGACCTTTGGTGCAGGTGCTGCATTACAAATACCATTTGCTATGCAAAT
        Fig badnavirus 1 NC_017830.1   (7141) -----------------------------------------------------------------------------------
```

```
                                                                                        Section 295
                                         (24403) 24403  24410     24420     24430     24440     24450     24460     24470     24485
SARS-CoV-2 Reference Genome NC_045512.2 (24268) GGCTTATAGGTTTAATGGTATTGGAGTTACACAGAATGTTCTCTATGAGAACCAAAAATTGATTGCCAACCAATTTAATAGTG
           Fig badnavirus 1 NC_017830.1  (7141) -----------------------------------------------------------------------------------
                                                                                        Section 296
                                         (24486) 24486         24500     24510     24520     24530     24540     24550          24568
SARS-CoV-2 Reference Genome NC_045512.2 (24351) CTATTGGCAAAATTCAAGACTCACTTTCTTCCACAGCAAGTGCACTTGGAAAACTTCAAGATGTGGTCAACCAAAATGCACAA
           Fig badnavirus 1 NC_017830.1  (7141) -----------------------------------------------------------------------------------
                                                                                        Section 297
                                         (24569) 24569      24580     24590     24600     24610     24620     24630     24640  24651
SARS-CoV-2 Reference Genome NC_045512.2 (24434) GCTTTAAACACGCTTGTTAAACAACTTAGCTCCAATTTTGGTGCAATTTCAAGTGTTTTAAATGATATCCTTTCACGTCTTGA
           Fig badnavirus 1 NC_017830.1  (7141) -----------------------------------------------------------------------------------
                                                                                        Section 298
                                         (24652) 24652   24660     24670     24680     24690     24700     24710     24720     24734
SARS-CoV-2 Reference Genome NC_045512.2 (24517) CAAAGTTGAGGCTGAAGTGCAAATTGATAGGTTGATCACAGGCAGACTTCAAAGTTTGCAGACATATGTGACTCAACAATTAA
           Fig badnavirus 1 NC_017830.1  (7141) -----------------------------------------------------------------------------------
                                                                                        Section 299
                                         (24735) 24735 24740     24750     24760     24770     24780     24790     24800       24817
SARS-CoV-2 Reference Genome NC_045512.2 (24600) TTAGAGCTGCAGAAATCAGAGCTTCTGCTAATCTTGCTGCTACTAAAATGTCAGAGTGTGTACTTGGACAATCAAAAAGAGTT
           Fig badnavirus 1 NC_017830.1  (7141) -----------------------------------------------------------------------------------
                                                                                        Section 300
                                         (24818) 24818       24830     24840     24850     24860     24870     24880     24890  24900
SARS-CoV-2 Reference Genome NC_045512.2 (24683) GATTTTTGTGGAAAGGGCTATCATCTTATGTCCTTCCCTCAGTCAGCACCTCATGGTGTAGTCTTCTTGCATGTGACTTATGT
           Fig badnavirus 1 NC_017830.1  (7141) -----------------------------------------------------------------------------------
                                                                                        Section 301
                                         (24901) 24901    24910     24920     24930     24940     24950     24960     24970     24983
SARS-CoV-2 Reference Genome NC_045512.2 (24766) CCCTGCACAAGAAAAGAACTTCACAACTGCTCCTGCCATTTGTCATGATGGAAAAGCACACTTTCCTCGTGAAGGTGTCTTTG
           Fig badnavirus 1 NC_017830.1  (7141) -----------------------------------------------------------------------------------
```

```
                                                                                                                Section 302
                                               (24984) 24984 24990     25000     25010     25020     25030     25040     25050           25066
SARS-CoV-2 Reference Genome NC_045512.2 (24849) TTTCAAATGGCACACACTGGTTTGTAACACAAAGGAATTTTTATGAACCACAAATCATTACTACAGACAACACATTTGTGTCT
          Fig badnavirus 1 NC_017830.1   (7141) -----------------------------------------------------------------------------------
                                                                                                                Section 303
                                               (25067) 25067        25080     25090     25100     25110     25120     25130               25149
SARS-CoV-2 Reference Genome NC_045512.2 (24932) GGTAACTGTGATGTTGTAATAGGAATTGTCAACAACACAGTTTATGATCCTTTGCAACCTGAATTAGACTCATTCAAGGAGGA
          Fig badnavirus 1 NC_017830.1   (7141) -----------------------------------------------------------------------------------
                                                                                                                Section 304
                                               (25150) 25150     25160     25170     25180     25190     25200     25210     25220       25232
SARS-CoV-2 Reference Genome NC_045512.2 (25015) GTTAGATAAATATTTTAAGAATCATACATCACCAGATGTTGATTTAGGTGACATCTCTGGCATTAATGCTTCAGTTGTAAACA
          Fig badnavirus 1 NC_017830.1   (7141) -----------------------------------------------------------------------------------
                                                                                                                Section 305
                                               (25233) 25233  25240     25250     25260     25270     25280     25290     25300          25315
SARS-CoV-2 Reference Genome NC_045512.2 (25098) TTCAAAAAGAAATTGACCGCCTCAATGAGGTTGCCAAGAATTTAAATGAATCTCTCATCGATCTCCAAGAACTTGGAAAGTAT
          Fig badnavirus 1 NC_017830.1   (7141) -----------------------------------------------------------------------------------
                                                                                                                Section 306
                                               (25316) 25316         25330     25340     25350     25360     25370     25380             25398
SARS-CoV-2 Reference Genome NC_045512.2 (25181) GAGCAGTATATAAAATGGCCATGGTACATTTGGCTAGGTTTTATAGCTGGCTTGATTGCCATAGTAATGGTGACAATTATGCT
          Fig badnavirus 1 NC_017830.1   (7141) -----------------------------------------------------------------------------------
                                                                                                                Section 307
                                               (25399) 25399      25410     25420     25430     25440     25450     25460     25470     25481
SARS-CoV-2 Reference Genome NC_045512.2 (25264) TTGCTGTATGACCAGTTGCTGTAGTTGTCTCAAGGGCTGTTGTTCTTGTGGATCCTGCTGCAAATTTGATGAAGACGACTCTG
          Fig badnavirus 1 NC_017830.1   (7141) -----------------------------------------------------------------------------------
                                                                                                                Section 308
                                               (25482) 25482   25490     25500     25510     25520     25530     25540     25550         25564
SARS-CoV-2 Reference Genome NC_045512.2 (25347) AGCCAGTGCTCAAAGGAGTCAAATTACATTACACATAAACGAACTTATGGATTTGTTTATGAGAATCTTCACAATTGGAACTG
          Fig badnavirus 1 NC_017830.1   (7141) -----------------------------------------------------------------------------------
```

```
                                                                                          Section 309
                                         (25565) 25565 25570     25580     25590     25600     25610     25620     25630          25647
SARS-CoV-2 Reference Genome NC_045512.2 (25430) TAACTTTGAAGCAAGGTGAAATCAAGGATGCTACTCCTTCAGATTTTGTTCGCGCTACTGCAACGATACCGATACAAGCCTCA
           Fig badnavirus 1 NC_017830.1  (7141) -----------------------------------------------------------------------------------
                                                                                          Section 310
                                         (25648) 25648       25660     25670     25680     25690     25700     25710     25720  25730
SARS-CoV-2 Reference Genome NC_045512.2 (25513) CTCCCTTTCGGATGGCTTATTGTTGGCGTTGCACTTCTTGCTGTTTTTCAGAGCGCTTCCAAAATCATAACCCTCAAAAAGAG
           Fig badnavirus 1 NC_017830.1  (7141) -----------------------------------------------------------------------------------
                                                                                          Section 311
                                         (25731) 25731    25740     25750     25760     25770     25780     25790     25800     25813
SARS-CoV-2 Reference Genome NC_045512.2 (25596) ATGGCAACTAGCACTCTCCAAGGGTGTTCACTTTGTTTGCAACTTGCTGTTGTTGTTTGTAACAGTTTACTCACACCTTTTGC
           Fig badnavirus 1 NC_017830.1  (7141) -----------------------------------------------------------------------------------
                                                                                          Section 312
                                         (25814) 25814 25820     25830     25840     25850     25860     25870     25880          25896
SARS-CoV-2 Reference Genome NC_045512.2 (25679) TCGTTGCTGCTGGCCTTGAAGCCCCTTTTCTCTATCTTTATGCTTTAGTCTACTTCTTGCAGAGTATAAACTTTGTAAGAATA
           Fig badnavirus 1 NC_017830.1  (7141) -----------------------------------------------------------------------------------
                                                                                          Section 313
                                         (25897) 25897        25910     25920     25930     25940     25950     25960           25979
SARS-CoV-2 Reference Genome NC_045512.2 (25762) ATAATGAGGCTTTGGCTTTGCTGGAAATGCCGTTCCAAAAACCCATTACTTTATGATGCCAACTATTTTCTTTGCTGGCATAC
           Fig badnavirus 1 NC_017830.1  (7141) -----------------------------------------------------------------------------------
                                                                                          Section 314
                                         (25980) 25980     25990     26000     26010     26020     26030     26040     26050    26062
SARS-CoV-2 Reference Genome NC_045512.2 (25845) TAATTGTTACGACTATTGTATACCTTACAATAGTGTAACTTCTTCAATTGTCATTACTTCAGGTGATGGCACAACAAGTCCTA
           Fig badnavirus 1 NC_017830.1  (7141) -----------------------------------------------------------------------------------
                                                                                          Section 315
                                         (26063) 26063  26070     26080     26090     26100     26110     26120     26130          26145
SARS-CoV-2 Reference Genome NC_045512.2 (25928) TTTCTGAACATGACTACCAGATTGGTGGTTATACTGAAAAATGGGAATCTGGAGTAAAAGACTGTGTTGTATTACACAGTTAC
           Fig badnavirus 1 NC_017830.1  (7141) -----------------------------------------------------------------------------------
```

```
                                                                                                                        Section 316
                                          (26146) 26146          26160     26170     26180     26190     26200     26210            26228
SARS-CoV-2 Reference Genome NC_045512.2 (26011) TTCACTTCAGACTATTACCAGCTGTACTCAACTCAATTGAGTACAGACACTGGTGTTGAACATGTTACCTTCTTCATCTACAA
          Fig badnavirus 1 NC_017830.1   (7141) -----------------------------------------------------------------------------------
                                                                                                                        Section 317
                                          (26229) 26229      26240     26250     26260     26270     26280     26290     26300   26311
SARS-CoV-2 Reference Genome NC_045512.2 (26094) TAAAATTGTTGATGAGCCTGAAGAACATGTCCAAATTCACACAATCGACGGTTCATCCGGAGTTGTTAATCCAGTAATGGAAC
          Fig badnavirus 1 NC_017830.1   (7141) -----------------------------------------------------------------------------------
                                                                                                                        Section 318
                                          (26312) 26312   26320     26330     26340     26350     26360     26370     26380        26394
SARS-CoV-2 Reference Genome NC_045512.2 (26177) CAATTTATGATGAACCGACGACGACTACTAGCGTGCCTTTGTAAGCACAAGCTGATGAGTACGAACTTATGTACTCATTCGTT
          Fig badnavirus 1 NC_017830.1   (7141) -----------------------------------------------------------------------------------
                                                                                                                        Section 319
                                          (26395) 26395 26400     26410     26420     26430     26440     26450     26460          26477
SARS-CoV-2 Reference Genome NC_045512.2 (26260) TCGGAAGAGACAGGTACGTTAATAGTTAATAGCGTACTTCTTTTTCTTGCTTTCGTGGTATTCTTGCTAGTTACACTAGCCAT
          Fig badnavirus 1 NC_017830.1   (7141) -----------------------------------------------------------------------------------
                                                                                                                        Section 320
                                          (26478) 26478       26490     26500     26510     26520     26530     26540     26550   26560
SARS-CoV-2 Reference Genome NC_045512.2 (26343) CCTTACTGCGCTTCGATTGTGTGCGTACTGCTGCAATATTGTTAACGTGAGTCTTGTAAAACCTTCTTTTTACGTTTACTCTC
          Fig badnavirus 1 NC_017830.1   (7141) -----------------------------------------------------------------------------------
                                                                                                                        Section 321
                                          (26561) 26561    26570     26580     26590     26600     26610     26620     26630       26643
SARS-CoV-2 Reference Genome NC_045512.2 (26426) GTGTTAAAAATCTGAATTCTTCTAGAGTTCCTGATCTTCTGGTCTAAACGAACTAAATATTATATTAGTTTTTCTGTTTGGAA
          Fig badnavirus 1 NC_017830.1   (7141) -----------------------------------------------------------------------------------
                                                                                                                        Section 322
                                          (26644) 26644 26650     26660     26670     26680     26690     26700     26710          26726
SARS-CoV-2 Reference Genome NC_045512.2 (26509) CTTTAATTTTAGCCATGGCAGATTCCAACGGTACTATTACCGTTGAAGAGCTTAAAAAGCTCCTTGAACAATGGAACCTAGTA
          Fig badnavirus 1 NC_017830.1   (7141) -----------------------------------------------------------------------------------
```

```
                                                                                                                          Section 323
                                           (26727) 26727        26740     26750     26760     26770     26780     26790              26809
SARS-CoV-2 Reference Genome NC_045512.2 (26592) ATAGGTTTCCTATTCCTTACATGGATTTGTCTTCTACAATTTGCCTATGCCAACAGGAATAGGTTTTTGTATATAATTAAGTT
           Fig badnavirus 1 NC_017830.1  (7141) -----------------------------------------------------------------------------------
                                                                                                                          Section 324
                                           (26810) 26810     26820     26830     26840     26850     26860     26870     26880       26892
SARS-CoV-2 Reference Genome NC_045512.2 (26675) AATTTTCCTCTGGCTGTTATGGCCAGTAACTTTAGCTTGTTTTGTGCTTGCTGCTGTTTACAGAATAAATTGGATCACCGGTG
           Fig badnavirus 1 NC_017830.1  (7141) -----------------------------------------------------------------------------------
                                                                                                                          Section 325
                                           (26893) 26893  26900     26910     26920     26930     26940     26950     26960          26975
SARS-CoV-2 Reference Genome NC_045512.2 (26758) GAATTGCTATCGCAATGGCTTGTCTTGTAGGCTTGATGTGGCTCAGCTACTTCATTGCTTCTTTCAGACTGTTTGCGCGTACG
           Fig badnavirus 1 NC_017830.1  (7141) -----------------------------------------------------------------------------------
                                                                                                                          Section 326
                                           (26976) 26976         26990     27000     27010     27020     27030     27040               27058
SARS-CoV-2 Reference Genome NC_045512.2 (26841) CGTTCCATGTGGTCATTCAATCCAGAAACTAACATTCTTCTCAACGTGCCACTCCATGGCACTATTCTGACCAGACCGCTTCT
           Fig badnavirus 1 NC_017830.1  (7141) -----------------------------------------------------------------------------------
                                                                                                                          Section 327
                                           (27059) 27059      27070     27080     27090     27100     27110     27120     27130    27141
SARS-CoV-2 Reference Genome NC_045512.2 (26924) AGAAAGTGAACTCGTAATCGGAGCTGTGATCCTTCGTGGACATCTTCGTATTGCTGGACACCATCTAGGACGCTGTGACATCA
           Fig badnavirus 1 NC_017830.1  (7141) -----------------------------------------------------------------------------------
                                                                                                                          Section 328
                                           (27142) 27142   27150     27160     27170     27180     27190     27200     27210         27224
SARS-CoV-2 Reference Genome NC_045512.2 (27007) AGGACCTGCCTAAAGAAATCACTGTTGCTACATCACGAACGCTTTCTTATTACAAATTGGGAGCTTCGCAGCGTGTAGCAGGT
           Fig badnavirus 1 NC_017830.1  (7141) -----------------------------------------------------------------------------------
                                                                                                                          Section 329
                                           (27225) 27225 27230     27240     27250     27260     27270     27280     27290             27307
SARS-CoV-2 Reference Genome NC_045512.2 (27090) GACTCAGGTTTTGCTGCATACAGTCGCTACAGGATTGGCAACTATAAATTAAACACAGACCATTCCAGTAGCAGTGACAATAT
           Fig badnavirus 1 NC_017830.1  (7141) -----------------------------------------------------------------------------------
```

Section 330

```
                                        (27308) 27308       27320     27330     27340     27350     27360     27370     27380     27390
SARS-CoV-2 Reference Genome NC_045512.2 (27173) TGCTTTGCTTGTACAGTAAGTGACAACAGATGTTTCATCTCGTTGACTTTCAGGTTACTATAGCAGAGATATTACTAATTATT
         Fig badnavirus 1 NC_017830.1   (7141)  -----------------------------------------------------------------------------------
```

Section 331

```
                                        (27391) 27391    27400     27410     27420     27430     27440     27450     27460     27473
SARS-CoV-2 Reference Genome NC_045512.2 (27256) ATGAGGACTTTTAAAGTTTCCATTTGGAATCTTGATTACATCATAAACCTCATAATTAAAAATTTATCTAAGTCACTAACTGA
         Fig badnavirus 1 NC_017830.1   (7141)  -----------------------------------------------------------------------------------
```

Section 332

```
                                        (27474) 27474 27480     27490     27500     27510     27520     27530     27540           27556
SARS-CoV-2 Reference Genome NC_045512.2 (27339) GAATAAATATTCTCAATTAGATGAAGAGCAACCAATGGAGATTGATTAAACGAACATGAAAATTATTCTTTTCTTGGCACTGA
         Fig badnavirus 1 NC_017830.1   (7141)  -----------------------------------------------------------------------------------
```

Section 333

```
                                        (27557) 27557        27570     27580     27590     27600     27610     27620              27639
SARS-CoV-2 Reference Genome NC_045512.2 (27422) TAACACTCGCTACTTGTGAGCTTTATCACTACCAAGAGTGTGTTAGAGGTACAACAGTACTTTTAAAAGAACCTTGCTCTTCT
         Fig badnavirus 1 NC_017830.1   (7141)  -----------------------------------------------------------------------------------
```

Section 334

```
                                        (27640) 27640     27650     27660     27670     27680     27690     27700     27710       27722
SARS-CoV-2 Reference Genome NC_045512.2 (27505) GGAACATACGAGGGCAATTCACCATTTCATCCTCTAGCTGATAACAAATTTGCACTGACTTGCTTTAGCACTCAATTTGCTTT
         Fig badnavirus 1 NC_017830.1   (7141)  -----------------------------------------------------------------------------------
```

Section 335

```
                                        (27723) 27723  27730     27740     27750     27760     27770     27780     27790          27805
SARS-CoV-2 Reference Genome NC_045512.2 (27588) TGCTTGTCCTGACGGCGTAAAACACGTCTATCAGTTACGTGCCAGATCAGTTTCACCTAAACTGTTCATCAGACAAGAGGAAG
         Fig badnavirus 1 NC_017830.1   (7141)  -----------------------------------------------------------------------------------
```

Section 336

```
                                        (27806) 27806         27820     27830     27840     27850     27860     27870             27888
SARS-CoV-2 Reference Genome NC_045512.2 (27671) TTCAAGAACTTTACTCTCCAATTTTTCTTATTGTTGCGGCAATAGTGTTTATAACACTTTGCTTCACACTCAAAAGAAAGACA
         Fig badnavirus 1 NC_017830.1   (7141)  -----------------------------------------------------------------------------------
```

```
                                                          Section 337
                                          (27889) 27889      27900      27910      27920      27930      27940      27950      27960  27971
SARS-CoV-2 Reference Genome NC_045512.2 (27754) GAATGATTGAACTTTCATTAATTGACTTCTATTTGTGCTTTTTAGCCTTTCTGCTATTCCTTGTTTTAATTATGCTTATTATC
        Fig badnavirus 1 NC_017830.1    (7141) -----------------------------------------------------------------------------------
                                                          Section 338
                                          (27972) 27972   27980      27990      28000      28010      28020      28030      28040    28054
SARS-CoV-2 Reference Genome NC_045512.2 (27837) TTTTGGTTCTCACTTGAACTGCAAGATCATAATGAAACTTGTCACGCCTAAACGAACATGAAATTTCTTGTTTTCTTAGGAAT
        Fig badnavirus 1 NC_017830.1    (7141) -----------------------------------------------------------------------------------
                                                          Section 339
                                          (28055) 28055 28060      28070      28080      28090      28100      28110      28120      28137
SARS-CoV-2 Reference Genome NC_045512.2 (27920) CATCACAACTGTAGCTGCATTTCACCAAGAATGTAGTTTACAGTCATGTACTCAACATCAACCATATGTAGTTGATGACCCGT
        Fig badnavirus 1 NC_017830.1    (7141) -----------------------------------------------------------------------------------
                                                          Section 340
                                          (28138) 28138       28150      28160      28170      28180      28190      28200    28210 28220
SARS-CoV-2 Reference Genome NC_045512.2 (28003) GTCCTATTCACTTCTATTCTAAATGGTATATTAGAGTAGGAGCTAGAAAATCAGCACCTTTAATTGAATTGTGCGTGGATGAG
        Fig badnavirus 1 NC_017830.1    (7141) -----------------------------------------------------------------------------------
                                                          Section 341
                                          (28221) 28221    28230      28240      28250      28260      28270      28280      28290    28303
SARS-CoV-2 Reference Genome NC_045512.2 (28086) GCTGGTTCTAAATCACCCATTCAGTACATCGATATCGGTAATTATACAGTTTCCTGTTTACCTTTTACAATTAATTGCCAGGA
        Fig badnavirus 1 NC_017830.1    (7141) -----------------------------------------------------------------------------------
                                                          Section 342
                                          (28304) 28304 28310      28320      28330      28340      28350      28360      28370      28386
SARS-CoV-2 Reference Genome NC_045512.2 (28169) ACCTAAATTGGGTAGTCTTGTAGTGCGTTGTTCGTTCTATGAAGACTTTTTAGAGTATCATGACGTTCGTGTTGTTTTAGATT
        Fig badnavirus 1 NC_017830.1    (7141) -----------------------------------------------------------------------------------
                                                          Section 343
                                          (28387) 28387       28400      28410      28420      28430      28440      28450          28469
SARS-CoV-2 Reference Genome NC_045512.2 (28252) TCATCTAAACGAACAAACTAAAATGTCTGATAATGGACCCCAAAATCAGCGAAATGCACCCCGCATTACGTTTGGTGGACCCT
        Fig badnavirus 1 NC_017830.1    (7141) -----------------------------------------------------------------------------------
```

```
                                                                                                  Section 344
                                       (28470) 28470     28480     28490     28500     28510     28520     28530     28540       28552
SARS-CoV-2 Reference Genome NC_045512.2 (28335) CAGATTCAACTGGCAGTAACCAGAATGGAGAACGCAGTGGGGCGCGATCAAAACAACGTCGGCCCCAAGGTTTACCCAATAAT
         Fig badnavirus 1 NC_017830.1  (7141) -----------------------------------------------------------------------------------
                                                                                                  Section 345
                                       (28553) 28553  28560     28570     28580     28590     28600     28610     28620          28635
SARS-CoV-2 Reference Genome NC_045512.2 (28418) ACTGCGTCTTGGTTCACCGCTCTCACTCAACATGGCAAGGAAGACCTTAAATTCCCTCGAGGACAAGGCGTTCCAATTAACAC
         Fig badnavirus 1 NC_017830.1  (7141) -----------------------------------------------------------------------------------
                                                                                                  Section 346
                                       (28636) 28636         28650     28660     28670     28680     28690     28700             28718
SARS-CoV-2 Reference Genome NC_045512.2 (28501) CAATAGCAGTCCAGATGACCAAATTGGCTACTACCGAAGAGCTACCAGACGAATTCGTGGTGGTGACGGTAAAATGAAAGATC
         Fig badnavirus 1 NC_017830.1  (7141) -----------------------------------------------------------------------------------
                                                                                                  Section 347
                                       (28719) 28719      28730     28740     28750     28760     28770     28780     28790      28801
SARS-CoV-2 Reference Genome NC_045512.2 (28584) TCAGTCCAAGATGGTATTTCTACTACCTAGGAACTGGGCCAGAAGCTGGACTTCCCTATGGTGCTAACAAAGACGGCATCATA
         Fig badnavirus 1 NC_017830.1  (7141) -----------------------------------------------------------------------------------
                                                                                                  Section 348
                                       (28802) 28802   28810     28820     28830     28840     28850     28860     28870         28884
SARS-CoV-2 Reference Genome NC_045512.2 (28667) TGGGTTGCAACTGAGGGAGCCTTGAATACACCAAAAGATCACATTGGCACCCGCAATCCTGCTAACAATGCTGCAATCGTGCT
         Fig badnavirus 1 NC_017830.1  (7141) -----------------------------------------------------------------------------------
                                                                                                  Section 349
                                       (28885) 28885 28890     28900     28910     28920     28930     28940     28950           28967
SARS-CoV-2 Reference Genome NC_045512.2 (28750) ACAACTTCCTCAAGGAACAACATTGCCAAAAGGCTTCTACGCAGAAGGGAGCAGAGGCGGCAGTCAAGCCTCTTCTCGTTCCT
         Fig badnavirus 1 NC_017830.1  (7141) -----------------------------------------------------------------------------------
                                                                                                  Section 350
                                       (28968) 28968       28980     28990     29000     29010     29020     29030     29040   29050
SARS-CoV-2 Reference Genome NC_045512.2 (28833) CATCACGTAGTCGCAACAGTTCAAGAAATTCAACTCCAGGCAGCAGTAGGGGAACTTCTCCTGCTAGAATGGCTGGCAATGGC
         Fig badnavirus 1 NC_017830.1  (7141) -----------------------------------------------------------------------------------
```

```
                                                                                                                           Section 351
                                         (29051) 29051    29060     29070     29080     29090     29100     29110     29120       29133
SARS-CoV-2 Reference Genome NC_045512.2 (28916) GGTGATGCTGCTCTTGCTTTGCTGCTGCTTGACAGATTGAACCAGCTTGAGAGCAAAATGTCTGGTAAAGGCCAACAACAACA
        Fig badnavirus 1 NC_017830.1  (7141) -----------------------------------------------------------------------------------
                                                                                                                           Section 352
                                         (29134) 29134 29140     29150     29160     29170     29180     29190     29200         29216
SARS-CoV-2 Reference Genome NC_045512.2 (28999) AGGCCAAACTGTCACTAAGAAATCTGCTGCTGAGGCTTCTAAGAAGCCTCGGCAAAAACGTACTGCCACTAAAGCATACAATG
        Fig badnavirus 1 NC_017830.1  (7141) -----------------------------------------------------------------------------------
                                                                                                                           Section 353
                                         (29217) 29217        29230     29240     29250     29260     29270     29280              29299
SARS-CoV-2 Reference Genome NC_045512.2 (29082) TAACACAAGCTTTCGGCAGACGTGGTCCAGAACAAACCCAAGGAAATTTTGGGGACCAGGAACTAATCAGACAAGGAACTGAT
        Fig badnavirus 1 NC_017830.1  (7141) -----------------------------------------------------------------------------------
                                                                                                                           Section 354
                                         (29300) 29300     29310     29320     29330     29340     29350     29360     29370       29382
SARS-CoV-2 Reference Genome NC_045512.2 (29165) TACAAACATTGGCCGCAAATTGCACAATTTGCCCCCAGCGCTTCAGCGTTCTTCGGAATGTCGCGCATTGGCATGGAAGTCAC
        Fig badnavirus 1 NC_017830.1  (7141) -----------------------------------------------------------------------------------
                                                                                                                           Section 355
                                         (29383) 29383  29390     29400     29410     29420     29430     29440     29450          29465
SARS-CoV-2 Reference Genome NC_045512.2 (29248) ACCTTCGGGAACGTGGTTGACCTACACAGGTGCCATCAAATTGGATGACAAAGATCCAAATTTCAAAGATCAAGTCATTTTGC
        Fig badnavirus 1 NC_017830.1  (7141) -----------------------------------------------------------------------------------
                                                                                                                           Section 356
                                         (29466) 29466         29480     29490     29500     29510     29520     29530           29548
SARS-CoV-2 Reference Genome NC_045512.2 (29331) TGAATAAGCATATTGACGCATACAAAACATTCCCACCAACAGAGCCTAAAAAGGACAAAAAGAAGAAGGCTGATGAAACTCAA
        Fig badnavirus 1 NC_017830.1  (7141) -----------------------------------------------------------------------------------
                                                                                                                           Section 357
                                         (29549) 29549      29560     29570     29580     29590     29600     29610     29620    29631
SARS-CoV-2 Reference Genome NC_045512.2 (29414) GCCTTACCGCAGAGACAGAAGAAACAGCAAACTGTGACTCTTCTTCCTGCTGCAGATTTGGATGATTTCTCCAAACAATTGCA
        Fig badnavirus 1 NC_017830.1  (7141) -----------------------------------------------------------------------------------
```

```
                                                          Section 358
                                    (29632) 29632   29640     29650     29660     29670     29680     29690     29700        29714
SARS-CoV-2 Reference Genome NC_045512.2 (29497) ACAATCCATGAGCAGTGCTGACTCAACTCAGGCCTAAACTCATGCAGACCACACAAGGCAGATGGGCTATATAAACGTTTTCG
       Fig badnavirus 1 NC_017830.1  (7141) -----------------------------------------------------------------------------------
                                                          Section 359
                                    (29715) 29715 29720     29730     29740     29750     29760     29770     29780          29797
SARS-CoV-2 Reference Genome NC_045512.2 (29580) CTTTTCCGTTTACGATATATAGTCTACTCTTGTGCAGAATGAATTCTCGTAACTACATAGCACAAGTAGATGTAGTTAACTTT
       Fig badnavirus 1 NC_017830.1  (7141) -----------------------------------------------------------------------------------
                                                          Section 360
                                    (29798) 29798       29810     29820     29830     29840     29850     29860     29870  29880
SARS-CoV-2 Reference Genome NC_045512.2 (29663) AATCTCACATAGCAATCTTTAATCAGTGTGTAACATTAGGGAGGACTTGAAAGAGCCACCACATTTTCACCGAGGCCACGCGG
       Fig badnavirus 1 NC_017830.1  (7141) -----------------------------------------------------------------------------------
                                                          Section 361
                                    (29881) 29881    29890     29900     29910     29920     29930     29940     29950     29963
SARS-CoV-2 Reference Genome NC_045512.2 (29746) AGTACGATCGAGTGTACAGTGAACAATGCTAGGGAGAGCTGCCTATATGGAAGAGCCCTAATGTGTAAAATTAATTTTAGTAG
       Fig badnavirus 1 NC_017830.1  (7141) -----------------------------------------------------------------------------------
                                                          Section 362
                                    (29964) 29964 29970     29980     29990     30000     30010     30020             30038
SARS-CoV-2 Reference Genome NC_045512.2 (29829) TGCTATCCCCATGTGATTTTAATAGCTTCTTAGGAGAATGACAAAAAAAAAAAAAAAAAAAAAAAAAAAAAAAAA
       Fig badnavirus 1 NC_017830.1  (7141) ---------------------------------------------------------------------------
```
