# Supplementary material for: Patch type nucleotide sequence identities between genomes from many different species facilitate illegitimate recombination
Source: Sci Rep. 2026 Mar 30;16:10524. doi: 10.1038/s41598-026-44124-0 (PMC13035915; doi:10.1038/s41598-026-44124-0)
Supplement: Supplementary file 11 — Supplementary Material 11 [file 41598_2026_44124_MOESM11_ESM.pdf]

Omicron BA.1

Section 1

```
                                                       (1) 1        10        20        30        40        50        60          72
SARS-CoV-2 Reference Genome NC_045512.2                (1) ATTAAAGGTTTATACCTTCCCAGGTAACAAACCAACCAACTTTCGATCTCTTGTAGATCTGTTCTCTAAACG
hCoV-19/Botswana/R69B55_BHP_916539/2021_EPI_ISL_90...  (1) ------------------------------------------------------AGATCTGTTCTCTAAACG
hCoV-19/India/HR-MDU-IGIB1210605800489930/2022_EPI...  (1) ------------TACCTTCCCAGGTAACAAACCAACCAACTTTCGATCTCTTGTAGATCTGTTCTCTAAACG
hCoV-19/England/PLYM-332B917/2022_EPI_ISL_9062229 ...  (1) --------------------------------------------------TTGTAGATCTGTTCTCTAAACG
hCoV-19/Germany/HE-RKI-I-438397/2022_EPI_ISL_905522... (1) ------------------------------------CAACTTTCGATCTCTTGTAGATCTGTTCTCTAAACG
hCoV-19/USA/DE-CDC-LC0472738/2021_EPI_ISL_9049423...   (1) ------------------------------------------------------------------------
```

Section 2

```
                                                       (73) 73     80        90        100       110       120       130         144
SARS-CoV-2 Reference Genome NC_045512.2                (73) AACTTTAAAATCTGTGTGGCTGTCACTCGGCTGCATGCTTAGTGCACTCACGCAGTATAATTAATAACTAAT
hCoV-19/Botswana/R69B55_BHP_916539/2021_EPI_ISL_90...  (19) AACTTTAAAATCTGTGTGGCTGTCACTCGGCTGCATGCTTAGTGCACTCACGCAGTATAATTAATAACTAAT
hCoV-19/India/HR-MDU-IGIB1210605800489930/2022_EPI...  (61) AACTTTAAAATCTGTGTGGCTGTCACTCGGCTGCATGCTTAGTGCACTCACGCAGTATAATTAATAACTAAT
hCoV-19/England/PLYM-332B917/2022_EPI_ISL_9062229 ...  (23) AACTTTAAAATCTGTGTGGCTGTCACTCGGCTGCATGCTTAGTGCACTCACGCAGTATAATTAATAACTAAT
hCoV-19/Germany/HE-RKI-I-438397/2022_EPI_ISL_905522... (37) AACTTTAAAATCTGTGTGGCTGTCACTCGGCTGCATGCTTAGTGCACTCACGCAGTATAATTAATAACTAAT
hCoV-19/USA/DE-CDC-LC0472738/2021_EPI_ISL_9049423...   (1) AACTTTAAAATCTGTGTGGCTGTCACTCGGCTGCATGCTTAGTGCACTCACGCAGTATAATTAATAACTAAT
```

Section 3

```
                                                       (145) 145  150       160       170       180       190       200         216
SARS-CoV-2 Reference Genome NC_045512.2                (145) TACTGTCGTTGACAGGACACGAGTAACTCGTCTATCTTCTGCAGGCTGCTTACGGTTTCGTCCGTGTTGCAG
hCoV-19/Botswana/R69B55_BHP_916539/2021_EPI_ISL_90...  (91) TACTGTCGTTGACAGGACACGAGTAACTCGTCTATCTTCTGCAGGCTGCTTACGGTTTCGTCCGTGTTGCAG
hCoV-19/India/HR-MDU-IGIB1210605800489930/2022_EPI...  (133) TACTGTCGTTGACAGGACACGAGTAACTCGTCTATCTTCTGCAGGCTGCTTACGGTTTCGTCCGTGTTGCAG
hCoV-19/England/PLYM-332B917/2022_EPI_ISL_9062229 ...  (95) TACTGTCGTTGACAGGACACGAGTAACTCGTCTATCTTCTGCAGGCTGCTTACGGTTTCGTCCGTGTTGCAG
hCoV-19/Germany/HE-RKI-I-438397/2022_EPI_ISL_905522... (109) TACTGTCGTTGACAGGACACGAGTAACTCGTCTATCTTCTGCAGGCTGCTTACGGTTTCGTCCGTGTTGCAG
hCoV-19/USA/DE-CDC-LC0472738/2021_EPI_ISL_9049423...   (73) TACTGTCGTTGACAGGACACGAGTAACTCGTCTATCTTCTGCAGGCTGCTTACGGTTTCGTCCGTGTTGCAG
```

Section 4

```
                                                       (217) 217          230       240       250       260       270         288
SARS-CoV-2 Reference Genome NC_045512.2                (217) CCGATCATCAGCACATCTAGGTTTCGTCCGGGTGTGACCGAAAGGTAAGATGGAGAGCCTTGTCCCTGGTTT
hCoV-19/Botswana/R69B55_BHP_916539/2021_EPI_ISL_90...  (163) CCGATCATCAGCACATCTAGGTTTTGTCCGGGTGTGACCGAAAGGTAAGATGGAGAGCCTTGTCCCTGGTTT
hCoV-19/India/HR-MDU-IGIB1210605800489930/2022_EPI...  (205) CCGATCATCAGCACATCTAGGTTTTGTCCGGGTGTGACCGAAAGGTAAGATGGAGAGCCTTGTCCCTGGTTT
hCoV-19/England/PLYM-332B917/2022_EPI_ISL_9062229 ...  (167) CCGATCATCAGCACATCTAGGTTTTGTCCGGGTGTGACCGAAAGGTAAGATGGAGAGCCTTGTCCCTGGTTT
hCoV-19/Germany/HE-RKI-I-438397/2022_EPI_ISL_905522... (181) CCGATCATCAGCACATCTAGGTTTTGTCCGGGTGTGACCGAAAGGTAAGATGGAGAGCCTTGTCCCTGGTTT
hCoV-19/USA/DE-CDC-LC0472738/2021_EPI_ISL_9049423...   (145) CCGATCATCAGCACATCTAGGTTTTGTCCGGGTGTGACCGAAAGGTAAGATGGAGAGCCTTGTCCCTGGTTT
```

Omicron BA.1

Section 5

Sequence	Start	289–360
	(289)	289 300 310 320 330 340 350 360
SARS-CoV-2 Reference Genome NC_045512.2	(289)	CAACGAGAAAACACACGTCCAACTCAGTTTGCCTGTTTTACAGGTTCGCGACGTGCTCGTACGTGGCTTTGG
hCoV-19/Botswana/R69B55_BHP_916539/2021_EPI_ISL_90...	(235)	CAACGAGAAAACACACGTCCAACTCAGTTTGCCTGTTTTACAGGTTCGCGACGTGCTCGTACGTGGCTTTGG
hCoV-19/India/HR-MDU-IGIB1210605800489930/2022_EPI...	(277)	CAACGAGAAAACACACGTCCAACTCAGTTTGCCTGTTTTACAGGTTCGCGACGTGCTCGTACGTGGCTTTGG
hCoV-19/England/PLYM-332B917/2022_EPI_ISL_9062229_...	(239)	CAACGAGAAAACACACGTCCAACTCAGTTTGCCTGTTTTACAGGTTCGCGACGTGCTCGTACGTGGCTTTGG
hCoV-19/Germany/HE-RKI-I-438397/2022_EPI_ISL_905522...	(253)	CAACGAGAAAACACACGTCCAACTCAGTTTGCCTGTTTTACAGGTTCGCGACGTGCTCGTACGTGGCTTTGG
hCoV-19/USA/DE-CDC-LC0472738/2021_EPI_ISL_9049423...	(217)	CAACGAGAAAACACACGTCCAACTCAGTTTGCCTGTTTTACAGGTTCGCGACGTGCTCGTACGTGGCTTTGG

Section 6

Sequence	Start	361–432
	(361)	361 370 380 390 400 410 420 432
SARS-CoV-2 Reference Genome NC_045512.2	(361)	AGACTCCGTGGAGGAGGTCTTATCAGAGGCACGTCAACATCTTAAAGATGGCACTTGTGGCTTAGTAGAAGT
hCoV-19/Botswana/R69B55_BHP_916539/2021_EPI_ISL_90...	(307)	AGACTCCGTGGAGGAGGTCTTATCAGAGGCACGTCAACATCTTAAAGATGGCACTTGTGGCTTAGTAGAAGT
hCoV-19/India/HR-MDU-IGIB1210605800489930/2022_EPI...	(349)	AGACTCCGTGGAGGAGGTCTTATCAGAGGCACGTCAACATCTTAAAGATGGCACTTGTGGCTTAGTAGAAGT
hCoV-19/England/PLYM-332B917/2022_EPI_ISL_9062229_...	(311)	AGACTCCGTGGAGGAGGTCTTATCAGAGGCACGTCAACATCTTAAAGATGGCACTTGTGGCTTAGTAGAAGT
hCoV-19/Germany/HE-RKI-I-438397/2022_EPI_ISL_905522...	(325)	AGACTCCGTGGAGGAGGTCTTATCAGAGGCACGTCAACATCTTAAAGATGGCACTTGTGGCTTAGTAGAAGT
hCoV-19/USA/DE-CDC-LC0472738/2021_EPI_ISL_9049423...	(289)	AGACTCCGTGGAGGAGGTCTTATCAGAGGCACGTCAACATCTTAAAGATGGCACTTGTGGCTTAGTAGAAGT

Section 7

Sequence	Start	433–504
	(433)	433 440 450 460 470 480 490 504
SARS-CoV-2 Reference Genome NC_045512.2	(433)	TGAAAAAGGCGTTTTGCCTCAACTTGAACAGCCCTATGTGTTCATCAAACGTTCGGATGCTCGAACTGCACC
hCoV-19/Botswana/R69B55_BHP_916539/2021_EPI_ISL_90...	(379)	TGAAAAAGGCGTTTTGCCTCAACTTGAACAGCCCTATGTGTTCATCAAACGTTCGGATGCTCGAACTGCACC
hCoV-19/India/HR-MDU-IGIB1210605800489930/2022_EPI...	(421)	TGAAAAAGGCGTTTTGCCTCAACTTGAACAGCCCTATGTGTTCATCAAACGTTCGGATGCTCGAACTGCACC
hCoV-19/England/PLYM-332B917/2022_EPI_ISL_9062229_...	(383)	TGAAAAAGGCGTTTTGCCTCAACTTGAACAGCCCTATGTGTTCATCAAACGTTCGGATGCTCGAACTGCACC
hCoV-19/Germany/HE-RKI-I-438397/2022_EPI_ISL_905522...	(397)	TGAAAAAGGCGTTTTGCCTCAACTTGAACAGCCCTATGTGTTCATCAAACGTTCGGATGCTCGAACTGCACC
hCoV-19/USA/DE-CDC-LC0472738/2021_EPI_ISL_9049423...	(361)	TGAAAAAGGCGTTTTGCCTCAACTTGAACAGCCCTATGTGTTCATCAAACGTTCGGATGCTCGAACTGCACC

Section 8

Sequence	Start	505–576
	(505)	505 510 520 530 540 550 560 576
SARS-CoV-2 Reference Genome NC_045512.2	(505)	TCATGGTCATGTTATGGTTGAGCTGGTAGCAGAACTCGAAGGCATTCAGTACGGTCGTAGTGGTGAGACACT
hCoV-19/Botswana/R69B55_BHP_916539/2021_EPI_ISL_90...	(451)	TCATGGTCATGTTATGGTTGAGCTGGTAGCAGAACTCGAAGGCATTCAGTACGGTCGTAGTGGTGAGACACT
hCoV-19/India/HR-MDU-IGIB1210605800489930/2022_EPI...	(493)	TCATGGTCATGTTATGGTTGAGCTGGTAGCAGAACTCGAAGGCATTCAGTACGGTCGTAGTGGTGAGACACT
hCoV-19/England/PLYM-332B917/2022_EPI_ISL_9062229_...	(455)	TCATGGTCATGTTATGGTTGAGCTGGTAGCAGAACTCGAAGGCATTCAGTACGGTCGTAGTGGTGAGACACT
hCoV-19/Germany/HE-RKI-I-438397/2022_EPI_ISL_905522...	(469)	TCATGGTCATGTTATGGTTGAGCTGGTAGCAGAACTCGAAGGCATTCAGTACGGTCGTAGTGGTGAGACACT
hCoV-19/USA/DE-CDC-LC0472738/2021_EPI_ISL_9049423...	(433)	TCATGGTCATGTTATGGTTGAGCTGGTAGCAGAACTCGAAGGCATTCAGTACGGTCGTAGTGGTGAGACACT

Section 9

	(577)	577 … 590 … 600 … 610 … 620 … 630 … 648
SARS-CoV-2 Reference Genome NC_045512.2	(577)	TGGTGTCCTTGTCCCTCATGTGGGCGAAATACCAGTGGCTTACCGCAAGGTTCTTCTTCGTAAGAACGGTAA
hCoV-19/Botswana/R69B55_BHP_916539/2021_EPI_ISL_90...	(523)	TGGTGTCCTTGTCCCTCATGTGGGCGAAATACCAGTGGCTTACCGCAAGGTTCTTCTTCGTAAGAACGGTAA
hCoV-19/India/HR-MDU-IGIB1210605800489930/2022_EPI...	(565)	TGGTGTCCTTGTCCCTCATGTGGGCGAAATACCAGTGGCTTACCGCAAGGTTCTTCTTCGTAAGAACGGTAA
hCoV-19/England/PLYM-332B917/2022_EPI_ISL_9062229_...	(527)	TGGTGTCCTTGTCCCTCATGTGGGCGAAATACCAGTGGCTTACCGCAAGGTTCTTCTTCGTAAGAACGGTAA
hCoV-19/Germany/HE-RKI-I-438397/2022_EPI_ISL_905522...	(541)	TGGTGTCCTTGTCCCTCATGTGGGCGAAATACCAGTGGCTTACCGCAAGGTTCTTCTTCGTAAGAACGGTAA
hCoV-19/USA/DE-CDC-LC0472738/2021_EPI_ISL_9049423...	(505)	TGGTGTCCTTGTCCCTCATGTGGGCGAAATACCAGTGGCTTACCGCAAGGTTCTTCTTCGTAAGAACGGTAA

Section 10

	(649)	649 … 660 … 670 … 680 … 690 … 700 … 710 … 720
SARS-CoV-2 Reference Genome NC_045512.2	(649)	TAAAGGAGCTGGTGGCCATAGTTACGGCGCCGATCTAAAGTCATTTGACTTAGGCGACGAGCTTGGCACTGA
hCoV-19/Botswana/R69B55_BHP_916539/2021_EPI_ISL_90...	(595)	TAAAGGAGCTGGTGGCCATAGTTACGGCGCCGATCTAAAGTCATTTGACTTAGGCGACGAGCTTGGCACTGA
hCoV-19/India/HR-MDU-IGIB1210605800489930/2022_EPI...	(637)	TAAAGGAGCTGGTGGCCATAGTTACGGCGCCGATCTAAAGTCATTTGACTTAGGCGACGAGCTTGGCACTGA
hCoV-19/England/PLYM-332B917/2022_EPI_ISL_9062229_...	(599)	TAAAGGAGCTGGTGGCCATAGTTACGGCGCCGATCTAAAGTCATTTGACTTAGGCGACGAGCTTGGCACTGA
hCoV-19/Germany/HE-RKI-I-438397/2022_EPI_ISL_905522...	(613)	TAAAGGAGCTGGTGGCCATAGTTACGGCGCCGATCTAAAGTCATTTGACTTAGGCGACGAGCTTGGCACTGA
hCoV-19/USA/DE-CDC-LC0472738/2021_EPI_ISL_9049423...	(577)	TAAAGGAGCTGGTGGCCATAGTTACGGCGCCGATCTAAAGTCATTTGACTTAGGCGACGAGCTTGGCACTGA

Section 11

	(721)	721 … 730 … 740 … 750 … 760 … 770 … 780 … 792
SARS-CoV-2 Reference Genome NC_045512.2	(721)	TCCTTATGAAGATTTTCAAGAAAACTGGAACACTAAACATAGCAGTGGTGTTACCCGTGAACTCATGCGTGA
hCoV-19/Botswana/R69B55_BHP_916539/2021_EPI_ISL_90...	(667)	TCCTTATGAAGATTTTCAAGAAAACTGGAACACTAAACATAGCAGTGGTGTTACCCGTGAACTCATGCGTGA
hCoV-19/India/HR-MDU-IGIB1210605800489930/2022_EPI...	(709)	TCCTTATGAAGATTTTCAAGAAAACTGGAACACTAAACATAGCAGTGGTGTTACCCGTGAACTCATGCGTGA
hCoV-19/England/PLYM-332B917/2022_EPI_ISL_9062229_...	(671)	TCCTTATGAAGATTTTCAAGAAAACTGGAACACTAAACATAGCAGTGGTGTTACCCGTGAACTCATGCGTGA
hCoV-19/Germany/HE-RKI-I-438397/2022_EPI_ISL_905522...	(685)	TCCTTATGAAGATTTTCAAGAAAACTGGAACACTAAACATAGCAGTGGTGTTACCCGTGAACTCATGCGTGA
hCoV-19/USA/DE-CDC-LC0472738/2021_EPI_ISL_9049423...	(649)	TCCTTATGAAGATTTTCAAGAAAACTGGAACACTAAACATAGCAGTGGTGTTACCCGTGAACTCATGCGTGA

Section 12

	(793)	793 … 800 … 810 … 820 … 830 … 840 … 850 … 864
SARS-CoV-2 Reference Genome NC_045512.2	(793)	GCTTAACGGAGGGGCATACACTCGCTATGTCGATAACAACTTCTGTGGCCCTGATGGCTACCCTCTTGAGTG
hCoV-19/Botswana/R69B55_BHP_916539/2021_EPI_ISL_90...	(739)	GCTTAACGGAGGGGCATACACTCGCTATGTCGATAACAACTTCTGTGGCCCTGATGGCTACCCTCTTGAGTG
hCoV-19/India/HR-MDU-IGIB1210605800489930/2022_EPI...	(781)	GCTTAACGGAGGGGCATACACTCGCTATGTCGATAACAACTTCTGTGGCCCTGATGGCTACCCTCTTGAGTG
hCoV-19/England/PLYM-332B917/2022_EPI_ISL_9062229_...	(743)	GCTTAACGGAGGGGCATACACTCGCTATGTCGATAACAACTTCTGTGGCCCTGATGGCTACCCTCTTGAGTG
hCoV-19/Germany/HE-RKI-I-438397/2022_EPI_ISL_905522...	(757)	GCTTAACGGAGGGGCATACACTCGCTATGTCGATAACAACTTCTGTGGCCCTGATGGCTACCCTCTTGAGTG
hCoV-19/USA/DE-CDC-LC0472738/2021_EPI_ISL_9049423...	(721)	GCTTAACGGAGGGGCATACACTCGCTATGTCGATAACAACTTCTGTGGCCCTGATGGCTACCCTCTTGAGTG

Section 13

		865 870 880 890 900 910 920 936
	(865)	
SARS-CoV-2 Reference Genome NC 045512.2	(865)	CATTAAAGACCTTCTAGCACGTGCTGGTAAAGCTTCATGCACTTTGTCCGAACAACTGGACTTTATTGACAC
hCoV-19/Botswana/R69B55 BHP 916539/2021 EPI ISL 90...	(811)	CATTAAAGACCTTCTAGCACGTGCTGGTAAAGCTTCATGCACTTTGTCCGAACAACTGGACTTTATTGACAC
hCoV-19/India/HR-MDU-IGIB1210605800489930/2022 EPI...	(853)	CATTAAAGACCTTCTAGCACGTGCTGGTAAAGCTTCATGCACTTTGTCCGAACAACTGGACTTTATTGACAC
hCoV-19/England/PLYM-332B917/2022 EPI ISL 9062229 ...	(815)	CATTAAAGACCTTCTAGCACGTGCTGGTAAAGCTTCATGCACTTTGTCCGAACAACTGGACTTTATTGACAC
hCoV-19/Germany/HE-RKI-I-438397/2022 EPI ISL 905522...	(829)	CATTAAAGACCTTCTAGCACGTGCTGGTAAAGCTTCATGCACTTTGTCCGAACAACTGGACTTTATTGACAC
hCoV-19/USA/DE-CDC-LC0472738/2021_EPI_ISL_9049423...	(793)	CATTAAAGACCTTCTAGCACGTGCTGGTAAAGCTTCATGCACTTTGTCCGAACAACTGGACTTTATTGACAC

Section 14

		937 950 960 970 980 990 1008
	(937)	
SARS-CoV-2 Reference Genome NC 045512.2	(937)	TAAGAGGGGTGTATACTGCTGCCGTGAACATGAGCATGAAATTGCTTGGTACACGGAACGTTCTGAAAAGAG
hCoV-19/Botswana/R69B55 BHP 916539/2021 EPI ISL 90...	(883)	TAAGAGGGGTGTATACTGTTGCCGTGAACATGAGCATGAAATTGCTTGGTACACGGAACGTTCTGAAAAGAG
hCoV-19/India/HR-MDU-IGIB1210605800489930/2022 EPI...	(925)	TAAGAGGGGTGTATACTGCTGCCGTGAACATGAGCATGAAATTGCTTGGTACACGGAACGTTCTGAAAAGAG
hCoV-19/England/PLYM-332B917/2022 EPI ISL 9062229 ...	(887)	TAAGAGGGGTGTATACTGCTGTCGTGAACATGAGCATGAAATTGCTTGGTACACGGAACGTTCTGAAAAGAG
hCoV-19/Germany/HE-RKI-I-438397/2022 EPI ISL 905522...	(901)	TAAGAGGGGTGTATACTGCTGCCGTGAACATGAGCATGAAATTGCTTGGTACACGGAACGTTCTGAAAAGAG
hCoV-19/USA/DE-CDC-LC0472738/2021_EPI_ISL_9049423...	(865)	TAAGAGGGGTGTATACTGCTGCCGTGAACATGAGCATGAAATTGCTTGGTACACGGAACGTTCTGAAAAGAG

Section 15

		1009 1020 1030 1040 1050 1060 1070 1080
	(1009)	
SARS-CoV-2 Reference Genome NC 045512.2	(1009)	CTATGAATTGCAGACACCTTTTGAAATTAAATTGGCAAAGAAATTTGACACCTTCAATGGGGAATGTCCAAA
hCoV-19/Botswana/R69B55 BHP 916539/2021 EPI ISL 90...	(955)	CTATGAATTGCAGACACCTTTTGAAATTAAATTGGCAAAGAAATTTGACACCTTCAATGGGGAATGTCCAAA
hCoV-19/India/HR-MDU-IGIB1210605800489930/2022 EPI...	(997)	CTATGAATTGCAGACACCTTTTGAAATTAAATTGGCAAAGAAATTTGACACCTTCAATGGGGAATGTCCAAA
hCoV-19/England/PLYM-332B917/2022 EPI ISL 9062229 ...	(959)	CTATGAATTGCAGACACCTTTTGAAATTAAATTGGCAAAGAAATTTGACACCTTCAATGGGGAATGTCCAAA
hCoV-19/Germany/HE-RKI-I-438397/2022 EPI ISL 905522...	(973)	CTATGAATTGCAGACACCTTTTGAAATTAAATTGGCAAAGAAATTTGACACCTTCAATGGGGAATGTCCAAA
hCoV-19/USA/DE-CDC-LC0472738/2021_EPI_ISL_9049423...	(937)	CTATGAATTGCAGACACCTTTTGAAATTAAATTGGCAAAGAAATTTGACACCTTCAATGGGGAATGTCCAAA

Section 16

		1081 1090 1100 1110 1120 1130 1140 1152
	(1081)	
SARS-CoV-2 Reference Genome NC 045512.2	(1081)	TTTTGTATTTCCCTTAAATTCCATAATCAAGACTATTCAACCAAGGGTTGAAAAGAAAAAGCTTGATGGCTT
hCoV-19/Botswana/R69B55 BHP 916539/2021 EPI ISL 90...	(1027)	TTTTGTATTTCCCTTAAATTCCATAATCAAGACTATTCAACCAAGGGTTGAAAAGAAAAAGCTTGATGGCTT
hCoV-19/India/HR-MDU-IGIB1210605800489930/2022 EPI...	(1069)	TTTTGTATTTCCCTTAAATTCCATAATCAAGACTATTCAACCAAGGGTTGAAAAGAAAAAGCTTGATGGCTT
hCoV-19/England/PLYM-332B917/2022 EPI ISL 9062229 ...	(1031)	TTTTGTATTTCCCTTAAATTCCATAATCAAGACTATTCAACCAAGGGTTGAAAAGAAAAAGCTTGATGGCTT
hCoV-19/Germany/HE-RKI-I-438397/2022 EPI ISL 905522...	(1045)	TTTTGTATTTCCCTTAAATTCCATAATCAAGACTATTCAACCAAGGGTTGAAAAGAAAAAGCTTGATGGCTT
hCoV-19/USA/DE-CDC-LC0472738/2021_EPI_ISL_9049423...	(1009)	TTTTGTATTTCCCTTAAATTCCATAATCAAGACTATTCAACCAAGGGTTGAAAAGAAAAAGCTTGATGGCTT

Omicron BA.1

Section 17

	(1153)	1153 1160 1170 1180 1190 1200 1210 1224
SARS-CoV-2 Reference Genome NC_045512.2	(1153)	TATGGGTAGAATTCGATCTGTCTATCCAGTTGCGTCACCAAATGAATGCAACCAAATGTGCCTTTCAACTCT
hCoV-19/Botswana/R69B55_BHP_916539/2021_EPI_ISL_90...	(1099)	TATGGGTAGAATTCGATCTGTCTATCCAGTTGCGTCACCAAATGAATGCAACCAAATGTGCCTTTCAACTCT
hCoV-19/India/HR-MDU-IGIB1210605800489930/2022_EPI...	(1141)	TATGGGTAGAATTCGATCTGTCTATCCAGTTGCGTCACCAAATGAATGCAACCAAATGTGCCTTTCAACTCT
hCoV-19/England/PLYM-332B917/2022_EPI_ISL_9062229 ...	(1103)	TATGGGTAGAATTCGATCTGTCTATCCAGTTGCGTCACCAAATGAATGCAACCAAATGTGCCTTTCAACTCT
hCoV-19/Germany/HE-RKI-I-438397/2022_EPI_ISL_905522...	(1117)	TATGGGTAGAATTCGATCTGTCTATCCAGTTGCGTCACCAAATGAATGCAACCAAATGTGCCTTTCAACTCT
hCoV-19/USA/DE-CDC-LC0472738/2021_EPI_ISL_9049423...	(1081)	TATGGGTAGAATTCGATCTGTCTATCCAGTTGCGTCACCAAATGAATGCAACCAAATGTGCCTTTCAACTCT

Section 18

	(1225)	1225 1230 1240 1250 1260 1270 1280 1296
SARS-CoV-2 Reference Genome NC_045512.2	(1225)	CATGAAGTGTGATCATTGTGGTGAAACTTCATGGCAGACGGGCGATTTTGTTAAAGCCACTTGCGAATTTTG
hCoV-19/Botswana/R69B55_BHP_916539/2021_EPI_ISL_90...	(1171)	CATGAAGTGTGATCATTGTGGTGAAACTTCATGGCAGACGGGCGATTTTGTTAAAGCCACTTGCGAATTTTG
hCoV-19/India/HR-MDU-IGIB1210605800489930/2022_EPI...	(1213)	CATGAAGTGTGATCATTGTGGTGAAACTTCATGGCAGACGGGCGATTTTGTTAAAGCCACTTGCGAATTTTG
hCoV-19/England/PLYM-332B917/2022_EPI_ISL_9062229 ...	(1175)	CATGAAGTGTGATCATTGTGGTGAAACTTCATGGCAGACGGGCGATTTTGTTAAAGCCACTTGCGAATTTTG
hCoV-19/Germany/HE-RKI-I-438397/2022_EPI_ISL_905522...	(1189)	CATGAAGTGTGATCATTGTGGTGAAACTTCATGGCAGACGGGCGATTTTGTTAAAGCCACTTGCGAATTTTG
hCoV-19/USA/DE-CDC-LC0472738/2021_EPI_ISL_9049423...	(1153)	CATGAAGTGTGATCATTGTGGTGAAACTTCATGGCAGACGGGCGATTTTGTTAAAGCCACTTGCGAATTTTG

Section 19

	(1297)	1297 1310 1320 1330 1340 1350 1368
SARS-CoV-2 Reference Genome NC_045512.2	(1297)	TGGCACTGAGAATTTGACTAAAGAAGGTGCCACTACTTGTGGTTACTTACCCCAAAATGCTGTTGTTAAAAT
hCoV-19/Botswana/R69B55_BHP_916539/2021_EPI_ISL_90...	(1243)	TGGCACTGAGAATTTGACTAAAGAAGGTGCCACTACTTGTGGTTACTTACCCCAAAATGCTGTTGTTAAAAT
hCoV-19/India/HR-MDU-IGIB1210605800489930/2022_EPI...	(1285)	TGGCACTGAGAATTTGACTAAAGAAGGTGCCACTACTTGTGGTTACTTACCCCAAAATGCTGTTGTTAAAAT
hCoV-19/England/PLYM-332B917/2022_EPI_ISL_9062229 ...	(1247)	TGGCACTGAGAATTTGACTAAAGAAGGTGCCACTACTTGTGGTTACTTACCCCAAAATGCTGTTGTTAAAAT
hCoV-19/Germany/HE-RKI-I-438397/2022_EPI_ISL_905522...	(1261)	TGGCACTGAGAATTTGACTAAAGAAGGTGCCACTACTTGTGGTTACTTACCCCAAAATGCTGTTGTTAAAAT
hCoV-19/USA/DE-CDC-LC0472738/2021_EPI_ISL_9049423...	(1225)	TGGCACTGAGAATTTGACTAAAGAAGGTGCCACTACTTGTGGTTACTTACCCCAAAATGCTGTTGTTAAAAT

Section 20

	(1369)	1369 1380 1390 1400 1410 1420 1430 1440
SARS-CoV-2 Reference Genome NC_045512.2	(1369)	TTATTGTCCAGCATGTCACAATTCAGAAGTAGGACCTGAGCATAGTCTTGCCGAATACCATAATGAATCTGG
hCoV-19/Botswana/R69B55_BHP_916539/2021_EPI_ISL_90...	(1315)	TTATTGTCCAGCATGTCACAATTCAGAAGTAGGACCTGAGCATAGTCTTGCCGAATACCATAATGAATCTGG
hCoV-19/India/HR-MDU-IGIB1210605800489930/2022_EPI...	(1357)	TTATTGTCCAGCATGTCACAATTCAGAAGTAGGACCTGAGCATAGTCTTGCCGAATACCATAATGAATCTGG
hCoV-19/England/PLYM-332B917/2022_EPI_ISL_9062229 ...	(1319)	TTATTGTCCAGCATGTCACAATTCAGAAGTAGGACCTGAGCATAGTCTTGCCGAATACCATAATGAATCTGG
hCoV-19/Germany/HE-RKI-I-438397/2022_EPI_ISL_905522...	(1333)	TTATTGTCCAGCATGTCACAATTCAGAAGTAGGACCTGAGCATAGTCTTGCCGAATACCATAATGAATCTGG
hCoV-19/USA/DE-CDC-LC0472738/2021_EPI_ISL_9049423...	(1297)	TTATTGTCCAGCATGTCACAATTCAGAAGTAGGACCTGAGCATAGTCTTGCCGAATACCATAATGAATCTGG

Section 21

		1441 1450 1460 1470 1480 1490 1500 1512
	(1441)	
SARS-CoV-2 Reference Genome NC_045512.2	(1441)	CTTGAAAACCATTCTTCGTAAGGGTGGTCGCACTATTGCCTTTGGAGGCTGTGTGTTCTCTTATGTTGGTTG
hCoV-19/Botswana/R69B55_BHP_916539/2021_EPI_ISL_90...	(1387)	CTTGAAAACCATTCTTCGTAAGGGTGGTCGCACTATTGCCTTTGGAGGCTGTGTGTTCTCTTATGTTGGTTG
hCoV-19/India/HR-MDU-IGIB1210605800489930/2022_EPI...	(1429)	CTTGAAAACCATTCTTCGTAAGGGTGGTCGCACTATTGCCTTTGGAGGCTGTGTGTTCTCTTATGTTGGTTG
hCoV-19/England/PLYM-332B917/2022_EPI_ISL_9062229_...	(1391)	CTTGAAAACCATTCTTCGTAAGGGTGGTCGCACTATTGCCTTTGGAGGCTGTGTGTTCTCTTATGTTGGTTG
hCoV-19/Germany/HE-RKI-I-438397/2022_EPI_ISL_905522...	(1405)	CTTGAAAACCATTCTTCGTAAGGGTGGTCGCACTATTGCCTTTGGAGGCTGTGTGTTCTCTTATGTTGGTTG
hCoV-19/USA/DE-CDC-LC0472738/2021_EPI_ISL_9049423...	(1369)	CTTGAAAACCATTCTTCGTAAGGGTGGTCGCACTATTGCCTTTGGAGGCTGTGTGTTCTCTTATGTTGGTTG

Section 22

		1513 1520 1530 1540 1550 1560 1570 1584
	(1513)	
SARS-CoV-2 Reference Genome NC_045512.2	(1513)	CCATAACAAGTGTGCCTATTGGGTTCCACGTGCTAGCGCTAACATAGGTTGTAACCATACAGGTGTTGTTGG
hCoV-19/Botswana/R69B55_BHP_916539/2021_EPI_ISL_90...	(1459)	CCATAACAAGTGTGCCTATTGGGTTCCACGTGCTAGCGCTAACATAGGTTGTAACCATACAGGTGTTGTTGG
hCoV-19/India/HR-MDU-IGIB1210605800489930/2022_EPI...	(1501)	CCATAACAAGTGTGCCTATTGGGTTCCACGTGCTAGCGCTAACATAGGTTGTAACCATACAGGTGTTGTTGG
hCoV-19/England/PLYM-332B917/2022_EPI_ISL_9062229_...	(1463)	CCATAACAAGTGTGCCTATTGGGTTCCACGTGCTAGCGCTAACATAGGTTGTAACCATACAGGTGTTGTTGG
hCoV-19/Germany/HE-RKI-I-438397/2022_EPI_ISL_905522...	(1477)	CCATAACAAGTGTGCCTATTGGGTTCCACGTGCTAGCGCTAACATAGGTTGTAACCATACAGGTGTTGTTGG
hCoV-19/USA/DE-CDC-LC0472738/2021_EPI_ISL_9049423...	(1441)	CCATAACAAGTGTGCCTATTGGGTTCCACGTGCTAGCGCTAACATAGGTTGTAACCATACAGGTGTTGTTGG

Section 23

		1585 1590 1600 1610 1620 1630 1640 1656
	(1585)	
SARS-CoV-2 Reference Genome NC_045512.2	(1585)	AGAAGGTTCCGAAGGTCTTAATGACAACCTTCTTGAAATACTCCAAAAAGAGAAAGTCAACATCAATATTGT
hCoV-19/Botswana/R69B55_BHP_916539/2021_EPI_ISL_90...	(1531)	AGAAGGTTCCGAAGGTCTTAATGACAACCTTCTTGAAATACTCCAAAAAGAGAAAGTCAACATCAATATTGT
hCoV-19/India/HR-MDU-IGIB1210605800489930/2022_EPI...	(1573)	AGAAGGTTCCGAAGGTCTTAATGACAACCTTCTTGAAATACTCCAAAAAGAGAAAGTCAACATCAATATTGT
hCoV-19/England/PLYM-332B917/2022_EPI_ISL_9062229_...	(1535)	AGAAGGTTCCGAAGGTCTTAATGACAACCTTCTTGAAATACTCCAAAAAGAGAAAGTCAACATCAATATTGT
hCoV-19/Germany/HE-RKI-I-438397/2022_EPI_ISL_905522...	(1549)	AGAAGGTTCCGAAGGTCTTAATGACAACCTTCTTGAAATACTCCAAAAAGAGAAAGTCAACATCAATATTGT
hCoV-19/USA/DE-CDC-LC0472738/2021_EPI_ISL_9049423...	(1513)	AGAAGGTTCCGAAGGTCTTAATGACAACCTTCTTGAAATACTCCAAAAAGAGAAAGTCAACATCAATATTGT

Section 24

		1657 1670 1680 1690 1700 1710 1728
	(1657)	
SARS-CoV-2 Reference Genome NC_045512.2	(1657)	TGGTGACTTTAAACTTAATGAAGAGATCGCCATTATTTTGGCATCTTTTTCTGCTTCCACAAGTGCTTTTGT
hCoV-19/Botswana/R69B55_BHP_916539/2021_EPI_ISL_90...	(1603)	TGGTGACTTTAAACTTAATGAAGAGATCGCCATTATTTTGGCATCTTTTTCTGCTTCCACAAGTGCTTTTGT
hCoV-19/India/HR-MDU-IGIB1210605800489930/2022_EPI...	(1645)	TGGTGACTTTAAACTTAATGAAGAGATCGCCATTATTTTGGCATCTTTTTCTGCTTCCACAAGTGCTTTTGT
hCoV-19/England/PLYM-332B917/2022_EPI_ISL_9062229_...	(1607)	TGGTGACTTTAAACTTAATGAAGAGATCGCCATTATTTTGGCATCTTTTTCTGCTTCCACAAGTGCTTTTGT
hCoV-19/Germany/HE-RKI-I-438397/2022_EPI_ISL_905522...	(1621)	TGGTGACTTTAAACTTAATGAAGAGATCGCCATTATTTTGGCATCTTTTTCTGCTTCCACAAGTGCTTTTGT
hCoV-19/USA/DE-CDC-LC0472738/2021_EPI_ISL_9049423...	(1585)	TGGTGACTTTAAACTTAATGAAGAGATCGCCATTATTTTGGCATCTTTTTCTGCTTCCACAAGTGCTTTTGT

Section 25

	(1729)	1729 1740 1750 1760 1770 1780 1790 1800
SARS-CoV-2 Reference Genome NC_045512.2	(1729)	GGAAACTGTGAAAGGTTTGGATTATAAAGCATTCAAACAAATTGTTGAATCCTGTGGTAATTTTAAAGTTAC
hCoV-19/Botswana/R69B55_BHP_916539/2021_EPI_ISL_90...	(1675)	GGAAACTGTGAAAGGTTTGGATTATAAAGCATTCAAACAAATTGTTGAATCCTGTGGTAATTTTAAAGTTAC
hCoV-19/India/HR-MDU-IGIB1210605800489930/2022_EPI...	(1717)	GGAAACTGTGAAAGGTTTGGATTATAAAGCATTCAAACAAATTGTTGAATCCTGTGGTAATTTTAAAGTTAC
hCoV-19/England/PLYM-332B917/2022_EPI_ISL_9062229 ...	(1679)	GGAAACTGTGAAAGGTTTGGATTATAAAGCATTCAAACAAATTGTTGAATCCTGTGGTAATTTTAAAGTTAC
hCoV-19/Germany/HE-RKI-I-438397/2022_EPI_ISL_905522...	(1693)	GGAAACTGTGAAAGGTTTGGATTATAAAGCATTCAAACAAATTGTTGAATCCTGTGGTAATTTTAAAGTTAC
hCoV-19/USA/DE-CDC-LC0472738/2021_EPI_ISL_9049423...	(1657)	GGAAACTGTGAAAGGTTTGGATTATAAAGCATTCAAACAAATTGTTGAATCCTGTGGTAATTTTAAAGTTAC

Section 26

	(1801)	1801 1810 1820 1830 1840 1850 1860 1872
SARS-CoV-2 Reference Genome NC_045512.2	(1801)	AAAAGGAAAAGCTAAAAAAGGTGCCTGGAATATTGGTGAACAGAAATCAATACTGAGTCCTCTTTATGCATT
hCoV-19/Botswana/R69B55_BHP_916539/2021_EPI_ISL_90...	(1747)	AAAAGGAAAAGCTAAAAAAGGTGCCTGGAATATTGGTGAACAGAAATCAATACTGAGTCCTCTTTATGCATT
hCoV-19/India/HR-MDU-IGIB1210605800489930/2022_EPI...	(1789)	AAAAGGAAAAGCTAAAAAAGGTGCCTGGAATATTGGTGAACAGAAATCAATACTGAGTCCTCTTTATGCATT
hCoV-19/England/PLYM-332B917/2022_EPI_ISL_9062229 ...	(1751)	AAAAGGAAAAGCTAAAAAAGGTGCCTGGAATATTGGTGAACAGAAATCAATACTGAGTCCTCTTTATGCATT
hCoV-19/Germany/HE-RKI-I-438397/2022_EPI_ISL_905522...	(1765)	AAAAGGAAAAGCTAAAAAAGGTGCCTGGAATATTGGTGAACAGAAATCAATACTGAGTCCTCTTTATGCATT
hCoV-19/USA/DE-CDC-LC0472738/2021_EPI_ISL_9049423...	(1729)	AAAAGGAAAAGCTAAAAAAGGTGCCTGGAATATTGGTGAACAGAAATCAATACTGAGTCCTCTTTATGCATT

Section 27

	(1873)	1873 1880 1890 1900 1910 1920 1930 1944
SARS-CoV-2 Reference Genome NC_045512.2	(1873)	TGCATCAGAGGCTGCTCGTGTTGTACGATCAATTTTCTCCCGCACTCTTGAAACTGCTCAAAATTCTGTGCG
hCoV-19/Botswana/R69B55_BHP_916539/2021_EPI_ISL_90...	(1819)	TGCATCAGAGGCTGCTCGTGTTGTACGATCAATTTTCTCCCGCACTCTTGAAACTGCTCAAAATTCTGTGCG
hCoV-19/India/HR-MDU-IGIB1210605800489930/2022_EPI...	(1861)	TGCATCAGAGGCTGCTCGTGTTGTACGATCAATTTTCTCCCGCACTCTTGAAACTGCTCAAAATTCTGTGCG
hCoV-19/England/PLYM-332B917/2022_EPI_ISL_9062229 ...	(1823)	TGCATCAGAGGCTGCTCGTGTTGTACGATCAATTTTCTCCCGCACTCTTGAAACTGCTCAAAATTCTGTGCG
hCoV-19/Germany/HE-RKI-I-438397/2022_EPI_ISL_905522...	(1837)	TGCATCAGAGGCTGCTCGTGTTGTACGATCAATTTTCTCCCGCACTCTTGAAACTGCTCAAAATTCTGTGCG
hCoV-19/USA/DE-CDC-LC0472738/2021_EPI_ISL_9049423...	(1801)	TGCATCAGAGGCTGCTCGTGTTGTACGATCAATTTTCTCCCGCACTCTTGAAACTGCTCAAAATTCTGTGCG

Section 28

	(1945)	1945 1950 1960 1970 1980 1990 2000 2016
SARS-CoV-2 Reference Genome NC_045512.2	(1945)	TGTTTTACAGAAGGCCGCTATAACAATACTAGATGGAATTTCACAGTATTCACTGAGACTCATTGATGCTAT
hCoV-19/Botswana/R69B55_BHP_916539/2021_EPI_ISL_90...	(1891)	TGTTTTACAGAAGGCCGCTATAACAATACTAGATGGAATTTCACAGTATTCACTGAGACTCATTGATGCTAT
hCoV-19/India/HR-MDU-IGIB1210605800489930/2022_EPI...	(1933)	TGTTTTACAGAAGGCCGCTATAACAATACTAGATGGAATTTCACAGTATTCACTGAGACTCATTGATGCTAT
hCoV-19/England/PLYM-332B917/2022_EPI_ISL_9062229 ...	(1895)	TGTTTTACAGAAGGCCGCTATAACAATACTAGATGGAATTTCACAGTATTCACTGAGACTCATTGATGCTAT
hCoV-19/Germany/HE-RKI-I-438397/2022_EPI_ISL_905522...	(1909)	TGTTTTACAGAAGGCCGCTATAACAATACTAGATGGAATTTCACAGTATTCACTGAGACTCATTGATGCTAT
hCoV-19/USA/DE-CDC-LC0472738/2021_EPI_ISL_9049423...	(1873)	TGTTTTACAGAAGGCCGCTATAACAATACTAGATGGAATTTCACAGTATTCACTGAGACTCATTGATGCTAT

Section 29

	(2017)	2017 2030 2040 2050 2060 2070 2088
SARS-CoV-2 Reference Genome NC_045512.2	(2017)	GATGTTCACATCTGATTTGGCTACTAACAATCTAGTTGTAATGGCCTACATTACAGGTGGTGTTGTTCAGTT
hCoV-19/Botswana/R69B55_BHP_916539/2021_EPI_ISL_90...	(1963)	GATGTTCACATCTGATTTGGCTACTAACAATCTAGTTGTAATGGCCTACATTACAGGTGGTGTTGTTCAGTT
hCoV-19/India/HR-MDU-IGIB1210605800489930/2022_EPI...	(2005)	GATGTTCACATCTGATTTGGCTACTAACAATCTAGTTGTAATGGCCTACATTACAGGTGGTGTTGTTCAGTT
hCoV-19/England/PLYM-332B917/2022_EPI_ISL_9062229_...	(1967)	GATGTTCACATCTGATTTGGCTACTAACAATCTAGTTGTAATGGCCTACATTACAGGTGGTGTTGTTCAGTT
hCoV-19/Germany/HE-RKI-I-438397/2022_EPI_ISL_905522...	(1981)	GATGTTCACATCTGATTTGGCTACTAACAATCTAGTTGTAATGGCCTACATTACAGGTGGTGTTGTTCAGTT
hCoV-19/USA/DE-CDC-LC0472738/2021_EPI_ISL_9049423...	(1945)	GATGTTCACATCTGATTTGGCTACTAACAATCTAGTTGTAATGGCCTACATTACAGGTGGTGTTGTTCAGTT

Section 30

	(2089)	2089 2100 2110 2120 2130 2140 2150 2160
SARS-CoV-2 Reference Genome NC_045512.2	(2089)	GACTTCGCAGTGGCTAACTAACATCTTTGGCACTGTTTATGAAAAACTCAAACCCGTCCTTGATTGGCTTGA
hCoV-19/Botswana/R69B55_BHP_916539/2021_EPI_ISL_90...	(2035)	GACTTCGCAGTGGCTAACTAACATCTTTGGCACTGTTTATGAAAAACTCAAACCCGTCCTTGATTGGCTTGA
hCoV-19/India/HR-MDU-IGIB1210605800489930/2022_EPI...	(2077)	GACTTCGCAGTGGCTAACTAACATCTTTGGCACTGTTTATGAAAAACTCAAACCCGTCCTTGATTGGCTTGA
hCoV-19/England/PLYM-332B917/2022_EPI_ISL_9062229_...	(2039)	GACTTCGCAGTGGCTAACTAACATCTTTGGCACTGTTTATGAAAAACTCAAACCCGTCCTTGATTGGCTTGA
hCoV-19/Germany/HE-RKI-I-438397/2022_EPI_ISL_905522...	(2053)	GACTTCGCAGTGGCTAACTAACATCTTTGGCACTGTTTATGAAAAACTCAAACCCGTCCTTGATTGGCTTGA
hCoV-19/USA/DE-CDC-LC0472738/2021_EPI_ISL_9049423...	(2017)	GACTTCGCAGTGGCTAACTAACATCTTTGGCACTGTTTATGAAAAACTCAAACCCGTCCTTGATTGGCTTGA

Section 31

	(2161)	2161 2170 2180 2190 2200 2210 2220 2232
SARS-CoV-2 Reference Genome NC_045512.2	(2161)	AGAGAAGTTTAAGGAAGGTGTAGAGTTTCTTAGAGACGGTTGGGAAATTGTTAAATTTATCTCAACCTGTGC
hCoV-19/Botswana/R69B55_BHP_916539/2021_EPI_ISL_90...	(2107)	AGAGAAGTTTAAGGAAGGTGTAGAGTTTCTTAGAGACGGTTGGGAAATTGTTAAATTTATCTCAACCTGTGC
hCoV-19/India/HR-MDU-IGIB1210605800489930/2022_EPI...	(2149)	AGAGAAGTTTAAGGAAGGTGTAGAGTTTCTTAGAGACGGTTGGGAAATTGTTAAATTTATCTCAACCTGTGC
hCoV-19/England/PLYM-332B917/2022_EPI_ISL_9062229_...	(2111)	AGAGAAGTTTAAGGAAGGTGTAGAGTTTCTTAGAGACGGTTGGGAAATTGTTAAATTTATCTCAACCTGTGC
hCoV-19/Germany/HE-RKI-I-438397/2022_EPI_ISL_905522...	(2125)	AGAGAAGTTTAAGGAAGGTGTAGAGTTTCTTAGAGACGGTTGGGAAATTGTTAAATTTATCTCAACCTGTGC
hCoV-19/USA/DE-CDC-LC0472738/2021_EPI_ISL_9049423...	(2089)	AGAGAAGTTTAAGGAAGGTGTAGAGTTTCTTAGAGACGGTTGGGAAATTGTTAAATTTATCTCAACCTGTGC

Section 32

	(2233)	2233 2240 2250 2260 2270 2280 2290 2304
SARS-CoV-2 Reference Genome NC_045512.2	(2233)	TTGTGAAATTGTCGGTGGACAAATTGTCACCTGTGCAAAGGAAATTAAGGAGAGTGTTCAGACATTCTTTAA
hCoV-19/Botswana/R69B55_BHP_916539/2021_EPI_ISL_90...	(2179)	TTGTGAAATTGTCGGTGGACAAATTGTCACCTGTGCAAAGGAAATTAAGGAGAGTGTTCAGACATTCTTTAA
hCoV-19/India/HR-MDU-IGIB1210605800489930/2022_EPI...	(2221)	TTGTGAAATTGTCGGTGGACAAATTGTCACCTGTGCAAAGGAAATTAAGGAGAGTGTTCAGACATTCTTTAA
hCoV-19/England/PLYM-332B917/2022_EPI_ISL_9062229_...	(2183)	TTGTGAAATTGTCGGTGGACAAATTGTCACCTGTGCAAAGGAAATTAAGGAGAGTGTTCAGACATTCTTTAA
hCoV-19/Germany/HE-RKI-I-438397/2022_EPI_ISL_905522...	(2197)	TTGTGAAATTGTCGGTGGACAAATTGTCACCTGTGCAAAGGAAATTAAGGAGAGTGTTCAGACATTCTTTAA
hCoV-19/USA/DE-CDC-LC0472738/2021_EPI_ISL_9049423...	(2161)	TTGTGAAATTGTCGGTGGACAAATTGTCACCTGTGCAAAGGAAATTAAGGAGAGTGTTCAGACATTCTTTAA

Section 33

	(2305)	2305 2310 2320 2330 2340 2350 2360 2376
SARS-CoV-2 Reference Genome NC_045512.2	(2305)	GCTTGTAAATAAATTTTTGGCTTTGTGTGCTGACTCTATCATTATTGGTGGAGCTAAACTTAAAGCCTTGAA
hCoV-19/Botswana/R69B55_BHP_916539/2021_EPI_ISL_90...	(2251)	GCTTGTAAATAAATTTTTGGCTTTGTGTGCTGACTCTATCATTATTGGTGGAGCTAAACTTAAAGCCTTGAA
hCoV-19/India/HR-MDU-IGIB1210605800489930/2022_EPI...	(2293)	GCTTGTAAATAAATTTTTGGCTTTGTGTGCTGACTCTATCATTATTGGTGGAGCTAAACTTAAAGCCTTGAA
hCoV-19/England/PLYM-332B917/2022_EPI_ISL_9062229_...	(2255)	GCTTGTAAATAAATTTTTGGCTTTGTGTGCTGACTCTATCATTATTGGTGGAGCTAAACTTAAAGCCTTGAA
hCoV-19/Germany/HE-RKI-I-438397/2022_EPI_ISL_905522...	(2269)	GCTTGTAAATAAATTTTTGGCTTTGTGTGCTGACTCTATCATTATTGGTGGAGCTAAACTTAAAGCCTTGAA
hCoV-19/USA/DE-CDC-LC0472738/2021_EPI_ISL_9049423...	(2233)	GCTTGTAAATAAATTTTTGGCTTTGTGTGCTGACTCTATCATTATTGGTGGAGCTAAACTTAAAGCCTTGAA

Section 34

	(2377)	2377 2390 2400 2410 2420 2430 2448
SARS-CoV-2 Reference Genome NC_045512.2	(2377)	TTTAGGTGAAACATTTGTCACGCACTCAAAGGGATTGTACAGAAAGTGTGTTAAATCCAGAGAAGAAACTGG
hCoV-19/Botswana/R69B55_BHP_916539/2021_EPI_ISL_90...	(2323)	TTTAGGTGAAACATTTGTCACGCACTCAAAGGGATTGTACAGAAAGTGTGTTAAATCCAGAGAAGAAACTGG
hCoV-19/India/HR-MDU-IGIB1210605800489930/2022_EPI...	(2365)	TTTAGGTGAAACATTTGTCACGCACTCAAAGGGATTGTACAGAAAGTGTGTTAAATCCAGAGAAGAAACTGG
hCoV-19/England/PLYM-332B917/2022_EPI_ISL_9062229_...	(2327)	TTTAGGTGAAACATTTGTCACGCACTCAAAGGGATTGTACAGAAAGTGTGTTAAATCCAGAGAAGAAACTGG
hCoV-19/Germany/HE-RKI-I-438397/2022_EPI_ISL_905522...	(2341)	TTTAGGTGAAACATTTGTCACGCACTCAAAGGGATTGTACAGAAAGTGTGTTAAATCCAGAGAAGAAACTGG
hCoV-19/USA/DE-CDC-LC0472738/2021_EPI_ISL_9049423...	(2305)	TTTAGGTGAAACATTTGTCACGCACTCAAAGGGATTGTACAGAAAGTGTGTTAAATCCAGAGAAGAAACTGG

Section 35

	(2449)	2449 2460 2470 2480 2490 2500 2510 2520
SARS-CoV-2 Reference Genome NC_045512.2	(2449)	CCTACTCATGCCTCTAAAAGCCCCAAAAGAAATTATCTTCTTAGAGGGAGAAACACTTCCCACAGAAGTGTT
hCoV-19/Botswana/R69B55_BHP_916539/2021_EPI_ISL_90...	(2395)	CCTACTCATGCCTCTAAAAGCCCCAAAAGAAATTATCTTCTTAGAGGGAGAAACACTTCCCACAGAAGTGTT
hCoV-19/India/HR-MDU-IGIB1210605800489930/2022_EPI...	(2437)	CCTACTCATGCCTCTAAAAGCCCCAAAAGAAATTATCTTCTTAGAGGGAGAAACACTTCCCACAGAAGTGTT
hCoV-19/England/PLYM-332B917/2022_EPI_ISL_9062229_...	(2399)	CCTACTCATGCCTCTAAAAGCCCCAAAAGAAATTATCTTCTTAGAGGGAGAAACACTTCCCACAGAAGTGTT
hCoV-19/Germany/HE-RKI-I-438397/2022_EPI_ISL_905522...	(2413)	CCTACTCATGCCTCTAAAAGCCCCAAAAGAAATTATCTTCTTAGAGGGAGAAACACTTCCCACAGAAGTGTT
hCoV-19/USA/DE-CDC-LC0472738/2021_EPI_ISL_9049423...	(2377)	CCTACTCATGCCTCTAAAAGCCCCAAAAGAAATTATCTTCTTAGAGGGAGAAACACTTCCCACAGAAGTGTT

Section 36

	(2521)	2521 2530 2540 2550 2560 2570 2580 2592
SARS-CoV-2 Reference Genome NC_045512.2	(2521)	AACAGAGGAAGTTGTCTTGAAAACTGGTGATTTACAACCATTAGAACAACCTACTAGTGAAGCTGTTGAAGC
hCoV-19/Botswana/R69B55_BHP_916539/2021_EPI_ISL_90...	(2467)	AACAGAGGAAGTTGTCTTGAAAACTGGTGATTTACAACCATTAGAACAACCTACTAGTGAAGCTGTTGAAGC
hCoV-19/India/HR-MDU-IGIB1210605800489930/2022_EPI...	(2509)	AACAGAGGAAGTTGTCTTGAAAACTGGTGATTTACAACCATTAGAACAACCTACTAGTGAAGCTGTTGAAGC
hCoV-19/England/PLYM-332B917/2022_EPI_ISL_9062229_...	(2471)	AACAGAGGAAGTTGTCTTGAAAACTGGTGATTTACAACCATTAGAACAACCTACTAGTGAAGCTGTTGAAGC
hCoV-19/Germany/HE-RKI-I-438397/2022_EPI_ISL_905522...	(2485)	AACAGAGGAAGTTGTCTTGAAAACTGGTGATTTACAACCATTAGAACAACCTACTAGTGAAGCTGTTGAAGC
hCoV-19/USA/DE-CDC-LC0472738/2021_EPI_ISL_9049423...	(2449)	AACAGAGGAAGTTGTCTTGAAAACTGGTGATTTACAACCATTAGAACAACCTACTAGTGAAGCTGTTGAAGC

Section 37

		2593 … 2664
	(2593)	
SARS-CoV-2 Reference Genome NC_045512.2	(2593)	TCCATTGGTTGGTACACCAGTTTGTATTAACGGGCTTATGTTGCTCGAAATCAAAGACACAGAAAAGTACTG
hCoV-19/Botswana/R69B55_BHP_916539/2021_EPI_ISL_90...	(2539)	TCCATTGGTTGGTACACCAGTTTGTATTAACGGGCTTATGTTGCTCGAAATCAAAGACACAGAAAAGTACTG
hCoV-19/India/HR-MDU-IGIB1210605800489930/2022_EPI...	(2581)	TCCATTGGTTGGTACACCAGTTTGTATTAACGGGCTTATGTTGCTCGAAATCAAAGACACAGAAAAGTACTG
hCoV-19/England/PLYM-332B917/2022_EPI_ISL_9062229_...	(2543)	TCCATTGGTTGGTACACCAGTTTGTATTAACGGGCTTATGTTGCTCGAAATCAAAGACACAGAAAAGTACTG
hCoV-19/Germany/HE-RKI-I-438397/2022_EPI_ISL_905522...	(2557)	TCCATTGGTTGGTACACCAGTTTGTATTAACGGGCTTATGTTGCTCGAAATCAAAGACACAGAAAAGTACTG
hCoV-19/USA/DE-CDC-LC0472738/2021_EPI_ISL_9049423...	(2521)	TCCATTGGTTGGTACACCAGTTTGTATTAACGGGCTTATGTTGCTCGAAATCAAAGACACAGAAAAGTACTG

Section 38

		2665 … 2736
	(2665)	
SARS-CoV-2 Reference Genome NC_045512.2	(2665)	TGCCCTTGCACCTAATATGATGGTAACAAACAATACCTTCACACTCAAAGGCGGTGCACCAACAAAGGTTAC
hCoV-19/Botswana/R69B55_BHP_916539/2021_EPI_ISL_90...	(2611)	TGCCCTTGCACCTAATATGATGGTAACAAACAATACCTTCACACTCAAAGGCGGTGCACCAACAAAGGTTAC
hCoV-19/India/HR-MDU-IGIB1210605800489930/2022_EPI...	(2653)	TGCCCTTGCACCTAATATGATGGTAACAAACAATACCTTCACACTCAAAGGCGGTGCACCAACAAAGGTTAC
hCoV-19/England/PLYM-332B917/2022_EPI_ISL_9062229_...	(2615)	TGCCCTTGCACCTAATATGATGGTAACAAACAATACCTTCACACTCAAAGGCGGTGCACCAACAAAGGTTAC
hCoV-19/Germany/HE-RKI-I-438397/2022_EPI_ISL_905522...	(2629)	TGCCCTTGCACCTAATATGATGGTAACAAACAATACCTTCACACTCAAAGGCGGTGCACCAACAAAGGTTAC
hCoV-19/USA/DE-CDC-LC0472738/2021_EPI_ISL_9049423...	(2593)	TGCCCTTGCACCTAATATGATGGTAACAAACAATACCTTCACACTCAAAGGCGGTGCACCAACAAAGGTTAC

Section 39

		2737 … 2808
	(2737)	
SARS-CoV-2 Reference Genome NC_045512.2	(2737)	TTTTGGTGATGACACTGTGATAGAAGTGCAAGGTTACAAGAGTGTGAATATCACTTTTGAACTTGATGAAAG
hCoV-19/Botswana/R69B55_BHP_916539/2021_EPI_ISL_90...	(2683)	TTTTGGTGATGACACTGTGATAGAAGTGCAAGGTTACAAGAGTGTGAATATCACTTTTGAACTTGATGAAAG
hCoV-19/India/HR-MDU-IGIB1210605800489930/2022_EPI...	(2725)	TTTTGGTGATGACACTGTGATAGAAGTGCAAGGTTACAAGAGTGTGAATATCACTTTTGAACTTGATGAAAG
hCoV-19/England/PLYM-332B917/2022_EPI_ISL_9062229_...	(2687)	TTTTGGTGATGACACTGTGATAGAAGTGCAAGGTTACAAGAGTGTGAATATCACTTTTGAACTTGATGAAAG
hCoV-19/Germany/HE-RKI-I-438397/2022_EPI_ISL_905522...	(2701)	TTTTGGTGATGACACTGTGATAGAAGTGCAAGGTTACAAGAGTGTGAATATCACTTTTGAACTTGATGAAAG
hCoV-19/USA/DE-CDC-LC0472738/2021_EPI_ISL_9049423...	(2665)	TTTTGGTGATGACACTGTGATAGAAGTGCAAGGTTACAAGAGTGTGAATATCACTTTTGAACTTGATGAAAG

Section 40

		2809 … 2880
	(2809)	
SARS-CoV-2 Reference Genome NC_045512.2	(2809)	GATTGATAAAGTACTTAATGAGAAGTGCTCTGCCTATACAGTTGAACTCGGTACAGAAGTAAATGAGTTCGC
hCoV-19/Botswana/R69B55_BHP_916539/2021_EPI_ISL_90...	(2755)	GATTGATAAAGTACTTAATGAGAGGTGCTCTGCCTATACAGTTGAACTCGGTACAGAAGTAAATGAGTTCGC
hCoV-19/India/HR-MDU-IGIB1210605800489930/2022_EPI...	(2797)	GATTGATAAAGTACTTAATGAGAGGTGCTCTGCCTATACAGTTGAACTCGGTACAGAAGTAAATGAGTTCGC
hCoV-19/England/PLYM-332B917/2022_EPI_ISL_9062229_...	(2759)	GATTGATAAAGTACTTAATGAGAGGTGCTCTGCCTATACAGTTGAACTCGGTACAGAAGTAAATGAGTTCGC
hCoV-19/Germany/HE-RKI-I-438397/2022_EPI_ISL_905522...	(2773)	GATTGATAAAGTACTTAATGAGAGGTGCTCTGCCTATACAGTTGAACTCGGTACAGAAGTAAATGAGTTCGC
hCoV-19/USA/DE-CDC-LC0472738/2021_EPI_ISL_9049423...	(2737)	GATTGATAAAGTACTTAATGAGAGGTGCTCTGCCTATACAGTTGAACTCGGTACAGAAGTAAATGAGTTCGC

Section 41

```
                                                                (2881) 2881    2890      2900      2910      2920      2930      2940        2952
          SARS-CoV-2 Reference Genome NC_045512.2               (2881) CTGTGTTGTGGCAGATGCTGTCATAAAAACTTTGCAACCAGTATCTGAATTACTTACACCACTGGGCATTGA
hCoV-19/Botswana/R69B55_BHP_916539/2021_EPI_ISL_90...           (2827) CTGTGTTGTGGCAGATGCTGTCATAAAAACTTTGCAACCAGTATCTGAATTACTTACACCACTGGGCATTGA
 hCoV-19/India/HR-MDU-IGIB1210605800489930/2022_EPI...          (2869) CTGTGTTGTGGCAGATGCTGTCATAAAAACTTTGCAACCAGTATCTGAATTACTTACACCACTGGGCATTGA
 hCoV-19/England/PLYM-332B917/2022_EPI_ISL_9062229 ...          (2831) CTGTGTTGTGGCAGATGCTGTCATAAAAACTTTGCAACCAGTATCTGAATTACTTACACCACTGGGCATTGA
hCoV-19/Germany/HE-RKI-I-438397/2022_EPI_ISL_905522...          (2845) CTGTGTTGTGGCAGATGCTGTCATAAAAACTTTGCAACCAGTATCTGAATTACTTACACCACTGGGCATTGA
 hCoV-19/USA/DE-CDC-LC0472738/2021_EPI_ISL_9049423...           (2809) CTGTGTTGTGGCAGATGCTGTCATAAAAACTTTGCAACCAGTATCTGAATTACTTACACCACTGGGCATTGA
```

Section 42

```
                                                                (2953) 2953   2960      2970      2980      2990      3000      3010          3024
          SARS-CoV-2 Reference Genome NC_045512.2               (2953) TTTAGATGAGTGGAGTATGGCTACATACTACTTATTTGATGAGTCTGGTGAGTTTAAATTGGCTTCACATAT
hCoV-19/Botswana/R69B55_BHP_916539/2021_EPI_ISL_90...           (2899) TTTAGATGAGTGGAGTATGGCTACATACTACTTATTTGATGAGTCTGGTGAGTTTAAATTGGCTTCACATAT
 hCoV-19/India/HR-MDU-IGIB1210605800489930/2022_EPI...          (2941) TTTAGATGAGTGGAGTATGGCTACATACTACTTATTTGATGAGTCTGGTGAGTTTAAATTGGCTTCACATAT
 hCoV-19/England/PLYM-332B917/2022_EPI_ISL_9062229 ...          (2903) TTTAGATGAGTGGAGTATGGCTACATACTACTTATTTGATGAGTCTGGTGAGTTTAAATTGGCTTCACATAT
hCoV-19/Germany/HE-RKI-I-438397/2022_EPI_ISL_905522...          (2917) TTTAGATGAGTGGAGTATGGCTACATACTACTTATTTGATGAGTCTGGTGAGTTTAAATTGGCTTCACATAT
 hCoV-19/USA/DE-CDC-LC0472738/2021_EPI_ISL_9049423...           (2881) TTTAGATGAGTGGAGTATGGCTACATACTACTTATTTGATGAGTCTGGTGAGTTTAAATTGGCTTCACATAT
```

Section 43

```
                                                                (3025) 3025 3030      3040      3050      3060      3070      3080            3096
          SARS-CoV-2 Reference Genome NC_045512.2               (3025) GTATTGTTCTTTCTACCCTCCAGATGAGGATGAAGAAGAAGGTGATTGTGAAGAAGAAGAGTTTGAGCCATC
hCoV-19/Botswana/R69B55_BHP_916539/2021_EPI_ISL_90...           (2971) GTATTGTTCTTTTTACCCTCCAGATGAGGATGAAGAAGAAGGTGATTGTGAAGAAGAAGAGTTTGAGCCATC
 hCoV-19/India/HR-MDU-IGIB1210605800489930/2022_EPI...          (3013) GTATTGTTCTTTTTACCCTCCAGATGAGGATGAAGAAGAAGGTGATTGTGAAGAAGAAGAGTTTGAGCCATC
 hCoV-19/England/PLYM-332B917/2022_EPI_ISL_9062229 ...          (2975) GTATTGTTCTTTTTACCCTCCAGATGAGGATGAAGAAGAAGGTGATTGTGAAGAAGAAGAGTTTGAGCCATC
hCoV-19/Germany/HE-RKI-I-438397/2022_EPI_ISL_905522...          (2989) GTATTGTTCTTTTTACCCTCCAGATGAGGATGAAGAAGAAGGTGATTGTGAAGAAGAAGAGTTTGAGCCATC
 hCoV-19/USA/DE-CDC-LC0472738/2021_EPI_ISL_9049423...           (2953) GTATTGTTCTTTTTACCCTCCAGATGAGGATGAAGAAGAAGGTGATTGTGAAGAAGAAGAGTTTGAGCCATC
```

Section 44

```
                                                                (3097) 3097         3110      3120      3130      3140      3150              3168
          SARS-CoV-2 Reference Genome NC_045512.2               (3097) AACTCAATATGAGTATGGTACTGAAGATGATTACCAAGGTAAACCTTTGGAATTTGGTGCCACTTCTGCTGC
hCoV-19/Botswana/R69B55_BHP_916539/2021_EPI_ISL_90...           (3043) AACTCAATATGAGTATGGTACTGAAGATGATTACCAAGGTAAACCTTTGGAATTTGGTGCCACTTCTGCTGC
 hCoV-19/India/HR-MDU-IGIB1210605800489930/2022_EPI...          (3085) AACTCAATATGAGTATGGTACTGAAGATGATTACCAAGGTAAACCTTTGGAATTTGGTGCCACTTCTGCTGC
 hCoV-19/England/PLYM-332B917/2022_EPI_ISL_9062229 ...          (3047) AACTCAATATGAGTATGGTACTGAAGATGATTACCAAGGTAAACCTTTGGAATTTGGTGCCACTTCTGCTGC
hCoV-19/Germany/HE-RKI-I-438397/2022_EPI_ISL_905522...          (3061) AACTCAATATGAGTATGGTACTGAAGATGATTACCAAGGTAAACCTTTGGAATTTGGTGCCACTTCTGCTGC
 hCoV-19/USA/DE-CDC-LC0472738/2021_EPI_ISL_9049423...           (3025) AACTCAATATGAGTATGGTACTGAAGATGATTACCAAGGTAAACCTTTGGAATTTGGTGCCACTTCTGCTGC
```

Section 45

```
                                                   (3169) 3169       3180           3190           3200           3210           3220           3230      3240
                SARS-CoV-2 Reference Genome NC 045512.2 (3169) TCTTCAACCTGAAGAAGAGCAAGAAGAAGATTGGTTAGATGATGATAGTCAACAAACTGTTGGTCAACAAGA
hCoV-19/Botswana/R69B55 BHP 916539/2021 EPI ISL 90... (3115) TCTTCAACCTGAAGAAGAGCAAGAAGAAGATTGGTTAGATGATGATAGTCAACAAACTGTTGGTCAACAAGA
 hCoV-19/India/HR-MDU-IGIB1210605800489930/2022 EPI... (3157) TCTTCAACCTGAAGAAGAGCAAGAAGAAGATTGGTTAGATGATGATAGTCAACAAACTGTTGGTCAACAAGA
 hCoV-19/England/PLYM-332B917/2022 EPI ISL 9062229 ... (3119) TCTTCAACCTGAAGAAGAGCAAGAAGAAGATTGGTTAGATGATGATAGTCAACAAACTGTTGGTCAACAAGA
hCoV-19/Germany/HE-RKI-I-438397/2022 EPI ISL 905522... (3133) TCTTCAACCTGAAGAAGAGCAAGAAGAAGATTGGTTAGATGATGATAGTCAACAAACTGTTGGTCAACAAGA
 hCoV-19/USA/DE-CDC-LC0472738/2021_EPI_ISL_9049423... (3097) TCTTCAACCTGAAGAAGAGCAAGAAGAAGATTGGTTAGATGATGATAGTCAACAAACTGTTGGTCAACAAGA
```

Section 46

```
                                                   (3241) 3241     3250           3260           3270           3280           3290           3300      3312
                SARS-CoV-2 Reference Genome NC 045512.2 (3241) CGGCAGTGAGGACAATCAGACAACTACTATTCAAACAATTGTTGAGGTTCAACCTCAATTAGAGATGGAACT
hCoV-19/Botswana/R69B55 BHP 916539/2021 EPI ISL 90... (3187) CGGCAGTGAGGACAATCAGACAACTACTATTCAAACAATTGTTGAGGTTCAACCTCAATTAGAGATGGAACT
 hCoV-19/India/HR-MDU-IGIB1210605800489930/2022 EPI... (3229) CGGCAGTGAGGACAATCAGACAACTACTATTCAAACAATTGTTGAGGTTCAACCTCAATTAGAGATGGAACT
 hCoV-19/England/PLYM-332B917/2022 EPI ISL 9062229 ... (3191) CGGCAGTGAGGACAATCAGACAACTACTATTCAAACAATTGTTGAGGTTCAACCTCAATTAGAGATGGAACT
hCoV-19/Germany/HE-RKI-I-438397/2022 EPI ISL 905522... (3205) CGGCAGTGAGGACAATCAGACAACTACTATTCAAACAATTGTTGAGGTTCAACCTCAATTAGAGATGGAACT
 hCoV-19/USA/DE-CDC-LC0472738/2021_EPI_ISL_9049423... (3169) CGGCAGTGAGGACAATCAGACAACTACTATTCAAACAATTGTTGAGGTTCAACCTCAATTAGAGATGGAACT
```

Section 47

```
                                                   (3313) 3313   3320           3330           3340           3350           3360           3370         3384
                SARS-CoV-2 Reference Genome NC 045512.2 (3313) TACACCAGTTGTTCAGACTATTGAAGTGAATAGTTTTAGTGGTTATTTAAAACTTACTGACAATGTATACAT
hCoV-19/Botswana/R69B55 BHP 916539/2021 EPI ISL 90... (3259) TACACCAGTTGTTCAGACTATTGAAGTGAATAGTTTTAGTGGTTATTTAAAACTTACTGACAATGTATACAT
 hCoV-19/India/HR-MDU-IGIB1210605800489930/2022 EPI... (3301) TACACCAGTTGTTCAGACTATTGAAGTGAATAGTTTTAGTGGTTATTTAAAACTTACTGACAATGTATACAT
 hCoV-19/England/PLYM-332B917/2022 EPI ISL 9062229 ... (3263) TACACCAGTTGTTCAGACTATTGAAGTGAATAGTTTTAGTGGTTATTTAAAACTTACTGACAATGTATACAT
hCoV-19/Germany/HE-RKI-I-438397/2022 EPI ISL 905522... (3277) TACACCAGTTGTTCAGACTATTGAAGTGAATAGTTTTAGTGGTTATTTAAAACTTACTGACAATGTATACAT
 hCoV-19/USA/DE-CDC-LC0472738/2021_EPI_ISL_9049423... (3241) TACACCAGTTGTTCAGACTATTGAAGTGAATAGTTTTAGTGGTTATTTAAAACTTACTGACAATGTATACAT
```

Section 48

```
                                                   (3385) 3385 3390           3400           3410           3420           3430           3440             3456
                SARS-CoV-2 Reference Genome NC 045512.2 (3385) TAAAAATGCAGACATTGTGGAAGAAGCTAAAAAGGTAAAACCAACAGTGGTTGTTAATGCAGCCAATGTTTA
hCoV-19/Botswana/R69B55 BHP 916539/2021 EPI ISL 90... (3331) TAAAAATGCAGACATTGTGGAAGAAGCTAAAAAGGTAAAACCAACAGTGGTTGTTAATGCAGCCAATGTTTA
 hCoV-19/India/HR-MDU-IGIB1210605800489930/2022 EPI... (3373) TAAAAATGCAGACATTGTGGAAGAAGCTAAAAAGGTAAAACCAACAGTGGTTGTTAATGCAGCCAATGTTTA
 hCoV-19/England/PLYM-332B917/2022 EPI ISL 9062229 ... (3335) TAAAAATGCAGACATTGTGGAAGAAGCTAAAAAGGTAAAACCAACAGTGGTTGTTAATGCAGCCAATGTTTA
hCoV-19/Germany/HE-RKI-I-438397/2022 EPI ISL 905522... (3349) TAAAAATGCAGACATTGTGGAAGAAGCTAAAAAGGTAAAACCAACAGTGGTTGTTAATGCAGCCAATGTTTA
 hCoV-19/USA/DE-CDC-LC0472738/2021_EPI_ISL_9049423... (3313) TAAAAATGCAGACATTGTGGAAGAAGCTAAAAAGGTAAAACCAACAGTGGTTGTTAATGCAGCCAATGTTTA
```

Section 49

	(3457)	3457 3470 3480 3490 3500 3510 3528
SARS-CoV-2 Reference Genome NC_045512.2	(3457)	CCTTAAACATGGAGGAGGTGTTGCAGGAGCCTTAAATAAGGCTACTAACAATGCCATGCAAGTTGAATCTGA
hCoV-19/Botswana/R69B55_BHP_916539/2021_EPI_ISL_90...	(3403)	CCTTAAACATGGAGGAGGTGTTGCAGGAGCCTTAAATAAGGCTACTAACAATGCCATGCAAGTTGAATCTGA
hCoV-19/India/HR-MDU-IGIB1210605800489930/2022_EPI...	(3445)	CCTTAAACATGGAGGAGGTGTTGCAGGAGCCTTAAATAAGGCTACTAACAATGCCATGCAAGTTGAATCTGA
hCoV-19/England/PLYM-332B917/2022_EPI_ISL_9062229_...	(3407)	CCTTAAACATGGAGGAGGTGTTGCAGGAGCCTTAAATAAGGCTACTAACAATGCCATGCAAGTTGAATCTGA
hCoV-19/Germany/HE-RKI-I-438397/2022_EPI_ISL_905522...	(3421)	CCTTAAACATGGAGGAGGTGTTGCAGGAGCCTTAAATAAGGCTACTAACAATGCCATGCAAGTTGAATCTGA
hCoV-19/USA/DE-CDC-LC0472738/2021_EPI_ISL_9049423...	(3385)	CCTTAAACATGGAGGAGGTGTTGCAGGAGCCTTAAATAAGGCTACTAACAATGCCATGCAAGTTGAATCTGA

Section 50

	(3529)	3529 3540 3550 3560 3570 3580 3590 3600
SARS-CoV-2 Reference Genome NC_045512.2	(3529)	TGATTACATAGCTACTAATGGACCACTTAAAGTGGGTGGTAGTTGTGTTTTAAGCGGACACAATCTTGCTAA
hCoV-19/Botswana/R69B55_BHP_916539/2021_EPI_ISL_90...	(3475)	TGATTACATAGCTACTAATGGACCACTTAAAGTGGGTGGTAGTTGTGTTTTAAGCGGACACAATCTTGCTAA
hCoV-19/India/HR-MDU-IGIB1210605800489930/2022_EPI...	(3517)	TGATTACATAGCTACTAATGGACCACTTAAAGTGGGTGGTAGTTGTGTTTTAAGCGGACACAATCTTGCTAA
hCoV-19/England/PLYM-332B917/2022_EPI_ISL_9062229_...	(3479)	TGATTACATAGCTACTAATGGACCACTTAAAGTGGGTGGTAGTTGTGTTTTAAGCGGACACAATCTTGCTAA
hCoV-19/Germany/HE-RKI-I-438397/2022_EPI_ISL_905522...	(3493)	TGATTACATAGCTACTAATGGACCACTTAAAGTGGGTGGTAGTTGTGTTTTAAGCGGACACAATCTTGCTAA
hCoV-19/USA/DE-CDC-LC0472738/2021_EPI_ISL_9049423...	(3457)	TGATTACATAGCTACTAATGGACCACTTAAAGTGGGTGGTAGTTGTGTTTTAAGCGGACACAATCTTGCTAA

Section 51

	(3601)	3601 3610 3620 3630 3640 3650 3660 3672
SARS-CoV-2 Reference Genome NC_045512.2	(3601)	ACACTGTCTTCATGTTGTCGGCCCAAATGTTAACAAAGGTGAAGACATTCAACTTCTTAAGAGTGCTTATGA
hCoV-19/Botswana/R69B55_BHP_916539/2021_EPI_ISL_90...	(3547)	ACACTGTCTTCATGTTGTCGGCCCAAATGTTAACAAAGGTGAAGACATTCAACTTCTTAAGAGTGCTTATGA
hCoV-19/India/HR-MDU-IGIB1210605800489930/2022_EPI...	(3589)	ACACTGTCTTCATGTTGTCGGCCCAAATGTTAACAAAGGTGAAGACATTCAACTTCTTAAGAGTGCTTATGA
hCoV-19/England/PLYM-332B917/2022_EPI_ISL_9062229_...	(3551)	ACACTGTCTTCATGTTGTCGGCCCAAATGTTAACAAAGGTGAAGACATTCAACTTCTTAAGAGTGCTTATGA
hCoV-19/Germany/HE-RKI-I-438397/2022_EPI_ISL_905522...	(3565)	ACACTGTCTTCATGTTGTCGGCCCAAATGTTAACAAAGGTGAAGACATTCAACTTCTTAAGAGTGCTTATGA
hCoV-19/USA/DE-CDC-LC0472738/2021_EPI_ISL_9049423...	(3529)	ACACTGTCTTCATGTTGTCGGCCCAAATGTTAACAAAGGTGAAGACATTCAACTTCTTAAGAGTGCTTATGA

Section 52

	(3673)	3673 3680 3690 3700 3710 3720 3730 3744
SARS-CoV-2 Reference Genome NC_045512.2	(3673)	AAATTTTAATCAGCACGAAGTTCTACTTGCACCATTATTATCAGCTGGTATTTTTGGTGCTGACCCTATACA
hCoV-19/Botswana/R69B55_BHP_916539/2021_EPI_ISL_90...	(3619)	AAATTTTAATCAGCACGAAGTTCTACTTGCACCATTATTATCAGCTGGTATTTTTGGTGCTGACCCTATACA
hCoV-19/India/HR-MDU-IGIB1210605800489930/2022_EPI...	(3661)	AAATTTTAATCAGCACGAAGTTCTACTTGCACCATTATTATCAGCTGGTATTTTTGGTGCTGACCCTATACA
hCoV-19/England/PLYM-332B917/2022_EPI_ISL_9062229_...	(3623)	AAATTTTAATCAGCACGAAGTTCTACTTGCACCATTATTATCAGCTGGTATTTTTGGTGCTGACCCTATACA
hCoV-19/Germany/HE-RKI-I-438397/2022_EPI_ISL_905522...	(3637)	AAATTTTAATCAGCACGAAGTTCTACTTGCACCATTATTATCAGCTGGTATTTTTGGTGCTGACCCTATACA
hCoV-19/USA/DE-CDC-LC0472738/2021_EPI_ISL_9049423...	(3601)	AAATTTTAATCAGCACGAAGTTCTACTTGCACCATTATTATCAGCTGGTATTTTTGGTGCTGACCCTATACA

Section 53

		3745 3750 3760 3770 3780 3790 3800 3816
	(3745)	
SARS-CoV-2 Reference Genome NC_045512.2	(3745)	TTCTTTAAGAGTTTGTGTAGATACTGTTCGCACAAATGTCTACTTAGCTGTCTTTGATAAAAATCTCTATGA
hCoV-19/Botswana/R69B55_BHP_916539/2021_EPI_ISL_90...	(3691)	TTCTTTAAGAGTTTGTGTAGATACTGTTCGCACAAATGTCTACTTAGCTGTCTTTGATAAAAATCTCTATGA
hCoV-19/India/HR-MDU-IGIB1210605800489930/2022_EPI...	(3733)	TTCTTTAAGAGTTTGTGTAGATACTGTTCGCACAAATGTCTACTTAGCTGTCTTTGATAAAAATCTCTATGA
hCoV-19/England/PLYM-332B917/2022_EPI_ISL_9062229 ...	(3695)	TTCTTTAAGAGTTTGTGTAGATACTGTTCGCACAAATGTCTACTTAGCTGTCTTTGATAAAAATCTCTATGA
hCoV-19/Germany/HE-RKI-I-438397/2022_EPI_ISL_905522...	(3709)	TTCTTTAAGAGTTTGTGTAGATACTGTTCGCACAAATGTCTACTTAGCTGTCTTTGATAAAAATCTCTATGA
hCoV-19/USA/DE-CDC-LC0472738/2021_EPI_ISL_9049423...	(3673)	TTCTTTAAGAGTTTGTGTAGATACTGTTCGCACAAATGTCTACTTAGCTGTCTTTGATAAAAATCTCTATGA

Section 54

		3817 3830 3840 3850 3860 3870 3888
	(3817)	
SARS-CoV-2 Reference Genome NC_045512.2	(3817)	CAAACTTGTTTCAAGCTTTTTGGAAATGAAGAGTGAAAAGCAAGTTGAACAAAAGATCGCTGAGATTCCTAA
hCoV-19/Botswana/R69B55_BHP_916539/2021_EPI_ISL_90...	(3763)	CAAACTTGTTTCAAGCTTTTTGGAAATGAAGAGTGAAAAGCAAGTTGAACAAAAGATCGCTGAGATTCCTAA
hCoV-19/India/HR-MDU-IGIB1210605800489930/2022_EPI...	(3805)	CAAACTTGTTTCAAGCTTTTTGGAAATGAAGAGTGAAAAGCAAGTTGAACAAAAGATCGCTGAGATTCCTAA
hCoV-19/England/PLYM-332B917/2022_EPI_ISL_9062229 ...	(3767)	CAAACTTGTTTCAAGCTTTTTGGAAATGAAGAGTGAAAAGCAAGTTGAACAAAAGATCGCTGAGATTCCTAA
hCoV-19/Germany/HE-RKI-I-438397/2022_EPI_ISL_905522...	(3781)	CAAACTTGTTTCAAGCTTTTTGGAAATGAAGAGTGAAAAGCAAGTTGAACAAAAGATCGCTGAGATTCCTAA
hCoV-19/USA/DE-CDC-LC0472738/2021_EPI_ISL_9049423...	(3745)	CAAACTTGTTTCAAGCTTTTTGGAAATGAAGAGTGAAAAGCAAGTTGAACAAAAGATCGCTGAGATTCCTAA

Section 55

		3889 3900 3910 3920 3930 3940 3950 3960
	(3889)	
SARS-CoV-2 Reference Genome NC_045512.2	(3889)	AGAGGAAGTTAAGCCATTTATAACTGAAAGTAAACCTTCAGTTGAACAGAGAAAACAAGATGATAAGAAAAT
hCoV-19/Botswana/R69B55_BHP_916539/2021_EPI_ISL_90...	(3835)	AGAGGAAGTTAAGCCATTTATAACTGAAAGTAAACCTTCAGTTGAACAGAGAAAACAAGATGATAAGAAAAT
hCoV-19/India/HR-MDU-IGIB1210605800489930/2022_EPI...	(3877)	AGAGGAAGTTAAGCCATTTATAACTGAAAGTAAACCTTCAGTTGAACAGAGAAAACAAGATGATAAGAAAAT
hCoV-19/England/PLYM-332B917/2022_EPI_ISL_9062229 ...	(3839)	AGAGGAAGTTAAGCCATTTATAACTGAAAGTAAACCTTCAGTTGAACAGAGAAAACAAGATGATAAGAAAAT
hCoV-19/Germany/HE-RKI-I-438397/2022_EPI_ISL_905522...	(3853)	AGAGGAAGTTAAGCCATTTATAACTGAAAGTAAACCTTCAGTTGAACAGAGAAAACAAGATGATAAGAAAAT
hCoV-19/USA/DE-CDC-LC0472738/2021_EPI_ISL_9049423...	(3817)	AGAGGAAGTTAAGCCATTTATAACTGAAAGTAAACCTTCAGTTGAACAGAGAAAACAAGATGATAAGAAAAT

Section 56

		3961 3970 3980 3990 4000 4010 4020 4032
	(3961)	
SARS-CoV-2 Reference Genome NC_045512.2	(3961)	CAAAGCTTGTGTTGAAGAAGTTACAACAACTCTGGAAGAAACTAAGTTCCTCACAGAAAACTTGTTACTTTA
hCoV-19/Botswana/R69B55_BHP_916539/2021_EPI_ISL_90...	(3907)	CAAAGCTTGTGTTGAAGAAGTTACAACAACTCTGGAAGAAACTAAGTTCCTCACAGAAAACTTGTTACTTTA
hCoV-19/India/HR-MDU-IGIB1210605800489930/2022_EPI...	(3949)	CAAAGCTTGTGTTGAAGAAGTTACAACAACTCTGGAAGAAACTAAGTTCCTCACAGAAAACTTGTTACTTTA
hCoV-19/England/PLYM-332B917/2022_EPI_ISL_9062229 ...	(3911)	CAAAGCTTGTGTTGAAGAAGTTACAACAACTCTGGAAGAAACTAAGTTCCTCACAGAAAACTTGTTACTTTA
hCoV-19/Germany/HE-RKI-I-438397/2022_EPI_ISL_905522...	(3925)	CAAAGCTTGTGTTGAAGAAGTTACAACAACTCTGGAAGAAACTAAGTTCCTCACAGAAAACTTGTTACTTTA
hCoV-19/USA/DE-CDC-LC0472738/2021_EPI_ISL_9049423...	(3889)	CAAAGCTTGTGTTGAAGAAGTTACAACAACTCTGGAAGAAACTAAGTTCCTCACAGAAAACTTGTTACTTTA

Omicron BA.1

```
                                                                                       Section 57
                                                      (4033) 4033   4040      4050      4060      4070      4080      4090          4104
           SARS-CoV-2 Reference Genome NC_045512.2    (4033) TATTGACATTAATGGCAATCTTCATCCAGATTCTGCCACTCTTGTTAGTGACATTGACATCACTTTCTTAAA
hCoV-19/Botswana/R69B55_BHP_916539/2021_EPI_ISL_90... (3979) TATTGACATTAATGGCAATCTTCATCCAGATTCTGCCACTCTTGTTAGTGACATTGACATCACTTTCTTAAA
 hCoV-19/India/HR-MDU-IGIB1210605800489930/2022_EPI... (4021) TATTGACATTAATGGCAATCTTCATCCAGATTCTGCCACTCTTGTTAGTGACATTGACATCACTTTCTTAAA
 hCoV-19/England/PLYM-332B917/2022_EPI_ISL_9062229 ... (3983) TATTGACATTAATGGCAATCTTCATCCAGATTCTGCCACTCTTGTTAGTGACATTGACATCACTTTCTTAAA
hCoV-19/Germany/HE-RKI-I-438397/2022_EPI_ISL_905522... (3997) TATTGACATTAATGGCAATCTTCATCCAGATTCTGCCACTCTTGTTAGTGACATTGACATCACTTTCTTAAA
 hCoV-19/USA/DE-CDC-LC0472738/2021_EPI_ISL_9049423... (3961) TATTGACATTAATGGCAATCTTCATCCAGATTCTGCCACTCTTGTTAGTGACATTGACATCACTTTCTTAAA
                                                                                       Section 58
                                                      (4105) 4105 4110      4120      4130      4140      4150      4160          4176
           SARS-CoV-2 Reference Genome NC_045512.2    (4105) GAAAGATGCTCCATATATAGTGGGTGATGTTGTTCAAGAGGGTGTTTTAACTGCTGTGGTTATACCTACTAA
hCoV-19/Botswana/R69B55_BHP_916539/2021_EPI_ISL_90... (4051) GAAAGATGCTCCATATATAGTGGGTGATGTTGTTCAAGAGGGTGTTTTAACTGCTGTGGTTATACCTACTAA
 hCoV-19/India/HR-MDU-IGIB1210605800489930/2022_EPI... (4093) GAAAGATGCTCCATATATAGTGGGTGATGTTGTTCAAGAGGGTGTTTTAACTGCTGTGGTTATACCTACTAA
 hCoV-19/England/PLYM-332B917/2022_EPI_ISL_9062229 ... (4055) GAAAGATGCTCCATATATAGTGGGTGATGTTGTTCAAGAGGGTGTTTTAACTGCTGTGGTTATACCTACTAA
hCoV-19/Germany/HE-RKI-I-438397/2022_EPI_ISL_905522... (4069) GAAAGATGCTCCATATATAGTGGGTGATGTTGTTCAAGAGGGTGTTTTAACTGCTGTGGTTATACCTACTAA
 hCoV-19/USA/DE-CDC-LC0472738/2021_EPI_ISL_9049423... (4033) GAAAGATGCTCCATATATAGTGGGTGATGTTGTTCAAGAGGGTGTTTTAACTGCTGTGGTTATACCTACTAA
                                                                                       Section 59
                                                      (4177) 4177         4190      4200      4210      4220      4230              4248
           SARS-CoV-2 Reference Genome NC_045512.2    (4177) AAAGGCTGGTGGCACTACTGAAATGCTAGCGAAAGCTTTGAGAAAAGTGCCAACAGACAATTATATAACCAC
hCoV-19/Botswana/R69B55_BHP_916539/2021_EPI_ISL_90... (4123) AAAGGCTGGTGGCACTACTGAAATGCTAGCGAAAGCTTTGAGAAAAGTGCCAACAGACAATTATATAACCAC
 hCoV-19/India/HR-MDU-IGIB1210605800489930/2022_EPI... (4165) AAAGGCTGGTGGCACTACTGAAATGCTAGCGAAAGCTTTGAGAAAAGTGCCAACAGACAATTATATAACCAC
 hCoV-19/England/PLYM-332B917/2022_EPI_ISL_9062229 ... (4127) AAAGGCTGGTGGCACTACTGAAATGCTAGCGAAAGCTTTGAGAAAAGTGCCAACAGACAATTATATAACCAC
hCoV-19/Germany/HE-RKI-I-438397/2022_EPI_ISL_905522... (4141) AAAGGCTGGTGGCACTACTGAAATGCTAGCGAAAGCTTTGAGAAAAGTGCCAACAGACAATTATATAACCAC
 hCoV-19/USA/DE-CDC-LC0472738/2021_EPI_ISL_9049423... (4105) AAAGGCTGGTGGCACTACTGAAATGCTAGCGAAAGCTTTGAGAAAAGTGCCAACAGACAATTATATAACCAC
                                                                                       Section 60
                                                      (4249) 4249       4260      4270      4280      4290      4300      4310    4320
           SARS-CoV-2 Reference Genome NC_045512.2    (4249) TTACCCGGGTCAGGGTTTAAATGGTTACACTGTAGAGGAGGCAAAGACAGTGCTTAAAAAGTGTAAAAGTGC
hCoV-19/Botswana/R69B55_BHP_916539/2021_EPI_ISL_90... (4195) TTACCCGGGTCAGGGTTTAAATGGTTACACTGTAGAGGAGGCAAAGACAGTGCTTAAAAAGTGTAAAAGTGC
 hCoV-19/India/HR-MDU-IGIB1210605800489930/2022_EPI... (4237) TTACCCGGGTCAGGGTTTAAATGGTTACACTGTAGAGGAGGCAAAGACAGTGCTTAAAAAGTGTAAAAGTGC
 hCoV-19/England/PLYM-332B917/2022_EPI_ISL_9062229 ... (4199) TTACCCGGGTCAGGGTTTAAATGGTTACACTGTAGAGGAGGCAAAGACAGTGCTTAAAAAGTGTAAAAGTGC
hCoV-19/Germany/HE-RKI-I-438397/2022_EPI_ISL_905522... (4213) TTACCCGGGTCAGGGTTTAAATGGTTACACTGTAGAGGAGGCAAAGACAGTGCTTAAAAAGTGTAAAAGTGC
 hCoV-19/USA/DE-CDC-LC0472738/2021_EPI_ISL_9049423... (4177) TTACCCGGGTCAGGGTTTAAATGGTTACACTGTAGAGGAGGCAAAGACAGTGCTTAAAAAGTGTAAAAGTGC
```

Section 61

		4321 4330 4340 4350 4360 4370 4380 4392
	(4321)	
SARS-CoV-2 Reference Genome NC_045512.2	(4321)	CTTTTACATTCTACCATCTATTATCTCTAATGAGAAGCAAGAAATTCTTGGAACTGTTTCTTGGAATTTGCG
hCoV-19/Botswana/R69B55_BHP_916539/2021_EPI_ISL_90...	(4267)	CTTTTACATTCTACCATCTATTATCTCTAATGAGAAGCAAGAAATTCTTGGAACTGTTTCTTGGAATTTGCG
hCoV-19/India/HR-MDU-IGIB1210605800489930/2022_EPI...	(4309)	CTTTTACATTCTACCATCTATTATCTCTAATGAGAAGCAAGAAATTCTTGGAACTGTTTCTTGGAATTTGCG
hCoV-19/England/PLYM-332B917/2022_EPI_ISL_9062229 ...	(4271)	CTTTTACATTCTACCATCTATTATCTCTAATGAGAAGCAAGAAATTCTTGGAACTGTTTCTTGGAATTTGCG
hCoV-19/Germany/HE-RKI-I-438397/2022_EPI_ISL_905522...	(4285)	CTTTTACATTCTACCATCTATTATCTCTAATGAGAAGCAAGAAATTCTTGGAACTGTTTCTTGGAATTTGCG
hCoV-19/USA/DE-CDC-LC0472738/2021_EPI_ISL_9049423...	(4249)	CTTTTACATTCTACCATCTATTATCTCTAATGAGAAGCAAGAAATTCTTGGAACTGTTTCTTGGAATTTGCG

Section 62

		4393 4400 4410 4420 4430 4440 4450 4464
	(4393)	
SARS-CoV-2 Reference Genome NC_045512.2	(4393)	AGAAATGCTTGCACATGCAGAAGAAACACGCAAATTAATGCCTGTCTGTGTGGAAACTAAAGCCATAGTTTC
hCoV-19/Botswana/R69B55_BHP_916539/2021_EPI_ISL_90...	(4339)	AGAAATGCTTGCACATGCAGAAGAAACACGCAAATTAATGCCTGTCTGTGTGGAAACTAAAGCCATAGTTTC
hCoV-19/India/HR-MDU-IGIB1210605800489930/2022_EPI...	(4381)	AGAAATGCTTGCACATGCAGAAGAAACACGCAAATTAATGCCTGTCTGTGTGGAAACTAAAGCCATAGTTTC
hCoV-19/England/PLYM-332B917/2022_EPI_ISL_9062229 ...	(4343)	AGAAATGCTTGCACATGCAGAAGAAACACGCAAATTAATGCCTGTCTGTGTGGAAACTAAAGCCATAGTTTC
hCoV-19/Germany/HE-RKI-I-438397/2022_EPI_ISL_905522...	(4357)	AGAAATGCTTGCACATGCAGAAGAAACACGCAAATTAATGCCTGTCTGTGTGGAAACTAAAGCCATAGTTTC
hCoV-19/USA/DE-CDC-LC0472738/2021_EPI_ISL_9049423...	(4321)	AGAAATGCTTGCACATGCAGAAGAAACACGCAAATTAATGCCTGTCTGTGTGGAAACTAAAGCCATAGTTTC

Section 63

		4465 4470 4480 4490 4500 4510 4520 4536
	(4465)	
SARS-CoV-2 Reference Genome NC_045512.2	(4465)	AACTATACAGCGTAAATATAAGGGTATTAAAATACAAGAGGGTGTGGTTGATTATGGTGCTAGATTTTACTT
hCoV-19/Botswana/R69B55_BHP_916539/2021_EPI_ISL_90...	(4411)	AACTATACAGCGTAAATATAAGGGTATTAAAATACAAGAGGGTGTGGTTGATTATGGTGCTAGATTTTACTT
hCoV-19/India/HR-MDU-IGIB1210605800489930/2022_EPI...	(4453)	AACTATACAGCGTAAATATAAGGGTATTAAAATACAAGAGGGTGTGGTTGATTATGGTGCTAGATTTTACTT
hCoV-19/England/PLYM-332B917/2022_EPI_ISL_9062229 ...	(4415)	AACTATACAGCGTAAATATAAGGGTATTAAAATACAAGAGGGTGTGGTTGATTATGGTGCTAGATTTTACTT
hCoV-19/Germany/HE-RKI-I-438397/2022_EPI_ISL_905522...	(4429)	AACTATACAGCGTAAATATAAGGGTATTAAAATACAAGAGGGTGTGGTTGATTATGGTGCTAGATTTTACTT
hCoV-19/USA/DE-CDC-LC0472738/2021_EPI_ISL_9049423...	(4393)	AACTATACAGCGTAAATATAAGGGTATTAAAATACAAGAGGGTGTGGTTGATTATGGTGCTAGATTTTACTT

Section 64

		4537 4550 4560 4570 4580 4590 4608
	(4537)	
SARS-CoV-2 Reference Genome NC_045512.2	(4537)	TTACACCAGTAAAACAACTGTAGCGTCACTTATCAACACACTTAACGATCTAAATGAAACTCTTGTTACAAT
hCoV-19/Botswana/R69B55_BHP_916539/2021_EPI_ISL_90...	(4483)	TTACACCAGTAAAACAACTGTAGCGTCACTTATCAACACACTTAACGATCTAAATGAAACTCTTGTTACAAT
hCoV-19/India/HR-MDU-IGIB1210605800489930/2022_EPI...	(4525)	TTACACCAGTAAAACAACTGTAGCGTCACTTATCAACACACTTAACGATCTAAATGAAACTCTTGTTACAAT
hCoV-19/England/PLYM-332B917/2022_EPI_ISL_9062229 ...	(4487)	TTACACCAGTAAAACAACTGTAGCGTCACTTATCAACACACTTAACGATCTAAATGAAACTCTTGTTACAAT
hCoV-19/Germany/HE-RKI-I-438397/2022_EPI_ISL_905522...	(4501)	TTACACCAGTAAAACAACTGTAGCGTCACTTATCAACACACTTAACGATCTAAATGAAACTCTTGTTACAAT
hCoV-19/USA/DE-CDC-LC0472738/2021_EPI_ISL_9049423...	(4465)	TTACACCAGTAAAACAACTGTAGCGTCACTTATCAACACACTTAACGATCTAAATGAAACTCTTGTTACAAT

Section 65

Sequence	Start	4609 … 4680
	(4609)	4609 4620 4630 4640 4650 4660 4670 4680
SARS-CoV-2 Reference Genome NC_045512.2	(4609)	GCCACTTGGCTATGTAACACATGGCTTAAATTTGGAAGAAGCTGCTCGGTATATGAGATCTCTCAAAGTGCC
hCoV-19/Botswana/R69B55_BHP_916539/2021_EPI_ISL_90...	(4555)	GCCACTTGGCTATGTAACACATGGCTTAAATTTGGAAGAAGCTGCTCGGTATATGAGATCTCTCAAAGTGCC
hCoV-19/India/HR-MDU-IGIB1210605800489930/2022_EPI...	(4597)	GCCACTTGGCTATGTAACACATGGCTTAAATTTGGAAGAAGCTGCTCGGTATATGAGATCTCTCAAAGTGCC
hCoV-19/England/PLYM-332B917/2022_EPI_ISL_9062229 ...	(4559)	GCCACTTGGCTATGTAACACATGGCTTAAATTTGGAAGAAGCTGCTCGGTATATGAGATCTCTCAAAGTGCC
hCoV-19/Germany/HE-RKI-I-438397/2022_EPI_ISL_905522...	(4573)	GCCACTTGGCTATGTAACACATGGCTTAAATTTGGAAGAAGCTGCTCGGTATATGAGATCTCTCAAAGTGCC
hCoV-19/USA/DE-CDC-LC0472738/2021_EPI_ISL_9049423...	(4537)	GCCACTTGGCTATGTAACACATGGCTTAAATTTGGAAGAAGCTGCTCGGTATATGAGATCTCTCAAAGTGCC

Section 66

Sequence	Start	4681 … 4752
	(4681)	4681 4690 4700 4710 4720 4730 4740 4752
SARS-CoV-2 Reference Genome NC_045512.2	(4681)	AGCTACAGTTTCTGTTTCTTCACCTGATGCTGTTACAGCGTATAATGGTTATCTTACTTCTTCTTCTAAAAC
hCoV-19/Botswana/R69B55_BHP_916539/2021_EPI_ISL_90...	(4627)	AGCTACAGTTTCTGTTTCTTCACCTGATGCTGTTACAGCGTATAATGGTTATCTTACTTCTTCTTCTAAAAC
hCoV-19/India/HR-MDU-IGIB1210605800489930/2022_EPI...	(4669)	AGCTACAGTTTCTGTTTCTTCACCTGATGCTGTTACAGCGTATAATGGTTATCTTACTTCTTCTTCTAAAAC
hCoV-19/England/PLYM-332B917/2022_EPI_ISL_9062229 ...	(4631)	AGCTACAGTTTCTGTTTCTTCACCTGATGCTGTTACAGCGTATAATGGTTATCTTACTTCTTCTTCTAAAAC
hCoV-19/Germany/HE-RKI-I-438397/2022_EPI_ISL_905522...	(4645)	AGCTACAGTTTCTGTTTCTTCACCTGATGCTGTTACAGCGTATAATGGTTATCTTACTTCTTCTTCTAAAAC
hCoV-19/USA/DE-CDC-LC0472738/2021_EPI_ISL_9049423...	(4609)	AGCTACAGTTTCTGTTTCTTCACCTGATGCTGTTACAGCGTATAATGGTTATCTTACTTCTTCTTCTAAAAC

Section 67

Sequence	Start	4753 … 4824
	(4753)	4753 4760 4770 4780 4790 4800 4810 4824
SARS-CoV-2 Reference Genome NC_045512.2	(4753)	ACCTGAAGAACATTTTATTGAAACCATCTCACTTGCTGGTTCCTATAAAGATTGGTCCTATTCTGGACAATC
hCoV-19/Botswana/R69B55_BHP_916539/2021_EPI_ISL_90...	(4699)	ACCTGAAGAACATTTTATTGAAACCATCTCACTTGCTGGTTCCTATAAAGATTGGTCCTATTCTGGACAATC
hCoV-19/India/HR-MDU-IGIB1210605800489930/2022_EPI...	(4741)	ACCTGAAGAACATTTTATTGAAACCATCTCACTTGCTGGTTCCTATAAAGATTGGTCCTATTCTGGACAATC
hCoV-19/England/PLYM-332B917/2022_EPI_ISL_9062229 ...	(4703)	ACCTGAAGAACATTTTATTGAAACCATCTCACTTGCTGGTTCCTATAAAGATTGGTCCTATTCTGGACAATC
hCoV-19/Germany/HE-RKI-I-438397/2022_EPI_ISL_905522...	(4717)	ACCTGAAGAACATTTTATTGAAACCATCTCACTTGCTGGTTCCTATAAAGATTGGTCCTATTCTGGACAATC
hCoV-19/USA/DE-CDC-LC0472738/2021_EPI_ISL_9049423...	(4681)	ACCTGAAGAACATTTTATTGAAACCATCTCACTTGCTGGTTCCTATAAAGATTGGTCCTATTCTGGACAATC

Section 68

Sequence	Start	4825 … 4896
	(4825)	4825 4830 4840 4850 4860 4870 4880 4896
SARS-CoV-2 Reference Genome NC_045512.2	(4825)	TACACAACTAGGTATAGAATTTCTTAAGAGAGGTGATAAAAGTGTATATTACACTAGTAATCCTACCACATT
hCoV-19/Botswana/R69B55_BHP_916539/2021_EPI_ISL_90...	(4771)	TACACAACTAGGTATAGAATTTCTTAAGAGAGGTGATAAAAGTGTATATTACACTAGTAATCCTACCACATT
hCoV-19/India/HR-MDU-IGIB1210605800489930/2022_EPI...	(4813)	TACACAACTAGGTATAGAATTTCTTAAGAGAGGTGATAAAAGTGTATATTACACTAGTAATCCTACCACATT
hCoV-19/England/PLYM-332B917/2022_EPI_ISL_9062229 ...	(4775)	TACACAACTAGGTATAGAATTTCTTAAGAGAGGTGATAAAAGTGTATATTACACTAGTAATCCTACCACATT
hCoV-19/Germany/HE-RKI-I-438397/2022_EPI_ISL_905522...	(4789)	TACACAACTAGGTATAGAATTTCTTAAGAGAGGTGATAAAAGTGTATATTACACTAGTAATCCTACCACATT
hCoV-19/USA/DE-CDC-LC0472738/2021_EPI_ISL_9049423...	(4753)	TACACAACTAGGTATAGAATTTCTTAAGAGAGGTGATAAAAGTGTATATTACACTAGTAATCCTACCACATT

Section 69

```
                                                          (4897) 4897         4910      4920      4930      4940      4950                 4968
                SARS-CoV-2 Reference Genome NC_045512.2   (4897) CCACCTAGATGGTGAAGTTATCACCTTTGACAATCTTAAGACACTTCTTTCTTTGAGAGAAGTGAGGACTAT
hCoV-19/Botswana/R69B55_BHP_916539/2021_EPI_ISL_90...     (4843) CCACCTAGATGGTGAAGTTATCACCTTTGACAATCTTAAGACACTTCTTTCTTTGAGAGAAGTGAGGACTAT
 hCoV-19/India/HR-MDU-IGIB1210605800489930/2022_EPI...    (4885) CCACCTAGATGGTGAAGTTATCACCTTTGACAATCTTAAGACACTTCTTTCTTTGAGAGAAGTGAGGACTAT
  hCoV-19/England/PLYM-332B917/2022_EPI_ISL_9062229 ...   (4847) CCACCTAGATGGTGAAGTTATCACCTTTGACAATCTTAAGACACTTCTTTCTTTGAGAGAAGTGAGGACTAT
 hCoV-19/Germany/HE-RKI-I-438397/2022_EPI_ISL_905522...   (4861) CCACCTAGATGGTGAAGTTATCACCTTTGACAATCTTAAGACACTTCTTTCTTTGAGAGAAGTGAGGACTAT
  hCoV-19/USA/DE-CDC-LC0472738/2021_EPI_ISL_9049423...    (4825) CCACCTAGATGGTGAAGTTATCACCTTTGACAATCTTAAGACACTTCTTTCTTTGAGAGAAGTGAGGACTAT
```

Section 70

```
                                                          (4969) 4969       4980      4990      5000      5010      5020      5030     5040
                SARS-CoV-2 Reference Genome NC_045512.2   (4969) TAAGGTGTTTACAACAGTAGACAACATTAACCTCCACACGCAAGTTGTGGACATGTCAATGACATATGGACA
hCoV-19/Botswana/R69B55_BHP_916539/2021_EPI_ISL_90...     (4915) TAAGGTGTTTACAACAGTAGACAACATTAACCTCCACACGCAAGTTGTGGACATGTCAATGACATATGGACA
 hCoV-19/India/HR-MDU-IGIB1210605800489930/2022_EPI...    (4957) TAAGGTGTTTACAACAGTAGACAACATTAACCTCCACACGCAAGTTGTGGACATGTCAATGACATATGGACA
  hCoV-19/England/PLYM-332B917/2022_EPI_ISL_9062229 ...   (4919) TAAGGTGTTTACAACAGTAGACAACATTAACCTCCACACGCAAGTTGTGGACATGTCAATGACATATGGACA
 hCoV-19/Germany/HE-RKI-I-438397/2022_EPI_ISL_905522...   (4933) TAAGGTGTTTACAACAGTAGACAACATTAACCTCCACACGCAAGTTGTGGACATGTCAATGACATATGGACA
  hCoV-19/USA/DE-CDC-LC0472738/2021_EPI_ISL_9049423...    (4897) TAAGGTGTTTACAACAGTAGACAACATTAACCTCCACACGCAAGTTGTGGACATGTCAATGACATATGGACA
```

Section 71

```
                                                          (5041) 5041     5050      5060      5070      5080      5090      5100         5112
                SARS-CoV-2 Reference Genome NC_045512.2   (5041) ACAGTTTGGTCCAACTTATTTGGATGGAGCTGATGTTACTAAAATAAAACCTCATAATTCACATGAAGGTAA
hCoV-19/Botswana/R69B55_BHP_916539/2021_EPI_ISL_90...     (4987) ACAGTTTGGTCCAACTTATTTGGATGGAGCTGATGTTACTAAAATAAAACCTCATAATTCACATGAAGGTAA
 hCoV-19/India/HR-MDU-IGIB1210605800489930/2022_EPI...    (5029) ACAGTTTGGTCCAACTTATTTGGATGGAGCTGATGTTACTAAAATAAAACCTCATAATTCACATGAAGGTAA
  hCoV-19/England/PLYM-332B917/2022_EPI_ISL_9062229 ...   (4991) ACAGTTTGGTCCAACTTATTTGGATGGAGCTGATGTTACTAAAATAAAACCTCATAATTCACATGAAGGTAA
 hCoV-19/Germany/HE-RKI-I-438397/2022_EPI_ISL_905522...   (5005) ACAGTTTGGTCCAACTTATTTGGATGGAGCTGATGTTACTAAAATAAAACCTCATAATTCACATGAAGGTAA
  hCoV-19/USA/DE-CDC-LC0472738/2021_EPI_ISL_9049423...    (4969) ACAGTTTGGTCCAACTTATTTGGATGGAGCTGATGTTACTAAAATAAAACCTCATAATTCACATGAAGGTAA
```

Section 72

```
                                                          (5113) 5113   5120      5130      5140      5150      5160      5170            5184
                SARS-CoV-2 Reference Genome NC_045512.2   (5113) AACATTTTATGTTTTACCTAATGATGACACTCTACGTGTTGAGGCTTTTGAGTACTACCACACAACTGATCC
hCoV-19/Botswana/R69B55_BHP_916539/2021_EPI_ISL_90...     (5059) AACATTTTATGTTTTACCTAATGATGACACTCTACGTGTTGAGGCTTTTGAGTACTACCACACAACTGATCC
 hCoV-19/India/HR-MDU-IGIB1210605800489930/2022_EPI...    (5101) AACATTTTATGTTTTACCTAATGATGACACTCTACGTGTTGAGGCTTTTGAGTACTACCACACAACTGATCC
  hCoV-19/England/PLYM-332B917/2022_EPI_ISL_9062229 ...   (5063) AACATTTTATGTTTTACCTAATGATGACACTCTACGTGTTGAGGCTTTTGAGTACTACCACACAACTGATCC
 hCoV-19/Germany/HE-RKI-I-438397/2022_EPI_ISL_905522...   (5077) AACATTTTATGTTTTACCTAATGATGACACTCTACGTGTTGAGGCTTTTGAGTACTACCACACAACTGATCC
  hCoV-19/USA/DE-CDC-LC0472738/2021_EPI_ISL_9049423...    (5041) AACATTTTATGTTTTACCTAATGATGACACTCTACGTGTTGAGGCTTTTGAGTACTACCACACAACTGATCC
```

```
Section 73
                                                        (5185) 5185 5190      5200      5210      5220      5230      5240            5256
SARS-CoV-2 Reference Genome NC_045512.2                 (5185) TAGTTTTCTGGGTAGGTACATGTCAGCATTAAATCACACTAAAAAGTGGAAATACCCACAAGTTAATGGTTT
hCoV-19/Botswana/R69B55_BHP_916539/2021_EPI_ISL_90...   (5131) TAGTTTTCTGGGTAGGTACATGTCAGCATTAAATCACACTAAAAAGTGGAAATACCCACAAGTTAATGGTTT
hCoV-19/India/HR-MDU-IGIB1210605800489930/2022_EPI...   (5173) TAGTTTTCTGGGTAGGTACATGTCAGCATTAAATCACACTAAAAAGTGGAAATACCCACAAGTTAATGGTTT
hCoV-19/England/PLYM-332B917/2022_EPI_ISL_9062229 ...   (5135) TAGTTTTCTGGGTAGGTACATGTCAGCATTAAATCACACTAAAAAGTGGAAATACCCACAAGTTAATGGTTT
hCoV-19/Germany/HE-RKI-I-438397/2022_EPI_ISL_905522...  (5149) TAGTTTTCTGGGTAGGTACATGTCAGCATTAAATCACACTAAAAAGTGGAAATACCCACAAGTTAATGGTTT
hCoV-19/USA/DE-CDC-LC0472738/2021_EPI_ISL_9049423...    (5113) TAGTTTTCTGGGTAGGTACATGTCAGCATTAAATCACACTAAAAAGTGGAAATACCCACAAGTTAATGGTTT

Section 74
                                                        (5257) 5257         5270      5280      5290      5300      5310            5328
SARS-CoV-2 Reference Genome NC_045512.2                 (5257) AACTTCTATTAAATGGGCAGATAACAACTGTTATCTTGCCACTGCATTGTTAACACTCCAACAAATAGAGTT
hCoV-19/Botswana/R69B55_BHP_916539/2021_EPI_ISL_90...   (5203) AACTTCTATTAAATGGGCAGATAACAACTGTTATCTTGCCACTGCATTGTTAACACTCCAACAAATAGAGTT
hCoV-19/India/HR-MDU-IGIB1210605800489930/2022_EPI...   (5245) AACTTCTATTAAATGGGCAGATAACAACTGTTATCTTGCCACTGCATTGTTAACACTCCAACAAATAGAGTT
hCoV-19/England/PLYM-332B917/2022_EPI_ISL_9062229 ...   (5207) AACTTCTATTAAATGGGCAGATAACAACTGTTATCTTGCCACTGCATTGTTAACACTCCAACAAATAGAGTT
hCoV-19/Germany/HE-RKI-I-438397/2022_EPI_ISL_905522...  (5221) AACTTCTATTAAATGGGCAGATAACAACTGTTATCTTGCCACTGCATTGTTAACACTCCAACAAATAGAGTT
hCoV-19/USA/DE-CDC-LC0472738/2021_EPI_ISL_9049423...    (5185) AACTTCTATTAAATGGGCAGATAACAACTGTTATCTTGCCACTGCATTGTTAACACTCCAACAAATAGAGTT

Section 75
                                                        (5329) 5329       5340      5350      5360      5370      5380      5390    5400
SARS-CoV-2 Reference Genome NC_045512.2                 (5329) GAAGTTTAATCCACCTGCTCTACAAGATGCTTATTACAGAGCAAGGGCTGGTGAAGCTGCTAACTTTTGTGC
hCoV-19/Botswana/R69B55_BHP_916539/2021_EPI_ISL_90...   (5275) GAAGTTTAATCCACCTGCTCTACAAGATGCTTATTACAGAGCAAGGGCTGGTGAAGCGGCTAACTTTTGTGC
hCoV-19/India/HR-MDU-IGIB1210605800489930/2022_EPI...   (5317) GAAGTTTAATCCACCTGCTCTACAAGATGCTTATTACAGAGCAAGGGCTGGTGAAGCGGCTAACTTTTGTGC
hCoV-19/England/PLYM-332B917/2022_EPI_ISL_9062229 ...   (5279) GAAGTTTAATCCACCTGCTCTACAAGATGCTTATTACAGAGCAAGGGCTGGTGAAGCGGCTAACTTTTGTGC
hCoV-19/Germany/HE-RKI-I-438397/2022_EPI_ISL_905522...  (5293) GAAGTTTAATCCACCTGCTCTACAAGATGCTTATTACAGAGCAAGGGCTGGTGAAGCGGCTAACTTTTGTGC
hCoV-19/USA/DE-CDC-LC0472738/2021_EPI_ISL_9049423...    (5257) GAAGTTTAATCCACCTGCTCTACAAGATGCTTATTACAGAGCAAGGGCTGGTGAAGCGGCTAACTTTTGTGC

Section 76
                                                        (5401) 5401     5410      5420      5430      5440      5450      5460      5472
SARS-CoV-2 Reference Genome NC_045512.2                 (5401) ACTTATCTTAGCCTACTGTAATAAGACAGTAGGTGAGTTAGGTGATGTTAGAGAAACAATGAGTTACTTGTT
hCoV-19/Botswana/R69B55_BHP_916539/2021_EPI_ISL_90...   (5347) ACTTATCTTAGCCTACTGTAATAAGACAGTAGGTGAGTTAGGTGATGTTAGAGAAACAATGAGTTACTTGTT
hCoV-19/India/HR-MDU-IGIB1210605800489930/2022_EPI...   (5389) ACTTATCTTAGCCTACTGTAATAAGACAGTAGGTGAGTTAGGTGATGTTAGAGAAACAATGAGTTACTTGTT
hCoV-19/England/PLYM-332B917/2022_EPI_ISL_9062229 ...   (5351) ACTTATCTTAGCCTACTGTAATAAGACAGTAGGTGAGTTAGGTGATGTTAGAGAAACAATGAGTTACTTGTT
hCoV-19/Germany/HE-RKI-I-438397/2022_EPI_ISL_905522...  (5365) ACTTATCTTAGCCTACTGTAATAAGACAGTAGGTGAGTTAGGTGATGTTAGAGAAACAATGAGTTACTTGTT
hCoV-19/USA/DE-CDC-LC0472738/2021_EPI_ISL_9049423...    (5329) ACTTATCTTAGCCTACTGTAATAAGACAGTAGGTGAGTTAGGTGATGTTAGAGAAACAATGAGTTACTTGTT
```

Omicron BA.1

Section 77

	(5473)	5473 5480 5490 5500 5510 5520 5530 5544
SARS-CoV-2 Reference Genome NC_045512.2	(5473)	TCAACATGCCAATTTAGATTCTTGCAAAAGAGTCTTGAACGTGGTGTGTAAAACTTGTGGACAACAGCAGAC
hCoV-19/Botswana/R69B55_BHP_916539/2021_EPI_ISL_90...	(5419)	TCAACATGCCAATTTAGATTCTTGCAAAAGAGTCTTGAACGTGGTGTGTAAAACTTGTGGACAACAGCAGAC
hCoV-19/India/HR-MDU-IGIB1210605800489930/2022_EPI...	(5461)	TCAACATGCCAATTTAGATTCTTGCAAAAGAGTCTTGAACGTGGTGTGTAAAACTTGTGGACAACAGCAGAC
hCoV-19/England/PLYM-332B917/2022_EPI_ISL_9062229_...	(5423)	TCAACATGCCAATTTAGATTCTTGCAAAAGAGTCTTGAACGTGGTGTGTAAAACTTGTGGACAACAGCAGAC
hCoV-19/Germany/HE-RKI-I-438397/2022_EPI_ISL_905522...	(5437)	TCAACATGCCAATTTAGATTCTTGCAAAAGAGTCTTGAACGTGGTGTGTAAAACTTGTGGACAACAGCAGAC
hCoV-19/USA/DE-CDC-LC0472738/2021_EPI_ISL_9049423...	(5401)	TCAACATGCCAATTTAGATTCTTGCAAAAGAGTCTTGAACGTGGTGTGTAAAACTTGTGGACAACAGCAGAC

Section 78

	(5545)	5545 5550 5560 5570 5580 5590 5600 5616
SARS-CoV-2 Reference Genome NC_045512.2	(5545)	AACCCTTAAGGGTGTAGAAGCTGTTATGTACATGGGCACACTTTCTTATGAACAATTTAAGAAAGGTGTTCA
hCoV-19/Botswana/R69B55_BHP_916539/2021_EPI_ISL_90...	(5491)	AACCCTTAAGGGTGTAGAAGCTGTTATGTACATGGGCACACTTTCTTATGAACAATTTAAGAAAGGTGTTCA
hCoV-19/India/HR-MDU-IGIB1210605800489930/2022_EPI...	(5533)	AACCCTTAAGGGTGTAGAAGCTGTTATGTACATGGGCACACTTTCTTATGAACAATTTAAGAAAGGTGTTCA
hCoV-19/England/PLYM-332B917/2022_EPI_ISL_9062229_...	(5495)	AACCCTTAAGGGTGTAGAAGCTGTTATGTACATGGGCACACTTTCTTATGAACAATTTAAGAAAGGTGTTCA
hCoV-19/Germany/HE-RKI-I-438397/2022_EPI_ISL_905522...	(5509)	AACCCTTAAGGGTGTAGAAGCTGTTATGTACATGGGCACACTTTCTTATGAACAATTTAAGAAAGGTGTTCA
hCoV-19/USA/DE-CDC-LC0472738/2021_EPI_ISL_9049423...	(5473)	AACCCTTAAGGGTGTAGAAGCTGTTATGTACATGGGCACACTTTCTTATGAACAATTTAAGAAAGGTGTTCA

Section 79

	(5617)	5617 5630 5640 5650 5660 5670 5688
SARS-CoV-2 Reference Genome NC_045512.2	(5617)	GATACCTTGTACGTGTGGTAAACAAGCTACAAAATATCTAGTACAACAGGAGTCACCTTTTGTTATGATGTC
hCoV-19/Botswana/R69B55_BHP_916539/2021_EPI_ISL_90...	(5563)	GATACCTTGTACGTGTGGTAAACAAGCTACAAAATATCTAGTACAACAGGAGTCACCTTTTGTTATGATGTC
hCoV-19/India/HR-MDU-IGIB1210605800489930/2022_EPI...	(5605)	GATACCTTGTACGTGTGGTAAACAAGCTACAAAATATCTAGTACAACAGGAGTCACCTTTTGTTATGATGTC
hCoV-19/England/PLYM-332B917/2022_EPI_ISL_9062229_...	(5567)	GATACCTTGTACGTGTGGTAAACAAGCTACAAAATATCTAGTACAACAGGAGTCACCTTTTGTTATGATGTC
hCoV-19/Germany/HE-RKI-I-438397/2022_EPI_ISL_905522...	(5581)	GATACCTTGTACGTGTGGTAAACAAGCTACAAAATATCTAGTACAACAGGAGTCACCTTTTGTTATGATGTC
hCoV-19/USA/DE-CDC-LC0472738/2021_EPI_ISL_9049423...	(5545)	GATACCTTGTACGTGTGGTAAACAAGCTACAAAATATCTAGTACAACAGGAGTCACCTTTTGTTATGATGTC

Section 80

	(5689)	5689 5700 5710 5720 5730 5740 5750 5760
SARS-CoV-2 Reference Genome NC_045512.2	(5689)	AGCACCACCTGCTCAGTATGAACTTAAGCATGGTACATTTACTTGTGCTAGTGAGTACACTGGTAATTACCA
hCoV-19/Botswana/R69B55_BHP_916539/2021_EPI_ISL_90...	(5635)	AGCACCACCTGCTCAGTATGAACTTAAGCATGGTACATTTATTTGTGCTAGTGAGTACACTGGTAATTACCA
hCoV-19/India/HR-MDU-IGIB1210605800489930/2022_EPI...	(5677)	AGCACCACCTGCTCAGTATGAACTTAAGCATGGTACATTTATTTGTGCTAGTGAGTACACTGGTAATTACCA
hCoV-19/England/PLYM-332B917/2022_EPI_ISL_9062229_...	(5639)	AGCACCACCTGCTCAGTATGAACTTAAGCATGGTACATTTACTTGTGCTAGTGAGTACACTGGTAATTACCA
hCoV-19/Germany/HE-RKI-I-438397/2022_EPI_ISL_905522...	(5653)	AGCACCACCTGCTCAGTATGAACTTAAGCATGGTACATTTACTTGTGCTAGTGAGTACACTGGTAATTACCA
hCoV-19/USA/DE-CDC-LC0472738/2021_EPI_ISL_9049423...	(5617)	AGCACCACCTGCTCAGTATGAACTTAAGCATGGTACATTTACTTGTGCTAGTGAGTACACTGGTAATTACCA

Section 81

```
                                                  (5761) 5761     5770      5780      5790      5800      5810      5820      5832
             SARS-CoV-2 Reference Genome NC_045512.2 (5761) GTGTGGTCACTATAAACATATAACTTCTAAAGAAACTTTGTATTGCATAGACGGTGCTTTACTTACAAAGTC
hCoV-19/Botswana/R69B55_BHP_916539/2021_EPI_ISL_90... (5707) GTGTGGTCACTATAAACATATAACTTCTAAAGAAACTTTGTATTGCATAGACGGTGCTTTACTTACAAAGTC
 hCoV-19/India/HR-MDU-IGIB1210605800489930/2022_EPI... (5749) GTGTGGTCACTATAAACATATAACTTCTAAAGAAACTTTGTATTGCATAGACGGTGCTTTACTTACAAAGTC
  hCoV-19/England/PLYM-332B917/2022_EPI_ISL_9062229_... (5711) GTGTGGTCACTATAAACATATAACTTCTAAAGAAACTTTGTATTGCATAGACGGTGCTTTACTTACAAAGTC
 hCoV-19/Germany/HE-RKI-I-438397/2022_EPI_ISL_905522... (5725) GTGTGGTCACTATAAACATATAACTTCTAAAGAAACTTTGTATTGCATAGACGGTGCTTTACTTACAAAGTC
  hCoV-19/USA/DE-CDC-LC0472738/2021_EPI_ISL_9049423... (5689) GTGTGGTCACTATAAACATATAACTTCTAAAGAAACTTTGTATTGCATAGACGGTGCTTTACTTACAAAGTC
```

Section 82

```
                                                  (5833) 5833  5840      5850      5860      5870      5880      5890          5904
             SARS-CoV-2 Reference Genome NC_045512.2 (5833) CTCAGAATACAAAGGTCCTATTACGGATGTTTTCTACAAAGAAAACAGTTACACAACAACCATAAAACCAGT
hCoV-19/Botswana/R69B55_BHP_916539/2021_EPI_ISL_90... (5779) CTCAGAATACAAAGGTCCTATTACGGATGTTTTCTACAAAGAAAACAGTTACACAACAACCATAAAACCAGT
 hCoV-19/India/HR-MDU-IGIB1210605800489930/2022_EPI... (5821) CTCAGAATACAAAGGTCCTATTACGGATGTTTTCTACAAAGAAAACAGTTACACAACAACCATAAAACCAGT
  hCoV-19/England/PLYM-332B917/2022_EPI_ISL_9062229_... (5783) CTCAGAATACAAAGGTCCTATTACGGATGTTTTCTACAAAGAAAACAGTTACACAACAACCATAAAACCAGT
 hCoV-19/Germany/HE-RKI-I-438397/2022_EPI_ISL_905522... (5797) CTCAGAATACAAAGGTCCTATTACGGATGTTTTCTACAAAGAAAACAGTTACACAACAACCATAAAACCAGT
  hCoV-19/USA/DE-CDC-LC0472738/2021_EPI_ISL_9049423... (5761) CTCAGAATACAAAGGTCCTATTACGGATGTTTTCTACAAAGAAAACAGTTACACAACAACCATAAAACCAGT
```

Section 83

```
                                                  (5905) 5905 5910      5920      5930      5940      5950      5960           5976
             SARS-CoV-2 Reference Genome NC_045512.2 (5905) TACTTATAAATTGGATGGTGTTGTTTGTACAGAAATTGACCCTAAGTTGGACAATTATTATAAGAAAGACAA
hCoV-19/Botswana/R69B55_BHP_916539/2021_EPI_ISL_90... (5851) TACTTATAAATTGGATGGTGTTGTTTGTACAGAAATTGACCCTAAGTTGGACAATTATTATAAGAAAGACAA
 hCoV-19/India/HR-MDU-IGIB1210605800489930/2022_EPI... (5893) TACTTATAAATTGGATGGTGTTGTTTGTACAGAAATTGACCCTAAGTTGGACAATTATTATAAGAAAGACAA
  hCoV-19/England/PLYM-332B917/2022_EPI_ISL_9062229_... (5855) TACTTATAAATTGGATGGTGTTGTTTGTACAGAAATTGACCCTAAGTTGGACAATTATTATAAGAAAGACAA
 hCoV-19/Germany/HE-RKI-I-438397/2022_EPI_ISL_905522... (5869) TACTTATAAATTGGATGGTGTTGTTTGTACAGAAATTGACCCTAAGTTGGACAATTATTATAAGAAAGACAA
  hCoV-19/USA/DE-CDC-LC0472738/2021_EPI_ISL_9049423... (5833) TACTTATAAATTGGATGGTGTTGTTTGTACAGAAATTGACCCTAAGTTGGACAATTATTATAAGAAAGACAA
```

Section 84

```
                                                  (5977) 5977         5990      6000      6010      6020      6030               6048
             SARS-CoV-2 Reference Genome NC_045512.2 (5977) TTCTTATTTCACAGAGCAACCAATTGATCTTGTACCAAACCAACCATATCCAAACGCAAGCTTCGATAATTT
hCoV-19/Botswana/R69B55_BHP_916539/2021_EPI_ISL_90... (5923) TTCTTATTTCACAGAGCAACCAATTGATCTTGTACCAAACCAACCATATCCAAACGCAAGCTTCGATAATTT
 hCoV-19/India/HR-MDU-IGIB1210605800489930/2022_EPI... (5965) TTCTTATTTCACAGAGCAACCAATTGATCTTGTACCAAACCAACCATATCCAAACGCAAGCTTCGATAATTT
  hCoV-19/England/PLYM-332B917/2022_EPI_ISL_9062229_... (5927) TTCTTATTTCACAGAGCAACCAATTGATCTTGTACCAAACCAACCATATCCAAACGCAAGCTTCGATAATTT
 hCoV-19/Germany/HE-RKI-I-438397/2022_EPI_ISL_905522... (5941) TTCTTATTTCACAGAGCAACCAATTGATCTTGTACCAAACCAACCATATCCAAACGCAAGCTTCGATAATTT
  hCoV-19/USA/DE-CDC-LC0472738/2021_EPI_ISL_9049423... (5905) TTCTTATTTCACAGAGCAACCAATTGATCTTGTACCAAACCAACCATATCCAAACGCAAGCTTCGATAATTT
```

Section 85

Sequence	Start	6049 … 6120
	(6049)	6049 6060 6070 6080 6090 6100 6110 6120
SARS-CoV-2 Reference Genome NC_045512.2	(6049)	TAAGTTTGTATGTGATAATATCAAATTTGCTGATGATTTAAACCAGTTAACTGGTTATAAGAAACCTGCTTC
hCoV-19/Botswana/R69B55_BHP_916539/2021_EPI_ISL_90...	(5995)	TAAGTTTGTATGTGATAATATCAAATTTGCTGATGATTTAAACCAGTTAACTGGTTATAAGAAACCTGCTTC
hCoV-19/India/HR-MDU-IGIB1210605800489930/2022_EPI...	(6037)	TAAGTTTGTATGTGATAATATCAAATTTGCTGATGATTTAAACCAGTTAACTGGTTATAAGAAACCTGCTTC
hCoV-19/England/PLYM-332B917/2022_EPI_ISL_9062229_...	(5999)	TAAGTTTGTATGTGATAATATCAAATTTGCTGATGATTTAAACCAGTTAACTGGTTATAAGAAACCTGCTTC
hCoV-19/Germany/HE-RKI-I-438397/2022_EPI_ISL_905522...	(6013)	TAAGTTTGTATGTGATAATATCAAATTTGCTGATGATTTAAACCAGTTAACTGGTTATAAGAAACCTGCTTC
hCoV-19/USA/DE-CDC-LC0472738/2021_EPI_ISL_9049423...	(5977)	TAAGTTTGTATGTGATAATATCAAATTTGCTGATGATTTAAACCAGTTAACTGGTTATAAGAAACCTGCTTC

Section 86

Sequence	Start	6121 … 6192
	(6121)	6121 6130 6140 6150 6160 6170 6180 6192
SARS-CoV-2 Reference Genome NC_045512.2	(6121)	AAGAGAGCTTAAAGTTACATTTTTCCCTGACTTAAATGGTGATGTGGTGGCTATTGATTATAAACACTACAC
hCoV-19/Botswana/R69B55_BHP_916539/2021_EPI_ISL_90...	(6067)	AAGAGAGCTTAAAGTTACATTTTTCCCTGACTTAAATGGTGATGTGGTGGCTATTGATTATAAACACTACAC
hCoV-19/India/HR-MDU-IGIB1210605800489930/2022_EPI...	(6109)	AAGAGAGCTTAAAGTTACATTTTTCCCTGACTTAAATGGTGATGTGGTGGCTATTGATTATAAACACTACAC
hCoV-19/England/PLYM-332B917/2022_EPI_ISL_9062229_...	(6071)	AAGAGAGCTTAAAGTTACATTTTTCCCTGACTTAAATGGTGATGTGGTGGCTATTGATTATAAACACTACAC
hCoV-19/Germany/HE-RKI-I-438397/2022_EPI_ISL_905522...	(6085)	AAGAGAGCTTAAAGTTACATTTTTCCCTGACTTAAATGGTGATGTGGTGGCTATTGATTATAAACACTACAC
hCoV-19/USA/DE-CDC-LC0472738/2021_EPI_ISL_9049423...	(6049)	AAGAGAGCTTAAAGTTACATTTTTCCCTGACTTAAATGGTGATGTGGTGGCTATTGATTATAAACACTACAC

Section 87

Sequence	Start	6193 … 6264
	(6193)	6193 6200 6210 6220 6230 6240 6250 6264
SARS-CoV-2 Reference Genome NC_045512.2	(6193)	ACCCTCTTTTAAGAAAGGAGCTAAATTGTTACATAAACCTATTGTTTGGCATGTTAACAATGCAACTAATAA
hCoV-19/Botswana/R69B55_BHP_916539/2021_EPI_ISL_90...	(6139)	ACCCTCTTTTAAGAAAGGAGCTAAATTGTTACATAAACCTATTGTTTGGCATGTTAACAATGCAACTAATAA
hCoV-19/India/HR-MDU-IGIB1210605800489930/2022_EPI...	(6181)	ACCCTCTTTTAAGAAAGGAGCTAAATTGTTACATAAACCTATTGTTTGGCATGTTAACAATGCAACTAATAA
hCoV-19/England/PLYM-332B917/2022_EPI_ISL_9062229_...	(6143)	ACCCTCTTTTAAGAAAGGAGCTAAATTGTTACATAAACCTATTGTTTGGCATGTTAACAATGCAACTAATAA
hCoV-19/Germany/HE-RKI-I-438397/2022_EPI_ISL_905522...	(6157)	ACCCTCTTTTAAGAAAGGAGCTAAATTGTTACATAAACCTATTGTTTGGCATGTTAACAATGCAACTAATAA
hCoV-19/USA/DE-CDC-LC0472738/2021_EPI_ISL_9049423...	(6121)	ACCCTCTTTTAAGAAAGGAGCTAAATTGTTACATAAACCTATTGTTTGGCATGTTAACAATGCAACTAATAA

Section 88

Sequence	Start	6265 … 6336
	(6265)	6265 6270 6280 6290 6300 6310 6320 6336
SARS-CoV-2 Reference Genome NC_045512.2	(6265)	AGCCACGTATAAACCAAATACCTGGTGTATACGTTGTCTTTGGAGCACAAAACCAGTTGAAACATCAAATTC
hCoV-19/Botswana/R69B55_BHP_916539/2021_EPI_ISL_90...	(6211)	AGCCACGTATAAACCAAATACCTGGTGTATACGTTGTCTTTGGAGCACAAAACCAGTTGAAACATCAAATTC
hCoV-19/India/HR-MDU-IGIB1210605800489930/2022_EPI...	(6253)	AGCCACGTATAAACCAAATACCTGGTGTATACGTTGTCTTTGGAGCACAAAACCAGTTGAAACATCAAATTC
hCoV-19/England/PLYM-332B917/2022_EPI_ISL_9062229_...	(6215)	AGCCACGTATAAACCAAATACCTGGTGTATACGTTGTCTTTGGAGCACAAAACCAGTTGAAACATCAAATTC
hCoV-19/Germany/HE-RKI-I-438397/2022_EPI_ISL_905522...	(6229)	AGCCACGTATAAACCAAATACCTGGTGTATACGTTGTCTTTGGAGCACAAAACCAGTTGAAACATCAAATTC
hCoV-19/USA/DE-CDC-LC0472738/2021_EPI_ISL_9049423...	(6193)	AGCCACGTATAAACCAAATACCTGGTGTATACGTTGTCTTTGGAGCACAAAACCAGTTGAAACATCAAATTC

```
                                                                    Section 89
                                                   (6337) 6337         6350       6360       6370       6380       6390          6408
              SARS-CoV-2 Reference Genome NC_045512.2 (6337) GTTTGATGTACTGAAGTCAGAGGACGCGCAGGGAATGGATAATCTTGCCTGCGAAGATCTAAAACCAGTCTC
hCoV-19/Botswana/R69B55_BHP_916539/2021_EPI_ISL_90... (6283) GTTTGATGTACTGAAGTCAGAGGACGCGCAGGGAATGGATAATCTTGCCTGCGAAGATCTAAAACCAGTCTC
 hCoV-19/India/HR-MDU-IGIB1210605800489930/2022_EPI... (6325) GTTTGATGTACTGAAGTCAGAGGACGCGCAGGGAATGGATAATCTTGCCTGCGAAGATCTAAAACCAGTCTC
  hCoV-19/England/PLYM-332B917/2022_EPI_ISL_9062229 ... (6287) GTTTGATGTACTGAAGTCAGAGGACGCGCAGGGAATGGATAATCTTGCCTGCGAAGATCTAAAACCAGTCTC
hCoV-19/Germany/HE-RKI-I-438397/2022_EPI_ISL_905522... (6301) GTTTGATGTACTGAAGTCAGAGGACGCGCAGGGAATGGATAATCTTGCCTGCGAAGATCTAAAACCAGTCTC
 hCoV-19/USA/DE-CDC-LC0472738/2021_EPI_ISL_9049423... (6265) GTTTGATGTACTGAAGTCAGAGGACGCGCAGGGAATGGATAATCTTGCCTGCGAAGATCTAAAACCAGTCTC

                                                                    Section 90
                                                   (6409) 6409       6420       6430       6440       6450       6460       6470    6480
              SARS-CoV-2 Reference Genome NC_045512.2 (6409) TGAAGAAGTAGTGGAAAATCCTACCATACAGAAAGACGTTCTTGAGTGTAATGTGAAAACTACCGAAGTTGT
hCoV-19/Botswana/R69B55_BHP_916539/2021_EPI_ISL_90... (6355) TGAAGAAGTAGTGGAAAATCCTACCATACAGAAAGACGTTCTTGAGTGTAATGTGAAAACTACCGAAGTTGT
 hCoV-19/India/HR-MDU-IGIB1210605800489930/2022_EPI... (6397) TGAAGAAGTAGTGGAAAATCCTACCATACAGAAAGACGTTCTTGAGTGTAATGTGAAAACTACCGAAGTTGT
  hCoV-19/England/PLYM-332B917/2022_EPI_ISL_9062229 ... (6359) TGAAGAAGTAGTGGAAAATCCTACCATACAGAAAGACGTTCTTGAGTGTAATGTGAAAACTACCGAAGTTGT
hCoV-19/Germany/HE-RKI-I-438397/2022_EPI_ISL_905522... (6373) TGAAGAAGTAGTGGAAAATCCTACCATACAGAAAGACGTTCTTGAGTGTAATGTGAAAACTACCGAAGTTGT
 hCoV-19/USA/DE-CDC-LC0472738/2021_EPI_ISL_9049423... (6337) TGAAGAAGTAGTGGAAAATCCTACCATACAGAAAGACGTTCTTGAGTGTAATGTGAAAACTACCGAAGTTGT

                                                                    Section 91
                                                   (6481) 6481     6490       6500       6510       6520       6530       6540     6552
              SARS-CoV-2 Reference Genome NC_045512.2 (6481) AGGAGACATTATACTTAAACCAGCAAATAATAGTTTAAAAATTACAGAAGAGGTTGGCCACACAGATCTAAT
hCoV-19/Botswana/R69B55_BHP_916539/2021_EPI_ISL_90... (6427) AGGAGACATTATACTTAAACCAGCAAATAATANNNTAAAAATTACAGAAGAGGTTGGCCACACAGATCTAAT
 hCoV-19/India/HR-MDU-IGIB1210605800489930/2022_EPI... (6469) AGGAGACATTATACTTAAACCAGCAAATAATAGTTTAAAAATTACAGAAGAGGTTGGCCACACAGATCTAAT
  hCoV-19/England/PLYM-332B917/2022_EPI_ISL_9062229 ... (6431) AGGAGACATTATACTTAAACCAGCAAATAATA---TAAAAATTACAGAAGAGGTTGGCCACACAGATCTAAT
hCoV-19/Germany/HE-RKI-I-438397/2022_EPI_ISL_905522... (6445) AGGAGACATTATACTTAAACCAGCAAATAATA---TAAAAATTACAGAAGAGGTTGGCCACACAGATCTAAT
 hCoV-19/USA/DE-CDC-LC0472738/2021_EPI_ISL_9049423... (6409) AGGAGACATTATACTTAAACCAGCAAATAATA---TAAAAATTACAGAAGAGGTTGGCCACACAGATCTAAT

                                                                    Section 92
                                                   (6553) 6553    6560       6570       6580       6590       6600       6610       6624
              SARS-CoV-2 Reference Genome NC_045512.2 (6553) GGCTGCTTATGTAGACAATTCTAGTCTTACTATTAAGAAACCTAATGAATTATCTAGAGTATTAGGTTTGAA
hCoV-19/Botswana/R69B55_BHP_916539/2021_EPI_ISL_90... (6499) GGCTGCTTATGTAGACAATTCTAGTCTTACTATTAAGAAACCTAATGAATTATCTAGAGTATTAGGTTTGAA
 hCoV-19/India/HR-MDU-IGIB1210605800489930/2022_EPI... (6541) GGCTGCTTATGTAGACAATTCTAGTCTTACTATTAAGAAACCTAATGAATTATCTAGAGTATTAGGTTTGAA
  hCoV-19/England/PLYM-332B917/2022_EPI_ISL_9062229 ... (6500) GGCTGCTTATGTAGACAATTCTAGTCTTACTATTAAGAAACCTAATGAATTATCTAGAGTATTAGGTTTGAA
hCoV-19/Germany/HE-RKI-I-438397/2022_EPI_ISL_905522... (6514) GGCTGCTTATGTAGACAATTCTAGTCTTACTATTAAGAAACCTAATGAATTATCTAGAGTATTAGGTTTGAA
 hCoV-19/USA/DE-CDC-LC0472738/2021_EPI_ISL_9049423... (6478) GGCTGCTTATGTAGACAATTCTAGTCTTACTATTAAGAAACCTAATGAATTATCTAGAGTATTAGGTTTGAA
```

Section 93

	(6625)	6625 6630 6640 6650 6660 6670 6680 6696
SARS-CoV-2 Reference Genome NC_045512.2	(6625)	AACCCTTGCTACTCATGGTTTAGCTGCTGTTAATAGTGTCCCTTGGGATACTATAGCTAATTATGCTAAGCC
hCoV-19/Botswana/R69B55_BHP_916539/2021_EPI_ISL_90...	(6571)	AACCCTTGCTACTCATGGTTTAGCTGCTGTTAATAGTGTCCCTTGGGATACTATAGCTAATTATGCTAAGCC
hCoV-19/India/HR-MDU-IGIB1210605800489930/2022_EPI...	(6613)	AACCCTTGCTACTCATGGTTTAGCTGCTGTTAATAGTGTCCCTTGGGATACTATAGCTAATTATGCTAAGCC
hCoV-19/England/PLYM-332B917/2022_EPI_ISL_9062229_...	(6572)	AACCCTTGCTACTCATGGTTTAGCTGCTGTTAATAGTGTCCCTTGGGATACTATAGCTAATTATGCTAAGCC
hCoV-19/Germany/HE-RKI-I-438397/2022_EPI_ISL_905522...	(6586)	AACCCTTGCTACTCATGGTTTAGCTGCTGTTAATAGTGTCCCTTGGGATACTATAGCTAATTATGCTAAGCC
hCoV-19/USA/DE-CDC-LC0472738/2021_EPI_ISL_9049423...	(6550)	AACCCTTGCTACTCATGGTTTAGCTGCTGTTAATAGTGTCCCTTGGGATACTATAGCTAATTATGCTAAGCC

Section 94

	(6697)	6697 6710 6720 6730 6740 6750 6768
SARS-CoV-2 Reference Genome NC_045512.2	(6697)	TTTTCTTAACAAAGTTGTTAGTACAACTACTAACATAGTTACACGGTGTTTAAACCGTGTTTGTACTAATTA
hCoV-19/Botswana/R69B55_BHP_916539/2021_EPI_ISL_90...	(6643)	TTTTCTTAACAAAGTTGTTAGTACAACTACTAATATAGTTACACGGTGTTTAAACCGTGTTTGTACTAATTA
hCoV-19/India/HR-MDU-IGIB1210605800489930/2022_EPI...	(6685)	TTTTCTTAACAAAGTTGTTAGTACAACTACTAACATAGTTACACGGTGTTTAAACCGTGTTTGTACTAATTA
hCoV-19/England/PLYM-332B917/2022_EPI_ISL_9062229_...	(6644)	TTTTCTTAACAAAGTTGTTAGTACAACTACTAACATAGTTACACGGTGTTTAAACCGTGTTTGTACTAATTA
hCoV-19/Germany/HE-RKI-I-438397/2022_EPI_ISL_905522...	(6658)	TTTTCTTAACAAAGTTGTTAGTACAACTACTAACATAGTTACACGGTGTTTAAACCGTGTTTGTACTAATTA
hCoV-19/USA/DE-CDC-LC0472738/2021_EPI_ISL_9049423...	(6622)	TTTTCTTAACAAAGTTGTTAGTACAACTACTAACATAGTTACACGGTGTTTAAACCGTGTTTGTACTAATTA

Section 95

	(6769)	6769 6780 6790 6800 6810 6820 6830 6840
SARS-CoV-2 Reference Genome NC_045512.2	(6769)	TATGCCTTATTTCTTTACTTTATTGCTACAATTGTGTACTTTTACTAGAAGTACAAATTCTAGAATTAAAGC
hCoV-19/Botswana/R69B55_BHP_916539/2021_EPI_ISL_90...	(6715)	TATGCCTTATTTCTTTACTTTATTGCTACAATTGTGTACTTTTACTAGAAGTACAAATTCTAGAATTAAAGC
hCoV-19/India/HR-MDU-IGIB1210605800489930/2022_EPI...	(6757)	TATGCCTTATTTCTTTACTTTATTGCTACAATTGTGTACTTTTACTAGAAGTACAAATTCTAGAATTAAAGC
hCoV-19/England/PLYM-332B917/2022_EPI_ISL_9062229_...	(6716)	TATGCCTTATTTCTTTACTTTATTGCTACAATTGTGTACTTTTACTAGAAGTACAAATTCTAGAATTAAAGC
hCoV-19/Germany/HE-RKI-I-438397/2022_EPI_ISL_905522...	(6730)	TATGCCTTATTTCTTTACTTTATTGCTACAATTGTGTACTTTTACTAGAAGTACAAATTCTAGAATTAAAGC
hCoV-19/USA/DE-CDC-LC0472738/2021_EPI_ISL_9049423...	(6694)	TATGCCTTATTTCTTTACTTTATTGCTACAATTGTGTACTTTTACTAGAAGTACAAATTCTAGAATTAAAGC

Section 96

	(6841)	6841 6850 6860 6870 6880 6890 6900 6912
SARS-CoV-2 Reference Genome NC_045512.2	(6841)	ATCTATGCCGACTACTATAGCAAAGAATACTGTTAAGAGTGTCGGTAAATTTTGTCTAGAGGCTTCATTTAA
hCoV-19/Botswana/R69B55_BHP_916539/2021_EPI_ISL_90...	(6787)	ATCTATGCCGACTACTATAGCAAAGAATACTGTTAAGAGTGTCGGTAAATTTTGTCTAGAGGCTTCATTTAA
hCoV-19/India/HR-MDU-IGIB1210605800489930/2022_EPI...	(6829)	ATCTATGCCGACTACTATAGCAAAGAATACTGTTAAGAGTGTCGGTAAATTTTGTCTAGAGGCTTCATTTAA
hCoV-19/England/PLYM-332B917/2022_EPI_ISL_9062229_...	(6788)	ATCTATGCCGACTACTATAGCAAAGAATACTGTTAAGAGTGTCGGTAAATTTTGTCTAGAGGCTTCATTTAA
hCoV-19/Germany/HE-RKI-I-438397/2022_EPI_ISL_905522...	(6802)	ATCTATGCCGACTACTATAGCAAAGAATACTGTTAAGAGTGTCGGTAAATTTTGTCTAGAGGCTTCATTTAA
hCoV-19/USA/DE-CDC-LC0472738/2021_EPI_ISL_9049423...	(6766)	ATCTATGCCGACTACTATAGCAAAGAATACTGTTAAGAGTGTCGGTAAATTTTGTCTAGAGGCTTCATTTAA

Section 97

		6913 6920 6930 6940 6950 6960 6970 6984
	(6913)	
SARS-CoV-2 Reference Genome NC_045512.2	(6913)	TTATTTGAAGTCACCTAATTTTTCTAAACTGATAAATATTATAATTTGGTTTTTACTATTAAGTGTTTGCCT
hCoV-19/Botswana/R69B55_BHP_916539/2021_EPI_ISL_90...	(6859)	TTATTTGAAGTCACCTAATTTTTCTAAACTGATAAATATTATAATTTGGTTTTTACTATTAAGTGTTTGCCT
hCoV-19/India/HR-MDU-IGIB1210605800489930/2022_EPI...	(6901)	TTATTTGAAGTCACCTAATTTTTCTAAACTGATAAATATTATAATTTGGTTTTTACTATTAAGTGTTTGCCT
hCoV-19/England/PLYM-332B917/2022_EPI_ISL_9062229_...	(6860)	TTATTTGAAGTCACCTAATTTTTCTAAACTGATAAATATTATAATTTGGTTTTTACTATTAAGTGTTTGCCT
hCoV-19/Germany/HE-RKI-I-438397/2022_EPI_ISL_905522...	(6874)	TTATTTGAAGTCACCTAATTTTTCTAAACTGATAAATATTATAATTTGGTTTTTACTATTAAGTGTTTGCCT
hCoV-19/USA/DE-CDC-LC0472738/2021_EPI_ISL_9049423...	(6838)	TTATTTGAAGTCACCTAATTTTTCTAAACTGATAAATATTATAATTTGGTTTTTACTATTAAGTGTTTGCCT

Section 98

		6985 6990 7000 7010 7020 7030 7040 7056
	(6985)	
SARS-CoV-2 Reference Genome NC_045512.2	(6985)	AGGTTCTTTAATCTACTCAACCGCTGCTTTAGGTGTTTTAATGTCTAATTTAGGCATGCCTTCTTACTGTAC
hCoV-19/Botswana/R69B55_BHP_916539/2021_EPI_ISL_90...	(6931)	AGGTTCTTTAATCTACTCAACCGCTGCTTTAGGTGTTTTAATGTCTAATTTAGGCATGCCTTCTTACTGTAC
hCoV-19/India/HR-MDU-IGIB1210605800489930/2022_EPI...	(6973)	AGGTTCTTTAATCTACTCAACCGCTGCTTTAGGTGTTTTAATGTCTAATTTAGGCATGCCTTCTTACTGTAC
hCoV-19/England/PLYM-332B917/2022_EPI_ISL_9062229_...	(6932)	AGGTTCTTTAATCTACTCAACCGCTGCTTTAGGTGTTTTAATGTCTAATTTAGGCATGCCTTCTTACTGTAC
hCoV-19/Germany/HE-RKI-I-438397/2022_EPI_ISL_905522...	(6946)	AGGTTCTTTAATCTACTCAACCGCTGCTTTAGGTGTTTTAATGTCTAATTTAGGCATGCCTTCTTACTGTAC
hCoV-19/USA/DE-CDC-LC0472738/2021_EPI_ISL_9049423...	(6910)	AGGTTCTTTAATCTACTCAACCGCTGCTTTAGGTGTTTTAATGTCTAATTTAGGCATGCCTTCTTACTGTAC

Section 99

		7057 7070 7080 7090 7100 7110 7128
	(7057)	
SARS-CoV-2 Reference Genome NC_045512.2	(7057)	TGGTTACAGAGAAGGCTATTTGAACTCTACTAATGTCACTATTGCAACCTACTGTACTGGTTCTATACCTTG
hCoV-19/Botswana/R69B55_BHP_916539/2021_EPI_ISL_90...	(7003)	TGGTTACAGAGAAGGCTATTTGAACTCTACTAATGTCACTATTGCAACCTACTGTACTGGTTCTATACCTTG
hCoV-19/India/HR-MDU-IGIB1210605800489930/2022_EPI...	(7045)	TGGTTACAGAGAAGGCTATTTGAACTCTACTAATGTCACTATTGCAACCTACTGTACTGGTTCTATACCTTG
hCoV-19/England/PLYM-332B917/2022_EPI_ISL_9062229_...	(7004)	TGGTTACAGAGAAGGCTATTTGAACTCTACTAATGTCACTATTGCAACCTACTGTACTGGTTCTATACCTTG
hCoV-19/Germany/HE-RKI-I-438397/2022_EPI_ISL_905522...	(7018)	TGGTTACAGAGAAGGCTATTTGAACTCTACTAATGTCACTATTGCAACCTACTGTACTGGTTCTATACCTTG
hCoV-19/USA/DE-CDC-LC0472738/2021_EPI_ISL_9049423...	(6982)	TGGTTACAGAGAAGGCTATTTGAACTCTACTAATGTCACTATTGCAACCTACTGTACTGGTTCTATACCTTG

Section 100

		7129 7140 7150 7160 7170 7180 7190 7200
	(7129)	
SARS-CoV-2 Reference Genome NC_045512.2	(7129)	TAGTGTTTGTCTTAGTGGTTTAGATTCTTTAGACACCTATCCTTCTTTAGAAACTATACAAATTACCATTTC
hCoV-19/Botswana/R69B55_BHP_916539/2021_EPI_ISL_90...	(7075)	TAGTGTTTGTCTTAGTGGTTTAGATTCTTTAGACACCTATCCTTCTTTAGAAACTATACAAATTACCATTTC
hCoV-19/India/HR-MDU-IGIB1210605800489930/2022_EPI...	(7117)	TAGTGTTTGTCTTAGTGGTTTAGATTCTTTAGACACCTATCCTTCTTTAGAAACTATACAAATTACCATTTC
hCoV-19/England/PLYM-332B917/2022_EPI_ISL_9062229_...	(7076)	TAGTGTTTGTCTTAGTGGTTTAGATTCTTTAGACACCTATCCTTCTTTAGAAACTATACAAATTACCATTTC
hCoV-19/Germany/HE-RKI-I-438397/2022_EPI_ISL_905522...	(7090)	TAGTGTTTGTCTTAGTGGTTTAGATTCTTTAGACACCTATCCTTCTTTAGAAACTATACAAATTACCATTTC
hCoV-19/USA/DE-CDC-LC0472738/2021_EPI_ISL_9049423...	(7054)	TAGTGTTTGTCTTAGTGGTTTAGATTCTTTAGACACCTATCCTTCTTTAGAAACTATACAAATTACCATTTC

Omicron BA.1

Section 101

```
                                                              (7201) 7201     7210      7220      7230      7240      7250      7260      7272
SARS-CoV-2 Reference Genome NC_045512.2                       (7201) ATCTTTTAAATGGGATTTAACTGCTTTTGGCTTAGTTGCAGAGTGGTTTTTGGCATATATTCTTTTCACTAG
hCoV-19/Botswana/R69B55_BHP_916539/2021_EPI_ISL_90...         (7147) ATCTTTTAAATGGGATTTAACTGCTTTTGGCTTAGTTGCAGAGTGGTTTTTGGCATATATTCTTTTCACTAG
hCoV-19/India/HR-MDU-IGIB1210605800489930/2022_EPI...         (7189) ATCTTTTAAATGGGATTTAACTGCTTTTGGCTTAGTTGCAGAGTGGTTTTTGGCATATATTCTTTTCACTAG
hCoV-19/England/PLYM-332B917/2022_EPI_ISL_9062229_...         (7148) ATCTTTTAAATGGGATTTAACTGCTTTTGGCTTAGTTGCAGAGTGGTTTTTGGCATATATTCTTTTCACTAG
hCoV-19/Germany/HE-RKI-I-438397/2022_EPI_ISL_905522...        (7162) ATCTTTTAAATGGGATTTAACTGCTTTTGGCTTAGTTGCAGAGTGGTTTTTGGCATATATTCTTTTCACTAG
hCoV-19/USA/DE-CDC-LC0472738/2021_EPI_ISL_9049423...          (7126) ATCTTTTAAATGGGATTTAACTGCTTTTGGCTTAGTTGCAGAGTGGTTTTTGGCATATATTCTTTTCACTAG
```

Section 102

```
                                                              (7273) 7273   7280      7290      7300      7310      7320      7330      7344
SARS-CoV-2 Reference Genome NC_045512.2                       (7273) GTTTTTCTATGTACTTGGATTGGCTGCAATCATGCAATTGTTTTTCAGCTATTTTGCAGTACATTTTATTAG
hCoV-19/Botswana/R69B55_BHP_916539/2021_EPI_ISL_90...         (7219) GTTTTTCTATGTACTTGGATTGGCTGCAATCATGCAATTGTTTTTCAGCTATTTTGCAGTACATTTTATTAG
hCoV-19/India/HR-MDU-IGIB1210605800489930/2022_EPI...         (7261) GTTTTTCTATGTACTTGGATTGGCTGCAATCATGCAATTGTTTTTCAGCTATTTTGCAGTACATTTTATTAG
hCoV-19/England/PLYM-332B917/2022_EPI_ISL_9062229_...         (7220) GTTTTTCTATGTACTTGGATTGGCTGCAATCATGCAATTGTTTTTCAGCTATTTTGCAGTACATTTTATTAG
hCoV-19/Germany/HE-RKI-I-438397/2022_EPI_ISL_905522...        (7234) GTTTTTCTATGTACTTGGATTGGCTGCAATCATGCAATTGTTTTTCAGCTATTTTGCAGTACATTTTATTAG
hCoV-19/USA/DE-CDC-LC0472738/2021_EPI_ISL_9049423...          (7198) GTTTTTCTATGTACTTGGATTGGCTGCAATCATGCAATTGTTTTTCAGCTATTTTGCAGTACATTTTATTAG
```

Section 103

```
                                                              (7345) 7345 7350      7360      7370      7380      7390      7400          7416
SARS-CoV-2 Reference Genome NC_045512.2                       (7345) TAATTCTTGGCTTATGTGGTTAATAATTAATCTTGTACAAATGGCCCCGATTTCAGCTATGGTTAGAATGTA
hCoV-19/Botswana/R69B55_BHP_916539/2021_EPI_ISL_90...         (7291) TAATTCTTGGCTTATGTGGTTAATAATTAATCTTGTACAAATGGCCCCGATTTCAGCTATGGTTAGAATGTA
hCoV-19/India/HR-MDU-IGIB1210605800489930/2022_EPI...         (7333) TAATTCTTGGCTTATGTGGTTAATAATTAATCTTGTACAAATGGCCCTGATTTCAGCTATGGTTAGAATGTA
hCoV-19/England/PLYM-332B917/2022_EPI_ISL_9062229_...         (7292) TAATTCTTGGCTTATGTGGTTAATAATTAATCTTGTACAAATGGCCCCGATTTCAGCTATGGTTAGAATGTA
hCoV-19/Germany/HE-RKI-I-438397/2022_EPI_ISL_905522...        (7306) TAATTCTTGGCTTATGTGGTTAATAATTAATCTTGTACAAATGGCCCCGATTTCAGCTATGGTTAGAATGTA
hCoV-19/USA/DE-CDC-LC0472738/2021_EPI_ISL_9049423...          (7270) TAATTCTTGGCTTATGTGGTTAATAATTAATCTTGTACAAATGGCCCCGATTTCAGCTATGGTTAGAATGTA
```

Section 104

```
                                                              (7417) 7417         7430      7440      7450      7460      7470              7488
SARS-CoV-2 Reference Genome NC_045512.2                       (7417) CATCTTCTTTGCATCATTTTATTATGTATGGAAAAGTTATGTGCATGTTGTAGACGGTTGTAATTCATCAAC
hCoV-19/Botswana/R69B55_BHP_916539/2021_EPI_ISL_90...         (7363) CATCTTCTTTGCATCATTTTATTATGTATGGAAAAGTTATGTGCATGTTGTAGACGGTTGTAATTCATCAAC
hCoV-19/India/HR-MDU-IGIB1210605800489930/2022_EPI...         (7405) CATCTTCTTTGCATCATTTTATTATGTATGGAAAAGTTATGTGCATGTTGTAGACGGTTGTAATTCATCAAC
hCoV-19/England/PLYM-332B917/2022_EPI_ISL_9062229_...         (7364) CATCTTCTTTGCATCATTTTATTATGTATGGAAAAGTTATGTGCATGTTGTAGACGGTTGTAATTCATCAAC
hCoV-19/Germany/HE-RKI-I-438397/2022_EPI_ISL_905522...        (7378) CATCTTCTTTGCATCATTTTATTATGTATGGAAAAGTTATGTGCATGTTGTAGACGGTTGTAATTCATCAAC
hCoV-19/USA/DE-CDC-LC0472738/2021_EPI_ISL_9049423...          (7342) CATCTTCTTTGCATCATTTTATTATGTATGGAAAAGTTATGTGCATGTTGTAGACGGTTGTAATTCATCAAC
```

Section 105

		7489 7500 7510 7520 7530 7540 7550 7560
	(7489)	
SARS-CoV-2 Reference Genome NC_045512.2	(7489)	TTGTATGATGTGTTACAAACGTAATAGAGCAACAAGAGTCGAATGTACAACTATTGTTAATGGTGTTAGAAG
hCoV-19/Botswana/R69B55_BHP_916539/2021_EPI_ISL_90...	(7435)	TTGTATGATGTGTTACAAACGTAATAGAGCAACAAGAGTCGAATGTACAACTATTGTTAATGGTGTTAGAAG
hCoV-19/India/HR-MDU-IGIB1210605800489930/2022_EPI...	(7477)	TTGTATGATGTGTTACAAACGTAATAGAGCAACAAGAGTCGAATGTACAACTATTGTTAATGGTGTTAGAAG
hCoV-19/England/PLYM-332B917/2022_EPI_ISL_9062229_...	(7436)	TTGTATGATGTGTTACAAACGTAATAGAGCAACAAGAGTCGAATGTACAACTATTGTTAATGGTGTTAGAAG
hCoV-19/Germany/HE-RKI-I-438397/2022_EPI_ISL_905522...	(7450)	TTGTATGATGTGTTACAAACGTAATAGAGCAACAAGAGTCGAATGTACAACTATTGTTAATGGTGTTAGAAG
hCoV-19/USA/DE-CDC-LC0472738/2021_EPI_ISL_9049423...	(7414)	TTGTATGATGTGTTACAAACGTAATAGAGCAACAAGAGTCGAATGTACAACTATTGTTAATGGTGTTAGAAG

Section 106

		7561 7570 7580 7590 7600 7610 7620 7632
	(7561)	
SARS-CoV-2 Reference Genome NC_045512.2	(7561)	GTCCTTTTATGTCTATGCTAATGGAGGTAAAGGCTTTTGCAAACTACACAATTGGAATTGTGTTAATTGTGA
hCoV-19/Botswana/R69B55_BHP_916539/2021_EPI_ISL_90...	(7507)	GTCCTTTTATGTCTATGCTAATGGAGGTAAAGGCTTTTGCAAACTACACAATTGGAATTGTGTTAATTGTGA
hCoV-19/India/HR-MDU-IGIB1210605800489930/2022_EPI...	(7549)	GTCCTTTTATGTCTATGCTAATGGAGGTAAAGGCTTTTGCAAACTACACAATTGGAATTGTGTTAATTGTGA
hCoV-19/England/PLYM-332B917/2022_EPI_ISL_9062229_...	(7508)	GTCCTTTTATGTCTATGCTAATGGAGGTAAAGGCTTTTGCAAACTACACAATTGGAATTGTGTTAATTGTGA
hCoV-19/Germany/HE-RKI-I-438397/2022_EPI_ISL_905522...	(7522)	GTCCTTTTATGTCTATGCTAATGGAGGTAAAGGCTTTTGCAAACTACACAATTGGAATTGTGTTAATTGTGA
hCoV-19/USA/DE-CDC-LC0472738/2021_EPI_ISL_9049423...	(7486)	GTCCTTTTATGTCTATGCTAATGGAGGTAAAGGCTTTTGCAAACTACACAATTGGAATTGTGTTAATTGTGA

Section 107

		7633 7640 7650 7660 7670 7680 7690 7704
	(7633)	
SARS-CoV-2 Reference Genome NC_045512.2	(7633)	TACATTCTGTGCTGGTAGTACATTTATTAGTGATGAAGTTGCGAGAGACTTGTCACTACAGTTTAAAAGACC
hCoV-19/Botswana/R69B55_BHP_916539/2021_EPI_ISL_90...	(7579)	TACATTCTGTGCTGGTAGTACATTTATTAGTGATGAAGTTGCGAGAGACTTGTCACTACAGTTTAAAAGACC
hCoV-19/India/HR-MDU-IGIB1210605800489930/2022_EPI...	(7621)	TACATTCTGTGCTGGTAGTACATTTATTAGTGATGAAGTTGCGAGAGACTTGTCACTACAGTTTAAAAGACC
hCoV-19/England/PLYM-332B917/2022_EPI_ISL_9062229_...	(7580)	TACATTCTGTGCTGGTAGTACATTTATTAGTGATGAAGTTGCGAGAGACTTGTCACTACAGTTTAAAAGACC
hCoV-19/Germany/HE-RKI-I-438397/2022_EPI_ISL_905522...	(7594)	TACATTCTGTGCTGGTAGTACATTTATTAGTGATGAAGTTGCGAGAGACTTGTCACTACAGTTTAAAAGACC
hCoV-19/USA/DE-CDC-LC0472738/2021_EPI_ISL_9049423...	(7558)	TACATTCTGTGCTGGTAGTACATTTATTAGTGATGAAGTTGCGAGAGACTTGTCACTACAGTTTAAAAGACC

Section 108

		7705 7710 7720 7730 7740 7750 7760 7776
	(7705)	
SARS-CoV-2 Reference Genome NC_045512.2	(7705)	AATAAATCCTACTGACCAGTCTTCTTACATCGTTGATAGTGTTACAGTGAAGAATGGTTCCATCCATCTTTA
hCoV-19/Botswana/R69B55_BHP_916539/2021_EPI_ISL_90...	(7651)	AATAAATCCTACTGACCAGTCTTCTTACATCGTTGATAGTGTTACAGTGAAGAATGGTTCCATCCATCTTTA
hCoV-19/India/HR-MDU-IGIB1210605800489930/2022_EPI...	(7693)	AATAAATCCTACTGACCAGTCTTCTTACATCGTTGATAGTGTTACAGTGAAGAATGGTTCCATCCATCTTTA
hCoV-19/England/PLYM-332B917/2022_EPI_ISL_9062229_...	(7652)	AATAAATCCTACTGACCAGTCTTCTTACATCGTTGATAGTGTTACAGTGAAGAATGGTTCCATCCATCTTTA
hCoV-19/Germany/HE-RKI-I-438397/2022_EPI_ISL_905522...	(7666)	AATAAATCCTACTGACCAGTCTTCTTACATCGTTGATAGTGTTACAGTGAAGAATGGTTCCATCCATCTTTA
hCoV-19/USA/DE-CDC-LC0472738/2021_EPI_ISL_9049423...	(7630)	AATAAATCCTACTGACCAGTCTTCTTACATCGTTGATAGTGTTACAGTGAAGAATGGTTCCATCCATCTTTA

Section 109

```
                                                           (7777) 7777         7790      7800      7810      7820      7830             7848
SARS-CoV-2 Reference Genome NC_045512.2                   (7777) CTTTGATAAAGCTGGTCAAAAGACTTATGAAAGACATTCTCTCTCTCATTTTGTTAACTTAGACAACCTGAG
hCoV-19/Botswana/R69B55_BHP_916539/2021_EPI_ISL_90...      (7723) CTTTGATAAAGCTGGTCAAAAGACTTATGAAAGACATTCTCTCTCTCATTTTGTTAACTTAGACAACCTGAG
hCoV-19/India/HR-MDU-IGIB1210605800489930/2022_EPI...      (7765) CTTTGATAAAGCTGGTCAAAAGACTTATGAAAGACATTCTCTCTCTCATTTTGTTAACTTAGACAACCTGAG
hCoV-19/England/PLYM-332B917/2022_EPI_ISL_9062229 ...      (7724) CTTTGATAAAGCTGGTCAAAAGACTTATGAAAGACATTCTCTCTCTCATTTTGTTAACTTAGACAACCTGAG
hCoV-19/Germany/HE-RKI-I-438397/2022_EPI_ISL_905522...     (7738) CTTTGATAAAGCTGGTCAAAAGACTTATGAAAGACATTCTCTCTCTCATTTTGTTAACTTAGACAACCTGAG
hCoV-19/USA/DE-CDC-LC0472738/2021_EPI_ISL_9049423...       (7702) CTTTGATAAAGCTGGTCAAAAGACTTATGAAAGACATTCTCTCTCTCATTTTGTTAACTTAGACAACCTGAG
```

Section 110

```
                                                           (7849) 7849       7860      7870      7880      7890      7900      7910     7920
SARS-CoV-2 Reference Genome NC_045512.2                   (7849) AGCTAATAACACTAAAGGTTCATTGCCTATTAATGTTATAGTTTTTGATGGTAAATCAAAATGTGAAGAATC
hCoV-19/Botswana/R69B55_BHP_916539/2021_EPI_ISL_90...      (7795) AGCTAATAACACTAAAGGTTCATTGCCTATTAATGTTATAGTTTTTGATGGTAAATCAAAATGTGAAGAATC
hCoV-19/India/HR-MDU-IGIB1210605800489930/2022_EPI...      (7837) AGCTAATAACACTAAAGGTTCATTGCCTATTAATGTTATAGTTTTTGATGGTAAATCAAAATGTGAAGAATC
hCoV-19/England/PLYM-332B917/2022_EPI_ISL_9062229 ...      (7796) AGCTAATAACACTAAAGGTTCATTGCCTATTAATGTTATAGTTTTTGATGGTAAATCAAAATGTGAAGAATC
hCoV-19/Germany/HE-RKI-I-438397/2022_EPI_ISL_905522...     (7810) AGCTAATAACACTAAAGGTTCATTGCCTATTAATGTTATAGTTTTTGATGGTAAATCAAAATGTGAAGAATC
hCoV-19/USA/DE-CDC-LC0472738/2021_EPI_ISL_9049423...       (7774) AGCTAATAACACTAAAGGTTCATTGCCTATTAATGTTATAGTTTTTGATGGTAAATCAAAATGTGAAGAATC
```

Section 111

```
                                                           (7921) 7921     7930      7940      7950      7960      7970      7980       7992
SARS-CoV-2 Reference Genome NC_045512.2                   (7921) ATCTGCAAAATCAGCGTCTGTTTACTACAGTCAGCTTATGTGTCAACCTATACTGTTACTAGATCAGGCATT
hCoV-19/Botswana/R69B55_BHP_916539/2021_EPI_ISL_90...      (7867) ATCTGCAAAATCAGCGTCTGTTTACTACAGTCAGCTTATGTGTCAACCTATACTGTTACTAGATCAGGCATT
hCoV-19/India/HR-MDU-IGIB1210605800489930/2022_EPI...      (7909) ATCTGCAAAATCAGCGTCTGTTTACTACAGTCAGCTTATGTGTCAACCTATACTGTTACTAGATCAGGCATT
hCoV-19/England/PLYM-332B917/2022_EPI_ISL_9062229 ...      (7868) ATCTGCAAAATCAGCGTCTGTTTACTACAGTCAGCTTATGTGTCAACCTATACTGTTACTAGATCAGGCATT
hCoV-19/Germany/HE-RKI-I-438397/2022_EPI_ISL_905522...     (7882) ATCTGCAAAATCAGCGTCTGTTTACTACAGTCAGCTTATGTGTCAACCTATACTGTTACTAGATCAGGCATT
hCoV-19/USA/DE-CDC-LC0472738/2021_EPI_ISL_9049423...       (7846) ATCTGCAAAATCAGCGTCTGTTTACTACAGTCAGCTTATGTGTCAACCTATACTGTTACTAGATCAGGCATT
```

Section 112

```
                                                           (7993) 7993   8000      8010      8020      8030      8040      8050         8064
SARS-CoV-2 Reference Genome NC_045512.2                   (7993) AGTGTCTGATGTTGGTGATAGTGCGGAAGTTGCAGTTAAAATGTTTGATGCTTACGTTAATACGTTTTCATC
hCoV-19/Botswana/R69B55_BHP_916539/2021_EPI_ISL_90...      (7939) AGTGTCTGATGTTGGTGATAGTGCGGAAGTTGCAGTTAAAATGTTTGATGCTTACGTTAATACGTTTTCATC
hCoV-19/India/HR-MDU-IGIB1210605800489930/2022_EPI...      (7981) AGTGTCTGATGTTGGTGATAGTGCGGAAGTTGCAGTTAAAATGTTTGATGCTTACGTTAATACGTTTTCATC
hCoV-19/England/PLYM-332B917/2022_EPI_ISL_9062229 ...      (7940) AGTGTCTGATGTTGGTGATAGTGCGGAAGTTGCAGTTAAAATGTTTGATGCTTACGTTAATACGTTTTCATC
hCoV-19/Germany/HE-RKI-I-438397/2022_EPI_ISL_905522...     (7954) AGTGTCTGATGTTGGTGATAGTGCGGAAGTTGCAGTTAAAATGTTTGATGCTTACGTTAATACGTTTTCATC
hCoV-19/USA/DE-CDC-LC0472738/2021_EPI_ISL_9049423...       (7918) AGTGTCTGATGTTGGTGATAGTGCGGAAGTTGCAGTTAAAATGTTTGATGCTTACGTTAATACGTTTTCATC
```

Section 113

Sequence	Start	8065 8070 8080 8090 8100 8110 8120 8136
	(8065)	AACTTTTAACGTACCAATGGAAAAACTCAAAACACTAGTTGCAACTGCAGAAGCTGAACTTGCAAAGAATGT
SARS-CoV-2 Reference Genome NC_045512.2	(8065)	AACTTTTAACGTACCAATGGAAAAACTCAAAACACTAGTTGCAACTGCAGAAGCTGAACTTGCAAAGAATGT
hCoV-19/Botswana/R69B55_BHP_916539/2021_EPI_ISL_90...	(8011)	AACTTTTAACGTACCAATGGAAAAACTCAAAACACTAGTTGCAACTGCAGAAGCTGAACTTGCAAAGAATGT
hCoV-19/India/HR-MDU-IGIB1210605800489930/2022_EPI...	(8053)	AACTTTTAACGTACCAATGGAAAAACTCAAAACACTAGTTGCAACTGCAGAAGCTGAACTTGCAAAGAATGT
hCoV-19/England/PLYM-332B917/2022_EPI_ISL_9062229_...	(8012)	AACTTTTAACGTACCAATGGAAAAACTCAAAACACTAGTTGCAACTGCAGAAGCTGAACTTGCAAAGAATGT
hCoV-19/Germany/HE-RKI-I-438397/2022_EPI_ISL_905522...	(8026)	AACTTTTAACGTACCAATGGAAAAACTCAAAACACTAGTTGCAACTGCAGAAGCTGAACTTGCAAAGAATGT
hCoV-19/USA/DE-CDC-LC0472738/2021_EPI_ISL_9049423...	(7990)	AACTTTTAACGTACCAATGGAAAAACTCAAAACACTAGTTGCAACTGCAGAAGCTGAACTTGCAAAGAATGT

Section 114

Sequence	Start	8137 8150 8160 8170 8180 8190 8208
SARS-CoV-2 Reference Genome NC_045512.2	(8137)	GTCCTTAGACAATGTCTTATCTACTTTTATTTCAGCAGCTCGGCAAGGGTTTGTTGATTCAGATGTAGAAAC
hCoV-19/Botswana/R69B55_BHP_916539/2021_EPI_ISL_90...	(8083)	GTCCTTAGACAATGTCTTATCTACTTTTATTTCAGCAGCTCGGCAAGGGTTTGTTGATTCAGATGTAGAAAC
hCoV-19/India/HR-MDU-IGIB1210605800489930/2022_EPI...	(8125)	GTCCTTAGACAATGTCTTATCTACTTTTATTTCAGCAGCTCGGCAAGGGTTTGTTGATTCAGATGTAGAAAC
hCoV-19/England/PLYM-332B917/2022_EPI_ISL_9062229_...	(8084)	GTCCTTAGACAATGTCTTATCTACTTTTATTTCAGCAGCTCGGCAAGGGTTTGTTGATTCAGATGTAGAAAC
hCoV-19/Germany/HE-RKI-I-438397/2022_EPI_ISL_905522...	(8098)	GTCCTTAGACAATGTCTTATCTACTTTTATTTCAGCAGCTCGGCAAGGGTTTGTTGATTCAGATGTAGAAAC
hCoV-19/USA/DE-CDC-LC0472738/2021_EPI_ISL_9049423...	(8062)	GTCCTTAGACAATGTCTTATCTACTTTTATTTCAGCAGCTCGGCAAGGGTTTGTTGATTCAGATGTAGAAAC

Section 115

Sequence	Start	8209 8220 8230 8240 8250 8260 8270 8280
SARS-CoV-2 Reference Genome NC_045512.2	(8209)	TAAAGATGTTGTTGAATGTCTTAAATTGTCACATCAATCTGACATAGAAGTTACTGGCGATAGTTGTAATAA
hCoV-19/Botswana/R69B55_BHP_916539/2021_EPI_ISL_90...	(8155)	TAAAGATGTTGTTGAATGTCTTAAATTGTCACATCAATCTGACATAGAAGTTACTGGCGATAGTTGTAATAA
hCoV-19/India/HR-MDU-IGIB1210605800489930/2022_EPI...	(8197)	TAAAGATGTTGTTGAATGTCTTAAATTGTCACATCAATCTGACATAGAAGTTACTGGCGATAGTTGTAATAA
hCoV-19/England/PLYM-332B917/2022_EPI_ISL_9062229_...	(8156)	TAAAGATGTTGTTGAATGTCTTAAATTGTCACATCAATCTGACATAGAAGTTACTGGCGATAGTTGTAATAA
hCoV-19/Germany/HE-RKI-I-438397/2022_EPI_ISL_905522...	(8170)	TAAAGATGTTGTTGAATGTCTTAAATTGTCACATCAATCTGACATAGAAGTTACTGGCGATAGTTGTAATAA
hCoV-19/USA/DE-CDC-LC0472738/2021_EPI_ISL_9049423...	(8134)	TAAAGATGTTGTTGAATGTCTTAAATTGTCACATCAATCTGACATAGAAGTTACTGGCGATAGTTGTAATAA

Section 116

Sequence	Start	8281 8290 8300 8310 8320 8330 8340 8352
SARS-CoV-2 Reference Genome NC_045512.2	(8281)	CTATATGCTCACCTATAACAAAGTTGAAAACATGACACCCCGTGACCTTGGTGCTTGTATTGACTGTAGTGC
hCoV-19/Botswana/R69B55_BHP_916539/2021_EPI_ISL_90...	(8227)	CTATATGCTCACCTATAACAAAGTTGAAAACATGACACCCCGTGACCTTGGTGCTTGTATTGACTGTAGTGC
hCoV-19/India/HR-MDU-IGIB1210605800489930/2022_EPI...	(8269)	CTATATGCTCACCTATAACAAAGTTGAAAACATGACACCCCGTGACCTTGGTGCTTGTATTGACTGTAGTGC
hCoV-19/England/PLYM-332B917/2022_EPI_ISL_9062229_...	(8228)	CTATATGCTCACCTATAACAAAGTTGAAAACATGACACCCCGTGACCTTGGTGCTTGTATTGACTGTAGTGC
hCoV-19/Germany/HE-RKI-I-438397/2022_EPI_ISL_905522...	(8242)	CTATATGCTCACCTATAACAAAGTTGAAAACATGACACCCCGTGACCTTGGTGCTTGTATTGACTGTAGTGC
hCoV-19/USA/DE-CDC-LC0472738/2021_EPI_ISL_9049423...	(8206)	CTATATGCTCACCTATAACAAAGTTGAAAACATGACACCCCGTGACCTTGGTGCTTGTATTGACTGTAGTGC

Section 117

	(8353)	8353 8360 8370 8380 8390 8400 8410 8424
SARS-CoV-2 Reference Genome NC_045512.2	(8353)	GCGTCATATTAATGCGCAGGTAGCAAAAAGTCACAACATTGCTTTGATATGGAACGTTAAAGATTTCATGTC
hCoV-19/Botswana/R69B55_BHP_916539/2021_EPI_ISL_90...	(8299)	GCGTCATATTAATGCGCAGGTAGCAAAAAGTCACAACATTACTTTGATATGGAACGTTAAAGATTTCATGTC
hCoV-19/India/HR-MDU-IGIB1210605800489930/2022_EPI...	(8341)	GCGTCATATTAATGCGCAGGTAGCAAAAAGTCACAACATTACTTTGATATGGAACGTTAAAGATTTCATGTC
hCoV-19/England/PLYM-332B917/2022_EPI_ISL_9062229_...	(8300)	GCGTCATATTAATGCGCAGGTAGCAAAAAGTCACAACATTACTTTGATATGGAACGTTAAAGATTTCATGTC
hCoV-19/Germany/HE-RKI-I-438397/2022_EPI_ISL_905522...	(8314)	GCGTCATATTAATGCGCAGGTAGCAAAAAGTCACAACATTACTTTGATATGGAACGTTAAAGATTTCATGTC
hCoV-19/USA/DE-CDC-LC0472738/2021_EPI_ISL_9049423...	(8278)	GCGTCATATTAATGCGCAGGTAGCAAAAAGTCACAACATTACTTTGATATGGAACGTTAAAGATTTCATGTC

Section 118

	(8425)	8425 8430 8440 8450 8460 8470 8480 8496
SARS-CoV-2 Reference Genome NC_045512.2	(8425)	ATTGTCTGAACAACTACGAAAACAAATACGTAGTGCTGCTAAAAAGAATAACTTACCTTTTAAGTTGACATG
hCoV-19/Botswana/R69B55_BHP_916539/2021_EPI_ISL_90...	(8371)	ATTGTCTGAACAACTACGAAAACAAATACGTAGTGCTGCTAAAAAGAATAACTTACCTTTTAAGTTGACATG
hCoV-19/India/HR-MDU-IGIB1210605800489930/2022_EPI...	(8413)	ATTGTCTGAACAACTACGAAAACAAATACGTAGTGCTGCTAAAAAGAATAACTTACCTTTTAAGTTGACATG
hCoV-19/England/PLYM-332B917/2022_EPI_ISL_9062229_...	(8372)	ATTGTCTGAACAACTACGAAAACAAATACGTAGTGCTGCTAAAAAGAATAACTTACCTTTTAAGTTGACATG
hCoV-19/Germany/HE-RKI-I-438397/2022_EPI_ISL_905522...	(8386)	ATTGTCTGAACAACTACGAAAACAAATACGTAGTGCTGCTAAAAAGAATAACTTACCTTTTAAGTTGACATG
hCoV-19/USA/DE-CDC-LC0472738/2021_EPI_ISL_9049423...	(8350)	ATTGTCTGAACAACTACGAAAACAAATACGTAGTGCTGCTAAAAAGAATAACTTACCTTTTAAGTTGACATG

Section 119

	(8497)	8497 8510 8520 8530 8540 8550 8568
SARS-CoV-2 Reference Genome NC_045512.2	(8497)	TGCAACTACTAGACAAGTTGTTAATGTTGTAACAACAAAGATAGCACTTAAGGGTGGTAAAATTGTTAATAA
hCoV-19/Botswana/R69B55_BHP_916539/2021_EPI_ISL_90...	(8443)	TGCAACTACTAGACAAGTTGTTAATGTTGTAACAACAAAGATAGCACTTAAGGGTGGTAAAATTGTTAATAA
hCoV-19/India/HR-MDU-IGIB1210605800489930/2022_EPI...	(8485)	TGCAACTACTAGACAAGTTGTTAATGTTGTAACAACAAAGATAGCACTTAAGGGTGGTAAAATTGTTAATAA
hCoV-19/England/PLYM-332B917/2022_EPI_ISL_9062229_...	(8444)	TGCAACTACTAGACAAGTTGTTAATGTTGTAACAACAAAGATAGCACTTAAGGGTGGTAAAATTGTTAATAA
hCoV-19/Germany/HE-RKI-I-438397/2022_EPI_ISL_905522...	(8458)	TGCAACTACTAGACAAGTTGTTAATGTTGTAACAACAAAGATAGCACTTAAGGGTGGTAAAATTGTTAATAA
hCoV-19/USA/DE-CDC-LC0472738/2021_EPI_ISL_9049423...	(8422)	TGCAACTACTAGACAAGTTGTTAATGTTGTAACAACAAAGATAGCACTTAAGGGTGGTAAAATTGTTAATAA

Section 120

	(8569)	8569 8580 8590 8600 8610 8620 8630 8640
SARS-CoV-2 Reference Genome NC_045512.2	(8569)	TTGGTTGAAGCAGTTAATTAAAGTTACACTTGTGTTCCTTTTTGTTGCTGCTATTTTCTATTTAATAACACC
hCoV-19/Botswana/R69B55_BHP_916539/2021_EPI_ISL_90...	(8515)	TTGGTTGAAGCAGTTAATTAAAGTTACACTTGTGTTCCTTTTTGTTGCTGCTATTTTCTATTTAATAACACC
hCoV-19/India/HR-MDU-IGIB1210605800489930/2022_EPI...	(8557)	TTGGTTGAAGCAGTTAATTAAAGTTACACTTGTGTTCCTTTTTGTTGCTGCTATTTTCTATTTAATAACACC
hCoV-19/England/PLYM-332B917/2022_EPI_ISL_9062229_...	(8516)	TTGGTTGAAGCAGTTAATTAAAGTTACACTTGTGTTCCTTTTTGTTGCTGCTATTTTCTATTTAATAACACC
hCoV-19/Germany/HE-RKI-I-438397/2022_EPI_ISL_905522...	(8530)	TTGGTTGAAGCAGTTAATTAAAGTTACACTTGTGTTCCTTTTTGTTGCTGCTATTTTCTATTTAATAACACC
hCoV-19/USA/DE-CDC-LC0472738/2021_EPI_ISL_9049423...	(8494)	TTGGTTGAAGCAGTTAATTAAAGTTACACTTGTGTTCCTTTTTGTTGCTGCTATTTTCTATTTAATAACACC

Section 121

	(8641)	8641 8650 8660 8670 8680 8690 8700 8712
SARS-CoV-2 Reference Genome NC_045512.2	(8641)	TGTTCATGTCATGTCTAAACATACTGACTTTTCAAGTGAAATCATAGGATACAAGGCTATTGATGGTGGTGT
hCoV-19/Botswana/R69B55_BHP_916539/2021_EPI_ISL_90...	(8587)	TGTTCATGTCATGTCTAAACATACTGACTTTTCAAGTGAAATCATAGGATACAAGGCTATTGATGGTGGTGT
hCoV-19/India/HR-MDU-IGIB1210605800489930/2022_EPI...	(8629)	TGTTCATGTCATGTCTAAACATACTGACTTTTCAAGTGAAATCATAGGATACAAGGCTATTGATGGTGGTGT
hCoV-19/England/PLYM-332B917/2022_EPI_ISL_9062229_...	(8588)	TGTTCATGTCATGTCTAAACATACTGACTTTTCAAGTGAAATCATAGGATACAAGGCTATTGATGGTGGTGT
hCoV-19/Germany/HE-RKI-I-438397/2022_EPI_ISL_905522...	(8602)	TGTTCATGTCATGTCTAAACATACTGACTTTTCAAGTGAAATCATAGGATACAAGGCTATTGATGGTGGTGT
hCoV-19/USA/DE-CDC-LC0472738/2021_EPI_ISL_9049423...	(8566)	TGTTCATGTCATGTCTAAACATACTGACTTTTCAAGTGAAATCATAGGATACAAGGCTATTGATGGTGGTGT

Section 122

	(8713)	8713 8720 8730 8740 8750 8760 8770 8784
SARS-CoV-2 Reference Genome NC_045512.2	(8713)	CACTCGTGACATAGCATCTACAGATACTTGTTTTGCTAACAAACATGCTGATTTTGACACATGGTTTAGCCA
hCoV-19/Botswana/R69B55_BHP_916539/2021_EPI_ISL_90...	(8659)	CACTCGTGACATAGCATCTACAGATACTTGTTTTGCTAACAAACATGCTGATTTTGACACATGGTTTAGCCA
hCoV-19/India/HR-MDU-IGIB1210605800489930/2022_EPI...	(8701)	CACTCGTGACATAGCATCTACAGATACTTGTTTTGCTAACAAACATGCTGATTTTGACACATGGTTTAGCCA
hCoV-19/England/PLYM-332B917/2022_EPI_ISL_9062229_...	(8660)	CACTCGTGACATAGCATCTACAGATACTTGTTTTGCTAACAAACATGCTGATTTTGACACATGGTTTAGCCA
hCoV-19/Germany/HE-RKI-I-438397/2022_EPI_ISL_905522...	(8674)	CACTCGTGACATAGCATCTACAGATACTTGTTTTGCTAACAAACATGCTGATTTTGACACATGGTTTAGCCA
hCoV-19/USA/DE-CDC-LC0472738/2021_EPI_ISL_9049423...	(8638)	CACTCGTGACATAGCATCTACAGATACTTGTTTTGCTAACAAACATGCTGATTTTGACACATGGTTTAGCCA

Section 123

	(8785)	8785 8790 8800 8810 8820 8830 8840 8856
SARS-CoV-2 Reference Genome NC_045512.2	(8785)	GCGTGGTGGTAGTTATACTAATGACAAAGCTTGCCCATTGATTGCTGCAGTCATAACAAGAGAAGTGGGTTT
hCoV-19/Botswana/R69B55_BHP_916539/2021_EPI_ISL_90...	(8731)	GCGTGGTGGTAGTTATACTAATGACAAAGCTTGCCCATTGATTGCTGCAGTCATAACAAGAGAAGTGGGTTT
hCoV-19/India/HR-MDU-IGIB1210605800489930/2022_EPI...	(8773)	GCGTGGTGGTAGTTATACTAATGACAAAGCTTGCCCATTGATTGCTGCAGTCATAACAAGAGAAGTGGGTTT
hCoV-19/England/PLYM-332B917/2022_EPI_ISL_9062229_...	(8732)	GCGTGGTGGTAGTTATACTAATGACAAAGCTTGCCCATTGATTGCTGCAGTCATAACAAGAGAAGTGGGTTT
hCoV-19/Germany/HE-RKI-I-438397/2022_EPI_ISL_905522...	(8746)	GCGTGGTGGTAGTTATACTAATGACAAAGCTTGCCCATTGATTGCTGCAGTCATAACAAGAGAAGTGGGTTT
hCoV-19/USA/DE-CDC-LC0472738/2021_EPI_ISL_9049423...	(8710)	GCGTGGTGGTAGTTATACTAATGACAAAGCTTGCCCATTGATTGCTGCAGTCATAACAAGAGAAGTGGGTTT

Section 124

	(8857)	8857 8870 8880 8890 8900 8910 8928
SARS-CoV-2 Reference Genome NC_045512.2	(8857)	TGTCGTGCCTGGTTTGCCTGGCACGATATTACGCACAACTAATGGTGACTTTTTGCATTTCTTACCTAGAGT
hCoV-19/Botswana/R69B55_BHP_916539/2021_EPI_ISL_90...	(8803)	TGTCGTGCCTGGTTTGCCTGGCACGATATTACGCACAACTAATGGTGACTTTTTGCATTTCTTACCTAGAGT
hCoV-19/India/HR-MDU-IGIB1210605800489930/2022_EPI...	(8845)	TGTCGTGCCTGGTTTGCCTGGCACGATATTACGCACAACTAATGGTGACTTTTTGCATTTCTTACCTAGAGT
hCoV-19/England/PLYM-332B917/2022_EPI_ISL_9062229_...	(8804)	TGTCGTGCCTGGTTTGCCTGGCACGATATTACGCACAACTAATGGTGACTTTTTGCATTTCTTACCTAGAGT
hCoV-19/Germany/HE-RKI-I-438397/2022_EPI_ISL_905522...	(8818)	TGTCGTGCCTGGTTTGCCTGGCACGATATTACGCACAACTAATGGTGACTTTTTGCATTTCTTACCTAGAGT
hCoV-19/USA/DE-CDC-LC0472738/2021_EPI_ISL_9049423...	(8782)	TGTCGTGCCTGGTTTGCCTGGCACGATATTACGCACAACTAATGGTGACTTTTTGCATTTCTTACCTAGAGT

Section 125

		8929 8940 8950 8960 8970 8980 8990 9000
	(8929)	
SARS-CoV-2 Reference Genome NC_045512.2	(8929)	TTTTAGTGCAGTTGGTAACATCTGTTACACACCATCAAAACTTATAGAGTACACTGACTTTGCAACATCAGC
hCoV-19/Botswana/R69B55_BHP_916539/2021_EPI_ISL_90...	(8875)	TTTTAGTGCAGTTGGTAACATCTGTTACACACCATCAAAACTTATAGAGTACACTGACTTTGCAACATCAGC
hCoV-19/India/HR-MDU-IGIB1210605800489930/2022_EPI...	(8917)	TTTTAGTGCAGTTGGTAACATCTGTTACACACCATCAAAACTTATAGAGTACACTGACTTTGCAACATCAGC
hCoV-19/England/PLYM-332B917/2022_EPI_ISL_9062229_...	(8876)	TTTTAGTGCAGTTGGTAACATCTGTTACACACCATCAAAACTTATAGAGTACACTGACTTTGCAACATCAGC
hCoV-19/Germany/HE-RKI-I-438397/2022_EPI_ISL_905522...	(8890)	TTTTAGTGCAGTTGGTAACATCTGTTACACACCATCAAAACTTATAGAGTACACTGACTTTGCAACATCAGC
hCoV-19/USA/DE-CDC-LC0472738/2021_EPI_ISL_9049423...	(8854)	TTTTAGTGCAGTTGGTAACATCTGTTACACACCATCAAAACTTATAGAGTACACTGACTTTGCAACATCAGC

Section 126

		9001 9010 9020 9030 9040 9050 9060 9072
	(9001)	
SARS-CoV-2 Reference Genome NC_045512.2	(9001)	TTGTGTTTTGGCTGCTGAATGTACAATTTTTAAAGATGCTTCTGGTAAGCCAGTACCATATTGTTATGATAC
hCoV-19/Botswana/R69B55_BHP_916539/2021_EPI_ISL_90...	(8947)	TTGTGTTTTGGCTGCTGAATGTACAATTTTTAAAGATGCTTCTGGTAAGCCAGTACCATATTGTTATGATAC
hCoV-19/India/HR-MDU-IGIB1210605800489930/2022_EPI...	(8989)	TTGTGTTTTGGCTGCTGAATGTACAATTTTTAAAGATGCTTCTGGTAAGCCAGTACCATATTGTTATGATAC
hCoV-19/England/PLYM-332B917/2022_EPI_ISL_9062229_...	(8948)	TTGTGTTTTGGCTGCTGAATGTACAATTTTTAAAGATGCTTCTGGTAAGCCAGTACCATATTGTTATGATAC
hCoV-19/Germany/HE-RKI-I-438397/2022_EPI_ISL_905522...	(8962)	TTGTGTTTTGGCTGCTGAATGTACAATTTTTAAAGATGCTTCTGGTAAGCCAGTACCATATTGTTATGATAC
hCoV-19/USA/DE-CDC-LC0472738/2021_EPI_ISL_9049423...	(8926)	TTGTGTTTTGGCTGCTGAATGTACAATTTTTAAAGATGCTTCTGGTAAGCCAGTACCATATTGTTATGATAC

Section 127

		9073 9080 9090 9100 9110 9120 9130 9144
	(9073)	
SARS-CoV-2 Reference Genome NC_045512.2	(9073)	CAATGTACTAGAAGGTTCTGTTGCTTATGAAAGTTTACGCCCTGACACACGTTATGTGCTCATGGATGGCTC
hCoV-19/Botswana/R69B55_BHP_916539/2021_EPI_ISL_90...	(9019)	CAATGTACTAGAAGGTTCTGTTGCTTATGAAAGTTTACGCCCTGACACACGTTATGTGCTCATGGATGGCTC
hCoV-19/India/HR-MDU-IGIB1210605800489930/2022_EPI...	(9061)	CAATGTACTAGAAGGTTCTGTTGCTTATGAAAGTTTACGCCCTGACACACGTTATGTGCTCATGGATGGCTC
hCoV-19/England/PLYM-332B917/2022_EPI_ISL_9062229_...	(9020)	CAATGTACTAGAAGGTTCTGTTGCTTATGAAAGTTTACGCCCTGACACACGTTATGTGCTCATGGATGGCTC
hCoV-19/Germany/HE-RKI-I-438397/2022_EPI_ISL_905522...	(9034)	CAATGTACTAGAAGGTTCTGTTGCTTATGAAAGTTTACGCCCTGACACACGTTATGTGCTCATGGATGGCTC
hCoV-19/USA/DE-CDC-LC0472738/2021_EPI_ISL_9049423...	(8998)	CAATGTACTAGAAGGTTCTGTTGCTTATGAAAGTTTACGCCCTGACACACGTTATGTGCTCATGGATGGCTC

Section 128

		9145 9150 9160 9170 9180 9190 9200 9216
	(9145)	
SARS-CoV-2 Reference Genome NC_045512.2	(9145)	TATTATTCAATTTCCTAACACCTACCTTGAAGGTTCTGTTAGAGTGGTAACAACTTTTGATTCTGAGTACTG
hCoV-19/Botswana/R69B55_BHP_916539/2021_EPI_ISL_90...	(9091)	TATTATTCAATTTCCTAACACCTACCTTGAAGGTTCTGTTAGAGTGGTAACAACTTTTGATTCTGAGTACTG
hCoV-19/India/HR-MDU-IGIB1210605800489930/2022_EPI...	(9133)	TATTATTCAATTTCCTAACACCTACCTTGAAGGTTCTGTTAGAGTGGTAACAACTTTTGATTCTGAGTACTG
hCoV-19/England/PLYM-332B917/2022_EPI_ISL_9062229_...	(9092)	TATTATTCAATTTCCTAACACCTACCTTGAAGGTTCTGTTAGAGTGGTAACAACTTTTGATTCTGAGTACTG
hCoV-19/Germany/HE-RKI-I-438397/2022_EPI_ISL_905522...	(9106)	TATTATTCAATTTCCTAACACCTACCTTGAAGGTTCTGTTAGAGTGGTAACAACTTTTGATTCTGAGTACTG
hCoV-19/USA/DE-CDC-LC0472738/2021_EPI_ISL_9049423...	(9070)	TATTATTCAATTTCCTAACACCTACCTTGAAGGTTCTGTTAGAGTGGTAACAACTTTTGATTCTGAGTACTG

Section 129

		9217 … 9230 … 9240 … 9250 … 9260 … 9270 … 9288
	(9217)	
SARS-CoV-2 Reference Genome NC_045512.2	(9217)	TAGGCACGGCACTTGTGAAAGATCAGAAGCTGGTGTTTGTGTATCTACTAGTGGTAGATGGGTACTTAACAA
hCoV-19/Botswana/R69B55_BHP_916539/2021_EPI_ISL_90...	(9163)	TAGGCACGGCACTTGTGAAAGATCAGAAGCTGGTGTTTGTGTATCTACTAGTGGTAGATGGGTACTTAACAA
hCoV-19/India/HR-MDU-IGIB1210605800489930/2022_EPI...	(9205)	TAGGCACGGCACTTGTGAAAGATCAGAAGCTGGTGTTTGTGTATCTACTAGTGGTAGATGGGTACTTAACAA
hCoV-19/England/PLYM-332B917/2022_EPI_ISL_9062229 ...	(9164)	TAGGCACGGCACTTGTGAAAGATCAGAAGCTGGTGTTTGTGTATCTACTAGTGGTAGATGGGTACTTAACAA
hCoV-19/Germany/HE-RKI-I-438397/2022_EPI_ISL_905522...	(9178)	TAGGCACGGCACTTGTGAAAGATCAGAAGCTGGTGTTTGTGTATCTACTAGTGGTAGATGGGTACTTAACAA
hCoV-19/USA/DE-CDC-LC0472738/2021_EPI_ISL_9049423...	(9142)	TAGGCACGGCACTTGTGAAAGATCAGAAGCTGGTGTTTGTGTATCTACTAGTGGTAGATGGGTACTTAACAA

Section 130

		9289 … 9300 … 9310 … 9320 … 9330 … 9340 … 9350 … 9360
	(9289)	
SARS-CoV-2 Reference Genome NC_045512.2	(9289)	TGATTATTACAGATCTTTACCAGGAGTTTTCTGTGGTGTAGATGCTGTAAATTTACTTACTAATATGTTTAC
hCoV-19/Botswana/R69B55_BHP_916539/2021_EPI_ISL_90...	(9235)	TGATTATTACAGATCTTTACCAGGAGTTTTCTGTGGTGTAGATGCTGTAAATTTACTTACTAATATGTTTAC
hCoV-19/India/HR-MDU-IGIB1210605800489930/2022_EPI...	(9277)	TGATTATTACAGATCTTTACCAGGAGTTTTCTGTGGTGTAGATGCTGTAAATTTACTTACTAATATGTTTAC
hCoV-19/England/PLYM-332B917/2022_EPI_ISL_9062229 ...	(9236)	TGATTATTACAGATCTTTACCAGGAGTTTTCTGTGGTGTAGATGCTGTAAATTTACTTACTAATATGTTTAC
hCoV-19/Germany/HE-RKI-I-438397/2022_EPI_ISL_905522...	(9250)	TGATTATTACAGATCTTTACCAGGAGTTTTCTGTGGTGTAGATGCTGTAAATTTATTTACTAATATGTTTAC
hCoV-19/USA/DE-CDC-LC0472738/2021_EPI_ISL_9049423...	(9214)	TGATTATTACAGATCTTTACCAGGAGTTTTTTGTGGTGTAGATGCTGTAAATTTACTTACTAATATGTTTAC

Section 131

		9361 … 9370 … 9380 … 9390 … 9400 … 9410 … 9420 … 9432
	(9361)	
SARS-CoV-2 Reference Genome NC_045512.2	(9361)	ACCACTAATTCAACCTATTGGTGCTTTGGACATATCAGCATCTATAGTAGCTGGTGGTATTGTAGCTATCGT
hCoV-19/Botswana/R69B55_BHP_916539/2021_EPI_ISL_90...	(9307)	ACCACTAATTCAACCTATTGGTGCTTTGGACATATCAGCATCTATAGTAGCTGGTGGTATTGTAGCTATCGT
hCoV-19/India/HR-MDU-IGIB1210605800489930/2022_EPI...	(9349)	ACCACTAATTCAACCTATTGGTGCTTTGGACATATCAGCATCTATAGTAGCTGGTGGTATTGTAGCTATCGT
hCoV-19/England/PLYM-332B917/2022_EPI_ISL_9062229 ...	(9308)	ACCACTAATTCAACCTATTGGTGCTTTGGACATATCAGCATCTATAGTAGCTGGTGGTATTGTAGCTATCGT
hCoV-19/Germany/HE-RKI-I-438397/2022_EPI_ISL_905522...	(9322)	ACCACTAATTCAACCTATTGGTGCTTTGGACATATCAGCATCTATAGTAGCTGGTGGTATTGTAGCTATCGT
hCoV-19/USA/DE-CDC-LC0472738/2021_EPI_ISL_9049423...	(9286)	ACCACTAATTCAACCTATTGGTGCTTTGGACATATCAGCATCTATAGTAGCTGGTGGTATTGTAGCTATCGT

Section 132

		9433 … 9440 … 9450 … 9460 … 9470 … 9480 … 9490 … 9504
	(9433)	
SARS-CoV-2 Reference Genome NC_045512.2	(9433)	AGTAACATGCCTTGCCTACTATTTTATGAGGTTTAGAAGAGCTTTTGGTGAATACAGTCATGTAGTTGCCTT
hCoV-19/Botswana/R69B55_BHP_916539/2021_EPI_ISL_90...	(9379)	AGTAACATGCCTTGCCTACTATTTTATGAGGTTTAGAAGAGCTTTTGGTGAATACAGTCATGTAGTTGCCTT
hCoV-19/India/HR-MDU-IGIB1210605800489930/2022_EPI...	(9421)	AGTAACATGCCTTGCCTACTATTTTATGAGGTTTAGAAGAGCTTTTGGTGAATACAGTCATGTAGTTGCCTT
hCoV-19/England/PLYM-332B917/2022_EPI_ISL_9062229 ...	(9380)	AGTAACATGCCTTGCCTACTATTTTATGAGGTTTAGAAGAGCTTTTGGTGAATACAGTCATGTAGTTGCCTT
hCoV-19/Germany/HE-RKI-I-438397/2022_EPI_ISL_905522...	(9394)	AGTAACATGCCTTGCCTACTATTTTATGAGGTTTAGAAGAGCTTTTGGTGAATACAGTCATGTAGTTGCCTT
hCoV-19/USA/DE-CDC-LC0472738/2021_EPI_ISL_9049423...	(9358)	AGTAACATGCCTTGCCTACTATTTTATGAGGTTTAGAAGAGCTTTTGGTGAATACAGTCATGTAGTTGCCTT

Section 133

		9505 9510 9520 9530 9540 9550 9560 9576
	(9505)	
SARS-CoV-2 Reference Genome NC_045512.2	(9505)	TAATACTTTACTATTCCTTATGTCATTCACTGTACTCTGTTTAACACCAGTTTACTCATTCTTACCTGGTGT
hCoV-19/Botswana/R69B55_BHP_916539/2021_EPI_ISL_90...	(9451)	TAATACTTTACTATTCCTTATGTCATTCACTGTACTCTGTTTAACACCAGTTTACTCATTCTTACCTGGTGT
hCoV-19/India/HR-MDU-IGIB1210605800489930/2022_EPI...	(9493)	TAATACTTTACTATTCCTTATGTCATTCACTGTACTCTGTTTAACACCAGTTTACTCATTCTTACCTGGTGT
hCoV-19/England/PLYM-332B917/2022_EPI_ISL_9062229_...	(9452)	TAATACTTTACTATTCCTTATGTCATTCACTGTACTCTGTTTAACACCAGTTTACTCATTCTTACCTGGTGT
hCoV-19/Germany/HE-RKI-I-438397/2022_EPI_ISL_905522...	(9466)	TAATACTTTACTATTCCTTATGTCATTCACTGTACTCTGTTTAACACCAGTTTACTCATTCTTACCTGGTGT
hCoV-19/USA/DE-CDC-LC0472738/2021_EPI_ISL_9049423...	(9430)	TAATACTTTACTATTCCTTATGTCATTCACTGTACTCTGTTTAACACCAGTTTACTCATTCTTACCTGGTGT

Section 134

		9577 9590 9600 9610 9620 9630 9648
	(9577)	
SARS-CoV-2 Reference Genome NC_045512.2	(9577)	TTATTCTGTTATTTACTTGTACTTGACATTTTATCTTACTAATGATGTTTCTTTTTTAGCACATATTCAGTG
hCoV-19/Botswana/R69B55_BHP_916539/2021_EPI_ISL_90...	(9523)	TTATTCTGTTATTTACTTGTACTTGACATTTTATCTTACTAATGATGTTTCTTTTTTAGCACATATTCAGTG
hCoV-19/India/HR-MDU-IGIB1210605800489930/2022_EPI...	(9565)	TTATTCTGTTATTTACTTGTACTTGACATTTTATCTTACTAATGATGTTTCTTTTTTAGCACATATTCAGTG
hCoV-19/England/PLYM-332B917/2022_EPI_ISL_9062229_...	(9524)	TTATTCTGTTATTTACTTGTACTTGACATTTTATCTTACTAATGATGTTTCTTTTTTAGCACATATTCAGTG
hCoV-19/Germany/HE-RKI-I-438397/2022_EPI_ISL_905522...	(9538)	TTATTCTGTTATTTACTTGTACTTGACATTTTATCTTACTAATGATGTTTCTTTTTTAGCACATATTCAGTG
hCoV-19/USA/DE-CDC-LC0472738/2021_EPI_ISL_9049423...	(9502)	TTATTCTGTTATTTACTTGTACTTGACATTTTATCTTACTAATGATGTTTCTTTTTTAGCACATATTCAGTG

Section 135

		9649 9660 9670 9680 9690 9700 9710 9720
	(9649)	
SARS-CoV-2 Reference Genome NC_045512.2	(9649)	GATGGTTATGTTCACACCTTTAGTACCTTTCTGGATAACAATTGCTTATATCATTTGTATTTCCACAAAGCA
hCoV-19/Botswana/R69B55_BHP_916539/2021_EPI_ISL_90...	(9595)	GATGGTTATGTTCACACCTTTAGTACCTTTCTGGATAACAATTGCTTATATCATTTGTATTTCCACAAAGCA
hCoV-19/India/HR-MDU-IGIB1210605800489930/2022_EPI...	(9637)	GATGGTTATGTTCACACCTTTAGTACCTTTCTGGATAACAATTGCTTATATCATTTGTATTTCCACAAAGCA
hCoV-19/England/PLYM-332B917/2022_EPI_ISL_9062229_...	(9596)	GATGGTTATGTTCACACCTTTAGTACCTTTCTGGATAACAATTGCTTATATCATTTGTATTTCCACAAAGCA
hCoV-19/Germany/HE-RKI-I-438397/2022_EPI_ISL_905522...	(9610)	GATGGTTATGTTCACACCTTTAGTACCTTTCTGGATAACAATTGCTTATATCATTTGTATTTCCACAAAGCA
hCoV-19/USA/DE-CDC-LC0472738/2021_EPI_ISL_9049423...	(9574)	GATGGTTATGTTCACACCTTTAGTACCTTTCTGGATAACAATTGCTTATATCATTTGTATTTCCACAAAGCA

Section 136

		9721 9730 9740 9750 9760 9770 9780 9792
	(9721)	
SARS-CoV-2 Reference Genome NC_045512.2	(9721)	TTTCTATTGGTTCTTTAGTAATTACCTAAAGAGACGTGTAGTCTTTAATGGTGTTTCCTTTAGTACTTTTGA
hCoV-19/Botswana/R69B55_BHP_916539/2021_EPI_ISL_90...	(9667)	TTTCTATTGGTTCTTTAGTAATTACCTAAAGAGACGTGTAGTCTTTAATGGTGTTTCCTTTAGTACTTTTGA
hCoV-19/India/HR-MDU-IGIB1210605800489930/2022_EPI...	(9709)	TTTCTATTGGTTCTTTAGTAATTACCTAAAGAGACGTGTAGTCTTTAATGGTGTTTCCTTTAGTACTTTTGA
hCoV-19/England/PLYM-332B917/2022_EPI_ISL_9062229_...	(9668)	TTTCTATTGGTTCTTTAGTAATTACCTAAAGAGACGTGTAGTCTTTAATGGTGTTTCCTTTAGTACTTTTGA
hCoV-19/Germany/HE-RKI-I-438397/2022_EPI_ISL_905522...	(9682)	TTTCTATTGGTTCTTTAGTAATTACCTAAAGAGACGTGTAGTCTTTAATGGTGTTTCCTTTAGTACTTTTGA
hCoV-19/USA/DE-CDC-LC0472738/2021_EPI_ISL_9049423...	(9646)	TTTCTATTGGTTCTTTAGTAATTACCTAAAGAGACGTGTAGTCTTTAATGGTGTTTCCTTTAGTACTTTTGA

Omicron BA.1

```
                                                                                                                                      Section 137
                                                              (9793) 9793   9800      9810      9820      9830      9840      9850          9864
                   SARS-CoV-2 Reference Genome NC_045512.2 (9793) AGAAGCTGCGCTGTGCACCTTTTTGTTAAATAAAGAAATGTATCTAAAGTTGCGTAGTGATGTGCTATTACC
hCoV-19/Botswana/R69B55_BHP_916539/2021_EPI_ISL_90...       (9739) AGAAGCTGCGCTGTGCACCTTTTTGTTAAATAAAGAAATGTATCTAAAGTTGCGTAGTGATGTGCTATTACC
hCoV-19/India/HR-MDU-IGIB1210605800489930/2022_EPI...       (9781) AGAAGCTGCGCTGTGCACCTTTTTGTTAAATAAAGAAATGTATCTAAAGTTGCGTAGTGATGTGCTATTACC
hCoV-19/England/PLYM-332B917/2022_EPI_ISL_9062229 ...       (9740) AGAAGCTGCGCTGTGCACCTTTTTGTTAAATAAAGAAATGTATCTAAAGTTGCGTAGTGATGTGCTATTACC
hCoV-19/Germany/HE-RKI-I-438397/2022_EPI_ISL_905522...      (9754) AGAAGCTGCGCTGTGCACCTTTTTGTTAAATAAAGAAATGTATCTAAAGTTGCGTAGTGATGTGCTATTACC
hCoV-19/USA/DE-CDC-LC0472738/2021_EPI_ISL_9049423...        (9718) AGAAGCTGCGCTGTGCACCTTTTTGTTAAATAAAGAAATGTATCTAAAGTTGCGTAGTGATGTGCTATTACC
                                                                                                                                      Section 138
                                                              (9865) 9865 9870      9880      9890      9900      9910      9920            9936
                   SARS-CoV-2 Reference Genome NC_045512.2 (9865) TCTTACGCAATATAATAGATACTTAGCTCTTTATAATAAGTACAAGTATTTTAGTGGAGCAATGGATACAAC
hCoV-19/Botswana/R69B55_BHP_916539/2021_EPI_ISL_90...       (9811) TCTTACGCAATATAATAGATACTTAGCTCTTTATAATAAGTACAAGTATTTTAGTGGAGCAATGGATACAAC
hCoV-19/India/HR-MDU-IGIB1210605800489930/2022_EPI...       (9853) TCTTACGCAATATAATAGATACTTAGCTCTTTATAATAAGTACAAGTATTTTAGTGGAGCAATGGATACAAC
hCoV-19/England/PLYM-332B917/2022_EPI_ISL_9062229 ...       (9812) TCTTACGCAATATAATAGATACTTAGCTCTTTATAATAAGTACAAGTATTTTAGTGGAGCAATGGATACAAC
hCoV-19/Germany/HE-RKI-I-438397/2022_EPI_ISL_905522...      (9826) TCTTACGCAATATAATAGATACTTAGCTCTTTATAATAAGTACAAGTATTTTAGTGGAGCAATGGATACAAC
hCoV-19/USA/DE-CDC-LC0472738/2021_EPI_ISL_9049423...        (9790) TCTTACGCAATATAATAGATACTTAGCTCTTTATAATAAGTACAAGTATTTTAGTGGAGCAATGGATACAAC
                                                                                                                                      Section 139
                                                              (9937) 9937         9950      9960      9970      9980      9990           10008
                   SARS-CoV-2 Reference Genome NC_045512.2 (9937) TAGCTACAGAGAAGCTGCTTGTTGTCATCTCGCAAAGGCTCTCAATGACTTCAGTAACTCAGGTTCTGATGT
hCoV-19/Botswana/R69B55_BHP_916539/2021_EPI_ISL_90...       (9883) TAGCTACAGAGAAGCTGCTTGTTGTCATCTCGCAAAGGCTCTCAATGACTTCAGTAACTCAGGTTCTGATGT
hCoV-19/India/HR-MDU-IGIB1210605800489930/2022_EPI...       (9925) TAGCTACAGAGAAGCTGCTTGTTGTCATCTCGCAAAGGCTCTCAATGACTTCAGTAACTCAGGTTCTGATGT
hCoV-19/England/PLYM-332B917/2022_EPI_ISL_9062229 ...       (9884) TAGCTACAGAGAAGCTGCTTGTTGTCATCTCGCAAAGGCTCTCAATGACTTCAGTAACTCAGGTTCTGATGT
hCoV-19/Germany/HE-RKI-I-438397/2022_EPI_ISL_905522...      (9898) TAGCTACAGAGAAGCTGCTTGTTGTCATCTCGCAAAGGCTCTCAATGACTTCAGTAACTCAGGTTCTGATGT
hCoV-19/USA/DE-CDC-LC0472738/2021_EPI_ISL_9049423...        (9862) TAGCTACAGAGAAGCTGCTTGTTGTCATCTCGCAAAGGCTCTCAATGACTTCAGTAACTCAGGTTCTGATGT
                                                                                                                                      Section 140
                                                             (10009) 10009      10020     10030     10040     10050     10060     10070  10080
                   SARS-CoV-2 Reference Genome NC_045512.2 (10009) TCTTTACCAACCACCACAAACCTCTATCACCTCAGCTGTTTTGCAGAGTGGTTTTAGAAAAATGGCATTCCC
hCoV-19/Botswana/R69B55_BHP_916539/2021_EPI_ISL_90...       (9955) TCTTTACCAACCACCACAAATCTCTATCACCTCAGCTGTTTTGCAGAGTGGTTTTAGAAAAATGGCATTCCC
hCoV-19/India/HR-MDU-IGIB1210605800489930/2022_EPI...       (9997) TCTTTACCAACCACCACAAATCTCTATCACCTCAGCTGTTTTGCAGAGTGGTTTTAGAAAAATGGCATTCCC
hCoV-19/England/PLYM-332B917/2022_EPI_ISL_9062229 ...       (9956) TCTTTACCAACCACCACAAATCTCTATCACCTCAGCTGTTTTGCAGAGTGGTTTTAGAAAAATGGCATTCCC
hCoV-19/Germany/HE-RKI-I-438397/2022_EPI_ISL_905522...      (9970) TCTTTACCAACCACCACAAATCTCTATCACCTCAGCTGTTTTGCAGAGTGGTTTTAGAAAAATGGCATTCCC
hCoV-19/USA/DE-CDC-LC0472738/2021_EPI_ISL_9049423...        (9934) TCTTTACCAACCACCACAAATCTCTATCACCTCAGCTGTTTTGCAGAGTGGTTTTAGAAAAATGGCATTCCC
```

Section 141

(10081)	10081 … 10090 … 10100 … 10110 … 10120 … 10130 … 10140 … 10152
SARS-CoV-2 Reference Genome NC_045512.2 (10081)	ATCTGGTAAAGTTGAGGGTTGTATGGTACAAGTAACTTGTGGTACAACTACACTTAACGGTCTTTGGCTTGA
hCoV-19/Botswana/R69B55_BHP_916539/2021_EPI_ISL_90... (10027)	ATCTGGTAAAGTTGAGGGTTGTATGGTACAAGTAACTTGTGGTACAACTACACTTAACGGTCTTTGGCTTGA
hCoV-19/India/HR-MDU-IGIB1210605800489930/2022_EPI... (10069)	ATCTGGTAAAGTTGAGGGTTGTATGGTACAAGTAACTTGTGGTACAACTACACTTAACGGTCTTTGGCTTGA
hCoV-19/England/PLYM-332B917/2022_EPI_ISL_9062229_... (10028)	ATCTGGTAAAGTTGAGGGTTGTATGGTACAAGTAACTTGTGGTACAACTACACTTAACGGTCTTTGGCTTGA
hCoV-19/Germany/HE-RKI-I-438397/2022_EPI_ISL_905522... (10042)	ATCTGGTAAAGTTGAGGGTTGTATGGTACAAGTAACTTGTGGTACAACTACACTTAACGGTCTTTGGCTTGA
hCoV-19/USA/DE-CDC-LC0472738/2021_EPI_ISL_9049423... (10006)	ATCTGGTAAAGTTGAGGGTTGTATGGTACAAGTAACTTGTGGTACAACTACACTCAACGGTCTTTGGCTTGA

Section 142

(10153)	10153 … 10160 … 10170 … 10180 … 10190 … 10200 … 10210 … 10224
SARS-CoV-2 Reference Genome NC_045512.2 (10153)	TGACGTAGTTTACTGTCCAAGACATGTGATCTGCACCTCTGAAGACATGCTTAACCCTAATTATGAAGATTT
hCoV-19/Botswana/R69B55_BHP_916539/2021_EPI_ISL_90... (10099)	TGACGTAGTTTACTGTCCAAGACATGTGATCTGCACCTCTGAAGACATGCTTAACCCTAATTATGAAGATTT
hCoV-19/India/HR-MDU-IGIB1210605800489930/2022_EPI... (10141)	TGACGTAGTTTACTGTCCAAGACATGTGATCTGCACCTCTGAAGACATGCTTAACCCTAATTATGAAGATTT
hCoV-19/England/PLYM-332B917/2022_EPI_ISL_9062229_... (10100)	TGACGTAGTTTACTGTCCAAGACATGTGATCTGCACCTCTGAAGACATGCTTAACCCTAATTATGAAGATTT
hCoV-19/Germany/HE-RKI-I-438397/2022_EPI_ISL_905522... (10114)	TGACGTAGTTTACTGTCCAAGACATGTGATCTGCACCTCTGAAGACATGCTTAACCCTAATTATGAAGATTT
hCoV-19/USA/DE-CDC-LC0472738/2021_EPI_ISL_9049423... (10078)	TGACGTAGTTTACTGTCCAAGACATGTGATCTGCACCTCTGAAGACATGCTTAACCCTAATTATGAAGATTT

Section 143

(10225)	10225 10230 … 10240 … 10250 … 10260 … 10270 … 10280 … 10296
SARS-CoV-2 Reference Genome NC_045512.2 (10225)	ACTCATTCGTAAGTCTAATCATAATTTCTTGGTACAGGCTGGTAATGTTCAACTCAGGGTTATTGGACATTC
hCoV-19/Botswana/R69B55_BHP_916539/2021_EPI_ISL_90... (10171)	ACTCATTCGTAAGTCTAATCATAATTTCTTGGTACAGGCTGGTAATGTTCAACTCAGGGTTATTGGACATTC
hCoV-19/India/HR-MDU-IGIB1210605800489930/2022_EPI... (10213)	ACTCATTCGTAAGTCTAATCATAATTTCTTGGTACAGGCTGGTAATGTTCAACTCAGGGTTATTGGACATTC
hCoV-19/England/PLYM-332B917/2022_EPI_ISL_9062229_... (10172)	ACTCATTCGTAAGTCTAATCATAATTTCTTGGTACAGGCTGGTAATGTTCAACTCAGGGTTATTGGACATTC
hCoV-19/Germany/HE-RKI-I-438397/2022_EPI_ISL_905522... (10186)	ACTCATTCGTAAGTCTAATCATAATTTCTTGGTACAGGCTGGTAATGTTCAACTCAGGGTTATTGGACATTC
hCoV-19/USA/DE-CDC-LC0472738/2021_EPI_ISL_9049423... (10150)	ACTCATTCGTAAGTCTAATCATAATTTCTTGGTACAGGCTGGTAATGTTCAACTCAGGGTTATTGGACATTC

Section 144

(10297)	10297 … 10310 … 10320 … 10330 … 10340 … 10350 … 10368
SARS-CoV-2 Reference Genome NC_045512.2 (10297)	TATGCAAAATTGTGTACTTAAGCTTAAGGTTGATACAGCCAATCCTAAGACACCTAAGTATAAGTTTGTTCG
hCoV-19/Botswana/R69B55_BHP_916539/2021_EPI_ISL_90... (10243)	TATGCAAAATTGTGTACTTAAGCTTAAGGTTGATACAGCCAATCCTAAGACACCTAAGTATAAGTTTGTTCG
hCoV-19/India/HR-MDU-IGIB1210605800489930/2022_EPI... (10285)	TATGCAAAATTGTGTACTTAAGCTTAAGGTTGATACAGCCAATCCTAAGACACCTAAGTATAAGTTTGTTCG
hCoV-19/England/PLYM-332B917/2022_EPI_ISL_9062229_... (10244)	TATGCAAAATTGTGTACTTAAGCTTAAGGTTGATACAGCCAATCCTAAGACACCTAAGTATAAGTTTGTTCG
hCoV-19/Germany/HE-RKI-I-438397/2022_EPI_ISL_905522... (10258)	TATGCAAAATTGTGTACTTAAGCTTAAGGTTGATACAGCCAATCCTAAGACACCTAAGTATAAGTTTGTTCG
hCoV-19/USA/DE-CDC-LC0472738/2021_EPI_ISL_9049423... (10222)	TATGCAAAATTGTGTACTTAAGCTTAAGGTTGATACAGCCAATCCTAAGACACCTAAGTATAAGTTTGTTCG

Section 145

```
                                                        (10369) 10369      10380     10390     10400     10410     10420     10430   10440
SARS-CoV-2 Reference Genome NC_045512.2                 (10369) CATTCAACCAGGACAGACTTTTTCAGTGTTAGCTTGTTACAATGGTTCACCATCTGGTGTTTACCAATGTGC
hCoV-19/Botswana/R69B55_BHP_916539/2021_EPI_ISL_90...   (10315) CATTCAACCAGGACAGACTTTTTCAGTGTTAGCTTGTTACAATGGTTCACCATCTGGTGTTTACCAATGTGC
hCoV-19/India/HR-MDU-IGIB1210605800489930/2022_EPI...   (10357) CATTCAACCAGGACAGACTTTTTCAGTGTTAGCTTGTTACAATGGTTCACCATCTGGTGTTTACCAATGTGC
hCoV-19/England/PLYM-332B917/2022_EPI_ISL_9062229_...   (10316) CATTCAACCAGGACAGACTTTTTCAGTGTTAGCTTGTTACAATGGTTCACCATCTGGTGTTTACCAATGTGC
hCoV-19/Germany/HE-RKI-I-438397/2022_EPI_ISL_905522...  (10330) CATTCAACCAGGACAGACTTTTTCAGTGTTAGCTTGTTACAATGGTTCACCATCTGGTGTTTACCAATGTGC
hCoV-19/USA/DE-CDC-LC0472738/2021_EPI_ISL_9049423...    (10294) CATTCAACCAGGACAGACTTTTTCAGTGTTAGCTTGTTACAATGGTTCACCATCTGGTGTTTACCAATGTGC
```

Section 146

```
                                                        (10441) 10441    10450     10460     10470     10480     10490     10500       10512
SARS-CoV-2 Reference Genome NC_045512.2                 (10441) TATGAGGCCCAATTTCACTATTAAGGGTTCATTCCTTAATGGTTCATGTGGTAGTGTTGGTTTTAACATAGA
hCoV-19/Botswana/R69B55_BHP_916539/2021_EPI_ISL_90...   (10387) TATGAGGCACAATTTCACTATTAAGGGTTCATTCCTTAATGGTTCATGTGGTAGTGTTGGTTTTAACATAGA
hCoV-19/India/HR-MDU-IGIB1210605800489930/2022_EPI...   (10429) TATGAGGCACAATTTCACTATTAAGGGTTCATTCCTTAATGGTTCATGTGGTAGTGTTGGTTTTAACATAGA
hCoV-19/England/PLYM-332B917/2022_EPI_ISL_9062229_...   (10388) TATGAGGCACAATTTCACTATTAAGGGTTCATTCCTTAATGGTTCATGTGGTAGTGTTGGTTTTAACATAGA
hCoV-19/Germany/HE-RKI-I-438397/2022_EPI_ISL_905522...  (10402) TATGAGGCACAATTTCACTATTAAGGGTTCATTCCTTAATGGTTCATGTGGTAGTGTTGGTTTTAACATAGA
hCoV-19/USA/DE-CDC-LC0472738/2021_EPI_ISL_9049423...    (10366) TATGAGGCACAATTTCACTATTAAGGGTTCATTCCTTAATGGTTCATGTGGTAGTGTTGGTTTTAACATAGA
```

Section 147

```
                                                        (10513) 10513  10520     10530     10540     10550     10560     10570         10584
SARS-CoV-2 Reference Genome NC_045512.2                 (10513) TTATGACTGTGTCTCTTTTTGTTACATGCACCATATGGAATTACCAACTGGAGTTCATGCTGGCACAGACTT
hCoV-19/Botswana/R69B55_BHP_916539/2021_EPI_ISL_90...   (10459) TTATGACTGTGTCTCTTTTTGTTACATGCACCATATGGAATTACCAACTGGAGTTCATGCTGGCACAGACTT
hCoV-19/India/HR-MDU-IGIB1210605800489930/2022_EPI...   (10501) TTATGACTGTGTCTCTTTTTGTTACATGCACCATATGGAATTACCAACTGGAGTTCATGCTGGCACAGACTT
hCoV-19/England/PLYM-332B917/2022_EPI_ISL_9062229_...   (10460) TTATGACTGTGTCTCTTTTTGTTACATGCACCATATGGAATTACCAACTGGAGTTCATGCTGGCACAGACTT
hCoV-19/Germany/HE-RKI-I-438397/2022_EPI_ISL_905522...  (10474) TTATGACTGTGTCTCTTTTTGTTACATGCACCATATGGAATTACCAACTGGAGTTCATGCTGGCACAGACTT
hCoV-19/USA/DE-CDC-LC0472738/2021_EPI_ISL_9049423...    (10438) TTATGACTGTGTCTCTTTTTGTTACATGCACCATATGGAATTACCAACTGGAGTTCATGCTGGCACAGACTT
```

Section 148

```
                                                        (10585) 10585 10590     10600     10610     10620     10630     10640          10656
SARS-CoV-2 Reference Genome NC_045512.2                 (10585) AGAAGGTAACTTTTATGGACCTTTTGTTGACAGGCAAACAGCACAAGCAGCTGGTACGGACACAACTATTAC
hCoV-19/Botswana/R69B55_BHP_916539/2021_EPI_ISL_90...   (10531) AGAAGGTAACTTTTATGGACCTTTTGTTGACAGGCAAACAGCACAAGCAGCTGGTACGGACACAACTATTAC
hCoV-19/India/HR-MDU-IGIB1210605800489930/2022_EPI...   (10573) AGAAGGTAACTTTTATGGACCTTTTGTTGACAGGCAAACAGCACAAGCAGCTGGTACGGACACAACTATTAC
hCoV-19/England/PLYM-332B917/2022_EPI_ISL_9062229_...   (10532) AGAAGGTAACTTTTATGGACCTTTTGTTGACAGGCAAACAGCACAAGCAGCTGGTACGGACACAACTATTAC
hCoV-19/Germany/HE-RKI-I-438397/2022_EPI_ISL_905522...  (10546) AGAAGGTAACTTTTATGGACCTTTTGTTGACAGGCAAACAGCACAAGCAGCTGGTACGGACACAACTATTAC
hCoV-19/USA/DE-CDC-LC0472738/2021_EPI_ISL_9049423...    (10510) AGAAGGTAACTTTTATGGACCTTTTGTTGACAGGCAAACAGCACAAGCAGCTGGTACGGACACAACTATTAC
```

Section 149

	(10657)	10657 10670 10680 10690 10700 10710 10728
SARS-CoV-2 Reference Genome NC_045512.2	(10657)	AGTTAATGTTTTAGCTTGGTTGTACGCTGCTGTTATAAATGGAGACAGGTGGTTTCTCAATCGATTTACCAC
hCoV-19/Botswana/R69B55_BHP_916539/2021_EPI_ISL_90...	(10603)	AGTTAATGTTTTAGCTTGGTTGTACGCTGCTGTTATAAATGGAGACAGGTGGTTTCTCAATCGATTTACCAC
hCoV-19/India/HR-MDU-IGIB1210605800489930/2022_EPI...	(10645)	AGTTAATGTTTTAGCTTGGTTGTACGCTGCTGTTATAAATGGAGACAGGTGGTTTCTCAATCGATTTACCAC
hCoV-19/England/PLYM-332B917/2022_EPI_ISL_9062229_...	(10604)	AGTTAATGTTTTAGCTTGGTTGTACGCTGCTGTTATAAATGGAGACAGGTGGTTTCTCAATCGATTTACCAC
hCoV-19/Germany/HE-RKI-I-438397/2022_EPI_ISL_905522...	(10618)	AGTTAATGTTTTAGCTTGGTTGTACGCTGCTGTTATAAATGGAGACAGGTGGTTTCTCAATCGATTTACCAC
hCoV-19/USA/DE-CDC-LC0472738/2021_EPI_ISL_9049423...	(10582)	AGTTAATGTTTTAGCTTGGTTGTACGCTGCTGTTATAAATGGAGACAGGTGGTTTCTCAATCGATTTACCAC

Section 150

	(10729)	10729 10740 10750 10760 10770 10780 10790 10800
SARS-CoV-2 Reference Genome NC_045512.2	(10729)	AACTCTTAATGACTTTAACCTTGTGGCTATGAAGTACAATTATGAACCTCTAACACAAGACCATGTTGACAT
hCoV-19/Botswana/R69B55_BHP_916539/2021_EPI_ISL_90...	(10675)	AACTCTTAATGACTTTAACCTTGTGGCTATGAAGTACAATTATGAACCTCTAACACAAGACCATGTTGACAT
hCoV-19/India/HR-MDU-IGIB1210605800489930/2022_EPI...	(10717)	AACTCTTAATGACTTTAACCTTGTGGCTATGAAGTACAATTATGAACCTCTAACACAAGACCATGTTGACAT
hCoV-19/England/PLYM-332B917/2022_EPI_ISL_9062229_...	(10676)	AACTCTTAATGACTTTAACCTTGTGGCTATGAAGTACAATTATGAACCTCTAACACAAGACCATGTTGACAT
hCoV-19/Germany/HE-RKI-I-438397/2022_EPI_ISL_905522...	(10690)	AACTCTTAATGACTTTAACCTTGTGGCTATGAAGTACAATTATGAACCTCTAACACAAGACCATGTTGACAT
hCoV-19/USA/DE-CDC-LC0472738/2021_EPI_ISL_9049423...	(10654)	AACTCTTAATGACTTTAACCTTGTGGCTATGAAGTACAATTATGAACCTCTAACACAAGACCATGTTGACAT

Section 151

	(10801)	10801 10810 10820 10830 10840 10850 10860 10872
SARS-CoV-2 Reference Genome NC_045512.2	(10801)	ACTAGGACCTCTTTCTGCTCAAACTGGAATTGCCGTTTTAGATATGTGTGCTTCATTAAAAGAATTACTGCA
hCoV-19/Botswana/R69B55_BHP_916539/2021_EPI_ISL_90...	(10747)	ACTAGGACCTCTTTCTGCTCAAACTGGAATTGCCGTTTTAGATATGTGTGCTTCATTAAAAGAATTACTGCA
hCoV-19/India/HR-MDU-IGIB1210605800489930/2022_EPI...	(10789)	ACTAGGACCTCTTTCTGCTCAAACTGGAATTGCCGTTTTAGATATGTGTGCTTCATTAAAAGAATTACTGCA
hCoV-19/England/PLYM-332B917/2022_EPI_ISL_9062229_...	(10748)	ACTAGGACCTCTTTCTGCTCAAACTGGAATTGCCGTTTTAGATATGTGTGCTTCATTAAAAGAATTACTGCA
hCoV-19/Germany/HE-RKI-I-438397/2022_EPI_ISL_905522...	(10762)	ACTAGGACCTCTTTCTGCTCAAACTGGAATTGCCGTTTTAGATATGTGTGCTTCATTAAAAGAATTACTGCA
hCoV-19/USA/DE-CDC-LC0472738/2021_EPI_ISL_9049423...	(10726)	ACTAGGACCTCTTTCTGCTCAAACTGGAATTGCCGTTTTAGATATGTGTGCTTCATTAAAAGAATTACTGCA

Section 152

	(10873)	10873 10880 10890 10900 10910 10920 10930 10944
SARS-CoV-2 Reference Genome NC_045512.2	(10873)	AAATGGTATGAATGGACGTACCATATTGGGTAGTGCTTTATTAGAAGATGAATTTACACCTTTTGATGTTGT
hCoV-19/Botswana/R69B55_BHP_916539/2021_EPI_ISL_90...	(10819)	AAATGGTATGAATGGACGTACCATATTGGGTAGTGCTTTATTAGAAGATGAATTTACACCTTTTGATGTTGT
hCoV-19/India/HR-MDU-IGIB1210605800489930/2022_EPI...	(10861)	AAATGGTATGAATGGACGTACCATATTGGGTAGTGCTTTATTAGAAGATGAATTTACACCTTTTGATGTTGT
hCoV-19/England/PLYM-332B917/2022_EPI_ISL_9062229_...	(10820)	AAATGGTATGAATGGACGTACCATATTGGGTAGTGCTTTATTAGAAGATGAATTTACACCTTTTGATGTTGT
hCoV-19/Germany/HE-RKI-I-438397/2022_EPI_ISL_905522...	(10834)	AAATGGTATGAATGGACGTACCATATTGGGTAGTGCTTTATTAGAAGATGAATTTACACCTTTTGATGTTGT
hCoV-19/USA/DE-CDC-LC0472738/2021_EPI_ISL_9049423...	(10798)	AAATGGTATGAATGGACGTACCATATTGGGTAGTGCTTTATTAGAAGATGAATTTACACCTTTTGATGTTGT

Section 153

(10945)		10945 10950 10960 10970 10980 10990 11000 11016
SARS-CoV-2 Reference Genome NC_045512.2	(10945)	TAGACAATGCTCAGGTGTTACTTTCCAAAGTGCAGTGAAAAGAACAATCAAGGGTACACACCACTGGTTGTT
hCoV-19/Botswana/R69B55_BHP_916539/2021_EPI_ISL_90...	(10891)	TAGACAATGCTCAGGTGTTACTTTCCAAAGTGCAGTGAAAAGAACAATCAAGGGTACACACCACTGGTTGTT
hCoV-19/India/HR-MDU-IGIB1210605800489930/2022_EPI...	(10933)	TAGACAATGCTCAGGTGTTACTTTCCAAAGTGCAGTGAAAAGAACAATCAAGGGTACACACCACTGGTTGTT
hCoV-19/England/PLYM-332B917/2022_EPI_ISL_9062229_...	(10892)	TAGACAATGCTCAGGTGTTACTTTCCAAAGTGCAGTGAAAAGAACAATCAAGGGTACACACCACTGGTTGTT
hCoV-19/Germany/HE-RKI-I-438397/2022_EPI_ISL_905522...	(10906)	TAGACAATGCTCAGGTGTTACTTTCCAAAGTGCAGTGAAAAGAACAATCAAGGGTACACACCACTGGTTGTT
hCoV-19/USA/DE-CDC-LC0472738/2021_EPI_ISL_9049423...	(10870)	TAGACAATGCTCAGGTGTTACTTTCCAAAGTGCAGTGAAAAGAACAATCAAGGGTACACACCACTGGTTGTT

Section 154

(11017)		11017 11030 11040 11050 11060 11070 11088
SARS-CoV-2 Reference Genome NC_045512.2	(11017)	ACTCACAATTTTGACTTCACTTTTAGTTTTAGTCCAGAGTACTCAATGGTCTTTGTTCTTTTTTTTGTATGA
hCoV-19/Botswana/R69B55_BHP_916539/2021_EPI_ISL_90...	(10963)	ACTCACAATTTTGACTTCACTTTTAGTTTTAGTCCAGAGTACTCAATGGTCTTTGTTCTTTTTTTTGTATGA
hCoV-19/India/HR-MDU-IGIB1210605800489930/2022_EPI...	(11005)	ACTCACAATTTTGACTTCACTTTTAGTTTTAGTCCAGAGTACTCAATGGTCTTTGTTCTTTTTTTTGTATGA
hCoV-19/England/PLYM-332B917/2022_EPI_ISL_9062229_...	(10964)	ACTCACAATTTTGACTTCACTTTTAGTTTTAGTCCAGAGTACTCAATGGTCTTTGTTCTTTTTTTTGTATGA
hCoV-19/Germany/HE-RKI-I-438397/2022_EPI_ISL_905522...	(10978)	ACTCACAATTTTGACTTCACTTTTAGTTTTAGTCCAGAGTACTCAATGGTCTTTGTTCTTTTTTTTGTATGA
hCoV-19/USA/DE-CDC-LC0472738/2021_EPI_ISL_9049423...	(10942)	ACTCACAATTTTGACTTCACTTTTAGTTTTAGTCCAGAGTACTCAATGGTCTTTGTTCTTTTTTTTGTATGA

Section 155

(11089)		11089 11100 11110 11120 11130 11140 11150 11160
SARS-CoV-2 Reference Genome NC_045512.2	(11089)	AAATGCCTTTTTACCTTTTGCTATGGGTATTATTGCTATGTCTGCTTTTGCAATGATGTTTGTCAAACATAA
hCoV-19/Botswana/R69B55_BHP_916539/2021_EPI_ISL_90...	(11035)	AAATGCCTTTTTACCTTTTGCTATGGGTATTATTGCTATGTCTGCTTTTGCAATGATGTTTGTCAAACATAA
hCoV-19/India/HR-MDU-IGIB1210605800489930/2022_EPI...	(11077)	AAATGCCTTTTTACCTTTTGCTATGGGTATTATTGCTATGTCTGCTTTTGCAATGATGTTTGTCAAACATAA
hCoV-19/England/PLYM-332B917/2022_EPI_ISL_9062229_...	(11036)	AAATGCCTTTTTACCTTTTGCTATGGGTATTATTGCTATGTCTGCTTTTGCAATGATGTTTGTCAAACATAA
hCoV-19/Germany/HE-RKI-I-438397/2022_EPI_ISL_905522...	(11050)	AAATGCCTTTTTACCTTTTGCTATGGGTATTATTGCTATGTCTGCTTTTGCAATGATGTTTGTCAAACATAA
hCoV-19/USA/DE-CDC-LC0472738/2021_EPI_ISL_9049423...	(11014)	AAATGCCTTTTTACCTTTTGCTATGGGTATTATTGCTATGTCTGCTTTTGCAATGATGTTTGTCAAACATAA

Section 156

(11161)		11161 11170 11180 11190 11200 11210 11220 11232
SARS-CoV-2 Reference Genome NC_045512.2	(11161)	GCATGCATTTCTCTGTTTGTTTTTGTTACCTTCTCTTGCCACTGTAGCTTATTTTAATATGGTCTATATGCC
hCoV-19/Botswana/R69B55_BHP_916539/2021_EPI_ISL_90...	(11107)	GCATGCATTTCTCTGTTTGTTTTTGTTACCTTCTCTTGCCACTGTAGCTTATTTTAATATGGTCTATATGCC
hCoV-19/India/HR-MDU-IGIB1210605800489930/2022_EPI...	(11149)	GCATGCATTTCTCTGTTTGTTTTTGTTACCTTCTCTTGCCACTGTAGCTTATTTTAATATGGTCTATATGCC
hCoV-19/England/PLYM-332B917/2022_EPI_ISL_9062229_...	(11108)	GCATGCATTTCTCTGTTTGTTTTTGTTACCTTCTCTTGCCACTGTAGCTTATTTTAATATGGTCTATATGCC
hCoV-19/Germany/HE-RKI-I-438397/2022_EPI_ISL_905522...	(11122)	GCATGCATTTCTCTGTTTGTTTTTGTTACCTTCTCTTGCCACTGTAGCTTATTTTAATATGGTCTATATGCC
hCoV-19/USA/DE-CDC-LC0472738/2021_EPI_ISL_9049423...	(11086)	GCATGCATTTCTCTGTTTGTTTTTGTTACCTTCTCTTGCCACTGTAGCTTATTTTAATATGGTCTATATGCC

Section 157

(11233)	11233 11240 11250 11260 11270 11280 11290 11304
SARS-CoV-2 Reference Genome NC_045512.2 (11233)	TGCTAGTTGGGTGATGCGTATTATGACATGGTTGGATATGGTTGATACTAGTTTGTCTGGTTTTAAGCTAAA
hCoV-19/Botswana/R69B55_BHP_916539/2021_EPI_ISL_90... (11179)	TGCTAGTTGGGTGATGCGTATTATGACATGGTTGGATATGGTTGATACTAGTNNNNNNNNNTTTAAGCTAAA
hCoV-19/India/HR-MDU-IGIB1210605800489930/2022_EPI... (11221)	TGCTAGTTGGGTGATGCGTATTATGACATGGTTGGATATGGTTGATACTAGTTTGTCTGGTTTTAAGCTAAA
hCoV-19/England/PLYM-332B917/2022_EPI_ISL_9062229_... (11180)	TGCTAGTTGGGTGATGCGTATTATGACATGGTTGGATATGGTTGATACTAG---------TTTTAAGCTAAA
hCoV-19/Germany/HE-RKI-I-438397/2022_EPI_ISL_905522... (11194)	TGCTAGTTGGGTGATGCGTATTATGACATGGTTGGATATGGTTGATACTAG---------TTTTAAGCTAAA
hCoV-19/USA/DE-CDC-LC0472738/2021_EPI_ISL_9049423... (11158)	TGCTAGTTGGGTGATGCGTATTATGACATGGTTGGATATGGTTGATACTAG---------TTTTAAGCTAAA

Section 158

(11305)	11305 11310 11320 11330 11340 11350 11360 11376
SARS-CoV-2 Reference Genome NC_045512.2 (11305)	AGACTGTGTTATGTATGCATCAGCTGTAGTGTTACTAATCCTTATGACAGCAAGAACTGTGTATGATGATGG
hCoV-19/Botswana/R69B55_BHP_916539/2021_EPI_ISL_90... (11251)	AGACTGTGTTATGTATGCATCAGCTGTAGTGTTACTAATCCTTATGACAGCAAGAACTGTGTATGATGATGG
hCoV-19/India/HR-MDU-IGIB1210605800489930/2022_EPI... (11293)	AGACTGTGTTATGTATGCATCAGCTGTAGTGTTACTAATCCTTATGACAGCAAGAACTGTGTATGATGATGG
hCoV-19/England/PLYM-332B917/2022_EPI_ISL_9062229_... (11243)	AGACTGTGTTATGTATGCATCAGCTGTAGTGTTACTAATCCTTATGACAGCAAGAACTGTGTATGATGATGG
hCoV-19/Germany/HE-RKI-I-438397/2022_EPI_ISL_905522... (11257)	AGACTGTGTTATGTATGCATCAGCTGTAGTGTTACTAATCCTTATGACAGCAAGAACTGTGTATGATGATGG
hCoV-19/USA/DE-CDC-LC0472738/2021_EPI_ISL_9049423... (11221)	AGACTGTGTTATGTATGCATCAGCTGTAGTGTTACTAATCCTTATGACAGCAAGAACTGTGTATGATGATGG

Section 159

(11377)	11377 11390 11400 11410 11420 11430 11448
SARS-CoV-2 Reference Genome NC_045512.2 (11377)	TGCTAGGAGAGTGTGGACACTTATGAATGTCTTGACACTCGTTTATAAAGTTTATTATGGTAATGCTTTAGA
hCoV-19/Botswana/R69B55_BHP_916539/2021_EPI_ISL_90... (11323)	TGCTAGGAGAGTGTGGACACTTATGAATGTCTTGACACTCGTTTATAAAGTTTATTATGGTAATGCTTTAGA
hCoV-19/India/HR-MDU-IGIB1210605800489930/2022_EPI... (11365)	TGCTAGGAGAGTGTGGACACTTATGAATGTCTTGACACTCGTTTATAAAGTTTATTATGGTAATGCTTTAGA
hCoV-19/England/PLYM-332B917/2022_EPI_ISL_9062229_... (11315)	TGCTAGGAGAGTGTGGACACTTATGAATGTCTTGACACTCGTTTATAAAGTTTATTATGGTAATGCTTTAGA
hCoV-19/Germany/HE-RKI-I-438397/2022_EPI_ISL_905522... (11329)	TGCTAGGAGAGTGTGGACACTTATGAATGTCTTGACACTCGTTTATAAAGTTTATTATGGTAATGCTTTAGA
hCoV-19/USA/DE-CDC-LC0472738/2021_EPI_ISL_9049423... (11293)	TGCTAGGAGAGTGTGGACACTTATGAATGTCTTGACACTCGTTTATAAAGTTTATTATGGTAATGCTTTAGA

Section 160

(11449)	11449 11460 11470 11480 11490 11500 11510 11520
SARS-CoV-2 Reference Genome NC_045512.2 (11449)	TCAAGCCATTTCCATGTGGGCTCTTATAATCTCTGTTACTTCTAACTACTCAGGTGTAGTTACAACTGTCAT
hCoV-19/Botswana/R69B55_BHP_916539/2021_EPI_ISL_90... (11395)	TCAAGCCATTTCCATGTGGGCTCTTATAATCTCTGTTACTTCTAACTACTCAGGTGTAGTTACAACTGTCAT
hCoV-19/India/HR-MDU-IGIB1210605800489930/2022_EPI... (11437)	TCAAGCCATTTCCATGTGGGCTCTTATAATCTCTGTTACTTCTAACTACTCAGGTGTAGTTACAACTGTCAT
hCoV-19/England/PLYM-332B917/2022_EPI_ISL_9062229_... (11387)	TCAAGCCATTTCCATGTGGGCTCTTATAATCTCTGTTACTTCTAACTACTCAGGTGTAGTTACAACTGTCAT
hCoV-19/Germany/HE-RKI-I-438397/2022_EPI_ISL_905522... (11401)	TCAAGCCATTTCCATGTGGGCTCTTATAATCTCTGTTACTTCTAACTACTCAGGTGTAGTTACAACTGTCAT
hCoV-19/USA/DE-CDC-LC0472738/2021_EPI_ISL_9049423... (11365)	TCAAGCCATTTCCATGTGGGCTCTTATAATCTCTGTTACTTCTAACTACTCAGGTGTAGTTACAACTGTCAT

Section 161

	(11521)	11521 … 11592
SARS-CoV-2 Reference Genome NC_045512.2	(11521)	GTTTTTGGCCAGAGGTATTGTTTTTATGTGTGTTGAGTATTGCCCTATTTTCTTCATAACTGGTAATACACT
hCoV-19/Botswana/R69B55_BHP_916539/2021_EPI_ISL_90...	(11467)	GTTTTTGGCCAGAGGTGTTGTTTTTATGTGTGTTGAGTATTGCCCTATTTTCTTCATAACTGGTAATACACT
hCoV-19/India/HR-MDU-IGIB1210605800489930/2022_EPI...	(11509)	GTTTTTGGCCAGAGGTGTTGTTTTTATGTGTGTTGAGTATTGCCCTATTTTCTTCATAACTGGTAATACACT
hCoV-19/England/PLYM-332B917/2022_EPI_ISL_9062229_...	(11459)	GTTTTTGGCCAGAGGTGTTGTTTTTATGTGTGTTGAGTATTGCCCTATTTTCTTCATAACTGGTAATACACT
hCoV-19/Germany/HE-RKI-I-438397/2022_EPI_ISL_905522...	(11473)	GTTTTTGGCCAGAGGTGTTGTTTTTATGTGTGTTGAGTATTGCCCTATTTTCTTCATAACTGGTAATACACT
hCoV-19/USA/DE-CDC-LC0472738/2021_EPI_ISL_9049423...	(11437)	GTTTTTGGCCAGAGGTGTTGTTTTTATGTGTGTTGAGTATTGCCCTATTTTCTTCATAACTGGTAATACACT

Section 162

	(11593)	11593 … 11664
SARS-CoV-2 Reference Genome NC_045512.2	(11593)	TCAGTGTATAATGCTAGTTTATTGTTTCTTAGGCTATTTTTGTACTTGTTACTTTGGCCTCTTTTGTTTACT
hCoV-19/Botswana/R69B55_BHP_916539/2021_EPI_ISL_90...	(11539)	TCAGTGTATAATGCTAGTTTATTGTTTCTTAGGCTATTTTTGTACTTGTTACTTTGGCCTCTTTTGTTTACT
hCoV-19/India/HR-MDU-IGIB1210605800489930/2022_EPI...	(11581)	TCAGTGTATAATGCTAGTTTATTGTTTCTTAGGCTATTTTTGTACTTGTTACTTTGGCCTCTTTTGTTTACT
hCoV-19/England/PLYM-332B917/2022_EPI_ISL_9062229_...	(11531)	TCAGTGTATAATGCTAGTTTATTGTTTCTTAGGCTATTTTTGTACTTGTTACTTTGGCCTCTTTTGTTTACT
hCoV-19/Germany/HE-RKI-I-438397/2022_EPI_ISL_905522...	(11545)	TCAGTGTATAATGCTAGTTTATTGTTTCTTAGGCTATTTTTGTACTTGTTACTTTGGCCTCTTTTGTTTACT
hCoV-19/USA/DE-CDC-LC0472738/2021_EPI_ISL_9049423...	(11509)	TCAGTGTATAATGCTAGTTTATTGTTTCTTAGGCTATTTTTGTACTTGTTACTTTGGCCTCTTTTGTTTACT

Section 163

	(11665)	11665 … 11736
SARS-CoV-2 Reference Genome NC_045512.2	(11665)	CAACCGCTACTTTAGACTGACTCTTGGTGTTTATGATTACTTAGTTTCTACACAGGAGTTTAGATATATGAA
hCoV-19/Botswana/R69B55_BHP_916539/2021_EPI_ISL_90...	(11611)	CAACCGCTACTTTAGACTGACTCTTGGTGTTTATGATTACTTAGTTTCTACACAGGAGTTTAGATATATGAA
hCoV-19/India/HR-MDU-IGIB1210605800489930/2022_EPI...	(11653)	CAACCGCTACTTTAGACTGACTCTTGGTGTTTATGATTACTTAGTTTCTACACAGGAGTTTAGATATATGAA
hCoV-19/England/PLYM-332B917/2022_EPI_ISL_9062229_...	(11603)	CAACCGCTACTTTAGACTGACTCTTGGTGTTTATGATTACTTAGTTTCTACACAGGAGTTTAGATATATGAA
hCoV-19/Germany/HE-RKI-I-438397/2022_EPI_ISL_905522...	(11617)	CAACCGCTACTTTAGACTGACTCTTGGTGTTTATGATTACTTAGTTTCTACACAGGAGTTTAGATATATGAA
hCoV-19/USA/DE-CDC-LC0472738/2021_EPI_ISL_9049423...	(11581)	CAACCGCTACTTTAGACTGACTCTTGGTGTTTATGATTACTTAGTTTCTACACAGGAGTTTAGATATATGAA

Section 164

	(11737)	11737 … 11808
SARS-CoV-2 Reference Genome NC_045512.2	(11737)	TTCACAGGGACTACTCCCACCCAAGAATAGCATAGATGCCTTCAAACTCAACATTAAATTGTTGGGTGTTGG
hCoV-19/Botswana/R69B55_BHP_916539/2021_EPI_ISL_90...	(11683)	TTCACAGGGACTACTCCCACCCAAGAATAGCATAGATGCCTTCAAACTCAACATTAAATTGTTGGGTGTTGG
hCoV-19/India/HR-MDU-IGIB1210605800489930/2022_EPI...	(11725)	TTCACAGGGACTACTCCCACCCAAGAATAGCATAGATGCCTTCAAACTCAACATTAAATTGTTGGGTGTTGG
hCoV-19/England/PLYM-332B917/2022_EPI_ISL_9062229_...	(11675)	TTCACAGGGACTACTCCCACCCAAGAATAGCATAGATGCCTTCAAACTCAACATTAAATTGTTGGGTGTTGG
hCoV-19/Germany/HE-RKI-I-438397/2022_EPI_ISL_905522...	(11689)	TTCACAGGGACTACTCCCACCCAAGAATAGCATAGATGCCTTCAAACTCAACATTAAATTGTTGGGTGTTGG
hCoV-19/USA/DE-CDC-LC0472738/2021_EPI_ISL_9049423...	(11653)	TTCACAGGGACTACTCCCACCCAAGAATAGCATAGATGCCTTCAAACTCAACATTAAATTGTTGGGTGTTGG

Section 165

	(11809)	11809 11820 11830 11840 11850 11860 11870 11880
SARS-CoV-2 Reference Genome NC_045512.2	(11809)	TGGCAAACCTTGTATCAAAGTAGCCACTGTACAGTCTAAAATGTCAGATGTAAAGTGCACATCAGTAGTCTT
hCoV-19/Botswana/R69B55_BHP_916539/2021_EPI_ISL_90...	(11755)	TGGCAAACCTTGTATCAAAGTAGCCACTGTACAGTCTAAAATGTCAGATGTAAAGTGCACATCAGTAGTCTT
hCoV-19/India/HR-MDU-IGIB1210605800489930/2022_EPI...	(11797)	TGGCAAACCTTGTATCAAAGTAGCCACTGTACAGTCTAAAATGTCAGATGTAAAGTGCACATCAGTAGTCTT
hCoV-19/England/PLYM-332B917/2022_EPI_ISL_9062229_...	(11747)	TGGCAAACCTTGTATCAAAGTAGCCACTGTACAGTCTAAAATGTCAGATGTAAAGTGCACATCAGTAGTCTT
hCoV-19/Germany/HE-RKI-I-438397/2022_EPI_ISL_905522...	(11761)	TGGCAAACCTTGTATCAAAGTAGCCACTGTACAGTCTAAAATGTCAGATGTAAAGTGCACATCAGTAGTCTT
hCoV-19/USA/DE-CDC-LC0472738/2021_EPI_ISL_9049423...	(11725)	TGGCAAACCTTGTATCAAAGTAGCCACTGTACAGTCTAAAATGTCAGATGTAAAGTGCACATCAGTAGTCTT

Section 166

	(11881)	11881 11890 11900 11910 11920 11930 11940 11952
SARS-CoV-2 Reference Genome NC_045512.2	(11881)	ACTCTCAGTTTTGCAACAACTCAGAGTAGAATCATCATCTAAATTGTGGGCTCAATGTGTCCAGTTACACAA
hCoV-19/Botswana/R69B55_BHP_916539/2021_EPI_ISL_90...	(11827)	ACTCTCAGTTTTGCAACAACTCAGAGTAGAATCATCATCTAAATTGTGGGCTCAATGTGTCCAGTTACACAA
hCoV-19/India/HR-MDU-IGIB1210605800489930/2022_EPI...	(11869)	ACTCTCAGTTTTGCAACAACTCAGAGTAGAATCATCATCTAAATTGTGGGCTCAATGTGTCCAGTTACACAA
hCoV-19/England/PLYM-332B917/2022_EPI_ISL_9062229_...	(11819)	ACTCTCAGTTTTGCAACAACTCAGAGTAGAATCATCATCTAAATTGTGGGCTCAATGTGTCCAGTTACACAA
hCoV-19/Germany/HE-RKI-I-438397/2022_EPI_ISL_905522...	(11833)	ACTCTCAGTTTTGCAACAACTCAGAGTAGAATCATCATCTAAATTGTGGGCTCAATGTGTCCAGTTACACAA
hCoV-19/USA/DE-CDC-LC0472738/2021_EPI_ISL_9049423...	(11797)	ACTCTCAGTTTTGCAACAACTCAGAGTAGAATCATCATCTAAATTGTGGGCTCAATGTGTCCAGTTACACAA

Section 167

	(11953)	11953 11960 11970 11980 11990 12000 12010 12024
SARS-CoV-2 Reference Genome NC_045512.2	(11953)	TGACATTCTCTTAGCTAAAGATACTACTGAAGCCTTTGAAAAAATGGTTTCACTACTTTCTGTTTTGCTTTC
hCoV-19/Botswana/R69B55_BHP_916539/2021_EPI_ISL_90...	(11899)	TGACATTCTCTTAGCTAAAGATACTACTGAAGCCTTTGAAAAAATGGTTTCACTACTTTCTGTTTTGCTTTC
hCoV-19/India/HR-MDU-IGIB1210605800489930/2022_EPI...	(11941)	TGACATTCTCTTAGCTAAAGATACTACTGAAGCCTTTGAAAAAATGGTTTCACTACTTTCTGTTTTGCTTTC
hCoV-19/England/PLYM-332B917/2022_EPI_ISL_9062229_...	(11891)	TGACATTCTCTTAGCTAAAGATACTACTGAAGCCTTTGAAAAAATGGTTTCACTACTTTCTGTTTTGCTTTC
hCoV-19/Germany/HE-RKI-I-438397/2022_EPI_ISL_905522...	(11905)	TGACATTCTCTTAGCTAAAGATACTACTGAAGCCTTTGAAAAAATGGTTTCACTACTTTCTGTTTTGCTTTC
hCoV-19/USA/DE-CDC-LC0472738/2021_EPI_ISL_9049423...	(11869)	TGACATTCTCTTAGCTAAAGATACTACTGAAGCCTTTGAAAAAATGGTTTCACTACTTTCTGTTTTGCTTTC

Section 168

	(12025)	12025 12030 12040 12050 12060 12070 12080 12096
SARS-CoV-2 Reference Genome NC_045512.2	(12025)	CATGCAGGGTGCTGTAGACATAAACAAGCTTTGTGAAGAAATGCTGGACAACAGGGCAACCTTACAAGCTAT
hCoV-19/Botswana/R69B55_BHP_916539/2021_EPI_ISL_90...	(11971)	CATGCAGGGTGCTGTAGACATAAACAAGCTTTGTGAAGAAATGCTGGACAACAGGGCAACCTTACAAGCTAT
hCoV-19/India/HR-MDU-IGIB1210605800489930/2022_EPI...	(12013)	CATGCAGGGTGCTGTAGACATAAACAAGCTTTGTGAAGAAATGCTGGACAACAGGGCAACCTTACAAGCTAT
hCoV-19/England/PLYM-332B917/2022_EPI_ISL_9062229_...	(11963)	CATGCAGGGTGCTGTAGACATAAACAAGCTTTGTGAAGAAATGCTGGACAACAGGGCAACCTTACAAGCTAT
hCoV-19/Germany/HE-RKI-I-438397/2022_EPI_ISL_905522...	(11977)	CATGCAGGGTGCTGTAGACATAAACAAGCTTTGTGAAGAAATGCTGGACAACAGGGCAACCTTACAAGCTAT
hCoV-19/USA/DE-CDC-LC0472738/2021_EPI_ISL_9049423...	(11941)	CATGCAGGGTGCTGTAGACATAAACAAGCTTTGTGAAGAAATGCTGGACAACAGGGCAACCTTACAAGCTAT

Section 169

	(12097)	12097 12110 12120 12130 12140 12150 12168
SARS-CoV-2 Reference Genome NC_045512.2	(12097)	AGCCTCAGAGTTTAGTTCCCTTCCATCATATGCAGCTTTTGCTACTGCTCAAGAAGCTTATGAGCAGGCTGT
hCoV-19/Botswana/R69B55_BHP_916539/2021_EPI_ISL_90...	(12043)	AGCCTCAGAGTTTAGTTCCCTTCCATCATATGCAGCTTTTGCTACTGCTCAAGAAGCTTATGAGCAGGCTGT
hCoV-19/India/HR-MDU-IGIB1210605800489930/2022_EPI...	(12085)	AGCCTCAGAGTTTAGTTCCCTTCCATCATATGCAGCTTTTGCTACTGCTCAAGAAGCTTATGAGCAGGCTGT
hCoV-19/England/PLYM-332B917/2022_EPI_ISL_9062229_...	(12035)	AGCCTCAGAGTTTAGTTCCCTTCCATCATATGCAGCTTTTGCTACTGCTCAAGAAGCTTATGAGCAGGCTGT
hCoV-19/Germany/HE-RKI-I-438397/2022_EPI_ISL_905522...	(12049)	AGCCTCAGAGTTTAGTTCCCTTCCATCATATGCAGCTTTTGCTACTGCTCAAGAAGCTTATGAGCAGGCTGT
hCoV-19/USA/DE-CDC-LC0472738/2021_EPI_ISL_9049423...	(12013)	AGCCTCAGAGTTTAGTTCCCTTCCATCATATGCAGCTTTTGCTACTGCTCAAGAAGCTTATGAGCAGGCTGT

Section 170

	(12169)	12169 12180 12190 12200 12210 12220 12230 12240
SARS-CoV-2 Reference Genome NC_045512.2	(12169)	TGCTAATGGTGATTCTGAAGTTGTTCTTAAAAAGTTGAAGAAGTCTTTGAATGTGGCTAAATCTGAATTTGA
hCoV-19/Botswana/R69B55_BHP_916539/2021_EPI_ISL_90...	(12115)	TGCTAATGGTGATTCTGAAGTTGTTCTTAAAAAGTTGAAGAAGTCTTTGAATGTGGCTAAATCTGAATTTGA
hCoV-19/India/HR-MDU-IGIB1210605800489930/2022_EPI...	(12157)	TGCTAATGGTGATTCTGAAGTTGTTCTTAAAAAGTTGAAGAAGTCTTTGAATGTGGCTAAATCTGAATTTGA
hCoV-19/England/PLYM-332B917/2022_EPI_ISL_9062229_...	(12107)	TGCTAATGGTGATTCTGAAGTTGTTCTTAAAAAGTTGAAGAAGTCTTTGAATGTGGCTAAATCTGAATTTGA
hCoV-19/Germany/HE-RKI-I-438397/2022_EPI_ISL_905522...	(12121)	TGCTAATGGTGATTCTGAAGTTGTTCTTAAAAAGTTGAAGAAGTCTTTGAATGTGGCTAAATCTGAATTTGA
hCoV-19/USA/DE-CDC-LC0472738/2021_EPI_ISL_9049423...	(12085)	TGCTAATGGTGATTCTGAAGTTGTTCTTAAAAAGTTGAAGAAGTCTTTGAATGTGGCTAAATCTGAATTTGA

Section 171

	(12241)	12241 12250 12260 12270 12280 12290 12300 12312
SARS-CoV-2 Reference Genome NC_045512.2	(12241)	CCGTGATGCAGCCATGCAACGTAAGTTGGAAAAGATGGCTGATCAAGCTATGACCCAAATGTATAAACAGGC
hCoV-19/Botswana/R69B55_BHP_916539/2021_EPI_ISL_90...	(12187)	CCGTGATGCAGCCATGCAACGTAAGTTGGAAAAGATGGCTGATCAAGCTATGACCCAAATGTATAAACAGGC
hCoV-19/India/HR-MDU-IGIB1210605800489930/2022_EPI...	(12229)	CCGTGATGCAGCCATGCAACGTAAGTTGGAAAAGATGGCTGATCAAGCTATGACCCAAATGTATAAACAGGC
hCoV-19/England/PLYM-332B917/2022_EPI_ISL_9062229_...	(12179)	CCGTGATGCAGCCATGCAACGTAAGTTGGAAAAGATGGCTGATCAAGCTATGACCCAAATGTATAAACAGGC
hCoV-19/Germany/HE-RKI-I-438397/2022_EPI_ISL_905522...	(12193)	CCGTGATGCAGCCATGCAACGTAAGTTGGAAAAGATGGCTGATCAAGCTATGACCCAAATGTATAAACAGGC
hCoV-19/USA/DE-CDC-LC0472738/2021_EPI_ISL_9049423...	(12157)	CCGTGATGCAGCCATGCAACGTAAGTTGGAAAAGATGGCTGATCAAGCTATGACCCAAATGTATAAACAGGC

Section 172

	(12313)	12313 12320 12330 12340 12350 12360 12370 12384
SARS-CoV-2 Reference Genome NC_045512.2	(12313)	TAGATCTGAGGACAAGAGGGCAAAAGTTACTAGTGCTATGCAGACAATGCTTTTCACTATGCTTAGAAAGTT
hCoV-19/Botswana/R69B55_BHP_916539/2021_EPI_ISL_90...	(12259)	TAGATCTGAGGACAAGAGGGCAAAAGTTACTAGTGCTATGCAGACAATGCTTTTCACTATGCTTAGAAAGTT
hCoV-19/India/HR-MDU-IGIB1210605800489930/2022_EPI...	(12301)	TAGATCTGAGGACAAGAGGGCAAAAGTTACTAGTGCTATGCAGACAATGCTTTTCACTATGCTTAGAAAGTT
hCoV-19/England/PLYM-332B917/2022_EPI_ISL_9062229_...	(12251)	TAGATCTGAGGACAAGAGGGCAAAAGTTACTAGTGCTATGCAGACAATGCTTTTCACTATGCTTAGAAAGTT
hCoV-19/Germany/HE-RKI-I-438397/2022_EPI_ISL_905522...	(12265)	TAGATCTGAGGACAAGAGGGCAAAAGTTACTAGTGCTATGCAGACAATGCTTTTCACTATGCTTAGAAAGTT
hCoV-19/USA/DE-CDC-LC0472738/2021_EPI_ISL_9049423...	(12229)	TAGATCTGAGGACAAGAGGGCAAAAGTTACTAGTGCTATGCAGACAATGCTTTTCACTATGCTTAGAAAGTT

Omicron BA.1

Section 173

	(12385)	12385 12390 12400 12410 12420 12430 12440 12456
SARS-CoV-2 Reference Genome NC_045512.2	(12385)	GGATAATGATGCACTCAACAACATTATCAACAATGCAAGAGATGGTTGTGTTCCCTTGAACATAATACCTCT
hCoV-19/Botswana/R69B55_BHP_916539/2021_EPI_ISL_90...	(12331)	GGATAATGATGCACTCAACAACATTATCAACAATGCAAGAGATGGTTGTGTTCCCTTGAACATAATACCTCT
hCoV-19/India/HR-MDU-IGIB1210605800489930/2022_EPI...	(12373)	GGATAATGATGCACTCAACAACATTATCAACAATGCAAGAGATGGTTGTGTTCCCTTGAACATAATACCTCT
hCoV-19/England/PLYM-332B917/2022_EPI_ISL_9062229_...	(12323)	GGATAATGATGCACTCAACAACATTATCAACAATGCAAGAGATGGTTGTGTTCCCTTGAACATAATACCTCT
hCoV-19/Germany/HE-RKI-I-438397/2022_EPI_ISL_905522...	(12337)	GGATAATGATGCACTCAACAACATTATCAACAATGCAAGAGATGGTTGTGTTCCCTTGAACATAATACCTCT
hCoV-19/USA/DE-CDC-LC0472738/2021_EPI_ISL_9049423...	(12301)	GGATAATGATGCACTCAACAACATTATCAACAATGCAAGAGATGGTTGTGTTCCCTTGAACATAATACCTCT

Section 174

	(12457)	12457 12470 12480 12490 12500 12510 12528
SARS-CoV-2 Reference Genome NC_045512.2	(12457)	TACAACAGCAGCCAAACTAATGGTTGTCATACCAGACTATAACACATATAAAAATACGTGTGATGGTACAAC
hCoV-19/Botswana/R69B55_BHP_916539/2021_EPI_ISL_90...	(12403)	TACAACAGCAGCCAAACTAATGGTTGTCATACCAGACTATAACACATATAAAAATACGTGTGATGGTACAAC
hCoV-19/India/HR-MDU-IGIB1210605800489930/2022_EPI...	(12445)	TACAACAGCAGCCAAACTAATGGTTGTCATACCAGACTATAACACATATAAAAATACGTGTGATGGTACAAC
hCoV-19/England/PLYM-332B917/2022_EPI_ISL_9062229_...	(12395)	TACAACAGCAGCCAAACTAATGGTTGTCATACCAGACTATAACACATATAAAAATACGTGTGATGGTACAAC
hCoV-19/Germany/HE-RKI-I-438397/2022_EPI_ISL_905522...	(12409)	TACAACAGCAGCCAAACTAATGGTTGTCATACCAGACTATAACACATATAAAAATACGTGTGATGGTACAAC
hCoV-19/USA/DE-CDC-LC0472738/2021_EPI_ISL_9049423...	(12373)	TACAACAGCAGCCAAACTAATGGTTGTCATACCAGACTATAACACATATAAAAATACGTGTGATGGTACAAC

Section 175

	(12529)	12529 12540 12550 12560 12570 12580 12590 12600
SARS-CoV-2 Reference Genome NC_045512.2	(12529)	ATTTACTTATGCATCAGCATTGTGGGAAATCCAACAGGTTGTAGATGCAGATAGTAAAATTGTTCAACTTAG
hCoV-19/Botswana/R69B55_BHP_916539/2021_EPI_ISL_90...	(12475)	ATTTACTTATGCATCAGCATTGTGGGAAATCCAACAGGTTGTAGATGCAGATAGTAAAATTGTTCAACTTAG
hCoV-19/India/HR-MDU-IGIB1210605800489930/2022_EPI...	(12517)	ATTTACTTATGCATCAGCATTGTGGGAAATCCAACAGGTTGTAGATGCAGATAGTAAAATTGTTCAACTTAG
hCoV-19/England/PLYM-332B917/2022_EPI_ISL_9062229_...	(12467)	ATTTACTTATGCATCAGCATTGTGGGAAATCCAACAGGTTGTAGATGCAGATAGTAAAATTGTTCAACTTAG
hCoV-19/Germany/HE-RKI-I-438397/2022_EPI_ISL_905522...	(12481)	ATTTACTTATGCATCAGCATTGTGGGAAATCCAACAGGTTGTAGATGCAGATAGTAAAATTGTTCAACTTAG
hCoV-19/USA/DE-CDC-LC0472738/2021_EPI_ISL_9049423...	(12445)	ATTTACTTATGCATCAGCATTGTGGGAAATCCAACAGGTTGTAGATGCAGATAGTAAAATTGTTCAACTTAG

Section 176

	(12601)	12601 12610 12620 12630 12640 12650 12660 12672
SARS-CoV-2 Reference Genome NC_045512.2	(12601)	TGAAATTAGTATGGACAATTCACCTAATTTAGCATGGCCTCTTATTGTAACAGCTTTAAGGGCCAATTCTGC
hCoV-19/Botswana/R69B55_BHP_916539/2021_EPI_ISL_90...	(12547)	TGAAATTAGTATGGACAATTCACCTAATTTAGCATGGCCTCTTATTGTAACAGCTTTAAGGGCCAATTCTGC
hCoV-19/India/HR-MDU-IGIB1210605800489930/2022_EPI...	(12589)	TGAAATTAGTATGGACAATTCACCTAATTTAGCATGGCCTCTTATTGTAACAGCTTTAAGGGCCAATTCTGC
hCoV-19/England/PLYM-332B917/2022_EPI_ISL_9062229_...	(12539)	TGAAATTAGTATGGACAATTCACCTAATTTAGCATGGCCTCTTATTGTAACAGCTTTAAGGGCCAATTCTGC
hCoV-19/Germany/HE-RKI-I-438397/2022_EPI_ISL_905522...	(12553)	TGAAATTAGTATGGACAATTCACCTAATTTAGCATGGCCTCTTATTGTAACAGCTTTAAGGGCCAATTCTGC
hCoV-19/USA/DE-CDC-LC0472738/2021_EPI_ISL_9049423...	(12517)	TGAAATTAGTATGGACAATTCACCTAATTTAGCATGGCCTCTTATTGTAACAGCTTTAAGGGCCAATTCTGC

Section 177

		12673 12680 12690 12700 12710 12720 12730 12744
	(12673)	
SARS-CoV-2 Reference Genome NC_045512.2	(12673)	TGTCAAATTACAGAATAATGAGCTTAGTCCTGTTGCACTACGACAGATGTCTTGTGCTGCCGGTACTACACA
hCoV-19/Botswana/R69B55_BHP_916539/2021_EPI_ISL_90...	(12619)	TGTCAAATTACAGAATAATGAGCTTAGTCCTGTTGCACTACGACAGATGTCTTGTGCTGCCGGTACTACACA
hCoV-19/India/HR-MDU-IGIB1210605800489930/2022_EPI...	(12661)	TGTCAAATTACAGAATAATGAGCTTAGTCCTGTTGCACTACGACAGATGTCTTGTGCTGCCGGTACTACACA
hCoV-19/England/PLYM-332B917/2022_EPI_ISL_9062229_...	(12611)	TGTCAAATTACAGAATAATGAGCTTAGTCCTGTTGCACTACGACAGATGTCTTGTGCTGCCGGTACTACACA
hCoV-19/Germany/HE-RKI-I-438397/2022_EPI_ISL_905522...	(12625)	TGTCAAATTACAGAATAATGAGCTTAGTCCTGTTGCACTACGACAGATGTCTTGTGCTGCCGGTACTACACA
hCoV-19/USA/DE-CDC-LC0472738/2021_EPI_ISL_9049423...	(12589)	TGTCAAATTACAGAATAATGAGCTTAGTCCTGTTGCACTACGACAGATGTCTTGTGCTGCCGGTACTACACA

Section 178

		12745 12750 12760 12770 12780 12790 12800 12816
	(12745)	
SARS-CoV-2 Reference Genome NC_045512.2	(12745)	AACTGCTTGCACTGATGACAATGCGTTAGCTTACTACAACACAACAAAGGGAGGTAGGTTTGTACTTGCACT
hCoV-19/Botswana/R69B55_BHP_916539/2021_EPI_ISL_90...	(12691)	AACTGCTTGCACTGATGACAATGCGTTAGCTTACTACAACACAACAAAGGGAGGTAGGTTTGTACTTGCACT
hCoV-19/India/HR-MDU-IGIB1210605800489930/2022_EPI...	(12733)	AACTGCTTGCACTGATGACAATGCGTTAGCTTACTACAACACAACAAAGGGAGGTAGGTTTGTACTTGCACT
hCoV-19/England/PLYM-332B917/2022_EPI_ISL_9062229_...	(12683)	AACTGCTTGCACTGATGACAATGCGTTAGCTTACTACAACACAACAAAGGGAGGTAGGTTTGTACTTGCACT
hCoV-19/Germany/HE-RKI-I-438397/2022_EPI_ISL_905522...	(12697)	AACTGCTTGCACTGATGACAATGCGTTAGCTTACTACAACACAACAAAGGGAGGTAGGTTTGTACTTGCACT
hCoV-19/USA/DE-CDC-LC0472738/2021_EPI_ISL_9049423...	(12661)	AACTGCTTGCACTGATGACAATGCGTTAGCTTACTACAACACAACAAAGGGAGGTAGGTTTGTACTTGCACT

Section 179

		12817 12830 12840 12850 12860 12870 12888
	(12817)	
SARS-CoV-2 Reference Genome NC_045512.2	(12817)	GTTATCCGATTTACAGGATTTGAAATGGGCTAGATTCCCTAAGAGTGATGGAACTGGTACTATCTATACAGA
hCoV-19/Botswana/R69B55_BHP_916539/2021_EPI_ISL_90...	(12763)	GTTATCCGATTTACAGGATTTGAAATGGGCTAGATTCCCTAAGAGTGATGGAACTGGTACTATCTATACAGA
hCoV-19/India/HR-MDU-IGIB1210605800489930/2022_EPI...	(12805)	GTTATCCGATTTACAGGATTTGAAATGGGCTAGATTCCCTAAGAGTGATGGAACTGGTACTATCTATACAGA
hCoV-19/England/PLYM-332B917/2022_EPI_ISL_9062229_...	(12755)	GTTATCCGATTTACAGGATTTGAAATGGGCTAGATTCCCTAAGAGTGATGGAACTGGTACTATCTATACAGA
hCoV-19/Germany/HE-RKI-I-438397/2022_EPI_ISL_905522...	(12769)	GTTATCCGATTTACAGGATTTGAAATGGGCTAGATTCCCTAAGAGTGATGGAACTGGTACTATCTATACAGA
hCoV-19/USA/DE-CDC-LC0472738/2021_EPI_ISL_9049423...	(12733)	GTTATCCGATTTACAGGATTTGAAATGGGCTAGATTCCCTAAGAGTGATGGAACTGGTACTATCTATACAGA

Section 180

		12889 12900 12910 12920 12930 12940 12950 12960
	(12889)	
SARS-CoV-2 Reference Genome NC_045512.2	(12889)	ACTGGAACCACCTTGTAGGTTTGTTACAGACACACCTAAAGGTCCTAAAGTGAAGTATTTATACTTTATTAA
hCoV-19/Botswana/R69B55_BHP_916539/2021_EPI_ISL_90...	(12835)	ACTGGAACCACCTTGTAGGTTTGTTACAGACACACCTAAAGGTCCTAAAGTGAAGTATTTATACTTTATTAA
hCoV-19/India/HR-MDU-IGIB1210605800489930/2022_EPI...	(12877)	ACTGGAACCACCTTGTAGGTTTGTTACAGACACACCTAAAGGTCCTAAAGTGAAGTATTTATACTTTATTAA
hCoV-19/England/PLYM-332B917/2022_EPI_ISL_9062229_...	(12827)	ACTGGAACCACCTTGTAGGTTTGTTACAGACACACCTAAAGGTCCTAAAGTGAAGTATTTATACTTTATTAA
hCoV-19/Germany/HE-RKI-I-438397/2022_EPI_ISL_905522...	(12841)	ACTGGAACCACCTTGTAGGTTTGTTACAGACACACCTAAAGGTCCTAAAGTGAAGTATTTATACTTTATTAA
hCoV-19/USA/DE-CDC-LC0472738/2021_EPI_ISL_9049423...	(12805)	ACTGGAACCACCTTGTAGGTTTGTTACAGACACACCTAAAGGTCCTAAAGTGAAGTATTTATACTTTATTAA

Omicron BA.1

```
                                                                                                                                   Section 181
                                                                  (12961) 12961    12970     12980     12990     13000     13010     13020      13032
                       SARS-CoV-2 Reference Genome NC_045512.2 (12961) AGGATTAAACAACCTAAATAGAGGTATGGTACTTGGTAGTTTAGCTGCCACAGTACGTCTACAAGCTGGTAA
hCoV-19/Botswana/R69B55_BHP_916539/2021_EPI_ISL_90... (12907) AGGATTAAACAACCTAAATAGAGGTATGGTACTTGGTAGTTTAGCTGCCACAGTACGTCTACAAGCTGGTAA
 hCoV-19/India/HR-MDU-IGIB1210605800489930/2022_EPI... (12949) AGGATTAAACAACCTAAATAGAGGTATGGTACTTGGTAGTTTAGCTGCCACAGTACGTCTACAAGCTGGTAA
  hCoV-19/England/PLYM-332B917/2022_EPI_ISL_9062229_... (12899) AGGATTAAACAACCTAAATAGAGGTATGGTACTTGGTAGTTTAGCTGCCACAGTACGTCTACAAGCTGGTAA
hCoV-19/Germany/HE-RKI-I-438397/2022_EPI_ISL_905522... (12913) AGGATTAAACAACCTAAATAGAGGTATGGTACTTGGTAGTTTAGCTGCCACAGTACGTCTACAAGCTGGTAA
 hCoV-19/USA/DE-CDC-LC0472738/2021_EPI_ISL_9049423... (12877) AGGATTAAACAACCTAAATAGAGGTATGGTACTTGGTAGTTTAGCTGCCACAGTACGTCTACAAGCTGGTAA
                                                                                                                                   Section 182
                                                                  (13033) 13033  13040     13050     13060     13070     13080     13090      13104
                       SARS-CoV-2 Reference Genome NC_045512.2 (13033) TGCAACAGAAGTGCCTGCCAATTCAACTGTATTATCTTTCTGTGCTTTTGCTGTAGATGCTGCTAAAGCTTA
hCoV-19/Botswana/R69B55_BHP_916539/2021_EPI_ISL_90... (12979) TGCAACAGAAGTGCCTGCCAATTCAACTGTATTATCTTTCTGTGCTTTTGCTGTAGATGCTGCTAAAGCTTA
 hCoV-19/India/HR-MDU-IGIB1210605800489930/2022_EPI... (13021) TGCAACAGAAGTGCCTGCCAATTCAACTGTATTATCTTTCTGTGCTTTTGCTGTAGATGCTGCTAAAGCTTA
  hCoV-19/England/PLYM-332B917/2022_EPI_ISL_9062229_... (12971) TGCAACAGAAGTGCCTGCCAATTCAACTGTATTATCTTTCTGTGCTTTTGCTGTAGATGCTGCTAAAGCTTA
hCoV-19/Germany/HE-RKI-I-438397/2022_EPI_ISL_905522... (12985) TGCAACAGAAGTGCCTGCCAATTCAACTGTATTATCTTTCTGTGCTTTTGCTGTAGATGCTGCTAAAGCTTA
 hCoV-19/USA/DE-CDC-LC0472738/2021_EPI_ISL_9049423... (12949) TGCAACAGAAGTGCCTGCCAATTCAACTGTATTATCTTTCTGTGCTTTTGCTGTAGATGCTGCTAAAGCTTA
                                                                                                                                   Section 183
                                                                  (13105) 13105 13110     13120     13130     13140     13150     13160       13176
                       SARS-CoV-2 Reference Genome NC_045512.2 (13105) CAAAGATTATCTAGCTAGTGGGGGACAACCAATCACTAATTGTGTTAAGATGTTGTGTACACACACTGGTAC
hCoV-19/Botswana/R69B55_BHP_916539/2021_EPI_ISL_90... (13051) CAAAGATTATCTAGCTAGTGGGGGACAACCAATCACTAATTGTGTTAAGATGTTGTGTACACACACTGGTAC
 hCoV-19/India/HR-MDU-IGIB1210605800489930/2022_EPI... (13093) CAAAGATTATCTAGCTAGTGGGGGACAACCAATCACTAATTGTGTTAAGATGTTGTGTACACACACTGGTAC
  hCoV-19/England/PLYM-332B917/2022_EPI_ISL_9062229_... (13043) CAAAGATTATCTAGCTAGTGGGGGACAACCAATCACTAATTGTGTTAAGATGTTGTGTACACACACTGGTAC
hCoV-19/Germany/HE-RKI-I-438397/2022_EPI_ISL_905522... (13057) CAAAGATTATCTAGCTAGTGGGGGACAACCAATCACTAATTGTGTTAAGATGTTGTGTACACACACTGGTAC
 hCoV-19/USA/DE-CDC-LC0472738/2021_EPI_ISL_9049423... (13021) CAAAGATTATCTAGCTAGTGGGGGACAACCAATCACTAATTGTGTTAAGATGTTGTGTACACACACTGGTAC
                                                                                                                                   Section 184
                                                                  (13177) 13177         13190     13200     13210     13220     13230         13248
                       SARS-CoV-2 Reference Genome NC_045512.2 (13177) TGGTCAGGCAATAACAGTTACACCGGAAGCCAATATGGATCAAGAATCCTTTGGTGGTGCATCGTGTTGTCT
hCoV-19/Botswana/R69B55_BHP_916539/2021_EPI_ISL_90... (13123) TGGTCAGGCAATAACAGTCACACCGGAAGCCAATATGGATCAAGAATCCTTTGGTGGTGCATCGTGTTGTCT
 hCoV-19/India/HR-MDU-IGIB1210605800489930/2022_EPI... (13165) TGGTCAGGCAATAACAGTCACACCGGAAGCCAATATGGATCAAGAATCCTTTGGTGGTGCATCGTGTTGTCT
  hCoV-19/England/PLYM-332B917/2022_EPI_ISL_9062229_... (13115) TGGTCAGGCAATAACAGTCACACCGGAAGCCAATATGGATCAAGAATCCTTTGGTGGTGCATCGTGTTGTCT
hCoV-19/Germany/HE-RKI-I-438397/2022_EPI_ISL_905522... (13129) TGGTCAGGCAATAACAGTCACACCGGAAGCCAATATGGATCAAGAATCCTTTGGTGGTGCATCGTGTTGTCT
 hCoV-19/USA/DE-CDC-LC0472738/2021_EPI_ISL_9049423... (13093) TGGTCAGGCAATAACAGTCACACCGGAAGCCAATATGGATCAAGAATCCTTTGGTGGTGCATCGTGTTGTCT
```

Section 185

	(13249)	13249 13260 13270 13280 13290 13300 13310 13320
SARS-CoV-2 Reference Genome NC_045512.2	(13249)	GTACTGCCGTTGCCACATAGATCATCCAAATCCTAAAGGATTTTGTGACTTAAAAGGTAAGTATGTACAAAT
hCoV-19/Botswana/R69B55_BHP_916539/2021_EPI_ISL_90...	(13195)	GTACTGCCGTTGCCACATAGATCATCCAAATCCTAAAGGATTTTGTGACTTAAAAGGTAAGTATGTACAAAT
hCoV-19/India/HR-MDU-IGIB1210605800489930/2022_EPI...	(13237)	GTACTGCCGTTGCCACATAGATCATCCAAATCCTAAAGGATTTTGTGACTTAAAAGGTAAGTATGTACAAAT
hCoV-19/England/PLYM-332B917/2022_EPI_ISL_9062229_...	(13187)	GTACTGCCGTTGCCACATAGATCATCCAAATCCTAAAGGATTTTGTGACTTAAAAGGTAAGTATGTACAAAT
hCoV-19/Germany/HE-RKI-I-438397/2022_EPI_ISL_905522...	(13201)	GTACTGCCGTTGCCACATAGATCATCCAAATCCTAAAGGATTTTGTGACTTAAAAGGTAAGTATGTACAAAT
hCoV-19/USA/DE-CDC-LC0472738/2021_EPI_ISL_9049423...	(13165)	GTACTGCCGTTGCCACATAGATCATCCAAATCCTAAAGGATTTTGTGACTTAAAAGGTAAGTATGTACAAAT

Section 186

	(13321)	13321 13330 13340 13350 13360 13370 13380 13392
SARS-CoV-2 Reference Genome NC_045512.2	(13321)	ACCTACAACTTGTGCTAATGACCCTGTGGGTTTTACACTTAAAAACACAGTCTGTACCGTCTGCGGTATGTG
hCoV-19/Botswana/R69B55_BHP_916539/2021_EPI_ISL_90...	(13267)	ACCTACAACTTGTGCTAATGACCCTGTGGGTTTTACACTTAAAAACACAGTCTGTACCGTCTGCGGTATGTG
hCoV-19/India/HR-MDU-IGIB1210605800489930/2022_EPI...	(13309)	ACCTACAACTTGTGCTAATGACCCTGTGGGTTTTACACTTAAAAACACAGTCTGTACCGTCTGCGGTATGTG
hCoV-19/England/PLYM-332B917/2022_EPI_ISL_9062229_...	(13259)	ACCTACAACTTGTGCTAATGACCCTGTGGGTTTTACACTTAAAAACACAGTCTGTACCGTCTGCGGTATGTG
hCoV-19/Germany/HE-RKI-I-438397/2022_EPI_ISL_905522...	(13273)	ACCTACAACTTGTGCTAATGACCCTGTGGGTTTTACACTTAAAAACACAGTCTGTACCGTCTGCGGTATGTG
hCoV-19/USA/DE-CDC-LC0472738/2021_EPI_ISL_9049423...	(13237)	ACCTACAACTTGTGCTAATGACCCTGTGGGTTTTACACTTAAAAACACAGTCTGTACCGTCTGCGGTATGTG

Section 187

	(13393)	13393 13400 13410 13420 13430 13440 13450 13464
SARS-CoV-2 Reference Genome NC_045512.2	(13393)	GAAAGGTTATGGCTGTAGTTGTGATCAACTCCGCGAACCCATGCTTCAGTCAGCTGATGCACAATCGTTTTT
hCoV-19/Botswana/R69B55_BHP_916539/2021_EPI_ISL_90...	(13339)	GAAAGGTTATGGCTGTAGTTGTGATCAACTCCGCGAACCCATGCTTCAGTCAGCTGATGCACAATCGTTTTT
hCoV-19/India/HR-MDU-IGIB1210605800489930/2022_EPI...	(13381)	GAAAGGTTATGGCTGTAGTTGTGATCAACTCCGCGAACCCATGCTTCAGTCAGCTGATGCACAATCGTTTTT
hCoV-19/England/PLYM-332B917/2022_EPI_ISL_9062229_...	(13331)	GAAAGGTTATGGCTGTAGTTGTGATCAACTCCGCGAACCCATGCTTCAGTCAGCTGATGCACAATCGTTTTT
hCoV-19/Germany/HE-RKI-I-438397/2022_EPI_ISL_905522...	(13345)	GAAAGGTTATGGCTGTAGTTGTGATCAACTCCGCGAACCCATGCTTCAGTCAGCTGATGCACAATCGTTTTT
hCoV-19/USA/DE-CDC-LC0472738/2021_EPI_ISL_9049423...	(13309)	GAAAGGTTATGGCTGTAGTTGTGATCAACTCCGCGAACCCATGCTTCAGTCAGCTGATGCACAATCGTTTTT

Section 188

	(13465)	13465 13470 13480 13490 13500 13510 13520 13536
SARS-CoV-2 Reference Genome NC_045512.2	(13465)	AAACGGGTTTGCGGTGTAAGTGCAGCCCGTCTTACACCGTGCGGCACAGGCACTAGTACTGATGTCGTATAC
hCoV-19/Botswana/R69B55_BHP_916539/2021_EPI_ISL_90...	(13411)	AAACGGGTTTGCGGTGTAAGTGCAGCCCGTCTTACACCGTGCGGCACAGGCACTAGTACTGATGTCGTATAC
hCoV-19/India/HR-MDU-IGIB1210605800489930/2022_EPI...	(13453)	AAACGGGTTTGCGGTGTAAGTGCAGCCCGTCTTACACCGTGCGGCACAGGCACTAGTACTGATGTCGTATAC
hCoV-19/England/PLYM-332B917/2022_EPI_ISL_9062229_...	(13403)	AAACGGGTTTGCGGTGTAAGTGCAGCCCGTCTTACACCGTGCGGCACAGGCACTAGTACTGATGTCGTATAC
hCoV-19/Germany/HE-RKI-I-438397/2022_EPI_ISL_905522...	(13417)	AAACGGGTTTGCGGTGTAAGTGCAGCCCGTCTTACACCGTGCGGCACAGGCACTAGTACTGATGTCGTATAC
hCoV-19/USA/DE-CDC-LC0472738/2021_EPI_ISL_9049423...	(13381)	AAACGGGTTTGCGGTGTAAGTGCAGCCCGTCTTACACCGTGCGGCACAGGCACTAGTACTGATGTCGTATAC

Section 189

	(13537)	13537 13550 13560 13570 13580 13590 13608
SARS-CoV-2 Reference Genome NC_045512.2	(13537)	AGGGCTTTTGACATCTACAATGATAAAGTAGCTGGTTTTGCTAAATTCCTAAAAACTAATTGTTGTCGCTTC
hCoV-19/Botswana/R69B55_BHP_916539/2021_EPI_ISL_90...	(13483)	AGGGCTTTTGACATCTACAATGATAAAGTAGCTGGTTTTGCTAAATTCCTAAAAACTAATTGTTGTCGCTTC
hCoV-19/India/HR-MDU-IGIB1210605800489930/2022_EPI...	(13525)	AGGGCTTTTGACATCTACAATGATAAAGTAGCTGGTTTTGCTAAATTCCTAAAAACTAATTGTTGTCGCTTC
hCoV-19/England/PLYM-332B917/2022_EPI_ISL_9062229_...	(13475)	AGGGCTTTTGACATCTACAATGATAAAGTAGCTGGTTTTGCTAAATTCCTAAAAACTAATTGTTGTCGCTTC
hCoV-19/Germany/HE-RKI-I-438397/2022_EPI_ISL_905522...	(13489)	AGGGCTTTTGACATCTACAATGATAAAGTAGCTGGTTTTGCTAAATTCCTAAAAACTAATTGTTGTCGCTTC
hCoV-19/USA/DE-CDC-LC0472738/2021_EPI_ISL_9049423...	(13453)	AGGGCTTTTGACATCTACAATGATAAAGTAGCTGGTTTTGCTAAATTCCTAAAAACTAATTGTTGTCGCTTC

Section 190

	(13609)	13609 13620 13630 13640 13650 13660 13670 13680
SARS-CoV-2 Reference Genome NC_045512.2	(13609)	CAAGAAAAGGACGAAGATGACAATTTAATTGATTCTTACTTTGTAGTTAAGAGACACACTTTCTCTAACTAC
hCoV-19/Botswana/R69B55_BHP_916539/2021_EPI_ISL_90...	(13555)	CAAGAAAAGGACGAAGATGACAATTTAATTGATTCTTACTTTGTAGTTAAGAGACACACTTTCTCTAACTAC
hCoV-19/India/HR-MDU-IGIB1210605800489930/2022_EPI...	(13597)	CAAGAAAAGGACGAAGATGACAATTTAATTGATTCTTACTTTGTAGTTAAGAGACACACTTTCTCTAACTAC
hCoV-19/England/PLYM-332B917/2022_EPI_ISL_9062229_...	(13547)	CAAGAAAAGGACGAAGATGACAATTTAATTGATTCTTACTTTGTAGTTAAGAGACACACTTTCTCTAACTAC
hCoV-19/Germany/HE-RKI-I-438397/2022_EPI_ISL_905522...	(13561)	CAAGAAAAGGACGAAGATGACAATTTAATTGATTCTTACTTTGTAGTTAAGAGACACACTTTCTCTAACTAC
hCoV-19/USA/DE-CDC-LC0472738/2021_EPI_ISL_9049423...	(13525)	CAAGAAAAGGACGAAGATGACAATTTAATTGATTCTTACTTTGTAGTTAAGAGACACACTTTCTCTAACTAC

Section 191

	(13681)	13681 13690 13700 13710 13720 13730 13740 13752
SARS-CoV-2 Reference Genome NC_045512.2	(13681)	CAACATGAAGAAACAATTTATAATTTACTTAAGGATTGTCCAGCTGTTGCTAAACATGACTTCTTTAAGTTT
hCoV-19/Botswana/R69B55_BHP_916539/2021_EPI_ISL_90...	(13627)	CAACATGAAGAAACAATTTATAATTTACTTAAGGATTGTCCAGCTGTTGCTAAACATGACTTCTTTAAGTTT
hCoV-19/India/HR-MDU-IGIB1210605800489930/2022_EPI...	(13669)	CAACATGAAGAAACAATTTATAATTTACTTAAGGATTGTCCAGCTGTTGCTAAACATGACTTCTTTAAGTTT
hCoV-19/England/PLYM-332B917/2022_EPI_ISL_9062229_...	(13619)	CAACATGAAGAAACAATTTATAATTTACTTAAGGATTGTCCAGCTGTTGCTAAACATGACTTCTTTAAGTTT
hCoV-19/Germany/HE-RKI-I-438397/2022_EPI_ISL_905522...	(13633)	CAACATGAAGAAACAATTTATAATTTACTTAAGGATTGTCCAGCTGTTGCTAAACATGACTTCTTTAAGTTT
hCoV-19/USA/DE-CDC-LC0472738/2021_EPI_ISL_9049423...	(13597)	CAACATGAAGAAACAATTTATAATTTACTTAAGGATTGTCCAGCTGTTGCTAAACATGACTTCTTTAAGTTT

Section 192

	(13753)	13753 13760 13770 13780 13790 13800 13810 13824
SARS-CoV-2 Reference Genome NC_045512.2	(13753)	AGAATAGACGGTGACATGGTACCACATATATCACGTCAACGTCTTACTAAATACACAATGGCAGACCTCGTC
hCoV-19/Botswana/R69B55_BHP_916539/2021_EPI_ISL_90...	(13699)	AGAATAGACGGTGACATGGTACCACATATATCACGTCAACGTCTTACTAAATACACAATGGCAGACCTCGTC
hCoV-19/India/HR-MDU-IGIB1210605800489930/2022_EPI...	(13741)	AGAATAGACGGTGACATGGTACCACATATATCACGTCAACGTCTTACTAAATACACAATGGCAGACCTCGTC
hCoV-19/England/PLYM-332B917/2022_EPI_ISL_9062229_...	(13691)	AGAATAGACGGTGACATGGTACCACATATATCACGTCAACGTCTTACTAAATACACAATGGCAGACCTCGTC
hCoV-19/Germany/HE-RKI-I-438397/2022_EPI_ISL_905522...	(13705)	AGAATAGACGGTGACATGGTACCACATATATCACGTCAACGTCTTACTAAATACACAATGGCAGACCTCGTC
hCoV-19/USA/DE-CDC-LC0472738/2021_EPI_ISL_9049423...	(13669)	AGAATAGACGGTGACATGGTACCACATATATCACGTCAACGTCTTACTAAATACACAATGGCAGACCTCGTC

Section 193

	(13825)	13825 13830 13840 13850 13860 13870 13880 13896
SARS-CoV-2 Reference Genome NC_045512.2	(13825)	TATGCTTTAAGGCATTTTGATGAAGGTAATTGTGACACATTAAAAGAAATACTTGTCACATACAATTGTTGT
hCoV-19/Botswana/R69B55_BHP_916539/2021_EPI_ISL_90...	(13771)	TATGCTTTAAGGCATTTTGATGAAGGTAATTGTGACACATTAAAAGAAATACTTGTCACATACAATTGTTGT
hCoV-19/India/HR-MDU-IGIB1210605800489930/2022_EPI...	(13813)	TATGCTTTAAGGCATTTTGATGAAGGTAATTGTGACACATTAAAAGAAATACTTGTCACATACAATTGTTGT
hCoV-19/England/PLYM-332B917/2022_EPI_ISL_9062229_...	(13763)	TATGCTTTAAGGCATTTTGATGAAGGTAATTGTGACACATTAAAAGAAATACTTGTCACATACAATTGTTGT
hCoV-19/Germany/HE-RKI-I-438397/2022_EPI_ISL_905522...	(13777)	TATGCTTTAAGGCATTTTGATGAAGGTAATTGTGACACATTAAAAGAAATACTTGTCACATACAATTGTTGT
hCoV-19/USA/DE-CDC-LC0472738/2021_EPI_ISL_9049423...	(13741)	TATGCTTTAAGGCATTTTGATGAAGGTAATTGTGACACATTAAAAGAAATACTTGTCACATACAATTGTTGT

Section 194

	(13897)	13897 13910 13920 13930 13940 13950 13968
SARS-CoV-2 Reference Genome NC_045512.2	(13897)	GATGATGATTATTTCAATAAAAAGGACTGGTATGATTTTGTAGAAAACCCAGATATATTACGCGTATACGCC
hCoV-19/Botswana/R69B55_BHP_916539/2021_EPI_ISL_90...	(13843)	GATGATGATTATTTCAATAAAAAGGACTGGTATGATTTTGTAGAAAACCCAGATATATTACGCGTATACGCC
hCoV-19/India/HR-MDU-IGIB1210605800489930/2022_EPI...	(13885)	GATGATGATTATTTCAATAAAAAGGACTGGTATGATTTTGTAGAAAACCCAGATATATTACGCGTATACGCC
hCoV-19/England/PLYM-332B917/2022_EPI_ISL_9062229_...	(13835)	GATGATGATTATTTCAATAAAAAGGACTGGTATGATTTTGTAGAAAACCCAGATATATTACGCGTATACGCC
hCoV-19/Germany/HE-RKI-I-438397/2022_EPI_ISL_905522...	(13849)	GATGATGATTATTTCAATAAAAAGGACTGGTATGATTTTGTAGAAAACCCAGATATATTACGCGTATACGCC
hCoV-19/USA/DE-CDC-LC0472738/2021_EPI_ISL_9049423...	(13813)	GATGATGATTATTTCAATAAAAAGGACTGGTATGATTTTGTAGAAAACCCAGATATATTACGCGTATACGCC

Section 195

	(13969)	13969 13980 13990 14000 14010 14020 14030 14040
SARS-CoV-2 Reference Genome NC_045512.2	(13969)	AACTTAGGTGAACGTGTACGCCAAGCTTTGTTAAAAACAGTACAATTCTGTGATGCCATGCGAAATGCTGGT
hCoV-19/Botswana/R69B55_BHP_916539/2021_EPI_ISL_90...	(13915)	AACTTAGGTGAACGTGTACGCCAAGCTTTGTTAAAAACAGTACAATTCTGTGATGCCATGCGAAATGCTGGT
hCoV-19/India/HR-MDU-IGIB1210605800489930/2022_EPI...	(13957)	AACTTAGGTGAACGTGTACGCCAAGCTTTGTTAAAAACAGTACAATTCTGTGATGCCATGCGAAATGCTGGT
hCoV-19/England/PLYM-332B917/2022_EPI_ISL_9062229_...	(13907)	AACTTAGGTGAACGTGTACGCCAAGCTTTGTTAAAAACAGTACAATTCTGTGATGCCATGCGAAATGCTGGT
hCoV-19/Germany/HE-RKI-I-438397/2022_EPI_ISL_905522...	(13921)	AACTTAGGTGAACGTGTACGCCAAGCTTTGTTAAAAACAGTACAATTCTGTGATGCCATGCGAAATGCTGGT
hCoV-19/USA/DE-CDC-LC0472738/2021_EPI_ISL_9049423...	(13885)	AACTTAGGTGAACGTGTACGCCAAGCTTTGTTAAAAACAGTACAATTCTGTGATGCCATGCGAAATGCTGGT

Section 196

	(14041)	14041 14050 14060 14070 14080 14090 14100 14112
SARS-CoV-2 Reference Genome NC_045512.2	(14041)	ATTGTTGGTGTACTGACATTAGATAATCAAGATCTCAATGGTAACTGGTATGATTTCGGTGATTTCATACAA
hCoV-19/Botswana/R69B55_BHP_916539/2021_EPI_ISL_90...	(13987)	ATTGTTGGTGTACTGACATTAGATAATCAAGATCTCAATGGTAACTGGTATGATTTCGGTGATTTCATACAA
hCoV-19/India/HR-MDU-IGIB1210605800489930/2022_EPI...	(14029)	ATTGTTGGTGTACTGACATTAGATAATCAAGATCTCAATGGTAACTGGTATGATTTCGGTGATTTCATACAA
hCoV-19/England/PLYM-332B917/2022_EPI_ISL_9062229_...	(13979)	ATTGTTGGTGTACTGACATTAGATAATCAAGATCTCAATGGTAACTGGTATGATTTCGGTGATTTCATACAA
hCoV-19/Germany/HE-RKI-I-438397/2022_EPI_ISL_905522...	(13993)	ATTGTTGGTGTACTGACATTAGATAATCAAGATCTCAATGGTAACTGGTATGATTTCGGTGATTTCATACAA
hCoV-19/USA/DE-CDC-LC0472738/2021_EPI_ISL_9049423...	(13957)	ATTGTTGGTGTACTGACATTAGATAATCAAGATCTCAATGGTAACTGGTATGATTTCGGTGATTTCATACAA

Section 197

(14113)	14113 14120 14130 14140 14150 14160 14170 14184
SARS-CoV-2 Reference Genome NC_045512.2 (14113)	ACCACGCCAGGTAGTGGAGTTCCTGTTGTAGATTCTTATTATTCATTGTTAATGCCTATATTAACCTTGACC
hCoV-19/Botswana/R69B55_BHP_916539/2021_EPI_ISL_90... (14059)	ACCACGCCAGGTAGTGGAGTTCCTGTTGTAGATTCTTATTATTCATTGTTAATGCCTATATTAACCTTGACC
hCoV-19/India/HR-MDU-IGIB1210605800489930/2022_EPI... (14101)	ACCACGCCAGGTAGTGGAGTTCCTGTTGTAGATTCTTATTATTCATTGTTAATGCCTATATTAACCTTGACC
hCoV-19/England/PLYM-332B917/2022_EPI_ISL_9062229_... (14051)	ACCACGCCAGGTAGTGGAGTTCCTGTTGTAGATTCTTATTATTCATTGTTAATGCCTATATTAACCTTGACC
hCoV-19/Germany/HE-RKI-I-438397/2022_EPI_ISL_905522... (14065)	ACCACGCCAGGTAGTGGAGTTCCTGTTGTAGATTCTTATTATTCATTGTTAATGCCTATATTAACCTTGACC
hCoV-19/USA/DE-CDC-LC0472738/2021_EPI_ISL_9049423... (14029)	ACCACGCCAGGTAGTGGAGTTCCTGTTGTAGATTCTTATTATTCATTGTTAATGCCTATATTAACCTTGACC

Section 198

(14185)	14185 14190 14200 14210 14220 14230 14240 14256
SARS-CoV-2 Reference Genome NC_045512.2 (14185)	AGGGCTTTAACTGCAGAGTCACATGTTGACACTGACTTAACAAAGCCTTACATTAAGTGGGATTTGTTAAAA
hCoV-19/Botswana/R69B55_BHP_916539/2021_EPI_ISL_90... (14131)	AGGGCTTTAACTGCAGAGTCACATGTTGACACTGACTTAACAAAGCCTTACATTAAGTGGGATTTGTTAAAA
hCoV-19/India/HR-MDU-IGIB1210605800489930/2022_EPI... (14173)	AGGGCTTTAACTGCAGAGTCACATGTTGACACTGACTTAACAAAGCCTTACATTAAGTGGGATTTGTTAAAA
hCoV-19/England/PLYM-332B917/2022_EPI_ISL_9062229_... (14123)	AGGGCTTTAACTGCAGAGTCACATGTTGACACTGACTTAACAAAGCCTTACATTAAGTGGGATTTGTTAAAA
hCoV-19/Germany/HE-RKI-I-438397/2022_EPI_ISL_905522... (14137)	AGGGCTTTAACTGCAGAGTCACATGTTGACACTGACTTAACAAAGCCTTACATTAAGTGGGATTTGTTAAAA
hCoV-19/USA/DE-CDC-LC0472738/2021_EPI_ISL_9049423... (14101)	AGGGCTTTAACTGCAGAGTCACATGTTGACACTGACTTAACAAAGCCTTACATTAAGTGGGATTTGTTAAAA

Section 199

(14257)	14257 14270 14280 14290 14300 14310 14328
SARS-CoV-2 Reference Genome NC_045512.2 (14257)	TATGACTTCACGGAAGAGAGGTTAAAACTCTTTGACCGTTATTTTAAATATTGGGATCAGACATACCACCCA
hCoV-19/Botswana/R69B55_BHP_916539/2021_EPI_ISL_90... (14203)	TATGACTTCACGGAAGAGAGGTTAAAACTCTTTGACCGTTATTTTAAATATTGGGATCAGACATACCACCCA
hCoV-19/India/HR-MDU-IGIB1210605800489930/2022_EPI... (14245)	TATGACTTCACGGAAGAGAGGTTAAAACTCTTTGACCGTTATTTTAAATATTGGGATCAGACATACCACCCA
hCoV-19/England/PLYM-332B917/2022_EPI_ISL_9062229_... (14195)	TATGACTTCACGGAAGAGAGGTTAAAACTCTTTGACCGTTATTTTAAATATTGGGATCAGACATACCACCCA
hCoV-19/Germany/HE-RKI-I-438397/2022_EPI_ISL_905522... (14209)	TATGACTTCACGGAAGAGAGGTTAAAACTCTTTGACCGTTATTTTAAATATTGGGATCAGACATACCACCCA
hCoV-19/USA/DE-CDC-LC0472738/2021_EPI_ISL_9049423... (14173)	TATGACTTCACGGAAGAGAGGTTAAAACTCTTTGACCGTTATTTTAAATATTGGGATCAGACATACCACCCA

Section 200

(14329)	14329 14340 14350 14360 14370 14380 14390 14400
SARS-CoV-2 Reference Genome NC_045512.2 (14329)	AATTGTGTTAACTGTTTGGATGACAGATGCATTCTGCATTGTGCAAACTTTAATGTTTTATTCTCTACAGTG
hCoV-19/Botswana/R69B55_BHP_916539/2021_EPI_ISL_90... (14275)	AATTGTGTTAACTGTTTGGATGACAGATGCATTCTGCATTGTGCAAACTTTAATGTTTTATTCTCTACAGTG
hCoV-19/India/HR-MDU-IGIB1210605800489930/2022_EPI... (14317)	AATTGTGTTAACTGTTTGGATGACAGATGCATTCTGCATTGTGCAAACTTTAATGTTTTATTCTCTACAGTG
hCoV-19/England/PLYM-332B917/2022_EPI_ISL_9062229_... (14267)	AATTGTGTTAACTGTTTGGATGACAGATGCATTCTGCATTGTGCAAACTTTAATGTTTTATTCTCTACAGTG
hCoV-19/Germany/HE-RKI-I-438397/2022_EPI_ISL_905522... (14281)	AATTGTGTTAACTGTTTGGATGACAGATGCATTCTGCATTGTGCAAACTTTAATGTTTTATTCTCTACAGTG
hCoV-19/USA/DE-CDC-LC0472738/2021_EPI_ISL_9049423... (14245)	AATTGTGTTAACTGTTTGGATGACAGATGCATTCTGCATTGTGCAAACTTTAATGTTTTATTCTCTACAGTG

Section 201

	(14401)	14401 14410 14420 14430 14440 14450 14460 14472
SARS-CoV-2 Reference Genome NC_045512.2	(14401)	TTCCCACCTACAAGTTTTGGACCACTAGTGAGAAAAATATTTGTTGATGGTGTTCCATTTGTAGTTTCAACT
hCoV-19/Botswana/R69B55_BHP_916539/2021_EPI_ISL_90...	(14347)	TTCCCACTTACAAGTTTTGGACCACTAGTGAGAAAAATATTTGTTGATGGTGTTCCATTTGTAGTTTCAACT
hCoV-19/India/HR-MDU-IGIB1210605800489930/2022_EPI...	(14389)	TTCCCACTTACAAGTTTTGGACCACTAGTGAGAAAAATATTTGTTGATGGTGTTCCATTTGTAGTTTCAACT
hCoV-19/England/PLYM-332B917/2022_EPI_ISL_9062229_...	(14339)	TTCCCACTTACAAGTTTTGGACCACTAGTGAGAAAAATATTTGTTGATGGTGTTCCATTTGTAGTTTCAACT
hCoV-19/Germany/HE-RKI-I-438397/2022_EPI_ISL_905522...	(14353)	TTCCCACTTACAAGTTTTGGACCACTAGTGAGAAAAATATTTGTTGATGGTGTTCCATTTGTAGTTTCAACT
hCoV-19/USA/DE-CDC-LC0472738/2021_EPI_ISL_9049423...	(14317)	TTCCCACTTACAAGTTTTGGACCACTAGTGAGAAAAATATTTGTTGATGGTGTTCCATTTGTAGTTTCAACT

Section 202

	(14473)	14473 14480 14490 14500 14510 14520 14530 14544
SARS-CoV-2 Reference Genome NC_045512.2	(14473)	GGATACCACTTCAGAGAGCTAGGTGTTGTACATAATCAGGATGTAAACTTACATAGCTCTAGACTTAGTTTT
hCoV-19/Botswana/R69B55_BHP_916539/2021_EPI_ISL_90...	(14419)	GGATACCACTTCAGAGAGCTAGGTGTTGTACATAATCAGGATGTAAACTTACATAGCTCTAGACTTAGTTTT
hCoV-19/India/HR-MDU-IGIB1210605800489930/2022_EPI...	(14461)	GGATACCACTTCAGAGAGCTAGGTGTTGTACATAATCAGGATGTAAACTTACATAGCTCTAGACTTAGTTTT
hCoV-19/England/PLYM-332B917/2022_EPI_ISL_9062229_...	(14411)	GGATACCACTTCAGAGAGCTAGGTGTTGTACATAATCAGGATGTAAACTTACATAGCTCTAGACTTAGTTTT
hCoV-19/Germany/HE-RKI-I-438397/2022_EPI_ISL_905522...	(14425)	GGATACCACTTCAGAGAGCTAGGTGTTGTACATAATCAGGATGTAAACTTACATAGCTCTAGACTTAGTTTT
hCoV-19/USA/DE-CDC-LC0472738/2021_EPI_ISL_9049423...	(14389)	GGATACCACTTCAGAGAGCTAGGTGTTGTACATAATCAGGATGTAAACTTACATAGCTCTAGACTTAGTTTT

Section 203

	(14545)	14545 14550 14560 14570 14580 14590 14600 14616
SARS-CoV-2 Reference Genome NC_045512.2	(14545)	AAGGAATTACTTGTGTATGCTGCTGACCCTGCTATGCACGCTGCTTCTGGTAATCTATTACTAGATAAACGC
hCoV-19/Botswana/R69B55_BHP_916539/2021_EPI_ISL_90...	(14491)	AAGGAATTACTTGTGTATGCTGCTGACCCTGCTATGCACGCTGCTTCTGGTAATCTATTACTAGATAAACGC
hCoV-19/India/HR-MDU-IGIB1210605800489930/2022_EPI...	(14533)	AAGGAATTACTTGTGTATGCTGCTGACCCTGCTATGCACGCTGCTTCTGGTAATCTATTACTAGATAAACGC
hCoV-19/England/PLYM-332B917/2022_EPI_ISL_9062229_...	(14483)	AAGGAATTACTTGTGTATGCTGCTGACCCTGCTATGCACGCTGCTTCTGGTAATCTATTACTAGATAAACGC
hCoV-19/Germany/HE-RKI-I-438397/2022_EPI_ISL_905522...	(14497)	AAGGAATTACTTGTGTATGCTGCTGACCCTGCTATGCACGCTGCTTCTGGTAATCTATTACTAGATAAACGC
hCoV-19/USA/DE-CDC-LC0472738/2021_EPI_ISL_9049423...	(14461)	AAGGAATTACTTGTGTATGCTGCTGACCCTGCTATGCACGCTGCTTCTGGTAATCTATTACTAGATAAACGC

Section 204

	(14617)	14617 14630 14640 14650 14660 14670 14688
SARS-CoV-2 Reference Genome NC_045512.2	(14617)	ACTACGTGCTTTTCAGTAGCTGCACTTACTAACAATGTTGCTTTTCAAACTGTCAAACCCGGTAATTTTAAC
hCoV-19/Botswana/R69B55_BHP_916539/2021_EPI_ISL_90...	(14563)	ACTACGTGCTTTTCAGTAGCTGCACTTACTAACAATGTTGCTTTTCAAACTGTCAAACCCGGTAATTTTAAC
hCoV-19/India/HR-MDU-IGIB1210605800489930/2022_EPI...	(14605)	ACTACGTGCTTTTCAGTAGCTGCACTTACTAACAATGTTGCTTTTCAAACTGTCAAACCCGGTAATTTTAAC
hCoV-19/England/PLYM-332B917/2022_EPI_ISL_9062229_...	(14555)	ACTACGTGCTTTTCAGTAGCTGCACTTACTAACAATGTTGCTTTTCAAACTGTCAAACCCGGTAATTTTAAC
hCoV-19/Germany/HE-RKI-I-438397/2022_EPI_ISL_905522...	(14569)	ACTACGTGCTTTTCAGTAGCTGCACTTACTAACAATGTTGCTTTTCAAACTGTCAAACCCGGTAATTTTAAC
hCoV-19/USA/DE-CDC-LC0472738/2021_EPI_ISL_9049423...	(14533)	ACTACGTGCTTTTCAGTAGCTGCACTTACTAACAATGTTGCTTTTCAAACTGTCAAACCCGGTAATTTTAAC

Omicron BA.1

Section 205

(14689)	14689 14700 14710 14720 14730 14740 14750 14760
SARS-CoV-2 Reference Genome NC_045512.2 (14689)	AAAGACTTCTATGACTTTGCTGTGTCTAAGGGTTTCTTTAAGGAAGGAAGTTCTGTTGAATTAAAACACTTC
hCoV-19/Botswana/R69B55_BHP_916539/2021_EPI_ISL_90... (14635)	AAAGACTTCTATGACTTTGCTGTGTCTAAGGGTTTCTTTAAGGAAGGAAGTTCTGTTGAATTAAAACACTTC
hCoV-19/India/HR-MDU-IGIB1210605800489930/2022_EPI... (14677)	AAAGACTTCTATGACTTTGCTGTGTCTAAGGGTTTCTTTAAGGAAGGAAGTTCTGTTGAATTAAAACACTTC
hCoV-19/England/PLYM-332B917/2022_EPI_ISL_9062229_... (14627)	AAAGACTTCTATGACTTTGCTGTGTCTAAGGGTTTCTTTAAGGAAGGAAGTTCTGTTGAATTAAAACACTTC
hCoV-19/Germany/HE-RKI-I-438397/2022_EPI_ISL_905522... (14641)	AAAGACTTCTATGACTTTGCTGTGTCTAAGGGTTTCTTTAAGGAAGGAAGTTCTGTTGAATTAAAACACTTC
hCoV-19/USA/DE-CDC-LC0472738/2021_EPI_ISL_9049423... (14605)	AAAGACTTCTATGACTTTGCTGTGTCTAAGGGTTTCTTTAAGGAAGGAAGTTCTGTTGAATTAAAACACTTC

Section 206

(14761)	14761 14770 14780 14790 14800 14810 14820 14832
SARS-CoV-2 Reference Genome NC_045512.2 (14761)	TTCTTTGCTCAGGATGGTAATGCTGCTATCAGCGATTATGACTACTATCGTTATAATCTACCAACAATGTGT
hCoV-19/Botswana/R69B55_BHP_916539/2021_EPI_ISL_90... (14707)	TTCTTTGCTCAGGATGGTAATGCTGCTATCAGCGATTATGACTACTATCGTTATAATCTACCAACAATGTGT
hCoV-19/India/HR-MDU-IGIB1210605800489930/2022_EPI... (14749)	TTCTTTGCTCAGGATGGTAATGCTGCTATCAGCGATTATGACTACTATCGTTATAATCTACCAACAATGTGT
hCoV-19/England/PLYM-332B917/2022_EPI_ISL_9062229_... (14699)	TTCTTTGCTCAGGATGGTAATGCTGCTATCAGCGATTATGACTACTATCGTTATAATCTACCAACAATGTGT
hCoV-19/Germany/HE-RKI-I-438397/2022_EPI_ISL_905522... (14713)	TTCTTTGCTCAGGATGGTAATGCTGCTATCAGCGATTATGACTACTATCGTTATAATCTACCAACAATGTGT
hCoV-19/USA/DE-CDC-LC0472738/2021_EPI_ISL_9049423... (14677)	TTCTTTGCTCAGGATGGTAATGCTGCTATCAGCGATTATGACTACTATCGTTATAATCTACCAACAATGTGT

Section 207

(14833)	14833 14840 14850 14860 14870 14880 14890 14904
SARS-CoV-2 Reference Genome NC_045512.2 (14833)	GATATCAGACAACTACTATTTGTAGTTGAAGTTGTTGATAAGTACTTTGATTGTTACGATGGTGGCTGTATT
hCoV-19/Botswana/R69B55_BHP_916539/2021_EPI_ISL_90... (14779)	GATATCAGACAACTACTATTTGTAGTTGAAGTTGTTGATAAGTACTTTGATTGTTACGATGGTGGCTGTATT
hCoV-19/India/HR-MDU-IGIB1210605800489930/2022_EPI... (14821)	GATATCAGACAACTACTATTTGTAGTTGAAGTTGTTGATAAGTACTTTGATTGTTACGATGGTGGCTGTATT
hCoV-19/England/PLYM-332B917/2022_EPI_ISL_9062229_... (14771)	GATATCAGACAACTACTATTTGTAGTTGAAGTTGTTGATAAGTACTTTGATTGTTACGATGGTGGCTGTATT
hCoV-19/Germany/HE-RKI-I-438397/2022_EPI_ISL_905522... (14785)	GATATCAGACAACTACTATTTGTAGTTGAAGTTGTTGATAAGTACTTTGATTGTTACGATGGTGGCTGTATT
hCoV-19/USA/DE-CDC-LC0472738/2021_EPI_ISL_9049423... (14749)	GATATCAGACAACTACTATTTGTAGTTGAAGTTGTTGATAAGTACTTTGATTGTTACGATGGTGGCTGTATT

Section 208

(14905)	14905 14910 14920 14930 14940 14950 14960 14976
SARS-CoV-2 Reference Genome NC_045512.2 (14905)	AATGCTAACCAAGTCATCGTCAACAACCTAGACAAATCAGCTGGTTTTCCATTTAATAAATGGGGTAAGGCT
hCoV-19/Botswana/R69B55_BHP_916539/2021_EPI_ISL_90... (14851)	AATGCTAACCAAGTCATCGTCAACAACCTAGACAAATCAGCTGGTTTTCCATTTAATAAATGGGGTAAGGCT
hCoV-19/India/HR-MDU-IGIB1210605800489930/2022_EPI... (14893)	AATGCTAACCAAGTCATCGTCAACAACCTAGACAAATCAGCTGGTTTTCCATTTAATAAATGGGGTAAGGCT
hCoV-19/England/PLYM-332B917/2022_EPI_ISL_9062229_... (14843)	AATGCTAACCAAGTCATCGTCAACAACCTAGACAAATCAGCTGGTTTTCCATTTAATAAATGGGGTAAGGCT
hCoV-19/Germany/HE-RKI-I-438397/2022_EPI_ISL_905522... (14857)	AATGCTAACCAAGTCATCGTCAACAACCTAGACAAATCAGCTGGTTTTCCATTTAATAAATGGGGTAAGGCT
hCoV-19/USA/DE-CDC-LC0472738/2021_EPI_ISL_9049423... (14821)	AATGCTAACCAAGTCATCGTCAACAACCTAGACAAATCAGCTGGTTTTCCATTTAATAAATGGGGTAAGGCT

Omicron BA.1

```
                                                                              Section 209
                                                    (14977) 14977        14990     15000     15010     15020     15030         15048
          SARS-CoV-2 Reference Genome NC_045512.2 (14977) AGACTTTATTATGATTCAATGAGTTATGAGGATCAAGATGCACTTTTCGCATATACAAAACGTAATGTCATC
hCoV-19/Botswana/R69B55_BHP_916539/2021_EPI_ISL_90... (14923) AGACTTTATTATGATTCAATGAGTTATGAGGATCAAGATGCACTTTTCGCATATACAAAACGTAATGTCATC
 hCoV-19/India/HR-MDU-IGIB1210605800489930/2022_EPI... (14965) AGACTTTATTATGATTCAATGAGTTATGAGGATCAAGATGCACTTTTCGCATATACAAAACGTAATGTCATC
  hCoV-19/England/PLYM-332B917/2022_EPI_ISL_9062229_... (14915) AGACTTTATTATGATTCAATGAGTTATGAGGATCAAGATGCACTTTTCGCATATACAAAACGTAATGTCATC
 hCoV-19/Germany/HE-RKI-I-438397/2022_EPI_ISL_905522... (14929) AGACTTTATTATGATTCAATGAGTTATGAGGATCAAGATGCACTTTTCGCATATACAAAACGTAATGTCATC
  hCoV-19/USA/DE-CDC-LC0472738/2021_EPI_ISL_9049423... (14893) AGACTTTATTATGATTCAATGAGTTATGAGGATCAAGATGCACTTTTCGCATATACAAAACGTAATGTCATC
                                                                              Section 210
                                                    (15049) 15049      15060     15070     15080     15090     15100     15110  15120
          SARS-CoV-2 Reference Genome NC_045512.2 (15049) CCTACTATAACTCAAATGAATCTTAAGTATGCCATTAGTGCAAAGAATAGAGCTCGCACCGTAGCTGGTGTC
hCoV-19/Botswana/R69B55_BHP_916539/2021_EPI_ISL_90... (14995) CCTACTATAACTCAAATGAATCTTAAGTATGCCATTAGTGCAAAGAATAGAGCTCGCACCGTAGCTGGTGTC
 hCoV-19/India/HR-MDU-IGIB1210605800489930/2022_EPI... (15037) CCTACTATAACTCAAATGAATCTTAAGTATGCCATTAGTGCAAAGAATAGAGCTCGCACCGTAGCTGGTGTC
  hCoV-19/England/PLYM-332B917/2022_EPI_ISL_9062229_... (14987) CCTACTATAACTCAAATGAATCTTAAGTATGCCATTAGTGCAAAGAATAGAGCTCGCACCGTAGCTGGTGTC
 hCoV-19/Germany/HE-RKI-I-438397/2022_EPI_ISL_905522... (15001) CCTACTATAACTCAAATGAATCTTAAGTATGCCATTAGTGCAAAGAATAGAGCTCGCACCGTAGCTGGTGTC
  hCoV-19/USA/DE-CDC-LC0472738/2021_EPI_ISL_9049423... (14965) CCTACTATAACTCAAATGAATCTTAAGTATGCCATTAGTGCAAAGAATAGAGCTCGCACCGTAGCTGGTGTC
                                                                              Section 211
                                                    (15121) 15121    15130     15140     15150     15160     15170     15180    15192
          SARS-CoV-2 Reference Genome NC_045512.2 (15121) TCTATCTGTAGTACTATGACCAATAGACAGTTTCATCAAAAATTATTGAAATCAATAGCCGCCACTAGAGGA
hCoV-19/Botswana/R69B55_BHP_916539/2021_EPI_ISL_90... (15067) TCTATCTGTAGTACTATGACCAATAGACAGTTTCATCAAAAATTATTGAAATCAATAGCCGCCACTAGAGGA
 hCoV-19/India/HR-MDU-IGIB1210605800489930/2022_EPI... (15109) TCTATCTGTAGTACTATGACCAATAGACAGTTTCATCAAAAATTATTGAAATCAATAGCCGCCACTAGAGGA
  hCoV-19/England/PLYM-332B917/2022_EPI_ISL_9062229_... (15059) TCTATCTGTAGTACTATGACCAATAGACAGTTTCATCAAAAATTATTGAAATCAATAGCCGCCACTAGAGGA
 hCoV-19/Germany/HE-RKI-I-438397/2022_EPI_ISL_905522... (15073) TCTATCTGTAGTACTATGACCAATAGACAGTTTCATCAAAAATTATTGAAATCAATAGCCGCCACTAGAGGA
  hCoV-19/USA/DE-CDC-LC0472738/2021_EPI_ISL_9049423... (15037) TCTATCTGTAGTACTATGACCAATAGACAGTTTCATCAAAAATTATTGAAATCAATAGCCGCCACTAGAGGA
                                                                              Section 212
                                                    (15193) 15193  15200     15210     15220     15230     15240     15250      15264
          SARS-CoV-2 Reference Genome NC_045512.2 (15193) GCTACTGTAGTAATTGGAACAAGCAAATTCTATGGTGGTTGGCACAACATGTTAAAAACTGTTTATAGTGAT
hCoV-19/Botswana/R69B55_BHP_916539/2021_EPI_ISL_90... (15139) GCTACTGTAGTAATTGGAACAAGCAAATTCTATGGTGGTTGGCACAATATGTTAAAAACTGTTTATAGTGAT
 hCoV-19/India/HR-MDU-IGIB1210605800489930/2022_EPI... (15181) GCTACTGTAGTAATTGGAACAAGCAAATTCTATGGTGGTTGGCACAATATGTTAAAAACTGTTTATAGTGAT
  hCoV-19/England/PLYM-332B917/2022_EPI_ISL_9062229_... (15131) GCTACTGTAGTAATTGGAACAAGCAAATTCTATGGTGGTTGGCACAATATGTTAAAAACTGTTTATAGTGAT
 hCoV-19/Germany/HE-RKI-I-438397/2022_EPI_ISL_905522... (15145) GCTACTGTAGTAATTGGAACAAGCAAATTCTATGGTGGTTGGCACAATATGTTAAAAACTGTTTATAGTGAT
  hCoV-19/USA/DE-CDC-LC0472738/2021_EPI_ISL_9049423... (15109) GCTACTGTAGTAATTGGAACAAGCAAATTCTATGGTGGTTGGCACAATATGTTAAAAACTGTTTATAGTGAT
```

Section 213

(15265)	15265 15270 15280 15290 15300 15310 15320 15336
SARS-CoV-2 Reference Genome NC_045512.2 (15265)	GTAGAAAACCCTCACCTTATGGGTTGGGATTATCCTAAATGTGATAGAGCCATGCCTAACATGCTTAGAATT
hCoV-19/Botswana/R69B55_BHP_916539/2021_EPI_ISL_90... (15211)	GTAGAAAACCCTCACCTTATGGGTTGGGATTATCCTAAATGTGATAGAGCCATGCCTAACATGCTTAGAATT
hCoV-19/India/HR-MDU-IGIB1210605800489930/2022_EPI... (15253)	GTAGAAAACCCTCACCTTATGGGTTGGGATTATCCTAAATGTGATAGAGCCATGCCTAACATGCTTAGAATT
hCoV-19/England/PLYM-332B917/2022_EPI_ISL_9062229_... (15203)	GTAGAAAACCCTCACCTTATGGGTTGGGATTATCCTAAATGTGATAGAGCCATGCCTAACATGCTTAGAATT
hCoV-19/Germany/HE-RKI-I-438397/2022_EPI_ISL_905522... (15217)	GTAGAAAACCCTCACCTTATGGGTTGGGATTATCCTAAATGTGATAGAGCCATGCCTAACATGCTTAGAATT
hCoV-19/USA/DE-CDC-LC0472738/2021_EPI_ISL_9049423... (15181)	GTAGAAAACCCTCACCTTATGGGTTGGGATTATCCTAAATGTGATAGAGCCATGCCTAACATGCTTAGAATT

Section 214

(15337)	15337 15350 15360 15370 15380 15390 15408
SARS-CoV-2 Reference Genome NC_045512.2 (15337)	ATGGCCTCACTTGTTCTTGCTCGCAAACATACAACGTGTTGTAGCTTGTCACACCGTTTCTATAGATTAGCT
hCoV-19/Botswana/R69B55_BHP_916539/2021_EPI_ISL_90... (15283)	ATGGCCTCACTTGTTCTTGCTCGCAAACATACAACGTGTTGTAGCTTGTCACACCGTTTCTATAGATTAGCT
hCoV-19/India/HR-MDU-IGIB1210605800489930/2022_EPI... (15325)	ATGGCCTCACTTGTTCTTGCTCGCAAACATACAACGTGTTGTAGCTTGTCACACCGTTTCTATAGATTAGCT
hCoV-19/England/PLYM-332B917/2022_EPI_ISL_9062229_... (15275)	ATGGCCTCACTTGTTCTTGCTCGCAAACATACAACGTGTTGTAGCTTGTCACACCGTTTCTATAGATTAGCT
hCoV-19/Germany/HE-RKI-I-438397/2022_EPI_ISL_905522... (15289)	ATGGCCTCACTTGTTCTTGCTCGCAAACATACAACGTGTTGTAGCTTGTCACACCGTTTCTATAGATTAGCT
hCoV-19/USA/DE-CDC-LC0472738/2021_EPI_ISL_9049423... (15253)	ATGGCCTCACTTGTTCTTGCTCGCAAACATACAACGTGTTGTAGCTTGTCACACCGTTTCTATAGATTAGCT

Section 215

(15409)	15409 15420 15430 15440 15450 15460 15470 15480
SARS-CoV-2 Reference Genome NC_045512.2 (15409)	AATGAGTGTGCTCAAGTATTGAGTGAAATGGTCATGTGTGGCGGTTCACTATATGTTAAACCAGGTGGAACC
hCoV-19/Botswana/R69B55_BHP_916539/2021_EPI_ISL_90... (15355)	AATGAGTGTGCTCAAGTATTGAGTGAAATGGTCATGTGTGGCGGTTCACTATATGTTAAACCAGGTGGAACC
hCoV-19/India/HR-MDU-IGIB1210605800489930/2022_EPI... (15397)	AATGAGTGTGCTCAAGTATTGAGTGAAATGGTCATGTGTGGCGGTTCACTATATGTTAAACCAGGTGGAACC
hCoV-19/England/PLYM-332B917/2022_EPI_ISL_9062229_... (15347)	AATGAGTGTGCTCAAGTATTGAGTGAAATGGTCATGTGTGGCGGTTCACTATATGTTAAACCAGGTGGAACC
hCoV-19/Germany/HE-RKI-I-438397/2022_EPI_ISL_905522... (15361)	AATGAGTGTGCTCAAGTATTGAGTGAAATGGTCATGTGTGGCGGTTCACTATATGTTAAACCAGGTGGAACC
hCoV-19/USA/DE-CDC-LC0472738/2021_EPI_ISL_9049423... (15325)	AATGAGTGTGCTCAAGTATTGAGTGAAATGGTCATGTGTGGCGGTTCACTATATGTTAAACCAGGTGGAACC

Section 216

(15481)	15481 15490 15500 15510 15520 15530 15540 15552
SARS-CoV-2 Reference Genome NC_045512.2 (15481)	TCATCAGGAGATGCCACAACTGCTTATGCTAATAGTGTTTTTAACATTTGTCAAGCTGTCACGGCCAATGTT
hCoV-19/Botswana/R69B55_BHP_916539/2021_EPI_ISL_90... (15427)	TCATCAGGAGATGCCACAACTGCTTATGCTAATAGTGTTTTTAACATTTGTCAAGCTGTCACGGCCAATGTT
hCoV-19/India/HR-MDU-IGIB1210605800489930/2022_EPI... (15469)	TCATCAGGAGATGCCACAACTGCTTATGCTAATAGTGTTTTTAACATTTGTCAAGCTGTCACGGCCAATGTT
hCoV-19/England/PLYM-332B917/2022_EPI_ISL_9062229_... (15419)	TCATCAGGAGATGCCACAACTGCTTATGCTAATAGTGTTTTTAACATTTGTCAAGCTGTCACGGCCAATGTT
hCoV-19/Germany/HE-RKI-I-438397/2022_EPI_ISL_905522... (15433)	TCATCAGGAGATGCCACAACTGCTTATGCTAATAGTGTTTTTAACATTTGTCAAGCTGTCACGGCCAATGTT
hCoV-19/USA/DE-CDC-LC0472738/2021_EPI_ISL_9049423... (15397)	TCATCAGGAGATGCCACAACTGCTTATGCTAATAGTGTTTTTAACATTTGTCAAGCTGTCACGGCCAATGTT

Omicron BA.1

Section 217

		15553 15560 15570 15580 15590 15600 15610 15624
	(15553)	
SARS-CoV-2 Reference Genome NC_045512.2	(15553)	AATGCACTTTTATCTACTGATGGTAACAAAATTGCCGATAAGTATGTCCGCAATTTACAACACAGACTTTAT
hCoV-19/Botswana/R69B55_BHP_916539/2021_EPI_ISL_90...	(15499)	AATGCACTTTTATCTACTGATGGTAACAAAATTGCCGATAAGTATGTCCGCAATTTACAACACAGACTTTAT
hCoV-19/India/HR-MDU-IGIB1210605800489930/2022_EPI...	(15541)	AATGCACTTTTATCTACTGATGGTAACAAAATTGCCGATAAGTATGTCCGCAATTTACAACACAGACTTTAT
hCoV-19/England/PLYM-332B917/2022_EPI_ISL_9062229_...	(15491)	AATGCACTTTTATCTACTGATGGTAACAAAATTGCCGATAAGTATGTCCGCAATTTACAACACAGACTTTAT
hCoV-19/Germany/HE-RKI-I-438397/2022_EPI_ISL_905522...	(15505)	AATGCACTTTTATCTACTGATGGTAACAAAATTGCCGATAAGTATGTCCGCAATTTACAACACAGACTTTAT
hCoV-19/USA/DE-CDC-LC0472738/2021_EPI_ISL_9049423...	(15469)	AATGCACTTTTATCTACTGATGGTAACAAAATTGCCGATAAGTATGTCCGCAATTTACAACACAGACTTTAT

Section 218

		15625 15630 15640 15650 15660 15670 15680 15696
	(15625)	
SARS-CoV-2 Reference Genome NC_045512.2	(15625)	GAGTGTCTCTATAGAAATAGAGATGTTGACACAGACTTTGTGAATGAGTTTTACGCATATTTGCGTAAACAT
hCoV-19/Botswana/R69B55_BHP_916539/2021_EPI_ISL_90...	(15571)	GAGTGTCTCTATAGAAATAGAGATGTTGACACAGACTTTGTGAATGAGTTTTACGCATATTTGCGTAAACAT
hCoV-19/India/HR-MDU-IGIB1210605800489930/2022_EPI...	(15613)	GAGTGTCTCTATAGAAATAGAGATGTTGACACAGACTTTGTGAATGAGTTTTACGCATATTTGCGTAAACAT
hCoV-19/England/PLYM-332B917/2022_EPI_ISL_9062229_...	(15563)	GAGTGTCTCTATAGAAATAGAGATGTTGACACAGACTTTGTGAATGAGTTTTACGCATATTTGCGTAAACAT
hCoV-19/Germany/HE-RKI-I-438397/2022_EPI_ISL_905522...	(15577)	GAGTGTCTCTATAGAAATAGAGATGTTGACACAGACTTTGTGAATGAGTTTTACGCATATTTGCGTAAACAT
hCoV-19/USA/DE-CDC-LC0472738/2021_EPI_ISL_9049423...	(15541)	GAGTGTCTCTATAGAAATAGAGATGTTGACACAGACTTTGTGAATGAGTTTTACGCATATTTGCGTAAACAT

Section 219

		15697 15710 15720 15730 15740 15750 15768
	(15697)	
SARS-CoV-2 Reference Genome NC_045512.2	(15697)	TTCTCAATGATGATACTCTCTGACGATGCTGTTGTGTGTTTCAATAGCACTTATGCATCTCAAGGTCTAGTG
hCoV-19/Botswana/R69B55_BHP_916539/2021_EPI_ISL_90...	(15643)	TTCTCAATGATGATACTCTCTGACGATGCTGTTGTGTGTTTCAATAGCACTTATGCATCTCAAGGTCTAGTG
hCoV-19/India/HR-MDU-IGIB1210605800489930/2022_EPI...	(15685)	TTCTCAATGATGATACTCTCTGACGATGCTGTTGTGTGTTTCAATAGCACTTATGCATCTCAAGGTCTAGTG
hCoV-19/England/PLYM-332B917/2022_EPI_ISL_9062229_...	(15635)	TTCTCAATGATGATACTCTCTGACGATGCTGTTGTGTGTTTCAATAGCACTTATGCATCTCAAGGTCTAGTG
hCoV-19/Germany/HE-RKI-I-438397/2022_EPI_ISL_905522...	(15649)	TTCTCAATGATGATACTCTCTGACGATGCTGTTGTGTGTTTCAATAGCACTTATGCATCTCAAGGTCTAGTG
hCoV-19/USA/DE-CDC-LC0472738/2021_EPI_ISL_9049423...	(15613)	TTCTCAATGATGATACTCTCTGACGATGCTGTTGTGTGTTTCAATAGCACTTATGCATCTCAAGGTCTAGTG

Section 220

		15769 15780 15790 15800 15810 15820 15830 15840
	(15769)	
SARS-CoV-2 Reference Genome NC_045512.2	(15769)	GCTAGCATAAAGAACTTTAAGTCAGTTCTTTATTATCAAAACAATGTTTTTATGTCTGAAGCAAAATGTTGG
hCoV-19/Botswana/R69B55_BHP_916539/2021_EPI_ISL_90...	(15715)	GCTAGCATAAAGAACTTTAAGTCAGTTCTTTATTATCAAAACAATGTTTTTATGTCTGAAGCAAAATGTTGG
hCoV-19/India/HR-MDU-IGIB1210605800489930/2022_EPI...	(15757)	GCTAGCATAAAGAACTTTAAGTCAGTTCTTTATTATCAAAACAATGTTTTTATGTCTGAAGCAAAATGTTGG
hCoV-19/England/PLYM-332B917/2022_EPI_ISL_9062229_...	(15707)	GCTAGCATAAAGAACTTTAAGTCAGTTCTTTATTATCAAAACAATGTTTTTATGTCTGAAGCAAAATGTTGG
hCoV-19/Germany/HE-RKI-I-438397/2022_EPI_ISL_905522...	(15721)	GCTAGCATAAAGAACTTTAAGTCAGTTCTTTATTATCAAAACAATGTTTTTATGTCTGAAGCAAAATGTTGG
hCoV-19/USA/DE-CDC-LC0472738/2021_EPI_ISL_9049423...	(15685)	GCTAGCATAAAGAACTTTAAGTCAGTTCTTTATTATCAAAACAATGTTTTTATGTCTGAAGCAAAATGTTGG

Omicron BA.1

Section 221

		15841 … 15912
	(15841)	15841 15850 15860 15870 15880 15890 15900 15912
SARS-CoV-2 Reference Genome NC_045512.2	(15841)	ACTGAGACTGACCTTACTAAAGGACCTCATGAATTTTGCTCTCAACATACAATGCTAGTTAAACAGGGTGAT
hCoV-19/Botswana/R69B55_BHP_916539/2021_EPI_ISL_90...	(15787)	ACTGAGACTGACCTTACTAAAGGACCTCATGAATTTTGCTCTCAACATACAATGCTAGTTAAACAGGGTGAT
hCoV-19/India/HR-MDU-IGIB1210605800489930/2022_EPI...	(15829)	ACTGAGACTGACCTTACTAAAGGACCTCATGAATTTTGCTCTCAACATACAATGCTAGTTAAACAGGGTGAT
hCoV-19/England/PLYM-332B917/2022_EPI_ISL_9062229_...	(15779)	ACTGAGACTGACCTTACTAAAGGACCTCATGAATTTTGCTCTCAACATACAATGCTAGTTAAACAGGGTGAT
hCoV-19/Germany/HE-RKI-I-438397/2022_EPI_ISL_905522...	(15793)	ACTGAGACTGACCTTACTAAAGGACCTCATGAATTTTGCTCTCAACATACAATGCTAGTTAAACAGGGTGAT
hCoV-19/USA/DE-CDC-LC0472738/2021_EPI_ISL_9049423...	(15757)	ACTGAGACTGACCTTACTAAAGGACCTCATGAATTTTGCTCTCAACATACAATGCTAGTTAAACAGGGTGAT

Section 222

		15913 … 15984
	(15913)	15913 15920 15930 15940 15950 15960 15970 15984
SARS-CoV-2 Reference Genome NC_045512.2	(15913)	GATTATGTGTACCTTCCTTACCCAGATCCATCAAGAATCCTAGGGGCCGGCTGTTTTGTAGATGATATCGTA
hCoV-19/Botswana/R69B55_BHP_916539/2021_EPI_ISL_90...	(15859)	GATTATGTGTACCTTCCTTACCCAGATCCATCAAGAATCCTAGGGGCCGGCTGTTTTGTAGATGATATCGTA
hCoV-19/India/HR-MDU-IGIB1210605800489930/2022_EPI...	(15901)	GATTATGTGTACCTTCCTTACCCAGATCCATCAAGAATCCTAGGGGCCGGCTGTTTTGTAGATGATATCGTA
hCoV-19/England/PLYM-332B917/2022_EPI_ISL_9062229_...	(15851)	GATTATGTGTACCTTCCTTACCCAGATCCATCAAGAATCCTAGGGGCCGGCTGTTTTGTAGATGATATCGTA
hCoV-19/Germany/HE-RKI-I-438397/2022_EPI_ISL_905522...	(15865)	GATTATGTGTACCTTCCTTACCCAGATCCATCAAGAATCCTAGGGGCCGGCTGTTTTGTAGATGATATCGTA
hCoV-19/USA/DE-CDC-LC0472738/2021_EPI_ISL_9049423...	(15829)	GATTATGTGTACCTTCCTTACCCAGATCCATCAAGAATCCTAGGGGCCGGCTGTTTTGTAGATGATATCGTA

Section 223

		15985 … 16056
	(15985)	15985 15990 16000 16010 16020 16030 16040 16056
SARS-CoV-2 Reference Genome NC_045512.2	(15985)	AAAACAGATGGTACACTTATGATTGAACGGTTCGTGTCTTTAGCTATAGATGCTTACCCACTTACTAAACAT
hCoV-19/Botswana/R69B55_BHP_916539/2021_EPI_ISL_90...	(15931)	AAAACAGATGGTACACTTATGATTGAACGGTTCGTGTCTTTAGCTATAGATGCTTACCCACTTACTAAACAT
hCoV-19/India/HR-MDU-IGIB1210605800489930/2022_EPI...	(15973)	AAAACAGATGGTACACTTATGATTGAACGGTTCGTGTCTTTAGCTATAGATGCTTACCCACTTACTAAACAT
hCoV-19/England/PLYM-332B917/2022_EPI_ISL_9062229_...	(15923)	AAAACAGATGGTACACTTATGATTGAACGGTTCGTGTCTTTAGCTATAGATGCTTACCCACTTACTAAACAT
hCoV-19/Germany/HE-RKI-I-438397/2022_EPI_ISL_905522...	(15937)	AAAACAGATGGTACACTTATGATTGAACGGTTCGTGTCTTTAGCTATAGATGCTTACCCACTTACTAAACAT
hCoV-19/USA/DE-CDC-LC0472738/2021_EPI_ISL_9049423...	(15901)	AAAACAGATGGTACACTTATGATTGAACGGTTCGTGTCTTTAGCTATAGATGCTTACCCACTTACTAAACAT

Section 224

		16057 … 16128
	(16057)	16057 16070 16080 16090 16100 16110 16128
SARS-CoV-2 Reference Genome NC_045512.2	(16057)	CCTAATCAGGAGTATGCTGATGTCTTTCATTTGTACTTACAATACATAAGAAAGCTACATGATGAGTTAACA
hCoV-19/Botswana/R69B55_BHP_916539/2021_EPI_ISL_90...	(16003)	CCTAATCAGGAGTATGCTGATGTCTTTCATTTGTACTTACAATACATAAGAAAGCTACATGATGAGTTAACA
hCoV-19/India/HR-MDU-IGIB1210605800489930/2022_EPI...	(16045)	CCTAATCAGGAGTATGCTGATGTCTTTCATTTGTACTTACAATACATAAGAAAGCTACATGATGAGTTAACA
hCoV-19/England/PLYM-332B917/2022_EPI_ISL_9062229_...	(15995)	CCTAATCAGGAGTATGCTGATGTCTTTCATTTGTACTTACAATACATAAGAAAGCTACATGATGAGTTAACA
hCoV-19/Germany/HE-RKI-I-438397/2022_EPI_ISL_905522...	(16009)	CCTAATCAGGAGTATGCTGATGTCTTTCATTTGTACTTACAATACATAAGAAAGCTACATGATGAGTTAACA
hCoV-19/USA/DE-CDC-LC0472738/2021_EPI_ISL_9049423...	(15973)	CCTAATCAGGAGTATGCTGATGTCTTTCATTTGTACTTACAATACATAAGAAAGCTACATGATGAGTTAACA

Section 225

(16129)		16129 16140 16150 16160 16170 16180 16190 16200
SARS-CoV-2 Reference Genome NC_045512.2	(16129)	GGACACATGTTAGACATGTATTCTGTTATGCTTACTAATGATAACACTTCAAGGTATTGGGAACCTGAGTTT
hCoV-19/Botswana/R69B55_BHP_916539/2021_EPI_ISL_90...	(16075)	GGACACATGTTAGACATGTATTCTGTTATGCTTACTAATGATAACACTTCAAGGTATTGGGAACCTGAGTTT
hCoV-19/India/HR-MDU-IGIB1210605800489930/2022_EPI...	(16117)	GGACACATGTTAGACATGTATTCTGTTATGCTTACTAATGATAACACTTCAAGGTATTGGGAACCTGAGTTT
hCoV-19/England/PLYM-332B917/2022_EPI_ISL_9062229_...	(16067)	GGACACATGTTAGACATGTATTCTGTTATGCTTACTAATGATAACACTTCAAGGTATTGGGAACCTGAGTTT
hCoV-19/Germany/HE-RKI-I-438397/2022_EPI_ISL_905522...	(16081)	GGACACATGTTAGACATGTATTCTGTTATGCTTACTAATGATAACACTTCAAGGTATTGGGAACCTGAGTTT
hCoV-19/USA/DE-CDC-LC0472738/2021_EPI_ISL_9049423...	(16045)	GGACACATGTTAGACATGTATTCTGTTATGCTTACTAATGATAACACTTCAAGGTATTGGGAACCTGAGTTT

Section 226

(16201)		16201 16210 16220 16230 16240 16250 16260 16272
SARS-CoV-2 Reference Genome NC_045512.2	(16201)	TATGAGGCTATGTACACACCGCATACAGTCTTACAGGCTGTTGGGGCTTGTGTTCTTTGCAATTCACAGACT
hCoV-19/Botswana/R69B55_BHP_916539/2021_EPI_ISL_90...	(16147)	TATGAGGCTATGTACACACCGCATACAGTCTTACAGGCTGTTGGGGCTTGTGTTCTTTGCAATTCACAGACT
hCoV-19/India/HR-MDU-IGIB1210605800489930/2022_EPI...	(16189)	TATGAGGCTATGTACACACCGCATACAGTCTTACAGGCTGTTGGGGCTTGTGTTCTTTGCAATTCACAGACT
hCoV-19/England/PLYM-332B917/2022_EPI_ISL_9062229_...	(16139)	TATGAGGCTATGTACACACCGCATACAGTCTTACAGGCTGTTGGGGCTTGTGTTCTTTGCAATTCACAGACT
hCoV-19/Germany/HE-RKI-I-438397/2022_EPI_ISL_905522...	(16153)	TATGAGGCTATGTACACACCGCATACAGTCTTACAGGCTGTTGGGGCTTGTGTTCTTTGCAATTCACAGACT
hCoV-19/USA/DE-CDC-LC0472738/2021_EPI_ISL_9049423...	(16117)	TATGAGGCTATGTACACACCGCATACAGTCTTACAGGCTGTTGGGGCTTGTGTTCTTTGCAATTCACAGACT

Section 227

(16273)		16273 16280 16290 16300 16310 16320 16330 16344
SARS-CoV-2 Reference Genome NC_045512.2	(16273)	TCATTAAGATGTGGTGCTTGCATACGTAGACCATTCTTATGTTGTAAATGCTGTTACGACCATGTCATATCA
hCoV-19/Botswana/R69B55_BHP_916539/2021_EPI_ISL_90...	(16219)	TCATTAAGATGTGGTGCTTGCATACGTAGACCATTCTTATGTTGTAAATGCTGTTACGACCATGTCATATCA
hCoV-19/India/HR-MDU-IGIB1210605800489930/2022_EPI...	(16261)	TCATTAAGATGTGGTGCTTGCATACGTAGACCATTCTTATGTTGTAAATGCTGTTACGACCATGTCATATCA
hCoV-19/England/PLYM-332B917/2022_EPI_ISL_9062229_...	(16211)	TCATTAAGATGTGGTGCTTGCATACGTAGACCATTCTTATGTTGTAAATGCTGTTACGACCATGTCATATCA
hCoV-19/Germany/HE-RKI-I-438397/2022_EPI_ISL_905522...	(16225)	TCATTAAGATGTGGTGCTTGCATACGTAGACCATTCTTATGTTGTAAATGCTGTTACGACCATGTCATATCA
hCoV-19/USA/DE-CDC-LC0472738/2021_EPI_ISL_9049423...	(16189)	TCATTAAGATGTGGTGCTTGCATACGTAGACCATTCTTATGTTGTAAATGCTGTTACGACCATGTCATATCA

Section 228

(16345)		16345 16350 16360 16370 16380 16390 16400 16416
SARS-CoV-2 Reference Genome NC_045512.2	(16345)	ACATCACATAAATTAGTCTTGTCTGTTAATCCGTATGTTTGCAATGCTCCAGGTTGTGATGTCACAGATGTG
hCoV-19/Botswana/R69B55_BHP_916539/2021_EPI_ISL_90...	(16291)	ACATCACATAAATTAGTCTTGTCTGTTAATCCGTATGTTTGCAATGCTCCAGGTTGTGATGTCACAGATGTG
hCoV-19/India/HR-MDU-IGIB1210605800489930/2022_EPI...	(16333)	ACATCACATAAATTAGTCTTGTCTGTTAATCCGTATGTTTGCAATGCTCCAGGTTGTGATGTCACAGATGTG
hCoV-19/England/PLYM-332B917/2022_EPI_ISL_9062229_...	(16283)	ACATCACATAAATTAGTCTTGTCTGTTAATCCGTATGTTTGCAATGCTCCAGGTTGTGATGTCACAGATGTG
hCoV-19/Germany/HE-RKI-I-438397/2022_EPI_ISL_905522...	(16297)	ACATCACATAAATTAGTCTTGTCTGTTAATCCGTATGTTTGCAATGCTCCAGGTTGTGATGTCACAGATGTG
hCoV-19/USA/DE-CDC-LC0472738/2021_EPI_ISL_9049423...	(16261)	ACATCACATAAATTAGTCTTGTCTGTTAATCCGTATGTTTGCAATGCTCCAGGTTGTGATGTCACAGATGTG

```
                                                                          Section 229
                                                   (16417) 16417      16430     16440     16450     16460     16470           16488
                SARS-CoV-2 Reference Genome NC_045512.2 (16417) ACTCAACTTTACTTAGGAGGTATGAGCTATTATTGTAAATCACATAAACCACCCATTAGTTTTCCATTGTGT
hCoV-19/Botswana/R69B55_BHP_916539/2021_EPI_ISL_90... (16363) ACTCAACTTTACTTAGGAGGTATGAGCTATTATTGTAAATCACATAAACCACCCATTAGTTTTCCATTGTGT
hCoV-19/India/HR-MDU-IGIB1210605800489930/2022_EPI... (16405) ACTCAACTTTACTTAGGAGGTATGAGCTATTATTGTAAATCACATAAACCACCCATTAGTTTTCCATTGTGT
hCoV-19/England/PLYM-332B917/2022_EPI_ISL_9062229 ... (16355) ACTCAACTTTACTTAGGAGGTATGAGCTATTATTGTAAATCACATAAACCACCCATTAGTTTTCCATTGTGT
hCoV-19/Germany/HE-RKI-I-438397/2022_EPI_ISL_905522... (16369) ACTCAACTTTACTTAGGAGGTATGAGCTATTATTGTAAATCACATAAACCACCCATTAGTTTTCCATTGTGT
hCoV-19/USA/DE-CDC-LC0472738/2021_EPI_ISL_9049423... (16333) ACTCAACTTTACTTAGGAGGTATGAGCTATTATTGTAAATCACATAAACCACCCATTAGTTTTCCATTGTGT
                                                                          Section 230
                                                   (16489) 16489     16500     16510     16520     16530     16540     16550 16560
                SARS-CoV-2 Reference Genome NC_045512.2 (16489) GCTAATGGACAAGTTTTTGGTTTATATAAAAATACATGTGTTGGTAGCGATAATGTTACTGACTTTAATGCA
hCoV-19/Botswana/R69B55_BHP_916539/2021_EPI_ISL_90... (16435) GCTAATGGACAAGTTTTTGGTTTATATAAAAATACATGTGTTGGTAGCGATAATGTTACTGACTTTAATGCA
hCoV-19/India/HR-MDU-IGIB1210605800489930/2022_EPI... (16477) GCTAATGGACAAGTTTTTGGTTTATATAAAAATACATGTGTTGGTAGCGATAATGTTACTGACTTTAATGCA
hCoV-19/England/PLYM-332B917/2022_EPI_ISL_9062229 ... (16427) GCTAATGGACAAGTTTTTGGTTTATATAAAAATACATGTGTTGGTAGCGATAATGTTACTGACTTTAATGCA
hCoV-19/Germany/HE-RKI-I-438397/2022_EPI_ISL_905522... (16441) GCTAATGGACAAGTTTTTGGTTTATATAAAAATACATGTGTTGGTAGCGATAATGTTACTGACTTTAATGCA
hCoV-19/USA/DE-CDC-LC0472738/2021_EPI_ISL_9049423... (16405) GCTAATGGACAAGTTTTTGGTTTATATAAAAATACATGTGTTGGTAGCGATAATGTTACTGACTTTAATGCA
                                                                          Section 231
                                                   (16561) 16561    16570     16580     16590     16600     16610     16620     16632
                SARS-CoV-2 Reference Genome NC_045512.2 (16561) ATTGCAACATGTGACTGGACAAATGCTGGTGATTACATTTTAGCTAACACCTGTACTGAAAGACTCAAGCTT
hCoV-19/Botswana/R69B55_BHP_916539/2021_EPI_ISL_90... (16507) ATTGCAACATGTGACTGGACAAATGCTGGTGATTACATTTTAGCTAACACCTGTACTGAAAGACTCAAGCTT
hCoV-19/India/HR-MDU-IGIB1210605800489930/2022_EPI... (16549) ATTGCAACATGTGACTGGACAAATGCTGGTGATTACATTTTAGCTAACACCTGTACTGAAAGACTCAAGCTT
hCoV-19/England/PLYM-332B917/2022_EPI_ISL_9062229 ... (16499) ATTGCAACATGTGACTGGACAAATGCTGGTGATTACATTTTAGCTAACACCTGTACTGAAAGACTCAAGCTT
hCoV-19/Germany/HE-RKI-I-438397/2022_EPI_ISL_905522... (16513) ATTGCAACATGTGACTGGACAAATGCTGGTGATTACATTTTAGCTAACACCTGTACTGAAAGACTCAAGCTT
hCoV-19/USA/DE-CDC-LC0472738/2021_EPI_ISL_9049423... (16477) ATTGCAACATGTGACTGGACAAATGCTGGTGATTACATTTTAGCTAACACCTGTACTGAAAGACTCAAGCTT
                                                                          Section 232
                                                   (16633) 16633  16640     16650     16660     16670     16680     16690         16704
                SARS-CoV-2 Reference Genome NC_045512.2 (16633) TTTGCAGCAGAAACGCTCAAAGCTACTGAGGAGACATTTAAACTGTCTTATGGTATTGCTACTGTACGTGAA
hCoV-19/Botswana/R69B55_BHP_916539/2021_EPI_ISL_90... (16579) TTTGCAGCAGAAACGCTCAAAGCTACTGAGGAGACATTTAAACTGTCTTATGGTATTGCTACTGTACGTGAA
hCoV-19/India/HR-MDU-IGIB1210605800489930/2022_EPI... (16621) TTTGCAGCAGAAACGCTCAAAGCTACTGAGGAGACATTTAAACTGTCTTATGGTATTGCTACTGTACGTGAA
hCoV-19/England/PLYM-332B917/2022_EPI_ISL_9062229 ... (16571) TTTGCAGCAGAAACGCTCAAAGCTACTGAGGAGACATTTAAACTGTCTTATGGTATTGCTACTGTACGTGAA
hCoV-19/Germany/HE-RKI-I-438397/2022_EPI_ISL_905522... (16585) TTTGCAGCAGAAACGCTCAAAGCTACTGAGGAGACATTTAAACTGTCTTATGGTATTGCTACTGTACGTGAA
hCoV-19/USA/DE-CDC-LC0472738/2021_EPI_ISL_9049423... (16549) TTTGCAGCAGAAACGCTCAAAGCTACTGAGGAGACATTTAAACTGTCTTATGGTATTGCTACTGTACGTGAA
```

Section 233

Sequence	Start	16705 – 16776
	(16705)	
SARS-CoV-2 Reference Genome NC_045512.2	(16705)	GTGCTGTCTGACAGAGAATTACATCTTTCATGGGAAGTTGGTAAACCTAGACCACCACTTAACCGAAATTAT
hCoV-19/Botswana/R69B55_BHP_916539/2021_EPI_ISL_90...	(16651)	GTGCTGTCTGACAGAGAATTACATCTTTCATGGGAAGTTGGTAAACCTAGACCACCACTTAACCGAAATTAT
hCoV-19/India/HR-MDU-IGIB1210605800489930/2022_EPI...	(16693)	GTGCTGTCTGACAGAGAATTACATCTTTCATGGGAAGTTGGTAAACCTAGACCACCACTTAACCGAAATTAT
hCoV-19/England/PLYM-332B917/2022_EPI_ISL_9062229 ...	(16643)	GTGCTGTCTGACAGAGAATTACATCTTTCATGGGAAGTTGGTAAACCTAGACCACCACTTAACCGAAATTAT
hCoV-19/Germany/HE-RKI-I-438397/2022_EPI_ISL_905522...	(16657)	GTGCTGTCTGACAGAGAATTACATCTTTCATGGGAAGTTGGTAAACCTAGACCACCACTTAACCGAAATTAT
hCoV-19/USA/DE-CDC-LC0472738/2021_EPI_ISL_9049423...	(16621)	GTGCTGTCTGACAGAGAATTACATCTTTCATGGGAAGTTGGTAAACCTAGACCACCACTTAACCGAAATTAT

Section 234

Sequence	Start	16777 – 16848
	(16777)	
SARS-CoV-2 Reference Genome NC_045512.2	(16777)	GTCTTTACTGGTTATCGTGTAACTAAAAACAGTAAAGTACAAATAGGAGAGTACACCTTTGAAAAAGGTGAC
hCoV-19/Botswana/R69B55_BHP_916539/2021_EPI_ISL_90...	(16723)	GTCTTTACTGGTTATCGTGTAACTAAAAACAGTAAAGTACAAATAGGAGAGTACACCTTTGAAAAAGGTGAC
hCoV-19/India/HR-MDU-IGIB1210605800489930/2022_EPI...	(16765)	GTCTTTACTGGTTATCGTGTAACTAAAAACAGTAAAGTACAAATAGGAGAGTACACCTTTGAAAAAGGTGAC
hCoV-19/England/PLYM-332B917/2022_EPI_ISL_9062229 ...	(16715)	GTCTTTACTGGTTATCGTGTAACTAAAAACAGTAAAGTACAAATAGGAGAGTACACCTTTGAAAAAGGTGAC
hCoV-19/Germany/HE-RKI-I-438397/2022_EPI_ISL_905522...	(16729)	GTCTTTACTGGTTATCGTGTAACTAAAAACAGTAAAGTACAAATAGGAGAGTACACCTTTGAAAAAGGTGAC
hCoV-19/USA/DE-CDC-LC0472738/2021_EPI_ISL_9049423...	(16693)	GTCTTTACTGGTTATCGTGTAACTAAAAACAGTAAAGTACAAATAGGAGAGTACACCTTTGAAAAAGGTGAC

Section 235

Sequence	Start	16849 – 16920
	(16849)	
SARS-CoV-2 Reference Genome NC_045512.2	(16849)	TATGGTGATGCTGTTGTTTACCGAGGTACAACAACTTACAAATTAAATGTTGGTGATTATTTTGTGCTGACA
hCoV-19/Botswana/R69B55_BHP_916539/2021_EPI_ISL_90...	(16795)	TATGGTGATGCTGTTGTTTACCGAGGTACAACAACTTACAAATTAAATGTTGGTGATTATTTTGTGCTGACA
hCoV-19/India/HR-MDU-IGIB1210605800489930/2022_EPI...	(16837)	TATGGTGATGCTGTTGTTTACCGAGGTACAACAACTTACAAATTAAATGTTGGTGATTATTTTGTGCTGACA
hCoV-19/England/PLYM-332B917/2022_EPI_ISL_9062229 ...	(16787)	TATGGTGATGCTGTTGTTTACCGAGGTACAACAACTTACAAATTAAATGTTGGTGATTATTTTGTGCTGACA
hCoV-19/Germany/HE-RKI-I-438397/2022_EPI_ISL_905522...	(16801)	TATGGTGATGCTGTTGTTTACCGAGGTACAACAACTTACAAATTAAATGTTGGTGATTATTTTGTGCTGACA
hCoV-19/USA/DE-CDC-LC0472738/2021_EPI_ISL_9049423...	(16765)	TATGGTGATGCTGTTGTTTACCGAGGTACAACAACTTACAAATTAAATGTTGGTGATTATTTTGTGCTGACA

Section 236

Sequence	Start	16921 – 16992
	(16921)	
SARS-CoV-2 Reference Genome NC_045512.2	(16921)	TCACATACAGTAATGCCATTAAGTGCACCTACACTAGTGCCACAAGAGCACTATGTTAGAATTACTGGCTTA
hCoV-19/Botswana/R69B55_BHP_916539/2021_EPI_ISL_90...	(16867)	TCACATACAGTAATGCCATTAAGTGCACCTACACTAGTGCCACAAGAGCACTATGTTAGAATTACTGGCTTA
hCoV-19/India/HR-MDU-IGIB1210605800489930/2022_EPI...	(16909)	TCACATACAGTAATGCCATTAAGTGCACCTACACTAGTGCCACAAGAGCACTATGTTAGAATTACTGGCTTA
hCoV-19/England/PLYM-332B917/2022_EPI_ISL_9062229 ...	(16859)	TCACATACAGTAATGCCATTAAGTGCACCTACACTAGTGCCACAAGAGCACTATGTTAGAATTACTGGCTTA
hCoV-19/Germany/HE-RKI-I-438397/2022_EPI_ISL_905522...	(16873)	TCACATACAGTAATGCCATTAAGTGCACCTACACTAGTGCCACAAGAGCACTATGTTAGAATTACTGGCTTA
hCoV-19/USA/DE-CDC-LC0472738/2021_EPI_ISL_9049423...	(16837)	TCACATACAGTAATGCCATTAAGTGCACCTACACTAGTGCCACAAGAGCACTATGTTAGAATTACTGGCTTA

Section 237

		16993 17000 17010 17020 17030 17040 17050 17064
	(16993)	
SARS-CoV-2 Reference Genome NC_045512.2	(16993)	TACCCAACACTCAATATCTCAGATGAGTTTTCTAGCAATGTTGCAAATTATCAAAAGGTTGGTATGCAAAAG
hCoV-19/Botswana/R69B55_BHP_916539/2021_EPI_ISL_90...	(16939)	TACCCAACACTCAATATCTCAGATGAGTTTTCTAGCAATGTTGCAAATTATCAAAAGGTTGGTATGCAAAAG
hCoV-19/India/HR-MDU-IGIB1210605800489930/2022_EPI...	(16981)	TACCCAACACTCAATATCTCAGATGAGTTTTCTAGCAATGTTGCAAATTATCAAAAGGTTGGTATGCAAAAG
hCoV-19/England/PLYM-332B917/2022_EPI_ISL_9062229_...	(16931)	TACCCAACACTCAATATCTCAGATGAGTTTTCTAGCAATGTTGCAAATTATCAAAAGGTTGGTATGCAAAAG
hCoV-19/Germany/HE-RKI-I-438397/2022_EPI_ISL_905522...	(16945)	TACCCAACACTCAATATCTCAGATGAGTTTTCTAGCAATGTTGCAAATTATCAAAAGGTTGGTATGCAAAAG
hCoV-19/USA/DE-CDC-LC0472738/2021_EPI_ISL_9049423...	(16909)	TACCCAACACTCAATATCTCAGATGAGTTTTCTAGCAATGTTGCAAATTATCAAAAGGTTGGTATGCAAAAG

Section 238

		17065 17070 17080 17090 17100 17110 17120 17136
	(17065)	
SARS-CoV-2 Reference Genome NC_045512.2	(17065)	TATTCTACACTCCAGGGACCACCTGGTACTGGTAAGAGTCATTTTGCTATTGGCCTAGCTCTCTACTACCCT
hCoV-19/Botswana/R69B55_BHP_916539/2021_EPI_ISL_90...	(17011)	TATTCTACACTCCAGGGACCACCTGGTACTGGTAAGAGTCATTTTGCTATTGGCCTAGCTCTCTACTACCCT
hCoV-19/India/HR-MDU-IGIB1210605800489930/2022_EPI...	(17053)	TATTCTACACTCCAGGGACCACCTGGTACTGGTAAGAGTCATTTTGCTATTGGCCTAGCTCTCTACTACCCT
hCoV-19/England/PLYM-332B917/2022_EPI_ISL_9062229_...	(17003)	TATTCTACACTCCAGGGACCACCTGGTACTGGTAAGAGTCATTTTGCTATTGGCCTAGCTCTCTACTACCCT
hCoV-19/Germany/HE-RKI-I-438397/2022_EPI_ISL_905522...	(17017)	TATTCTACACTCCAGGGACCACCTGGTACTGGTAAGAGTCATTTTGCTATTGGCCTAGCTCTCTACTACCCT
hCoV-19/USA/DE-CDC-LC0472738/2021_EPI_ISL_9049423...	(16981)	TATTCTACACTCCAGGGACCACCTGGTACTGGTAAGAGTCATTTTGCTATTGGCCTAGCTCTCTACTACCCT

Section 239

		17137 17150 17160 17170 17180 17190 17208
	(17137)	
SARS-CoV-2 Reference Genome NC_045512.2	(17137)	TCTGCTCGCATAGTGTATACAGCTTGCTCTCATGCCGCTGTTGATGCACTATGTGAGAAGGCATTAAAATAT
hCoV-19/Botswana/R69B55_BHP_916539/2021_EPI_ISL_90...	(17083)	TCTGCTCGCATAGTGTATACAGCTTGCTCTCATGCCGCTGTTGATGCACTATGTGAGAAGGCATTAAAATAT
hCoV-19/India/HR-MDU-IGIB1210605800489930/2022_EPI...	(17125)	TCTGCTCGCATAGTGTATACAGCTTGCTCTCATGCCGCTGTTGATGCACTATGTGAGAAGGCATTAAAATAT
hCoV-19/England/PLYM-332B917/2022_EPI_ISL_9062229_...	(17075)	TCTGCTCGCATAGTGTATACAGCTTGCTCTCATGCCGCTGTTGATGCACTATGTGAGAAGGCATTAAAATAT
hCoV-19/Germany/HE-RKI-I-438397/2022_EPI_ISL_905522...	(17089)	TCTGCTCGCATAGTGTATACAGCTTGCTCTCATGCCGCTGTTGATGCACTATGTGAGAAGGCATTAAAATAT
hCoV-19/USA/DE-CDC-LC0472738/2021_EPI_ISL_9049423...	(17053)	TCTGCTCGCATAGTGTATACAGCTTGCTCTCATGCCGCTGTTGATGCACTATGTGAGAAGGCATTAAAATAT

Section 240

		17209 17220 17230 17240 17250 17260 17270 17280
	(17209)	
SARS-CoV-2 Reference Genome NC_045512.2	(17209)	TTGCCTATAGATAAATGTAGTAGAATTATACCTGCACGTGCTCGTGTAGAGTGTTTTGATAAATTCAAAGTG
hCoV-19/Botswana/R69B55_BHP_916539/2021_EPI_ISL_90...	(17155)	TTGCCTATAGATAAATGTAGTAGAATTATACCTGCACGTGCTCGTGTAGAGTGTTTTGATAAATTCAAAGTG
hCoV-19/India/HR-MDU-IGIB1210605800489930/2022_EPI...	(17197)	TTGCCTATAGATAAATGTAGTAGAATTATACCTGCACGTGCTCGTGTAGAGTGTTTTGATAAATTCAAAGTG
hCoV-19/England/PLYM-332B917/2022_EPI_ISL_9062229_...	(17147)	TTGCCTATAGATAAATGTAGTAGAATTATACCTGCACGTGCTCGTGTAGAGTGTTTTGATAAATTCAAAGTG
hCoV-19/Germany/HE-RKI-I-438397/2022_EPI_ISL_905522...	(17161)	TTGCCTATAGATAAATGTAGTAGAATTATACCTGCACGTGCTCGTGTAGAGTGTTTTGATAAATTCAAAGTG
hCoV-19/USA/DE-CDC-LC0472738/2021_EPI_ISL_9049423...	(17125)	TTGCCTATAGATAAATGTAGTAGAATTATACCTGCACGTGCTCGTGTAGAGTGTTTTGATAAATTCAAAGTG

Omicron BA.1

Section 241

	(17281)	17281 … 17352
SARS-CoV-2 Reference Genome NC_045512.2	(17281)	AATTCAACATTAGAACAGTATGTCTTTTGTACTGTAAATGCATTGCCTGAGACGACAGCAGATATAGTTGTC
hCoV-19/Botswana/R69B55_BHP_916539/2021_EPI_ISL_90...	(17227)	AATTCAACATTAGAACAGTATGTCTTTTGTACTGTAAATGCATTGCCTGAGACGACAGCAGATATAGTTGTC
hCoV-19/India/HR-MDU-IGIB1210605800489930/2022_EPI...	(17269)	AATTCAACATTAGAACAGTATGTCTTTTGTACTGTAAATGCATTGCCTGAGACGACAGCAGATATAGTTGTC
hCoV-19/England/PLYM-332B917/2022_EPI_ISL_9062229_...	(17219)	AATTCAACATTAGAACAGTATGTCTTTTGTACTGTAAATGCATTGCCTGAGACGACAGCAGATATAGTTGTC
hCoV-19/Germany/HE-RKI-I-438397/2022_EPI_ISL_905522...	(17233)	AATTCAACATTAGAACAGTATGTCTTTTGTACTGTAAATGCATTGCCTGAGACGACAGCAGATATAGTTGTC
hCoV-19/USA/DE-CDC-LC0472738/2021_EPI_ISL_9049423...	(17197)	AATTCAACATTAGAACAGTATGTCTTTTGTACTGTAAATGCATTGCCTGAGACGACAGCAGATATAGTTGTC

Section 242

	(17353)	17353 … 17424
SARS-CoV-2 Reference Genome NC_045512.2	(17353)	TTTGATGAAATTTCAATGGCCACAAATTATGATTTGAGTGTTGTCAATGCCAGATTACGTGCTAAGCACTAT
hCoV-19/Botswana/R69B55_BHP_916539/2021_EPI_ISL_90...	(17299)	TTTGATGAAATTTCAATGGCCACAAATTATGATTTGAGTGTTGTCAATGCCAGATTACGTGCTAAGCACTAT
hCoV-19/India/HR-MDU-IGIB1210605800489930/2022_EPI...	(17341)	TTTGATGAAATTTCAATGGCCACAAATTATGATTTGAGTGTTGTCAATGCCAGATTACGTGCTAAGCACTAT
hCoV-19/England/PLYM-332B917/2022_EPI_ISL_9062229_...	(17291)	TTTGATGAAATTTCAATGGCCACAAATTATGATTTGAGTGTTGTCAATGCCAGATTACGTGCTAAGCACTAT
hCoV-19/Germany/HE-RKI-I-438397/2022_EPI_ISL_905522...	(17305)	TTTGATGAAATTTCAATGGCCACAAATTATGATTTGAGTGTTGTCAATGCCAGATTACGTGCTAAGCACTAT
hCoV-19/USA/DE-CDC-LC0472738/2021_EPI_ISL_9049423...	(17269)	TTTGATGAAATTTCAATGGCCACAAATTATGATTTGAGTGTTGTCAATGCCAGATTACGTGCTAAGCACTAT

Section 243

	(17425)	17425 … 17496
SARS-CoV-2 Reference Genome NC_045512.2	(17425)	GTGTACATTGGCGACCCTGCTCAATTACCTGCACCACGCACATTGCTAACTAAGGGCACACTAGAACCAGAA
hCoV-19/Botswana/R69B55_BHP_916539/2021_EPI_ISL_90...	(17371)	GTGTACATTGGCGACCCTGCTCAATTACCTGCACCACGCACATTGCTAACTAAGGGCACACTAGAACCAGAA
hCoV-19/India/HR-MDU-IGIB1210605800489930/2022_EPI...	(17413)	GTGTACATTGGCGACCCTGCTCAATTACCTGCACCACGCACATTGCTAACTAAGGGCACACTAGAACCAGAA
hCoV-19/England/PLYM-332B917/2022_EPI_ISL_9062229_...	(17363)	GTGTACATTGGCGACCCTGCTCAATTACCTGCACCACGCACATTGCTAACTAAGGGCACACTAGAACCAGAA
hCoV-19/Germany/HE-RKI-I-438397/2022_EPI_ISL_905522...	(17377)	GTGTACATTGGCGACCCTGCTCAATTACCTGCACCACGCACATTGCTAACTAAGGGCACACTAGAACCAGAA
hCoV-19/USA/DE-CDC-LC0472738/2021_EPI_ISL_9049423...	(17341)	GTGTACATTGGCGACCCTGCTCAATTACCTGCACCACGCACATTGCTAACTAAGGGCACACTAGAACCAGAA

Section 244

	(17497)	17497 … 17568
SARS-CoV-2 Reference Genome NC_045512.2	(17497)	TATTTCAATTCAGTGTGTAGACTTATGAAAACTATAGGTCCAGACATGTTCCTCGGAACTTGTCGGCGTTGT
hCoV-19/Botswana/R69B55_BHP_916539/2021_EPI_ISL_90...	(17443)	TATTTCAATTCAGTGTGTAGACTTATGAAAACTATAGGTCCAGACATGTTCCTCGGAACTTGTCGGCGTTGT
hCoV-19/India/HR-MDU-IGIB1210605800489930/2022_EPI...	(17485)	TATTTCAATTCAGTGTGTAGACTTATGAAAACTATAGGTCCAGACATGTTCCTCGGAACTTGTCGGCGTTGT
hCoV-19/England/PLYM-332B917/2022_EPI_ISL_9062229_...	(17435)	TATTTCAATTCAGTGTGTAGACTTATGAAAACTATAGGTCCAGACATGTTCCTCGGAACTTGTCGGCGTTGT
hCoV-19/Germany/HE-RKI-I-438397/2022_EPI_ISL_905522...	(17449)	TATTTCAATTCAGTGTGTAGACTTATGAAAACTATAGGTCCAGACATGTTCCTCGGAACTTGTCGGCGTTGT
hCoV-19/USA/DE-CDC-LC0472738/2021_EPI_ISL_9049423...	(17413)	TATTTCAATTCAGTGTGTAGACTTATGAAAACTATAGGTCCAGACATGTTCCTCGGAACTTGTCGGCGTTGT

Section 245

		17569 … 17640
	(17569)	17569 17580 17590 17600 17610 17620 17630 17640
SARS-CoV-2 Reference Genome NC_045512.2	(17569)	CCTGCTGAAATTGTTGACACTGTGAGTGCTTTGGTTTATGATAATAAGCTTAAAGCACATAAAGACAAATCA
hCoV-19/Botswana/R69B55_BHP_916539/2021_EPI_ISL_90...	(17515)	CCTGCTGAAATTGTTGACACTGTGAGTGCTTTGGTTTATGATAATAAGCTTAAAGCACATAAAGACAAATCA
hCoV-19/India/HR-MDU-IGIB1210605800489930/2022_EPI...	(17557)	CCTGCTGAAATTGTTGACACTGTGAGTGCTTTGGTTTATGATAATAAGCTTAAAGCACATAAAGACAAATCA
hCoV-19/England/PLYM-332B917/2022_EPI_ISL_9062229_...	(17507)	CCTGCTGAAATTGTTGACACTGTGAGTGCTTTGGTTTATGATAATAAGCTTAAAGCACATAAAGACAAATCA
hCoV-19/Germany/HE-RKI-I-438397/2022_EPI_ISL_905522...	(17521)	CCTGCTGAAATTGTTGACACTGTGAGTGCTTTGGTTTATGATAATAAGCTTAAAGCACATAAAGACAAATCA
hCoV-19/USA/DE-CDC-LC0472738/2021_EPI_ISL_9049423...	(17485)	CCTGCTGAAATTGTTGACACTGTGAGTGCTTTGGTTTATGATAATAAGCTTAAAGCACATAAAGACAAATCA

Section 246

		17641 … 17712
	(17641)	17641 17650 17660 17670 17680 17690 17700 17712
SARS-CoV-2 Reference Genome NC_045512.2	(17641)	GCTCAATGCTTTAAAATGTTTTATAAGGGTGTTATCACGCATGATGTTTCATCTGCAATTAACAGGCCACAA
hCoV-19/Botswana/R69B55_BHP_916539/2021_EPI_ISL_90...	(17587)	GCTCAATGCTTTAAAATGTTTTATAAGGGTGTTATCACGCATGATGTTTCATCTGCAATTAACAGGCCACAA
hCoV-19/India/HR-MDU-IGIB1210605800489930/2022_EPI...	(17629)	GCTCAATGCTTTAAAATGTTTTATAAGGGTGTTATCACGCATGATGTTTCATCTGCAATTAACAGGCCACAA
hCoV-19/England/PLYM-332B917/2022_EPI_ISL_9062229_...	(17579)	GCTCAATGCTTTAAAATGTTTTATAAGGGTGTTATCACGCATGATGTTTCATCTGCAATTAACAGGCCACAA
hCoV-19/Germany/HE-RKI-I-438397/2022_EPI_ISL_905522...	(17593)	GCTCAATGCTTTAAAATGTTTTATAAGGGTGTTATCACGCATGATGTTTCATCTGCAATTAACAGGCCACAA
hCoV-19/USA/DE-CDC-LC0472738/2021_EPI_ISL_9049423...	(17557)	GCTCAATGCTTTAAAATGTTTTATAAGGGTGTTATCACGCATGATGTTTCATCTGCAATTAACAGGCCACAA

Section 247

		17713 … 17784
	(17713)	17713 17720 17730 17740 17750 17760 17770 17784
SARS-CoV-2 Reference Genome NC_045512.2	(17713)	ATAGGCGTGGTAAGAGAATTCCTTACACGTAACCCTGCTTGGAGAAAAGCTGTCTTTATTTCACCTTATAAT
hCoV-19/Botswana/R69B55_BHP_916539/2021_EPI_ISL_90...	(17659)	ATAGGCGTGGTAAGAGAATTCCTTACACGTAACCCTGCTTGGAGAAAAGCTGTCTTTATTTCACCTTATAAT
hCoV-19/India/HR-MDU-IGIB1210605800489930/2022_EPI...	(17701)	ATAGGCGTGGTAAGAGAATTCCTTACACGTAACCCTGCTTGGAGAAAAGCTGTCTTTATTTCACCTTATAAT
hCoV-19/England/PLYM-332B917/2022_EPI_ISL_9062229_...	(17651)	ATAGGCGTGGTAAGAGAATTCCTTACACGTAACCCTGCTTGGAGAAAAGCTGTCTTTATTTCACCTTATAAT
hCoV-19/Germany/HE-RKI-I-438397/2022_EPI_ISL_905522...	(17665)	ATAGGCGTGGTAAGAGAATTCCTTACACGTAACCCTGCTTGGAGAAAAGCTGTCTTTATTTCACCTTATAAT
hCoV-19/USA/DE-CDC-LC0472738/2021_EPI_ISL_9049423...	(17629)	ATAGGCGTGGTAAGAGAATTCCTTACACGTAACCCTGCTTGGAGAAAAGCTGTCTTTATTTCACCTTATAAT

Section 248

		17785 … 17856
	(17785)	17785 17790 17800 17810 17820 17830 17840 17856
SARS-CoV-2 Reference Genome NC_045512.2	(17785)	TCACAGAATGCTGTAGCCTCAAAGATTTTGGGACTACCAACTCAAACTGTTGATTCATCACAGGGCTCAGAA
hCoV-19/Botswana/R69B55_BHP_916539/2021_EPI_ISL_90...	(17731)	TCACAGAATGCTGTAGCCTCAAAGATTTTGGGACTACCAACTCAAACTGTTGATTCATCACAGGGCTCAGAA
hCoV-19/India/HR-MDU-IGIB1210605800489930/2022_EPI...	(17773)	TCACAGAATGCTGTAGCCTCAAAGATTTTGGGACTACCAACTCAAACTGTTGATTCATCACAGGGCTCAGAA
hCoV-19/England/PLYM-332B917/2022_EPI_ISL_9062229_...	(17723)	TCACAGAATGCTGTAGCCTCAAAGATTTTGGGACTACCAACTCAAACTGTTGATTCATCACAGGGCTCAGAA
hCoV-19/Germany/HE-RKI-I-438397/2022_EPI_ISL_905522...	(17737)	TCACAGAATGCTGTAGCCTCAAAGATTTTGGGACTACCAACTCAAACTGTTGATTCATCACAGGGCTCAGAA
hCoV-19/USA/DE-CDC-LC0472738/2021_EPI_ISL_9049423...	(17701)	TCACAGAATGCTGTAGCCTCAAAGATTTTGGGACTACCAACTCAAACTGTTGATTCATCACAGGGCTCAGAA

Section 249

Sequence	Start	17857 … 17928
	(17857)	17857 17870 17880 17890 17900 17910 17928
SARS-CoV-2 Reference Genome NC_045512.2	(17857)	TATGACTATGTCATATTCACTCAAACCACTGAAACAGCTCACTCTTGTAATGTAAACAGATTTAATGTTGCT
hCoV-19/Botswana/R69B55_BHP_916539/2021_EPI_ISL_90...	(17803)	TATGACTATGTCATATTCACTCAAACCACTGAAACAGCTCACTCTTGTAATGTAAACAGATTTAATGTTGCT
hCoV-19/India/HR-MDU-IGIB1210605800489930/2022_EPI...	(17845)	TATGACTATGTCATATTCACTCAAACCACTGAAACAGCTCACTCTTGTAATGTAAACAGATTTAATGTTGCT
hCoV-19/England/PLYM-332B917/2022_EPI_ISL_9062229 ...	(17795)	TATGACTATGTCATATTCACTCAAACCACTGAAACAGCTCACTCTTGTAATGTAAACAGATTTAATGTTGCT
hCoV-19/Germany/HE-RKI-I-438397/2022_EPI_ISL_905522...	(17809)	TATGACTATGTCATATTCACTCAAACCACTGAAACAGCTCACTCTTGTAATGTAAACAGATTTAATGTTGCT
hCoV-19/USA/DE-CDC-LC0472738/2021_EPI_ISL_9049423...	(17773)	TATGACTATGTCATATTCACTCAAACCACTGAAACAGCTCACTCTTGTAATGTAAACAGATTTAATGTTGCT

Section 250

Sequence	Start	17929 … 18000
	(17929)	17929 17940 17950 17960 17970 17980 17990 18000
SARS-CoV-2 Reference Genome NC_045512.2	(17929)	ATTACCAGAGCAAAAGTAGGCATACTTTGCATAATGTCTGATAGAGACCTTTATGACAAGTTGCAATTTACA
hCoV-19/Botswana/R69B55_BHP_916539/2021_EPI_ISL_90...	(17875)	ATTACCAGAGCAAAAGTAGGCATACTTTGCATAATGTCTGATAGAGACCTTTATGACAAGTTGCAATTTACA
hCoV-19/India/HR-MDU-IGIB1210605800489930/2022_EPI...	(17917)	ATTACCAGAGCAAAAGTAGGCATACTTTGCATAATGTCTGATAGAGACCTTTATGACAAGTTGCAATTTACA
hCoV-19/England/PLYM-332B917/2022_EPI_ISL_9062229 ...	(17867)	ATTACCAGAGCAAAAGTAGGCATACTTTGCATAATGTCTGATAGAGACCTTTATGACAAGTTGCAATTTACA
hCoV-19/Germany/HE-RKI-I-438397/2022_EPI_ISL_905522...	(17881)	ATTACCAGAGCAAAAGTAGGCATACTTTGCATAATGTCTGATAGAGACCTTTATGACAAGTTGCAATTTACA
hCoV-19/USA/DE-CDC-LC0472738/2021_EPI_ISL_9049423...	(17845)	ATTACCAGAGCAAAAGTAGGCATACTTTGCATAATGTCTGATAGAGACCTTTATGACAAGTTGCAATTTACA

Section 251

Sequence	Start	18001 … 18072
	(18001)	18001 18010 18020 18030 18040 18050 18060 18072
SARS-CoV-2 Reference Genome NC_045512.2	(18001)	AGTCTTGAAATTCCACGTAGGAATGTGGCAACTTTACAAGCTGAAAATGTAACAGGACTCTTTAAAGATTGT
hCoV-19/Botswana/R69B55_BHP_916539/2021_EPI_ISL_90...	(17947)	AGTCTTGAAATTCCACGTAGGAATGTGGCAACTTTACAAGCTGAAAATGTAACAGGACTCTTTAAAGATTGT
hCoV-19/India/HR-MDU-IGIB1210605800489930/2022_EPI...	(17989)	AGTCTTGAAATTCCACGTAGGAATGTGGCAACTTTACAAGCTGAAAATGTAACAGGACTCTTTAAAGATTGT
hCoV-19/England/PLYM-332B917/2022_EPI_ISL_9062229 ...	(17939)	AGTCTTGAAATTCCACGTAGGAATGTGGCAACTTTACAAGCTGAAAATGTAACAGGACTCTTTAAAGATTGT
hCoV-19/Germany/HE-RKI-I-438397/2022_EPI_ISL_905522...	(17953)	AGTCTTGAAATTCCACGTAGGAATGTGGCAACTTTACAAGCTGAAAATGTAACAGGACTCTTTAAAGATTGT
hCoV-19/USA/DE-CDC-LC0472738/2021_EPI_ISL_9049423...	(17917)	AGTCTTGAAATTCCACGTAGGAATGTGGCAACTTTACAAGCTGAAAATGTAACAGGACTCTTTAAAGATTGT

Section 252

Sequence	Start	18073 … 18144
	(18073)	18073 18080 18090 18100 18110 18120 18130 18144
SARS-CoV-2 Reference Genome NC_045512.2	(18073)	AGTAAGGTAATCACTGGGTTACATCCTACACAGGCACCTACACACCTCAGTGTTGACACTAAATTCAAAACT
hCoV-19/Botswana/R69B55_BHP_916539/2021_EPI_ISL_90...	(18019)	AGTAAGGTAATCACTGGGTTACATCCTACACAGGCACCTACACACCTCAGTGTTGACACTAAATTCAAAACT
hCoV-19/India/HR-MDU-IGIB1210605800489930/2022_EPI...	(18061)	AGTAAGGTAATCACTGGGTTACATCCTACACAGGCACCTACACACCTCAGTGTTGACACTAAATTCAAAACT
hCoV-19/England/PLYM-332B917/2022_EPI_ISL_9062229 ...	(18011)	AGTAAGGTAATCACTGGGTTACATCCTACACAGGCACCTACACACCTCAGTGTTGACACTAAATTCAAAACT
hCoV-19/Germany/HE-RKI-I-438397/2022_EPI_ISL_905522...	(18025)	AGTAAGGTAATCACTGGGTTACATCCTACACAGGCACCTACACACCTCAGTGTTGACACTAAATTCAAAACT
hCoV-19/USA/DE-CDC-LC0472738/2021_EPI_ISL_9049423...	(17989)	AGTAAGGTAATCACTGGGTTACATCCTACACAGGCACCTACACACCTCAGTGTTGACACTAAATTCAAAACT

Section 253

```
                                                                  (18145) 18145 18150          18160          18170          18180          18190          18200               18216
SARS-CoV-2 Reference Genome NC_045512.2                           (18145) GAAGGTTTATGTGTTGACATACCTGGCATACCTAAGGACATGACCTATAGAAGACTCATCTCTATGATGGGT
hCoV-19/Botswana/R69B55_BHP_916539/2021_EPI_ISL_90...             (18091) GAAGGTTTATGTGTTGACGTACCTGGCATACCTAAGGACATGACCTATAGAAGACTCATCTCTATGATGGGT
hCoV-19/India/HR-MDU-IGIB1210605800489930/2022_EPI...             (18133) GAAGGTTTATGTGTTGACGTACCTGGCATACCTAAGGACATGACCTATAGAAGACTCATCTCTATGATGGGT
hCoV-19/England/PLYM-332B917/2022_EPI_ISL_9062229_...             (18083) GAAGGTTTATGTGTTGACGTACCTGGCATACCTAAGGACATGACCTATAGAAGACTCATCTCTATGATGGGT
hCoV-19/Germany/HE-RKI-I-438397/2022_EPI_ISL_905522...            (18097) GAAGGTTTATGTGTTGACGTACCTGGCATACCTAAGGACATGACCTATAGAAGACTCATCTCTATGATGGGT
hCoV-19/USA/DE-CDC-LC0472738/2021_EPI_ISL_9049423...              (18061) GAAGGTTTATGTGTTGACGTACCTGGCATACCTAAGGACATGACCTATAGAAGACTCATCTCTATGATGGGT
```

Section 254

```
                                                                  (18217) 18217       18230          18240          18250          18260          18270               18288
SARS-CoV-2 Reference Genome NC_045512.2                           (18217) TTTAAAATGAATTATCAAGTTAATGGTTACCCTAACATGTTTATCACCCGCGAAGAAGCTATAAGACATGTA
hCoV-19/Botswana/R69B55_BHP_916539/2021_EPI_ISL_90...             (18163) TTTAAAATGAATTATCAAGTTAATGGTTACCCTAACATGTTTATCACCCGCGAAGAAGCTATAAGACATGTA
hCoV-19/India/HR-MDU-IGIB1210605800489930/2022_EPI...             (18205) TTTAAAATGAATTATCAAGTTAATGGTTACCCTAACATGTTTATCACCCGCGAAGAAGCTATAAGACATGTA
hCoV-19/England/PLYM-332B917/2022_EPI_ISL_9062229_...             (18155) TTTAAAATGAATTATCAAGTTAATGGTTACCCTAACATGTTTATCACCCGCGAAGAAGCTATAAGACATGTA
hCoV-19/Germany/HE-RKI-I-438397/2022_EPI_ISL_905522...            (18169) TTTAAAATGAATTATCAAGTTAATGGTTACCCTAACATGTTTATCACCCGCGAAGAAGCTATAAGACATGTA
hCoV-19/USA/DE-CDC-LC0472738/2021_EPI_ISL_9049423...              (18133) TTTAAAATGAATTATCAAGTTAATGGTTACCCTAACATGTTTATCACCCGCGAAGAAGCTATAAGACATGTA
```

Section 255

```
                                                                  (18289) 18289     18300          18310          18320          18330          18340          18350    18360
SARS-CoV-2 Reference Genome NC_045512.2                           (18289) CGTGCATGGATTGGCTTCGATGTCGAGGGGTGTCATGCTACTAGAGAAGCTGTTGGTACCAATTTACCTTTA
hCoV-19/Botswana/R69B55_BHP_916539/2021_EPI_ISL_90...             (18235) CGTGCATGGATTGGCTTCGATGTCGAGGGGTGTCATGCTACTAGAGAAGCTGTTGGTACCAATTTACCTTTA
hCoV-19/India/HR-MDU-IGIB1210605800489930/2022_EPI...             (18277) CGTGCATGGATTGGCTTCGATGTCGAGGGGTGTCATGCTACTAGAGAAGCTGTTGGTACCAATTTACCTTTA
hCoV-19/England/PLYM-332B917/2022_EPI_ISL_9062229_...             (18227) CGTGCATGGATTGGCTTCGATGTCGAGGGGTGTCATGCTACTAGAGAAGCTGTTGGTACCAATTTACCTTTA
hCoV-19/Germany/HE-RKI-I-438397/2022_EPI_ISL_905522...            (18241) CGTGCATGGATTGGCTTCGATGTCGAGGGGTGTCATGCTACTAGAGAAGCTGTTGGTACCAATTTACCTTTA
hCoV-19/USA/DE-CDC-LC0472738/2021_EPI_ISL_9049423...              (18205) CGTGCATGGATTGGCTTCGATGTCGAGGGGTGTCATGCTACTAGAGAAGCTGTTGGTACCAATTTACCTTTA
```

Section 256

```
                                                                  (18361) 18361    18370          18380          18390          18400          18410          18420      18432
SARS-CoV-2 Reference Genome NC_045512.2                           (18361) CAGCTAGGTTTTTCTACAGGTGTTAACCTAGTTGCTGTACCTACAGGTTATGTTGATACACCTAATAATACA
hCoV-19/Botswana/R69B55_BHP_916539/2021_EPI_ISL_90...             (18307) CAGCTAGGTTTTTCTACAGGTGTTAACCTAGTTGCTGTACCTACAGGTTATGTTGATACACCTAATAATACA
hCoV-19/India/HR-MDU-IGIB1210605800489930/2022_EPI...             (18349) CAGCTAGGTTTTTCTACAGGTGTTAACCTAGTTGCTGTACCTACAGGTTATGTTGATACACCTAATAATACA
hCoV-19/England/PLYM-332B917/2022_EPI_ISL_9062229_...             (18299) CAGCTAGGTTTTTCTACAGGTGTTAACCTAGTTGCTGTACCTACAGGTTATGTTGATACACCTAATAATACA
hCoV-19/Germany/HE-RKI-I-438397/2022_EPI_ISL_905522...            (18313) CAGCTAGGTTTTTCTACAGGTGTTAACCTAGTTGCTGTACCTACAGGTTATGTTGATACACCTAATAATACA
hCoV-19/USA/DE-CDC-LC0472738/2021_EPI_ISL_9049423...              (18277) CAGCTAGGTTTTTCTACAGGTGTTAACCTAGTTGCTGTACCTACAGGTTATGTTGATACACCTAATAATACA
```

Section 257

		18433 18440 18450 18460 18470 18480 18490 18504
	(18433)	
SARS-CoV-2 Reference Genome NC_045512.2	(18433)	GATTTTTCCAGAGTTAGTGCTAAACCACCGCCTGGAGATCAATTTAAACACCTCATACCACTTATGTACAAA
hCoV-19/Botswana/R69B55_BHP_916539/2021_EPI_ISL_90...	(18379)	GATTTTTCCAGAGTTAGTGCTAAACCACCGCCTGGAGATCAATTTAAACACCTCATACCACTTATGTACAAA
hCoV-19/India/HR-MDU-IGIB1210605800489930/2022_EPI...	(18421)	GATTTTTCCAGAGTTAGTGCTAAACCACCGCCTGGAGATCAATTTAAACACCTCATACCACTTATGTACAAA
hCoV-19/England/PLYM-332B917/2022_EPI_ISL_9062229 ...	(18371)	GATTTTTCCAGAGTTAGTGCTAAACCACCGCCTGGAGATCAATTTAAACACCTCATACCACTTATGTACAAA
hCoV-19/Germany/HE-RKI-I-438397/2022_EPI_ISL_905522...	(18385)	GATTTTTCCAGAGTTAGTGCTAAACCACCGCCTGGAGATCAATTTAAACACCTCATACCACTTATGTACAAA
hCoV-19/USA/DE-CDC-LC0472738/2021_EPI_ISL_9049423...	(18349)	GATTTTTCCAGAGTTAGTGCTAAACCACCGCCTGGAGATCAATTTAAACACCTCATACCACTTATGTACAAA

Section 258

		18505 18510 18520 18530 18540 18550 18560 18576
	(18505)	
SARS-CoV-2 Reference Genome NC_045512.2	(18505)	GGACTTCCTTGGAATGTAGTGCGTATAAAGATTGTACAAATGTTAAGTGACACACTTAAAAATCTCTCTGAC
hCoV-19/Botswana/R69B55_BHP_916539/2021_EPI_ISL_90...	(18451)	GGACTTCCTTGGAATGTAGTGCGTATAAAGATTGTACAAATGTTAAGTGACACACTTAAAAATCTCTCTGAC
hCoV-19/India/HR-MDU-IGIB1210605800489930/2022_EPI...	(18493)	GGACTTCCTTGGAATGTAGTGCGTATAAAGATTGTACAAATGTTAAGTGACACACTTAAAAATCTCTCTGAC
hCoV-19/England/PLYM-332B917/2022_EPI_ISL_9062229 ...	(18443)	GGACTTCCTTGGAATGTAGTGCGTATAAAGATTGTACAAATGTTAAGTGACACACTTAAAAATCTCTCTGAC
hCoV-19/Germany/HE-RKI-I-438397/2022_EPI_ISL_905522...	(18457)	GGACTTCCTTGGAATGTAGTGCGTATAAAGATTGTACAAATGTTAAGTGACACACTTAAAAATCTCTCTGAC
hCoV-19/USA/DE-CDC-LC0472738/2021_EPI_ISL_9049423...	(18421)	GGACTTCCTTGGAATGTAGTGCGTATAAAGATTGTACAAATGTTAAGTGACACACTTAAAAATCTCTCTGAC

Section 259

		18577 18590 18600 18610 18620 18630 18648
	(18577)	
SARS-CoV-2 Reference Genome NC_045512.2	(18577)	AGAGTCGTATTTGTCTTATGGGCACATGGCTTTGAGTTGACATCTATGAAGTATTTTGTGAAAATAGGACCT
hCoV-19/Botswana/R69B55_BHP_916539/2021_EPI_ISL_90...	(18523)	AGAGTCGTATTTGTCTTATGGGCACATGGCTTTGAGTTGACATCTATGAAGTATTTTGTGAAAATAGGACCT
hCoV-19/India/HR-MDU-IGIB1210605800489930/2022_EPI...	(18565)	AGAGTCGTATTTGTCTTATGGGCACATGGCTTTGAGTTGACATCTATGAAGTATTTTGTGAAAATAGGACCT
hCoV-19/England/PLYM-332B917/2022_EPI_ISL_9062229 ...	(18515)	AGAGTCGTATTTGTCTTATGGGCACATGGCTTTGAGTTGACATCTATGAAGTATTTTGTGAAAATAGGACCT
hCoV-19/Germany/HE-RKI-I-438397/2022_EPI_ISL_905522...	(18529)	AGAGTCGTATTTGTCTTATGGGCACATGGCTTTGAGTTGACATCTATGAAGTATTTTGTGAAAATAGGACCT
hCoV-19/USA/DE-CDC-LC0472738/2021_EPI_ISL_9049423...	(18493)	AGAGTCGTATTTGTCTTATGGGCACATGGCTTTGAGTTGACATCTATGAAGTATTTTGTGAAAATAGGACCT

Section 260

		18649 18660 18670 18680 18690 18700 18710 18720
	(18649)	
SARS-CoV-2 Reference Genome NC_045512.2	(18649)	GAGCGCACCTGTTGTCTATGTGATAGACGTGCCACATGCTTTTCCACTGCTTCAGACACTTATGCCTGTTGG
hCoV-19/Botswana/R69B55_BHP_916539/2021_EPI_ISL_90...	(18595)	GAGCGCACCTGTTGTCTATGTGATAGACGTGCCACATGCTTTTCCACTGCTTCAGACACTTATGCCTGTTGG
hCoV-19/India/HR-MDU-IGIB1210605800489930/2022_EPI...	(18637)	GAGCGCACCTGTTGTCTATGTGATAGACGTGCCACATGCTTTTCCACTGCTTCAGACACTTATGCCTGTTGG
hCoV-19/England/PLYM-332B917/2022_EPI_ISL_9062229 ...	(18587)	GAGCGCACCTGTTGTCTATGTGATAGACGTGCCACATGCTTTTCCACTGCTTCAGACACTTATGCCTGTTGG
hCoV-19/Germany/HE-RKI-I-438397/2022_EPI_ISL_905522...	(18601)	GAGCGCACCTGTTGTCTATGTGATAGACGTGCCACATGCTTTTCCACTGCTTCAGACACTTATGCCTGTTGG
hCoV-19/USA/DE-CDC-LC0472738/2021_EPI_ISL_9049423...	(18565)	GAGCGCACCTGTTGTCTATGTGATAGACGTGCCACATGCTTTTCCACTGCTTCAGACACTTATGCCTGTTGG

Section 261

(18721)	18721 18730 18740 18750 18760 18770 18780 18792
SARS-CoV-2 Reference Genome NC_045512.2 (18721)	CATCATTCTATTGGATTTGATTACGTCTATAATCCGTTTATGATTGATGTTCAACAATGGGGTTTTACAGGT
hCoV-19/Botswana/R69B55_BHP_916539/2021_EPI_ISL_90... (18667)	CATCATTCTATTGGATTTGATTACGTCTATAATCCGTTTATGATTGATGTTCAACAATGGGGTTTTACAGGT
hCoV-19/India/HR-MDU-IGIB1210605800489930/2022_EPI... (18709)	CATCATTCTATTGGATTTGATTACGTCTATAATCCGTTTATGATTGATGTTCAACAATGGGGTTTTACAGGT
hCoV-19/England/PLYM-332B917/2022_EPI_ISL_9062229_... (18659)	CATCATTCTATTGGATTTGATTACGTCTATAATCCGTTTATGATTGATGTTCAACAATGGGGTTTTACAGGT
hCoV-19/Germany/HE-RKI-I-438397/2022_EPI_ISL_905522... (18673)	CATCATTCTATTGGATTTGATTACGTCTATAATCCGTTTATGATTGATGTTCAACAATGGGGTTTTACAGGT
hCoV-19/USA/DE-CDC-LC0472738/2021_EPI_ISL_9049423... (18637)	CATCATTCTATTGGATTTGATTACGTCTATAATCCGTTTATGATTGATGTTCAACAATGGGGTTTTACAGGT

Section 262

(18793)	18793 18800 18810 18820 18830 18840 18850 18864
SARS-CoV-2 Reference Genome NC_045512.2 (18793)	AACCTACAAAGCAACCATGATCTGTATTGTCAAGTCCATGGTAATGCACATGTAGCTAGTTGTGATGCAATC
hCoV-19/Botswana/R69B55_BHP_916539/2021_EPI_ISL_90... (18739)	AACCTACAAAGCAACCATGATCTGTATTGTCAAGTCCATGGTAATGCACATGTAGCTAGTTGTGATGCAATC
hCoV-19/India/HR-MDU-IGIB1210605800489930/2022_EPI... (18781)	AACCTACAAAGCAACCATGATCTGTATTGTCAAGTCCATGGTAATGCACATGTAGCTAGTTGTGATGCAATC
hCoV-19/England/PLYM-332B917/2022_EPI_ISL_9062229_... (18731)	AACCTACAAAGCAACCATGATCTGTATTGTCAAGTCCATGGTAATGCACATGTAGCTAGTTGTGATGCAATC
hCoV-19/Germany/HE-RKI-I-438397/2022_EPI_ISL_905522... (18745)	AACCTACAAAGCAACCATGATCTGTATTGTCAAGTCCATGGTAATGCACATGTAGCTAGTTGTGATGCAATC
hCoV-19/USA/DE-CDC-LC0472738/2021_EPI_ISL_9049423... (18709)	AACCTACAAAGCAACCATGATCTGTATTGTCAAGTCCATGGTAATGCACATGTAGCTAGTTGTGATGCAATC

Section 263

(18865)	18865 18870 18880 18890 18900 18910 18920 18936
SARS-CoV-2 Reference Genome NC_045512.2 (18865)	ATGACTAGGTGTCTAGCTGTCCACGAGTGCTTTGTTAAGCGTGTTGACTGGACTATTGAATATCCTATAATT
hCoV-19/Botswana/R69B55_BHP_916539/2021_EPI_ISL_90... (18811)	ATGACTAGGTGTCTAGCTGTCCACGAGTGCTTTGTTAAGCGTGTTGACTGGACTATTGAATATCCTATAATT
hCoV-19/India/HR-MDU-IGIB1210605800489930/2022_EPI... (18853)	ATGACTAGGTGTCTAGCTGTCCACGAGTGCTTTGTTAAGCGTGTTGACTGGACTATTGAATATCCTATAATT
hCoV-19/England/PLYM-332B917/2022_EPI_ISL_9062229_... (18803)	ATGACTAGGTGTCTAGCTGTCCACGAGTGCTTTGTTAAGCGTGTTGACTGGACTATTGAATATCCTATAATT
hCoV-19/Germany/HE-RKI-I-438397/2022_EPI_ISL_905522... (18817)	ATGACTAGGTGTCTAGCTGTCCACGAGTGCTTTGTTAAGCGTGTTGACTGGACTATTGAATATCCTATAATT
hCoV-19/USA/DE-CDC-LC0472738/2021_EPI_ISL_9049423... (18781)	ATGACTAGGTGTCTAGCTGTCCACGAGTGCTTTGTTAAGCGTGTTGACTGGACTATTGAATATCCTATAATT

Section 264

(18937)	18937 18950 18960 18970 18980 18990 19008
SARS-CoV-2 Reference Genome NC_045512.2 (18937)	GGTGATGAACTGAAGATTAATGCGGCTTGTAGAAAGGTTCAACACATGGTTGTTAAAGCTGCATTATTAGCA
hCoV-19/Botswana/R69B55_BHP_916539/2021_EPI_ISL_90... (18883)	GGTGATGAACTGAAGATTAATGCGGCTTGTAGAAAGGTTCAACACATGGTTGTTAAAGCTGCATTATTAGCA
hCoV-19/India/HR-MDU-IGIB1210605800489930/2022_EPI... (18925)	GGTGATGAACTGAAGATTAATGCGGCTTGTAGAAAGGTTCAACACATGGTTGTTAAAGCTGCATTATTAGCA
hCoV-19/England/PLYM-332B917/2022_EPI_ISL_9062229_... (18875)	GGTGATGAACTGAAGATTAATGCGGCTTGTAGAAAGGTTCAACACATGGTTGTTAAAGCTGCATTATTAGCA
hCoV-19/Germany/HE-RKI-I-438397/2022_EPI_ISL_905522... (18889)	GGTGATGAACTGAAGATTAATGCGGCTTGTAGAAAGGTTCAACACATGGTTGTTAAAGCTGCATTATTAGCA
hCoV-19/USA/DE-CDC-LC0472738/2021_EPI_ISL_9049423... (18853)	GGTGATGAACTGAAGATTAATGCGGCTTGTAGAAAGGTTCAACACATGGTTGTTAAAGCTGCATTATTAGCA

```
                                                                 Section 265
                                                 (19009) 19009      19020     19030     19040     19050     19060     19070   19080
              SARS-CoV-2 Reference Genome NC_045512.2 (19009) GACAAATTCCCAGTTCTTCACGACATTGGTAACCCTAAAGCTATTAAGTGTGTACCTCAAGCTGATGTAGAA
         hCoV-19/Botswana/R69B55_BHP_916539/2021_EPI_ISL_90... (18955) GACAAATTCCCAGTTCTTCACGACATTGGTAACCCTAAAGCTATTAAGTGTGTACCTCAAGCTGATGTAGAA
      hCoV-19/India/HR-MDU-IGIB1210605800489930/2022_EPI... (18997) GACAAATTCCCAGTTCTTCACGACATTGGTAACCCTAAAGCTATTAAGTGTGTACCTCAAGCTGATGTAGAA
       hCoV-19/England/PLYM-332B917/2022_EPI_ISL_9062229_... (18947) GACAAATTCCCAGTTCTTCACGACATTGGTAACCCTAAAGCTATTAAGTGTGTACCTCAAGCTGATGTAGAA
     hCoV-19/Germany/HE-RKI-I-438397/2022_EPI_ISL_905522... (18961) GACAAATTCCCAGTTCTTCACGACATTGGTAACCCTAAAGCTATTAAGTGTGTACCTCAAGCTGATGTAGAA
   hCoV-19/USA/DE-CDC-LC0472738/2021_EPI_ISL_9049423... (18925) GACAAATTCCCAGTTCTTCACGACATTGGTAACCCTAAAGCTATTAAGTGTGTACCTCAAGCTGATGTAGAA
                                                                 Section 266
                                                 (19081) 19081    19090     19100     19110     19120     19130     19140    19152
              SARS-CoV-2 Reference Genome NC_045512.2 (19081) TGGAAGTTCTATGATGCACAGCCTTGTAGTGACAAAGCTTATAAAATAGAAGAATTATTCTATTCTTATGCC
         hCoV-19/Botswana/R69B55_BHP_916539/2021_EPI_ISL_90... (19027) TGGAAGTTCTATGATGCACAGCCTTGTAGTGACAAAGCTTATAAAATAGAAGAATTATTCTATTCTTATGCC
      hCoV-19/India/HR-MDU-IGIB1210605800489930/2022_EPI... (19069) TGGAAGTTCTATGATGCACAGCCTTGTAGTGACAAAGCTTATAAAATAGAAGAATTATTCTATTCTTATGCC
       hCoV-19/England/PLYM-332B917/2022_EPI_ISL_9062229_... (19019) TGGAAGTTCTATGATGCACAGCCTTGTAGTGACAAAGCTTATAAAATAGAAGAATTATTCTATTCTTATGCC
     hCoV-19/Germany/HE-RKI-I-438397/2022_EPI_ISL_905522... (19033) TGGAAGTTCTATGATGCACAGCCTTGTAGTGACAAAGCTTATAAAATAGAAGAATTATTCTATTCTTATGCC
   hCoV-19/USA/DE-CDC-LC0472738/2021_EPI_ISL_9049423... (18997) TGGAAGTTCTATGATGCACAGCCTTGTAGTGACAAAGCTTATAAAATAGAAGAATTATTCTATTCTTATGCC
                                                                 Section 267
                                                 (19153) 19153  19160     19170     19180     19190     19200     19210      19224
              SARS-CoV-2 Reference Genome NC_045512.2 (19153) ACACATTCTGACAAATTCACAGATGGTGTATGCCTATTTTGGAATTGCAATGTCGATAGATATCCTGCTAAT
         hCoV-19/Botswana/R69B55_BHP_916539/2021_EPI_ISL_90... (19099) ACACATTCTGACAAATTCACAGATGGTGTATGCCTATTTTGGAATTGCAATGTCGATAGATATCCTGCTAAT
      hCoV-19/India/HR-MDU-IGIB1210605800489930/2022_EPI... (19141) ACACATTCTGACAAATTCACAGATGGTGTATGCCTATTTTGGAATTGCAATGTCGATAGATATCCTGCTAAT
       hCoV-19/England/PLYM-332B917/2022_EPI_ISL_9062229_... (19091) ACACATTCTGACAAATTCACAGATGGTGTATGCCTATTTTGGAATTGCAATGTCGATAGATATCCTGCTAAT
     hCoV-19/Germany/HE-RKI-I-438397/2022_EPI_ISL_905522... (19105) ACACATTCTGACAAATTCACAGATGGTGTATGCCTATTTTGGAATTGCAATGTCGATAGATATCCTGCTAAT
   hCoV-19/USA/DE-CDC-LC0472738/2021_EPI_ISL_9049423... (19069) ACACATTCTGACAAATTCACAGATGGTGTATGCCTATTTTGGAATTGCAATGTCGATAGATATCCTGCTAAT
                                                                 Section 268
                                                 (19225) 19225 19230     19240     19250     19260     19270     19280       19296
              SARS-CoV-2 Reference Genome NC_045512.2 (19225) TCCATTGTTTGTAGATTTGACACTAGAGTGCTATCTAACCTTAACTTGCCTGGTTGTGATGGTGGCAGTTTG
         hCoV-19/Botswana/R69B55_BHP_916539/2021_EPI_ISL_90... (19171) TCCATTGTTTGTAGATTTGACACTAGAGTGCTATCTAACCTTAACTTGCCTGGTTGTGATGGTGGCAGTTTG
      hCoV-19/India/HR-MDU-IGIB1210605800489930/2022_EPI... (19213) TCCATTGTTTGTAGATTTGACACTAGAGTGCTATCTAACCTTAACTTGCCTGGTTGTGATGGTGGCAGTTTG
       hCoV-19/England/PLYM-332B917/2022_EPI_ISL_9062229_... (19163) TCCATTGTTTGTAGATTTGACACTAGAGTGCTATCTAACCTTAACTTGCCTGGTTGTGATGGTGGCAGTTTG
     hCoV-19/Germany/HE-RKI-I-438397/2022_EPI_ISL_905522... (19177) TCCATTGTTTGTAGATTTGACACTAGAGTGCTATCTAACCTTAACTTGCCTGGTTGTGATGGTGGCAGTTTG
   hCoV-19/USA/DE-CDC-LC0472738/2021_EPI_ISL_9049423... (19141) TCCATTGTTTGTAGATTTGACACTAGAGTGCTATCTAACCTTAACTTGCCTGGTTGTGATGGTGGCAGTTTG
```

Section 269

Sequence	Start	19297 … 19368
	(19297)	19297 19310 19320 19330 19340 19350 19368
SARS-CoV-2 Reference Genome NC_045512.2	(19297)	TATGTAAATAAACATGCATTCCACACACCAGCTTTTGATAAAAGTGCTTTTGTTAATTTAAAACAATTACCA
hCoV-19/Botswana/R69B55_BHP_916539/2021_EPI_ISL_90...	(19243)	TATGTAAATAAACATGCATTCCACACACCAGCTTTTGATAAAAGTGCTTTTGTTAATTTAAAACAATTACCA
hCoV-19/India/HR-MDU-IGIB1210605800489930/2022_EPI...	(19285)	TATGTAAATAAACATGCATTCCACACACCAGCTTTTGATAAAAGTGCTTTTGTTAATTTAAAACAATTACCA
hCoV-19/England/PLYM-332B917/2022_EPI_ISL_9062229_...	(19235)	TATGTAAATAAACATGCATTCCACACACCAGCTTTTGATAAAAGTGCTTTTGTTAATTTAAAACAATTACCA
hCoV-19/Germany/HE-RKI-I-438397/2022_EPI_ISL_905522...	(19249)	TATGTAAATAAACATGCATTCCACACACCAGCTTTTGATAAAAGTGCTTTTGTTAATTTAAAACAATTACCA
hCoV-19/USA/DE-CDC-LC0472738/2021_EPI_ISL_9049423...	(19213)	TATGTAAATAAACATGCATTCCACACACCAGCTTTTGATAAAAGTGCTTTTGTTAATTTAAAACAATTACCA

Section 270

Sequence	Start	19369 … 19440
	(19369)	19369 19380 19390 19400 19410 19420 19430 19440
SARS-CoV-2 Reference Genome NC_045512.2	(19369)	TTTTTCTATTACTCTGACAGTCCATGTGAGTCTCATGGAAAACAAGTAGTGTCAGATATAGATTATGTACCA
hCoV-19/Botswana/R69B55_BHP_916539/2021_EPI_ISL_90...	(19315)	TTTTTCTATTACTCTGACAGTCCATGTGAGTCTCATGGAAAACAAGTAGTGTCAGATATAGATTATGTACCA
hCoV-19/India/HR-MDU-IGIB1210605800489930/2022_EPI...	(19357)	TTTTTCTATTACTCTGACAGTCCATGTGAGTCTCATGGAAAACAAGTAGTGTCAGATATAGATTATGTACCA
hCoV-19/England/PLYM-332B917/2022_EPI_ISL_9062229_...	(19307)	TTTTTCTATTACTCTGACAGTCCATGTGAGTCTCATGGAAAACAAGTAGTGTCAGATATAGATTATGTACCA
hCoV-19/Germany/HE-RKI-I-438397/2022_EPI_ISL_905522...	(19321)	TTTTTCTATTACTCTGACAGTCCATGTGAGTCTCATGGAAAACAAGTAGTGTCAGATATAGATTATGTACCA
hCoV-19/USA/DE-CDC-LC0472738/2021_EPI_ISL_9049423...	(19285)	TTTTTCTATTACTCTGACAGTCCATGTGAGTCTCATGGAAAACAAGTAGTGTCAGATATAGATTATGTACCA

Section 271

Sequence	Start	19441 … 19512
	(19441)	19441 19450 19460 19470 19480 19490 19500 19512
SARS-CoV-2 Reference Genome NC_045512.2	(19441)	CTAAAGTCTGCTACGTGTATAACACGTTGCAATTTAGGTGGTGCTGTCTGTAGACATCATGCTAATGAGTAC
hCoV-19/Botswana/R69B55_BHP_916539/2021_EPI_ISL_90...	(19387)	CTAAAGTCTGCTACGTGTATAACACGTTGCAATTTAGGTGGTGCTGTCTGTAGACATCATGCTAATGAGTAC
hCoV-19/India/HR-MDU-IGIB1210605800489930/2022_EPI...	(19429)	CTAAAGTCTGCTACGTGTATAACACGTTGCAATTTAGGTGGTGCTGTCTGTAGACATCATGCTAATGAGTAC
hCoV-19/England/PLYM-332B917/2022_EPI_ISL_9062229_...	(19379)	CTAAAGTCTGCTACGTGTATAACACGTTGCAATTTAGGTGGTGCTGTCTGTAGACATCATGCTAATGAGTAC
hCoV-19/Germany/HE-RKI-I-438397/2022_EPI_ISL_905522...	(19393)	CTAAAGTCTGCTACGTGTATAACACGTTGCAATTTAGGTGGTGCTGTCTGTAGACATCATGCTAATGAGTAC
hCoV-19/USA/DE-CDC-LC0472738/2021_EPI_ISL_9049423...	(19357)	CTAAAGTCTGCTACGTGTATAACACGTTGCAATTTAGGTGGTGCTGTCTGTAGACATCATGCTAATGAGTAC

Section 272

Sequence	Start	19513 … 19584
	(19513)	19513 19520 19530 19540 19550 19560 19570 19584
SARS-CoV-2 Reference Genome NC_045512.2	(19513)	AGATTGTATCTCGATGCTTATAACATGATGATCTCAGCTGGCTTTAGCTTGTGGGTTTACAAACAATTTGAT
hCoV-19/Botswana/R69B55_BHP_916539/2021_EPI_ISL_90...	(19459)	AGATTGTATCTCGATGCTTATAACATGATGATCTCAGCTGGCTTTAGCTTGTGGGTTTACAAACAATTTGAT
hCoV-19/India/HR-MDU-IGIB1210605800489930/2022_EPI...	(19501)	AGATTGTATCTCGATGCTTATAACATGATGATCTCAGCTGGCTTTAGCTTGTGGGTTTACAAACAATTTGAT
hCoV-19/England/PLYM-332B917/2022_EPI_ISL_9062229_...	(19451)	AGATTGTATCTCGATGCTTATAACATGATGATCTCAGCTGGCTTTAGCTTGTGGGTTTACAAACAATTTGAT
hCoV-19/Germany/HE-RKI-I-438397/2022_EPI_ISL_905522...	(19465)	AGATTGTATCTCGATGCTTATAACATGATGATCTCAGCTGGCTTTAGCTTGTGGGTTTACAAACAATTTGAT
hCoV-19/USA/DE-CDC-LC0472738/2021_EPI_ISL_9049423...	(19429)	AGATTGTATCTCGATGCTTATAACATGATGATCTCAGCTGGCTTTAGCTTGTGGGTTTACAAACAATTTGAT

Section 273

		19585 19590 19600 19610 19620 19630 19640 19656
	(19585)	
SARS-CoV-2 Reference Genome NC_045512.2	(19585)	ACTTATAACCTCTGGAACACTTTTACAAGACTTCAGAGTTTAGAAAATGTGGCTTTTAATGTTGTAAATAAG
hCoV-19/Botswana/R69B55_BHP_916539/2021_EPI_ISL_90...	(19531)	ACTTATAACCTCTGGAACACTTTTACAAGACTTCAGAGTTTAGAAAATGTGGCTTTTAATGTTGTAAATAAG
hCoV-19/India/HR-MDU-IGIB1210605800489930/2022_EPI...	(19573)	ACTTATAACCTCTGGAACACTTTTACAAGACTTCAGAGTTTAGAAAATGTGGCTTTTAATGTTGTAAATAAG
hCoV-19/England/PLYM-332B917/2022_EPI_ISL_9062229_...	(19523)	ACTTATAACCTCTGGAACACTTTTACAAGACTTCAGAGTTTAGAAAATGTGGCTTTTAATGTTGTAAATAAG
hCoV-19/Germany/HE-RKI-I-438397/2022_EPI_ISL_905522...	(19537)	ACTTATAACCTCTGGAACACTTTTACAAGACTTCAGAGTTTAGAAAATGTGGCTTTTAATGTTGTAAATAAG
hCoV-19/USA/DE-CDC-LC0472738/2021_EPI_ISL_9049423...	(19501)	ACTTATAACCTCTGGAACACTTTTACAAGACTTCAGAGTTTAGAAAATGTGGCTTTTAATGTTGTAAATAAG

Section 274

		19657 19670 19680 19690 19700 19710 19728
	(19657)	
SARS-CoV-2 Reference Genome NC_045512.2	(19657)	GGACACTTTGATGGACAACAGGGTGAAGTACCAGTTTCTATCATTAATAACACTGTTTACACAAAAGTTGAT
hCoV-19/Botswana/R69B55_BHP_916539/2021_EPI_ISL_90...	(19603)	GGACACTTTGATGGACAACAGGGTGAAGTACCAGTTTCTATCATTAATAACACTGTTTACACAAAAGTTGAT
hCoV-19/India/HR-MDU-IGIB1210605800489930/2022_EPI...	(19645)	GGACACTTTGATGGACAACAGGGTGAAGTACCAGTTTCTATCATTAATAACACTGTTTACACAAAAGTTGAT
hCoV-19/England/PLYM-332B917/2022_EPI_ISL_9062229_...	(19595)	GGACACTTTGATGGACAACAGGGTGAAGTACCAGTTTCTATCATTAATAACACTGTTTACACAAAAGTTGAT
hCoV-19/Germany/HE-RKI-I-438397/2022_EPI_ISL_905522...	(19609)	GGACACTTTGATGGACAACAGGGTGAAGTACCAGTTTCTATCATTAATAACACTGTTTACACAAAAGTTGAT
hCoV-19/USA/DE-CDC-LC0472738/2021_EPI_ISL_9049423...	(19573)	GGACACTTTGATGGACAACAGGGTGAAGTACCAGTTTCTATCATTAATAACACTGTTTACACAAAAGTTGAT

Section 275

		19729 19740 19750 19760 19770 19780 19790 19800
	(19729)	
SARS-CoV-2 Reference Genome NC_045512.2	(19729)	GGTGTTGATGTAGAATTGTTTGAAAATAAAACAACATTACCTGTTAATGTAGCATTTGAGCTTTGGGCTAAG
hCoV-19/Botswana/R69B55_BHP_916539/2021_EPI_ISL_90...	(19675)	GGTGTTGATGTAGAATTGTTTGAAAATAAAACAACATTACCTGTTAATGTAGCATTTGAGCTTTGGGCTAAG
hCoV-19/India/HR-MDU-IGIB1210605800489930/2022_EPI...	(19717)	GGTGTTGATGTAGAATTGTTTGAAAATAAAACAACATTACCTGTTAATGTAGCATTTGAGCTTTGGGCTAAG
hCoV-19/England/PLYM-332B917/2022_EPI_ISL_9062229_...	(19667)	GGTGTTGATGTAGAATTGTTTGAAAATAAAACAACATTACCTGTTAATGTAGCATTTGAGCTTTGGGCTAAG
hCoV-19/Germany/HE-RKI-I-438397/2022_EPI_ISL_905522...	(19681)	GGTGTTGATGTAGAATTGTTTGAAAATAAAACAACATTACCTGTTAATGTAGCATTTGAGCTTTGGGCTAAG
hCoV-19/USA/DE-CDC-LC0472738/2021_EPI_ISL_9049423...	(19645)	GGTGTTGATGTAGAATTGTTTGAAAATAAAACAACATTACCTGTTAATGTAGCATTTGAGCTTTGGGCTAAG

Section 276

		19801 19810 19820 19830 19840 19850 19860 19872
	(19801)	
SARS-CoV-2 Reference Genome NC_045512.2	(19801)	CGCAACATTAAACCAGTACCAGAGGTGAAAATACTCAATAATTTGGGTGTGGACATTGCTGCTAATACTGTG
hCoV-19/Botswana/R69B55_BHP_916539/2021_EPI_ISL_90...	(19747)	CGCAACATTAAACCAGTACCAGAGGTGAAAATACTCAATAATTTGGGTGTGGACATTGCTGCTAATACTGTG
hCoV-19/India/HR-MDU-IGIB1210605800489930/2022_EPI...	(19789)	CGCAACATTAAACCAGTACCAGAGGTGAAAATACTCAATAATTTGGGTGTGGACATTGCTGCTAATACTGTG
hCoV-19/England/PLYM-332B917/2022_EPI_ISL_9062229_...	(19739)	CGCAACATTAAACCAGTACCAGAGGTGAAAATACTCAATAATTTGGGTGTGGACATTGCTGCTAATACTGTG
hCoV-19/Germany/HE-RKI-I-438397/2022_EPI_ISL_905522...	(19753)	CGCAACATTAAACCAGTACCAGAGGTGAAAATACTCAATAATTTGGGTGTGGACATTGCTGCTAATACTGTG
hCoV-19/USA/DE-CDC-LC0472738/2021_EPI_ISL_9049423...	(19717)	CGCAACATTAAACCAGTACCAGAGGTGAAAATACTCAATAATTTGGGTGTGGACATTGCTGCTAATACTGTG

Section 277

		19873 19880 19890 19900 19910 19920 19930 19944
	(19873)	
SARS-CoV-2 Reference Genome NC_045512.2	(19873)	ATCTGGGACTACAAAAGAGATGCTCCAGCACATATATCTACTATTGGTGTTTGTTCTATGACTGACATAGCC
hCoV-19/Botswana/R69B55_BHP_916539/2021_EPI_ISL_90...	(19819)	ATCTGGGACTACAAAAGAGATGCTCCAGCACATATATCTACTATTGGTGTTTGTTCTATGACTGACATAGCC
hCoV-19/India/HR-MDU-IGIB1210605800489930/2022_EPI...	(19861)	ATCTGGGACTACAAAAGAGATGCTCCAGCACATATATCTACTATTGGTGTTTGTTCTATGACTGACATAGCC
hCoV-19/England/PLYM-332B917/2022_EPI_ISL_9062229_...	(19811)	ATCTGGGACTACAAAAGAGATGCTCCAGCACATATATCTACTATTGGTGTTTGTTCTATGACTGACATAGCC
hCoV-19/Germany/HE-RKI-I-438397/2022_EPI_ISL_905522...	(19825)	ATCTGGGACTACAAAAGAGATGCTCCAGCACATATATCTACTATTGGTGTTTGTTCTATGACTGACATAGCC
hCoV-19/USA/DE-CDC-LC0472738/2021_EPI_ISL_9049423...	(19789)	ATCTGGGACTACAAAAGAGATGCTCCAGCACATATATCTACTATTGGTGTTTGTTCTATGACTGACATAGCC

Section 278

		19945 19950 19960 19970 19980 19990 20000 20016
	(19945)	
SARS-CoV-2 Reference Genome NC_045512.2	(19945)	AAGAAACCAACTGAAACGATTTGTGCACCACTCACTGTCTTTTTTGATGGTAGAGTTGATGGTCAAGTAGAC
hCoV-19/Botswana/R69B55_BHP_916539/2021_EPI_ISL_90...	(19891)	AAGAAACCAACTGAAACGATTTGTGCACCACTCACTGTCTTTTTTGATGGTAGAGTTGATGGTCAAGTAGAC
hCoV-19/India/HR-MDU-IGIB1210605800489930/2022_EPI...	(19933)	AAGAAACCAACTGAAACGATTTGTGCACCACTCACTGTCTTTTTTGATGGTAGAGTTGATGGTCAAGTAGAC
hCoV-19/England/PLYM-332B917/2022_EPI_ISL_9062229_...	(19883)	AAGAAACCAACTGAAACGATTTGTGCACCACTCACTGTCTTTTTTGATGGTAGAGTTGATGGTCAAGTAGAC
hCoV-19/Germany/HE-RKI-I-438397/2022_EPI_ISL_905522...	(19897)	AAGAAACCAACTGAAACGATTTGTGCACCACTCACTGTCTTTTTTGATGGTAGAGTTGATGGTCAAGTAGAC
hCoV-19/USA/DE-CDC-LC0472738/2021_EPI_ISL_9049423...	(19861)	AAGAAACCAACTGAAACGATTTGTGCACCACTCACTGTCTTTTTTGATGGTAGAGTTGATGGTCAAGTAGAC

Section 279

		20017 20030 20040 20050 20060 20070 20088
	(20017)	
SARS-CoV-2 Reference Genome NC_045512.2	(20017)	TTATTTAGAAATGCCCGTAATGGTGTTCTTATTACAGAAGGTAGTGTTAAAGGTTTACAACCATCTGTAGGT
hCoV-19/Botswana/R69B55_BHP_916539/2021_EPI_ISL_90...	(19963)	TTATTTAGAAATGCCCGTAATGGTGTTCTTATTACAGAAGGTAGTGTTAAAGGTTTACAACCATCTGTAGGT
hCoV-19/India/HR-MDU-IGIB1210605800489930/2022_EPI...	(20005)	TTATTTAGAAATGCCCGTAATGGTGTTCTTATTACAGAAGGTAGTGTTAAAGGTTTACAACCATCTGTAGGT
hCoV-19/England/PLYM-332B917/2022_EPI_ISL_9062229_...	(19955)	TTATTTAGAAATGCCCGTAATGGTGTTCTTATTACAGAAGGTAGTGTTAAAGGTTTACAACCATCTGTAGGT
hCoV-19/Germany/HE-RKI-I-438397/2022_EPI_ISL_905522...	(19969)	TTATTTAGAAATGCCCGTAATGGTGTTCTTATTACAGAAGGTAGTGTTAAAGGTTTACAACCATCTGTAGGT
hCoV-19/USA/DE-CDC-LC0472738/2021_EPI_ISL_9049423...	(19933)	TTATTTAGAAATGCCCGTAATGGTGTTCTTATTACAGAAGGTAGTGTTAAAGGTTTACAACCATCTGTAGGT

Section 280

		20089 20100 20110 20120 20130 20140 20150 20160
	(20089)	
SARS-CoV-2 Reference Genome NC_045512.2	(20089)	CCCAAACAAGCTAGTCTTAATGGAGTCACATTAATTGGAGAAGCCGTAAAAACACAGTTCAATTATTATAAG
hCoV-19/Botswana/R69B55_BHP_916539/2021_EPI_ISL_90...	(20035)	CCCAAACAAGCTAGTCTTAATGGAGTCACATTAATTGGAGAAGCCGTAAAAACACAGTTCAATTATTATAAG
hCoV-19/India/HR-MDU-IGIB1210605800489930/2022_EPI...	(20077)	CCCAAACAAGCTAGTCTTAATGGAGTCACATTAATTGGAGAAGCCGTAAAAACACAGTTCAATTATTATAAG
hCoV-19/England/PLYM-332B917/2022_EPI_ISL_9062229_...	(20027)	CCCAAACAAGCTAGTCTTAATGGAGTCACATTAATTGGAGAAGCCGTAAAAACACAGTTCAATTATTATAAG
hCoV-19/Germany/HE-RKI-I-438397/2022_EPI_ISL_905522...	(20041)	CCCAAACAAGCTAGTCTTAATGGAGTCACATTAATTGGAGAAGCCGTAAAAACACAGTTCAATTATTATAAG
hCoV-19/USA/DE-CDC-LC0472738/2021_EPI_ISL_9049423...	(20005)	CCCAAACAAGCTAGTCTTAATGGAGTCACATTAATTGGAGAAGCCGTAAAAACACAGTTCAATTATTATAAG

Section 281

	(20161)	20161 20170 20180 20190 20200 20210 20220 20232
SARS-CoV-2 Reference Genome NC_045512.2	(20161)	AAAGTTGATGGTGTTGTCCAACAATTACCTGAAACTTACTTTACTCAGAGTAGAAATTTACAAGAATTTAAA
hCoV-19/Botswana/R69B55_BHP_916539/2021_EPI_ISL_90...	(20107)	AAAGTTGATGGTGTTGTCCAACAATTACCTGAAACTTACTTTACTCAGAGTAGAAATTTACAAGAATTTAAA
hCoV-19/India/HR-MDU-IGIB1210605800489930/2022_EPI...	(20149)	AAAGTTGATGGTGTTGTCCAACAATTACCTGAAACTTACTTTACTCAGAGTAGAAATTTACAAGAATTTAAA
hCoV-19/England/PLYM-332B917/2022_EPI_ISL_9062229_...	(20099)	AAAGTTGATGGTGTTGTCCAACAATTACCTGAAACTTACTTTACTCAGAGTAGAAATTTACAAGAATTTAAA
hCoV-19/Germany/HE-RKI-I-438397/2022_EPI_ISL_905522...	(20113)	AAAGTTGATGGTGTTGTCCAACAATTACCTGAAACTTACTTTACTCAGAGTAGAAATTTACAAGAATTTAAA
hCoV-19/USA/DE-CDC-LC0472738/2021_EPI_ISL_9049423...	(20077)	AAAGTTGATGGTGTTGTCCAACAATTACCTGAAACTTACTTTACTCAGAGTAGAAATTTACAAGAATTTAAA

Section 282

	(20233)	20233 20240 20250 20260 20270 20280 20290 20304
SARS-CoV-2 Reference Genome NC_045512.2	(20233)	CCCAGGAGTCAAATGGAAATTGATTTCTTAGAATTAGCTATGGATGAATTCATTGAACGGTATAAATTAGAA
hCoV-19/Botswana/R69B55_BHP_916539/2021_EPI_ISL_90...	(20179)	CCCAGGAGTCAAATGGAAATTGATTTCTTAGAATTAGCTATGGATGAATTCATTGAACGGTATAAATTAGAA
hCoV-19/India/HR-MDU-IGIB1210605800489930/2022_EPI...	(20221)	CCCAGGAGTCAAATGGAAATTGATTTCTTAGAATTAGCTATGGATGAATTCATTGAACGGTATAAATTAGAA
hCoV-19/England/PLYM-332B917/2022_EPI_ISL_9062229_...	(20171)	CCCAGGAGTCAAATGGAAATTGATTTCTTAGAATTAGCTATGGATGAATTCATTGAACGGTATAAATTAGAA
hCoV-19/Germany/HE-RKI-I-438397/2022_EPI_ISL_905522...	(20185)	CCCAGGAGTCAAATGGAAATTGATTTCTTAGAATTAGCTATGGATGAATTCATTGAACGGTATAAATTAGAA
hCoV-19/USA/DE-CDC-LC0472738/2021_EPI_ISL_9049423...	(20149)	CCCAGGAGTCAAATGGAAATTGATTTCTTAGAATTAGCTATGGATGAATTCATTGAACGGTATAAATTAGAA

Section 283

	(20305)	20305 20310 20320 20330 20340 20350 20360 20376
SARS-CoV-2 Reference Genome NC_045512.2	(20305)	GGCTATGCCTTCGAACATATCGTTTATGGAGATTTTAGTCATAGTCAGTTAGGTGGTTTACATCTACTGATT
hCoV-19/Botswana/R69B55_BHP_916539/2021_EPI_ISL_90...	(20251)	GGCTATGCCTTCGAACATATCGTTTATGGAGATTTTAGTCATAGTCAGTTAGGTGGTTTACATCTACTGATT
hCoV-19/India/HR-MDU-IGIB1210605800489930/2022_EPI...	(20293)	GGCTATGCCTTCGAACATATCGTTTATGGAGATTTTAGTCATAGTCAGTTAGGTGGTTTACATCTACTGATT
hCoV-19/England/PLYM-332B917/2022_EPI_ISL_9062229_...	(20243)	GGCTATGCCTTCGAACATATCGTTTATGGAGATTTTAGTCATAGTCAGTTAGGTGGTTTACATCTACTGATT
hCoV-19/Germany/HE-RKI-I-438397/2022_EPI_ISL_905522...	(20257)	GGCTATGCCTTCGAACATATCGTTTATGGAGATTTTAGTCATAGTCAGTTAGGTGGTTTACATCTACTGATT
hCoV-19/USA/DE-CDC-LC0472738/2021_EPI_ISL_9049423...	(20221)	GGCTATGCCTTCGAACATATCGTTTATGGAGATTTTAGTCATAGTCAGTTAGGTGGTTTACATCTACTGATT

Section 284

	(20377)	20377 20390 20400 20410 20420 20430 20448
SARS-CoV-2 Reference Genome NC_045512.2	(20377)	GGACTAGCTAAACGTTTTAAGGAATCACCTTTTGAATTAGAAGATTTTATTCCTATGGACAGTACAGTTAAA
hCoV-19/Botswana/R69B55_BHP_916539/2021_EPI_ISL_90...	(20323)	GGACTAGCTAAACGTTTTAAGGAATCACCTTTTGAATTAGAAGATTTTATTCCTATGGACAGTACAGTTAAA
hCoV-19/India/HR-MDU-IGIB1210605800489930/2022_EPI...	(20365)	GGACTAGCTAAACGTTTTAAGGAATCACCTTTTGAATTAGAAGATTTTATTCCTATGGACAGTACAGTTAAA
hCoV-19/England/PLYM-332B917/2022_EPI_ISL_9062229_...	(20315)	GGACTAGCTAAACGTTTTAAGGAATCACCTTTTGAATTAGAAGATTTTATTCCTATGGACAGTACAGTTAAA
hCoV-19/Germany/HE-RKI-I-438397/2022_EPI_ISL_905522...	(20329)	GGACTAGCTAAACGTTTTAAGGAATCACCTTTTGAATTAGAAGATTTTATTCCTATGGACAGTACAGTTAAA
hCoV-19/USA/DE-CDC-LC0472738/2021_EPI_ISL_9049423...	(20293)	GGACTAGCTAAACGTTTTAAGGAATCACCTTTTGAATTAGAAGATTTTATTCCTATGGACAGTACAGTTAAA

Section 285

```
                                                        (20449) 20449      20460     20470     20480     20490     20500     20510   20520
SARS-CoV-2 Reference Genome NC_045512.2                 (20449) AACTATTTCATAACAGATGCGCAAACAGGTTCATCTAAGTGTGTGTGTTCTGTTATTGATTTATTACTTGAT
hCoV-19/Botswana/R69B55_BHP_916539/2021_EPI_ISL_90...   (20395) AACTATTTCATAACAGATGCGCAAACAGGTTCATCTAAGTGTGTGTGTTCTGTTATTGATTTATTACTTGAT
hCoV-19/India/HR-MDU-IGIB1210605800489930/2022_EPI...   (20437) AACTATTTCATAACAGATGCGCAAACAGGTTCATCTAAGTGTGTGTGTTCTGTTATTGATTTATTACTTGAT
hCoV-19/England/PLYM-332B917/2022_EPI_ISL_9062229_...   (20387) AACTATTTCATAACAGATGCGCAAACAGGTTCATCTAAGTGTGTGTGTTCTGTTATTGATTTATTACTTGAT
hCoV-19/Germany/HE-RKI-I-438397/2022_EPI_ISL_905522...  (20401) AACTATTTCATAACAGATGCGCAAACAGGTTCATCTAAGTGTGTGTGTTCTGTTATTGATTTATTACTTGAT
hCoV-19/USA/DE-CDC-LC0472738/2021_EPI_ISL_9049423...    (20365) AACTATTTCATAACAGATGCGCAAACAGGTTCATCTAAGTGTGTGTGTTCTGTTATTGATTTATTACTTGAT
```

Section 286

```
                                                        (20521) 20521    20530     20540     20550     20560     20570     20580      20592
SARS-CoV-2 Reference Genome NC_045512.2                 (20521) GATTTTGTTGAAATAATAAAATCCCAAGATTTATCTGTAGTTTCTAAGGTTGTCAAAGTGACTATTGACTAT
hCoV-19/Botswana/R69B55_BHP_916539/2021_EPI_ISL_90...   (20467) GATTTTGTTGAAATAATAAAATCCCAAGATTTATCTGTAGTTTCTAAGGTTGTCAAAGTGACTATTGACTAT
hCoV-19/India/HR-MDU-IGIB1210605800489930/2022_EPI...   (20509) GATTTTGTTGAAATAATAAAATCCCAAGATTTATCTGTAGTTTCTAAGGTTGTCAAAGTGACTATTGACTAT
hCoV-19/England/PLYM-332B917/2022_EPI_ISL_9062229_...   (20459) GATTTTGTTGAAATAATAAAATCCCAAGATTTATCTGTAGTTTCTAAGGTTGTCAAAGTGACTATTGACTAT
hCoV-19/Germany/HE-RKI-I-438397/2022_EPI_ISL_905522...  (20473) GATTTTGTTGAAATAATAAAATCCCAAGATTTATCTGTAGTTTCTAAGGTTGTCAAAGTGACTATTGACTAT
hCoV-19/USA/DE-CDC-LC0472738/2021_EPI_ISL_9049423...    (20437) GATTTTGTTGAAATAATAAAATCCCAAGATTTATCTGTAGTTTCTAAGGTTGTCAAAGTGACTATTGACTAT
```

Section 287

```
                                                        (20593) 20593  20600     20610     20620     20630     20640     20650        20664
SARS-CoV-2 Reference Genome NC_045512.2                 (20593) ACAGAAATTTCATTTATGCTTTGGTGTAAAGATGGCCATGTAGAAACATTTTACCCAAAATTACAATCTAGT
hCoV-19/Botswana/R69B55_BHP_916539/2021_EPI_ISL_90...   (20539) ACAGAAATTTCATTTATGCTTTGGTGTAAAGATGGCCATGTAGAAACATTTTACCCAAAATTACAATCTAGT
hCoV-19/India/HR-MDU-IGIB1210605800489930/2022_EPI...   (20581) ACAGAAATTTCATTTATGCTTTGGTGTAAAGATGGCCATGTAGAAACATTTTACCCAAAATTACAATCTAGT
hCoV-19/England/PLYM-332B917/2022_EPI_ISL_9062229_...   (20531) ACAGAAATTTCATTTATGCTTTGGTGTAAAGATGGCCATGTAGAAACATTTTACCCAAAATTACAATCTAGT
hCoV-19/Germany/HE-RKI-I-438397/2022_EPI_ISL_905522...  (20545) ACAGAAATTTCATTTATGCTTTGGTGTAAAGATGGCCATGTAGAAACATTTTACCCAAAATTACAATCTAGT
hCoV-19/USA/DE-CDC-LC0472738/2021_EPI_ISL_9049423...    (20509) ACAGAAATTTCATTTATGCTTTGGTGTAAAGATGGCCATGTAGAAACATTTTACCCAAAATTACAATCTAGT
```

Section 288

```
                                                        (20665) 20665 20670     20680     20690     20700     20710     20720           20736
SARS-CoV-2 Reference Genome NC_045512.2                 (20665) CAAGCGTGGCAACCGGGTGTTGCTATGCCTAATCTTTACAAAATGCAAAGAATGCTATTAGAAAAGTGTGAC
hCoV-19/Botswana/R69B55_BHP_916539/2021_EPI_ISL_90...   (20611) CAAGCGTGGCAACCGGGTGTTGCTATGCCTAATCTTTACAAAATGCAAAGAATGCTATTAGAAAAGTGTGAC
hCoV-19/India/HR-MDU-IGIB1210605800489930/2022_EPI...   (20653) CAAGCGTGGCAACCGGGTGTTGCTATGCCTAATCTTTACAAAATGCAAAGAATGCTATTAGAAAAGTGTGAC
hCoV-19/England/PLYM-332B917/2022_EPI_ISL_9062229_...   (20603) CAAGCGTGGCAACCGGGTGTTGCTATGCCTAATCTTTACAAAATGCAAAGAATGCTATTAGAAAAGTGTGAC
hCoV-19/Germany/HE-RKI-I-438397/2022_EPI_ISL_905522...  (20617) CAAGCGTGGCAACCGGGTGTTGCTATGCCTAATCTTTACAAAATGCAAAGAATGCTATTAGAAAAGTGTGAC
hCoV-19/USA/DE-CDC-LC0472738/2021_EPI_ISL_9049423...    (20581) CAAGCGTGGCAACCGGGTGTTGCTATGCCTAATCTTTACAAAATGCAAAGAATGCTATTAGAAAAGTGTGAC
```

Section 289

	(20737)	20737 20750 20760 20770 20780 20790 20808
SARS-CoV-2 Reference Genome NC_045512.2	(20737)	CTTCAAAATTATGGTGATAGTGCAACATTACCTAAAGGCATAATGATGAATGTCGCAAAATATACTCAACTG
hCoV-19/Botswana/R69B55_BHP_916539/2021_EPI_ISL_90...	(20683)	CTTCAAAATTATGGTGATAGTGCAACATTACCTAAAGGCATAATGATGAATGTCGCAAAATATACTCAACTG
hCoV-19/India/HR-MDU-IGIB1210605800489930/2022_EPI...	(20725)	CTTCAAAATTATGGTGATAGTGCAACATTACCTAAAGGCATAATGATGAATGTCGCAAAATATACTCAACTG
hCoV-19/England/PLYM-332B917/2022_EPI_ISL_9062229_...	(20675)	CTTCAAAATTATGGTGATAGTGCAACATTACCTAAAGGCATAATGATGAATGTCGCAAAATATACTCAACTG
hCoV-19/Germany/HE-RKI-I-438397/2022_EPI_ISL_905522...	(20689)	CTTCAAAATTATGGTGATAGTGCAACATTACCTAAAGGCATAATGATGAATGTCGCAAAATATACTCAACTG
hCoV-19/USA/DE-CDC-LC0472738/2021_EPI_ISL_9049423...	(20653)	CTTCAAAATTATGGTGATAGTGCAACATTACCTAAAGGCATAATGATGAATGTCGCAAAATATACTCAACTG

Section 290

	(20809)	20809 20820 20830 20840 20850 20860 20870 20880
SARS-CoV-2 Reference Genome NC_045512.2	(20809)	TGTCAATATTTAAACACATTAACATTAGCTGTACCCTATAATATGAGAGTTATACATTTTGGTGCTGGTTCT
hCoV-19/Botswana/R69B55_BHP_916539/2021_EPI_ISL_90...	(20755)	TGTCAATATTTAAACACATTAACATTAGCTGTACCCTATAATATGAGAGTTATACATTTTGGTGCTGGTTCT
hCoV-19/India/HR-MDU-IGIB1210605800489930/2022_EPI...	(20797)	TGTCAATATTTAAACACATTAACATTAGCTGTACCCTATAATATGAGAGTTATACATTTTGGTGCTGGTTCT
hCoV-19/England/PLYM-332B917/2022_EPI_ISL_9062229_...	(20747)	TGTCAATATTTAAACACATTAACATTAGCTGTACCCTATAATATGAGAGTTATACATTTTGGTGCTGGTTCT
hCoV-19/Germany/HE-RKI-I-438397/2022_EPI_ISL_905522...	(20761)	TGTCAATATTTAAACACATTAACATTAGCTGTACCCTATAATATGAGAGTTATACATTTTGGTGCTGGTTCT
hCoV-19/USA/DE-CDC-LC0472738/2021_EPI_ISL_9049423...	(20725)	TGTCAATATTTAAACACATTAACATTAGCTGTACCCTATAATATGAGAGTTATACATTTTGGTGCTGGTTCT

Section 291

	(20881)	20881 20890 20900 20910 20920 20930 20940 20952
SARS-CoV-2 Reference Genome NC_045512.2	(20881)	GATAAAGGAGTTGCACCAGGTACAGCTGTTTTAAGACAGTGGTTGCCTACGGGTACGCTGCTTGTCGATTCA
hCoV-19/Botswana/R69B55_BHP_916539/2021_EPI_ISL_90...	(20827)	GATAAAGGAGTTGCACCAGGTACAGCTGTTTTAAGACAGTGGTTGCCTACGGGTACGCTGCTTGTCGATTCA
hCoV-19/India/HR-MDU-IGIB1210605800489930/2022_EPI...	(20869)	GATAAAGGAGTTGCACCAGGTACAGCTGTTTTAAGACAGTGGTTGCCTACGGGTACGCTGCTTGTCGATTCA
hCoV-19/England/PLYM-332B917/2022_EPI_ISL_9062229_...	(20819)	GATAAAGGAGTTGCACCAGGTACAGCTGTTTTAAGACAGTGGTTGCCTACGGGTACGCTGCTTGTCGATTCA
hCoV-19/Germany/HE-RKI-I-438397/2022_EPI_ISL_905522...	(20833)	GATAAAGGAGTTGCACCAGGTACAGCTGTTTTAAGACAGTGGTTGCCTACGGGTACGCTGCTTGTCGATTCA
hCoV-19/USA/DE-CDC-LC0472738/2021_EPI_ISL_9049423...	(20797)	GATAAAGGAGTTGCACCAGGTACAGCTGTTTTAAGACAGTGGTTGCCTACGGGTACGCTGCTTGTCGATTCA

Section 292

	(20953)	20953 20960 20970 20980 20990 21000 21010 21024
SARS-CoV-2 Reference Genome NC_045512.2	(20953)	GATCTTAATGACTTTGTCTCTGATGCAGATTCAACTTTGATTGGTGATTGTGCAACTGTACATACAGCTAAT
hCoV-19/Botswana/R69B55_BHP_916539/2021_EPI_ISL_90...	(20899)	GATCTTAATGACTTTGTCTCTGATGCAGATTCAACTTTGATTGGTGATTGTGCAACTGTACATACAGCTAAT
hCoV-19/India/HR-MDU-IGIB1210605800489930/2022_EPI...	(20941)	GATCTTAATGACTTTGTCTCTGATGCAGATTCAACTTTGATTGGTGATTGTGCAACTGTACATACAGCTAAT
hCoV-19/England/PLYM-332B917/2022_EPI_ISL_9062229_...	(20891)	GATCTTAATGACTTTGTCTCTGATGCAGATTCAACTTTGATTGGTGATTGTGCAACTGTACATACAGCTAAT
hCoV-19/Germany/HE-RKI-I-438397/2022_EPI_ISL_905522...	(20905)	GATCTTAATGACTTTGTCTCTGATGCAGATTCAACTTTGATTGGTGATTGTGCAACTGTACATACAGCTAAT
hCoV-19/USA/DE-CDC-LC0472738/2021_EPI_ISL_9049423...	(20869)	GATCTTAATGACTTTGTCTCTGATGCAGATTCAACTTTGATTGGTGATTGTGCAACTGTACATACAGCTAAT

Section 293

	(21025)	21025 21030 21040 21050 21060 21070 21080 21096
SARS-CoV-2 Reference Genome NC_045512.2	(21025)	AAATGGGATCTCATTATTAGTGATATGTACGACCCTAAGACTAAAAATGTTACAAAAGAAAATGACTCTAAA
hCoV-19/Botswana/R69B55_BHP_916539/2021_EPI_ISL_90...	(20971)	AAATGGGATCTCATTATTAGTGATATGTACGACCCTAAGACTAAAAATGTTACAAAAGAAAATGACTCTAAA
hCoV-19/India/HR-MDU-IGIB1210605800489930/2022_EPI...	(21013)	AAATGGGATCTCATTATTAGTGATATGTACGACCCTAAGACTAAAAATGTTACAAAAGAAAATGACTCTAAA
hCoV-19/England/PLYM-332B917/2022_EPI_ISL_9062229_...	(20963)	AAATGGGATCTCATTATTAGTGATATGTACGACCCTAAGACTAAAAATGTTACAAAAGAAAATGACTCTAAA
hCoV-19/Germany/HE-RKI-I-438397/2022_EPI_ISL_905522...	(20977)	AAATGGGATCTCATTATTAGTGATATGTACGACCCTAAGACTAAAAATGTTACAAAAGAAAATGACTCTAAA
hCoV-19/USA/DE-CDC-LC0472738/2021_EPI_ISL_9049423...	(20941)	AAATGGGATCTCATTATTAGTGATATGTACGACCCTAAGACTAAAAATGTTACAAAAGAAAATGACTCTAAA

Section 294

	(21097)	21097 21110 21120 21130 21140 21150 21168
SARS-CoV-2 Reference Genome NC_045512.2	(21097)	GAGGGTTTTTTCACTTACATTTGTGGGTTTATACAACAAAAGCTAGCTCTTGGAGGTTCCGTGGCTATAAAG
hCoV-19/Botswana/R69B55_BHP_916539/2021_EPI_ISL_90...	(21043)	GAGGGTTTTTTCACTTACATTTGTGGGTTTATACAACAAAAGCTAGCTCTTGGAGGTTCCGTGGCTATAAAG
hCoV-19/India/HR-MDU-IGIB1210605800489930/2022_EPI...	(21085)	GAGGGTTTTTTCACTTACATTTGTGGGTTTATACAACAAAAGCTAGCTCTTGGAGGTTCCGTGGCTATAAAG
hCoV-19/England/PLYM-332B917/2022_EPI_ISL_9062229_...	(21035)	GAGGGTTTTTTCACTTACATTTGTGGGTTTATACAACAAAAGCTAGCTCTTGGAGGTTCCGTGGCTATAAAG
hCoV-19/Germany/HE-RKI-I-438397/2022_EPI_ISL_905522...	(21049)	GAGGGTTTTTTCACTTACATTTGTGGGTTTATACAACAAAAGCTAGCTCTTGGAGGTTCCGTGGCTATAAAG
hCoV-19/USA/DE-CDC-LC0472738/2021_EPI_ISL_9049423...	(21013)	GAGGGTTTTTTCACTTACATTTGTGGGTTTATACAACAAAAGCTAGCTCTTGGAGGTTCCGTGGCTATAAAG

Section 295

	(21169)	21169 21180 21190 21200 21210 21220 21230 21240
SARS-CoV-2 Reference Genome NC_045512.2	(21169)	ATAACAGAACATTCTTGGAATGCTGATCTTTATAAGCTCATGGGACACTTCGCATGGTGGACAGCCTTTGTT
hCoV-19/Botswana/R69B55_BHP_916539/2021_EPI_ISL_90...	(21115)	ATAACAGAACATTCTTGGAATGCTGATCTTTATAAGCTCATGGGACACTTCGCATGGTGGACAGCCTTTGTT
hCoV-19/India/HR-MDU-IGIB1210605800489930/2022_EPI...	(21157)	ATAACAGAACATTCTTGGAATGCTGATCTTTATAAGCTCATGGGACACTTCGCATGGTGGACAGCCTTTGTT
hCoV-19/England/PLYM-332B917/2022_EPI_ISL_9062229_...	(21107)	ATAACAGAACATTCTTGGAATGCTGATCTTTATAAGCTCATGGGACACTTCGCATGGTGGACAGCCTTTGTT
hCoV-19/Germany/HE-RKI-I-438397/2022_EPI_ISL_905522...	(21121)	ATAACAGAACATTCTTGGAATGCTGATCTTTATAAGCTCATGGGACACTTCGCATGGTGGACAGCCTTTGTT
hCoV-19/USA/DE-CDC-LC0472738/2021_EPI_ISL_9049423...	(21085)	ATAACAGAACATTCTTGGAATGCTGATCTTTATAAGCTCATGGGACACTTCGCATGGTGGACAGCCTTTGTT

Section 296

	(21241)	21241 21250 21260 21270 21280 21290 21300 21312
SARS-CoV-2 Reference Genome NC_045512.2	(21241)	ACTAATGTGAATGCGTCATCATCTGAAGCATTTTTAATTGGATGTAATTATCTTGGCAAACCACGCGAACAA
hCoV-19/Botswana/R69B55_BHP_916539/2021_EPI_ISL_90...	(21187)	ACTAATGTGAATGCGTCATCATCTGAAGCATTTTTAATTGGATGTAATTATCTTGGCAAACCACGCGAACAA
hCoV-19/India/HR-MDU-IGIB1210605800489930/2022_EPI...	(21229)	ACTAATGTGAATGCGTCATCATCTGAAGCATTTTTAATTGGATGTAATTATCTTGGCAAACCACGCGAACAA
hCoV-19/England/PLYM-332B917/2022_EPI_ISL_9062229_...	(21179)	ACTAATGTGAATGCGTCATCATCTGAAGCATTTTTAATTGGATGTAATTATCTTGGCAAACCACGCGAACAA
hCoV-19/Germany/HE-RKI-I-438397/2022_EPI_ISL_905522...	(21193)	ACTAATGTGAATGCGTCATCATCTGAAGCATTTTTAATTGGATGTAATTATCTTGGCAAACCACGCGAACAA
hCoV-19/USA/DE-CDC-LC0472738/2021_EPI_ISL_9049423...	(21157)	ACTAATGTGAATGCGTCATCATCTGAAGCATTTTTAATTGGATGTAATTATCTTGGCAAACCACGCGAACAA

Section 297

		21313 21320 21330 21340 21350 21360 21370 21384
	(21313)	
SARS-CoV-2 Reference Genome NC_045512.2	(21313)	ATAGATGGTTATGTCATGCATGCAAATTACATATTTTGGAGGAATACAAATCCAATTCAGTTGTCTTCCTAT
hCoV-19/Botswana/R69B55_BHP_916539/2021_EPI_ISL_90...	(21259)	ATAGATGGTTATGTCATGCATGCAAATTACATATTTTGGAGGAATACAAATCCAATTCAGTTGTCTTCCTAT
hCoV-19/India/HR-MDU-IGIB1210605800489930/2022_EPI...	(21301)	ATAGATGGTTATGTCATGCATGCAAATTACATATTTTGGAGGAATACAAATCCAATTCAGTTGTCTTCCTAT
hCoV-19/England/PLYM-332B917/2022_EPI_ISL_9062229_...	(21251)	ATAGATGGTTATGTCATGCATGCAAATTACATATTTTGGAGGAATACAAATCCAATTCAGTTGTCTTCCTAT
hCoV-19/Germany/HE-RKI-I-438397/2022_EPI_ISL_905522...	(21265)	ATAGATGGTTATGTCATGCATGCAAATTACATATTTTGGAGGAATACAAATCCAATTCAGTTGTCTTCCTAT
hCoV-19/USA/DE-CDC-LC0472738/2021_EPI_ISL_9049423...	(21229)	ATAGATGGTTATGTCATGCATGCAAATTACATATTTTGGAGGAATACAAATCCAATTCAGTTGTCTTCCTAT

Section 298

		21385 21390 21400 21410 21420 21430 21440 21456
	(21385)	
SARS-CoV-2 Reference Genome NC_045512.2	(21385)	TCTTTATTTGACATGAGTAAATTTCCCCTTAAATTAAGGGGTACTGCTGTTATGTCTTTAAAAGAAGGTCAA
hCoV-19/Botswana/R69B55_BHP_916539/2021_EPI_ISL_90...	(21331)	TCTTTATTTGACATGAGTAAATTTCCCCTTAAATTAAGGGGTACTGCTGTTATGTCTTTAAAAGAAGGTCAA
hCoV-19/India/HR-MDU-IGIB1210605800489930/2022_EPI...	(21373)	TCTTTATTTGACATGAGTAAATTTCCCCTTAAATTAAGGGGTACTGCTGTTATGTCTTTAAAAGAAGGTCAA
hCoV-19/England/PLYM-332B917/2022_EPI_ISL_9062229_...	(21323)	TCTTTATTTGACATGAGTAAATTTCCCCTTAAATTAAGGGGTACTGCTGTTATGTCTTTAAAAGAAGGTCAA
hCoV-19/Germany/HE-RKI-I-438397/2022_EPI_ISL_905522...	(21337)	TCTTTATTTGACATGAGTAAATTTCCCCTTAAATTAAGGGGTACTGCTGTTATGTCTTTAAAAGAAGGTCAA
hCoV-19/USA/DE-CDC-LC0472738/2021_EPI_ISL_9049423...	(21301)	TCTTTATTTGACATGAGTAAATTTCCCCTTAAATTAAGGGGTACTGCTGTTATGTCTTTAAAAGAAGGTCAA

Section 299

		21457 21470 21480 21490 21500 21510 21528
	(21457)	
SARS-CoV-2 Reference Genome NC_045512.2	(21457)	ATCAATGATATGATTTTATCTCTTCTTAGTAAAGGTAGACTTATAATTAGAGAAAACAACAGAGTTGTTATT
hCoV-19/Botswana/R69B55_BHP_916539/2021_EPI_ISL_90...	(21403)	ATCAATGATATGATTTTATCTCTTCTTAGTAAAGGTAGACTTATAATTAGAGAAAACAACAGAGTTGTTATT
hCoV-19/India/HR-MDU-IGIB1210605800489930/2022_EPI...	(21445)	ATCAATGATATGATTTTATCTCTTCTTAGTAAAGGTAGACTTATAATTAGAGAAAACAACAGAGTTGTTATT
hCoV-19/England/PLYM-332B917/2022_EPI_ISL_9062229_...	(21395)	ATCAATGATATGATTTTATCTCTTCTTAGTAAAGGTAGACTTATAATTAGAGAAAACAACAGAGTTGTTATT
hCoV-19/Germany/HE-RKI-I-438397/2022_EPI_ISL_905522...	(21409)	ATCAATGATATGATTTTATCTCTTCTTAGTAAAGGTAGACTTATAATTAGAGAAAACAACAGAGTTGTTATT
hCoV-19/USA/DE-CDC-LC0472738/2021_EPI_ISL_9049423...	(21373)	ATCAATGATATGATTTTATCTCTTCTTAGTAAAGGTAGACTTATAATTAGAGAAAACAACAGAGTTGTTATT

Section 300

		21529 21540 21550 21560 21570 21580 21590 21600
	(21529)	
SARS-CoV-2 Reference Genome NC_045512.2	(21529)	TCTAGTGATGTTCTTGTTAACAACTAAACGAACAATGTTTGTTTTTCTTGTTTTATTGCCACTAGTCTCTAG
hCoV-19/Botswana/R69B55_BHP_916539/2021_EPI_ISL_90...	(21475)	TCTAGTGATGTTCTTGTTAACAACTAAACGAACAATGTTTGTTTTTCTTGTTTTATTGCCACTAGTCTCTAG
hCoV-19/India/HR-MDU-IGIB1210605800489930/2022_EPI...	(21517)	TCTAGTGATGTTCTTGTTAACAACTAAACGAACAATGTTTGTTTTTCTTGTTTTATTGCCACTAGTCTCTAG
hCoV-19/England/PLYM-332B917/2022_EPI_ISL_9062229_...	(21467)	TCTAGTGATGTTCTTGTTAACAACTAAACGAACAATGTTTGTTTTTCTTGTTTTATTGCCACTAGTCTCTAG
hCoV-19/Germany/HE-RKI-I-438397/2022_EPI_ISL_905522...	(21481)	TCTAGTGATGTTCTTGTTAACAACTAAACGAACAATGTTTGTTTTTCTTGTTTTATTGCCACTAGTCTCTAG
hCoV-19/USA/DE-CDC-LC0472738/2021_EPI_ISL_9049423...	(21445)	TCTAGTGATGTTCTTGTTAACAACTAAACGAACAATGTTTGTTTTTCTTGTTTTATTGCCACTAGTCTCTAG

```
                                                                        Section 301
                                                                (21601) 21601    21610      21620      21630      21640      21650      21660     21672
SARS-CoV-2 Reference Genome NC_045512.2                         (21601) TCAGTGTGTTAATCTTACAACCAGAACTCAATTACCCCCTGCATACACTAATTCTTTCACACGTGGTGTTTA
hCoV-19/Botswana/R69B55_BHP_916539/2021_EPI_ISL_90...           (21547) TCAGTGTGTTAATCTTACAACCAGAACTCAATTACCCCCTGCATACACTAATTCTTTCACACGTGGTGTTTA
hCoV-19/India/HR-MDU-IGIB1210605800489930/2022_EPI...           (21589) TCAGTGTGTTAATCTTACAACCAGAACTCAATTACCCCCTGCATACACTAATTCTTTCACACGTGGTGTTTA
hCoV-19/England/PLYM-332B917/2022_EPI_ISL_9062229_...           (21539) TCAGTGTGTTAATCTTACAACCAGAACTCAATTACCCCCTGCATACACTAATTCTTTCACACGTGGTGTTTA
hCoV-19/Germany/HE-RKI-I-438397/2022_EPI_ISL_905522...          (21553) TCAGTGTGTTAATCTTACAACCAGAACTCAATTACCCCCTGCATACACTAATTCTTTCACACGTGGTGTTTA
hCoV-19/USA/DE-CDC-LC0472738/2021_EPI_ISL_9049423...            (21517) TCAGTGTGTTAATCTTACAACCAGAACTCAATTACCCCCTGCATACACTAATTCTTTCACACGTGGTGTTTA
                                                                        Section 302
                                                                (21673) 21673  21680      21690      21700      21710      21720      21730       21744
SARS-CoV-2 Reference Genome NC_045512.2                         (21673) TTACCCTGACAAAGTTTTCAGATCCTCAGTTTTACATTCAACTCAGGACTTGTTCTTACCTTTCTTTTCCAA
hCoV-19/Botswana/R69B55_BHP_916539/2021_EPI_ISL_90...           (21619) TTACCCTGACAAAGTTTTCAGATCCTCAGTTTTACATTCAACTCAGGACTTGTTCTTACCTTTCTTTTCCAA
hCoV-19/India/HR-MDU-IGIB1210605800489930/2022_EPI...           (21661) TTACCCTGACAAAGTTTTCAGATCCTCAGTTTTACATTCAACTCAGGACTTGTTCTTACCTTTCTTTTCCAA
hCoV-19/England/PLYM-332B917/2022_EPI_ISL_9062229_...           (21611) TTACCCTGACAAAGTTTTCAGATCCTCAGTTTTACATTCAACTCAGGACTTGTTCTTACCTTTCTTTTCCAA
hCoV-19/Germany/HE-RKI-I-438397/2022_EPI_ISL_905522...          (21625) TTACCCTGACAAAGTTTTCAGATCCTCAGTTTTACATTCAACTCAGGACTTGTTCTTACCTTTCTTTTCCAA
hCoV-19/USA/DE-CDC-LC0472738/2021_EPI_ISL_9049423...            (21589) TTACCCTGACAAAGTTTTCAGATCCTCAGTTTTACATTCAACTCAGGACTTGTTCTTACCTTTCTTTTCCAA
                                                                        Section 303
                                                                (21745) 21745 21750     21760      21770      21780      21790      21800         21816
SARS-CoV-2 Reference Genome NC_045512.2                         (21745) TGTTACTTGGTTCCATGCTATACATGTCTCTGGGACCAATGGTACTAAGAGGTTTGATAACCCTGTCCTACC
hCoV-19/Botswana/R69B55_BHP_916539/2021_EPI_ISL_90...           (21691) TGTTACTTGGTTCCATGTTANNNNNNTCTCTGGGACCAATGGTACTAAGAGGTTTGATAACCCTGTCCTACC
hCoV-19/India/HR-MDU-IGIB1210605800489930/2022_EPI...           (21733) TGTTACTTGGTTCCATGTTATACATGTCTCTGGGACCAATGGTACTAAGAGGTTTGATAACCCTGTCCTACC
hCoV-19/England/PLYM-332B917/2022_EPI_ISL_9062229_...           (21683) TGTTACTTGGTTCCATGTTAT------CTCTGGGACCAATGGTACTAAGAGGTTTGATAACCCTGTCCTACC
hCoV-19/Germany/HE-RKI-I-438397/2022_EPI_ISL_905522...          (21697) TGTTACTTGGTTCCATGTTAT------CTCTGGGACCAATGGTACTAAGAGGTTTGATAACCCTGTCCTACC
hCoV-19/USA/DE-CDC-LC0472738/2021_EPI_ISL_9049423...            (21661) TGTTACTTGGTTCCATGTTAT------CTCTGGGACCAATGGTACTAAGAGGTTTGATAACCCTGTCCTACC
                                                                        Section 304
                                                                (21817) 21817        21830      21840      21850      21860      21870            21888
SARS-CoV-2 Reference Genome NC_045512.2                         (21817) ATTTAATGATGGTGTTTATTTTGCTTCCACTGAGAAGTCTAACATAATAAGAGGCTGGATTTTTGGTACTAC
hCoV-19/Botswana/R69B55_BHP_916539/2021_EPI_ISL_90...           (21763) ATTTAATGATGGTGTTTATTTTGCTTCCATTGAGAAGTCTAACATAATAAGAGGCTGGATTTTTGGTACTAC
hCoV-19/India/HR-MDU-IGIB1210605800489930/2022_EPI...           (21805) ATTTAATGATGGTGTTTATTTTGCTTCCATTGAGAAGTCTAACATAATAAGAGGCTGGATTTTTGGTACTAC
hCoV-19/England/PLYM-332B917/2022_EPI_ISL_9062229_...           (21749) ATTTAATGATGGTGTTTATTTTGCTTCCATTGAGAAGTCTAACATAATAAGAGGCTGGATTTTTGGTACTAC
hCoV-19/Germany/HE-RKI-I-438397/2022_EPI_ISL_905522...          (21763) ATTTAATGATGGTGTTTATTTTGCTTCCATTGAGAAGTCTAACATAATAAGAGGCTGGATTTTTGGTACTAC
hCoV-19/USA/DE-CDC-LC0472738/2021_EPI_ISL_9049423...            (21727) ATTTAATGATGGTGTTTATTTTGCTTCCATTGAGAAGTCTAACATAATAAGAGGCTGGATTTTTGGTACTAC
```

Omicron BA.1

```
                                                                                     Section 305
                                                           (21889) 21889 ... 21960
SARS-CoV-2 Reference Genome NC_045512.2 (21889)            TTTAGATTCGAAGACCCAGTCCCTACTTATTGTTAATAACGCTACTAATGTTGTTATTAAAGTCTGTGAATT
hCoV-19/Botswana/R69B55_BHP_916539/2021_EPI_ISL_90... (21835) TTTAGATTCGAAGACCCAGTCCCTACTTATTGTTAATAACGCTACTAATGTTGTTATTAAAGTCTGTGAATT
hCoV-19/India/HR-MDU-IGIB1210605800489930/2022_EPI... (21877) TTTAGATTCGAAGACCCAGTCCCTACTTATTGTTAATAACGCTACTAATGTTGTTATTAAAGTCTGTGAATT
hCoV-19/England/PLYM-332B917/2022_EPI_ISL_9062229 ... (21821) TTTAGATTCGAAGACCCAGTCCCTACTTATTGTTAATAACGCTACTAATGTTGTTATTAAAGTCTGTGAATT
hCoV-19/Germany/HE-RKI-I-438397/2022_EPI_ISL_905522... (21835) TTTAGATTCGAAGACCCAGTCCCTACTTATTGTTAATAACGCTACTAATGTTGTTATTAAAGTCTGTGAATT
hCoV-19/USA/DE-CDC-LC0472738/2021_EPI_ISL_9049423... (21799) TTTAGATTCGAAGACCCAGTCCCTACTTATTGTTAATAACGCTACTAATGTTGTTATTAAAGTCTGTGAATT
                                                                                     Section 306
                                                           (21961) 21961 ... 22032
SARS-CoV-2 Reference Genome NC_045512.2 (21961)            TCAATTTTGTAATGATCCATTTTTGGGTGTTTATTACCACAAAAACAACAAAAGTTGGATGGAAAGTGAGTT
hCoV-19/Botswana/R69B55_BHP_916539/2021_EPI_ISL_90... (21907) TCAATTTTGTAATGATCCATTTTTGGGNNNNNNNNACCACAAAAACAACAAAAGTTGGATGGAAAGTGAGTT
hCoV-19/India/HR-MDU-IGIB1210605800489930/2022_EPI... (21949) TCAATTTTGTAATGATCCATTTTTGGATGTTTATTACCACAAAAACAACAAAAGTTGGATGGAAAGTGAGTT
hCoV-19/England/PLYM-332B917/2022_EPI_ISL_9062229 ... (21893) TCAATTTTGTAATGATCCATTTTTGG---------ACCACAAAAACAACAAAAGTTGGATGGAAAGTGAGTT
hCoV-19/Germany/HE-RKI-I-438397/2022_EPI_ISL_905522... (21907) TCAATTTTGTAATGATCCATTTTTGG---------ACCACAAAAACAACAAAAGTTGGATGGAAAGTGAGTT
hCoV-19/USA/DE-CDC-LC0472738/2021_EPI_ISL_9049423... (21871) TCAATTTTGTAATGATCCATTTTTGG---------ACCACAAAAACAACAAAAGTTGGATGGAAAGTGAGTT
                                                                                     Section 307
                                                           (22033) 22033 ... 22104
SARS-CoV-2 Reference Genome NC_045512.2 (22033)            CAGAGTTTATTCTAGTGCGAATAATTGCACTTTTGAATATGTCTCTCAGCCTTTTCTTATGGACCTTGAAGG
hCoV-19/Botswana/R69B55_BHP_916539/2021_EPI_ISL_90... (21979) CAGAGTTTATTCTAGTGCGAATAATTGCACTTTTGAATATGTCTCTCAGCCTTTTCTTATGGACCTTGAAGG
hCoV-19/India/HR-MDU-IGIB1210605800489930/2022_EPI... (22021) CAGAGTTTATTCTAGTGCGAATAATTGCACTTTTGAATATGTCTCTCAGCCTTTTCTTATGGACCTTGAAGG
hCoV-19/England/PLYM-332B917/2022_EPI_ISL_9062229 ... (21956) CAGAGTTTATTCTAGTGCGAATAATTGCACTTTTGAATATGTCTCTCAGCCTTTTCTTATGGACCTTGAAGG
hCoV-19/Germany/HE-RKI-I-438397/2022_EPI_ISL_905522... (21970) NNNNNNNNNNNNNNNNNNNNNNNNNNNNNNNNNNNNNNNNNNNNNNNNNNNNNNNNNNNNNNNNNNNNNNNN
hCoV-19/USA/DE-CDC-LC0472738/2021_EPI_ISL_9049423... (21934) CAGAGTTTATTCTAGTGCGAATAATTGCACTTTTGAATATGTCTCTCAGCCTTTTCTTATGGACCTTGAAGG
                                                                                     Section 308
                                                           (22105) 22105 ... 22176
SARS-CoV-2 Reference Genome NC_045512.2 (22105)            AAAACAGGGTAATTTCAAAAATCTTAGGGAATTTGTGTTTAAGAATATTGATGGTTATTTTAAAATATATTC
hCoV-19/Botswana/R69B55_BHP_916539/2021_EPI_ISL_90... (22051) AAAACAGGGTAATTTCAAAAATCTTAGGGAATTTGTGTTTAAGAATATTGATGGTTATTTTAAAATATATTC
hCoV-19/India/HR-MDU-IGIB1210605800489930/2022_EPI... (22093) AAAACAGGGTAATTTCAAAAATCTTAGGGAATTTGTGTTTAAGAATATTGATGGTTATTTTAAAATATATTC
hCoV-19/England/PLYM-332B917/2022_EPI_ISL_9062229 ... (22028) AAAACAGGGTAATTTCAAAAATCTTAGGGAATTTGTGTTTAAGAATATTGATGGTTATTTTAAAATATATTC
hCoV-19/Germany/HE-RKI-I-438397/2022_EPI_ISL_905522... (22042) NNNNNNNNNNNNNNNNNNNNNNNNNNNNNNNNNNNNNNNNNNNNNNNNNNNNNNNNNNNNNNNNNNNNNNNN
hCoV-19/USA/DE-CDC-LC0472738/2021_EPI_ISL_9049423... (22006) AAAACAGGGTAATTTCAAAAATCTTAGGGAATTTGTGTTTAAGAATATTGATGGTTATTTTAAAATATATTC
```

Omicron BA.1

Section 309

```
                                                                (22177) 22177        22190      22200      22210      22220      22230        22248
SARS-CoV-2 Reference Genome NC_045512.2                          (22177) TAAGCACACGCCTATTAATTTA-GTGCGT-----GATCTCCCTCAGGGTTTTTCGGCTTTAGAACCATTGGT
hCoV-19/Botswana/R69B55_BHP_916539/2021_EPI_ISL_90...            (22123) TAAGCACACGCCTATTANNNTA-GTGCGT-----GATCTCCCTCAGGGTTTTTCGGCTTTAGAACCATTGGT
hCoV-19/India/HR-MDU-IGIB1210605800489930/2022_EPI...            (22165) TAAGCACACGCCTATTAATTTA-GTGCGT-----GATCTCCCTCAGGGTTTTTCGGCTTTAGAACCATTGGT
hCoV-19/England/PLYM-332B917/2022_EPI_ISL_9062229 ...            (22100) TAAGCACACGCCTATTATAGTGCGTGAGCCAGAAGATCTCCCTCAGGGTTTTTCGGCTTTAGAACCATTGGT
hCoV-19/Germany/HE-RKI-I-438397/2022_EPI_ISL_905522...           (22114) NNNNNNNNNNNNNNNNNNNNNN-NNNNNN-----NNNNNNNNNNNNNNNNNNNNNNNNNNAGAACCATTGGT
hCoV-19/USA/DE-CDC-LC0472738/2021_EPI_ISL_9049423...             (22078) TAAGCACACGCCTATTATAGTGCGTGAGCCAGAAGATCTCCCTCAGGGTTTTTCGGCTTTAGAACCATTGGT
```

Section 310

```
                                                                (22249) 22249      22260      22270      22280      22290      22300      22310  22320
SARS-CoV-2 Reference Genome NC_045512.2                          (22243) AGATTTGCCAATAGGTATTAACATCACTAGGTTTCAAACTTTACTTGCTTTACATAGAAGTTATTTGACTCC
hCoV-19/Botswana/R69B55_BHP_916539/2021_EPI_ISL_90...            (22189) AGATTTGCCAATAGGTATTAACATCACTAGGTTTCAAACTTTACTTGCTTTACATAGAAGTTATTTGACTCC
hCoV-19/India/HR-MDU-IGIB1210605800489930/2022_EPI...            (22231) AGATTTGCCAATAGGTATTAACATCACTAGGTTTCAAACTTTACTTGCTTTACATAGAAGTTATTTGACTCC
hCoV-19/England/PLYM-332B917/2022_EPI_ISL_9062229 ...            (22172) AGATTTGCCAATAGGTATTAACATCACTAGGTTTCAAACTTTACTTGCTTTACATAGAAGTTATTTGACTCC
hCoV-19/Germany/HE-RKI-I-438397/2022_EPI_ISL_905522...           (22180) AGATTTGCNNNNNNNNNNNNNCNNNNNNNGGTTTCAAACTTTACTTGCTTTACATAGAAGTTATTTGACTCC
hCoV-19/USA/DE-CDC-LC0472738/2021_EPI_ISL_9049423...             (22150) AGATTTGCCAATAGGTATTAACATCACTAGGTTTCAAACTTTACTTGCTTTACATAGAAGTTATTTGACTCC
```

Section 311

```
                                                                (22321) 22321    22330      22340      22350      22360      22370      22380    22392
SARS-CoV-2 Reference Genome NC_045512.2                          (22315) TGGTGATTCTTCTTCAGGTTGGACAGCTGGTGCTGCAGCTTATTATGTGGGTTATCTTCAACCTAGGACTTT
hCoV-19/Botswana/R69B55_BHP_916539/2021_EPI_ISL_90...            (22261) TGGTGATTCTTCTTCAGGTTGGACAGCTGGTGCTGCAGCTTATTATGTGGGTTATCTTCAACCTAGGACTTT
hCoV-19/India/HR-MDU-IGIB1210605800489930/2022_EPI...            (22303) TGGTGATTCTTCTTCAGGTTGGACAGCTGGTGCTGCAGCTTATTATGTGGGTTATCTTCAACCTAGGACTTT
hCoV-19/England/PLYM-332B917/2022_EPI_ISL_9062229 ...            (22244) TGGTGATTCTTCTTCAGGTTGGACAGCTGGTGCTGCAGCTTATTATGTGGGTTATCTTCAACCTAGGACTTT
hCoV-19/Germany/HE-RKI-I-438397/2022_EPI_ISL_905522...           (22252) TGGTGATTCTTCTTCAGGTTGGACAGCTGGTGCTGCAGCTTATTATGTGGGTTATCTTCAACCTANNNNTTT
hCoV-19/USA/DE-CDC-LC0472738/2021_EPI_ISL_9049423...             (22222) TGGTGATTCTTCTTCAGGTTGGACAGCTGGTGCTGCAGCTTATTATGTGGGTTATCTTCAACCTAGGACTTT
```

Section 312

```
                                                                (22393) 22393   22400      22410      22420      22430      22440      22450      22464
SARS-CoV-2 Reference Genome NC_045512.2                          (22387) TCTATTAAAATATAATGAAAATGGAACCATTACAGATGCTGTAGACTGTGCACTTGACCCTCTCTCAGAAAC
hCoV-19/Botswana/R69B55_BHP_916539/2021_EPI_ISL_90...            (22333) TCTATTAAAATATAATGAAAATGGAACCATTACAGATGCTGTAGACTGTGCACTTGACCCTCTCTCAGAAAC
hCoV-19/India/HR-MDU-IGIB1210605800489930/2022_EPI...            (22375) TCTATTAAAATATAATGAAAATGGAACCATTACAGATGCTGTAGACTGTGCACTTGACCCTCTCTCAGAAAC
hCoV-19/England/PLYM-332B917/2022_EPI_ISL_9062229 ...            (22316) TCTATTAAAATATAATGAAAATGGAACCATTACAGATGCTGTAGACTGTGCACTTGACCCTCTCTCAGAAAC
hCoV-19/Germany/HE-RKI-I-438397/2022_EPI_ISL_905522...           (22324) TCTATTAAAATATAATGAAAATGGAACCATTACAGATGCTGTAGACTGTGCACTTGACCCTCTCTCAGAAAC
hCoV-19/USA/DE-CDC-LC0472738/2021_EPI_ISL_9049423...             (22294) TCTATTAAAATATAATGAAAATGGAACCATTACAGATGCTGTAGACTGTGCACTTGACCCTCTCTCAGAAAC
```

Section 313

	(22465)	22465 … 22536
SARS-CoV-2 Reference Genome NC_045512.2	(22459)	AAAGTGTACGTTGAAATCCTTCACTGTAGAAAAAGGAATCTATCAAACTTCTAACTTTAGAGTCCAACCAAC
hCoV-19/Botswana/R69B55_BHP_916539/2021_EPI_ISL_90...	(22405)	AAAGTGTACGTTGAAATCCTTCACTGTAGAAAAAGGAATCTATCAAACTTCTAACTTTAGAGTCCAACCAAC
hCoV-19/India/HR-MDU-IGIB1210605800489930/2022_EPI...	(22447)	AAAGTGTACGTTGAAATCCTTCACTGTAGAAAAAGGAATCTATCAAACTTCTAACTTTAGAGTCCAACCAAC
hCoV-19/England/PLYM-332B917/2022_EPI_ISL_9062229_...	(22388)	AAAGTGTACGTTGAAATCCTTCACTGTAGAAAAAGGAATCTATCAAACTTCTAACTTTAGAGTCCAACCAAC
hCoV-19/Germany/HE-RKI-I-438397/2022_EPI_ISL_905522...	(22396)	AAAGNNNNCNNNNNNNNNNNTCACTGTAGAAAAAGGAATCTATCAAACTTCTAACTTTAGAGTCCAACCAAC
hCoV-19/USA/DE-CDC-LC0472738/2021_EPI_ISL_9049423...	(22366)	AAAGTGTACGTTGAAATCCTTCACTGTAGAAAAAGGAATCTATCAAACTTCTAACTTTAGAGTCCAACCAAC

Section 314

	(22537)	22537 … 22608
SARS-CoV-2 Reference Genome NC_045512.2	(22531)	AGAATCTATTGTTAGATTTCCTAATATTACAAACTTGTGCCCTTTTGGTGAAGTTTTTAACGCCACCAGATT
hCoV-19/Botswana/R69B55_BHP_916539/2021_EPI_ISL_90...	(22477)	AGAATCTATTGTTAGATTTCCTAATATTACAAACTTGTGCCCTTTTGATGAAGTTTTTAACGCCACCAGATT
hCoV-19/India/HR-MDU-IGIB1210605800489930/2022_EPI...	(22519)	AGAATCTATTGTTAGATTTCCTAATATTACAAACTTGTGCCCTTTTGATGAAGTTTTTAACGCCACCAGATT
hCoV-19/England/PLYM-332B917/2022_EPI_ISL_9062229_...	(22460)	AGAATCTATTGTTAGATTTCCTAATATTACAAACTTGTGCCCTTTTGATGAAGTTTTTAACGCCACCAGATT
hCoV-19/Germany/HE-RKI-I-438397/2022_EPI_ISL_905522...	(22468)	AGANNNNNNNNNNAGANTTCCTAATNNNNNNNNNNNNNNNNNNNNNNNNNNNGTTTTTAACGCCACCAGATT
hCoV-19/USA/DE-CDC-LC0472738/2021_EPI_ISL_9049423...	(22438)	AGAATCTATTGTTAGATTTCCTAATATTACAAACTTGTGCCCTTTTGATGAAGTTTTTAACGCCACCAGATT

Section 315

	(22609)	22609 … 22680
SARS-CoV-2 Reference Genome NC_045512.2	(22603)	TGCATCTGTTTATGCTTGGAACAGGAAGAGAATCAGCAACTGTGTTGCTGATTATTCTGTCCTATATAATTC
hCoV-19/Botswana/R69B55_BHP_916539/2021_EPI_ISL_90...	(22549)	TGCATCTGTTTATGCTTGGAACAGGAAGAGAATCAGCAACTGTGTTGCTGATTATTCTGTCCTATATAATCT
hCoV-19/India/HR-MDU-IGIB1210605800489930/2022_EPI...	(22591)	TGCATCTGTTTATGCTTGGAACAGGAAGAGAATCAGCAACTGTGTTGCTGATTATTCTGTCCTATATAATCT
hCoV-19/England/PLYM-332B917/2022_EPI_ISL_9062229_...	(22532)	TGCATCTGTTTATGCTTGGAACAGGAAGAGAATCAGCAACTGTGTTGCTGATTATTCTGTCCTATATAATCT
hCoV-19/Germany/HE-RKI-I-438397/2022_EPI_ISL_905522...	(22540)	TGCATCTGTTTATGCTTGGAACAGGAAGAGNNNNNNNNNNNNNNNNNNNNNNNNNNNNNNNNNNNNNNNNNN
hCoV-19/USA/DE-CDC-LC0472738/2021_EPI_ISL_9049423...	(22510)	TGCATCTGTTTATGCTTGGAACAGGAAGAGAATCAGCAACTGTGTTGCTGATTATTCTGTCCTATATAATCT

Section 316

	(22681)	22681 … 22752
SARS-CoV-2 Reference Genome NC_045512.2	(22675)	CGCATCATTTTCCACTTTTAAGTGTTATGGAGTGTCTCCTACTAAATTAAATGATCTCTGCTTTACTAATGT
hCoV-19/Botswana/R69B55_BHP_916539/2021_EPI_ISL_90...	(22621)	CGCACCATTTTTCACTTTTAAGTGTTATGGAGTGTCTCCTACTAAATTAAATGATCTCTGCTTTACTAATGT
hCoV-19/India/HR-MDU-IGIB1210605800489930/2022_EPI...	(22663)	CGCACCATTTTTCACTTTTAAGTGTTATGGAGTGTCTCCTACTAAATTAAATGATCTCTGCTTTACTAATGT
hCoV-19/England/PLYM-332B917/2022_EPI_ISL_9062229_...	(22604)	CGCACCATTTTTCACTTTTAAGTGTTATGGAGTGTCTCCTACTAAATTAAATGATCTCTGCTTTACTAATGT
hCoV-19/Germany/HE-RKI-I-438397/2022_EPI_ISL_905522...	(22612)	NNNNNNNNNNNNNNNNNNNNNNNNNNNNNNNNNNNNNNNNNNNNNNNNNNNNNNNNNNNNNNNNNNNNNNNN
hCoV-19/USA/DE-CDC-LC0472738/2021_EPI_ISL_9049423...	(22582)	CGCACCATTTTTCACTTTTAAGTGTTATGGAGTGTCTCCTACTAAATTAAATGATCTCTGCTTTACTAATGT

Section 317

(22753) 22753 22760 22770 22780 22790 22800 22810 22824
SARS-CoV-2 Reference Genome NC_045512.2 (22747) CTATGCAGATTCATTTGTAATTAGAGGTGATGAAGTCAGACAAATCGCTCCAGGGCAAACTGGAAAGATTGC
hCoV-19/Botswana/R69B55_BHP_916539/2021_EPI_ISL_90... (22693) CTATGCAGATTCATTTGTAATTAGAGGTGATGAAGTCAGACAAATCGCTCCAGGGCAAACTGGAAATATTGC
hCoV-19/India/HR-MDU-IGIB1210605800489930/2022_EPI... (22735) CTATGCAGATTCATTTGTAATTAGAGGTGATGAAGTCAGACAAATCGCTCCAGGGCAAACTGGAAATATTGC
hCoV-19/England/PLYM-332B917/2022_EPI_ISL_9062229_... (22676) CTATGCAGATTCATTTGTAATTAGAGGTGATGAAGTCAGACAAATCGCTCCAGGGCAAACTGGAAATATTGC
hCoV-19/Germany/HE-RKI-I-438397/2022_EPI_ISL_905522... (22684) NNNNNNNNNNNNNNNNNNNNNNNNNNNNNNNNNNNNNNNNNNNNNNNNNNNNNNNNNNNNNNNNNNNNNNNN
hCoV-19/USA/DE-CDC-LC0472738/2021_EPI_ISL_9049423... (22654) CTATGCAGATTCATTTGTAATTAGAGGTGATGAAGTCAGACAAATCGCTCCAGGGCAAACTGGAAATATTGC

Section 318

(22825) 22825 22830 22840 22850 22860 22870 22880 22896
SARS-CoV-2 Reference Genome NC_045512.2 (22819) TGATTATAATTATAAATTACCAGATGATTTTACAGGCTGCGTTATAGCTTGGAATTCTAACAATCTTGATTC
hCoV-19/Botswana/R69B55_BHP_916539/2021_EPI_ISL_90... (22765) TGATTATAATTATAAATTACCAGATGATTTTACAGGCTGCGTTATAGCTTGGAATTCTAACAAGCTTGATTC
hCoV-19/India/HR-MDU-IGIB1210605800489930/2022_EPI... (22807) TGATTATAATTATAAATTACCAGATGATTTTACAGGCTGCGTTATAGCTTGGAATTCTAACAAGCTTGATTC
hCoV-19/England/PLYM-332B917/2022_EPI_ISL_9062229_... (22748) TGATTATAATTATAAATTACCAGATGATTTTACAGGCTGCGTTATAGCTTGGAATTCTAACAAGCTTGATTC
hCoV-19/Germany/HE-RKI-I-438397/2022_EPI_ISL_905522... (22756) NNNNNNNNNNNNNNNNNNNNNNNNNNNNNNNNNNNNNNNNNNNNNNNNNNNNNNNNNNNNNNNNNNNNNNNN
hCoV-19/USA/DE-CDC-LC0472738/2021_EPI_ISL_9049423... (22726) TGATTATAATTATAAATTACCAGATGATTTTACAGGCTGCGTTATAGCTTGGAATTCTAACAAGCTTGATTC

Section 319

(22897) 22897 22910 22920 22930 22940 22950 22968
SARS-CoV-2 Reference Genome NC_045512.2 (22891) TAAGGTTGGTGGTAATTATAATTACCTGTATAGATTGTTTAGGAAGTCTAATCTCAAACCTTTTGAGAGAGA
hCoV-19/Botswana/R69B55_BHP_916539/2021_EPI_ISL_90... (22837) TAAGGTTAGTGGTAATTATAATTACCTGTATAGATTGTTTAGGAAGTCTAATCTCAAACCTTTTGAGAGAGA
hCoV-19/India/HR-MDU-IGIB1210605800489930/2022_EPI... (22879) TAAGGTTAGTGGTAATTATAATTACCTGTATAGATTGTTTAGGAAGTCTAATCTCAAACCTTTTGAGAGAGA
hCoV-19/England/PLYM-332B917/2022_EPI_ISL_9062229_... (22820) TAAGGTTAGTGGTAATTATAATTACCTGTATAGATTGTTTAGGAAGTCTAATCTCAAACCTTTTGAGAGAGA
hCoV-19/Germany/HE-RKI-I-438397/2022_EPI_ISL_905522... (22828) NNNNNNNNNNNNNNNNNNNNNNNNNNNNNNNNNNNNNNNNNNNNNNNNNNNNNNNNNNNNNNNNNNNNNNNN
hCoV-19/USA/DE-CDC-LC0472738/2021_EPI_ISL_9049423... (22798) TAAGGTTAGTGGTAATTATAATTACCTGTATAGATTGTTTAGGAAGTCTAATCTCAAACCTTTTGAGAGAGA

Section 320

(22969) 22969 22980 22990 23000 23010 23020 23030 23040
SARS-CoV-2 Reference Genome NC_045512.2 (22963) TATTTCAACTGAAATCTATCAGGCCGGTAGCACACCTTGTAATGGTGTTGAAGGTTTTAATTGTTACTTTCC
hCoV-19/Botswana/R69B55_BHP_916539/2021_EPI_ISL_90... (22909) TATTTCAACTGAAATCTATCAGGCCGGTAACAAACCTTGTAATGGTGTTGCAGGTTTTAATTGTTACTTTCC
hCoV-19/India/HR-MDU-IGIB1210605800489930/2022_EPI... (22951) TATTTCAACTGAAATCTATCAGGCCGGTAACAAACCTTGTAATGGTGTTGCAGGTTTTAATTGTTACTTTCC
hCoV-19/England/PLYM-332B917/2022_EPI_ISL_9062229_... (22892) TATTTCAACTGAAATCTATCAGGCCGGTAACAAACCTTGTAATGGTGTTGCAGGTTTTAATTGTTACTTTCC
hCoV-19/Germany/HE-RKI-I-438397/2022_EPI_ISL_905522... (22900) NNNNNNNNNNNNNNNNNNNNNNNNNNNNNNNNNNNNNNNNNNNNNNNNNNNNNNNNNNNNNNNNNNNNNNNN
hCoV-19/USA/DE-CDC-LC0472738/2021_EPI_ISL_9049423... (22870) TATTTCAACTGAAATCTATCAGGCCGGTAACAAACCTTGTAATGGTGTTGCAGGTTTTAATTGTTACTTTCC

Section 321

```
                                                          (23041) 23041    23050      23060      23070      23080      23090      23100     23112
SARS-CoV-2 Reference Genome NC_045512.2                   (23035) TTTACAATCATATGGTTTCCAACCCACTAATGGTGTTGGTTACCAACCATACAGAGTAGTAGTACTTTCTTT
hCoV-19/Botswana/R69B55_BHP_916539/2021_EPI_ISL_90...     (22981) TTTACGATCATATAGTTTCCGACCCACTTATGGTGTTGGTCACCAACCATACAGAGTAGTAGTACTTTCTTT
hCoV-19/India/HR-MDU-IGIB1210605800489930/2022_EPI...     (23023) TTTACGATCATATAGTTTCCGACCCACTTATGGTGTTGGTCACCAACCATACAGAGTAGTAGTACTTTCTTT
hCoV-19/England/PLYM-332B917/2022_EPI_ISL_9062229_...     (22964) TTTACGATCATATAGTTTCCGACCCACTTATGGTGTTGGTCACCAACCATACAGAGTAGTAGTACTTTCTTT
hCoV-19/Germany/HE-RKI-I-438397/2022_EPI_ISL_905522...    (22972) NNNNNNNNNNNNNNNNNNNNNNNNNNNNNNNNNNNNNNNNNNNNNNNNNNNNNNNNNNNNNNNNNNNNNNNN
hCoV-19/USA/DE-CDC-LC0472738/2021_EPI_ISL_9049423...      (22942) TTTACGATCATATAGTTTCCGACCCACTTATGGTGTTGGTCACCAACCATACAGAGTAGTAGTACTTTCTTT
```

Section 322

```
                                                          (23113) 23113  23120      23130      23140      23150      23160      23170     23184
SARS-CoV-2 Reference Genome NC_045512.2                   (23107) TGAACTTCTACATGCACCAGCAACTGTTTGTGGACCTAAAAAGTCTACTAATTTGGTTAAAAACAAATGTGT
hCoV-19/Botswana/R69B55_BHP_916539/2021_EPI_ISL_90...     (23053) TGAACTTCTACATGCACCAGCAACTGTTTGTGGACCTAAAAAGTCTACTAATTTGGTTAAAAACAAATGTGT
hCoV-19/India/HR-MDU-IGIB1210605800489930/2022_EPI...     (23095) TGAACTTCTACATGCACCAGCAACTGTTTGTGGACCTAAAAAGTCTACTAATTTGGTTAAAAACAAATGTGT
hCoV-19/England/PLYM-332B917/2022_EPI_ISL_9062229_...     (23036) TGAACTTCTACATGCACCAGCAACTGTTTGTGGACCTAAAAAGTCTACTAATTTGGTTAAAAACAAATGTGT
hCoV-19/Germany/HE-RKI-I-438397/2022_EPI_ISL_905522...    (23044) NNNNNNNNNNNNNNNNNNNNNAACTGTTTGTGGACCTAAAAAGTCTACTAATTTGGTTAAAAACAAATGTGT
hCoV-19/USA/DE-CDC-LC0472738/2021_EPI_ISL_9049423...      (23014) TGAACTTCTACATGCACCAGCAACTGTTTGTGGACCTAAAAAGTCTACTAATTTGGTTAAAAACAAATGTGT
```

Section 323

```
                                                          (23185) 23185 23190      23200      23210      23220      23230      23240     23256
SARS-CoV-2 Reference Genome NC_045512.2                   (23179) CAATTTCAACTTCAATGGTTTAACAGGCACAGGTGTTCTTACTGAGTCTAACAAAAAGTTTCTGCCTTTCCA
hCoV-19/Botswana/R69B55_BHP_916539/2021_EPI_ISL_90...     (23125) CAATTTCAACTTCAATGGTTTAAAAGGCACAGGTGTTCTTACTGAGTCTAACAAAAAGTTTCTGCCTTTCCA
hCoV-19/India/HR-MDU-IGIB1210605800489930/2022_EPI...     (23167) CAATTTCAACTTCAATGGTTTAAAAGGCACAGGTGTTCTTACTGAGTCTAACAAAAAGTTTCTGCCTTTCCA
hCoV-19/England/PLYM-332B917/2022_EPI_ISL_9062229_...     (23108) CAATTTCAACTTCAATGGTTTAAAAGGCACAGGTGTTCTTACTGAGTCTAACAAAAAGTTTCTGCCTTTCCA
hCoV-19/Germany/HE-RKI-I-438397/2022_EPI_ISL_905522...    (23116) CAATTTCAACTTCAATGGTTTAAAAGGCACAGGTGTTCTTACTGAGTCTAACAAAAAGTTTCTGCCTTTCCA
hCoV-19/USA/DE-CDC-LC0472738/2021_EPI_ISL_9049423...      (23086) CAATTTCAACTTCAATGGTTTAAAAGGCACAGGTGTTCTTACTGAGTCTAACAAAAAGTTTCTGCCTTTCCA
```

Section 324

```
                                                          (23257) 23257         23270      23280      23290      23300      23310         23328
SARS-CoV-2 Reference Genome NC_045512.2                   (23251) ACAATTTGGCAGAGACATTGCTGACACTACTGATGCTGTCCGTGATCCACAGACACTTGAGATTCTTGACAT
hCoV-19/Botswana/R69B55_BHP_916539/2021_EPI_ISL_90...     (23197) ACAATTTGGCAGAGACATTGCTGACACTACTGATGCTGTCCGTGATCCACAGACACTTGAGATTCTTGACAT
hCoV-19/India/HR-MDU-IGIB1210605800489930/2022_EPI...     (23239) ACAATTTGGCAGAGACATTGCTGACACTACTGATGCTGTCCGTGATCCACAGACACTTGAGATTCTTGACAT
hCoV-19/England/PLYM-332B917/2022_EPI_ISL_9062229_...     (23180) ACAATTTGGCAGAGACATTGCTGACACTACTGATGCTGTCCGTGATCCACAGACACTTGAGATTCTTGACAT
hCoV-19/Germany/HE-RKI-I-438397/2022_EPI_ISL_905522...    (23188) ACAATTTGGCAGAGACATTGCTGACACTACTGATGCTGTCCGTGATCCACAGACACTTGAGATTCTTGACAT
hCoV-19/USA/DE-CDC-LC0472738/2021_EPI_ISL_9049423...      (23158) ACAATTTGGCAGAGACATTGCTGACACTACTGATGCTGTCCGTGATCCACAGACACTTGAGATTCTTGACAT
```

Section 325

```
                                                         (23329) 23329      23340       23350       23360       23370       23380       23390   23400
SARS-CoV-2 Reference Genome NC_045512.2                  (23323) TACACCATGTTCTTTTGGTGGTGTCAGTGTTATAACACCAGGAACAAATACTTCTAACCAGGTTGCTGTTCT
hCoV-19/Botswana/R69B55_BHP_916539/2021_EPI_ISL_90...    (23269) TACACCATGTTCTTTTGGTGGTGTCAGTGTTATAACACCAGGAACAAATACTTCTAACCAGGTTGCTGTTCT
hCoV-19/India/HR-MDU-IGIB1210605800489930/2022_EPI...    (23311) TACACCATGTTCTTTTGGTGGTGTCAGTGTTATAACACCAGGAACAAATACTTCTAACCAGGTTGCTGTTCT
hCoV-19/England/PLYM-332B917/2022_EPI_ISL_9062229 ...    (23252) TACACCATGTTCTTTTGGTGGTGTCAGTGTTATAACACCAGGAACAAATACTTCTAACCAGGTTGCTGTTCT
hCoV-19/Germany/HE-RKI-I-438397/2022_EPI_ISL_905522...   (23260) TACACCATGTTCTTTTGGTGGTGTCAGTGTTATAACACCAGGAACAAATACTTCTAACCAGGTTGCTGTTCT
hCoV-19/USA/DE-CDC-LC0472738/2021_EPI_ISL_9049423...     (23230) TACACCATGTTCTTTTGGTGGTGTCAGTGTTATAACACCAGGAACAAATACTTCTAACCAGGTTGCTGTTCT
```

Section 326

```
                                                         (23401) 23401     23410       23420       23430       23440       23450       23460     23472
SARS-CoV-2 Reference Genome NC_045512.2                  (23395) TTATCAGGATGTTAACTGCACAGAAGTCCCTGTTGCTATTCATGCAGATCAACTTACTCCTACTTGGCGTGT
hCoV-19/Botswana/R69B55_BHP_916539/2021_EPI_ISL_90...    (23341) TTATCAGGGTGTTAACTGCACAGAAGTCCCTGTTGCTATTCATGCAGATCAACTTACTCCTACTTGGCGTGT
hCoV-19/India/HR-MDU-IGIB1210605800489930/2022_EPI...    (23383) TTATCAGGGTGTTAACTGCACAGAAGTCCCTGTTGCTATTCATGCAGATCAACTTACTCCTACTTGGCGTGT
hCoV-19/England/PLYM-332B917/2022_EPI_ISL_9062229 ...    (23324) TTATCAGGGTGTTAACTGCACAGAAGTCCCTGTTGCTATTCATGCAGATCAACTTACTCCTACTTGGCGTGT
hCoV-19/Germany/HE-RKI-I-438397/2022_EPI_ISL_905522...   (23332) TTATCAGGGTGTTAACTGCACAGAAGTCCCTGTTGCTATTCATGCAGATCAACTTACTCCTACTTGGCGTGT
hCoV-19/USA/DE-CDC-LC0472738/2021_EPI_ISL_9049423...     (23302) TTATCAGGGTGTTAACTGCACAGAAGTCCCTGTTGCTATTCATGCAGATCAACTTACTCCTACTTGGCGTGT
```

Section 327

```
                                                         (23473) 23473  23480       23490       23500       23510       23520       23530     23544
SARS-CoV-2 Reference Genome NC_045512.2                  (23467) TTATTCTACAGGTTCTAATGTTTTTCAAACACGTGCAGGCTGTTTAATAGGGGCTGAACATGTCAACAACTC
hCoV-19/Botswana/R69B55_BHP_916539/2021_EPI_ISL_90...    (23413) TTATTCTACAGGTTCTAATGTTTTTCAAACACGTGCAGGCTGTTTAATAGGGGCTGAATATGTCAACAACTC
hCoV-19/India/HR-MDU-IGIB1210605800489930/2022_EPI...    (23455) TTATTCTACAGGTTCTAATGTTTTTCAAACACGTGCAGGCTGTTTAATAGGGGCTGAATATGTCAACAACTC
hCoV-19/England/PLYM-332B917/2022_EPI_ISL_9062229 ...    (23396) TTATTCTACAGGTTCTAATGTTTTTCAAACACGTGCAGGCTGTTTAATAGGGGCTGAATATGTCAACAACTC
hCoV-19/Germany/HE-RKI-I-438397/2022_EPI_ISL_905522...   (23404) TTATTCTACAGGTTCTAATGTTTTTCAAACACGTGCAGGCTGTTTAATAGGGGCTGAATATGTCAACAACTC
hCoV-19/USA/DE-CDC-LC0472738/2021_EPI_ISL_9049423...     (23374) TTATTCTACAGGTTCTAATGTTTTTCAAACACGTGCAGGCTGTTTAATAGGGGCTGAATATGTCAACAACTC
```

Section 328

```
                                                         (23545) 23545 23550       23560       23570       23580       23590       23600         23616
SARS-CoV-2 Reference Genome NC_045512.2                  (23539) ATATGAGTGTGACATACCCATTGGTGCAGGTATATGCGCTAGTTATCAGACTCAGACTAATTCTCCTCGGCG
hCoV-19/Botswana/R69B55_BHP_916539/2021_EPI_ISL_90...    (23485) ATATGAGTGTGACATACCCATTGGTGCAGGTATATGCGCTAGTTATCAGACTCAGACTAAGTCTCATCGGCG
hCoV-19/India/HR-MDU-IGIB1210605800489930/2022_EPI...    (23527) ATATGAGTGTGACATACCCATTGGTGCAGGTATATGCGCTAGTTATCAGACTCAGACTAAGTCTCATCGGCG
hCoV-19/England/PLYM-332B917/2022_EPI_ISL_9062229 ...    (23468) ATATGAGTGTGACATACCCATTGGTGCAGGTATATGCGCTAGTTATCAGACTCAGACTAAGTCTCATCGGCG
hCoV-19/Germany/HE-RKI-I-438397/2022_EPI_ISL_905522...   (23476) ATATGAGTGTGACATACCCATTGGTGCAGGTATATGCGCTAGTTATCAGACTCAGACTAAGTCTCATCGGCG
hCoV-19/USA/DE-CDC-LC0472738/2021_EPI_ISL_9049423...     (23446) ATATGAGTGTGACATACCCATTGGTGCAGGTATATGCGCTAGTTATCAGACTCAGACTAAGTCTCATCGGCG
```

Omicron BA.1

```
                                                                    Section 329
                                                    (23617) 23617         23630         23640         23650         23660         23670         23688
             SARS-CoV-2 Reference Genome NC_045512.2 (23611) GGCACGTAGTGTAGCTAGTCAATCCATCATTGCCTACACTATGTCACTTGGTGCAGAAAATTCAGTTGCTTA
hCoV-19/Botswana/R69B55_BHP_916539/2021_EPI_ISL_90... (23557) GGCACGTAGTGTAGCTAGTCAATCCATCATTGCCTACACTATGTCACTTGGTGCAGAAAATTCAGTTGCTTA
 hCoV-19/India/HR-MDU-IGIB1210605800489930/2022_EPI... (23599) GGCACGTAGTGTAGCTAGTCAATCCATCATTGCCTACACTATGTCACTTGGTGCAGAAAATTCAGTTGCTTA
 hCoV-19/England/PLYM-332B917/2022_EPI_ISL_9062229_... (23540) GGCACGTAGTGTAGCTAGTCAATCCATCATTGCCTACACTATGTCACTTGGTGCAGAAAATTCAGTTGCTTA
hCoV-19/Germany/HE-RKI-I-438397/2022_EPI_ISL_905522... (23548) GGCACGTAGTGTAGCTAGTCAATCCATCATTGCCTACACTATGTCACTTGGTGCAGAAAATTCAGTTGCTTA
 hCoV-19/USA/DE-CDC-LC0472738/2021_EPI_ISL_9049423... (23518) GGCACGTAGTGTAGCTAGTCAATCCATCATTGCCTACACTATGTCACTTGGTGCAGAAAATTCAGTTGCTTA
                                                                    Section 330
                                                    (23689) 23689       23700         23710         23720         23730         23740         23750   23760
             SARS-CoV-2 Reference Genome NC_045512.2 (23683) CTCTAATAACTCTATTGCCATACCCACAAATTTTACTATTAGTGTTACCACAGAAATTCTACCAGTGTCTAT
hCoV-19/Botswana/R69B55_BHP_916539/2021_EPI_ISL_90... (23629) CTCTAATAACTCTATTGCCATACCCACAAATTTTACTATTAGTGTTACCACAGAAATTCTACCAGTGTCTAT
 hCoV-19/India/HR-MDU-IGIB1210605800489930/2022_EPI... (23671) CTCTAATAACTCTATTGCCATACCCACAAATTTTACTATTAGTGTTACCACAGAAATTCTACCAGTGTCTAT
 hCoV-19/England/PLYM-332B917/2022_EPI_ISL_9062229_... (23612) CTCTAATAACTCTATTGCCATACCCACAAATTTTACTATTAGTGTTACCACAGAAATTCTACCAGTGTCTAT
hCoV-19/Germany/HE-RKI-I-438397/2022_EPI_ISL_905522... (23620) CTCTAATAACTCTATTGCCATACCCACAAATTTTACTATTAGTGTTACCACAGAAATTCTACCAGTGTCTAT
 hCoV-19/USA/DE-CDC-LC0472738/2021_EPI_ISL_9049423... (23590) CTCTAATAACTCTATTGCCATACCCACAAATTTTACTATTAGTGTTACCACAGAAATTCTACCAGTGTCTAT
                                                                    Section 331
                                                    (23761) 23761     23770         23780         23790         23800         23810         23820     23832
             SARS-CoV-2 Reference Genome NC_045512.2 (23755) GACCAAGACATCAGTAGATTGTACAATGTACATTTGTGGTGATTCAACTGAATGCAGCAATCTTTTGTTGCA
hCoV-19/Botswana/R69B55_BHP_916539/2021_EPI_ISL_90... (23701) GACCAAGACATCAGTAGATTGTACAATGTACATTTGTGGTGATTCAACTGAATGCAGCAATCTTTTGTTGCA
 hCoV-19/India/HR-MDU-IGIB1210605800489930/2022_EPI... (23743) GACCAAGACATCAGTAGATTGTACAATGTACATTTGTGGTGATTCAACTGAATGCAGCAATCTTTTGTTGCA
 hCoV-19/England/PLYM-332B917/2022_EPI_ISL_9062229_... (23684) GACCAAGACATCAGTAGATTGTACAATGTACATTTGTGGTGATTCAACTGAATGCAGCAATCTTTTGTTGCA
hCoV-19/Germany/HE-RKI-I-438397/2022_EPI_ISL_905522... (23692) GACCAAGACATCAGTAGATTGTACAATGTACATTTGTGGTGATTCAACTGAATGCAGCAATCTTTTGTTGCA
 hCoV-19/USA/DE-CDC-LC0472738/2021_EPI_ISL_9049423... (23662) GACCAAGACATCAGTAGATTGTACAATGTACATTTGTGGTGATTCAACTGAATGCAGCAATCTTTTGTTGCA
                                                                    Section 332
                                                    (23833) 23833   23840         23850         23860         23870         23880         23890     23904
             SARS-CoV-2 Reference Genome NC_045512.2 (23827) ATATGGCAGTTTTTGTACACAATTAAACCGTGCTTTAACTGGAATAGCTGTTGAACAAGACAAAAACACCCA
hCoV-19/Botswana/R69B55_BHP_916539/2021_EPI_ISL_90... (23773) ATATGGCAGTTTTTGTACACAATTAAAACGTGCTTTAACTGGAATAGCTGTTGAACAAGACAAAAACACCCA
 hCoV-19/India/HR-MDU-IGIB1210605800489930/2022_EPI... (23815) ATATGGCAGTTTTTGTACACAATTAAAACGTGCTTTAACTGGAATAGCTGTTGAACAAGACAAAAACACCCA
 hCoV-19/England/PLYM-332B917/2022_EPI_ISL_9062229_... (23756) ATATGGCAGTTTTTGTACACAATTAAAACGTGCTTTAACTGGAATAGCTGTTGAACAAGACAAAAACACCCA
hCoV-19/Germany/HE-RKI-I-438397/2022_EPI_ISL_905522... (23764) ATATGGCAGTTTTTGTACACAATTAAAACGTGCTTTAACTGGAATAGCTGTTGAACAAGACAAAAACACCCA
 hCoV-19/USA/DE-CDC-LC0472738/2021_EPI_ISL_9049423... (23734) ATATGGCAGTTTTTGTACACAATTAAAACGTGCTTTAACTGGAATAGCTGTTGAACAAGACAAAAACACCCA
```

Section 333

(23905)	23905 23910 23920 23930 23940 23950 23960 23976
SARS-CoV-2 Reference Genome NC_045512.2 (23899)	AGAAGTTTTTGCACAAGTCAAACAAATTTACAAAACACCACCAATTAAAGATTTTGGTGGTTTTAATTTTTC
hCoV-19/Botswana/R69B55_BHP_916539/2021_EPI_ISL_90... (23845)	AGAAGTTTTTGCACAAGTCAAACAAATTTACAAAACACCACCAATTAAATATTTTGGTGGTTTTAATTTTTC
hCoV-19/India/HR-MDU-IGIB1210605800489930/2022_EPI... (23887)	AGAAGTTTTTGCACAAGTCAAACAAATTTACAAAACACCACCAATTAAATATTTTGGTGGTTTTAATTTTTC
hCoV-19/England/PLYM-332B917/2022_EPI_ISL_9062229_... (23828)	AGAAGTTTTTGCACAAGTCAAACAAATTTACAAAACACCACCAATTAAATATTTTGGTGGTTTTAATTTTTC
hCoV-19/Germany/HE-RKI-I-438397/2022_EPI_ISL_905522... (23836)	AGAAGTTTTTGCACAAGTCAAACAAATTTACAAAACACCACCAATTAAATATTTTGGTGGTTTTAATTTTTC
hCoV-19/USA/DE-CDC-LC0472738/2021_EPI_ISL_9049423... (23806)	AGAAGTTTTTGCACAAGTCAAACAAATTTACAAAACACCACCAATTAAATATTTTGGTGGTTTTAATTTTTC

Section 334

(23977)	23977 23990 24000 24010 24020 24030 24048
SARS-CoV-2 Reference Genome NC_045512.2 (23971)	ACAAATATTACCAGATCCATCAAAACCAAGCAAGAGGTCATTTATTGAAGATCTACTTTTCAACAAAGTGAC
hCoV-19/Botswana/R69B55_BHP_916539/2021_EPI_ISL_90... (23917)	ACAAATATTACCAGATCCATCAAAACCAAGCAAGAGGTCATTTATTGAAGATCTACTTTTCAACAAAGTGAC
hCoV-19/India/HR-MDU-IGIB1210605800489930/2022_EPI... (23959)	ACAAATATTACCAGATCCATCAAAACCAAGCAAGAGGTCATTTATTGAAGATCTACTTTTCAACAAAGTGAC
hCoV-19/England/PLYM-332B917/2022_EPI_ISL_9062229_... (23900)	ACAAATATTACCAGATCCATCAAAACCAAGCAAGAGGTCATTTATTGAAGATCTACTTTTCAACAAAGTGAC
hCoV-19/Germany/HE-RKI-I-438397/2022_EPI_ISL_905522... (23908)	ACAAATATTACCAGATCCATCAAAACCAAGCAAGAGGTCATTTATTGAAGATCTACTTTTCAACAAAGTGAC
hCoV-19/USA/DE-CDC-LC0472738/2021_EPI_ISL_9049423... (23878)	ACAAATATTACCAGATCCATCAAAACCAAGCAAGAGGTCATTTATTGAAGATCTACTTTTCAACAAAGTGAC

Section 335

(24049)	24049 24060 24070 24080 24090 24100 24110 24120
SARS-CoV-2 Reference Genome NC_045512.2 (24043)	ACTTGCAGATGCTGGCTTCATCAAACAATATGGTGATTGCCTTGGTGATATTGCTGCTAGAGACCTCATTTG
hCoV-19/Botswana/R69B55_BHP_916539/2021_EPI_ISL_90... (23989)	ACTTGCAGATGCTGGCTTCATCAAACAATATGGTGATTGCCTTGGTGATATTGCTGCTAGAGACCTCATTTG
hCoV-19/India/HR-MDU-IGIB1210605800489930/2022_EPI... (24031)	ACTTGCAGATGCTGGCTTCATCAAACAATATGGTGATTGCCTTGGTGATATTGCTGCTAGAGACCTCATTTG
hCoV-19/England/PLYM-332B917/2022_EPI_ISL_9062229_... (23972)	ACTTGCAGATGCTGGCTTCATCAAACAATATGGTGATTGCCTTGGTGATATTGCTGCTAGAGACCTCATTTG
hCoV-19/Germany/HE-RKI-I-438397/2022_EPI_ISL_905522... (23980)	ACTTGCAGATGCTGGCTTCATCAAACAATATGGTGATTGCCTTGGTGATATTGCTGCTAGAGACCTCATTTG
hCoV-19/USA/DE-CDC-LC0472738/2021_EPI_ISL_9049423... (23950)	ACTTGCAGATGCTGGCTTCATCAAACAATATGGTGATTGCCTTGGTGATATTGCTGCTAGAGACCTCATTTG

Section 336

(24121)	24121 24130 24140 24150 24160 24170 24180 24192
SARS-CoV-2 Reference Genome NC_045512.2 (24115)	TGCACAAAAGTTTAACGGCCTTACTGTTTTGCCACCTTTGCTCACAGATGAAATGATTGCTCAATACACTTC
hCoV-19/Botswana/R69B55_BHP_916539/2021_EPI_ISL_90... (24061)	TGCACAAAAGTTTAAAGGCCTTACTGTTTTGCCACCTTTGCTCACAGATGAAATGATTGCTCAATACACTTC
hCoV-19/India/HR-MDU-IGIB1210605800489930/2022_EPI... (24103)	TGCACAAAAGTTTAAAGGCCTTACTGTTTTGCCACCTTTGCTCACAGATGAAATGATTGCTCAATACACTTC
hCoV-19/England/PLYM-332B917/2022_EPI_ISL_9062229_... (24044)	TGCACAAAAGTTTAAAGGCCTTACTGTTTTGCCACCTTTGCTCACAGATGAAATGATTGCTCAATACACTTC
hCoV-19/Germany/HE-RKI-I-438397/2022_EPI_ISL_905522... (24052)	TGCACAAAAGTTTAAAGGCCTTACTGTTTTGCCACCTTTGCTCACAGATGAAATGATTGCTCAATACACTTC
hCoV-19/USA/DE-CDC-LC0472738/2021_EPI_ISL_9049423... (24022)	TGCACAAAAGTTTAAAGGCCTTACTGTTTTGCCACCTTTGCTCACAGATGAAATGATTGCTCAATACACTTC

Section 337

	(24193)	24193 24200 24210 24220 24230 24240 24250 24264
SARS-CoV-2 Reference Genome NC_045512.2	(24187)	TGCACTGTTAGCGGGTACAATCACTTCTGGTTGGACCTTTGGTGCAGGTGCTGCATTACAAATACCATTTGC
hCoV-19/Botswana/R69B55_BHP_916539/2021_EPI_ISL_90...	(24133)	TGCACTGTTAGCGGGTACAATCACTTCTGGTTGGACCTTTGGTGCAGGTGCTGCATTACAAATACCATTTGC
hCoV-19/India/HR-MDU-IGIB1210605800489930/2022_EPI...	(24175)	TGCACTGTTAGCGGGTACAATCACTTCTGGTTGGACCTTTGGTGCAGGTGCTGCATTACAAATACCATTTGC
hCoV-19/England/PLYM-332B917/2022_EPI_ISL_9062229_...	(24116)	TGCACTGTTAGCGGGTACAATCACTTCTGGTTGGACCTTTGGTGCAGGTGCTGCATTACAAATACCATTTGC
hCoV-19/Germany/HE-RKI-I-438397/2022_EPI_ISL_905522...	(24124)	TGCACTGTTAGCGGGTACAATCACTTCTGGTTGGACCTTTGGTGCAGGTGCTGCATTACAAATACCATTTGC
hCoV-19/USA/DE-CDC-LC0472738/2021_EPI_ISL_9049423...	(24094)	TGCACTGTTAGCGGGTACAATCACTTCTGGTTGGACCTTTGGTGCAGGTGCTGCATTACAAATACCATTTGC

Section 338

	(24265)	24265 24270 24280 24290 24300 24310 24320 24336
SARS-CoV-2 Reference Genome NC_045512.2	(24259)	TATGCAAATGGCTTATAGGTTTAATGGTATTGGAGTTACACAGAATGTTCTCTATGAGAACCAAAAATTGAT
hCoV-19/Botswana/R69B55_BHP_916539/2021_EPI_ISL_90...	(24205)	TATGCAAATGGCTTATAGGTTTAATGGTATTGGAGTTACACAGAATGTTCTCTATGAGAACCAAAAATTGAT
hCoV-19/India/HR-MDU-IGIB1210605800489930/2022_EPI...	(24247)	TATGCAAATGGCTTATAGGTTTAATGGTATTGGAGTTACACAGAATGTTCTCTATGAGAACCAAAAATTGAT
hCoV-19/England/PLYM-332B917/2022_EPI_ISL_9062229_...	(24188)	TATGCAAATGGCTTATAGGTTTAATGGTATTGGAGTTACACAGAATGTTCTCTATGAGAACCAAAAATTGAT
hCoV-19/Germany/HE-RKI-I-438397/2022_EPI_ISL_905522...	(24196)	TATGCAAATGGCTTATAGGTTTAATGGTATTGGAGTTACACAGAATGTTCTCTATGAGAACCAAAAATTGAT
hCoV-19/USA/DE-CDC-LC0472738/2021_EPI_ISL_9049423...	(24166)	TATGCAAATGGCTTATAGGTTTAATGGTATTGGAGTTACACAGAATGTTCTCTATGAGAACCAAAAATTGAT

Section 339

	(24337)	24337 24350 24360 24370 24380 24390 24408
SARS-CoV-2 Reference Genome NC_045512.2	(24331)	TGCCAACCAATTTAATAGTGCTATTGGCAAAATTCAAGACTCACTTTCTTCCACAGCAAGTGCACTTGGAAA
hCoV-19/Botswana/R69B55_BHP_916539/2021_EPI_ISL_90...	(24277)	TGCCAACCAATTTAATAGTGCTATTGGCAAAATTCAAGACTCACTTTCTTCCACAGCAAGTGCACTTGGAAA
hCoV-19/India/HR-MDU-IGIB1210605800489930/2022_EPI...	(24319)	TGCCAACCAATTTAATAGTGCTATTGGCAAAATTCAAGACTCACTTTCTTCCACAGCAAGTGCACTTGGAAA
hCoV-19/England/PLYM-332B917/2022_EPI_ISL_9062229_...	(24260)	TGCCAACCAATTTAATAGTGCTATTGGCAAAATTCAAGACTCACTTTCTTCCACAGCAAGTGCACTTGGAAA
hCoV-19/Germany/HE-RKI-I-438397/2022_EPI_ISL_905522...	(24268)	TGCCAACCAATTTAATAGTGCTATTGGCAAAATTCAAGACTCACTTTCTTCCACAGCAAGTGCACTTGGAAA
hCoV-19/USA/DE-CDC-LC0472738/2021_EPI_ISL_9049423...	(24238)	TGCCAACCAATTTAATAGTGCTATTGGCAAAATTCAAGACTCACTTTCTTCCACAGCAAGTGCACTTGGAAA

Section 340

	(24409)	24409 24420 24430 24440 24450 24460 24470 24480
SARS-CoV-2 Reference Genome NC_045512.2	(24403)	ACTTCAAGATGTGGTCAACCAAAATGCACAAGCTTTAAACACGCTTGTTAAACAACTTAGCTCCAATTTTGG
hCoV-19/Botswana/R69B55_BHP_916539/2021_EPI_ISL_90...	(24349)	ACTTCAAGATGTGGTCAACCATAATGCACAAGCTTTAAACACGCTTGTTAAACAACTTAGCTCCAAATTTGG
hCoV-19/India/HR-MDU-IGIB1210605800489930/2022_EPI...	(24391)	ACTTCAAGATGTGGTCAACCATAATGCACAAGCTTTAAACACGCTTGTTAAACAACTTAGCTCCAAATTTGG
hCoV-19/England/PLYM-332B917/2022_EPI_ISL_9062229_...	(24332)	ACTTCAAGATGTGGTCAACCATAATGCACAAGCTTTAAACACGCTTGTTAAACAACTTAGCTCCAAATTTGG
hCoV-19/Germany/HE-RKI-I-438397/2022_EPI_ISL_905522...	(24340)	ACTTCAAGATGTGGTCAACCATAATGCACAAGCTTTAAACACGCTTGTTAAACAACTTAGCTCCAAATTTGG
hCoV-19/USA/DE-CDC-LC0472738/2021_EPI_ISL_9049423...	(24310)	ACTTCAAGATGTGGTCAACCATAATGCACAAGCTTTAAACACGCTTGTTAAACAACTTAGCTCCAAATTTGG

Section 341

(24481)	24481 24490 24500 24510 24520 24530 24540 24552
SARS-CoV-2 Reference Genome NC_045512.2 (24475)	TGCAATTTCAAGTGTTTTAAATGATATCCTTTCACGTCTTGACAAAGTTGAGGCTGAAGTGCAAATTGATAG
hCoV-19/Botswana/R69B55_BHP_916539/2021_EPI_ISL_90... (24421)	TGCAATTTCAAGTGTTTTAAATGATATCTTTTCACGTCTTGACAAAGTTGAGGCTGAAGTGCAAATTGATAG
hCoV-19/India/HR-MDU-IGIB1210605800489930/2022_EPI... (24463)	TGCAATTTCAAGTGTTTTAAATGATATCTTTTCACGTCTTGACAAAGTTGAGGCTGAAGTGCAAATTGATAG
hCoV-19/England/PLYM-332B917/2022_EPI_ISL_9062229_... (24404)	TGCAATTTCAAGTGTTTTAAATGATATCTTTTCACGTCTTGACAAAGTTGAGGCTGAAGTGCAAATTGATAG
hCoV-19/Germany/HE-RKI-I-438397/2022_EPI_ISL_905522... (24412)	TGCAATTTCAAGTGTTTTAAATGATATCTTTTCACGTCTTGACAAAGTTGAGGCTGAAGTGCAAATTGATAG
hCoV-19/USA/DE-CDC-LC0472738/2021_EPI_ISL_9049423... (24382)	TGCAATTTCAAGTGTTTTAAATGATATCTTTTCACGTCTTGACAAAGTTGAGGCTGAAGTGCAAATTGATAG

Section 342

(24553)	24553 24560 24570 24580 24590 24600 24610 24624
SARS-CoV-2 Reference Genome NC_045512.2 (24547)	GTTGATCACAGGCAGACTTCAAAGTTTGCAGACATATGTGACTCAACAATTAATTAGAGCTGCAGAAATCAG
hCoV-19/Botswana/R69B55_BHP_916539/2021_EPI_ISL_90... (24493)	GTTGATCACAGGCAGACTTCAAAGTTTGCAGACATATGTGACTCAACAATTAATTAGAGCTGCAGAAATCAG
hCoV-19/India/HR-MDU-IGIB1210605800489930/2022_EPI... (24535)	GTTGATCACAGGCAGACTTCAAAGTTTGCAGACATATGTGACTCAACAATTAATTAGAGCTGCAGAAATCAG
hCoV-19/England/PLYM-332B917/2022_EPI_ISL_9062229_... (24476)	GTTGATCACAGGCAGACTTCAAAGTTTGCAGACATATGTGACTCAACAATTAATTAGAGCTGCAGAAATCAG
hCoV-19/Germany/HE-RKI-I-438397/2022_EPI_ISL_905522... (24484)	GTTGATCACAGGCAGACTTCAAAGTTTGCAGACATATGTGACTCAACAATTAATTAGAGCTGCAGAAATCAG
hCoV-19/USA/DE-CDC-LC0472738/2021_EPI_ISL_9049423... (24454)	GTTGATCACAGGCAGACTTCAAAGTTTGCAGACATATGTGACTCAACAATTAATTAGAGCTGCAGAAATCAG

Section 343

(24625)	24625 24630 24640 24650 24660 24670 24680 24696
SARS-CoV-2 Reference Genome NC_045512.2 (24619)	AGCTTCTGCTAATCTTGCTGCTACTAAAATGTCAGAGTGTGTACTTGGACAATCAAAAAGAGTTGATTTTTG
hCoV-19/Botswana/R69B55_BHP_916539/2021_EPI_ISL_90... (24565)	AGCTTCTGCTAATCTTGCTGCTACTAAAATGTCAGAGTGTGTACTTGGACAATCAAAAAGAGTTGATTTTTG
hCoV-19/India/HR-MDU-IGIB1210605800489930/2022_EPI... (24607)	AGCTTCTGCTAATCTTGCTGCTACTAAAATGTCAGAGTGTGTACTTGGACAATCAAAAAGAGTTGATTTTTG
hCoV-19/England/PLYM-332B917/2022_EPI_ISL_9062229_... (24548)	AGCTTCTGCTAATCTTGCTGCTACTAAAATGTCAGAGTGTGTACTTGGACAATCAAAAAGAGTTGATTTTTG
hCoV-19/Germany/HE-RKI-I-438397/2022_EPI_ISL_905522... (24556)	AGCTTCTGCTAATCTTGCTGCTACTAAAATGTCAGAGTGTGTACTTGGACAATCAAAAAGAGTTGATTTTTG
hCoV-19/USA/DE-CDC-LC0472738/2021_EPI_ISL_9049423... (24526)	AGCTTCTGCTAATCTTGCTGCTACTAAAATGTCAGAGTGTGTACTTGGACAATCAAAAAGAGTTGATTTTTG

Section 344

(24697)	24697 24710 24720 24730 24740 24750 24768
SARS-CoV-2 Reference Genome NC_045512.2 (24691)	TGGAAAGGGCTATCATCTTATGTCCTTCCCTCAGTCAGCACCTCATGGTGTAGTCTTCTTGCATGTGACTTA
hCoV-19/Botswana/R69B55_BHP_916539/2021_EPI_ISL_90... (24637)	TGGAAAGGGCTATCATCTTATGTCCTTCCCTCAGTCAGCACCTCATGGTGTAGTCTTCTTGCATGTGACTTA
hCoV-19/India/HR-MDU-IGIB1210605800489930/2022_EPI... (24679)	TGGAAAGGGCTATCATCTTATGTCCTTCCCTCAGTCAGCACCTCATGGTGTAGTCTTCTTGCATGTGACTTA
hCoV-19/England/PLYM-332B917/2022_EPI_ISL_9062229_... (24620)	TGGAAAGGGCTATCATCTTATGTCCTTCCCTCAGTCAGCACCTCATGGTGTAGTCTTCTTGCATGTGACTTA
hCoV-19/Germany/HE-RKI-I-438397/2022_EPI_ISL_905522... (24628)	TGGAAAGGGCTATCATCTTATGTCCTTCCCTCAGTCAGCACCTCATGGTGTAGTCTTCTTGCATGTGACTTA
hCoV-19/USA/DE-CDC-LC0472738/2021_EPI_ISL_9049423... (24598)	TGGAAAGGGCTATCATCTTATGTCCTTCCCTCAGTCAGCACCTCATGGTGTAGTCTTCTTGCATGTGACTTA

Section 345

		24769 24780 24790 24800 24810 24820 24830 24840
	(24769)	
SARS-CoV-2 Reference Genome NC_045512.2	(24763)	TGTCCCTGCACAAGAAAAGAACTTCACAACTGCTCCTGCCATTTGTCATGATGGAAAAGCACACTTTCCTCG
hCoV-19/Botswana/R69B55_BHP_916539/2021_EPI_ISL_90...	(24709)	TGTCCCTGCACAAGAAAAGAACTTCACAACTGCTCCTGCCATTTGTCATGATGGAAAAGCACACTTTCCTCG
hCoV-19/India/HR-MDU-IGIB1210605800489930/2022_EPI...	(24751)	TGTCCCTGCACAAGAAAAGAACTTCACAACTGCTCCTGCCATTTGTCATGATGGAAAAGCACACTTTCCTCG
hCoV-19/England/PLYM-332B917/2022_EPI_ISL_9062229_...	(24692)	TGTCCCTGCACAAGAAAAGAACTTCACAACTGCTCCTGCCATTTGTCATGATGGAAAAGCACACTTTCCTCG
hCoV-19/Germany/HE-RKI-I-438397/2022_EPI_ISL_905522...	(24700)	TGTCCCTGCACAAGAAAAGAACTTCACAACTGCTCCTGCCATTTGTCATGATGGAAAAGCACACTTTCCTCG
hCoV-19/USA/DE-CDC-LC0472738/2021_EPI_ISL_9049423...	(24670)	TGTCCCTGCACAAGAAAAGAACTTCACAACTGCTCCTGCCATTTGTCATGATGGAAAAGCACACTTTCCTCG

Section 346

		24841 24850 24860 24870 24880 24890 24900 24912
	(24841)	
SARS-CoV-2 Reference Genome NC_045512.2	(24835)	TGAAGGTGTCTTTGTTTCAAATGGCACACACTGGTTTGTAACACAAAGGAATTTTTATGAACCACAAATCAT
hCoV-19/Botswana/R69B55_BHP_916539/2021_EPI_ISL_90...	(24781)	TGAAGGTGTCTTTGTTTCAAATGGCACACACTGGTTTGTAACACAAAGGAATTTTTATGAACCACAAATCAT
hCoV-19/India/HR-MDU-IGIB1210605800489930/2022_EPI...	(24823)	TGAAGGTGTCTTTGTTTCAAATGGCACACACTGGTTTGTAACACAAAGGAATTTTTATGAACCACAAATCAT
hCoV-19/England/PLYM-332B917/2022_EPI_ISL_9062229_...	(24764)	TGAAGGTGTCTTTGTTTCAAATGGCACACACTGGTTTGTAACACAAAGGAATTTTTATGAACCACAAATCAT
hCoV-19/Germany/HE-RKI-I-438397/2022_EPI_ISL_905522...	(24772)	TGAAGGTGTCTTTGTTTCAAATGGCACACACTGGTTTGTAACACAAAGGAATTTTTATGAACCACAAATCAT
hCoV-19/USA/DE-CDC-LC0472738/2021_EPI_ISL_9049423...	(24742)	TGAAGGTGTCTTTGTTTCAAATGGCACACACTGGTTTGTAACACAAAGGAATTTTTATGAACCACAAATCAT

Section 347

		24913 24920 24930 24940 24950 24960 24970 24984
	(24913)	
SARS-CoV-2 Reference Genome NC_045512.2	(24907)	TACTACAGACAACACATTTGTGTCTGGTAACTGTGATGTTGTAATAGGAATTGTCAACAACACAGTTTATGA
hCoV-19/Botswana/R69B55_BHP_916539/2021_EPI_ISL_90...	(24853)	TACTACAGACAACACATTTGTGTCTGGTAACTGTGATGTTGTAATAGGAATTGTCAACAACACAGTTTATGA
hCoV-19/India/HR-MDU-IGIB1210605800489930/2022_EPI...	(24895)	TACTACAGACAACACATTTGTGTCTGGTAACTGTGATGTTGTAATAGGAATTGTCAACAACACAGTTTATGA
hCoV-19/England/PLYM-332B917/2022_EPI_ISL_9062229_...	(24836)	TACTACAGACAACACATTTGTGTCTGGTAACTGTGATGTTGTAATAGGAATTGTCAACAACACAGTTTATGA
hCoV-19/Germany/HE-RKI-I-438397/2022_EPI_ISL_905522...	(24844)	TACTACAGACAACACATTTGTGTCTGGTAACTGTGATGTTGTAATAGGAATTGTCAACAACACAGTTTATGA
hCoV-19/USA/DE-CDC-LC0472738/2021_EPI_ISL_9049423...	(24814)	TACTACAGACAACACATTTGTGTCTGGTAACTGTGATGTTGTAATAGGAATTGTCAACAACACAGTTTATGA

Section 348

		24985 24990 25000 25010 25020 25030 25040 25056
	(24985)	
SARS-CoV-2 Reference Genome NC_045512.2	(24979)	TCCTTTGCAACCTGAATTAGACTCATTCAAGGAGGAGTTAGATAAATATTTTAAGAATCATACATCACCAGA
hCoV-19/Botswana/R69B55_BHP_916539/2021_EPI_ISL_90...	(24925)	TCCTTTGCAACCTGAATTAGATTCATTCAAGGAGGAGTTAGATAAATATTTTAAGAATCATACATCACCAGA
hCoV-19/India/HR-MDU-IGIB1210605800489930/2022_EPI...	(24967)	TCCTTTGCAACCTGAATTAGATTCATTCAAGGAGGAGTTAGATAAATATTTTAAGAATCATACATCACCAGA
hCoV-19/England/PLYM-332B917/2022_EPI_ISL_9062229_...	(24908)	TCCTTTGCAACCTGAATTAGATTCATTCAAGGAGGAGTTAGATAAATATTTTAAGAATCATACATCACCAGA
hCoV-19/Germany/HE-RKI-I-438397/2022_EPI_ISL_905522...	(24916)	TCCTTTGCAACCTGAATTAGATTCATTCAAGGAGGAGTTAGATAAATATTTTAAGAATCATACATCACCAGA
hCoV-19/USA/DE-CDC-LC0472738/2021_EPI_ISL_9049423...	(24886)	TCCTTTGCAACCTGAATTAGATTCATTCAAGGAGGAGTTAGATAAATATTTTAAGAATCATACATCACCAGA

```
                                                                          Section 349
                                                    (25057) 25057        25070     25080     25090     25100     25110           25128
                SARS-CoV-2 Reference Genome NC_045512.2 (25051) TGTTGATTTAGGTGACATCTCTGGCATTAATGCTTCAGTTGTAAACATTCAAAAAGAAATTGACCGCCTCAA
  hCoV-19/Botswana/R69B55_BHP_916539/2021_EPI_ISL_90... (24997) TGTTGATTTAGGTGACATCTCTGGCATTAATGCTTCAGTTGTAAACATTCAAAAAGAAATTGACCGCCTCAA
hCoV-19/India/HR-MDU-IGIB1210605800489930/2022_EPI... (25039) TGTTGATTTAGGTGACATCTCTGGCATTAATGCTTCAGTTGTAAACATTCAAAAAGAAATTGACCGCCTCAA
 hCoV-19/England/PLYM-332B917/2022_EPI_ISL_9062229_... (24980) TGTTGATTTAGGTGACATCTCTGGCATTAATGCTTCAGTTGTAAACATTCAAAAAGAAATTGACCGCCTCAA
  hCoV-19/Germany/HE-RKI-I-438397/2022_EPI_ISL_905522... (24988) TGTTGATTTAGGTGACATCTCTGGCATTAATGCTTCAGTTGTAAACATTCAAAAAGAAATTGACCGCCTCAA
hCoV-19/USA/DE-CDC-LC0472738/2021_EPI_ISL_9049423... (24958) TGTTGATTTAGGTGACATCTCTGGCATTAATGCTTCAGTTGTAAACATTCAAAAAGAAATTGACCGCCTCAA
                                                                          Section 350
                                                    (25129) 25129      25140     25150     25160     25170     25180     25190  25200
                SARS-CoV-2 Reference Genome NC_045512.2 (25123) TGAGGTTGCCAAGAATTTAAATGAATCTCTCATCGATCTCCAAGAACTTGGAAAGTATGAGCAGTATATAAA
  hCoV-19/Botswana/R69B55_BHP_916539/2021_EPI_ISL_90... (25069) TGAGGTTGCCAAGAATTTAAATGAATCTCTCATCGATCTCCAAGAACTTGGAAAGTATGAGCAGTATATAAA
hCoV-19/India/HR-MDU-IGIB1210605800489930/2022_EPI... (25111) TGAGGTTGCCAAGAATTTAAATGAATCTCTCATCGATCTCCAAGAACTTGGAAAGTATGAGCAGTATATAAA
 hCoV-19/England/PLYM-332B917/2022_EPI_ISL_9062229_... (25052) TGAGGTTGCCAAGAATTTAAATGAATCTCTCATCGATCTCCAAGAACTTGGAAAGTATGAGCAGTATATAAA
  hCoV-19/Germany/HE-RKI-I-438397/2022_EPI_ISL_905522... (25060) TGAGGTTGCCAAGAATTTAAATGAATCTCTCATCGATCTCCAAGAACTTGGAAAGTATGAGCAGTATATAAA
hCoV-19/USA/DE-CDC-LC0472738/2021_EPI_ISL_9049423... (25030) TGAGGTTGCCAAGAATTTAAATGAATCTCTCATCGATCTCCAAGAACTTGGAAAGTATGAGCAGTATATAAA
                                                                          Section 351
                                                    (25201) 25201     25210     25220     25230     25240     25250     25260    25272
                SARS-CoV-2 Reference Genome NC_045512.2 (25195) ATGGCCATGGTACATTTGGCTAGGTTTTATAGCTGGCTTGATTGCCATAGTAATGGTGACAATTATGCTTTG
  hCoV-19/Botswana/R69B55_BHP_916539/2021_EPI_ISL_90... (25141) ATGGCCATGGTACATTTGGCTAGGTTTTATAGCTGGCTTGATTGCCATAGTAATGGTGACAATTATGCTTTG
hCoV-19/India/HR-MDU-IGIB1210605800489930/2022_EPI... (25183) ATGGCCATGGTACATTTGGCTAGGTTTTATAGCTGGCTTGATTGCCATAGTAATGGTGACAATTATGCTTTG
 hCoV-19/England/PLYM-332B917/2022_EPI_ISL_9062229_... (25124) ATGGCCATGGTACATTTGGCTAGGTTTTATAGCTGGCTTGATTGCCATAGTAATGGTGACAATTATGCTTTG
  hCoV-19/Germany/HE-RKI-I-438397/2022_EPI_ISL_905522... (25132) ATGGCCATGGTACATTTGGCTAGGTTTTATAGCTGGCTTGATTGCCATAGTAATGGTGACAATTATGCTTTG
hCoV-19/USA/DE-CDC-LC0472738/2021_EPI_ISL_9049423... (25102) ATGGCCATGGTACATTTGGCTAGGTTTTATAGCTGGCTTGATTGCCATAGTAATGGTGACAATTATGCTTTG
                                                                          Section 352
                                                    (25273) 25273  25280     25290     25300     25310     25320     25330        25344
                SARS-CoV-2 Reference Genome NC_045512.2 (25267) CTGTATGACCAGTTGCTGTAGTTGTCTCAAGGGCTGTTGTTCTTGTGGATCCTGCTGCAAATTTGATGAAGA
  hCoV-19/Botswana/R69B55_BHP_916539/2021_EPI_ISL_90... (25213) CTGTATGACCAGTTGCTGTAGTTGTCTCAAGGGCTGTTGTTCTTGTGGATCCTGCTGCAAATTTGATGAAGA
hCoV-19/India/HR-MDU-IGIB1210605800489930/2022_EPI... (25255) CTGTATGACCAGTTGCTGTAGTTGTCTCAAGGGCTGTTGTTCTTGTGGATCCTGCTGCAAATTTGATGAAGA
 hCoV-19/England/PLYM-332B917/2022_EPI_ISL_9062229_... (25196) CTGTATGACCAGTTGCTGTAGTTGTCTCAAGGGCTGTTGTTCTTGTGGATCCTGCTGCAAATTTGATGAAGA
  hCoV-19/Germany/HE-RKI-I-438397/2022_EPI_ISL_905522... (25204) CTGTATGACCAGTTGCTGTAGTTGTCTCAAGGGCTGTTGTTCTTGTGGATCCTGCTGCAAATTTGATGAAGA
hCoV-19/USA/DE-CDC-LC0472738/2021_EPI_ISL_9049423... (25174) CTGTATGACCAGTTGCTGTAGTTGTCTCAAGGGCTGTTGTTCTTGTGGATCCTGCTGCAAATTTGATGAAGA
```

Section 353

(25345)	25345 25350 25360 25370 25380 25390 25400 25416
SARS-CoV-2 Reference Genome NC_045512.2 (25339)	CGACTCTGAGCCAGTGCTCAAAGGAGTCAAATTACATTACACATAAACGAACTTATGGATTTGTTTATGAGA
hCoV-19/Botswana/R69B55_BHP_916539/2021_EPI_ISL_90... (25285)	CGACTCTGAGCCAGTGCTCAAAGGAGTCAAATTACATTACACATAAACGAACTTATGGATTTGTTTATGAGA
hCoV-19/India/HR-MDU-IGIB1210605800489930/2022_EPI... (25327)	CGACTCTGAGCCAGTGCTCAAAGGAGTCAAATTACATTACACATAAACGAACTTATGGATTTGTTTATGAGA
hCoV-19/England/PLYM-332B917/2022_EPI_ISL_9062229_... (25268)	CGACTCTGAGCCAGTGCTCAAAGGAGTCAAATTACATTACACATAAACGAACTTATGGATTTGTTTATGAGA
hCoV-19/Germany/HE-RKI-I-438397/2022_EPI_ISL_905522... (25276)	CGACTCTGAGCCAGTGCTCAAAGGAGTCAAATTACATTACACATAAACGAACTTATGGATTTGTTTATGAGA
hCoV-19/USA/DE-CDC-LC0472738/2021_EPI_ISL_9049423... (25246)	CGACTCTGAGCCAGTGCTCAAAGGAGTCAAATTACATTACACATAAACGAACTTATGGATTTGTTTATGAGA

Section 354

(25417)	25417 25430 25440 25450 25460 25470 25488
SARS-CoV-2 Reference Genome NC_045512.2 (25411)	ATCTTCACAATTGGAACTGTAACTTTGAAGCAAGGTGAAATCAAGGATGCTACTCCTTCAGATTTTGTTCGC
hCoV-19/Botswana/R69B55_BHP_916539/2021_EPI_ISL_90... (25357)	ATCTTCACAATTGGAACTGTAACTTTGAAGCAAGGTGAAATCAAGGATGCTACTCCTTCAGATTTTGTTCGC
hCoV-19/India/HR-MDU-IGIB1210605800489930/2022_EPI... (25399)	ATCTTCACAATTGGAACTGTAACTTTGAAGCAAGGTGAAATCAAGGATGCTACTCCTTCAGATTTTGTTCGC
hCoV-19/England/PLYM-332B917/2022_EPI_ISL_9062229_... (25340)	ATCTTCACAATTGGAACTGTAACTTTGAAGCAAGGTGAAATCAAGGATGCTACTCCTTCAGATTTTGTTCGC
hCoV-19/Germany/HE-RKI-I-438397/2022_EPI_ISL_905522... (25348)	ATCTTCACAATTGGAACTGTAACTTTGAAGCAAGGTGAAATCAAGGATGCTACTCCTTCAGATTTTGTTCGC
hCoV-19/USA/DE-CDC-LC0472738/2021_EPI_ISL_9049423... (25318)	ATCTTCACAATTGGAACTGTAACTTTGAAGCAAGGTGAAATCAAGGATGCTACTCCTTCAGATTTTGTTCGC

Section 355

(25489)	25489 25500 25510 25520 25530 25540 25550 25560
SARS-CoV-2 Reference Genome NC_045512.2 (25483)	GCTACTGCAACGATACCGATACAAGCCTCACTCCCTTTCGGATGGCTTATTGTTGGCGTTGCACTTCTTGCT
hCoV-19/Botswana/R69B55_BHP_916539/2021_EPI_ISL_90... (25429)	GCTACTGCAACGATACCGATACAAGCCTCACTCCCTTTCGGATGGCTTATTGTTGGCGTTGCACTTCTTGCT
hCoV-19/India/HR-MDU-IGIB1210605800489930/2022_EPI... (25471)	GCTACTGCAACGATACCGATACAAGCCTCACTCCCTTTCGGATGGCTTATTGTTGGCGTTGCACTTCTTGCT
hCoV-19/England/PLYM-332B917/2022_EPI_ISL_9062229_... (25412)	GCTACTGCAACGATACCGATACAAGCCTCACTCCCTTTCGGATGGCTTATTGTTGGCGTTGCACTTCTTGCT
hCoV-19/Germany/HE-RKI-I-438397/2022_EPI_ISL_905522... (25420)	GCTACTGCAACGATACCGATACAAGCCTCACTCCCTTTCGGATGGCTTATTGTTGGCGTTGCACTTCTTGCT
hCoV-19/USA/DE-CDC-LC0472738/2021_EPI_ISL_9049423... (25390)	GCTACTGCAACGATACCGATACAAGCCTCACTCCCTTTCGGATGGCTTATTGTTGGCGTTGCACTTCTTGCT

Section 356

(25561)	25561 25570 25580 25590 25600 25610 25620 25632
SARS-CoV-2 Reference Genome NC_045512.2 (25555)	GTTTTTCAGAGCGCTTCCAAAATCATAACCCTCAAAAAGAGATGGCAACTAGCACTCTCCAAGGGTGTTCAC
hCoV-19/Botswana/R69B55_BHP_916539/2021_EPI_ISL_90... (25501)	GTTTTTCAGAGCGCTTCCAAAATCATAACTCTCAAAAAGAGATGGCAACTAGCACTCTCCAAGGGTGTTCAC
hCoV-19/India/HR-MDU-IGIB1210605800489930/2022_EPI... (25543)	GTTTTTCAGAGCGCTTCCAAAATCATAACTCTCAAAAAGAGATGGCAACTAGCACTCTCCAAGGGTGTTCAC
hCoV-19/England/PLYM-332B917/2022_EPI_ISL_9062229_... (25484)	GTTTTTCAGAGCGCTTCCAAAATCATAACTCTCAAAAAGAGATGGCAACTAGCACTCTCCAAGGGTGTTCAC
hCoV-19/Germany/HE-RKI-I-438397/2022_EPI_ISL_905522... (25492)	GTTTTTCAGAGCGCTTCCAAAATCATAACTCTCAAAAAGAGATGGCAACTAGCACTCTCCAAGGGTGTTCAC
hCoV-19/USA/DE-CDC-LC0472738/2021_EPI_ISL_9049423... (25462)	GTTTTTCAGAGCGCTTCCAAAATCATAACTCTCAAAAAGAGATGGCAACTAGCACTCTCCAAGGGTGTTCAC

Section 357

		25633 25640 25650 25660 25670 25680 25690 25704
	(25633)	
SARS-CoV-2 Reference Genome NC_045512.2	(25627)	TTTGTTTGCAACTTGCTGTTGTTGTTTGTAACAGTTTACTCACACCTTTTGCTCGTTGCTGCTGGCCTTGAA
hCoV-19/Botswana/R69B55_BHP_916539/2021_EPI_ISL_90...	(25573)	TTTGTTTGCAACTTGCTGTTGTTGTTTGTAACAGTTTACTCACACCTTTTGCTCGTTGCTGCTGGCCTTGAA
hCoV-19/India/HR-MDU-IGIB1210605800489930/2022_EPI...	(25615)	TTTGTTTGCAACTTGCTGTTGTTGTTTGTAACAGTTTACTCACACCTTTTGCTCGTTGCTGCTGGCCTTGAA
hCoV-19/England/PLYM-332B917/2022_EPI_ISL_9062229_...	(25556)	TTTGTTTGCAACTTGCTGTTGTTGTTTGTAACAGTTTACTCACACCTTTTGCTCGTTGCTGCTGGCCTTGAA
hCoV-19/Germany/HE-RKI-I-438397/2022_EPI_ISL_905522...	(25564)	TTTGTTTGCAACTTGCTGTTGTTGTTTGTAACAGTTTACTCACACCTTTTGCTCGTTGCTGCTGGCCTTGAA
hCoV-19/USA/DE-CDC-LC0472738/2021_EPI_ISL_9049423...	(25534)	TTTGTTTGCAACTTGCTGTTGTTGTTTGTAACAGTTTACTCACACCTTTTGCTCGTTGCTGCTGGCCTTGAA

Section 358

		25705 25710 25720 25730 25740 25750 25760 25776
	(25705)	
SARS-CoV-2 Reference Genome NC_045512.2	(25699)	GCCCCTTTTCTCTATCTTTATGCTTTAGTCTACTTCTTGCAGAGTATAAACTTTGTAAGAATAATAATGAGG
hCoV-19/Botswana/R69B55_BHP_916539/2021_EPI_ISL_90...	(25645)	GCCCCTTTTCTCTATCTTTATGCTTTAGTCTACTTCTTGCAGAGTATAAACTTTGTAAGAATAATAATGAGG
hCoV-19/India/HR-MDU-IGIB1210605800489930/2022_EPI...	(25687)	GCCCCTTTTCTCTATCTTTATGCTTTAGTCTACTTCTTGCAGAGTATAAACTTTGTAAGAATAATAATGAGG
hCoV-19/England/PLYM-332B917/2022_EPI_ISL_9062229_...	(25628)	GCCCCTTTTCTCTATCTTTATGCTTTAGTCTACTTCTTGCAGAGTATAAACTTTGTAAGAATAATAATGAGG
hCoV-19/Germany/HE-RKI-I-438397/2022_EPI_ISL_905522...	(25636)	GCCCCTTTTCTCTATCTTTATGCTTTAGTCTACTTCTTGCAGAGTATAAACTTTGTAAGAATAATAATGAGG
hCoV-19/USA/DE-CDC-LC0472738/2021_EPI_ISL_9049423...	(25606)	GCCCCTTTTTTCTATCTTTATGCTTTAGTCTACTTCTTGCAGAGTATAAACTTTGTAAGAATAATAATGAGG

Section 359

		25777 25790 25800 25810 25820 25830 25848
	(25777)	
SARS-CoV-2 Reference Genome NC_045512.2	(25771)	CTTTGGCTTTGCTGGAAATGCCGTTCCAAAAACCCATTACTTTATGATGCCAACTATTTTCTTTGCTGGCAT
hCoV-19/Botswana/R69B55_BHP_916539/2021_EPI_ISL_90...	(25717)	CTTTGGCTTTGCTGGAAATGCCGTTCCAAAAACCCATTACTTTATGATGCCAACTATTTTCTTTGCTGGCAT
hCoV-19/India/HR-MDU-IGIB1210605800489930/2022_EPI...	(25759)	CTTTGGCTTTGCTGGAAATGCCGTTCCAAAAACCCATTACTTTATGATGCCAACTATTTTCTTTGCTGGCAT
hCoV-19/England/PLYM-332B917/2022_EPI_ISL_9062229_...	(25700)	CTTTGGCTTTGCTGGAAATGCCGTTCCAAAAACCCATTACTTTATGATGCCAACTATTTTCTTTGCTGGCAT
hCoV-19/Germany/HE-RKI-I-438397/2022_EPI_ISL_905522...	(25708)	CTTTGGCTTTGCTGGAAATGCCGTTCCAAAAACCCATTACTTTATGATGCCAACTATTTTCTTTGCTGGCAT
hCoV-19/USA/DE-CDC-LC0472738/2021_EPI_ISL_9049423...	(25678)	CTTTGGCTTTGCTGGAAATGCCGTTCCAAAAACCCATTACTTTATGATGCCAACTATTTTCTTTGCTGGCAT

Section 360

		25849 25860 25870 25880 25890 25900 25910 25920
	(25849)	
SARS-CoV-2 Reference Genome NC_045512.2	(25843)	ACTAATTGTTACGACTATTGTATACCTTACAATAGTGTAACTTCTTCAATTGTCATTACTTCAGGTGATGGC
hCoV-19/Botswana/R69B55_BHP_916539/2021_EPI_ISL_90...	(25789)	ACTAATTGTTACGACTATTGTATACCTTACAATAGTGTAACTTCTTCAATTGTCATTACTTCAGGTGATGGC
hCoV-19/India/HR-MDU-IGIB1210605800489930/2022_EPI...	(25831)	ACTAATTGTTACGACTATTGTATACCTTACAATAGTGTAACTTCTTCAATTGTCATTACTTCAGGTGATGGC
hCoV-19/England/PLYM-332B917/2022_EPI_ISL_9062229_...	(25772)	ACTAATTGTTACGACTATTGTATACCTTACAATAGTGTAACTTCTTCAATTGTCATTACTTCAGGTGATGGC
hCoV-19/Germany/HE-RKI-I-438397/2022_EPI_ISL_905522...	(25780)	ACTAATTGTTACGACTATTGTATACCTTACAATAGTGTAACTTCTTCAATTGTCATTACTTCAGGTGATGGC
hCoV-19/USA/DE-CDC-LC0472738/2021_EPI_ISL_9049423...	(25750)	ACTAATTGTTACGACTATTGTATACCTTACAATAGTGTAACTTCTTCAATTGTCATTACTTCAGGTGATGGC

Omicron BA.1

Section 361

Sequence	Start	25921 … 25992
	(25921)	
SARS-CoV-2 Reference Genome NC_045512.2	(25915)	ACAACAAGTCCTATTTCTGAACATGACTACCAGATTGGTGGTTATACTGAAAAATGGGAATCTGGAGTAAAA
hCoV-19/Botswana/R69B55_BHP_916539/2021_EPI_ISL_90...	(25861)	ACAACAAGTCCTATTTCTGAACATGACTACCAGATTGGTGGTTATACTGAAAAATGGGAATCTGGAGTAAAA
hCoV-19/India/HR-MDU-IGIB1210605800489930/2022_EPI...	(25903)	ACAACAAGTCCTATTTCTGAACATGACTACCAGATTGGTGGTTATACTGAAAAATGGGAATCTGGAGTAAAA
hCoV-19/England/PLYM-332B917/2022_EPI_ISL_9062229_...	(25844)	ACAACAAGTCCTATTTCTGAACATGACTACCAGATTGGTGGTTATACTGAAAAATGGGAATCTGGAGTAAAA
hCoV-19/Germany/HE-RKI-I-438397/2022_EPI_ISL_905522...	(25852)	ACAACAAGTCCTATTTCTGAACATGACTACCAGATTGGTGGTTATACTGAAAAATGGGAATCTGGAGTAAAA
hCoV-19/USA/DE-CDC-LC0472738/2021_EPI_ISL_9049423...	(25822)	ACAACAAGTCCTATTTCTGAACATGACTACCAGATTGGTGGTTATACTGAAAAATGGGAATCTGGAGTAAAA

Section 362

Sequence	Start	25993 … 26064
	(25993)	
SARS-CoV-2 Reference Genome NC_045512.2	(25987)	GACTGTGTTGTATTACACAGTTACTTCACTTCAGACTATTACCAGCTGTACTCAACTCAATTGAGTACAGAC
hCoV-19/Botswana/R69B55_BHP_916539/2021_EPI_ISL_90...	(25933)	GACTGTGTTGTATTACACAGTTACTTCACTTCAGACTATTACCAGCTGTACTCAACTCAATTGAGTACAGAC
hCoV-19/India/HR-MDU-IGIB1210605800489930/2022_EPI...	(25975)	GACTGTGTTGTATTACACAGTTACTTCACTTCAGACTATTACCAGCTGTACTCAACTCAATTGAGTACAGAC
hCoV-19/England/PLYM-332B917/2022_EPI_ISL_9062229_...	(25916)	GACTGTGTTGTATTACACAGTTACTTCACTTCAGACTATTACCAGCTGTACTCAACTCAATTGAGTACAGAC
hCoV-19/Germany/HE-RKI-I-438397/2022_EPI_ISL_905522...	(25924)	GACTGTGTTGTATTACACAGTTACTTCACTTCAGACTATTACCAGCTGTACTCAACTCAATTGAGTACAGAC
hCoV-19/USA/DE-CDC-LC0472738/2021_EPI_ISL_9049423...	(25894)	GACTGTGTTGTATTACACAGTTACTTCACTTCAGACTATTACCAGCTGTACTCAACTCAATTGAGTACAGAC

Section 363

Sequence	Start	26065 … 26136
	(26065)	
SARS-CoV-2 Reference Genome NC_045512.2	(26059)	ACTGGTGTTGAACATGTTACCTTCTTCATCTACAATAAAATTGTTGATGAGCCTGAAGAACATGTCCAAATT
hCoV-19/Botswana/R69B55_BHP_916539/2021_EPI_ISL_90...	(26005)	ACTGGTGTTGAACATGTTACCTTCTTCATCTACAATAAAATTGTTGATGAGCCTGAAGAACATGTCCAAATT
hCoV-19/India/HR-MDU-IGIB1210605800489930/2022_EPI...	(26047)	ACTGGTGTTGAACATGTTACCTTCTTCATCTACAATAAAATTGTTGATGAGCCTGAAGAACATGTCCAAATT
hCoV-19/England/PLYM-332B917/2022_EPI_ISL_9062229_...	(25988)	ACTGGTGTTGAACATGTTACCTTCTTCATCTACAATAAAATTGTTGATGAGCCTGAAGAACATGTCCAAATT
hCoV-19/Germany/HE-RKI-I-438397/2022_EPI_ISL_905522...	(25996)	ACTGGTGTTGAACATGTTACCTTCTTCATCTACAATAAAATTGTTGATGAGCCTGAAGAACATGTCCAAATT
hCoV-19/USA/DE-CDC-LC0472738/2021_EPI_ISL_9049423...	(25966)	ACTGGTGTTGAACATGTTACCTTCTTCATCTACAATAAAATTGTTGATGAGCCTGAAGAACATGTCCAAATT

Section 364

Sequence	Start	26137 … 26208
	(26137)	
SARS-CoV-2 Reference Genome NC_045512.2	(26131)	CACACAATCGACGGTTCATCCGGAGTTGTTAATCCAGTAATGGAACCAATTTATGATGAACCGACGACGACT
hCoV-19/Botswana/R69B55_BHP_916539/2021_EPI_ISL_90...	(26077)	CACACAATCGACGGTTCATCCGGAGTTGTTAATCCAGTAATGGAACCAATTTATGATGAACCGACGACGACT
hCoV-19/India/HR-MDU-IGIB1210605800489930/2022_EPI...	(26119)	CACACAATCGACGGTTCATCCGGAGTTGTTAATCCAGTAATGGAACCAATTTATGATGAACCGACGACGACT
hCoV-19/England/PLYM-332B917/2022_EPI_ISL_9062229_...	(26060)	CACACAATCGACGGTTCATCCGGAGTTGTTAATCCAGTAATGGAACCAATTTATGATGAACCGACGACGACT
hCoV-19/Germany/HE-RKI-I-438397/2022_EPI_ISL_905522...	(26068)	CACACAATCGACGGTTCATCCGGAGTTGTTAATCCAGTAATGGAACCAATTTATGATGAACCGACGACGACT
hCoV-19/USA/DE-CDC-LC0472738/2021_EPI_ISL_9049423...	(26038)	CACACAATCGACGGTTCATCCGGAGTTGTTAATCCAGTAATGGAACCAATTTATGATGAACCGACGACGACT

Section 365

(26209)	26209 26220 26230 26240 26250 26260 26270 26280
SARS-CoV-2 Reference Genome NC_045512.2 (26203)	ACTAGCGTGCCTTTGTAAGCACAAGCTGATGAGTACGAACTTATGTACTCATTCGTTTCGGAAGAGACAGGT
hCoV-19/Botswana/R69B55_BHP_916539/2021_EPI_ISL_90... (26149)	ACTAGCGTGCCTTTGTAAGCACAAGCTGATGAGTACGAACTTATGTACTCATTCGTTTCGGAAGAGATAGGT
hCoV-19/India/HR-MDU-IGIB1210605800489930/2022_EPI... (26191)	ACTAGCGTGCCTTTGTAAGCACAAGCTGATGAGTACGAACTTATGTACTCATTCGTTTCGGAAGAGATAGGT
hCoV-19/England/PLYM-332B917/2022_EPI_ISL_9062229_... (26132)	ACTAGCGTGCCTTTGTAAGCACAAGCTGATGAGTACGAACTTATGTACTCATTCGTTTCGGAAGAGATAGGT
hCoV-19/Germany/HE-RKI-I-438397/2022_EPI_ISL_905522... (26140)	ACTAGCGTGCCTTTGTAAGCACAAGCTGATGAGTACGAACTTATGTACTCATTCGTTTCGGAAGAGATAGGT
hCoV-19/USA/DE-CDC-LC0472738/2021_EPI_ISL_9049423... (26110)	ACTAGCGTGCCTTTGTAAGCACAAGCTGATGAGTACGAACTTATGTACTCATTCGTTTCGGAAGAGATAGGT

Section 366

(26281)	26281 26290 26300 26310 26320 26330 26340 26352
SARS-CoV-2 Reference Genome NC_045512.2 (26275)	ACGTTAATAGTTAATAGCGTACTTCTTTTTCTTGCTTTCGTGGTATTCTTGCTAGTTACACTAGCCATCCTT
hCoV-19/Botswana/R69B55_BHP_916539/2021_EPI_ISL_90... (26221)	ACGTTAATAGTTAATAGCGTACTTCTTTTTCTTGCTTTCGTGGTATTCTTGCTAGTTACACTAGCCATCCTT
hCoV-19/India/HR-MDU-IGIB1210605800489930/2022_EPI... (26263)	ACGTTAATAGTTAATAGCGTACTTCTTTTTCTTGCTTTCGTGGTATTCTTGCTAGTTACACTAGCCATCCTT
hCoV-19/England/PLYM-332B917/2022_EPI_ISL_9062229_... (26204)	ACGTTAATAGTTAATAGCGTACTTCTTTTTCTTGCTTTCGTGGTATTCTTGCTAGTTACACTAGCCATCCTT
hCoV-19/Germany/HE-RKI-I-438397/2022_EPI_ISL_905522... (26212)	ACGTTAATAGTTAATAGCGTACTTCTTTTTCTTGCTTTCGTGGTATTCTTGCTAGTTACACTAGCCATCCTT
hCoV-19/USA/DE-CDC-LC0472738/2021_EPI_ISL_9049423... (26182)	ACGTTAATAGTTAATAGCGTACTTCTTTTTCTTGCTTTCGTGGTATTCTTGCTAGTTACACTAGCCATCCTT

Section 367

(26353)	26353 26360 26370 26380 26390 26400 26410 26424
SARS-CoV-2 Reference Genome NC_045512.2 (26347)	ACTGCGCTTCGATTGTGTGCGTACTGCTGCAATATTGTTAACGTGAGTCTTGTAAAACCTTCTTTTTACGTT
hCoV-19/Botswana/R69B55_BHP_916539/2021_EPI_ISL_90... (26293)	ACTGCGCTTCGATTGTGTGCGTACTGCTGCAATATTGTTAACGTGAGTCTTGTAAAACCTTCTTTTTACGTT
hCoV-19/India/HR-MDU-IGIB1210605800489930/2022_EPI... (26335)	ACTGCGCTTCGATTGTGTGCGTACTGCTGCAATATTGTTAACGTGAGTCTTGTAAAACCTTCTTTTTACGTT
hCoV-19/England/PLYM-332B917/2022_EPI_ISL_9062229_... (26276)	ACTGCGCTTCGATTGTGTGCGTACTGCTGCAATATTGTTAACGTGAGTCTTGTAAAACCTTCTTTTTACGTT
hCoV-19/Germany/HE-RKI-I-438397/2022_EPI_ISL_905522... (26284)	ACTGCGCTTCGATTGTGTGCGTACTGCTGCAATATTGTTAACGTGAGTCTTGTAAAACCTTCTTTTTACGTT
hCoV-19/USA/DE-CDC-LC0472738/2021_EPI_ISL_9049423... (26254)	ACTGCGCTTCGATTGTGTGCGTACTGCTGCAATATTGTTAACGTGAGTCTTGTAAAACCTTCTTTTTACGTT

Section 368

(26425)	26425 26430 26440 26450 26460 26470 26480 26496
SARS-CoV-2 Reference Genome NC_045512.2 (26419)	TACTCTCGTGTTAAAAATCTGAATTCTTCTAGAGTTCCTGATCTTCTGGTCTAAACGAACTAAATATTATAT
hCoV-19/Botswana/R69B55_BHP_916539/2021_EPI_ISL_90... (26365)	TACTCTCGTGTTAAAAATCTGAATTCTTCTAGAGTTCCTGATCTTCTGGTCTAAACGAACTAAATATTATAT
hCoV-19/India/HR-MDU-IGIB1210605800489930/2022_EPI... (26407)	TACTCTCGTGTTAAAAATCTGAATTCTTCTAGAGTTCCTGATCTTCTGGTCTAAACGAACTAAATATTATAT
hCoV-19/England/PLYM-332B917/2022_EPI_ISL_9062229_... (26348)	TACTCTCGTGTTAAAAATCTGAATTCTTCTAGAGTTCCTGATCTTCTGGTCTAAACGAACTAAATATTATAT
hCoV-19/Germany/HE-RKI-I-438397/2022_EPI_ISL_905522... (26356)	TACTCTCGTGTTAAAAATCTGAATTCTTCTAGAGTTCCTGATCTTCTGGTCTAAACGAACTAAATATTATAT
hCoV-19/USA/DE-CDC-LC0472738/2021_EPI_ISL_9049423... (26326)	TACTCTCGTGTTAAAAATCTGAATTCTTCTAGAGTTCCTGATCTTCTGGTCTAAACGAACTAAATATTATAT

Section 369

	(26497)	26497 26510 26520 26530 26540 26550 26568
SARS-CoV-2 Reference Genome NC_045512.2	(26491)	TAGTTTTTCTGTTTGGAACTTTAATTTTAGCCATGGCAGATTCCAACGGTACTATTACCGTTGAAGAGCTTA
hCoV-19/Botswana/R69B55_BHP_916539/2021_EPI_ISL_90...	(26437)	TAGTTTTTCTGTTTGGAACTTTAATTTTAGCCATGGCAGGTTCCAACGGTACTATTACCGTTGAAGAGCTTA
hCoV-19/India/HR-MDU-IGIB1210605800489930/2022_EPI...	(26479)	TAGTTTTTCTGTTTGGAACTTTAATTTTAGCCATGGCAGGTTCCAACGGTACTATTACCGTTGAAGAGCTTA
hCoV-19/England/PLYM-332B917/2022_EPI_ISL_9062229_...	(26420)	TAGTTTTTCTGTTTGGAACTTTAATTTTAGCCATGGCAGGTTCCAACGGTACTATTACCGTTGAAGAGCTTA
hCoV-19/Germany/HE-RKI-I-438397/2022_EPI_ISL_905522...	(26428)	TAGTTTTTCTGTTTGGAACTTTAATTTTAGCCATGGCAGGTTCCAACGGTACTATTACCGTTGAAGAGCTTA
hCoV-19/USA/DE-CDC-LC0472738/2021_EPI_ISL_9049423...	(26398)	TAGTTTTTCTGTTTGGAACTTTAATTTTAGCCATGGCAGGTTCCAACGGTACTATTACCGTTGAAGAGCTTA

Section 370

	(26569)	26569 26580 26590 26600 26610 26620 26630 26640
SARS-CoV-2 Reference Genome NC_045512.2	(26563)	AAAAGCTCCTTGAACAATGGAACCTAGTAATAGGTTTCCTATTCCTTACATGGATTTGTCTTCTACAATTTG
hCoV-19/Botswana/R69B55_BHP_916539/2021_EPI_ISL_90...	(26509)	AAAAGCTCCTTGAAGAATGGAACCTAGTAATAGGTTTCCTATTCCTTACATGGATTTGTCTTCTACAATTTG
hCoV-19/India/HR-MDU-IGIB1210605800489930/2022_EPI...	(26551)	AAAAGCTCCTTGAAGAATGGAACCTAGTAATAGGTTTCCTATTCCTTACATGGATTTGTCTTCTACAATTTG
hCoV-19/England/PLYM-332B917/2022_EPI_ISL_9062229_...	(26492)	AAAAGCTCCTTGAAGAATGGAACCTAGTAATAGGTTTCCTATTCCTTACATGGATTTGTCTTCTACAATTTG
hCoV-19/Germany/HE-RKI-I-438397/2022_EPI_ISL_905522...	(26500)	AAAAGCTCCTTGAAGAATGGAACCTAGTAATAGGTTTCCTATTCCTTACATGGATTTGTCTTCTACAATTTG
hCoV-19/USA/DE-CDC-LC0472738/2021_EPI_ISL_9049423...	(26470)	AAAAGCTCCTTGAAGAATGGAACCTAGTAATAGGTTTCCTATTCCTTACATGGATTTGTCTTCTACAATTTG

Section 371

	(26641)	26641 26650 26660 26670 26680 26690 26700 26712
SARS-CoV-2 Reference Genome NC_045512.2	(26635)	CCTATGCCAACAGGAATAGGTTTTTGTATATAATTAAGTTAATTTTCCTCTGGCTGTTATGGCCAGTAACTT
hCoV-19/Botswana/R69B55_BHP_916539/2021_EPI_ISL_90...	(26581)	CCTATGCCAACAGGAATAGGTTTTTGTATATAATTAAGTTAATTTTCCTCTGGCTGTTATGGCCAGTAACTT
hCoV-19/India/HR-MDU-IGIB1210605800489930/2022_EPI...	(26623)	CCTATGCCAACAGGAATAGGTTTTTGTATATAATTAAGTTAATTTTCCTCTGGCTGTTATGGCCAGTAACTT
hCoV-19/England/PLYM-332B917/2022_EPI_ISL_9062229_...	(26564)	CCTATGCCAACAGGAATAGGTTTTTGTATATAATTAAGTTAATTTTCCTCTGGCTGTTATGGCCAGTAACTT
hCoV-19/Germany/HE-RKI-I-438397/2022_EPI_ISL_905522...	(26572)	CCTATGCCAACAGGAATAGGTTTTTGTATATAATTAAGTTAATTTTCCTCTGGCTGTTATGGCCAGTAACTT
hCoV-19/USA/DE-CDC-LC0472738/2021_EPI_ISL_9049423...	(26542)	CCTATGCCAACAGGAATAGGTTTTTGTATATAATTAAGTTAATTTTCCTCTGGCTGTTATGGCCAGTAACTT

Section 372

	(26713)	26713 26720 26730 26740 26750 26760 26770 26784
SARS-CoV-2 Reference Genome NC_045512.2	(26707)	TAGCTTGTTTTGTGCTTGCTGCTGTTTACAGAATAAATTGGATCACCGGTGGAATTGCTATCGCAATGGCTT
hCoV-19/Botswana/R69B55_BHP_916539/2021_EPI_ISL_90...	(26653)	TAACTTGTTTTGTGCTTGCTGCTGTTTACAGAATAAATTGGATCACCGGTGGAATTGCTATCGCAATGGCTT
hCoV-19/India/HR-MDU-IGIB1210605800489930/2022_EPI...	(26695)	TAACTTGTTTTGTGCTTGCTGCTGTTTACAGAATAAATTGGATCACCGGTGGAATTGCTATCGCAATGGCTT
hCoV-19/England/PLYM-332B917/2022_EPI_ISL_9062229_...	(26636)	TAACTTGTTTTGTGCTTGCTGCTGTTTACAGAATAAATTGGATCACCGGTGGAATTGCTATCGCAATGGCTT
hCoV-19/Germany/HE-RKI-I-438397/2022_EPI_ISL_905522...	(26644)	TAACTTGTTTTGTGCTTGCTGCTGTTTACAGAATAAATTGGATCACCGGTGGAATTGCTATCGCAATGGCTT
hCoV-19/USA/DE-CDC-LC0472738/2021_EPI_ISL_9049423...	(26614)	TAACTTGTTTTGTGCTTGCTGCTGTTTACAGAATAAATTGGATCACCGGTGGAATTGCTATCGCAATGGCTT

Section 373

		26785 26790 26800 26810 26820 26830 26840 26856
	(26785)	
SARS-CoV-2 Reference Genome NC_045512.2	(26779)	GTCTTGTAGGCTTGATGTGGCTCAGCTACTTCATTGCTTCTTTCAGACTGTTTGCGCGTACGCGTTCCATGT
hCoV-19/Botswana/R69B55_BHP_916539/2021_EPI_ISL_90...	(26725)	GTCTTGTAGGCTTGATGTGGCTCAGCTACTTCATTGCTTCTTTCAGACTGTTTGCGCGTACGCGTTCCATGT
hCoV-19/India/HR-MDU-IGIB1210605800489930/2022_EPI...	(26767)	GTCTTGTAGGCTTGATGTGGCTCAGCTACTTCATTGCTTCTTTCAGACTGTTTGCGCGTACGCGTTCCATGT
hCoV-19/England/PLYM-332B917/2022_EPI_ISL_9062229_...	(26708)	GTCTTGTAGGCTTGATGTGGCTCAGCTACTTCATTGCTTCTTTCAGACTGTTTGCGCGTACGCGTTCCATGT
hCoV-19/Germany/HE-RKI-I-438397/2022_EPI_ISL_905522...	(26716)	GTCTTGTAGGCTTGATGTGGCTCAGCTACTTCATTGCTTCTTTCAGACTGTTTGCGCGTACGCGTTCCATGT
hCoV-19/USA/DE-CDC-LC0472738/2021_EPI_ISL_9049423...	(26686)	GTCTTGTAGGCTTGATGTGGCTCAGCTACTTCATTGCTTCTTTCAGACTGTTTGCGCGTACGCGTTCCATGT

Section 374

		26857 26870 26880 26890 26900 26910 26928
	(26857)	
SARS-CoV-2 Reference Genome NC_045512.2	(26851)	GGTCATTCAATCCAGAAACTAACATTCTTCTCAACGTGCCACTCCATGGCACTATTCTGACCAGACCGCTTC
hCoV-19/Botswana/R69B55_BHP_916539/2021_EPI_ISL_90...	(26797)	GGTCATTCAATCCAGAAACTAACATTCTTCTCAACGTGCCACTCCATGGCACTATTCTGACCAGACCGCTTC
hCoV-19/India/HR-MDU-IGIB1210605800489930/2022_EPI...	(26839)	GGTCATTCAATCCAGAAACTAACATTCTTCTCAACGTGCCACTCCATGGCACTATTCTGACCAGACCGCTTC
hCoV-19/England/PLYM-332B917/2022_EPI_ISL_9062229_...	(26780)	GGTCATTCAATCCAGAAACTAACATTCTTCTCAACGTGCCACTCCATGGCACTATTCTGACCAGACCGCTTC
hCoV-19/Germany/HE-RKI-I-438397/2022_EPI_ISL_905522...	(26788)	GGTCATTCAATCCAGAAACTAACATTCTTCTCAACGTGCCACTCCATGGCACTATTCTGACCAGACCGCTTC
hCoV-19/USA/DE-CDC-LC0472738/2021_EPI_ISL_9049423...	(26758)	GGTCATTCAATCCAGAAACTAACATTCTTCTCAACGTGCCACTCCATGGCACTATTCTGACCAGACCGCTTC

Section 375

		26929 26940 26950 26960 26970 26980 26990 27000
	(26929)	
SARS-CoV-2 Reference Genome NC_045512.2	(26923)	TAGAAAGTGAACTCGTAATCGGAGCTGTGATCCTTCGTGGACATCTTCGTATTGCTGGACACCATCTAGGAC
hCoV-19/Botswana/R69B55_BHP_916539/2021_EPI_ISL_90...	(26869)	TAGAAAGTGAACTCGTAATCGGAGCTGTGATCCTTCGTGGACATCTTCGTATTGCTGGACACCATCTAGGAC
hCoV-19/India/HR-MDU-IGIB1210605800489930/2022_EPI...	(26911)	TAGAAAGTGAACTCGTAATCGGAGCTGTGATCCTTCGTGGACATCTTCGTATTGCTGGACACCATCTAGGAC
hCoV-19/England/PLYM-332B917/2022_EPI_ISL_9062229_...	(26852)	TAGAAAGTGAACTCGTAATCGGAGCTGTGATCCTTCGTGGACATCTTCGTATTGCTGGACACCATCTAGGAC
hCoV-19/Germany/HE-RKI-I-438397/2022_EPI_ISL_905522...	(26860)	TAGAAAGTGAACTCGTAATCGGAGCTGTGATCCTTCGTGGACATCTTCGTATTGCTGGACACCATCTAGGAC
hCoV-19/USA/DE-CDC-LC0472738/2021_EPI_ISL_9049423...	(26830)	TAGAAAGTGAACTCGTAATCGGAGCTGTGATCCTTCGTGGACATCTTCGTATTGCTGGACACCATCTAGGAC

Section 376

		27001 27010 27020 27030 27040 27050 27060 27072
	(27001)	
SARS-CoV-2 Reference Genome NC_045512.2	(26995)	GCTGTGACATCAAGGACCTGCCTAAAGAAATCACTGTTGCTACATCACGAACGCTTTCTTATTACAAATTGG
hCoV-19/Botswana/R69B55_BHP_916539/2021_EPI_ISL_90...	(26941)	GCTGTGACATCAAGGACCTGCCTAAAGAAATCACTGTTGCTACATCACGAACGCTTTCTTATTACAAATTGG
hCoV-19/India/HR-MDU-IGIB1210605800489930/2022_EPI...	(26983)	GCTGTGACATCAAGGACCTGCCTAAAGAAATCACTGTTGCTACATCACGAACGCTTTCTTATTACAAATTGG
hCoV-19/England/PLYM-332B917/2022_EPI_ISL_9062229_...	(26924)	GCTGTGACATCAAGGACCTGCCTAAAGAAATCACTGTTGCTACATCACGAACGCTTTCTTATTACAAATTGG
hCoV-19/Germany/HE-RKI-I-438397/2022_EPI_ISL_905522...	(26932)	GCTGTGACATCAAGGACCTGCCTAAAGAAATCACTGTTGCTACATCACGAACGCTTTCTTATTACAAATTGG
hCoV-19/USA/DE-CDC-LC0472738/2021_EPI_ISL_9049423...	(26902)	GCTGTGACATCAAGGACCTGCCTAAAGAAATCACTGTTGCTACATCACGAACGCTTTCTTATTACAAATTGG

Omicron BA.1

Section 377

```
(27073)                                               27073  27080     27090     27100     27110     27120     27130     27144
SARS-CoV-2 Reference Genome NC_045512.2 (27067)             GAGCTTCGCAGCGTGTAGCAGGTGACTCAGGTTTTGCTGCATACAGTCGCTACAGGATTGGCAACTATAAAT
hCoV-19/Botswana/R69B55_BHP_916539/2021_EPI_ISL_90... (27013) GAGCTTCGCAGCGTGTAGCAGGTGACTCAGGTTTTGCTGCATACAGTCGCTACAGGATTGGCAACTATAAAT
hCoV-19/India/HR-MDU-IGIB1210605800489930/2022_EPI... (27055) GAGCTTCGCAGCGTGTAGCAGGTGACTCAGGTTTTGCTGCATACAGTCGCTACAGGATTGGCAACTATAAAT
hCoV-19/England/PLYM-332B917/2022_EPI_ISL_9062229_... (26996) GAGCTTCGCAGCGTGTAGCAGGTGACTCAGGTTTTGCTGCATACAGTCGCTACAGGATTGGCAACTATAAAT
hCoV-19/Germany/HE-RKI-I-438397/2022_EPI_ISL_905522... (27004) GAGCTTCGCAGCGTGTAGCAGGTGACTCAGGTTTTGCTGCATACAGTCGCTACAGGATTGGCAACTATAAAT
hCoV-19/USA/DE-CDC-LC0472738/2021_EPI_ISL_9049423... (26974) GAGCTTCGCAGCGTGTAGCAGGTGACTCAGGTTTTGCTGCATACAGTCGCTACAGGATTGGCAACTATAAAT
```

Section 378

```
(27145)                                               27145 27150     27160     27170     27180     27190     27200     27216
SARS-CoV-2 Reference Genome NC_045512.2 (27139)             TAAACACAGACCATTCCAGTAGCAGTGACAATATTGCTTTGCTTGTACAGTAAGTGACAACAGATGTTTCAT
hCoV-19/Botswana/R69B55_BHP_916539/2021_EPI_ISL_90... (27085) TAAACACAGACCATTCCAGTAGCAGTGACAATATTGCTTTGCTTGTACAGTAAGTGACAACAGATGTTTCAT
hCoV-19/India/HR-MDU-IGIB1210605800489930/2022_EPI... (27127) TAAACACAGACCATTCCAGTAGCAGTGACAATATTGCTTTGCTTGTACAGTAAGTGACAACAGATGTTTCAT
hCoV-19/England/PLYM-332B917/2022_EPI_ISL_9062229_... (27068) TAAACACAGACCATTCCAGTAGCAGTGACAATATTGCTTTGCTTGTACAGTAAGTGACAACAGATGTTTCAT
hCoV-19/Germany/HE-RKI-I-438397/2022_EPI_ISL_905522... (27076) TAAACACAGACCATTCCAGTAGCAGTGACAATATTGCTTTGCTTGTACAGTAAGTGACAACAGATGTTTCAT
hCoV-19/USA/DE-CDC-LC0472738/2021_EPI_ISL_9049423... (27046) TAAACACAGACCATTCCAGTAGCAGTGACAATATTGCTTTGCTTGTACAGTAAGTGACAACAGATGTTTCAT
```

Section 379

```
(27217)                                               27217      27230     27240     27250     27260     27270          27288
SARS-CoV-2 Reference Genome NC_045512.2 (27211)             CTCGTTGACTTTCAGGTTACTATAGCAGAGATATTACTAATTATTATGAGGACTTTTAAAGTTTCCATTTGG
hCoV-19/Botswana/R69B55_BHP_916539/2021_EPI_ISL_90... (27157) CTCGTTGACTTTCAGGTTACTATAGCAGAGATATTACTAATTATTATGCGGACTTTTAAAGTTTCCATTTGG
hCoV-19/India/HR-MDU-IGIB1210605800489930/2022_EPI... (27199) CTCGTTGACTTTCAGGTTACTATAGCAGAGATATTACTAATTATTATGCGGACTTTTAAAGTTTCCATTTGG
hCoV-19/England/PLYM-332B917/2022_EPI_ISL_9062229_... (27140) CTCGTTGACTTTCAGGTTACTATAGCAGAGATATTACTAATTATTATGCGGACTTTTAAAGTTTCCATTTGG
hCoV-19/Germany/HE-RKI-I-438397/2022_EPI_ISL_905522... (27148) CTCGTTGACTTTCAGGTTACTATAGCAGAGATATTACTAATTATTATGCGGACTTTTAAAGTTTCCATTTGG
hCoV-19/USA/DE-CDC-LC0472738/2021_EPI_ISL_9049423... (27118) CTCGTTGACTTTCAGGTTACTATAGCAGAGATATTACTAATTATTATGCGGACTTTTAAAGTTTCCATTTGG
```

Section 380

```
(27289)                                               27289     27300     27310     27320     27330     27340     27350  27360
SARS-CoV-2 Reference Genome NC_045512.2 (27283)             AATCTTGATTACATCATAAACCTCATAATTAAAAATTTATCTAAGTCACTAACTGAGAATAAATATTCTCAA
hCoV-19/Botswana/R69B55_BHP_916539/2021_EPI_ISL_90... (27229) AATCTTGATTACATCATAAACCTCATAATTAAAAATTTATCTAAGTCACTAACTGAGAATAAATATTCTCAA
hCoV-19/India/HR-MDU-IGIB1210605800489930/2022_EPI... (27271) AATCTTGATTACATCATAAACCTCATAATTAAAAATTTATCTAAGTCACTAACTGAGAATAAATATTCTCAA
hCoV-19/England/PLYM-332B917/2022_EPI_ISL_9062229_... (27212) AATCTTGATTACATCATAAACCTCATAATTAAAAATTTATCTAAGTCACTAACTGAGAATAAATATTCTCAA
hCoV-19/Germany/HE-RKI-I-438397/2022_EPI_ISL_905522... (27220) AATCTTGATTACATCATAAACCTCATAATTAAAAATTTATCTAAGTCACTAACTGAGAATAAATATTCTCAA
hCoV-19/USA/DE-CDC-LC0472738/2021_EPI_ISL_9049423... (27190) AATCTTGATTACATCATAAACCTCATAATTAAAAATTTATCTAAGTCACTAACTGAGAATAAATATTCTCAA
```

```
                                                               Section 381
                                                  (27361) 27361    27370     27380     27390     27400     27410     27420   27432
          SARS-CoV-2 Reference Genome NC_045512.2 (27355) TTAGATGAAGAGCAACCAATGGAGATTGATTAAACGAACATGAAAATTATTCTTTTCTTGGCACTGATAACA
hCoV-19/Botswana/R69B55_BHP_916539/2021_EPI_ISL_90... (27301) TTAGATGAAGAGCAACCAATGGAGATTGATTAAACGAACATGAAAATTATTCTTTTCTTGGCACTGATAACA
hCoV-19/India/HR-MDU-IGIB1210605800489930/2022_EPI... (27343) TTAGATGAAGAGCAACCAATGGAGATTGATTAAACGAACATGAAAATTATTCTTTTCTTGGCACTGATAACA
hCoV-19/England/PLYM-332B917/2022_EPI_ISL_9062229_... (27284) TTAGATGAAGAGCAACCAATGGAGATTGATTAAACGAACATGAAAATTATTCTTTTCTTGGCACTGATAACA
hCoV-19/Germany/HE-RKI-I-438397/2022_EPI_ISL_905522... (27292) TTAGATGAAGAGCAACCAATGGAGATTGATTAAACGAACATGAAAATTATTCTTTTCTTGGCACTGATAACA
hCoV-19/USA/DE-CDC-LC0472738/2021_EPI_ISL_9049423... (27262) TTAGATGAAGAGCAACCAATGGAGATTGATTAAACGAACATGAAAATTATTCTTTTCTTGGCACTGATAACA
```

```
                                                               Section 382
                                                  (27433) 27433 27440    27450     27460     27470     27480     27490     27504
          SARS-CoV-2 Reference Genome NC_045512.2 (27427) CTCGCTACTTGTGAGCTTTATCACTACCAAGAGTGTGTTAGAGGTACAACAGTACTTTTAAAAGAACCTTGC
hCoV-19/Botswana/R69B55_BHP_916539/2021_EPI_ISL_90... (27373) CTCGCTACTTGTGAGCTTTATCACTACCAAGAGTGTGTTAGAGGTACAACAGTACTTTTAAAAGAACCTTGC
hCoV-19/India/HR-MDU-IGIB1210605800489930/2022_EPI... (27415) CTCGCTACTTGTGAGCTTTATCACTACCAAGAGTGTGTTAGAGGTACAACAGTACTTTTAAAAGAACCTTGC
hCoV-19/England/PLYM-332B917/2022_EPI_ISL_9062229_... (27356) CTCGCTACTTGTGAGCTTTATCACTACCAAGAGTGTGTTAGAGGTACAACAGTACTTTTAAAAGAACCTTGC
hCoV-19/Germany/HE-RKI-I-438397/2022_EPI_ISL_905522... (27364) CTCGCTACTTGTGAGCTTTATCACTACCAAGAGTGTGTTAGAGGTACAACAGTACTTTTAAAAGAACCTTGC
hCoV-19/USA/DE-CDC-LC0472738/2021_EPI_ISL_9049423... (27334) CTCGCTACTTGTGAGCTTTATCACTACCAAGAGTGTGTTAGAGGTACAACAGTACTTTTAAAAGAACCTTGC
```

```
                                                               Section 383
                                                  (27505) 27505 27510     27520     27530     27540     27550     27560     27576
          SARS-CoV-2 Reference Genome NC_045512.2 (27499) TCTTCTGGAACATACGAGGGCAATTCACCATTTCATCCTCTAGCTGATAACAAATTTGCACTGACTTGCTTT
hCoV-19/Botswana/R69B55_BHP_916539/2021_EPI_ISL_90... (27445) TCTTCTGGAACATACGAGGGCAATTCACCATTTCATCCTCTAGCTGATAACAAATTTGCACTGACTTGCTTT
hCoV-19/India/HR-MDU-IGIB1210605800489930/2022_EPI... (27487) TCTTCTGGAACATACGAGGGCAATTCACCATTTCATCCTCTAGCTGATAACAAATTTGCACTGACTTGCTTT
hCoV-19/England/PLYM-332B917/2022_EPI_ISL_9062229_... (27428) TCTTCTGGAACATACGAGGGCAATTCACCATTTCATCCTCTAGCTGATAACAAATTTGCACTGACTTGCTTT
hCoV-19/Germany/HE-RKI-I-438397/2022_EPI_ISL_905522... (27436) TCTTCTGGAACATACGAGGGCAATTCACCATTTCATCCTCTAGCTGATAACAAATTTGCACTGACTTGCTTT
hCoV-19/USA/DE-CDC-LC0472738/2021_EPI_ISL_9049423... (27406) TCTTCTGGAACATACGAGGGCAATTCACCATTTCATCCTCTAGCTGATAACAAATTTGCACTGACTTGCTTT
```

```
                                                               Section 384
                                                  (27577) 27577        27590     27600     27610     27620     27630         27648
          SARS-CoV-2 Reference Genome NC_045512.2 (27571) AGCACTCAATTTGCTTTTGCTTGTCCTGACGGCGTAAAACACGTCTATCAGTTACGTGCCAGATCAGTTTCA
hCoV-19/Botswana/R69B55_BHP_916539/2021_EPI_ISL_90... (27517) AGCACTCAATTTGCTTTTGCTTGTCCTGACGGCGTAAAACACGTCTATCAGTTACGTGCCAGATCAGTTTCA
hCoV-19/India/HR-MDU-IGIB1210605800489930/2022_EPI... (27559) AGCACTCAATTTGCTTTTGCTTGTCCTGACGGCGTAAAACACGTCTATCAGTTACGTGCCAGATCAGTTTCA
hCoV-19/England/PLYM-332B917/2022_EPI_ISL_9062229_... (27500) AGCACTCAATTTGCTTTTGCTTGTCCTGACGGCGTAAAACACGTCTATCAGTTACGTGCCAGATCAGTTTCA
hCoV-19/Germany/HE-RKI-I-438397/2022_EPI_ISL_905522... (27508) AGCACTCAATTTGCTTTTGCTTGTCCTGACGGCGTAAAACACGTCTATCAGTTACGTGCCAGATCAGTTTCA
hCoV-19/USA/DE-CDC-LC0472738/2021_EPI_ISL_9049423... (27478) AGCACTCAATTTGCTTTTGCTTGTCCTGACGGCGTAAAACACGTCTATCAGTTACGTGCCAGATCAGTTTCA
```

```
                                                                                   Section 385
                                                          (27649) 27649      27660     27670     27680     27690     27700     27710  27720
           SARS-CoV-2 Reference Genome NC_045512.2 (27643) CCTAAACTGTTCATCAGACAAGAGGAAGTTCAAGAACTTTACTCTCCAATTTTTCTTATTGTTGCGGCAATA
  hCoV-19/Botswana/R69B55_BHP_916539/2021_EPI_ISL_90... (27589) CCTAAACTGTTCATCAGACAAGAGGAAGTTCAAGAACTTTACTCTCCAATTTTTCTTATTGTTGCGGCAATA
  hCoV-19/India/HR-MDU-IGIB1210605800489930/2022_EPI... (27631) CCTAAACTGTTCATCAGACAAGAGGAAGTTCAAGAACTTTACTCTCCAATTTTTCTTATTGTTGCGGCAATA
   hCoV-19/England/PLYM-332B917/2022_EPI_ISL_9062229 ... (27572) CCTAAACTGTTCATCAGACAAGAGGAAGTTCAAGAACTTTACTCTCCAATTTTTCTTATTGTTGCGGCAATA
  hCoV-19/Germany/HE-RKI-I-438397/2022_EPI_ISL_905522... (27580) CCTAAACTGTTCATCAGACAAGAGGAAGTTCAAGAACTTTACTCTCCAATTTTTCTTATTGTTGCGGCAATA
   hCoV-19/USA/DE-CDC-LC0472738/2021_EPI_ISL_9049423... (27550) CCTAAACTGTTCATCAGACAAGAGGAAGTTCAAGAACTTTACTCTCCAATTTTTCTTATTGTTGCGGCAATA
                                                                                   Section 386
                                                          (27721) 27721    27730     27740     27750     27760     27770     27780    27792
           SARS-CoV-2 Reference Genome NC_045512.2 (27715) GTGTTTATAACACTTTGCTTCACACTCAAAAGAAAGACAGAATGATTGAACTTTCATTAATTGACTTCTATT
  hCoV-19/Botswana/R69B55_BHP_916539/2021_EPI_ISL_90... (27661) GTGTTTATAACACTTTGCTTCACACTCAAAAGAAAGACAGAATGATTGAACTTTCATTAATTGACTTCTATT
  hCoV-19/India/HR-MDU-IGIB1210605800489930/2022_EPI... (27703) GTGTTTATAACACTTTGCTTCACACTCAAAAGAAAGACAGAATGATTGAACTTTCATTAATTGACTTCTATT
   hCoV-19/England/PLYM-332B917/2022_EPI_ISL_9062229 ... (27644) GTGTTTATAACACTTTGCTTCACACTCAAAAGAAAGACAGAATGATTGAACTTTCATTAATTGACTTCTATT
  hCoV-19/Germany/HE-RKI-I-438397/2022_EPI_ISL_905522... (27652) GTGTTTATAACACTTTGCTTCACACTCAAAAGAAAGACAGAATGATTGAACTTTCATTAATTGACTTCTATT
   hCoV-19/USA/DE-CDC-LC0472738/2021_EPI_ISL_9049423... (27622) GTGTTTATAACACTTTGCTTCACACTCAAAAGAAAGACAGAATGATTGAACTTTCATTAATTGACTTCTATT
                                                                                   Section 387
                                                          (27793) 27793   27800     27810     27820     27830     27840     27850      27864
           SARS-CoV-2 Reference Genome NC_045512.2 (27787) TGTGCTTTTTAGCCTTTCTGCTATTCCTTGTTTTAATTATGCTTATTATCTTTTGGTTCTCACTTGAACTGC
  hCoV-19/Botswana/R69B55_BHP_916539/2021_EPI_ISL_90... (27733) TGTGCTTTTTAGCCTTTCTGTTATTCCTTGTTTTAATTATGCTTATTATCTTTTGGTTCTCACTTGAACTGC
  hCoV-19/India/HR-MDU-IGIB1210605800489930/2022_EPI... (27775) TGTGCTTTTTAGCCTTTCTGTTATTCCTTGTTTTAATTATGCTTATTATCTTTTGGTTCTCACTTGAACTGC
   hCoV-19/England/PLYM-332B917/2022_EPI_ISL_9062229 ... (27716) TGTGCTTTTTAGCCTTTCTGTTATTCCTTGTTTTAATTATGCTTATTATCTTTTGGTTCTCACTTGAACTGC
  hCoV-19/Germany/HE-RKI-I-438397/2022_EPI_ISL_905522... (27724) TGTGCTTTTTAGCCTTTCTGTTATTCCTTGTTTTAATTATGCTTATTATCTTTTGGTTCTCACNNNNNNNNN
   hCoV-19/USA/DE-CDC-LC0472738/2021_EPI_ISL_9049423... (27694) TGTGCTTTTTAGCCTTTCTGTTATTCCTTGTTTTAATTATGCTTATTATCTTTTGGTTCTCACTTGAACTGC
                                                                                   Section 388
                                                          (27865) 27865 27870     27880     27890     27900     27910     27920      27936
           SARS-CoV-2 Reference Genome NC_045512.2 (27859) AAGATCATAATGAAACTTGTCACGCCTAAACGAACATGAAATTTCTTGTTTTCTTAGGAATCATCACAACTG
  hCoV-19/Botswana/R69B55_BHP_916539/2021_EPI_ISL_90... (27805) AAGATCATAATGAANNNNNNNNNNNNNNAACGAACATGAAATTTCTTGTTTTCTTAGGAATCATCACAACTG
  hCoV-19/India/HR-MDU-IGIB1210605800489930/2022_EPI... (27847) AAGATCATAATGAAACTTGTCACGCCTAAACGAACATGAAATTTCTTGTTTTCTTAGGAATCATCACAACTG
   hCoV-19/England/PLYM-332B917/2022_EPI_ISL_9062229 ... (27788) AAGATCATAATGAAACTTGTCACGCCTAAACGAACATGAAATTTCTTGTTTTCTTAGGAATCATCACAACTG
  hCoV-19/Germany/HE-RKI-I-438397/2022_EPI_ISL_905522... (27796) NNNNNNNNNNNNNNNNNNNNNNNNNNNNNNNNNNNNNNNNNNNNNNNNNNNNNNNNNNNNNNNNNNNNNNNN
   hCoV-19/USA/DE-CDC-LC0472738/2021_EPI_ISL_9049423... (27766) AAGATCATAATGAAACTTGTCACGCCTAAACGAACATGAAATTTCTTGTTTTCTTAGGAATCATCACAACTG
```

Omicron BA.1

Section 389

```
                                                                      27937                                                                  28008
SARS-CoV-2 Reference Genome NC_045512.2 (27931)                       TAGCTGCATTTCACCAAGAATGTAGTTTACAGTCATGTACTCAACATCAACCATATGTAGTTGATGACCCGT
hCoV-19/Botswana/R69B55_BHP_916539/2021_EPI_ISL_90... (27877)         TAGCTGCATTTCACCAAGAATGTAGTTTACAGTCATGTACTCAACATCAACCATATGTAGTTGATGACCCGT
hCoV-19/India/HR-MDU-IGIB1210605800489930/2022_EPI... (27919)         TAGCTGCATTTCACCAAGAATGTAGTTTACAGTCATGTACTCAACATCAACCATATGTAGTTGATGACCCGT
hCoV-19/England/PLYM-332B917/2022_EPI_ISL_9062229_... (27860)         TAGCTGCATTTCACCAAGAATGTAGTTTACAGTCATGTACTCAACATCAACCATATGTAGTTGATGACCCGT
hCoV-19/Germany/HE-RKI-I-438397/2022_EPI_ISL_905522... (27868)        NNNNNNNNNNNNNNNNNNNNNNTAGTTTACAGTCATGTACTCAACNNNNNCCATATGTNNNNNNNNNNNNNN
hCoV-19/USA/DE-CDC-LC0472738/2021_EPI_ISL_9049423... (27838)          TAGCTGCATTTCACCAAGAATGTAGTTTACAGTCATGTACTCAACATCAACCATATGTAGTTGATGACCCGT
```

Section 390

```
                                                                      28009                                                                  28080
SARS-CoV-2 Reference Genome NC_045512.2 (28003)                       GTCCTATTCACTTCTATTCTAAATGGTATATTAGAGTAGGAGCTAGAAAATCAGCACCTTTAATTGAATTGT
hCoV-19/Botswana/R69B55_BHP_916539/2021_EPI_ISL_90... (27949)         GTCCTATTCACTTCTATTCTAAATGGTATATTAGAGTAGGAGCTAGAAAATCAGCACCTTTAATTGAATTGT
hCoV-19/India/HR-MDU-IGIB1210605800489930/2022_EPI... (27991)         GTCCTATTCACTTCTATTCTAAATGGTATATTAGAGTAGGAGCTAGAAAATCAGCACCTTTAATTGAATTGT
hCoV-19/England/PLYM-332B917/2022_EPI_ISL_9062229_... (27932)         GTCCTATTCACTTCTATTCTAAATGGTATATTAGAGTAGGAGCTAGAAAATCAGCACCTTTAATTGAATTGT
hCoV-19/Germany/HE-RKI-I-438397/2022_EPI_ISL_905522... (27940)        NNNNTATTCACTNNNNNNNNNNNNNNNNNNNNNNNNNNNNNNNNNNNNNNNNNNNNNNNNNNNNNNNNNNNN
hCoV-19/USA/DE-CDC-LC0472738/2021_EPI_ISL_9049423... (27910)          GTCCTATTCACTTCTATTCTAAATGGTATATTAGAGTAGGAGCTAGAAAATCAGCACCTTTAATTGAATTGT
```

Section 391

```
                                                                      28081                                                                  28152
SARS-CoV-2 Reference Genome NC_045512.2 (28075)                       GCGTGGATGAGGCTGGTTCTAAATCACCCATTCAGTACATCGATATCGGTAATTATACAGTTTCCTGTTTAC
hCoV-19/Botswana/R69B55_BHP_916539/2021_EPI_ISL_90... (28021)         GCGTGGATGAGGCTGGTTCTAAATCACCCATTCAGTACATCGATATCGGTAATTATACAGTTTCCTGTTTAC
hCoV-19/India/HR-MDU-IGIB1210605800489930/2022_EPI... (28063)         GCGTGGATGAGGCTGGTTCTAAATCACCCATTCAGTACATCGATATCGGTAATTATACAGTTTCCTGTTTAC
hCoV-19/England/PLYM-332B917/2022_EPI_ISL_9062229_... (28004)         GCGTGGATGAGGCTGGTTCTAAATCACCCATTCAGTACATCGATATCGGTAATTATACAGTTTCCTGTTTAC
hCoV-19/Germany/HE-RKI-I-438397/2022_EPI_ISL_905522... (28012)        NNNTGGATGAGGCNGGTTCTAAATCACCCATTCAGTACATCGATATCGGTAATTATACAGTTTCCTGTTTAC
hCoV-19/USA/DE-CDC-LC0472738/2021_EPI_ISL_9049423... (27982)          GCGTGGATGAGGCTGGTTCTAAATCACCCATTCAGTACATCGATATCGGTAATTATACAGTTTCCTGTTTAC
```

Section 392

```
                                                                      28153                                                                  28224
SARS-CoV-2 Reference Genome NC_045512.2 (28147)                       CTTTTACAATTAATTGCCAGGAACCTAAATTGGGTAGTCTTGTAGTGCGTTGTTCGTTCTATGAAGACTTTT
hCoV-19/Botswana/R69B55_BHP_916539/2021_EPI_ISL_90... (28093)         CTTTTACAATTAATTGCCAGGAACCTAAATTGGGTAGTCTTGTAGTGCGTTGTTCGTTCTATGAAGACTTTT
hCoV-19/India/HR-MDU-IGIB1210605800489930/2022_EPI... (28135)         CTTTTACAATTAATTGCCAGGAACCTAAATTGGGTAGTCTTGTAGTGCGTTGTTCGTTCTATGAAGACTTTT
hCoV-19/England/PLYM-332B917/2022_EPI_ISL_9062229_... (28076)         CTTTTACAATTAATTGCCAGGAACCTAAATTGGGTAGTCTTGTAGTGCGTTGTTCGTTCTATGAAGACTTTT
hCoV-19/Germany/HE-RKI-I-438397/2022_EPI_ISL_905522... (28084)        CTTTTACAATTAATTGCCAGGAACCTAAATTGGGTAGTCTTGTAGTGCGTTGTTCGTTCTATGAAGACTTTT
hCoV-19/USA/DE-CDC-LC0472738/2021_EPI_ISL_9049423... (28054)          CTTTTACAATTAATTGCCAGGAACCTAAATTGGGTAGTCTTGTAGTGCGTTGTTCGTTCTATGAAGACTTTT
```

Section 393

```
                                               (28225) 28225 28230     28240     28250     28260     28270     28280     28296
SARS-CoV-2 Reference Genome NC_045512.2        (28219) TAGAGTATCATGACGTTCGTGTTGTTTTAGATTTCATCTAAACGAACAAACTAAAATGTCTGATAATGGACC
hCoV-19/Botswana/R69B55_BHP_916539/2021_EPI_ISL_90... (28165) TAGAGTATCATGACGTTCGTGTTGTTTTAGATTTCATCTAAACGAACAAACTTAAATGTCTGATAATGGACC
hCoV-19/India/HR-MDU-IGIB1210605800489930/2022_EPI... (28207) TAGAGTATCATGACGTTCGTGTTGTTTTAGATTTCATCTAAACGAACAAACTTAAATGTCTGATAATGGACC
hCoV-19/England/PLYM-332B917/2022_EPI_ISL_9062229 ... (28148) TAGAGTATCATGACGTTCGTGTTGTTTTAGATTTCATCTAAACGAACAAACTTAAATGTCTGATAATGGACC
hCoV-19/Germany/HE-RKI-I-438397/2022_EPI_ISL_905522... (28156) TAGAGTATCATGACGTTCGTGTTGTTTTAGATTTCATCTAAACGAACAAACTTAAATGTCTGATAATGGACC
hCoV-19/USA/DE-CDC-LC0472738/2021_EPI_ISL_9049423... (28126) TAGAGTATCATGACGTTCGTGTTGTTTTAGATTTCATCTAAACGAACAAACTTAAATGTCTGATAATGGACC
```

Section 394

```
                                               (28297) 28297          28310     28320     28330     28340     28350          28368
SARS-CoV-2 Reference Genome NC_045512.2        (28291) CCAAAATCAGCGAAATGCACCCCGCATTACGTTTGGTGGACCCTCAGATTCAACTGGCAGTAACCAGAATGG
hCoV-19/Botswana/R69B55_BHP_916539/2021_EPI_ISL_90... (28237) CCAAAATCAGCGAAATGCACTCCGCATTACGTTTGGTGGACCCTCAGATTCAACTGGCAGTAACCAGAATGN
hCoV-19/India/HR-MDU-IGIB1210605800489930/2022_EPI... (28279) CCAAAATCAGCGAAATGCACCCCGCATTACGTTTGGTGGACCCTCAGATTCAACTGGCAGTAACCAGAATGG
hCoV-19/England/PLYM-332B917/2022_EPI_ISL_9062229 ... (28220) CCAAAATCAGCGAAATGCACTCCGCATTACGTTTGGTGGACCCTCAGATTCAACTGGCAGTAACCAGAATGG
hCoV-19/Germany/HE-RKI-I-438397/2022_EPI_ISL_905522... (28228) CCAAAATCAGCGAAATGCACTCCGCATTACGTTTGGTGGACCCTCAGATTCAACTGGCAGTAACCAGAATGG
hCoV-19/USA/DE-CDC-LC0472738/2021_EPI_ISL_9049423... (28198) CCAAAATCAGCGAAATGCACTCCGCATTACGTTTGGTGGACCCTCAGATTCAACTGGCAGTAACCAGAATGG
```

Section 395

```
                                               (28369) 28369       28380     28390     28400     28410     28420     28430   28440
SARS-CoV-2 Reference Genome NC_045512.2        (28363) AGAACGCAGTGGGGCGCGATCAAAACAACGTCGGCCCCAAGGTTTACCCAATAATACTGCGTCTTGGTTCAC
hCoV-19/Botswana/R69B55_BHP_916539/2021_EPI_ISL_90... (28309) NNNNNNNNGTGGGGCGCGATCAAAACAACGTCGGCCCCAAGGTTTACCCAATAATACTGCGTCTTGGTTCAC
hCoV-19/India/HR-MDU-IGIB1210605800489930/2022_EPI... (28351) AGAACGCAGTGGGGCGCGATCAAAACAACGTCGGCCCCAAGGTTTACCCAATAATACTGCGTCTTGGTTCAC
hCoV-19/England/PLYM-332B917/2022_EPI_ISL_9062229 ... (28292) ---------TGGGGCGCGATCAAAACAACGTCGGCCCCAAGGTTTACCCAATAATACTGCGTCTTGGTTCAC
hCoV-19/Germany/HE-RKI-I-438397/2022_EPI_ISL_905522... (28300) ---------TGGGGCGCGATCAAAACAACGTCGGCCCCAAGGTTTACCCAATAATACTGCGTCTTGGTTCAC
hCoV-19/USA/DE-CDC-LC0472738/2021_EPI_ISL_9049423... (28270) ---------TGGGGCGCGATCAAAACAACGTCGGCCCCAAGGTTTACCCAATAATACTGCGTCTTGGTTCAC
```

Section 396

```
                                               (28441) 28441    28450     28460     28470     28480     28490     28500    28512
SARS-CoV-2 Reference Genome NC_045512.2        (28435) CGCTCTCACTCAACATGGCAAGGAAGACCTTAAATTCCCTCGAGGACAAGGCGTTCCAATTAACACCAATAG
hCoV-19/Botswana/R69B55_BHP_916539/2021_EPI_ISL_90... (28381) CGCTCTCACTCAACATGGCAAGGAAGACCTTAAATTCCCTCGAGGACAAGGCGTTCCAATTAACACCAATAG
hCoV-19/India/HR-MDU-IGIB1210605800489930/2022_EPI... (28423) CGCTCTCACTCAACATGGCAAGGAAGGCCTTAAATTCCCTCGAGGACAAGGCGTTCCAATTAACACCAATAG
hCoV-19/England/PLYM-332B917/2022_EPI_ISL_9062229 ... (28355) CGCTCTCACTCAACATGGCAAGGAAGACCTTAAATTCCCTCGAGGACAAGGCGTTCCAATTAACACCAATAG
hCoV-19/Germany/HE-RKI-I-438397/2022_EPI_ISL_905522... (28363) CGCTCTCACTCAACATGGCAAGGAAGACCTTAAATTCCCTCGAGGACAAGGCGTTCCAATTAACACCAATAG
hCoV-19/USA/DE-CDC-LC0472738/2021_EPI_ISL_9049423... (28333) CGCTCTCACTCAACATGGCAAGGAAGACCTTAAATTCCCTCGAGGACAAGGCGTTCCAATTAACACCAATAG
```

Section 397

(28513)	28513 28520 28530 28540 28550 28560 28570 28584
SARS-CoV-2 Reference Genome NC_045512.2 (28507)	CAGTCCAGATGACCAAATTGGCTACTACCGAAGAGCTACCAGACGAATTCGTGGTGGTGACGGTAAAATGAA
hCoV-19/Botswana/R69B55_BHP_916539/2021_EPI_ISL_90... (28453)	CAGTCCAGATGACCAAATTGGCTACTACCGAAGAGCTACCAGACGAATTCGTGGTGGTGACGGTAAAATGAA
hCoV-19/India/HR-MDU-IGIB1210605800489930/2022_EPI... (28495)	CAGTCCAGATGACCAAATTGGCTACTACCGAAGAGCTACCAGACGAATTCGTGGTGGTGACGGTAAAATGAA
hCoV-19/England/PLYM-332B917/2022_EPI_ISL_9062229 ... (28427)	CAGTCCAGATGACCAAATTGGCTACTACCGAAGAGCTACCAGACGAATTCGTGGTGGTGACGGTAAAATGAA
hCoV-19/Germany/HE-RKI-I-438397/2022_EPI_ISL_905522... (28435)	CAGTCCAGATGACCAAATTGGCTACTACCGAAGAGCTACCAGACGAATTCGTGGTGGTGACGGTAAAATGAA
hCoV-19/USA/DE-CDC-LC0472738/2021_EPI_ISL_9049423... (28405)	CAGTCCAGATGACCAAATTGGCTACTACCGAAGAGCTACCAGACGAATTCGTGGTGGTGACGGTAAAATGAA

Section 398

(28585)	28585 28590 28600 28610 28620 28630 28640 28656
SARS-CoV-2 Reference Genome NC_045512.2 (28579)	AGATCTCAGTCCAAGATGGTATTTCTACTACCTAGGAACTGGGCCAGAAGCTGGACTTCCCTATGGTGCTAA
hCoV-19/Botswana/R69B55_BHP_916539/2021_EPI_ISL_90... (28525)	AGATCTCAGTCCAAGATGGTATTTCTACTACCTAGGAACTGGGCCAGAAGCTGGACTTCCCTATGGTGCTAA
hCoV-19/India/HR-MDU-IGIB1210605800489930/2022_EPI... (28567)	AGATCTCAGTCCAAGATGGTATTTCTACTACCTAGGAACTGGGCCAGAAGCTGGACTTCCCTATGGTGCTAA
hCoV-19/England/PLYM-332B917/2022_EPI_ISL_9062229 ... (28499)	AGATCTCAGTCCAAGATGGTATTTCTACTACCTAGGAACTGGGCCAGAAGCTGGACTTCCCTATGGTGCTAA
hCoV-19/Germany/HE-RKI-I-438397/2022_EPI_ISL_905522... (28507)	AGATCTCAGTCCAAGATGGTATTTCTACTACCTAGGAACTGGGCCAGAAGCTGGACTTCCCTATGGTGCTAA
hCoV-19/USA/DE-CDC-LC0472738/2021_EPI_ISL_9049423... (28477)	AGATCTCAGTCCAAGATGGTATTTCTACTACCTAGGAACTGGGCCAGAAGCTGGACTTCCCTATGGTGCTAA

Section 399

(28657)	28657 28670 28680 28690 28700 28710 28728
SARS-CoV-2 Reference Genome NC_045512.2 (28651)	CAAAGACGGCATCATATGGGTTGCAACTGAGGGAGCCTTGAATACACCAAAAGATCACATTGGCACCCGCAA
hCoV-19/Botswana/R69B55_BHP_916539/2021_EPI_ISL_90... (28597)	CAAAGACGGCATCATATGGGTTGCAACTGAGGGAGCCTTGAATACACCAAAAGATCACATTGGCACCCGCAA
hCoV-19/India/HR-MDU-IGIB1210605800489930/2022_EPI... (28639)	CAAAGACGGCATCATATGGGTTGCAACTGAGGGAGCCTTGAATACACCAAAAGATCACATTGGCACCCGCAA
hCoV-19/England/PLYM-332B917/2022_EPI_ISL_9062229 ... (28571)	CAAAGACGGCATCATATGGGTTGCAACTGAGGGAGCCTTGAATACACCAAAAGATCACATTGGCACCCGCAA
hCoV-19/Germany/HE-RKI-I-438397/2022_EPI_ISL_905522... (28579)	CAAAGACGGCATCATATGGGTTGCAACTGAGGGAGCCTTGAATACACCAAAAGATCACATTGGCACCCGCAA
hCoV-19/USA/DE-CDC-LC0472738/2021_EPI_ISL_9049423... (28549)	CAAAGACGGCATCATATGGGTTGCAACTGAGGGAGCCTTGAATACACCAAAAGATCATATTGGCACCCGCAA

Section 400

(28729)	28729 28740 28750 28760 28770 28780 28790 28800
SARS-CoV-2 Reference Genome NC_045512.2 (28723)	TCCTGCTAACAATGCTGCAATCGTGCTACAACTTCCTCAAGGAACAACATTGCCAAAAGGCTTCTACGCAGA
hCoV-19/Botswana/R69B55_BHP_916539/2021_EPI_ISL_90... (28669)	TCCTGCTAACAATGCTGCAATCGTGCTACAACTTCCTCAAGGAACAACATTGCCAAAAGGCTTCTACGCAGA
hCoV-19/India/HR-MDU-IGIB1210605800489930/2022_EPI... (28711)	TCCTGCTAACAATGCTGCAATCGTGCTACAACTTCCTCAAGGAACAACATTGCCAAAAGGCTTCTACGCAGA
hCoV-19/England/PLYM-332B917/2022_EPI_ISL_9062229 ... (28643)	TCCTGCTAACAATGCTGCAATCGTGCTACAACTTCCTCAAGGAACAACATTGCCAAAAGGCTTCTACGCAGA
hCoV-19/Germany/HE-RKI-I-438397/2022_EPI_ISL_905522... (28651)	TCCTGCTAACAATGCTGCAATCGTGCTACAACTTCCTCAAGGAACAACATTGCCAAAAGGCTTCTACGCAGA
hCoV-19/USA/DE-CDC-LC0472738/2021_EPI_ISL_9049423... (28621)	TCCTGCTAACAATGCTGCAATCGTGCTACAACTTCCTCAAGGAACAACATTGCCAAAAGGCTTCTACGCAGA

Omicron BA.1

Section 401

	(28801)	28801 28810 28820 28830 28840 28850 28860 28872
SARS-CoV-2 Reference Genome NC_045512.2	(28795)	AGGGAGCAGAGGCGGCAGTCAAGCCTCTTCTCGTTCCTCATCACGTAGTCGCAACAGTTCAAGAAATTCAAC
hCoV-19/Botswana/R69B55_BHP_916539/2021_EPI_ISL_90...	(28741)	AGGGAGCAGAGGCGGCAGTCAAGCCTCTTCTCGTTCCTCATCACGTAGTCGCAACAGTTCAAGAAATTCAAC
hCoV-19/India/HR-MDU-IGIB1210605800489930/2022_EPI...	(28783)	AGGGAGCAGAGGCGGCAGTCAAGCCTCTTCTCGTTCCTCATCACGTAGTCGCAACAGTTCAAGAAATTCAAC
hCoV-19/England/PLYM-332B917/2022_EPI_ISL_9062229_...	(28715)	AGGGAGCAGAGGCGGCAGTCAAGCCTCTTCTCGTTCCTCATCACGTAGTCGCAACAGTTCAAGAAATTCAAC
hCoV-19/Germany/HE-RKI-I-438397/2022_EPI_ISL_905522...	(28723)	AGGGAGCAGAGGCGGCAGTCAAGCCTCTTCTCGTTCCTCATCACGTAGTCGCAACAGTTCAAGAAATTCAAC
hCoV-19/USA/DE-CDC-LC0472738/2021_EPI_ISL_9049423...	(28693)	AGGGAGCAGAGGCGGCAGTCAAGCCTCTTCTCGTTCCTCATCACGTAGTCGCAACAGTTCAAGAAATTCAAC

Section 402

	(28873)	28873 28880 28890 28900 28910 28920 28930 28944
SARS-CoV-2 Reference Genome NC_045512.2	(28867)	TCCAGGCAGCAGTAGGGGAACTTCTCCTGCTAGAATGGCTGGCAATGGCGGTGATGCTGCTCTTGCTTTGCT
hCoV-19/Botswana/R69B55_BHP_916539/2021_EPI_ISL_90...	(28813)	TCCAGGCAGCAGTAAACGAACTTCTCCTGCTAGAATGGCTGGCAATGGCGGTGATGCTGCTCTTGCTTTGCT
hCoV-19/India/HR-MDU-IGIB1210605800489930/2022_EPI...	(28855)	TCCAGGCAGCAGTAAACGAACTTCTCCTGCTAGAATGGCTGGCAATGGCGGTGATGCTGCTCTTGCTTTGCT
hCoV-19/England/PLYM-332B917/2022_EPI_ISL_9062229_...	(28787)	TCCAGGCAGCAGTAAACGAACTTCTCCTGCTAGAATGGCTGGCAATGGCGGTGATGCTGCTCTTGCTTTGCT
hCoV-19/Germany/HE-RKI-I-438397/2022_EPI_ISL_905522...	(28795)	TCCAGGCAGCAGTAAACGAACTTCTCCTGCTAGAATGGCTGGCAATGGCGGTGATGCTGCTCTTGCTTTGCT
hCoV-19/USA/DE-CDC-LC0472738/2021_EPI_ISL_9049423...	(28765)	TCCAGGCAGCAGTAAACGAACTTCTCCTGCTAGAATGGCTGGCAATGGCGGTGATGCTGCTCTTGCTTTGCT

Section 403

	(28945)	28945 28950 28960 28970 28980 28990 29000 29016
SARS-CoV-2 Reference Genome NC_045512.2	(28939)	GCTGCTTGACAGATTGAACCAGCTTGAGAGCAAAATGTCTGGTAAAGGCCAACAACAACAAGGCCAAACTGT
hCoV-19/Botswana/R69B55_BHP_916539/2021_EPI_ISL_90...	(28885)	GCTGCTTGACAGATTGAACCAGCTTGAGAGCAAAATGTCTGGTAAAGGCCAACAACAACAAGGCCAAACTGT
hCoV-19/India/HR-MDU-IGIB1210605800489930/2022_EPI...	(28927)	GCTGCTTGACAGATTGAACCAGCTTGAGAGCAAAATGTCTGGTAAAGGCCAACAACAACAAGGCCAAACTGT
hCoV-19/England/PLYM-332B917/2022_EPI_ISL_9062229_...	(28859)	GCTGCTTGACAGATTGAACCAGCTTGAGAGCAAAATGTCTGGTAAAGGCCAACAACAACAAGGCCAAACTGT
hCoV-19/Germany/HE-RKI-I-438397/2022_EPI_ISL_905522...	(28867)	GCTGCTTGACAGATTGAACCAGCTTGAGAGCAAAATGTCTGGTAAAGGCCAACAACAACAAGGCCAAACTGT
hCoV-19/USA/DE-CDC-LC0472738/2021_EPI_ISL_9049423...	(28837)	GCTGCTTGACAGATTGAACCAGCTTGAGAGCAAAATGTCTGGTAAAGGCCAACAACAACAAGGCCAAACTGT

Section 404

	(29017)	29017 29030 29040 29050 29060 29070 29088
SARS-CoV-2 Reference Genome NC_045512.2	(29011)	CACTAAGAAATCTGCTGCTGAGGCTTCTAAGAAGCCTCGGCAAAAACGTACTGCCACTAAAGCATACAATGT
hCoV-19/Botswana/R69B55_BHP_916539/2021_EPI_ISL_90...	(28957)	CACTAAGAAATCTGCTGCTGAGGCTTCTAAGAAGCCTCGGCAAAAACGTACTGCCACTAAAGCATACAATGT
hCoV-19/India/HR-MDU-IGIB1210605800489930/2022_EPI...	(28999)	CACTAAGAAATCTGCTGCTGAGGCTTCTAAGAAGCCTCGGCAAAAACGTACTGCCACTAAAGCATACAATGT
hCoV-19/England/PLYM-332B917/2022_EPI_ISL_9062229_...	(28931)	CACTAAGAAATCTGCTGCTGAGGCTTCTAAGAAGCCTCGGCAAAAACGTACTGCCACTAAAGCATACAATGT
hCoV-19/Germany/HE-RKI-I-438397/2022_EPI_ISL_905522...	(28939)	CACTAAGAAATCTGCTGCTGAGGCTTCTAAGAAGCCTCGGCAAAAACGTACTGCCACTAAAGCATACAATGT
hCoV-19/USA/DE-CDC-LC0472738/2021_EPI_ISL_9049423...	(28909)	CACTAAGAAATCTGCTGCTGAGGCTTCTAAGAAGCCTCGGCAAAAACGTACTGCCACTAAAGCATACAATGT

Omicron BA.1

Section 405

Sequence	Start	29089 … 29160
	(29089)	
SARS-CoV-2 Reference Genome NC_045512.2	(29083)	AACACAAGCTTTCGGCAGACGTGGTCCAGAACAAACCCAAGGAAATTTTGGGGACCAGGAACTAATCAGACA
hCoV-19/Botswana/R69B55_BHP_916539/2021_EPI_ISL_90...	(29029)	AACACAAGCTTTCGGCAGACGTGGTCCAGAACAAACCCAAGGAAATTTTGGGGACCAGGAACTAATCAGACA
hCoV-19/India/HR-MDU-IGIB1210605800489930/2022_EPI...	(29071)	AACACAAGCTTTCGGCAGACGTGGTCCAGAACAAACCCAAGGAAATTTTGGGGACCAGGAACTAATCAGACA
hCoV-19/England/PLYM-332B917/2022_EPI_ISL_9062229_...	(29003)	AACACAAGCTTTCGGCAGACGTGGTCCAGAACAAACCCAAGGAAATTTTGGGGACCAGGAACTAATCAGACA
hCoV-19/Germany/HE-RKI-I-438397/2022_EPI_ISL_905522...	(29011)	AACACAAGCTTTCGGCAGACGTGGTCCAGAACAAACCCAAGGAAATTTTGGGGACCAGGAACTAATCAGACA
hCoV-19/USA/DE-CDC-LC0472738/2021_EPI_ISL_9049423...	(28981)	AACACAAGCTTTCGGCAGACGTGGTCCAGAACAAACCCAAGGAAATTTTGGGGACCAGGAACTAATCAGACA

Section 406

Sequence	Start	29161 … 29232
	(29161)	
SARS-CoV-2 Reference Genome NC_045512.2	(29155)	AGGAACTGATTACAAACATTGGCCGCAAATTGCACAATTTGCCCCCAGCGCTTCAGCGTTCTTCGGAATGTC
hCoV-19/Botswana/R69B55_BHP_916539/2021_EPI_ISL_90...	(29101)	AGGAACTGATTACAAACATTGGCCGCAAATTGCACAATTTGCCCCCAGCGCTTCAGCGTTCTTCGGAATGTC
hCoV-19/India/HR-MDU-IGIB1210605800489930/2022_EPI...	(29143)	AGGAACTGATTACAAACATTGGCCGCAAATTGCACAATTTGCCCCCAGCGCTTCAGCGTTCTTCGGAATGTC
hCoV-19/England/PLYM-332B917/2022_EPI_ISL_9062229_...	(29075)	AGGAACTGATTACAAACATTGGCCGCAAATTGCACAATTTGCCCCCAGCGCTTCAGCGTTCTTCGGAATGTC
hCoV-19/Germany/HE-RKI-I-438397/2022_EPI_ISL_905522...	(29083)	AGGAACTGATTACAAACATTGGCCGCAAATTGCACAATTTGCCCCCAGCGCTTCAGCGTTCTTCGGAATGTC
hCoV-19/USA/DE-CDC-LC0472738/2021_EPI_ISL_9049423...	(29053)	AGGAACTGATTACAAACATTGGCCGCAAATTGCACAATTTGCCCCCAGCGCTTCAGCGTTCTTCGGAATGTC

Section 407

Sequence	Start	29233 … 29304
	(29233)	
SARS-CoV-2 Reference Genome NC_045512.2	(29227)	GCGCATTGGCATGGAAGTCACACCTTCGGGAACGTGGTTGACCTACACAGGTGCCATCAAATTGGATGACAA
hCoV-19/Botswana/R69B55_BHP_916539/2021_EPI_ISL_90...	(29173)	GCGCATTGGCATGGAAGTCACACCTTCGGGAACGTGGTTGACCTACACAGGTGCCATCAAATTGGATGACAA
hCoV-19/India/HR-MDU-IGIB1210605800489930/2022_EPI...	(29215)	GCGCATTGGCATGGAAGTCACACCTTCGGGAACGTGGTTGACCTACACAGGTGCCATCAAATTGGATGACAA
hCoV-19/England/PLYM-332B917/2022_EPI_ISL_9062229_...	(29147)	GCGCATTGGCATGGAAGTCACACCTTCGGGAACGTGGTTGACCTACACAGGTGCCATCAAATTGGATGACAA
hCoV-19/Germany/HE-RKI-I-438397/2022_EPI_ISL_905522...	(29155)	GCGCATTGGCATGGAAGTCACACCTTCGGGAACGTGGTTGACCTACACAGGTGCCATCAAATTGGATGACAA
hCoV-19/USA/DE-CDC-LC0472738/2021_EPI_ISL_9049423...	(29125)	GCGCATTGGCATGGAAGTCACACCTTCGGGAACGTGGTTGACCTACACAGGTGCCATCAAATTGGATGACAA

Section 408

Sequence	Start	29305 … 29376
	(29305)	
SARS-CoV-2 Reference Genome NC_045512.2	(29299)	AGATCCAAATTTCAAAGATCAAGTCATTTTGCTGAATAAGCATATTGACGCATACAAAACATTCCCACCAAC
hCoV-19/Botswana/R69B55_BHP_916539/2021_EPI_ISL_90...	(29245)	AGATCCAAATTTCAAAGATCAAGTCATTTTGCTGAATAAGCATATTGACGCATACAAAACATTCCCACCAAC
hCoV-19/India/HR-MDU-IGIB1210605800489930/2022_EPI...	(29287)	AGATCCAAATTTCAAAGATCAAGTCATTTTGCTGAATAAGCATATTGACGCATACAAAACATTCCCACCAAC
hCoV-19/England/PLYM-332B917/2022_EPI_ISL_9062229_...	(29219)	AGATCCAAATTTCAAAGATCAAGTCATTTTGCTGAATAAGCATATTGACGCATACAAAACATTCCCACCAAC
hCoV-19/Germany/HE-RKI-I-438397/2022_EPI_ISL_905522...	(29227)	AGATCCAAATTTCAAAGATCAAGTCATTTTGCTGAATAAGCATATTGACGCATACAAAACATTCCCACCAAC
hCoV-19/USA/DE-CDC-LC0472738/2021_EPI_ISL_9049423...	(29197)	AGGTCCAAATTTCAAAGATCAAGTCATTTTGCTGAATAAGCATATTGACGCATACAAAACATTCCCACCAAC

Omicron BA.1

Section 409

```
                                                        (29377) 29377        29390     29400     29410     29420     29430              29448
SARS-CoV-2 Reference Genome NC_045512.2                 (29371) AGAGCCTAAAAAGGACAAAAAGAAGAAGGCTGATGAAACTCAAGCCTTACCGCAGAGACAGAAGAAACAGCA
hCoV-19/Botswana/R69B55_BHP_916539/2021_EPI_ISL_90...   (29317) AGAGCCTAAAAAGGACAAAAAGAAGAAGGCTGATGAAACTCAAGCCTTACCGCAGAGACAGAAGAAACAGCA
hCoV-19/India/HR-MDU-IGIB1210605800489930/2022_EPI...   (29359) AGAGCCTAAAAAGGACAAAAAGAAGAAGGCTGATGAAACTCAAGCCTTACCGCAGAGACAGAAGAAACAGCA
hCoV-19/England/PLYM-332B917/2022_EPI_ISL_9062229_...   (29291) AGAGCCTAAAAAGGACAAAAAGAAGAAGGCTGATGAAACTCAAGCCTTACCGCAGAGACAGAAGAAACAGCA
hCoV-19/Germany/HE-RKI-I-438397/2022_EPI_ISL_905522...  (29299) AGAGCCTAAAAAGGACAAAAAGAAGAAGGCTGATGAAACTCAAGCCTTACCGCAGAGACAGAAGAAACAGCA
hCoV-19/USA/DE-CDC-LC0472738/2021_EPI_ISL_9049423...    (29269) AGAGCCTAAAAAGGACAAAAAGAAGAAGGCTGATGAAACTCAAGCCTTACCGCAGAGACAGAAGAAACAGCA
```

Section 410

```
                                                        (29449) 29449      29460     29470     29480     29490     29500     29510   29520
SARS-CoV-2 Reference Genome NC_045512.2                 (29443) AACTGTGACTCTTCTTCCTGCTGCAGATTTGGATGATTTCTCCAAACAATTGCAACAATCCATGAGCAGTGC
hCoV-19/Botswana/R69B55_BHP_916539/2021_EPI_ISL_90...   (29389) AACTGTGACTCTTCTTCCTGCTGCAGATTTGGATGATTTCTCCAAACAATTGCAACAATCCATGAGCAGTGC
hCoV-19/India/HR-MDU-IGIB1210605800489930/2022_EPI...   (29431) AACTGTGACTCTTCTTCCTGCTGCAGATTTGGATGATTTCTCCAAACAATTGCAACAATCCATGAGCAGTGC
hCoV-19/England/PLYM-332B917/2022_EPI_ISL_9062229_...   (29363) AACTGTGACTCTTCTTCCTGCTGCAGATTTGGATGATTTCTCCAAACAATTGCAACAATCCATGAGCAGTGC
hCoV-19/Germany/HE-RKI-I-438397/2022_EPI_ISL_905522...  (29371) AACTGTGACTCTTCTTCCTGCTGCAGATTTGGATGATTTCTCCAAACAATTGCAACAATCCATGAGCAGTGC
hCoV-19/USA/DE-CDC-LC0472738/2021_EPI_ISL_9049423...    (29341) AACTGTGACTCTTCTTCCTGCTGCAGATTTGGATGATTTCTCCAAACAATTGCAACAATCCATGAGCAGTGC
```

Section 411

```
                                                        (29521) 29521    29530     29540     29550     29560     29570     29580      29592
SARS-CoV-2 Reference Genome NC_045512.2                 (29515) TGACTCAACTCAGGCCTAAACTCATGCAGACCACACAAGGCAGATGGGCTATATAAACGTTTTCGCTTTTCC
hCoV-19/Botswana/R69B55_BHP_916539/2021_EPI_ISL_90...   (29461) TGACTCAACTCAGGCCTAAACTCATGCAGACCACACAAGGCAGATGGGCTATATAAACGTTTTCGCTTTTCC
hCoV-19/India/HR-MDU-IGIB1210605800489930/2022_EPI...   (29503) TGACTCAACTCAGGCCTAAACTCATGCAGACCACACAAGGCAGATGGGCTATATAAACGTTTTCGCTTTTCC
hCoV-19/England/PLYM-332B917/2022_EPI_ISL_9062229_...   (29435) TGACTCAACTCAGGCCTAAACTCATGCAGACCACACAAGGCAGATGGGCTATATAAACGTTTTCGCTTTTCC
hCoV-19/Germany/HE-RKI-I-438397/2022_EPI_ISL_905522...  (29443) TGACTCAACTCAGGCCTAAACTCATGCAGACCACACAAGGCAGATGGGCTATATAAACGTTTTCGCTTTTCC
hCoV-19/USA/DE-CDC-LC0472738/2021_EPI_ISL_9049423...    (29413) TGACTCAACTCAGGCCTAAACTCATGCAGACCACACAAGGCAGATGGGCTATATAAACGTTTTCGCTTTTCC
```

Section 412

```
                                                        (29593) 29593  29600     29610     29620     29630     29640     29650      29664
SARS-CoV-2 Reference Genome NC_045512.2                 (29587) GTTTACGATATATAGTCTACTCTTGTGCAGAATGAATTCTCGTAACTACATAGCACAAGTAGATGTAGTTAA
hCoV-19/Botswana/R69B55_BHP_916539/2021_EPI_ISL_90...   (29533) GTTTACGATATATAGTCTACTCTTGTGCAGAATGAATTCTCGTAACTACATAGCACAAGTAGATGTAGTTAA
hCoV-19/India/HR-MDU-IGIB1210605800489930/2022_EPI...   (29575) GTTTACGATATATAGTCTACTCTTGTGCAGAATGAATTCTCGTAACTACATAGCACAAGTAGATGTAGTTAA
hCoV-19/England/PLYM-332B917/2022_EPI_ISL_9062229_...   (29507) GTTTACGATATATAGTCTACTCTTGTGCAGAATGAATTCTCGTAATTACATAGCACAAGTAGATGTAGTTAA
hCoV-19/Germany/HE-RKI-I-438397/2022_EPI_ISL_905522...  (29515) GTTTACGATATATAGTCTACTCTTGTGCAGAATGAATTCTCGTAACTACATAGCACAAGTAGATGTAGTTAA
hCoV-19/USA/DE-CDC-LC0472738/2021_EPI_ISL_9049423...    (29485) GTTTACGATATATAGTCTACTCTTGTGCAGAATGAATTCTCGTAACTACATAGCACAAGTAGATGTAGTTAA
```

Omicron BA.1

Section 413

	(29665)	29665 29670 29680 29690 29700 29710 29720 29736
SARS-CoV-2 Reference Genome NC_045512.2	(29659)	CTTTAATCTCACATAGCAATCTTTAATCAGTGTGTAACATTAGGGAGGACTTGAAAGAGCCACCACATTTTC
hCoV-19/Botswana/R69B55_BHP_916539/2021_EPI_ISL_90...	(29605)	CTTTAATCTCACATAGCAATCTTTAATCAGTGTGTAACATTAGGGAGGACTTGAAAGAGCCACCACATTTTC
hCoV-19/India/HR-MDU-IGIB1210605800489930/2022_EPI...	(29647)	CTTTAATCTCACATAGCAATCTTTAATCAGTGTGTAACATTAGGGAGGACTTGAAAGAGCCACCACATTTTC
hCoV-19/England/PLYM-332B917/2022_EPI_ISL_9062229_...	(29579)	CTTTAATCTCACATAGCAATCTTTAATCAGTGTGTAACATTAGGGAGGACTTGAAAGAGCCACCACATTTTC
hCoV-19/Germany/HE-RKI-I-438397/2022_EPI_ISL_905522...	(29587)	CTTTAATCTCACATAGCAATCTTTAATCAGTGTGTAACATTAGGGAGGACTTGAAAGAGCCACCACATTTTC
hCoV-19/USA/DE-CDC-LC0472738/2021_EPI_ISL_9049423...	(29557)	CTTTAATCTCACATAGCAATCTTTAATCAGTGTGTAACATTAGGGAGGACTTGAAAGAGCCACCACATTTTC

Section 414

	(29737)	29737 29750 29760 29770 29780 29790 29808
SARS-CoV-2 Reference Genome NC_045512.2	(29731)	ACCGAGGCCACGCGGAGTACGATCGAGTGTACAGTGAACAATGCTAGGGAGAGCTGCCTATATGGAAGAGCC
hCoV-19/Botswana/R69B55_BHP_916539/2021_EPI_ISL_90...	(29677)	ACCGAGGCCACGCGGAGTACGATCGAGTGTACAGTGAA----------------------------------
hCoV-19/India/HR-MDU-IGIB1210605800489930/2022_EPI...	(29719)	ACCGAGGCCACGCGGAGTACGATCGAGTGTACAGTGAACAATGCTAGGGAGAGCTNNNNNNNNNNNNNNNNN
hCoV-19/England/PLYM-332B917/2022_EPI_ISL_9062229_...	(29651)	ACCGAGGCCACGCGGAGTACGATCGAGTGTACAGTGAACAATGCTAGGGAGAGCTGCCTATATGGAAGAGCC
hCoV-19/Germany/HE-RKI-I-438397/2022_EPI_ISL_905522...	(29659)	ACCGAGGCCACGCGGAGTACGATCGAGTGTACAGTGAACAATGCTAGGGAGAGCTGCCTATATGGAAGAGCC
hCoV-19/USA/DE-CDC-LC0472738/2021_EPI_ISL_9049423...	(29629)	ACCGAGGCCACGCGGAGTACGATCGAGTGTACAGTGAACAATGCTAGGGAGAGCTGCCTATATGGAAGAGCC

Section 415

	(29809)	29809 29820 29830 29840 29850 29860 29870 29880
SARS-CoV-2 Reference Genome NC_045512.2	(29803)	CTAATGTGTAAAATTAATTTTAGTAGTGCTATCCCCATGTGATTTTAATAGCTTCTTAGGAGAATGACAAAA
hCoV-19/Botswana/R69B55_BHP_916539/2021_EPI_ISL_90...	(29715)	--
hCoV-19/India/HR-MDU-IGIB1210605800489930/2022_EPI...	(29791)	NNNNNNNNNNNNNNNNNNNNNNNNNNNNNNNNNNNNNNNNNNNNNNNNNNNNNNNNNNNNNNNNNNNCAAAA
hCoV-19/England/PLYM-332B917/2022_EPI_ISL_9062229_...	(29723)	CTAATGTGTAAAATTAATTTTAGTA---
hCoV-19/Germany/HE-RKI-I-438397/2022_EPI_ISL_905522...	(29731)	CTAATGTGTAAAATTAATTTTAGTAGTGCTATCCCCATGTGATTTTAATAGCTTC-----------------
hCoV-19/USA/DE-CDC-LC0472738/2021_EPI_ISL_9049423...	(29701)	CTAATGTGTAAAATTAA---

Section 416

	(29881)	29881 29890 29909
SARS-CoV-2 Reference Genome NC_045512.2	(29875)	AAAAAAAAAAAAAAAAAAAAAAAAAAAAA
hCoV-19/Botswana/R69B55_BHP_916539/2021_EPI_ISL_90...	(29715)	-----------------------------
hCoV-19/India/HR-MDU-IGIB1210605800489930/2022_EPI...	(29863)	AAAAAAAAAAAAAAAAAAAAAAAAAAAAA
hCoV-19/England/PLYM-332B917/2022_EPI_ISL_9062229_...	(29748)	-----------------------------
hCoV-19/Germany/HE-RKI-I-438397/2022_EPI_ISL_905522...	(29786)	-----------------------------
hCoV-19/USA/DE-CDC-LC0472738/2021_EPI_ISL_9049423...	(29718)	-----------------------------
