# Supplementary material for: Patch type nucleotide sequence identities between genomes from many different species facilitate illegitimate recombination
Source: Sci Rep. 2026 Mar 30;16:10524. doi: 10.1038/s41598-026-44124-0 (PMC13035915; doi:10.1038/s41598-026-44124-0)
Supplement: Supplementary file 12 — Supplementary Material 12 [file 41598_2026_44124_MOESM12_ESM.pdf]

Homo sapiens chromosome 17 vs. Lycium barbarum isolate Lr01 chromosome 6

	Start	Section 1 (1–75)
	(1)	1 … 75
Homo sapiens chromosome 17 NC_000017.11 region fr...	(1)	AGAGCCACTTTTTTCAGTTGAAGTCTGAGTTCTTATGGGCCATGACCAAGGCTGCTGCTTCCTCAAAGGCACGA
Lycium barbarum isolate Lr01 chromosome 6 NC_08334...	(1)	--------GAGTTTAAGAT--ATGATGTGTTGGCCTTGGGCAACACCTATAGTGAAGAGATTTCACTTCTTACAA

	Start	Section 2 (76–150)
	(76)	76 … 150
Homo sapiens chromosome 17 NC_000017.11 region fr...	(76)	AGGGGGCCGCGTGCGGTGGCTCACGCCTGTAATCCCAGCACTTTGGGAGGCCAAGGCGGGC--GGATCACGAGGT
Lycium barbarum isolate Lr01 chromosome 6 NC_08334...	(66)	ACAAAAAGAGCTAATTTGGTTCTATCTTTAAA--CTACTAAGTTGTGAGTGAATTTTAAACTTGTTTAAACTGTT

	Start	Section 3 (151–225)
	(151)	151 … 225
Homo sapiens chromosome 17 NC_000017.11 region fr...	(149)	CAGGAGTTTGAGATCAGCCTGGCAAACATGGCGACACCCTGTCTCTACTAAAAATACAAACAT-TAGCCCGGCGT
Lycium barbarum isolate Lr01 chromosome 6 NC_08334...	(139)	TGGTTGTTCATGACTAGGC-------CCTGAATAGATTTTGTAAC-ACTAAAAAGAGGAATATATAGACAGTCTT

	Start	Section 4 (226–300)
	(226)	226 … 300
Homo sapiens chromosome 17 NC_000017.11 region fr...	(223)	GGTGGCGTGTGCCTGTAGTCCCAGCTACTTGGGAGGCTGAGACAGGAGACTTGCTTGAATGTGGGAGGCGGAGGT
Lycium barbarum isolate Lr01 chromosome 6 NC_08334...	(206)	CTTGTTGT-TGTGAATCGTCTTTTGAAGTTTGTAG-CTAATTTGG-----TTCTGTCCATATATGTGACTTTGTT

	Start	Section 5 (301–375)
	(301)	301 … 375
Homo sapiens chromosome 17 NC_000017.11 region fr...	(298)	TGCAGTGAGTCAGATCGTGCCACTGCACTCCAGCCTGGGTGACAAAAGTGAAACTCTTGTCTTTAAAAAAAAAAA
Lycium barbarum isolate Lr01 chromosome 6 NC_08334...	(274)	TC---TCTGTTGAATGTATCCCATGC--TCAGTCTTGTGT---AGATGTTCTAAGAATGCTTTCACACTGTGTGT

	Start	Section 6 (376–450)
	(376)	376 … 450
Homo sapiens chromosome 17 NC_000017.11 region fr...	(373)	AAAGAAATGCAGGAAGG--GAAC---AGGGAACAAGGCTGGGAGGGAGGA-GCCGACGGCTGCAGCAAGCTCCG
Lycium barbarum isolate Lr01 chromosome 6 NC_08334...	(341)	AATTTTGTGTACGGAGTCTGATCTTGAGAGTAAATGTCTGAGATGCTTCACTGTCAAGATGTTCTGTATGGATCT

	Start	Section 7 (451–525)
	(451)	451 … 525
Homo sapiens chromosome 17 NC_000017.11 region fr...	(442)	GCCTCAGCCTCCCTTCCCTGCTCCTCTGAGATGCCGAGGGCGGGGCGTACTCACTGCAAGCCTGCCAAGTAGGAC
Lycium barbarum isolate Lr01 chromosome 6 NC_08334...	(416)	GTCTTCACCTAAGT---CTATATCTTAGGTGTTTAGATGGCTGGG-GTCCTATCT------CTTTTATTTATTTC

Homo sapiens chromosome 17 vs. Lycium barbarum isolate Lr01 chromosome 6

Section 8

	(526)	526 … 540 … 550 … 560 … 570 … 580 … 590 … 600
Homo sapiens chromosome 17 NC_000017.11 region fr...	(517)	CTCCATGTCCTCTGACATTCGA--GAGATGGATTTTTTGGCCCTGAGATCACGTCTCCTACTGTGTCGTCATTCT
Lycium barbarum isolate Lr01 chromosome 6 NC_08334...	(481)	-TCAATATGAT-TGAAAGTCTGTTTAGCTCAAGGTAGAGGTCTAGTGATT--GTTTAACACAT----GCCTTTTT

Section 9

	(601)	601 … 610 … 620 … 630 … 640 … 650 … 660 … 675
Homo sapiens chromosome 17 NC_000017.11 region fr...	(590)	AGCACCTGTGGGTGACTTCCTCATGTGAGTGAGGCATAAGCACCCGGAATCCGGACTGGAGGCCGTCTCTGCCTC
Lycium barbarum isolate Lr01 chromosome 6 NC_08334...	(548)	AGCATTATATGGGGTTTGCCTGAAATAAAATGGG--TAATTTCCT---ATACTAACTCCTCTCCTACTTTGTTTA

Section 10

	(676)	676 … 690 … 700 … 710 … 720 … 730 … 740 … 750
Homo sapiens chromosome 17 NC_000017.11 region fr...	(665)	TGGATTGCACACTGGCCTACAGACCTGGGGACAGAGTCTTTCACGAGTTGTGTATCGTTTAACAGAAACCCAACA
Lycium barbarum isolate Lr01 chromosome 6 NC_08334...	(618)	TT--TTGCA---TGACC---ACACC-GGAGCCGAAGAGTTTCC-----------TCTTTGAAACTCAACGACGTT

Section 11

	(751)	751 … 760 … 770 … 780 … 790 … 800 … 810 … 825
Homo sapiens chromosome 17 NC_000017.11 region fr...	(740)	GTT-CCACCCAAACCTCCTGACACATTCTCGGGCTTGGGAAGTGTCCCAAAGCACCAAGGGGGTGAGACACACGC
Lycium barbarum isolate Lr01 chromosome 6 NC_08334...	(673)	GTTACCACATGGAGCCCGTG---CATCCTCGATCAATTCCAGTTTTATATCGCGCAAAGAGAACCACAAAATAAT

Section 12

	(826)	826 … 840 … 850 … 860 … 870 … 880 … 890 … 900
Homo sapiens chromosome 17 NC_000017.11 region fr...	(814)	CTTGAAAAATGGCAGTAGGCATACTTACCTGTCTTCATGCGCTCACATACGAGGTGTACCTAGAACGCCCTCATG
Lycium barbarum isolate Lr01 chromosome 6 NC_08334...	(745)	C---AAATCTTTCTGTCGGAG------CCTATTTTGGCTAATTTATTTATTTGTTGATGCT-GCTTGTTTAAATG

Section 13

	(901)	901 … 910 … 920 … 930 … 940 … 950 … 960 … 975
Homo sapiens chromosome 17 NC_000017.11 region fr...	(889)	TTATATTCCCAGCACTAATGAAGCTACTGGTCAACAGCT-ATTTGTTCCCAAAGAATTTTCCCTTGCGCA-GCCG
Lycium barbarum isolate Lr01 chromosome 6 NC_08334...	(810)	TT-TGGTGTGACTAACATTCTACCTAATTGTGTGGTTATTATAAAATGCTTAACTGTTTTATTTTAACCAAGTCC

Section 14

	(976)	976 … 990 … 1000 … 1010 … 1020 … 1030 … 1040 … 1050
Homo sapiens chromosome 17 NC_000017.11 region fr...	(962)	CAGCCTGGAGGAAGCAGGGCGTGTGCCCTCGTCTGCTGGCGGCAGCAGTGAGGGGTGTAGCCAAGTGCCCACTTC
Lycium barbarum isolate Lr01 chromosome 6 NC_08334...	(884)	CATTTTATCCATACTAGGATGTCTTAACCAGCAATTTAGGTCTTAAATCAAGTGGAGTTTAATTGTTCCT-CTTG

Homo sapiens chromosome 17 vs. Lycium barbarum isolate Lr01 chromosome 6

Section 15

	(1051)	1051 … 1060 … 1070 … 1080 … 1090 … 1100 … 1110 … 1125
Homo sapiens chromosome 17 NC_000017.11 region fr...	(1037)	TCTCATT--CAGCTGCTTCGGGGCAAACTGGAAAGATCCTTTGAGCTCACAGCCCCCTGCTCAGTGCTACTCCTG
Lycium barbarum isolate Lr01 chromosome 6 NC_08334...	(958)	TGAGTTTGGCACCTTTTTTTAGTTAGAAAGAAAAGATA---TGCTTTCAAAGTATCATA---AATTTTACACACT

Section 16

	(1126)	1126 … 1140 … 1150 … 1160 … 1170 … 1180 … 1190 … 1200
Homo sapiens chromosome 17 NC_000017.11 region fr...	(1110)	AACCTGCGGGCCTGTGTGCACAGCCACCCACAGCGCGC-ACAACACATGGCCTGCCCAGGGCAACGGCACCGTC
Lycium barbarum isolate Lr01 chromosome 6 NC_08334...	(1027)	AAAC-GTTAGCTTGTTTTGAGAAATAAAAA-AGCAAACTAGTTTTTATGGATAACTTTTTCACTTTAGCCCACTT

Section 17

	(1201)	1201 … 1210 … 1220 … 1230 … 1240 … 1250 … 1260 … 1275
Homo sapiens chromosome 17 NC_000017.11 region fr...	(1184)	AACAGCGTGTGCCCTCGCTCAGGGCCCTGCTGGTACTACGCTTCCCAGTGTGCAGGGTTCCCAGACGTGTGTCCT
Lycium barbarum isolate Lr01 chromosome 6 NC_08334...	(1100)	TCCAATATTTGAAA-----CATGTATTTATTAAAACAAC-CTTTGCATAGTTTATATTGAACTGATA---GTCTT

Section 18

	(1276)	1276 … 1290 … 1300 … 1310 … 1320 … 1330 … 1340 … 1350
Homo sapiens chromosome 17 NC_000017.11 region fr...	(1259)	CTCATACATTGCATTTCCTCCCTAAGCTGCACGGGGCAACACCGTGTTAGGTCCCGCGCATCTGCTCCTGGGCCA
Lycium barbarum isolate Lr01 chromosome 6 NC_08334...	(1166)	TTCATAAGTC---TTTCATGT-TAAGCTAATC-----ACAAGTTTCGAAATACACAAACGTTTTC-CCTAAACTT

Section 19

	(1351)	1351 … 1360 … 1370 … 1380 … 1390 … 1400 … 1410 … 1425
Homo sapiens chromosome 17 NC_000017.11 region fr...	(1334)	CATCCGGCTCCGTCTGTATTGTTCCTGAATTCAGTTTGCTGTCCTAGAACAAGGCAGCAGGAGGGGAGAACAGCT
Lycium barbarum isolate Lr01 chromosome 6 NC_08334...	(1231)	TATTTAGTTCACTACTTTTCATA--TGATATTTGTATTTTTTTTTAAAATGCTTCCACATTTGTATACAACAA--

Section 20

	(1426)	1426 … 1440 … 1450 … 1460 … 1470 … 1480 … 1490 … 1500
Homo sapiens chromosome 17 NC_000017.11 region fr...	(1409)	TTTCAGTGTCTTTAATTAGGCAACAGGAAAAAACAAAGTCTGGCTGAAAATTACTATTTGAAGCTTGGCCGATGC
Lycium barbarum isolate Lr01 chromosome 6 NC_08334...	(1302)	--TCAGCCTC---AATTTTTCTCAACTTAAGTCCG--GTCAGT-TAACCATTATTAATGGAT-CTTAAAGGATGC

Section 21

	(1501)	1501 … 1510 … 1520 … 1530 … 1540 … 1550 … 1560 … 1575
Homo sapiens chromosome 17 NC_000017.11 region fr...	(1484)	CAACACAGCACTCGCTCCCTGCGTTCTGGTAAGTGATGGCCATTGATTTCAAGGACGTACCAAGGGGCGAAAAGC
Lycium barbarum isolate Lr01 chromosome 6 NC_08334...	(1368)	TTA------ATACCTTCCCT---TTAGATTAATTGAACCC------TGTCACGACCCAATTAAGGGGCCGTGACG

Homo sapiens chromosome 17 vs. Lycium barbarum isolate Lr01 chromosome 6

```
                                                             Section 22
                                                 (1576) 1576          1590      1600      1610      1620      1630      1640      1650
  Homo sapiens chromosome 17 NC_000017.11 region fr...  (1559) AAGAGCAGGAAGTCACCTCGCTCTGGAAAAGA-AGCTGGTTTTCATCTGCTGTATGTGCTGGTCCCAAACA-TAC
Lycium barbarum isolate Lr01 chromosome 6 NC_08334...  (1428) AGCACCCGGTGCTAGCCTACCCGGGCACCCCTTAACTCATTTTTATGCT-TACATCTAGGTGACCCATACAATTA
                                                             Section 23
                                                 (1651) 1651      1660      1670      1680      1690      1700      1710          1725
  Homo sapiens chromosome 17 NC_000017.11 region fr...  (1632) CCTTTTCTCTTTTTTAGTATTTTTTTTC---CCAATTAGGAATTCAATGTGGAAAACATACCCTTTTTCTTCAA
Lycium barbarum isolate Lr01 chromosome 6 NC_08334...  (1502) AACATACAAATCTATTCGTCCATCATACCAGTCCCATT-GGACGATAATGCCTTTAT-ATAATGATTGGCATTTA
                                                             Section 24
                                                 (1726) 1726          1740      1750      1760      1770      1780      1790      1800
  Homo sapiens chromosome 17 NC_000017.11 region fr...  (1704) CAGGCTTTTAATGT---TGGGAAGACA-GGTTAAAAAAACAACAACAACAGGCTGGCTGTGCCGGCGC-ACGCC
Lycium barbarum isolate Lr01 chromosome 6 NC_08334...  (1575) TGCCACATCAACGTACATGAGCCGACAAGGCTATCAAAATGATATACAAAATATGGGCCGACAAATCTAGACATA
                                                             Section 25
                                                 (1801) 1801      1810      1820      1830      1840      1850      1860          1875
  Homo sapiens chromosome 17 NC_000017.11 region fr...  (1774) TGGAATCCCAGCACTGTGGG-AGGCCAAGGCAGGCAGATCACCTGAGGTCAGGAGTTCGAGACCAGCCTGGCCAA
Lycium barbarum isolate Lr01 chromosome 6 NC_08334...  (1650) TCTAACCATATACAAGTGACTACGAGACTCTAAGAAGAGTATAACATATCACATGAGCGGGACAGGACAAGACCC
                                                             Section 26
                                                 (1876) 1876          1890      1900      1910      1920      1930      1940      1950
  Homo sapiens chromosome 17 NC_000017.11 region fr...  (1848) CATGGCGAAACCCCGTCTCTACTAAAAATACAAAAATTAGTCAGGCGCGGTGGTGTCGCTTGTAAT----CTCAG
Lycium barbarum isolate Lr01 chromosome 6 NC_08334...  (1725) CGCTGTGCCTATAATTATGTACAAAAAAGA--ATGAGTACCCAAAAGCTATGGCTCCGAATGAAATGGAGCTCTG
                                                             Section 27
                                                 (1951) 1951      1960      1970      1980      1990      2000      2010          2025
  Homo sapiens chromosome 17 NC_000017.11 region fr...  (1919) CTACTTGG-----GAGACTGAGGCA--GGAGAATGGCT-TGAACCTGTG-AGCTGGGATCGTGCCACTGCA--CT
Lycium barbarum isolate Lr01 chromosome 6 NC_08334...  (1798) CTAGGTGGTCTTTGAGAATACCGCTATGGATCAAGCCTGTCTCCCTGTGCACCTGCGGGCATGGCGCAGCGTCCA
                                                             Section 28
                                                 (2026) 2026          2040      2050      2060      2070      2080      2090      2100
  Homo sapiens chromosome 17 NC_000017.11 region fr...  (1983) CTAGCCG--GGGCGACAGAGCGAGA------CTCTGTCTCAAAAAAA-AA--AAAAAAAAAAAACCCTCATCGCT
Lycium barbarum isolate Lr01 chromosome 6 NC_08334...  (1873) CAAACCAAAGGACGTCAGTACGAAAAATGTACTGAGTATGTAAAGCATAGTCAATATCAATATAGAAGCATAGAT
```

Homo sapiens chromosome 17 vs. Lycium barbarum isolate Lr01 chromosome 6

```
                                                                 Section 29
                                                   (2101) 2101     2110      2120      2130      2140      2150      2160           2175
Homo sapiens chromosome 17 NC_000017.11 region fr... (2047) GTTGTGAGGATGAAT------GGATGGGATCTGGAAGT--CACTGTGGTAAACACAAATCTGGAACCACAGAGT
Lycium barbarum isolate Lr01 chromosome 6 NC_08334... (1948) AGCAACATAAGAAATAACATAGGATGGGAAATGGCAATATCATCGTCATATGCACTTACTTGCCTTTCATAG---
                                                                 Section 30
                                                   (2176) 2176          2190      2200      2210      2220      2230      2240     2250
Homo sapiens chromosome 17 NC_000017.11 region fr... (2114) GGCGTTTCACAGCCGCCCCCAGCCTCCACCTGCAGGGCCTGGTTTCCTGGCTGCCGGAGGAAGCCCTTGAATA
Lycium barbarum isolate Lr01 chromosome 6 NC_08334... (2020) GAACTTTCCCTTGCTAATAC--GTATTTGTGTACATA-CATCACTATCCGTACTCCACCATAATACCCGT-ACCA
                                                                 Section 31
                                                   (2251) 2251     2260      2270      2280      2290      2300      2310           2325
Homo sapiens chromosome 17 NC_000017.11 region fr... (2189) TGCCACAGCTGGTGCTGAACCTGCTTCCAATTAGCAACAAGAATCTGAAGGTGGAGCTGCTTGGCGGGATTATC
Lycium barbarum isolate Lr01 chromosome 6 NC_08334... (2091) TATTTGTGCTCGTATTCGTG-TACATGCCCTTATTCACATTCATATACATGGTCATATCATATTCG----TATTC
                                                                 Section 32
                                                   (2326) 2326          2340      2350      2360      2370      2380      2390     2400
Homo sapiens chromosome 17 NC_000017.11 region fr... (2264) ATGCCACCCACTTCGCCTGCATCTGTGGCCTTTACGACCTCTCACTATCGATCCCTGACACTCAGAA---GTGAT
Lycium barbarum isolate Lr01 chromosome 6 NC_08334... (2161) ATATCA-----TATGCATAATCATGTCATACACATAACATTTACATAGCATACCCGAACACGTAGGATCGGTGTT
                                                                 Section 33
                                                   (2401) 2401     2410      2420      2430      2440      2450      2460           2475
Homo sapiens chromosome 17 NC_000017.11 region fr... (2336) TCTTCCTAACGGTTTCCCGCAAAGCAGTCACTGTATAAACCTGAACCTTTGGAGTAAACCTTCCCCACGGGTCT
Lycium barbarum isolate Lr01 chromosome 6 NC_08334... (2231) CCATACATATCCTTATATAACATACCCGACCACGTAGGATCGGTA---TTTCACGTACCCGATCATATATAGGCT
                                                                 Section 34
                                                   (2476) 2476          2490      2500      2510      2520      2530      2540     2550
Homo sapiens chromosome 17 NC_000017.11 region fr... (2411) TAAGGAAGCTACAGACCCACTCTAAAAGGGGAATTCTGGTGTCAAGAATCCAGT--TGCAATGACTTAGGGCAAC
Lycium barbarum isolate Lr01 chromosome 6 NC_08334... (2303) CGGTGATCATACATACCTGGCCAACCAAGGC--TCAGTGTTACACGTACCTGGCCCTACCAAGGCTCAGTGTTGT
                                                                 Section 35
                                                   (2551) 2551     2560      2570      2580      2590      2600      2610           2625
Homo sapiens chromosome 17 NC_000017.11 region fr... (2484) CC---CCTCGCTGGACTTTGTTCCTCCTGCCCCAAAGGTAAGCAGCTGCTAAGAGAACCC-ACCTTTCCTCCGAG
Lycium barbarum isolate Lr01 chromosome 6 NC_08334... (2376) CCATACCCAACTGCAGTGGTGTGCGCGCGTTACGTAAATATACA--TACATATACATCTATACATATACTACCCG
```

Homo sapiens chromosome 17 vs. Lycium barbarum isolate Lr01 chromosome 6

Section 36

(2626)	2626 2640 2650 2660 2670 2680 2690 2700
Homo sapiens chromosome 17 NC_000017.11 region fr... (2555)	GCCATC-ATGATACATGGAGAGAAATCCTGCCACCTCC-CACATTCAGGTCGGCCACATTCATCCCACCCACCCC
Lycium barbarum isolate Lr01 chromosome 6 NC_08334... (2449)	GCCATATAAGCTCGGTGTTCCATAATAGTCATACATATACACAAT-AGCTCATAAGCATCTTTACTTTTAACCCC

Section 37

(2701)	2701 2710 2720 2730 2740 2750 2760 2775
Homo sapiens chromosome 17 NC_000017.11 region fr... (2628)	GACCCCTAAGGGAGACTCTGGCAGCGTTCCCAGAGAAGCCTTGAGCCTGGGACCTGCTGGGTTTAAGAGGCTCCC
Lycium barbarum isolate Lr01 chromosome 6 NC_08334... (2523)	AT----TATCATAATCGTCATCGTTATTATCATTATAGCCATCATCAAT--ACCAT-TATCACTATCATCATCAT

Section 38

(2776)	2776 2790 2800 2810 2820 2830 2840 2850
Homo sapiens chromosome 17 NC_000017.11 region fr... (2703)	ACACTTGGCCCATTCTTCACAAGGAGAC---CCGTGCGTGATTCCTCCTGTTCTCCCAACCAGCTTTCTCAGGCT
Lycium barbarum isolate Lr01 chromosome 6 NC_08334... (2591)	TCATTTCATCTAC-CTTAATAGGCCTACTCGCCATGCGAGAAACTTAGTACAATCGTAGCGAA-----TCAAGAA

Section 39

(2851)	2851 2860 2870 2880 2890 2900 2910 2925
Homo sapiens chromosome 17 NC_000017.11 region fr... (2775)	TCAGAGGACTCGGCTCCCCTGGAGGGAGGAAATGGGCAAGAATGTCCACAGCAAGGTACGCGATCTGAGGATTCA
Lycium barbarum isolate Lr01 chromosome 6 NC_08334... (2660)	TCG------TGAGCTTACAAGCTCGGAAGATCA----AATCATTTCAAAGACAACATACTCGTATAGAGGATTTA

Section 40

(2926)	2926 2940 2950 2960 2970 2980 2990 3000
Homo sapiens chromosome 17 NC_000017.11 region fr... (2850)	CCCTCCGTCCAGGGCCAAGTCAAACCAGGAGAGACGGTGGCCAGGGCTGCAGGAGGGATCTGCCTCTGCCACGGT
Lycium barbarum isolate Lr01 chromosome 6 NC_08334... (2725)	GA-----TGTTTGGCCAAAG--AACCATGCCTTATGAAAGAAGGGTTAGCCTTACATACCTTTCTGTTGCACTAT

Section 41

(3001)	3001 3010 3020 3030 3040 3050 3060 3075
Homo sapiens chromosome 17 NC_000017.11 region fr... (2925)	CATCCAAATGAGCTTCCTGGCTGCCGGAAGCCAAGAGGATGTGACAGATGCTACATCCTGGGGGTCTCAAAACTC
Lycium barbarum isolate Lr01 chromosome 6 NC_08334... (2793)	TCTACACTTGCGCGTTCTC----CTCTAATGCTAGCGTTTCTATCTTCATTGAAGTCATACTATCATTAGAATTG

Section 42

(3076)	3076 3090 3100 3110 3120 3130 3140 3150
Homo sapiens chromosome 17 NC_000017.11 region fr... (3000)	CTGGGACTCAGCTCCCAGCTA--CCAGGTATCCTCCACTGCCCACCCCGATAGAGCACAGGACCACAAGCAAAAA
Lycium barbarum isolate Lr01 chromosome 6 NC_08334... (2864)	ATAGCTAGCATATATGACTAAAGCTAGAGAAAATCGGACAGCATCTCCTCCATAACGTATCTCAACTCCCAAACG

Homo sapiens chromosome 17 vs. Lycium barbarum isolate Lr01 chromosome 6

		Section 43
	(3151)	3151 3160 3170 3180 3190 3200 3210 3225
Homo sapiens chromosome 17 NC_000017.11 region fr...	(3073)	CAACTTTTGTGATCAAGGACAGCAGAGGCCCAGGGTGGATGAGGTCCAGAGATGCCTGCTTTATTTCCTACGGGG
Lycium barbarum isolate Lr01 chromosome 6 NC_08334...	(2939)	TCAATAATAAATTCATG-ATGCCATAACAATAGTCATTACTCATTCACATTATCCATATTTCTAGACTTACATTC

		Section 44
	(3226)	3226 3240 3250 3260 3270 3280 3290 3300
Homo sapiens chromosome 17 NC_000017.11 region fr...	(3148)	TGATCGCATTTCAGGCTGGTAACATCACATAACAAGTCTAATCGCCAAC-TAGTTGTACGCCTCTGAACTACAAG
Lycium barbarum isolate Lr01 chromosome 6 NC_08334...	(3013)	AATTCATCCACAACCATGGCCATAACACACACGACGTCTATTTACATACATTGTTCATCTCATGTTCTCAACATC

		Section 45
	(3301)	3301 3310 3320 3330 3340 3350 3360 3375
Homo sapiens chromosome 17 NC_000017.11 region fr...	(3222)	TCAACAATCCAAT--TCAGCAAGGAACAACAGTGTCAACAGCTCATTAGCGGGTGGGGACCGGATTTC-CAAAT
Lycium barbarum isolate Lr01 chromosome 6 NC_08334...	(3088)	ATTTATATCGTATCTACATCACAATACATCAAGACCCATAGCTCAATCCATACTATTTCTCAAAATGACACTATT

		Section 46
	(3376)	3376 3390 3400 3410 3420 3430 3440 3450
Homo sapiens chromosome 17 NC_000017.11 region fr...	(3294)	CAATGGTCTTGGCTGGCAGAGTGCTGAGTGGGGCCTGAGTGAATGAGGCTAAAACGCTTGGCCGAAGCCGTC--C
Lycium barbarum isolate Lr01 chromosome 6 NC_08334...	(3163)	CCATATTCATGACT--CATTTTTCTATACTCTTCTACAATCCAAGTATTTCAA-CTCTTAACTACCTTCAACAAC

		Section 47
	(3451)	3451 3460 3470 3480 3490 3500 3510 3525
Homo sapiens chromosome 17 NC_000017.11 region fr...	(3367)	ATCCCCA-ATCCTGAGAACAGAGTTGTCTGTTTCTCTTTCGGGGGTCAGGTCAG---CC-CAAGTAAGAGGCCAA
Lycium barbarum isolate Lr01 chromosome 6 NC_08334...	(3235)	ATAGAAATATCATGAATCTTACCTTAAATGTTGTAGGAACAAGCTTTAGTTGATAATACTCCACTTTGAG--CAA

		Section 48
	(3526)	3526 3540 3550 3560 3570 3580 3590 3600
Homo sapiens chromosome 17 NC_000017.11 region fr...	(3437)	CAACCTACCTGGGC-CGAT----ACAAGGCGACACAAGGCCCACCCG-TGGCTGACGCGCCCTCCA--CCCTCAC
Lycium barbarum isolate Lr01 chromosome 6 NC_08334...	(3308)	AACCCTAGTTTATCTCCATTTGGATTTCTTGACCTTGGATGATCTTGATGGTTTTCTTACACTTGATTCCCTTGA

		Section 49
	(3601)	3601 3610 3620 3630 3640 3650 3660 3675
Homo sapiens chromosome 17 NC_000017.11 region fr...	(3504)	TCCATCCCGGGGCACAGCTCCT-TCCGCTGGTGCTTA-GGAA---AAGGCGAGGCTTCTGGTAATTTCTCATCTT
Lycium barbarum isolate Lr01 chromosome 6 NC_08334...	(3383)	TTTATGTTGTTGATCACTAAATATCCTTGAATTCTTATGGAATATATGTAGAGAGATGTTCTAGAGAGA-AGGGG

Homo sapiens chromosome 17 vs. Lycium barbarum isolate Lr01 chromosome 6

Section 50

```
                                                      (3676) 3676          3690      3700      3710      3720      3730      3740     3750
Homo sapiens chromosome 17 NC_000017.11 region fr...  (3574) TGTAGTTCTGCTTTGAAAAGAAAAGGGGGCCGGGCACGGCGGCTCACGCCTGTAATCCCAGCACTTTGGGAGGCC
Lycium barbarum isolate Lr01 chromosome 6 NC_08334... (3457) TGTGATGTAGAAGTGAAATGAAATAAGCCTTGGTCCCCTTATTTAATGACTTAAAAATCTGATTTGAAGTGCACA
```

Section 51

```
                                                      (3751) 3751     3760      3770      3780      3790      3800      3810          3825
Homo sapiens chromosome 17 NC_000017.11 region fr...  (3649) GAGGCGGGTGGATCACCTGAGGTCAGGAGTTCGAGACCA--GCCTGGCCAACATGGT-----GAAACCCCATCTC
Lycium barbarum isolate Lr01 chromosome 6 NC_08334... (3532) GTTGTCGTAAACTTGGTTTACGGCCCGTATTCCAGTTTACGGACCGTCCTTCGTGGTCGTATTTAATCATATCAG
```

Section 52

```
                                                      (3826) 3826          3840      3850      3860      3870      3880      3890     3900
Homo sapiens chromosome 17 NC_000017.11 region fr...  (3717) TACTAAAAATACAAA-----AATTAGCCAGGTACGGTGGCGGGT----GCCTGTAATCCCAGCTAC--TCGGGAG
Lycium barbarum isolate Lr01 chromosome 6 NC_08334... (3607) AACCAGAAACTCAAACTGAGATGTGGTGATTTACGGACGCAGTTTACGGGCCGTAAACCACTTTACGGTCCGTAC
```

Section 53

```
                                                      (3901) 3901     3910      3920      3930      3940      3950      3960          3975
Homo sapiens chromosome 17 NC_000017.11 region fr...  (3781) GCTGAGGCAGGAGAATCACTTGAACCTTGGGGGCGGAGGTTGAAGTGAGCCAAGATGGTGCCACTGCACTCCAGC
Lycium barbarum isolate Lr01 chromosome 6 NC_08334... (3682) ATTGGGTCGTTTTTAGCCATTTGCTCATTTGACAAAAAGTTGAAGTTTTGGAAGCCAAAAT-ACGACATGGCAGT
```

Section 54

```
                                                      (3976) 3976          3990      4000      4010      4020      4030      4040     4050
Homo sapiens chromosome 17 NC_000017.11 region fr...  (3856) CTGGGCGACAGAGCAAGACTCCATCTCAAAAAAAAGAAAATAAAAGGGAATGGCAGGGGTGATCGAACGTGGGTG
Lycium barbarum isolate Lr01 chromosome 6 NC_08334... (3756) TTACG-GACCGTATACCACTTTACAGTCCGTATTTCATTTTACGACC-ACTGGCCAGAATG--CAAACTTG----
```

Section 55

```
                                                      (4051) 4051     4060      4070      4080      4090      4100      4110          4125
Homo sapiens chromosome 17 NC_000017.11 region fr...  (3931) TGGGCACCTCAGCAGAGATCAGGGGCCTTGGGCACAGAGCTGGGTGGAGCGAGAGGTCCGTGCTGGTGCAGAGGT
Lycium barbarum isolate Lr01 chromosome 6 NC_08334... (3823) ----CAACTTTTCACATTTCCAAAACCTATA--ATTATTCATTATGGAGCATAACCTCTCTCTTATTGCGATTGC
```

Section 56

```
                                                      (4126) 4126          4140      4150      4160      4170      4180      4190     4200
Homo sapiens chromosome 17 NC_000017.11 region fr...  (4006) GCTCCTCCTAGGACTCAGGG----CTAAGTTTCTTGGGAAGATGTCCAAAGA--GAAGGAT--TCGTTAACAGAA
Lycium barbarum isolate Lr01 chromosome 6 NC_08334... (3892) CTTCGGAATCTCACTTAGGGTCGTCAAATGTTATTTCTTACTCGCGGAAACATCGTACGCTCGTACTCATCACTA
```

Section 57

	(4201)	4201 4210 4220 4230 4240 4250 4260 4275
Homo sapiens chromosome 17 NC_000017.11 region fr...	(4073)	TTTCAGGATCGTGGGAGC--------TGTTTGTATGATATGAACCATTA------CCTTAGCTACATTTTTCTTC
Lycium barbarum isolate Lr01 chromosome 6 NC_08334...	(3967)	GTTCATTCACTTGCGTACCAACGGAATATTTTTCCGAGGTGTAACATTCTTCCCCCCTTTAGAACATTCGTCCTC

Section 58

	(4276)	4276 4290 4300 4310 4320 4330 4340 4350
Homo sapiens chromosome 17 NC_000017.11 region fr...	(4134)	TCACCCAAAAAACCTCTACAAGCTTCCTTTTGCATTTGGAGATCCCCTGCCTGAGAACACAAGAGTGGGCTGCTT
Lycium barbarum isolate Lr01 chromosome 6 NC_08334...	(4042)	GAATGTTTAAGTCTTCGGGGA--TTCTATAAAAATTTCGCCAGAGTTTCCCCTATAATATGGCACTACCATCCTG

Section 59

	(4351)	4351 4360 4370 4380 4390 4400 4410 4425
Homo sapiens chromosome 17 NC_000017.11 region fr...	(4209)	TTCTCTCAGACCGAGGAAGACTGTGTTGCTTAGAAA-AGGACGTCCTCACCCGCAGCTCTAAGC--GGAGGCCCT
Lycium barbarum isolate Lr01 chromosome 6 NC_08334...	(4115)	TCACAACA-ACCCATAATACCGATGCCTCACAGGGCCACAACGCAATAGCATACAAGTTTGGCCACGCACGACCC

Section 60

	(4426)	4426 4440 4450 4460 4470 4480 4490 4500
Homo sapiens chromosome 17 NC_000017.11 region fr...	(4281)	TTCTCAGCTCAGCCTCCACGCAGGTGTCACCCGGCGGTTCCCCTCTGGC----CGTCCGGGGCCGTGTGTGCCGA
Lycium barbarum isolate Lr01 chromosome 6 NC_08334...	(4189)	AAAAAGCATAAAAGTGAGTTAAGGGGTACCCGGGTGGGCTAGCACCGGGTGCTCGTCACGGCCCCTTAATTGGGT

Section 61

	(4501)	4501 4510 4520 4530 4540 4550 4560 4575
Homo sapiens chromosome 17 NC_000017.11 region fr...	(4352)	CGT--CAGAGTCAGAGCCCGAGCGG----GACCCACCGGCCCTCCACGCTCACTCTGCCCAGGACACCCTGGCAG
Lycium barbarum isolate Lr01 chromosome 6 NC_08334...	(4264)	CGTGACAAAAGTGGTATCAGAGCAGTTCAGTCCTAGGGTGTGTCTACGAGCCGTGT--CTAGTAGAGTCTTGGTT

Section 62

	(4576)	4576 4590 4600 4610 4620 4630 4640 4650
Homo sapiens chromosome 17 NC_000017.11 region fr...	(4421)	ATCCTTCACCAAAAGCTAGTGACTTTGCGAAAGCCATGCAGCCCCGCTC-TGGCCGGGCCCCCGGCCATCCCGTG
Lycium barbarum isolate Lr01 chromosome 6 NC_08334...	(4337)	ATGGGTGTGTT---GCACGCCACACTTATAAA--CAAGAAGCTGCGGACATTTTAGGAATAAATGGCCTTCTTTT

Section 63

	(4651)	4651 4660 4670 4680 4690 4700 4710 4725
Homo sapiens chromosome 17 NC_000017.11 region fr...	(4495)	CTCCCAAACAGAGTCTCCAAACACGGTGGACTCTGCCGCTGCTGATTCCACACCCCAGGGTGCTGGCTG--AGGA
Lycium barbarum isolate Lr01 chromosome 6 NC_08334...	(4407)	CTTGATAAGAATCGTGCGATAGAGCTATGATGTGAGAAATTCTTGTTCCTTAATCGTGTGTTGTGTATTTCAGAA

Homo sapiens chromosome 17 vs. Lycium barbarum isolate Lr01 chromosome 6

```
                                                                Section 64
                                                   (4726) 4726          4740      4750      4760      4770      4780      4790    4800
 Homo sapiens chromosome 17 NC_000017.11 region fr... (4568) GTTTACTGCA-GAAACAAGGCACCACAGAGAGGCTGGACACTTGGGTGGTGGGATTCGGGGCACGCTGG-GTAAG
Lycium barbarum isolate Lr01 chromosome 6 NC_08334... (4482) ATTCCTAGAAAGAGAAAAGCTACAGCTGCCCCAAAGGGTAAGACGGTGGCAGAAAAGCGGGCTGAAAAAAGCACC
                                                                Section 65
                                                   (4801) 4801     4810      4820      4830      4840      4850      4860           4875
 Homo sapiens chromosome 17 NC_000017.11 region fr... (4641) TCCAAGGGCGGAAGGAAGGAGGGGGAGGAGGAGAAGGCTGAGTTTTAAATAACGTCTCAGGAGGCCGGAGCC-AC
Lycium barbarum isolate Lr01 chromosome 6 NC_08334... (4557) GCCACCGGTAGTAG--AGAAAAGTGAGTCCCAGAGTGCGGCTCAATCCCAATCCTCTC----GTTCAGTGCCTAT
                                                                Section 66
                                                   (4876) 4876          4890      4900      4910      4920      4930      4940    4950
 Homo sapiens chromosome 17 NC_000017.11 region fr... (4715) GCTAGATGGGCTCCGGTTTGAGCTTCTCTGCTAGGAAGTCATTCACAAAGGCCTCCAC--GATGGCGGGGGTGAT
Lycium barbarum isolate Lr01 chromosome 6 NC_08334... (4626) ATTAGAGGAGCGTGAGG--GAGCCTCAGCCCCAGCTCCAACACCTCCAGCTCCTCCACCGGATGCTTCGGGCCAA
                                                                Section 67
                                                   (4951) 4951     4960      4970      4980      4990      5000      5010           5025
 Homo sapiens chromosome 17 NC_000017.11 region fr... (4788) CTCCTCCACGT--CCATCACGTACTTGGCCCGGGCTGACATGTCCAGGATGGTGAG-CAAAG----GGGCAGCCT
Lycium barbarum isolate Lr01 chromosome 6 NC_08334... (4699) TATGTGAAAGAGGCCATTAATTTGCTGACTCAATTGGTTACGGCCCAGACTCAGAGGCAAAGTTCAGGGCAAGGT
                                                                Section 68
                                                   (5026) 5026          5040      5050      5060      5070      5080      5090    5100
 Homo sapiens chromosome 17 NC_000017.11 region fr... (4856) C--AGGCAGGTTGGTGTAATCTCGCAGGGAGTCAGTCATGT----CATCCTGAAGCC----G-TC--AGGAGGGA
Lycium barbarum isolate Lr01 chromosome 6 NC_08334... (4774) GATAGGGCTGTTAGTGCAAGGGCCCGTGACTTCATTACTTTAAACCCTCCGGAATTCTTTGGGTCAAAGCCGGAA
                                                                Section 69
                                                   (5101) 5101     5110      5120      5130      5140      5150      5160           5175
 Homo sapiens chromosome 17 NC_000017.11 region fr... (4918) GAGAAAACCAAGAAGTT--TTAAAGAAAGGAGGGGGAGAGTCCCCTGGGCCCAGTCTCC----CCAGGTGTGGTA
Lycium barbarum isolate Lr01 chromosome 6 NC_08334... (4849) GAGGACCCTCAGGATTTCATTGATGGTATGTTGAGGACGCTTCAATTGATATATGCTTCGGACACCGAATCGGTG
                                                                Section 70
                                                   (5176) 5176          5190      5200      5210      5220      5230      5240    5250
 Homo sapiens chromosome 17 NC_000017.11 region fr... (4987) GCTCCCGCACCCTGAAGAGCCGGCGTT-TCCCAAGCAGAGGGCTCCCAGCCTGGGAAGGA------GGGAACCC-
Lycium barbarum isolate Lr01 chromosome 6 NC_08334... (4924) GTGTTAGCGTCATATAGATTGAGAGATGTCTCGATCC-AGTGGTATACGGTTTGGATGGCTTCACGGGGAGCCAA
```

Homo sapiens chromosome 17 vs. Lycium barbarum isolate Lr01 chromosome 6

Section 71

	(5251)	5251 5260 5270 5280 5290 5300 5310 5325
Homo sapiens chromosome 17 NC_000017.11 region fr...	(5054)	TGCCCTGCCTTCCGGA-GGAAGGGGCTGCTTTGTAAAATTCTCT--CTTTGCATCCGCCCTGCAAACCA-CTAGG
Lycium barbarum isolate Lr01 chromosome 6 NC_08334...	(4998)	TGCACCTCCCCCGGTATGGCAAGAGTTCGTTGATGCTTTTCTCCGACACTATATGCCTCCGGAAGTTCGGCGAGC

Section 72

	(5326)	5326 5340 5350 5360 5370 5380 5390 5400
Homo sapiens chromosome 17 NC_000017.11 region fr...	(5125)	TA-AGTCG-TCTGTATTTAATTATTTATGTATTTATTAGCATCAACACCATTTTAATACGTCCTCAGCATTTTAG
Lycium barbarum isolate Lr01 chromosome 6 NC_08334...	(5073)	TAGAGCCGATAAGTTCTTAAAT-TTGAGGCAAGG--TAGTATGAGTGCTCTAG-AGTACAGTCTCCGCTTCAATT

Section 73

	(5401)	5401 5410 5420 5430 5440 5450 5460 5475
Homo sapiens chromosome 17 NC_000017.11 region fr...	(5198)	AAATGTCACATTCTTTTTTTTTTTTTTTTTTTTGAGATGG-AGTCTCGCTCTGTCACCCAGGCTGGAGTGCAGTG
Lycium barbarum isolate Lr01 chromosome 6 NC_08334...	(5144)	CCTTGGC---TAGGTATGCTTCGGCCATGGTAGCAGATGTCACACCCCTTTTTTTAACCGCGGAGAGTTAAAGTT

Section 74

	(5476)	5476 5490 5500 5510 5520 5530 5540 5550
Homo sapiens chromosome 17 NC_000017.11 region fr...	(5272)	GCACGATCTCGGCTCACTGCAAGCTCCACCTCCCGG-------GTTTAAGCAATTCTCCTGCCTCAGCCTCCTGA
Lycium barbarum isolate Lr01 chromosome 6 NC_08334...	(5216)	AGAAG-TAT-GACGTATTGGAGATTCCTGTTTTTGTTTATTTTGTTTAAGGAGT-CGCCACCTAATTATTTATGG

Section 75

	(5551)	5551 5560 5570 5580 5590 5600 5610 5625
Homo sapiens chromosome 17 NC_000017.11 region fr...	(5340)	GTAGCTGGGACTACAGGCGCCCACCAC-GCCCGGCTAATTTTTTACATT-TTTAGTAGAGACGGGGTTTCACCAT
Lycium barbarum isolate Lr01 chromosome 6 NC_08334...	(5288)	TGAATTAGGACACCTAAAGTTTATTAAAGTTATGTTTAAAGTTAACTCTGTTTAAGGTCTGCGAAACTTAAGATT

Section 76

	(5626)	5626 5640 5650 5660 5670 5680 5690 5700
Homo sapiens chromosome 17 NC_000017.11 region fr...	(5413)	GTTGGTCAGGCTGGTCTCCAACTCCTGACCTCAGGTGATCCGCCTACCTTGGCCTCCCAAAGTGCTAGGATTACA
Lycium barbarum isolate Lr01 chromosome 6 NC_08334...	(5363)	CTAGGTAAGGGT--TCAATTAGTCTAAAGGGAAGGT-ATTAGGCACCCTTTAAGACCCAT----------TAACA

Section 77

	(5701)	5701 5710 5720 5730 5740 5750 5760 5775
Homo sapiens chromosome 17 NC_000017.11 region fr...	(5488)	GGCGTCAGCCACCGTGCCAGTCTAGAAATGTCATAGCCTC--TGATTGTTTATTTTTGTAACCAACTGTCAATGA
Lycium barbarum isolate Lr01 chromosome 6 NC_08334...	(5425)	ATGGTTA---ACCGA-CCGGACTTAAAATTAATTAGGCTAAATGCGAATATAGTATTATAGGAAAAAGGAAAGTA

Homo sapiens chromosome 17 vs. Lycium barbarum isolate Lr01 chromosome 6

Section 78

```
                                                    (5776) 5776          5790      5800      5810      5820      5830      5840     5850
Homo sapiens chromosome 17 NC_000017.11 region fr... (5561) --TTTGCAGCCCCACTGTGGTTA--TCTGAGGCCAATCTG---GACTTCAACTCTGGAATGTTTTTAAACAGAG
Lycium barbarum isolate Lr01 chromosome 6 NC_08334... (5496) ATTTTGTA-----AGTATGGCTAAAGTTATAGATAAATATGTAAGAATGCTATTTAAAATAGAACTTGTAAAATA
```

Section 79

```
                                                    (5851) 5851     5860      5870      5880      5890      5900      5910          5925
Homo sapiens chromosome 17 NC_000017.11 region fr... (5629) ACAGCTCGCTGTGTTCCA-ACTCCCACCCCTGTGGTTTCAGGCAGGACAGGAGCTGCACA-GCCAGCCAGTTCAG
Lycium barbarum isolate Lr01 chromosome 6 NC_08334... (5566) ATAATAATTTGTATAAAAGAGTACTTTTAACGTTATTTT--GAAAAATACGACTTATAAATGTTGTTAAGATTTT
```

Section 80

```
                                                    (5926) 5926          5940      5950      5960      5970      5980      5990     6000
Homo sapiens chromosome 17 NC_000017.11 region fr... (5702) AAGACAGCGCTGCTTAAGCACTGTGGAAGCTTACGCTGCCTCACTACTTATCTGAGAAAGAAGCTGGCACTTTAT
Lycium barbarum isolate Lr01 chromosome 6 NC_08334... (5639) TAAACGAAAGTGTAATAATAATGTTTAAGATAATACTTGTAGAAAATGTAGTGATTTAATAAGAGTTTAGTTATA
```

Section 81

```
                                                    (6001) 6001     6010      6020      6030      6040      6050      6060          6075
Homo sapiens chromosome 17 NC_000017.11 region fr... (5777) CCTACATTCCCAGCAGCTGTCCTGGTGAATTTCCCTAATAGCAGACATAGCCTCATGCAGACACCAAGGCAAACA
Lycium barbarum isolate Lr01 chromosome 6 NC_08334... (5714) AATAAATCAAAAGAAACGGAATGAGTAATATTCATATGTATCCCGAATAGGA--ATTGCGCCAACTAGC------
```

Section 82

```
                                                    (6076) 6076          6090      6100      6110      6120      6130      6140     6150
Homo sapiens chromosome 17 NC_000017.11 region fr... (5852) CAGAAATCCAAAGACAAGGAGAAAGGATTTGTAAGGGAGCTACTTTTTTTTTTGGAGATAGAGTCTCTTTCTGT
Lycium barbarum isolate Lr01 chromosome 6 NC_08334... (5781) ---AAATTAAAATGTATTTGTAAAGTTATTGTAAGATTAATA-TTAATGTTTTAACAAAT---GTGTATTTCAAG
```

Section 83

```
                                                    (6151) 6151     6160      6170      6180      6190      6200      6210          6225
Homo sapiens chromosome 17 NC_000017.11 region fr... (5927) TGCCCCAGCTGGAGTGAAGTGGCGCAGTCATAGCTCACTGCAGCCTCCACCTCCTGGGCTCAAGCAATCCTCCCA
Lycium barbarum isolate Lr01 chromosome 6 NC_08334... (5849) CGTTCTA-----AGATAAAAGAGTGAAT-ATTGATTCTTTTTGACTCAAATATATAG--TCATGCTATT-TAAAA
```

Section 84

```
                                                    (6226) 6226          6240      6250      6260      6270      6280      6290     6300
Homo sapiens chromosome 17 NC_000017.11 region fr... (6002) CCTCAGCCTCCTGAGTAGCTGGGACTCTAGCCATGCAGCACCATGCCTGGCTAATTTTTGTACATTTTTTGGAGA
Lycium barbarum isolate Lr01 chromosome 6 NC_08334... (5915) ATTATTCCAAATAAATGTTATAGATGCTATATAAGTAGTTTAGAACATAACTAGAAGT-GAATGTAATTTGTGGA
```

Homo sapiens chromosome 17 vs. Lycium barbarum isolate Lr01 chromosome 6

Section 85

```
                                                         (6301) 6301     6310      6320      6330      6340      6350      6360          6375
Homo sapiens chromosome 17 NC_000017.11 region fr...     (6077) CACAAAAGTCCAGCCCAGCCAGACGGGAGCCAGCAAACTGCCTT-GGAGCAGCCCCTCGTCCACTCTGAACCTTG
Lycium barbarum isolate Lr01 chromosome 6 NC_08334...    (5989) ATTAACTACCCAT---AACTAAACTAAAACCCTTAATGTCACTAAGGTTTATCTAAATTAACAAACAACAAAATG
```

Section 86

```
                                                         (6376) 6376          6390      6400      6410      6420      6430      6440     6450
Homo sapiens chromosome 17 NC_000017.11 region fr...     (6151) CCTCCCTCCCCTGTGAAACAGGACAGCGACTACGTTCCCAGCCAGAGAGCCAGGAGCGAAGGAGACAGCCCAGGT
Lycium barbarum isolate Lr01 chromosome 6 NC_08334...    (6061) TTAGTCATACAAGGTAAACAAGAAATACAACAAAATAAGATAGGAGGAAAGAAGAGAAAATGGAACAGTCCATTT
```

Section 87

```
                                                         (6451) 6451     6460      6470      6480      6490      6500      6510          6525
Homo sapiens chromosome 17 NC_000017.11 region fr...     (6226) --G-AAATATGC----GGCTCTGTGCCTGGCGTACAGAAAACCTGCAAATGCTCCTTCCAGACTTGACACCTCTC
Lycium barbarum isolate Lr01 chromosome 6 NC_08334...    (6136) ATGGACTGCTGCTGCAGCCTATTTTGCTATTGGGCTTCAGCCCAGAAAGTCC-CTTTCTTG--TTGTTGTTGTTG
```

Section 88

```
                                                         (6526) 6526          6540      6550      6560      6570      6580      6590     6600
Homo sapiens chromosome 17 NC_000017.11 region fr...     (6294) AAGGCCGCCCCCGCCCGTGCCCGGCCCCTGCTGTGCTGCAGCCATCTTTTCTCAGAGCCATTCTGCAGGGGTTGG
Lycium barbarum isolate Lr01 chromosome 6 NC_08334...    (6208) TGGGCAGGCT--GGACAAGAAGAGGCCATGTTGGGCT-------TTTATCCCAACACCAAACACGGAGGAAGACG
```

Section 89

```
                                                         (6601) 6601     6610      6620      6630      6640      6650      6660          6675
Homo sapiens chromosome 17 NC_000017.11 region fr...     (6369) AGTCACTGCATGCCGCGCAGCCTCCCTGGGGCTGGGCTTGGT-GTGCAGTGGCCAGT--GCCACCCCGAGGCAGG
Lycium barbarum isolate Lr01 chromosome 6 NC_08334...    (6274) AGTCCGAGGGACTCGTATGCGATAGTCATGCATAAACACGAAAGAAAAACGATTAGTATACAATCAATAAACAAG
```

Section 90

```
                                                         (6676) 6676          6690      6700      6710      6720      6730      6740     6750
Homo sapiens chromosome 17 NC_000017.11 region fr...     (6441) GCCTGTGAACCTCTTTGCTCCCCTCCACCCCCGGGCTGAGCAGGTGACTGGAGCCGCCTGGGAAGGACCCAGGTG
Lycium barbarum isolate Lr01 chromosome 6 NC_08334...    (6349) G--TTTAAACATGTAAAATATAACTCAAAACAAATC-GAT-AGATTA-TGTATACAC-TGTGTATAGTCGAGTAT
```

Section 91

```
                                                         (6751) 6751     6760      6770      6780      6790      6800      6810          6825
Homo sapiens chromosome 17 NC_000017.11 region fr...     (6516) CTGGGCTGGGCAGGGCCGGGGGCTGAGGTGATGAATGGGTGGCACAGGG-TGT-CTGGCTGCCGCCTGCCTCCTC
Lycium barbarum isolate Lr01 chromosome 6 NC_08334...    (6418) ATCTCATGTATACACATTCATGAAGAAAAGATTAA-GGAAAGCACATGAATATACATTCAGTCATATGCCCAAAC
```

Section	Sequence	Start	Alignment
Section 92		(6826)	6826 … 6840 … 6850 … 6860 … 6870 … 6880 … 6890 … 6900
	Homo sapiens chromosome 17 NC_000017.11 region fr...	(6589)	CGTGCCTCTCCTGGGATCCAAACACAGCGTGAAATCAGCACAGGGCTCTGACCAACTGGAGGTACAGATGGGTGG
	Lycium barbarum isolate Lr01 chromosome 6 NC_08334...	(6492)	CGC-CTTGACCAATTTT--ATGGAAAGCACTTAAT--GTTCAGCG----GACATAGTCAACGAACAGCAACATGG
Section 93		(6901)	6901 … 6910 … 6920 … 6930 … 6940 … 6950 … 6960 … 6975
	Homo sapiens chromosome 17 NC_000017.11 region fr...	(6664)	ATGGAGGGCAGGGGGCGGCCTCCAAAACTGTCTGT-CAGTCAGTGGGGTGGGGTGGGGTGGGGAGGTGGCAGCGC
	Lycium barbarum isolate Lr01 chromosome 6 NC_08334...	(6558)	AT------CAAGTAGAGCATTATGAATCTGATTAAGCATATAGCAAGAAAAAGCAAGCTCTTA--GAGAAATCGT
Section 94		(6976)	6976 … 6990 … 7000 … 7010 … 7020 … 7030 … 7040 … 7050
	Homo sapiens chromosome 17 NC_000017.11 region fr...	(6738)	AGGCATG-GAGGAGGCTCCT--GGGTTGGAGGAGCTCAAGAATGAGAGCCTGCACCTGTGCTGAGTCGCGGAGAC
	Lycium barbarum isolate Lr01 chromosome 6 NC_08334...	(6625)	AGACATTTGTGGAAGCACTTTCAGGATGCAAGAAAGCAAATACCAAT---TGTAGAAGGACATAGCAGTTCTATA
Section 95		(7051)	7051 … 7060 … 7070 … 7080 … 7090 … 7100 … 7110 … 7125
	Homo sapiens chromosome 17 NC_000017.11 region fr...	(6810)	GCCCGTCTGCAGGGTGCAGGGTGCAGGGTTCCAGGCCCTGGATAACTGGGTACCTGCGGGAGGGCGCTCTGCTGA
	Lycium barbarum isolate Lr01 chromosome 6 NC_08334...	(6697)	TAACATTTAGACAGT--AAGGCAAACAGATTGAACCACT--ATGGATGGGT-----CTTAAGTACAGAATCTAGA
Section 96		(7126)	7126 … 7140 … 7150 … 7160 … 7170 … 7180 … 7190 … 7200
	Homo sapiens chromosome 17 NC_000017.11 region fr...	(6885)	AAGCCCCACTTTCCCACCCTGTGAAGAGTCTCCTGCCTGGTCCAGCCCCGCTGGAGACCTCAAAGGGACAGC-CT
	Lycium barbarum isolate Lr01 chromosome 6 NC_08334...	(6763)	CAGTCAAACAACCTCTCCAGGTGCT-AGT----TAACCCATCAAGCAT-GCTGCCTAAGTGTTCTCGGCAGCTCT
Section 97		(7201)	7201 … 7210 … 7220 … 7230 … 7240 … 7250 … 7260 … 7275
	Homo sapiens chromosome 17 NC_000017.11 region fr...	(6959)	GGGTGAAACAGACGGGGAGCGGCAGCAATTTC--CAACCTCAGCACTCAC---CGGGC-CGGCTGGGTG--ACCG
	Lycium barbarum isolate Lr01 chromosome 6 NC_08334...	(6832)	GACTAAACAAGATATCCAACCAGCGCAATGTAATCAAACTAATCACTCACACAAGTACACAGATTCCTGCTAGCG
Section 98		(7276)	7276 … 7290 … 7300 … 7310 … 7320 … 7330 … 7340 … 7350
	Homo sapiens chromosome 17 NC_000017.11 region fr...	(7026)	CAG--GTATAAGCAGTAGCCCCAGCCAGGATCCCTCTGATGAGATCGGCCCACTGCAGCCAGCGCGGAGGCACGA
	Lycium barbarum isolate Lr01 chromosome 6 NC_08334...	(6907)	CAGGAGTGTAAATAACATACCCTTCTA---TACTTTACATGAGCTCACATAAATCCAACAG-GCATAATCATGA

Homo sapiens chromosome 17 vs. Lycium barbarum isolate Lr01 chromosome 6

Section 99

(7351) 7351 7360 7370 7380 7390 7400 7410 7425
Homo sapiens chromosome 17 NC_000017.11 region fr... (7099) ACCCAGCATTTTGTAAAGACTCAAGGGCCTGGTTGTGACCCAGGAGGTGTGGATCACACTTTGCAGCCACGGCCA
Lycium barbarum isolate Lr01 chromosome 6 NC_08334... (6978) ATTAAACAACTTG--ATGACCAGCTTACCCTTTTTCGGACTCAACAGAAACAGCTGTCA-TATAAAGTAATACCAA

Section 100

(7426) 7426 7440 7450 7460 7470 7480 7490 7500
Homo sapiens chromosome 17 NC_000017.11 region fr... (7174) CTCTCCCCAGCCGCTCAGGCGGGAAGCAGCCTGGGGACGGCTCCCTGCTGACCTGAGGCTGCCGAGCATGGACGG
Lycium barbarum isolate Lr01 chromosome 6 NC_08334... (7050) ATGAAAAGATTT-CACATTATCAAAGGA-CTTGGATACAACACAAAGGGGAAATG-GCATGTGTTTCAACCATAG

Section 101

(7501) 7501 7510 7520 7530 7540 7550 7560 7575
Homo sapiens chromosome 17 NC_000017.11 region fr... (7249) GAAGAGGAAGCAGCAGGGGAGGGAATGGTTTTCCATGGCGAATGGCTACCTTTCCTTGCCACATTTGTCTGTGGG
Lycium barbarum isolate Lr01 chromosome 6 NC_08334... (7122) GGGACTGATTCATCATACGAA---------TTTAAA---CACAAAGATCAATTAGAGTG--ACATATGCAAATGAG

Section 102

(7576) 7576 7590 7600 7610 7620 7630 7640 7650
Homo sapiens chromosome 17 NC_000017.11 region fr... (7324) CTCTCTCAGAAGCACAGGCAGCCCTGACCCTGG--GGTCCTTTCAGGCAACCCTGGGGCCAGGATCAGTCCTGTG
Lycium barbarum isolate Lr01 chromosome 6 NC_08334... (7184) CTTACT-AGCA-CATGAGCAGGCCTAAGTATGCAAGAGAGATGCAGGAGGAAATGCATAAGTGTATACATATGAA

Section 103

(7651) 7651 7660 7670 7680 7690 7700 7710 7725
Homo sapiens chromosome 17 NC_000017.11 region fr... (7397) CCAGAAGTCCCAGCTGAGCCAGCCCTGGGCCAAGCCTCTCCTCCGCACTCACCTCCCCGGCTACGAAGAACAGAA
Lycium barbarum isolate Lr01 chromosome 6 NC_08334... (7257) ATTCATATCAAAGACCTTAATGACACCAAACAATAATAACCAAATCAAACACATACTCCTTTGAAGGGCAAAGAA

Section 104

(7726) 7726 7740 7750 7760 7770 7780 7790 7800
Homo sapiens chromosome 17 NC_000017.11 region fr... (7472) GGGGTGCCTCCTCCTCT-TTGGCTTTGTACTTGGCAATGATTTTCT--CAGCTATCGGCTGAATCAGCTGCTTGG
Lycium barbarum isolate Lr01 chromosome 6 NC_08334... (7332) GAGTAGTAACAGCAGCTGTAGGCAAAGCAAAAGGGAAGAGGTAACAAACAGCAGT-GACAGAATTAG-TGCAGGG

Section 105

(7801) 7801 7810 7820 7830 7840 7850 7860 7875
Homo sapiens chromosome 17 NC_000017.11 region fr... (7544) CCGCCTCGGAC-TCTCCGTCATCCTCAG----------AATCTGTGATTGGGTCGG--G----GGTAGAAAAAGA
Lycium barbarum isolate Lr01 chromosome 6 NC_08334... (7405) GGGGTTGATAAATTTGGGTTGTTAGCAGGGCAAGGTACAAGTAGCAATTATAACATGTGAATTGGCAGAGATTTG

Homo sapiens chromosome 17 vs. Lycium barbarum isolate Lr01 chromosome 6

```
                                                         Section 106
                                          (7876) 7876          7890      7900      7910      7920      7930      7940     7950
Homo sapiens chromosome 17 NC_000017.11 region fr... (7602) CGGGGAGAAAAAGCACTGGCTTGTCAAACAGAGCGGCACCCGCCGAGGGGGCCTGAGCTGCAGAAACGCCCGCCT
Lycium barbarum isolate Lr01 chromosome 6 NC_08334... (7480) CGAGTATAACATGATCTTAAG-GTTAAATATAACTGTAACAATCAAGCAA-CAAGAT-TACAGATAACTCCTATT
                                                         Section 107
                                          (7951) 7951     7960      7970      7980      7990      8000      8010           8025
Homo sapiens chromosome 17 NC_000017.11 region fr... (7677) GAGCG----GCCCCTGAACGGAAATGAA--------CTTCCCCTTGGATGCAGGTCTTGCTCCCCGCATGCATGG
Lycium barbarum isolate Lr01 chromosome 6 NC_08334... (7552) GATCCTTATGAAAGTTAACTTAAATAATTTTTTAAGCATGATATTGTAT-CATATCAGTTTCGCAACAGAACAAA
                                                         Section 108
                                          (8026) 8026          8040      8050      8060      8070      8080      8090     8100
Homo sapiens chromosome 17 NC_000017.11 region fr... (7740) GTGTGTGTGCACATGCGTCCAGCAGCAAAGCTCAC-AGTGCTCTCCTGCACCGGGCTCCCCAGGAGGAACCTGC
Lycium barbarum isolate Lr01 chromosome 6 NC_08334... (7626) GAGAATGAGATCTTGAATCAAACTATAAGTGGAAAATAAGGCAGGCTTTAAGCATAATTCATCATAGAG---TGC
                                                         Section 109
                                          (8101) 8101     8110      8120      8130      8140      8150      8160           8175
Homo sapiens chromosome 17 NC_000017.11 region fr... (7814) CTCACTGAGCAGCTCTGCTCTCCCAGGTAGACAGTCT---TCCCATTGAATTCCTCACAAACCCGCCTGGGAGGC
Lycium barbarum isolate Lr01 chromosome 6 NC_08334... (7698) ATCTGAGAACACATTTAGACTATAAACTAGGAAGGAAGAGTCCTATTCTATTCCAAACTAACCCATCACAAATGT
                                                         Section 110
                                          (8176) 8176          8190      8200      8210      8220      8230      8240     8250
Homo sapiens chromosome 17 NC_000017.11 region fr... (7886) TGA-------GCTTCCCCTAA-GCC------AGGACCTGAGGCGCCTGCTAGTGTTTCTCTCAGCCACAG---AA
Lycium barbarum isolate Lr01 chromosome 6 NC_08334... (7773) CAAAAAAAGTGAATCATCTAATGTCTTTAAAAAGATCACACAAACTATTATTTATTTCTTTGAACAGAAGGGTAA
                                                         Section 111
                                          (8251) 8251     8260      8270      8280      8290      8300      8310           8325
Homo sapiens chromosome 17 NC_000017.11 region fr... (7944) TGAGAAGCC-----ATGACCTCCGACCTCCGACTTCCCAGTGAAA--ATGGC-AGAA-----GAACTGGTGGTGA
Lycium barbarum isolate Lr01 chromosome 6 NC_08334... (7848) AGAGAAGACGATAAATGAGCATATTGCAGTCACTTGACTAAAAAATGAAGGCTAGAAAGATTCAAATGAAACAGC
                                                         Section 112
                                          (8326) 8326          8340      8350      8360      8370      8380      8390     8400
Homo sapiens chromosome 17 NC_000017.11 region fr... (8006) GCGGGAGGGGGTGAGGACTGTGGTCTGGTCAGCGGCTTTTTTTTTTTTTTTTTTTTTTT--TTGAGACAGAGTC
Lycium barbarum isolate Lr01 chromosome 6 NC_08334... (7923) AAAGTAGAGATTAAAGAGAATGTTTTGGAAATGTGCTCATTTGTTCACTTATAAACAGCAAGGTAAAGCAAAGTA
```

Homo sapiens chromosome 17 vs. Lycium barbarum isolate Lr01 chromosome 6

Section 113

```
                                                             (8401) 8401     8410      8420      8430      8440      8450      8460          8475
      Homo sapiens chromosome 17 NC_000017.11 region fr...  (8079) TCGCTCTGTTGCCCAG----GC--------TGGAGTGCAGTGGTGCCATCTGGGCTCACTACA-----ACTTCTG
Lycium barbarum isolate Lr01 chromosome 6 NC_08334...       (7998) AAAC-CTTTATCAAAGTAAAGCATTTCTCATTTTGATCACACGACAAAAGGGGGAAAAGTAAACAGCGAATTAAG
```

Section 114

```
                                                             (8476) 8476          8490      8500      8510      8520      8530      8540     8550
      Homo sapiens chromosome 17 NC_000017.11 region fr...  (8137) CCTCCCCGGTTCAAGCGATTC--TCGTGCCTCAGCCTCCCGAGTAGCTGGGATTACAGGCGTGAGCCACCACACC
Lycium barbarum isolate Lr01 chromosome 6 NC_08334...       (8072) CATATT-GATCCAGATCATTCAATCGTGACTCAACCTGAA-ACTAAACATGATGA-AGGCAA-ACTAACAACAAA
```

Section 115

```
                                                             (8551) 8551     8560      8570      8580      8590      8600      8610          8625
      Homo sapiens chromosome 17 NC_000017.11 region fr...  (8210) CAGGCTCGT---ACAGTGATTTTGAGTTGTTGGAGGGGAGCCACAAGTCCAAGGCAGAGGGCCAGGTTT-TGCAA
Lycium barbarum isolate Lr01 chromosome 6 NC_08334...       (8143) CAGCTCTGTAAGACAGTGAAACTACGTCACATAAGCACTTAAGCGCGTCCTAGTAGCATGTTAAACTAAATAGCA
```

Section 116

```
                                                             (8626) 8626          8640      8650      8660      8670      8680      8690     8700
      Homo sapiens chromosome 17 NC_000017.11 region fr...  (8281) AAGGAACCAGGGTCATCCCATCAGACTGCAGGGAGCCTGGCATGGATGCGCCTGTGTCCCCCTGGCCCGGCTGGG
Lycium barbarum isolate Lr01 chromosome 6 NC_08334...       (8218) AATTAGGCATAGA-ACCACACCACACAA-AGGTTCAATTATACAGAGGCGAGAACATTCAATATACTTACATGCA
```

Section 117

```
                                                             (8701) 8701     8710      8720      8730      8740      8750      8760          8775
      Homo sapiens chromosome 17 NC_000017.11 region fr...  (8356) CAGTTACCCACGGAGCTCAGACTGGGTGAGA------TGGGAGACGGGGAGGTGCAAGCGAGGACAAAGGAGCCA
Lycium barbarum isolate Lr01 chromosome 6 NC_08334...       (8291) C---TACATACAG-GCATAAACATGCTAATAAAAACTTAAAATTAGCTGAACTG-AATTAAGGAATGAGCAGACA
```

Section 118

```
                                                             (8776) 8776          8790      8800      8810      8820      8830      8840     8850
      Homo sapiens chromosome 17 NC_000017.11 region fr...  (8425) AGGCAGCCACCGGGCTGGAGCAGCTCTTCAGAGCACAGAGGATCCGCAGAAGCAGCCTTGGTGAGCCTCGGCCAG
Lycium barbarum isolate Lr01 chromosome 6 NC_08334...       (8361) GATTCTTCTGGTAGCTA-AGCTGGAAACTAAACCATATTAAAACACGAATATCATTCAAG-TAAGATGCTGGACA
```

Section 119

```
                                                             (8851) 8851     8860      8870      8880      8890      8900      8910          8925
      Homo sapiens chromosome 17 NC_000017.11 region fr...  (8500) AGGGGCCCAGCCCCTGCAGCTGCCGTGTGT---GCCACCCGCCCTTTCCAACAATGGGTCTGCGCATGGGGGCCC
Lycium barbarum isolate Lr01 chromosome 6 NC_08334...       (8434) ACATCTACAAACACATAATCAAACAATACTCAAACTAAACGAATTTGCAAACACCACTGCTTCAC-TAACTAAAA
```

Homo sapiens chromosome 17 vs. Lycium barbarum isolate Lr01 chromosome 6

Section 120

```
                                                     (8926) 8926          8940      8950      8960      8970      8980      8990    9000
Homo sapiens chromosome 17 NC_000017.11 region fr... (8572) CGAGGGCATCCTCCCGGCCTGACCTATTCTGCCCAGCTGGCAGCTGGGGAGCAGCTAGGAGGGAAAGTGCAGCTG
Lycium barbarum isolate Lr01 chromosome 6 NC_08334... (8508) AAAAAAAAAACTGACACAATCATCTATTAATATTAACAAATATCAA---ACCAACCTAACGGCTAACTATCGAAC
```

Section 121

```
                                                     (9001) 9001     9010      9020      9030      9040      9050      9060          9075
Homo sapiens chromosome 17 NC_000017.11 region fr... (8647) CAGCTCCCACAGGGGCCAGCCATGAATAAATCATTGGGGTCAAAAGCAAGGCTTCAGGGAGAGACAAAGGCCCAG
Lycium barbarum isolate Lr01 chromosome 6 NC_08334... (8580) CAACGAA-ACACTT-CGACATATGAAATAAACAGAAAGTACTGAAGCTGCTAATAAACAGATAACGAAGGGAGAT
```

Section 122

```
                                                     (9076) 9076          9090      9100      9110      9120      9130      9140    9150
Homo sapiens chromosome 17 NC_000017.11 region fr... (8722) GCTT-GCACTGCTGGTGACAGAGAGAAGCGGCAGGGACAGGCTCCTCCCAAGTGAGGCCCCTCCTGTCCCGCCCC
Lycium barbarum isolate Lr01 chromosome 6 NC_08334... (8653) TTATCGACCTTTTGTGGGTACAGTGAAGTGG--GGCGTCGACGTCGTCGAATCTACGCTCGAACTCGAATGAAAT
```

Section 123

```
                                                     (9151) 9151     9160      9170      9180      9190      9200      9210          9225
Homo sapiens chromosome 17 NC_000017.11 region fr... (8796) CCAGCCACCCCTCGCCCCCTGCCCCCGTGAGCTTCATACCTACAAAAAGGACGAGGCAGGGGCCCTCGT-TAAGC
Lycium barbarum isolate Lr01 chromosome 6 NC_08334... (8726) AAACGATGCTTTCGATTAAAACCCA---GAATTGATTGTGTGAAAATATGGTGATTTAGAGTAATCCGGCTAAAG
```

Section 124

```
                                                     (9226) 9226          9240      9250      9260      9270      9280      9290    9300
Homo sapiens chromosome 17 NC_000017.11 region fr... (8870) TGCGCGGCGTTGGAGTCGGAGAGCTCCAGCACGGGCTTGGGGTGCCAGGGGAACTCCCGGCAGTCCTCGTCGTTC
Lycium barbarum isolate Lr01 chromosome 6 NC_08334... (8798) TTCAC--CGGAAAAGT--GAAAACTATGGTTGGAAAATGGTG----AGTGTCCCTAAACACTGAATCAATCCCTC
```

Section 125

```
                                                     (9301) 9301     9310      9320      9330      9340      9350      9360          9375
Homo sapiens chromosome 17 NC_000017.11 region fr... (8945) AGCACCTCCACCCGCCCCTGCCGCGTGATCACCTCGCCCTGC-----GGGTCCAGCATGA-TGAGCGTGGGGATG
Lycium barbarum isolate Lr01 chromosome 6 NC_08334... (8865) AACTCCAAAAATCCCCCCTTTTTCGATTCGACCTCGACCCATTTATAGGGTTTTGTTTCTGTTCTAGTGGAGAGA
```

Section 126

```
                                                     (9376) 9376          9390      9400      9410      9420      9430      9440    9450
Homo sapiens chromosome 17 NC_000017.11 region fr... (9014) CCTGCAGGGAAGAGGGGG-TGCTTGGCCGCTGGCCCGGGAGATGCTGGCCCTGCCTGGCAGGAGGCTCGCGGGGT
Lycium barbarum isolate Lr01 chromosome 6 NC_08334... (8940) GGAGCGTGGGAGAGAAGTATGGTGTCGGGTGAAGTGGGAGGAACGTGAGGAGCAGG--AGCAGGCGTGGGGGGG
```

```
                                                                 Section 127
                                                      (9451) 9451     9460      9470      9480      9490      9500      9510          9525
Homo sapiens chromosome 17 NC_000017.11 region fr...  (9088) CCAGCCCGGGGTCCCCCCGACCGTGGTGGAGCCCACCGAGGACCT-GGTACAGGCTCACAATGAAGAGGCCA---
Lycium barbarum isolate Lr01 chromosome 6 NC_08334... (9013) AA---CAAAAGTAAGTGGAGCAGCGATGGATGGCAGAGAAGAGGTAGGAAGAACGTGAAATGGAGGAGAGAAAAG
                                                                 Section 128
                                                      (9526) 9526          9540      9550      9560      9570      9580      9590     9600
Homo sapiens chromosome 17 NC_000017.11 region fr...  (9159) GGTCTAAAGTCACAAAGCCTGGGGAGAATCCAGGCCTGGCCAGG--GGGAGCCATGGAACCCCCTCGGGTCAGCC
Lycium barbarum isolate Lr01 chromosome 6 NC_08334... (9085) GGAGTAGAGAGATAGAGATGAAGGAGTGAGCGGCGGAGGTGAAGGAGGGGGTGAAGGGATTAGGTTTTGTTTGGT
                                                                 Section 129
                                                      (9601) 9601     9610      9620      9630      9640      9650      9660          9675
Homo sapiens chromosome 17 NC_000017.11 region fr...  (9232) T--GTAACCTGTCACCTTCGCCTT--TCAGGCCTCAGGATCCTCAGCTGCAGCCTGATGAGTGTGTTGTCGTTC
Lycium barbarum isolate Lr01 chromosome 6 NC_08334... (9160) TTAGGAAATAGAAAGTGTGGGCCGGGTCGGGTGATAAGGTT--TGGGCTGGA--CTGAACCGGGTTAGGTAGTTG
                                                                 Section 130
                                                      (9676) 9676          9690      9700      9710      9720      9730      9740     9750
Homo sapiens chromosome 17 NC_000017.11 region fr...  (9303) CTACCTTATTCCG-CT-ACAGAAAGATCAATGAGTTC----ATGCGGTTAAAGAACTTAGAGTTTGGGAGGCTGA
Lycium barbarum isolate Lr01 chromosome 6 NC_08334... (9231) GTGTAGGATTTGGGCTGATATAATGTGGGTTGGGTATTAAAATGTGGGTTGTTTATTTGGGTAGGGGAATAATAT
                                                                 Section 131
                                                      (9751) 9751     9760      9770      9780      9790      9800      9810          9825
Homo sapiens chromosome 17 NC_000017.11 region fr...  (9372) GGTGGATGGA--T----CACTTGA--GGTCAGGAGTTGGAGA-CCAGCCTG-GCCAACATGGTGAAACCCCCGTC
Lycium barbarum isolate Lr01 chromosome 6 NC_08334... (9306) TGTATTTGTATTTTAAGCCATTAATTGGCTGAAAATTGTTGATCCTTCCTCCGCTATTTTAATTATTTTCGGGCT
                                                                 Section 132
                                                      (9826) 9826          9840      9850      9860      9870      9880      9890     9900
Homo sapiens chromosome 17 NC_000017.11 region fr...  (9437) TCTACTAAAAATACAAAAACTAGCTGGGCGTGGTGGCGGGCGCCTGTAGTCCC-AGCTACTC-GGGAGGCTGAGG
Lycium barbarum isolate Lr01 chromosome 6 NC_08334... (9381) TCTAATTTAATAACTAGTACCATATTTATTATGTGAAGATAAATAGTAATTAGTATATAGAAAGTAAGGATTTTA
                                                                 Section 133
                                                      (9901) 9901     9910      9920      9930      9940      9950      9960          9975
Homo sapiens chromosome 17 NC_000017.11 region fr...  (9510) CAGGAGAATCGCTCGAACTCGGGAGGTGGAGGTTGCAGTGAGCCAGGATTGCACCACTGCACTCCAGCCTGGACA
Lycium barbarum isolate Lr01 chromosome 6 NC_08334... (9456) CAAAGTGTTGTCTTGTAATTA--ATTTAACGATTCCAG--ACCCGAAAATGAAACGATG-ACTAACCGTTTAAAA
```

Homo sapiens chromosome 17 vs. Lycium barbarum isolate Lr01 chromosome 6

```
Section 134
                                                      9976                   9990           10000           10010           10020           10030           10040    10050
Homo sapiens chromosome 17 NC_000017.11 region fr... (9976)
                                                      (9585) ACAGAGCAAGATTCTGTCTCTAACAAATAAATAAATAAATAAGAACTTAGCACAGGGTCATGGCGCATCGGA-G
Lycium barbarum isolate Lr01 chromosome 6 NC_08334... (9526) TTTGTGATAAAGTAATGCTCGTAATGTTTTAAAAAA-AAATAAGAGTAGTGAAAATAATAGTAGTGAAAATAAAG

Section 135
                                                      10051         10060           10070           10080           10090           10100           10110          10125
Homo sapiens chromosome 17 NC_000017.11 region fr... (10051)
                                                      (9659) CAATCA-CTCACTGCTGGTCTCCCAAAGACAC--AGAAGACCTTCCCCAGCTGC-----TGGGGGTCAAACACGG
Lycium barbarum isolate Lr01 chromosome 6 NC_08334... (9600) TATTTAGCTCGTTAGTAAATTTAGAAACCCGGGTAAATTAAATAGGAGAGGGACAAAATTGGGTGTCAACAGCAG

Section 136
                                                      10126                  10140          10150           10160           10170           10180           10190    10200
Homo sapiens chromosome 17 NC_000017.11 region fr... (10126)
                                                      (9726) A-ACAGGCAGTGCAGTG-ACCACACGGCGGACTTGCTGTGGCCACCGATGCAAGACGGGTCCTCGCCCGGCCACA
Lycium barbarum isolate Lr01 chromosome 6 NC_08334... (9675) ATATAGGCGACTGAGTGCACCGATTTGTAAAAGGCCTAGGGCCACACTTGATGGATAGATGCTTGACTGCGTCCC

Section 137
                                                      10201         10210           10220           10230           10240           10250           10260          10275
Homo sapiens chromosome 17 NC_000017.11 region fr... (10201)
                                                      (9799) GTGAGCGC---AGGGCCAGTGC-CGCCGGGGGGAACCTGAGGCTGGCCGCACCGGAGAC-TCGAAGAGGAGGACA
Lycium barbarum isolate Lr01 chromosome 6 NC_08334... (9750) TTCAGGACGATATGGATACTGCACGCATTCAGGCCCATGCTCAAAACTTAGAAGAAAACCTGCCACAGCAAAGAA

Section 138
                                                      10276                  10290          10300           10310           10320           10330           10340    10350
Homo sapiens chromosome 17 NC_000017.11 region fr... (10276)
                                                      (9869) GCTGACTGCAGCCCCCGCCGTCTGCACCCCAGCCCTCCCCGCTCCCCAGGGCTGCAGCCCCCGCCGTCTGCACCC
Lycium barbarum isolate Lr01 chromosome 6 NC_08334... (9825) GCGAACGTGAGCAAGATAGGGGC-CATAGCAAAGGGCCAGATCTTCGGGTGCGATGAGTGAGATTAGAGGTTGG

Section 139
                                                      10351         10360           10370           10380           10390           10400           10410          10425
Homo sapiens chromosome 17 NC_000017.11 region fr... (10351)
                                                      (9944) CAGCACAACCCCCTCCCCAGGGCTGCAGCCCCCGCCGTCTGCACCCCAGCACAACCCC-----------------
Lycium barbarum isolate Lr01 chromosome 6 NC_08334... (9899) CACAGGCAGCAGTTTCCCGGGCATTCAGGGTACTCTATGACCAGTGCACCTCCACGGTTTTTCAGGCTAGAGAT

Section 140
                                                      10426                  10440         10453
Homo sapiens chromosome 17 NC_000017.11 region fr... (10426)
                                                     (10002) ----------------------------
Lycium barbarum isolate Lr01 chromosome 6 NC_08334... (9974) TTGATAGATCTGCTCGTGTGGGGCCGAG
```
