# Supplementary material for: Patch type nucleotide sequence identities between genomes from many different species facilitate illegitimate recombination
Source: Sci Rep. 2026 Mar 30;16:10524. doi: 10.1038/s41598-026-44124-0 (PMC13035915; doi:10.1038/s41598-026-44124-0)
Supplement: Supplementary file 14 — Supplementary Material 14 [file 41598_2026_44124_MOESM14_ESM.pdf]

Homo sapiens chromosome 1 NC_000001.11; 11783698-11817823 vs. SARS-CoV-2 Shuffle No.1

Section 1

```
                                                        (1) 1       10              20              30              40              50              60              74
Homo sapiens chromosome 1 NC_000001.11: 11783698-...    (1) CTGCCCTCTGAGGCTGAGGTTGCAAGATGGA-AAGCCAGTGCTTTCACCTGTTACTGGTCGTTTACTGTTTTGT
SARS-CoV-2 Reference Genome Shuffle No.1                (1) --CGGCACTGACGACATGGCATGTAGATTAATAAGACCATAGATTTAGAGTAGATTCGATGCCTTTCAAGATGT
```

Section 2

```
                                                        (75) 75  80              90              100             110             120             130             148
Homo sapiens chromosome 1 NC_000001.11: 11783698-...    (74) GGGAGATTTGCAGGTGTAACTTTGAATAAATG---TGACTTTTGACTC--TGAGAACGAGA-----CAAGCCCT
SARS-CoV-2 Reference Genome Shuffle No.1                (73) AGCAG-TATAAAATTATAAATTAACTTGAATGCTATGATTTTTGAAACATTCAGTACAAGCTGGATCGGACCCT
```

Section 3

```
                                                        (149) 149        160             170             180             190             200             210         222
Homo sapiens chromosome 1 NC_000001.11: 11783698-...    (138) CCCA---TGCATCTGTTTTTGTTTTGTTTCATTTTGTTTTTTTGAGATGGAGTTTCAC---TCTTGTCACCCAG
SARS-CoV-2 Reference Genome Shuffle No.1                (146) TCCAGTTGGGAACTATATATGATCACGTTAATACCTGATTGTTCAGAAGTATTCACAGTTATCGTTTGAACGGC
```

Section 4

```
                                                        (223) 223    230             240             250             260             270             280             296
Homo sapiens chromosome 1 NC_000001.11: 11783698-...    (206) GCTGGAGTGCACTTGCGCG-ATCT----CGGCTTACTGCAACCTCTGCCTCCTGGGTTCAGGCAATTCTTCTGC
SARS-CoV-2 Reference Genome Shuffle No.1                (220) GCCAGACATCTTTATCGCCTAACTTTTGCACAGTCTTATAAACTAAAATACCT---TTTCGAGAAGTACTGTCG
```

Section 5

```
                                                        (297) 297           310             320             330             340             350             360     370
Homo sapiens chromosome 1 NC_000001.11: 11783698-...    (275) CTCAGACTCCCAAGTAGCTGGGATTACAGGTGCCCACTACCAGGCCTGGCTAATTTTTGTATTTTTAATAGAGA
SARS-CoV-2 Reference Genome Shuffle No.1                (291) CTATCGCTACAGAGT-GTAATGATTTTAGA--CTGACTATTTAGTTGAACGTGGTTTGAGGTTCTTATAAACGT
```

Section 6

```
                                                        (371) 371      380             390             400             410             420             430         444
Homo sapiens chromosome 1 NC_000001.11: 11783698-...    (349) CGGGGTTTCACCACGTTGGTCAGGCTGGTCTC--AAACTGACTTCAGGTGATCCACTCACCTTG-GCTTCCCAA
SARS-CoV-2 Reference Genome Shuffle No.1                (362) CGAA--TCCGTGGGGGATATTACGCTCTTCCCCTTAATTAACACTAGGCCCATCACTTAAAAAATGCATAAAAA
```

Section 7

```
                                                        (445) 445  450             460             470             480             490             500             518
Homo sapiens chromosome 1 NC_000001.11: 11783698-...    (420) AGTGCTGGGATTACA------AGCATGAGCCACCACACCTGGCCCAATGTGTCTGTTTTGCAGCTACTTCCAGG
SARS-CoV-2 Reference Genome Shuffle No.1                (434) CATACCTTTATTTCAGGGTCTAGCTTGA-CGGTCGTATTTATAATAATA-GACTATTTT----CTTCTTAGAAT
```

Homo sapiens chromosome 1 NC_000001.11; 11783698-11817823 vs. SARS-CoV-2 Shuffle No.1

Section 8
```
                                                     (519) 519        530            540            550            560            570            580            592
Homo sapiens chromosome 1 NC_000001.11: 11783698-... (488) CCTGGTGTGAGGTTGTAAGAGACACGGGGGTGCTCCGGGCCCAGCATCAAGCCTTTCAGGATGGCCTGAGGAGA
      SARS-CoV-2 Reference Genome Shuffle No.1       (502) CGCTTT-TCTGCTGGGAAGCTAATCAAG-----TAAGAACACAATGTCCGAATTGAAAGGATGTACTC-GTACA
```

Section 9
```
                                                     (593) 593    600            610            620            630            640            650            666
Homo sapiens chromosome 1 NC_000001.11: 11783698-... (562) AGAG-----CACTGGGGGCCGTGT---TTGCCACATGGCGGGAAGCCCAGGAAGTGGCAGCCGGGGCACAGG--
      SARS-CoV-2 Reference Genome Shuffle No.1       (569) ATACTTAGACCCAAGGAGCATTTTAAATTCCTAATTACATCCTAGCTGATGTTTCTTAATCCTAAACACAAGTA
```

Section 10
```
                                                     (667) 667          680            690            700            710            720            730            740
Homo sapiens chromosome 1 NC_000001.11: 11783698-... (626) AGCAGCGTGTGG---CCCAG-GCCTCCCTTGCCCGCTGGAGAAGCTGCGGGCAGCAA--GGCCAGGAAG-ATGG
      SARS-CoV-2 Reference Genome Shuffle No.1       (643) AGCTCCATAAGGAAAACTAATGTTTTTTTAGTGCTTTTTATACG-TGCCTTTATCAATTGGGTATACAGTATCG
```

Section 11
```
                                                     (741) 741          750            760            770            780            790            800            814
Homo sapiens chromosome 1 NC_000001.11: 11783698-... (693) GCAGCAGAAGAAGGCCCGGGCCCCACAGGCCTTCCCAGCATGGCCAGTGGCCCCGGGCATGCACCATGAGGCCC
      SARS-CoV-2 Reference Genome Shuffle No.1       (716) CAATTGGATAAAAATCTTATTGCGATTTTCCGCCTGTTAAATATCAATAATCGCAGCGTTTTACAAT-ACTCTC
```

Section 12
```
                                                     (815) 815  820            830            840            850            860            870            888
Homo sapiens chromosome 1 NC_000001.11: 11783698-... (767) AGCAGCAGGCAGGAGAGAGCGCTGGGGCCCAGGCAGCCCAGTGC-TGGACTTGGTGCTGGGCTCTGTGGGTGCA
      SARS-CoV-2 Reference Genome Shuffle No.1       (789) AAAATATGG--GGCTATAGTCTTT---CCCAGTTTGACGGTTGGATGCTCATTTTATTACGTCTTATATGAATT
```

Section 13
```
                                                     (889) 889        900            910            920            930            940            950            962
Homo sapiens chromosome 1 NC_000001.11: 11783698-... (840) TGAGT-CCTGTCGGGGCCAGGTCAGCCGAGCCCATGCTTCCTGGAAGCCG--CGAGCCTGGTGAG-TGCTGTG-
      SARS-CoV-2 Reference Genome Shuffle No.1       (858) TGTGTTCGCGTCTTGCTGATATATTGGTATGAATTAAGAGACGGTTGTCATCCTTGCCTCTTAAGATTCTGTGA
```

Section 14
```
                                                     (963) 963    970            980            990            1000           1010           1020           1036
Homo sapiens chromosome 1 NC_000001.11: 11783698-... (909) GTCTAAGTGCAGCCCCACTCTGTGCTTCCAG-CTCA---GGCCTCAAGCC-TGTTTTGGCAGGGGTAGAGCGT
      SARS-CoV-2 Reference Genome Shuffle No.1       (932) GACTAAGTGCCTTTGTGCTATGCTCTTCTATACTAAATTGTTCTATATCTATACTGCTAACTCTCCTTCTCAGC
```

Homo sapiens chromosome 1 NC_000001.11; 11783698-11817823 vs. SARS-CoV-2 Shuffle No.1

```
                                                                Section 15
                                                 (1037) 1037        1050      1060      1070      1080      1090      1100    1110
Homo sapiens chromosome 1 NC_000001.11: 11783698-...  (978) AATTCATGGCTGGGTGGCAGGGCAAAGGCTCAAGAGGGTGGTTGGGGGTGACAGGGAGAGGCCGCCCTG---GC
         SARS-CoV-2 Reference Genome Shuffle No.1 (1006) TATTTACGCCTGTACG--ATGTACGAAGCCAAAATTATTATATAAACCTCAAAAG-AGGCGCTGTATTGTCTGC
                                                                Section 16
                                                 (1111) 1111     1120      1130      1140      1150      1160      1170       1184
Homo sapiens chromosome 1 NC_000001.11: 11783698-... (1049) CATCTAGTCCTGGGCCACATCCTGACACCGCCTCTTGCTGTGTGTCCCCTCCTTGGGGAAGTCTCTAATGGTCA
         SARS-CoV-2 Reference Genome Shuffle No.1 (1077) TCGATATGACGTTTAAGCATTTTGATATGA--TTGTGTTGGAGGTTGC-TCATGCGATAAATTGTAAGCGGTCT
                                                                Section 17
                                                 (1185) 1185 1190      1200      1210      1220      1230      1240          1258
Homo sapiens chromosome 1 NC_000001.11: 11783698-... (1123) T--GCCTTGCAGTCACCAGCACAGCCAGCCACTCAGCA-AGCGCCATAAACGCGCTCTTGCCAGGGAAGGGGAG
         SARS-CoV-2 Reference Genome Shuffle No.1 (1148) TTTACTTTGCAGCACGAATCACTGGTATTAAATTTGTTCATTAACTTTTTTAACCTTATAACTTCGTTGGTGTT
                                                                Section 18
                                                 (1259) 1259       1270      1280      1290      1300      1310      1320    1332
Homo sapiens chromosome 1 NC_000001.11: 11783698-... (1194) GCAAGTGGCACTGGCAGGACTGTCCCC----TCTCTGCCCCTAAACAGG-ATGCTTGTCCCCGTCTGTGATTT
         SARS-CoV-2 Reference Genome Shuffle No.1 (1222) GCTAGGGGCCTAATGAAATCCACCAAACGTAATTTGTGACATTAATTTTTCAAAAAAGGGCTCCTCAGTATTTA
                                                                Section 19
                                                 (1333) 1333  1340      1350      1360      1370      1380      1390         1406
Homo sapiens chromosome 1 NC_000001.11: 11783698-... (1263) TCTCCTTCCAGGTCGCTCTCCATCCTGTTTACCCAGATTCTCCCTCCAGAAAAGGGAGTGTGGGAGGTGGGTGG
         SARS-CoV-2 Reference Genome Shuffle No.1 (1296) ATTGCATAGCGGT---TTTCCATGA-GTTTATCTATAA-----CGCACGAATTCTAATTTTCCAGTATGTAGAA
                                                                Section 20
                                                 (1407) 1407        1420      1430      1440      1450      1460      1470    1480
Homo sapiens chromosome 1 NC_000001.11: 11783698-... (1337) GCCCCAGCCTGGGGCTGGGCCCGGAAGGCCCCCTCCCGCAGGGCACTGGGGGGGCT---CCCTGCAGA-GAGTC
         SARS-CoV-2 Reference Genome Shuffle No.1 (1361) GTCCCAATATTCAGCTGTTGCTTTTGAGAC---TAACTTATGTCATTGGGATAGCTATACCAAACATACGTGTG
                                                                Section 21
                                                 (1481) 1481     1490      1500      1510      1520      1530      1540       1554
Homo sapiens chromosome 1 NC_000001.11: 11783698-... (1407) CTGGCCTTTATG-GCCCTG---GCTGCAGACTCCTTCCTCTCCCGCAGGGTCCTAGAGGCCT--CGGTGCAGTC
         SARS-CoV-2 Reference Genome Shuffle No.1 (1432) ATGGGCTGAACGTGAACAGAAAGTTCGAAGCAGCGAATACGCGGGGTATGTTAAATATGTCCGCCAGTATGGAT
```

Section 22

```
                                                       (1555) 1555 1560      1570      1580      1590      1600      1610              1628
Homo sapiens chromosome 1 NC_000001.11: 11783698-...  (1475) GGCGGTGCGCGGCGGTGTCCAGCGAGCCATCCTCACCCAGCTCCG-GCCGGCTGAGCTCAGGCGCTTC-CTGCG
         SARS-CoV-2 Reference Genome Shuffle No.1  (1506) GAGGAGACCATACTTAGTCAAATGGCCCAACGTTTTATAATGCGAAGCAATATGATCTTTACCTTTTAGCTTGG
```

Section 23

```
                                                       (1629) 1629       1640      1650      1660      1670      1680      1690          1702
Homo sapiens chromosome 1 NC_000001.11: 11783698-...  (1547) GACAGTGCAGCTCAGGGTGCGGCTGG--GACTGC-CAGGGGCCGGCAAGGTACGCCCCAAGCCCCAGACTGACC
         SARS-CoV-2 Reference Genome Shuffle No.1  (1580) TAATACCGACCACAGGTTGATGTAGTTCTACTCCGCCGGGTCCGGTA--GTAAAGATTACGTTGAAGCTTATTG
```

Section 24

```
                                                       (1703) 1703   1710      1720      1730      1740      1750      1760              1776
Homo sapiens chromosome 1 NC_000001.11: 11783698-...  (1618) TCA-CGC-TCTGGCCCCGGATGGCCAGCAGGGGAGCCTGCTGCTGGACGCCCCAGCCCCTTGGAAACAGGTGA
         SARS-CoV-2 Reference Genome Shuffle No.1  (1652) TAAACGAATATAGTATAGTATATGGA-CAGAGTCCAGTTCCAAC-GAATACCACAGC----TATCATTAAGGAC
```

Section 25

```
                                                       (1777) 1777         1790      1800      1810      1820      1830      1840      1850
Homo sapiens chromosome 1 NC_000001.11: 11783698-...  (1690) AGGAGCCTAGGCAGGGCGGGAGGTCCAAAAATGACCCTCGGACCACCGGCCTTAGAATCAGCTTGATTCAGCC
         SARS-CoV-2 Reference Genome Shuffle No.1  (1720) TGCGGGATAATGACGAGTGGAAACTACTAAGTTGT---TCAATCTATCTTGATCACAATACGCAT-ATCC----
```

Section 26

```
                                                       (1851) 1851     1860      1870      1880      1890      1900      1910          1924
Homo sapiens chromosome 1 NC_000001.11: 11783698-...  (1764) GGTGGTGGCAGCATGGCTGGATGCCTCC-CCAGGGGCCCAGCAACCTCGTATGCACCACCTCTCCCCAGTTCTC
         SARS-CoV-2 Reference Genome Shuffle No.1  (1786) -GTGGTT-----AGGGCTGTATACGTGCACCTAAAGGAGAAGGCTTTCGGTTGCAACTCGAGTGTTTTGACCTA
```

Section 27

```
                                                       (1925) 1925 1930      1940      1950      1960      1970      1980              1998
Homo sapiens chromosome 1 NC_000001.11: 11783698-...  (1837) AGTCAGCCTCTGACAAGCAACTCTGAATCCATCAGC-AAAGATGAACCTGGCACCTCTCGAGTGCCAGGCCACT
         SARS-CoV-2 Reference Genome Shuffle No.1  (1854) TCATAGCCC---ACAAGTTACCGTTG-TCGATCTGGGAAATTCGCGCAAGTTATCCCACTTGTATACAGGATGA
```

Section 28

```
                                                       (1999) 1999       2010      2020      2030      2040      2050      2060          2072
Homo sapiens chromosome 1 NC_000001.11: 11783698-...  (1910) GCAGGTCCGG--CCGC--AAAGAC-CAGCATGTTGGGTCACAGCCTGTTAGGGAGACATTGAAATGCATGCTTT
         SARS-CoV-2 Reference Genome Shuffle No.1  (1924) GCAGGTAGTCTTGCGTTATAAGTTACACTATTATCTCTCGTTATCCGTTAGATAGTTAGT-AAGTGAATGCAGC
```

```
                                                                    Section 29
                                                (2073) 2073   2080            2090            2100            2110            2120            2130            2146
Homo sapiens chromosome 1 NC_000001.11: 11783698-... (1979) CTCACGGTGCAGTTGAGGATCAGAATAGGTACACGAGGACAGTAGCATTTTTTTGTCA-AGTTTTTTGTTTAT
     SARS-CoV-2 Reference Genome Shuffle No.1 (1997) TACTCTATTC--TACGCTATTGCTTTACGTAGTTGTCATTATTTTGAGGCTATAGGAGGAGTTGTCTGGTTTGG
                                                                    Section 30
                                                (2147) 2147            2160            2170            2180            2190            2200            2210    2220
Homo sapiens chromosome 1 NC_000001.11: 11783698-... (2052) TTTTGAGACAGAGTCTGGCT-CAATTGCCCAGGCT-GAAGCAGAGGAGTGATCTCAGCTCAC-TGCAACCTCTG
     SARS-CoV-2 Reference Genome Shuffle No.1 (2069) ATTCGAAACGGGCTCTTTTTTCAAAGAATCTAGCTAGAAACGAATCTGTTTTCATGACACATGTGGATACCCTA
                                                                    Section 31
                                                (2221) 2221    2230            2240            2250            2260            2270            2280            2294
Homo sapiens chromosome 1 NC_000001.11: 11783698-... (2123) CCTCCCGGGTT------CAAGTGATT--CTCCCGCCTCAGCTTCCTGAG---TAGCTGGGACTACAAG-----T
     SARS-CoV-2 Reference Genome Shuffle No.1 (2143) CCTTAAGTTCTGATAAACAAGTGTATAACACCAGTATCTGACTAAAGAAATATTCCATAAACGCGAAGGACGAT
                                                                    Section 32
                                                (2295) 2295 2300            2310            2320            2330            2340            2350            2368
Homo sapiens chromosome 1 NC_000001.11: 11783698-... (2181) GCACGCCACCACGCCTGGCTAATTTTGTATTTTTAGTAGAGACGGGGTTTCACCATATTGCCCAAGCTGGTCT
     SARS-CoV-2 Reference Genome Shuffle No.1 (2217) ACATAGGTCCACCCAGTGGGAGCTTCAGGATGCCCTGATGTATTGAACGTCTACTCTATTTCGTTTTTCGTTCA
                                                                    Section 33
                                                (2369) 2369          2380            2390            2400            2410            2420            2430    2442
Homo sapiens chromosome 1 NC_000001.11: 11783698-... (2255) CGAACTCCTGAACTCAAG--TGATCCTCCCACCTCGACTTCCCAAAGTGCTGGGATTACAGG-TGTGAGCCACC
     SARS-CoV-2 Reference Genome Shuffle No.1 (2291) AGTGGTCTTGACATGGGTTTTGGTGGTAGCGCTTAAACGACACAACCT------ATTAAGGGCTATAAGCT--C
                                                                    Section 34
                                                (2443) 2443   2450            2460            2470            2480            2490            2500            2516
Homo sapiens chromosome 1 NC_000001.11: 11783698-... (2326) GCGCCAGGCCAAGTTTTTTTTTTCTTATTGGAAAAGTGAAATAT--G--CTTATTACAGAAACCTCAGAAAAT
     SARS-CoV-2 Reference Genome Shuffle No.1 (2357) ACGCAAG--CAAAATATTCTATCGGAGCTGCCAAGACTTTTTCGTCGGGGATTCTTACAAAATCGTTTCAAGGT
                                                                    Section 35
                                                (2517) 2517            2530            2540            2550            2560            2570            2580    2590
Homo sapiens chromosome 1 NC_000001.11: 11783698-... (2396) ATAAAGTAGAAAAATACCAAAATTTCTTTCATAATTCTCTCTACCCAAAGGCATCGGTCAGTCCCTCTCCAGAC
     SARS-CoV-2 Reference Genome Shuffle No.1 (2429) CTGTTGGACAGATATATTTGCACAAAATTTGGATTACTTACGAGGCGGAAAAATCTTT---TTCTTATAAACTG
```

Section 36

```
                                                    (2591) 2591     2600           2610           2620           2630           2640           2650        2664
Homo sapiens chromosome 1 NC_000001.11: 11783698-... (2470) CAGAAGCAG-TTAGTTCTGACACCAACAAGTGGTGATAAGAGGTTGATAGCCTAGCAAGGGGAAGAAGCCACC
         SARS-CoV-2 Reference Genome Shuffle No.1 (2500) TATTCGGGGATTATTAGTATCTCCAGCAAGTATTTA----ACGCTTATGGCATCAAACGGTTATCCACTCAGTG
```

Section 37

```
                                                    (2665) 2665 2670           2680           2690           2700           2710           2720             2738
Homo sapiens chromosome 1 NC_000001.11: 11783698-... (2543) ACCAAACCAAACACAAAAAATGACACTGCACAGCAGTCTGGGACATGTCCTTGAAGGACACAGCCCCAAAGAT
         SARS-CoV-2 Reference Genome Shuffle No.1 (2570) ATTTTTTTATGTTGGGGTTCCCTATTTGTACTGTTAATTGTTCACAGGGCTT--AAGGTTAAACCCATAAATAA
```

Section 38

```
                                                    (2739) 2739       2750           2760           2770           2780           2790           2800       2812
Homo sapiens chromosome 1 NC_000001.11: 11783698-... (2617) ACTGGCAGG--TGAGG-TAGTGTCCCTAAGATCAACAGGCATCATCATGAATCCTATTTCAGAATGCCACAAAG
         SARS-CoV-2 Reference Genome Shuffle No.1 (2642) CTTAGGAGGACTGTGGGTATGGGCGCTATGAGTATCAAGAAAGATACT--AGTGTCTTTACGTGTCCCAGTAAG
```

Section 39

```
                                                    (2813) 2813   2820           2830           2840           2850           2860           2870            2886
Homo sapiens chromosome 1 NC_000001.11: 11783698-... (2688) --AAT-ACGTTTTAGA--GAAGATTCTAAGAGAAAAAGCTAAGGTGATTTTTTTTTCAGTCGTTTTTTGTTCT
         SARS-CoV-2 Reference Genome Shuffle No.1 (2714) CGGATTACTCGTTACCAGGTAGGCTCCATGTTGCACAA-TAAACACCCTGGAGCTATCGTTGGTTAAAAAATGT
```

Section 40

```
                                                    (2887) 2887          2900           2910           2920           2930           2940           2950    2960
Homo sapiens chromosome 1 NC_000001.11: 11783698-... (2757) CAGACAGT-GTCTCACTCTGTCGCCCAGGCCAGAGTGCAGTGGTGCGATCACAGCTCACTGCAGCCTCAACCTC
         SARS-CoV-2 Reference Genome Shuffle No.1 (2787) GGGAGGTACGTGCAAGACTGTT----AGGAAAT-GTGAAGACGGAGGGTCTAAAC--ACCGATACTACACCCGT
```

Section 41

```
                                                    (2961) 2961     2970           2980           2990           3000           3010           3020        3034
Homo sapiens chromosome 1 NC_000001.11: 11783698-... (2830) CAGGGCTCAAACGATCCTCCCACCTCAGCCACCTGAGTAG-CTGAGACTACAGGCGTGTGCCACCACACCCGGC
         SARS-CoV-2 Reference Genome Shuffle No.1 (2854) CATTGGTT----GATCTGACAACTGGTGTTTCTTTATTAAACTAAAGCTTCGGGAACTTGGCACCGGCCTTTTC
```

Section 42

```
                                                    (3035) 3035 3040           3050           3060           3070           3080           3090             3108
Homo sapiens chromosome 1 NC_000001.11: 11783698-... (2903) TAATTTTTTTTTTTTTTTTTTTGTAGAAATGGGATCTCACTATGTTGCCAGGGCTTGTCTCAAACTCCTGGGCT
         SARS-CoV-2 Reference Genome Shuffle No.1 (2924) TAGATTAATAATAATTACTTTAC--GAAGTTCTACGTCATCATAT-GCCTTTAAATATCGGATACCTATGTACT
```

Section 43

```
                                                      (3109) 3109       3120      3130      3140      3150      3160      3170      3182
Homo sapiens chromosome 1 NC_000001.11: 11783698-...  (2977) CAAGCGATCTACCTGCCTTGGCCTCCCAAAGCAT-TGGGC------AGGCCCATTACGCCATGAGCCACCACAC
       SARS-CoV-2 Reference Genome Shuffle No.1  (2995) GTAT---TTTCATTGTATTAT--TTCTGACGCATGTGGGCGCATCGCGACATTTTAGGAAAACAGGTATAATAG
```

Section 44

```
                                                      (3183) 3183   3190      3200      3210      3220      3230      3240      3256
Homo sapiens chromosome 1 NC_000001.11: 11783698-...  (3044) CTGCT---GGTTTTTGTTTTTAAATGGAATTGAAAGGAACTAGCCTTGGCTAGTTG--CAGAATTCACACATA
       SARS-CoV-2 Reference Genome Shuffle No.1  (3064) TTTTATAGTTTTTTTATTATTAAAACTAAATCTCATTAATTAGTGTTGTCAACGTGTTCGTATTTAAGTTCTT
```

Section 45

```
                                                      (3257) 3257         3270      3280      3290      3300      3310      3320    3330
Homo sapiens chromosome 1 NC_000001.11: 11783698-...  (3113) TGGGGCTATGGCAACAGCAGATTCTGGAGTCTCAGGGTCTTTTCTTTCTAGATAAGTTAC-AGGGCAATTGCTT
       SARS-CoV-2 Reference Genome Shuffle No.1  (3138) TGAAGCAATGTCTATTTTATAG-CTCTATTAATAAGGTTTTCAAGTATTGGTGGTGATACTAGAATCATTCAGT
```

Section 46

```
                                                      (3331) 3331     3340      3350      3360      3370      3380      3390      3404
Homo sapiens chromosome 1 NC_000001.11: 11783698-...  (3186) TTCAGAGTACTTT-TACATTTCTATTCCATTCTTGT-CAGT---AAAGACAAGGATCCCATAAGC-TGATGGAG
       SARS-CoV-2 Reference Genome Shuffle No.1  (3211) TGGTGTGTTTGAGGTAAAGCTCCCC-CGATAGTTATACAGTTTCACGTTCAAACAGAATATAATCGTGACTTTC
```

Section 47

```
                                                      (3405) 3405 3410      3420      3430      3440      3450      3460          3478
Homo sapiens chromosome 1 NC_000001.11: 11783698-...  (3254) AAACTGAGGCC--TCTAGAAACTAAACGCTGGGAATGGCACCCAGGGTGCTGAAT-TCCAGGGTCTCCTCTTTT
       SARS-CoV-2 Reference Genome Shuffle No.1  (3284) TAATGGAAGCAGTTATAACAATTCAACCAATTAAACCACAATAAC--TGCATAATGTCCAGTGAGACAAAACTA
```

Section 48

```
                                                      (3479) 3479       3490      3500      3510      3520      3530      3540      3552
Homo sapiens chromosome 1 NC_000001.11: 11783698-...  (3325) CTGCCACAGGCACACATGCCCCAGCAGCCCTTTGGAGA------GCCCCCTCTCTA-TAGGGTACTAGCCGGG
       SARS-CoV-2 Reference Genome Shuffle No.1  (3356) C--CAGCAAGCGGATATTTATTAG-AGCTCTTAAGGACATAAAATGCCGGTTTAATAGTATAGTTGTGATCTAC
```

Section 49

```
                                                      (3553) 3553   3560      3570      3580      3590      3600      3610      3626
Homo sapiens chromosome 1 NC_000001.11: 11783698-...  (3392) GGCTCAGACTCTGGAGTCAGACGGCTTCCGAG-GGATGAGGCCTCAGACAAGTAACTCAGCTTTGAAAT---GT
       SARS-CoV-2 Reference Genome Shuffle No.1  (3427) GCCCCCTTATGTTTCCTCACTCGACTGCAGAACGGAATAGCGTAGGTCGGGCATC-CTGAAATGCCATTCCGC
```

```
                                                                   Section 50
                                                 (3627) 3627         3640     3650     3660     3670     3680     3690   3700
Homo sapiens chromosome 1 NC_000001.11: 11783698-... (3462) TTTCAGTGGGGAGAGAAATAGCCCCCTCCCAGGGCTGTGCTCCGG--GAACTGACTGAGGGCTAACTATTATGG
    SARS-CoV-2 Reference Genome Shuffle No.1 (3500) ACCCTTAGTGGATCGAATAGGCTTTTTCAGATTCATATATAAAGAGTGAATATTTACAGGCCAAATCGTTTATG
                                                                   Section 51
                                                 (3701) 3701     3710     3720     3730     3740     3750     3760     3774
Homo sapiens chromosome 1 NC_000001.11: 11783698-... (3534) GCCCACCCCATTGTTGGAATGTCTTCAGCCACATGGGGGAATGGGTGGAAGAGGCCGGGATGTGTCCTGCCAGC
    SARS-CoV-2 Reference Genome Shuffle No.1 (3574) ATTCGCCTAATTGT--GTATACTGTAACTTACTTGGTATTTGGAGCGTAATTC-CTAGAATATGTGTCACGATG
                                                                   Section 52
                                                 (3775) 3775 3780     3790     3800     3810     3820     3830     3848
Homo sapiens chromosome 1 NC_000001.11: 11783698-... (3608) TAGAGCAGCGAAGAAATCCCAG----CGGGGCCCCAGGAAGTCCAAGCCCATGGGGTTCAGGTGCTCCTCTCTG
    SARS-CoV-2 Reference Genome Shuffle No.1 (3645) TGTCTAAGACGATTAATCGCAAAAGACAGTGTATTAATAAATTAAACCTACTTCTGGGGAAATGCTAGTATCCT
                                                                   Section 53
                                                 (3849) 3849       3860     3870     3880     3890     3900     3910   3922
Homo sapiens chromosome 1 NC_000001.11: 11783698-... (3678) GCTATTCCTTCAGACCCGCAGCTGCT----GGACACAGGCCACAGAGCTG---GTGCCTCCCGCGCCAT-----
    SARS-CoV-2 Reference Genome Shuffle No.1 (3719) ACATATTCATCTGACGAATGTTGGATTATAGTACTGCGTCTTCATTTCTTTGCGTGCCTCGCAGGCCACATAGA
                                                                   Section 54
                                                 (3923) 3923   3930     3940     3950     3960     3970     3980      3996
Homo sapiens chromosome 1 NC_000001.11: 11783698-... (3740) -CACTGCAGTGCAGCCTGGGTGGACGGAGGAAGCCAAGGGGAAC---GGCC-TGGGCTCAGAGTAAGGAGACCT
    SARS-CoV-2 Reference Genome Shuffle No.1 (3793) ACACATAAATCCCTCAAGTCTACGGCCCACAATTTAGATGCAATTTTTGCAATGTGTTCCCTCAAAGATGACGT
                                                                   Section 55
                                                 (3997) 3997         4010     4020     4030     4040     4050     4060   4070
Homo sapiens chromosome 1 NC_000001.11: 11783698-... (3809) T----GCCCCGGGGGACAA--ACGGTGTCTGAAGTGCAGGGGTG-AAGCGGTCTCCAGTCCTCAGGGGCCTG--
    SARS-CoV-2 Reference Genome Shuffle No.1 (3867) TCATAGGTCAGGGTCACAAATACGCCAGCTATAATGGACTCATGCACTTCATCTCTCGATTTCAGCTCGCTGTC
                                                                   Section 56
                                                 (4071) 4071     4080     4090     4100     4110     4120     4130     4144
Homo sapiens chromosome 1 NC_000001.11: 11783698-... (3874) -GAAATCAGCTCCTTGGGACACGGTCTTATGTGTATTGA--CCATTCTCCCCATCCATTCCCTCTTCGGAATG-
    SARS-CoV-2 Reference Genome Shuffle No.1 (3941) TGAGAACA-CTATTTAGGA---GTTCTCATATATACTCAAATCGTACTTAAAGTGGATGAGGTTTTTCCAATCG
```

Section 57

```
                                                (4145) 4145 4150      4160      4170      4180      4190      4200             4218
Homo sapiens chromosome 1 NC_000001.11: 11783698-... (3944) ---TTTCAGCTGTTCTGAGAAAACACTTTGCAT----TCC-TGCCCCGCCCCTGTGATTTCTCTTTGGGCGGCA
SARS-CoV-2 Reference Genome Shuffle No.1             (4011) TAATTAGAACAAGTCA-AGGACACATTTGGTCTATACTCCGTGGCTTGCAGCCGAGAA--CGATAAGGCCCGAA
```

Section 58

```
                                                (4219) 4219       4230      4240      4250      4260      4270      4280         4292
Homo sapiens chromosome 1 NC_000001.11: 11783698-... (4010) GGCAGCCG-GGAGAGGCTGGAGGCCTGGGGAGATGGGGGCAGGGCACAAGGCT-AAGCTCAGCACCTTCCTCCA
SARS-CoV-2 Reference Genome Shuffle No.1             (4082) AAAATTAAAGGTGAGGATTTTC-CCAGAGTTACTGATGGTAGTTGATAATAATTAAAATTTCTATGCTACGTCT
```

Section 59

```
                                                (4293) 4293   4300      4310      4320      4330      4340      4350            4366
Homo sapiens chromosome 1 NC_000001.11: 11783698-... (4082) CCCTCCTCACTGTACTCAACTCCTAGGCGGG--CACTAAGAGC---AGCTGCCTTCCTGGACCCACCTTAACCT
SARS-CoV-2 Reference Genome Shuffle No.1             (4155) GACGTGTCCTGAAAGTTAACTATTAAGGTTATTTACAAATATCGTTACGAGAAAACTTGGAAGAATGTTAACGT
```

Section 60

```
                                                (4367) 4367         4380      4390      4400      4410      4420      4430      4440
Homo sapiens chromosome 1 NC_000001.11: 11783698-... (4151) CTTGTGACTCAA-----CTCCCCTTTCCAGGGT-----GCAGGGAACA-----TTCCCTCT-GCCCTG-CCTTC
SARS-CoV-2 Reference Genome Shuffle No.1             (4229) CGAATAATTGATGGAATCGCCCTATACCAGTGCCACAAGCCGAGATTAATAGTTTCATTATAGTGGTGTCCTAG
```

Section 61

```
                                                (4441) 4441     4450      4460      4470      4480      4490      4500          4514
Homo sapiens chromosome 1 NC_000001.11: 11783698-... (4208) CAGCTCTGGCCACAGT--GGCCTGG-------ACAGAGT------A----------GCTGGGTCCCAGGC--CT
SARS-CoV-2 Reference Genome Shuffle No.1             (4303) CAACTTCGGGTAATGTGTGGCTTATGATTGATATATAGTCTTATAACATCATTAATGCTGGTAACAAATTATCT
```

Section 62

```
                                                (4515) 4515 4520      4530      4540      4550      4560      4570             4588
Homo sapiens chromosome 1 NC_000001.11: 11783698-... (4255) GCCCCTGTGGACGAGGGACCAGGGACCAAGAGCGCAC------TCCA-GCCCTGAGCCCA--GAGCCTGCAAAG
SARS-CoV-2 Reference Genome Shuffle No.1             (4377) ATCCGTTTATAGCGGGATAAAGTAATTAACATTGCAAAGGAGTTCCATGTCAATATCTATGTGAGTCTGTAATT
```

Section 63

```
                                                (4589) 4589       4600      4610      4620      4630      4640      4650         4662
Homo sapiens chromosome 1 NC_000001.11: 11783698-... (4320) C---------CCAAAGC---AAGGCCCATAAACGGAGGCTACGGTAAGAGG--AGCTGTGTGTGC-AGTTTGGT
SARS-CoV-2 Reference Genome Shuffle No.1             (4451) CGATATGGACCCTATGTCTTAAGCAAAAAAAATTTCAAATTCGGTTATAGTGAAGCCGACCTAGCTACTATGGA
```

Section 64

```
                                                      4663   4670            4680            4690            4700            4710            4720              4736
Homo sapiens chromosome 1 NC_000001.11: 11783698-... (4379) GGGTCCGAGGGATG---GGGGCAAGGGC--TGAGGGGCTTGCCTG--AGCCATGGCTACA---GCTGGGCCCTC
SARS-CoV-2 Reference Genome Shuffle No.1             (4525) ACTTCTCAGTTATGTGTGGGTTAACTACATTTCTAGTCTAGACTATCATGCAAGGTTCAAAGGGTTGTGAGCTC
```

Section 65

```
                                                      4737         4750            4760            4770            4780            4790            4800       4810
Homo sapiens chromosome 1 NC_000001.11: 11783698-... (4443) ----TCTGGCCAGTGCTCGT-TCCTGCAGGATCCTGGAAAAGCAGGCCCAGGCC-------------CATGGCT
SARS-CoV-2 Reference Genome Shuffle No.1             (4599) CTGCTCTGAGGATAGAAGTTATGCGACGGCTTCCGATAGACTTTGGCGTAAGCCGAAACGAACAATACATTGTT
```

Section 66

```
                                                      4811     4820            4830            4840            4850            4860            4870              4884
Homo sapiens chromosome 1 NC_000001.11: 11783698-... (4499) CTGCCCTCCTTCTGGCCCTG---AAGGGTCACGATGCTCTTGGCCATCAGGAGGAAGTACCTGCAGCGCCGGTG
SARS-CoV-2 Reference Genome Shuffle No.1             (4673) CGCCCCTTATTATCAATGTTGATAAGGTAGACTTCCCATGAGGCGACTTGTACCTTTTAACTGAAGACCTGAAG
```

Section 67

```
                                                      4885 4890            4900            4910            4920            4930            4940                  4958
Homo sapiens chromosome 1 NC_000001.11: 11783698-... (4570) CC-TCGAGGCACTGCTTCACGGGCTGCGGGGTTCCCAGCAGGCCAGGTGCCTGGCAGTGGCATGGCAGCACTGG
SARS-CoV-2 Reference Genome Shuffle No.1             (4747) CCATTCAATCTGTCATTTACGTGCAATGAAGTTGTCTATAC-CGAATTGCGTTGAAGTTTGCTCATAGCTATTC
```

Section 68

```
                                                      4959        4970            4980            4990            5000            5010            5020            5032
Homo sapiens chromosome 1 NC_000001.11: 11783698-... (4643) GTGGATGCCCAAGGGGCAGAGCAGCTGGTGCG-GACCCTGGTG-AGTGGGTGCACCCCTC-TCCACACTGACCA
SARS-CoV-2 Reference Genome Shuffle No.1             (4820) GATTTTGGCTAATCCG-ATTCCTGTGAGGGCGAGTTAATGACGTACTGGGTATGCTTTTTGTAAACACTTCCGG
```

Section 69

```
                                                      5033   5040            5050            5060            5070            5080            5090              5106
Homo sapiens chromosome 1 NC_000001.11: 11783698-... (4714) TCTCCCATT--TCACCTCATACTTAACCCAAACCCCTCTCAAAAGTATGGCCCTTGGA------CCTACTGACT
SARS-CoV-2 Reference Genome Shuffle No.1             (4893) ATACCAATTCGTCACGCCAGTTTCTGCGGACAATGTTTTTAAATTTGAGACACAATGAAGATGTCATTACGGAT
```

Section 70

```
                                                      5107         5120            5130            5140            5150            5160            5170       5180
Homo sapiens chromosome 1 NC_000001.11: 11783698-... (4780) CCAAAACCTTGGGGGACAGGCCCAGGAATCTGCATTCTTAATACT-CCTCAGATGACACCCCCTTGCCATAGCC
SARS-CoV-2 Reference Genome Shuffle No.1             (4967) ACAATATCTCGATACACTGACTGTAGG-TCGGTTCCCTTGTTTGTACCTCAGACATTAAGTGGTTTACTTATTA
```

Section 71

```
                                          (5181) 5181     5190      5200      5210      5220      5230      5240          5254
Homo sapiens chromosome 1 NC_000001.11: 11783698-... (4853) CTTTCACTCTGGTGCAGCAGTGTCGGGGGAGAAAGGCAGAAGG-GCTGGGGACTGCGGGGGAGCGTGAGCACAA
SARS-CoV-2 Reference Genome Shuffle No.1 (5040) GTATTATAAATTTAATTCAAAAGCAAAGTATTCGGGTAGAAAGCGCTGTGCATTTTGGTCTAATAAAAGAACAA
```

Section 72

```
                                          (5255) 5255 5260      5270      5280      5290      5300      5310              5328
Homo sapiens chromosome 1 NC_000001.11: 11783698-... (4926) GAGGCCT----TCTC---TGCCAACTGT--CCCCA--AAGCTCAGGGAGTGG--CACCTGAGATGGCCTGGCA
SARS-CoV-2 Reference Genome Shuffle No.1 (5114) AGACCCTAGGATGTTCATTGCTTATTTTGACTCCATTAAGTACGGCTGGTGCTTAATCTAAACTAAGCATATC-
```

Section 73

```
                                          (5329) 5329       5340      5350      5360      5370      5380      5390        5402
Homo sapiens chromosome 1 NC_000001.11: 11783698-... (4987) GACGTGGTGGCAGCGGATCCTGC-----GACTGCGAGTGGCTCAGCAGTTACAGCAGCAGGAAGATGGGTGG--
SARS-CoV-2 Reference Genome Shuffle No.1 (5187) GAGAACTTTTCAATAAAAAATGCCTAATGAGTTTCAGGAAATCATAACAAATTTAAATATGTGTCTAGGTTGAG
```

Section 74

```
                                          (5403) 5403   5410      5420      5430      5440      5450      5460            5476
Homo sapiens chromosome 1 NC_000001.11: 11783698-... (5054) -GTCCTTTCCCAGGT----------ACTGCCCT-CTTCCCTGC---ATTCCACTCAGCCAGCCTTCC----AGAA
SARS-CoV-2 Reference Genome Shuffle No.1 (5261) AGACCGTGTCATGTTTAAACGATAAATGCTCAACGTGTCTAACTGAGTACAATAACGTATCCTCCTGCTAATAT
```

Section 75

```
                                          (5477) 5477         5490      5500      5510      5520      5530      5540    5550
Homo sapiens chromosome 1 NC_000001.11: 11783698-... (5110) GTCTGTGCCACAGCC-ACGCACCGTGGAGCTTTC--CCTGCT---TGCCAGCCCCGGGGCCCCTTCGTTTTCAG
SARS-CoV-2 Reference Genome Shuffle No.1 (5335) GACTCCCCCAAAGTATACTCACTTAAGACTTGTACACCTATTTAGTGCAAAAAGTAGGGCTGTTTAGATAACAG
```

Section 76

```
                                          (5551) 5551     5560      5570      5580      5590      5600      5610          5624
Homo sapiens chromosome 1 NC_000001.11: 11783698-... (5178) GGGAGGG--GGTCAGTTCC-ATTACAGAACACACAGGCTGGATGCTGGAGCCCTTGCTTTGATTTGTGGCCCCA
SARS-CoV-2 Reference Genome Shuffle No.1 (5409) TATAGAAAAGTTCGGATAATATTCTAGTGTTTTGATAGTGATAGAATAAGGCGAT-CTATAATTATCACACATA
```

Section 77

```
                                          (5625) 5625 5630      5640      5650      5660      5670      5680              5698
Homo sapiens chromosome 1 NC_000001.11: 11783698-... (5249) ---GCTCAGGCCAGG--CCCACCCGGTCCATCAGTTTGCGAAGAAGGTGTGGCATGAGGCATGGCTGCCACTCA
SARS-CoV-2 Reference Genome Shuffle No.1 (5482) AATGGTGACGCGTCGTACCCAGCCTGTAGAATAAATTAACCACATTCCTTTAAATAT--CTAGACTAGAAATTT
```

Section 78

```
                                                     5699       5710      5720      5730      5740      5750      5760         5772
Homo sapiens chromosome 1 NC_000001.11: 11783698-... (5318) CACTAGCCCATGGAGGGCCTGGGTCCTGAGCGCCCCCTCCC-TCATTTGCTCTGCTCTGAGTACAGCTTCCCAG
SARS-CoV-2 Reference Genome Shuffle No.1             (5554) CA-TAGCCATTGGCGAAAACCTTTTATCAACGATCAATGATATTAACTTATTTAAAGGAATTATATATTCTTAG
```

Section 79

```
                                                     5773   5780      5790      5800      5810      5820      5830           5846
Homo sapiens chromosome 1 NC_000001.11: 11783698-... (5391) CTCACCATCAGATCTATTCTGAGCTTGTGCATTTGGCCTAGGCAAGACTGGCTGGCTGCTTGGGGCAGAGGGAG
SARS-CoV-2 Reference Genome Shuffle No.1             (5627) -TCTGTTAAATGTGGATTCGGCGAGTAGCAAAATAGCATCGGTTAAACATCATTCCCGTTTGATACGGGCTTAT
```

Section 80

```
                                                     5847         5860      5870      5880      5890      5900      5910   5920
Homo sapiens chromosome 1 NC_000001.11: 11783698-... (5465) GGGCCCTGGGGACAAAAGGAGCTGGGTACAGGGGTTGCTCTAGCAGGCACAGCACAGACCCCGGAGAAAGCCCA
SARS-CoV-2 Reference Genome Shuffle No.1             (5700) ATCAAATCTGCAAGCAAGATGCTG-----ACGTCTCGTTTTCGGAGTCACAGTATATTGTC---ATTGAGTTTT
```

Section 81

```
                                                     5921     5930      5940      5950      5960      5970      5980         5994
Homo sapiens chromosome 1 NC_000001.11: 11783698-... (5539) CAACAATAGCATTTCCTCTCCTCCCTGGTTCCAGGCCTTTAAGAA-------GTGGCACCAACGCCTG---GCA
SARS-CoV-2 Reference Genome Shuffle No.1             (5766) CAACAGTTGGGCTGATGTTAATTCCAG-TTCTAGATTAATCAGAATATATATTTCGATATAGAGACTAACTTCA
```

Section 82

```
                                                     5995 6000      6010      6020      6030      6040      6050           6068
Homo sapiens chromosome 1 NC_000001.11: 11783698-... (5603) GCCAGGAGCCCAAGGAGAGGAGCTGCCA--GTAGC--CCAAGACCCTGGAGCAAGCCAGGCCCCAAG---GGCC
SARS-CoV-2 Reference Genome Shuffle No.1             (5839) GGTCTGAACATTTGTCAATACTCTTCAACTGTACCTACCTATTATCTTTCACATTCC-GTCCATATGTATTGCG
```

Section 83

```
                                                     6069       6080      6090      6100      6110      6120      6130        6142
Homo sapiens chromosome 1 NC_000001.11: 11783698-... (5670) CCGAGAGTGGACAGGAAGCCGCCAGAGCACCGCGGGGTTGGGGGCTTG--GGGCA--GAGCATGGGGCCCAGCT
SARS-CoV-2 Reference Genome Shuffle No.1             (5912) ACGGTATTCCTCGCTCAACATTTAAATCATGCAAAATCAGTTGTCTTGTCGCGCACCGGGTAGATGGCAGAGTA
```

Section 84

```
                                                     6143   6150      6160      6170      6180      6190      6200           6216
Homo sapiens chromosome 1 NC_000001.11: 11783698-... (5740) GCAGC------TGTGACTTGTTCTCATAGAA----TAAAAAAC-AGAACTGAACTTAGCCTTCC---CAGGGAA
SARS-CoV-2 Reference Genome Shuffle No.1             (5986) GTAGTATACCTTCTTACATGTACTACGAGCAATACTAGAAAACGAGTTGGGATCTTCGCATTCCATTCTGTATC
```

Section 85

		6217 … 6290
	(6217)	6217 6230 6240 6250 6260 6270 6280 6290
Homo sapiens chromosome 1 NC_000001.11: 11783698-...	(5800)	GGAACCATGCC---CCACACATCCAGGGAGTGATGACAGAGGGGACAGCTTGAAGAGCTCTGAAGGACAATAAA
SARS-CoV-2 Reference Genome Shuffle No.1	(6060)	GGATTCCTTCGGAAACAAAAATTTATGCA-TGATCACCAAAGTAATTACTGAATGCATTAAGCATGATCATGAT

Section 86

		6291 … 6364
	(6291)	6291 6300 6310 6320 6330 6340 6350 6364
Homo sapiens chromosome 1 NC_000001.11: 11783698-...	(5871)	ACCCACTCCTCAGTCCTAGCTGCT---GCTTTCAGTCTCTC-CTAGCGGCTGCCCCTCTGCCCCAGCCAGTGAG
SARS-CoV-2 Reference Genome Shuffle No.1	(6133)	AAGTTAAAGTCACTAATTGAGGAATAAGCATATAAGCACTCACTCAACGTTACTGCTTTTGTCGTATAAGATTG

Section 87

		6365 … 6438
	(6365)	6365 6370 6380 6390 6400 6410 6420 6438
Homo sapiens chromosome 1 NC_000001.11: 11783698-...	(5941)	GCCAGGAAGGGAGTAAAGAGGACATCCCACAATTGCGTCCACCCCCTTGGCCATCAGGCTGCTCTTCCACTACA
SARS-CoV-2 Reference Genome Shuffle No.1	(6207)	---AAAAACAGTATAAAGAGTGATTTCTAG--TTCAGACGAACG---TGGGCAATTTGCATCACTCCGTATGTT

Section 88

		6439 … 6512
	(6439)	6439 6450 6460 6470 6480 6490 6500 6512
Homo sapiens chromosome 1 NC_000001.11: 11783698-...	(6015)	CGAAACAGCCCATCTGCACCTGCCAGTCACTGCCCCCAGGCCCTGGGGCTTGCAGGTGACTCGTGGGGAGCCAG
SARS-CoV-2 Reference Genome Shuffle No.1	(6273)	TGAAGCAAGGTGGCCGCTGTTG---GTT-CGGCTTCAAA---CTGTTAGTTACAGG-GAGAGAAGGTCTTCGAC

Section 89

		6513 … 6586
	(6513)	6513 6520 6530 6540 6550 6560 6570 6586
Homo sapiens chromosome 1 NC_000001.11: 11783698-...	(6089)	GCCAGGCAGATAACCTCTGGGACAGGACGAGGGGATGAGGAAGGCAGCTGGCAGGAC---AAGGGCCTACCTGA
SARS-CoV-2 Reference Genome Shuffle No.1	(6339)	TATTATCGGGAATGTGCATGGGTATCGGGTAATTTTTTCGTAATCTTTAGCCACGACTTCACGTTGAAATGAGA

Section 90

		6587 … 6660
	(6587)	6587 6600 6610 6620 6630 6640 6650 6660
Homo sapiens chromosome 1 NC_000001.11: 11783698-...	(6160)	AAAATAC-ACCAGGAAGCTGGGCTGCTGCACAGGCCCAGGCAGATCTGGGGCCCCTGTGCTGGCAAATCT---C
SARS-CoV-2 Reference Genome Shuffle No.1	(6413)	AAAGTGCTACTAAATATTTCGACTAGTGGTGTAAAATGGGCT-ATATTTCCCGCCAGTACTTGTTGATTTATTA

Section 91

		6661 … 6734
	(6661)	6661 6670 6680 6690 6700 6710 6720 6734
Homo sapiens chromosome 1 NC_000001.11: 11783698-...	(6230)	ATCT--TAGCCTCTGACAGGGAACGGGGCAGCATCAGGCCTCCACAGCCAGGCAAAAGGGGAAGGCCACGAGCC
SARS-CoV-2 Reference Genome Shuffle No.1	(6486)	ATGTAGTGGCCTATCATACTTACGAGTCTTTTTTTAATTCATCGTGTGGAGAATACTTGTAAGGGCGTTCCGTC

Section 92

```
                                                   (6735) 6735 6740      6750      6760      6770      6780      6790              6808
Homo sapiens chromosome 1 NC_000001.11: 11783698-... (6302) CCACCAAGCACAAGGGTTCAGACTGCCTTCAACCCTTAAGGGGCACTTTT-----TCTCTCAAGGCT---CAGA
      SARS-CoV-2 Reference Genome Shuffle No.1 (6560) GGTTACTTTTCAAAATTTTTG--TGACTTCAAGAGTAAATCATAAGTTTATGAAATCTATGAATCCTTTACAGC
```

Section 93

```
                                                   (6809) 6809       6820      6830      6840      6850      6860      6870         6882
Homo sapiens chromosome 1 NC_000001.11: 11783698-... (6368) ATAGACAACTGTGCCAAAAAGCAGCATGGTAGGTGTCAGCCCAAT--GCCCATCCAGGGGGCTGGTGCCATGGA
      SARS-CoV-2 Reference Genome Shuffle No.1 (6632) GAAGATTAAAGGCACAATAATGGGTCTGTTATGGGACATCGGTATCAGATGTGCCTGGTTAAGTGTGCGTGAGA
```

Section 94

```
                                                   (6883) 6883   6890      6900      6910      6920      6930      6940             6956
Homo sapiens chromosome 1 NC_000001.11: 11783698-... (6440) CG-GCTAAGTGGGCAGGCAGGGCAGTGGTGT---GCCC-CTTCAGTC-ACAA-GTAGGTGGGGCCCAGCTGCCC
      SARS-CoV-2 Reference Genome Shuffle No.1 (6706) AGTGCTCGGATTCGACGCGTCTCTAACGTATATCGCCGTCTACCGTGTACAACGATGGCGGTTCCATACTATCC
```

Section 95

```
                                                   (6957) 6957         6970      6980      6990      7000      7010      7020     7030
Homo sapiens chromosome 1 NC_000001.11: 11783698-... (6507) ACCTCTAGGAGGTGGAAGGGTACA---GCACTTTGCCACATCTCTTCTACGATGCCACCAGTGCCTGATGCCCT
      SARS-CoV-2 Reference Genome Shuffle No.1 (6780) ACAT-TAGACTGGTTAAGGGAACATTAGCACTTCGGATTAAACCATCCACGCAGA-ATCA-TGCACAATTCCTC
```

Section 96

```
                                                   (7031) 7031     7040      7050      7060      7070      7080      7090          7104
Homo sapiens chromosome 1 NC_000001.11: 11783698-... (6578) TGCGTGTTTTGCCTGTACTGCACGGGCTCC-AAGTGTAAGTTCACTGGGAGTCCCAGGCTGGCTCTGGAGGCTG
      SARS-CoV-2 Reference Genome Shuffle No.1 (6851) TTGACGTTA---CTATCGTGCCCAGTGTAGGAAGTCTTAAAAGA-TGGG-GTATTGTGCCCATTCTG-----TC
```

Section 97

```
                                                   (7105) 7105 7110      7120      7130      7140      7150      7160              7178
Homo sapiens chromosome 1 NC_000001.11: 11783698-... (6651) CCTTGGTTCGAGG-GCTTAGTTGGCAACAACCACTCTCCTCGCCTTCTCAGGAGGTGCCACC--CACTGTGGTC
      SARS-CoV-2 Reference Genome Shuffle No.1 (6915) CCTGAGTCCGAGGCGATTACGTGGCATGTAAAAGATGCCTAAGCA-CGAAAGACGTACGGTCACCATAGTTATC
```

Section 98

```
                                                   (7179) 7179       7190      7200      7210      7220      7230      7240         7252
Homo sapiens chromosome 1 NC_000001.11: 11783698-... (6722) CTCTGCAGGAC---GCAAGTGC-----TCCCCACCAAAAGAGCACAGACTCCTGGCTAGAGCAGAGAGTACTAG
      SARS-CoV-2 Reference Genome Shuffle No.1 (6988) ATTCCTAATATATAGTAACTGCATGTTTCTCCTGCAAAATATTTCAAAAAGGTG---ATGTCAGAGTTAAACAG
```

Section 99

```
                                                            7253   7260                7270                7280                7290                7300                7310                7326
Homo sapiens chromosome 1 NC_000001.11: 11783698-... (6788) GTTCCCATGTGGGGCCGACTCAGGTCCAGGAAGAGCCTGGAAGCCTCACTCCAGTCTAGCTGCCATTGTCAGGT
SARS-CoV-2 Reference Genome Shuffle No.1            (7059) TTTCGCATTAGATGATAATGCTGACCCTTTAAGTAGGGTGCATTTTCAATGAA-TCTTAATATCCT----AGAT
```

Section 100

```
                                                    (7327) 7327 7340 7350 7360 7370 7380 7390 7400
Homo sapiens chromosome 1 NC_000001.11: 11783698-... (6862) GGGGGAGTGGAGGCCGGGGTGGGAGAGACACGAAG---GAGAGTGGAGTTCCCAAGAGAAGCAGCACTGTGGAG
SARS-CoV-2 Reference Genome Shuffle No.1            (7128) GAGAAACAACAGGT-GCTGTGGTGCACATACGAGGTTCGTGTTTGGACTT---ATGCCATCTAGAAAGCTTTAG
```

Section 101

```
                                                    (7401) 7401 7410 7420 7430 7440 7450 7460 7474
Homo sapiens chromosome 1 NC_000001.11: 11783698-... (6933) GAGGAAGGCGGGACAGGAGTGGCTCCAACGCAGGGCGTCAGGACGCAGGGTCA--TGGAGCCTCCGTTTCTCTC
SARS-CoV-2 Reference Genome Shuffle No.1            (7198) CAACACATCGGTTT-----TACCTGAAAATTATATCGGTGCTTC-CAGGGAGAATTTGAGCAATAGCTCCTCGA
```

Section 102

```
                                                    (7475) 7475 7480 7490 7500 7510 7520 7530 7548
Homo sapiens chromosome 1 NC_000001.11: 11783698-... (7005) GCATTCTGGGTGGGCCTGTTGAGAA-GCTCCAATGTGTCTTCCA---CCACCTGCCAGAGGCAGTTGTCCAGTG
SARS-CoV-2 Reference Genome Shuffle No.1            (7266) GCGG---GTATAAGGGTGTTTAGGCCGTACCGATGGGGCTAAGGAATACAACTGAATTCTGGATTTTACAAATT
```

Section 103

```
                                                    (7549) 7549 7560 7570 7580 7590 7600 7610 7622
Homo sapiens chromosome 1 NC_000001.11: 11783698-... (7075) GGAAGTCATTGTCCACCAGGT----TGACCAGGAAGTAGTTGTCGTGGATGTAC-TGGATGATGGTGCGGGACG
SARS-CoV-2 Reference Genome Shuffle No.1            (7337) TGGACGTTTTTCACACGAGGACTGATAACGAGAATATCGGCAGCCTGAGCGACTATTCAAAACTATATTCTATT
```

Section 104

```
                                                    (7623) 7623 7630 7640 7650 7660 7670 7680 7696
Homo sapiens chromosome 1 NC_000001.11: 11783698-... (7144) GGGACTCCTCCTCATACAGCTTTCCCCACCGCTCAATC-CACAGGGCAAAGGCCTCGT--CCTACACACACATA
SARS-CoV-2 Reference Genome Shuffle No.1            (7411) GGTATAGGGATAGAGAAGGCTAGCATCAGAGTTTTATTGCGTTGCTAAAATGTTTTGTTACAGACAGTCTCATA
```

Section 105

```
                                                    (7697) 7697 7710 7720 7730 7740 7750 7760 7770
Homo sapiens chromosome 1 NC_000001.11: 11783698-... (7215) CCCCCGCACACACGCACACATGCACACACACACAGAGGCAAGATTAACAGGGCTCTCCAAGGGCTCTGCTCAAC
SARS-CoV-2 Reference Genome Shuffle No.1            (7485) TTGAGGCT-ACA-GCGAATCTCCTCACTAACGTCTAGTTATTCTTATCAAGCCCCAATTATAAATTAGTAGTTC
```

Section 106

```
(7771)                                                      7771     7780      7790      7800      7810      7820      7830          7844
Homo sapiens chromosome 1 NC_000001.11: 11783698-... (7289) AGGACATTT--CCCGGAATAGAACAAGGCCCACTGCCAGTATCACTTCCCACCCCTGCCCAGAAATAAACACCC
SARS-CoV-2 Reference Genome Shuffle No.1            (7557) TTGACTTTTAAGCATGCATA-ATCAATAT--AGTTCCAGTAGA--TTGGAGCTGCTATAGATTGATTTATATGA
```

Section 107

```
(7845)                                                      7845 7850      7860      7870      7880      7890      7900              7918
Homo sapiens chromosome 1 NC_000001.11: 11783698-... (7361) TGCATCAGCAGTGACATGGGTGAGTCAGA---GCTGGG-TGTGCCCTGAAGGCTGGGTCCAG----TGTGGCCT
SARS-CoV-2 Reference Genome Shuffle No.1            (7626) CTGATAA--ATTGAAATGGTACAGACTCAATGGCTACAATAAGCTAGCAAAAATCCGTAAAAATCTTGTCCCCT
```

Section 108

```
(7919)                                                      7919       7930      7940      7950      7960      7970      7980      7992
Homo sapiens chromosome 1 NC_000001.11: 11783698-... (7427) TCAGCACACGCTTGGGACTTGGTCCCAATCCCTCCCCA--CGGTTTTCCAGGTGGGCGGGG-CAAGCTTGCCCC
SARS-CoV-2 Reference Genome Shuffle No.1            (7698) TAAAATTATCAGAAAAACACGGTATATAGCACGGCAGAAATGGCTCTACTTATGGCAGAAATCCCGACTTTCAA
```

Section 109

```
(7993)                                                      7993   8000      8010      8020      8030      8040      8050          8066
Homo sapiens chromosome 1 NC_000001.11: 11783698-... (7498) CGGCTCCTT---TACCTTCCAGAACATGAAGCTGACGGGATCC-ACTACGGTGGGCTGGATGATCTCTCGCCCA
SARS-CoV-2 Reference Genome Shuffle No.1            (7772) AAACTCGTTAGTGGCACACCGGTAGGTAACTCGGATGTGAAAATAAGACCGGTCGTTTGATAAATTTTATTTTA
```

Section 110

```
(8067)                                                      8067         8080      8090      8100      8110      8120      8130   8140
Homo sapiens chromosome 1 NC_000001.11: 11783698-... (7568) GGGAAGATGCCCCAAGTGACAGCA-TTCGGCTGCAGTTCA---GGGGCAT-TGGTGATGTTTTCACCCTGAGGG
SARS-CoV-2 Reference Genome Shuffle No.1            (7846) G--AAGCTGTGCAACGTTATAGCAGTACCTCTGTATCCCAATAACGGAATATTATAATCGTTTGTCACTGTATC
```

Section 111

```
(8141)                                                      8141     8150      8160      8170      8180      8190      8200          8214
Homo sapiens chromosome 1 NC_000001.11: 11783698-... (7637) GATGTGGGGTGGGG---GTAA----GACCTGGGCCAA--GAACAACTGGA-GTCCCACTGCCTG-GACAGCCTT
SARS-CoV-2 Reference Genome Shuffle No.1            (7918) TTTCTAGTCTGAACTTAGTAATGATAACCAGAGTTTACGGAATACTTTCAAGTGTATTTGTATGTGATAGGAGG
```

Section 112

```
(8215)                                                      8215 8220      8230      8240      8250      8260      8270              8288
Homo sapiens chromosome 1 NC_000001.11: 11783698-... (7700) -GACTCAATGAGGCTGTCTGG-GCTCAATACCCTCTGCCTGCGCCTCCACTCCCGGCCTCCACTCTCCCTGCGC
SARS-CoV-2 Reference Genome Shuffle No.1            (7992) AGATTTTTTCAAGCTTTGTAGAGTTAGAAAAACTAATCCGGGGCTCAGGATCGGAATCTTCGGGCAAGATAACA
```

Section 113

(8289) 8289 8300 8310 8320 8330 8340 8350 8362
Homo sapiens chromosome 1 NC_000001.11: 11783698-... (7772) CTCCACTCCCCTCTCCCTGTGCCTCCGCTCCTGGC-CTCCTCTGCCTGCATCTGTGAATGATATCTCTCCCCAC
SARS-CoV-2 Reference Genome Shuffle No.1 (8066) CACTGTTTATATCCAGTTATATCTAGGTTGGTCGAACTTCAGTAGATTAAAACGAATATTTTTTGTATGTTGAA

Section 114

(8363) 8363 8370 8380 8390 8400 8410 8420 8436
Homo sapiens chromosome 1 NC_000001.11: 11783698-... (7845) ACAAGCCTGAACCTAGAGGCGTC--CTGGCCACTTCCCCTCTCCTCCTTGAGCACCCCCTGCTCACTCTCTGAT
SARS-CoV-2 Reference Genome Shuffle No.1 (8140) AGACAGTTTGATTGAAAGTATTCTGCTACACAGTTACAGTTTGAGCAGTAAA-ACTACATTTGAAAACAATTAT

Section 115

(8437) 8437 8450 8460 8470 8480 8490 8500 8510
Homo sapiens chromosome 1 NC_000001.11: 11783698-... (7917) CCATTCCCTCTTGTCTAGCCCACCTCCAGG-------A-GTGTGTTTGGCTTGGGTCACTCCAGGGGTCTCTTC
SARS-CoV-2 Reference Genome Shuffle No.1 (8213) TTTGTACC-CTTTCCTAGTCTGCATGTATGTCATTCCAAGAAGGGTTGGAGTACGGGATTTTAGTCATTTGTTA

Section 116

(8511) 8511 8520 8530 8540 8550 8560 8570 8584
Homo sapiens chromosome 1 NC_000001.11: 11783698-... (7983) ACTGGCCT--CCCCGGCTCTAGCCGTCCCCAATCTCTCTCATCCGTATGACTATCCACTGT--GATTTGCCTAA
SARS-CoV-2 Reference Genome Shuffle No.1 (8286) AGTGTTTTGTACCCGTCGGCACTTATTCAAATTTTAAATGCACCATTTGTTTATTCTCGCAAGCAATCGAGTCT

Section 117

(8585) 8585 8590 8600 8610 8620 8630 8640 8658
Homo sapiens chromosome 1 NC_000001.11: 11783698-... (8053) AATAAAGCAGGAACCAAGCCACCCCACTGCTGGAGAAAGGCTTCCCTTGCATT---GAGGGAAAACTCAAATTC
SARS-CoV-2 Reference Genome Shuffle No.1 (8360) GTTTTCCTAAGATTGA-GTTATCC-AGTGAAGATAATAAATTATCTTTGCGTTATCGAAGCGAAACAAGCATAA

Section 118

(8659) 8659 8670 8680 8690 8700 8710 8720 8732
Homo sapiens chromosome 1 NC_000001.11: 11783698-... (8124) CAGCAGGACAACCATGATCTCTGCGGGCCTGGCCCCTGCTTAACACTCCCGCCTCCTCCTGCTCATGTTCCCCG
SARS-CoV-2 Reference Genome Shuffle No.1 (8432) TATCT--ATCGCAATGATCACA-CAGGATTGTGGGGGCCTAACGTCACTTGACTGGTCATAGACGTTTAAACCG

Section 119

(8733) 8733 8740 8750 8760 8770 8780 8790 8806
Homo sapiens chromosome 1 NC_000001.11: 11783698-... (8198) TTTCCCCAGATGGTGCAGCTCACTGCCTCTGCCTGTCCTCTGCCCTGTCCCAGGAATACCCTTCTCTCCTGTTG
SARS-CoV-2 Reference Genome Shuffle No.1 (8503) ATTAC--AGTTGGA--ATTTTTTTTTCTATACCCCGTGTATATTTTG---CTGTTTTAAAATTTGCGCTTTTTA

Section 120

```
(8807) 8807         8820      8830      8840      8850      8860      8870    8880
Homo sapiens chromosome 1 NC_000001.11: 11783698-... (8272) GCCAACTCCTCTTTGTCCTCAGTG-ATTCCCACAGATGACCCTCTGAGAAGCCTTCCCTGGCCTCCCAGGTGAG
SARS-CoV-2 Reference Genome Shuffle No.1 (8570) TCCGAGACGTGTAGAAACTGATTTTATAGCTAGAGTTGATA----GACACGCGTGGAATTGGTTCGTCAGTGC-
```

Section 121

```
(8881) 8881     8890      8900      8910      8920      8930      8940        8954
Homo sapiens chromosome 1 NC_000001.11: 11783698-... (8345) ATATCCCTCCTCTGGACCCGACAGCATCCTCTCCTGAACTTGATTTTCCCACTTA--CACTGGCTGGTGGTCAC
SARS-CoV-2 Reference Genome Shuffle No.1 (8639) -TTTCAGTT-TCTTGATT-GATAAT-TCGTATTCTGCCCTTG-TTATCCGACTTATGTATATAATAGCCCTCAT
```

Section 122

```
(8955) 8955 8960      8970      8980      8990      9000      9010              9028
Homo sapiens chromosome 1 NC_000001.11: 11783698-... (8417) ACATTGATCTGCCTCCCTCACTCTAGGCTGGAGCTCCTTAAGAGCAGGCCCCAGTTCTGCCTGTATCTCTCT
SARS-CoV-2 Reference Genome Shuffle No.1 (8708) AACAGGAGCCTCTTTTCGCCTTTTATACCGTCTTCCATCTAAGGGCCAGGCCTT--TACTGA-TGTATGT-TGA
```

Section 123

```
(9029) 9029       9040      9050      9060      9070      9080      9090      9102
Homo sapiens chromosome 1 NC_000001.11: 11783698-... (8491) GTCTTGCACAATGCCTAGCCCAGGCTAGGTGC-TGGGTGTTGC-TCAACGAAGGGCCTGGTACTCTGTGGGAA
SARS-CoV-2 Reference Genome Shuffle No.1 (8778) GTCTTGTCG--TGGTTAGCC--GGATGGGTGGATGATTTTACGCATAAAGGAAAATTCGCGTTATACATGTCTT
```

Section 124

```
(9103) 9103   9110      9120      9130      9140      9150      9160            9176
Homo sapiens chromosome 1 NC_000001.11: 11783698-... (8563) CCCTGGGGCCTGGCCTACCTTCACA----TTGACAAGGTGGTAATTAACCCGGAGC----TCGTACTTCTTCAG
SARS-CoV-2 Reference Genome Shuffle No.1 (8848) TC--GGTTCACCGACTAAATTATAAAGTTTTGACAGGGGTAGATTAAAAGCTAACCAATATTATACTATAGCGG
```

Section 125

```
(9177) 9177         9190      9200      9210      9220      9230      9240    9250
Homo sapiens chromosome 1 NC_000001.11: 11783698-... (8629) CACTTGCAGAAGTGCT-------TCCGCTGTCTCGCGGGAAGTGAAAAACTCTAAGTAGGCCTGTGGGAGAGAC
SARS-CoV-2 Reference Genome Shuffle No.1 (8920) AGATGACGGAGGAGCTCAGAGTCTTTAATTTATACAATTATTTGAGAATATTTAAAGAAAGTAGCGGAGGCGTC
```

Section 126

```
(9251) 9251     9260      9270      9280      9290      9300      9310        9324
Homo sapiens chromosome 1 NC_000001.11: 11783698-... (8696) AGGTGCCTGTCACCAACCCCAGCCCTGCCAGACGCCCTTTCGCAGAC---ACCTAAGGTATCAGGACTACTGGC
SARS-CoV-2 Reference Genome Shuffle No.1 (8994) --GTGGGTAGAATTTTTTACAGTTC-GAAAGACGC--TTTCAATTACTAGATTTAATATATTGAATCGA-TCAC
```

Section 127

```
(9325) 9325 9330 9340 9350 9360 9370 9380 9398
Homo sapiens chromosome 1 NC_000001.11: 11783698-... (8767) AGCCATGTGGGCGGAATAATGAACTGTGGCGTTGGCACGTCCATAAGCAAAGGGTGAGGGGAGTGGGGACGTGA
SARS-CoV-2 Reference Genome Shuffle No.1 (9062) TTCTAAGAGT-CGAAATACTCAATAAATACTATTGAGAGTGAATTGTGATATTATCATTATACGTAATTAGTAA
```

Section 128

```
(9399) 9399 9410 9420 9430 9440 9450 9460 9472
Homo sapiens chromosome 1 NC_000001.11: 11783698-... (8841) GTTTCTCAGTCTGGACAGGTCTGAGGGCTATCAGCCACCCCTCTCCCAATCA-GTCAGGGAAGACTCC-ACGAA
SARS-CoV-2 Reference Genome Shuffle No.1 (9135) --TCCATTGACGCGAAATTGCAAACATGTATCAGTTTGATACGACAAACTCGTGTCTCTTTAGATACATATGGT
```

Section 129

```
(9473) 9473 9480 9490 9500 9510 9520 9530 9546
Homo sapiens chromosome 1 NC_000001.11: 11783698-... (8913) GAAGCCATTTCTGAACAGCAGCAACTGGTTTGACAGG---CCGAGTCATGGGAGAGAGGCCCCACACATTCCAT
SARS-CoV-2 Reference Genome Shuffle No.1 (9207) AATCGCGGTTCTGTC--GTTGACACTATACAAACATGTAACTGTTACTTTGCACAACTTAACCTGAAATTACGG
```

Section 130

```
(9547) 9547 9560 9570 9580 9590 9600 9610 9620
Homo sapiens chromosome 1 NC_000001.11: 11783698-... (8984) CCTCCCCAGTGCAAGCTGACAATGACATTGACATCTCCTCTAGAACCAGTGCCTATCAGG--GATTAAGCTGGG
SARS-CoV-2 Reference Genome Shuffle No.1 (9279) TATATTAATCGCGTGTATAAGTGTACACTGAT-TCTTATCTAACAT-AGGGTTAAACGGAATGTTCAATTGAGA
```

Section 131

```
(9621) 9621 9630 9640 9650 9660 9670 9680 9694
Homo sapiens chromosome 1 NC_000001.11: 11783698-... (9056) TTTACTCAA---ACTCATTTGCTAGAATGCTGATAATA------CGGGAGGCTGGGCA-CAGTGAGCCCCACGT
SARS-CoV-2 Reference Genome Shuffle No.1 (9351) ATTACTGGACTGAATTTTTTCTGAGGATGTTAACCATCTTGTCTAGAAAGGTTCAGAATCGGTAATCTCGAAGT
```

Section 132

```
(9695) 9695 9700 9710 9720 9730 9740 9750 9768
Homo sapiens chromosome 1 NC_000001.11: 11783698-... (9120) GAGGAAAGTGGGTTCAGGGAAGTTGAGCACGTTGGCTACTCTGACAGTAAACAGTAGAGCTGGTCCTTAAACTC
SARS-CoV-2 Reference Genome Shuffle No.1 (9425) G-GGATCTTGCTATGAGTCAACCGGGCCTAGTAAACGATTGACTTATTTGAATATTGAATTAGTCATGATTTTG
```

Section 133

```
(9769) 9769 9780 9790 9800 9810 9820 9830 9842
Homo sapiens chromosome 1 NC_000001.11: 11783698-... (9194) AGGTCTTTCTGCTCCAGGGCCTGTGGG-CCTTAGATCAGAACATAATGTTAA-CTCCAGCAGCCCTGGGAGAAA
SARS-CoV-2 Reference Genome Shuffle No.1 (9498) ATATTTTTATTCT--AGCTCATGAGTCTCATAATATTGTTAGCTAAGACGAATCGAAATCGCGGCTTAGGGTCG
```

Section 134

```
                                                (9843)  9843   9850      9860      9870      9880      9890      9900          9916
Homo sapiens chromosome 1 NC_000001.11: 11783698-... (9266)  GGTGTCAACTCAGGATAGTAACAGCAGCTGCCTTTCAGGGA---GTGCTTCCTGCTCTTACCATGTGCCAAGCA
SARS-CoV-2 Reference Genome Shuffle No.1             (9570)  GCTTCTGTCTGAGTATGGAAAAGTTAGCTACAATTCTAATATCTCTATTTAAGATCTAGAGGAAGCCCATTCT
```

Section 135

```
                                                (9917)  9917        9930      9940      9950      9960      9970      9980     9990
Homo sapiens chromosome 1 NC_000001.11: 11783698-... (9337)  CGTTACATGCTTTGTTAGGTTTCATGCCCAGAACACAA-GCTACATCCCATTTTACAGACGAGAAAACCAGGCT
SARS-CoV-2 Reference Genome Shuffle No.1             (9644)  GGTGTCATGGTAATTGAATTCCCATGGTCTATTTATTGCGCAGCCGTTCAGTCTC--GATGTTTTCTTTAGATC
```

Section 136

```
                                                (9991)  9991     10000     10010     10020     10030     10040     10050      10064
Homo sapiens chromosome 1 NC_000001.11: 11783698-... (9410)  CAGAGAGG---TCGAGTGACCTGCCTCCATAGGTCTGATTTC--TATCTTTTTTTTTTTTTTTTTGAGATGGA-
SARS-CoV-2 Reference Genome Shuffle No.1             (9716)  AAAAGGGGGCTTAGTTTATTCTGCCTTCTTAGCGAGAAAACGAGTAGGAAATTTTATTAATTTAGAACATACAT
```

Section 137

```
                                                (10065)  10065 10070    10080     10090     10100     10110     10120          10138
Homo sapiens chromosome 1 NC_000001.11: 11783698-... (9478)  --GTCTTGCTCTG----TTGCCCAGGCTGGAGTGCA---------GTGGCATGATCTCA---GCTCACTGCAACC
SARS-CoV-2 Reference Genome Shuffle No.1             (9790)  TTGACTACCTTTACTAATTCAGAATACTCGACTGTTACTTTTACGTACCAAAATATTATGGCCGCACTGGAGCA
```

Section 138

```
                                                (10139)  10139     10150     10160     10170     10180     10190     10200    10212
Homo sapiens chromosome 1 NC_000001.11: 11783698-... (9535)  TCCGTCTCCCGGGTTCAAGCGATTCTCCTGCCTCAGCCTCCCAAGTAGCTGCAACT--ACAGGTGCCCGCCACC
SARS-CoV-2 Reference Genome Shuffle No.1             (9864)  GTCGTTTTGGGTAAGATGTAGAATTAAATGATTCAA--TACCT--TTGCTCCACCTTTGCAGGGTTCGGCCAAA
```

Section 139

```
                                                (10213)  10213 10220     10230     10240     10250     10260     10270          10286
Homo sapiens chromosome 1 NC_000001.11: 11783698-... (9607)  -ACACCCGGCTAATTGTTGTATT-TTTAGTAGAGATGGGGTTTCATCATGTT----TGTCAGGCTGGTCTCGAA
SARS-CoV-2 Reference Genome Shuffle No.1             (9934)  GATGTTCGTTAAAATTTCGGGTTCTTTCCTGGAAACATAATTTTAATAAGTAGACATGTCAAACTTTGCGATAG
```

Section 140

```
                                                (10287)  10287       10300     10310     10320     10330     10340     10350 10360
Homo sapiens chromosome 1 NC_000001.11: 11783698-... (9675)  CTCCTGACCTTGTGAT--CTGCCCACCTTGGCCTCCTAAAGTGCTGGGATTACAGGCATGAGCCACCAAGCCCA
SARS-CoV-2 Reference Genome Shuffle No.1            (10008)  GGCCTTAACTTTTAAAAGATTACAAGCGGAAGGCAGTTGCCATTTTGTAGAGTAAGCATAAGAAAACAAGACCA
```

```
                                                                                Section 141
                                                      (10361) 10361    10370     10380     10390     10400     10410     10420     10434
Homo sapiens chromosome 1 NC_000001.11: 11783698-...   (9747) GC---CAATCTTTTTTTTTTTTTTTTTTTTTTTTTTTTGAGACAGGGTCTCACTTTGTGGGCCAGGC-TGGAGT
SARS-CoV-2 Reference Genome Shuffle No.1              (10082) GAGCGCTATAACTGATATTGCGGTGTGCTTTCTCAAATGCATCAGTGTTGCACATCATAAGAGAGATATTAAAC
                                                                                Section 142
                                                      (10435) 10435 10440     10450     10460     10470     10480     10490             10508
Homo sapiens chromosome 1 NC_000001.11: 11783698-...   (9817) GCAGAGGCACAAATATG-GCTTACTGGAGGCTCAACCTCTCGGACTCAAGCGATCCTCTCACCCCAGCCCCCTA
SARS-CoV-2 Reference Genome Shuffle No.1              (10156) GTGAACACTCTACTAGCCGAGTTCAGGAG-CGAACCATGGGGGAATGTATTAACTC-CTTATTGGGGTTAATGT
                                                                                Section 143
                                                      (10509) 10509      10520     10530     10540     10550     10560     10570     10582
Homo sapiens chromosome 1 NC_000001.11: 11783698-...   (9890) AGTACCTGGGACTACAGGTGTACACCACCATGCCTCGGTCATTTTTGTATTTT-------TTGTAGAGAATGGG
SARS-CoV-2 Reference Genome Shuffle No.1              (10228) TTTATATATGTCTACAGCCATACATGTTAGTGCCATTGTGCGTATTCCATATTCCCAACATTGTCGT-AATGGA
                                                                                Section 144
                                                      (10583) 10583   10590     10600     10610     10620     10630     10640           10656
Homo sapiens chromosome 1 NC_000001.11: 11783698-...   (9957) TTTCACCATGTTGCCCAGGCTGGTCTCGAACTCCTGGG---CTCAAGTGATCCACCCGCCTTGGCCTCCCAA--
SARS-CoV-2 Reference Genome Shuffle No.1              (10301) TCTCGCCCTA--GACTACGCCCGCATTAAACCCATATAAAACATATTTTATTATTTGTTTCAAACGTACCCTTT
                                                                                Section 145
                                                      (10657) 10657         10670     10680     10690     10700     10710     10720  10730
Homo sapiens chromosome 1 NC_000001.11: 11783698-...  (10026) AGTGCTGGCCGGGTTCCGTGCCCAGCCAGGTCTATCTGACTTAAAGCATGAGCTCCCATCCGCTCCAATCCATG
SARS-CoV-2 Reference Genome Shuffle No.1              (10373) AGTGATTAGCGGGATT--TAGTTGGTTATGTCAAACTAGTAAGAAGTTAGTACCCAAATTCCCTAGGCATGTCG
                                                                                Section 146
                                                      (10731) 10731    10740     10750     10760     10770     10780     10790     10804
Homo sapiens chromosome 1 NC_000001.11: 11783698-...  (10100) CTCATTCAAAATTTAAGAGCCCAG-GAGAGGCAGGCTGCTC--TGCCCT---AAGCTCTGGGGAACCCA----C
SARS-CoV-2 Reference Genome Shuffle No.1             (10445) CTATCTACAAAGCGATTTGGTTATAGATTGTATCGTTTCTCGATGCGGTCTGAAATTATTCTAAAACTTTGTTC
                                                                                Section 147
                                                      (10805) 10805 10810     10820     10830     10840     10850     10860             10878
Homo sapiens chromosome 1 NC_000001.11: 11783698-...  (10164) GGGTGCCGGTCAAGAGAGGGGGG--CACCCCGTGCCTCCCTACCAC--ACCTTCTGGAAGACATAGCCCC-CGC
SARS-CoV-2 Reference Genome Shuffle No.1             (10519) GTGTAAAAATTAAAATTGGTGGATCAAATTAGTAGCAACGTAAAACGGAATTTAATAATTATTTAGGTTTGCAC
```

```
                                                                Section 148
                                                         (10879) 10879      10890     10900     10910     10920     10930     10940       10952
Homo sapiens chromosome 1 NC_000001.11: 11783698-... (10233) TGGGGCCCCAGCCCACGATGGGGTCGGAGGACGGCTTCCCGTTGATGTTGGGCTGTGAGTTGATGGTG-AGGAT
SARS-CoV-2 Reference Genome Shuffle No.1 (10593) TGTAATCTTTGTCCGTTAAGATATAT-ACGACAG-TGATCGGAATCGGTGATGAGGGCGTTGAGGATGGATTAT
                                                                Section 149
                                                         (10953) 10953  10960     10970     10980     10990     11000     11010           11026
Homo sapiens chromosome 1 NC_000001.11: 11783698-... (10306) GCCCTGGCGGTTCACCCGCAGCAGCTCCTCCTTCAGCAGGCTGGTCTCAGCCGCCAGGGGCTCAT-CGTTCCAG
SARS-CoV-2 Reference Genome Shuffle No.1 (10665) GGGAT--CATTTCAAGAGAAGCAGGTATACGCT-AGATGGATGATGGAGCACGACAGAATGTAGTACTTTCTAC
                                                                Section 150
                                                         (11027) 11027         11040     11050     11060     11070     11080     11090  11100
Homo sapiens chromosome 1 NC_000001.11: 11783698-... (10379) --GGCAGGCAAGTCACCTGGGAGAGACGGTGAGCTGG---------CTGGGGCGACCATCAGGTTTGGCACCCT
SARS-CoV-2 Reference Genome Shuffle No.1 (10736) TATGCACCTAAATTCCAAGTTCGTATAGATGACCTGCAAATACCATCTTAGACGACCGTTGTTCAAGTGAACGT
                                                                Section 151
                                                         (11101) 11101    11110     11120     11130     11140     11150     11160        11174
Homo sapiens chromosome 1 NC_000001.11: 11783698-... (10442) GAGTCCCTCTCACGGCCCC--CAACAAAGACCCAGCCTGTCTTTGCCTCCCTAAGCCCTTCCAGGTGGAG-GTC
SARS-CoV-2 Reference Genome Shuffle No.1 (10810) GGTTGGTTTATACTGAATGTGCTAAGATATGCAAGATCGTCGTTTCGTCT-TAAGACGATCTTGATTAACTGTC
                                                                Section 152
                                                         (11175) 11175 11180     11190     11200     11210     11220     11230           11248
Homo sapiens chromosome 1 NC_000001.11: 11783698-... (10513) TCCCA-----ACT---TACCCTTC----TCCCTTTGCCA-TG---TCCACAGCATGGAGGGGAGGGCACAGGAT
SARS-CoV-2 Reference Genome Shuffle No.1 (10883) TTGCGTATAAAATATTTAAACTTTAATTTTATTATACTAATGGCTTTCAAACGATGTATTGATTTGGTTACAGT
                                                                Section 153
                                                         (11249) 11249      11260     11270     11280     11290     11300     11310       11322
Homo sapiens chromosome 1 NC_000001.11: 11783698-... (10571) GGGGAAGTCACAGCCC---CGCAGCCTGGCCTGCAGCTGGGGTCAGGCCAGGGGCAGGGGATGAACCAGGGTCC
SARS-CoV-2 Reference Genome Shuffle No.1 (10957) TGGTATCAAACTGCTATTATTCGGTGTTAATTGCGGATCGCTTCAATATCGA---AGAGAGTTATATTGTGTAA
                                                                Section 154
                                                         (11323) 11323  11330     11340     11350     11360     11370     11380           11396
Homo sapiens chromosome 1 NC_000001.11: 11783698-... (10642) CCACTCCAGCATCACTCA---CTTTGTGACCATTC-CGGTTTGGTTCTCCC-GAGAGGTAAAGAACGAAGA--C
SARS-CoV-2 Reference Genome Shuffle No.1 (11028) GC-CACAAGCACTACTTGTGCGTATGCGTCAGTGCACGCGTTATCGCTACCAGCTATATAGCATATGAATTATC
```

Section 155

		(11397) 11397 11410 11420 11430 11440 11450 11460 11470
Homo sapiens chromosome 1 NC_000001.11: 11783698-...	(10709)	TTCAAAGACACTTTCTTCACTGGTCAGCTCCTCCCCCCACATCTTCAGCAGCTCCTCCTTGGGGACTTGCTCT
SARS-CoV-2 Reference Genome Shuffle No.1	(11101)	TACATGGACACTTGTGTC--TGG---GATCGATGACGCTAGTC----GAAGGAGTTGACTGCAGGCCCATATTT

Section 156

		(11471) 11471 11480 11490 11500 11510 11520 11530 11544
Homo sapiens chromosome 1 NC_000001.11: 11783698-...	(10783)	TCAGGTAGAA--GAGGTAGTAGTCCTTCAGCTCCCCAAAGGCAGGGGAAGAGGAATTGCCCCTGGCAGAGGGGT
SARS-CoV-2 Reference Genome Shuffle No.1	(11166)	ATCTGTAGAACTGAGGTTCTGTGCACAGTCCTCGTCTTAGCAAAAGAGGCATGATATAACCGTGACACAAACAT

Section 157

		(11545) 11545 11550 11560 11570 11580 11590 11600 11618
Homo sapiens chromosome 1 NC_000001.11: 11783698-...	(10855)	GCCCAGAGGTC-AGGGCAC--ACTCCTGACAGAGGGCAGTGCCACCACATGCCCAGGAGGCCATTCCTGTAAAT
SARS-CoV-2 Reference Genome Shuffle No.1	(11240)	ATAAAAATTACGAAGGCACCGAGTCCAGATACTAGATATGGTGAAAAATTAATTATGTCGAC--TC------AT

Section 158

		(11619) 11619 11630 11640 11650 11660 11670 11680 11692
Homo sapiens chromosome 1 NC_000001.11: 11783698-...	(10926)	TCTGCCCCTGACTCCTCCCAGGTCAACCACAAGCATGCAAACTTCTTCTGCCCTCCCGCTCCCAAGAACAAAGA
SARS-CoV-2 Reference Genome Shuffle No.1	(11306)	TATGTCCCCGATTTTAGTCCGG-CATGTGAAGACATGGTAAGGATGTTTTAAATATGATGTCAACGTCTCTAAA

Section 159

		(11693) 11693 11700 11710 11720 11730 11740 11750 11766
Homo sapiens chromosome 1 NC_000001.11: 11783698-...	(11000)	TGTATTTGCAAGGAAGGTCTGCAGGCCCTCACCAGCGGCCGTT--AGGGAAC-TCGTCCCACTCCTGGGTA--C
SARS-CoV-2 Reference Genome Shuffle No.1	(11379)	TGTTTATATGATTAATGTTTAGAA----TTATAATCTGTCATTCCACAAAACATTTTCTAAAAATATGAAACTC

Section 160

		(11767) 11767 11780 11790 11800 11810 11820 11830 11840
Homo sapiens chromosome 1 NC_000001.11: 11783698-...	(11069)	GGTAGATGTAACT-CTTTGGT---CTGGAGGCCCAGAAGATGGGACGTACATCTTCC-TCTCGGCGCTTGGGGT
SARS-CoV-2 Reference Genome Shuffle No.1	(11449)	GTAATTTACAACAACTTTAGTAATCAGCAGTATGTTTACAATTGTCGTCAATGTTTAATATTTGTTATATGCGG

Section 161

		(11841) 11841 11850 11860 11870 11880 11890 11900 11914
Homo sapiens chromosome 1 NC_000001.11: 11783698-...	(11138)	GGGCGCTGAGAGCCCAGGGTAGGGGACGCC-TGGGTGAGGATGGGGACAGAGAATTGAGACAAGGGATTGGCTA
SARS-CoV-2 Reference Genome Shuffle No.1	(11523)	ACCCGTTGCAA-CCTTGTGTCTTCGTGGTAATTGCGGTGGGTTTAAAATCAAAACTAATAAATCGGTTCG--TT

Section 162

```
                                                           11915 11920     11930     11940     11950     11960     11970             11988
                                                   (11915)
Homo sapiens chromosome 1 NC_000001.11: 11783698-... (11211) GAGGGAGCCGGAA-GCAGGGGAGCACCC-----CTGAGGCTGAGTTCTTCCA--TCTGATGCCCTCTGAGACTG
SARS-CoV-2 Reference Genome Shuffle No.1             (11594) CATGTACCTTGATTGCTTGACCGTAAGCGCTTTCTCTGTCCGTGTCTTGCCAGCTCTTATTACCTTTTCTACGC
```

Section 163

```
                                                           11989      12000     12010     12020     12030     12040     12050       12062
                                                   (11989)
Homo sapiens chromosome 1 NC_000001.11: 11783698-... (11277) GAGCCTTCGATGTGCCC---AGCTAGCCATGGCACAGACATCCAT---CT----GTACCACCG-TGGGGAGCCA
SARS-CoV-2 Reference Genome Shuffle No.1             (11668) GAAAATGCGCTCCTCCCGAAACCACGTTGTTTCATCGACATACTTTAACTTAACGCACCAACTCTTGGTATTGA
```

Section 164

```
                                                           12063  12070     12080     12090     12100     12110     12120           12136
                                                   (12063)
Homo sapiens chromosome 1 NC_000001.11: 11783698-... (11340) GCACTGCAGCCGGGGCTGCTCTTGGACCCTCCT-CTGGGGATCTCTGG--GCCACTGCCCTCACCTGGGGTCCT
SARS-CoV-2 Reference Genome Shuffle No.1             (11742) GCACTACACTTATAGCTGTTAATCAACCATAATGCTTGAGACC-CTGGTAGGTTGTGCCGTAA----GAACCGA
```

Section 165

```
                                                           12137        12150     12160     12170     12180     12190     12200   12210
                                                   (12137)
Homo sapiens chromosome 1 NC_000001.11: 11783698-... (11411) CAGTCCACATCCCCAGGCGCTT---CAGCACCTCTGTGGTAGCCATCTCGCGGTTGAGGGTGTAGAAGTGGAGG
SARS-CoV-2 Reference Genome Shuffle No.1             (11811) CAACACTTTTAGCGAAATGTTTCGCCAGCAAGTCTCTGG-AGCTTATTA-----TATGAGTGAAATCATAGTAC
```

Section 166

```
                                                           12211    12220     12230     12240     12250     12260     12270         12284
                                                   (12211)
Homo sapiens chromosome 1 NC_000001.11: 11783698-... (11482) CCTGGCACCAAGCCACTGGCCAGAAGCTCCTGGCACAGGCTCACGGCCAGCTCGATGCCATAGTTGCGGATGGC
SARS-CoV-2 Reference Genome Shuffle No.1             (11879) CCT---ACTCTGTCCTTGGTTCGGTTGAGTTAGCAAACCCCTTAG---AGGTAGTTACCTGAACTAAG-AT--C
```

Section 167

```
                                                           12285 12290     12300     12310     12320     12330     12340             12358
                                                   (12285)
Homo sapiens chromosome 1 NC_000001.11: 11783698-... (11556) AGCATC-GTTGTCTTTGATTGG--CTCAATCACGTCCTTGATCTCCTG-TGGCACCTCCAGCTTGGACAGCTTC
SARS-CoV-2 Reference Genome Shuffle No.1             (11944) ATCATTAGTTCTCTTTTATAGCGACTTATTTATATA---GAACTTCTGCTTGCTGAAACTAATAGGATACATTT
```

Section 168

```
                                                           12359      12370     12380     12390     12400     12410     12420       12432
                                                   (12359)
Homo sapiens chromosome 1 NC_000001.11: 11783698-... (11626) ACAAGCTGCCGAAGGGAGTGGTAGCCCTGTCCAAGACATGAGGGGCTGTGGCGGCTGCTGGCCAGCCCGTCTCC
SARS-CoV-2 Reference Genome Shuffle No.1             (12015) ATTATATCCTTCTGATAGCTGT---CTTGGACTGTAACTGAACGAATACGAAATCA--TTGACATACCA-CTCT
```

Section 169

(12433) 12433 12440 12450 12460 12470 12480 12490 12506
Homo sapiens chromosome 1 NC_000001.11: 11783698-... (11700) CACCCTAAATAC-TTCTCCC-CCTTCCATCATCCATGCCAACCACAGTGTCAGGCAGAGGGACCCACGAA----
SARS-CoV-2 Reference Genome Shuffle No.1 (12083) TACTAAAAATAAATGCTAAAGCATATCATCA-CCTTGCAATTTCTTCTATCAA-CACATAAACGTAATAATACT

Section 170

(12507) 12507 12520 12530 12540 12550 12560 12570 12580
Homo sapiens chromosome 1 NC_000001.11: 11783698-... (11768) AGTCCGATCATATTCTAGGAGCACACCTGGGCTTGGCCTGCCG-TTTTCTCCTCTTTTTTCTGTTTTTAGA-GA
SARS-CoV-2 Reference Genome Shuffle No.1 (12155) AGTTCTATGAAGCTTAACAATTACGAACATACAAAAAATACATCTTATGTACTGGAGACTATAAGTTTAAACGA

Section 171

(12581) 12581 12590 12600 12610 12620 12630 12640 12654
Homo sapiens chromosome 1 NC_000001.11: 11783698-... (11840) CTGAGTCTCACTGCTCTCAAACGCCAGGGCTCAAGCCATCCTCCTGCCTCAG-CTTCT-CAAGTAGCCGGGACT
SARS-CoV-2 Reference Genome Shuffle No.1 (12229) TTTAGTTGTTGCAATATCGCAATCA--GGCTCATACCTGCTTTATCGATCAGACTTTTACATGTATATTGTTTT

Section 172

(12655) 12655 12660 12670 12680 12690 12700 12710 12728
Homo sapiens chromosome 1 NC_000001.11: 11783698-... (11912) ACAGG-CAC---GTGCCACTATGCCCAGCTTTCCCACTTTTTAC-AGAGTAGAGCAGC-TGC-----CCTAAGC
SARS-CoV-2 Reference Genome Shuffle No.1 (12301) GAAGTTCACCTAGTACCTCAATTT--AGTTTATTTACAATTGACGAGATTAGCGCATTATGTATATACGTCAGC

Section 173

(12729) 12729 12740 12750 12760 12770 12780 12790 12802
Homo sapiens chromosome 1 NC_000001.11: 11783698-... (11975) ATTCCAGCCTT-----GGGTTGAGCCAGCCTTT--TAGCCCTAGATGTGTCTGTTGGTAACATGGTGTCATCTA
SARS-CoV-2 Reference Genome Shuffle No.1 (12373) -TTCCAAAATCCAAAACAGTTCACAAAGTCTTTCGTACGGCGCGCATTAATTATTTTCAGCTATATTTATTCAA

Section 174

(12803) 12803 12810 12820 12830 12840 12850 12860 12876
Homo sapiens chromosome 1 NC_000001.11: 11783698-... (12042) ACCCAGCTGTTCCCATGCTGCACTGATCATCCGAATCACCTGCCAAGCTCAAGAAGTAAAACACACATTCCGAG
SARS-CoV-2 Reference Genome Shuffle No.1 (12446) TTCAGACTTTTCTATCTTTGCAAAAATC-TACGAATATTCTTGTAA-CTATTCTTATATTTGATGCAGTATGTT

Section 175

(12877) 12877 12890 12900 12910 12920 12930 12940 12950
Homo sapiens chromosome 1 NC_000001.11: 11783698-... (12116) GACCTCCCTCAGATTTACTGAGTGAGAACCTCCAGGGGTGGACCTGGAGATCCACTTTTTGAAAAAGCTTATC
SARS-CoV-2 Reference Genome Shuffle No.1 (12518) GCCCAGATTGAGTAATCTAAAGTGTTCAGTTACTAAACATATTAATAGAAACCCACGGCTTAAACGTGGTTGCG

Section 176

```
                                                (12951) 12951   12960     12970     12980     12990     13000     13010        13024
Homo sapiens chromosome 1 NC_000001.11: 11783698-... (12190) A-AGTGGTTCTGATGA--CAGCCACCTTTGGGAAACACTGATTTAAGCAGGATTTGTAATGAGAATTAGAATCC
SARS-CoV-2 Reference Genome Shuffle No.1 (12592) GGAACGACCATGGTTATGCAGTCAC---TGGTACCTTTTACTTTGCGTAGAATTCC----GAGCATTGGC-TAA
```

Section 177

```
                                                (13025) 13025 13030     13040     13050     13060     13070     13080          13098
Homo sapiens chromosome 1 NC_000001.11: 11783698-... (12261) CTTTTGGTGATGCTTGTTGGCCAAAGTACAACAAACCCCTCAACAGACACTGTTGCTGGGTTTTGGGGGAAAA
SARS-CoV-2 Reference Genome Shuffle No.1 (12658) ATTATTATATTGCAGAATAG----AGTGCGA-AAAGGTCAGTGGAGACACATTTACATG--TATAGGGGAACCT
```

Section 178

```
                                                (13099) 13099       13110     13120     13130     13140     13150     13160    13172
Homo sapiens chromosome 1 NC_000001.11: 11783698-... (12335) TTAGAGGTAACCAAAATGGGGTGGCCAAGCAACGC-TGTGCAAGTTCTGGACCTG-AGAGGAGATCTGGGAAGA
SARS-CoV-2 Reference Genome Shuffle No.1 (12725) TTAGATGTTTACTTAATATTCTGTAATACTAAAACATGGTCAACAACAGCCGCTGTAGACGGGCTC--------
```

Section 179

```
                                                (13173) 13173  13180     13190     13200     13210     13220     13230          13246
Homo sapiens chromosome 1 NC_000001.11: 11783698-... (12407) ACTCAGCGAACTCAGCACTCCACCCAGAGCCCCCAGCCTGTGCGAGGACGGTGCGGTGAGAGTGGGGT--GGAG
SARS-CoV-2 Reference Genome Shuffle No.1 (12791) ACTAGTCAATCGCTGA--TCCACATTTATTAGGCTTTATATTCGA--ATTTTTTAATAACACCAGGATTCGTCA
```

Section 180

```
                                                (13247) 13247         13260     13270     13280     13290     13300     13310  13320
Homo sapiens chromosome 1 NC_000001.11: 11783698-... (12479) GGAGCTTATGGGCT---CTCCTGGGCCCCTCAC---CTGG-ATGGGAAAGATCCCGGGGACGATGGGGCAAGTG
SARS-CoV-2 Reference Genome Shuffle No.1 (12861) TGAAATCATCTACTTTTGTCATCTGACCTTTAGTTTCTGGTATTCCTAAGTACTTG---ACTATTTTATAAGAG
```

Section 181

```
                                                (13321) 13321   13330     13340     13350     13360     13370     13380        13394
Homo sapiens chromosome 1 NC_000001.11: 11783698-... (12546) ATGCCCATGTCGGTGCATGCCTTCACAAAGCGGAAGAATGTG-TCAGCCTCAAAGAAAAGCTGCGTGATGATGA
SARS-CoV-2 Reference Genome Shuffle No.1 (12932) CGGTTTTTGATTGTGG-TGCAATAA-ATTGCTACAAATTGTGCTTTGACTCCATGGAAGAATCAACGCCAAACG
```

Section 182

```
                                                (13395) 13395 13400     13410     13420     13430     13440     13450          13468
Homo sapiens chromosome 1 NC_000001.11: 11783698-... (12619) AATCGGCTCCCGCAGACACCTTCTCCTTCAAGTGCTTC--AGGTCAGCCTCAAAGCTCCCTG--CTTCGGGGTG
SARS-CoV-2 Reference Genome Shuffle No.1 (13004) TAGCG----CTACTAATTCTTTCTCCAGTTATTGGTCGGAAGTTAACGAAGTAATGTGGGTGTCCTGCTATTTG
```

```
                                                          Section 183
(13469)  13469      13480     13490     13500     13510     13520     13530     13542
Homo sapiens chromosome 1 NC_000001.11: 11783698-... (12689) GCCT-TTGGGGTAACCTGCCAATAGGGATGACAGTCAGGAGAGGCTGGCCTCCACCTGTTCAAGGCGAGGGATG
SARS-CoV-2 Reference Genome Shuffle No.1 (13074) TCATGTTGTAGCTTCGTTACAATCTT-ATAACTTTATGTTATGACCATCCAGAACTTATTGAAAAATAGAACAC

                                                          Section 184
(13543)  13543  13550     13560     13570     13580     13590     13600          13616
Homo sapiens chromosome 1 NC_000001.11: 11783698-... (12762) AAGAGA----CCACAGGGACTGGGCAG-AGAGAGTCCTCTGCTT--TGGGGCTCTGATCTTGCACCTTCCAAC
SARS-CoV-2 Reference Genome Shuffle No.1 (13147) CATAGTTTAGCGATAACCACAGTGCTCTATTGGCCGCGTAGCTCGATAGATGCTTTAGCCTTAGTTTCGTTAAA

                                                          Section 185
(13617)  13617        13630     13640     13650     13660     13670     13680 13690
Homo sapiens chromosome 1 NC_000001.11: 11783698-... (12829) AGCACAGCACAGCCTGAACTAAAACAGTGAGTGGCTGGCTCATGAC---TGATATGCTT-CTGACCCCAGAGA
SARS-CoV-2 Reference Genome Shuffle No.1 (13221) AGCATAGTGCAATTAGTAATTGAGGAGAT-GTAGCTAGACTTGTGACGATTGGTAAGCTTTCCGTTAACA-AGA

                                                          Section 186
(13691)  13691   13700     13710     13720     13730     13740     13750          13764
Homo sapiens chromosome 1 NC_000001.11: 11783698-... (12899) TGAC----TCTCAGGGGAGGTGGCTCAGA----GCCCAGTTCCAAGAGTGGGGGTGGAT-TGCTCCAA----AC
SARS-CoV-2 Reference Genome Shuffle No.1 (13293) TGACGTTATGCCACTCGATCTGGTTGTAATGTTGTGCTGATC-AATTGTTTGAGCGTATGTGTTACAATTCTAT

                                                          Section 187
(13765)  13765 13770     13780     13790     13800     13810     13820              13838
Homo sapiens chromosome 1 NC_000001.11: 11783698-... (12960) TCCCACACTGTC-CTGCCTTGAGCAGCC-AAGCAAA--T--CAGG--CCTGCACTTGGTGGCCATAGAAAAGAG
SARS-CoV-2 Reference Genome Shuffle No.1 (13366) TATTACTATGTGTCCAAGTAACGGATCCGAAGTATAAATTTCTTGATCGTTAACTCGCTACCAATAGCCTCAAA

                                                          Section 188
(13839)  13839      13850     13860     13870     13880     13890     13900     13912
Homo sapiens chromosome 1 NC_000001.11: 11783698-... (13026) AAAATGCTACAGTTAGCACTGGCTGGGCAGCATCTGGGGTAGTAAGCTTGACTTTTCTCTAGGCACCGGATTAA
SARS-CoV-2 Reference Genome Shuffle No.1 (13440) ATCAGGTTCCTTCTC-CGCTG--TTATCTCCTTCTATGGTGCTAGATTATCGTTGTTGCTATGTTGCCTATTAT

                                                          Section 189
(13913)  13913  13920     13930     13940     13950     13960     13970          13986
Homo sapiens chromosome 1 NC_000001.11: 11783698-... (13100) ACATTGTTTTTAAT-GTAACACTGAAATTACTCAGGGGTCCAAGATAGTGAAAAGCTAAAAACCAAGTTACACA
SARS-CoV-2 Reference Genome Shuffle No.1 (13511) TCTGCGGTTTCAGTTGTGTTAAAGGAGTGGCAGAAGC-TCCTTGAGA-TACATAGCTCTATAAGGAGATT-ATA
```

Section 190

```
                                                            (13987) 13987        14000     14010     14020     14030     14040     14050   14060
Homo sapiens chromosome 1 NC_000001.11: 11783698-...        (13173) AGGAAGAACTGAAGAAAGTGATGTTTTCTTCAAAGAAAATAATGGATG-GCAGGGTGCGGTGGCTCA--TGTCT
SARS-CoV-2 Reference Genome Shuffle No.1                    (13582) AGTGTGGCTTCATGGCTTTCATAACCAATGGAGTATTTCAATACGATGCGTCAGTTGGGGTGCCTAAATTGATA
```

Section 191

```
                                                            (14061) 14061    14070     14080     14090     14100     14110     14120       14134
Homo sapiens chromosome 1 NC_000001.11: 11783698-...        (13244) GTAATCCCAGCACTTTGGGAGGCCAAGGCAGGAGGATGACCTGAGGTCAGGAGTTCGAGACCAGCCTG---GCC
SARS-CoV-2 Reference Genome Shuffle No.1                    (13656) ACAATTCCGGCTGACTACCAGTTCATAATATGAAGGGGGCGCGATAAAACATGATAAGTAACACACTTTTTTCG
```

Section 192

```
                                                            (14135) 14135 14140     14150     14160     14170     14180     14190        14208
Homo sapiens chromosome 1 NC_000001.11: 11783698-...        (13315) AACATGGTAAAACC--CCGTCTC----TACCAAAAATA--CAAAAATTAGCCAGGTG-TGGTGGTGTATGCCTG
SARS-CoV-2 Reference Genome Shuffle No.1                    (13730) ATGAGGCTAAAAACATCAGCTTAATGTTACGACAAGGAGTCAAAAGTACTATAAACGATATAAGTGTGTGGCGC
```

Section 193

```
                                                            (14209) 14209      14220     14230     14240     14250     14260     14270      14282
Homo sapiens chromosome 1 NC_000001.11: 11783698-...        (13380) TA-ATAGCTACTCAGGAGGCTGAGTCAGGAAAATTG-CTTGAACCCA--CGAGACAGAGGTT-GCAGTGAGCCG
SARS-CoV-2 Reference Genome Shuffle No.1                    (13804) TACATAGTTACGAATGTGTAACTATTACACTTATTCACCTCATTCCAATCGTGCAAGTCTTAAGCAGCATGAAT
```

Section 194

```
                                                            (14283) 14283  14290     14300     14310     14320     14330     14340        14356
Homo sapiens chromosome 1 NC_000001.11: 11783698-...        (13449) AGATCGTACCATTGCACTCTAGC------CTGGGCAACGAGAGCGAAACTCCGTCTCAAAAAAAAAAAAAAAAA
SARS-CoV-2 Reference Genome Shuffle No.1                    (13878) AATTTTTAT-ATTGATCACTTGCTGAGTTCTTAGCTTCGACATGTA--CTCTGCTGCAGAAATCCTAGTCGTTA
```

Section 195

```
                                                            (14357) 14357        14370     14380     14390     14400     14410     14420   14430
Homo sapiens chromosome 1 NC_000001.11: 11783698-...        (13517) AAAGAATGAAAAGAATACAGAAACTCCTTTCAAATGTCCAAAGGTCTGTCCTGGGAAAAAGGATCCACCATAG
SARS-CoV-2 Reference Genome Shuffle No.1                    (13949) AATGCGTTCTATTCG-ACAGAAACAGATTTATAGATTTTTGACTACT-TGCTGCT-AACTCGAATATTCGTTTT
```

Section 196

```
                                                            (14431) 14431    14440     14450     14460     14470     14480     14490       14504
Homo sapiens chromosome 1 NC_000001.11: 11783698-...        (13591) -GTGAGTTTGTTTTGTTTTGTTCAGAGACAGGGTCTTGCTCTGTCACCCAGGCTAGAGTGCAGTGATACA-AT
SARS-CoV-2 Reference Genome Shuffle No.1                    (14020) TGCTACTCTGGCCTGCTTTGGTTTTCTAAC---GTCAT------TTATAGAGCTTAGTGGGAA-TGAAATAGAT
```

Section 197

```
                                                        (14505) 14505 14510      14520      14530      14540      14550      14560           14578
Homo sapiens chromosome 1 NC_000001.11: 11783698-... (13663) CATGGCTCA-CTGTAGCCTTGAACTCCC--GGGCACAAGTGATCTCCCACTTCA--GTCTCCTGAGTAGCTTGG
  SARS-CoV-2 Reference Genome Shuffle No.1 (14084) CGTTATTCTTCTGAAGTCGCTTACTCCCATGGGCTCGATACATGACCATATTCAAGGTCTCAT-ATTAGATAAC
```

Section 198

```
                                                        (14579) 14579      14590      14600      14610      14620      14630      14640      14652
Homo sapiens chromosome 1 NC_000001.11: 11783698-... (13732) ACTACAGATGCAAGCCACCACATCTG-GCTAATTAAAAAAAATTTTTTTAATTAAT-TATT----TATTTTTTA
  SARS-CoV-2 Reference Genome Shuffle No.1 (14157) -CTAGA-ATCAATGTAATGGCTTCAAAGCTAAATGTAGAGATGGTCCTCGAACATTGTACTCTGGTTCTTGTAC
```

Section 199

```
                                                        (14653) 14653  14660      14670      14680      14690      14700      14710         14726
Homo sapiens chromosome 1 NC_000001.11: 11783698-... (13800) GAGATGG-GGTCTTGCTATCTTGTCCAGGTTTGTCTGGAACTTCTGGGCTCAAGCAATCCTCCCACC-TTGGCC
  SARS-CoV-2 Reference Genome Shuffle No.1 (14229) GAAATGACGGCCTTACTGCATTTGTCACTTTTAAAAAGAGGTCAAGAG-TCAAGCCTAACGATTACCATTGTTC
```

Section 200

```
                                                        (14727) 14727        14740      14750      14760      14770      14780      14790 14800
Homo sapiens chromosome 1 NC_000001.11: 11783698-... (13872) TCCCAAAGTGCTGAGATTACAGGCATGAGCCACTGTACCCAGCCCCCTAGTGAAGTTCTTTATTAGTCATATTC
  SARS-CoV-2 Reference Genome Shuffle No.1 (14302) GCGCTTATGG--GAACGTACAGA-ATTTCCTATAATTTAGCGTTTTTCAAGGATAAAAACGCCTTGGAATACCC
```

Section 201

```
                                                        (14801) 14801   14810      14820      14830      14840      14850      14860         14874
Homo sapiens chromosome 1 NC_000001.11: 11783698-... (13946) TGTGTTGTTTCTAATGGCAGAACCAGCATCCGGTTCAGA---GAGCTGGCTAGAGTACT------ATAAGCCAC
  SARS-CoV-2 Reference Genome Shuffle No.1 (14373) ATTCTGCATTCTCGACTCACTACTTCTCGTCTTTTTAGATTTGAACATGCTGAATTAGTTCCAGTATTAGGCAG
```

Section 202

```
                                                        (14875) 14875 14880      14890      14900      14910      14920      14930           14948
Homo sapiens chromosome 1 NC_000001.11: 11783698-... (14011) TCACAAAACAATGAGCTCCTGGAGTCTGAAAACACAGGGGCAGTGGATGCTGAAGA-CAAGATTCCAGAATTGC
  SARS-CoV-2 Reference Genome Shuffle No.1 (14447) CCTTTAAGGATTACTCTAATTTATTACGAAGTTATCCGTCTATAATAAGCTTAACTGCTAATTTCCGGTACAGC
```

Section 203

```
                                                        (14949) 14949      14960      14970      14980      14990      15000      15010      15022
Homo sapiens chromosome 1 NC_000001.11: 11783698-... (14084) TTAGGAGGTGGGATCAGATGTCCCATAGGCTCCTTACTGGCTCTGTGAACTCTCTGACCCCTTTCCTCAATGAC
  SARS-CoV-2 Reference Genome Shuffle No.1 (14521) TTCGCT------ATATGACGTCCTGACCCAGGTATCATGCCCCTTAGTA-TCTAGAAGTCCAATGACTAATTA-
```

Section 204

(15023) 15023 15030 15040 15050 15060 15070 15080 15096
Homo sapiens chromosome 1 NC_000001.11: 11783698-... (14158) TAAACTTACTAGCCGCCTTGCTCGGTCAGGCAAGCAGGA-TGACCATAGGCCTTCATGAAACTACATGGAGTGC
SARS-CoV-2 Reference Genome Shuffle No.1 (14587) TAGGCTGTTTAGCC-CATAAAACAGACATAAGAGCATTCGTTACAACTAAATCTCATAATAGCATA---ATAGT

Section 205

(15097) 15097 15110 15120 15130 15140 15150 15160 15170
Homo sapiens chromosome 1 NC_000001.11: 11783698-... (14231) TGGTTTCATCAGCCCAAGCCCAGGGCAGGACTCTGAGCCAGGCGGAAGGCCAAGCTGCTTTGCTCTGATAAGGC
SARS-CoV-2 Reference Genome Shuffle No.1 (14657) TGGTGAC-TCTGCTCTTACATATGGTATTAGGAGGAAAAACACTCAAAGCGATATAA--TTGATTTGAAGATGT

Section 206

(15171) 15171 15180 15190 15200 15210 15220 15230 15244
Homo sapiens chromosome 1 NC_000001.11: 11783698-... (14305) AGTTCCGTTCTTAAGAAACCCAGAGTCATCAAAAAATGAATATAAAAACTATTGTTTCAAGGGGTAAGGGAGTG
SARS-CoV-2 Reference Genome Shuffle No.1 (14728) GGTTAATTT-TGATCCGACCGAATGTATTTTA----TGCATTTCCATAGTA--GTTGAAATTATGACCTCTGTG

Section 207

(15245) 15245 15250 15260 15270 15280 15290 15300 15318
Homo sapiens chromosome 1 NC_000001.11: 11783698-... (14379) GGGGATAAAGCAATGGGGCTTCACACTTGCAAT----AACAAAGCTA----TTTTCCCAGTAGGAATTTCAGAA
SARS-CoV-2 Reference Genome Shuffle No.1 (14795) TCGGTTACAAAACGAATGCTTAACTGGTCCTATTCCGACCAATCCGACGTCTTACGCCTGCGGTTTGTTCACAA

Section 208

(15319) 15319 15330 15340 15350 15360 15370 15380 15392
Homo sapiens chromosome 1 NC_000001.11: 11783698-... (14445) ACAAC-AAGGCTTTTTAATAGCTGAAAAGTATAGCTACAATACCTAAATATCACCGAACAAATCTAGCATTTGA
SARS-CoV-2 Reference Genome Shuffle No.1 (14869) ATTTTGAATACCTCATGCTTG--GATTATTACAACTA-ATTACGATATTGTGTTGGGAGGACAGGAGC--TTGG

Section 209

(15393) 15393 15400 15410 15420 15430 15440 15450 15466
Homo sapiens chromosome 1 NC_000001.11: 11783698-... (14518) TTACGTATAGATTTTGCTATTTAC-TCTATTTTTCTATCATGAACAGCTGAGGGCTTTCTTTTTTAAAATGTTT
SARS-CoV-2 Reference Genome Shuffle No.1 (14938) AGGCAAATGTATTCTTCGATGTACATATGAGTTAGTGTACTTACCAG-TACTGTCTTATGCGTGTACAGTAGGA

Section 210

(15467) 15467 15480 15490 15500 15510 15520 15530 15540
Homo sapiens chromosome 1 NC_000001.11: 11783698-... (14591) TTATTTTTTGTAGAGACACGGTCTCACTGTGTTGCCCAGGCTGGTCTTGATCTCCTGCCTCAGTCTCCCAAAGC
SARS-CoV-2 Reference Genome Shuffle No.1 (15011) TT--TTATTAGCGAAACAAAGCACATGCAAGATCTTCATATTGTTCTACTACTGCGGAGATGATAGCTAACTAC

```
                                                                  Section 211
                                                   (15541) 15541    15550     15560     15570     15580     15590     15600         15614
Homo sapiens chromosome 1 NC_000001.11: 11783698-... (14665) TCTGGGATTACAGGCATGA-----GCTACCA----TGCCCA-ACAGGTTGGTGTTTTC--TAATCCACTCATCT
SARS-CoV-2 Reference Genome Shuffle No.1 (15083) CATGGGACATTTGGCATGATATTTGCTGGTATATATACCCATACATGTAAGTAGTACGGATATTTCAAGATCCT
                                                                  Section 212
                                                   (15615) 15615 15620    15630     15640     15650     15660     15670             15688
Homo sapiens chromosome 1 NC_000001.11: 11783698-... (14727) TCATTACCCACTTGATAAGCAAAGTAAGAAACCATTCAGCAACTGTGAGCTCACAGCTTCACTAGTTTCCAATC
SARS-CoV-2 Reference Genome Shuffle No.1 (15157) TGATT--CTAATTCATAA-CATTGATAAAAACCAAAGTTTACATTT-AGTTGGGA----CACTATGTTGAACTC
                                                                  Section 213
                                                   (15689) 15689      15700     15710     15720     15730     15740     15750     15762
Homo sapiens chromosome 1 NC_000001.11: 11783698-... (14801) ATTATCACCTAACGCCCATCACGGGTGCATATTCCCCCAGTAACT--GACTATATAATGGCTGATACAAGCGAT
SARS-CoV-2 Reference Genome Shuffle No.1 (15223) CTTGTAAGCATTCGAT-ATTA-----GCATACTAATACATATTCTAGTACGAGAAAAAGGGTAATCAATACGCT
                                                                  Section 214
                                                   (15763) 15763  15770     15780     15790     15800     15810     15820         15836
Homo sapiens chromosome 1 NC_000001.11: 11783698-... (14873) GACAATTTAACAGTTGGAAATGTCTGTTCTCTGACTCCTAATCAGTGCTTCCCAGACTTAGCAC--CTAAACAC
SARS-CoV-2 Reference Genome Shuffle No.1 (15291) AACTGCTGTTAAGCCGTA----TCTTTAATCTT--TCGTATATAG-GCAACC---ACTGAGCACTCCGTATGAC
                                                                  Section 215
                                                   (15837) 15837         15850     15860     15870     15880     15890     15900 15910
Homo sapiens chromosome 1 NC_000001.11: 11783698-... (14945) CCCT-GTTGACAGAATGGACATGTCCATCGGGGGTTAGGGACAAGGCCAAAGTTACTCACAACATGTAATTTTC
SARS-CoV-2 Reference Genome Shuffle No.1 (15355) CCTTTGTAGTACTAATATAAATTAGTAGAAACCCAAAATGCAATGACCCGAGA-ACTCCCCTGTTTATATAATA
                                                                  Section 216
                                                   (15911) 15911    15920     15930     15940     15950     15960     15970         15984
Homo sapiens chromosome 1 NC_000001.11: 11783698-... (15018) TTGAAGCCACGTGGTTTGTTTTTGGGTTTTTTGTTTTGTTTTTTTGAGACGGAGTCTGGC-TCTGTTG-CCC
SARS-CoV-2 Reference Genome Shuffle No.1 (15428) TT-ACGTTTAGTTAAACGTATTC-GTCTTTCAAGGATTGATCTAATCATACCATTGTCTAAAATCAGATGACCC
                                                                  Section 217
                                                   (15985) 15985 15990    16000     16010     16020     16030     16040             16058
Homo sapiens chromosome 1 NC_000001.11: 11783698-... (15090) AGGCTACAGTGCATTGGTGCGATCTCGGCTCACTGCAAGCTCCGCCTC-CTGGGTTCAAGCGATTCTCCTGCCT
SARS-CoV-2 Reference Genome Shuffle No.1 (15500) ATTCCTAGTATCAACGGTACACAGTCATGTTC--GAAAGCTAATCATCTCTTATTTACAGGGATCTGCTCCC-
```

Section 218

```
                                                      (16059) 16059      16070     16080     16090     16100     16110     16120       16132
Homo sapiens chromosome 1 NC_000001.11: 11783698-... (15163) CAGCCTACCAAGTAGCTGGGACTACAGGCACGCGCCACCATGCCCGGCTAATTTTTGTATTTTTA-GTAGAGAC
   SARS-CoV-2 Reference Genome Shuffle No.1 (15571) -AATCTACATAGTTGGT-----TTAAGAC-CAAACCACTA-----GTCTAAAATAGGTCTATATACGTAGATTG
```

Section 219

```
                                                      (16133) 16133 16140     16150     16160     16170     16180     16190          16206
Homo sapiens chromosome 1 NC_000001.11: 11783698-... (15236) GGGGTT-TCACTATGTTGGACAGGATGGTCTCAAACTCCTGATCTCATGATCCACCCGCCTTGGCCTCCCAAAG
   SARS-CoV-2 Reference Genome Shuffle No.1 (15633) AAAGTTGTCTGTACAATTGAGAGG--GATTTCATA----TGATCAATTGAACT------------CTATTAAAG
```

Section 220

```
                                                      (16207) 16207        16220     16230     16240     16250     16260     16270  16280
Homo sapiens chromosome 1 NC_000001.11: 11783698-... (15309) TGCTGGGATTATAGGCTTCAGCCACCGCACCTGGCCTTTTTTTTTAGTTCATATCACTTTAGATGCTACTCCA
   SARS-CoV-2 Reference Genome Shuffle No.1 (15689) AGTAACTAAACTTGTCATCGG--ACTAAAGCCGACGATCGTAAAGTAGCTTTTGCCCCTCCAAAAGATATTCCT
```

Section 221

```
                                                      (16281) 16281   16290     16300     16310     16320     16330     16340        16354
Homo sapiens chromosome 1 NC_000001.11: 11783698-... (15383) T-----AACATA-GCCATGT--TTAATATA-AAAATGGTAGGATTTCTCCCAGAATATTGGCAAAAAAAC-TGT
   SARS-CoV-2 Reference Genome Shuffle No.1 (15761) TGTGTCAACGTACGCCCTGATATTAATACACAGACAGGTGAACAATTTCACGGGCTGTTGTCCCCAAGTCATTT
```

Section 222

```
                                                      (16355) 16355 16360     16370     16380     16390     16400     16410          16428
Homo sapiens chromosome 1 NC_000001.11: 11783698-... (15447) CATCCCAGAAAATGTGCCAA-TCCCGGGACT-TCCTGTCCCTGGTGCACACTGTCTTGCCATACCTGAGCGCTG
   SARS-CoV-2 Reference Genome Shuffle No.1 (15835) TAAAGACGGAAATGAATAATGTAAAATGAATATATAGTACGACCTGGGTTATGAAATTCCTTATTATGGCACCC
```

Section 223

```
                                                      (16429) 16429      16440     16450     16460     16470     16480     16490       16502
Homo sapiens chromosome 1 NC_000001.11: 11783698-... (15519) TCTGAGACGCTGAGGGCTCAATGCCAGCCTTCAGCTTCAAGGGTGGGAGAGAAGTCCAACCCACCCATTTCTGT
   SARS-CoV-2 Reference Genome Shuffle No.1 (15909) TTACAAACACGAACCGCGTGTTCTTATCTAT----TTAGCGGTTACTTTCTAAAATCAACGCCGTC-TAAATGT
```

Section 224

```
                                                      (16503) 16503 16510     16520     16530     16540     16550     16560          16576
Homo sapiens chromosome 1 NC_000001.11: 11783698-... (15593) TGGGGAACCTGGTCTTGTGGCTGTGGCCAATGGTGATTCTCTTTCCTGGGAATTTGGAACGGAGGCCTGTCCA
   SARS-CoV-2 Reference Genome Shuffle No.1 (15978) -GGTGGACGTTGTTTTTCTCTACGTTAACTAATGTT-TTATAATTATTG-----TAGAATACGAG---TTTTTG
```

Section 225

```
                                                          (16577) 16577        16590     16600     16610     16620     16630     16640  16650
Homo sapiens chromosome 1 NC_000001.11: 11783698-... (15667) TTAGCCTGGGGAACTGGCTGGAACTGACACTCTGCACACTCAGGAGCAGGCCAGGTGTGTGGAAAAGCATGGGT
SARS-CoV-2 Reference Genome Shuffle No.1 (16042) TAAAACGTAGTAACTGTAAAGA--TTACGGGCTAGATTTTC---------CTAGATGAATGAAAACTGTTG---
```

Section 226

```
                                                          (16651) 16651    16660     16670     16680     16690     16700     16710      16724
Homo sapiens chromosome 1 NC_000001.11: 11783698-... (15741) ATCAGGTCTCCCAG---ATGAAGCACAGATGTGCAGTGGGAAGCGGAGATGGGGGACCCTGTGGCCCCAGAGGG
SARS-CoV-2 Reference Genome Shuffle No.1 (16102) -TAAGGTATCGAAGTCAACGTCGCATAAAAG-GTTTTACATTACCACTATGGAAAATCATC--GCCCTAGACTT
```

Section 227

```
                                                          (16725) 16725 16730     16740     16750     16760     16770     16780      16798
Homo sapiens chromosome 1 NC_000001.11: 11783698-... (15812) AATGAGGGAGGGCCTTGCTTTTTGACCTCTTCCAGTGCCTTGTGAGGTCTGAGGCCATCTCCTGATCTTGGGTT
SARS-CoV-2 Reference Genome Shuffle No.1 (16172) TATTATA--------TTTTTATTGAGATTTTTCAGATATCCCTTTGCTTATTCAACATGACATGAT--TCAGTT
```

Section 228

```
                                                          (16799) 16799       16810     16820     16830     16840     16850     16860  16872
Homo sapiens chromosome 1 NC_000001.11: 11783698-... (15886) CCCTGCCCTCTGGAATCCTGTTAGTAAATATC-CCTCTTTTGTGGGGT-GTTGTTTTTTTTTTTTTTTTGAGAT
SARS-CoV-2 Reference Genome Shuffle No.1 (16236) GCTATAAATC---ACTACTGATGATACATCTGACGTCTTTTATTACATAGTCATATTTTCGCTTTACTTGCGCC
```

Section 229

```
                                                          (16873) 16873  16880     16890     16900     16910     16920     16930      16946
Homo sapiens chromosome 1 NC_000001.11: 11783698-... (15958) GGAGTCTTGCTCTGCCACTGAGGCTGGACTGCAGCGGCACCATCTCGGCTCACTGCAACCTCCGCCTCTTGGGT
SARS-CoV-2 Reference Genome Shuffle No.1 (16307) GGACACAAGAGGTTGTATTCAAGTCACCGCTCACCTG----ATTTGAAAAATTTACAAAATCAGCCCATTACCT
```

Section 230

```
                                                          (16947) 16947        16960     16970     16980     16990     17000     17010  17020
Homo sapiens chromosome 1 NC_000001.11: 11783698-... (16032) CCAAGAGATTCTCCTGGCTCAGCCTCCTGAG-TAGCTGGGACTACAGCCGTGCACCACCACACCTGG--CTAAT
SARS-CoV-2 Reference Genome Shuffle No.1 (16377) ACGTCACTAGCAC-TGGTGCTTCATCTTCAACTAGTTAAGTATCCGGCCCCGGGTGGCTAGATCGGAAAATAAA
```

Section 231

```
                                                          (17021) 17021    17030     17040     17050     17060     17070     17080      17094
Homo sapiens chromosome 1 NC_000001.11: 11783698-... (16103) TTTTGT-ATTTTTAGTAGAGACG---------GGGTTTCGCTATGTTGGCCAGGCAGGTCTCAAACTCCTGACC
SARS-CoV-2 Reference Genome Shuffle No.1 (16450) CTTTATCAGTTGTAGTAGAGCCTTTACAATATGATATTATTTATCACATCTTCATTCGTCTCGTTCTTGTCAAT
```

Homo sapiens chromosome 1 NC_000001.11; 11783698-11817823 vs. SARS-CoV-2 Shuffle No.1

```
                                                                          Section 232
                                               (17095) 17095 17100           17110           17120           17130           17140           17150                17168
       Homo sapiens chromosome 1 NC_000001.11: 11783698-... (16167) TCAGGTGATCTGCCTGCCTCGGCCTCCCAAAGTGTTGGGGTTACA---GGTGTGAGCCACCGCGCCCAGACCCC
           SARS-CoV-2 Reference Genome Shuffle No.1 (16524) GCTTGTGTTACCCATTTTACTGCAATAGATCTTACTCCGTATCCAAAAGAGGCTGGATACAGTTACCACACCCA
                                                                          Section 233
                                               (17169) 17169      17180           17190           17200           17210           17220           17230        17242
       Homo sapiens chromosome 1 NC_000001.11: 11783698-... (16238) TCTTTCGTTTTAAAGAAAAGAATAAGGCCAAGCAGGCTAAGGGCACAGCCAGGCAGAGGAACAAAAAGCTAGGG
           SARS-CoV-2 Reference Genome Shuffle No.1 (16598) T-TGTCTTGTTTTATAATTTTATCGATGTCTTTAGTAATTTGGAAGATACATGTA----AACTGATTCCAAGGG
                                                                          Section 234
                                               (17243) 17243   17250           17260           17270           17280           17290           17300          17316
       Homo sapiens chromosome 1 NC_000001.11: 11783698-... (16312) GTCGATGGTGGCAGGCCCAGGAGGGGAGTGGGTGATACAAAAGGAGCAAGGACATGGTGCAGTTCACCTCTGCA
           SARS-CoV-2 Reference Genome Shuffle No.1 (16667) GAGGCTACCCAATG----ATAAGCCGACTCGCACTCACTTAAGG-GCGACTAG-TGGTATCGT--AAGTA-GAG
                                                                          Section 235
                                               (17317) 17317        17330           17340           17350           17360           17370           17380   17390
       Homo sapiens chromosome 1 NC_000001.11: 11783698-... (16386) TGTCCTTCATCCAGCATGGACCCATGAGTGCCCCTGATCACTGTGTCCTGAACCGAACACCGACAGGCTCTCTG
           SARS-CoV-2 Reference Genome Shuffle No.1 (16732) TTTGCGTAATAGATTCTGGTTTAATTATTGACGG-GATAA---------GAAACAATTACTGAGGGGGGCACTA
                                                                          Section 236
                                               (17391) 17391     17400           17410           17420           17430           17440           17450         17464
       Homo sapiens chromosome 1 NC_000001.11: 11783698-... (16460) GGCATCTCAGCCTCCCTAGCTCCATCCCCGCCACCAGGATGATCCAGCCACTCACCT-GCCACACAGATGTCAA
           SARS-CoV-2 Reference Genome Shuffle No.1 (16796) GACAAC--------CTTTGCTCATTCTATACAAGGGAAATCACTCCGCTGTCCAAATCGCTACTCCTA-GTCTC
                                                                          Section 237
                                               (17465) 17465 17470           17480           17490           17500           17510           17520                17538
       Homo sapiens chromosome 1 NC_000001.11: 11783698-... (16533) AGTAGTCACCAAACTCACTTCGGATGTGCTTCACCAGGTCCACTGCGTAGTTGAAGCCTCCCTCCTCCTCTTCC
           SARS-CoV-2 Reference Genome Shuffle No.1 (16861) TATGGTAACGTTGGCAAATCTGGGTATAATAATCTTATTAC---GCCTAATGGAAAGCCACTATTAAATGATAC
                                                                          Section 238
                                               (17539) 17539      17550           17560           17570           17580           17590           17600        17612
       Homo sapiens chromosome 1 NC_000001.11: 11783698-... (16607) CACTGGTCACCTATTGGGTCTGCAGGGTTGGTGGAGGTCATAGTCACTGGGTCTCACCCAACCTCCAG--AGAC
           SARS-CoV-2 Reference Genome Shuffle No.1 (16932) AATCTGTGAATTAATATATCGGCCAAAACACCTTAGT--ATAGGCAGCCGGGAAAAAAAAACTTCAACGTAGCC
```

```
                                                                        Section 239
                                                        (17613) 17613  17620     17630     17640     17650     17660     17670          17686
Homo sapiens chromosome 1 NC_000001.11: 11783698-... (16679) CCTTTCTGTTCCCTCTACCTAGCCCCGACACCCAGGACTGCGAGTCCCTGGAAGGCAGAAACTTCATATTATTT
SARS-CoV-2 Reference Genome Shuffle No.1 (17004) CTATTCTGTTCT-TGTTTCTATAACTGTAAGTCAAGG--GCGTAGATTTCGAA--CATAACTATCAC--TAGGT

                                                                        Section 240
                                                        (17687) 17687        17700     17710     17720     17730     17740     17750  17760
Homo sapiens chromosome 1 NC_000001.11: 11783698-... (16753) AGGTTTGGTTCGTCTCTTTCTAGCACTTGG-TAAGACAGCAGGAGGGAAAAAAGCAGCCCACCAGTTCAATATT
SARS-CoV-2 Reference Genome Shuffle No.1 (17071) TGAATTAGTTAACTTCTTTTTACTCCTTATCTATTATATCAGTCTA-AAATACTCTGAGGAA-AGATTATTTGG

                                                                        Section 241
                                                        (17761) 17761    17770     17780     17790     17800     17810     17820       17834
Homo sapiens chromosome 1 NC_000001.11: 11783698-... (16826) CACTGCATGCTGACCCTGTGCTGGCGGTGGAGAATAGGAGACCACCGCTGCTTCTCACCGTGCCTGGAGGATTG
SARS-CoV-2 Reference Genome Shuffle No.1 (17143) ATAAGATCTCTTAGGCGGAACTTAGGAGCGAAACTCAGGAAAGAACGTTGGTAAG-ACTTTGACGCGCGGAACG

                                                                        Section 242
                                                        (17835) 17835 17840     17850     17860     17870     17880     17890          17908
Homo sapiens chromosome 1 NC_000001.11: 11783698-... (16900) GAGAGCCCTTCCCTACTTCTACC-TGGGCATGCCTGCCACCCTCTCCCATCCCACG-----GCCTTGGGGCTCC
SARS-CoV-2 Reference Genome Shuffle No.1 (17216) CAATACTTTGCTTTTAACATAATATGATCCGCAGTAGGAAGTACTGGCGATCTATGTTTGAGCGTTAGTTATCA

                                                                        Section 243
                                                        (17909) 17909      17920     17930     17940     17950     17960     17970    17982
Homo sapiens chromosome 1 NC_000001.11: 11783698-... (16968) TGCCTCCTGGGAGAATTTTCTACTCATCTCAAGTGAAG-----GGGTCTTGTGGTAGGGAG---CACGAGGCCA
SARS-CoV-2 Reference Genome Shuffle No.1 (17290) ATCCCCCTGTTC---TTTACGACTAAAGTTACTTGAAGTTTAAGAATTTAGAATTCTATATTTACCTAAAGCTA

                                                                        Section 244
                                                        (17983) 17983  17990     18000     18010     18020     18030     18040          18056
Homo sapiens chromosome 1 NC_000001.11: 11783698-... (17034) TAGGATCCCACCCATCAGGCTCCCTCTTCCCACTCTTAGGTGGCCTGACAACAGCGGG--CACCAGAGGCCTGA
SARS-CoV-2 Reference Genome Shuffle No.1 (17361) ATGCAGAGTACCCGGAAGTTTATGCGTACCAAATTGCAGCCACCTGTAGAGTAGCTGTGTAACCACAAAAACGA

                                                                        Section 245
                                                        (18057) 18057        18070     18080     18090     18100     18110     18120  18130
Homo sapiens chromosome 1 NC_000001.11: 11783698-... (17106) CCGGCTATTCTCACTCCTGGATACTCTGTTACTCTGTGATTGGGCCAAGT----GCTGGGTGCAGC-CAGCAAT
SARS-CoV-2 Reference Genome Shuffle No.1 (17435) AAAGTGGGCTCCTCAGCAGGCTACAAATAATCGAAGTGTGGTTACCAAGTATATGCTAATTTCATTACAGCTTT
```

Section 246

(18131) 18131 18140 18150 18160 18170 18180 18190 18204

Homo sapiens chromosome 1 NC_000001.11: 11783698-... (17175) CGATGCAGTGGTGTAGGCAAGGCAGAGGGTTATCAGGCCGCGACTGCATTCCTGACGGAA-CTCCTATGTCGAG

SARS-CoV-2 Reference Genome Shuffle No.1 (17509) -GAT-CACCCGTTTCTACGGTGACTCGCTCTACCGAGGCTAGCAACAATAGATTACTTTAGCTCTTGTGTGTTT

Section 247

(18205) 18205 18210 18220 18230 18240 18250 18260 18278

Homo sapiens chromosome 1 NC_000001.11: 11783698-... (17248) GTCAAGAGGTGGAAGAGGTGGCTGCCTGTGTGTGGTGCCCAGGATGACTCACTACCCACAATGCCTACTGACAC

SARS-CoV-2 Reference Genome Shuffle No.1 (17581) CTTAGAACA--GTTTAAGTCGCTGACACTTGAACATGGGTAAT-TGGCGAGATAACTAAAAAACAT-CAGACAC

Section 248

(18279) 18279 18290 18300 18310 18320 18330 18340 18352

Homo sapiens chromosome 1 NC_000001.11: 11783698-... (17322) TACTGGCGGGATGGGATCCTGCGGATCCTCAGCGTGGG--GCCCAGGA-AGAAGTAAAGGACCATTTCAGGAA

SARS-CoV-2 Reference Genome Shuffle No.1 (17651) T--TAAAAGAGATAACAATTGTCGTTTAGATGTAGTTGGAAGCACGCGACAGAAATAGAGATGCATT----GAT

Section 249

(18353) 18353 18360 18370 18380 18390 18400 18410 18426

Homo sapiens chromosome 1 NC_000001.11: 11783698-... (17393) CCAAGTTCAGGCTACCAAGTGGCCTCCGGGAAAGCCAGAACCCAGAGTGTAGGGGAGTGCTGGCTCCACACCTC

SARS-CoV-2 Reference Genome Shuffle No.1 (17719) CG-----CAGGC-ACATAGGAGCGCTAAATAAATCGTGGACTCCGAGTCCACTCAACAATTGGTTTGA-ACTTG

Section 250

(18427) 18427 18440 18450 18460 18470 18480 18490 18500

Homo sapiens chromosome 1 NC_000001.11: 11783698-... (17467) CCCGCAGCGCCATGATGTTCTTCAGGCCCAGCTGCTTAGCTTTGTGCAGATGGCC------CGTGATCT-CCTC

SARS-CoV-2 Reference Genome Shuffle No.1 (17786) TATG-AGAATAATTAGAATCTTTTAACAA---TACTTGACGT-GCGGAGATTGCCTCTTGAGTTGATCTGCCTT

Section 251

(18501) 18501 18510 18520 18530 18540 18550 18560 18574

Homo sapiens chromosome 1 NC_000001.11: 11783698-... (17534) CAGGCGCTGACGGCAGCAGGTCATGTGCAGGATGGTCTCCAGGCCACAGT-AGTTCACGGCGGTG-CTGGCGAT

SARS-CoV-2 Reference Genome Shuffle No.1 (17855) AAACCATAAACTTGTCCAGCATAAAACACTGATATATTTTAATTTACGGTGAGTAAATCGTAGTGTCTATAAGT

Section 252

(18575) 18575 18580 18590 18600 18610 18620 18630 18648

Homo sapiens chromosome 1 NC_000001.11: 11783698-... (17606) CATCATGGAGGAGGTCTCCTT--GTCTGAGCCAGGGTCACCTGCTGGGTGCCAGGTCACGTCTATGTAGAGGGG

SARS-CoV-2 Reference Genome Shuffle No.1 (17929) GATACCGTAGGGATACTTCGTGGGTATTTAGCTGTTTTCTATGCATAAGTCAGGATGGAATCGGTGAACTCGAC

Section 253

```
                                                    (18649) 18649      18660     18670     18680     18690     18700     18710     18722
Homo sapiens chromosome 1 NC_000001.11: 11783698-... (17678) GCCA--CCTGCTGCCATC---CGGTCAAACCTGTAGGGGTTTGTTTCTGAAGAGAGGTTCCAGGAGAATACC
     SARS-CoV-2 Reference Genome Shuffle No.1 (18003) ACCATTTCTGCTAAAAACTACTGATCTAACTTGTCGTGCGAGTAGGACATCCTACGTTTTC----ATCATTACA
```

Section 254

```
                                                    (18723) 18723  18730     18740     18750     18760     18770     18780          18796
Homo sapiens chromosome 1 NC_000001.11: 11783698-... (17747) ATCCGTCACTGCCTTCCCATGGGGCCCGGGAGTTAGAGAAGCATCAATGAAGGTTGGAGACAGACCA--ATCAG
     SARS-CoV-2 Reference Genome Shuffle No.1 (18073) ATTCTTCCTAACTTGAGCATGGTGTTGCTTATTTGACGTTTCAAAATGCAACTCACTTAAGTATCACCATACT
```

Section 255

```
                                                    (18797) 18797        18810     18820     18830     18840     18850     18860  18870
Homo sapiens chromosome 1 NC_000001.11: 11783698-... (17819) CGTTCATATCCTGGCTTTGCTACTTA----TAGCTGTGTGATCTTGGGTACATTACTTAATCTCTGTGAGTTCT
     SARS-CoV-2 Reference Genome Shuffle No.1 (18147) CGCACATGGGCTGTCGTTAAGAAGTTGGGGTAGAAATCCAAGTAGTGAGACCCCCTGTATTGACGCCGACATAG
```

Section 256

```
                                                    (18871) 18871   18880     18890     18900     18910     18920     18930        18944
Homo sapiens chromosome 1 NC_000001.11: 11783698-... (17889) GTTTCCTTATTTATA-AGACACGGCAGATAGTAGTACCCACTTCATAA-TAACACTA--GGATGCTGGGCGTGG
     SARS-CoV-2 Reference Genome Shuffle No.1 (18221) TCTACCTGTTTCATTTAGTGTTGTAAGGTCGTCTTATCGTCTACCTTCCTAACACAACTGTCAGATGAGCGCGA
```

Section 257

```
                                                    (18945) 18945 18950     18960     18970     18980     18990     19000          19018
Homo sapiens chromosome 1 NC_000001.11: 11783698-... (17959) TGGCTCACGCCTGTA-ATCCCAGCACTTCAGGAGGC----TGAAGTGAGTGGATCACTTGAGGTCAGGAGTTCC
     SARS-CoV-2 Reference Genome Shuffle No.1 (18295) ATATACAAAACTCAAGATCCAATTAGTATAAAATTCACCTTGAATTTGGTGCCAT-CTCAACGTCAGCTGTACC
```

Section 258

```
                                                    (19019) 19019      19030     19040     19050     19060     19070     19080     19092
Homo sapiens chromosome 1 NC_000001.11: 11783698-... (18028) AGACCGC---CTGGCCAA--CATAGTGAAACCT--CATCTCTACTAAAAATA-------CAAAAATT--AGCTG
     SARS-CoV-2 Reference Genome Shuffle No.1 (18368) GCATGTAATATTGTCCGAATCCTTGGGCGTTGTTCCATTCCTATTTAATAAATCCTTGGCATGTATTCAATTTG
```

Section 259

```
                                                    (19093) 19093  19100     19110     19120     19130     19140     19150          19166
Homo sapiens chromosome 1 NC_000001.11: 11783698-... (18086) GGTGTGGTGG--TGGGCACCTGTAATCCCAGCTACTTGAGAGGCTGAGGCAGGAGAATTGCTTGAGCCAGGAGG
     SARS-CoV-2 Reference Genome Shuffle No.1 (18442) GTTCTGTTACCCTGGGCACTGAAGATGTATGCTAACATTGTAATTCAACAAGGTACGTT--TTGAGC--GGA--
```

Section 260

(19167) 19167 19180 19190 19200 19210 19220 19230 19240
Homo sapiens chromosome 1 NC_000001.11: 11783698-... (18158) CAGAGGTTGCAATGAGCCAAGATCACACCACTGCACTCCAGCCTGGGCCACAGAATGAGACTCTGTCTCAAAAA
SARS-CoV-2 Reference Genome Shuffle No.1 (18510) CAAACTCTACGTTTATTCATTATAATAGGTCTGTTATTCAA--TTGGCCGTA-AATAATATTCC-TCAGATTTA

Section 261

(19241) 19241 19250 19260 19270 19280 19290 19300 19314
Homo sapiens chromosome 1 NC_000001.11: 11783698-... (18232) AAAAAAAAAAAAAAAAAAAGCAACAACAACAACAAAAAACCATAACACTAGGAGAATATGACGGCAGCTGGTTC
SARS-CoV-2 Reference Genome Shuffle No.1 (18580) AGCGTCATGTGGGACGCAAGTCGAATACTTTTCTCGAAACCGAACCAAAAGACCAATTTGCGAGCGGTGGTGAA

Section 262

(19315) 19315 19320 19330 19340 19350 19360 19370 19388
Homo sapiens chromosome 1 NC_000001.11: 11783698-... (18306) CACAGAACTTAGAACAGTGCCTGGTGTACAAACCGAATACTCAGTATATGTGAGGTGCCGTTATAATC---ACT
SARS-CoV-2 Reference Genome Shuffle No.1 (18654) GCCTGCTCTTGCTACAT--CCG-----ACAAACAGCGTGG---GTATAAGGGACGACGTGTTATAATCTACACA

Section 263

(19389) 19389 19400 19410 19420 19430 19440 19450 19462
Homo sapiens chromosome 1 NC_000001.11: 11783698-... (18377) ATTACAGAGGTAAGACACAACAACAGCGCTATCATTTTGTGTGAGAGCAGCACTT-----CAG--ACTTTTCAC
SARS-CoV-2 Reference Genome Shuffle No.1 (18718) ATTTGTGGTTTAAACCGTCCCTCTTGTGATACTTTTTTGTTTATTAT-ATCTCTTATATGAAGCAACAATTCCA

Section 264

(19463) 19463 19470 19480 19490 19500 19510 19520 19536
Homo sapiens chromosome 1 NC_000001.11: 11783698-... (18444) AGCGCTTGC--CTGTTTATTATCTCAGGTGAGTTAAGACATCATGATTGAGAGTTAGGGGAATCCTAACTGTGG
SARS-CoV-2 Reference Genome Shuffle No.1 (18791) TGAGCTGAGTGCTCCTTCTGATAACGTATTCAAACAGACGCGTTGGAATAAATTTAGGAGTCTTGCAAAGCTAG

Section 265

(19537) 19537 19550 19560 19570 19580 19590 19600 19610
Homo sapiens chromosome 1 NC_000001.11: 11783698-... (18516) CAAGCA-CAGCCTCGCTGTAGGAACAGTTAGAAAGAGGGTCAGCAGACTCACTTTCTTTCATGCCACCCCCCAA
SARS-CoV-2 Reference Genome Shuffle No.1 (18865) TAAATATCTGGACCTGTCCAGCACCGTGCGCGCTGATGTTCAACTTGTCGACTTCAAAACAATACTAGCTCAAT

Section 266

(19611) 19611 19620 19630 19640 19650 19660 19670 19684
Homo sapiens chromosome 1 NC_000001.11: 11783698-... (18589) GCCCTG-TGCAGCAGCTTACAGTTATGCAGACCAAGGCCAAGTTCCGAAACGAGACTCAAAAAAAGCCTGGAT
SARS-CoV-2 Reference Genome Shuffle No.1 (18939) GTACTGATGACATAGGTTCCAAGTAT----AACAAGCTAAGGATAAGACCTTTCTTTCAACAGAAAGCTA--AT

Section 267

		19685 19690 19700 19710 19720 19730 19740 19758
	(19685)	
Homo sapiens chromosome 1 NC_000001.11: 11783698-...	(18662)	GTGCA-CGAGTACTTGTGTTACAAGCTATCAACGGTGTATTTAAACATCCTTGTGCATTTAAACTTCAAAAACT
SARS-CoV-2 Reference Genome Shuffle No.1	(19007)	CTGCATCTACCCCGTCAGTCCCTTT-TCTCTACGTA--AGTTAAAAGTCCAT----AATTGTACTTAGACATCG

Section 268

		19759 19770 19780 19790 19800 19810 19820 19832
	(19759)	
Homo sapiens chromosome 1 NC_000001.11: 11783698-...	(18735)	TGGGAGTGACACGTGAGACGTGCATACAGTAAAAGGCATAAACTTAAAAAAAAAAC----TGCAATTCATGAAG
SARS-CoV-2 Reference Genome Shuffle No.1	(19074)	TAATAGTAGTTATCAATATGTTTAAAGAGATTTTCCCC---ACTTATAACGGAGGCATGTTGCAACACGTCAAT

Section 269

		19833 19840 19850 19860 19870 19880 19890 19906
	(19833)	
Homo sapiens chromosome 1 NC_000001.11: 11783698-...	(18805)	TGCTAATGTAATTCAGTTGGCATTTCTAGAGTTCAAGAAGCATAATCCAAATTTCTAACTTGCTGAAAACAATC
SARS-CoV-2 Reference Genome Shuffle No.1	(19145)	TGGCACGATTTGTGCATAGGCTTTTCGTTATTTGGTAAACGTTAATTT---TTTTGTGCGTGCAGATTTCG---

Section 270

		19907 19920 19930 19940 19950 19960 19970 19980
	(19907)	
Homo sapiens chromosome 1 NC_000001.11: 11783698-...	(18879)	TCACTGCTTTGTGACTTCCTAAACTGCACTCGTGATCCAGCCCCCAAGTTCCCTCAAAGAATACAGTGTGAGAA
SARS-CoV-2 Reference Genome Shuffle No.1	(19213)	TCAAAGTTTTAT-AGTAG---AACTAACCTCTGCAATTTGAAAAAGATTAGCCTTT--GATGACTGTGCCA-AA

Section 271

		19981 19990 20000 20010 20020 20030 20040 20054
	(19981)	
Homo sapiens chromosome 1 NC_000001.11: 11783698-...	(18953)	TCAACATT----TTTAACCCATTGAAGAGTGTGTCTAAAATACCTTGACACTGTGGAGGGACTTTTACAGGCA-
SARS-CoV-2 Reference Genome Shuffle No.1	(19280)	TTATCATTGGGATTTATGATATAGCTTGTTATGTGTTACATAG-TAGGCGCTCACGAACAAATTGCA-AGGCAC

Section 272

		20055 20060 20070 20080 20090 20100 20110 20128
	(20055)	
Homo sapiens chromosome 1 NC_000001.11: 11783698-...	(19022)	-A-CAGAGAAGTTGCAGATCAGATGACCCACTCTGCCTTCTCCTTCCTGCCCCCTCCGGCTG-CTT----TTTT
SARS-CoV-2 Reference Genome Shuffle No.1	(19352)	AAACCTAATAATACGAGCACAGAT--CTCGATTTATCTTTGACT-CGTAAGCCTGATGGATATCGTAAACTCTT

Section 273

		20129 20140 20150 20160 20170 20180 20190 20202
	(20129)	
Homo sapiens chromosome 1 NC_000001.11: 11783698-...	(19089)	CTGCCGGGTTAAAGGGCAGCCCAGCCTCAGGGCTGACAGTTTGCTCCCCAGGCACCACCACTCCCGCCTCCCAC
SARS-CoV-2 Reference Genome Shuffle No.1	(19423)	TTACTTGGTTGTAGGTC----CAGATT--GGTCGTGCAATTTGAATTACTCGCTTGACGA-TAACGAAGGGTTT

```
Section 274
(20203) 20203 20210 20220 20230 20240 20250 20260 20276
Homo sapiens chromosome 1 NC_000001.11: 11783698-... (19163) CTTAACCTTGCATGAGTTTACCTTGAGATGAGATTGACAGC-TCCCTCAGCAGTTCGAGGAGGGAAGAATTCCA
SARS-CoV-2 Reference Genome Shuffle No.1 (19490) -TGATACTAATAGGTGGTTACGATTTGGTG-GAT--ACAGCGTCGATGCGCACGTCTCGCGTTAATCTTGTTCT

Section 275
(20277) 20277 20290 20300 20310 20320 20330 20340 20350
Homo sapiens chromosome 1 NC_000001.11: 11783698-... (19236) GGGAGAACCACTTGTCACCAGATTCCAATCGCCGCCTCATCTTCTCCGGAGTCTCTCATGCCGCTCGG---GG
SARS-CoV-2 Reference Genome Shuffle No.1 (19560) GGTTCAAGTAAATG--AGTAGAATTTTAGGTCCACT--ATCGTA-CGCGAGGGATATAATGGATAATAAATTGG

Section 276
(20351) 20351 20360 20370 20380 20390 20400 20410 20424
Homo sapiens chromosome 1 NC_000001.11: 11783698-... (19307) TCCAGGCCCGGGGTGGAACATCTCGAACTATCTTTGG-AGCTCTC-ACTGCCACTGCTGGCACTGCCCTCCAAG
SARS-CoV-2 Reference Genome Shuffle No.1 (19629) ACTTGGAAAGTTTTAGTTGTTCTTATTGTATGGTAGATATGCCTCGAGTAAAAGTACTTTTAGCGCGTTCCATG

Section 277
(20425) 20425 20430 20440 20450 20460 20470 20480 20498
Homo sapiens chromosome 1 NC_000001.11: 11783698-... (19379) CAGGGGTTGAG-GCTGCTGTTTCCTCTGGCTTCGTTCACCATGGCTGGGTTCC--TACTGCACAGGGTGGGGCA
SARS-CoV-2 Reference Genome Shuffle No.1 (19703) --GGTGTAGAATGAACCTTTTAGATATATCTTATTATCTAATGCCCATCCTCGATTACTCTACTTCGCAGATGT

Section 278
(20499) 20499 20510 20520 20530 20540 20550 20560 20572
Homo sapiens chromosome 1 NC_000001.11: 11783698-... (19450) GGGAGCATCAGGGGGCAGGCAGCCACACCCCCGACACATCAAG-ACACCTGAGTGGCAGGTTCAAGCCGGAGGC
SARS-CoV-2 Reference Genome Shuffle No.1 (19775) GAAAATATCCGTTTGCTGATCAAAAAAGAATGTACTTGGCAAATATTACTAAAT---ATATGTAATCTTGATTT

Section 279
(20573) 20573 20580 20590 20600 20610 20620 20630 20646
Homo sapiens chromosome 1 NC_000001.11: 11783698-... (19523) GCTGTATTTCC--ACACAGGAAGAAGGCCAA-AAAAGGTGACACTGCCCCCTCCCAGTGGCTCCATGCTCCTCA
SARS-CoV-2 Reference Genome Shuffle No.1 (19846) AATATAAGCGCGAAAAATCGCACATATCAATTAATAACTTACGCTTAACGTTACGGTTGTTTGTATATTGATCT

Section 280
(20647) 20647 20660 20670 20680 20690 20700 20710 20720
Homo sapiens chromosome 1 NC_000001.11: 11783698-... (19594) GCTATGGCTGTCCGGGCCGCCTCACTCAA-AGCCTTGCC---CTCCGCTGC-TGCCAGGCTCCTTGCATGCAAG
SARS-CoV-2 Reference Genome Shuffle No.1 (19920) TGGATGGGGATGGAGACAGTGAAACTTACTATATTTGGGTTATTCCTATACCTAACAGTCCAGATTTAAGTGGG
```

Section 281

(20721) 20721 20730 20740 20750 20760 20770 20780 20794
Homo sapiens chromosome 1 NC_000001.11: 11783698-... (19663) GCAGCCCCCACCCGGCACTGCAACATGCTCCTCGGTGACAGCTACCTGATGGAAGGTGGCACAGAGGACCTCAC
SARS-CoV-2 Reference Genome Shuffle No.1 (19994) GAACTAAATAATAAGTTTTTCCTAATTCTATCGGGTGA-----ACCCGGTCG---GTGA-AGAATTAACCTTAA

Section 282

(20795) 20795 20800 20810 20820 20830 20840 20850 20868
Homo sapiens chromosome 1 NC_000001.11: 11783698-... (19737) GCTGC-CGACCTGCGAGGCCCATATTCCTAGCGAGGGCAGTAGTGCCCAGCTGGAGGACCCTGGCTTTTCGA
SARS-CoV-2 Reference Genome Shuffle No.1 (20059) TCTTAACCAGCGCCCAGTCCC-TATTGTTACTACGTGTCGTTGTTTTTCA--TGTTATAACA-TACAATTACGT

Section 283

(20869) 20869 20880 20890 20900 20910 20920 20930 20942
Homo sapiens chromosome 1 NC_000001.11: 11783698-... (19810) TGGTCCATCTCTGTCAGCTCAGGCCCAGAGTGCGTGGGGAGGGAACAGAGGCACCTTCCCCAGCGGGTGTGTTC
SARS-CoV-2 Reference Genome Shuffle No.1 (20129) ATCTGGCTGTGAGTTACATATGTAAATGAGAT-GTGGTGACTCAACAAA------TATGACAG-----GATACC

Section 284

(20943) 20943 20950 20960 20970 20980 20990 21000 21016
Homo sapiens chromosome 1 NC_000001.11: 11783698-... (19884) AACTGGCGGAGCTGCGACAAAGGAAGAGGCTTGAGCTCCAGCCAGCACAAGGGGCGGGGGCAAGGCTTCCAG
SARS-CoV-2 Reference Genome Shuffle No.1 (20191) TCCTGGAATAATC-GAGAGTGAGGAAGATACTTG--------CCAGTGCGAGTGATCCAG--CAAGACATATAT

Section 285

(21017) 21017 21030 21040 21050 21060 21070 21080 21090
Homo sapiens chromosome 1 NC_000001.11: 11783698-... (19958) GCGAGGAGGGAGGGCTGAG----GCTCTAGTAAGGGAGGCGGATGGA--AGGAGGG-GGACTCAGGGGCAGAG
SARS-CoV-2 Reference Genome Shuffle No.1 (20254) TCAAATTCCAATAACGGAGTCGTGCCCTGTCAAGAGCAGCCGCCTACACTACGACATAGGGCCAATTGACACAT

Section 286

(21091) 21091 21100 21110 21120 21130 21140 21150 21164
Homo sapiens chromosome 1 NC_000001.11: 11783698-... (20025) ACTGCAGGGGTTGGTGGAGATGAGGCCAACAGGCGTTCAGGGATACCAGATCACCCTCCAGAGAAGGAACAGCT
SARS-CoV-2 Reference Genome Shuffle No.1 (20328) ATTACTATC-TCGGCACAGGATTGGC--ATAGG-GTTTAAGCATACCTAATGAAAACATTGATAAT-AGTTGCT

Section 287

(21165) 21165 21170 21180 21190 21200 21210 21220 21238
Homo sapiens chromosome 1 NC_000001.11: 11783698-... (20099) CACTCTGGCTGGCTCTGCCTGGAACAT-CTGTTACTTGAAACAGTTCGTGCACAGGATGGAAGGGGTGAGGCT
SARS-CoV-2 Reference Genome Shuffle No.1 (20397) -ACTCTT-CACATTCTACATTTTCCGTGCTCTTAGTAGTACGGTTATGAGCAAAGGCGTTAA--TGAAAGCT

```
                                                                         Section 288
                                                          (21239) 21239      21250          21260          21270          21280          21290          21300     21312
Homo sapiens chromosome 1 NC_000001.11: 11783698-... (20172) TGAC----TATGCAGCCAGATTTCTCCTGAATTCATGCTACTCCAAAAAGACCGTGTTGTGGGTTAGAACTTGA
       SARS-CoV-2 Reference Genome Shuffle No.1 (20467) TGTTGTGTTAAGCAGC-AGAGTTACAGTGGAACCCCGCCATATTAGGATGTTATTTAAGTA--TTTGTTCTCAA
                                                                         Section 289
                                                          (21313) 21313 21320          21330          21340          21350          21360          21370          21386
Homo sapiens chromosome 1 NC_000001.11: 11783698-... (20242) TGCTGGCCATGTTCCAAAAATAGCCAGACC-----CAAGTTCAGTAAGGGCA-CGAATTT--CTCAGAACAAAA
       SARS-CoV-2 Reference Genome Shuffle No.1 (20538) CAATTTAAAAAGGCTTTTAATAGCAAGTCCACTGACAAGCCCACAGGTCGCAGCGTAGGTAAGTCAGATCCCAC
                                                                         Section 290
                                                          (21387) 21387        21400          21410          21420          21430          21440          21450  21460
Homo sapiens chromosome 1 NC_000001.11: 11783698-... (20308) GCTGCCCCATCTGTGCTTGCCTTGCAGGCTGCTCTTCTTACACAT-CACAGAATTCAATTCAAATCATCACAGT
       SARS-CoV-2 Reference Genome Shuffle No.1 (20612) TATTACACAT---TGAGTTAATTAGATTACCCGAGTATAACGGAAACACCGGCTCCAAGTGGTACATTCGC-GT
                                                                         Section 291
                                                          (21461) 21461   21470          21480          21490          21500          21510          21520          21534
Homo sapiens chromosome 1 NC_000001.11: 11783698-... (20381) GACCCAGC-TACTGGGAAGGCTGAGGCAGAAGGATTGCCT-GAGCC-CAGGAGT-TCAAGGCCAGCCTGGGCAA
       SARS-CoV-2 Reference Genome Shuffle No.1 (20682) AAGCTGGAATAATAAGTAAGATCTTATACAATTGGTGCATAGATTATCTGGAATATCAAACGCTCGTACGTCCA
                                                                         Section 292
                                                          (21535) 21535 21540          21550          21560          21570          21580          21590          21608
Homo sapiens chromosome 1 NC_000001.11: 11783698-... (20451) CATAGCGAGACTC----CATCTCTTAAAAAAACAAA---AAACACACCTGTAATCCCAGCACTTTGGGAGGCCG
       SARS-CoV-2 Reference Genome Shuffle No.1 (20756) GTTAAGCAGACATAAGTCAATACTTAACTATATCAGCCCAATCTAACCGAGAGTACACTCAAGCGACCGGGCAG
                                                                         Section 293
                                                          (21609) 21609      21620          21630          21640          21650          21660          21670     21682
Homo sapiens chromosome 1 NC_000001.11: 11783698-... (20518) AGGTGGGTGGATCACCTGAGGCCAGGAGTTTGAGACCAGCCTGGCCAACATGGT-GAAATCCCGACTCTACTAA
       SARS-CoV-2 Reference Genome Shuffle No.1 (20830) ATAATTTTAGGTTAAAATATATTATGCATTAGGCAAGAGCGAAGAGTACAGGTTAGCTTTGTCCAAGGAGCCCG
                                                                         Section 294
                                                          (21683) 21683 21690          21700          21710          21720          21730          21740          21756
Homo sapiens chromosome 1 NC_000001.11: 11783698-... (20591) AAATATAAAAATTAGCCAGGTTTGGTGGCGGGCGCCTGTAATCCCAGCTACTCTGGAGGCTGAGGCAGGAGAAT
       SARS-CoV-2 Reference Genome Shuffle No.1 (20904) CCATAGAATGATTTTTAATAAATTATAGTGGGGGGTT--AAGCATTTCTGGTATCAACTTTT-GGCAGCACCTT
```

```
                                                                        Section 295
                                            (21757) 21757        21770     21780     21790     21800     21810     21820   21830
Homo sapiens chromosome 1 NC_000001.11: 11783698-... (20665) CGCTTGAACCCGGAAGGCAGAGGTTGCAGTGAACCAAGATTG--TGCCATAGCACTCCAGCCTG-------GGC
         SARS-CoV-2 Reference Genome Shuffle No.1 (20975) ATTCTGTTACCAGTGAAAATATTTTGACGTAATCATATAACGGATGCCCAGGTGCTAGTTCGTGTTTTGTTGTC
                                                                        Section 296
                                            (21831) 21831    21840     21850     21860     21870     21880     21890         21904
Homo sapiens chromosome 1 NC_000001.11: 11783698-... (20730) GACAAAAGCAA--AACTCCATCTCAAAAAAAAAAACAAAAACAAAAAACAGAAACAAGTAATCAGAGCCACCAG
         SARS-CoV-2 Reference Genome Shuffle No.1 (21049) GTTTGATCGATTTTACTAAATTTAAATTAATTACACCAAATCCTTTATTGAAATCAATTTTGCAGTCAAACAAG
                                                                        Section 297
                                            (21905) 21905 21910     21920     21930     21940     21950     21960         21978
Homo sapiens chromosome 1 NC_000001.11: 11783698-... (20802) GGC-ACAGACAGGCCAACAGAGGGCATAGGGTCATCTGGCAGTCTGGTTA-AGAAAATAAGACAAACACTTAA
         SARS-CoV-2 Reference Genome Shuffle No.1 (21123) AGAGATATTCGTCCCAATTTAGCGAAGTTGATTTTTTCA-AGTAAGGTTTTAACAAATTATAATTTCATGCGAA
                                                                        Section 298
                                            (21979) 21979       21990     22000     22010     22020     22030     22040   22052
Homo sapiens chromosome 1 NC_000001.11: 11783698-... (20874) AACAAACAAAACACTTGCACAGCAA-TGGGCAAACACACAGTAGTGAATGGGTGTGTGGTGACATGGCACAACA
         SARS-CoV-2 Reference Genome Shuffle No.1 (21196) AACGATTGGAATAATTATGCGGATGGTGGACCAAAACACTCATATAA-------TGTTCAGATACAATTTCTGT
                                                                        Section 299
                                            (22053) 22053  22060     22070     22080     22090     22100     22110         22126
Homo sapiens chromosome 1 NC_000001.11: 11783698-... (20947) GAGGAAAGCCCAGGTGAGGTTCATTTCTTTCAGACTGTCCACCACTAACCCTGTGTACCTAACCATTTGGGGTC
         SARS-CoV-2 Reference Genome Shuffle No.1 (21263) GCGTATAAGACAACTTTGTTTTGCACCGTAGACACATAGGCAGACTGAACATATAAATTGAATCTACTCGGAAC
                                                                        Section 300
                                            (22127) 22127        22140     22150     22160     22170     22180     22190   22200
Homo sapiens chromosome 1 NC_000001.11: 11783698-... (21021) TTAGACTCT----TTTGACAATCTGATTAAATG-CACT-GCTCCCTTCCCCTGAAATACACACACAGGTGCACA
         SARS-CoV-2 Reference Genome Shuffle No.1 (21337) TTCTCCGTTATGCTTCCAAGATCTGATTTTTCGTCACTCGCATAGGACCCGCGTATTAAATCAGTCTTTAATAA
                                                                        Section 301
                                            (22201) 22201    22210     22220     22230     22240     22250     22260         22274
Homo sapiens chromosome 1 NC_000001.11: 11783698-... (21089) GACTACACGGGGATACATCCAAAGACAAGGAGTTGATTCCAGACTAAGAACCCTGGGCCAAAGCTTTCTCCCTC
         SARS-CoV-2 Reference Genome Shuffle No.1 (21411) GGCAAAACATT--TACTTC---AGGTATAGAAATCTTTGCGGTC-AGGAAACTAATAACTACTTGTTTT---TT
```

```
                                                                                   Section 302
                                                      (22275) 22275 22280     22290     22300     22310     22320     22330         22348
Homo sapiens chromosome 1 NC_000001.11: 11783698-... (21163) TAGGGCTGCTCTCTTTCATTCTGACCTCAAACTCCAGATTCTCAGTGGGG---ATGGCTGTGGGGAGTGAGGAA
          SARS-CoV-2 Reference Genome Shuffle No.1 (21476) GAAGCCTGGT-TATTCACGTCCGCCATTATCCATCA-ATGCTCAGTGAGGTTTATCGATTAACATCGTAAGTAT
                                                                                   Section 303
                                                      (22349) 22349      22360     22370     22380     22390     22400     22410        22422
Homo sapiens chromosome 1 NC_000001.11: 11783698-... (21234) CGGGGGCGGGGGGGAGGAGTAACAGCAAGAGCAGCCAGTTGGCTTTCCTCCAGCGT--TTAAGGGCGCACCTCG
          SARS-CoV-2 Reference Genome Shuffle No.1 (21548) CCATT-CACATATGATTTG-ACTTGCCAAAG-AGATATTTGGATTTAAGCCAAAGTGGTTAACGAGTAAAACAT
                                                                                   Section 304
                                                      (22423) 22423  22430     22440     22450     22460     22470     22480           22496
Homo sapiens chromosome 1 NC_000001.11: 11783698-... (21306) TTAATTGCTTGCTG-GAACAGAACCTAGAGTAGGAGGAAGCTGAACCACCTGTAAACCCTCTGATTTTCAAAGA
          SARS-CoV-2 Reference Genome Shuffle No.1 (21619) GCAATAGCCTACAGCGAACATTCTTGTTACGAATATTAAGCGCATTAAAGAAGACAGCA-CTTTTGTTAGATTA
                                                                                   Section 305
                                                      (22497) 22497        22510     22520     22530     22540     22550     22560   22570
Homo sapiens chromosome 1 NC_000001.11: 11783698-... (21379) CTTATTGCA--TTCCTAG-TATATGCCACGCATGTGCCATATTTG-TAAGCCAACTCCCACGAT-TTATCCG--
          SARS-CoV-2 Reference Genome Shuffle No.1 (21692) CTTCTTAGAAGTTACAAGATATCAGAACATCAGGTGTGGTACTCGCTCATGAATTATCTAAGTTCTTACCCGAG
                                                                                   Section 306
                                                      (22571) 22571    22580     22590     22600     22610     22620     22630          22644
Homo sapiens chromosome 1 NC_000001.11: 11783698-... (21446) ---TTATCAGCTGGACTCCCATAGCACCCTGGGTTGTAACTGTGGGTGAAAAGGCCTGTCTCCTTTCTCTTTCC
          SARS-CoV-2 Reference Genome Shuffle No.1 (21766) CGAAGAACAGCCTG-CTTATATAATGCTCGGGATTATGTCCGTGCTTGAAATT-CGAAAATCAGTGATATTACG
                                                                                   Section 307
                                                      (22645) 22645 22650     22660     22670     22680     22690     22700         22718
Homo sapiens chromosome 1 NC_000001.11: 11783698-... (21517) CC-AGTTCCCTGAGACAGGACCGGTGTCCTGCCTGGGGTATCCCCAGAGTTTGGCACACGGTGATAGCCAACAT
          SARS-CoV-2 Reference Genome Shuffle No.1 (21838) GAGAATTCTCGTCTACCTCAA-GAAATCTAGTTTGTTGTAAGAGTACATGTTAGCCGTGTTATACATCTATACT
                                                                                   Section 308
                                                      (22719) 22719      22730     22740     22750     22760     22770     22780        22792
Homo sapiens chromosome 1 NC_000001.11: 11783698-... (21590) TCACTGAGCGCCAAAGGGCCAGGTGCTGCCACTCTCTCAAAATAAGCCTCTGCCACTTACTGAACAACTAGTCT
          SARS-CoV-2 Reference Genome Shuffle No.1 (21911) TAAGT-ATTGGAATGTGGTCAGTTTCAATCGCGAAGACATGAAATCTCTTTTCAGTGCGGTCACCACTGAAACT
```

Section 309

(22793)		22793 22800 22810 22820 22830 22840 22850 22866
Homo sapiens chromosome 1 NC_000001.11: 11783698-...	(21664)	GCGCCAAGCACTGGGATACTAAGCCTACGACTC-CCAGAAAGGTCCCGGCGGACCCCCGTGCGC-AGCGGGACG
SARS-CoV-2 Reference Genome Shuffle No.1	(21984)	GCATACATCAGCTGTACCCTTTCGATAGAGTTCTCTCCTGAGATCGGTTTCTAATTACGCGAGATAGATAGA-G

Section 310

(22867)		22867 22880 22890 22900 22910 22920 22930 22940
Homo sapiens chromosome 1 NC_000001.11: 11783698-...	(21736)	CAGTGGGCGCCAGGGACCGCAGTGCCCCGGGCCCAGCGCTCTTCTCCAGGTGATCGCCGGGGAGGCAGGTTTAA
SARS-CoV-2 Reference Genome Shuffle No.1	(22057)	CAGTTAA-GCCATG---CTAAATTTTGCGCACAGAGAATAGTCAGCGAGGGGATCTACGGGT-------TTTGA

Section 311

(22941)		22941 22950 22960 22970 22980 22990 23000 23014
Homo sapiens chromosome 1 NC_000001.11: 11783698-...	(21810)	AAGGCAGGAGCCGGAAGCCGTCTCGGCGCCCAGGTGGCCGAGAATCCAGTCACCAGGTCACTGAGTCACCGATG
SARS-CoV-2 Reference Genome Shuffle No.1	(22120)	AGCTTAGGCGAGTGA-GCTTTCATTTTTGGCCTGTGAGAGCGTTTCCATTC-CGTTGTTTCTAACTTAAGGCTT

Section 312

(23015)		23015 23020 23030 23040 23050 23060 23070 23088
Homo sapiens chromosome 1 NC_000001.11: 11783698-...	(21884)	GGGGCGAGGACACGGGCCTGGGCCGGTCAGAGGGGCGGGATCGTCACCCCTGGCTCAGGGGCCCCTCCGTCCAG
SARS-CoV-2 Reference Genome Shuffle No.1	(22192)	CTGG--AGGAAAGTCACCTTAAAC---------------AAAGTTAAAGCTATATCTAG----CATTTTTCCTC

Section 313

(23089)		23089 23100 23110 23120 23130 23140 23150 23162
Homo sapiens chromosome 1 NC_000001.11: 11783698-...	(21958)	GCAGGGAGCCAAAGTCAGTCTTCGCTTGAGGGTTGGCGGTCGCTGGAAGTGGTAG-----CCATTGGGAGTTAC
SARS-CoV-2 Reference Genome Shuffle No.1	(22245)	GACGGGTT--AGGTTCAACATTCTCTCGAGCATTGAAGAGT-CTGAAATTCTTTGTTATACTATAGTTTTTGAT

Section 314

(23163)		23163 23170 23180 23190 23200 23210 23220 23236
Homo sapiens chromosome 1 NC_000001.11: 11783698-...	(22027)	ACTAATCCCGCGAAGGGTGCGCAAGGGAGGCGGCAGCCCCCCCCAAGAAGAGAGGC--AGGCCGG-CCTCCAGC
SARS-CoV-2 Reference Genome Shuffle No.1	(22316)	GCCAATTCCGCTTAAATTG----AGATATACAGCAGACTTCTACAATAAAATGTGCTTAAGATAATCCTATAAG

Section 315

(23237)		23237 23250 23260 23270 23280 23290 23300 23310
Homo sapiens chromosome 1 NC_000001.11: 11783698-...	(22098)	GCTCCCCGCCCACAAGCACGTCCTTGCCCCCGGGAGGTTGTTTGCCGGCTCCAGGATCTCCCTCCCGGCGACCC
SARS-CoV-2 Reference Genome Shuffle No.1	(22386)	TAGGCAATCTTAGAA-----TATTTGCAA----GGGGATAAAGACACGCTACAATCTGCCCCTTCTGAGGCCTA

```
                                                              Section 316
                                                  (23311) 23311    23320     23330     23340     23350     23360     23370       23384
Homo sapiens chromosome 1 NC_000001.11: 11783698-... (22172) CGGCCTCGCCGTCACTCACCCTCCAGTCCCGGGTGACTCAGAACGCTCGGCCGCCGGCCGCACCACTGTGGCCG
       SARS-CoV-2 Reference Genome Shuffle No.1 (22451) TGGACAAAACATGAC-CATCTTCGCGTGTTTAAAAACATAATACAC-CACCCTGCTTATAAAGTATACATACCT

                                                              Section 317
                                                  (23385) 23385 23390     23400     23410     23420     23430     23440        23458
Homo sapiens chromosome 1 NC_000001.11: 11783698-... (22246) CTGCAGGGGCGGG---GAACGCAGTGA-AGGGGCGCGGCGCAGGTGTCAGGTTGCTGGAGAGGCGGGCCTACAG
       SARS-CoV-2 Reference Genome Shuffle No.1 (22523) CACTATGAGTTCGAATGAACTAAGTTTCATGAGCTTTACACTCAAGACTTTTTTGTGAATACAATC-CTTTCAT

                                                              Section 318
                                                  (23459) 23459      23470     23480     23490     23500     23510     23520     23532
Homo sapiens chromosome 1 NC_000001.11: 11783698-... (22316) GGCGGA--AACCACCGGCGCGGGTGACCGGAAGCCCCGCCCCCAACATGGCCGTGCCCACAGGTCTCGTTGGAG
       SARS-CoV-2 Reference Genome Shuffle No.1 (22596) TTCTTCCCAATTATTAGAGCTATTGATCTGTGCGGTAG----------TGG--GATACTCCGGGACGGCCATGAG

                                                              Section 319
                                                  (23533) 23533  23540     23550     23560     23570     23580     23590       23606
Homo sapiens chromosome 1 NC_000001.11: 11783698-... (22388) GCCGTGCCTTTATCGTCATGTGACGCGAGTCCTCGCCCCACCCGTCTGCAGCCACTCCTGGTCTC-AGTCCCAG
       SARS-CoV-2 Reference Genome Shuffle No.1 (22659) CCCTTTCCTTTGATTAATTGTTAGTGTAGTATTAATAACAAAAAGACTAAG----TAGTGATATGTACTACCAC

                                                              Section 320
                                                  (23607) 23607        23620     23630     23640     23650     23660     23670 23680
Homo sapiens chromosome 1 NC_000001.11: 11783698-... (22461) AG-CTCGAGAGGGCCACGTGACCGTCCCGGGGCCAGTCACGTGAGGCGCAGAT-CCTGGCTGGGA-------GG
       SARS-CoV-2 Reference Genome Shuffle No.1 (22729) AAACAATACACTGTAATCTTTACGAGTAGGCATTTAAAAACTGATTCATATATACGTAGTTGTGACTATACCGG

                                                              Section 321
                                                  (23681) 23681    23690     23700     23710     23720     23730     23740       23754
Homo sapiens chromosome 1 NC_000001.11: 11783698-... (22526) GGGTTGGTAGAGGGGTCCAGAGTGGCAGTAAAGGAGGAAGATGGCGGGGTGCAGGGGGTCTCTGTGCTGCTGCT
       SARS-CoV-2 Reference Genome Shuffle No.1 (22803) ACACTCGTAGATGAGCTGATAGT--CATTGACGATGCGTTATCAAAGTCTTCAGATCATAAGTTATATCAAACC

                                                              Section 322
                                                  (23755) 23755 23760     23770     23780     23790     23800     23810        23828
Homo sapiens chromosome 1 NC_000001.11: 11783698-... (22600) GCAGGTGGTGCTGCTGCTGCGGTGAGCGTGAGACCCGCACCCCCGAGGAGCTGGTAAGAAGCCGGCGG--AGTC
       SARS-CoV-2 Reference Genome Shuffle No.1 (22875) ACCGCATATACTTCCGTACACCATAGAAGTAGTAGTAATCATTGCGTTAATTTATGAGAAACGTGTCATAATTA
```

```
                                                          Section 323
                                                    (23829) 23829      23840     23850     23860     23870     23880     23890     23902
Homo sapiens chromosome 1 NC_000001.11: 11783698-... (22672) GGCC---TGGGGAGGGGGCGGTTGCTAAGGGACTGAGAGAGGCGTTGCCGGGGTGGGGCCGGGCCAATCTGGG
      SARS-CoV-2 Reference Genome Shuffle No.1 (22949) TGACGTATATCTAAGCTTAGTAATATAACTCTCTTAGGTACTCGCTACCCGGGAAATTAAACGGGGAGAAAGTT
                                                          Section 324
                                                    (23903) 23903  23910      23920     23930     23940     23950     23960          23976
Homo sapiens chromosome 1 NC_000001.11: 11783698-... (22743) CCCGCAGGTGGCAGCGGGTGGGGCCTGGGGACCGCAGCCAGGGCTCTCGAAGCGTCTACCCTGCTTCA------
      SARS-CoV-2 Reference Genome Shuffle No.1 (23023) ATCTAATGCGACCGCTG-TCGGGCCTTAAATGCACAGTTATAGAAGATATGACCGGTTCCATGTCTCATAAAAG
                                                          Section 325
                                                    (23977) 23977        23990     24000     24010     24020     24030     24040 24050
Homo sapiens chromosome 1 NC_000001.11: 11783698-... (22811) --CGTGCCTAAGTCTGGGGTCTTTTCTCATAACCCCTCCTGGAATCTGAGGGAGAATCCAGGCCAGCTCCAGTA
      SARS-CoV-2 Reference Genome Shuffle No.1 (23096) ATCATGTCAAAGACTTGCCAGCGATCTCCCATGCAC--CTACCACCTTCGATTCCTTCGAAGATAACACGTGTA
                                                          Section 326
                                                    (24051) 24051    24060     24070     24080     24090     24100     24110        24124
Homo sapiens chromosome 1 NC_000001.11: 11783698-... (22883) AATCCCACCCATGTTTCCTTTTATTTCCATCTGAAAAGAGTTTGGAATGAAACAAGCGTTTTCAGA-ATGAGCT
      SARS-CoV-2 Reference Genome Shuffle No.1 (23168) GTT---ACTCAAACTAGATGAGAATAATATCTTATTACCGTTAAATAAGGTCCG-GCGATTGCACTTATGAGCA
                                                          Section 327
                                                    (24125) 24125 24130      24140     24150     24160     24170     24180          24198
Homo sapiens chromosome 1 NC_000001.11: 11783698-... (22956) TCC-TCACAGGACTTTGTGCTTATC---ATGCTGCTGTTTTAGCTTCTCCAGTCCTTTCTCACTCTATCCCTCA
      SARS-CoV-2 Reference Genome Shuffle No.1 (23238) TGAGTCTGAATATGTTCCGATACCCCATACGCCGCTTTACTAGGTCCAACCACAGCTAGTAATGAAGTTTGTGA
                                                          Section 328
                                                    (24199) 24199      24210     24220     24230     24240     24250     24260     24272
Homo sapiens chromosome 1 NC_000001.11: 11783698-... (23026) CCCTTTCTGGGTCACTGACA--GTCCAGCTTCCCCCTGCAAAGCATCCTGGGAAAACTTGAGTTGTGTTCCCAG
      SARS-CoV-2 Reference Genome Shuffle No.1 (23312) CCGG----GGGCCAAAAGTAATGTACTGTTCTCCTCTTTTTACAATA-TGCTTAAA-TAAAGGTGTGTATG-AT
                                                          Section 329
                                                    (24273) 24273  24280      24290     24300     24310     24320     24330          24346
Homo sapiens chromosome 1 NC_000001.11: 11783698-... (23098) AGTTGACAAGTCCTTTCTGAT---TTCAGATACGTCACACTGTCAAAT-GGAACCTGCATTTTTACCCCCCTTT
      SARS-CoV-2 Reference Genome Shuffle No.1 (23379) TGTACGTATGTAATCTGGGATCGATGGAAAGCTAAGAAACTATTACTTAGTAAACAATAAGATTTCTCATGGTA
```

Section 330

```
                                                     (24347) 24347        24360          24370          24380          24390          24400          24410    24420
Homo sapiens chromosome 1 NC_000001.11: 11783698-... (23168) TCAG-CTGG--CGTTGGACATCTTTGAGGTG--T--CCTGAAGAGGAGGGTGGTTA-TTTGGAGACTATCAGTG
SARS-CoV-2 Reference Genome Shuffle No.1             (23453) GCTGTCGGAACCGATGTAAAAAATTCGGTTGAATCTTCGGGATAGCTGCCAGACCCGTATTGAGAGCATAAAAG
```

Section 331

```
                                                     (24421) 24421       24430          24440          24450          24460          24470          24480       24494
Homo sapiens chromosome 1 NC_000001.11: 11783698-... (23234) GAAATGAAGAGCTCAGCT-GATGGCTGTAGATCCTCACCCACATGGTCCCTCACCCCTGAAAGGTTTTTTTTGA
SARS-CoV-2 Reference Genome Shuffle No.1             (23527) CCGATCACTGACTGGGATTGGTTGCGTCTAAGACTAAGTTAC--GGTATATCACCCTAAGATCCTTTTAATTAC
```

Section 332

```
                                                     (24495) 24495 24500          24510          24520          24530          24540          24550           24568
Homo sapiens chromosome 1 NC_000001.11: 11783698-... (23307) GGGCTGGTTCCTATTTGATAATGGCTCCAGATGATTTAGAAGCTAG--CCA-CTGATTTCAGTAAATACTTCTG
SARS-CoV-2 Reference Genome Shuffle No.1             (23599) GCACT--TTTCTTTGCCCGACGGGCGAAGAATTAAATATACGCTACGCCCATCGAATTTTTGTTGTTGCTTATC
```

Section 333

```
                                                     (24569) 24569           24580          24590          24600          24610          24620          24630   24642
Homo sapiens chromosome 1 NC_000001.11: 11783698-... (23378) CCTCCTTCCACTCCTAACCACTAAAAAAGGCTCCCTCTGCGGATCTGTTTTTCTAGACCATCCTTGGAGAAACA
SARS-CoV-2 Reference Genome Shuffle No.1             (23671) CCGCGGTACTCGATTTACCATAAGACTTCCTTATCTCCATACTTTAAAAGATCTCAACCATCATCCGATAAA--
```

Section 334

```
                                                     (24643) 24643  24650          24660          24670          24680          24690          24700           24716
Homo sapiens chromosome 1 NC_000001.11: 11783698-... (23452) CAGGAGGAGGAGGATGAGATTCTTCCAAGGAAAGACTATGAGGTGAGCTCCTTTGATACTGCTTGGGCAACTAA
SARS-CoV-2 Reference Genome Shuffle No.1             (23743) -ATGAAGACACG--TGATA-------AAGCAGGCGCTATTTATTGAACTC--TTAATCACCCTTGACGCAGTAC
```

Section 335

```
                                                     (24717) 24717        24730          24740          24750          24760          24770          24780    24790
Homo sapiens chromosome 1 NC_000001.11: 11783698-... (23526) AGTAGTGAGTGGCCTGGTCACTTCAGGCCGAAGCATTATGATTCCAACCAGAGATGCTC-TTTGACCTTCTGTC
SARS-CoV-2 Reference Genome Shuffle No.1             (23805) AGG-GTCAGA----TTGTCAATACCCAATGATGTATTATCAGAAATATTGTATTAACTCATTTTACTATATTAC
```

Section 336

```
                                                     (24791) 24791       24800          24810          24820          24830          24840          24850       24864
Homo sapiens chromosome 1 NC_000001.11: 11783698-... (23599) CCAGGGAGTGAGTGGGTAAGAGAACTTCCTTCTAGGAAAGAGACTGTTTAGCAAGCCCTTTTCTCACCTTAACC
SARS-CoV-2 Reference Genome Shuffle No.1             (23874) GCTAAGCTT-AGAAGGAACTATCACTTGGA-CTTGCACAGATAG-----AGCGGCCTCATAACGTGAGTTGACA
```

Section 337

```
                                                          24865 24870     24880     24890     24900     24910     24920            24938
                                                  (24865)
Homo sapiens chromosome 1 NC_000001.11: 11783698-... (23673) CCAAAACAAGGTCTGGTGTTTTTACACGTTTATAAATCTGTGGCACCTTAACCTGGATAGAGTATGTATTTGCC
SARS-CoV-2 Reference Genome Shuffle No.1 (23941) CGCCA-CTAGGGATGCCAGTGGTTCACGCTT-TCATTTTTAAAAACCAT---CTGTATCCGGTGCAAACAT--C
```

Section 338

```
                                                          24939      24950     24960     24970     24980     24990     25000      25012
                                                  (24939)
Homo sapiens chromosome 1 NC_000001.11: 11783698-... (23747) ATTAAACTGTTTGCTTTTACAGCTTACAGAGAGACTT-AGAGTGAAAGGCACTGGCAGGCAGTATGTGCCCCTA
SARS-CoV-2 Reference Genome Shuffle No.1 (24008) GTTA--CCGTTGGAGCATACATATAAAGGAGGTCCGAGAGGGTGCAAGGATTTCGGGTCTACT-TATCGTGGTC
```

Section 339

```
                                                          25013   25020     25030     25040     25050     25060     25070          25086
                                                  (25013)
Homo sapiens chromosome 1 NC_000001.11: 11783698-... (23820) ACAGGTGTGTGCTTATAAA-CTGTTAAGGAGGCCTTGCTTCTCTTCTAGGAGAGCTTGCTTTTCAGATATTTTC
SARS-CoV-2 Reference Genome Shuffle No.1 (24079) ACATATCGCTATCTATGTTGCTTTTTTTCCATAGTATG--TCCACTCATTGAATGTATGAAGAACTCA-ATTTAG
```

Section 340

```
                                                          25087         25100     25110     25120     25130     25140     25150  25160
                                                  (25087)
Homo sapiens chromosome 1 NC_000001.11: 11783698-... (23893) TTGCTCAAGCAGCTTCTCTCTGACATGTTCTT-GAT----TAAGTTCCACCTTTCATGTATGGGATTCTTAGAG
SARS-CoV-2 Reference Genome Shuffle No.1 (24150) TATTACTATC-GATTATGTCTGATATACACTTAGATATCATAAGGTAGCGAACTGCCCCATGAACTGCCACGAC
```

Section 341

```
                                                          25161    25170     25180     25190     25200     25210     25220         25234
                                                  (25161)
Homo sapiens chromosome 1 NC_000001.11: 11783698-... (23962) TATGTGTTTTTAGTAAAGCCAACACTGCTGGGGTGAAACAACATGCGTGACTTTTTATCATTAAATTCTTAAAC
SARS-CoV-2 Reference Genome Shuffle No.1 (24223) -ATGTACATTCTGTAAGT--AACGGATCTGGGACGACCCTATTTAACTCCCCATTAATAGTAACCGCCTTTAAG
```

Section 342

```
                                                          25235 25240     25250     25260     25270     25280     25290            25308
                                                  (25235)
Homo sapiens chromosome 1 NC_000001.11: 11783698-... (24036) GTAATAAAGA--AGGTAGAGAAAAGCCTATAACTCCATTCATTAAAAGATGACTGTTAACACCTTACTAAGTAT
SARS-CoV-2 Reference Genome Shuffle No.1 (24294) TTTAAACTGATGACGCAGAGGTCAGTTT-TAATCGCATTGCG-----GAT-AGTGCTAGAGCCGTACAGCGTTT
```

Section 343

```
                                                          25309      25320     25330     25340     25350     25360     25370      25382
                                                  (25309)
Homo sapiens chromosome 1 NC_000001.11: 11783698-... (24108) CTTTCTACATCCTTACCCATGCTTTTATATACATGATACATGCTTTTTTTAAGTAACAAAAATGAGATTCCACT
SARS-CoV-2 Reference Genome Shuffle No.1 (24361) ATTT-TATTTATTTACCCACTTGTTTCTTTCTATTTCACAAGCGACGATT---TGACTAAACCGTGAGT-----
```

Section 344

(25383)	25383 25390 25400 25410 25420 25430 25440 25456
Homo sapiens chromosome 1 NC_000001.11: 11783698-... (24182)	AAATCATCTATTTTAACCTGTCCCACTCACCCCCTTTATTATACTTTTTGTATCATTAGGTAATTTTCCACAAC
SARS-CoV-2 Reference Genome Shuffle No.1 (24426)	---TTAT-TGTTGTTACAT---CAGCTCATTTCCTGGTTGAGACCGAGAAAGGTATTACTGTTGACGCCAAATC

Section 345

(25457)	25457 25470 25480 25490 25500 25510 25520 25530
Homo sapiens chromosome 1 NC_000001.11: 11783698-... (24256)	CTTTTCTTTTTTTTG-ACAGAGTCTCAC-TCTTCCATAAATTCTTCATGCTGTAGAATT---TAGTGTCAGCGT
SARS-CoV-2 Reference Genome Shuffle No.1 (24493)	CGTTTAGTACGTTTGTACTAAGGTTGAGGTTTTTCACTACTAA--CATACGCTAATATTGCATAATCTAACTTT

Section 346

(25531)	25531 25540 25550 25560 25570 25580 25590 25604
Homo sapiens chromosome 1 NC_000001.11: 11783698-... (24325)	TAGGAT--TTGTAGCACTTTTTTGTTTAACTTTTATTATGAAGGATTTCAACTATATGCAAAAACAGAATAATC
SARS-CoV-2 Reference Genome Shuffle No.1 (24565)	TAACTTCCCTTCAGTGGGCTAGAGCTCAA--TTTATAGTGA--GAACTCA-CTA-AGGTAAAAAGAAGAAACAC

Section 347

(25605)	25605 25610 25620 25630 25640 25650 25660 25678
Homo sapiens chromosome 1 NC_000001.11: 11783698-... (24397)	TGTTGAAACCCCTTGTCCTCATCACACAGCTTCCACAGTGATTTAGCTCAAGCCCAGTCTTCTTTTGTCT-GTG
SARS-CoV-2 Reference Genome Shuffle No.1 (24633)	GGTGCTAATC-----TAGTCTATAAAGAGGGTGTGTAGACCTTTCTGAATAGATGTGTGTACTGGGGTAAAGAA

Section 348

(25679)	25679 25690 25700 25710 25720 25730 25740 25752
Homo sapiens chromosome 1 NC_000001.11: 11783698-... (24470)	CTTTCGTTATCCACTTCTCCTTTCTCATATTATTATTTTTTATTGTATGTGTGTGTGTGTTTGTGTGTGTGTGT
SARS-CoV-2 Reference Genome Shuffle No.1 (24702)	ACTTCATTTTGAGTGTGTGGCTGA--AAATTAGCATATGGTCGA-TAAAAATGTTTATGGTTATCTACACTTCT

Section 349

(25753)	25753 25760 25770 25780 25790 25800 25810 25826
Homo sapiens chromosome 1 NC_000001.11: 11783698-... (24544)	GTGTGTGTGTGTGTGTGTGTGTGTGTGTGTTTAAAGTAAAGACAGTCTCCCTATGTTGCCCAGGCTGTTCTCG
SARS-CoV-2 Reference Genome Shuffle No.1 (24773)	AATCCTGTGTTCAACAGGTAGTCGTTATAATTGAACATACATTAAAACTAGCC--GGTACATTGGCTGAACTG-

Section 350

(25827)	25827 25840 25850 25860 25870 25880 25890 25900
Homo sapiens chromosome 1 NC_000001.11: 11783698-... (24618)	AACTCCTTGACACCAGCGATCCTCCCATGTCAGCCTCCCAAAGTTCTGGGATTACAGTCATGATCTACCATACC
SARS-CoV-2 Reference Genome Shuffle No.1 (24844)	----CGGTATAACTAATCAGTCCCCTTAATTATATTTCGAA---TAAGATATAATGGTCGT-ATTTCAGATTGT

```
                                                                      Section 351
                                                          (25901) 25901    25910     25920     25930     25940     25950     25960       25974
Homo sapiens chromosome 1 NC_000001.11: 11783698-... (24692) TGGCCGCCCTTCCCATGTTATTTTGAAAGAAATTCTGTACATCATATTATTTCACCCATAATTCTATATGCATC
SARS-CoV-2 Reference Genome Shuffle No.1 (24910) GGTAAGATAAT----TGATAAGTTGCTATAGAGTCAACGAAT-AGGCTATTAAA---ATAACTCGCAGTATATT

                                                                      Section 352
                                                          (25975) 25975 25980     25990     26000     26010     26020     26030       26048
Homo sapiens chromosome 1 NC_000001.11: 11783698-... (24766) -TCTAATGGATAAGGACTTTTAAAAAGCATAACCCAATACTATTACCACATCTAAAAAATTAGGAATTTCTTGA
SARS-CoV-2 Reference Genome Shuffle No.1 (24976) GTCTTGTGCTGTTGGGCTGGTAAATACCGTTTTTATACAGCTGGGGGACACTGGCCTTTTTAGGATCATATGG-

                                                                      Section 353
                                                          (26049) 26049      26060     26070     26080     26090     26100     26110    26122
Homo sapiens chromosome 1 NC_000001.11: 11783698-... (24839) TATCAAATATCTACTGTCCAGATTTTCCAGTTTTCTTGTAAATG-TCATATGTATTTTA-ACTTTATT--TGAA
SARS-CoV-2 Reference Genome Shuffle No.1 (25049) TAT----TATTTCTGGTTTGGATAACGCAAAATTAATACAAATAATCGGGGTCAGATTACAGTACATTCATGAA

                                                                      Section 354
                                                          (26123) 26123  26130     26140     26150     26160     26170     26180       26196
Homo sapiens chromosome 1 NC_000001.11: 11783698-... (24909) TTAGGTTCCAAAAGTCCTTTTTTATTTGTTTGATAAATCTGTTAAATCTCTTTTAATCTGTAGCTTCTTCCTTC
SARS-CoV-2 Reference Genome Shuffle No.1 (25119) ATATATAATAATAAGAGTCTAGTATATGTTGAATTA-TCGATCA---CGCCGTAAGAGTTTAACTAAATTATGA

                                                                      Section 355
                                                          (26197) 26197        26210     26220     26230     26240     26250     26260 26270
Homo sapiens chromosome 1 NC_000001.11: 11783698-... (24983) ATCTTTTTTTTTTTTTTGTAAGTTAT---TTGACAACATAATTAGGACCTGGGATTTT-----CTACTGTCTAG
SARS-CoV-2 Reference Genome Shuffle No.1 (25189) ATGAGATTGATTGGAGTGTACTGGATCGATTGCTAATA-ATTCAGCAGATACGAATGTTCAGACATCGGTATAG

                                                                      Section 356
                                                          (26271) 26271    26280     26290     26300     26310     26320     26330       26344
Homo sapiens chromosome 1 NC_000001.11: 11783698-... (25049) ATTTTGCCATTTGGAGCCGTTTGTA-TAGTTTAATATGCTAC---TCTGATGGGACT-GCTTTGAATGAACCAA
SARS-CoV-2 Reference Genome Shuffle No.1 (25262) ACAATTA-ATTACTATACCTAACTAGTAGTGTAATCTGCCGCAGATAACAAGAGTGTTGCCTTGAGTTTTTCTT

                                                                      Section 357
                                                          (26345) 26345 26350     26360     26370     26380     26390     26400       26418
Homo sapiens chromosome 1 NC_000001.11: 11783698-... (25118) AAAGCACTGTATCCACTGCTGTGTTTGTTCTATTTCTTTTTTTTTCTTTTTTTTA-AATTTGAGATGGAGTC-
SARS-CoV-2 Reference Genome Shuffle No.1 (25335) CACGA--TGTGTCCATGACCAGATATCGTACCACTGGAGGAGTTCTCTTTCCACAATAACTCCATAGTCGGTAG
```

Section 358

```
                                                     (26419) 26419      26430     26440     26450     26460     26470     26480   26492
Homo sapiens chromosome 1 NC_000001.11: 11783698-... (25190) TCACTCTTAGTGCCCAGGTTGGAGTGCAATGGCTCGATCTCAGCTCACTGCAACCTCCACCCCACCCTAGGTTC
   SARS-CoV-2 Reference Genome Shuffle No.1 (25407) TATATCCGAGCTATCTAATTTTATCCAAGAGCCTCAATGTTAAGCGTTTGCTGACTTTAGAAACCCCGGCTATC
```

Section 359

```
                                                     (26493) 26493  26500     26510     26520     26530     26540     26550         26566
Homo sapiens chromosome 1 NC_000001.11: 11783698-... (25264) AAGCGGTTCTTCTGCCCCAGCCTCCCGAGTAGATGGGATTACAGGCGCCCACCACCATGCCCGGCTAATTTTTG
   SARS-CoV-2 Reference Genome Shuffle No.1 (25481) CA-CTATTATTATGTGCCGGTTATT----TAGATTTGGTATCGCACGGGCTTCTCTAGACCATG-TAACGTTAG
```

Section 360

```
                                                     (26567) 26567        26580     26590     26600     26610     26620     26630 26640
Homo sapiens chromosome 1 NC_000001.11: 11783698-... (25338) TATTTTTAG--TAGAGATAGGGTTTCACCATGTTGGCCAGGCTGGTCTCGAACTCCTGACCTCAGGTGATACTG
   SARS-CoV-2 Reference Genome Shuffle No.1 (25549) ACCTTACAGGATAAAAAGAGAAGTAAGCTATGTCGACAAGCGCAAAGAAGTTTTTAGCAAGTAATCTGGAATGG
```

Section 361

```
                                                     (26641) 26641    26650     26660     26670     26680     26690     26700      26714
Homo sapiens chromosome 1 NC_000001.11: 11783698-... (25410) CTTCGGTCTCCCAGAGTGCTGGGATTACAGGTATGAGCCACTATAC---CTGGCCATTTTCTTCTGTTTCGTAA
   SARS-CoV-2 Reference Genome Shuffle No.1 (25623) ATACACTCTGC---AGTGTTGTGGTGCTAGAGATCAGACTCTACACAATCAGGCGGAACTTTT-TGATTGCTAG
```

Section 362

```
                                                     (26715) 26715 26720     26730     26740     26750     26760     26770         26788
Homo sapiens chromosome 1 NC_000001.11: 11783698-... (25481) GTATTTGTTT--TCTTGTACATAGTTGA----GAAGTTTGCAGGAATTAGTATAAGATAC-TCCATATTGTTCT
   SARS-CoV-2 Reference Genome Shuffle No.1 (25693) GTACTAACTCGACCTAGTTCGTTTCTTATGTGGAAATTGCAAGGAAAGCTAATTAGTTAAATGGATAAGGATCA
```

Section 363

```
                                                     (26789) 26789      26800     26810     26820     26830     26840     26850   26862
Homo sapiens chromosome 1 NC_000001.11: 11783698-... (25548) GTCACCTTGCACTCCCTCACTGAACCCTGTTACATCATCCTGACTGTATCACCAGGACTGAGACTGTCTCACAG
   SARS-CoV-2 Reference Genome Shuffle No.1 (25767) TGGGCGTAGGATGATCATACAG-----TGGTTTAT-AT----AATTTATTCCCTTGACTCCTAAGAATTATAAA
```

Section 364

```
                                                     (26863) 26863  26870     26880     26890     26900     26910     26920         26936
Homo sapiens chromosome 1 NC_000001.11: 11783698-... (25622) CCTTCATCCTACTTCTAGGAGCTCACCAAGGCTTCC--CCATTGACACCCAGGCTCCCTCACCCCT--TCTACT
   SARS-CoV-2 Reference Genome Shuffle No.1 (25831) CC--CATCCCTCTTTAAGATTTATATGCCAGCTAGCTACCTTTGTCT----GTATCACGAGACCCTGATCTTGA
```

Homo sapiens chromosome 1 NC_000001.11; 11783698-11817823 vs. SARS-CoV-2 Shuffle No.1

Section 365

(26937) 26937 26950 26960 26970 26980 26990 27000 27010
Homo sapiens chromosome 1 NC_000001.11: 11783698-... (25692) AAATTGTAC-TTACA-TTTGTA-ATGTGCTTGCCTTCAGCTGATATGCATA-TTCAT--GTAAATTTTTGTTGT
SARS-CoV-2 Reference Genome Shuffle No.1 (25899) AATTTTTAGGTGTCAGTGTGTATACGTTACATCATACATATTAAAGATTTTGTTCCTTAGAGAATACCTGACTT

Section 366

(27011) 27011 27020 27030 27040 27050 27060 27070 27084
Homo sapiens chromosome 1 NC_000001.11: 11783698-... (25760) TCTTGT-CATTGTTTGAGACAGAGTCTCACTCTGTCGCCCAGGCTGGAATGCAGTGGTACGGTCTTGGATCACT
SARS-CoV-2 Reference Genome Shuffle No.1 (25973) TATAGCGCATATCTTAAAGTTTCTTGGTGCTCCGTCACTCTAGTTGACATG-AGTACGAAGTTTTTCGATAACC

Section 367

(27085) 27085 27090 27100 27110 27120 27130 27140 27158
Homo sapiens chromosome 1 NC_000001.11: 11783698-... (25833) GCGACCTCCACCCCCGCCTCTGAGTTCAAGCTGTTCTCCTACCT-CAGCCTCCCAAGTAGCTGGGACTACATGC
SARS-CoV-2 Reference Genome Shuffle No.1 (26046) GGGTATGTATGAACTGACGCTATAGGGGATATTTCTTTGTATATGCGGAATTGCTTGT-GCAGG---TACATGC

Section 368

(27159) 27159 27170 27180 27190 27200 27210 27220 27232
Homo sapiens chromosome 1 NC_000001.11: 11783698-... (25906) ATACACCACCATGCCTGGCT-AAGTTTTGTATTTTTAGTAGAGATGGGGTTTCACCATGTTGGTCAGGCTGGTC
SARS-CoV-2 Reference Genome Shuffle No.1 (26116) -----CAACCCTGGCTGCATTAAAGTGAATACCGATATCTGCTAGTCTCTAAGAAAAACCTGATCTAGCTGATA

Section 369

(27233) 27233 27240 27250 27260 27270 27280 27290 27306
Homo sapiens chromosome 1 NC_000001.11: 11783698-... (25979) TTGAACTCCTGACCTCAGGTGATCCACCCACCTCA----GCCTCCCAAAGTACTGGGATTACAAGCGTGAGCCA
SARS-CoV-2 Reference Genome Shuffle No.1 (26185) TGGCTTTTCAGTGGTTTTTTGACACAGTCGTTTGAACTAGGCTCGAAAGGTACC----TAACAACTGAGAGCTA

Section 370

(27307) 27307 27320 27330 27340 27350 27360 27370 27380
Homo sapiens chromosome 1 NC_000001.11: 11783698-... (26049) CCAC-GCGTGGCCATATTCATGTACATTTCATGG---GTTAATGTTCTTTTGATATTGGTCAT--TGATATTTC
SARS-CoV-2 Reference Genome Shuffle No.1 (26255) TCATTGTGAATCAATCTGTCTCATCATTGCCCGTCCAATCAATCTCCGTATGAAGTTTGTCCCCATCAAATGCG

Section 371

(27381) 27381 27390 27400 27410 27420 27430 27440 27454
Homo sapiens chromosome 1 NC_000001.11: 11783698-... (26117) TCAACCATCTTCAAGAATGAGCTTCTTGAGAGCACTTGCTTTGTGTAAGAACAAATTCAGGCACTTAAACAGAA
SARS-CoV-2 Reference Genome Shuffle No.1 (26329) TTGATCATCATCGT---TGTGTACCCAGACAGCGAGTATAATGTCTTGTCCGCAAGGCTACCATGAATTCGTGT

Section 372

(27455) 27455 27460 27470 27480 27490 27500 27510 27528
Homo sapiens chromosome 1 NC_000001.11: 11783698-... (26191) AAGTCTGGATTTT-ATTTAT-TCAATAAATAAATCAACTTGACACAAAAGTATAATAAAGAGGGGA-GTGAGT
SARS-CoV-2 Reference Genome Shuffle No.1 (26400) ATCTGCGGATCCTCATATAACTGAGAAAAGAACTGTCCTCGTAATCAAAATGA--ATGAATAGGTCAAGTAACA

Section 373

(27529) 27529 27540 27550 27560 27570 27580 27590 27602
Homo sapiens chromosome 1 NC_000001.11: 11783698-... (26262) GGGACAGGTTAAAAT--AGTTGATTTAGTGGAATCTCATTTTTGTTATTTAAAACACGT--GTTAAAAGCATGT
SARS-CoV-2 Reference Genome Shuffle No.1 (26472) GCCCCCCGTTCCGCCCAAGCGGTGTTGGTTTTTGCGAATAGTTGAATGTCGAAGCTTTTACGTTTAAGTAACGA

Section 374

(27603) 27603 27610 27620 27630 27640 27650 27660 27676
Homo sapiens chromosome 1 NC_000001.11: 11783698-... (26332) GTATAAAAACGTGGGTAAGGATGTAGAAGGATATTTAGTAAGGTATTAATAGTCATATTTTA-ATGAATGGAGT
SARS-CoV-2 Reference Genome Shuffle No.1 (26546) ATG---ACACGTGATTACGAACGGACAGGTATAA--ACTACCGTTCTAAGTGCCAGAAGAGAGATTATACTACC

Section 375

(27677) 27677 27690 27700 27710 27720 27730 27740 27750
Homo sapiens chromosome 1 NC_000001.11: 11783698-... (26405) TATAGGGTTTTCTCTACCTT-GATAGTCAGTCAACTA-GAAAACCCTTCT-GTTG-AATATCTTTCTAAAAAGT
SARS-CoV-2 Reference Genome Shuffle No.1 (26615) TAACCGAGGAACCCAGCGGACGTTTACCAATCAATGAAGAAAACCAAACTCGTAGTAATACATCTGTAATAGGT

Section 376

(27751) 27751 27760 27770 27780 27790 27800 27810 27824
Homo sapiens chromosome 1 NC_000001.11: 11783698-... (26475) -GCCCCTTCTTCCTG-TCTCAGGAAGAGTTCATTGCACATATTTGACTCTTTATTTGATAAATTTAGGGTCTTA
SARS-CoV-2 Reference Genome Shuffle No.1 (26689) TGCTGAATCTACATTATACCTTTATGATTTCTCTA-ACGAATGAAA---TTTCGCTATAAAACAAAAAGTCTTT

Section 377

(27825) 27825 27830 27840 27850 27860 27870 27880 27898
Homo sapiens chromosome 1 NC_000001.11: 11783698-... (26547) CAAAATGTTAGAATCCCATTTTGACATCCATTCTCATTATAGGTGGTTTGAAAAA---TGGTAACTTTCTGTGA
SARS-CoV-2 Reference Genome Shuffle No.1 (26759) CGCAATTTTATAA-CTCATTGCTACTA--ATCGTGTTCATAGGTTGGTTTAGATCGTCTGGTTAATAATACTGA

Section 378

(27899) 27899 27910 27920 27930 27940 27950 27960 27972
Homo sapiens chromosome 1 NC_000001.11: 11783698-... (26618) GATGTGCCATTGCAAACAGGAATGTTTCATGCAGGTCACACAGTCTATTTATATTGTACCTTTGAATTAGAAA
SARS-CoV-2 Reference Genome Shuffle No.1 (26830) -ACG---CAATGTAGATATCAATCATT--AT--AGGTAACGGACCCC-------TTATACTTTGGAAATAGACA

```
                                                                        Section 379
                                                    (27973) 27973  27980           27990           28000           28010           28020           28030             28046
Homo sapiens chromosome 1 NC_000001.11: 11783698-... (26692) GAGGCACACTCAGAACGTAAGACTTAGAA--TTAAAGGCCTCCCCACTGGCCATAGCAGCACCCCTAGCTCAC
      SARS-CoV-2 Reference Genome Shuffle No.1 (26889) CGGAATTAATAAAATCTTGTTTCA-AGAAGGTTACGAAATTCAGCGACTATTTACCTTA-------TATATTAT
                                                                        Section 380
                                                    (28047) 28047        28060           28070           28080           28090           28100           28110   28120
Homo sapiens chromosome 1 NC_000001.11: 11783698-... (26764) AACCAGTTGAAGGTGGTTTTGGTGTGAACACTAAGA-TGCCCCTTCCTGGTTGAATTGGGGCTGGTTCAGCCCC
      SARS-CoV-2 Reference Genome Shuffle No.1 (26955) GTTTCTTTAACGCAAGTTTTCACTAGAATAACAAGCGTATAATCTGCGTGTTTTCGATGAACCGATAAACTACG
                                                                        Section 381
                                                    (28121) 28121     28130           28140           28150           28160           28170           28180         28194
Homo sapiens chromosome 1 NC_000001.11: 11783698-... (26837) CACCTCCCTCCCGGCCAGG--TGCCATTTTGCTCGTAACCCCCTGTAACAGCCCTGCAAGACACAGATAACATT
      SARS-CoV-2 Reference Genome Shuffle No.1 (27029) TAGCTACGAGCAGTGCATGAATTCGTACTTCTACGTAAGCCTCTTTGAG-----TGATAGAGGCAGGCGGAATT
                                                                        Section 382
                                                    (28195) 28195 28200           28210           28220           28230           28240           28250             28268
Homo sapiens chromosome 1 NC_000001.11: 11783698-... (26909) TTATTTTTTTAAAAAGTTGTTAGTCCCCTAAACCAAAGAGCAACATATACTGCAGAAATAAAAAGTTAGTCATA
      SARS-CoV-2 Reference Genome Shuffle No.1 (27098) CTGTGTCGG-ACAACGTTATC--TCAGTTTTA--ATAGGGCAATCAATAG-GATTACCTTTCA--TTAGTACTC
                                                                        Section 383
                                                    (28269) 28269          28280           28290           28300           28310           28320           28330        28342
Homo sapiens chromosome 1 NC_000001.11: 11783698-... (26983) GCAAAGGAGAAGTAACAGTATCTGGTTTTAATAATAGCTCATATATTCCATTGGTAC-ATATTTACAAAGGGCC
      SARS-CoV-2 Reference Genome Shuffle No.1 (27164) GGTAATAATGAATGTAAATTTTATGACTTAATTAGCACTCAATTATCTCATTCAAACCACAGTTGTATAA--CA
                                                                        Section 384
                                                    (28343) 28343  28350           28360           28370           28380           28390           28400             28416
Homo sapiens chromosome 1 NC_000001.11: 11783698-... (27056) AAACTCAACTAATTTATTTAAAAAAAAAAGAAGCGTGGCACAGTGGCTCACGCCTATAT--GCTCAGCACTTTG
      SARS-CoV-2 Reference Genome Shuffle No.1 (27236) AATACCAACGAGGTTTTGGATTTGAGATCATCATGAGGAATGGTATCTACCGGCCATCTAAGATTAAATGCATT
                                                                        Section 385
                                                    (28417) 28417        28430           28440           28450           28460           28470           28480   28490
Homo sapiens chromosome 1 NC_000001.11: 11783698-... (27128) GGAGGCCAAGGCAGGAGGATCGCTTAAAGCCAGGAGTTCAAGACCAT-CCTGGGCAATAAAGTGAGACTTTGTT
      SARS-CoV-2 Reference Genome Shuffle No.1 (27310) GTACTGTTCGTATTTGTGATGAATTAAGTTAGTGTATTTAAACCCACACGTGTTCCGGTTTGTGGCCGATCCTA
```

```
                                                        Section 386
                                          (28491) 28491    28500     28510     28520     28530     28540     28550        28564
Homo sapiens chromosome 1 NC_000001.11: 11783698-... (27201) CCTACAAAAAAATTTGTTCAAAATTAGCAGGGGGCCGGGTGC-AGTGGCCCACACCTGTTACCCCCACACTTTA
  SARS-CoV-2 Reference Genome Shuffle No.1 (27384) CCTCCGTTAAAACGT----AGGATTCACGGTTTCCTTTGTTCTATTGACGGATACAGTTTGCGATGACTTGTGT

                                                        Section 387
                                          (28565) 28565 28570     28580     28590     28600     28610     28620             28638
Homo sapiens chromosome 1 NC_000001.11: 11783698-... (27274) GGAGGCTGAGGC--AGGTGGATCACTTGAGCCCAGGACTTCATACCAGCCTGGGCAACATGGCGAGACTCCATC
  SARS-CoV-2 Reference Genome Shuffle No.1 (27454) CATCATTGGGCCTTAAATGGGTGA---GAGAGCTGAATTACAACGGAAAAAGGACGACTTTGAGCATCTTGAGC

                                                        Section 388
                                          (28639) 28639      28650     28660     28670     28680     28690     28700       28712
Homo sapiens chromosome 1 NC_000001.11: 11783698-... (27346) TCAACAACAAATACGAAAAAATTAACGAGGTGTGGTGGCTAACCTGTGGTCCCCCCTACTCAGGAGATCGAAGC
  SARS-CoV-2 Reference Genome Shuffle No.1 (27525) TGTGTGGCATTTGCGCAAACAGTAC----GCGAGTAAGCGGAACCAAGATTACGTGAACTTAAG-GATTGGTAA

                                                        Section 389
                                          (28713) 28713  28720     28730     28740     28750     28760     28770           28786
Homo sapiens chromosome 1 NC_000001.11: 11783698-... (27420) AGGTGAATTGCTTGAGCCCAGGAGACGGAGGTTGCAGAGAGCTGTGATCACGCCACTGCACTCCAGCCTGGGTG
  SARS-CoV-2 Reference Genome Shuffle No.1 (27594) ACCCGAGTGACTATTG----GGTAAAGAAAGAACCGGAGACGTGACTATTCACTATTCCAAT--AGAAATAATC

                                                        Section 390
                                          (28787) 28787        28800     28810     28820     28830     28840     28850 28860
Homo sapiens chromosome 1 NC_000001.11: 11783698-... (27494) ACAGAGCGAGACCCTGACAAAAAAAAAAGGATTCTGGTGTTCT-AGCGTTGCGGTTGTT----TCTAAATGCC
  SARS-CoV-2 Reference Genome Shuffle No.1 (27662) TTTG-GCGATTATTTCAGTAAAGGCAAAGG--TTACAGTGTCCTCAGTTTAACCACTGGTGAGATCTGCATTCT

                                                        Section 391
                                          (28861) 28861    28870     28880     28890     28900     28910     28920        28934
Homo sapiens chromosome 1 NC_000001.11: 11783698-... (27563) CTTCTTAGCAGTTTTATTGCACTATAATTGG-CATACTATTCA--GTTCACCCATGTAGAGTGTACAATTCAAT
  SARS-CoV-2 Reference Genome Shuffle No.1 (27733) CATTATTTGAGTCAATAGGTACGGTTACAGGACATCTCGCTGATACTTTAAGGATTAAGACT-TATATTCTCAG

                                                        Section 392
                                          (28935) 28935 28940     28950     28960     28970     28980     28990             29008
Homo sapiens chromosome 1 NC_000001.11: 11783698-... (27634) GGG----GTTTTAAAAATTAGTATATTCACAGAGTTGTACAACCATTCCTACAGTCAATTTTTTTT--TTTTT
  SARS-CoV-2 Reference Genome Shuffle No.1 (27806) GAGATAAGTTTTAGAGGTAAGTATTATCGTTTTTTAGGTGGTAGATATATACTATATATTTTTGTTCGTCTATT
```

```
Section 393
                                                            (29009) 29009      29020     29030     29040     29050     29060     29070     29082
Homo sapiens chromosome 1 NC_000001.11: 11783698-... (27702) TGAGACGGAGTTTCGCTCTTGTTGCCCAGGCAAGAGTGCAATGTTGTGATCTCAGCTCACTGCAACCTCTGCCT
 SARS-CoV-2 Reference Genome Shuffle No.1 (27880) TCATAGTCAATGCTTCTTTTAAT-CTCAATTAA---TTCAA----GAGTTTCGAACATACTGTTAAATGTAGCG

Section 394
                                                            (29083) 29083  29090     29100     29110     29120     29130     29140         29156
Homo sapiens chromosome 1 NC_000001.11: 11783698-... (27776) CCCGGGTTCAAGTGATTCTCCTGCCTCAGCCTCCCAAGTAGCTGGGATTACAGGCGTCTGCCACCACACCCGGC
 SARS-CoV-2 Reference Genome Shuffle No.1 (27946) AATTGGT---AGTTAAAGT--TGACTCTAAGTCG-AACTCAATACTAAAAGACGCAGTGGAATTTATGCGATAT

Section 395
                                                            (29157) 29157        29170     29180     29190     29200     29210     29220  29230
Homo sapiens chromosome 1 NC_000001.11: 11783698-... (27850) TAATTTTGTATTTTTAGTAGAGACGAGGCTTCTCCATGTTGGTCAGGCTGGTCTTGAACTCCTGGCCTCGGGTG
 SARS-CoV-2 Reference Genome Shuffle No.1 (28014) TGATTTC-TCTATTTGGTTGGTAGGAAATTTCTGG-TGAACTTCAGTATATTGCTACGCAGCTGGAGAGGAAT-

Section 396
                                                            (29231) 29231    29240     29250     29260     29270     29280     29290         29304
Homo sapiens chromosome 1 NC_000001.11: 11783698-... (27924) ATCCGCCCGCCTTGGCCTCCCAAAGTGCTGGGATTACAGACGTGA---GCCACTGCGCCCAGCTAATTCCTACA
 SARS-CoV-2 Reference Genome Shuffle No.1 (28085) AGATGATATGCTTGTCGGAA-AAAGTCTATTGAAAACTGAGGTGTTTCGACAAAGTCCTCTTCAGGTTTACATA

Section 397
                                                            (29305) 29305 29310     29320     29330     29340     29350     29360           29378
Homo sapiens chromosome 1 NC_000001.11: 11783698-... (27995) GTCAATTTTAGAATATTTTCATCACCT-GAGAGGAAACCCTGCACTCATTAGCAGCCACTGC-CTGTTCTCCTT
 SARS-CoV-2 Reference Genome Shuffle No.1 (28158) TTGCACTACAACATCCATTGCTGTCATTGTGATTGGGCACGCCGCTGTTGAGTAGACTCTGTTCTCTCCTCAAT

Section 398
                                                            (29379) 29379      29390     29400     29410     29420     29430     29440     29452
Homo sapiens chromosome 1 NC_000001.11: 11783698-... (28067) GCCCCTGACTCCAGG-----CAACCAGTGATCTCCTTTCT-GTCTATTTGCCTATTCTAGAT---ATCTAAAAA
 SARS-CoV-2 Reference Genome Shuffle No.1 (28232) GGTAGTAACGTGGGCAATCTCAACACGGAAGCAAATCTATTGTGAATTCCTAAGTTCTAGAGTGGGTATCATAG

Section 399
                                                            (29453) 29453  29460     29470     29480     29490     29500     29510         29526
Homo sapiens chromosome 1 NC_000001.11: 11783698-... (28132) CCTTCTAAATGTTTACGGAACCATATCATTATTAGATGGAACCATCAATGAAGTACTTGACCAGTA-ATTTCCA
 SARS-CoV-2 Reference Genome Shuffle No.1 (28306) TCTTCGCAGAATTCCAATCTACTAATGATTAGGACAAGAAATAAGATATCATGTACGCGAGCTTCATACTGTAG
```

```
                                                                 Section 400
                                                  (29527) 29527        29540     29550     29560     29570     29580     29590   29600
Homo sapiens chromosome 1 NC_000001.11: 11783698-... (28205) TACATATTTTGGATGAAGGCAAGGGTATTGTTTCTCCTCTGCTCTCAGGCACCACTCGCGGAACACTGCTAACA
      SARS-CoV-2 Reference Genome Shuffle No.1 (28380) GAGGTATCTCTTCAAAAATCAATAGCGTCGCCTAACTT-TAC-CGGAGGTCACGCTATAACATGTTTTCTAAAA
                                                                 Section 401
                                                  (29601) 29601    29610     29620     29630     29640     29650     29660        29674
Homo sapiens chromosome 1 NC_000001.11: 11783698-... (28279) CCGATATGTGGAGGTTGTTT--CCCCACTAC----TCAGGAGGC----TGAGGTGGGAGGATGGCTTAAGCCCA
      SARS-CoV-2 Reference Genome Shuffle No.1 (28452) ATTACTTGCCAGGGCTCGTTGGCTCCTGTGCCATTTGTGAAATCCAAATAGAGTACCAGCCAAATTTATTCACA
                                                                 Section 402
                                                  (29675) 29675 29680     29690     29700     29710     29720     29730          29748
Homo sapiens chromosome 1 NC_000001.11: 11783698-... (28343) GGA--GTTGGAGGTTGCAGTGAACCGAGATTATGC---CACTGCACTCCATGCTGGGTGACAGAGAAAGACCCC
      SARS-CoV-2 Reference Genome Shuffle No.1 (28526) ACATTGTTTGTTTTCGTAGGATAACGCGGTTCATCATGCTCAAGAGTTCTTTGAGGTTTAATTAGAGGGATGCG
                                                                 Section 403
                                                  (29749) 29749       29760     29770     29780     29790     29800     29810    29822
Homo sapiens chromosome 1 NC_000001.11: 11783698-... (28412) ATCTCAAAAAAAAAAAGTAGTAGGTTGTCACCTGCATTTCTCAGCAGCTGGCAGTGAATTGAAGG--TTCACAG
      SARS-CoV-2 Reference Genome Shuffle No.1 (28600) ATATTAAAACCATTCAGTAAAACCAGATCGCGTTACTTTT-----AGATAAC--TGAATTTCATGCTTACGCAG
                                                                 Section 404
                                                  (29823) 29823  29830     29840     29850     29860     29870     29880          29896
Homo sapiens chromosome 1 NC_000001.11: 11783698-... (28484) GACTCCCTCCTTGAGTTTG--GTTAACTTGCTAGAGCGGGTCAGAAAACACAGAAATGCTACTTACATTTGCTA
      SARS-CoV-2 Reference Genome Shuffle No.1 (28667) TGTTGGATCATTTCGTTCGCCATTAATAACTTAGTG-GTATCATCACGCACTGATGT--------CATGCGCTT
                                                                 Section 405
                                                  (29897) 29897        29910     29920     29930     29940     29950     29960   29970
Homo sapiens chromosome 1 NC_000001.11: 11783698-... (28556) GTTTATTAATAAAGGATATCATAAAGGATACAGAAGAACAGCCAGATGGAGAGATTCAAGGAACGTGGGAGGGG
      SARS-CoV-2 Reference Genome Shuffle No.1 (28732) TTGAAAAAGTAATG--TATTA-AAAGTAAAGGGAAGAGAATACAGCTTGATGTA------GAACGTGTTCGGTT
                                                                 Section 406
                                                  (29971) 29971    29980     29990     30000     30010     30020     30030        30044
Homo sapiens chromosome 1 NC_000001.11: 11783698-... (28630) GCACGGAGCTTTCATGCCCTCTCCAGGCACGCTACCCTCCAGGAACCTCCAAACGGTTCAGCTGTCTGGAAGCT
      SARS-CoV-2 Reference Genome Shuffle No.1 (28797) TCACGC-----TCGTGCGC-----AGCCGCGGGACAGATAAGTAATATTATA-----TGAGATAATTATAACCT
```

```
                                                                  Section 407
                                                       (30045) 30045 30050 30060 30070 30080 30090 30100 30118
Homo sapiens chromosome 1 NC_000001.11: 11783698-... (28704) CTCTGAACCTTGTCATTTTGGAGTTTTATGGAAGCTTTTTTATGCAGTCATGATTGATTACATCACCAGCCCTT
SARS-CoV-2 Reference Genome Shuffle No.1 (28856) TTTTGCAGAC-GACTTTGGCTCGTGATGTCCTAATTTACGTCTAAATAGACAATAGAAAAAGTGACACTGCAAA

                                                                  Section 408
                                                       (30119) 30119 30130 30140 30150 30160 30170 30180 30192
Homo sapiens chromosome 1 NC_000001.11: 11783698-... (28778) GGTGATCAGCTTAACCTTCAGCTTCGGTCTCCTCCCTGGAGGGATGGGTGTCCCACCCCGCTAATCCT--GCCT
SARS-CoV-2 Reference Genome Shuffle No.1 (28929) TATGGTCAGCAACTAATTGCACTCGGGACTAACACAACGCGCTAAGAA-GTGCTAGCTATCATAGCATTTTCTC

                                                                  Section 409
                                                       (30193) 30193 30200 30210 30220 30230 30240 30250 30266
Homo sapiens chromosome 1 NC_000001.11: 11783698-... (28850) TGGTCTTTCTGGTGACC----AGCCCCTTTTCTGAAGCCATCTAAGGGCCCCCAGCCGCCACTCATCT----CA
SARS-CoV-2 Reference Genome Shuffle No.1 (29002) GGGACATGTTTGTTACCAAAAAGGCAAAATACACGAATCACCAAATTTTTGCCGGACAATA-TAATGTTCAACA

                                                                  Section 410
                                                       (30267) 30267 30280 30290 30300 30310 30320 30330 30340
Homo sapiens chromosome 1 NC_000001.11: 11783698-... (28916) TTAGCA-AACAAAAGATACCACGGCTCAAAAAAAAAAAAAGACAAAGTATGTTTGTGTGTGTGTGTG-TGTGTG
SARS-CoV-2 Reference Genome Shuffle No.1 (29075) TTAGAAGAACAAGAGTTGCGA-GTCCCGTACGAATTCATTTTTATTCTTTTATAGATGGTACGAAAGATACAAC

                                                                  Section 411
                                                       (30341) 30341 30350 30360 30370 30380 30390 30400 30414
Homo sapiens chromosome 1 NC_000001.11: 11783698-... (28988) TGTGTGTGT-GTGTGTGTGTGTGTGTGTATTGTTGGGGCTCAGAAAAT-GATACCTGGAAATATGGTGCTTTGA
SARS-CoV-2 Reference Genome Shuffle No.1 (29148) AATAAATACAGAGTAGCTTAGCGTATAGATTCCAGGTGTTAGGAATCTTGAGCCGTGAGACTAATTAAAATCGT

                                                                  Section 412
                                                       (30415) 30415 30420 30430 30440 30450 30460 30470 30488
Homo sapiens chromosome 1 NC_000001.11: 11783698-... (29060) TATGCTGAACTCTCCACCCTCACACTTTGTCTCTCCCAAAGCACAGGATGAGGCTCTTCTCTGCCGTTCCCTTA
SARS-CoV-2 Reference Genome Shuffle No.1 (29222) CTAGTAGGACTCGGGCACCTATTCCAATACGTC-CGGAAATCAGATTATTATAC-CTAGTGGATTTAAGGTTAA

                                                                  Section 413
                                                       (30489) 30489 30500 30510 30520 30530 30540 30550 30562
Homo sapiens chromosome 1 NC_000001.11: 11783698-... (29134) TCTACCTAGAAACTGGACCTACCAGAG-AGGAACACAGTTGTCTTTGATCCCTTTCCTGAAATGTCATTAACCA
SARS-CoV-2 Reference Genome Shuffle No.1 (29294) TCTTGTTCAGATCCGTAACAACCACTGCACTCGCTCACAACGGTTGGAATTGTTTACCCAGCTGGT-TGAACCG
```

Homo sapiens chromosome 1 NC_000001.11; 11783698-11817823 vs. SARS-CoV-2 Shuffle No.1

Section 414

(30563) 30563 30570 30580 30590 30600 30610 30620 30636
Homo sapiens chromosome 1 NC_000001.11: 11783698-... (29207) GGGAACATTAAAACTCATCTTACAGAGGAGACTGAAGAC--CAAACAACATACCTAGAGCCTGGACAAACTGTC
SARS-CoV-2 Reference Genome Shuffle No.1 (29367) CGGACCCTGTATCTGAAGCTCGTGAAGTACTCAAAATCCTTCTCAGAAAAAACCTCTGATGCGGACTAGATA--

Section 415

(30637) 30637 30650 30660 30670 30680 30690 30700 30710
Homo sapiens chromosome 1 NC_000001.11: 11783698-... (29279) CCAACCGTTGTTTGTTCACTGTCCCATACAATTTCCAAAGAGAATCATTCACAAGATGATATCTGCTTCCCAGA
SARS-CoV-2 Reference Genome Shuffle No.1 (29439) --AAGGGGAGCATAT--------CTATTAAAAACCAAAAGAATATTATGTCCCAAATGATACTGGAATAGACGA

Section 416

(30711) 30711 30720 30730 30740 30750 30760 30770 30784
Homo sapiens chromosome 1 NC_000001.11: 11783698-... (29353) TCCATTCATTCCCCACTAAGAATTATTTCCTCCCACATCCCTATCTCCCCTTCCCCTGTGAAGAAGAGT-ATAG
SARS-CoV-2 Reference Genome Shuffle No.1 (29503) GATATTAGTTTTCCGGTTAGGTGCAGTTGCTAGACTATTTGTTACAAAGCTGTAGCTATAAATTCGCCTTACTT

Section 417

(30785) 30785 30790 30800 30810 30820 30830 30840 30858
Homo sapiens chromosome 1 NC_000001.11: 11783698-... (29426) AAGCGTCTGTACCTC-GCTGGCATTA---TTGGGTAATCATTCTGCAGTTCCCCTGTGCAATGCATGTTAAATA
SARS-CoV-2 Reference Genome Shuffle No.1 (29577) TATCGTCCGGACACCAGTTGACATGAGAGTTGCGTAA-CATATATTAGTTT----------------TTACGTA

Section 418

(30859) 30859 30870 30880 30890 30900 30910 30920 30932
Homo sapiens chromosome 1 NC_000001.11: 11783698-... (29496) AGTGGATGCCTTTGCTCCCATTAATCTGCCTTTTGTCGGTTCACTTTCAGCAGACCTTTAGAGAGTGAAAGGGG
SARS-CoV-2 Reference Genome Shuffle No.1 (29634) AGCATAGTCCTTTG-----ATTAATATACGATAT----------TTAATGAAGAGTATTATACATTTCATTTCT

Section 419

(30933) 30933 30940 30950 30960 30970 30980 30990 31006
Homo sapiens chromosome 1 NC_000001.11: 11783698-... (29570) AAGCTT-TTCCTCTTGGCCGCTACAATACATATGTAGGTGCATATATGTTTAATCACTTATGGATGAGTCTAGT
SARS-CoV-2 Reference Genome Shuffle No.1 (29693) AAGGTAGTTACTCTATTGC---AAACAACGCGCGTAAG---ATACCCCTCTAGTATCGTCA--ACGAATCTTCA

Section 420

(31007) 31007 31020 31030 31040 31050 31060 31070 31080
Homo sapiens chromosome 1 NC_000001.11: 11783698-... (29643) TTCTAAAAGTACTTTTTTCATCACTGTGTTTCTTTTCTGATCAGTAAAAAAGATTTTTTTTTTTTTTTTGAGAC
SARS-CoV-2 Reference Genome Shuffle No.1 (29759) TGC--ACACTGCTATCGTCACAGCGCTACCAATTTTCATATCACTGGCGATGAATCTGTATTT-------AGAA

Section 421

```
                                                        (31081) 31081    31090     31100     31110     31120     31130     31140         31154
Homo sapiens chromosome 1 NC_000001.11: 11783698-... (29717) CGAGTCTCACTCTGTCGCCTAGGCTGGAGTGCAGTAGCACAATCTCGGCTCACTGCAGCCTCTGCCTCCTGGGT
      SARS-CoV-2 Reference Genome Shuffle No.1 (29824) TATCTCAAACGCTTGCGGC-ATTCTATTGTGTGTTTAAATTAGCCAGTCTCCTAGTAGTTGCAGACTAGACGGA
```

Section 422

```
                                                        (31155) 31155 31160     31170     31180     31190     31200     31210             31228
Homo sapiens chromosome 1 NC_000001.11: 11783698-... (29791) TCAAGCGATTCTCCTGCCTCAGCCACCCTAGTAGCTGGGATTACAAGTGTGTGCCACCACACCCACCTAATTTG
      SARS-CoV-2 Reference Genome Shuffle No.1 (29897) TATTGCT-------------------------------------------------------------------
```

Section 423

```
                                                        (31229) 31229      31240     31250             31268
Homo sapiens chromosome 1 NC_000001.11: 11783698-... (29865) TGTATTTTTGGTAGAGACGGGGTTTCGCCATGTTGGCCGG
      SARS-CoV-2 Reference Genome Shuffle No.1 (29904) ----------------------------------------
```
