# Supplementary material for: Patch type nucleotide sequence identities between genomes from many different species facilitate illegitimate recombination
Source: Sci Rep. 2026 Mar 30;16:10524. doi: 10.1038/s41598-026-44124-0 (PMC13035915; doi:10.1038/s41598-026-44124-0)
Supplement: Supplementary file 15 — Supplementary Material 15 [file 41598_2026_44124_MOESM15_ESM.pdf]

Homo sapiens chromosome 13 NC_000013.11; 34882059-34911962 vs. SARS-CoV-2 Shuffle No.1

```
                                                          Section 1
                                          (1) 1        10        20        30        40        50        60            74
Homo sapiens chromosome 13 NC_000013.11: 34882059...  (1) TTTTATCCAGATTAACTTCTTCTCTTAAATGTACAGCAGTCCAGAGCTAAAAATGAAACACAA-AAAAATTGCA
      SARS-CoV-2 Reference Genome Shuffle No.1  (1) ----------------------------CGGCACTGACGACATGGCATGTAGATTAATAAGACCATAGATTTAG
                                                          Section 2
                                         (75) 75   80        90        100       110       120       130               148
Homo sapiens chromosome 13 NC_000013.11: 34882059... (74) ATTACTTTTTAAATGAAAAAATCCCTGTACTGCCATGAGATTAGTCCTTAAACCTGTAAGATTTGGGAAAGAA
      SARS-CoV-2 Reference Genome Shuffle No.1 (47) AGTAGATTCGATGCCTTTCAAGA--TGTAGCAGTATAAAATTATAAATTAA-CTTGAATGCTATGATTTTTGAA
                                                          Section 3
                                        (149) 149        160       170       180       190       200       210           222
Homo sapiens chromosome 13 NC_000013.11: 34882059... (148) ATAATTTTTTGCTCCATTACCCTAAAACATTCCAAAGGAATATTAAATTTGAATGAGAACATTTAAAAAT-ACC
      SARS-CoV-2 Reference Genome Shuffle No.1 (118) ACATTCAGTACAAGCTGGATCGGAC--CCTTCCAGTTGGGAACTATAT----ATGATCACGTTAATACCTGATT
                                                          Section 4
                                        (223) 223    230       240       250       260       270       280               296
Homo sapiens chromosome 13 NC_000013.11: 34882059... (221) ATTTAAAAAATTTTTCACTGAT-GATTAAACTGATGTTAA-ATTATTA-CACTTATTTTCTTGACAATTTATAA
      SARS-CoV-2 Reference Genome Shuffle No.1 (186) GTTCAGAAGTATTCACAGTTATCGTTTGAACGGCGCCAGACATCTTTATCGCCTAACTTTTGCACAGTCTTATA
                                                          Section 5
                                        (297) 297          310       320       330       340       350       360         370
Homo sapiens chromosome 13 NC_000013.11: 34882059... (292) AACTAACCTCTCTCTTTCAAACTCCATTATATTTTTAGTCCTGTAGAATTCCATTTGGATGTTATCTTGTTATT
      SARS-CoV-2 Reference Genome Shuffle No.1 (260) AACTAAAATACCT-TTTCGAGAAGTACTGTCGCTATCG--CTACAGAGTGTAAT---GATTTTAGACTGACTAT
                                                          Section 6
                                        (371) 371      380       390       400       410       420       430             444
Homo sapiens chromosome 13 NC_000013.11: 34882059... (366) TTTGGTCA--GTCTTTGAAGAATAACACCAACGATTA-TGCACCAGCAACCTGCCACTAAGTCACCTGCTTAAA
      SARS-CoV-2 Reference Genome Shuffle No.1 (328) TTAGTTGAACGTGGTTTGAGGTTCTTATAAACGTCGAATCCGTGGGGATATTACGCT----CTTCCCCTTAAT
                                                          Section 7
                                        (445) 445  450       460       470       480       490       500               518
Homo sapiens chromosome 13 NC_000013.11: 34882059... (437) TAAGCGAGAGGCCGTCACTACTCAATACACTCTTCTTTTAATGAGCTAATAATTAAAGCTTTATTTTGAATATA
      SARS-CoV-2 Reference Genome Shuffle No.1 (398) TAA-CACTAGGCC--CATCACTTAAAAAATGCATAAAAACAT-ACCTT-TATTTCAGGGTCTAGCTTGACG---
```

Homo sapiens chromosome 13 NC_000013.11; 34882059-34911962 vs. SARS-CoV-2 Shuffle No.1

Section 8

	(519)	519 530 540 550 560 570 580 592
Homo sapiens chromosome 13 NC_000013.11: 34882059...	(511)	AATAGTGCCATTTATACACAGAAACACACATGAAGATTATTTACTAAATCTCCATATTGGTGTTTATGAATCAA
SARS-CoV-2 Reference Genome Shuffle No.1	(464)	----GTCGTATTTATAATAATAGACT-------ATTTTCTTCTTAGAATCGCTTTTCTGCTGG----GAAGCTA

Section 9

	(593)	593 600 610 620 630 640 650 666
Homo sapiens chromosome 13 NC_000013.11: 34882059...	(585)	AATTTCTTCATTAAAATATTACCCTATTTAGTATATGAATTTTAAAAAATAAAAAATTATTTCCCAAGTCCATT
SARS-CoV-2 Reference Genome Shuffle No.1	(523)	A-TCAAGTAAGAACACAATGTCCGAATTGAAAGGATGTACTCGTACAAT-----ACTTAGACCCAAGGAGCATT

Section 10

	(667)	667 680 690 700 710 720 730 740
Homo sapiens chromosome 13 NC_000013.11: 34882059...	(659)	CTAAAACCCTCCCTTGGTTATTATAAGGTTAATATATTTAAGAATTTGTCTGAACTTATTTCCATCTCTCCTAC
SARS-CoV-2 Reference Genome Shuffle No.1	(591)	TTAAATTCCTAA-TTACATCCTA----GCTGATGTTTCTTAATCCTAAACACAAGTAAGCTCCATAAGGAAAAC

Section 11

	(741)	741 750 760 770 780 790 800 814
Homo sapiens chromosome 13 NC_000013.11: 34882059...	(733)	ACAAAGATTAAGCATGAAACAATATATCCCCAGAGTAGACTGAATTCTGTGTGCAGACTAAATAACTGTCTACA
SARS-CoV-2 Reference Genome Shuffle No.1	(660)	TAATGTTTTTTTAGTG---CTTTTTATACGT-GCCTTTA-TCAATTGGGTATACAGTATCGC-AATTGGATAAA

Section 12

	(815)	815 820 830 840 850 860 870 888
Homo sapiens chromosome 13 NC_000013.11: 34882059...	(807)	CAACTTGAT----TTTAAAGATTGTCCAACTCTCAACATTGAAATCTCTTAATACTA-TCTGAAAGTAGGGACC
SARS-CoV-2 Reference Genome Shuffle No.1	(728)	AATCTTATTGCGATTTTCCGCCTGTTAAA-TATCAATAATCGCAGCGTTTTACAATACTCTCAAAATATGGGGC

Section 13

	(889)	889 900 910 920 930 940 950 962
Homo sapiens chromosome 13 NC_000013.11: 34882059...	(876)	TTTGTACCTCCTTGCACTGTTATTGTCAACTGCTAACACCTGGCCATGGTAGATTAACAAATTAGATTTCCTAT
SARS-CoV-2 Reference Genome Shuffle No.1	(801)	TATAGTCTTTCC--CAGTTTGACGGTTGGATGCT----------CATTTTATTACGTCTTATATGAATTTGTGT

Section 14

	(963)	963 970 980 990 1000 1010 1020 1036
Homo sapiens chromosome 13 NC_000013.11: 34882059...	(950)	TAGCGTCAAAC--ACTAAAGGAACAGAACAGAGAATCAATGAGTTTCATAGAGTAGAAGACAGGCAGGAAGAAA
SARS-CoV-2 Reference Genome Shuffle No.1	(863)	TCGCGTCTTGCTGATATATTGGTATGAATTAAGAGACGGTTGTCATCCTTGCCTCTTA-AGATTCTGTGAGA--

Section 15

```
                                                  (1037) 1037         1050      1060      1070      1080      1090      1100     1110
Homo sapiens chromosome 13 NC_000013.11: 34882059... (1022) GGTCAGAATGACTGCATGG--AGCTCTGAGCAGCTAGGAAGCATCAGAAAAGCACTGGCCACTAAACTTCTGA
SARS-CoV-2 Reference Genome Shuffle No.1            (934) ---CTAAGTGCCTTTGTGCTATGCTCTTCTATACTAAATTGTTCTATATCTATACTGCTA-ACTCTCCTTCTCA
```

Section 16

```
                                                  (1111) 1111     1120      1130      1140      1150      1160      1170          1184
Homo sapiens chromosome 13 NC_000013.11: 34882059... (1094) G--AGTAGGGAAGGCACTAAATAAAAGGTAGGGAATGTCAAAAGACACTCTAAAGAGAAATCCACGAGCTTCAC
SARS-CoV-2 Reference Genome Shuffle No.1           (1004) GCTATTTACGCCTGTACGATGTACGAAGCCAAAATTATTATATAAACCTCAAAAGAG--------GCGCTGTAT
```

Section 17

```
                                                  (1185) 1185 1190      1200      1210      1220      1230      1240              1258
Homo sapiens chromosome 13 NC_000013.11: 34882059... (1166) TGAATCCAC-ATTTTAAATAGCATTATTTATATTTAGTGACATTTTCAAAATTTAATGTCAGAATCATGACGTT
SARS-CoV-2 Reference Genome Shuffle No.1           (1070) TGTCTGCTCGATATGACGTTTAAGCATTTTGATATGATTGTGTTGGAGGTTGCTCATGCGATAAA-------TT
```

Section 18

```
                                                  (1259) 1259       1270      1280      1290      1300      1310      1320        1332
Homo sapiens chromosome 13 NC_000013.11: 34882059... (1239) GAAAATAGATTCTGAAACTAAGGGGGAAAAATA--TTCCCTTAATTAAGCTCATGGAGGTAGTTGGC-TGACTT
SARS-CoV-2 Reference Genome Shuffle No.1           (1137) GTAAGCGG--TCTTTTACTTTGCAGCACGAATCACTGGTATTAAATTTGTTCATTAACTTTTTTAACCTTATAA
```

Section 19

```
                                                  (1333) 1333   1340      1350      1360      1370      1380      1390          1406
Homo sapiens chromosome 13 NC_000013.11: 34882059... (1310) TGGCAATGAAATTTAACTTATTCTTTTTTCCATTCTCTCATACGTACACTCTGCTACACCTTACTGAACAAGCT
SARS-CoV-2 Reference Genome Shuffle No.1           (1209) CTTCGTTGGTGTTG--CTAGGGGCCTAATGAAATCCACCAAACGTAATTTGTGACATTAATTTTTCAAAAAAGG
```

Section 20

```
                                                  (1407) 1407         1420      1430      1440      1450      1460      1470     1480
Homo sapiens chromosome 13 NC_000013.11: 34882059... (1384) GAAAATTCAATAAATAAATGCATT-CGTTAATGATTTAGTATAACCCGAAAGGAAAGGAAAGCTGCTGGAGCCA
SARS-CoV-2 Reference Genome Shuffle No.1           (1281) GCTCCT-CAGTATTTAATTGCATAGCGGTTTTCCATGAGTTTATCTATAACGCAC--GAATTCTAATTTT-CCA
```

Section 21

```
                                                  (1481) 1481     1490      1500      1510      1520      1530      1540          1554
Homo sapiens chromosome 13 NC_000013.11: 34882059... (1457) CTCCACAGGTCTCCCACTTTGCCAAAAGCGATTACTGTGTGTCAGGGCTGATGGCATGAACCCAGCTGTGGGAG
SARS-CoV-2 Reference Genome Shuffle No.1           (1351) GTATGTAGAAGTCCCAATATTC----AGCTGTTGCTTTTGAGACTAACTTATGTCATTGGGATAGCTATACCAA
```

Homo sapiens chromosome 13 NC_000013.11; 34882059-34911962 vs. SARS-CoV-2 Shuffle No.1

Section 22

```
                                                  (1555) 1555 1560      1570      1580      1590      1600      1610          1628
Homo sapiens chromosome 13 NC_000013.11: 34882059... (1531) GCC-AGGGATGCGGGGAGGCTGCTGCCCCTGCAGCT-GTTGCTGTGAGAGATGCTGAAGGCCTTAGAATCTGCA
          SARS-CoV-2 Reference Genome Shuffle No.1 (1421) ACATACGTGTGATGGGCTGAACGTGAACAGAAAGTTCGAAGCAGCGA-ATACGCGGGGTATGTTA-AATATG--
```

Section 23

```
                                                  (1629) 1629       1640      1650      1660      1670      1680      1690       1702
Homo sapiens chromosome 13 NC_000013.11: 34882059... (1603) CTCCCACAGTGACTGAGAAATGAGGGATTTGAATATGTTTATGTGAAGGTTCTTCCTTCTTCCCCTTCATTAT
          SARS-CoV-2 Reference Genome Shuffle No.1 (1491) -TCCGCCAGT-----ATGGATGAGGA----GACCATACTTAGTCAAATGGCCCAACGTTTT---------ATAAT
```

Section 24

```
                                                  (1703) 1703   1710      1720      1730      1740      1750      1760          1776
Homo sapiens chromosome 13 NC_000013.11: 34882059... (1677) GCGCCAAAGGATGTTCTAGTAAAACTCAAGACATCAGCTTAGAATATGTTACTGATCATAAATTAAATT-GCTC
          SARS-CoV-2 Reference Genome Shuffle No.1 (1547) GCGAAGCAATATGATCT--TTACCTT-------TTAGCTTGGTAA----TACCGACCACAGGTTGATGTAGTTC
```

Section 25

```
                                                  (1777) 1777        1790      1800      1810      1820      1830      1840     1850
Homo sapiens chromosome 13 NC_000013.11: 34882059... (1750) -ACAGAAGGTGATCAATTTTTAATGTTTACACTCAAGATGTTCTTTCTAATTAACACTTAGCTACATTTGTATT
          SARS-CoV-2 Reference Genome Shuffle No.1 (1608) TACTCCGCCGGGTCCGGTAGTAAAGATTACGTTGAAGCT--TATTGTAAACGAATA--TAGTATAGTATATGGA
```

Section 26

```
                                                  (1851) 1851     1860      1870      1880      1890      1900      1910          1924
Homo sapiens chromosome 13 NC_000013.11: 34882059... (1823) CTGTGTTCACTGT-AGAAAATGTCTGAGCTTGCA--GAGAACTGCAATAAGTTGTAAGACATCAAACTGCCATA
          SARS-CoV-2 Reference Genome Shuffle No.1 (1678) CAGAGTCCAGTTCCAACGAATACCACAGCTATCATTAAGGACTGCGGGATAATG-ACGAGTGGAAACTACTAAG
```

Section 27

```
                                                  (1925) 1925 1930      1940      1950      1960      1970      1980              1998
Homo sapiens chromosome 13 NC_000013.11: 34882059... (1894) ATTTTCAAAGTTACACTCTTCAGCTAAAGAAAAAGGATTTAGCTTCAGAGCACTGACACCATAAAGGGACCTAA
          SARS-CoV-2 Reference Genome Shuffle No.1 (1751) TTGTTCAATCTA----TCTTGATC---ACAATACGCATATCCGTGGTTAGGGCTGT----ATACGTGCACCTAA
```

Section 28

```
                                                  (1999) 1999       2010      2020      2030      2040      2050      2060       2072
Homo sapiens chromosome 13 NC_000013.11: 34882059... (1968) GTTATTCTTCGGAAGTAGTGGGAAGTTACTCTGTATGAATAGATATTTTTAGAAAGTTACTTATATTCCTGATG
          SARS-CoV-2 Reference Genome Shuffle No.1 (1814) AGGA------GAAGGCTTTCGGTTGCAACTC-GAGTGTTTTGACCTATC---ATAGCC-CACAAGTTACCGTTG
```

Homo sapiens chromosome 13 NC_000013.11; 34882059-34911962 vs. SARS-CoV-2 Shuffle No.1

```
                                                                 Section 29
                                              (2073) 2073   2080           2090           2100           2110           2120           2130           2146
Homo sapiens chromosome 13 NC_000013.11: 34882059... (2042) TGGAGGTGGAAATTACCCATCCATAATGCCTATGATGTACTGAGACATATATGTGAAAATTAAAAGTGATTTCC
       SARS-CoV-2 Reference Genome Shuffle No.1 (1877) TCGATCTGGGAAATTCGCGCAAGTTATCCCACTTGTATACAG-GATGAGCAGGTAGTCTTGCGTTATAAGTTAC
                                                                 Section 30
                                              (2147) 2147          2160           2170           2180           2190           2200           2210       2220
Homo sapiens chromosome 13 NC_000013.11: 34882059... (2116) AAATTTAGCAAAAGCTCATTAATTTAGAATCTATTGATTTTTAATTAATGCTAATTGGCCACTTGTGAGCTAT-
       SARS-CoV-2 Reference Genome Shuffle No.1 (1950) ACTATTATCT----CTCGTTA---TCCGTTAGATAGTTAGTAAGTGAATGCAGCTACTCTA-TTCTACGCTATT
                                                                 Section 31
                                              (2221) 2221     2230           2240           2250           2260           2270           2280          2294
Homo sapiens chromosome 13 NC_000013.11: 34882059... (2189) -ATAGAACTATTT----TAAAAAACAGATTATTT-ACTAGCTGTGTGACCTTGA---GAAATTGAC-CTAACTC
       SARS-CoV-2 Reference Genome Shuffle No.1 (2016) GCTTTACGTAGTTGTCATTATTTTGAGGCTATAGGAGGAGTTGTCTGGTTTGGATTCGAAACGGGCTCTTTTTT
                                                                 Section 32
                                              (2295) 2295 2300           2310           2320           2330           2340           2350             2368
Homo sapiens chromosome 13 NC_000013.11: 34882059... (2253) CTCAGATTGAAAGTGGGAGTAATATCTATATGACGGTTTCTATGAGGATTAAATAACATATGTGAAGTACATAT
       SARS-CoV-2 Reference Genome Shuffle No.1 (2090) CAAAGAATCTAGCTAGAAACGA-ATCTGTTTTCATGACAC-ATGTGGATACCCTACCTTAAGTTCTG-ATAAAC
                                                                 Section 33
                                              (2369) 2369        2380           2390           2400           2410           2420           2430        2442
Homo sapiens chromosome 13 NC_000013.11: 34882059... (2327) CAGTGCTTCACATATACTATTAGTTTCTTTCCTGTGTATATTACCATATTTACTTGCATTGTGCTTC-TTGGTA
       SARS-CoV-2 Reference Genome Shuffle No.1 (2161) AAGTG-------TATAACACCAGTATCTGACTAAAGAAATATTCCATAA--ACGCGAAGGACGATACATAGGTC
                                                                 Section 34
                                              (2443) 2443   2450           2460           2470           2480           2490           2500             2516
Homo sapiens chromosome 13 NC_000013.11: 34882059... (2400) CTTTCTCTTTAAAAATGGGAATAACAGTTGATCAGGAAATATTTATGTATCTCATTGTGGAAAATACCGCTTCT
       SARS-CoV-2 Reference Genome Shuffle No.1 (2226) CACCCAGTGGGAGCTTCAGGATGCCCTGATGTATTGAACGTCTACTCTATTTCGTTTTTCGTTCAAGTGGTCTT
                                                                 Section 35
                                              (2517) 2517          2530           2540           2550           2560           2570           2580       2590
Homo sapiens chromosome 13 NC_000013.11: 34882059... (2474) GGCA-GTGTCTGGGGTCCTGCGCC---AAGACAGGAACAATTCAGGGAAGCAGGCCAAGGACTTTGACTTACTC
       SARS-CoV-2 Reference Genome Shuffle No.1 (2300) GACATGGGTTTTGGTGGTAGCGCTTAAACGACACAACCTATTAAGGGCTATAAGCTCACGCAAGCAAAATATTC
```

Section 36

```
                                               (2591) 2591     2600      2610      2620      2630      2640      2650          2664
Homo sapiens chromosome 13 NC_000013.11: 34882059... (2544) TGCAAAGGAAAAGGTACTATATGTCAGTTATTTATGTATTTATTGGAGACAGGACCCCACTCTGCCACTCAGGC
SARS-CoV-2 Reference Genome Shuffle No.1 (2374) T--ATCGGAGCTGCCAAGACTTTTTCGTCGGGGATTCTTACAAAATCGTTTCAAGGTCTGTTGGACAGATATAT
```

Section 37

```
                                               (2665) 2665 2670      2680      2690      2700      2710      2720              2738
Homo sapiens chromosome 13 NC_000013.11: 34882059... (2618) TGGAGTGCAGTGGTGAGATCATAATTCATTGCAACCTCAAACTCCTGGGTCCAAGCAATCCTCCTTCATCAGTC
SARS-CoV-2 Reference Genome Shuffle No.1 (2446) TTGCACAAAATTTGGATTACTTACGAGGCGGAAAAATCTTTTTCTTATAAACT-GTATTCGGGGATTATTAGTA
```

Section 38

```
                                               (2739) 2739       2750      2760      2770      2780      2790      2800       2812
Homo sapiens chromosome 13 NC_000013.11: 34882059... (2692) TCCCAAGTAGCTAGGACTAGAGACATTCTCCATCAT--GACCAGCTAATTTGTTTTCCCCTTTTCATGCAGAGA
SARS-CoV-2 Reference Genome Shuffle No.1 (2519) TCTCCAGCA---AGTATTTAACGCTTATGGCATCAAACGGTTATCCACTCAGTGATT---TTTTTATGTTGGG-
```

Section 39

```
                                               (2813) 2813   2820      2830      2840      2850      2860      2870            2886
Homo sapiens chromosome 13 NC_000013.11: 34882059... (2764) CAGGCTCTCACTATGTTGCCCAAGCTGGTC-TTGAACTCTTGGCCTCAAGCTATCCTCCCACCTTGGCCTCTAG
SARS-CoV-2 Reference Genome Shuffle No.1 (2586) ---GTTCCCTATTTGTA-------CTGTTAATTGTTCACAGGGCTTAAGGTTAAACCCATAAATAACTTAGGAG
```

Section 40

```
                                               (2887) 2887         2900      2910      2920      2930      2940      2950     2960
Homo sapiens chromosome 13 NC_000013.11: 34882059... (2837) AGTTGCTGAGATTACAGGCATGAGCCACCACACCCAGACATGAGTCATTTAGAGGAAAACTAGTGTCCACAGAA
SARS-CoV-2 Reference Genome Shuffle No.1 (2650) GACTGTGGGTATGGGCGCTATGAGTATCAAGAAAGATACTAGTGTC-TTTAC----------GTGTCC-CAGTA
```

Section 41

```
                                               (2961) 2961     2970      2980      2990      3000      3010      3020          3034
Homo sapiens chromosome 13 NC_000013.11: 34882059... (2911) AACAGACATTTGCCGGACACAGTGGCTCATGCCTGTAATCCTAACACTTTGGGAG--TCCAGGCAGGAAGATCG
SARS-CoV-2 Reference Genome Shuffle No.1 (2712) AGCGGATTACTCGTTACCAGGTAGGCTCCATGTTGCACAATAAACACCCTGGAGCTATCGTTGGTTAAAAAATG
```

Section 42

```
                                               (3035) 3035 3040      3050      3060      3070      3080      3090              3108
Homo sapiens chromosome 13 NC_000013.11: 34882059... (2983) CTTGAGCTCAGGAGTTCAAGAATGGCTTGGGCAACATGGTGAGACCTCGTGTCTACAGGAAATAAAATTAGCCG
SARS-CoV-2 Reference Genome Shuffle No.1 (2786) TGGGAGGTACG---TGCAAGACTG--TTAGGAAATGTGA--AGACGGAGGGTCTAAAC-ACCGATACTACACCC
```

Section 43

```
                                                      (3109) 3109       3120           3130           3140           3150           3160           3170        3182
Homo sapiens chromosome 13 NC_000013.11: 34882059...  (3057) GGCAT-GGTGGACC--ACACCTGTAGTCCCAGCTACTCGGGAGGCTGAGGCAGGAGGATTGCTTGAGCCCAGGA
  SARS-CoV-2 Reference Genome Shuffle No.1            (2852) GTCATTGGTTGATCTGACAACTGGTGTTTCTT-TATT----AAACTAAAGCTTCGGGAA--CTTG-GCACCGGC
```

Section 44

```
                                                      (3183) 3183   3190           3200           3210           3220           3230           3240        3256
Homo sapiens chromosome 13 NC_000013.11: 34882059...  (3128) GGTAGAGGCTGCAGTGAGCTGAGATCCCTTCACTGCACTCCAGCCTGGTGAAACCCAGCCTGAG-TGACAGAGC
  SARS-CoV-2 Reference Genome Shuffle No.1            (2918) CTTTT---CTAGATTAATAATA-ATTACTTTAC-GAAGTTCTACGTCATCATATGCCTTTAAATATCGGATACC
```

Section 45

```
                                                      (3257) 3257         3270           3280           3290           3300           3310           3320   3330
Homo sapiens chromosome 13 NC_000013.11: 34882059...  (3201) GAGAGCCTGCCCCTGCACCCCAAAAAATGGAAAGAA-----AAGAAAACAGATATCCATGGAGCATGGGTACAA
  SARS-CoV-2 Reference Genome Shuffle No.1            (2987) TATGTACTGTATTTTCATTGTATTATTTCTGACGCATGTGGGCGCATCGCGACATTTTAGGAAAACAGGTATAA
```

Section 46

```
                                                      (3331) 3331     3340           3350           3360           3370           3380           3390        3404
Homo sapiens chromosome 13 NC_000013.11: 34882059...  (3270) TGGCTTTCATGGTGC---CATTGCCAATTCTATAGAAAAGAAGATTAGAAGAGTACAAATAATTGTTTAAAATA
  SARS-CoV-2 Reference Genome Shuffle No.1            (3061) TAGTTTTTATAGTTTTTTATTATTAAAACTAAATCTCATTA-ATTAGTGTTGTCAACGTGTTCGTATTTAAGT
```

Section 47

```
                                                      (3405) 3405 3410           3420           3430           3440           3450           3460           3478
Homo sapiens chromosome 13 NC_000013.11: 34882059...  (3341) TAATGGACTCAGTAAGGCGCGGCAGATCACGCCTGTAATCCCAACACTTTGGGAAGCCAAGGTGG-----GCAG
  SARS-CoV-2 Reference Genome Shuffle No.1            (3134) TCTTTGA---AGCAATGTCTATTTTATAGCTCTATTAAT---AAGGTTTTC--AAGTATTGGTGGTGATACTAG
```

Section 48

```
                                                      (3479) 3479       3490           3500           3510           3520           3530           3540        3552
Homo sapiens chromosome 13 NC_000013.11: 34882059...  (3410) ATCACCTGAGGTCAGGAGTTCGAGACCAGCCTGGCCAACATGGCAAAACCCTGTCTCTACTAAA---AATACAA
  SARS-CoV-2 Reference Genome Shuffle No.1            (3200) AATCATTCAGTTGGTGTGTTTGAGGTAAAGCTCCCCCG-ATAGTTATACAGTTTCACGTTCAAACAGAATATAA
```

Section 49

```
                                                      (3553) 3553   3560           3570           3580           3590           3600           3610        3626
Homo sapiens chromosome 13 NC_000013.11: 34882059...  (3481) AAATTAGCCGAGCATGGTGGCA-------------CAAGCCTGTAATCC-CAGCTACTCGGGAGGCTGAGGTATG
  SARS-CoV-2 Reference Genome Shuffle No.1            (3273) TCGTGACTTTCTAATGGAAGCAGTTATAACAATTCAACCAATTAAACCACAATAACTGCATAATGTCCAGTGAG
```

Homo sapiens chromosome 13 NC_000013.11; 34882059-34911962 vs. SARS-CoV-2 Shuffle No.1

Section 50

```
                                                  (3627) 3627         3640      3650      3660      3670      3680      3690     3700
Homo sapiens chromosome 13 NC_000013.11: 34882059... (3542) AGAATCACTTGAACCCAGGAGGCAGAGGTTACAGTGAGT--GGAGATCACACCACTGCACTCCAGCCTGG--GC
      SARS-CoV-2 Reference Genome Shuffle No.1 (3347) ACAAA-ACTACCAGCAAGCGGATATTTATTAGAGCTCTTAAGGACATAAAATGCCGGTTTAATAGTATAGTTGT
```

Section 51

```
                                                  (3701) 3701     3710      3720      3730      3740      3750      3760          3774
Homo sapiens chromosome 13 NC_000013.11: 34882059... (3612) AATGGAGTAAGACTTCATTGAAAGAAAGAGAGAGAGAGAGGAAGGAAGGAGGGAGGGAGGGAGGGAGGGAA
      SARS-CoV-2 Reference Genome Shuffle No.1 (3420) GATCTACGCCCCCTTATGTTTCCTCACTCGACTGC-AGAACGGAAATAGCGTAGGTCGGGCATCCTGAAATGCC
```

Section 52

```
                                                  (3775) 3775 3780      3790      3800      3810      3820      3830              3848
Homo sapiens chromosome 13 NC_000013.11: 34882059... (3686) GGGAAGGAAGGGGAAAAGAAAAAAGAAAGAAAGAGAAAGAAAGAAAGAAAGAAGAAAGAAAGAGAAAGAAA
      SARS-CoV-2 Reference Genome Shuffle No.1 (3493) ATTCCGCACCCTTAGTGGATCGAATAGGCTTTTTCAGATTCATATATAAAGAGTGAAT-ATTTACAGGCC-AAA
```

Section 53

```
                                                  (3849) 3849        3860      3870      3880      3890      3900      3910      3922
Homo sapiens chromosome 13 NC_000013.11: 34882059... (3760) GAAAGAAAGAAAGAAAGAAAGAAAGAGAGAGAGAGGAAGGAAGGAAGGGAAGGAAGGGGAAAAGAAAAAGAAAG
      SARS-CoV-2 Reference Genome Shuffle No.1 (3565) TCGTTTATGATTCGCCTAATTGTGTATACTGTAACTTACTTGGTATTTGGAGCGTAATTCCTAGAATATGTGTC
```

Section 54

```
                                                  (3923) 3923   3930      3940      3950      3960      3970      3980          3996
Homo sapiens chromosome 13 NC_000013.11: 34882059... (3834) AAGAAAGAGAAAAAAGGA--AAGAGAGAAAGAAAGGAAAGAAAGAAAGAAAGAGAAAGAAAGAAAGAAAGAAAG
      SARS-CoV-2 Reference Genome Shuffle No.1 (3639) ACGATGTGTCTAAGACGATTAATCGCAAAAGACAGTGTATTAATAAATTAAACCTACTTCTGGGGAAATGCTAG
```

Section 55

```
                                                  (3997) 3997         4010      4020      4030      4040      4050      4060     4070
Homo sapiens chromosome 13 NC_000013.11: 34882059... (3906) AAAGAAAGAAAGAAAGAAAGAACGAACGAAAGAAAGAAAGAAAGAAAGAACGAACGAAAGAAAGAAAGAAA-GA
      SARS-CoV-2 Reference Genome Shuffle No.1 (3713) TATCCTACATATTCAT------CTGACGAATGTTGGATTATAGTACTGCGTCTTCATTTCTTTGCGTGCCTCGC
```

Section 56

```
                                                  (4071) 4071     4080      4090      4100      4110      4120      4130          4144
Homo sapiens chromosome 13 NC_000013.11: 34882059... (3979) AAGAAAGAAAGAAAGAAAGAGAAAGGAAAGAAAAGTGAGGGAGGGAGGGAGGCAGGAAGGAAGGAAGGAAGAGA
      SARS-CoV-2 Reference Genome Shuffle No.1 (3781) AGGCCACATAGAAC---ACATAAATCCCTCAAGTCTACGGCCCACA---ATTTAG-ATGCAATTTTTGCAATGT
```

```
                                                          Section 57
                                              (4145) 4145 4150      4160      4170      4180      4190      4200              4218
Homo sapiens chromosome 13 NC_000013.11: 34882059... (4053) AGTAAATAAAATAGCATGGACTCTTCTCA---TC-CTTATATTCTCTCTAAATTGCATTTTTGTT-TTCTTTTG
   SARS-CoV-2 Reference Genome Shuffle No.1 (3848) GTTCCCTCAAAGATGACGTTCATAGGTCAGGGTCACAAATACGCCAGCTATAATGGACTCATGCACTTCATCTC
                                                          Section 58
                                              (4219) 4219       4230      4240      4250      4260      4270      4280        4292
Homo sapiens chromosome 13 NC_000013.11: 34882059... (4122) T-GTTTTTAAGGATCTGCAAGAATATCTCTAACATGGAGATTTCCAAGTTTTCTTAGTTTGATGATAGAGGAAA
   SARS-CoV-2 Reference Genome Shuffle No.1 (3922) TCGATTTCAGCTCGCTGTCTGAGAA-CACTATTTAGGAGTTCTCATA-TATACTCAAATCGTACTTAAAGTGGA
                                                          Section 59
                                              (4293) 4293   4300      4310      4320      4330      4340      4350            4366
Homo sapiens chromosome 13 NC_000013.11: 34882059... (4195) TTTCAGGCTTAGACGATGATAGT---AACAAAGGA--GAAAAATTGGCACTAGGATAGGTGGGTGG-AGCCAAA
   SARS-CoV-2 Reference Genome Shuffle No.1 (3994) TG--AGGTTTTTCCAATCGTAATTAGAACAAGTCAAGGACACATTTGGTCTATACTCCGTGGCTTGCAGCCGAG
                                                          Section 60
                                              (4367) 4367         4380      4390      4400      4410      4420      4430    4440
Homo sapiens chromosome 13 NC_000013.11: 34882059... (4263) ACAGATTTTACAGACTGATGAAAGTATGATTTTGGCAAAATGGACAGAAATTTTTGGCTCTGTATATGCTCAAA
   SARS-CoV-2 Reference Genome Shuffle No.1 (4066) AACGAT---AAGGCCCGAAAAAATTA-------------AAGGTGAGGATTTT----CCCAGAGT-TACTGATG
                                                          Section 61
                                              (4441) 4441     4450      4460      4470      4480      4490      4500          4514
Homo sapiens chromosome 13 NC_000013.11: 34882059... (4337) ATAGCTGTTGGGATGATAAAATATTAAATCATAAATGTATAATTTTTACTAGTGGTGCACTAACTTCCTAGGAA
   SARS-CoV-2 Reference Genome Shuffle No.1 (4119) GTAGTTGATA--ATAATTAAAATTTCTATGCTAC--GTCTGACGTGTCCTGAAAGTTAACTAT-----TAAGGT
                                                          Section 62
                                              (4515) 4515 4520      4530      4540      4550      4560      4570              4588
Homo sapiens chromosome 13 NC_000013.11: 34882059... (4411) GATCTCAAGACATGACTCTGAAATTAAGTATCCTAAAGTGTGTTTCTAGAGCTATAAATAACCTCCTTCTATGT
   SARS-CoV-2 Reference Genome Shuffle No.1 (4184) TATTTACAAATATCGTTACGAGA--AAACTTGGAAGAATGTTAACGTCGAATAATTGATGGAATCGCCCTATAC
                                                          Section 63
                                              (4589) 4589       4600      4610      4620      4630      4640      4650        4662
Homo sapiens chromosome 13 NC_000013.11: 34882059... (4485) GTAATTCCAATAATCCATGCAAAT-GTTGCTTTC---AGATATCCATGGAAATTTTTGGTACATGACTAA-TTA
   SARS-CoV-2 Reference Genome Shuffle No.1 (4256) C-AGTGCCACAAGCCGAGATTAATAGTTTCATTATAGTGGTGTCC-TAGCAACTTCGGGTA-ATGTGTGGCTTA
```

```
                                                     Section 64
                                             (4663)  4663  4670  4680  4690  4700  4710  4720  4736
Homo sapiens chromosome 13 NC_000013.11: 34882059... (4554) AAATTATTGTTTAAGCCCATGGCAAGTCACTGGTGCACTAAACAAGACAATGAGAGTGTGCATGATTGTCATAT
SARS-CoV-2 Reference Genome Shuffle No.1             (4327) TGATTGATATATAGTCTTATAACA--TCATTAATGCTGGTAACAA-ATTATCTATCCGTTTAT-AGCGGGATAA

                                                     Section 65
                                             (4737)  4737  4750  4760  4770  4780  4790  4800  4810
Homo sapiens chromosome 13 NC_000013.11: 34882059... (4628) TTAAACCAAAGCTGTGAGAGAGCTTCCTGGTCCACTGTTTCTGGGATATCTATTATAATTTGCCACACGTTGAA
SARS-CoV-2 Reference Genome Shuffle No.1             (4397) AGTAATTAACATTGCAAAGGAG-TTCCATGTCAA-TATCTATGTGAG-TCTGT---AATTCGATA-----TGGA

                                                     Section 66
                                             (4811)  4811  4820  4830  4840  4850  4860  4870  4884
Homo sapiens chromosome 13 NC_000013.11: 34882059... (4702) CTATAAAAAATGATGTGTCTTTAGCTAACTCAACTTTAATTTTC-TTGTATCTTACCCAATTTCCCAAATATTT
SARS-CoV-2 Reference Genome Shuffle No.1             (4460) CCCTA----------TGTCTTAAGCAAAAAAAAATTTCAAATTCGGTTATAGTGAAGCCGA---CCTAGCTACTA

                                                     Section 67
                                             (4885)  4885  4890  4900  4910  4920  4930  4940  4958
Homo sapiens chromosome 13 NC_000013.11: 34882059... (4775) TCTAAGTATTGCAATGTGAATAAATTTTCTAACAGTAAAACAGGATATAGTTTATGGAGTTTTATTTTCTGAA
SARS-CoV-2 Reference Genome Shuffle No.1             (4521) TGGAACTTCTCAGTTATGTGTGGGTTAACTA-CATTTCTA--GTCTAGACTATCATGCA-----AGGTTCAAAG

                                                     Section 68
                                             (4959)  4959  4970  4980  4990  5000  5010  5020  5032
Homo sapiens chromosome 13 NC_000013.11: 34882059... (4849) G-TAGATAACTCAAGAATGTAAGGAAGTAAAGCAAAAAAAGAGACTCAAGTGATTTTTTTGTTAGCCTGAT--
SARS-CoV-2 Reference Genome Shuffle No.1             (4587) GGTTGTGAGCTCCTGC-TCTGAGGATAGAAGTTATGCGACGGCTTC-CGATAGACTTTGGCGTAAGCCGAAACG

                                                     Section 69
                                             (5033)  5033  5040  5050  5060  5070  5080  5090  5106
Homo sapiens chromosome 13 NC_000013.11: 34882059... (4920) AATTAGTTTTTGTTTTCTTTTTTAATCTGAAGATCTGTGGAGGAAATGATTTTACTTTATAAATAAGAAACTTG
SARS-CoV-2 Reference Genome Shuffle No.1             (4659) AACAATACATTGTTCGCCCCTT-ATTATCAATGT-TGATAAGGTA--GACTTCCCATGAG------GCGACTTG

                                                     Section 70
                                             (5107)  5107  5120  5130  5140  5150  5160  5170  5180
Homo sapiens chromosome 13 NC_000013.11: 34882059... (4994) GAC-TTATATCAGCAAGAATTCTATTGGCTGGGCTCACACAATCTGG-ATTAACAGGGAAC--ACTCCTTGAAA
SARS-CoV-2 Reference Genome Shuffle No.1             (4723) TACCTTTTAACTG-AAGAC---------CTGAAGCCATTCAATCTGTCATTTACGTGCAATGAAGTTGTCTATA
```

Section 71

		(5181) 5181 – 5254
Homo sapiens chromosome 13 NC_000013.11: 34882059...	(5064)	CTTTTTATTCTTTTTATTTTGTTAA--ACTATTTCAGATTTACTTTTTATATGACACTT-GAGTCACTATGAGT
SARS-CoV-2 Reference Genome Shuffle No.1	(4787)	CCGAAT-TGCGTTGAAGTTTGCTCATAGCTATTCGATTTTGGCTAATCCGATTCCTGTGAGGGCGAGTTAATGA

Section 72

		(5255) 5255 – 5328
Homo sapiens chromosome 13 NC_000013.11: 34882059...	(5135)	CAAACTGGGTAGTGATATCATCTAACCTCTGGAAGGGTAAATAGTTCAAAAAATAGTAGCTCTGATCAATGCTT
SARS-CoV-2 Reference Genome Shuffle No.1	(4860)	CGTACTGGGTA-TGCTTTT-TGTAAACACTTCC-GGATACCAATTCGTCACGCCAGTTTCTGCGGACAATGTTT

Section 73

		(5329) 5329 – 5402
Homo sapiens chromosome 13 NC_000013.11: 34882059...	(5209)	CAAAATTCTGTTGCACATTAGATTCAGCAGAGTACCATTCAAAAACATATAATACCCAGGTCCCAGGTGGGT-C
SARS-CoV-2 Reference Genome Shuffle No.1	(4931)	TTAAATT-TGAGACACAATGAAGATGTCAT--TACGGATACAATATCTCGA-TACACTGACTGTAGGTCGGTTC

Section 74

		(5403) 5403 – 5476
Homo sapiens chromosome 13 NC_000013.11: 34882059...	(5282)	ATGTCTTAGTAT-TAGTTCTTTAAAAACGCTACTCAGTTG-ATTTTAATGTGCAGATAGAATTAAAAACTACTG
SARS-CoV-2 Reference Genome Shuffle No.1	(5001)	CCTTGTTTGTACCTCAGACATTAAGTGGTTTACTTATTAGTATTATAAATTTAATTCAAAAGCAAAGTATTCGG

Section 75

		(5477) 5477 – 5550
Homo sapiens chromosome 13 NC_000013.11: 34882059...	(5354)	CTTCAGATA---AAGCATTTTTTTTTTTTTACATAGAAAAGAGCACTTCAAAGGGTTATGTCCATCACAGTTCC
SARS-CoV-2 Reference Genome Shuffle No.1	(5075)	GTAGAAAGCGCTGTGCATTTTGGTCTAATAAAAGAACAAAGACC-CTAGGATGTTCATTGCTTATTTTGACTCC

Section 76

		(5551) 5551 – 5624
Homo sapiens chromosome 13 NC_000013.11: 34882059...	(5425)	CAGAA-TATGGAGAATGATCAATA-AAAGTTAGAACTTCAACTA--TTTGAATCCATCAGGT-TAAACTTTTTC
SARS-CoV-2 Reference Genome Shuffle No.1	(5148)	ATTAAGTACGGCTGGTGCTTAATCTAAACTAAGCATATCGAGAACTTTTCAATAAAAAATGCCTAATGAGTTTC

Section 77

		(5625) 5625 – 5698
Homo sapiens chromosome 13 NC_000013.11: 34882059...	(5494)	TGTAATGGGTCTGACAGT-AAATACCT---TAGGTGTAGA-AGGTTATAGGGTGCCTGTCGCAGCTACTCAAC-
SARS-CoV-2 Reference Genome Shuffle No.1	(5222)	AGGAAATCATAACAAATTTAAATATGTGTCTAGGTTGAGAGACCGTGTCATGTTTAAACGATAAATGCTCAACG

```
                                                                                   Section 78
                                           (5699) 5699      5710      5720      5730      5740      5750      5760         5772
Homo sapiens chromosome 13 NC_000013.11: 34882059... (5562) --TCTGCTGGTATTCAAT--CCTATCATTGTAGCACAAAAGCAGCCACAGACAATATGAACCAAATA--TGTCT
         SARS-CoV-2 Reference Genome Shuffle No.1 (5296) TGTCTAACTGAGTACAATAACGTATCCTCCTGCTAATATGACTCCCCCAAAGTATACTCACTTAAGACTTGTAC
                                                                                   Section 79
                                           (5773) 5773   5780      5790      5800      5810      5820      5830         5846
Homo sapiens chromosome 13 NC_000013.11: 34882059... (5630) AGCT-----GTGCTCCAA-TAAAACTTTATTTACCAAAACAGGCAACATGTTGGATT-TGGGCCACTGGTC--A
         SARS-CoV-2 Reference Genome Shuffle No.1 (5370) ACCTATTTAGTGCAAAAAAGTAGGGCTGTTTAGATAACAGTATAGAAAAGTTCGGATAATATTCTAGTGTTTTGA
                                                                                   Section 80
                                           (5847) 5847        5860      5870      5880      5890      5900      5910   5920
Homo sapiens chromosome 13 NC_000013.11: 34882059... (5695) TAGT--TTGCC-AACCCTGGCAATA--CATCACCTCTCAATTTTTGCAACTTTTTGCAATTTTATCACTATAGC
         SARS-CoV-2 Reference Genome Shuffle No.1 (5444) TAGTGATAGAATAAGGCGATCTATAATTATCACACATAAATGGTGACGCGTCGTACCCAGCCTGT-AGAATAAA
                                                                                   Section 81
                                           (5921) 5921     5930      5940      5950      5960      5970      5980         5994
Homo sapiens chromosome 13 NC_000013.11: 34882059... (5764) TTTATTTTATAGTTTAAAATATTATTTGTTTATAAAAT--ATTGCCAT-GGATTAAACCACT----AACTAGCT
         SARS-CoV-2 Reference Genome Shuffle No.1 (5517) TTAACCACATTCCTTTAAATATC--TAGACTAGAAATTTCATAGCCATTGGCGAAAACCTTTTATCAACGATCA
                                                                                   Section 82
                                           (5995) 5995 6000      6010      6020      6030      6040      6050            6068
Homo sapiens chromosome 13 NC_000013.11: 34882059... (5831) ATTATATGTGATTATT--AAAGAGTTA-----GTGTAGCCAGACACGT-TGGCTCACGGCTGT-----AATCCC
         SARS-CoV-2 Reference Genome Shuffle No.1 (5589) ATGATATTAACTTATTTAAAGGAATTATATATTCTTAGTCTGTTAAATGTGGATTCGGCGAGTAGCAAAATAGC
                                                                                   Section 83
                                           (6069) 6069      6080      6090      6100      6110      6120      6130         6142
Homo sapiens chromosome 13 NC_000013.11: 34882059... (5892) AGCACTTTAGGA-----GGC---TGAGGCGGG--TGGATCACCTGAGGTCAGGAGTTCAAGACCAGCCTGGCCA
         SARS-CoV-2 Reference Genome Shuffle No.1 (5663) ATCGGTTAAACATCATTCCCGTTTGATACGGGCTTATATCAAATCTGCAAGCAAGATGCTGAC--GTCTCGTTT
                                                                                   Section 84
                                           (6143) 6143   6150      6160      6170      6180      6190      6200         6216
Homo sapiens chromosome 13 NC_000013.11: 34882059... (5956) ACATGGTGAAACCCCATCTTTACTAAAAATACAAAAAATTAGCTGGACGTGGTGGCATGCACCTG--TAATTTCA
         SARS-CoV-2 Reference Genome Shuffle No.1 (5735) TCGGAGTCACAGTATATTGTCATTGAGTTTTCAACAGTTGGGCTGATGTTAATTCCAGTTCTAGATTAATCAGA
```

Homo sapiens chromosome 13 NC_000013.11; 34882059-34911962 vs. SARS-CoV-2 Shuffle No.1

Section 85

```
                                                    (6217) 6217         6230      6240      6250      6260      6270      6280   6290
Homo sapiens chromosome 13 NC_000013.11: 34882059... (6028) GT-TAT-TTGGGAGGCTGAGGCAGGAGAATCGCTTGAACCCAGGAGACAGAGGTTGCAGTGAGCCGAGATCACA
       SARS-CoV-2 Reference Genome Shuffle No.1 (5809) ATATATATTTCGATATAGAGACTAACTTCAGGTCTGAACATTTGTCAATACTCTTCAACTGTACCTACCTATTA
```

Section 86

```
                                                    (6291) 6291     6300      6310      6320      6330      6340      6350         6364
Homo sapiens chromosome 13 NC_000013.11: 34882059... (6100) CCACTG-CACTCTAGCCTGGGCAACAGAGCAAGACTCCATCTCAAAAAAAAATAAGTAAATAAAATTTAAAAAA
       SARS-CoV-2 Reference Genome Shuffle No.1 (5883) TCTTTCACATTCCGTCCATATGTATTGCGACGGTATTCCTCGCTCAACATTTAAATCATGCAAAATC----AGT
```

Section 87

```
                                                    (6365) 6365 6370      6380      6390      6400      6410      6420             6438
Homo sapiens chromosome 13 NC_000013.11: 34882059... (6173) TAAAGAGTTAGTATAAATAAAAATGACCAAATAATAACCTCTGCCCTTTTGATGAGATTACCATCTCTAAAGAT
       SARS-CoV-2 Reference Genome Shuffle No.1 (5953) TGTCTTGTCGCGCACCGGGTAGATGGCAGAGTAGTAG--TATACCTTCTTACATGTACTACGAGCAATACTAGA
```

Section 88

```
                                                    (6439) 6439       6450      6460      6470      6480      6490      6500       6512
Homo sapiens chromosome 13 NC_000013.11: 34882059... (6247) ACTGGTTTTTAGAGAATTTAATTTAACTTTATTTAGCCTAGCT------AATATAATTTATAAATTATACTTAC
       SARS-CoV-2 Reference Genome Shuffle No.1 (6025) AAACGAGTTGGGATCTTCGCATTCCATTCTGTATCGGATTCCTTCGGAAACAAAAATTTATGCATGATCACCAA
```

Section 89

```
                                                    (6513) 6513   6520      6530      6540      6550      6560      6570           6586
Homo sapiens chromosome 13 NC_000013.11: 34882059... (6315) TGGGATA--TGAAGTGATTAAATA-GAAATTTCCTAGTGCTACT-ACTTCTAGAGGACAAGAGCTCTTTAATTG
       SARS-CoV-2 Reference Genome Shuffle No.1 (6099) AGTAATTACTGAATGCATTAAGCATGATCATGATAAGTTAAAGTCACTAATTGAGGAATA-AGCATATAAGCAC
```

Section 90

```
                                                    (6587) 6587         6600      6610      6620      6630      6640      6650   6660
Homo sapiens chromosome 13 NC_000013.11: 34882059... (6385) TATTTCATAGAAAGTACTGTAGTCAAGAGTAGAAAATATGAAATATAC-ATAAGGGCCACTTTCCTCTCTAGGA
       SARS-CoV-2 Reference Genome Shuffle No.1 (6172) TCACTCAACGTTACTGCTTTTGTC----GTATAAGAT-TGAAAAACAGTATAAAGAGTGATTTCTAGTTCAGAC
```

Section 91

```
                                                    (6661) 6661     6670      6680      6690      6700      6710      6720         6734
Homo sapiens chromosome 13 NC_000013.11: 34882059... (6458) AAGACATTGCCAATCA--ATTATACCTGATCCTGGATGTGATCTC-CCAATACTTCTTACTATTACTCCAGGCA
       SARS-CoV-2 Reference Genome Shuffle No.1 (6241) GA-ACGTGGGCAATTTGCATCACTCCGTATGTTTGAAGCAAGGTGGCCGCTGTTGGTTCGGCTTCAAACTGTTA
```

Section 92

(6735) 6735 6740 6750 6760 6770 6780 6790 6808
Homo sapiens chromosome 13 NC_000013.11: 34882059... (6529) GTCACCACAAATCAAAGTTTGCAGACAAGATGAAATCTATGTGACATTCTTAACACAAGAGGAGATGTGTTTCC
SARS-CoV-2 Reference Genome Shuffle No.1 (6314) GTTACAGGGAGAGAAGGTCTTC-GACTAT-TATCGGGAATGTG-CATGGGTATC----GGGTAATTTTTTCGTA

Section 93

(6809) 6809 6820 6830 6840 6850 6860 6870 6882
Homo sapiens chromosome 13 NC_000013.11: 34882059... (6603) AGCTCCAACAACGTGCAGGAAATTATTCTTTGCTCCAAGGGTATAAATGCCCTTCCCACCACCACACTGAAGAG
SARS-CoV-2 Reference Genome Shuffle No.1 (6381) ATCTTTAGCCACGA-CTTCACGTTGAAATGAGAAAAGTGCTACTAAATA---TTTCGACTAGTGG--TGTAAAA

Section 94

(6883) 6883 6890 6900 6910 6920 6930 6940 6956
Homo sapiens chromosome 13 NC_000013.11: 34882059... (6677) TGTGCTGATCTTACAG--AGGAAGCATAGAATCA--CATGTTAATGTTTGTTAGAGATGCAGAAATTGCAGCA
SARS-CoV-2 Reference Genome Shuffle No.1 (6449) TGGGCTATATTTCCCGCCAGTACTTGTTGATTTATTAATGTAGTGGCCTATCATACTTAC-GAGTCTTTTTTTA

Section 95

(6957) 6957 6970 6980 6990 7000 7010 7020 7030
Homo sapiens chromosome 13 NC_000013.11: 34882059... (6747) AGTCACATCTTATCCAAAGTAGGTAGTCAGCATTTTGCATAGCATGCTTGGAAGAAAGAAAA-GATTCCCATAG
SARS-CoV-2 Reference Genome Shuffle No.1 (6522) ATTCA--TCGTGTGGAGAATACTT-GTAAGGCGTTCCGTCGGTTACTTTTCAAAATTTTTGTGACTTCAAGAG

Section 96

(7031) 7031 7040 7050 7060 7070 7080 7090 7104
Homo sapiens chromosome 13 NC_000013.11: 34882059... (6820) -AAATCAATTGATTAAAAAAAAAATCTATAGCTAAAGGCTGTAATTTAAACTTAAGCAAAAGGAAATAGTAAAA
SARS-CoV-2 Reference Genome Shuffle No.1 (6593) TAAATCATAAGTTTATGAAATCTATGAATCCTTTACAGC-GAAGATTAAAGGCACAATAATGGGTCTGTTATGG

Section 97

(7105) 7105 7110 7120 7130 7140 7150 7160 7178
Homo sapiens chromosome 13 NC_000013.11: 34882059... (6893) -ACTTTTCTATAATTTACATAAAATAAACTCTGAAGCAGAGGTTTTGGGG--CAGCTAGTATGGGAC---TAGA
SARS-CoV-2 Reference Genome Shuffle No.1 (6666) GACATCGGTATCAGATGTGCCTGGTTAAGTGTGCGTGAGAAGTGCTCGGATTCGACGCGTCTCTAACGTATATC

Section 98

(7179) 7179 7190 7200 7210 7220 7230 7240 7252
Homo sapiens chromosome 13 NC_000013.11: 34882059... (6961) GAAGTGT--GGGGCACACAAAAGGAGATTTAGATTGAACCGGAACTTTTTCCTGTCTCATTCTTCATGACTGGT
SARS-CoV-2 Reference Genome Shuffle No.1 (6740) GCCGTCTACCGTGTACAACGATGGCGGTTCCATACTATCC---ACATTAGACTGGTTAAGGGAACATTA---GC

Section 99

```
                                                      (7253) 7253   7260      7270      7280      7290      7300      7310         7326
Homo sapiens chromosome 13 NC_000013.11: 34882059...  (7033) AGCTGGCACTGG--CTTTAACCGAGAA--AT-CTCAATTCTCCTCTGGCTTCTCATCCT--CCAGTGTACTAGA
           SARS-CoV-2 Reference Genome Shuffle No.1  (6808) ACTTCGGATTAAACCATCCACGCAGAATCATGCACAATTCCTCTTGACGTTACTATCGTGCCCAGTGTAGGAAG
```

Section 100

```
                                                      (7327) 7327         7340      7350      7360      7370      7380      7390    7400
Homo sapiens chromosome 13 NC_000013.11: 34882059...  (7100) TC-----AGCTTCCTTATA-TGAGAGTGGTGT---TGGGGGACATTCCAAGAAAGCAAAGGCCTTTGAGGCCTA
           SARS-CoV-2 Reference Genome Shuffle No.1  (6882) TCTTAAAAGATGGGGTATTGTGCCCATTCTGTCCCTGAGTCCGAGGCGATTACGTGGCATGTAAAAGATGCCTA
```

Section 101

```
                                                      (7401) 7401     7410      7420      7430      7440      7450      7460         7474
Homo sapiens chromosome 13 NC_000013.11: 34882059...  (7165) GATTCCTGA-ACTTAC--ACAACATCAGTTCAATTACATAATCTATTCGAA--GCAAGATTC------AAGAG-
           SARS-CoV-2 Reference Genome Shuffle No.1  (6956) AGCACGAAAGACGTACGGTCACCAT-AGTTATCATTCCTAATATATAGTAACTGCATGTTTCTCCTGCAAAATA
```

Section 102

```
                                                      (7475) 7475 7480      7490      7500      7510      7520      7530             7548
Homo sapiens chromosome 13 NC_000013.11: 34882059...  (7227) -GTAGGAAAAGAGACTCCACCTCTTGGTAGGAG-GCACTATAAAATACTGGAGTTATGTTTTTCAATCTGCCAC
           SARS-CoV-2 Reference Genome Shuffle No.1  (7029) TTTCAAAAAGGTGATGTCAGAGTTAAACAGTTTCGCATTAGATGATAATGCTGACCCTTTAAGTAGGGTGC-AT
```

Section 103

```
                                                      (7549) 7549       7560      7570      7580      7590      7600      7610       7622
Homo sapiens chromosome 13 NC_000013.11: 34882059...  (7299) TCACAGTGGAGCTGTAGTCTTACTTAACACATTCTTCTGATTCT-TCATAAAGTTCTGAATTAAATTATTCTGA
           SARS-CoV-2 Reference Genome Shuffle No.1  (7102) TTTCAATGAATCT-TAATATCCTAGATGAGAAACAACAGGTGCTGTGGTGCACATACGAGGTTCGTGTTTGGAC
```

Section 104

```
                                                      (7623) 7623   7630      7640      7650      7660      7670      7680           7696
Homo sapiens chromosome 13 NC_000013.11: 34882059...  (7372) TTATTATGTATGGACAGC---AGGACCAAATGGGGCCTCTCTGACAACATCTTAAGATTCTGATGCTCAGAGCA
           SARS-CoV-2 Reference Genome Shuffle No.1  (7175) TTATGCCATCTAGAAAGCTTTAGCAACACATCGGTTTTACCTGAAAA-----TTATATC--GGTGCTTCCAG--
```

Section 105

```
                                                      (7697) 7697         7710      7720      7730      7740      7750      7760    7770
Homo sapiens chromosome 13 NC_000013.11: 34882059...  (7443) GCAAAAATTGTCTAATAAAAT-TCCATCAGAAAGAAGCATATTTCTGTC-TAAGGATGCTACATCTTACATATG
           SARS-CoV-2 Reference Genome Shuffle No.1  (7240) GGAGAATTTGAGCAATAGCTCCTCGAGCGGGTATAAGGGTGTTTAGGCCGTACCGATGGGGC---TAAGGAATA
```

Homo sapiens chromosome 13 NC_000013.11; 34882059-34911962 vs. SARS-CoV-2 Shuffle No.1

Section 106

```
                                                    (7771) 7771     7780            7790      7800      7810      7820      7830          7844
Homo sapiens chromosome 13 NC_000013.11: 34882059... (7515) TAAGATCTTTCATGTTTGATTTCAATCTTTCATGTTTGATTTCATA-GAATCTTAATATTAAGAAAAAGAAAGA
     SARS-CoV-2 Reference Genome Shuffle No.1 (7311) CAACTGAATTCTGGATT--TTACAAATTTGGACGTTT---TTCACACGAGGACTGATAACGAGAATATCGGC-A
```

Section 107

```
                                                    (7845) 7845 7850      7860      7870      7880      7890      7900              7918
Homo sapiens chromosome 13 NC_000013.11: 34882059... (7588) AACTTAAATATCATCCAATTCCACGTTAAGCAAAATTGCCAATTCACAAA--AAATATACAACTTTTATTTCTT
     SARS-CoV-2 Reference Genome Shuffle No.1 (7379) GCCTGAGCGACTATTCAAAACTATATTCTATTGGTATAGGGATAGAGAAGGCTAGCATCAGAGTTTTATTGCGT
```

Section 108

```
                                                    (7919) 7919       7930      7940      7950      7960      7970      7980        7992
Homo sapiens chromosome 13 NC_000013.11: 34882059... (7660) TAAAAGAATAGATACAGGCCCACGGAGGGGAATAATATTTTT-CTACAATTATGCTACCTTCTTTATTGAATTG
     SARS-CoV-2 Reference Genome Shuffle No.1 (7453) TGCTAAAATGTTTTGTTACAGACAGTC-----TCATATTGAGGCTACA-----GCGAATCTCCTCACTAACGTC
```

Section 109

```
                                                    (7993) 7993   8000      8010      8020      8030      8040      8050            8066
Homo sapiens chromosome 13 NC_000013.11: 34882059... (7733) TACAATACAGTTATTTTAGT--AGCTCAAGGTTTAATGTCTGGTTATACTTCAGGAG-AAGCAGCCAGGTGGGC
     SARS-CoV-2 Reference Genome Shuffle No.1 (7517) TA-------GTTATTCTTATCAAGCCCCAATTATAAATTA--GTAGTTCTTGACTTTTAAGCATGCA--TAATC
```

Section 110

```
                                                    (8067) 8067         8080      8090      8100      8110      8120      8130    8140
Homo sapiens chromosome 13 NC_000013.11: 34882059... (7804) AACATGACCAGTTCCTTGATTTTCCAGGTAAGAAACAGAGACAGGGAAACTAAGGGTCTTGCTCAAGGTTACAG
     SARS-CoV-2 Reference Genome Shuffle No.1 (7580) AATAT----AGTTCCAGTAGATTGGAGCT--GCTATAGATTGATTTATATGACTGATAAATTGAAATGGTACAG
```

Section 111

```
                                                    (8141) 8141     8150      8160      8170      8180      8190      8200          8214
Homo sapiens chromosome 13 NC_000013.11: 34882059... (7878) AACTAACTACTGGCAAAATTGATGCCACAAGAC--AACACCCTTGCTCCCAAAACAATGTTTCCTCATTGCTCC
     SARS-CoV-2 Reference Genome Shuffle No.1 (7648) ACTCAATGGCTACAATAAGCTA-GCAAAAATCCGTAAAAATCTTGTCCCCTTAAAATTATCAGAAAAACACGGT
```

Section 112

```
                                                    (8215) 8215 8220      8230      8240      8250      8260      8270              8288
Homo sapiens chromosome 13 NC_000013.11: 34882059... (7950) AT-TAGGACAG------TTACTCTAATTCAGGAA-AAGTCATTATTCTCTAAGCCTTACTGATCTGGTGAGACC
     SARS-CoV-2 Reference Genome Shuffle No.1 (7721) ATATAGCACGGCAGAAATGGCTCTACTTATGGCAGAAATCCCGACTTTCAAAAACTCGTTAGT---GGCACACC
```

```
                                                                                                                  Section 113
                                                  (8289) 8289       8300      8310      8320      8330      8340      8350    8362
Homo sapiens chromosome 13 NC_000013.11: 34882059... (8016) AG-AGGTCAGCAGCCTGTTTCTGGAAAG--GGCC---ACATAAAAATTACTTTAGAT--TTTGCAAGCCTGGAA
      SARS-CoV-2 Reference Genome Shuffle No.1 (7792) GGTAGGTAACTCGGATGTGAAAATAAGACCGGTCGTTTGATAAATTTTATTTTAGAAGCTGTGCAACGTTATAG
                                                                                                                  Section 114
                                                  (8363) 8363   8370      8380      8390      8400      8410      8420          8436
Homo sapiens chromosome 13 NC_000013.11: 34882059... (8082) -AGGGCCAC---ATAAAAATTACTTTAGATTTTGTAAGCCATACAGTCTCTGGTTGCCACTATGCAACCCCAGC
      SARS-CoV-2 Reference Genome Shuffle No.1 (7866) CAGTACCTCTGTATCCCAATAACGGAATATTATAATCGTTTGTCACTGTATCTTT-CTAGTCTGAA--CTTAGT
                                                                                                                  Section 115
                                                  (8437) 8437         8450      8460      8470      8480      8490      8500  8510
Homo sapiens chromosome 13 NC_000013.11: 34882059... (8152) ATTGTACCACAAAAGCAGACTGACAATACATTTTTTTAAATG-ACCATTCTGTGGTCCAATCAAATTGTTT---
      SARS-CoV-2 Reference Genome Shuffle No.1 (7937) AATG-ATAACCAGAGTTTACGGA--ATAC----TTTCAAGTGTATTTGTATGTGATAGGAGGAGATTTTTTCAA
                                                                                                                  Section 116
                                                  (8511) 8511     8520      8530      8540      8550      8560      8570      8584
Homo sapiens chromosome 13 NC_000013.11: 34882059... (8222) ---TTATAGAAACAGGCAAC--AGGCCAGGTTTGACCCACAGCCAATACTTTTCCAATACCACGCACTTGAT-T
      SARS-CoV-2 Reference Genome Shuffle No.1 (8004) GCTTTGTAGAGTTAGAAAAACTAATCCGGGGCTCAGGATCGGAATCTTCGGGCAAGATAACACACTGTTTATAT
                                                                                                                  Section 117
                                                  (8585) 8585 8590      8600      8610      8620      8630      8640          8658
Homo sapiens chromosome 13 NC_000013.11: 34882059... (8290) AAAGTCTAAACTATTAAGACCCCAATTCCTCATCCCTTTATGCATTCACATCCTTTGCCATGTCATCATTATGA
      SARS-CoV-2 Reference Genome Shuffle No.1 (8078) CCAGTTATATCTAGGTTGGTCGAACTTCAGTAGATTAAAACGAAT----ATTTTTTGT-ATGT--TGAAAGACA
                                                                                                                  Section 118
                                                  (8659) 8659       8670      8680      8690      8700      8710      8720    8732
Homo sapiens chromosome 13 NC_000013.11: 34882059... (8364) GTTCAGTTTGTAAGTCCTTTATTTTGGGCTTGACCCTTTATTATGGGTCAAATTGTTTCCCCCCAGAATTCATA
      SARS-CoV-2 Reference Genome Shuffle No.1 (8145) GTTT-GATTGAAAGTATTCTGCTACACAGTT-ACAGTTTG--AGCAGTAAAACTACATTTGAAAACAATTATTT
                                                                                                                  Section 119
                                                  (8733) 8733   8740      8750      8760      8770      8780      8790          8806
Homo sapiens chromosome 13 NC_000013.11: 34882059... (8438) TGT-CGAAGTCCGAA-CTCCCAGTACCTCAGAATATGACTGTATGTGGACATAGGGTCTTTATAGAGGTAATCA
      SARS-CoV-2 Reference Genome Shuffle No.1 (8215) TGTACCCTTTCCTAGTCTGCATGTATGTCATTCCAAGAAGGTTG--GAGTACGGGATTTTA-----GTCATTT
```

Section 120

```
                                                       8807         8820      8830      8840      8850      8860      8870    8880
                                               (8807)
Homo sapiens chromosome 13 NC_000013.11: 34882059... (8510) GCTAAAATGAGGTTATCAAGATAGGCCCAATCCAA--TCTGACTGGGTCCTTGTAAAAAGGAGAAAT--TTGG
   SARS-CoV-2 Reference Genome Shuffle No.1 (8282) GTTAAG-TGTTTTGTACCCGTCGGCACTTATTCAAATTTTAAATGCACCATTTGTTTATTCTCGCAAGCAATCG
```

Section 121

```
                                                       8881     8890      8900      8910      8920      8930      8940          8954
                                               (8881)
Homo sapiens chromosome 13 NC_000013.11: 34882059... (8580) AAACAGACACGCATACAGGGAAAACACCACGTGAACATGAACACAGCCATCTCCAAGGCAAGAGAGGGACCTGG
   SARS-CoV-2 Reference Genome Shuffle No.1 (8355) AGTCTGTTTTCCTAAGATTGAGTTATCCA-GTGAAGATAATAAATTATCTTTGCGTTATCGAAGCGAAACAAGC
```

Section 122

```
                                                       8955 8960      8970      8980      8990      9000      9010            9028
                                               (8955)
Homo sapiens chromosome 13 NC_000013.11: 34882059... (8654) AACAGATCCTTCCC-----TCACAGCCTTCAGAAGGAACCAAACCTGTCAACCTCTTGGTCTTGGACTTTTAGT
   SARS-CoV-2 Reference Genome Shuffle No.1 (8428) ATAATATCTATCGCAATGATCACACAGGATTGTGGGGCCTAAC--GTCACTTGACTGGTCATAGACGTTTAAA
```

Section 123

```
                                                       9029       9040      9050      9060      9070      9080      9090      9102
                                               (9029)
Homo sapiens chromosome 13 NC_000013.11: 34882059... (8723) CTCCAGAGCTGCGAGATAATAAATTTCTGTTGTTGAAGCCACCCAGTCTGTAGACATTTTTTAGGACAGCCATA
   SARS-CoV-2 Reference Genome Shuffle No.1 (8500) C-CGATTACAGTTGGAATTTTTTTTTCTATACCCCGTGTATATTTTGCTGTTTTAAAATTTGCGCTTTTT-ATC
```

Section 124

```
                                                       9103   9110      9120      9130      9140      9150      9160          9176
                                               (9103)
Homo sapiens chromosome 13 NC_000013.11: 34882059... (8797) GGACACTAATATACCATGTGACTTGTTGTGCCCAGTGATGCTGGGCTAGAAGTGGCAAATGCCAGTCTGTAGAC
   SARS-CoV-2 Reference Genome Shuffle No.1 (8572) CGAGACGTGTAGA--AACTGATTT--TATAGCTAGAGTTGATAGACACGC-GTGGAATTGGTTCGTCAGTGCTT
```

Section 125

```
                                                       9177         9190      9200      9210      9220      9230      9240    9250
                                               (9177)
Homo sapiens chromosome 13 NC_000013.11: 34882059... (8871) TAGGT--CTTAAAAAACTTTGAGTTTT-TCCACTTGCTGTCTCACCCACCTTCC--ATTGCTATGAGAAAATTA
   SARS-CoV-2 Reference Genome Shuffle No.1 (8641) TCAGTTTCTTGATTGATAATTCGTATTCTGCCCTTGTTATCCGACTTATGTATATAATAGCCCTCATAACAGGA
```

Section 126

```
                                                       9251     9260      9270      9280      9290      9300      9310          9324
                                               (9251)
Homo sapiens chromosome 13 NC_000013.11: 34882059... (8940) TGCCCTAAT-GCTATTCTGCTGGTCTAAGAAGCATGAGACACATTTGAACCAGAGCCATTCCAGCCAATCCCTA
   SARS-CoV-2 Reference Genome Shuffle No.1 (8715) GCCTCTTTTCGCCTTTTATACCGTCTTCCATCTAAGGGCCAGGCCTTTACTGATGTATGTTGAGTCTTGTCGTG
```

Section 127

		9325 – 9398
	(9325)	9325 9330 9340 9350 9360 9370 9380 9398
Homo sapiens chromosome 13 NC_000013.11: 34882059...	(9013)	CCT--CCGGATGACCTGCAGATATACCAGCCATAATAAATTATTTCTGTAAAGCCACTGAGTTTTAGAATAGAT
SARS-CoV-2 Reference Genome Shuffle No.1	(8789)	GTTAGCCGGATGGGTGGATGATTTTACG--CATAAAGGAAAATTCGCGTTATACATGTC---TTTCGGTTCACC

Section 128

		9399 – 9472
	(9399)	9399 9410 9420 9430 9440 9450 9460 9472
Homo sapiens chromosome 13 NC_000013.11: 34882059...	(9085)	TACTACATAGCAACAACTAATTGATTTAAATGTTAAATTTAACATTAGTTAAATTCCCAAAACACAGCTGTTTA
SARS-CoV-2 Reference Genome Shuffle No.1	(8858)	GACTAAATT---ATAAAGTTTTGAC---AGGGGTAGATTAAAAGCTAACCAATATTATACTATAGCGGAGATGA

Section 129

		9473 – 9546
	(9473)	9473 9480 9490 9500 9510 9520 9530 9546
Homo sapiens chromosome 13 NC_000013.11: 34882059...	(9159)	CATTCAATATAGAACTATGACTTTTTTCTGCATGTGTTTTGCCTTTTCCACTGAAAATTTAAGTCAATTCCTAA
SARS-CoV-2 Reference Genome Shuffle No.1	(8926)	CGGAGGAGCTCAGAGTCTT--TAATTTATACAATTATTTGAGAATATTTAAAGAAAGTAGCGGAGGCGTCGTGG

Section 130

		9547 – 9620
	(9547)	9547 9560 9570 9580 9590 9600 9610 9620
Homo sapiens chromosome 13 NC_000013.11: 34882059...	(9233)	ACACG-TGGTTTGCTATCCACAAG-CCTTCTCTCTTCCTTTTTTCCTTCCAGTGAA---AATAGTGATATGAAC
SARS-CoV-2 Reference Genome Shuffle No.1	(8998)	GTAGAATTTTTTACAGTTCGAAAGACGCTTTCAATTACTAGATTTAATATATTGAATCGATCACTTCTAAGAGT

Section 131

		9621 – 9694
	(9621)	9621 9630 9640 9650 9660 9670 9680 9694
Homo sapiens chromosome 13 NC_000013.11: 34882059...	(9302)	TGATGAATAAATGAAGTCGGCTTGCTCGTCTTCTTCTAACTTAGGCCGGCAAAATTGCTATGTAATCCCACTCT
SARS-CoV-2 Reference Genome Shuffle No.1	(9072)	CGAAATACTCAATAAATACTATTGAGAGTGAATTGTGATATTAT--CATTATACGTAATTAGTAATCCAT---T

Section 132

		9695 – 9768
	(9695)	9695 9700 9710 9720 9730 9740 9750 9768
Homo sapiens chromosome 13 NC_000013.11: 34882059...	(9376)	TTTCCACAGTTGCAAAAATGTGCTCCGTTTTGCTTTCAGCAGCCTCGTTTCATCAGTCAGACTATTTCTTAAGA
SARS-CoV-2 Reference Genome Shuffle No.1	(9141)	GACGCGAAATTGCAAACATGTA-TCAGTTTGA--TACGACAAACTCGTGTCTCTTTAGATAC-ATATGGTAATC

Section 133

		9769 – 9842
	(9769)	9769 9780 9790 9800 9810 9820 9830 9842
Homo sapiens chromosome 13 NC_000013.11: 34882059...	(9450)	GATGTATCTATAGAGGG----ATAGTTTGCTGTTGTTGTTATTGTTGTTAATTAAA--TAATGCGATCCATTTT
SARS-CoV-2 Reference Genome Shuffle No.1	(9211)	GCGGT-TCTGTCGTTGACACTATACAAACATGTAACTGTTACTTTGCACAACTTAACCTGAAATTACGGTATAT

Section 134

	(9843)	9843 … 9916
Homo sapiens chromosome 13 NC_000013.11: 34882059...	(9518)	TAAACAC----ACAGTTGTA-AA--ATACCCTTTGAACAGAC----ACACAGTTTGTTTGTTGGTTTGTTTGTT
SARS-CoV-2 Reference Genome Shuffle No.1	(9284)	TAATCGCGTGTATAAGTGTACACTGATTCTTATCTAACATAGGGTTAAACGGAATGTTCAATTGAGAATTACTG

Section 135

	(9917)	9917 … 9990
Homo sapiens chromosome 13 NC_000013.11: 34882059...	(9581)	TGTTTGTTTGTTTGTGACAGGGTCTCTCTCTGTTGCCTAGGCTGGAGTCAGTGATCATAC-CTCACTGCAGCAT
SARS-CoV-2 Reference Genome Shuffle No.1	(9358)	GACTGAATTTTTTCTGAGGATGT-TAACCATCTTGTCTAGAAAGGT-TCAGAATCGGTAATCTCGAAGTGGGAT

Section 136

	(9991)	9991 … 10064
Homo sapiens chromosome 13 NC_000013.11: 34882059...	(9654)	TCAGCTCCTGGGCTCAAGTGATCCTCCCACCCCAGTCTCTGAGTAGC---TAGGACTACAAGCACGTACCACCA
SARS-CoV-2 Reference Genome Shuffle No.1	(9430)	CTTGCTA-TGAG-TCAACCGGGCCTAGTAAACGATTGACTTATTTGAATATTGAATTAGTCATGATTTTGATAT

Section 137

	(10065)	10065 … 10138
Homo sapiens chromosome 13 NC_000013.11: 34882059...	(9725)	TGCTCAGCTAATTAAAAAAAAAAAAAAAATTGT-AGAGACAGGGTCTCTCTAT-GTTGCCTAGGCTAGTCTTGA
SARS-CoV-2 Reference Genome Shuffle No.1	(9502)	TTTTATTCTAGCTCATGAGTCTCATAATATTGTTAGCTAAGACGAATCGAAATCGCGGCTTAGGGTCGGCTTCT

Section 138

	(10139)	10139 … 10212
Homo sapiens chromosome 13 NC_000013.11: 34882059...	(9797)	ACTCCTGGCCTTAAAACAATCCTCTAGCCTCTGAAAGTGCTTGGATTGCAGGCATGAGCCACCACTCCCAGCCA
SARS-CoV-2 Reference Genome Shuffle No.1	(9576)	GT--CTGAGTATGGAAAAGTTAGCTACAATTCTAATATCTCTATTTTAAGATCTAGAGGAAGCCCATTCTGGTG

Section 139

	(10213)	10213 … 10286
Homo sapiens chromosome 13 NC_000013.11: 34882059...	(9871)	TACACATAGATTCTTAACTTGTTGGTACTTTTCACAAATTACCTAACATTTTGGTCTTAGTTTTTGATTGGCAG
SARS-CoV-2 Reference Genome Shuffle No.1	(9648)	T---CATGGTAATTGAATTCCCATGGTCTATTTA----TTGCGCAGCCGTTCAGTCTCGATGTTTTCTTT--AG

Section 140

	(10287)	10287 … 10360
Homo sapiens chromosome 13 NC_000013.11: 34882059...	(9945)	AATGAAATTAGACTAATTGGTGTGATGTATGGTGGGGGCCTTGAGCCATGATGTGTCAGTTTGACCTCAGGAGG
SARS-CoV-2 Reference Genome Shuffle No.1	(9713)	ATCAAAAGGGGGCTTAGTT-TATTCTGCCTTCTTAGCG-----AGAAAACGAGTAGGAAATTTTATTAATTTAG

Section 141

```
                                                  (10361) 10361    10370     10380     10390     10400     10410     10420        10434
Homo sapiens chromosome 13 NC_000013.11: 34882059... (10019) AACTGGAAACCGAGTAACAAAGGTCAGTCACACCATGAACTTCATGCCTACATGTCCCAA----TTAAAACC--
SARS-CoV-2 Reference Genome Shuffle No.1              (9781) AACATACATTTGACTACCTTTACTAATTCAGAATACTCGACTGTTACTTTTACGTACCAAAATATTATGGCCGC
```

Section 142

```
                                                  (10435) 10435 10440     10450     10460     10470     10480     10490            10508
Homo sapiens chromosome 13 NC_000013.11: 34882059... (10087) -CTGGACTGGC----TGGGTATGGTGGTTCATGCCTGTAATCCCAGCACTTTGGGATGCTGAGGCGGGCAGAT
SARS-CoV-2 Reference Genome Shuffle No.1              (9855) ACTGGAGCAGTCGTTTTGGGTAAGATGTAGAATTAAA-TGATTCAATACCTTTGCTCCACCTTTGCAGG--GTT
```

Section 143

```
                                                  (10509) 10509      10520     10530     10540     10550     10560     10570      10582
Homo sapiens chromosome 13 NC_000013.11: 34882059... (10156) CATGAGATCAGGAAATAGAGACCACCCTGTCTAATACAATGAAACCTCATCTCTATTAAAAATAAAAATACAAA
SARS-CoV-2 Reference Genome Shuffle No.1              (9926) CGGCCAAAGATGTTCGTTAAAATTTCGGGT-TCTTTCCTGGAAACATAATTTTAAT---AAGTAGACATGTCAA
```

Section 144

```
                                                  (10583) 10583  10590     10600     10610     10620     10630     10640          10656
Homo sapiens chromosome 13 NC_000013.11: 34882059... (10230) AAATTAGCCAAGCATGGTGGCACGTGCCTGTAGTCCCAGC--TACTCAGGAGGC-TGAAGTAGGAGAATC-TCT
SARS-CoV-2 Reference Genome Shuffle No.1              (9996) ACTTTGCGATAGGGCCTTAACTTTTAAAAG-ATTACAAGCGGAAGGCAGTTGCCATTTTGTAGAGTAAGCATAA
```

Section 145

```
                                                  (10657) 10657       10670     10680     10690     10700     10710     10720   10730
Homo sapiens chromosome 13 NC_000013.11: 34882059... (10300) TGAA------CCTGGAGACGGAGCTTGCAGTGAGCCGAGATTGCACCACTGCACTCCAGCCTGGCAACAGAA
SARS-CoV-2 Reference Genome Shuffle No.1             (10069) GAAAACAAGACCAGAGCGCTATAACTGATATTGCGGTGTGCTTTCTCAAATGCA-TCAGTGTTGCACATCATAA
```

Section 146

```
                                                  (10731) 10731    10740     10750     10760     10770     10780     10790        10804
Homo sapiens chromosome 13 NC_000013.11: 34882059... (10368) TGAGATTCCGTCTCAAAAAAAAACAAAAAACCCTGGAACCAAAGTTTGGGTGAACCTCTCATTTGGCAATACT
SARS-CoV-2 Reference Genome Shuffle No.1             (10142) -GAGAG----------ATATTAAACGTGAACACTCTACTAGCCGAGTTCAGGAG--CGAACCATGGGGGAAT---
```

Section 147

```
                                                  (10805) 10805 10810     10820     10830     10840     10850     10860            10878
Homo sapiens chromosome 13 NC_000013.11: 34882059... (10442) CTGTGCTGTCACACATCATTGCTGGAAAAATTCAGTACTGCTCATATGACTTCACAGGGAAGGGACAGCTGGAA
SARS-CoV-2 Reference Genome Shuffle No.1             (10201) ----GTATTAACTCCTTATTGGGGTTAATGTTTTATATATGTCTACAGCCAT-ACATGTTAGTGCCATTGTGCG
```

Homo sapiens chromosome 13 NC_000013.11; 34882059-34911962 vs. SARS-CoV-2 Shuffle No.1

Section 148

```
                                                              (10879) 10879      10890      10900      10910      10920      10930      10940      10952
Homo sapiens chromosome 13 NC_000013.11: 34882059... (10516) ACTTGAACTTGTTCTTTCCTGGTG-ACTTTAACTTG---TACCCTTTTGCTGTAATAAACCATAACTGTGAGTA
     SARS-CoV-2 Reference Genome Shuffle No.1 (10270) TATTCCATATTCCCAACATTGTCGTAATGGATCTCGCCCTAGACTACGCCCGCATTAAACCCATA--------TA
```

Section 149

```
                                                              (10953) 10953  10960      10970      10980      10990      11000      11010      11026
Homo sapiens chromosome 13 NC_000013.11: 34882059... (10586) TAACAGCTTTTGCCAGTTCTGTGAGCCCTTCTAGCAAGTCATTGAAACTGAGGGTGTTCTTGGGGACTCCCAAA
     SARS-CoV-2 Reference Genome Shuffle No.1 (10337) AAACATATTTTATTATTTGTTTCAAACGTACCCTTTAGTGATTA-----GCGGGATTTAGTTGGTTATGTCAAA
```

Section 150

```
                                                              (11027) 11027         11040      11050      11060      11070      11080      11090  11100
Homo sapiens chromosome 13 NC_000013.11: 34882059... (10660) CACATAACTATGCAAACATACATGAAAAGAAAATA--TAAGATTGTGATTAATAACTTCTCCATTTG-TTATA-
     SARS-CoV-2 Reference Genome Shuffle No.1 (10406) CTAGTAAG------AAGTTA---GTACCCAAATTCCCTAGGCATGTCGCTATCTACAAAGCGATTTGGTTATAG
```

Section 151

```
                                                              (11101) 11101    11110      11120      11130      11140      11150      11160      11174
Homo sapiens chromosome 13 NC_000013.11: 34882059... (10730) --TGTGTAATCACTGTACCCTGCCCCACTGTATGCAAAGACTGTGACATGTGTGCAAAGACTCCCA-------A
     SARS-CoV-2 Reference Genome Shuffle No.1 (10471) ATTGTATCGTTTCTCGATGCGGTCTGAAATTATTCTAAAACTTTG---TTCGTGTAAAAATTAAAATTGGTGGA
```

Section 152

```
                                                              (11175) 11175 11180      11190      11200      11210      11220      11230        11248
Homo sapiens chromosome 13 NC_000013.11: 34882059... (10795) GCCCATGTGTAAGTGAGGAAGCCACTGAAGCTCGTGAGAGCCTAAGTTAGGACT-TACCATTTGA----TAAAA
     SARS-CoV-2 Reference Genome Shuffle No.1 (10542) TCAAATTAGTAGCAACGTAAA--ACGGAATTTAATAATTATTTAGGTTTGCACTGTAATCTTTGTCCGTTAAGA
```

Section 153

```
                                                              (11249) 11249      11260      11270      11280      11290      11300      11310      11322
Homo sapiens chromosome 13 NC_000013.11: 34882059... (10864) TGATGTCAACATTACCATGGCTTCCC----CAGTCCTTAGAGGATG------TGGATCACTCTTTAACCAGTC
     SARS-CoV-2 Reference Genome Shuffle No.1 (10614) TATATACGACAGTG--ATCGGAATCGGTGATGAGGGCGTTGAGGATGGATTATGGGATCA---TTTCAAGAGAA
```

Section 154

```
                                                              (11323) 11323  11330      11340      11350      11360      11370      11380      11396
Homo sapiens chromosome 13 NC_000013.11: 34882059... (10928) GCACGGAAAACTTGCCCTCCATGGTCTTGCAAGACCTAA-GAACAAGGGTTAGCAATTCCCAGTAAACC--ATT
     SARS-CoV-2 Reference Genome Shuffle No.1 (10683) GCA-GGTATACGCTAGATGGATGATGGAGCACGACAGAATGTAGTACTTTCTACTATGCACCTAAATTCCAAGT
```

Section 155

(11397) 11397 11410 11420 11430 11440 11450 11460 11470
Homo sapiens chromosome 13 NC_000013.11: 34882059... (10999) TGCTATTTATGAGATCCTGG--GCAAATTCCATGAACTCTCTGAACCTTAGGTTCCTGGTTTTAAATAGGAAAG
SARS-CoV-2 Reference Genome Shuffle No.1 (10756) TCGTATAGATGACCTGCAAATACCATCTTAGACGACCGTTGTTCAAGTGAACGTGGTTGGTTTATACTG-AATG

Section 156

(11471) 11471 11480 11490 11500 11510 11520 11530 11544
Homo sapiens chromosome 13 NC_000013.11: 34882059... (11071) CAATAAGTACCACACAGAATTATTATTAAGTTTAACAAAGATAATGCATGTGTGA-TTGAGTAAAAATTAATAA
SARS-CoV-2 Reference Genome Shuffle No.1 (10829) TGCTAAGAT--ATGCAAGATCGTCGTTTCGTCTTAAGACGATCTTGATTAACTGTCTTGCGTATAAAATATTTA

Section 157

(11545) 11545 11550 11560 11570 11580 11590 11600 11618
Homo sapiens chromosome 13 NC_000013.11: 34882059... (11144) CAGGATAGCTCTTCACCACCAACTTCCTTGTGAAAGAAG-ATTTTGTCTGTCTCATCTTCTCCCCCTTCCCCAC
SARS-CoV-2 Reference Genome Shuffle No.1 (10901) AACTTTAATTTTATTATACTAA-TGGCTTTCAAACGATGTATTGATTTGGTTACAGTTGGTATCAAACTGCTAT

Section 158

(11619) 11619 11630 11640 11650 11660 11670 11680 11692
Homo sapiens chromosome 13 NC_000013.11: 34882059... (11217) CTTCCGAGAAGGATTCTGCTTTGCCCGTTGATTATTTG-GAATTTGATAGTGCAGAATCAGAAAAGAGAAAGTG
SARS-CoV-2 Reference Genome Shuffle No.1 (10974) TATTCGGTGTTAATTGCGGATCGC---TTCAATATCGAAGAGAGTTATATTGTGTAAGCCACAAGCACTACTTG

Section 159

(11693) 11693 11700 11710 11720 11730 11740 11750 11766
Homo sapiens chromosome 13 NC_000013.11: 34882059... (11290) GTAGAAGGAGAAAAGGTATATATATAGACTAAGGCTGTGGGACAAGCTTTTCAGGAAGACTTTGAGAGACAGAG
SARS-CoV-2 Reference Genome Shuffle No.1 (11045) TGCGTATGCGTCAGTGCACGCGT------TATCGCT-----ACCAGCTATATAGCATATGAATTATCTACATGG

Section 160

(11767) 11767 11780 11790 11800 11810 11820 11830 11840
Homo sapiens chromosome 13 NC_000013.11: 34882059... (11364) AGAGATCTGGCAGCCCCTACAGAGG--AGTAGAAAGTGAATGGGATATAAAAGGAAGAATGTTAAGAAGTGCGT
SARS-CoV-2 Reference Genome Shuffle No.1 (11108) ACACTTGTGTCTGGGATCGATGACGCTAGTCGAAGGAGTTGACTGCAGGCCCATATTTATCTGTAGAACTGAGG

Section 161

(11841) 11841 11850 11860 11870 11880 11890 11900 11914
Homo sapiens chromosome 13 NC_000013.11: 34882059... (11436) ATCCTCTTTATAAGTAATATTTGAGAATCAGAAATATAGACCTGAG-TAACC-TCAAACCAACTTATATAAAAA
SARS-CoV-2 Reference Genome Shuffle No.1 (11182) TTC-------TGTGCACAGTCCTCGTCTTAGCAAAAGAGGCATGATATAACCGTGACACAAACATATA-AAAAT

Section 162

```
                                                   (11915) 11915 11920          11930          11940          11950          11960          11970             11988
Homo sapiens chromosome 13 NC_000013.11: 34882059... (11508) TCCTATTTGAGAGAAATAGTACAAACTGAAGAATAGCCTTTCTATTTTTCTGTGGATTAGGCCAGATCACATCA
   SARS-CoV-2 Reference Genome Shuffle No.1 (11248) TACGA----AGGCACCGAGTCCAGATACTAGA-TATGGTGAAAAATTA--------ATTATGTC-GA-CTCATTA
```

Section 163

```
                                                   (11989) 11989       12000          12010          12020          12030          12040          12050      12062
Homo sapiens chromosome 13 NC_000013.11: 34882059... (11582) AGCTTCATATGCTTCTATTTGTCTTGC-TTTCCAGAAATTCTTTCAGAGTTTCCCTTAAGAT--CCATGCCATT
   SARS-CoV-2 Reference Genome Shuffle No.1 (11308) TGTCCCCGATT------TTAGTCCGGCATGTGAAGACATGGTAAGGATGTTTTAAATATGATGTCAACGTCTCT
```

Section 164

```
                                                   (12063) 12063 12070          12080          12090          12100          12110          12120          12136
Homo sapiens chromosome 13 NC_000013.11: 34882059... (11653) AACTTATACTGAAGGAATAATAAACTCAAAAAACATATTTTATTTCGGGAGATTTTTTTTCAGTGTGATGTCA
   SARS-CoV-2 Reference Genome Shuffle No.1 (11376) AAATGTTTAT--ATGATTAATGTTTAGAATTATAATCTGTCATTCCACAAAACATTTTCTAAAAAT--ATGAAA
```

Section 165

```
                                                   (12137) 12137           12150          12160          12170          12180          12190          12200 12210
Homo sapiens chromosome 13 NC_000013.11: 34882059... (11727) CGCTCAAGAAATTTCACTTAGAACATAAAGCCAGAGGAATTT--CTCTCCTAAAGGTGTAATACGTTTTCCCTT
   SARS-CoV-2 Reference Genome Shuffle No.1 (11446) CTCGTAATTTACAACAACTTTAGTAATCAGCAGTATGTTTACAATTGTCGTCAATGTTTAATATTTGTTATATG
```

Section 166

```
                                                   (12211) 12211    12220          12230          12240          12250          12260          12270          12284
Homo sapiens chromosome 13 NC_000013.11: 34882059... (11799) CTGTAACGCTGACAAACGTGTGATTT--TGG--CTACCATTAAGTTCAGAGTCAAATTTC-TAGCTCGACTTTT
   SARS-CoV-2 Reference Genome Shuffle No.1 (11520) CGGACCCGTTG-CAACCTTGTGTCTTCGTGGTAATTGCGGTGGGTTTAAAATCAAAACTAATAAATCGG--TTC
```

Section 167

```
                                                   (12285) 12285 12290          12300          12310          12320          12330          12340             12358
Homo sapiens chromosome 13 NC_000013.11: 34882059... (11868) GTTAATGATTTAAATTAAATTTAAATGATTTTTAAACTTCTGGCCGGATGTGGTGGCTCACACCTGTAGTTCCA
   SARS-CoV-2 Reference Genome Shuffle No.1 (11591) GTTCATG---TACCTTGA--TTGCTTGACCGTAAGCGCTTTCTCTGTCCGTGTCT--TGCCAGCTCTTATTAC-
```

Section 168

```
                                                   (12359) 12359       12370          12380          12390          12400          12410          12420      12432
Homo sapiens chromosome 13 NC_000013.11: 34882059... (11942) GCACTTTGGGAGGCCAAGCGGGGAGGATTGCTTGAGCCCAGGAGTTTGAGACCAGCCTATACAACTGAGACCTC
   SARS-CoV-2 Reference Genome Shuffle No.1 (11657) ---CTTTTCTACGCGAAAATGCGCTCCTCCC--GAAACCACGTTGTTTCATCGACATACTTTAACTTAA--CGC
```

Homo sapiens chromosome 13 NC_000013.11; 34882059-34911962 vs. SARS-CoV-2 Shuffle No.1

```
Section 169
                                                       (12433) 12433  12440      12450      12460      12470      12480      12490         12506
Homo sapiens chromosome 13 NC_000013.11: 34882059... (12016) ATCTCAAAAAAAATTAAAATTAGCCAAGCATGGTGTTGCCCACCTGTAGTCCCAGCTACTTGGGAGACTGAG
      SARS-CoV-2 Reference Genome Shuffle No.1 (11724) ACCAACTCTTGGTATTGAGCACTACACTTATA-GCTGTTAATCAACCATAATGC-------TTGAGACCCTG-G

Section 170
                                                       (12507) 12507        12520      12530      12540      12550      12560      12570  12580
Homo sapiens chromosome 13 NC_000013.11: 34882059... (12090) GTGGAGGATCGTTAGAGCCCATGACATCAAGGCTGCAGTGAGTTGTGATCATGCCACTGTA-CTGGGCAACAG
      SARS-CoV-2 Reference Genome Shuffle No.1 (11789) TAGGTTGTGCCGTAAGAACCGACAACACTTTTAGCGAAATGTTTCGCCAGCAAGTCTCTGGAGCTTATTATATG

Section 171
                                                       (12581) 12581    12590      12600      12610      12620      12630      12640         12654
Homo sapiens chromosome 13 NC_000013.11: 34882059... (12163) AGTGAG---A----ACCTGTCTCAAAGAAAACTTAAAATTAAAAATAAATAATTCTAAGTTCTATCTATCTGAA
      SARS-CoV-2 Reference Genome Shuffle No.1 (11863) AGTGAAATCATAGTACCCTACTCTGTCCTTGGTTCGGTTGAGTTAGCAA-ACCCCTTAGAGGTAGTTACCTGAA

Section 172
                                                       (12655) 12655 12660      12670      12680      12690      12700      12710          12728
Homo sapiens chromosome 13 NC_000013.11: 34882059... (12230) TTTGAGCTGCCTTCTTCCCACTTTCACTTGCTTACATCAGATCATTGGGAATGGATGTAGATAATAATACCAGA
      SARS-CoV-2 Reference Genome Shuffle No.1 (11936) ------CTAAGATCATCATTAGTTCTCTT--TTATAGCGACTTATT-----TATATAGAACTTCTGCTTGCTGA

Section 173
                                                       (12729) 12729      12740      12750      12760      12770      12780      12790     12802
Homo sapiens chromosome 13 NC_000013.11: 34882059... (12304) AC--AGGAGGATTTATTTATTTATTTATTTATTTATTTAGATAGAGTCTCACTCTGTCACCCAGGCTGGAGTA
      SARS-CoV-2 Reference Genome Shuffle No.1 (11997) AACTAATAGGATACATTTATT-----ATATCCTTCTGATAGCT---GTCTTGGACTGTAACTGAACGAATACGA

Section 174
                                                       (12803) 12803  12810      12820      12830      12840      12850      12860         12876
Homo sapiens chromosome 13 NC_000013.11: 34882059... (12376) CAGTGGTGCAATCTCAA-CTCACTGAAATCTCTGCCTGCTGGGCTTAAGCAATTCTTCTGCCTCAGCCCCCAAA
      SARS-CoV-2 Reference Genome Shuffle No.1 (12063) AATCATTGACATACCACTCTTACTAAAAA---TAAATGCTA-----AAGCATATCATCACCTTGCAATTTCTTC

Section 175
                                                       (12877) 12877        12890      12900      12910      12920      12930      12940  12950
Homo sapiens chromosome 13 NC_000013.11: 34882059... (12449) GTAGCTGAGATTACAGGCACCTGCCACCATGCCTGACTATTTTTTTTTTTTGAGGTGAAATTTCATTCTTGTT
      SARS-CoV-2 Reference Genome Shuffle No.1 (12129) -TATCAACACATAAACGTAA-TAATACTAGTTCTATGAAGCTTAACAATTACGAACATACAAAAAATACATCTT
```

```
                                                                Section 176
                                                  (12951) 12951    12960     12970     12980     12990     13000     13010        13024
Homo sapiens chromosome 13 NC_000013.11: 34882059... (12523) GCCCAGGCTGGAGTGCAGTGGCACAATC--TCGACTCACTGCAACCTCCACCTCCTGGATTCAAGAGATTCTCC
        SARS-CoV-2 Reference Genome Shuffle No.1 (12201) ATGTA--CTGGAGACTATAAGTTTAAACGATTTAGTTGTTGCAATATCGCAATCAGGCTCATACCTGCTTTATC
                                                                Section 177
                                                  (13025) 13025 13030     13040     13050     13060     13070     13080          13098
Homo sapiens chromosome 13 NC_000013.11: 34882059... (12595) TGTCTCAGCCTCCCAAGTAGCTGGGATTACAGGTGCCCGCCACCATGCCCAGCTAATTTTTTGTATTTTTAGTA
        SARS-CoV-2 Reference Genome Shuffle No.1 (12273) GATCAGACTTTTACATGTATATTGTTTTGAAGTT------CACCTAGTACCTCAA---TTTAGTTTATTTACAA
                                                                Section 178
                                                  (13099) 13099       13110     13120     13130     13140     13150     13160   13172
Homo sapiens chromosome 13 NC_000013.11: 34882059... (12669) TAGACGGGGTTTCACCATGTTGGCCA---GGCTGGTCTCAACCTGCTGACCTCAGGTGATCA-GCCCACCTTAG
        SARS-CoV-2 Reference Genome Shuffle No.1 (12338) TTGACGAGATTAGCGCATTATGTATATACGTCAGCTTCCAAAATCCAAAAC--AGTTCACAAAGTCTTTCGTA-
                                                                Section 179
                                                  (13173) 13173  13180     13190     13200     13210     13220     13230         13246
Homo sapiens chromosome 13 NC_000013.11: 34882059... (12739) CCTCCCAAAGTGCTGGAATTACAGGCAT---GAGCCACCAC-GCCTGGCCCAGAACAGAGGGATTTT--GAAGT
        SARS-CoV-2 Reference Genome Shuffle No.1 (12409) CGGCGCGCATTAATT-ATTTTCAGCTATATTTATTCAATTCAGACTTTTCTATCTTTGCAAAAATCTACGAATA
                                                                Section 180
                                                  (13247) 13247         13260     13270     13280     13290     13300     13310 13320
Homo sapiens chromosome 13 NC_000013.11: 34882059... (12807) TGCTGGGAGAGGACAAAGAGGTAACAGACTTCCTTTGTGTGTCGGTGGGAGGAGTGTCA-GTGGGAGTAGCCTT
        SARS-CoV-2 Reference Genome Shuffle No.1 (12482) TTCTTGTA-ACTATTCTTA--TATTTGATGCAGTATGTTGCCCAGATTGAGTAATCTAAAGTGTTCAGTTACTA
                                                                Section 181
                                                  (13321) 13321    13330     13340     13350     13360     13370     13380        13394
Homo sapiens chromosome 13 NC_000013.11: 34882059... (12880) AAGATAATAATCAATCAACACACATTTAA-GAGCTATAGTAAGCCAACAAGAGTAGAGCAGTTTATTGGAGCTT
        SARS-CoV-2 Reference Genome Shuffle No.1 (12553) AACATATTAATAGAAACCCACGGCTTAAACGTGGTTGCGGGAACGACCATGGTTAT-GCAGTCACTGGTACCTT
                                                                Section 182
                                                  (13395) 13395 13400     13410     13420     13430     13440     13450          13468
Homo sapiens chromosome 13 NC_000013.11: 34882059... (12953) CAAGAGT-CAAGGCTAAATGACACAAGGCTAAAAGATCCTTATGAATCTTGACCCAGCTTAAAAGATCTAAGTG
        SARS-CoV-2 Reference Genome Shuffle No.1 (12626) TTACTTTGCGTAGAATTCCGAGCATTGGCTAAATTATTATATTGCAGAAT-AGAGTGCG-AAAAGTC--AGTG
```

Homo sapiens chromosome 13 NC_000013.11; 34882059-34911962 vs. SARS-CoV-2 Shuffle No.1

Section 183

	(13469)	13469 13480 13490 13500 13510 13520 13530 13542
Homo sapiens chromosome 13 NC_000013.11: 34882059...	(13026)	ATGAGA-----ACAACTTAAAGTGT---TTTGGTTCTAGAATTTAGAAT-TCTTCTGCGTGAAGAATTTAATCA
SARS-CoV-2 Reference Genome Shuffle No.1	(12696)	GAGACACATTTACATGTATAGGGGAACCTTTAGATGTTTACTTAATATTCTGTAATACTAAAACATGGTCAACA

Section 184

	(13543)	13543 13550 13560 13570 13580 13590 13600 13616
Homo sapiens chromosome 13 NC_000013.11: 34882059...	(13091)	GGTGAC--TGCAAA-GTGTACAGTTTTCATTTTAAAATAACAGGAGATTGAACCTTGGAGCCCCACCAGAAAAG
SARS-CoV-2 Reference Genome Shuffle No.1	(12770)	ACAGCCGCTGTAGACGGGCTCACTAGTCAATCGCTGATCCACATTTATTAGGCTTTATATTCGAATTTTTTAAT

Section 185

	(13617)	13617 13630 13640 13650 13660 13670 13680 13690
Homo sapiens chromosome 13 NC_000013.11: 34882059...	(13162)	ACTAAATGTGCTTAGGGATGATGGCTTAGAGAGGTTTAACCAG--CTGGAGGT----GAAACAGTTTGCAACTG
SARS-CoV-2 Reference Genome Shuffle No.1	(12844)	AACACCAG-GATTCGTCATGAAATCATCTACTTTTGTCATCTGACCTTTAGTTTCTGGTATTCCTAAGTACTTG

Section 186

	(13691)	13691 13700 13710 13720 13730 13740 13750 13764
Homo sapiens chromosome 13 NC_000013.11: 34882059...	(13230)	AACAATCT---AGATGGTTATGTGAAGTACTGTGCCATGCAGAGTCCTCATAATTTATCTCTG---TAATTATA
SARS-CoV-2 Reference Genome Shuffle No.1	(12917)	ACTATTTTATAAGAGCGGTTTTTGAT-TGTGGTGCAAT--AAATTGCTACAAATTGTGCTTTGACTCCATGGAA

Section 187

	(13765)	13765 13770 13780 13790 13800 13810 13820 13838
Homo sapiens chromosome 13 NC_000013.11: 34882059...	(13298)	ATATCAACCCTAGACCTTCATTTAAAAGAAGAAAATATTTGAACCATTATTG-----AC-TCCAGAAGGCAATA
SARS-CoV-2 Reference Genome Shuffle No.1	(12988)	GAATCAACGCCAAACGTAGCGCTACTA------ATTCTTTCTCCAGTTATTGGTCGGAAGTTAACGAAGTAATG

Section 188

	(13839)	13839 13850 13860 13870 13880 13890 13900 13912
Homo sapiens chromosome 13 NC_000013.11: 34882059...	(13366)	TAAGGAGCCTTTTTTTTTTTTTTTTTTTTTTTTTCAGAAACCTTTTA--TTGGTTTCATTAACAGAATCTGGCA
SARS-CoV-2 Reference Genome Shuffle No.1	(13056)	TGGGTGTCCTGCTATTTGTCATGTTGTAGCTTCGTTACAATCTTATAACTTTATGTTATGACCATCCAGAACTT

Section 189

	(13913)	13913 13920 13930 13940 13950 13960 13970 13986
Homo sapiens chromosome 13 NC_000013.11: 34882059...	(13438)	TGTCCACTTTAGTTCTCCTTAGCTAAACT-TAAACAGACATTTCAAT-GTAAGTCTACTTAAGTAAA--CTAAA
SARS-CoV-2 Reference Genome Shuffle No.1	(13130)	ATTGAAAAATAGAACACCATAGTTTAGCGATAACCACAGTGCTCTATTGGCCGCGTAGCTCGATAGATGCTTTA

Section 190

(13987) 13987 14000 14010 14020 14030 14040 14050 14060
Homo sapiens chromosome 13 NC_000013.11: 34882059... (13508) AACA-AGACACAT--AGAGTCACGCCCAACAAGAACCTTAGAAACCATCTAGTCCTAGGCCGGGCACAGTGGCT
SARS-CoV-2 Reference Genome Shuffle No.1 (13204) GCCTTAGTTTCGTTAAAAGCATAGTGCAATTAGTAATTGAGGAG--ATGTAG--CTAGACTTGTGACGATTGGT

Section 191

(14061) 14061 14070 14080 14090 14100 14110 14120 14134
Homo sapiens chromosome 13 NC_000013.11: 34882059... (13579) CA--TGTCTGT-AATCCCAGCACTTTGGGAGGCTGAGGCAGGC--TAATCACCTGA-GGTCAGGAGTTCGAGAC
SARS-CoV-2 Reference Genome Shuffle No.1 (13274) AAGCTTTCCGTTAACAAGATGACGTTATGCCACTCGATCTGGTTGTAATGTTGTGCTGATCAATTGTTTGAGCG

Section 192

(14135) 14135 14140 14150 14160 14170 14180 14190 14208
Homo sapiens chromosome 13 NC_000013.11: 34882059... (13647) CAGCCTGGCCAACACAGTTAA-ACTCCATCTCTATTAAA--------AATACAAAATTAGCTGGGCGTGGTGGT
SARS-CoV-2 Reference Genome Shuffle No.1 (13348) TATGTGTTACAATTCTATTATTACTATGTGTCCAAGTAACGGATCCGAAGTATAAATTTCTTGATCGTTAACTC

Section 193

(14209) 14209 14220 14230 14240 14250 14260 14270 14282
Homo sapiens chromosome 13 NC_000013.11: 34882059... (13712) GC-----AT-GCCTGTAATCCCAG--C-TACTCAGGAGGCAGGAGAATCTCTTGAACTCGGGAAGCAGAGGTTG
SARS-CoV-2 Reference Genome Shuffle No.1 (13422) GCTACCAATAGCCTCAAAATCAGGTTCCTTCTCCGCTGTTATCTCCTTCTATGGTGCTAGATTATC-GTTGTTG

Section 194

(14283) 14283 14290 14300 14310 14320 14330 14340 14356
Homo sapiens chromosome 13 NC_000013.11: 34882059... (13777) CAGTGA-GCCGAG-ATTGCGCCATTGCACTCCAGCCTGGGCAACAAGAGCGAAACTCTATCTCAAAAATTAATA
SARS-CoV-2 Reference Genome Shuffle No.1 (13495) CTATGTTGCCTATTATTCTGCGGTTTCAGT------TGTGTTAAAGGAGTGGCAGAAGCTCCTTGAGATACATA

Section 195

(14357) 14357 14370 14380 14390 14400 14410 14420 14430
Homo sapiens chromosome 13 NC_000013.11: 34882059... (13849) AATAAATAAATAAATAAGAAAAGAAA----AGAAATTTCCTAGTC---CTAGCTCCTCA------TTTTACAGG
SARS-CoV-2 Reference Genome Shuffle No.1 (13563) GCTCTATAAGGAGATTATAAGTGTGGCTTCATGGCTTTCATAACCAATGGAGTATTTCAATACGATGCGTCAGT

Section 196

(14431) 14431 14440 14450 14460 14470 14480 14490 14504
Homo sapiens chromosome 13 NC_000013.11: 34882059... (13910) TTGGAAACCAAAATTGATAA-AGGCCAGAATCACATGTCTTAGCTAATGGCAAGAA--CCTGAGGCCTCTGGAT
SARS-CoV-2 Reference Genome Shuffle No.1 (13637) TGGGGTGCCTAAATTGATAACAATTCCGGCTGACTACCAGTTCATAATATGAAGGGGGCGCGATAAAACATGAT

```
Section 197
                                                   (14505) 14505 14510      14520      14530      14540      14550      14560        14578
Homo sapiens chromosome 13 NC_000013.11: 34882059... (13981) GCTCTGCGATCCTGTT-CTGTCAG---AAATGCATGAGGTTACTCTGCCCAAACCCCACCAATGCAAGTTATAA
         SARS-CoV-2 Reference Genome Shuffle No.1 (13711) AAGTAACACACTTTTTTCGATGAGGCTAAAAACATCAGCTTAATGTTACGACAAGGAGTCAA---AAGTACTAT

Section 198
                                                   (14579) 14579      14590      14600      14610      14620      14630      14640     14652
Homo sapiens chromosome 13 NC_000013.11: 34882059... (14051) AAATGATTTTGAAGCTGTTTCTGAGGCAAAAACAGAGACCCACAGCTCTG-CAATTCCATTTGCCAGCCCCAGT
         SARS-CoV-2 Reference Genome Shuffle No.1 (13782) AAACGATAT--AAG---TGTGTGGCGCTA---CATAGTTACGAATGTGTAACTATTACACTTATTCACCTCATT

Section 199
                                                   (14653) 14653  14660      14670      14680      14690      14700      14710       14726
Homo sapiens chromosome 13 NC_000013.11: 34882059... (14124) TCACATTTGCTTTTTTT------TCT--ATCAATTATACAAATTAAACTTCTCTAGTTTTCAAATTCCAGAA-T
         SARS-CoV-2 Reference Genome Shuffle No.1 (13848) CCAATCGTGCAAGTCTTAAGCAGCATGAATAATTTTTATATTGATCACTTGCTGAGTTCTTAGCTTCGACATGT

Section 200
                                                   (14727) 14727        14740      14750      14760      14770      14780      14790  14800
Homo sapiens chromosome 13 NC_000013.11: 34882059... (14189) AATTTGCATATTCCAATAAATTCCCAGGTGACTTTATTGAGCA-TAGGCGTTAGCAGTATAGACAAATTTCATC
         SARS-CoV-2 Reference Genome Shuffle No.1 (13922) ACTCTGC----TGCAGAAA--TCCTAGTCG--TTAAATGCGTTCTATTCGACAGAAACAGATTTATAGATTTT

Section 201
                                                   (14801) 14801    14810      14820      14830      14840      14850      14860      14874
Homo sapiens chromosome 13 NC_000013.11: 34882059... (14262) TTTTGACTTGTTGCTACAT---GTCTTCGTTTAGGAATCCCTT-CTGGCTTTTATGACTCAGGATTTTCTTTAA
         SARS-CoV-2 Reference Genome Shuffle No.1 (13988) GACT-ACTTGCTGCTAACTCGAATATTCGTTTTTGCTACTCTGGCCTGCTTTGGTTTTCTAACGTCATTTATAG

Section 202
                                                   (14875) 14875 14880      14890      14900      14910      14920      14930        14948
Homo sapiens chromosome 13 NC_000013.11: 34882059... (14332) AATCTCCTTGGACC--AATATTTCTATATTTTCACTAAG--GTTCAGTTAGCTCTTTTAAATTCAAAACTGTAA
         SARS-CoV-2 Reference Genome Shuffle No.1 (14061) AGCTTAGTGGGAATGAAATAGATCGTTATTCTTCTGAAGTCGCTTACTCCCATGGGCTCGATACATGACCATAT

Section 203
                                                   (14949) 14949      14960      14970      14980      14990      15000      15010     15022
Homo sapiens chromosome 13 NC_000013.11: 34882059... (14402) TCTGCAGCTATGAAATCTGTTGCCCAGCAAGGTAAGCATACTTTGCAATTCTTTGGCAACCTTCTTTTTCTC-T
         SARS-CoV-2 Reference Genome Shuffle No.1 (14135) TCA--AGGTCTCATATTAGATAACCTAGAATCAATGTAATGGCTTCAAAGCTA----AATGTAGAGATGGTCCT
```

Homo sapiens chromosome 13 NC_000013.11; 34882059-34911962 vs. SARS-CoV-2 Shuffle No.1

Section 204

(15023) 15023 15030 15040 15050 15060 15070 15080 15096

Homo sapiens chromosome 13 NC_000013.11: 34882059... (14475) CCAACTTCTTCCATCCCATAATATTTACACTAA--GTCTTATTTG--TTCTTACTTTCACTCCATGAGTATTTC

SARS-CoV-2 Reference Genome Shuffle No.1 (14203) CGAACATTGTACTCTGGTTCTTGTACGAAATGACGGCCTTACTGCATTTGTCACTTTTAAAAAGAGGTCAAGAG

Section 205

(15097) 15097 15110 15120 15130 15140 15150 15160 15170

Homo sapiens chromosome 13 NC_000013.11: 34882059... (14545) TTCTACCTACCAAGCACCCTCAGTCCAAGTTCAAGGAAAGATACAGAA-----CAGAATCAAAAGTACATAAA-

SARS-CoV-2 Reference Genome Shuffle No.1 (14277) TCAAGCCTAACGATTACCATT-GTTCGCGCTTATGGGAACGTACAGAATTTCCTATAATTTAGCGTTTTTCAAG

Section 206

(15171) 15171 15180 15190 15200 15210 15220 15230 15244

Homo sapiens chromosome 13 NC_000013.11: 34882059... (14613) -ATAATAGCAG------ATACATTTTCAGGATAATTGTTAATCCTTGTTTTGTGTACTTTACAATGTGTGGGCA

SARS-CoV-2 Reference Genome Shuffle No.1 (14350) GATAAAAACGCCTTGGAATACCCATTCTGCATTCTCG--ACTCACTACTTCTCGTCTTTTTAGATTTGAACATG

Section 207

(15245) 15245 15250 15260 15270 15280 15290 15300 15318

Homo sapiens chromosome 13 NC_000013.11: 34882059... (14680) CACCATTTTGTTTTTTAATAACAATATCTTTCATCAGTTAAAAAACGTAGTTTCTATTTTTCCTATTATAATA-

SARS-CoV-2 Reference Genome Shuffle No.1 (14422) CTGAATTAGTTCCAGTATTAGGCAGC-CTTTAAGGATTACTCTAATTTATTACGAAGTTATCCGTCTATAATAA

Section 208

(15319) 15319 15330 15340 15350 15360 15370 15380 15392

Homo sapiens chromosome 13 NC_000013.11: 34882059... (14753) ---TGGCCGGTTATTTCCTCTT-ATTTTTTAAAAATACCATTTTTGC---TGTATCCTGTGTAACTG-ATACAG

SARS-CoV-2 Reference Genome Shuffle No.1 (14495) GCTTAACTGCTAATTTCCGGTACAGCTTCGCTATATGACGTCCTGACCCAGGTATCATGCCCCTTAGTATCTAG

Section 209

(15393) 15393 15400 15410 15420 15430 15440 15450 15466

Homo sapiens chromosome 13 NC_000013.11: 34882059... (14819) AAATAAGTTCACAAAAGATTATAAATTTTTTTTTGCAATAAGGAGTTAGTCAAGGCCAGTCGCGGTGGCTCACA

SARS-CoV-2 Reference Genome Shuffle No.1 (14569) AAGTCCAATGACTAA---TTATAGGCTGTTTAGCCCATAAAACAGACAT--AAGAGCATTCGTTACAACTAAAT

Section 210

(15467) 15467 15480 15490 15500 15510 15520 15530 15540

Homo sapiens chromosome 13 NC_000013.11: 34882059... (14893) CTTGTAATCCCAT------------CTCAAAACAAAAACAAACAA--ACAAACAAACAAAAACAAAGAGTTAGT

SARS-CoV-2 Reference Genome Shuffle No.1 (14638) CTCATAATAGCATAATAGTTGGTGACTCTGCTCTTACATATGGTATTAGGAGGAAAAACACTCAAAGCGATATA

```
                                                                Section 211
                                                    (15541) 15541    15550     15560     15570     15580     15590     15600        15614
Homo sapiens chromosome 13 NC_000013.11: 34882059... (14953) CAAAACAAGTAGCT-TGGCCGGATATGGTGCTCACGCCTGTAATCCCAGCACCTTTGGAGGCCGAGGCAGGCTG
         SARS-CoV-2 Reference Genome Shuffle No.1 (14712) ATTGATTTGAAGATGTGGTTAATTTTGATCCGACCGAATGTATTTTATGCATTTCCATAGTAGTTGAAA---TT
                                                                Section 212
                                                    (15615) 15615 15620     15630     15640     15650     15660     15670             15688
Homo sapiens chromosome 13 NC_000013.11: 34882059... (15026) ATCACCTGAGGTCAGGAGTTCGAGACCAGCCTGGCCAACATGGTGAAACCTCATCTCTACTAAAAAT-ACAAAA
         SARS-CoV-2 Reference Genome Shuffle No.1 (14783) ATGACCTCTGTGTCGGT-TACAAAACGAA--TGCTTAACTGGTCCTATTCCGACCAATCCGACGTCTTACGCCT
                                                                Section 213
                                                    (15689) 15689     15700     15710     15720     15730     15740     15750       15762
Homo sapiens chromosome 13 NC_000013.11: 34882059... (15099) ACAAATTAGCCAGGCATCCACTGTGGCACAAGCCTG--TAATCCCAGCTACTCAGGGAGCTGAG---GCAGGAG
         SARS-CoV-2 Reference Genome Shuffle No.1 (14854) GCGGTTTGTTCACAAATTTTGAATACCTCATGCTTGGATTATTACAACTAATTACGATATTGTGTTGGGAGGAC
                                                                Section 214
                                                    (15763) 15763  15770     15780     15790     15800     15810     15820           15836
Homo sapiens chromosome 13 NC_000013.11: 34882059... (15168) AATTGCTTGAA--CCTAGGAAGTG---GAGGTTGTGGTCAGCT-GTGGTCAAGCCACTGCACTCCAGCCTGGGT
         SARS-CoV-2 Reference Genome Shuffle No.1 (14928) AGGAGCTTGGAGGCAAATGTATTCTTCGATGTACATATGAGTTAGTGTACTTACCAGTACTGTCTTA--TGCGT
                                                                Section 215
                                                    (15837) 15837        15850     15860     15870     15880     15890     15900 15910
Homo sapiens chromosome 13 NC_000013.11: 34882059... (15236) GACACAGTGAGACTCCATCTCAAAAAAACAAAACAAACAAGTAGCTAATTTTTGGAAGACTTTTTGCAAGGATT
         SARS-CoV-2 Reference Genome Shuffle No.1 (15000) GT-ACAGTAGGATTTTATTAGCGAAACAAAGCACATGCAAG-ATCTTCATATTGTTCTACTACT-GCGGAGATG
                                                                Section 216
                                                    (15911) 15911    15920     15930     15940     15950     15960     15970        15984
Homo sapiens chromosome 13 NC_000013.11: 34882059... (15310) AAATTCACAAATCTTAGATGAGCCAAGAGCTGGAAACTTCCAGCAGATGCAGTCAGAGAAGTGAGTAAGAATGA
         SARS-CoV-2 Reference Genome Shuffle No.1 (15071) ATAGCTAACTACCATGG--GACATTTGGCATGATATTTGCTGGTATATATACCCATACATGTAAGTAGTACGGA
                                                                Section 217
                                                    (15985) 15985 15990     16000     16010     16020     16030     16040             16058
Homo sapiens chromosome 13 NC_000013.11: 34882059... (15384) GTCTTACCCCTTGCATTTATTGGGTTCTCTTATGAGATTTAATATGATAAATGAGCACGGTGGCTCAGGCCTGT
         SARS-CoV-2 Reference Genome Shuffle No.1 (15143) -TATTTCAAGATCC-TTGATTCTAAT---TCATAACATTGATAAAAACCAAAGTTTACATTTAGTTGGGACACT
```

Homo sapiens chromosome 13 NC_000013.11; 34882059-34911962 vs. SARS-CoV-2 Shuffle No.1

Section 218

```
                                                           (16059) 16059      16070     16080     16090     16100     16110     16120       16132
Homo sapiens chromosome 13 NC_000013.11: 34882059... (15458) AATCACAGCACTTTGGGAGACCGAGGAGGGTAGATCCCTTGAAGACAGGAATTTCAGACCAGCCTGGCCACCAT
SARS-CoV-2 Reference Genome Shuffle No.1 (15212) ATGTTGAACTCCTTGTAAG-CATTCGATATTAGCATACT--AATACAT-ATTCTAGTACGAGA------AAAAG
```

Section 219

```
                                                           (16133) 16133  16140     16150     16160     16170     16180     16190         16206
Homo sapiens chromosome 13 NC_000013.11: 34882059... (15532) GGTGA--AACCCCGTCTCTACTAAAAATACAAAAATTAGCTGGACTTGGTGGCACGTATCTGTAATC-CTAGCT
SARS-CoV-2 Reference Genome Shuffle No.1 (15276) GGTAATCAATACGCTAACTGCTGTTAAGCCG-----TATCTTTAATCTTTCGTA--TATAGGCAACCACTGAGC
```

Section 220

```
                                                           (16207) 16207        16220     16230     16240     16250     16260     16270 16280
Homo sapiens chromosome 13 NC_000013.11: 34882059... (15603) ACTCAGGAGGCTGACACTTG--AGATTCACTTGAACCTGGGAGGTTGCAAGATTGCACCACTACACTCCAGCCT
SARS-CoV-2 Reference Genome Shuffle No.1 (15343) ACTCCGTA---TGACCCTTTGTAGTACTAATATAAATTAGTAGAAACCCAAAATGCA--ATGACCCGAGAAC--
```

Section 221

```
                                                           (16281) 16281    16290     16300     16310     16320     16330     16340         16354
Homo sapiens chromosome 13 NC_000013.11: 34882059... (15675) GTCTCAAAAAAAGAAAAAATTAATTAATTAATTAAATATATGTATATATTTATCATATTTTTACATGATTT--T
SARS-CoV-2 Reference Genome Shuffle No.1 (15410) -TCCCCTGTTTATATAATATTACGT--TTAGTTAAACGTATTCGTCTTTCAAGGATTGATCTA-ATCATACCAT
```

Section 222

```
                                                           (16355) 16355 16360     16370     16380     16390     16400     16410            16428
Homo sapiens chromosome 13 NC_000013.11: 34882059... (15747) TATATATGAT-AAATGAACCAAT--TAGACTCCATTATGTTTTTCAATGCCCCACTCCCCAAATTTGCCACCAT
SARS-CoV-2 Reference Genome Shuffle No.1 (15480) TGTCTAAAATCAGATGACCCATTCCTAGTATCAACGGTACACAGTCATGTTCGAAAGCT--AATCATCTCTTAT
```

Section 223

```
                                                           (16429) 16429      16440     16450     16460     16470     16480     16490       16502
Homo sapiens chromosome 13 NC_000013.11: 34882059... (15818) TTAAAATTCACCAAAAGCTAAAATTTGGATCATTGCTTTATGACTAAA--AAGTCTGTGAATCTGCTACCAACT
SARS-CoV-2 Reference Genome Shuffle No.1 (15552) TTTACAGGGATCTGCTCCCAA--TCTACATAGTTGGTTTAAGACCAAACCACTAGTCTAAAATAGGT-----CT
```

Section 224

```
                                                           (16503) 16503  16510     16520     16530     16540     16550     16560         16576
Homo sapiens chromosome 13 NC_000013.11: 34882059... (15890) ATAAAA-TCCCTTCACAAGTGTATATTCCCTTAGGAAAGATCAAAT-TCATCAGAAGGGCAACTTTCAAGAGTA
SARS-CoV-2 Reference Genome Shuffle No.1 (15619) ATATACGTAGATTGAAAGTTGTCTGTACAATTGAGAGGGATTTCATATGATCAATTGAACTCTATTAAAGAGTA
```

Section 225

(16577) 16577 16590 16600 16610 16620 16630 16640 16650
Homo sapiens chromosome 13 NC_000013.11: 34882059... (15962) --TGAATTT-TAAGCCATATTAAGA-GTTCTTCCCAAAGCATCTCAGAGAAACATGTTCAGAAGTCCAGCCTCA
SARS-CoV-2 Reference Genome Shuffle No.1 (15693) ACTAAACTTGTCATCGGACTAAAGCCGACGATCGTAAAGTAGCTTTTG----CCCCTCCAAAAGATATTCCTTG

Section 226

(16651) 16651 16660 16670 16680 16690 16700 16710 16724
Homo sapiens chromosome 13 NC_000013.11: 34882059... (16032) TGGCCACA---GCCCAAACAATCCTGG--AGTTATTTTAGTGCTCTGCGATATTTTCGTCCCCA--TCCTTTGT
SARS-CoV-2 Reference Genome Shuffle No.1 (15763) TGTCAACGTACGCCCTGATATTAATACACAGACAGGTGAACAATTTCACGGGCTGTTGTCCCCAAGTCATTTTA

Section 227

(16725) 16725 16730 16740 16750 16760 16770 16780 16798
Homo sapiens chromosome 13 NC_000013.11: 34882059... (16099) CCTCCTTTTCT--ATAGTATCGATTTCAGGATTAGTATCACT---ATCATATAACTACACACACACACACACAT
SARS-CoV-2 Reference Genome Shuffle No.1 (15837) AAGACGGAAATGAATAATGTAAAATGAATATATAGTACGACCTGGGTTATGAAATTCCTTATTATGGCACCC-T

Section 228

(16799) 16799 16810 16820 16830 16840 16850 16860 16872
Homo sapiens chromosome 13 NC_000013.11: 34882059... (16168) TAAAACCTGTATGTAGGTATATTTTCATGTTGCCATTTGCTTCTGATTATC--CTTGATCTACATGCATATAT
SARS-CoV-2 Reference Genome Shuffle No.1 (15910) TACAAACACGAACCGCGTGTTCTTATCTATTTAGCGGTTACTTTCTAAAATCAACGCCGTCTAAATG--TGGTG

Section 229

(16873) 16873 16880 16890 16900 16910 16920 16930 16946
Homo sapiens chromosome 13 NC_000013.11: 34882059... (16240) GCTTTTAAAATAATGAGCCTTATTTATTTTTCCAATTTCATTTTACCTTAGAACTGTTCATTTGCATAAGAGTA
SARS-CoV-2 Reference Genome Shuffle No.1 (15982) GACGTTGTTTTTCTCTACGTTAACTAATGTTTTATAATTATTGTAGAATACGA--GTT--TTTGTAAAAC-GTA

Section 230

(16947) 16947 16960 16970 16980 16990 17000 17010 17020
Homo sapiens chromosome 13 NC_000013.11: 34882059... (16314) GTCAGTGAAATGTATACTT-CTAATTGTTC--------ATTTTTACTTGTGTCA--AAACCAGATATCCTTCAT
SARS-CoV-2 Reference Genome Shuffle No.1 (16051) GTAACTGTAAAGATTACGGGCTAGATTTTCCTAGATGAATGAAAACTGTTGTAAGGTATCGAAGTCAACGTCGC

Section 231

(17021) 17021 17030 17040 17050 17060 17070 17080 17094
Homo sapiens chromosome 13 NC_000013.11: 34882059... (16377) CTTTTTTTCCTGACTTTACATCGTTC-ACAAGCACAACCCATTTCCTTCAAAATTTTCTCACTGCACACAGTAA
SARS-CoV-2 Reference Genome Shuffle No.1 (16125) ATAAAAGGTTTTACATTACCACTATGGAAAATCATCGCCC-TAGACTTTATTATATTTTTATTGAGATTTTTCA

Homo sapiens chromosome 13 NC_000013.11; 34882059-34911962 vs. SARS-CoV-2 Shuffle No.1

Section 232

```
                                                        17095 17100     17110     17120     17130     17140     17150         17168
                                                (17095)
Homo sapiens chromosome 13 NC_000013.11: 34882059... (16450) --TTTCCAGTGGT-AGGAAAGAATGCTTCATGG-GTATTTCAAAACATCTTCTG-TCCTAACCCTTCGTTCTT
       SARS-CoV-2 Reference Genome Shuffle No.1 (16198) GATATCCCTTTGCTTATTCAACATGACATGATTCAGTTGCTATAAATCACTACTGATGATACATCTGACGTCTT
```

Section 233

```
                                                        17169      17180     17190     17200     17210     17220     17230      17242
                                                (17169)
Homo sapiens chromosome 13 NC_000013.11: 34882059... (16519) TTCTCACTTTG---GATCTCTGGTGT-CTTCC--CACACAAAAGGGAATATGCCA-TCTCCCTTTAG--GAA
       SARS-CoV-2 Reference Genome Shuffle No.1 (16272) TTATTACATAGTCATATTTTCGCTTTACTTGCGCCGGACACAAGAGGTTGTATTCAAGTCACCGCTCACCTGAT
```

Section 234

```
                                                        17243  17250     17260     17270     17280     17290     17300          17316
                                                (17243)
Homo sapiens chromosome 13 NC_000013.11: 34882059... (16584) ATCAAGATTCTAAAATATCTTTCCTCTCTTGACCGTTTTGGACTGTGGGTATTCATAACCTCACATA-TAAAT
       SARS-CoV-2 Reference Genome Shuffle No.1 (16346) TTGAAAAATTTACAAAATCAGCCCATTACCTACGTCACTAG-CACTGGTGCTTCAT--CTTCAACTAGTTAAGT
```

Section 235

```
                                                        17317        17330     17340     17350     17360     17370     17380  17390
                                                (17317)
Homo sapiens chromosome 13 NC_000013.11: 34882059... (16657) TGGCATAAATGTATTTATTTATTTATTTATTTATTTATTTATTTATTTATTTTGAGACAGTGTCTCGCT
       SARS-CoV-2 Reference Genome Shuffle No.1 (16417) ATCCGGCCCCGGGTGGCTAGATCGGAAAATAAACTTTATCAGTTGTAGTAGAGCCTTT---ACAATATGATATT
```

Section 236

```
                                                        17391    17400     17410     17420     17430     17440     17450        17464
                                                (17391)
Homo sapiens chromosome 13 NC_000013.11: 34882059... (16731) CTGTCGCCCAGGCTGGAGTGCAGTGGCGCGATCTCAGCTCACTGCAAGCTCTGCCTCCCGGGTTCCTGCCATTC
       SARS-CoV-2 Reference Genome Shuffle No.1 (16488) ATTTATCACAT-CTTCATTC----GTCTCGTTCTTG--TCAATGCTTG---TGTTACCCATTTTACTGCAATAG
```

Section 237

```
                                                        17465 17470     17480     17490     17500     17510     17520         17538
                                                (17465)
Homo sapiens chromosome 13 NC_000013.11: 34882059... (16805) TCCTGCCTCAGCCTCCTGAGTAGCTGGGACTACATGCGCCCGCCACCACGCCTGGCT----AATTTTTTGTATT
       SARS-CoV-2 Reference Genome Shuffle No.1 (16552) ATCTTACTCCGTATCCAAAAGAGGCTGGA-TACAGTTACCACACCCATTGTCTTGTTTTATAATTTTATCGATG
```

Section 238

```
                                                        17539      17550     17560     17570     17580     17590     17600      17612
                                                (17539)
Homo sapiens chromosome 13 NC_000013.11: 34882059... (16875) T--TTAGTAGAGACGGGGTTTCACCGTGTTAGCCAGGATGGTCTCGATCTCCTGACCTCGTGATCTGCCCGCCT
       SARS-CoV-2 Reference Genome Shuffle No.1 (16625) TCTTTAGTAATTTGGAAGATACA---TGTAAACT--GATTCCAAGGGGAGGCT-ACCCAATGATAAGCC-GACT
```

Section 239

```
                                                       17613  17620           17630           17640           17650           17660           17670           17686
Homo sapiens chromosome 13 NC_000013.11: 34882059... (16947) CGGCCTC-CCAAAGTGCTGGGATTACAGGCGTGAG-ACACCACGCCTGGC-----CTAATTTATTT-TTTAAGT
SARS-CoV-2 Reference Genome Shuffle No.1 (16692) CGCACTCACTTAAGGGCGACTAGTGGTATCGTAAGTAGAGTTTGCGTAATAGATTCTGGTTTAATTATTGACGG
```

Section 240

```
                                                       17687  17700  17710  17720  17730  17740  17750  17760
Homo sapiens chromosome 13 NC_000013.11: 34882059... (17013) AATAAGCTTTAATCAGTTATGA---TAAAACATCAGTTATGAACCACATTGTTAGTTTTTGTTACTCTTCTTTC
SARS-CoV-2 Reference Genome Shuffle No.1 (16766) GATAAGAAACAATTACTGAGGGGGGCACTAGACAACCTTTGCTCATTCTATACAAGGGAAATCACTCCGCTGTC
```

Section 241

```
                                                       17761  17770  17780  17790  17800  17810  17820  17834
Homo sapiens chromosome 13 NC_000013.11: 34882059... (17084) ---------ACTATTA-TATTTCAGTAAATATTGCCAAATGCATTT-TAATTGTCTTTGATATTTTAATAGCTT
SARS-CoV-2 Reference Genome Shuffle No.1 (16840) CAAATCGCTACTCCTAGTCTCTATGGTAACGTTGGCAAATCTGGGTATAATAATCTTATTACGCCTAATGGAA-
```

Section 242

```
                                                       17835  17840  17850  17860  17870  17880  17890  17908
Homo sapiens chromosome 13 NC_000013.11: 34882059... (17147) CATGTACCCTTGAA-GAGATGTGCTGCCAGTCTAATTATTTTCAAAA-------AGTTAAATTATCCCTCAAAT
SARS-CoV-2 Reference Genome Shuffle No.1 (16913) -AGCCACTATTAAATGATACAATCTGTGAATTAATATATCGGCCAAAACACCTTAGTATAGGCAGCCGGGAAAA
```

Section 243

```
                                                       17909  17920  17930  17940  17950  17960  17970  17982
Homo sapiens chromosome 13 NC_000013.11: 34882059... (17213) GAGACTTTCTTCATTTTAAAATT-TCCTCATCTACCCATAG-TGTTTGTTGA-----TAGATTTC---CTCAAC
SARS-CoV-2 Reference Genome Shuffle No.1 (16986) AAAAACTTCAACGTAGCCCTATTCTGTTCTTGTTTCTATAACTGTAAGTCAAGGGCGTAGATTTCGAACATAAC
```

Section 244

```
                                                       17983  17990  18000  18010  18020  18030  18040  18056
Homo sapiens chromosome 13 NC_000013.11: 34882059... (17277) TATTTTCTAGGTCTCTTGTTTTCTCTTGTGTTGTAAT--TAATCAGTTAGTGTTTTCATGCATAGGGAAACACA
SARS-CoV-2 Reference Genome Shuffle No.1 (17060) TATCA-CTAGGTTGAATTAGTTAACTTCTTTT-TACTCCTTATCTATTATATCAGTCTAAAATACTCTGAGGAA
```

Section 245

```
                                                       18057  18070  18080  18090  18100  18110  18120  18130
Homo sapiens chromosome 13 NC_000013.11: 34882059... (17349) TGTTGTTCTCATCAAGTTTCTTTCAGTTGCTTACTTAATCTTCTTCACAGCTTCCAGAAATATCTTTACTCCAT
SARS-CoV-2 Reference Genome Shuffle No.1 (17132) AGATTATTTGGATAAGAT-CTCTTAG--GCGGAACTTAGGAGCGAAAC----TCAGGAAAGAACGTTGGTAAGA
```

Section 246

(18131) 18131 18140 18150 18160 18170 18180 18190 18204

Homo sapiens chromosome 13 NC_000013.11: 34882059... (17423) CTGTAGAATTACAGGTTTAGAATACAGTTTCCTTTCCAGGTTATTGACTCTGTTTTCCCCTGTGATGTTG--CA

SARS-CoV-2 Reference Genome Shuffle No.1 (17199) CTTT-GACGCGC-GGAACGCAATAC--TTTGCTTTTAACATAATATGATCCGCAGTAGGAAGTACTGGCGATCT

Section 247

(18205) 18205 18210 18220 18230 18240 18250 18260 18278

Homo sapiens chromosome 13 NC_000013.11: 34882059... (17495) ATTTTTGTTCACTAGC---CAAGGAGTATTAACTGGTGTACTCATTCGTTTGGGAACTTAATTTTTTGGG-TTC

SARS-CoV-2 Reference Genome Shuffle No.1 (17269) ATGTTTGAGCGTTAGTTATCAATCCCCCTGTTCTTTACGACTAAAGTTACTTGAAGTTTAAGAATTTAGAATTC

Section 248

(18279) 18279 18290 18300 18310 18320 18330 18340 18352

Homo sapiens chromosome 13 NC_000013.11: 34882059... (17565) TATTATTTTCCTTTTT-TAGGTGACATTATTTTTTTTTTACTGCATATAGAAATAAGGAATAACCTG-ACACTA

SARS-CoV-2 Reference Genome Shuffle No.1 (17343) TAT-ATTTACCTAAAGCTAATGCAGAGTACCCGGAAGTTTATGCGTACCAAATT--GCAGCCACCTGTAGAGTA

Section 249

(18353) 18353 18360 18370 18380 18390 18400 18410 18426

Homo sapiens chromosome 13 NC_000013.11: 34882059... (17637) GATGT-TACTTCCTCTACCGTACCATCTTTCCAGAACGCCATAGACTTGTACCACAAAATACAGTCATTTTTAA

SARS-CoV-2 Reference Genome Shuffle No.1 (17414) GCTGTGTAACCACAAAAACGAAAAGT-------GGGCTCCTCAGCAGGCTACAAATAATCGAAGTG-TGGTTAC

Section 250

(18427) 18427 18440 18450 18460 18470 18480 18490 18500

Homo sapiens chromosome 13 NC_000013.11: 34882059... (17710) ATAGT-TGTGCTTGATTTTTTTC----TTGTTAAC---TTCCTATGATGA-TTGCTGCACGGTATAGTAAAATA

SARS-CoV-2 Reference Genome Shuffle No.1 (17480) CAAGTATATGCTAATTTCATTACAGCTTTGATCACCCGTTTCTACGGTGACTCGCTCTACCG-AGGCTAGCAAC

Section 251

(18501) 18501 18510 18520 18530 18540 18550 18560 18574

Homo sapiens chromosome 13 NC_000013.11: 34882059... (17775) GATGAGCATATTATTTTAAAAGTATTTTTCATGCCTTTAACAGGCTTTGTTTGCTGTGTAAGGTTTGAACACTA

SARS-CoV-2 Reference Genome Shuffle No.1 (17553) AAT----AGATTACTTTAGCTCT-TGTGTGTTTCTTAGAACAGTTTAAGTC-GCTG----ACACTTGAACA-TG

Section 252

(18575) 18575 18580 18590 18600 18610 18620 18630 18648

Homo sapiens chromosome 13 NC_000013.11: 34882059... (17849) AGTGATAATGACCCATAATATAAACTAATTCATGCTCATAGATCCC-TCCAAGTAGGGATTTCGAGTAAATTAG

SARS-CoV-2 Reference Genome Shuffle No.1 (17616) GGTAAT--TGGCGAGATAACTAAAAAACATCAGACACTTAAAAGAGATAACAATTGTCGTTTAGATGTAGTTGG

Section 253

```
                                                           18649      18660     18670     18680     18690     18700     18710       18722
                                                   (18649)
Homo sapiens chromosome 13 NC_000013.11: 34882059... (17922) AGGCAATCTTTTTCAACCGAGCAACTTTTCTTAGAG-CACAGAACAACTGCCATCCTCTGCCTTGA-TGCGGGG
        SARS-CoV-2 Reference Genome Shuffle No.1 (17688) AAGCACGCGACAGAAATAGAGATGCATTGATCGCAGGCACATAGGAGC-GCTAAATAAATCGTGGACTCCGAGT
```

Section 254

```
                                                           18723  18730     18740     18750     18760     18770     18780           18796
                                                   (18723)
Homo sapiens chromosome 13 NC_000013.11: 34882059... (17994) AGCTGCAATTGCTGAAGGTAGATACGTTCACGACCTAAGTTAGAGTTTAGCCTTTAACCTTACCAAGGGTATCA
        SARS-CoV-2 Reference Genome Shuffle No.1 (17761) CCACTCAACAATTG--GTTTGA-ACTTGTATGAGAATAATTAGAATCTTTTAACAATACTTGACGTGCGGAGAT
```

Section 255

```
                                                           18797        18810     18820     18830     18840     18850     18860  18870
                                                   (18797)
Homo sapiens chromosome 13 NC_000013.11: 34882059... (18068) TTCCAACAGC---CATCTACCTCACCACCTAAAGCAGGAAAACG-AAATGTCTTTTCTTTTGTCATTG--GCTT
        SARS-CoV-2 Reference Genome Shuffle No.1 (17832) TGCCTCTTGAGTTGATCTGCCTTAAACCATAAACTTGTCCAGCATAAAACACTGATATATTTTAATTTACGGTG
```

Section 256

```
                                                           18871    18880     18890     18900     18910     18920     18930         18944
                                                   (18871)
Homo sapiens chromosome 13 NC_000013.11: 34882059... (18136) TCTACTCCATTAAAACTATAGAGAATA--GTTGGTATAACTGTAAAATATAGAGCCTCTTCCCAAGGTTAAAAT
        SARS-CoV-2 Reference Genome Shuffle No.1 (17906) AGTAAATCGTAGTGTCTATAAGTGATACCGTAGGGATACTTCGTGGGTATTTAGCTGTTTTCTATGCATAAGTC
```

Section 257

```
                                                           18945 18950     18960     18970     18980     18990     19000           19018
                                                   (18945)
Homo sapiens chromosome 13 NC_000013.11: 34882059... (18208) GGGTTGTTATATTTTGTCTTT-CACACACACAAAAAAAGACCTCCTG--CTTCTTTTTCCTTCAAGCAGGGAAAA
        SARS-CoV-2 Reference Genome Shuffle No.1 (17980) AGGATGGAATCGGTGAACTCGACACCATTTCTGCTAAAAACTACTGATCTAACTTGTCGTGCGAGTAGGACATC
```

Section 258

```
                                                           19019      19030     19040     19050     19060     19070     19080       19092
                                                   (19019)
Homo sapiens chromosome 13 NC_000013.11: 34882059... (18279) GTGGCAAGTCTGTCTTGAACCATTGTGCCTTTCACATTCTCCTCCCCAAAGCCATTTAAGCTCCTTCGACCTGC
        SARS-CoV-2 Reference Genome Shuffle No.1 (18054) CTACGTTTTCATCATT--ACAATTCTTCCTAAC---TTGAGCATGGTGTTGCTTATTTTGACGTTTCAAAATGC
```

Section 259

```
                                                           19093  19100     19110     19120     19130     19140     19150           19166
                                                   (19093)
Homo sapiens chromosome 13 NC_000013.11: 34882059... (18353) TTCTCTATTTCAAGAGGTCTTTTCTTT-ACATTTGCATAACTTAA-A----GGTATCTATCTCCATAACAAAGA
        SARS-CoV-2 Reference Genome Shuffle No.1 (18123) AACTC-ACTTAAGTATCACCATACTCGCACATGGGCTGTCGTTAAGAAGTTGGGGTAGAAATCCA-AGTAGTGA
```

```
Section 260
                                              (19167) 19167        19180     19190     19200     19210     19220     19230  19240
Homo sapiens chromosome 13 NC_000013.11: 34882059... (18421) AACCCATTGCCTGGAAGGTGTGTTGGAGTTTCCACTTCTCACCTA-----ATAAAGACTTCTTGCAGTTT--TT
    SARS-CoV-2 Reference Genome Shuffle No.1 (18195) GACCCCCTGTATTGACGCCGACAT--AGTCTACCTGTTTCATTTAGTGTTGTAAGGTCGTCTTATCGTCTACCT

Section 261
                                              (19241) 19241   19250     19260     19270     19280     19290     19300      19314
Homo sapiens chromosome 13 NC_000013.11: 34882059... (18488) AAATAATGAAATGTGAGAGACTGGGGAGATTACACTCTGTCATCCATGCCTCCGGCTTTTTTCAAAACC--CAT
    SARS-CoV-2 Reference Genome Shuffle No.1 (18267) TCCTAACACAAC-TGTCAGATGAGCGCGAATATACAAA---ACTCAAGA-TCCAATTAGTATAAAATTCACCTT

Section 262
                                              (19315) 19315 19320     19330     19340     19350     19360     19370        19388
Homo sapiens chromosome 13 NC_000013.11: 34882059... (18560) GGGTCTGG-GAAATCTGATACAATTCTGCATAGTCTGCAAGA----CATAGTGCTATGCATTTATTGCATTCAA
    SARS-CoV-2 Reference Genome Shuffle No.1 (18336) GAATTTGGTGCCATCTCAACGTCAGCTGTACCGCATGTAATATTGTCCGAATCCTTGGGCGTTGTTCCATTCCT

Section 263
                                              (19389) 19389       19400     19410     19420     19430     19440     19450   19462
Homo sapiens chromosome 13 NC_000013.11: 34882059... (18629) GTTGTTTTCATCC----CA---------ATTGAGTGCTG--ACTGTGTAC-CTGGCTCTGTCTGTCAA---TGAA
    SARS-CoV-2 Reference Genome Shuffle No.1 (18410) ATTTAATAAATCCTTGGCATGTATTCAATTTGGTTCTGTTACCCTGGCACTGAAGATGTATGCTAACATTGTA

Section 264
                                              (19463) 19463 19470     19480     19490     19500     19510     19520        19536
Homo sapiens chromosome 13 NC_000013.11: 34882059... (18685) ---CAACAA-----------AGACAAAACTTTCTCCCCTCATCCAACTAGTAAAAGGAG------CACCTGTCC
    SARS-CoV-2 Reference Genome Shuffle No.1 (18484) ATTCAACAAGGTACGTTTTGAGCGGACAAACTCTACGTTTATTCAT-TATAATAGGTCTGTTATTCAATTGGCC

Section 265
                                              (19537) 19537        19550     19560     19570     19580     19590     19600  19610
Homo sapiens chromosome 13 NC_000013.11: 34882059... (18739) -TGAACAAAATGAGGAAGTTAAATGTGTATTAT---ATAGCTGT-GAATACTATTAAAAAAAAAAAAAGGAAAAA
    SARS-CoV-2 Reference Genome Shuffle No.1 (18557) GTAAATAATATTCCTCAGATTTAAGCGTCATGTGGGACGCAAGTCGAATACTTTTCTCGAAACCGAACCAAAA-

Section 266
                                              (19611) 19611   19620     19630     19640     19650     19660     19670      19684
Homo sapiens chromosome 13 NC_000013.11: 34882059... (18808) GGGGAGATTTGGAGCTAGGGTAGAGAGAGCTGTAAGTCATTCAGGCAAAGTTCC------AAAGGAAGGAAGAG
    SARS-CoV-2 Reference Genome Shuffle No.1 (18630) GACCAATTTGCGAGCGGTGGTGAAGCCTGCTCTTGCTACATCCGACAAACAGCGTGGGTATAAGGGACGACGTG
```

Section 267

(19685) 19685 19690 19700 19710 19720 19730 19740 19758

Homo sapiens chromosome 13 NC_000013.11: 34882059... (18876) TGTTACAGGCAAAGACTAATGGAAGGAACAGGCC---T-G-GAGACTGGAGCATGCCTGGGGTTT--TAGAAGA

SARS-CoV-2 Reference Genome Shuffle No.1 (18704) TTATAATCTACACAATTTGTGGTTTAAACCGTCCCTCTTGTGATACTTTTTTGTTTATTATATCTCTTATATGA

Section 268

(19759) 19759 19770 19780 19790 19800 19810 19820 19832

Homo sapiens chromosome 13 NC_000013.11: 34882059... (18943) ACTGCAAGGAAGCTAA-AGATAAGAAC-CCAGAAACTACAGCACTTAGA---ACTCCTATGACTAA-TATATGA

SARS-CoV-2 Reference Genome Shuffle No.1 (18778) A--GCAACAATTCCATGAGCTGAGTGCTCCTTCTGATAACGTATTCAAACAGACGCGTTGGAATAAATTTAGGA

Section 269

(19833) 19833 19840 19850 19860 19870 19880 19890 19906

Homo sapiens chromosome 13 NC_000013.11: 34882059... (19011) TT-TCGCATTTTCTTTTTTTATTTTATTTTAT---------T------TTAT-TTTATTTTATTTATTT-ATTT

SARS-CoV-2 Reference Genome Shuffle No.1 (18850) GTCTTGCAAAGCTAGTAAATATCTGGACCTGTCCAGCACCGTGCGCGCTGATGTTCAACTTGTCGACTTCAAAA

Section 270

(19907) 19907 19920 19930 19940 19950 19960 19970 19980

Homo sapiens chromosome 13 NC_000013.11: 34882059... (19067) ATTTATTA--TTATTATACTTTAAGTTTTAGGGTACATGTGCACAATGTGCAGGTTACTTACATA-TGTATACA

SARS-CoV-2 Reference Genome Shuffle No.1 (18924) CAATACTAGCTCAATGTACTG-ATGACATAGGTTCCAAGTATAACAAGCTAAGGATAAGACCTTTCTTTCAACA

Section 271

(19981) 19981 19990 20000 20010 20020 20030 20040 20054

Homo sapiens chromosome 13 NC_000013.11: 34882059... (19138) TGT-GCCATGCTGGTGTGTTGCACCCACTAACTTGTCATCTAGCATTAGGTATATCTCCCAATGCTATCCCTCC

SARS-CoV-2 Reference Genome Shuffle No.1 (18997) GAAAGCTAATCTGCATCTACCCCGTCAGTCCCTTTTC-TCTA-CGTAAGTTAAAAGTCC--ATAATTGTACTTA

Section 272

(20055) 20055 20060 20070 20080 20090 20100 20110 20128

Homo sapiens chromosome 13 NC_000013.11: 34882059... (19211) CCCCTCCCCCCACCCCACAACAGTCCCCAGAGTGTGATGTTCCCCTTCCTGTGTCCATGG---GTTCTCATTGT

SARS-CoV-2 Reference Genome Shuffle No.1 (19067) GACATCGTAATAGTAGTTATCAATATGTTTAAAGAGATTTTCCCCA-CTTATAACGGAGGCATGTTGCAACACG

Section 273

(20129) 20129 20140 20150 20160 20170 20180 20190 20202

Homo sapiens chromosome 13 NC_000013.11: 34882059... (19282) TTAATTCCCACCTATGAGTG---AGAATATGCGGTGTTTGGT-----T------TTTTGTTCTTGC-GAT----

SARS-CoV-2 Reference Genome Shuffle No.1 (19140) TCAATTGGCACG-ATTTGTGCATAGGCTTTTCGTTATTTGGTAAACGTTAATTTTTTTGTGCGTGCAGATTTCG

```
                                                                                    Section 274
                                                    (20203) 20203   20210     20220     20230     20240     20250     20260     20276
Homo sapiens chromosome 13 NC_000013.11: 34882059... (19337) ---A--GTTTACTG-AAAATGATGATTTCCAATTTCATCCATGTC--CCTACAAAGGACGTG--AACTCATCAT
      SARS-CoV-2 Reference Genome Shuffle No.1 (19213) TCAAAGTTTTATAGTAGAACTAACCTCTGCAATTTGAAAAAGATTAGCCTTTGATGACTGTGCCAAATTATCAT
                                                                                    Section 275
                                                    (20277) 20277        20290     20300     20310     20320     20330     20340   20350
Homo sapiens chromosome 13 NC_000013.11: 34882059... (19401) TTTTAAGCTTTCTTGACCCTGTTGCATCTGAGATC-TCTAAGCTGTCTTAAGAACTCCGTAGCACACAAAAGGA
      SARS-CoV-2 Reference Genome Shuffle No.1 (19287) TGGGATTTATGATATAGCTTGTTATGTGTTACATAGTAGGCGCTCACGAACAAATTGCAAGGCACAAACCTAAT
                                                                                    Section 276
                                                    (20351) 20351    20360     20370     20380     20390     20400     20410     20424
Homo sapiens chromosome 13 NC_000013.11: 34882059... (19474) GTTAGAAGCATCCTTCAGCATT---CCCTGAAGCTTAGGCCAGTGTATTCTGAGCAACCATTTCTTCTAAATGC
      SARS-CoV-2 Reference Genome Shuffle No.1 (19361) AATACGAGCACAGATCTCGATTTATCTTTGACTCGTAAGCCTGATGGATATCGTAAACTCTTTTACTTGGTTG-
                                                                                    Section 277
                                                    (20425) 20425 20430     20440     20450     20460     20470     20480     20498
Homo sapiens chromosome 13 NC_000013.11: 34882059... (19545) ATAGACCCTCATGAAACCTTTT-TTGGATCTATTTTTGTTTTTCTTACCCCTGGCTGTGTTCGTAATTGCTTCA
      SARS-CoV-2 Reference Genome Shuffle No.1 (19434) -TAGGTCCAGATTGGTCGTGCAATTTGAATTACTCGCTTGACGATAACGAAGGGTTTTGATACTAATAGGTG--
                                                                                    Section 278
                                                    (20499) 20499      20510     20520     20530     20540     20550     20560   20572
Homo sapiens chromosome 13 NC_000013.11: 34882059... (19618) CGATGACATCTCATTGACAGATGCAAGGCTGCTGACACCACGCCAGACTC----TCTGA---AATGAAATCATT
      SARS-CoV-2 Reference Genome Shuffle No.1 (19505) -GTTACGATTTGGTGGATACA-GCGTCGATGCGCACGTCTCGCGTTAATCTTGTTCTGGTTCAAGTAAATGAGT
                                                                                    Section 279
                                                    (20573) 20573   20580     20590     20600     20610     20620     20630     20646
Homo sapiens chromosome 13 NC_000013.11: 34882059... (19685) TGACACCTGAGGGGATCT------------GGGCCTTCTGGACTCTTCATT--CCTGGCTGAGAACTCTGTGCC
      SARS-CoV-2 Reference Genome Shuffle No.1 (19577) AGA-ATTTTAGGTCCACTATCGTACGCGAGGGATATAATGGATAATAAATTGGACTTGGAAAGTTTTAGTTGTT
                                                                                    Section 280
                                                    (20647) 20647        20660     20670     20680     20690     20700     20710   20720
Homo sapiens chromosome 13 NC_000013.11: 34882059... (19745) CTACTTTGAGGGTTTGAACCCTCAGGGAAGAAGCTAACTTAG-GAATTCTCGTTGCCCTCATTGCTCATCTCTG
      SARS-CoV-2 Reference Genome Shuffle No.1 (19650) CTTATTGTATGGTAGATATGCCTCGAGTAAAAGTACTTTTAGCGCGTTCCATGGGTGTAGAATGAACCTTTTAG
```

Section 281

```
                                                      (20721) 20721    20730           20740           20750           20760           20770           20780           20794
Homo sapiens chromosome 13 NC_000013.11: 34882059... (19818) AGCTACCTTAGCACCTCAAAAAGGGCCCGCAAATCCTGTCCCTTTCTTTATTCACAAACCTCAAATTTTGGTGA
     SARS-CoV-2 Reference Genome Shuffle No.1 (19724) ATATATCTTATTATCT-AATGCCCATCCTCGATTACTCTACTTCGCAGATGTGAAAATATCCGTTTGCTGATCA
```

Section 282

```
                                                      (20795) 20795 20800          20810           20820           20830           20840           20850           20868
Homo sapiens chromosome 13 NC_000013.11: 34882059... (19892) C---AGAGTGCAGGTGCCACAGGG-ACTTA-------GAGTCATGGTTGAACAAGGAAGGGCTGTA---CGTTC
     SARS-CoV-2 Reference Genome Shuffle No.1 (19797) AAAAAGAATGTACTTGGCAAATATTACTAAATATATGTAATCTTGATTTAATAT--AAGCGCGAAAAATCGCAC
```

Section 283

```
                                                      (20869) 20869      20880           20890           20900           20910           20920           20930           20942
Homo sapiens chromosome 13 NC_000013.11: 34882059... (19952) AGATGCCTACAGAGGCTACTCCGGTCACATATGTCAGCGAGAAGTTAGATAAGTGAGATCCACAGCAATTGTGA
     SARS-CoV-2 Reference Genome Shuffle No.1 (19869) ATATCAATTAATAACTTACGCTTAACGT-TACGGTTGTTTGTATATTGATCTTGGATGGGGATGGAGACAGTGA
```

Section 284

```
                                                      (20943) 20943  20950           20960           20970           20980           20990           21000           21016
Homo sapiens chromosome 13 NC_000013.11: 34882059... (20026) GCTTGTGTGTCTTTAGGGGAGAGCGAATTCTCAGATCTAGCCAGTCATTGAAATGTGGAAATAACAGTCCAGTG
     SARS-CoV-2 Reference Genome Shuffle No.1 (19942) AACTTACTATATTTGGGTTATTCCTATACCTAACAGT---CCAG--ATTTAAGTGGGGAACTAAATAATAAGTT
```

Section 285

```
                                                      (21017) 21017        21030           21040           21050           21060           21070           21080  21090
Homo sapiens chromosome 13 NC_000013.11: 34882059... (20100) TTTCCAAATCTTATCGTAAAAGACAAGACAGCAATCCA--AATATTTGTATGAAATTTCCTGATGTTTTAATGC
     SARS-CoV-2 Reference Genome Shuffle No.1 (20011) TTTCCTAATTCTATCGGGTGAACCCGGTCGGTGAAGAATTAACCTTAATCTTAACCAGCGCCCAGTCCCTATTG
```

Section 286

```
                                                      (21091) 21091    21100           21110           21120           21130           21140           21150           21164
Homo sapiens chromosome 13 NC_000013.11: 34882059... (20172) TGACAACTAGTAATCCCCCCAAAACAAACAAACAAACAAACAAACAAAACCACTGTTGAATTACACCAAACAAG
     SARS-CoV-2 Reference Genome Shuffle No.1 (20085) TTACTACGTGTCGTTGTTTTTCATGTTATAA-CATACAATTA--CGTATCTGGCTGTGAGTTACAT-ATGTAAA
```

Section 287

```
                                                      (21165) 21165 21170          21180           21190           21200           21210           21220                 21238
Homo sapiens chromosome 13 NC_000013.11: 34882059... (20246) AGCTATCTGTAGCCTGGGT---TCTGCCTGGGGGCTCTCATTCTATGA-CTTCTGAAACAATCATCTTTGATCC
     SARS-CoV-2 Reference Genome Shuffle No.1 (20155) TGAGATGTGGTGACTCAACAAATATGACAGGATAC-CTCCTGGAATAATCGAGAGTGAGGAAGATACTTGCCAG
```

```
                                                                    Section 288
                                                   (21239) 21239      21250          21260          21270          21280          21290          21300        21312
Homo sapiens chromosome 13 NC_000013.11: 34882059... (20316) TGTCACTCATTGTCCATTTCATGGTGTT---------TGTGGGAGTTTAAAATTTTTTAAATAAGATGTGGA-A
        SARS-CoV-2 Reference Genome Shuffle No.1 (20228) TGCGAGTGATCCAGCAAGACATATATTCAAATTCCAATAACGGAGTCGTGCCCTGTCAAGAGCAGCCGCCTACA
                                                                    Section 289
                                                   (21313) 21313  21320          21330          21340          21350          21360          21370             21386
Homo sapiens chromosome 13 NC_000013.11: 34882059... (20380) CAAGGAT----GGCAAATAATTTCTAAATTGCATGTCACCTCTGATTTATTAGCAGTGGCTGCCGTGAGCACTA
        SARS-CoV-2 Reference Genome Shuffle No.1 (20302) CTACGACATAGGGCCAATTGACACATATTACTATCTCGGCACAGG---ATTGGCATAGGGTT---TAAGCATAC
                                                                    Section 290
                                                   (21387) 21387         21400          21410          21420          21430          21440          21450  21460
Homo sapiens chromosome 13 NC_000013.11: 34882059... (20450) C-ATTTAGGATTCTGAGGCT-GTTTCTAGGTCTGCAGAAAAAGCATGTGTTAAATTATCAATGTTGAATGATGG
        SARS-CoV-2 Reference Genome Shuffle No.1 (20370) CTAATGAAAACATTGATAATAGTTGCTAC-TCTTCACATTCTACATTT--TCCGTGCTCTTAGTAGTACGGTTA
                                                                    Section 291
                                                   (21461) 21461    21470          21480          21490          21500          21510          21520        21534
Homo sapiens chromosome 13 NC_000013.11: 34882059... (20522) TA--CACAAGGCCTGTATTTGCAA--TTCCCCAACCAAACAATCCCCATGAAGGTTAGTGTTGGTGCTTGAATA
        SARS-CoV-2 Reference Genome Shuffle No.1 (20441) TGAGCAAAAGGCGT-TAAATGAAAGCTTGTTGTGTTAAGCAGCAGAGTTACAGTGGAACCCCGCCATATTAGGA
                                                                    Section 292
                                                   (21535) 21535 21540          21550          21560          21570          21580          21590             21608
Homo sapiens chromosome 13 NC_000013.11: 34882059... (20592) AATGATTTCAG--TTACTCCTCCTCCTTTCTTA---GCTCTTTAATTCAAGTGTGCTTCTCTCATCTACTGTTC
        SARS-CoV-2 Reference Genome Shuffle No.1 (20514) TGTTATTTAAGTATTTGTTCTCAACAATTTAAAAAGGCTTTTAATAGCAAGTCCACTGA-CAAGCCCACAGGTC
                                                                    Section 293
                                                   (21609) 21609      21620          21630          21640          21650          21660          21670        21682
Homo sapiens chromosome 13 NC_000013.11: 34882059... (20661) CCCAAGGCCTGGGAAAATGGAGCACTGACT-TT---CAGCGAATGGCGGAGGCGGGGGTGGGGTGGGGGTCCTG
        SARS-CoV-2 Reference Genome Shuffle No.1 (20587) GCA---GCGTAGGTAAGTC-AGATCCCACTATTACACATTGAGTTAATTAGATTACCCGAGTATAACGGAAACA
                                                                    Section 294
                                                   (21683) 21683  21690          21700          21710          21720          21730          21740             21756
Homo sapiens chromosome 13 NC_000013.11: 34882059... (20731) CCAGGTC-ATCAGGTGTCTTTGCTTATAACTCATGAGGCATAAATCAAGCCAATGCATTCAACACTACAGGAGG
        SARS-CoV-2 Reference Genome Shuffle No.1 (20657) CCGGCTCCAAGTGGTACATTCGCGTA-AGCT-----GGAATAAT--AAGTAAGATC-TTATACAATTGGTGCAT
```

```
                                                                Section 295
                                              (21757) 21757        21770     21780     21790     21800     21810     21820   21830
Homo sapiens chromosome 13 NC_000013.11: 34882059... (20804) AGAGTAACTGATCTATCACTCAGACACCTGTTATAATTTTTTCAGATTTCTTGTGATGTAAAAATATCAGAGCA
   SARS-CoV-2 Reference Genome Shuffle No.1 (20722) AGATTATCTGGAATATCAAACGCTCGTACGTCCAGTTAAGCAGACATAAGTCAATACTTAACTATATCAGCCCA

                                                                Section 296
                                              (21831) 21831   21840     21850     21860     21870     21880     21890       21904
Homo sapiens chromosome 13 NC_000013.11: 34882059... (20878) AAGTTGTACTACTCTAGACCAATCAGGCTCCAGATGATAAATATTTTAATCTGAGTTGGTATTCTAGCTCATTT
   SARS-CoV-2 Reference Genome Shuffle No.1 (20796) ATCTA--AC--CGAGAGTACACTCAAGCGACCG--GGCAGATAATTTTAGGTTAAAATATATTATG---CATTA

                                                                Section 297
                                              (21905) 21905 21910     21920     21930     21940     21950     21960         21978
Homo sapiens chromosome 13 NC_000013.11: 34882059... (20952) TAATATATCAAAATG---AGTTTACTTTGGAATAAAAATGACCTTAAATACAAATTTCAGATACTTTATTTTAA
   SARS-CoV-2 Reference Genome Shuffle No.1 (20861) GGCAAGAGCGAAGAGTACAGGTTAGCTTTGTCCAAGGAGCCCGCCATAGAATGATTTTTAATAAATTATAGTGG

                                                                Section 298
                                              (21979) 21979      21990     22000     22010     22020     22030     22040     22052
Homo sapiens chromosome 13 NC_000013.11: 34882059... (21023) AGAG--AAACAATAAAA--ACCAACACTGGAAAACAACCTACAACATTTAATTATTTTTAGTGTTTTAGTTCTT
   SARS-CoV-2 Reference Genome Shuffle No.1 (20935) GGGGTTAAGCATTTCTGGTATCAACTTTTGGCAGCACCTTATT-CTGTTACCAGTGAAAATATTTTGACGTAAT

                                                                Section 299
                                              (22053) 22053  22060     22070     22080     22090     22100     22110       22126
Homo sapiens chromosome 13 NC_000013.11: 34882059... (21093) GATAGGGAAACTGAACCCAAGAAAGTAATT-GAAAAATGTTATCCTCCACAAGTATATCTTAGAGGTAAGAGCA
   SARS-CoV-2 Reference Genome Shuffle No.1 (21008) CATAT---AACGGATGCCCAGGTGCTAGTTCGTGTTTTGTTGTCGTTTGATCGATTTTACTAAATTTAAATTAA

                                                                Section 300
                                              (22127) 22127        22140     22150     22160     22170     22180     22190   22200
Homo sapiens chromosome 13 NC_000013.11: 34882059... (21166) TTTGA-------ATGTTAAGAAATATATATT---TTTAAACGTCC-ACTTATGTTTAC----TTTG-GAAGGA-
   SARS-CoV-2 Reference Genome Shuffle No.1 (21079) TTACACCAAATCCTTTATTGAAATCAATTTTGCAGTCAAACAAGAGAGATATTCGTCCCAATTTAGCGAAGTTG

                                                                Section 301
                                              (22201) 22201   22210     22220     22230     22240     22250     22260       22274
Homo sapiens chromosome 13 NC_000013.11: 34882059... (21223) ATTTCTTATAGAAAAATGCTAGCAAATAGATAAGTTCTTACCTA--CTAATTCACTCTGTTTTCTCAT------
   SARS-CoV-2 Reference Genome Shuffle No.1 (21153) ATTTTTTCAAGTAAGGTTTTAACAAATT-ATAATTTCATGCGAAAACGATTGGAATAATTATGCGGATGGTGGA
```

```
                                                                                 Section 302
                                                       (22275) 22275 22280     22290     22300     22310     22320     22330          22348
Homo sapiens chromosome 13 NC_000013.11: 34882059... (21289) --AATCTTCTGACAAAAT-TTCAGTCAAAGTAATCAATG-GTCTGCAAGCTCCCAGTTTCCAACAATTGCTAAC
        SARS-CoV-2 Reference Genome Shuffle No.1 (21226) CCAAAACACTCATATAATGTTCAGATACAATT-TCTGTGCGTATAAGACAACTTTGTTTTGCACCGTAGACACA
                                                                                 Section 303
                                                       (22349) 22349      22360     22370     22380     22390     22400     22410      22422
Homo sapiens chromosome 13 NC_000013.11: 34882059... (21359) TCAGTA-ACT-AACATCAACTACTTGAGTCAATAGAATAGGTAGTAGTTGG----CCTATATGTGATTTTAGAC
        SARS-CoV-2 Reference Genome Shuffle No.1 (21299) TAGGCAGACTGAACATATA--AATTGAATCTACTCGGAACTTCTCCGTTATGCTTCCAAGATCTGATTTTTCGT
                                                                                 Section 304
                                                       (22423) 22423   22430     22440     22450     22460     22470     22480        22496
Homo sapiens chromosome 13 NC_000013.11: 34882059... (21427) CACATATAGAGGATTTGA-TATAAAAAAATCTCCCCACAATGCCCCAAATCTCTTCTTCA--TCAACACGATGT
        SARS-CoV-2 Reference Genome Shuffle No.1 (21371) CACTCGCATAGGACCCGCGTATTAAATCAGTCTTTAATAAGGCA--AAACATTTACTTCAGGTATAGAAATCTT
                                                                                 Section 305
                                                       (22497) 22497        22510     22520     22530     22540     22550     22560  22570
Homo sapiens chromosome 13 NC_000013.11: 34882059... (21498) TGATGCCTGAAACTCAGCAATCAGACTCCTTCTGAATTTTTATAATTCATTATTGCAATATTGTTTAATTGGAG
        SARS-CoV-2 Reference Genome Shuffle No.1 (21443) TGCGGTCAGGAAACTAATAACTACTTGTTTTTTGAAGCCTGGTTATTCACGTCCGCCATTATCCATCAATGCTC
                                                                                 Section 306
                                                       (22571) 22571    22580     22590     22600     22610     22620     22630      22644
Homo sapiens chromosome 13 NC_000013.11: 34882059... (21572) TATCAAAATG-TC-ATTGTCTTTGAGACTATTAAA--AAATAGTGCTTCAATA--TAAACTATTTATTGTCTAC
        SARS-CoV-2 Reference Genome Shuffle No.1 (21517) AGTGAGGTTTATCGATTAACATCGTAAGTATCCATTCACATATGATTTGACTTGCCAAAGAGATATTTGATTT
                                                                                 Section 307
                                                       (22645) 22645 22650     22660     22670     22680     22690     22700          22718
Homo sapiens chromosome 13 NC_000013.11: 34882059... (21640) AGTACATTCTTCCATTTATTTGATTCTGATTATATAGATTTCTTCCAATTGTAACTTGTAGGTAGCTAATGGCA
        SARS-CoV-2 Reference Genome Shuffle No.1 (21591) AAGCCAAAGTGG--TTAACGAGTAAAACATGCAATAGCCTACAGCGAACATTCTTGTTACGAATATTAAGCGCA
                                                                                 Section 308
                                                       (22719) 22719      22730     22740     22750     22760     22770     22780      22792
Homo sapiens chromosome 13 NC_000013.11: 34882059... (21714) TTGCAAAATA----ATTTTTGTCCATAGACATGCAACTGAACTGTTCTCCCGCTGTGGAAAAAATAGTTTTGTC
        SARS-CoV-2 Reference Genome Shuffle No.1 (21663) TTAAAGAAGACAGCACTTTTGT---TAGAT-TACTTCTTAGAAGTTA-CAAGATATCAGAACATCAGGTGTGGT
```

```
Section 309
                                                           (22793) 22793  22800     22810     22820     22830     22840     22850     22866
Homo sapiens chromosome 13 NC_000013.11: 34882059...       (21784) TCCATTTGCACATTTATT-TCAATAGTCTTGATCTAG---AAATGTGTCTGTTTA---ATTGTTTGTTTTTGGA
SARS-CoV-2 Reference Genome Shuffle No.1                   (21732) AC---TCGCTCATGAATTATCTAAGTTCTTACCCGAGCGAAGAACAGCCTGCTTATATAATGCTCGGGATTATG

Section 310
                                                           (22867) 22867        22880     22890     22900     22910     22920     22930 22940
Homo sapiens chromosome 13 NC_000013.11: 34882059...       (21851) TTTTCTTTTGAATTGCTTA------TAATATCT-GCCTAGTTCTCTCACTGAACAATGAGTTAAGTAAGATCTT
SARS-CoV-2 Reference Genome Shuffle No.1                   (21803) TCCGTGCTTGAAATTCGAAAATCAGTGATATTACGGAGAATTCTCGTCTACCTCAAGAAATCTAGTTTGTTGTA

Section 311
                                                           (22941) 22941    22950     22960     22970     22980     22990     23000     23014
Homo sapiens chromosome 13 NC_000013.11: 34882059...       (21918) T-AATACATGTTTGC-----TTTATATCTGTA--TGAGTCATGATTTTACTTCTTTTTGAAAATTATCTTTTAA
SARS-CoV-2 Reference Genome Shuffle No.1                   (21877) AGAGTACATGTTAGCCGTGTTATACATCTATACTTAAGTATTGGAATGTGGTCAGTTTCAA--TCGCGAAGACA

Section 312
                                                           (23015) 23015 23020     23030     23040     23050     23060     23070         23088
Homo sapiens chromosome 13 NC_000013.11: 34882059...       (21984) TGATAT---TTTCCTATTTGATCAATATAAATTCTTGACAAGTAAT-TCCAATATTTATCCACATTTATGAAAA
SARS-CoV-2 Reference Genome Shuffle No.1                   (21949) TGAAATCTCTTTTCAGTGCGGTCACCACTGAAACTGCATACATCAGCTGTACCCTTTCGATAGAGTTCTCTCCT

Section 313
                                                           (23089) 23089      23100     23110     23120     23130     23140     23150   23162
Homo sapiens chromosome 13 NC_000013.11: 34882059...       (22054) AAGACCCTTTGAAAAAGTAAGAAACTTGATTAATAATTT--GCAATTTTATTTTATTCTACCAAAATGTTGTT-
SARS-CoV-2 Reference Genome Shuffle No.1                   (22023) GAGATCGGTTTCTAATTACGCGAGATAGATAGAGCAGTTAAGCCATGCTAAATTTTGCGCACAGAGAATAGTCA

Section 314
                                                           (23163) 23163  23170     23180     23190     23200     23210     23220     23236
Homo sapiens chromosome 13 NC_000013.11: 34882059...       (22125) -------GATAAATGTGTTTCTGAAAAGTATATTCCACAGCTATGTGTCCTGACCTATTTTATCCTTAAAATCT
SARS-CoV-2 Reference Genome Shuffle No.1                   (22097) GCGAGGGGATCTACGGGTTT-TGAAGCTTAGGCGAGTGAGCTTTCATTTTTGGCCTGTGAGAGCGTTTCCAT-T

Section 315
                                                           (23237) 23237        23250     23260     23270     23280     23290     23300 23310
Homo sapiens chromosome 13 NC_000013.11: 34882059...       (22192) TATTTAACTTCAAATTTTGGAAAATGTAATAAGGAAA--ATAAACAATTAAAAAGCAATTTAAAAAGTAATTAT
SARS-CoV-2 Reference Genome Shuffle No.1                   (22169) CCGTTGTTTCTAACTTAAGGCTTCTGGAGGAAAGTCACCTTAAACAAAGTTAAAGCTATATCTA--GCATTTTT
```

Section 316

```
(23311) 23311 23320 23330 23340 23350 23360 23370 23384
Homo sapiens chromosome 13 NC_000013.11: 34882059... (22264) TGAGCACGTGCTCTATTCCAGACTTGCTCAAATATCTGACCAAGGATCCCGGTATGCGTCTCACTTTTGAAAAG
SARS-CoV-2 Reference Genome Shuffle No.1 (22241) CCTCGACGGGTTAGGTTCAACATTCTCTCGAGCAT-TGAAGA----------GTCTGAAATTCTTTGTTATACTA
```

Section 317

```
(23385) 23385 23390 23400 23410 23420 23430 23440 23458
Homo sapiens chromosome 13 NC_000013.11: 34882059... (22338) CCCTTTTAAAGTCCATCTAAGAGCAAATTCATTTCTCTAGCAT-CTACTGAAAAACAGCCAGTCAAAGAATATG
SARS-CoV-2 Reference Genome Shuffle No.1 (22305) TAGTTTTTGATGCCAATTCCGCTTAAATTGAGATATACAGCAGACTTCTACAATAAAATGTGCTTAAGATAATC
```

Section 318

```
(23459) 23459 23470 23480 23490 23500 23510 23520 23532
Homo sapiens chromosome 13 NC_000013.11: 34882059... (22411) TTCCACA----CCCTCCAAGTGTTTCTCCAATGTAACAGCCACTGTCAGCAGGGTGACTGGGCCCCTTCCCCAG
SARS-CoV-2 Reference Genome Shuffle No.1 (22379) CTATAAGTAGGCAATCTTAGAATATTTGCAAGGGGATAAAGACACGCTACAATCTG------CCCCTTCTGAGG
```

Section 319

```
(23533) 23533 23540 23550 23560 23570 23580 23590 23606
Homo sapiens chromosome 13 NC_000013.11: 34882059... (22481) GCTGTCACGTCTGATGCTTCCTTCAGTGACCCGGCCTTCTAATTCAGCTTAACCCTTCTCCCT-TCAACCT--C
SARS-CoV-2 Reference Genome Shuffle No.1 (22447) CCTATG--GACAAAAACATGAC--CATCTTCGCGTGTTTAAAAACATAATACACCACCCTGCTTATAAAGTATAC
```

Section 320

```
(23607) 23607 23620 23630 23640 23650 23660 23670 23680
Homo sapiens chromosome 13 NC_000013.11: 34882059... (22552) AGCCCTGTCACCAGGACAGCCTCTCATTCCACTCAGTCCTGCCTTCCAGTGG-GAATCGGATGAGCG-ACTCTA
SARS-CoV-2 Reference Genome Shuffle No.1 (22517) ATACCT--CACTATGAGTTCGAATGAACTAAGTTTCATGAGCTTTACACTCAAGACTTTTTTGTGAATACAATC
```

Section 321

```
(23681) 23681 23690 23700 23710 23720 23730 23740 23754
Homo sapiens chromosome 13 NC_000013.11: 34882059... (22624) CATCCATGG-TGGTCAAGGAAATGGGTTGCCGTGATGT--GGGAGTGGAATTCGAGGTGCAGACCTT----CTA
SARS-CoV-2 Reference Genome Shuffle No.1 (22589) CTTTCATTTCTTCCCAATTATTAGAGCTATTGATCTGTGCGGTAGTGGGATACTCCGGGACGGCCATGAGCCCT
```

Section 322

```
(23755) 23755 23760 23770 23780 23790 23800 23810 23828
Homo sapiens chromosome 13 NC_000013.11: 34882059... (22691) TTGTTTAGAAACAGAGCTATTGAATCCCCCAGGGTAAACCAAAACTAAG-AGTGCTGAGGAAGACCTAGAGTCA
SARS-CoV-2 Reference Genome Shuffle No.1 (22663) TTCCTTTGATTAATTGTTAGTGTAGTATTAATAACAAA--AAGACTAAGTAGTGATATGTACTACCACAAACAA
```

```
                                                                     Section 323
                                                   (23829) 23829      23840      23850      23860      23870      23880      23890     23902
Homo sapiens chromosome 13 NC_000013.11: 34882059... (22764) GAGGCCGTAACACTCCCATGAATGTAGGAAAGTAACTTGAGAACAGTTGACGTAGT---AAATAAAAGAGGAGC
   SARS-CoV-2 Reference Genome Shuffle No.1 (22735) TACACTGTAATCTTTACGAGTAGGCATTTAA--AAACTGATTCATATATACGTAGTTGTGACTATACCGGACAC
                                                                     Section 324
                                                   (23903) 23903  23910      23920      23930      23940      23950      23960         23976
Homo sapiens chromosome 13 NC_000013.11: 34882059... (22835) TTTTGCATGATAGCAGAATAGTCTAGAATCAGCAGTGGGAGCAAGATAAGCAAGATGGCAGCACTACTCTCCTG
   SARS-CoV-2 Reference Genome Shuffle No.1 (22807) TCGTAGATGA--GCTGA-TAGTCATTGACGATGCGTT--ATCAAAGTCTTCA-GAT--CATAAGTTATATCAAA
                                                                     Section 325
                                                   (23977) 23977        23990      24000      24010      24020      24030      24040  24050
Homo sapiens chromosome 13 NC_000013.11: 34882059... (22909) CCCTAGAGGGATTACAGTCTTGAGAGGGAGACAGACCAACAAACAAGCAATTACAAT-ATTGTCTGATCTGTAG
   SARS-CoV-2 Reference Genome Shuffle No.1 (22873) CC----ACCGCATATA--CTT----------CCGTACACCATAGAAGTAGTAGTAATCATTGCGTTAATTTATG
                                                                     Section 326
                                                   (24051) 24051    24060      24070      24080      24090      24100      24110         24124
Homo sapiens chromosome 13 NC_000013.11: 34882059... (22982) ATGATCAGGGAATTCTAAGGATGCCCACCGAAGGTAAATGGATCCCAGTCTGGGTCAGTGTGGAGTATTCACAA
   SARS-CoV-2 Reference Genome Shuffle No.1 (22931) AGAAACGTGTCATAATTATGACGTATATCTAAGCTTAGTA-ATATAACTCTC--TTAG------GTACTCGCTA
                                                                     Section 327
                                                   (24125) 24125 24130      24140      24150      24160      24170      24180            24198
Homo sapiens chromosome 13 NC_000013.11: 34882059... (23056) AGGGAAGAATTAATGGGGAACTGGGCACTGTTTATTCAACATGATCCAGAGAGAAACACTAAATATGAGCAAA-
   SARS-CoV-2 Reference Genome Shuffle No.1 (22996) CCCGGGAAATTAAACGGGGAGAAAGTTATCTAATGCGACCGCTGTCGGGCCTTAAATGCACAGTTATAGAAGAT
                                                                     Section 328
                                                   (24199) 24199      24210      24220      24230      24240      24250      24260     24272
Homo sapiens chromosome 13 NC_000013.11: 34882059... (23129) ATGAACTTTCCAGTCACTGGGAAAGCATCTCTGTAGGTGAAGTAGAAATGATTGAATAAGCATCG-CCACAGAG
   SARS-CoV-2 Reference Genome Shuffle No.1 (23070) ATGACCGGTTCCATGTCTCATAAAAGATCA-TGTCAAAGACTTGCCAGCGATCTCCCATGCACCTACCACCTTC
                                                                     Section 329
                                                   (24273) 24273  24280      24290      24300      24310      24320      24330         24346
Homo sapiens chromosome 13 NC_000013.11: 34882059... (23202) CAATGAATCTC---TCCCATGCTTACTGATCAACATTCTGATTATTAGTGTATCTTCCTGG-GTACCCTAATCT
   SARS-CoV-2 Reference Genome Shuffle No.1 (23143) GATTCCTTCGAAGATAACACGTGTAGTTACTCAAACTA-GATGAGAATAATATCTTATTACCGTTAAATAAGGT
```

Section 330

```
(24347)                                                          24347        24360     24370     24380     24390     24400     24410  24420
Homo sapiens chromosome 13 NC_000013.11: 34882059... (23272)     CAG-CAAAGGAACATCCTGGATGGGGATACATGCACTCCAGCAACTAGAAC------TGGATATCTCCCACTA
SARS-CoV-2 Reference Genome Shuffle No.1 (23216)                 CCGGCGATTGCACTTATGAGCATGAGTCTGAATATGTTCCGATACCCCATACGCCGCTTTACTAGGTCCAACCA
```

Section 331

```
(24421)                                                          24421    24430     24440     24450     24460     24470     24480      24494
Homo sapiens chromosome 13 NC_000013.11: 34882059... (23339)     TAAATTACCCAGGACTCTTGCGAGTAGATTGCCATGAATCTTCTTTCCTGAGGCTTTTCCACTTTGAGAAATTG
SARS-CoV-2 Reference Genome Shuffle No.1 (23290)                 CAGCT-AGTAATGAAGTTTGTGACCGGGG-GCCA--AAAGTAATGTACTG----TTCTCCTCTTTTTACAATAT
```

Section 332

```
(24495)                                                          24495 24500     24510     24520     24530     24540     24550         24568
Homo sapiens chromosome 13 NC_000013.11: 34882059... (23413)     ACAGGGCTGAAGGCCAGACTGGGGCCTTTCTAAATTCACCAAAGGTAATGCCACCTGTGAGGCTTCTTGGCAG
SARS-CoV-2 Reference Genome Shuffle No.1 (23356)                 GCTTAAATAAAGGTGTGTATGATTGTACGTATGTAATCTGGGATCG-ATGGAAAGCTAAGAAACTATTACTTAG
```

Section 333

```
(24569)                                                          24569      24580     24590     24600     24610     24620     24630      24642
Homo sapiens chromosome 13 NC_000013.11: 34882059... (23487)     GCAGTTTTCAGCATTCTCCTGGAAGCACCCT-AACCT-TCTCTACAGCGCCTTTGAAATTTTTAACAATGGGCA
SARS-CoV-2 Reference Genome Shuffle No.1 (23429)                 TAAACAATAAGATTTCTCATGGTAGCTGTCGGAACCGATGTAAAAAATTCGGTTGAATCTTCGGGATAGCTGCC
```

Section 334

```
(24643)                                                          24643  24650     24660     24670     24680     24690     24700          24716
Homo sapiens chromosome 13 NC_000013.11: 34882059... (23559)     TAGTGAATATGAATTAGATGTCACTATGTCACC-AATCAGCTCTGTGGAAACATAAAACCTCTTT--GTTTGA
SARS-CoV-2 Reference Genome Shuffle No.1 (23503)                 AGACCCGTATTGAGAGCATAAAAGCCGATCACTGACTGGGATTGGTTGCGTC-TAAGACTAAGTTACGGTATAT
```

Section 335

```
(24717)                                                          24717        24730     24740     24750     24760     24770     24780  24790
Homo sapiens chromosome 13 NC_000013.11: 34882059... (23630)     TGGCTACACATACTTTTATTTGACAAGCCCTGTAACTGCTCAGCACCAGAAAAACTAAATT---GGTCCATTCA
SARS-CoV-2 Reference Genome Shuffle No.1 (23576)                 CACCCTAAGATCCTTTTAATTACGCA--CTTTTCTTTGCCCGACGGGCGAAGAATTAAATATACGCTACGCCCA
```

Section 336

```
(24791)                                                          24791    24800     24810     24820     24830     24840     24850      24864
Homo sapiens chromosome 13 NC_000013.11: 34882059... (23701)     --GAAAACTTGGT----------CCAACCTAGTAAATTTTTC---AGTTTCTGCTAGCTTAAAA-TTGAGCATC
SARS-CoV-2 Reference Genome Shuffle No.1 (23648)                 TCGAATTTTTGTTGTTGCTTATCCCGCGGTACTCGATTTACCATAAGACTTCCTTATCTCCATACTTTAAAAGA
```

```
                                                                     Section 337
                                                   (24865) 24865 24870     24880     24890     24900     24910     24920            24938
Homo sapiens chromosome 13 NC_000013.11: 34882059... (23759) TCTCAGGTAAATATTCAATCAAATG----CAAGTG-TAATTCTGTCACTGTCCAGTTCAATGCTCGTTCTCTTC
  SARS-CoV-2 Reference Genome Shuffle No.1 (23722) TCTCAACCATC-ATCCGATAAAATGAAGACACGTGATAAAGCAGGCGCTATTTA-TTGAA--CTCTTAATCACC
                                                                     Section 338
                                                   (24939) 24939      24950     24960     24970     24980     24990     25000      25012
Homo sapiens chromosome 13 NC_000013.11: 34882059... (23828) CTACAATCCCTCCACTGTCC-AT--CCAACTCTCA--GGAGGCCTCTGAGAGGCCTCTGGCCCAACACCTGTGC
  SARS-CoV-2 Reference Genome Shuffle No.1 (23792) CTTGACGCAGTACAGGGTCAGATTGTCAATACCCAATGATGTATTATCAGAAATAT-TGTATTAACTCATTTT-
                                                                     Section 339
                                                   (25013) 25013  25020     25030     25040     25050     25060     25070           25086
Homo sapiens chromosome 13 NC_000013.11: 34882059... (23897) AGTGGAAGACCTAAATTAAGACCCAATACTATGTGCTGCCTTGACATCTGGAAAAATCAGCAGGACCTCAAAT
  SARS-CoV-2 Reference Genome Shuffle No.1 (23864) ACTATATTACGCTAAGCTTAGAAGGAA--CTATCACTTG----GACTT--GCACAGAT-AGAGCGGCCTCATAA
                                                                     Section 340
                                                   (25087) 25087        25100     25110     25120     25130     25140     25150  25160
Homo sapiens chromosome 13 NC_000013.11: 34882059... (23971) GGCCTGATCCCAAGTTCACCTCCTGCTCCCA-TGGTTAAAGTCCCCCAGTCCAAAAATTCTCCTTAGCCAGTGG
  SARS-CoV-2 Reference Genome Shuffle No.1 (23929) CGTGAGTTGACACG--CCACTAGGGATGCCAGTGGTTCACGCTTTCATTTTTAAAAACCATCTGTATCCGGTG-
                                                                     Section 341
                                                   (25161) 25161    25170     25180     25190     25200     25210     25220         25234
Homo sapiens chromosome 13 NC_000013.11: 34882059... (24044) ACCAGGCACAGTTTCTGCTTATCCCTAAGTAGTGGGTTTCAGTTCCCAGCCTACCTAAGGAATTATTCACACAA
  SARS-CoV-2 Reference Genome Shuffle No.1 (24000) --CAAACATCGTTACCGTTGGAGCATACATATAAAGG---AGGTCCGAGAGGGTGCAAGGATTTCGGGTCT---
                                                                     Section 342
                                                   (25235) 25235 25240     25250     25260     25270     25280     25290            25308
Homo sapiens chromosome 13 NC_000013.11: 34882059... (24118) ACCAATCACATCCTCTCACAGAAATC-AGGAGTCACCTCACCCTCTTGATACTACAGTCTGCCTTTCCCA-GCC
  SARS-CoV-2 Reference Genome Shuffle No.1 (24066) ACTTATCGTGGTCACATATCGCTATCTATGTTGCTTTTTTCCATAGTATGTCCACTCATTGAATGTATGAAGAA
                                                                     Section 343
                                                   (25309) 25309      25320     25330     25340     25350     25360     25370      25382
Homo sapiens chromosome 13 NC_000013.11: 34882059... (24190) CTGGCTCGTTCACTCAGTTTTCGAGTGGAACTCTCATGTGACCCTGCATGGCGTGCTGTGTTCTCCTTCCCCAG
  SARS-CoV-2 Reference Genome Shuffle No.1 (24140) CTCAATTTAGTATTAC--TATCGATT--ATGTCTGATATACACTTAGATATCATAAGGTAGCGAACTGCCCCAT
```

Section 344

(25383) 25383 25390 25400 25410 25420 25430 25440 25456
Homo sapiens chromosome 13 NC_000013.11: 34882059... (24264) GTTATG--AGGACATGTAACTAATAAAGTTACAACACCTCCTCTGCCCAGTGTTGGGAGTCATGTGTTTGGCTA
SARS-CoV-2 Reference Genome Shuffle No.1 (24210) GAACTGCCACGACATGTACATTCTGTAAGTA--ACGGATC-------------TGGGACGACCCTATTTAACTC

Section 345

(25457) 25457 25470 25480 25490 25500 25510 25520 25530
Homo sapiens chromosome 13 NC_000013.11: 34882059... (24336) TCCCAGTAACTCTAAA-GCATCAAAGGGTAAATAGGAGGTGATTAAAACACCCTGCACTAAGCAGCAGGACGGA
SARS-CoV-2 Reference Genome Shuffle No.1 (24269) -CCCATTAATAGTAACCGCCTTTAAGTTTAAACTGATGACGCAGAGGTCAGTTT----TAATC-GCATTGCGGA

Section 346

(25531) 25531 25540 25550 25560 25570 25580 25590 25604
Homo sapiens chromosome 13 NC_000013.11: 34882059... (24409) ATGGAGATGCGTCACCTGACCGGCTGCATGGTGGCTGTAAGGTGGCTTCCTCAGGTGGGTCTTGCTGGTAG-CT
SARS-CoV-2 Reference Genome Shuffle No.1 (24337) TAG----TGCTAGAGCCGTACAGC-GTTTATTTTATTTAT------TTACCCACTTGTTTCTTTCTATTTCACA

Section 347

(25605) 25605 25610 25620 25630 25640 25650 25660 25678
Homo sapiens chromosome 13 NC_000013.11: 34882059... (24482) AGAGTCCTTCCCTAGTCTAGTCTCTGGCTCTGGTGTTTTATCACTACCAGCTATT-TGGAGTGGTCTGGTAGGT
SARS-CoV-2 Reference Genome Shuffle No.1 (24400) AGCGACGAT---TTGACTAAACCGTGAGTTTATTGTTGTTACATCAGCTCATTTCCTGGTTGAGACCGAGAAAG

Section 348

(25679) 25679 25690 25700 25710 25720 25730 25740 25752
Homo sapiens chromosome 13 NC_000013.11: 34882059... (24555) GGTGGATAACAGCCTCGAAAGGC--TTAGGGCGGCT-TCC--AGGTTTGGGTAATCCAATAAGGTGATCCTTAC
SARS-CoV-2 Reference Genome Shuffle No.1 (24471) GTATTACTGTTGACGCCAAATCCGTTTAGTACGTTTGTACTAAGGTTGAGGTTTTTCACTACTAACATACGCTA

Section 349

(25753) 25753 25760 25770 25780 25790 25800 25810 25826
Homo sapiens chromosome 13 NC_000013.11: 34882059... (24624) ATGCTAAATCCTCCAAGGTCTACCTCACAGGCTTAGGACATGAGGTCTTGTCCCCACTGAGAAACTTCTTGTG-
SARS-CoV-2 Reference Genome Shuffle No.1 (24545) ATATTGCATAATCTAACTTTTAACTTCCCTTCAGTGGGCTAGAGCTCAATTTAT-AGTGAGAACTCACTAAGGT

Section 350

(25827) 25827 25840 25850 25860 25870 25880 25890 25900
Homo sapiens chromosome 13 NC_000013.11: 34882059... (24697) AGATGGCTGACAAGCCAGCAAGGACCTCAGCTACCTA---GTTCCCTAGTTAATTCCAAGCCTACAGGAAACCT
SARS-CoV-2 Reference Genome Shuffle No.1 (24618) AAAAAGAAGA-AACACGGTGCTAATCTAGTCTATAAAGAGGGTGTGTAGACCTTTCTGAATAGATGTGTGTACT

Section 351

```
                                                      (25901) 25901    25910     25920     25930     25940     25950     25960       25974
Homo sapiens chromosome 13 NC_000013.11: 34882059... (24768) -------AGAAATTTCCCCCAGCTGAGCTTGGCTCCAGAGGAAGCTGAAGGTG-------ATCTTTGCC-TTCT
SARS-CoV-2 Reference Genome Shuffle No.1 (24691) GGGGTAAAGAAACTTCATTTTGA-GTGTGTGGCTGAAAATTA-GCATATGGTCGATAAAAATGTTTATGGTTAT
```

Section 352

```
                                                      (25975) 25975 25980     25990     26000     26010     26020     26030           26048
Homo sapiens chromosome 13 NC_000013.11: 34882059... (24827) CTTGCCTCATATTGCTATCTCCTCAGGGTCCTTCTTGCTCCAAAGGCGACAGAGGATTCTGCAGATTCTATTGC
SARS-CoV-2 Reference Genome Shuffle No.1 (24763) CTACACTTCTAATCCTGTGTTCAACAGGTAGTCGTTATAATTGAACATACATTAAAACTAGCCGGTACATTGGC
```

Section 353

```
                                                      (26049) 26049       26060     26070     26080     26090     26100     26110     26122
Homo sapiens chromosome 13 NC_000013.11: 34882059... (24901) TCAGCAAACTCTAGAAATTGTGTGTCATTTTCTTACTTATCTGCCTAC--AGCTGCAAAACTAAAAATGATGCT
SARS-CoV-2 Reference Genome Shuffle No.1 (24837) TGAACTG-CGGTATAACTAATCAGTCCC---CTTAATTATATTTCGAATAAGATATAATGGTCGTATTTCAGAT
```

Section 354

```
                                                      (26123) 26123  26130     26140     26150     26160     26170     26180           26196
Homo sapiens chromosome 13 NC_000013.11: 34882059... (24973) T-TAGAATGTTTCTCG-TGTGTAGACATGTGGTCAGAATAAATCTTCTCACTCAAATGCTCTCTTTTCCTCCTT
SARS-CoV-2 Reference Genome Shuffle No.1 (24907) TGTGGTAAGATAATTGATAAGTTGCTATAGAGTCAACGAATAGGCTATTAAAATAACTCGCAGTATATTGTCTT
```

Section 355

```
                                                      (26197) 26197         26210     26220     26230     26240     26250     26260  26270
Homo sapiens chromosome 13 NC_000013.11: 34882059... (25045) TTAGTGTCCT-CTCCTGACTCTCCTTCCTTTCACAGCCAAGCCTCAAGAATGGTTCTATGCTCACTTCCCCTAC
SARS-CoV-2 Reference Genome Shuffle No.1 (24981) GTGCTGTTGGGCTGGTAAATACCGTTTTTAT-ACAGCTGGGGGACACTGGCCTTTTTAGGATCATATGGTATTA
```

Section 356

```
                                                      (26271) 26271    26280     26290     26300     26310     26320     26330       26344
Homo sapiens chromosome 13 NC_000013.11: 34882059... (25118) TTCCTCCC----ATCACATTCACTCTTTGCTGC-AGTCTGACTCTAGTCCCTGAAACTCCTTTCCATAAAGTCA
SARS-CoV-2 Reference Genome Shuffle No.1 (25054) TTTCTGGTTTGGATAACGCAAAATTAATACAAATAATCGGGGTCAGATTACAGTACATTCATGAAATATA-TAA
```

Section 357

```
                                                      (26345) 26345 26350     26360     26370     26380     26390     26400           26418
Homo sapiens chromosome 13 NC_000013.11: 34882059... (25187) TAATTT-ATCCT--CAAATTGTGAAAT-TCAATGACTATGTCCTGGCCTTATTGAACTAC-AGTGTGGCACAAT
SARS-CoV-2 Reference Genome Shuffle No.1 (25127) TAATAAGAGTCTAGTATATGTTGAATTATCGATCACGCCGTAAGAGTTTAACTAAATTATGAATGAGATTGATT
```

Homo sapiens chromosome 13 NC_000013.11; 34882059-34911962 vs. SARS-CoV-2 Shuffle No.1

Section 358

```
                                                       (26419) 26419      26430     26440     26450     26460     26470     26480   26492
Homo sapiens chromosome 13 NC_000013.11: 34882059... (25256) GTGCTGCCCAC--TCCCTCTTTAA-AACTCT-CACTTTAGTGT-TAGTGACATCATTCTTGCCTAAGTATCA-T
   SARS-CoV-2 Reference Genome Shuffle No.1 (25201) GGAGTGTACTGGATCGATTGCTAATAATTCAGCAGATACGAATGTTCAGACATCGGTATAGACAATTAATTACT
```

Section 359

```
                                                       (26493) 26493  26500     26510     26520     26530     26540     26550       26566
Homo sapiens chromosome 13 NC_000013.11: 34882059... (25324) CTCCTTCCCTGAGCATCTT-TCTGTCTCATGCATGCAGGCTCTTCCAATTTCCCCTCAGTGATGGTGCTGCCAC
   SARS-CoV-2 Reference Genome Shuffle No.1 (25275) ATACCTAACTAGTAGTGTAATCTGCCGCAGATAACAAGAGTGTTGCCTTGAGTTTTTTCTTCACGATGTGTCCAT
```

Section 360

```
                                                       (26567) 26567        26580     26590     26600     26610     26620     26630  26640
Homo sapiens chromosome 13 NC_000013.11: 34882059... (25397) TGCT-GAGGACCTGCTTTTTGCACTGTGCTCTTCCATCCATATAGTTCTCATTAGGTGACCACATGGAAGCC-T
   SARS-CoV-2 Reference Genome Shuffle No.1 (25349) GACCAGATATCGTACCACTGGAGGAGTTCTCTTTCCACAATA-ACTCCATAGTCGGT-AGTATATCCGAGCTAT
```

Section 361

```
                                                       (26641) 26641    26650     26660     26670     26680     26690     26700       26714
Homo sapiens chromosome 13 NC_000013.11: 34882059... (25469) CTGTTTTCCTTCAACTACCACTGTGTATGACCCAATGC---CTCCAGCTCCTTCTACTGAGCTCAGACATCATC
   SARS-CoV-2 Reference Genome Shuffle No.1 (25421) CTAATTTTATCCAAGAGCCTCAATGT-TAAGCGTTTGCTGACTTTAGAAACCCCGGCTATCCACTATTATTATG
```

Section 362

```
                                                       (26715) 26715 26720     26730     26740     26750     26760     26770         26788
Homo sapiens chromosome 13 NC_000013.11: 34882059... (25540) CCCGGGTTCTGGACCCACATAATGTGTTCTTTATTTGAAGAGAAATGATGACTTTATGGAAAAAGTTTCTGCC
   SARS-CoV-2 Reference Genome Shuffle No.1 (25494) TGCCGGTTATTTAG--ATTTGGTATCGCACGGGCTTCTCTAGACCATGTAACGTTAGACCTTACAGGATAAAAA
```

Section 363

```
                                                       (26789) 26789      26800     26810     26820     26830     26840     26850   26862
Homo sapiens chromosome 13 NC_000013.11: 34882059... (25614) TGAAACTTGCCTAGGCACTTTATACTTAACATGGATTATAAGCCCTTCTTCCCTCTGTATTCCCCTAGTTGAAG
   SARS-CoV-2 Reference Genome Shuffle No.1 (25566) GAGAAGTAAGCTATGT-CGACAAGCGCAAAGAAGTTTTTAGCAAGTAATCTGGAATGGATACACTC---TGCAG
```

Section 364

```
                                                       (26863) 26863  26870     26880     26890     26900     26910     26920       26936
Homo sapiens chromosome 13 NC_000013.11: 34882059... (25688) GCACATTCATCCAAGGACCTAGTATTCTAGCCATTCTGTCTG--CCTCTTCTATCCTCATTGCCTAAGAGTCTG
   SARS-CoV-2 Reference Genome Shuffle No.1 (25636) TGTTGTGGTGCTAGAGATC-AG-ACTCTACACAATCAGGCGGAACTTTTTGATTGCTAGGTACTAACTCGACCT
```

Homo sapiens chromosome 13 NC_000013.11; 34882059-34911962 vs. SARS-CoV-2 Shuffle No.1

Section 365

```
                                                    (26937) 26937        26950     26960     26970     26980     26990     27000   27010
Homo sapiens chromosome 13 NC_000013.11: 34882059... (25760) ATTTTACTTCCTAAATGTACATGTTTGGGCTCTTTTCTTCCCTTCTATTGCACTGC--CATGGTCTTGGTTTAG
         SARS-CoV-2 Reference Genome Shuffle No.1 (25708) AGTTCGTTTCTTATGTGGAAATTGCAAGGAAAGCTAATTAG-TTAAATGGATAAGGATCATGGGCGTAGGATGA
```

Section 366

```
                                                    (27011) 27011    27020     27030     27040     27050     27060     27070       27084
Homo sapiens chromosome 13 NC_000013.11: 34882059... (25832) GCCCTCATTGCTTTTTGCCAGGATTATTGTTCTCTCAGCCTCTAGACTACACAAGCCCTTTCTCCTACCCAACC
         SARS-CoV-2 Reference Genome Shuffle No.1 (25781) TCATACAGTGGTTTAT---ATAATT--TATTCCCTTGACTCCTAAGAATTATAAACCCATCC-----------C
```

Section 367

```
                                                    (27085) 27085 27090     27100     27110     27120     27130     27140          27158
Homo sapiens chromosome 13 NC_000013.11: 34882059... (25906) TCTACCAGAGTGATTTTTTAACTG--TAAATCTGCTCGTGTCAGGTTACCTTCCTGCTTAAAACCTT-----TC
         SARS-CoV-2 Reference Genome Shuffle No.1 (25839) TCTTTAAGATTTATATGCCAGCTAGCTACCTTTGTCTGTATCACGAGACCCTGATCTTGAAATTTTTAGGTGTC
```

Section 368

```
                                                    (27159) 27159       27170     27180     27190     27200     27210     27220     27232
Homo sapiens chromosome 13 NC_000013.11: 34882059... (25973) AGGAT---TCCGTCACATT---CAGGATAAAGGTCAAACTCCTCAGCTAGG----CACACTA-AGCCCATCATT
         SARS-CoV-2 Reference Genome Shuffle No.1 (25913) AGTGTGTATACGTTACATCATACATATTAAAGATTTTGTTCCTTAGAGAATACCTGACTTTATAGCGCATATCT
```

Section 369

```
                                                    (27233) 27233   27240     27250     27260     27270     27280     27290         27306
Homo sapiens chromosome 13 NC_000013.11: 34882059... (26036) AATACTTTGGCATCACACTCCACAGTACTAGTTGTGA-GAGTGTAGACAAGTTACTTAACCTTACTATGCCTCA
         SARS-CoV-2 Reference Genome Shuffle No.1 (25987) TAAAGTTT--CTTGGTGCTCCGTCACTCTAGTTGACATGAGTACGAAGTTTTTCGATAACCGGG-TATGTATGA
```

Section 370

```
                                                    (27307) 27307        27320     27330     27340     27350     27360     27370   27380
Homo sapiens chromosome 13 NC_000013.11: 34882059... (26109) GTTTCCTCCATGTGTAAAATA------GAGATAAGATAGCACGTGTGATAAGATCATTGTCAGCACAGAATGAA
         SARS-CoV-2 Reference Genome Shuffle No.1 (26058) ACTGACGCTATAGGGGATATTTCTTTGTATATGCGGAATTGCTTGTGC--AGGTACATGCCAACCCTGGCTGCA
```

Section 371

```
                                                    (27381) 27381    27390     27400     27410     27420     27430     27440       27454
Homo sapiens chromosome 13 NC_000013.11: 34882059... (26177) CTAAGGCATATAAAGTACTTAGGCACGTAGTAAGCAATCTGTTTTTTTGCTTCTTTGTTTGTTTA---TTTTTT
         SARS-CoV-2 Reference Genome Shuffle No.1 (26130) TTAAAGTGAATACCGATATCTGCTAGTCTCTAAGAAAAACCTGATCTAGCTGATATGGCTTTTCAGTGGTTTTT
```

Homo sapiens chromosome 13 NC_000013.11; 34882059-34911962 vs. SARS-CoV-2 Shuffle No.1

```
                                                                                  Section 372
                                                    (27455) 27455 27460     27470     27480     27490     27500     27510              27528
Homo sapiens chromosome 13 NC_000013.11: 34882059... (26248) AGACAGGGTCTTACTCTGTCACCAAGGCTGGAGTACAGTGACATGATCTCAGCTCACTGCAACCTCTTTGCCTC
    SARS-CoV-2 Reference Genome Shuffle No.1 (26204) TGACACAGTCGTT-----TGAACTAGGCTCGAA---AGGTACCTAA---CAACTGAGAGCTATCATTGTGAATC
                                                                                  Section 373
                                                    (27529) 27529      27540     27550     27560     27570     27580     27590       27602
Homo sapiens chromosome 13 NC_000013.11: 34882059... (26322) AGCCTCTCAAGTAGCTGGGACTACAGGAGCATGCCACCATGCCCAGCTAATC-------AATGTTTGTTTGTTT
    SARS-CoV-2 Reference Genome Shuffle No.1 (26267) AATCTGTCTCATCATTGCCCGTCCA--ATCAATCT-CCGTATGAAGTTTGTCCCCATCAAATGCGTTGATCATC
                                                                                  Section 374
                                                    (27603) 27603   27610     27620     27630     27640     27650     27660        27676
Homo sapiens chromosome 13 NC_000013.11: 34882059... (26389) GTTTGTTTGTTTGTTTCTTTCTTTCTTTCTTTCTT-TCTTTCTTTCTTTCTTTCTTTC-TCTTTCTTTCTCTCT
    SARS-CoV-2 Reference Genome Shuffle No.1 (26338) ATCGTTGTGTACCCAGACAGCGAGTATAATGTCTTGTCCGCAAGGCTACCATGAATTCGTGTATCTGCGGATCC
                                                                                  Section 375
                                                    (27677) 27677        27690     27700     27710     27720     27730     27740  27750
Homo sapiens chromosome 13 NC_000013.11: 34882059... (26461) TTCTCTCTTTCTCTCTCTTTCTTCCCTCCCTCCCTTTTTCCTTCCCTCCCTCTC-TTCCTTCCTTCCTTCCCTC
    SARS-CoV-2 Reference Genome Shuffle No.1 (26412) TCATATAACTGAGAAAAGAACTGTCCTCGTAATCAAAATGAATGAATAGGTCAAGTAACAGCCCCCCGTTCCGC
                                                                                  Section 376
                                                    (27751) 27751    27760     27770     27780     27790     27800     27810       27824
Homo sapiens chromosome 13 NC_000013.11: 34882059... (26534) CCTCCCTCTCTCCCTCCTTCCCT-TCCTTCCTTCCCTCCTTCTTTCCTTTT---TTTTGTA-GATATGGGCTTT
    SARS-CoV-2 Reference Genome Shuffle No.1 (26486) CCAAGCGGTGTTGGTTTTTGCGAATAGTTGAATGTCGAAGCTTTTACGTTTAAGTAACGAATGACACGTGATTA
                                                                                  Section 377
                                                    (27825) 27825 27830     27840     27850     27860     27870     27880              27898
Homo sapiens chromosome 13 NC_000013.11: 34882059... (26603) C--ACTGTGTTGCCCAAGCTGC--TCCAGAACTCCTGAGCTCAAGTGATCC-ACC-AGCCTAGGCCTCCCAAAG
    SARS-CoV-2 Reference Genome Shuffle No.1 (26560) CGAACGGACAGGTATAAACTACCGTTCTAAGTGCCAGAAGAGAGATTATACTACCTAACCGAGGAACCC---AG
                                                                                  Section 378
                                                    (27899) 27899      27910     27920     27930     27940     27950     27960       27972
Homo sapiens chromosome 13 NC_000013.11: 34882059... (26671) TGCTGGGATTACAGGCA-TGAGCCACTGCACCCAGCCAGCAATCTGTTTTTGATAG-TAGCT--ATCTTTATTA
    SARS-CoV-2 Reference Genome Shuffle No.1 (26631) CGGACGTTTACCAATCAATGAAGAAAACCAAACTCGTAGTAATACATCTGTAATAGGTTGCTGAATCTACATTA
```

Homo sapiens chromosome 13 NC_000013.11; 34882059-34911962 vs. SARS-CoV-2 Shuffle No.1

```
                                                        Section 379
                                             (27973) 27973  27980      27990      28000      28010      28020      28030      28046
Homo sapiens chromosome 13 NC_000013.11: 34882059... (26741) CCATTTTAAAATCTCACCTCCC--------TT-CTCTTCCCTTCCCTTCGGCCACCATACTGGTCTACTTGACAT
          SARS-CoV-2 Reference Genome Shuffle No.1 (26705) TACCTTTATGATTTCTCTAACGAATGAAATTTCGCTATAAAACAAAAAGTCTTTCGCAATTTTATAACTCATTG

                                                        Section 380
                                             (28047) 28047         28060      28070      28080      28090      28100      28110  28120
Homo sapiens chromosome 13 NC_000013.11: 34882059... (26807) TTCTGAATATTGCCTGTGAGTTCATCCTCCTGGGCTTACTTATGGTCCTGCTTCCTCTGTGTGT---AATACTT
          SARS-CoV-2 Reference Genome Shuffle No.1 (26779) CTACTAATCGTGTTCATAGGTTGGTTTAGATCGTCTGGTTAATAATACTGAACGCAATGTAGATATCAATCATT

                                                        Section 381
                                             (28121) 28121   28130      28140      28150      28160      28170      28180      28194
Homo sapiens chromosome 13 NC_000013.11: 34882059... (26878) -----TCCCCCTCTTTCTTCATCTAGCTAACAGGTACTTAGTTT-TAAAGACCCTTCTCACA----TCACTTCT
          SARS-CoV-2 Reference Genome Shuffle No.1 (26853) ATAGGTAACGGACCCCTTATACTTTGGAAATAGACACGGAATTAATAAAATCTTGTTTCAAGAAGGTTACGAAA

                                                        Section 382
                                             (28195) 28195 28200      28210      28220      28230      28240      28250      28268
Homo sapiens chromosome 13 NC_000013.11: 34882059... (26942) TCCAGAAACTCTT--CCTTG---ATCTTCTTGCTCCAGT-CGGGGTTTAATTTTCCATGTTTTTGTA-ACTCTT
          SARS-CoV-2 Reference Genome Shuffle No.1 (26927) TTCAGCGACTATTTACCTTATATATTATGTTTCTTTAACGCAAGTTTTCACTAGAATAACAAGCGTATAATCTG

                                                        Section 383
                                             (28269) 28269      28280      28290      28300      28310      28320      28330      28342
Homo sapiens chromosome 13 NC_000013.11: 34882059... (27009) CATTAATACATTTATCCCA-ACACTCAATAACTCTGCATTGGGCTTGATTACATGTTTGTCTCCTATACAAGAC
          SARS-CoV-2 Reference Genome Shuffle No.1 (27001) CGTGTTTTCGATGAACCGATAAACTACGTAGCTACGAGCAGTGCATGAATTCGTACTTCTA-CGTAAGCCTCTT

                                                        Section 384
                                             (28343) 28343  28350      28360      28370      28380      28390      28400      28416
Homo sapiens chromosome 13 NC_000013.11: 34882059... (27082) TGTGGATTCCTTAAAGGCAGAAT-----------ACAACTTTGTCTGAGTTCCAACGAAAGGGCTTGTCAAT-GT
          SARS-CoV-2 Reference Genome Shuffle No.1 (27074) TGAGTGATAGAGGCAGGCGGAATTCTGTGTCGGACAACGTTATCTCAGTTTTAAT---AGGGCAA-TCAATAGG

                                                        Section 385
                                             (28417) 28417         28430      28440      28450      28460      28470      28480  28490
Homo sapiens chromosome 13 NC_000013.11: 34882059... (27145) TGAACCAGGGATTCAGTAAATG--ACTGCTG--GGTGAATTAAAAGATTGCATCAGGTGCTCAGAGA-CTCTAG
          SARS-CoV-2 Reference Genome Shuffle No.1 (27144) ATTACCTTTCATT-AGTACTCGGTAATAATGAATGTAAATTTTATGACTTAATTAGCA-CTCAATTATCTCATT
```

```
                                                                  Section 386
                                                     (28491) 28491    28500    28510    28520    28530    28540    28550    28564
Homo sapiens chromosome 13 NC_000013.11: 34882059... (27214) CACACCTTTGCTTCAAAAAGCACCTTACTTCAAAATTATT-ATCTCATTTCTTCCTCTATTTAAAGTCCCTATT
         SARS-CoV-2 Reference Genome Shuffle No.1 (27216) CAAACCACAGTTGTATAA--CAAATACCAACGAGGTTTTGGATTTGAGATCATCATG-AGGAATGGTATCTACC
                                                                  Section 387
                                                     (28565) 28565 28570    28580    28590    28600    28610    28620    28638
Homo sapiens chromosome 13 NC_000013.11: 34882059... (27287) TGTAAATTGTGAAATTAGGCACTATTAGTTTACTGTCAGCGATGAAACTAAGCTAGTCAGCTGAAA-----ATT
         SARS-CoV-2 Reference Genome Shuffle No.1 (27287) GGCCATCTAAGATTAAATGCATTGTACTGTTCGTATTTGTGATGAAT-TAAGTTAGTGTATTTAAACCCACACG
                                                                  Section 388
                                                     (28639) 28639    28650    28660    28670    28680    28690    28700    28712
Homo sapiens chromosome 13 NC_000013.11: 34882059... (27356) TATTACAATT------CATTTATACTTC---TAAAGCGTAT-ATTTACCCTTTCAAA----CAAGGAACTTGTA
         SARS-CoV-2 Reference Genome Shuffle No.1 (27360) TGTTCCGGTTTGTGGCCGATCCTACCTCCGTTAAAACGTAGGATTCACGGTTTCCTTTGTTCTATTGACGGATA
                                                                  Section 389
                                                     (28713) 28713 28720    28730    28740    28750    28760    28770    28786
Homo sapiens chromosome 13 NC_000013.11: 34882059... (27416) AAGATAATAGGATGAATTTTAGTGATGAGTTTAAGGAAATATAATAGGAGGAAAGGGTGTAGTGTGAGAAGAT
         SARS-CoV-2 Reference Genome Shuffle No.1 (27434) CAGTT--TGCGATGACTTGT-GTCATCATT-----GGGCCTTAAATGGGTGAGAGAGCTGAATT---ACAACGG
                                                                  Section 390
                                                     (28787) 28787    28800    28810    28820    28830    28840    28850    28860
Homo sapiens chromosome 13 NC_000013.11: 34882059... (27490) AGGAAGAATAATTTTTTTCTGCCCAGGCACAGTGGCGCATGCCTATAATCCCAGCACTTTGGGAGGCCAAAGCA
         SARS-CoV-2 Reference Genome Shuffle No.1 (27497) AAAAAGGACGACTTTGAGCATCTTGAGCTGTGTGGCATTTGCGCAAA----CAGTACGCGAGTAAGCGGAACCA
                                                                  Section 391
                                                     (28861) 28861    28870    28880    28890    28900    28910    28920    28934
Homo sapiens chromosome 13 NC_000013.11: 34882059... (27564) GGAGGATC---ACTTGAGGCCAG-TAGTTCAAGGCTAC-AGTGAGCAATGATCATGCCATTGCACTCCAGTCTG
         SARS-CoV-2 Reference Genome Shuffle No.1 (27567) AGATTACGTGAACTTAAGGATTGGTAAACCCGAGTGACTATTGGGTAAAGAAAGAACCGGAG-ACGTGACTATT
                                                                  Section 392
                                                     (28935) 28935 28940    28950    28960    28970    28980    28990    29008
Homo sapiens chromosome 13 NC_000013.11: 34882059... (27633) GGCCACAGAGGAAGACTCAGTTTCTAAAAAAAAAAATTAAATACAGAAAAAATAAAAAAATAAAAAATAAATTTT
         SARS-CoV-2 Reference Genome Shuffle No.1 (27640) CACTATTCCAATAGAAATAATCTTTGGCGATTATTTCAGTAAAGGCAAAGGTTACAGTGTCCTCAGTTTAACCA
```

```
                                                                                                                Section 393
                                                             (29009) 29009      29020          29030          29040          29050          29060          29070      29082
Homo sapiens chromosome 13 NC_000013.11: 34882059... (27707) ATGG-GAGAGTTGCTTGGAACTCCTTAAATAA--CATCAAGTGGGAATCCAG--CCTCTA-CTGGTAGGGGTAA
         SARS-CoV-2 Reference Genome Shuffle No.1 (27714) CTGGTGAGATCTGCATT---CTCATTATTTGAGTCAATAGGTACGGTTACAGGACATCTCGCTGATACTTTAAG
                                                                                                                Section 394
                                                             (29083) 29083  29090          29100          29110          29120          29130          29140          29156
Homo sapiens chromosome 13 NC_000013.11: 34882059... (27775) GCTGATGGCCTACTATATGAGGGGATTTGACTGGAAAAGCACCCAGAAGCTTTTCATAATTAAACTAGTAGCTG
         SARS-CoV-2 Reference Genome Shuffle No.1 (27785) GATTAAGACTTATATTCTCAGGAGATAAGTTTT-AGAGGTA---AGTA--TTATCGTTTTTAGGTGGTAGAT-
                                                                                                                Section 395
                                                             (29157) 29157          29170          29180          29190          29200          29210          29220  29230
Homo sapiens chromosome 13 NC_000013.11: 34882059... (27849) AAATACTGAA----CTAATATCACTAATAAAAAGGCAGC-CTGACAACAAAAGAACTAAAATCTTGATACATGA
         SARS-CoV-2 Reference Genome Shuffle No.1 (27852) ATATACTATATATTTTTGTTCGTCTATTTCATAGTCAATGCTTCTTTTAATCTCAATTAATTCAAGAGTTTCGA
                                                                                                                Section 396
                                                             (29231) 29231      29240          29250          29260          29270          29280          29290      29304
Homo sapiens chromosome 13 NC_000013.11: 34882059... (27918) TACAACATGCTAAGTGAAG-GAA--GCCAGACAAAAAA-ACCCATATGCTTCATGTGTATGAAATGTC-CAGAA
         SARS-CoV-2 Reference Genome Shuffle No.1 (27926) -ACATACTGTTAAATGTAGCGAATTGGTAGTTAAAGTTGACTCTAAGTCGAACTCAATACTAAAAGACGCAGTG
                                                                                                                Section 397
                                                             (29305) 29305 29310          29320          29330          29340          29350          29360          29378
Homo sapiens chromosome 13 NC_000013.11: 34882059... (27987) TAAGCAAACCCATAGTCAGTGAGTGGATCAGGGCTGGGGAACAGGGAGTGATTTCTAATGAGTATGGGTTTCT
         SARS-CoV-2 Reference Genome Shuffle No.1 (27999) GAATTTATGCGATATTGATTTCTCTATTTGGTTGGTAGGAAATTTCTGGTGAACT----TCAGTATA--TTGCT
                                                                                                                Section 398
                                                             (29379) 29379      29390          29400          29410          29420          29430          29440      29452
Homo sapiens chromosome 13 NC_000013.11: 34882059... (28061) TTGGGGCTGATAAAAAATGTTCTGAAATTAAATAGTGGTGATGGGTGCAT--AACCCT---TTGAATATACTAAA
         SARS-CoV-2 Reference Genome Shuffle No.1 (28067) ACGCAGCTGGAGAGGAATAGATGATATGC--TTGTCGGAAAAAGTCTATTGAAAACTGAGGTGTTTCGACAAAG
                                                                                                                Section 399
                                                             (29453) 29453  29460          29470          29480          29490          29500          29510          29526
Homo sapiens chromosome 13 NC_000013.11: 34882059... (28130) ATCTACTGAATTGTACATTTTA-AA-----------AGTGTTATTTTTATGATATGTTAAATTATATTGCAATAA
         SARS-CoV-2 Reference Genome Shuffle No.1 (28139) TCCTCTTCAGGTTTACATATTGCACTACAACATCCATTGCTGTCATTGTGATTGGGCACGCCGCTGTTGAGTAG
```

```
                                                                  Section 400
                                             (29527) 29527        29540     29550     29560     29570     29580     29590   29600
Homo sapiens chromosome 13 NC_000013.11: 34882059... (28193) AAATATGTCAGAAAAAAGTGATATT--CCAGGAAATACTGACAATAGAAAAAAAC-AGCATGAGT---TATTT
        SARS-CoV-2 Reference Genome Shuffle No.1 (28213) ACTCTGTTCTCTCCTCAATGGTAGTAACGTGGGCAATCTCAACACGGAAGCAAATCTATTGTGAATTCCTAAGT
                                                                  Section 401
                                             (29601) 29601    29610     29620     29630     29640     29650     29660      29674
Homo sapiens chromosome 13 NC_000013.11: 34882059... (28261) ATTATACT--ATTTGAAGGTCCACAAATAAAATTATATTTTCTAAGTATGAAGG-ATTATATAGCAAGTCATTT
        SARS-CoV-2 Reference Genome Shuffle No.1 (28287) TCTAGAGTGGGTATCATAGTCTTCGCAGAATTCCA-ATCTACTAATGATTAGGACAAGAAATAAGATATCATGT
                                                                  Section 402
                                             (29675) 29675 29680     29690     29700     29710     29720     29730        29748
Homo sapiens chromosome 13 NC_000013.11: 34882059... (28332) TAAT-AGCTTACATA---AAGCAGTTTCATATTTCTTTTCCTCTAGTTTCACAGAACTCTTTTGATGTATTGTG
        SARS-CoV-2 Reference Genome Shuffle No.1 (28360) ACGCGAGCTT-CATACTGTAGGAGGTATCTCTTCAAAAATCAATAGCGTCGCCTAACTTTACCGGAG----GTC
                                                                  Section 403
                                             (29749) 29749      29760     29770     29780     29790     29800     29810    29822
Homo sapiens chromosome 13 NC_000013.11: 34882059... (28402) TCATTAATTAATAGGCTCTAAAAACTACCGTTGTGTTAGTTGGTGTTTTGTTTCTGTTTTAAACTTTCAATTTT
        SARS-CoV-2 Reference Genome Shuffle No.1 (28429) ACGCTATAACATGTTTTCTAAAAATTAC--TTGCCAGGGCTCGT---TGGCTCCTGTGC--------CATTTGT
                                                                  Section 404
                                             (29823) 29823   29830     29840     29850     29860     29870     29880        29896
Homo sapiens chromosome 13 NC_000013.11: 34882059... (28476) TTTTTTTTTTTTGAGATGGAGTCTCACTCTGTCACCCAGGCTGGAGTGCAGTGGCTGGAGTGCAACCTCCACCT
        SARS-CoV-2 Reference Genome Shuffle No.1 (28490) GAAATCCAAATAGAGTACCAGCCAAATTTATTCAC--AACATTGTTTGTTTTCGTAGGATAACGCGGTTCATC-
                                                                  Section 405
                                             (29897) 29897        29910     29920     29930     29940     29950     29960   29970
Homo sapiens chromosome 13 NC_000013.11: 34882059... (28550) CCCAGGTTCAAGTGATTCTCCTGCCTGAGCTTAACAAGTAACTGGGATTACAGGCACCTGCCACCAAACCCGGC
        SARS-CoV-2 Reference Genome Shuffle No.1 (28561) ---ATGCTCAAGAGTTCTTTGAGGTTTAATT--AGAGGGATGCGATATTAAA---ACCATTCAGTAAAACCAGA
                                                                  Section 406
                                             (29971) 29971    29980     29990     30000     30010     30020     30030      30044
Homo sapiens chromosome 13 NC_000013.11: 34882059... (28624) TAATTTTTGTATTTTAGTAGAGACGGAGTTTCACTATGTTAGCCAGGTTGCTCTCAAACTCCTGACCTGAAGTG
        SARS-CoV-2 Reference Genome Shuffle No.1 (28627) TCGCGTTACT--TTTAG-ATA-ACTGAATTTCATGCT--TACGCAGTGTTGGATCATTTCGTTCGCCATTAATA
```

Homo sapiens chromosome 13 NC_000013.11; 34882059-34911962 vs. SARS-CoV-2 Shuffle No.1

```
                                                                      Section 407
                                                       (30045) 30045 30050     30060     30070     30080     30090     30100             30118
Homo sapiens chromosome 13 NC_000013.11: 34882059... (28698) ATCCACCCGCCTCAGCCTCCCAAGGTGCCAGGATTACAGGTGTGAGCCACCACGCCTGGCCCTAAACTCAATTT
       SARS-CoV-2 Reference Genome Shuffle No.1 (28695) ACTTAGTGGTATCATCACGCACTGATGTCATG----CGCTTTTGAAAAGTAA---TGTATTAAAAGTAAAGGG
                                                                      Section 408
                                                       (30119) 30119      30130     30140     30150     30160     30170     30180    30192
Homo sapiens chromosome 13 NC_000013.11: 34882059... (28772) TTTAAAAAATAATTTATTTTGGTTTTATAATGGTCTCAATTTAGCTCATTCAATCAAAACA-ATAACCTATACT
       SARS-CoV-2 Reference Genome Shuffle No.1 (28762) AAGAGAATACAGCTTGATGTAGAACGTGTTCGGTTTCACGCTCGTGCGCAGCCGCGGGACAGATAAGTAATATT
                                                                      Section 409
                                                       (30193) 30193  30200     30210     30220     30230     30240     30250        30266
Homo sapiens chromosome 13 NC_000013.11: 34882059... (28845) ACTTTCGGTTCAATTCAAATGTTCCT---------TTTGACTATTAATCCCAGTTTTAAGATGAACATTTATAA
       SARS-CoV-2 Reference Genome Shuffle No.1 (28836) ATATGAGAT--AATTATAACCTTTTTGCAGACGACTTTGGCTCGTGATGTCCTAATTTACGTCTAAATAGACAA
                                                                      Section 410
                                                       (30267) 30267        30280     30290     30300     30310     30320     30330 30340
Homo sapiens chromosome 13 NC_000013.11: 34882059... (28910) CTATCCAAGTTGATGCCACAGATATGCT--GTAATTAATAACATTTTATGTTTCCTTTTATTTATCATA-GTGA
       SARS-CoV-2 Reference Genome Shuffle No.1 (28908) -TAGAAAAAGTGACACTGCAAATATGGTCAGCAACTAATTGCACTCGGGACTAACACAACGCGCTAAGAAGTGC
                                                                      Section 411
                                                       (30341) 30341    30350     30360     30370     30380     30390     30400        30414
Homo sapiens chromosome 13 NC_000013.11: 34882059... (28981) TATAATTTTATATTACTGTCCACCCATAAATGTCAGTTGAACAGAGGCTTAATTGAAACATTCTTTAGATCACAG
       SARS-CoV-2 Reference Genome Shuffle No.1 (28981) TAGC-TATCATAGCATTTTCT-CGGGACATGTTTGTTACCAAAAAGGCAAAATACACGAATCACCAAATTTTTG
                                                                      Section 412
                                                       (30415) 30415 30420     30430     30440     30450     30460     30470             30488
Homo sapiens chromosome 13 NC_000013.11: 34882059... (29055) AAAAAGATAGTGATGTTATGCTTGAAATATTTCTCGAAGATTATACAAGGTTAAGAAGTCATTGAACAAATGTT
       SARS-CoV-2 Reference Genome Shuffle No.1 (29053) CCGGACAATATAATGTTCAACATTAGA-AGAACAAGA-GTTGCGAGTCCCGTACGAATTCATTTTTATTCT-TT
                                                                      Section 413
                                                       (30489) 30489      30500     30510     30520     30530     30540     30550    30562
Homo sapiens chromosome 13 NC_000013.11: 34882059... (29129) TATTGGAGGTCTACTATGTA-AACTATGGCTCTCGGCCTTCTTA---TAT---TTCCAAGTGT-----ACCTTG
       SARS-CoV-2 Reference Genome Shuffle No.1 (29124) TATAGATGGTACGAAAGATACAACAATAAATACAGAGTAGCTTAGCGTATAGATTCCAGGTGTTAGGAATCTTG
```

```
                                                                  Section 414
                                                       (30563) 30563 30570 30580 30590 30600 30610 30620 30636
Homo sapiens chromosome 13 NC_000013.11: 34882059... (29191) AAAACCAACTCTTCTGAGCAACAACTTGGAGAGATT----AAATGTGCCACACACGTCAGCACTAATTACACTG
          SARS-CoV-2 Reference Genome Shuffle No.1 (29198) AGCCGTGAGACTAATTAAAATCGTCTAGTAGGACTCGGGCACCTATTCCA-ATACGTCCGGA--AATCAGATTA
                                                                  Section 415
                                                       (30637) 30637 30650 30660 30670 30680 30690 30700 30710
Homo sapiens chromosome 13 NC_000013.11: 34882059... (29261) AAAT-CCCAGAAAATC-ATGGCTCATAGTGT------CTGTGAAAGTGTC--CAGTCAGCACACTA----TTTA
          SARS-CoV-2 Reference Genome Shuffle No.1 (29269) TTATACCTAGTGGATTTAAGGTTAATCTTGTTCAGATCCGTAACAACCACTGCACTC-GCTCACAACGGTTGGA
                                                                  Section 416
                                                       (30711) 30711 30720 30730 30740 30750 30760 30770 30784
Homo sapiens chromosome 13 NC_000013.11: 34882059... (29321) GTTGTTTACACTT----TTACACTGAGG-CCTTGT---TGAAGAAGAAGAAATAGTCT-----CTTCCTTGACA
          SARS-CoV-2 Reference Genome Shuffle No.1 (29342) ATTGTTTACCCAGCTGGTTGAACCGCGGACCCTGTATCTGAAGCTCGTGAAGTACTCAAAATCCTTCTCAGAAA
                                                                  Section 417
                                                       (30785) 30785 30790 30800 30810 30820 30830 30840 30858
Homo sapiens chromosome 13 NC_000013.11: 34882059... (29382) AT-CCTCTTTTATATACTCAGACATAAGGGTTATTTTATGTTATAGTAAACACACATAATCCAGATATCCCAAA
          SARS-CoV-2 Reference Genome Shuffle No.1 (29416) AAACCTCTGATGCGGACT-AGATAAAGGGGAGCATATCTATTAAA---AACCAAAAGAATATT-ATGTCCCAAA
                                                                  Section 418
                                                       (30859) 30859 30870 30880 30890 30900 30910 30920 30932
Homo sapiens chromosome 13 NC_000013.11: 34882059... (29455) ATTTTCT--AAAATTTGATGTCTTGTTTTTTATAATACCCAAATAAAACAGCTCCTCCTCTTTACACT-CTCTG
          SARS-CoV-2 Reference Genome Shuffle No.1 (29485) TGATACTGGAATAGACGAGATATTAGTTTTCCGGTTAGGTGCAGTTGCTAGA--CTATTTGTTACAAAGCTGTA
                                                                  Section 419
                                                       (30933) 30933 30940 30950 30960 30970 30980 30990 31006
Homo sapiens chromosome 13 NC_000013.11: 34882059... (29526) GG-ATACCAGCACCCTCCCTTCTCCTCCATCAGTATTTTCTTGCACCTTTCAGAGGTGCACATTTTCTCAGATG
          SARS-CoV-2 Reference Genome Shuffle No.1 (29557) GCTATAAATTCGCCTTACTTTATCGTCC----GGACACCAGTTGACATGAGAGTTGCGTAACATATATTAGTTT
                                                                  Section 420
                                                       (31007) 31007 31020 31030 31040 31050 31060 31070 31080
Homo sapiens chromosome 13 NC_000013.11: 34882059... (29599) TTGCAGCTGCTTTCCCACTGTGGTTTGACCATGAACCATTATTTGTGGCTTCCATGTTAGTTGTTATCCAGTTA
          SARS-CoV-2 Reference Genome Shuffle No.1 (29627) TTACGTAAGCATA------GTCCTTTGATTAATATACGATATTTA--------ATGAAGAGTATTATACATTTC
```

Section 421

```
                                                        (31081) 31081    31090          31100          31110          31120          31130          31140        31154
Homo sapiens chromosome 13 NC_000013.11: 34882059... (29673) AAAGCAGAAAACAGTCTAGGGTTCTGTATTTGTCAGCTTGAAC-TACTATAACAAAATGTCACAGACAGGGTGA
      SARS-CoV-2 Reference Genome Shuffle No.1 (29687) ATTTCT-AAGGTAGT------TACTCTATTGCAAACAACGCGCGTAAGATACCCCTCTAGTATCGTCAACGAAT
```

Section 422

```
                                                        (31155) 31155 31160          31170          31180          31190          31200          31210              31228
Homo sapiens chromosome 13 NC_000013.11: 34882059... (29746) CTTAAGGAACAGAGATTTATTTATTATCTATTTTATTTATATCTCACAGTTTTAGACTCTAAAAAATTCAAGAA
      SARS-CoV-2 Reference Genome Shuffle No.1 (29754) CTTCATGCACACTGCTATCGTCACAGCGCTACCAATTTTCATATCACTGGCGATGAATCTGTATT-TAGAATAT
```

Section 423

```
                                                        (31229) 31229      31240          31250          31260          31270          31280          31290      31302
Homo sapiens chromosome 13 NC_000013.11: 34882059... (29820) CAAGATGCCTTGGTCTTACCTTCTCTCTGCGTCCTCACCTGGCAGACACTCTGTTGTCTCTTCTTTTAGGACAC
      SARS-CoV-2 Reference Genome Shuffle No.1 (29827) CTCAAACGCTTGCGG---CATTCTAT-TGTGTGTTTAAATT--AGCCAGTCTC----CTAGTAGTTGCAGACTA
```

Section 424

```
                                                        (31303) 31303     31315
Homo sapiens chromosome 13 NC_000013.11: 34882059... (29894) CAACGCTGTTG--
      SARS-CoV-2 Reference Genome Shuffle No.1 (29891) GACGGATATTGCT
```
