# Supplementary material for: Patch type nucleotide sequence identities between genomes from many different species facilitate illegitimate recombination
Source: Sci Rep. 2026 Mar 30;16:10524. doi: 10.1038/s41598-026-44124-0 (PMC13035915; doi:10.1038/s41598-026-44124-0)
Supplement: Supplementary file 16 — Supplementary Material 16 [file 41598_2026_44124_MOESM16_ESM.docx]

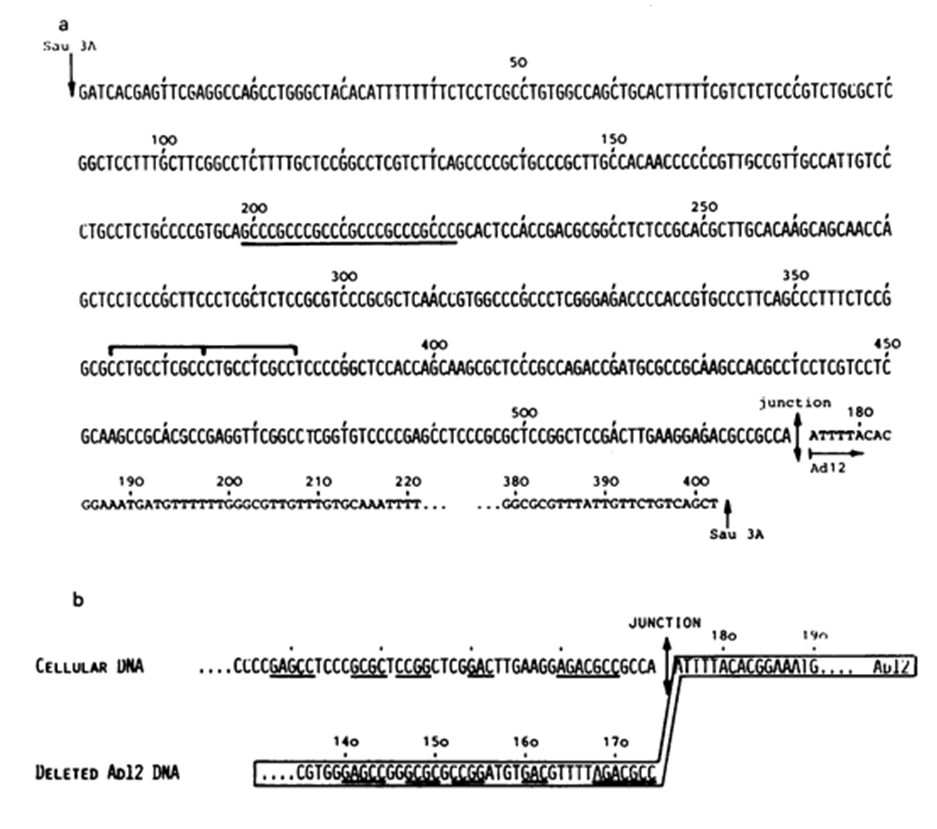


**Fig. SA** Junction site between the left terminus of *Ad12* DNA and hamster cell DNA.

1. Total of 529 nucleotides of cellular and of 128 of Ad12 origin. have been determined. Internal cellular repeats in the cellular DNA are underlined/ brackets.
2. 70 base pairs of *Ad12* DNA (bottom). Junction - vertical arrow. **Patch-type sequence identities** between the *Ad12* and hamster DNAs: **One tri-, two tetra-, one penta-, and one hepta-nucleotide**. Stabel & Doerfler (**1982**). **Nucl Acids Res 10**:8007-23. [doi:10.1093/nar/10.24.8007](https://doi.org/10.1093/nar/10.24.8007)
