# Supplementary material for: Patch type nucleotide sequence identities between genomes from many different species facilitate illegitimate recombination
Source: Sci Rep. 2026 Mar 30;16:10524. doi: 10.1038/s41598-026-44124-0 (PMC13035915; doi:10.1038/s41598-026-44124-0)
Supplement: Supplementary file 17 — Supplementary Material 17 [file 41598_2026_44124_MOESM17_ESM.docx]

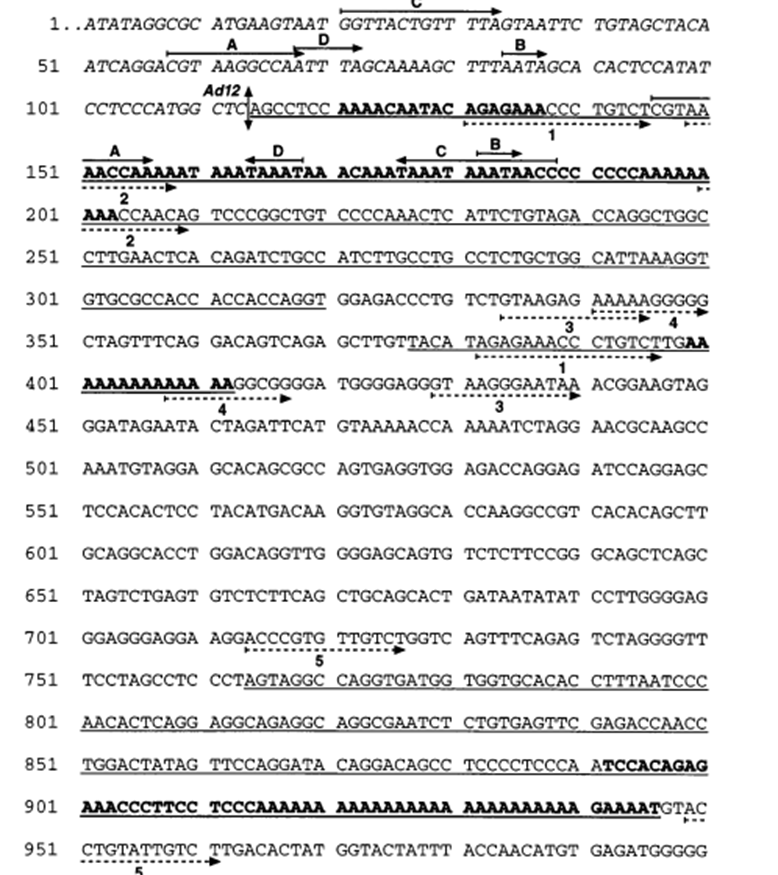


**Fig. SB** Nucleotide sequence at and adjacent to the junction site (double-headed vertical arrow) between *Ad12* DNA and hamster cell DNA in the *Ad12*-induced hamster tumor *T191*. *Ad12 113 nucleotides* in *italics*. **Patchy homologies** to known hamster DNA sequences (**4 to 13 nucleotides long**) arrows A to D). Knoblauch et al. (**1996**). - **J Virol 70**:3788-3796 [doi: 10.1128/JVI.70.6.3788-3796.1996](https://doi.org/10.1128/JVI.70.6.3788-3796.1996)
