# Supplementary material for: Patch type nucleotide sequence identities between genomes from many different species facilitate illegitimate recombination
Source: Sci Rep. 2026 Mar 30;16:10524. doi: 10.1038/s41598-026-44124-0 (PMC13035915; doi:10.1038/s41598-026-44124-0)
Supplement: Supplementary file 18 — Supplementary Material 18 [file 41598_2026_44124_MOESM18_ESM.pdf]

Legends: Supporting material: Sequence comparisons in **Figures S1 to S13** have been submitted online alongside this manuscript. These alignments can be scrolled along their entire lengths.

Figure S1: *SARS-CoV-2 Wuhan-Hu-1 & HERV-E*; **Figure S2**: *SARS-CoV-2 Wuhan-Hu-1* and human chromosome 1, start position 11,783,698; **Figure S3** : *SARS-CoV-2 Wuhan-Hu-1* and human chromosome 13, start position 34,882,059; **Figure S4** : *SARS-CoV-2 Wuhan-Hu-1* and *Oryza sativa, chromosome 1;* **Figure S5:** *Bombus pascuorum* chromosome 14 vs. *Oryza sativa* chromosome 11*;* **Figure S6**: *Homo sapiens* mitochondrion, complete genome vs. *Latimeria chalumnae* mitochondrion; **Figure S7** *Mus musculus* strain C57BL/6J chromosome 19 vs. *Triticum aestivum* cultivar Chinese Spring chromosome 6D; **Figure S8:** *Mycobacterium tuberculosis* vs. *Oryza sativa* chromosome 8; **Figure S9**: *SARS-CoV-2 Wuhan-Hu-1 & Fig badnavirus*; **Figure S10**: *SARS-CoV-2 Wuhan-Hu-1 & Fig badnavirus 2*; **Figure S11**: *SARS-CoV-2 Omicron BA.1* different isolates; **Figure S12**: *Homo sapiens* chromosome 17 vs. *Lycium barbarum* isolate Lr01 chromosome 6; **Figure S13:** *SARS-CoV-2* vs. *Adenovirus type 12* (*Ad12*) – See Graphical Abstract and manuscript page 35; **Figures S14 and S15:** In these experiments, shuffled SARS-Co-2 sequences were aligned to certain unaltered sequences on human chromosomes 1 and 13, respectively. Details and the actual results of comparative sequence alignments have been presented in **Table 2**.

By scrolling through the alignments, the high degree of sequence identities in patchy patterns will be apparent. Further alignments of the *SARS-CoV-2* sequence with nucleotide sequences of additional species are available from the authors.
