# Supplementary material for: Patch type nucleotide sequence identities between genomes from many different species facilitate illegitimate recombination
Source: Sci Rep. 2026 Mar 30;16:10524. doi: 10.1038/s41598-026-44124-0 (PMC13035915; doi:10.1038/s41598-026-44124-0)
Supplement: Supplementary file 19 — Supplementary Material 19 [file 41598_2026_44124_MOESM19_ESM.pdf]

Table S1 - ~~**Delete those examples in Table S1 used for New Table 1**~~

**Alignment**	**VNTI® Identity Positions**	**BLAST® Percent Identities**
Acidianus rod-shaped virus 1 (NC-009965.1; alignment complete genome) **vs.** ***SARS-CoV-2 Wuhan-Hu-1*** (NC_045512.2; alignment from 3,352-29,903nt) *	46.2%	45%
Adenovirus type 2 (J01917.1; alignment complete genome) **vs.** ***SARS-CoV-2 Wuhan-Hu-1*** (NC_045512.2; alignment complete genome) *	43.2%	43%
Adenovirus type 5 (AC_000008.1; alignment complete genome) **vs.** ***SARS-CoV-2 Wuhan-Hu-1*** (NC_045512.2; alignment complete genome) *	43.4%	43%
Adenovirus type 12 (X73487; alignment from 556-33,354nt) **vs.** ***SARS-CoV-2 Wuhan-Hu-1*** (NC_045512.2; alignment complete genome) *	44.9%	45%
Adenovirus type 12 (X73487; alignment from 12,239-24,660nt) **vs.** ***HERV-E*** (AB062274.1; alignment complete genome) **	44.9%	45%
Adenovirus type 12 (X73487; alignment from 13,005-33,725nt) **vs.** ***HERV-K*** (AF074086; alignment complete genome) **	44.8%	45%
Adenovirus type 12 (X73487; alignment from 25,874-29,259nt) **vs.** ***HERV-W*** (NM_014590.4; alignment from 1-3,044nt) **	46.1%	46%
Adenovirus type 12 (X73487; alignment from 15,503-22,066nt) **vs.** ***LINE 1.2*** (M80343; alignment complete genome) **	45.7%	45%
Adenovirus type 12 (X73487; alignment from 1-34,125nt) **vs.** ***Homo sapiens* chromosome 1** (NC_000001.11; alignment from 11,783,698-11,817,823nt) **	44.3%	44%
Adenovirus type 12 (X73487; alignment complete genome) **vs.** ***Homo sapiens* chromosome 13** (NC_000013.11; alignment from 34,882,059-34,916,184nt) **	44.1%	44%

Alternaria brassicicola endornavirus (NC_026136.1; alignment complete genome) **vs. *HERV-E*** (AB062274.1; complete genome) **	44.2%	45%
Alternaria brassicicola endornavirus (NC_026136.1; alignment complete genome) **vs. *HERV-K*** (AF074086; alignment from 5,027-16,155nt) **	45%	45%
Alternaria brassicicola endornavirus (NC_026136.1; alignment from 180-3,554nt) **vs. *HERV-W*** (NM_014590.4; alignment from 1-3,044nt) **	45.6%	45%
Alternaria brassicicola endornavirus (NC_026136.1; alignment from 3,503-10,069nt) **vs. *LINE 1.2*** (M80343; alignment complete genome) **	45.8%	46%
Alternaria brassicicola endornavirus (NC_026136.1; alignment complete genome) **vs. *SARS-CoV-2 Wuhan-Hu-1*** (NC_045512.2; alignment from 353-10,639nt) *	44.5%	44%
Autographa californica nuclear polyhedrosis virus (NC_001623.1; alignment from 42,843-75,223nt) **vs. *SARS-CoV-2 Wuhan-Hu-1*** (NC_045512.2; alignment complete genome) *	45.6%	45%
Candidatus Carsonella ruddii* strain BT** (CP024798.1; alignment from 135,557-167,442nt) **vs. *SARS-CoV-2 Wuhan-Hu-1 (NC_045512.2; alignment complete genome) *	46%	46%
Carrot mottle mimic umbravirus (ds-RNA) (NC_001726.1; alignment complete genome) **vs. *SARS-CoV-2 Wuhan-Hu-1*** (NC_045512.2; alignment from 1,990-6,319nt) *	45%	45%
Chlorocebus sabaeus* mitochondrion** (NC_008066.1; alignment from 2,779-15,177nt) **vs. *HERV-E (AB062274.1; alignment complete genome) **	44.5%	44%
Chlorocebus sabaeus* mitochondrion** (NC_008066.1; alignment complete genome) **vs. *HERV-K (AF074086; alignment from 636-19,135nt) **	44.6%	44%
Chlorocebus sabaeus* mitochondrion** (NC_008066.1; alignment from 5,862-9,251nt) **vs. *HERV-W (NM_014590.4; alignment from 1-3,044nt) **	46.8%	47%

Chlorocebus sabaeus* mitochondrion** (NC_008066.1; alignment from 128-6,484nt) **vs. *LINE 1.2 (M80343; alignment complete genome) **	46%	46%
***Chlorocebus sabaeus* mitochondrion** (NC_008066.1; alignment complete genome) **vs. *Homo sapiens* mitochondrion** (NC_012920.1; alignment complete genome) **	78.7%	79%
Escherichia coli* K12** (NC_000913.3; 129,062–161,212 nt) **vs. *SARS-CoV-2 Wuhan-Hu-1 (NC_045512.2; alignment complete genome) *	45.2%	45%
Fig badnavirus 1 (NC_017830.1; alignment complete genome) **vs. *SARS-CoV-2 Wuhan-Hu-1*** (NC_045512.2; alignment from 1,978-9,647nt) *	45.5%	45%
Hepatitis B Virus (NC_003977.2; alignment complete genome) **vs. *SARS-CoV-2 Wuhan-Hu-1*** (NC_045512.2; alignment from 21,047-24,558nt) *	45.7%	46%
HPV 16 (NC_001526; alignment from 1-7,905nt) **vs. *HERV- E*** (AB062274.1; alignment from 1,618-10,463nt) **	45%	44%
HPV 16 (NC_001526; alignment from 1-7,905nt) **vs. *HERV- K*** (AF074086; alignment from 5,016-13,700nt) **	46%	45%
HPV 16 (NC_001526; alignment from 2,800-6,032nt) **vs. *HERV- W*** (NM_014590.4; alignment from 1-3,044nt) **	44.9%	45%
HPV 16 (NC_001526; alignment from 25-6,598nt) **vs. *LINE 1.2*** (M80343; alignment complete genome) **	45.8%	45%
***Homo sapiens* chromosome 1** (NC_000001.11; alignment from 11,783,698-11,817,823nt) **vs. *Homo sapiens* chromosome 13** (NC_000013.11; alignment from 34,882,059-34,916,184nt) **	44.5 %	45%
Homo sapiens* mitochondrial DNA** (NC_012920.1; alignment complete genome) **vs. *SARS-CoV-2 Wuhan-Hu-1 (NC_045512.2; alignment 11,168-29,055nt) *	45.3%	45%
Human Immunodeficiency Virus 1 (K03455.1; alignment complete genome) **vs. *SARS-CoV-2 Wuhan-Hu-1*** (NC_045512.2; alignment from 699-11,486nt) *	45.8%	46%

Nitrosopumilus spindle-shaped virus (NC_048199.1; alignment complete genome) **vs. *SARS-CoV-2 Wuhan-Hu-1*** (NC_045512.2; alignment complete genome)*	47%	46%
**Phage *lambda*** (*λ*) (NC_001416.1; alignment from 14,752-47,353nt) **vs. *SARS-CoV-2 Wuhan-Hu-1*** (NC_045512.2; alignment complete genome) *	45.3%	45%
**Phage *T4*** (NC_000866.4; alignment from 9,803-42,013nt) **vs. *SARS-CoV-2 Wuhan-Hu-1*** (NC_045512.2; alignment complete genome) *	46.4%	46%
**Phage *mu*** (NC_000929.1; alignment from 3,019-35,926nt) **vs. *SARS-CoV-2 Wuhan-Hu-1*** (NC_045512.2; alignment complete genome) *	44.2%	44%
SARS-CoV-2 Wuhan-Hu-1 (NC_045512.2; alignment from 17,170-29,465nt) **vs. *HERV- E*** (AB062274.1; alignment complete genome) **	45.2%	45%
SARS-CoV-2 Wuhan-Hu-1 (NC_045512.2; alignment from 3,949-25,100nt) **vs. *HERV- K*** (AF074086; alignment complete genome) **	45.6%	46%
SARS-CoV-2 Wuhan-Hu-1 (NC_045512.2; alignment from 24,378-27,503nt) **vs. *HERV- W*** (NM_014590.4; alignment from 1-3,044nt) **	46%	46%
SARS-CoV-2 Wuhan-Hu-1 (NC_045512.2; alignment from 234-6,608nt) **vs. *LINE 1.2*** (M80343; alignment complete genome) **	45.9%	46%
SARS-CoV-2 Wuhan-Hu-1 (NC_045512.2; alignment complete genome) **vs. *Homo sapiens* chromosome 1** (NC_000001.11; alignment from 11,783,698-11,813,601nt) **	44%	44%
SARS-CoV-2 Wuhan-Hu-1 (NC_045512.2; alignment complete genome) **vs. *Homo sapiens* chromosome 13** (NC_000013.11; alignment from 34,882,059-34,911,962nt)**	49.4%	45%
SARS-CoV-2 Omicron BA.1 (#alignment complete genome) **vs. *SARS-CoV-2 Wuhan*** (NC_045512.2; alignment complete genome) ** **(Figure S11)**	98.8%	
SARS-CoV-2 Omicron BA.2 (##; alignment complete genome) **vs. *SARS-CoV-2 Wuhan-Hu-1*** (NC_045512.2; alignment complete genome) **	98.8%	

SARS-CoV-2 Omicron BA.2.75 (EPI_ISL_13989997; alignment complete genome) **vs.** ***SARS-CoV-2 Wuhan-Hu-1*** (NC_045512.2; alignment complete genome) **	98.8%	99%
SARS-CoV-2 Omicron BA.3 (EPI_ISL_10327378; alignment complete genome) **vs.** ***SARS-CoV-2 Wuhan-Hu-1*** (NC_045512.2; alignment complete genome) **	98.8%	99%
SARS-CoV-2 Omicron BA.4 (EPI_ISL_14834909; alignment complete genome) **vs.** ***SARS-CoV-2 Wuhan-Hu-1*** (NC_045512.2; alignment complete genome) **	98.6%	99%
SARS-CoV-2 Omicron BA.5 (EPI_ISL_13035233; alignment complete genome) **vs.** ***SARS-CoV-2 Wuhan-Hu-1*** (NC_045512.2; alignment complete genome) **	98.8%	99%
SARS-CoV-2 Omicron BA.4 (EPI_ISL_14834909; alignment complete genome) **vs.** ***SARS-CoV-2 Omicron BA.5*** (EPI_ISL_13035233; alignment complete genome) **	98.5%	99%
SARS-CoV-2 Wuhan-Hu-1 (NC_045512.2; alignment complete genome) **vs.** ***SARS-CoV-2 XBB.1.5*** (EPI_ISL_16154660; alignment complete genome)	99.4%	99%
SARS-CoV-2 Wuhan-Hu-1 (NC_045512.2; alignment complete genome) **vs.** ***Arabidopsis thaliana*** **chromosome 5** (NC_003076.8; alignment from 653,301-686,079nt) **	50.8%	46%
SARS-CoV-2 Wuhan-Hu-1 (NC_045512.2; alignment complete genome) **vs.** ***Oryza sativa*** **chromosome 1** (BA000010.8; alignment from 9,891-41,663nt) **	49.2%	46%
SARS-CoV-2 Wuhan-Hu-1 (NC_045512.2; alignment from 16,644-25,718nt) **vs.** ***HTLV-1*** (AF033817.1; alignment complete genome) **	43.8%	44%
Sulfolobus turreted icosahedral virus (NC_005892; alignment complete genome) **vs.** ***SARS-CoV-2 Wuhan-Hu-1*** (NC_045512.2; alignment from 3,368-22,160nt) *	45.5%	46%
Homo sapiens **chromosome 7** (NC_000007.14; alignment from 143,456-153,536nt) **vs.** ***Ilex aquifolium*** **chromosome 11** (OX637401.1; alignment from 1,289-11,294nt) **	43.7%	44%

***Oryza sativa* chromosome 2** (NC_029257.1; alignment from 35,937-45,937nt) **vs. *Ilex aquifolium* chromosome 11** (OX637401.1; alignment from 1,289-11,293nt) **	42.9%	43%
***Homo sapiens* mitochondrion** (NC_012920.1; alignment complete genome) **vs. *Latimeria chalumnae* mitochondrion** (NC_001804.1; alignment complete genome) **	62.8%	63%
***Bombus pascuorum* chromosome 14** (NC_083501.1; alignment from 114,012-124,012nt) **vs. *Oryza sativa* chromosome 11** (NC_029266.1; alignment from 82,863-92,863nt) **	44.2%	44%
***Cylas formicarius* mitochondrion** (NC_046580.1; alignment complete genome) **vs. *Homo sapiens* chromosome 3** (NC_000003.12; alignment from 45,687-62,837nt) **	46.5%	46%
***Homo sapiens* chromosome 17** (NC_000017.11; alignment from 796,234-806,234nt) **vs. *Lycium barbarum* isolate Lr01 chromosome 6** (NC_083342.1; alignment from 8,334,652-8,344,652nt) ** (**Figure S12**)	42.2%	42%
***Homo sapiens* chromosome X** (NC_000023.11; alignment from 604,089-614,089nt) **vs. *Zootoca vivipara* chromosome W** (NC_083293.1; alignment from 563,489-573,489nt) **	42.2%	42%
Mycobacterium tuberculosis (AP018036.1; alignment from 4,403,362-4,413,362nt) **vs. *Oryza sativa* chromosome 8** (NC_029263.1; alignment from 844,302-854,302nt) **	40.2%	40%
Ilex aquifolium* chromosome 11** (OX637401.1; alignment from 1,289-11,293nt) **vs. *Sus scrofa isolate TJ Tabasco breed Duroc **chromosome 13** (NC_010455.5; alignment from 334,590-344,590nt) **	43.9%	44%
Mus musculus strain C57BL/6J **chromosome 19** (NC_000085.7; alignment from 7,159,736-7,169,736nt) **vs. *Triticum aestivum*** cultivar Chinese Spring **chromosome 6D** (NC_057811.1; alignment from 5,380,293-5,390,293nt) **	43.1%	43%

* Data from Weber et al., 2022 [29]. ** New data. # EPI_ISL_9002822 (alignment from 1-29,714nt), EPI_ISL_9021871(alignment from 1-29,891nt), EPI_ISL_9049423 (alignment from 1-29,717nt), EPI_ISL_9055228(alignment from 1-29,785nt), EPI_ISL_9062229(alignment from 1-29,747nt), EPI_ISL_9064167, EPI_ISL_9070358, EPI_ISL_9070380, EPI_ISL_9070606, EPI_ISL_90710188; Some of the entire alignments can be inspected under **Supplemental Materials, Figures S1** to **S15**.

Table 1. - Nucleotide sequence comparisons among 65 genome pairs from a wide range of taxonomically diverse species. Common names for selected species include: *Ilex aquifolium* (English holly), *Bombus pascuorum* (common carder bee; see Figure S5), *Latimeria chalumnae* (coelacanth, closely related to lungfish; **Figure S6**), *Cylas formicarius* (sweet potato weevil), *Lycium barbarum* (matrimony vine), *Zootoca vivipara* (viviparous lizard), *Sus scrofa* (wild boar), and *Triticum aestivum* (common wheat). Genomic coordinates for each alignment are provided in the leftmost column. All comparisons span the full length of the shorter sequence in each pair. As detailed in the Materials and Methods section, alignments were conducted using two independent programs—Vector NTI and BLAST. The results from both methods are presented in adjacent columns and show high concordance, often yielding identical identity percentages.
