# Supplementary material for: Patch type nucleotide sequence identities between genomes from many different species facilitate illegitimate recombination
Source: Sci Rep. 2026 Mar 30;16:10524. doi: 10.1038/s41598-026-44124-0 (PMC13035915; doi:10.1038/s41598-026-44124-0)
Supplement: Supplementary file 20 — Supplementary Material 20 [file 41598_2026_44124_MOESM20_ESM.docx]

| **Alignment** | **GC content** | **VNTI®**  **Identity Positions** | **BLAST®**  **Percent Identities** |
| --- | --- | --- | --- |
| ***Acidianus rod-shaped virus 1*** (NC-009965.1; alignment complete genome) **vs. *SARS-CoV-2*** ***Wuhan-Hu-1*** (NC_045512.2; alignment from 3,352-29,903nt) *** | 39% vs. 38% | 46.2% | 45% |
| ***Adenovirus type 2*** (J01917.1; alignment complete genome) **vs.**  ***SARS-CoV-2*** ***Wuhan-Hu-1*** (NC_045512.2; alignment complete genome) *** | 55% vs. 38% | 43.2% | 43% |
| ***Adenovirus type 5*** (AC_000008.1; alignment complete genome) **vs.**  ***SARS-CoV-2 Wuhan-Hu-1*** (NC_045512.2; alignment complete genome) *** | 55% vs. 38% | 43.4% | 43% |
| ***Adenovirus type 12*** (X73487; alignment from 556-33,354nt) **vs.**  ***SARS-CoV-2* *Wuhan-Hu-1*** (NC_045512.2; alignment complete genome) *** | 46.5% vs. 38% | 44.9% | 45% |
| ***Adenovirus type 12*** (X73487; alignment from 12,239-24,660nt) **vs.**  ***HERV-E*** (AB062274.1; alignment complete genome) ** | 46.5% vs. 47.71% | 44.9% | 45% |
| ***Adenovirus type 12*** (X73487; alignment from 13,005-33,725nt) **vs.**  ***HERV-K*** (AF074086; alignment complete genome) ** | 46.5% vs. 41.76% | 44.8% | 45% |
| ***Adenovirus type 12*** (X73487; alignment from 25,874-29,259nt) **vs.**  ***HERV-W*** (NM_014590.4; alignment from 1-3,044nt) ** | 46.5% vs. 46.75% | 46.1% | 46% |
| ***Adenovirus type 12*** (X73487; alignment from 15,503-22,066nt) **vs.**  ***LINE 1.2*** (M80343; alignment complete genome) ** | 46.5% vs. 42.43% | 45.7% | 45% |
| ***Adenovirus type 12*** (X73487; alignment from 1-34,125nt) **vs.** ***Homo sapiens* chromosome 1** (NC_000001.11; alignment from 11,783,698-11,817,823nt) ** | 46.5% vs. 51.27% | 44.3% | 44% |
| ***Adenovirus type 12*** (X73487; alignment complete genome) **vs. *Homo sapiens* chromosome 13** (NC_000013.11; alignment from 34,882,059-34,916,184nt) ** | 46.5% vs. 39.23% | 44.1% | 44% |
| ***Alternaria brassicicola endornavirus*** (NC_026136.1; alignment complete genome) **vs.** ***HERV-E*** (AB062274.1; complete genome) ** | 48.5% vs. 47.71% | 44.2% | 45% |
| ***Alternaria brassicicola endornavirus*** (NC_026136.1; alignment complete genome) **vs.** ***HERV-K*** (AF074086; alignment from 5,027-16,155nt) ** | 48.5% vs. 41.76% | 45% | 45% |
| ***Alternaria brassicicola endornavirus*** (NC_026136.1; alignment from 180-3,554nt) **vs. *HERV-W*** (NM_014590.4; alignment from 1-3,044nt) ** | 48.5% vs. 46.75% | 45.6% | 45% |
| ***Alternaria brassicicola endornavirus*** (NC_026136.1; alignment from 3,503-10,069nt) **vs. *LINE 1.2*** (M80343; alignment complete genome) ** | 48.5% vs. 42.43% | 45.8% | 46% |
| ***Alternaria brassicicola endornavirus*** (NC_026136.1; alignment complete genome) **vs. *SARS-CoV-2 Wuhan-Hu-1*** (NC_045512.2; alignment from 353-10,639nt) *** | 48.5% vs. 38% | 44.5% | 44% |
| ***Autographa californica nuclear polyhedrosis virus*** (NC_001623.1; alignment from 42,843-75,223nt) **vs.** ***SARS-CoV-2 Wuhan-Hu-1*** (NC_045512.2; alignment complete genome) *** | 39.03% vs. 38% | 45.6% | 45% |
| ***Candidatus Carsonella ruddii* strain BT** (CP024798.1; alignment from 135,557-167,442nt) **vs. *SARS-CoV-2 Wuhan-Hu-1*** (NC_045512.2; alignment complete genome) *** | 14.9% vs. 38% | 46% | 46% |
| ***Carrot mottle mimic umbravirus*** (ds-RNA) (NC_001726.1; alignment complete genome) **vs.** ***SARS-CoV-2 Wuhan-Hu-1*** (NC_045512.2; alignment from 1,990-6,319nt) *** | 52.5% vs. 38% | 45% | 45% |
| ***Chlorocebus sabaeus* mitochondrion** (NC_008066.1; alignment from 2,779-15,177nt) **vs. *HERV-E*** (AB062274.1; alignment complete genome) ** | 42.5% vs. 47.71% | 44.5% | 44% |
| ***Chlorocebus sabaeus* mitochondrion** (NC_008066.1; alignment complete genome) **vs. *HERV-K*** (AF074086; alignment from 636-19,135nt) ** | 42.5% vs. 41.76% | 44.6% | 44% |
| ***Chlorocebus sabaeus* mitochondrion** (NC_008066.1; alignment from 5,862-9,251nt) **vs. *HERV-W*** (NM_014590.4; alignment from 1-3,044nt) ** | 42.5% vs. 46.75% | 46.8% | 47% |
| ***Chlorocebus sabaeus* mitochondrion** (NC_008066.1; alignment from 128-6,484nt) **vs. *LINE 1.2*** (M80343; alignment complete genome) ** | 42.5% vs. 42.43% | 46% | 46% |
| ***Chlorocebus sabaeus* mitochondrion** (NC_008066.1; alignment complete genome) **vs. *Homo sapiens* mitochondrion** (NC_012920.1; alignment complete genome) ** | 42.5% vs. 44.36% | 78.7% | 79% |
| ***Escherichia coli* K12** (NC_000913.3; 129,062–161,212 nt) **vs.**  ***SARS-CoV-2 Wuhan-Hu-1*** (NC_045512.2; alignment complete genome) *** | 49.6% vs. 38% | 45.2% | 45% |
| ***Fig badnavirus 1*** (NC_017830.1; alignment complete genome) **vs.**  ***SARS-CoV-2 Wuhan-Hu-1*** (NC_045512.2; alignment from 1,978-9,647nt) *** | 43% vs. 38% | 45.5% | 45% |
| ***Hepatitis B Virus*** (NC_003977.2; alignment complete genome) **vs. *SARS-CoV-2 Wuhan-Hu-1*** (NC_045512.2; alignment from 21,047-24,558nt) *** | 48.49% vs. 38% | 45.7% | 46% |
| ***HPV 16*** (NC_001526; alignment from 1-7,905nt) **vs.**  ***HERV- E*** (AB062274.1; alignment from 1,618-10,463nt) ** | 36.5% vs. 47.71% | 45% | 44% |
| ***HPV 16*** (NC_001526; alignment from 1-7,905nt) **vs.**  ***HERV- K*** (AF074086; alignment from 5,016-13,700nt) ** | 36.5% vs. 41.76% | 46% | 45% |
| ***HPV 16*** (NC_001526; alignment from 2,800-6,032nt) **vs.**  ***HERV- W*** (NM_014590.4; alignment from 1-3,044nt) ** | 36.5% vs. 46.75% | 44.9% | 45% |
| ***HPV 16*** (NC_001526; alignment from 25-6,598nt) **vs.**  ***LINE 1.2*** (M80343; alignment complete genome) ** | 36.5% vs. 42.43% | 45.8% | 45% |
| ***Homo sapiens* chromosome 1** (NC_000001.11; alignment from 11,783,698-11,817,823nt) **vs. *Homo sapiens* chromosome 13** (NC_000013.11; alignment from 34,882,059-34,916,184nt) ** | 51.27% vs. 39.23% | 44.5 % | 45% |
| ***Homo sapiens* mitochondrial DNA** (NC_012920.1; alignment complete genome) **vs. *SARS-CoV-2 Wuhan-Hu-1*** (NC_045512.2; alignment 11,168-29,055nt) *** | 44.36% vs. 38% | 45.3% | 45% |
| ***Human Immunodeficiency Virus 1*** (K03455.1; alignment complete genome) **vs. *SARS-CoV-2 Wuhan-Hu-1*** (NC_045512.2; alignment from 699-11,486nt) *** | 42.5% vs. 38% | 45.8% | 46% |
| ***Nitrosopumilus spindle-shaped virus*** (NC_048199.1; alignment complete genome) **vs. *SARS-CoV-2 Wuhan-Hu-1*** (NC_045512.2; alignment complete genome)*** | 29.81% vs. 38% | 47% | 46% |
| **Phage *lambda*** (*λ*) (NC_001416.1; alignment from 14,752-47,353nt) **vs. *SARS-CoV-2 Wuhan-Hu-1*** (NC_045512.2; alignment complete genome) *** | 46.97% vs. 38% | 45.3% | 45% |
| **Phage *T4*** (NC_000866.4; alignment from 9,803-42,013nt) **vs. *SARS-CoV-2 Wuhan-Hu-1*** (NC_045512.2; alignment complete genome) *** | 33.5% vs. 38% | 46.4% | 46% |
| **Phage *mu*** (NC_000929.1; alignment from 3,019-35,926nt) **vs. *SARS-CoV-2 Wuhan-Hu-1*** (NC_045512.2; alignment complete genome) *** | 52.72% vs. 38% | 44.2% | 44% |
| ***SARS-CoV-2* *Wuhan-Hu-1*** (NC_045512.2; alignment from 17,170-29,465nt) **vs. *HERV- E*** (AB062274.1; alignment complete genome) ** | 38% vs. 47.71% | 45.2% | 45% |
| ***SARS-CoV-2* *Wuhan-Hu-1*** (NC_045512.2; alignment from 3,949-25,100nt) **vs. *HERV- K*** (AF074086; alignment complete genome) ** | 38% vs. 41.76% | 45.6% | 46% |
| ***SARS-CoV-2* *Wuhan-Hu-1*** (NC_045512.2; alignment from 24,378-27,503nt) **vs. *HERV- W*** (NM_014590.4; alignment from 1-3,044nt) ** | 38% vs. 46.75% | 46% | 46% |
| ***SARS-CoV-2* *Wuhan-Hu-1*** (NC_045512.2; alignment from 234-6,608nt) **vs. *LINE 1.2*** (M80343; alignment complete genome) ** | 38% vs. 42.43% | 45.9% | 46% |
| ***SARS-CoV-2* *Wuhan-Hu-1*** (NC_045512.2; alignment complete genome) **vs. *Homo sapiens* chromosome 1** (NC_000001.11; alignment from 11,783,698-11,813,601nt) ** | 38% vs. 51.27% | 44% | 44% |
| ***SARS-CoV-2* *Wuhan-Hu-1*** (NC_045512.2; alignment complete genome) **vs. *Homo sapiens* chromosome 13** (NC_000013.11; alignment from 34,882,059-34,911,962nt )** | 38% vs. 39.23% | 49.4% | 45% |
| ***SARS-CoV-2* *Omicron BA.1*** (#alignment complete genome) **vs. *SARS-CoV-2* *Wuhan*** (NC_045512.2; alignment complete genome) ** **(Figure S11**) | 37.97% vs. 38% | 98.8% |  |
| ***SARS-CoV-2* *Omicron BA.2*** (##; alignment complete genome) **vs. *SARS-CoV-2* *Wuhan-Hu-1*** (NC_045512.2; alignment complete genome) ** | 37.98% vs. 38% | 98.8% |  |
| ***SARS-CoV-2* *Omicron BA.2.75*** (EPI_ISL_13989997; alignment complete genome) **vs. *SARS-CoV-2* *Wuhan-Hu-1*** (NC_045512.2; alignment complete genome) ** | 37.93% vs. 38% | 98.8% | 99% |
| ***SARS-CoV-2* *Omicron BA.3*** (EPI_ISL_10327378; alignment complete genome) **vs. *SARS-CoV-2* *Wuhan-Hu-1*** (NC_045512.2; alignment complete genome) ** | 38.01% vs. 38% | 98.8% | 99% |
| ***SARS-CoV-2* *Omicron BA.4*** (EPI_ISL_14834909; alignment complete genome) **vs. *SARS-CoV-2* *Wuhan-Hu-1*** (NC_045512.2; alignment complete genome) ** | 37.98% vs. 38% | 98.6% | 99% |
| ***SARS-CoV-2* *Omicron BA.5*** (EPI_ISL_13035233; alignment complete genome) **vs. *SARS-CoV-2* *Wuhan-Hu-1*** (NC_045512.2; alignment complete genome) ** | 37.92% vs. 38% | 98.8% | 99% |
| ***SARS-CoV-2* *Omicron BA.4*** (EPI_ISL_14834909; alignment complete genome) **vs. *SARS-CoV-2* *Omicron BA.5*** (EPI_ISL_13035233; alignment complete genome) ** | 37.92% vs. 37.92% | 98.5% | 99% |
| ***SARS-CoV-2* *Wuhan-Hu-1*** (NC_045512.2; alignment complete genome) **vs. *SARS-CoV-2 XBB.1.5*** (EPI_ISL_16154660; alignment complete genome) | 38% vs. 37.91% | 99.4% | 99% |
| ***SARS-CoV-2* *Wuhan-Hu-1*** (NC_045512.2; alignment complete genome) **vs. *Arabidopsis thaliana* chromosome 5** (NC_003076.8; alignment from 653,301-686,079nt) ** | 38% vs. 36.92% | 50.8% | 46% |
| ***SARS-CoV-2* *Wuhan-Hu-1*** (NC_045512.2; alignment complete genome) **vs. *Oryza sativa* chromosome 1** (BA000010.8; alignment from 9,891-41,663nt) ** | 38% vs. 41.52% | 49.2% | 46% |
| ***SARS-CoV-2* *Wuhan-Hu-1*** (NC_045512.2; alignment from 16,644-25,718nt) **vs. *HTLV-1*** (AF033817.1; alignment complete genome) ** | 38% vs. 53.5% | 43.8% | 44% |
| ***Sulfolobus turreted icosahedral virus*** (NC_005892; alignment complete genome) **vs. *SARS-CoV-2 Wuhan-Hu-1*** (NC_045512.2; alignment from 3,368-22,160nt) *** | 36% vs. 38% | 45.5% | 46% |
| ***Homo sapiens* chromosome 7** (NC_000007.14; alignment from 143,456-153,536nt) **vs.** ***Ilex aquifolium*** **chromosome 11** (OX637401.1; alignment from 1,289-11,294nt) ** | 48.98% vs. 53.72% | 43.7% | 44% |
| ***Oryza sativa*** **chromosome 2** (NC_029257.1; alignment from 35,937-45,937nt) **vs.** ***Ilex aquifolium*** **chromosome 11** (OX637401.1; alignment from 1,289-11,293nt) ** | 42.73% vs. 53.72% | 42.9% | 43% |
| ***Homo sapiens* mitochondrion** (NC_012920.1; alignment complete genome) **vs.** ***Latimeria chalumnae*** **mitochondrion** (NC_001804.1; alignment complete genome) ** | 44.36% vs. 41.67% | 62.8% | 63% |
| ***Bombus pascuorum* chromosome 14** (NC_083501.1; alignment from 114,012-124,012nt) **vs.** ***Oryza sativa* chromosome 11** (NC_029266.1; alignment from 82,863-92,863nt) ** | 37.68% vs. 39.1% | 44.2% | 44% |
| ***Cylas formicarius* mitochondrion** (NC_046580.1; alignment complete genome) **vs.** ***Homo sapiens*** **chromosome 3** (NC_000003.12; alignment from 45,687-62,837nt) ** | 21.6% vs. 36.85% | 46.5% | 46% |
| ***Homo sapiens* chromosome 17** (NC_000017.11; alignment from 796,234-806,234nt) **vs. *Lycium barbarum* isolate Lr01 chromosome 6** (NC_083342.1; alignment from 8,334,652-8,344,652nt) ** (**Figure S12**) | 57.03% vs. 38.05% | 42.2% | 42% |
| ***Homo sapiens* chromosome X** (NC_000023.11; alignment from 604,089-614,089nt) **vs.** ***Zootoca vivipara* chromosome W** (NC_083293.1; alignment from 563,489-573,489nt) ** | 41.77% vs. 50.11% | 42.2% | 42% |
| ***Mycobacterium tuberculosis*** (AP018036.1; alignment from 4,403,362-4,413,362nt) **vs.** ***Oryza sativa* chromosome 8** (NC_029263.1; alignment from 844,302-854,302nt) ** | 63.09% vs. 33.19% | 40.2% | 40% |
| ***Ilex aquifolium*** **chromosome 11** (OX637401.1; alignment from 1,289-11,293nt) **vs.** ***Sus scrofa*** isolate TJ Tabasco breed Duroc **chromosome 13** (NC_010455.5; alignment from 334,590-344,590nt) ** | 53.72% vs. 41.28% | 43.9% | 44% |
| ***Mus musculus*** strain C57BL/6J **chromosome 19** (NC_000085.7; alignment from 7,159,736-7,169,736nt) **vs.** ***Triticum aestivum*** cultivar Chinese Spring **chromosome 6D** (NC_057811.1; alignment from 5,380,293-5,390,293nt) ** | 55.1% vs. 44.91% | 43.1% | 43% |
