# Supplementary material for: Patch type nucleotide sequence identities between genomes from many different species facilitate illegitimate recombination
Source: Sci Rep. 2026 Mar 30;16:10524. doi: 10.1038/s41598-026-44124-0 (PMC13035915; doi:10.1038/s41598-026-44124-0)
Supplement: Supplementary file 21 — Supplementary Material 21 [file 41598_2026_44124_MOESM21_ESM.docx]

**Table S3****:** Summary of DNA alignments between SARS-CoV-2 Wuhan-Hu-1 and shuffled DNAs from Mycobacterium tuberculosis (region from 4,322,553 to 4,354,863nt).

| **DNA Alignments** | **GC content** | **VNTI®**  **Identity Positions** |
| --- | --- | --- |
| *SARS-CoV-2 Wuhan-Hu-1* (NC_045512.2; alignment complete genome) vs. *Mycobacterium tuberculosis* (AP018036.1; alignment from 4,322,553 to 4,354,863nt) | 38%*** vs. 63.86% | 42.2% |
| *SARS-CoV-2 Wuhan-Hu-1* (NC_045512.2; alignment complete genome) vs *Mycobacterium* *tuberculosis* shuffled 1 (DNA shuffled from region 4,322,553 to 4,354,863nt) | 38%*** vs. 63.86% | 41.3% |
| *SARS-CoV-2 Wuhan-Hu-1* (NC_045512.2; alignment complete genome) vs *Mycobacterium* *tuberculosis* shuffled 2 (DNA shuffled from region 4,322,553 to 4,354,863nt) | 38%*** vs. 63.86% | 41.8% |
| *SARS-CoV-2 Wuhan-Hu-1* (NC_045512.2; alignment complete genome) vs *Mycobacterium* *tuberculosis* shuffled 3 (DNA shuffled from region 4,322,553 to 4,354,863nt) | 38%*** vs. 63.86% | 41.3% |
| *SARS-CoV-2 Wuhan-Hu-1* (NC_045512.2; alignment complete genome) vs *Mycobacterium* *tuberculosis* shuffled 4 (DNA shuffled from region 4,322,553 to 4,354,863nt) | 38%*** vs. 63.86% | 41.2% |
| *SARS-CoV-2 Wuhan-Hu-1* (NC_045512.2; alignment complete genome) vs *Mycobacterium* *tuberculosis* shuffled 5 (DNA shuffled from region 4,322,553 to 4,354,863nt) | 38%*** vs. 63.86% | 41.1% |
| *SARS-CoV-2 Wuhan-Hu-1* (NC_045512.2; alignment complete genome) vs *Mycobacterium* *tuberculosis* shuffled 6 (DNA shuffled from region 4,322,553 to 4,354,863nt) | 38%*** vs. 63.86% | 41.6% |
| *SARS-CoV-2 Wuhan-Hu-1* (NC_045512.2; alignment complete genome) vs *Mycobacterium* *tuberculosis* shuffled 7 (DNA shuffled from region 4,322,553 to 4,354,863nt) | 38%*** vs. 63.86% | 41.1% |
| *SARS-CoV-2 Wuhan-Hu-1* (NC_045512.2; alignment complete genome) vs *Mycobacterium* *tuberculosis* shuffled 8 (DNA shuffled from region 4,322,553 to 4,354,863nt) | 38%*** vs. 63.86% | 41.1% |
| *SARS-CoV-2 Wuhan-Hu-1* (NC_045512.2; alignment complete genome) vs *Mycobacterium* *tuberculosis* shuffled 9 (DNA shuffled from region 4,322,553 to 4,354,863nt) | 38%*** vs. 63.86% | 41% |
| *SARS-CoV-2 Wuhan-Hu-1* (NC_045512.2; alignment complete genome) vs *Mycobacterium* *tuberculosis* shuffled 10 (DNA shuffled from region 4,322,553 to 4,354,863nt) | 38%*** vs. 63.86% | 41.6% |

*Mycobacterium tuberculosis* (AP018036.1) complete genome: 65,61%

*Mycobacterium tuberculosis* (AP018036.1; alignment from 4,322,553 to 4,354,863nt): 63.86%

*SARS-CoV-2 Wuhan-Hu-1* (NC_045512.2) complete genome: 38%***

*** The GC content of the DNA sequence is taken from the NCBI database. All other GC values were determined using the online tool “GC Content Calculator” from VectorBuilder (<https://en.vectorbuilder.com/tool/gc-content-calculator.html>)

**Table S4:** Summary of DNA alignments between shuffled DNAs from SARS-CoV-2 Wuhan-Hu-1 and shuffled DNAs from Mycobacterium tuberculosis (region from 4,322,553 to 4,354,863nt)

| **DNA Alignments** | **GC content** | **VNTI®**  **Identity Positions** |
| --- | --- | --- |
| *SARS-CoV-2 Wuhan-Hu-1* (NC_045512.2; alignment complete genome) vs. *Mycobacterium tuberculosis* (AP018036.1; alignment from 4,322,553 to 4,354,863nt) | 38%*** vs. 63.86% | 42.2% |
| *SARS-CoV-2 Wuhan-Hu-1* shuffled DNA 1 (NC_045512.2; alignment complete genome) vs *Mycobacterium* *tuberculosis* shuffled 1 (DNA shuffled from region 4,322,553 to 4,354,863nt) | 38%*** vs. 63.86% | 41.5% |
| *SARS-CoV-2 Wuhan-Hu-1* shuffled DNA 2 (NC_045512.2; alignment complete genome) vs *Mycobacterium* *tuberculosis* shuffled 2 (DNA shuffled from region 4,322,553 to 4,354,863nt) | 38%*** vs. 63.86% | 41.6% |
| *SARS-CoV-2 Wuhan-Hu-1* shuffled DNA 3 (NC_045512.2; alignment complete genome) vs *Mycobacterium* *tuberculosis* shuffled 3 (DNA shuffled from region 4,322,553 to 4,354,863nt) | 38%*** vs. 63.86% | 41.4% |
| *SARS-CoV-2 Wuhan-Hu-1* shuffled DNA 4 (NC_045512.2; alignment complete genome) vs *Mycobacterium* *tuberculosis* shuffled 4 (DNA shuffled from region 4,322,553 to 4,354,863nt) | 38%*** vs. 63.86% | 41.7% |
| *SARS-CoV-2 Wuhan-Hu-1* shuffled DNA 5 (NC_045512.2; alignment complete genome) vs *Mycobacterium* *tuberculosis* shuffled 5 (DNA shuffled from region 4,322,553 to 4,354,863nt) | 38%*** vs. 63.86% | 40.9% |
| *SARS-CoV-2 Wuhan-Hu-1* shuffled DNA 6 (NC_045512.2; alignment complete genome) vs *Mycobacterium* *tuberculosis* shuffled 6 (DNA shuffled from region 4,322,553 to 4,354,863nt) | 38%*** vs. 63.86% | 41.6% |
| *SARS-CoV-2 Wuhan-Hu-1* shuffled DNA 7 (NC_045512.2; alignment complete genome) vs *Mycobacterium* *tuberculosis* shuffled 7 (DNA shuffled from region 4,322,553 to 4,354,863nt) | 38%*** vs. 63.86% | 41.8% |
| *SARS-CoV-2 Wuhan-Hu-1* shuffled DNA 8 (NC_045512.2; alignment complete genome) vs *Mycobacterium* *tuberculosis* shuffled 8 (DNA shuffled from region 4,322,553 to 4,354,863nt) | 38%*** vs. 63.86% | 41% |
| *SARS-CoV-2 Wuhan-Hu-1* shuffled DNA 9 (NC_045512.2; alignment complete genome) vs *Mycobacterium* *tuberculosis* shuffled 9 (DNA shuffled from region 4,322,553 to 4,354,863nt) | 38%*** vs. 63.86% | 42% |
| *SARS-CoV-2 Wuhan-Hu-1* shuffled DNA 10 (NC_045512.2; alignment complete genome) vs *Mycobacterium* *tuberculosis* shuffled 10 (DNA shuffled from region 4,322,553 to 4,354,863nt) | 38%*** vs. 63.86% | 41.1% |

*Mycobacterium tuberculosis* (AP018036.1) complete genome: 65,61%

*Mycobacterium tuberculosis* (AP018036.1; alignment from 4,322,553 to 4,354,863nt): 63.86%

*SARS-CoV-2 Wuhan-Hu-1* (NC_045512.2) complete genome: 38%***

*** The GC content of the DNA sequence is taken from the NCBI database. All other GC values were determined using the online tool “GC Content Calculator” from VectorBuilder (https://en.vectorbuilder.com/tool/gc-content-calculator.html).
