# Supplementary material for: Patch type nucleotide sequence identities between genomes from many different species facilitate illegitimate recombination
Source: Sci Rep. 2026 Mar 30;16:10524. doi: 10.1038/s41598-026-44124-0 (PMC13035915; doi:10.1038/s41598-026-44124-0)
Supplement: Supplementary file 22 — Supplementary Material 22 [file 41598_2026_44124_MOESM22_ESM.docx]

| **Pair** | **Pattern Segment** | **Percent Identity Real** | **Percent Identity RC** | **Median Identity- shuffled** | **Identity IQR- shuffled** |
| --- | --- | --- | --- | --- | --- |
| *SARS2* Wuhan vs *Adenovirus 2* | 9952-19951 (10000 bp) | 46,057 | 46,232 | 17,863 | 0,130 |
| *SARS2* Wuhan vs *Arabidopsis* cp | 9952-19951 (10000 bp) | 48,459 | 48,588 | 16,480 | 0,093 |
| *SARS2* Wuhan vs Human mtDNA | 3285-13284 (10000 bp) | 46,411 | 47,699 | 26,965 | 0,202 |
| *SARS2* Wuhan vs *Lambda* phage | 9952-19951 (10000 bp) | 48,016 | 46,889 | 15,900 | 0,090 |
| *SARS2* Wuhan vs  *M. tuberculosis* | 9952-19951 (10000 bp) | 45,155 | 44,861 | 18,303 | 0,095 |
| *SARS2* Wuhan vs  *O. sativa* chr 8 | 9952-19951 (10000 bp) | 48,624 | 48,459 | 16,832 | 0,094 |
| *SARS2* Wuhan vs *T7*_phage | 9952-19951 (10000 bp) | 47,896 | 47,105 | 17,903 | 0,089 |

**Table S5 -** Overall identities in random regions are low (26.5% and 30.5% for *M. tuberculosis* and *P. falciparum,* respectively. For *Ad2*, there is a 21-nucleotide region of 95.2% identity. Real identities lie between 45 and 48% as expected.
